# Quantitative proteomic biomarkers from extracellular vesicles of human seminal plasma in the differential diagnosis of azoospermia

**DOI:** 10.1002/ctm2.423

**Published:** 2021-05-28

**Authors:** Liping Yao, Yueshuai Guo, Xiangzheng Zhang, Chen Xu, Yichun Wang, Xiaofei Liu, Yue Wang, Yan Li, Yaling Qi, Jiahao Sha, Chao Qin, Xiaoyu Yang, Xuejiang Guo

**Affiliations:** ^1^ State Key Laboratory of Reproductive Medicine Department of Histology and Embryology Nanjing Medical University Nanjing China; ^2^ Department of Urology The First Affiliated Hospital of Nanjing Medical University Nanjing China; ^3^ Clinical Center of Reproductive Medicine The First Hospital of Nanjing Medical University Nanjing China

Dear Editor,

Azoospermia, which accounts for about 10%–15% of infertile men, is generally classified as obstructive azoospermia (OA) due to male reproductive tract obstruction and nonobstructive azoospermia (NOA) due to testicular failure.^1^ NOA has three major forms: hypospermatogenesis (HS), germ cell arrest (MA), and Sertoli cell–only (SCO) based on the histopathological examination of testicular tissue.^2^ An accurate diagnosis of the subtype of azoospermia is crucial and mandatory, as the treatment approach differs between NOA and OA. However, the choices for high sensitivity and specificity in the noninvasive differential diagnosis of azoospermia are limited.^3^ Extracellular vesicles (EVs) are increasingly being considered as a promising source of novel diagnostic biomarkers due to their noninvasive nature and high reproducibility.^4^ In this study, we revealed that two proteins SLC5A12 and HIST1H2BA from human seminal plasma extracellular vesicle (spEV) could differentially diagnose azoospermia with high sensitivity and specificity.

To explore proteome and phosphoproteome changes in spEV from healthy individuals with normal sperm (NS), NOA, and OA patients, spEVs were purified by ultracentrifugation method, and high purity of EVs were obtained according to Western blot, Nano sight technology, and transmission electron microscopy analysis (Figure 1A–D). Proteomic and phosphoproteomic analyses of spEV from nine NS, nine NOA, and nine OA patients by tandem mass tag 10‐plex (Figure S1) identified 3785 proteins and 1533 phosphorylation sites from 748 phosphoproteins (Figure 1E; Tables S1A and S2‐3). Fifty proteins were differentially expressed, and they showed three main clusters (Figure 1F) with C2 cluster high in NS and NOA patients related to defense response to bacteria and fertilization, and C3 cluster low in NOA and OA patients related to sperm motility (Figure 1F and G; Table S4‐5). Near half (6/13) of the proteins in C2 were epididymis specific and more than half (18/34) of the proteins in C3 were testis specific in male reproductive system^5^ (Figure 1H), suggesting that they are sources of biomarkers to distinguish NOA from OA. The spEV phosphoproteome data showed 33 differential phosphorylated sites corresponding to 32 phosphoproteins, and are related to spermatid development and differentiation (Figure 1I and J; Table S6). Enrichment of proline or arginine at the +1 and +3 sites was observed in all identified phosphorylated sites, respectively, but not in the differential phosphosites (Figure S2).

**FIGURE 1:**
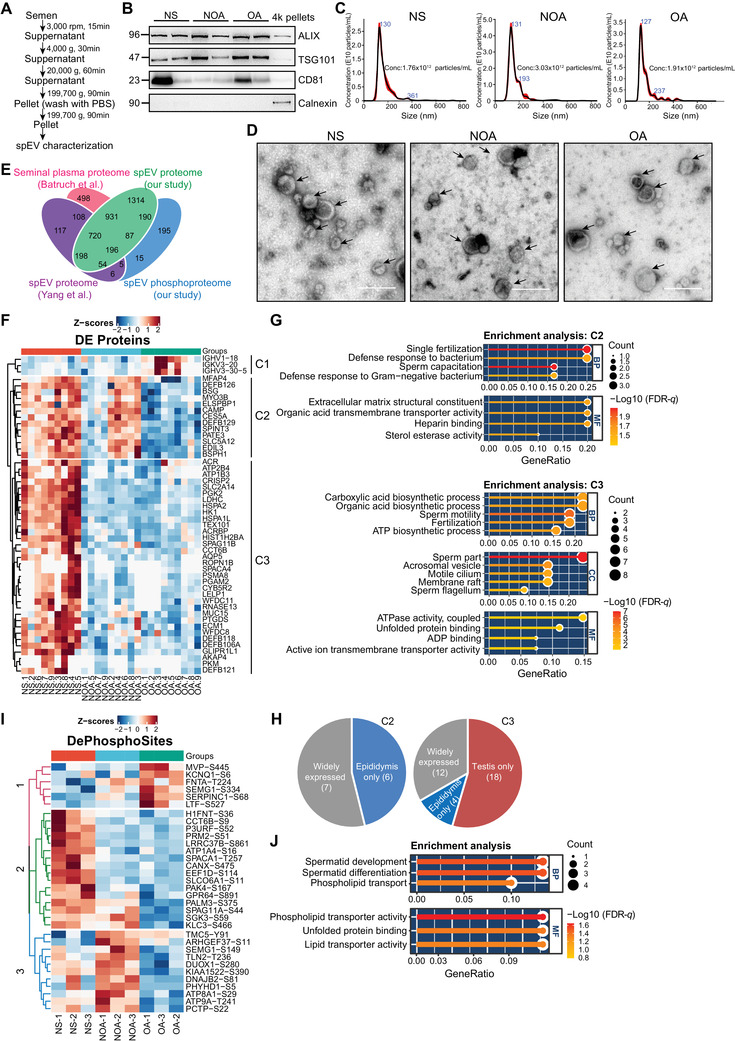
Proteomic and phosphoproteomic analyses of spEVs from NS, OA, and NOA patients. (A) The workflow for the isolation of spEV. (B) Immunoblots showing expression levels of known EV makers (CD81, ALIX, and TSG101) and absence of endoplasmic reticulum marker Calnexin in isolated spEVs, with pellets centrifuged at 4000 *g* for 30 min from the seminal plasma serving as the control. (C) Particle size distribution of spEVs measured by the Nano sight instrument had diameters mainly ranging from 30 to 150 nm with concentrations > 10^12^/mL. (D) Representative transmission electron microscopy (TEM) images of isolated spEVs. Black arrows indicated the purified round‐shaped spEVs with lipid bilayer membranes. Scale bar = 200 nm. (E) Comparison of the identified proteome and in our study with Yang et al.’s spEV proteome and Batruch et al.’s seminal plasma proteome, showing overlaps of 83% and 76%, respectively; 221 proteins only in phosphoproteome but not proteome, meaning phosophrylation enrichment can improve the sensitivity of protein identification. (F) Cluster and heatmap analysis of differentially expressed spEV proteins. Cluster 1 (C1) consisted of immunoglobulin heavy chain variable region proteins and showed the highest level in OA patients. Cluster 2 (C2) exhibited the highest level in NS and NOA patients. Cluster 3 (C3) showed the lowest level in NOA and OA patients. (G) Enriched gene ontology terms in differentially expressed proteins from cluster 2 (C2) and cluster 3 (C3), as shown in (F). (H) Tissue distribution in the male reproductive system of proteins from clusters 2 and 3 according to data in the Human Protein Atlas. (I) Cluster and heatmap analysis of spEV phosphoproteins with differentially phosphorylation levels in NS, OA, and NOA patients. (J) Enriched gene ontology terms in spEV phosphoproteins with differentially phosphorylation levels. NS, healthy individuals with normal sperm; NOA, nonobstructive azoospermia; OA, obstructive azoospermia

To identify potential biomarkers, multi‐step analysis of differentially expressed spEV (phospho)proteins was performed (Figure 2B). In total, 21 candidate proteins (19 proteins and two phosphoproteins) were subjected to validation in 42 spEV samples by relative quantification using parallel reaction monitoring (PRM)^6^ (Figure 2A–C and Figure S3A; Tables S1B and S7A). In NS>OA and NOA>OA group, SLC5A12 could differentiate NOA from OA with 100% specificity and sensitivity. In NS>OA and NS>NOA group, compared with other candidate proteins, including known biomarker TEX101,^7^ HIST1H2BA could be detected in most HS and MA samples (6/7, 86%), but not in SCO and OA samples (0/25, 0%) and was the best biomarker for distinguishing NOA subtypes (Figure 2C and Figure S3B). Phosphoproteins, SPACA1 (pT257), and SPAG11A (pS44) were not detected in the absence of phosphorylation enrichment (Figure 2C and Figure S3B), and were not further considered.

**FIGURE 2:**
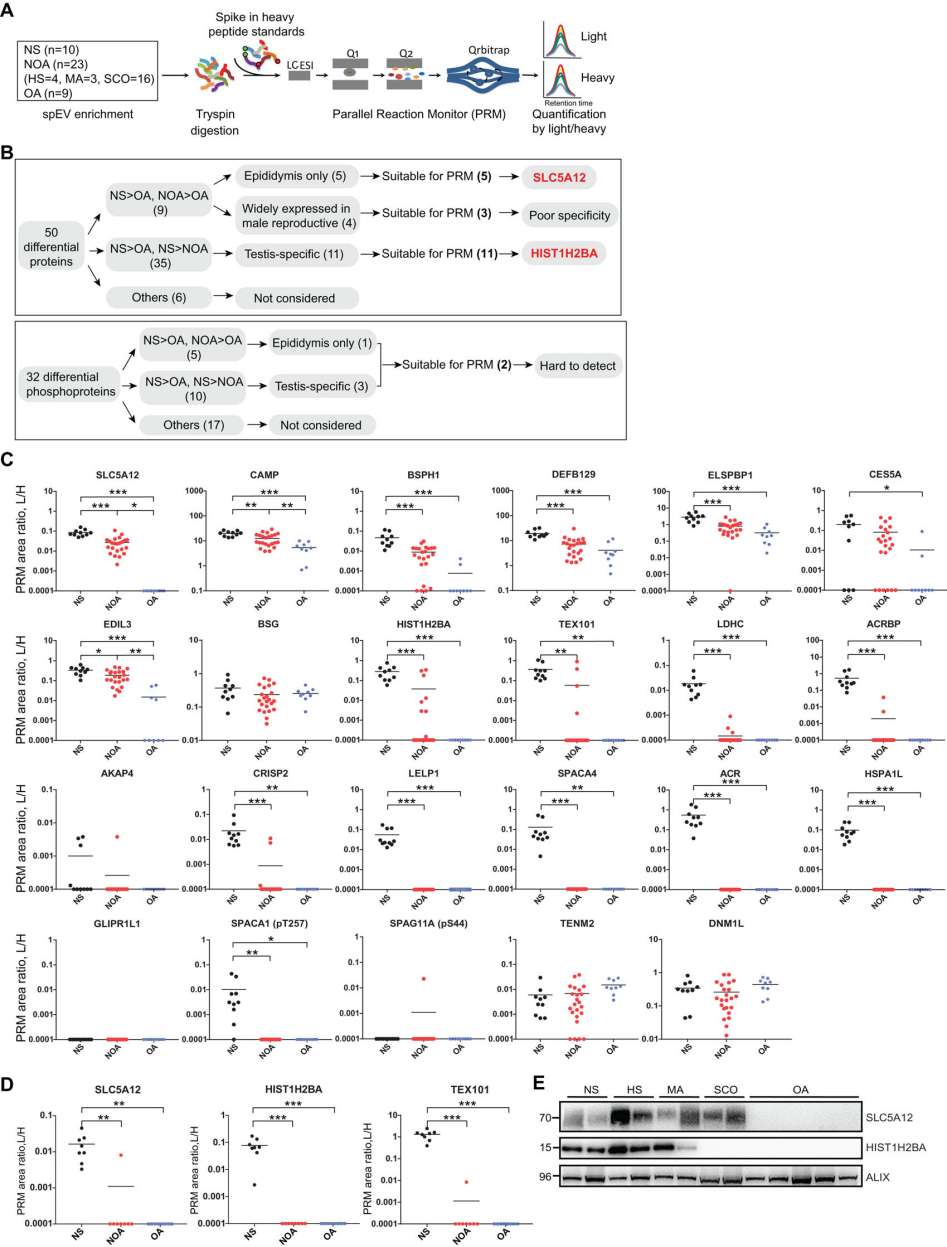
Validation of differentially expressed spEV proteins in NS, OA, and NOA patients by PRM‐MS. (A) The quantification of spEV protein abundances by PRM. LC‐ESI, liquid chromatography‐electrospray ionization; Q1, first quadrupole; Q2, second quadrupole. (B) Multi‐step strategy for candidate biomarker screening. (C) Relative quantification of 19 differentially expressed proteins and two differentially expressed phosphorylated proteins, with two unchanged proteins, TENM2 and DNM1L, serving as the control proteins in 42 spEV samples from 10 NS, 23 NOA (4 HS, 3 MA, 16 SCO), and 9 OA patients using PRM. L‐light peptide, H‐heavy peptide; (phospho)protein levels are quantified as the PRM area ratio of L/H peptides. Horizontal lines represent the median value in each sample set. Proteins not detected by LC‐MS/MS are shown as the lowest value of 0.0001. (D) Relative abundances of SLC5A12, HIST1H2BA, and TEX101 in seminal plasma samples from eight NS, eight NOA (two HS, one MA, five SCO), and eight OA patients by PRM. (E) Immunoblots showing expression levels of SLC5A12 and HIST1H2BA in spEV samples from NS, NOA, and OA patients. The EV biomarker, ALIX, served as the control. ***p ≤ 0.001; **p ≤ 0.01; *p ≤ 0.05

Direct quantification of HIST1H2BA, SLC5A12, and the reported biomarker TEX101^7^ in seminal plasma is hampered due to the high abundance proteins^8^ (Figure 2D). Thus, SLC5A12 and HIST1H2BA are enriched in spEVs and can be good biomarkers.

To better understand the roles of SLC5A12 and HIST1H2BA in the diagnosis of the different types of azoospermia, we performed expression analysis of SLC5A12 and HIST1H2BA by western blotting and found consistent results with those of PRM (Figure 2E). Further immunolocalization analysis showed that SLC5A12 was mainly expressed in the lumen‐facing apical membrane of epithelial cells in the human epididymis and was not detected in the human testis (Figure 3A, Figure S4A and C). spEV SLC5A12 may be derived from SLC5A12‐positive epithelial cells. Thus, significant decrease of spEV SLC5A12 is expected in OA patients, but not NOA patients. HIST1H2BA was expressed in spermatogenic cells and was absent in testicular somatic cells in the human testis and absent in the epididymis (Figure 3B, Figure S4B and C). The physical obstruction (OA) and the absence of germ cells (SCO) in testes led to the undetectable level of HIST1H2BA in spEV samples. Because spermatogenic cells can be found in MA and HS testes, HIST1H2BA may be secreted by these spermatogenic cells, making the differential diagnosis of the SCO subtype possible.

**FIGURE 3:**
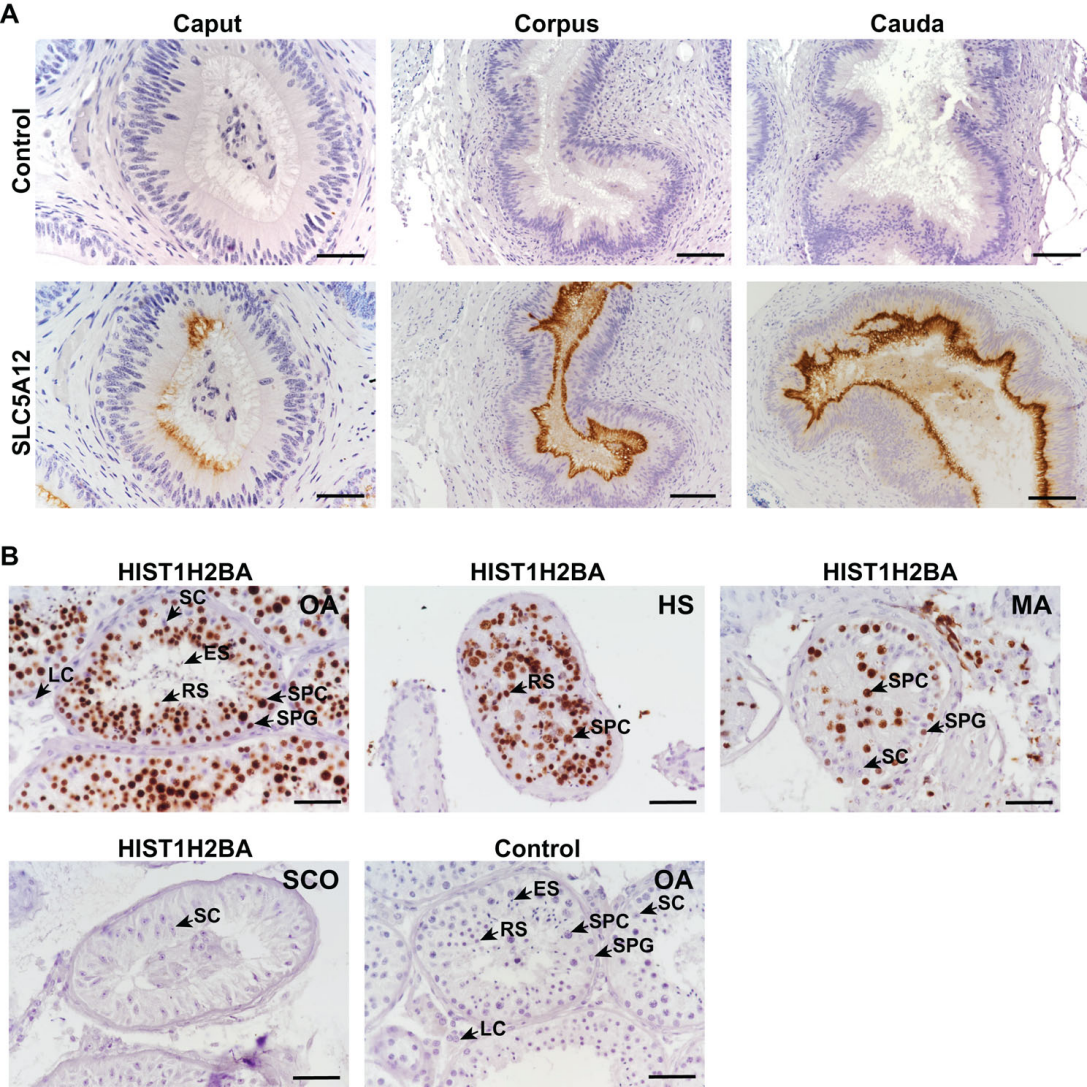
Immunolocalization of SLC5A12 and HIST1H2BA in the human testis and epididymis. (A) Immunohistochemical staining of SLC5A12 in the caput, corpus, and cauda epididymis with IgG serving as the negative control. Caput, scale bar = 50 μm; Corpus and cauda, scale bar = 100 μm. (B) Immunohistochemical staining of HIST1H2BA in the testis with active spermatogenesis (OA), NOA with decreased spermatogenesis (HS), NOA with germ cell arrest (MA), and NOA with Sertoli cell‐only (SCO), IgG serving as the negative control. Scale bar = 50 μm. Spermatogenic cells, including spermatogonia (SPG), spermatocytes (SPC), round spermatids (RS), and elongated spermatids (ES); testicular somatic cells, including Sertoli cells (SC) and Leydig cells (LC)

To evaluate the clinical value of diagnosis, we performed absolute quantification of SLC5A12 and HIST1H2BA using purified isotope‐labeled heavy synthetic peptides in 74 spEV samples by mass spectrometry (Table S1D). The limits of detection (0.00097 and 0.00094 μg/mg (spEVs)), limits of quantification (0.00243 and 0.00236 μg/mg (spEVs)), and linear response (*R* ^2^ = 0.9943 and *R* ^2^ = 0.9966) were obtained for SLC5A12 and HIST1H2BA by dilution series using isotope‐labeled heavy synthetic peptides (Figures S5 and S6). A cutoff value of 0.00243 μg/mg (spEVs) provided a specificity and sensitivity of 100% (area under the curve (AUC) of the receiver operating characteristic ROC = 1.00) for SLC5A12 to distinguish OA from NOA, and OA from NS (Figure 4A and B and Figure S7A). For HIST1H2BA, a cutoff value of 0.00236 μg/mg (spEVs) provided 100% specificity at 100% sensitivity for distinguishing OA from NS, and 81.25% specificity at 100% sensitivity (AUC = 0.906) for distinguishing SCO from HS and MA among NOA patients (Figure 4C and D and Figure S7B). Patients with SCO are not likely to undergo successful sperm retrieval by microdissection testicular sperm extraction (mTESE), while those without SCO are more likely to undergo successful sperm retrieval by mTESE for assisted reproductive technology treatment.^9^

**FIGURE 4:**
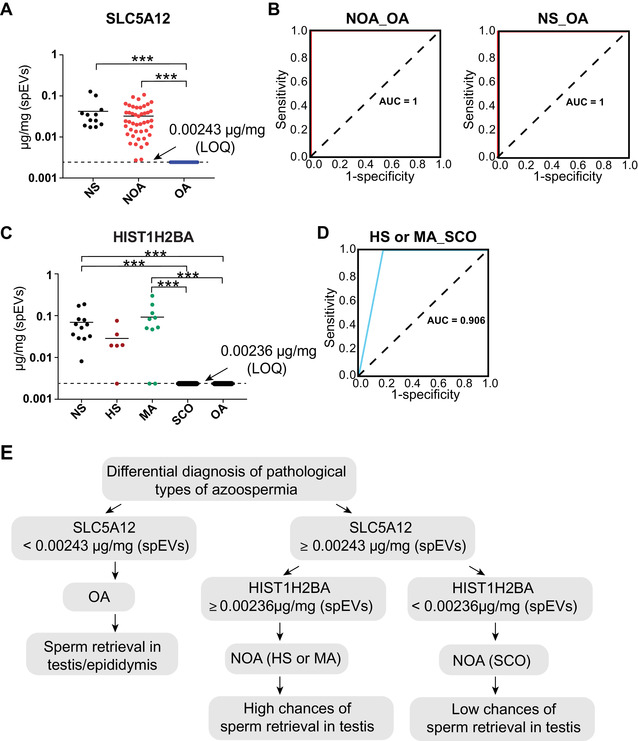
Evaluation of diagnostic values of SLC5A12 and HIST1H2BA in spEVs by PRM‐based absolute quantification. (A) Concentration of SLC5A12 in spEV samples from 12 NS, 45 NOA (6 HS, 10 MA, 29 SCO), and 17 OA patients, NS: 0.042 ± 0.0098 μg/mg (spEVs) (mean ± SEM); NOA: 0.032 ± 0.0037 μg/mg (spEVs) (mean ± SEM); OA: undetected (below LOQ). (B) ROC curves comparing the diagnostic power of SLC5A12 (solid red line) in spEV samples from NOA and OA patients, and in those from NS and OA patients. The dotted black line is the reference line. (C) Concentration of HIST1H2BA in spEV samples from 12 NS, 45 NOA (6 HS, 10 MA, 29 SCO), and 17 OA patients. NS: 0.069 ± 0.0156 μg/mg (spEVs) (mean ± SEM); NOA‐HS: 0.029 ± 0.0094 μg/mg (spEVs) (mean ± SEM); NOA‐MA: 0.093 ± 0.0272 μg/mg (spEVs) (mean ± SEM); NOA‐SCO and OA: undetected (below LOQ). (D) ROC curves comparing the diagnostic power of HIST1H2BA (solid blue line) in spEV samples from SCO and non‐SCO (HS or MA) patients. The dotted black line is the reference line. ***p ≤ 0.001. (E) Two spEV protein biomarker for noninvasive differential diagnosis of azoospermia (NOA vs. OA) and prediction of NOA subtypes

In conclusion, we developed two spEV protein biomarker for the noninvasive differential diagnosis of NOA and OA with 100% sensitivity and specificity, and for the differential diagnosis of the SCO subtype in NOA patients (Figure 4E), which can replace invasive diagnostic testicular biopsies and predict the presence of germ cells in the testis of NOA patients, thus increasing the success rate of surgical sperm extraction and reducing patient pain and costs.

## CONFLICT OF INTEREST

The authors declare no conflict of interest.

## DATA AVAILABILITY STATEMENT

The proteomics and phosphoproteomics data have been deposited in the ProteomeXchange Consortium via the proteomics identifications (PRIDE) database (Identifier PXD022843). Most data relevant to the study are included in the article or uploaded as the Supporting Information.

## Supporting information

Supporting Information

Supporting Information

Supporting Information

Supporting Information

Supporting Information

Supporting Information

Supporting Information

Supporting Information

Supporting Information
