# Supplementary material for: Quantitative proteomic biomarkers from extracellular vesicles of human seminal plasma in the differential diagnosis of azoospermia
Source: Clin Transl Med. 2021 May 28;11(5):e423. doi: 10.1002/ctm2.423 (PMC8161617; doi:10.1002/ctm2.423)
Supplement: Supplementary file 1 — Supporting Information [file CTM2-11-e423-s007.docx]

**MATERIALS AND METHODS**

**Patient information**

Prior to sample collection, approval was granted by the bioethics committees of Nanjing Medical University (Permission Number (2018)562) and the First Affiliated Hospital of Nanjing Medical University (Permission Number 2012-SR-128), and informed consent was obtained from all participants. Healthy individuals with normal sperm (NS) seminal plasma samples were obtained from healthy semen donors according to the 2010 guidelines of the World Health Organization.[^1^](#_ENREF_1) The absence of sperm in at least three semen analyses (centrifugation at 3,000 rpm for 15 min) was defined as azoospermia. In this study, nonobstructive azoospermia (NOA) patients included men with azoospermia by semen analysis that was confirmed by testicular biopsy or an elevated Follicle-stimulating hormone (FSH) level (>12 IU/L) accompanied by a low testicular volume (<12 mL).[^2^](#_ENREF_2)^,^ [^3^](#_ENREF_3) Patients with all known acquired (e.g., chemotherapy, bilateral cryptorchidism, testicular torsion) and genetic (karyotype aberrations, Y-chromosomal AZF deletions) causes of azoospermia were excluded. NOA subtypes were determined by hematoxylin and eosin (H&E)-stained multi-point puncture biopsy. Patients with a high number of sperm retrieved by testicular biopsy or percutaneous epididymal sperm aspiration (PESA) were enrolled in the obstructive azoospermia (OA) group, and those with congenital absence of vas deferens (CBAVD) were excluded. The discovery group consisted of 9 NS, 9 OA, and 9 NOA patients. Ten NS, 23 NOA (4 HS, 3 MA, 16 SCO), and 9 OA patients were verified by relative quantification, whereas 12 NS, 45 NOA (6 HS, 10 MA, 29 SCO), and 17 OA patients were verified by absolute targeted quantification using PRM. The relative levels of SLC5A12, HIST1H2BA and TEX101 in the seminal plasma were determined in 8 NS, 8 NOA (2 HS, 1 MA, 5 SCO), and 8 OA patients. Epididymal samples were from patients undergoing radical inguinal orchiectomy. The clinical data of all patients used to screen and validate protein candidates was summarized in Table S1.

**Isolation and identification of spEVs**

Semen samples were obtained by masturbation after 3–5 days of sexual abstinence. After liquefaction, the semen samples were centrifuged at 3,000 rpm for 15 min at 4°C, and the supernatants were stored at -80°C until further use. The isolation of seminal plasma extracellular vesicles (spEVs) was performed by differential centrifugation as previously described with minor modifications.[^4^](#_ENREF_4) In brief, the supernatants were sequentially centrifugated at 4,000 g for 30 min to obtain the seminal plasma fraction and at 20,000 g for 60 min at 4°C to obtain the microvesicle fraction, followed by centrifugation at 199,700 g for 90 min at 4°C using a Beckman SW60Ti rotor. The vesicles were resuspended in phosphate buffered saline (PBS) and centrifuged at 199,700 g for 90 min at 4°C, and the pellet of spEVs were collected and processed further.

The spEV concentration and size were determined using the NanoSight instrument (NTA, Malvern, NS300) according to the manufacturer’s instructions, and the spEV morphology was observed by transmission electron microscopy (TEM, TecnaiG2 Spirit 120 kV). In brief, spEVs were deposited on formvar-coated grids stabilized with evaporated carbon film and glow discharged before sample application. Neutral uranyl acetate (2% in AD) was used for staining, and the spEVs were then observed by TEM.

**Western blotting**

The spEVs or tissues were resuspended in 200 μL of protein lysis buffer (8 M Urea, 75 mM NaCl, 50 mM Tris, pH 8.2, 1% EDTA-free protease inhibitor, 1 mM NaF, 1 mM β-glycerophosphate, 1 mM sodium orthovanadate, 10 mM sodium pyrophosphate), clarified by spinning for 60 min at 10 rpm, and then centrifuged at 40,000 g for 60 min at 4°C. Equivalent amounts of protein (10 μg) were separated by SDS/PAGE and transferred onto polyvinylidene difluoride membranes. The primary antibodies were as follows: mouse anti-CD81 (1:1000, sc-166029, Santa Cruz Biotechnology), mouse anti-TSG101 (1:1000, ab83, Abcam), rabbit anti-ALIX (1:1000, 12422-1-AP, Proteintech), rabbit anti-Calnexin (1:1000, 10427-2-AP, Proteintech), mouse anti-SLC5A12 (1:1000, sc-515141, Santa Cruz Biotechnology), rabbit anti-HIST1H2BA (1:1000, ab178426, Abcam), and mouse anti-GAPDH (1:1000, 60004-1-Ig, Proteintech). The secondary antibodies were as follows: goat anti-mouse IgG (1:5000, 31430, Thermo Fisher Scientific) and goat anti-rabbit IgG (1:5000, 31460, Thermo Fisher Scientific).

**Protein sample preparation for TMT-based LC-MS/MS**

The spEV proteins were reduced, trypsin digested, and desalted using an OASIS HLB Vac cartridge (Waters, 186000383) as previously described.[^5^](#_ENREF_5) Purified peptides were reconstituted in 200 mM triethylammonium bicarbonate (TEAB) and labeled with tandem mass tag (TMT) 10-plex (A37725, Thermo Fisher Scientific) according to the manufacturer’s instructions.[^6^](#_ENREF_6) The labeled peptide samples were combined, purified using an OASIS HLB Vac cartridge, and lyophilized with a SpeedVac concentrator (Labconco). In total, three sets of TMT 10-plex experiments quantifying 9 NS, 9 NOA, and 9 OA samples were performed, with each TMT 10-plex labeling containing 3 NS, 3 NOA, and 3 OA samples, as well as a pooled sample which served as the internal standard (Figure S1, Table S1 A). Approximately 5% of each TMT sample set was used for proteomic analysis, and the remaining 95% of each TMT sample set was pooled for phosphoproteomic analysis due to the high sample amount required for analysis.

**High-pH reverse phase fractionation**

The TMT-labeled peptides were fractionated by high-pH reverse phase column as previously described[^7^](#_ENREF_7) using an XBridge BEH130 C18 column (300 μm × 150 mm, 1.7 μm, Waters) and the M-class HPLC system (Waters). Ten fractions were collected using a nonadjacent pooling scheme with a 73-min gradient of 3% buffer B (A: 20 mM ammonium formate, pH 10; B: 100% acetonitrile (ACN) for 14 min, 3%–8% B for 1 min, 8%–29% B for 24 min, 29%–41% B for 4 min, 41%–100% B for 1 min, 100% B for 8 min, and 100%–3% B for 1 min, followed by 20 min at 3% B), and the fractions were then dried.

For phosphoproteomic quantification, 1 mg of the TMT-labeled peptide mixture was fractionated using an XBridge BEH300 C18 column (10 × 250 mm, 5 μm, Waters) and the Agilent 1260 system. Five fractions were collected using a nonadjacent pooling scheme with a 22-min gradient of 0%–12% buffer B (5 mM ammonium formate/90% ACN, pH 10) for 1.7 min, 12%–32% B for 10.6 min, 32%–37% B for 0.7 min, 37%–48% B for 2.6 min, 48%–70% B for 2.4 min, followed by 4 min at 70% B. The fractions were then dried by vacuum concentration for further enrichment of the phosphopeptides.

**Phosphopeptide enrichment**

TMT-labeled phosphopeptides were enriched by immobilized metal affinity chromatography (Ti-IMAC, JK Chemical) as previously described.[^8^](#_ENREF_8) In brief, peptides were dissolved in loading buffer (80% ACN, 6% trifluoroacetic acid (TFA)) and incubated with IMAC beads for 30 min at room temperature, rinsed with wash buffers I (50% ACN, 200 mM NaCl, 6% TFA) and II (30% ACN, 0.1% TFA) for 30 min, and eluted with elution buffer (10% NH_4_OH) for 15 min. The phosphopeptide eluates were dried and desalted using C18 StageTips.

**TMT-based quantification by LC-MS/MS analysis**

For LC-MS/MS analysis, peptides were resuspended in 0.1% formic acid (FA) and analyzed using an Orbitrap Fusion Lumos mass spectrometer (Thermo Fisher Scientific) coupled to the Easy-nLC 1200 system (Thermo Fisher Scientific). The trap column (75 μm × 2 cm, Acclaim PepMap100 C18 column, 3 μm, 100 Å; DIONEX) effluent was transferred to a reverse-phase microcapillary column (75 μm × 25 cm, Acclaim PepMap RSLC C18 column, 2 μm, 100 Å; DIONEX). A 95-min linear gradient (3%–5% B for 5 sec, 5%–15% B for 40 min, 15%–28% B for 34 min and 50 sec, 28%–38% B for 12 min, 30%–100% B for 5 sec, and 100% B for 8 min) was applied while using the following buffer: 0.1% FA (buffer A) and 80% ACN, 0.1% FA (buffer B). The Orbitrap Fusion Lumos mass spectrometer was operated in the data-dependent mode. A full-survey scan was obtained for the m/z range of 350–1500, and the resolution of higher energy collisional dissociation (HCD) MS/MS was 50,000.

Raw files were searched against human protein sequences obtained from the Universal Protein Resource (UniProt) database (release 2018_07) using MaxQuant software (1.6.5.0).[^9^](#_ENREF_9) The FDR cut-off was set to 0.01 for proteins, peptides, and sites. Enzyme specificity was considered full cleavage by trypsin, and a maximum of two missed cleavage sites was permitted. Carbamidomethyl (C) on cysteine-fixed modifications, with TMT reagent adducts on lysine and peptide amino termini, were considered fixed modifications. Variable modifications included oxidation (M) and acetylation (protein N-term). A dynamic modification of +79.996 Da (phosphorylation) was observed on serine, threonine, and tyrosine residues. The corrected TMT reporter intensities were used for TMT-based quantification.

The protein expression levels were calculated by dividing each target protein value by the pooled internal standard value in the TMT 10-plex experiments, and then log2 converted. Phosphorylation sites were filtered by a localization probability >0.75, and only singly phosphorylated peptides were selected for downstream analysis. One-way analysis of variance (ANOVA), followed by Permutation-based correction, was performed using Perseus software.[^10^](#_ENREF_10) A protein was considered significant if it had a fold-change >2 and an FDR-*q* ≤0.05. A phosphorylation site was considered significant if it had a fold-change >1.5 and a p-value ≤0.05.

**Bioinformatics analysis**

Gene ontology enrichment analysis, including biological process (BP) and cellular component terms (CC), molecular function (MF), was carried out using the clusterProfiler R package,[^11^](#_ENREF_11) and an FDR-*q* ≤0.05 was considered significant. Heatmap analysis was performed using the ComplexHeatmap R package.[^12^](#_ENREF_12) Motif analysis of pS/T phosphorylation sites was carried out using the Motif-X package (https://motif-x.med.harvard.edu/).[^13^](#_ENREF_13) Protein distribution in tissues was determined by referring to the Human Protein Atlas (http://www.proteinatlas.org).[^14^](#_ENREF_14)

**Protein quantification by parallel reaction monitoring**

PRM was performed as previously described.[^15^](#_ENREF_15) In brief, crude isotope-labeled heavy synthetic peptides were used for relative targeted quantification, and the purified isotope-labeled heavy synthetic peptides (purity, >95%) were used for absolute targeted quantification. They were purchased from Synpeptide (Table S7). Each peptide sample (600 ng) was combined with the isotope-labeled peptides and separated by an analytical column (1.9 μm × 16 cm, 26350-3, Dr. Maisch) using a flow rate of 300 nL/min on an easy-nLC 1200 HPLC system (Thermo Fisher Scientific) and a 60-min gradient (3%–5% B for 5 sec, 5%–15% B for 23 min and 55 sec, 15%–28% B for 21 min, 28%–38% B for 7 min and 30 sec, 38%–100% B for 5 sec, and 100% B for 7 min and 25 sec) or a 30-min gradient (3%–5% B for 3 sec, 5%–15% B for 11 min and 56 sec, 15%–28% B for 10 min and 30 sec, 28%–38% B for 3 min and 45 sec, 38%–100% B for 3 sec, and 100% B for 3 min and 43 sec). Analysis was conducted using a scheduled method on the LTQ Fusion Lumos mass spectrometer with the following parameters: a higher-energy collision of 30 eV, an AGC target of 5.0E4, a maximal injection time of 54 ms, and a scan range (m/z) of 150–2,000.

PRM data were processed with Skyline Daily software.[^16^](#_ENREF_16) At least three transitions per precursor were used to quantify the targeted peptides in the samples. For relative quantification, the protein expression was calculated as the ratio of the endogenous to the heavy peptide, which was then added to the transition peak areas. For absolute quantification, the amount of the endogenous protein was calculated by its purified heavy-labeled peptide counterparts with known amounts. ANOVA was used for multi-group comparisons, and an FDR-*q* ≤0.05 was considered significant.

**Hematoxylin and eosin staining and immunostaining of testis and epididymis sections**

For H&E staining, testes and epididymis were fixed with modified Davidson's fluid fixative for 48 h. Tissues were then dehydrated with increasing concentrations of ethanol (70%, 80%, 90%, 100%), cleared in xylene, embedded in paraffin, and serially sectioned to generate 5-μm-thick sections. Sections were then deparaffinized, rehydrated, stained with H&E, dehydrated, and mounted.

For immunostaining, endogenous peroxidase activity was quenched by incubating the sections in 3% H_2_O_2_ (diluted with methanol) for 10 min at 37°C. Heat-induced antigen retrieval was carried out, and sections were blocked with 1% BSA for 2 h. Sections were incubated in primary antibodies overnight at 4°C as follows: mouse anti-SLC5A12 (1:100, sc-515141, Santa Cruz Biotechnology), rabbit anti-HIST1H2BA (1:200, ab178426, Abcam), mouse anti-IgG (1:500, 12-371, Sigma), and rabbit anti-IgG (1:500, 2729, Cell Signaling Technology). After incubation with goat anti-mouse IgG (1:500, 31430, Thermo Fisher Scientific) or goat anti-rabbit IgG (1:500, 31460, Thermo Fisher Scientific) secondary antibodies, immunoreactive sites were visualized with diaminobenzidine (ZLI-9018, SolelyBio) and mounted for bright-field microscopy (Ni-E, Nikon).

**REFERENCES**

**1.** Cooper TG, Noonan E, von Eckardstein S, et al. World Health Organization reference values for human semen characteristics. *Human reproduction update.* 2010;16:231-245.

**2.** Jungwirth A, Giwercman A, Tournaye H, et al. European Association of Urology guidelines on Male Infertility: the 2012 update. *European urology.* 2012;62:324-332.

**3.** Evaluation of the azoospermic male: a committee opinion. *Fertility and sterility.* 2018;109:777-782.

**4.** Andaluz Aguilar H, Iliuk AB, Chen IH, Tao WA. Sequential phosphoproteomics and N-glycoproteomics of plasma-derived extracellular vesicles. *Nature protocols.* 2020;15:161-180.

**5.** Wang J, Qi L, Huang S, et al. Quantitative phosphoproteomics analysis reveals a key role of insulin growth factor 1 receptor (IGF1R) tyrosine kinase in human sperm capacitation. *Molecular & cellular proteomics : MCP.* 2015;14:1104-1112.

**6.** Fan Y, Cheng Y, Li Y, et al. Phosphoproteomic Analysis of Neonatal Regenerative Myocardium Revealed Important Roles of Checkpoint Kinase 1 via Activating Mammalian Target of Rapamycin C1/Ribosomal Protein S6 Kinase b-1 Pathway. *Circulation.* 2020;141:1554-1569.

**7.** Hao P, Ren Y, Dutta B, Sze SK. Comparative evaluation of electrostatic repulsion-hydrophilic interaction chromatography (ERLIC) and high-pH reversed phase (Hp-RP) chromatography in profiling of rat kidney proteome. *Journal of proteomics.* 2013;82:254-262.

**8.** Zhou H, Ye M, Dong J, et al. Robust phosphoproteome enrichment using monodisperse microsphere-based immobilized titanium (IV) ion affinity chromatography. *Nature protocols.* 2013;8:461-480.

**9.** Tyanova S, Temu T, Cox J. The MaxQuant computational platform for mass spectrometry-based shotgun proteomics. *Nature protocols.* 2016;11:2301-2319.

**10.** Tyanova S, Temu T, Sinitcyn P. The Perseus computational platform for comprehensive analysis of (prote)omics data. 2016;13:731-740.

**11.** Yu G, Wang L-G, Han Y, He Q-Y. clusterProfiler: an R package for comparing biological themes among gene clusters. *OMICS.* 2012;16:284-287.

**12.** Gu Z, Eils R, Schlesner M. Complex heatmaps reveal patterns and correlations in multidimensional genomic data. *Bioinformatics.* 2016;32:2847-2849.

**13.** Schwartz D, Gygi SP. An iterative statistical approach to the identification of protein phosphorylation motifs from large-scale data sets. *Nat Biotechnol.* 2005;23:1391-1398.

**14.** Uhlén M, Fagerberg L, Hallström BM, et al. Proteomics. Tissue-based map of the human proteome. *Science.* 2015;347:1260419.

**15.** Peterson AC, Russell JD, Bailey DJ, Westphall MS, Coon JJ. Parallel reaction monitoring for high resolution and high mass accuracy quantitative, targeted proteomics. *Mol Cell Proteomics.* 2012;11:1475-1488.

**16.** MacLean B, Tomazela DM, Shulman N, et al. Skyline: an open source document editor for creating and analyzing targeted proteomics experiments. *Bioinformatics.* 2010;26:966-968.
