# Supplementary material for: Quantitative proteomic biomarkers from extracellular vesicles of human seminal plasma in the differential diagnosis of azoospermia
Source: Clin Transl Med. 2021 May 28;11(5):e423. doi: 10.1002/ctm2.423 (PMC8161617; doi:10.1002/ctm2.423)
Supplement: Supplementary file 2 — Supporting Information [file CTM2-11-e423-s008.pdf]

Supplementary Figures and Tables

Figures S1-S7

Supplementary Figure 1. TMT 10-plex-labelled LC-MS/MS workflow

Supplementary Figure 2. Motif analysis of phosphorylation sites identified in the spEVs phosphoproteome

Supplementary Figure 3. Validation of differential (phospho)proteins in different types of azoospermia by PRM-MS

Supplementary Figure 4. Expression of SLC5A12 and HIST1H2BA in human testis and epididymis

Supplementary Figure 5. Calibration curves used to establish LOD and LOQ values of SLC5A12 in PRM

Supplementary Figure 6. Calibration curves used to establish LOD and LOQ values of HIST1H2BA in PRM

Supplementary Figure 7. Extracted ion chromatograms of SLC5A12 and HIST1H2BA in spEV from NS, NOA, and OA patients by PRM-based absolute quantification measurements

Tables S1-S7

Supplementary Table 1. Clinical data of individuals used in this study.

Supplementary Table 2. Quantitative proteome of spEV from NS, NOA and OA patients.

Supplementary Table 3. Quantitative phosphoproteome of spEV from NS, NOA and OA patients.

Supplementary Table 4. Differential proteins and phosphoproteins of spEV from NS, NOA and OA patients.

Supplementary Table 5. Gene ontology analysis of differential proteins of spEV in cluster 2 and cluster 3 from NS, NOA and OA patients.

Supplementary Table 6. Gene ontology analysis of differential

phosphoproteins of spEV form NS, NOA and OA patients.

Supplementary Table 7. Crude or purified isotope-labeled heavy synthetic peptide sequences used for relative or absolute targeted quantification by PRM.

Supplementary Figure 1

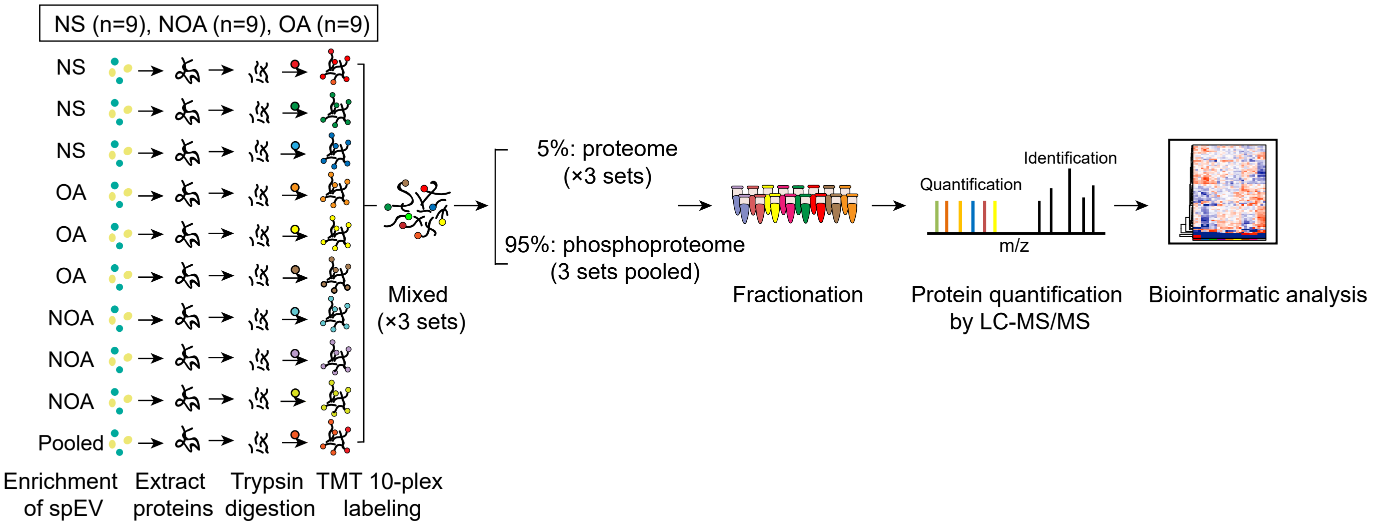

Supplementary Figure 1. TMT 10-plex-labelled LC-MS/MS workflow

The workflow using TMT 10-plex-labelled LC-MS/MS to analyze spEV samples from 9 NS, 9 NOA, and 9 OA patients.

NS, healthy individuals with normal sperm; NOA, nonobstructive azoospermia; and OA, obstructive azoospermia.

Supplementary Figure 2

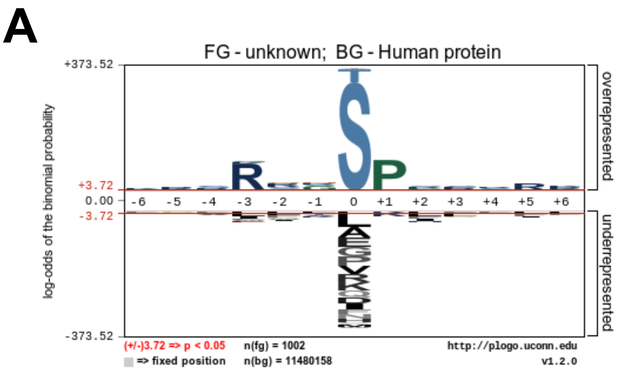

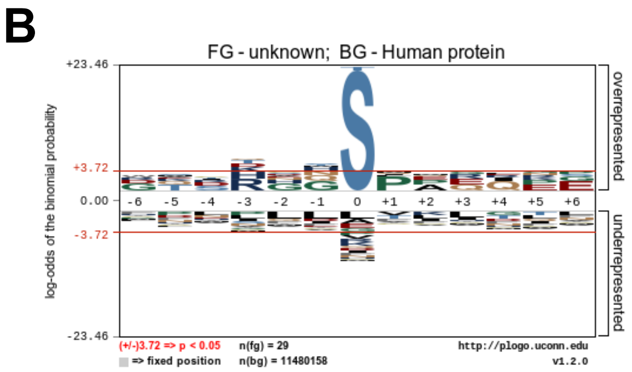

Supplementary Figure 2. Motif analysis of phosphorylation sites identified in the spEVs phosphoproteome

(A) Enrichment of amino acids by motif analysis surrounding phosphorylation sites identified in the spEV phosphoproteome.

(B) Enrichment of amino acids by motif analysis of differential phosphorylation sites in spEV from NS, OA, and NOA patients.

Supplementary Figure 3

A

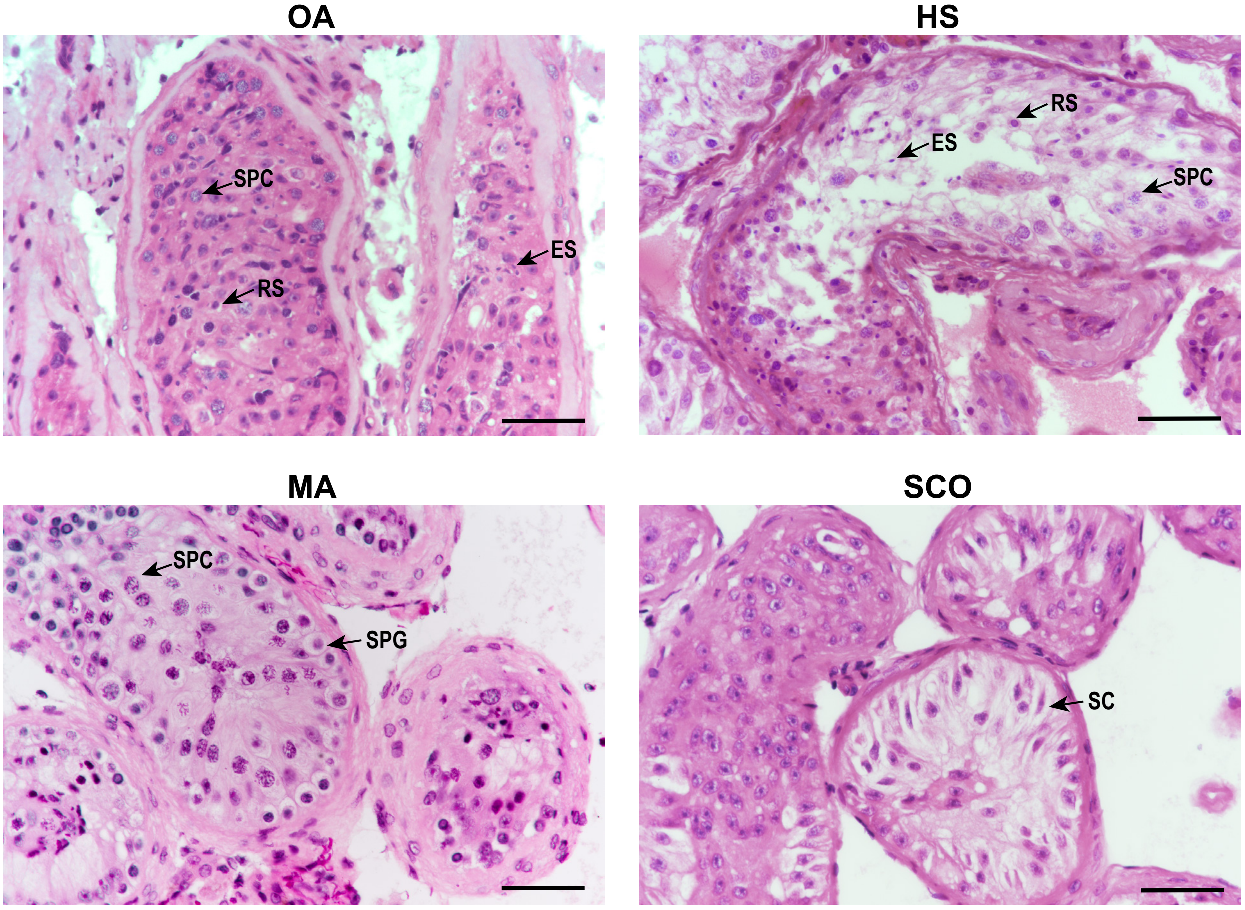

B

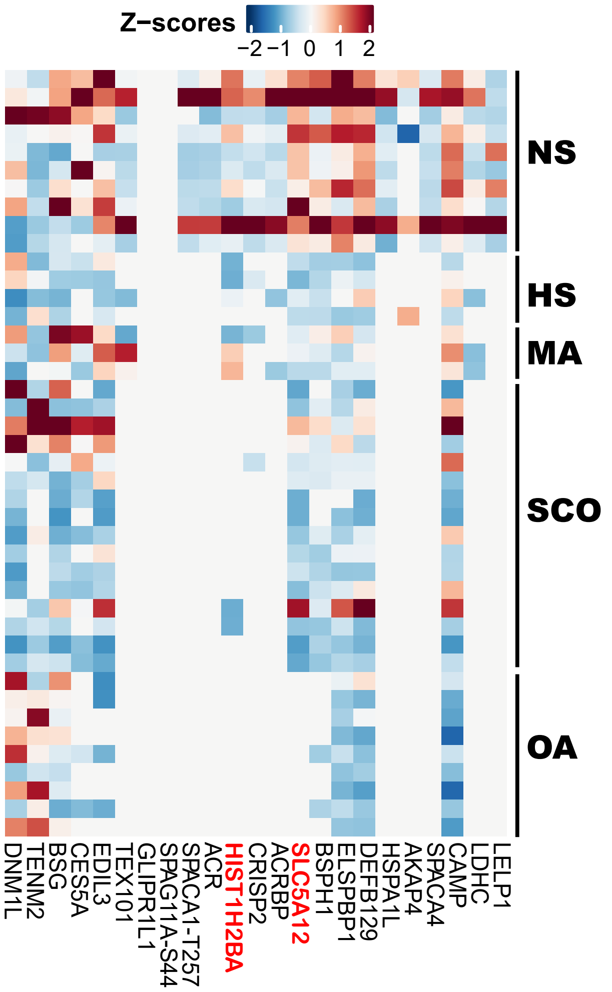

Supplementary Figure 3. Validation of differential (phospho)proteins in different types of azoospermia by PRM-MS

(A) H&E staining of OA, HS, MA, and SCO testes. Scale bar = 50 μm.

(B) Heatmap of Z-score transformed expression values of differential (phospho)proteins by PRM with undetected expression shown in white.

HS, hypospermatogenesis; MA, germ cell arrest; SCO, Sertoli cell–only syndrome.

Supplementary Figure 4

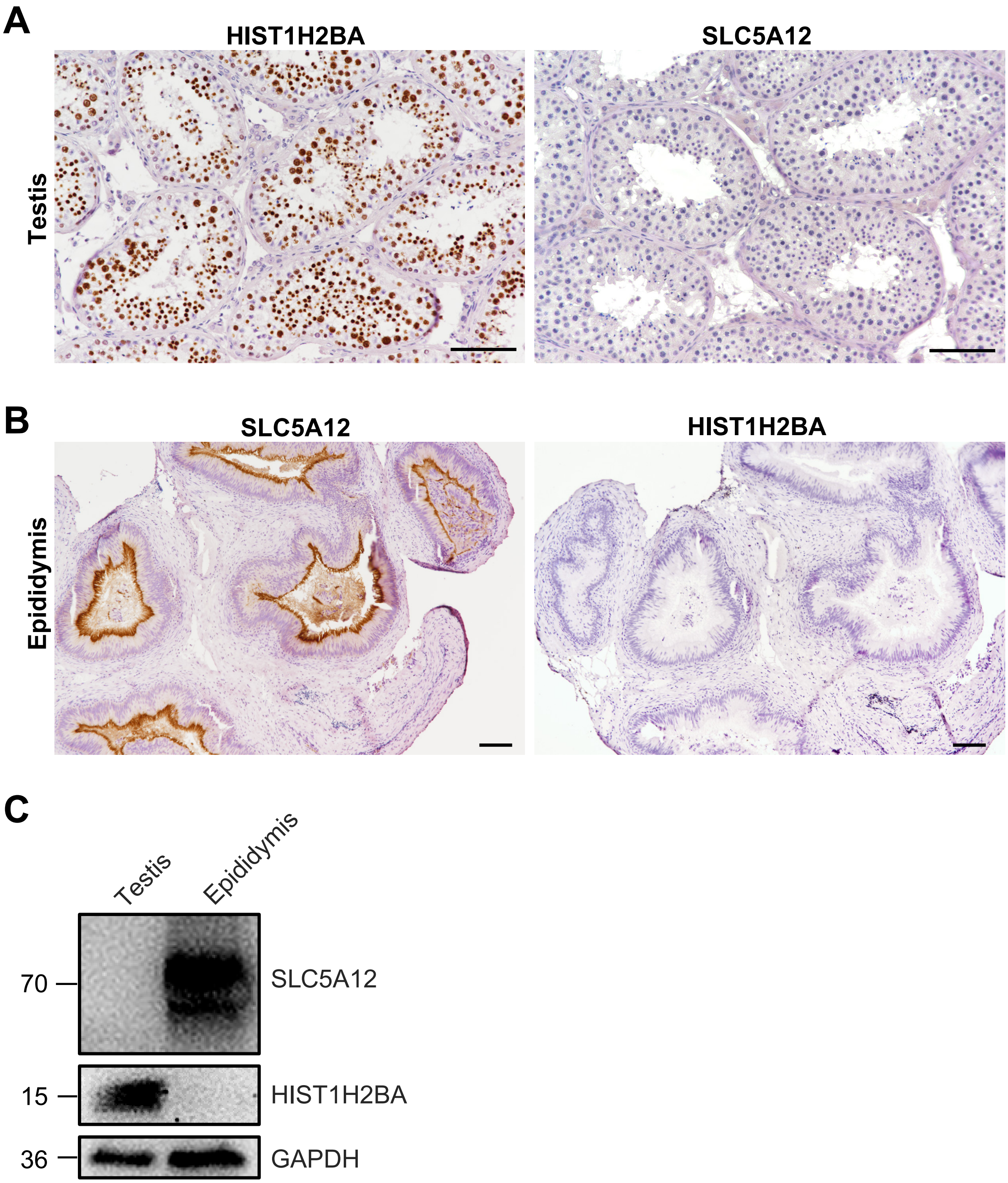

Supplementary Figure 4. Expression of SLC5A12 and HIST1H2BA in human testis and epididymis

(A-B) Immunohistochemical staining of SLC5A12 and HIST1H2BA in the testis (A) and epididymis (B) and Scale bar = 100 μm.

(C) Western blotting results of SLC5A12 and HIST1H2BA in the human testis and epididymis.

Supplementary Figure 5

A

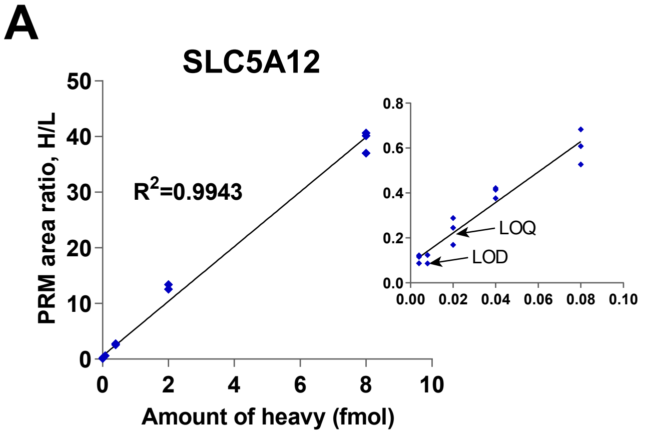

B

Protein	H/L	CV(%)	H (fmol)	MW(g/mol)	μg/mg(sEV)	
SLC5A12	0.122408	17.34967	0.004	72897	0.00049	
	0.123588	18.86749	0.008	72897	0.00097	LOD
	0.288334	25.77982	0.02	72897	0.00243	LOQ
	0.414319	6.001259	0.04	72897	0.00486	
	0.608347	12.87452	0.08	72897	0.00972	
	2.770851	4.592782	0.4	72897	0.04860	
	13.40483	3.540485	2	72897	0.24299	
	40.16064	4.989866	8	72897	0.97196	

C

SLC5A12: TWPLPLSTDQCI**K**($^{13}C_6$,$^{15}N_2$)

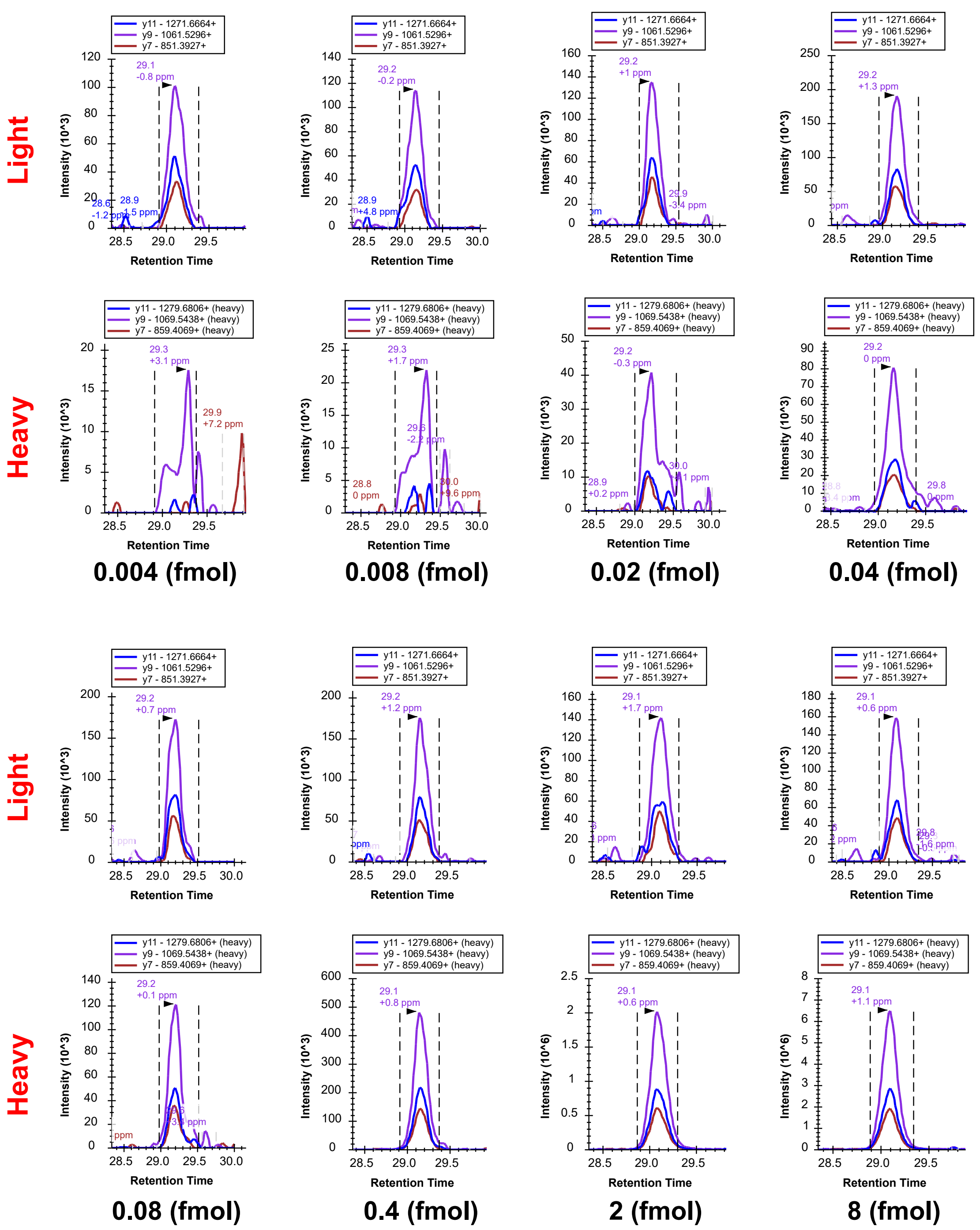

Supplementary Figure 5. Calibration curves used to establish LOD and LOQ values of SLC5A12 in PRM

(A-B) LOD and LOQ values for SLC5A12 were defined as the lowest concentrations of target proteins in the calibration curve at which the signal to noise ratio (S/N) of surrogate peptides was at least 3 and 10, respectively. Heavy to light peptide ratio (H/L) was used to generate the calibration curves and to evaluate reproducibility.

SLC5A12 had a calibration curve with an R^2 =0.9943 and LOD and LOQ values of 0.00097 µg/mg (spEVs) and 0.00243 µg/mg (spEVs), respectively.

(C) Representative extracted ion chromatograms of SLC5A12 in serial dilution experiments by PRM.

Supplementary Figure 6

A

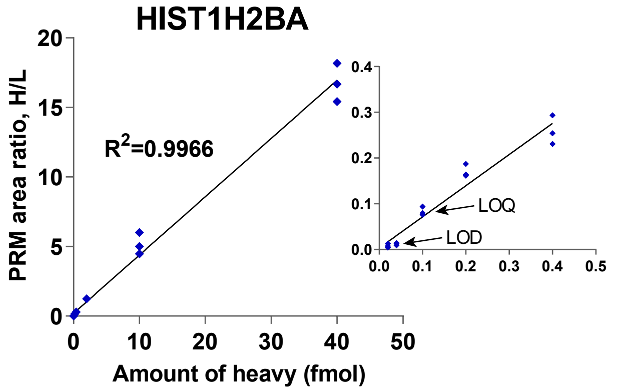

B

Protein	H/L	CV(%)	H (fmol)	MW(g/mol)	µg/mg(sEV)	
HIST1H2BA	0.004252	52.44084	0.02	14167	0.00047	
	0.009207	22.05115	0.04	14167	**0.00094**	LOD
	0.079926	11.24647	0.1	14167	**0.00236**	LOQ
	0.161384	8.292217	0.2	14167	0.00472	
	0.293393	12.17467	0.4	14167	0.00944	
	1.23793	1.25375	2	14167	0.04722	
	5.005005	15.02675	10	14167	0.23612	
	18.18182	8.217348	40	14167	0.94447	

C

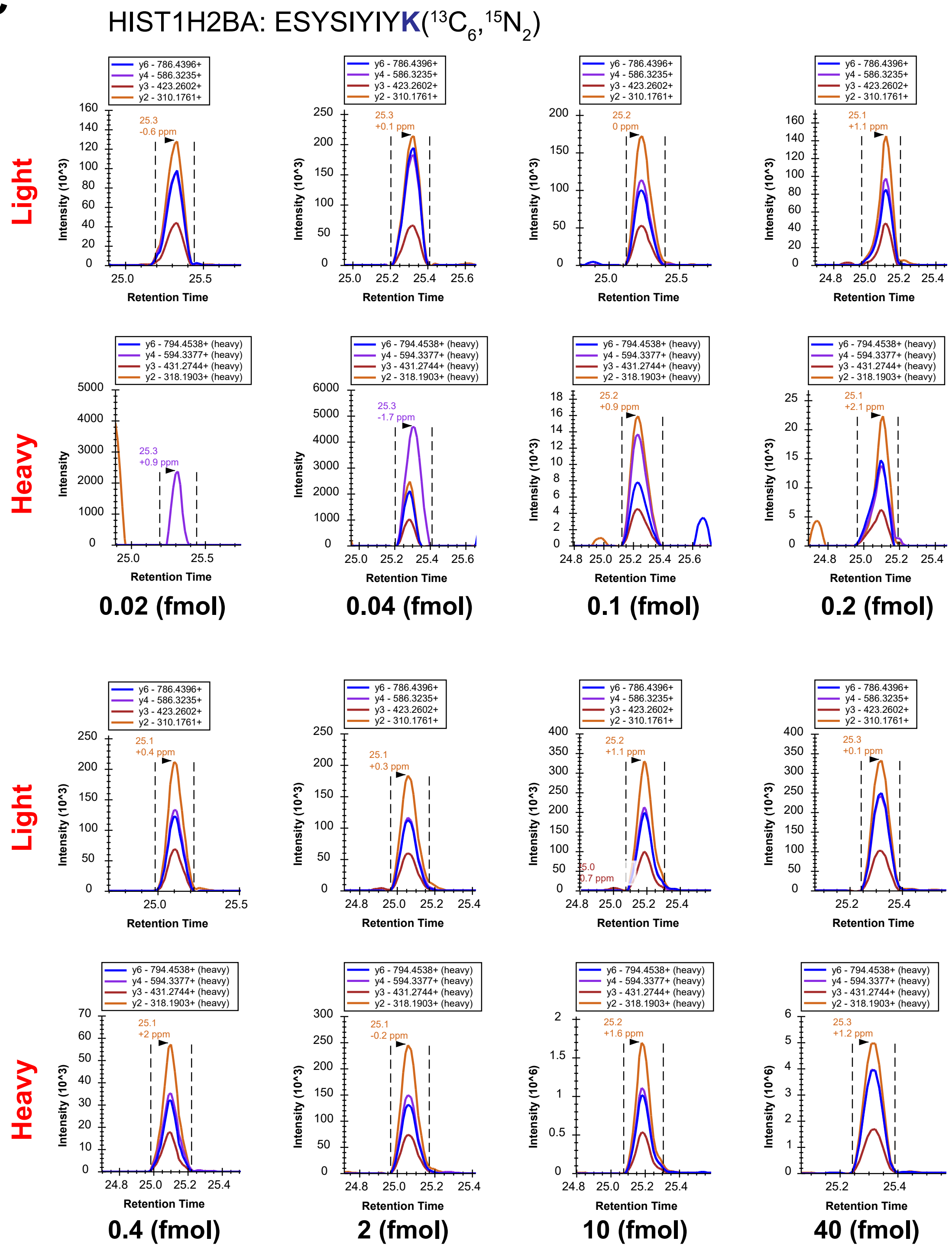

Supplementary Figure 6. Calibration curves used to establish LOD and LOQ values of HIST1H2BA in PRM

(A-B) LOD and LOQ values for HIST1H2BA were defined as the lowest concentrations of target proteins in the calibration curve at which the S/N of surrogate peptides was at least 3 and 10, respectively. H/L was used to generate the calibration curves and to evaluate reproducibility.

HIST1H2BA had a calibration curve with an R^2 =0.9966 and LOD and LOQ values of 0.00094 μg/mg (spEVs) and 0.00236 μg/mg (spEVs), respectively.

(C) Representative extracted ion chromatograms of HIST1H2BA in serial dilution experiments by PRM.

Supplementary Figure 7

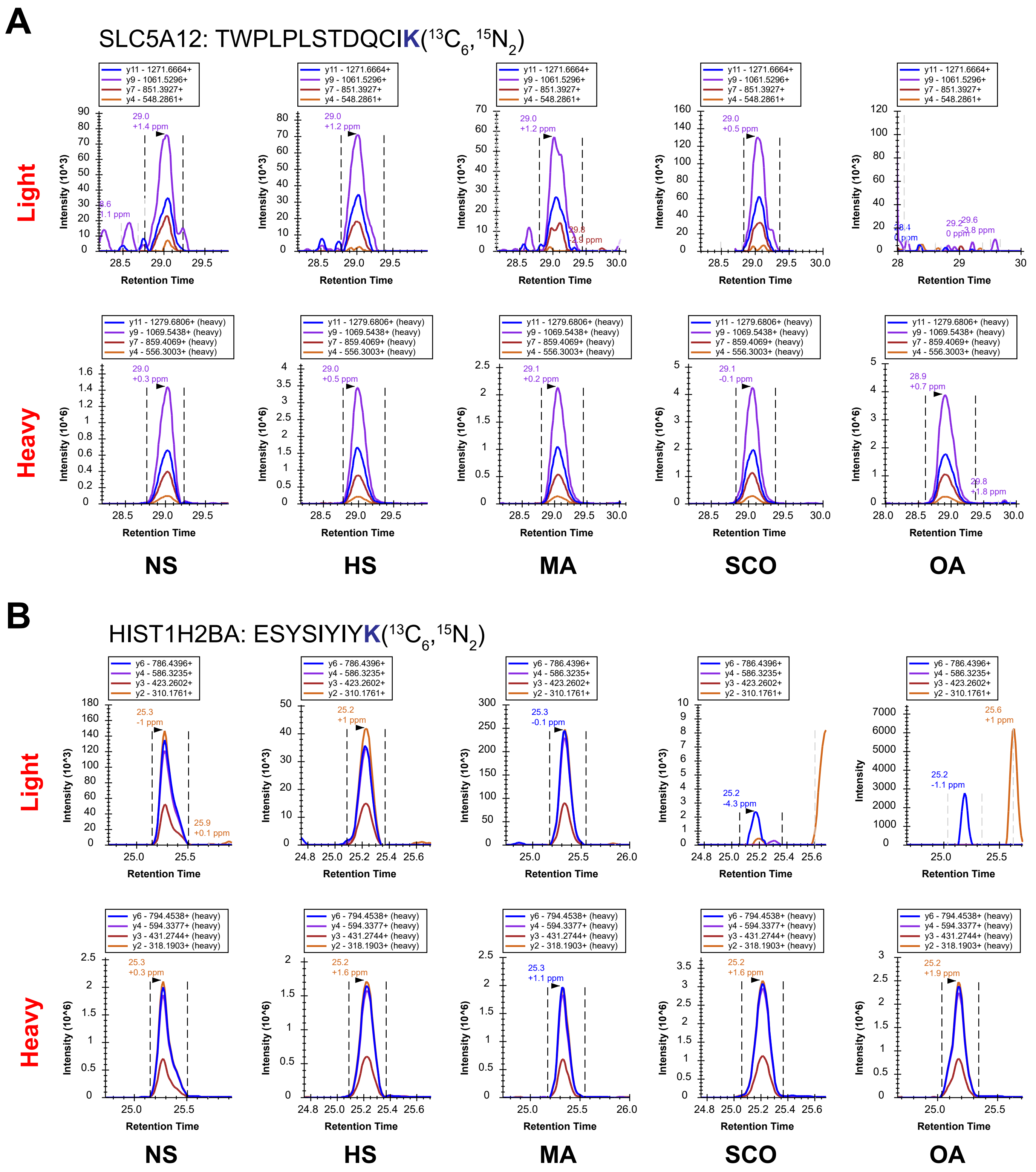

Supplementary Figure 7. Extracted ion chromatograms of SLC5A12 and HIST1H2BA in spEV from NS, NOA, and OA patients by PRM-based absolute quantification measurements

Representative extracted ion chromatograms of SLC5A12 (A) and HIST1H2BA (B) in spEV from NS, NOA (HS, MA, SCO), and OA patients.
