# Supplementary material for: Quantitative proteomic biomarkers from extracellular vesicles of human seminal plasma in the differential diagnosis of azoospermia
Source: Clin Transl Med. 2021 May 28;11(5):e423. doi: 10.1002/ctm2.423 (PMC8161617; doi:10.1002/ctm2.423)
Supplement: Supplementary file 3 — Supporting Information [file CTM2-11-e423-s004.pdf]

Supplementary Table 1A. The clinical data of patients enrolled in the spEV discovery group.

Patient group	Number of samples	Average age (years)	Average FSH (IU/L)	Average sperm concentration (millions/mL)	Average left testicular volume (mL)	Average right testicular volume (mL)	Y chromosome microdeletion status (AZFa, AZFb and AZFc regions)
NS	9	32 (27-38)		126.1			
NOA	9	27 (22-34)	21.1	0	7	7.1	negative
OA	9	28 (23-33)	3.5	0	15.6	15.6	4 patients-negative, 5 patients-N/A

Supplementary Table 1B. The clinical data of patients enrolled in the spEV relative quantification group.

Patient group	Number of samples	Average age (years)	Average FSH (IU/L)	Average sperm concentration (millions/mL)	Average left testicular volume (mL)	Average right testicular volume (mL)	Y chromosome microdeletion status (AZFa, AZFb and AZFc regions)
NS	10	32 (26-40)		148.9			
NOA	23 (4 HS, 3 MA,16 SCO	30 (24-39)	18.7	0	9.1	9.1	negative
OA	9	31 (26-38)	4.5	0	15.9	15.9	7 patients-negative, 2 patients-N/A

Supplementary Table 1C. The clinical data of patients enrolled in the validation using seminal plasma proteins.

Patient group	Number of samples	Average age (years)	Average FSH (IU/L)	Average sperm concentration (millions/mL)	Average left testicular volume (mL)	Average right testicular volume (mL)	Y chromosome microdeletion status (AZFa, AZFb and AZFc regions)
NS	8	32 (27-40)		138			
NOA	8 (2 HS, 1 MA, 5 SCO)	28 (25-33)	22.5	0	8.3	8.3	negative
OA	8	31 (26-38)	3.9	0	16.5	16.5	6 patients-negative, 2 patients-N/A

Supplementary Table 1D. The clinical data of patients enrolled in the spEV absolute quantification group.

Patient group	Number of samples	Average age (years)	Average FSH (IU/L)	Average sperm concentration (millions/mL)	Average left testicular volume (mL)	Average right testicular volume (mL)	Y chromosome microdeletion status (AZFa, AZFb and AZFc regions)
NS	12	32 (26-41)		142.5			
NOA	45 (6 HS, 10 MA,29 SCO)	29 (23-36)	19.4	0	8.7	8.6	negative
OA	17	31 (26-38)	4.5	0	14.4	14.7	15 patients-negative, 2 patients-N/A
