# Supplementary material for: Quantitative proteomic biomarkers from extracellular vesicles of human seminal plasma in the differential diagnosis of azoospermia
Source: Clin Transl Med. 2021 May 28;11(5):e423. doi: 10.1002/ctm2.423 (PMC8161617; doi:10.1002/ctm2.423)
Supplement: Supplementary file 4 — Supporting Information [file CTM2-11-e423-s001.pdf]

Supplementary Table 2. Quantitative proteomic analysis of spEV from NS, NOA and OA patients.

Id	UniprotID	Gene Symbol	Gene name	ENTREZID	ENSEMBL gene	Peptides	NS.1	NS.2	NS.3	NS.4	NS.5	NS.6	NS.7	NS.8	NS.9	NOA.1	NOA.2	NOA.3	NOA.4	NOA.5	NOA.6	NOA.7	NOA.8	NOA.9	OA.1	OA.2	OA.3	OA.4	OA.5	OA.6	OA.7	OA.8	OA.9	ANOVA FDR-*q*	Fold change (NS/OA)	Fold change (NS/NOA)	Fold change (NOA/OA)	ANOVA Significant	Differential proteins	Significant pairs
68	A0A087WY4	AC010330	epididymal sperm binding protein 1	64100	ENSG00000169393	11	0.446	0.111	-3.01	1.149	0.932	-0.16	0.197	0.293	0.902	-0.905	0.2421	0.6817	1.1713	-1.235	0.5968	-0.438	0.1402	-1.472	-2.02	-2.8	-2.56	-1.4	-1.61	-1.69	-1.69	-0.29	-1.97	0.021303	1.876267	0.230334351	1.645932741	+	Yes	NOA_OA; NS_OA
2566	Q1EHB4	SLC5A12	solute carrier family 5 member 12	159963	ENSG00000148942	3	0.253	-0.25	1.599	1.331	0.441	0.462	0.193	0.274	-0.39	-1.106	0.5331	0.9831	0.97	-1.749	0.9858	-0.647	0.1813	-0.504	-2.2	-2.35	-1.92	-1.7	-2	-2.53	-1.35	0.299	-1.39	0.001902	2.118535	0.473894462	1.644640696	+	Yes	NOA_OA; NS_OA
1596	P13796	LCP1	lymphocyte cytosolic protein 1	3936	ENSG00000136167	44	0.504	0.186	0.251	0.323	0.53	-0.05	0.282	0.272	0.317	-0.109	0.0525	0.1625	0.2388	0.111	0.0145	0.1014	-0.02	-0.105	-0.28	-1.1	-0.16	-0.51	-0.07	-0.17	-0.18	0.269	0.056	0.02	0.529974	0.241046347	0.288927281	+		
909	J3KNB4	CAMP	cathelicidin antimicrobial peptide	820	ENSG00000164047	15	0.023	-0.43	0.59	0.268	0.022	0.192	0.121	0.626	0.05	-0.441	0.8953	0.9356	0.761	-0.92	0.5965	0.143	0.6287	-0.642	-1.33	-2.32	-1.17	-0.41	-0.87	-1.86	-1.22	-0.55	-2.04	0.000958	1.470079	-0.054657346	1.524735966	+	Yes	NS_OA; NOA_OA
3360	Q9H1M3	DEFB129	defensin beta 129	140881	ENSG00000125903	9	0.762	0.125	0.828	1.232	0.964	0.247	-0.09	0.443	0.248	-1.053	0.7825	0.3094	0.6439	-2.063	0.7164	-1.055	0.1631	-1.496	-1.15	-1.41	-1.31	-1.53	-1.29	-2.39	-1.63	-1.21	-2.68	0.000926	2.151446	0.86794765	1.283498848	+	Yes	NOA_OA; NS_OA
1499	P08253	MMP2	matrix metallopeptidase 2	4313	ENSG00000087245	41	-0.08	-0.58	0.222	-0.44	-0.2	-0.23	-0.78	0.085	-0.45	0.1699	0.2902	-1	-0.756	-0.439	-1.042	-0.558	-0.084	-0.519	0.26	0.955	0.884	0.496	0.033	0.364	0.745	0.168	-0.37	0.018667	-0.66289	0.166820741	-0.829706974	+		
2332	Q075Z2	BSPH1	binder of sperm protein homolog 1	100131137	ENSG00000188334	5	0.965	-0.79	0.789	0.953	0.689	-0.07	-0.35	0.708	0.315	-1.508	-0.108	1.8344	0.5625	-1.111	0.2478	-0.872	-0.608	-1.131	-1.32	-1.78	-2.43	-1.05	-1.51	-1.67	-1.68	-0.05	-1.75	0.009058	1.82816	0.655248481	1.172911674	+	Yes	NOA_OA; NS_OA
1703	P21266	GSTM3	glutathione S-transferase mu 3	2947	ENSG00000134202	23	0.26	0.264	0.172	0.639	0.875	0.457	-0.42	0.043	0.043	0.0386	-0.27	0.2048	-0.056	-0.076	-0.183	-0.416	0.0286	-0.049	-0.43	-0.85	-0.13	-0.67	-0.22	-0.21	-0.05	-0.31	-0.48	0.012696	0.631357	0.345878507	0.285478403	+		
1142	O43854	EDIL3	EGF like repeats and discoidin domains 3	10085	ENSG00000164176	27	0.226	-0.43	0.934	0.158	0.445	0.156	-0.1	0.415	0.461	-0.981	0.5399	1.2921	0.786	-0.728	0.1428	-0.88	0.1498	-0.358	-1.51	-1.32	-1.46	-1.22	-1.13	-1.04	-1.17	-0.16	-1.07	0.001925	1.373458	0.256113354	1.117344898	+	Yes	NOA_OA; NS_OA
2849	Q8IWA5	SLC44A2	solute carrier family 44 member 2	57153	ENSG00000129353	19	-0.09	0.452	0.03	-0.31	-0.11	-0.23	-0.22	-0.16	-0	0.036	0.1612	0.1388	0.2001	0.0069	0.0588	-0.051	0.273	0.2111	-0.24	-0.13	-0.3	-0.36	-0.38	-0.04	-0.14	-0.1	-0.22	0.0226	0.138879	-0.187072807	0.325951594	+		
2342	Q08431	MFGE8	milk fat globule-EGF factor 8 protein	4240	ENSG00000140545	19	0.279	-0.36	0.596	0.131	0.172	-0.05	-0	0.296	0.343	-0.733	0.0502	0.3462	0.9037	-0.609	0.2433	-0.342	0.2795	-0.346	-0.6	-0.65	-0.47	-0.63	-0.44	-0.71	-0.47	-0.33	-0.87	0.016862	0.731511	0.179879311	0.551631648	+		
1987	P49223	SPINT3	serine peptidase inhibitor, Kunitz type 3	10816	ENSG00000101446	4	0.367	-0.37	0.384	1.352	0.667	0.03	0.603	1.593	0.764	-0.819	0.2808	0.404	0.6888	-0.931	0.0252	-0.161	-0.145	-1.354	-1.16	-1.39	-1.38	-0.89	-0.93	-1.8	-1.03	-0.58	-1.89	0.000294	1.827923	0.822353451	1.005569165	+	Yes	NOA_OA; NS_OA; NS_NOA
1761	P26641	EEF1G	eukaryotic translation elongation factor 1 gar	1937	ENSG00000254772	20	0.195	-0.27	0.108	1.439	0.991	0.084	0.273	0.409	0.577	-0.403	-0.517	-0.358	-0.392	-0.575	-0.342	-0.415	-0.147	-0.2	-0.39	-0.92	-0.42	-0.35	-0.47	-0.27	-0.14	-0.36	-0.3	0.001188	0.826503	0.795188195	0.031314504	+		
261	E5RFG9	CES5A	carboxylesterase 5A	221223	ENSG00000159398	12	-0.25	0.608	0.432	0.291	0.444	-0.15	-0.14	0.734	0.729	-0.076	0.3872	0.4174	1.0726	-0.552	0.245	-0.516	0.4645	-0.227	-1.07	-1.1	-0.99	-0.38	-0.49	-0.79	-0.6	-0.78	-0.86	0.000897	1.082338	0.1637174	0.918620827	+	Yes	NOA_OA; NS_OA
1059	O14964	HGS	hepatocyte growth factor-regulated tyrosine	9146	ENSG00000185359	22	0.091	0.177	-0.03	0.12	-0.08	-0.27	-0.1	0.002	0.11	0.0161	0.1493	0.2165	0.1392	-1E-04	0.1717	0.2002	0.1148	-0.004	-0.31	-0.5	-0.31	-0.37	-0.32	-0.02	-0.16	0.126	0.153	0.046702	0.191013	-0.109679347	0.300692445	+		
3321	Q9BYW3	DEFB126	defensin beta 126	81623	ENSG00000125788	9	1.008	0.009	1.112	1.3	0.796	0.12	0.765	1.024	0.341	-1.787	-3.377	0.8742	0.3082	-1.426	0.4808	-0.698	0.0458	-1.067	-2.28	-1.89	-2.28	-0.93	-1.42	-1.39	-1.48	-0.93	-2.05	0.001457	2.347948	1.457751204	0.890197256	+	Yes	NS_OA; NS_NOA
2650	Q5TFE4	NT5DC1	5'-nucleotidase domain containing 1	221294	ENSG00000178425	11	-0.13	0.125	0.143	-0.09	-0.08	-0.38	-0.17	-0.01	0.044	0.246	0.2051	0.151	0.047	0.1188	-0.087	0.0641	0.126	0.0994	-0.29	-0.58	-0.33	-0.36	-0.36	0.079	0.042	-0.1	-0.06	0.033843	0.157417	-0.168597484	0.326014743	+		
971	K7ES70	MFAP4	microfibril associated protein 4	4239	ENSG00000166482	4	-0.13	-0.36	0.534	0.669	0.358	-0.27	0.136	0.907	0.305	-0.631	0.3551	0.7095	0.192	-0.858	0.1288	-0.526	0.4042	-0.404	0.157	-0.69	-0.17	-2.08	-1.71	-1.79	-0.79	-0.05	-1.04	0.039208	1.143951	0.308804964	0.835145615	+	Yes	NOA_OA; NS_OA
1786	P29218	IMPA1	inositol monophosphatase 1	3612	ENSG00000133731	16	0.355	0.289	0.14	0.327	0.742	-0.04	-0.05	0.239	0.164	-0.222	-0.159	0.1519	-0.212	-0.237	-0.216	-0.201	-0.214	-0.172	-0.45	-0.72	-0.48	-0.59	-0.25	-0.32	-0.07	-0.1	-0.1	0.000885	0.583545	0.406059287	0.177485492	+		
3698	Q9Y230	RUVBL2	RuvB like AAA ATPase 2	10856	ENSG00000183207	22	0.505	0.132	0.208	0.89	1.334	0.036	0.178	0.685	0.056	-0.422	-0.473	-0.358	-0.408	-0.365	-0.409	-0.276	-0.358	-0.268	-0.47	-0.64	-0.43	-0.39	-0.52	-0.41	-0.08	-0.41	-0.4	0.0002	0.863755	0.817823878	0.045931211	+		
3442	Q9HCN3	TMEM8A	transmembrane protein 8A	58986	ENSG00000129925	19	-0.26	-0.08	0.318	0.223	0.175	-0.08	0.287	0.52	0.439	-0.107	0.0912	0.3987	0.3454	-0.092	0.1031	-0.062	-0.113	0.22	-0.79	-0.3	-0.28	-0.32	-0.04	-0.29	-0.22	0.116	-0.1	0.049417	0.4174	0.084613887	0.332785901	+		
2293	Q01813	PFKP	phosphofructokinase, platelet	5214	ENSG00000067057	28	0.465	0.127	-0.09	0.926	0.985	-0.09	0.493	0.776	0.561	-0.366	-0.283	-0.27	-0.152	-0.389	-0.426	-0.041	-0.19	-0.027	-0.28	-0.64	-0.44	-0.53	-0.66	-0.33	0.269	-0.19	-0.12	0.000967	0.785863	0.699765422	0.086097287	+		
484	C9J0K6	SRI	sorcin	6717	ENSG00000075142	17	-0.21	0.176	-0.06	0.081	0.06	-0.52	-0.13	-0.29	-0.21	0.1024	0.1183	-0.074	0.4572	-0.132	0.247	-0.013	0.0594	-0.076	-0.22	-0.22	-0.46	-0.42	-0.38	-0.08	-0.08	-0.2	-0.28	0.04666	0.1373	-0.199598876	0.336898428	+		
120	A0A087X2B	BSG	basigin (Ok blood group)	682	ENSG00000172270	6	0.376	0.005	1.213	0.654	0.708	0.163	-0.51	0.155	-0.55	-0.452	0.2722	0.3409	0.6966	-0.559	0.2806	-0.306	0.227	-0.486	-1.1	-1.48	-1.22	-0.47	-0.41	-0.44	-0.59	-0.33	-1.01	0.01277	1.029747	0.244363014	0.785384451	+	Yes	NOA_OA; NS_OA
425	B3GLJ2	PATE3	prostate and testis expressed 3	100169851	ENSG00000236027	7	0.223	-0.09	0.71	0.71	1.069	0.165	0.31	1.29	0.93	-0.979	0.1738	0.0471	0.0196	-0.723	0.2257	-0.534	0.2811	-0.798	-1.24	-0.91	-0.99	-0.98	-1.15	-1.53	-0.58	-0.53	-1.17	0	1.600863	0.845356713	0.755505878	+	Yes	NOA_OA; NS_OA; NS_NOA
1007	O00410	IPO5	importin 5	3843	ENSG00000065150	27	-0.02	0.125	0.079	0.319	0.446	0.045	0.343	0.299	0.273	0.0957	-0.037	-0.014	-0.085	-0.296	-0.029	0.1126	0.1022	0.1712	-0.28	-0.58	0.037	-0.2	-0.28	-0.08	-0.04	-0.03	0.237	0.039021	0.348389	0.210140156	0.138248428	+		
708	F5H2J1	MYO3B	myosin IIIB	140469	ENSG00000071909	3	0.185	-0.12	0.583	0.514	0.892	0.621				-0.922	-0.524	0.3262	0.5055	-0.577	-0.173				-1.57	-1.2	-0.94	-0.21	-0.72	-0.74				0.018882	1.341178	0.672751283	0.668426382	+	Yes	NS_OA
2424	Q13618	CUL3	cullin 3	8452	ENSG00000036257	26	0.28	0.189	-0.02	0.526	0.67	-0.01	0.216	0.367	0.302	-0.066	-0.033	-0.169	-0.218	-0.199	-0.176	-0.058	-0.102	-0.083	-0.31	-0.73	-0.31	-0.36	-0.48	-0.26	-0.03	-0.14	0.13	0.000893	0.556925	0.40279128	0.154133828	+		
1334	Q5T9B7	AK1	adenylate kinase 1	203	ENSG00000106992	16	0.146	0.227	0.384	0.701	0.761	-0.1	-0.01	0.712	0.379	-0.214	-0.178	-0.338	-0.685	-0.405	-0.147	-0.505	-0.211	-0.174	-0.6	-0.57	-0.13	-1.04	-0.6	-0.29	0.007	-0.37	-0.27	0.000545	0.782126	0.67234503	0.109780912	+		
231	A0A0G2JNE	SPAG11B	sperm associated antigen 11B	10407	ENSG00000274405	1	-0.55	0.335	-0.1	2.646	0.132	0.16	0.182	1.137	-0.34	-1.647	-0.738	0.5882	-0.353	-0.767	0.0852	-1.34	-0.666	-1.055	-1.72	-1.88	-1.53	-1.01	-1.19	-1.05	-1.1	-0.5	-0.77	0.012931	1.595128	1.054463379	0.540664376	+	Yes	NS_OA; NS_NOA
2558	Q16610	ECM1	extracellular matrix protein 1	1893	ENSG00000143369	31	0.496	-0.79	1.013	0.787	-0.07	0.513	0.3	0.841	0.251	-0.456	0.3461	0.7548	-0.244	-0.789	-0.484	-0.169	-0.319	-0.969	-0.57	-0.83	-0.3	-0.7	-0.23	-1.24	-0.5	-0.38	-1.19	0.018388	1.032245	0.630459328	0.401786124	+	Yes	NS_OA; NS_NOA
2275	P98160	HSPG2	heparan sulfate proteoglycan 2	3339	ENSG00000142798	39	-0.49	-0.64	-0.54	-0.27	-0.32	-0.55	-0.22	-0.04	-0.18	-0.26	-0.018	0.0435	-0.316	0.525	-0.166	0.0096	-0.237	0.198	-0.01	0.226	0.328	0.258	-0.36	0.222	0.426	-0	0.343	0.012883	-0.52018	-0.336649895	-0.183530643	+		
2603	Q5GAN3	RNASE13	ribonuclease A family member 13 (inactive)	440163	ENSG00000206150	3				0.888	1.053	0.22	0.849	1.556	1.302				0.0106	-0.496	0.3108	-0.64	-0.679	-0.478				-0.78	-0.86	-0.89	-0.34	0.052	-1.39	0.0015	1.678005	1.306677183	0.371327487	+	Yes	NS_OA; NS_NOA
2371	Q12974	PTP4A2	protein tyrosine phosphatase 4A2;protein tyr	8073	ENSG00000184007	9	-0.05	0.17	-0.02	0.016	0.027	-0.37	-0.19	-0.01	-0.19	0.1709	0.007	0.0153	0.0764	-0.299	0.1058	0.0477	0.288	0.0103	-0.37	-0.74	-0.43	-0.59	-0.41	-0.03	-0.35	-0.15	0.093	0.035901	0.263094	-0.115370547	0.378464786	+		
161	A0A0A0MT6	MUC15	mucin 15, cell surface associated	143662	ENSG00000169550	7	-0.21	-0.41	1.941	0.738	0.151	0.293	0.177	0.967	-0.23	-0.797	-0.087	0.2671	0.2677	-0.848	0.1205	-0.5	-0.103	-0.68	-1.03	-0.27	-0.85	-0.36	-0.97	-0.95	-0.59	-0.08	-0.58	0.040165	1.01133	0.642983099	0.368346725	+	Yes	NS_OA; NS_NOA
1908	P41222	PTGDS;AL	prostaglandin D2 synthase;novel transcript	5730	ENSG00000107317	5	0.655	-0.33	1.812	1.443	1.396	-0.17	0.873	1.666	-0.31	-0.872	-1.067	1.4018	-1.431	-2.738	-2.218	-1.071	-1.348	-0.899	-1.67	-1.21	-1.52	-1.93	-2.42	-2.44	-0.64	-0.14	-1.29	0.002119	2.251848	1.918756038	0.33309193	+	Yes	NS_OA; NS_NOA
113	A0A087X1X	EEF1D	eukaryotic translation elongation factor 1 del	1936	ENSG00000273594	12	0.184	0.251	-0.02	0.837	0.662	-0.15	0.281	0.295	0.388	0.0261	-0.073	0.0207	-0.091	-0.398	-0.039	0.2327	-0.056	0.0241	-0.21	-1.09	-0.13	-0.3	-0.42	-0.19	0.142	-0.15	-0.12	0.022141	0.577757	0.342549894	0.235207426	+		
1597	P13797	PLS3	plastin 3	5358	ENSG00000102024	21	0.342	-0.18	0.226	0.244	0.818	0.217	0.184	0.382	0.465	-0.661	-0.089	0.239	0.695	-0.39	0.1037	-0.175	0.0265	-0.217	-0.71	-0.74	-0.54	-0.34	-0.38	-0.45	-0.2	-0.36	-0.54	0.001605	0.774163	0.352015878	0.422146792	+		
2832	Q8IUA0	WFDC8	WAP four-disulfide core domain 8	90199	ENSG00000158901	16	0.928	0.145	1.096	0.957	1.249	0.461	0.348	1.327	0.855	-1.423	-0.395	-0.076	-0.242	-0.829	0.2504	-0.754	-0.473	-0.978	-1.49	-1.02	0.878	-0.86	-1.13	-1.52	-0.65	-0.62	-1.22	0.000313	1.666553	1.36491209	0.301640932	+	Yes	NS_OA; NS_NOA
1330	P00491	PNP	purine nucleoside phosphorylase	4860	ENSG00000198805	15	0.187	0.052	0.174	0.383	0.644	-0.4	0.234	0.277	0.255	-0.131	0.1312	0.3646	0.2365	-0.38	-0.207	-0.14	-0.209	-0.165	-0.38	-0.68	-0.07	-0.27	-0.41	-0.3	-0.13	-0.05	-0.15	0.03686	0.471095	0.255964304	0.2151311	+		
2632	Q5T123	SH3BGRL	SH3 domain binding glutamate rich protein l	83442	ENSG00000142669	6	-0.11	0.247	0.004	0.128	0.255	-0.58	0.257	0.214	0.221	0.1286	0.0843	0.1669	0.256	-0.202	0.1874	0.1148	0.2988	-0.034	-0.43	-1.17	-0.34	-0.57	-0.71	-0.13	-0.16	-0.03	-0.01	0.030897	0.465912	-0.040273943	0.506185929	+		
1197	O75264	SMIM24	small integral membrane protein 24	284422	ENSG00000095932	4	0.424	-0.04	1.046	0.214	0.118	0.136	-0	0.075	-0.08	-0.649	0.1609	0.8245	0.9202	-0.599	0.4142	-0.725	-0.042	-0.636	-0.81	-0.83	-0.86	-0.15	-0.57	-0.76	-0.9	-0.47	-1.18	0.020197	0.932288	0.246378529	0.685909042	+		
1773	P27824	CANX	calnexin	821	ENSG00000127022	8	0.232	0.218	0.064	0.152	0.497	-0.09	0.536	0.545	0.196	-0.462	-0.334	-0.305	-0.217	-0.094	-0.451	-0.266	-0.344	-0.382	-0.31	0.06	0.22	-0.02	-0.3	-0.26	0.165	-0.19	-0.35	0.000571	0.370157	0.5779248	-0.207767733	+		
1297	O95630	STAMBP	STAM binding protein	10617	ENSG00000124356	18	0.034	0.238	0.179	0.085	-0.07	-0.33	-0.01	0.449	0.13	-0.078	0.1483	0.2558	0.0391	0.1515	0.1415	0.0546	0.1327	0.0293	-0.21	-0.55	-0.25	-0.35	-0.23	-0.13	-0.18	-0.1	0.003	0.015875	0.298651	-0.019078725	0.317729899	+		
3704	Q9Y265	RUVBL1	RuvB like AAA ATPase 1	8607	ENSG00000175792	16	0.302	0.012	0.074	0.775	1.451	0.022	0.44	0.681	0.291	-0.581	-0.591	-0.574	-0.595	-0.35	-0.348	-0.226	-0.384	-0.503	-0.58	-1.03	-0.48	-0.49	-0.52	-0.28	0.2	-0.4	-0.49	0.000316	0.901965	0.911199055	-0.009233857	+		
1617	P15104	GLUL	glutamate-ammonia ligase	2752	ENSG00000135821	9	-0.11	-0.04	0.362	0.458	0.602	-0.06	2E-04	0.588	0.181	-0.567	-0.364	-0.266	-0.123	-0.576	-0.144	0.111	0.1094	-0.217	-0.78	-0.55	-0.07	-0.23	-0.32	-0.33	0.373	-0.51	-0.34	0.034056	0.52587	0.446379486	0.079490246	+		
3643	Q9UKG1	APPL1	adaptor protein, phosphotyrosine interacting	26060	ENSG00000157500	25	-0.04	0.049	-0.07	0.096	-0.01	-0.35	-0.21	-0	0.039	0.081	0.2123	0.1216	0.0331	0.104	0.0704	0.039	0.2411	0.0995	-0.2	-0.48	-0.1	-0.45	-0.29	-0.09	-0.12	0.023	0.089	0.027376	0.124741	-0.166687404	0.291427911	+		
1760	P26640	VARS;VAF	valyl-tRNA synthetase;valyl-tRNA synthetas	7407	ENSG00000231945	21	0.16	-0.05	0.09	1.336	1.35	0.083	0.174	0.365	0.235	-0.289	-0.289	-0.268	-0.342	-0.535	-0.485	-0.232	-0.226	-0.168	-0.41	-0.5	-0.29	-0.28	-0.44	-0.3	-0.13	-0.16	-0.22	0.002535	0.7181	0.730509433	-0.012409566	+		
1242	O75970	MPDZ	multiple PDZ domain crumbs cell polarity cc	8777	ENSG00000107186	18	0.033	0.322	-0.22	0.091	-0.17	0.009	-0.11	0.005	0.017	-0.181	0.0508	-0.112	-0.047	0.1685	0.3561	0.1057	0.2846	0.2118	-0.46	-0.71	-0.4	-0.38	-0.3	-0.26	-0.06	-0.11	0.112	0.028988	0.285133	-0.094583873	0.379717009	+		
3176	Q96PH6	DEFB118	defensin beta 118	117285	ENSG00000131068	3	0.548	-0.38	1.547	0.416	1.693	-1.91	1.42	2.26	0.66	-1.368	-0.816	0.2194	-1.322	-1.863	-1.083	-3.025	-1.932	-2.391	-1.59	-1.21	-1.53	-0.96	-1.68	-2.28	-1.64	-1.09	-3.16	0.001622	2.376643	2.203392102	0.173250745	+	Yes	NS_OA; NS_NOA
1307	O95757	HSPA4L	heat shock protein family A (Hsp70) membe	22824	ENSG00000164070	26	0.275	0.006	0.175	0.702	0.957	0.005	0.639	0.939	0.241	-0.35	-0.458	-0.552	-0.342	-0.336	0.1118	0.0934	0.0405	-0.223	-0.59	-0.71	-0.45	-0.27	-0.62	-0.24	-0.07	-0.42	-0.23	0.00055	0.838011	0.661601799	0.176409598	+		
945	K7EIU8	SMAD4	SMAD family member 4	4089	ENSG00000141646	9	0.222	0.197	-0.05	0.201	-0.21	-0.18	-0.11	-0.2	-0.02	0.0954	0.1683	0.257	-0.096	-0.093	0.0617	-0.218	0.0414	-0.098	-0.36	-0.93	-0.46	-0.46	-0.46	-0.22	0.093	-0.21	-0.14	0.041949	0.334006	-0.029985196	0.363990782	+		
1838	P33121	ACSL1	acyl-CoA synthetase long chain family mem	2180	ENSG00000151726	9	0.406	0.143	0.119	0.696	0.748	-0.12	0.41	0.576	0.42	-0.135	-0.29	-0.172	-0.277	-0.473	-0.279	-0.197	-0.231	-0.208	-0.55	-0.68	-0.25	0.131	-0.43	-0.33	0.233	-0.16	-0.11	0.00092	0.616877	0.629234345	-0.01235726	+		
54	A0A087WW	SH3GLB1	SH3 domain containing GRB2 like, endophil	51100	ENSG00000097033	11	0.253	0.204	0.161	0.351	0.624	-0.33	0.181	0.189	0.408	0.1841	0.1553	0.2078	-0.072	-0.087	0.1763	0.0031	-0.045	-0.097	-0.18	-0.7	-0.15	-0.62	-0.21	0.037	-0.12	-0.15	0.062	0.026159	0.451738	0.179799017	0.271939242	+		
2608	Q5J5C9	DEFB121	defensin beta 121	245934	ENSG00000204548	3	-0.09	0.23	0.947				-0.26	1.384	0.196	-0.953	-0.894	-0.322				-1.264	-1.405	-1.379	-0.89	-0.67	-1.24				-1.3	-1.02	-1.58	0.002733	1.516245	1.436141079	0.080104103	+	Yes	NS_OA; NS_NOA
603	E7EP66	ACRBP	acrosin binding protein	84519	ENSG00000111644	5	1.558	-0.63	0.309	1.164	0.712	0.086	0.177	1.848	0.377	-1.12	-0.556	-1.33	-1.023	-0.476	-0.705	-0.634	-0.251	-0.128	-1	-1.03	-1.17	-0.65	-0.9	-0.97	0.069	-0.36	-0.72	0.001212	1.370045	1.313602587	0.056442546	+	Yes	NS_OA; NS_NOA
1464	P07205	PGK2	phosphoglycerate kinase 2	5232	ENSG00000170950	22	1.209	0.447	0.575	1.628	2.145	0.19	0.536	0.823	0.938	-1.324	-1.275	-1.33	-1.04	-0.942	-1.008	-0.932	-1.288	-1.1	-1.51	-1.62	-1.64	-0.61	-1.14	-0.88	-0.88	-1.01	-1.23	0	2.111579	2.081253946	0.03032534	+	Yes	NS_OA; NS_NOA
3512	Q9NUP9	LIN7C	lin-7 homolog C, crumbs cell polarity comple	55327	ENSG00000148943	10	-0.01	0.256	-0.17	0.348	-0.17	-0.28	-0.35	-0.42	-0.31	-0.043	0.3276	0.1932	0.2011	-0.063	0.1544	0.0243	0.1757	-0.034	-0.41	-0.29	-0.46	-0.39	-0.35	-0.05	-0.19	-0.26	-0.18	0.022247	0.163811	-0.227807867	0.39161886	+		
1735	P23634	ATP2B4	ATPase plasma membrane Ca2+ transporti	493	ENSG00000058668	5	1.288	0.428	0.926	1.208	1.798	0.423				-1.472	-1.698	-1.659	-0.271	-0.325	-0.331				-2.06	-1.3	-1.69	-0.25	0.011	-0.58				0.007804	1.990968	1.971444587	0.019523398	+	Yes	NS_OA; NS_NOA
2704	Q6P597	KLC3	kinesin light chain 3	147700	ENSG00000104892	12	0.084	0.137	-0.14	-0.13	-0.04	-0.27	0.013	-0.07	0.069	0.1807	0.4031	0.2912	-0.156	0.3142	0.3139	0.1107	0.6031	-0.084	-0.41	-1.16	-0.16	-0.64	-0.89	-0.16	0.055	0.032	0.147	0.04598	0.315111	-0.258272745	0.57338365	+		
3309	Q9BY14	TEX101	testis expressed 101	83639	ENSG00000131126	2	0.95	-0.42	0.744	1.556	1.401	0.054	0.266	3.343	-0.67	-2.043	-2.066	-2.537	-1.782	-1.142	-1.57	-0.941	-2.197	-1.452	-2.38	-2.47	-2.44	-1.65	-1.08	-2	-0.36	-1.49	-1.66	0.00025	2.529022	2.550706565	-0.021685008	+	Yes	NS_NOA; NS_OA
2886	Q8N104	DEFB106A	defensin beta 106A;defensin beta 106B	245909;503	ENSG00000186579	1	1.032	0.693	0.959	2.224	1.184	0.739	0.167	1.592	0.046	-1.38	-0.523	-0.387	-0.55	-1.057	0.2072	-2.381	-1.973	-2.274	-1.13	-0.71	-1.19	-0.67	-0.74	-1.03	-1.34	-0.5	-2.67	0.000267	2.069114	2.106005441	-0.03689174	+	Yes	NS_NOA; NS_OA
2943	Q8NEX6	WFDC11	WAP four-disulfide core domain 11	259239	ENSG00000180083	1				1.322	0.598	1.586	0.731	0.303	2.545				-2.906	-2.815	-0.495	-1.435	-1.519	-1.468				-2.07	-1.66	-3.58	-1.28	-0.24	-1.56	0.002872	2.913107	2.953720391	-0.040612943	+	Yes	NS_NOA; NS_OA
3032	Q92526	CCT6B	chaperonin containing TCP1 subunit 6B	10693	ENSG00000132141	9	0.488	-0.48	0.168	2.796	0.089	-0.28	0.701	0.962	-0.07	-0.634	-0.73	-0.183	-0.738	-1.055	-0.514	-0.316	-0.873	-0.675	-0.71	-0.59	-0.77	-0.98	-0.85	-1	0.258	-0.45	-0.22	0.027512	1.076055	1.121435047	-0.045379624	+	Yes	NS_NOA; NS_OA
2018	P50238	CRIP1;AL	cysteine rich protein 1;novel protein	1396	ENSG00000213145	3	-0	-0.02	0.845	0.125	0.456	0.12	-0.34	-0.05	-0.2	-0.801	0.1181	0.009	-0.106	-0.379	0.1862	-0.617	0.1134	-0.006	-1.21	-0.13	-0.42	-0.4	-0.64	-0.66	-0.51	-0.77	-1.05	0.014286	0.747249	0.268145351	0.479103262	+		
3478	Q9NRA1	PDGFC	platelet derived growth factor C	56034	ENSG00000145431	3	-0.37	-0.62	-0.33	-0.24	0.073	-0.26	-0.02	-0.09	0.057	0.1141	0.0468	0.1186	0.0702	0.0937	-0.027	0.3065	0.1381	-0.007	-0.14	0.126	0.238	0.067	0.338	-0	-0.06	0.07	-0.08	0.039316	-0.26202	-0.294971163	0.032946912	+		
2787	Q7Z4H3	HDDC2	HD domain containing 2	51020	ENSG00000111906	8	-0.16	-0.22	-0.38	0.866	1.144	-0.17	0.253	0.788	0.385	-0.755	-0.569	-0.214	-0.6	-0.451	-0.511	-0.215	-0.159	-0.192	-0.82	-0.7	-0.8	-0.56	-0.52	-0.45	-0.36	-0.06	-0.23	0.010278	0.778522	0.685753264	0.092768459	+		
2238	P63261	ACTG1	actin gamma 1	71	ENSG00000184009	33	0.32	0.45	0.122	0.036	0.591	-0.3	0.087	0.175	0.337	0.0746	-0.095	-0.033	-0.078	-0.387	-0.331	0.1045	0.2048	0.2374	-0.35	-1.11	-0.2	-0.5	-0.49	-0.18	-0.21	0.022	0.054	0.045574	0.530604	0.235639906	0.294964261	+		
2844	Q8IVL8	CPO	carboxypeptidase O	130749	ENSG00000144410	7	-0.18	-0.47	0.111	-0.29	-0.33	-0.07	-0.46	-0.2	0.025	0.3793	-0.076	0.3739	0.1627	-0.456	-0.006	0.0372	0.7793	-0.119	-0.12	0.028	-0.37	-1.01	-0.76	-0.8	-0.41	-0.52	-0.58	0.02262	0.298424	-0.326452894	0.624876432	+		
3462	Q9NQ48	LZTFL1	leucine zipper transcription factor like 1	54585	ENSG00000163818	10	0.255	0.043	0.21	0.054	0.561	-0.18	0.227	0.414	0.287	-0.074	0.0234	-0.065	-0.134	-0.059	-0.026	-0.067	0.2578	-0.049	-0.14	-0.47	-0.16	-0.4	-0.34	-0.14	0.013	0.088	-0.01	0.01697	0.3821	0.229633671	0.152466719	+		
1641	P16562	CRISP2	cysteine rich secretory protein 2	7180	ENSG00000124490	3	0.778	-0.31	0.335	1.31	1.987	0.537	0.448	1.142	0.839	-0.365	-1.04	-0.433	-0.908	-0.429	-1.277	-0.908	-0.937	-1.067	-0.77	-1.02	-1.51	-0.6	0.031	-1.45	-0.05	-0.82	-0.75	0.000182	1.557569	1.604013138	-0.046444618	+	Yes	NS_NOA; NS_OA
2009	P49841	GSK3B	glycogen synthase kinase 3 beta	2932	ENSG00000082701	6	0.261	0.121	0.266	-0.23	-0.35	-0.41	0.266	0.554	0.119	-0.147	0.4049	0.1901	-0.128	-0.42	0.0044	-0.198	0.6524	-0.316	-0.59	-0.89	-0.72	-0.7	-0.58	-0.36	-1.04	-0.14	-0.74	0.003833	0.708004	0.0618676	0.646136536	+		

2066	P52943	CRIP2	cysteine rich protein 2	1397	ENSG00000182809	6	-0.36	-0.06	-0.02	-0.12	0.23	0.053	-0.16	-0.36	-0.28	0.2074	0.0126	0.0468	0.2555	-0.199	0.3565	-0.021	0.1278	-0.325	-0.46	-0.56	-0.28	-0.58	-0.33	-0.18	0.006	-0.24	-0.3	0.045534	0.205007	-0.170401334	0.375408645	+		
496	D6RA85	ROPN1B	rhophilin associated tail protein 1B	152015	ENSG00000114547	1				1.343	1.964	0.341							-1.02	-1.132	-1.153							-0.74	-1.1	-1.27				0.040582	2.25429	2.317740778	-0.063450754	+	Yes	NS_NOA; NS_OA
274	A0A1B0GUI	ESPN	espin	83715	ENSG00000187017	4	0.189	0.166	0.561	0.396	0.838	0.739	-0.05	0.149	0.168	-0.697	-0.106	0.3343	0.4466	-0.393	0.0385	-0.33	-0.105	-0.095	-0.92	-0.72	-0.7	-0.58	-0.68	-0.94	0.05	-0.08	-0.15	0.002795	0.874615	0.451559616	0.423055664	+		
1671	P19367	HK1	hexokinase 1	3098	ENSG00000156515	25	0.895	0.232	0.248	1.128	1.942	0.143	0.519	1.287	0.507	-0.842	-0.953	-0.926	-0.846	-0.685	-0.7	-0.716	-0.949	-0.813	-0.86	-0.93	-0.92	-0.38	-0.94	-0.9	-0.33	-0.65	-0.94	0	1.528031	1.592393776	-0.064363182	+	Yes	NS_NOA; NS_OA
2107	P55064	AQP5	aquaporin 5	362	ENSG00000161798	2	0.909	0.002	0.147	1.707	2.605	0.295				-1.072	-1.504	-1.138	-1.18	-1.055	-1.794				-1.33	-0.74	-1.38	-0.75	-1.23	-1.78				0.001528	2.144377	2.234478574	-0.090101818	+	Yes	NS_NOA; NS_OA
2093	P54709	ATP1B3	ATPase Na+/K+ transporting subunit beta 3	483	ENSG00000069849	4	0.962	0.317	0.407	0.839	1.019	-0.1				-0.878	-0.721	-0.904	-0.317	-0.274	-0.483				-1.06	-0.45	-0.67	-0.38	-0.07	-0.26				0.002717	1.05659	1.17003075	-0.113440288	+	Yes	NS_NOA; NS_OA
2367	Q12907	LMAN2	lectin, mannose binding 2	10960	ENSG00000169223	9	0.188	0.3	0.12	0.829	1.351	0.332	0.097	-0.05	0.138	-0.087	-0.038	-0.084	-0.004	-0.262	-0.317	-0.161	0.0002	-0.117	-0.44	-0.3	-0.28	-0.2	-0.11	-0.25	-0.15	-0.11	0.043	0.013088	0.566879	0.485755116	0.081124095	+		
2678	Q6BCY4	CYB5R2	cytochrome b5 reductase 2	51700	ENSG00000166394	8				1.504	1.776	0.293	0.567	1.116	0.382				-0.658	-0.559	-0.577	-0.556	-0.769	-0.651				-0.61	-0.6	-0.7	-0.21	-0.57	-0.38	0.000278	1.451189	1.568090493	-0.116901261	+	Yes	NS_NOA; NS_OA
2725	Q6UWP2	DHRS11	dehydrogenase/reductase 11	79154	ENSG00000278535	6	0.012	-0.11	-0.06	0.423	0.307	-0.07	0.109	0.207	0.356	-0.176	-0.165	-0.214	-0.109	-0.194	-0.138	0.0005	-0.086	-0.085	-0.19	-0.38	0.171	-0.31	-0.12	-0.11	-0.01	-0.06	-0.01	0.046738	0.242659	0.258859214	-0.01619979	+		
377	A6NIR2	HSPB11	heat shock protein family B (small) member	51668	ENSG00000081870	2	0.241	0.44	0.199	0.054	0.082	-0.2	-0.65	-0.19	0.047	0.3467	0.2353	0.2358	0.2111	0.0753	0.0622	0.0633	0.1546	0.0284	-0.35	-0.74	-0.38	-0.24	-0.26	0.054	-0.39	-0.3	-0.12	0.025556	0.304201	-0.155225191	0.459425921	+		
3392	Q9H4B8	DPEP3	dipeptidase 3	64180	ENSG00000141096	5	0.411	0.095	0.634	0.431	0.489	-0.27	0.438	1.724	0.458	-0.614	-0.902	-0.822	-0.358	-0.118	0.1507	-0.184	-0.571	-0.27	-0.43	-0	-0.47	-0.64	-0.58	-0.36	0.299	-0.38	-0.16	0.005347	0.792578	0.89927295	-0.106695151	+		
2609	Q5JQC9	AKAP4	A-kinase anchoring protein 4	8852	ENSG00000147081	1							1.118	1.499	1.026							-0.401	-0.927	-0.446							-0.19	-0.54	-0.67	0.009698	1.682457	1.80566752	-0.123210768	+	Yes	NS_NOA; NS_OA
1336	P00750	PLAT	plasminogen activator, tissue type	5327	ENSG00000104368	5	-0.41	0.158	-0.47	-0.67	-0.22	-0.22	-0.45	-0.27	0.056	-0.039	-0.187	-0.002	-0.305	-0.129	-0.286	-0.112	-0.902	-0.232	-0.4	0.51	0.477	0.522	0.036	0.349	0.307	0.027	0.32	0.020933	-0.51523	-0.03220487	-0.483024617	+		
1610	P14618	PKM	pyruvate kinase M1/2	5315	ENSG00000067225	49							0.501	0.778	0.223							-0.825	-0.791	-0.778							-0.62	-0.57	-0.73	0.00572	1.141565	1.298360492	-0.15679546	+	Yes	NS_NOA; NS_OA
953	K7EQ55	DAZAP1	DAZ associated protein 1	26528	ENSG00000071626	2	0.19	-0.01	0.048				0.445	0.144	0.213	-0.149	-0.507	-0.305				-0.293	-0.514	-0.353	-0.09	-0.49	-0.19				0.135	-0.58	-0.27	0.020443	0.420266	0.524709071	-0.104443147	+		
1847	Q53FA3	HSPA1L	heat shock protein family A (Hsp70) membe	3305	ENSG00000226704	31	0.839	0.155	0.319	1.027	1.038	0.542	0.493	1.737	0.419	-1.58	-0.918	-1.31	-0.666	-0.198	-0.567	-1.179	-1.171	-1.371	-0.94	-1.24	-0.97	-0.28	-0.27	-0.68	-0.9	-1.06	-1.14	0	1.561659	1.725695819	-0.164037209	+	Yes	NS_NOA; NS_OA
2092	P54652	HSPA2	heat shock protein family A (Hsp70) membe	3306	ENSG00000126803	38	0.543	-0.11	0.67	1.675	2.02	0.336	0.705	1.384	0.218	-1.224	-1.016	-1.292	-1.104	-1.109	-1.142	-1.005	-1.162	-1.088	-1.41	-1.18	-0.76	-0.44	-1.1	-1.21	-0.54	-0.77	-0.93	0	1.750951	1.953229684	-0.202278922	+	Yes	NS_NOA; NS_OA
2981	Q8TDB8	SLC2A14	solute carrier family 2 member 14	144195	ENSG00000173262	9	1.594	0.581	1.004	0.693	2.435	-0.35	0.5	1.567	0.974	-2.203	-1.71	-1.923	-1.434	-0.905	-1.22	-1.502	-1.727	-1.865	-1.73	-1.54	-2.04	-0.69	-1.1	-1.34	-0.99	-1.3	-1.78	0	2.391753	2.609874113	-0.218120899	+	Yes	NS_NOA; NS_OA
43	A0A087WV2	SSNA1	SS nuclear autoantigen 1	8636	ENSG00000176101	1	0.502	0.244	0.191				0.229	0.614	0.356	-0.22	-0.039	-0.099				-0.381	-0.225	-0.349	-0.52	-1.28	-0.36				-0.06	-0.29	-0.23	0.013548	0.812489	0.574997811	0.237491102	+		
2431	Q13733	ATP1A4	ATPase Na+/K+ transporting subunit alpha	480	ENSG00000132681	6	0.411	-0.17	0.583	0.842	1.056	0.02	0.009	1.177	0.491	-0.713	-0.761	-0.861	-0.391	-0.35	-0.386	-0.492	-0.306	-0.252	-0.65	-0.06	-0.78	-0.38	-0.57	-0.43	-0.14	-0.45	-0.31	0.000231	0.908957	0.992706516	-0.083749452	+		
2723	Q6UWM5	GLIPR1L1	GLIPR1 like 1	256710	ENSG00000173401	4	0.863	0.137	0.65	1.971	-0.55	1.407	1.724	1.903	1.159	0.0342	-0.167	-0.779	-1.039	-0.336	-0.636	-0.9	-1.141	-1.234	-0.41	-0.13	0.014	-0.75	-0.46	-0.87	-0.38	-0.55	-0.39	0.000286	1.464374	1.717479335	-0.253105747	+	Yes	NS_NOA; NS_OA
2961	Q8TAA3	PSMA8	proteasome subunit alpha 8	143471	ENSG00000154611	8				1.541	2.364	0.173	0.419	0.845	0.568				-1.715	-1.461	-1.623	-0.485	-0.908	-0.658				-0.64	-1.46	-1.38	-0.39	-0.61	-0.74	0.001821	1.855999	2.126394642	-0.270395507	+	Yes	NS_NOA; NS_OA
1726	P23280	CA6	carbonic anhydrase 6	765	ENSG00000131686	3	0.975	-0.19	0.422				0.074	0.189	0.627	-0.4	-0.278	-0.079				-0.581	-0.677	-1.08	-0.59	-0.3	-0.22				-0.77	-0.27	-0.45	0.019784	0.78349	0.866061414	-0.082571785	+		
2642	Q5T871	LELP1	late cornified envelope like proline rich 1	149018	ENSG00000203784	2				2.005	2.11	0.657	1.194	1.095	1.332				-0.918	-0.923	-0.867	-0.724	-0.957	-0.706				-1	-0.89	-0.71	-0.19	-0.31	-0.13	0	1.937492	2.247880538	-0.310388106	+	Yes	NS_NOA; NS_OA
2501	Q15147	PLCB4	phospholipase C beta 4	5332	ENSG00000101333	4	0.446	0.19	0.559				0.024	0.486	0.425	-0.141	0.1998	0.3522				0.1523	0.4586	0.0206	-0.37	-0.6	-0.51				-0.3	-0.23	-0.83	0.001226	0.828249	0.181110475	0.647138814	+		
3292	Q9BVM2	DPCD	deleted in primary ciliary dyskinesia homolog	25911	ENSG00000166171	5	0.936	-0.02	0.759	1.17	1.363	0.738	0.072	0.109	-0.2	-0.767	-0.47	-0.626	-0.404	-0.18	-0.08	1.027	0.1086	-0.064	-0.69	-0.52	-0.6	-0.33	-0.57	-0.45	0.102	-0.23	0.126	0.032125	0.898958	0.709131304	0.189826478	+		
3091	Q96AQ6	PBXIP1	PBX homeobox interacting protein 1	57326	ENSG00000163346	6	-0.22	-0.27	-0.66	-0.34	-0.12	-0.15	-0.38	-0.61	-0.27	0.0154	-0.149	-0.175	-0.302	0.4703	-0.101	0.183	-0.278	0.3865	0.455	0.552	0.068	0.155	0.307	0.085	-0.17	0.136	0.014	0.012712	-0.51208	-0.33992586	-0.172158574	+		
1482	P07864	LDHC	lactate dehydrogenase C	3948	ENSG00000166796	17	1.105	0.114	0.521	1.632	2.357	0.299	0.463	1.506	0.819	-2.163	-2.316	-2.32	-1.362	-0.982	-1.373	-1.133	-1.344	-1.283	-2.17	-1.99	-1.93	-0.45	-1.09	-1.24	-0.6	-0.81	-1.04	0	2.238364	2.565850266	-0.327485955	+	Yes	NS_NOA; NS_OA
3080	Q96A08	H2BC1;HIS	H2B clustered histone 1;histone cluster 1 H	255626	ENSG00000146047	6	0.897	0.121	0.69	2.297	2.079	0.063	1.047	1.04	1.155	-2.89	-2.643	-2.955	-2.604	-2.484	-2.614	-0.797	-1.291	-1.036	-2.15	-2.04	-2.42	-1.96	-2.52	-2.56	-0.44	-0.49	-1.63	0	2.845118	3.189083568	-0.343966011	+	Yes	NS_NOA; NS_OA
1541	P10323	ACR	acrosin	49	ENSG00000100312	8	1.371	-0.13	0.304	0.567	0.612	-0.19	0.445	1.644	0.963	-1.206	-1.08	-0.882	-0.683	-0.964	-1.187	-0.276	-0.461	-0.339	-0.95	-1.09	-1.5	1.341	-0.27	0.239	0.019	-0.25	-0.32	0.011436	0.929949	1.407068696	-0.477119965	+	Yes	NS_NOA; NS_OA
2983	Q8TDM5	SPACA4	sperm acrosome associated 4	171169	ENSG00000177202	1				1.039	0.867	-0.07							-1.472	-1.24	-1.667							-0.69	-0.95	-1.2				0.036609	1.556716	2.070263418	-0.513547262	+	Yes	NS_NOA; NS_OA
1622	P15259	PGAM2	phosphoglycerate mutase 2	5224	ENSG00000164708	11				1.601	2.545	0.23	0.47	1.299	0.74				-1.809	-1.45	-1.715	-0.88	-1.01	-1.276				-0.13	-0.98	-1.38	-0.39	-0.76	-1.12	0.000739	1.941322	2.50419338	-0.562871625	+	Yes	NS_NOA; NS_OA
1359	P01619	IGKV3-20	immunoglobulin kappa variable 3-20	28912	ENSG00000239951	3	-0.82	-0.92	-0.33	-1.18	-0.74	-0.48				-0.552	-0.349	-0.479	-0.698	-0.826	-0.983				-0.12	-0.26	1.804	2.286	-0.11	1.081				0.04251	-1.52265	-0.094707722	-1.427940937	+	Yes	OA_NS; OA_NOA
202	A0A0C4DH3	IGHV1-18	immunoglobulin heavy variable 1-18	28468	ENSG00000211945	1	-0.92	-1.37	-0.31	-2.11	-1.05	-1	-1.07	-0.93	-0.88	0.2702	-0.792	-0.65	-1.213	-1.412	-2.366	-0.268	-0.908	-1.275	0.046	-0.31	2.046	2.743	1.044	0.723	0.073	-0.47	-1.14	0.026446	-1.59874	-0.113435944	-1.485304234	+	Yes	OA_NS; OA_NOA
245	P0DP03	IGHV3-30;	immunoglobulin heavy variable 3-30;immunc	28439;8977	ENSG00000270550	4	-2.66	-2.17	-2.41	-2.78	-2.74	-1.96				-4.894	-1.338	-1.617	-2.855	-2.188	-2.913				-1.02	-0.71	3.742	1.169	-0.72	2.092				0.019726	-3.20976	0.183837295	-3.393602083	+	Yes	OA_NOA; OA_NS
885	H7C2R6	CFAP298	cilia and flagella associated protein 298	56683	ENSG00000159079	1	0.213	0.27	0.226							0.3613	0.3176	0.4113							-0.01	-0.42	-0.26							0.047086	0.465592	-0.126998633	0.592590962	+		
1609	P14618	PKM	pyruvate kinase M1/2	5315	ENSG00000067225	53	-0.11	0.26	-0.11	0.058	0.086	-0.49	0.069	-0.18	0.031	0.0935	0.0861	-0.012	-0.031	-0.094	0.0562	0.1948	0.1772	-0.104	-0.18	-0.76	0.062	-0.31	-0.36	0.137	0.026	0.061	0.157	0.442687	0.087315	-0.084168549	0.171483571			
2299	Q02383	SEMG2	semenogelin 2	6407	ENSG00000124157	109	-0.13	-1.43	-0.51	-0.81	-0.35	0.67	0.244	0.28	0.033	-0.067	0.1046	-0.463	-0.939	0.1863	0.0066	-0.249	-0.497	-0.29	0.455	0.816	0.635	-0.34	0.404	-0.36	0.751	-0.22	-0.27	0.271684	-0.43018	0.022760754	-0.452943906			
1417	P04279	SEMG1	semenogelin 1	6406	ENSG00000124233	95	0.009	-1.23	-0.7	-0.41	-0.68	0.277	-0.24	-0.15	-0.18	-0.18	-0.004	-0.186	-0.566	1.116	-0.262	-0.415	-1.511	-0.593	0.619	0.761	0.519	-0.2	0.207	-0.05	0.055	-0.02	-0.33	0.214197	-0.54006	-0.077425663	-0.462631472			
1397	P02788	LTF	lactotransferrin	4057	ENSG00000012223	88	-0.31	-1.65	-0.92	-0.67	-0.96	0.414	0.267	-0.57	0.199	-0.313	-0.421	0.3903	-2.146	-1.189	-1.17	-0.462	-0.988	-0.233	-0.68	0.516	1.492	2.361	0.177	0.251	-0.02	-0.52	-1.74	0.236346	-0.67085	0.259102975	-0.929954915			
1390	P02751	FN1	fibronectin 1	2335	ENSG00000115414	162	0.047	-0.46	0.524	-0.14	-0.96	1.249	0.466	-1.11	-0.05	-0.545	-0.353	-0.697	-0.912	0.2449	-0.501	-1.476	-1.009	-0.327	1.289	-0.74	0.54	0.05	0.158	-0.3	1.065	-0.14	-1	0.212309	-0.15197	0.569950906	-0.721921109			
312	A6NNI4	CD9	CD9 molecule	928	ENSG00000010278	13	-0.55	0.519	0.096	0.027	-0.19	-0.51	0.069	0.126	0.157	0.0164	0.2165	0.1644	0.1646	-0.233	-0.162	0.4454	0.7286	0.7664	-0.6	-0.05	-0.48	-0.94	-0.65	0.028	0.089	0.334	0.542	0.235402	0.164365	-0.262174851	0.426539523			
1550	P10909	CLU	clusterin	1191	ENSG00000120885	46	-0.68	-1.09	-0.58	-0.5	-0.59	-0.58	-0.03	0.111	0.151	-0.264	-0.145	0.135	-0.498	0.6457	-0.79	0.0951	-0.465	0.318	-0.38	0.229	0.018	-0.03	-0.1	-0.27	0.64	0.223	-0.54	0.255404	-0.40009	-0.314791345	-0.085298579			
1619	P15144	ANPEP	alanyl aminopeptidase, membrane	290	ENSG00000166825	70	-0.19	0.792	-0.76	0.354	0.577	-0.71	-0.63	-0.39	-0.64	-0.325	-0.157	0.4027	0.0676	-0.148	-0.48	0.5951	0.3332	0.8209	-0.44	-0.17	-0.48	-0.8	-0.61	-0.47	-0.17	0.082	0.33	0.32324	0.124453	-0.30158925	0.426042537			
1572	P12273	PIP	prolactin induced protein	5304	ENSG00000159763	15	-0.07	-1.25	-0.84	0.018	-0.33	0.059	0.102	0.371	0.115	0.3212	0.5586	0.2494	-0.678	0.5414	-0.129	0.3288	-1.009	0.6002	0.374	1.239	-0.6	0.025	-0.11	-0.54	0.74	-0.65	-0.19	0.665251	-0.23552	-0.289531961	0.054010691			
1625	P15309	ACPP	acid phosphatase, prostate	55	ENSG00000014257	34	0.005	-0.18	0.035	0.081	-0.26	-0.51	-0.47	-0.18	0.016	0.7175	-0.181	0.5636	-0.052	-0.287	-0.78	0.3203	0.4892	-0.026	-0.46	-0.19	0.435	-0.44	-0.27	-0.07	-0.91	-0	-0.57	0.286403	0.112915	-0.248038308	0.360953311			
1986	P49221	TGM4	transglutaminase 4	7047	ENSG00000163810	52	0.834	-2.74	0.96	0.617	-1.72	-0.16	2.724	-1.72	-0.69	-2.106	-2.21	-2.437	1.0597	-1.044	-1.305	-1.677	-1.899	0.7309	-1.91	0.655	-2.04	1.277	0.422	-1.04	0.537	-1.81	0.056	0.469229	0.217877	0.999400142	-0.781523521			
1768	P27487	DPP4	dipeptidyl peptidase 4	1803	ENSG00000197635	61	-0.34	0.557	-0.23	0.45	-0.29	-0.52	-0.14	-0.34	0.197	-0.051	0.1369	0.443	0.1351	-0.695	0.2218	0.2301	0.0321	0.1576	-0.24	0.079	-0.66	-0.55	-0.7	-0.47	-0.09	0.258	0.583	0.50379	0.128156	-0.139452299	0.267608124			
1503	P08473	MME	membrane metalloendopeptidase	4311	ENSG00000196549	75	0.243	0.177	-0.36	-0.3	-0.21	-0.26	-0.72	0.064	0.218	-0.664	0.4658	0.2975	-0.242	-0.571	-0.057	0.4159	-0.361	1.1674	-0.54	-1.01	0.169	-0.67	0.029	-0.34	-0.67	0.326	0.579	0.613344	0.108847	-0.177772655	0.286619403			
1466	P07288	KLK3	kallikrein related peptidase 3	354	ENSG00000142515	21	-0.11	-0.43	-0.34	0.547	-0.79	-1.07	-0.31	-0.3	0.422	0.3639	-0.132	0.2723	-0.16	-0.27	-0.388	1.6861	-0.387	0.1118	-1.04	-0.3	-0.57	-0.38	-0.57	-0.18	0.446	0.91	-1	0.433388	0.034219	-0.387833146	0.422051853			
1490	P08118	MSMB	microseminoprotein beta	4477	ENSG00000263639	6	-2.93	-2.06	-1.13	0.926	-0.35	-0.91	0.743	0.474	0.165	-1.184	-1.922	-0.824	-0.694	-0.814	-1.611	1.7918	0.2935	1.1938	-1.89	-2.19	-0.31	-0.94	-1.48	-0.64	0.051	1.001	0.263	0.932919	0.117992	-0.145532749	0.263524277			
3006	Q8WUM4	PDCD6IP	programmed cell death 6 interacting protein	10015	ENSG00000170248	89	-0.1	0.323	-0.47	0.094	-0.09	-0.34	-0.22	0.05	-0.15	-0.363	0.0383	0.3522	-0.087	-0.222	-0.188	0.2196	0.0528	0.4269	-0.15	-0.04	-0.43	-0.58	-0.45	-0.32	-0.29	0.062	0.566	0.479464	0.081244	-0.125640623	0.206884944			
3749	Q9Y3R5	DOP1B	DOP1 leucine zipper like protein B	9980	ENSG00000142197	139	-0.29	0.509	-0.18	-0.01	-0.28	-0.41	0.134	-0.15	0.109	0.3972	0.11	0.1614	0.3411	-0.346	-0.18	0.5033	0.2235	0.3573	-0.33	-0.79	-0.22	-0.89	-0.69	0.228	-0.35	-0.1	0.094	0.095651	0.277163	-0.237183194	0.51434664			
220	A0A0G2JIW	HSPA1B	heat shock protein family A (Hsp70) membe	3304	ENSG00000212866	65	0.13	0.121	0.045	0.061	0.193	-0.59	0.034	-0.25	0.339	0.0343	0.1239	0.0137	0.0956	-0.343	0.1146	-0.089	0.3968	0.1562	-0.35	-0.78	-0.22	-0.79	-0.56	-0.13	-0.07	-0.01	0.271	0.147911	0.302814	-0.046873779	0.349687487			
1231	O75874	IDH1	isocitrate dehydrogenase (NADP(+)) 1, cyto	3417	ENSG00000138413	44	0.18	0.022	-0.08	0.131	0.029	-0.79	0.306	-0.14	0.276	0.1429	0.074	-0.011	-0.032	-0.31	0.0957	0.031	0.2199	-0.213	-0.3	-1.29	-0.24	-0.84	-0.26	-0.05	0.017	0.25	0.335	0.398631	0.256952	-0.006927499	0.263879894			
2221	P62937	PPIA	peptidylprolyl isomerase A	5478	ENSG00000196262	14	-0.02	0.344	0.038	0.068	-0.12	-0.52	0.077	-0.26	0.112	0.0295	0.0697	-0.006	0.1372	-0.122	0.1753	0.0503	0.4192	0.0762	-0.39	-1.38	-0.47	-0.87	-0.48	0.066	-0.3	-0.03	0.39	0.125452	0.353084	-0.122831581	0.47591586			
1573	P12277	CKB	creatine kinase B	1152	ENSG00000166165	29	-0.12	0.058	-0.07	-0.39	-0.28	-0.4	0.079	0.169	0.236	0.482	0.1254	-0.018	-0.239	-0.062	0.0404	0.2673	0.2348	-5E-04	0.214	-1.04	-0.32	-0.75	-0.4	0.117	-0.34	0.176	0.618	0.403712	0.111216	-0.173072377	0.284288757			
1989	P49327	FASN	fatty acid synthase	2194	ENSG00000169710	148	-0.02	0.353	0.213	-0.56	-0.05	-0.61	-0.07	-0.16	0.128	0.347	-0.132	-0.063	0.2817	-0.187	-0.197	-0.111	0.0365	0.3172	-0.16	-0.68	-0.25	-0.44	-0.21	0.022	0.158	-0.07	-0.02	0.413515	0.098166	-0.11814576	0.216311335			
229	A0A0G2JN4	MUC6	mucin 6, oligomeric mucus/gel-forming	4588	ENSG00000184956	71	-0.53	-0.52	0.161	-1.27	0.172	0.047	-0.67	-0.32	-0.26	0.5507	0.0551	0.0304	-0.441	0.1397	-0.732	-1.179	0.2476	-0.878	0.376	0.518	-0.18	-0.67	0.577	0.084	0.191	1.023	-1	0.379684	-0.456	-0.109269361	-0.346727518			
2341	Q08380	LGALS3BF	galectin 3 binding protein	3959	ENSG00000108679	26	-0.8	-0.3	0.496	-0.84	0.105	-0.25	-0.01	0.1	0.09	0.3959	0.024	0.0294	-0.313	0.0982	-0.799	-0.098	0.106	0.0674	-0.58	0.72	-0.45	0.313	-0.12	-0.16	0.538	0.493	-0.96	0.875589	-0.1353	-0.103852722	-0.03144477			
2223	P62979	RPS27A	ribosomal protein S27a	6233	ENSG00000143947	13	-0.31	0.288	-0.05	0.112	-0.31	-0.17	-0.14	0.168	-0.26	-0.319	0.0476	-0.013	0.152	-0.198	-0.078	0.1095	0.3506	0.0595	-0.38	0.075	-0.37	-0.45	-0.34	-0.26	-0.52	0.125	0.421	0.409837	0.114558	-0.086914306	0.201472532			
1684	P20142	PGC	progastricsin	5225	ENSG00000096088	8	-0.54	-1.28	0.152	-2.27	-0.41	-0.66	-0.48	0.165	-0.93	-0.397	1.283	-0.938	-1.709	0.71	-0.824	-0.65	-0.074	-0.526	-0.82	0.188	1.239	-1.47	-0.6	0.032	1.274	0.736	-0.73	0.435522	-0.67823	-0.348060917	-0.330169287			
1626	P15311	EZR	ezrin	7430	ENSG00000092820	71	0.1	0.334	-0.07	0.18	0.193	-0.6	-0.05	0.013	0.006	-0.397	0.0543	0.2315	0.2436	-0.388	0.2134	0.0688	0.2127	0.1477	-0.46	-0.38	-0.71	-0.55	-0.69	-0.4	-0.37	-0.16	0.264	0.068079	0.395786	-0.031390439	0.427176351			
2285	Q00796	SORD	sorbitol dehydrogenase	6652	ENSG00000140263	36	0.135	0.274	-0.09	-0.17	-0.17	-0.59	0.195	-0.12	0.173	0.2423	0.0871	0.0023	-0.164	0.1706	-0.043	0.2311	0.3229	0.0813	-0.32	-1.38	-0.09	-0.66	-0.54	-0.09	-0.1	0.061	0.386	0.189249	0.261791	-0.144885188	0.406676264			
2135	P60709	ACTB	actin beta	60	ENSG00000075624	33	0.112	-0.06	-0.08	0.059	0.375	-0.24	0.175	0.184	0.285	-0.16	-0.184	0.803	0.1271	-0.307	-0.06	-3E-04	0.0385	0.0515	-0.47	-0.98	0.09	0.205	-0.44	-0.2	-0.22	-0.15	-0.1	0.172284	0.340978	0.055299438	0.285678889			
2263	P80303	NUCB2	nucleobindin 2	4925	ENSG00000070081	56	0.162	-0.75	-0.81	-0.62	-0.12	-0.26	0.287	0.775	-0.04	-0.138	-0.817	-0.715	-0.506	0.5822	-0.209	-0.18	-1.061	-0.39	0.591	0.78	-0.33	-0.24	0.74	-0.31	0.337	-0.4	-0.1	0.269995	-0.27001	0.229430205	-0.499438338			
1475	P07602	PSAP	prosaposin	5660	ENSG00000197746	40	-0.42	-0.31	-0.05	-1.02	-0.25	-0.23	0.108	-0.35	0.328	0.7921	0.3711	0.0724	-0.683	0.0431	-0.571	-0.012	-0.247	0.0611	-0.07	0.474	-0	-0.38	0.384	-0.22	0.974	0.22	-0.57	0.433969	-0.33347	-0.224201775	-0.109264503			
1410	P04083	ANXA1	annexin A1	301	ENSG00000135046	35	0.403	0.137	0.044	-0.09	0.06	-0.48	0.383	-0.17	0.177	-0.438	0.1151	0.3551	0.1389	-0.325	0.2559	-0.01	0.3673	0.0409	0.047	-1.17	-0.27	-0.54	-0.47	-0.19	0.198	0.061	0.476	0.387824	0.258257	-0.004093952	0.262350766			
1419	P04406	GAPDH	glyceraldehyde-3-phosphate dehydrogenase	2597	ENSG00000111640	26	-0.01	0.053	8E-04	-0.11	0.071	-0.57	0.36	0.122	0.238	0.1106	0.038	0.0412	-0.177	-0.221	0.0575	0.2521	0.2421	0.0961	-0.22	-0.87	0.146	-0.03	-0.39	0.013	0.179	0.294	0.186	0.721313	0.094015	-0.032809687	0.126824825			
1553	P11142	HSPA8	heat shock protein family A (Hsp70) membe	3312	ENSG00000109971	57	0.098	0.368	-0.09	-0.08	0.104	-0.6	0.193	0.013	0.192	0.0131	-0.041	0.0514	-0.027	-0.127	0.0725	0.0338	0.2419	0.1375	-0.22	-0.97	-0.24	-0.67	-0.34	-0	-0.08	-0.05	0.248	0.17572	0.279867	-0.017783008	0.297649733			
1483	P07900	HSP90AA1	heat shock protein 90 alpha family class A m	3320	ENSG00000080824	69	0.178	0.119	-0.05	0.063	0.37	-0.41	0.088	-0.06	0.149	-0.065	-0.011	-0.135	-0.014	-0.109	0.0096	-0.086	0.1727	0.0028	-0.2	-1.18	-0.4	-0.74	-0.43	0.053	0.104	-0.2	0.043	0.125952	0.376353	0.074667102	0.301686066			
2447	Q14204	DYNC1H1	dynein cytoplasmic 1 heavy chain 1	1778	ENSG00000197102	265	-0	0.09	-0.12	-0.04	0.108	-0.35	0.003	-0.09	0.078	0.0299	0.045	0.0851	0.0873	-0.141	0.116	0.0807	0.2051	0.1636	-0.21	-0.67	-0.22	-0.42	-0.27	0.019	0.127	0.042	0.223	0.192032	0.117306	-0.110463344	0.227769494			
2312	Q04609	FOLH1	folate hydrolase 1	2346	ENSG00000086205	56	-0.51	-0.04	-0.39	-1.15	0.26	-0.43	-0	0.084	0.919	-0.252	-0.15	0.98	-0.271	-0.092	-0.549	-0.924	-0.513	0.3111	-0.63	1.274	0.091	0.072	-0.67	0.029	0.002	0.035	-0.94	0.970799	-0.05843	0.021822132	-0.080248238			
3325	Q9BZQ8	NIBAN1;FA	niban apoptosis regulator 1;family with sequ	116496	ENSG00000135842	54	0.243	0.09	0.207	-0.15	-0.18	-0.72	-0.01	-0.4	0.579	0.306	-0.191	0.3557	0.2132	-0.236	-0.12	0.0981	0.3214	0.2317	-0.06	-1.02	-0.5	-0.6	-0.92	0.018	-0.17	0.125	0.132	0.171517	0.29549	-0.145315445	0.440805697			
1083	O15230	LAMA5	laminin subunit alpha 5	3911	ENSG00000130702	140	-0.44	-0.39	-0.36	-0.27	-0.52	-0.17	0.071	0.039	-0.17	0.0019	0.2724	0.0191	-0.52	0.685	0.0165	0.4472	-0.44	0.3233	0.225	0.49	0.163	-0.39	-0.31	0.075	0.3	-0.24	-0.05	0.202961	-0.2754	-0.336314999	0.060911841			
2246	Q5VTE0	EEF1A1P5	eukaryotic translation elongation factor 1 alp	158078	ENSG00000196205	31	-0.06	-0.04	-0.04	-0.1	-0.25	-0.62	0.235	0.243	0.43	0.0294	-0.127	-0.024	-0.202	-0.219	-0.028	0.1189	0.3994	0.3779	-0.25	-0.96	-0.29	-0.74	-0.46	-0.11	0.127	0.074	0.357	0.324548	0.227502	-0.058149254	0.28565136			
1839	P33176	KIF5B	kinesin family member 5B	3799	ENSG00000170759	117	0.11	0.437	-0.03	-0.14	-0.37	-0.45	0.159	-0.08	0.298	0.2811	0.0396	-0.021	-0.152	-0.044	0.1793	0.0861	0.3695	-0.133	-0.32	-1.33	-0.06	-0.63	-0.41	0.055	-0.13	-0.1	0.413	0.243406	0.270792	-0.075377834	0.346169742			
1672	P19440	GGT1	gamma-glutamyltransferase 1	2678	ENSG00000100031	26	-0.51	0.142	0.254	-0.23	0.09	-0.22	-0.09	-0.06	-0.24	0.0759	0.4642	0.085	0.1288	0.0136	-0.328	0.0667	-0.103	0.0114	-0.87	0.051	0.043	-0.57	-0.16	-0.08	-0.36	0.032	-0.29	0.211079	0.14723	-0.143176717	0.290406523			
1552	P11047	LAMC1	laminin subunit gamma 1	3915	ENSG00000135862	100	-0.58	-0.48	-0.43	-0.23	-0.6	-0.2	0.041	0.014	-0.27	-0.091	0.2785	-0.005	-0.514	0.7375	0.037	0.4453	-0.523	0.2819	0.207	0.459	0.094	-0.55	-0.3	0.077	0.236	-0.34	-0.14	0.203187	-0.27379	-0.37507542	0.101289227			

1508	P08758	ANXA5	annexin A5	308	ENSG00000164111	41	0.154	0.116	-0.11	-0.07	-0.05	-0.62	-0.52	-0.28	-0.07	0.0645	-0.018	-0.002	0.0393	-0.191	0.2388	-0.196	0.259	-0.075	-0.11	-1.09	-0.31	-0.68	-0.4	-0.02	-0.17	-0.11	0.216	0.230536	0.135273	-0.174060682	0.309333313
1301	O95716	RAB3D	RAB3D, member RAS oncogene family	9545	ENSG00000105514	27	0.068	0.485	0.102	0.256	-0.22	-0.52	0.112	-0.19	0.197	0.2338	0.2106	0.2091	0.3577	-0.434	-0.246	0.5487	0.3217	0.2946	-0.44	-1.03	-0.29	-0.77	-0.63	0.131	-0.37	-0.29	0.268	0.08446	0.411738	-0.134474151	0.546212316
2313	Q04760	GLO1	glyoxalase I	2739	ENSG00000124767	16	0.286	-0.03	-0.31	-0.02	-0.15	-0.22	0.381	0.246	0.291	0.3111	-0.04	-0.044	-0.279	-0.342	0.0015	-0.012	0.3398	-0.486	-0.25	-0.66	-0.09	-0.79	-0.72	0.86	-0.31	0.081	0.513	0.669822	0.20382	0.114354655	0.089465774
1862	P35579	MYH9	myosin heavy chain 9	4627	ENSG00000100345	167	-0.08	0.037	-0.08	-0.11	0.05	-0.39	-0.07	-0.26	0.024	0.1589	0.0334	-0.059	0.0491	-0.101	0.1292	0.0935	0.1941	-0.129	-0.15	-0.81	0.039	-0.01	-0.31	0.073	0.128	0.031	0.098	0.418358	0.002991	-0.138549354	0.141540018
1829	P31947	SFN	stratifin	2810	ENSG00000175793	29	0.136	0.124	-0.2	-0.08	-0.15	-0.54	2E-04	-0.27	0.051	-0.046	0.1322	0.0788	-0.024	-0.131	0.23	0.0318	0.2201	0.0518	-0.17	-1.6	-0.25	-0.96	-0.35	-0.05	0.049	-0.13	0.365	0.221406	0.241495	-0.164086997	0.405582357
2113	P55268	LAMB2	laminin subunit beta 2	3913	ENSG00000172037	101	-0.26	-0.4	-0.4	-0.3	-0.54	-0.16	-0.04	0.035	-0.17	-0.01	0.1432	-0.115	-0.456	0.556	-0.003	0.3188	-0.6	0.0581	0.292	0.716	0.22	-0.32	-0.09	0.089	0.253	-0.28	-0.14	0.208223	-0.3303	-0.236593914	-0.093701277
2019	P50395	GDI2	GDP dissociation inhibitor 2	2665	ENSG00000057608	52	-0.03	0.186	-0.02	0.121	0.022	-0.37	0.283	1E-03	0.293	-0.027	-0.055	-0.033	0.0435	-0.329	0.0549	0.1631	0.1894	0.109	-0.29	-0.94	-0.31	-0.67	-0.43	0.012	-0.12	-0.02	0.288	0.139319	0.326581	0.039707257	0.286873752
1454	P06733	ENO1	enolase 1	2023	ENSG00000074800	38	0.15	0.141	-0.3	0.173	-0.09	-0.5	0.408	0.16	0.177	0.1404	-0.015	0.1122	-0.325	-0.066	0.0482	0.1871	0.2544	-0.211	-0.11	-1.04	-0.19	-0.67	-0.4	0.073	-0.38	0.001	0.361	0.236121	0.297651	0.020655441	0.276995882
2265	P80723	BASP1	brain abundant membrane attached signal p	10409	ENSG00000176788	32	-0.12	0.216	0.317	0.115	0.174	-0.35	0.004	-0.32	-0.26	0.2805	-0.012	-0.006	0.13	-0.552	0.1486	-0.222	0.1692	-0.641	-0.07	-0.31	-0.18	-0.82	-0.4	0.463	-0.82	-0.27	-0.28	0.343562	0.273969	0.053337336	0.220631575
1152	O60282	KIF5C	kinesin family member 5C	3800	ENSG00000276734	111	0.078	0.448	-0.18	-0.09	-0.28	-0.4	0.012	-0.13	0.236	0.3152	-0.121	-0.161	-0.081	-0.14	0.2568	0.1419	0.4058	-0.243	-0.46	-1.6	-0.26	-0.77	-0.35	-0.05	-0.2	-0	0.301	0.192999	0.343823	-0.075196069	0.41901935
1586	P13639	EEF2	eukaryotic translation elongation factor 2	1938	ENSG00000167658	66	0.147	0.201	0.059	0.069	0.072	-0.33	0.008	-0.01	0.248	0.186	0.0611	0.0612	0.0649	-0.261	0.049	0.098	0.2073	0.125	-0.27	-1.14	-0.3	-0.5	-0.25	0.157	-0	-0.04	0.162	0.149729	0.292129	-0.0139789	0.306107839
1332	P00558	PGK1	phosphoglycerate kinase 1	5230	ENSG00000102144	41	0.064	0.112	-0.08	0.058	-0.08	-0.15	0.226	-0.1	0.27	-0.128	-0.015	-0.056	-0.139	-0.029	0.0871	0.1223	0.3566	0.1809	-0.33	-1.2	-0.26	-0.72	-0.31	-0.13	-0.1	0.125	0.423	0.176099	0.314874	-0.006573383	0.32144725
1469	P07355	ANXA2	annexin A2	302	ENSG00000182718	40	-0.14	0.046	-0.02	-0.48	0.301	-0.91	0.505	-0.08	-0.21	0.6429	-0.467	-0.412	-0.355	0.0472	0.2739	0.2249	0.0471	0.5461	-0.25	-0.1	-0.22	0.082	-0.27	-0.26	-0.25	-0.56	0.479	0.594863	0.041372	-0.170654103	0.212025757
1459	P06858	LPL	lipoprotein lipase	4023	ENSG00000175445	30	-0.26	-0.39	-0.57	-0.66	-0.69	0.114	0.063	0.083	-0.37	0.1455	-0.413	-0.593	-0.248	0.6498	0.0622	-0.067	-0.802	-0.538	0.736	0.67	0.463	0.055	0.401	-0.04	0.207	-0.35	-0.22	0.130527	-0.51188	-0.09733801	-0.414546466
1689	P20337	RAB3B	RAB3B, member RAS oncogene family	5865	ENSG00000169213	26	-0.13	0.111	-0.15	0.109	-0.22	-0.34	0.292	0.132	0.534	-0.339	-0.366	-0.156	-0.111	-0.352	-0.123	0.7533	0.5013	0.6592	-0.6	-0.97	-0.62	-0.87	-0.61	0.046	-0.23	-0	0.624	0.224524	0.396835	-0.014301545	0.411136832
2329	Q06830	PRDX1	peroxiredoxin 1	5052	ENSG00000117450	24	0.197	0.137	-0.12	-0.09	-0.03	-0.61	0.192	0.014	0.198	0.1281	-0.051	-0.108	-0.166	-0.167	0.1951	0.0367	0.1967	-0.049	-0.24	-0.98	-0.45	-0.88	-0.43	-0.1	-0.03	0.028	0.307	0.19684	0.295798	-0.013677675	0.309475706
1601	P14174	MIF	macrophage migration inhibitory factor	4282	ENSG00000240972	6	-0.28	0.483	-0.1	0.513	-0.08	-0.66	0.28	-0.03	0.519	0.1369	0.0793	0.0768	0.1127	-0.638	-0.115	0.469	0.4644	0.0669	-0.12	-1.25	-0.22	-0.85	-0.58	-0.19	-0.03	0.124	0.187	0.212143	0.397128	3.54072E-05	0.397092634
1578	P12821	ACE	angiotensin I converting enzyme	1636	ENSG00000159640	58	-0.42	0.274	0.272	-0.67	0.461	0.315	-0.14	-0.44	-0.13	0.0743	0.3348	-0.093	-0.349	-0.075	-0.461	-0.713	0.268	-0.062	-0.32	1.036	-0.27	-0.06	-0.07	-0.53	0.246	0.199	-0.63	0.943651	-0.00973	0.066149173	-0.075879721
2437	Q14008	CKAP5	cytoskeleton associated protein 5	9793	ENSG00000175216	121	0.14	0.335	-0.18	-0.02	-0.25	-0.3	-0.03	-0.26	0.04	0.073	0.0665	0.0686	-0.095	-0.076	0.1228	0.1013	0.3065	-0.04	-0.46	-1.27	-0.32	-0.68	-0.47	-0.08	-0.18	-0.12	0.354	0.121596	0.301254	-0.116005533	0.417259506
2038	P51159	RAB27A	RAB27A, member RAS oncogene family	5873	ENSG00000069974	20	-0.12	0.498	0.022	0.182	-0.32	-0.16	-0.12	-0.32	0.444	0.4677	0.0505	0.2253	0.2247	-0.285	-0.207	0.2724	-0.068	0.0256	-0.29	-1.12	-0.47	-0.88	-0.69	0.214	-0.33	-0.03	0.225	0.132567	0.385963	-0.067277444	0.45324013
1495	P08238	HSP90AB	heat shock protein 90 alpha family class B m	3326	ENSG00000096384	67	0.155	0.123	-0.04	0.039	0.095	-0.38	0.103	0.003	0.202	0.0041	-0.045	0.0504	0.0948	-0.086	0.0616	0.0512	0.1538	0.0753	-0.16	-0.99	-0.24	-0.71	-0.27	0.017	0.13	0.021	0.205	0.193302	0.255148	-0.006009509	0.261157488
1745	P25311	AZGP1	alpha-2-glycoprotein 1, zinc-binding	563	ENSG00000160862	31	-0.18	-0.02	-0.37	0.284	-0.39	-0.41	-0.24	0.14	0.264	1.1886	-0.065	0.4455	-0.049	0.0632	-0.898	0.4328	-0.06	-0.329	-0.49	-0.77	-0.15	-0.59	0.119	0.129	-0.29	0.641	-0.74	0.50418	0.134254	-0.183878197	0.318131707
2758	Q71U36	TUBA1A	tubulin alpha 1a	7846	ENSG00000167552	34	0.296	0.486	-0.17	0.063	-0.05	-0.54	0.209	0.133	0.296	0.2947	0.1334	0.1953	-0.2	0.0241	0.1284	0.1872	0.2163	0.1988	-0.48	-1.42	-0.31	-0.81	-0.5	-0.2	-0.02	0.052	0.445	0.121552	0.439384	-0.051817938	0.491202353
1784	P28907	CD38	CD38 molecule	952	ENSG00000004468	27	0.279	-0.06	-0.22	-0.45	-0.39	-0.26	0.002	0.2	-0.36	0.2823	0.2106	0.1501	0.531	-0.23	0.1749	0.2782	0.3606	-0.233	-0.14	-0.33	-0.04	-1.06	-0.15	0.302	-0.09	-0.35	-0.65	0.117837	0.14025	-0.30927466	0.449525133
2249	P68371	TUBB4B	tubulin beta 4B class IVb	10383	ENSG00000188229	29	0.097	0.342	-0.2	0.103	0.108	-0.41	0.467	0.251	0.468	0.107	-0.04	0.0522	-0.069	0.0611	0.2554	0.278	0.2473	0.1998	-0.46	-1.26	-0.23	-0.62	-0.4	-0.12	0.098	0.183	0.663	0.200962	0.376154	0.015083319	0.361070959
1093	O15393	TMPRSS2	transmembrane serine protease 2	7113	ENSG00000184012	23	-0.22	0.748	-0.45	-0.4	-0.37	-0.03	-0.08	0.189	-0.07	0.2105	0.3901	0.1673	-0.527	-0.237	-0.237	0.6011	0.1023	1.0562	-0.07	-0.31	-0.39	-0.14	-0.64	-0.34	-0.19	-0.08	0.743	0.393358	0.085215	-0.244356189	0.329571209
1021	O00560	SDCBP	syndecan binding protein	6386	ENSG00000137575	25	-0.52	0.494	-0.12	-0.14	-0.16	-0.17	-0.07	0.038	-0.03	-0.098	0.0525	0.2067	0.0979	-0.143	-0.35	0.2488	0.1889	0.5245	-0.19	0.655	-0.16	-0.45	-0.23	-0.09	-0.37	0.127	0.401	0.649251	-0.04056	-0.156306359	0.11575011
993	O00194	RAB27B	RAB27B, member RAS oncogene family	5874	ENSG00000041353	20	-0.29	0.411	-0.25	-0.06	-0.2	-0.33	-0.08	-0.26	0.18	0.2117	0.129	0.1662	0.0406	-0.35	0.0209	0.2444	0.4012	0.1508	-0.28	-0.78	-0.54	-0.68	-0.44	0.148	-0.32	-0.15	0.267	0.109956	0.211407	-0.21007171	0.42147886
2909	Q8N6Q3	CD177	CD177 molecule	57126	ENSG00000204936	13	0.405	0.462	-1.07	-0.1	0.291	-0.2	-0.3	-0.64	0.528	-0.202	-0.291	0.1171	-0.263	0.1202	-0.475	0.1965	0.099	0.2962	-0.83	-0.98	0.028	-0.04	0.651	-0.21	0.662	0.205	0.189	0.99141	-0.03365	-0.024599464	-0.009047181
2130	P60174	TPI1	triosephosphate isomerase 1	7167	ENSG00000111669	28	-0.1	0.066	-0.09	0.009	0.033	-0.31	0.04	-0.11	0.168	-0.065	-0.111	0.0725	-0.066	-0.065	0.0304	-0.015	0.0305	-0.025	-0.33	-1.04	-0.16	-0.45	-0.29	-0.02	0.037	0.132	0.067	0.264897	0.196001	-0.007938305	0.203938937
1734	P23528	CFL1	cofilin 1	1072	ENSG00000172757	27	0.007	0.324	0.05	-0.03	-0.13	-0.46	0.137	-0.05	0.203	0.0612	0.0647	-0.079	-0.001	-0.187	0.1922	0.1457	0.2252	0.1047	-0.26	-1.36	-0.27	-0.71	-0.42	0.098	-0.19	0.037	0.291	0.172455	0.31348	-0.054407505	0.367887729
303	A0A286YF2	PHGDH	phosphoglycerate dehydrogenase	26227	ENSG00000092621	36	0.156	0.058	-0.2	0.029	0.019	-0.81	-0.03	-0.05	0.039	-0.149	-0.089	-0.053	0.0573	-0.165	0.25	0.0477	0.2678	0.0441	-0.39	-1.29	-0.43	-0.53	-0.58	-0.12	0.199	0.281	0.43	0.400121	0.180879	-0.111572672	0.292451592
1408	P04075	ALDOA;AC	aldolase, fructose-bisphosphate A;uncharat	226;112694	ENSG00000149925	42	0.116	0.177	-0.02	0.015	0.059	-0.61	0.478	0.102	0.27	0.2044	-0.024	0.0891	-0.148	-0.265	-0.017	0.2549	0.0614	0.0428	-0.19	-0.52	6E-04	-0.5	-0.34	-0.13	0.201	0.347	0.432	0.667557	0.143457	0.043343497	0.100113947
1522	P09543	CNP	2',3'-cyclic nucleotide 3' phosphodiesterase	1267	ENSG00000173786	46	-0.17	0.316	-0.06	0.047	-0.23	-0.52	-0.02	-0.18	0.201	0	0.1466	0.1941	0.1921	-0.439	0.1823	0.1305	0.1019	0.1726	-0.38	-0.85	-0.4	-0.72	-0.59	0.019	-0.19	-0.02	0.35	0.146119	0.240823	-0.145041121	0.385864382
49	A0A087WV	CLTC	clathrin heavy chain	1213	ENSG00000141367	89	0.091	0.07	0.011	0.465	0.186	-0.4	-0.05	0.074	0.184	-0.007	-0.05	0.0663	0.0341	-0.035	-0.093	-0.094	-0.012	0.0931	-0.18	-0.75	-0.21	-0.38	-0.25	-0.06	-0.06	-0.07	0.104	0.126146	0.276883	0.081110788	0.195771786
2070	P53396	ACLY	ATP citrate lyase	47	ENSG00000131473	67	0.268	0.171	-0.03	-0.11	0.036	-0.42	-0.02	0.04	0.307	0.0541	-0.04	0.0398	-0.008	-0.074	-0.032	0.1703	0.114	0.2763	-0.06	-0.87	-0.19	-0.61	-0.49	-0.1	0.159	0.077	0.212	0.212492	0.23416	-0.028360813	0.262520967
3162	Q96KP4	CNDP2	carnosine dipeptidase 2	55748	ENSG00000133313	43	0.235	0.007	-0.07	0.09	-0.01	-0.45	0.017	-0.26	0.029	-0.154	0.1736	-0.011	0.0911	-0.025	-0.014	0.0867	0.1839	0.0352	-0.36	-1.47	-0.32	-0.92	-0.26	0.044	-0.22	0.21	0.265	0.20574	0.291404	-0.086650398	0.378054847
2189	P62258	YWHAE	tyrosine 3-monooxygenase/tryptophan 5-mo	7531	ENSG00000108953	28	0.172	0.219	-0	-0.19	0.005	-0.49	-0.24	-0.38	-0.15	0.1503	0.1429	0.0921	0.0621	0.0817	-0.008	-0.294	-0.143	-0.408	-0.02	-0.96	-0.25	-0.75	-0.36	0.051	-0.08	-0.32	-0.02	0.269911	0.184191	-0.081942199	0.266133261
1642	P16870	CPE	carboxypeptidase E	1363	ENSG00000109472	32	-0.15	-0.42	-0.19	-0.25	-0.18	-0.62	-0.16	0.413	0.505	0.7669	-0.315	0.2424	-0.441	-0.333	-0.893	0.6419	0.0495	-0.027	0.17	0.298	-0.26	-0.32	-0.27	-0.14	0.653	0.658	-0.78	0.903648	-0.11992	-0.083225032	-0.036698366
1797	P30041	PRDX6	peroxiredoxin 6	9588	ENSG00000117592	28	0.055	0.445	0.026	0.038	0.113	-0.43	0.301	0.133	0.423	0.0366	0.0504	0.0438	-0.198	-0.189	0.1821	0.1324	0.5464	0.3306	-0.36	-1.29	-0.38	-0.48	-0.44	-0.2	-0.08	0.163	0.537	0.157679	0.40431	0.018317999	0.385991657
3205	Q99497	PARK7	Parkinsonism associated deglycase	11315	ENSG00000116288	21	0.214	0.404	0.162	0.116	0.156	-0.53	0.074	0.091	0.262	-0.089	-0.059	0.059	0.0404	0.0178	0.1426	0.1126	0.2355	0.0176	-0.31	-1.21	-0.31	-0.82	-0.42	0.046	-0.09	0.089	0.142	0.112364	0.425095	0.052545174	0.372549944
1765	P27348	YWHAQ	tyrosine 3-monooxygenase/tryptophan 5-mo	10971	ENSG00000134308	27	-0.03	0.096	-0.15	-0.07	-0.09	-0.7	-0.03	-0.1	0.359	-0.103	-0.002	0.0705	-0.033	-0.204	-0.01	0.0845	0.1427	0.2363	-0.36	-1.08	-0.33	-0.84	-0.55	-0	-0.04	0.07	0.177	0.194825	0.250139	-0.098542339	0.348681165
607	E7ER45	MGAM	maltase-glucoamylase	8972	ENSG00000257335	74	0.169	0.067	1.284	0.213	0.118	0.002	-0.47	0.063	-0.27	-0.743	0.0119	1.1049	1.0481	-1.407	0.4564	-0.587	0.2858	-0.472	-0.88	-1.25	-0.73	0.182	-0.78	-1.38	-1.25	-0.49	-1.38	0.073694	1.016172	0.164305987	0.851865678
347	E9PNW4	AL049629.	novel protein;CD59 molecule (CD59 blood g	966	ENSG00000284969	7	-0.32	0.134	-0.02	-0.34	0.021	-0.37	0.058	-0.08	0.356	0.1508	0.1764	0.0541	-0.055	-0.198	-0.179	-0.299	-0.006	0.3669	-0.3	0.409	-0.03	-0.24	-0.39	-0.03	-0.32	0.32	-0.22	0.815546	0.026761	-0.063722633	0.0904835
3720	Q9Y2Q0	ATP8A1	ATPase phospholipid transporting 8A1	10396	ENSG00000124406	60	-0.04	0.159	0.188	-0.12	-0.23	-0.49	0.187	-0.11	0.177	0.2569	0.2538	0.0948	0.071	-0.177	0.2255	0.1721	0.3098	0.2738	-0.34	-0.9	-0.41	-0.57	-0.35	0.069	-0.09	-0.05	0.078	0.052886	0.252515	-0.195758982	0.448274007
1583	P13473	LAMP2	lysosomal associated membrane protein 2	3920	ENSG00000005893	12	-0.32	0.056	-0.24	0.081	-0	-0.57	0.363	-0.23	0.394	0.1304	0.4987	-0.283	0.5122	-0.477	0.2013	0.2493	0.4793	0.5986	-0.67	-0.94	-0.4	-0.56	-0.44	0.482	-0.49	-0.29	0.382	0.138685	0.271453	-0.265028909	0.536482309
1803	P30086	PEBP1	phosphatidylethanolamine binding protein 1	5037	ENSG00000089220	17	0.153	0.433	0.206	-0.09	-0.13	-0.39	0.456	0.198	0.415	0.2058	0.0865	0.1274	0.0004	-0.042	0.1159	0.3417	0.4206	0.1787	-0.03	-1.05	-0.45	-0.79	-0.63	-0.2	0.047	0.228	0.443	0.127641	0.408725	-0.020081918	0.428806678
1599	P13861	PRKAR2A	protein kinase cAMP-dependent type II regu	5576	ENSG00000114302	35	0.123	0.322	-0.15	0.257	0.169	-0.51	0.199	0.019	0.261	-0.39	0.0121	-0.211	0.0121	-0.405	-0.08	0.4216	0.2527	0.5777	-0.38	-0.75	-0.35	-0.61	-0.52	-0.16	-0.2	-0.14	0.226	0.124655	0.396785	0.055761851	0.341023244
3797	Q9Y6R7	FCGBP	Fc fragment of IgG binding protein	8857	ENSG00000275395	100	-0.8	-0.86	-0.28	-1.48	-0.49	-1.14	-0.01	-0.13	-0.73	0.0862	-0.233	0.019	0.1737	-0.689	-0.879	-0.267	0.1995	0.0587	-0.82	0.363	0.684	2.26	0.278	0.379	-0.86	-0.61	-1.22	0.264416	-0.70918	-0.488022182	-0.221162345
2868	Q8IYJ3	SYTL1	synaptotagmin like 1	84958	ENSG00000142765	38	-0.07	0.163	-0.05	#####	-0.15	-0.43	-0.01	0.011	0.424	0.1017	0.155	0.0553	0.0901	-0.359	0.0551	0.3336	0.325	0.2705	-0.37	-0.75	-0.26	-0.78	-0.57	0.202	-0.09	-0.13	0.141	0.115206	0.276814	-0.127069566	0.403883354
602	O95359	TACC2	transforming acidic coiled-coil containing pr	10579	ENSG00000138162	114	-0.12	0.254	-0.26	-0.04	-0.13	-0.27	0.445	-0.05	0.302	0.0896	0.0694	0.2368	-0.129	-0.019	0.2405	0.1614	0.3195	0.0311	-0.51	-1.22	-0.31	-0.75	-0.62	-0.18	0.264	0.009	0.415	0.147603	0.335198	-0.097934383	0.433132592
1902	P40925	MDH1	malate dehydrogenase 1	4190	ENSG00000014641	26	0.109	0.277	-0.08	0.065	0.047	-0.74	0.203	0.058	0.309	-0.089	-0.029	0.0871	0.0629	-0.163	-0.109	0.0764	0.1914	0.0013	-0.29	-1.26	-0.45	-0.91	-0.54	-0.05	0.02	0.244	0.328	0.226301	0.350746	0.024779915	0.325965651
2058	P52565	ARHGDIA	Rho GDP dissociation inhibitor alpha	396	ENSG00000141522	17	0.125	0.308	-0.09	-0.07	0.089	-0.57	0.142	0.03	0.179	0.1246	0.0998	-0.039	-0.054	0.0398	0.393	0.1551	0.4066	0.1609	-0.16	-1.15	-0.29	-0.95	-0.48	-0.1	-0.13	0.04	0.383	0.12595	0.328719	-0.127636878	0.456356088
3475	Q9NR45	S	N-acetylneuraminate synthase	54187	ENSG00000095380	25	0.043	0.261	-0.17	-0.18	-0.04	-0.56	0.297	0.32	0.453	-0.056	-0.314	-0.098	0.0012	-0.014	-3E-04	0.289	0.4192	0.301	-0.05	-1.15	-0.4	-0.84	-0.49	-0.18	0.141	0.101	0.48	0.282808	0.312027	-0.01158975	0.323616593
1453	P06727	APOA4	apolipoprotein A4	337	ENSG00000110244	46	-0.17	-0.91	-0.74	-0.76	0.089	-0.1	0.328	0.164	-0.4	-0.749	-0.05	-0.754	-0.658	0.4471	-0.403	-0.265	-0.582	-0.18	0.381	-0.62	0.914	1.351	0.127	0.018	-0.1	-0.3	-0.38	0.225926	-0.43332	0.076441887	-0.509761917
1491	P08133	ANXA6	annexin A6	309	ENSG00000197043	57	-0.09	-0.1	-0.28	-0.1	-0	-0.59	-0.26	0.109	-0.02	-0.284	-0.038	-0.017	0.2047	0.0508	0.2021	0.0245	0.6656	0.2043	-0.26	-0.9	-0.24	-0.67	-0.41	-0.24	0.214	0.132	0.045	0.146711	0.111424	-0.260066134	0.371490424
1492	Q5JR08	RHOC	ras homolog family member C	389	ENSG00000155366	19	-0.02	0.383	0.093	0.16	-0.15	-0.41	0.131	0.038	0.149	0.1448	0.1012	-0.01	0.1511	-0.206	0.197	0.1113	0.3157	0.099	-0.28	-0.87	-0.37	-0.6	-0.44	0.036	-0.36	0.042	0.316	0.098356	0.323144	-0.05878207	0.381925968
1131	O43657	TSPAN6	tetraspanin 6	7105	ENSG00000000003	17	-0.56	0.189	0.377	0.19	-0.1	-0.76	-0.25	-0.62	0.22	-0.078	0.3622	-0.098	0.1483	-0.282	-0.068	-0.084	0.5933	0.1021	-0.51	-0.08	-0.66	-0.52	-0.52	0.485	-0.03	0.085	0.125	0.472787	0.03517	-0.21219932	0.247369426
1456	P06744	GPI	glucose-6-phosphate isomerase	2821	ENSG00000282019	41	0.096	0.138	-0.12	0.129	0.357	-0.35	0.151	-0.02	0.071	0.0012	-0.132	0.1892	-0.078	-0.256	-0.127	0.122	0.0493	-0.164	-0.2	-0.86	-0.22	0.04	-0.36	-0.05	0.088	0.014	-0.04	0.256968	0.226625	0.094877577	0.131746944
1574	P12429	ANXA3	annexin A3	306	ENSG00000138772	38	0.014	0.23	0.182	-0	-0.18	-0.64	0.159	-0.18	0.269	-0.077	0.1405	0.0348	0.081	0.0577	0.0857	-0.006	0.3792	-0.085	-0.08	-0.66	-0.12	-0.8	-0.43	0.006	-0.06	0.111	0.114	0.229788	0.197148	-0.084104532	0.28125277
1612	P14625	HSP90B1	heat shock protein 90 beta family member 1	7184	ENSG00000166598	59	0.38	-0.36	-0.35	0.285	0.402	-0.13	0.034	0.251	0.026	-0.413	0.0165	-0.189	-0.489	0.3415	-0.403	-0.014	-0.572	-0.231	-0.16	0.496	0.237	0.027	-0.35	-0.07	0.245	0.139	-0.54	0.294065	0.056296	0.276743319	-0.220447028
1189	O75110	ATP9A	ATPase phospholipid transporting 9A (putat	10079	ENSG00000054793	53	-0.33	0.266	-0.08	0.081	-0.33	-0.31	0.131	-0.17	0.124	0.264	0.0854	-0.004	0.1306	-0.203	-0.089	0.3387	0.2211	0.2138	-0.41	-0.76	-0.35	-0.67	-0.47	0.067	-0.17	-0.15	0.162	0.081782	0.236709	-0.17552643	0.412235697
2084	P54107	CRISP1	cysteine rich secretory protein 1	167	ENSG00000124812	16	-0.03	-1.45	-0.37	-0.31	-0.61	-0.61	-0.55	0.239	0.436	-0.383	-0.633	3.0953	-0.47	-1.19	-0.408	-0.384	-1.056	-1.281	-0.55	-0.93	-0.7	0.034	-0.59	-1.46	-1.08	-0.16	-2.04	0.561773	0.467852	-0.061790588	0.529642624
1563	P11766	ADH5	alcohol dehydrogenase 5 (class III), chi poly	128	ENSG00000197894	22	0.213	0.042	-0	0.179	-0.04	-0.3	0.221	0.043	0.301	-0.216	-0.022	0.0411	0.0824	-0.111	0.0167	0.1997	0.298	0.2677	-0.4	-1.43	-0.39	-0.64	-0.33	-0.04	-0.02	0.026	0.399	0.146881	0.386479	0.010832955	0.375646484
3776	Q9Y5X9	LIPG	lipase G, endothelial type	9388	ENSG00000101670	22	-0.46	-0.53	-0.44	-0.73	-0.55	0.047	-0.23	-0.07	-0.11	0.3975	0.1131	-0.693	-0.409	-0.75	0.1864	0.078	-0.559	-0.221	0.971	1.173	0.136	0.348	0.37	-0.09	0.004	-0.25	-0.91	0.180739	-0.53722	-0.13560654	-0.40160909
0	A0A024QZX	SERPINB6	serpin family B member 6	5269	ENSG00000124570	25	0.186	0.292	-0.06	0.248	0.005	-0.42	0.206	0.089	0.246	0.078	0.0373	-0.189	0.0299	-0.283	0.1571	0.2642	0.2651	0.2408	-0.3	-1.24	-0.25	-0.79	-0.43	-0.08	0.012	0.094	0.386	0.161284	0.375945	0.021026862	0.354917682
2108	P55072	VCP	valosin containing protein	7415	ENSG00000165280	67	-0.05	0.062	0.011	-0.09	0.368	-0.52	0.114	0.069	0.135	0.0367	-0.008	-0.181	0.0899	-0.178	-0.012	0.3543	0.28	0.0728	-0.29	-0.52	0.029	-0.33	-0.22	0.006	0.338	0.016	0.02	0.491643	0.116475	-0.039178919	0.155653875
2389	Q13217	DNAJC3	DnaJ heat shock protein family (Hsp40) mer	5611	ENSG00000102580	38	-0.18	-0.71	-0.86	-0.65	-0.12	-0.29	-0.24	-0.07	0.404	0.3608	-0.091	-0.319	-0.898	0.5503	-0.267	0.1106	-0.819	0.0959	0.203	0.929	0.223	-0.21	-0.2	-0.12	0.377	-0.12	-0.1	0.286215	-0.40927	-0.159279951	-0.249993758
1403	P03973	SLPI	secretory leukocyte peptidase inhibitor	6590	ENSG00000124107	13	-0.88	-0.57	-0.81	-0.67	-1.36	-0.33	-0.81	-0.17	0.902	0.0287	0.0295	-0.284	-1.191	0.1282	-1.55	0.5002	-1.249	0.5287	-0.91	-0.78	0.308	0.727	-0.1	1.555	-0.75	1.686	-1.73	0.600908	-0.52258	-0.182742793	-0.339834875
1733	P23526	AHCY	adenosylhomocysteinase	191	ENSG00000101444	28	0.222	0.146	-0.13	-0.04	0.179	-0.33	0.058	0.078	0.09	0.2621	0.0017	0.1339	-0.063	-0.121	-0.078	0.1785	-0.049	0.2479	-0.01	-0.76	-0.19	-0.37	-0.11	0.07	0.225	0.076	0.004	0.357016	0.146978	-0.027340882	0.174319261
2231	P63104	YWHAZ	tyrosine 3-monooxygenase/tryptophan 5-mo	7534	ENSG00000164924	26	0.068	0.101	-0.21	0.134	0.093	-0.42	0.324	0.084	0.406	-0.022	-0.012	0.0423	-0.049	-0.201	0.2709	0.3167	0.3425	0.2716	-0.35	-1.46	-0.35	-0.65	-0.32	-0.03	0.076	0.112	0.447	0.202388	0.346077	-0.041323169	0.387399767
3208	Q99536	VAT1	vesicle amine transport 1	10493	ENSG00000108828	19	-0.36	0.326	-0.09	0.227	-0.25	-0.55	0.4	-0.14	0.386	0.1998	0.2233	-0.109	0.0376	-0.225	0.1596	0.295	0.4281	0.1726	-0.06	-0.65	-0.47	-0.64	-0.86	-0.03	0.054	0.037	0.373	0.203003	0.245005	-0.137173158	0.382178431
2290	Q01518	CAP1	cyclase associated actin cytoskeleton regula	10487	ENSG00000131236	32	0.079	0.09	-0.21	0.048	0.102	-0.53	0.177	-0.08	0.202	-0.229	-0.081	-0.101	-0.085	-0.014	0.0637	0.1439	0.1728	0.0156	-0.13	-0.74	-0.08	-0.44	-0.36	-0.08	-0.04	-0	0.162	0.321289	0.175334	-0.001814522	0.177148855
1607	P14550	AKR1A1	aldo-keto reductase family 1 member A1	10327	ENSG00000117448	28	0.024	0.129	-0.17	-0.23	-0.26	-0.66	0.096	-0.05	0.338	0.12	0.0681	0.0693	-0.212	-0.24	0.0974	0.1235	0.3478	0.2592	-0.39	-1.53	-0.42	-0.7	-0.59	-0.16	-0.09	0.023	0.496	0.193818	0.286001	-0.157682093	0.443683517
3345	Q9H0B6	KLC2	kinesin light chain 2	64837	ENSG00000174996	62	0.252	0.463	0.04	-0.25	-0.47	-0.36	0.003	-0.08	0.105	0.3781	0.0394	0.0042	-0.183	-0.06	0.0132	0.1185	0.2271	-0.127	-0.26	-1.26	-0.03	-0.2	-0.35	0.113	-0.32	-0.14	0.237	0.30604	0.21122	-0.079197613	0.290417313
2147	P61019	RAB2A	RAB2A, member RAS oncogene family	5862	ENSG00000104388	21	0.072	0.08	-0.01	0.117	-0.15	-0.32	0.109	0.186	0.147	-0.031	-0.003	-0.203	-0.001	-0.381	0.2277	0.121	0.2763	0.0141	-0.35	-1.12	-0.18	-0.58	-0.39	-0.02	-0.08	0.004	0.331	0.2073	0.289621	0.02392766	0.26569316
2546	Q15907	RAB11B	RAB11B, member RAS oncogene family	9230	ENSG00000185236	17	-0.19	0.439	0.018	0.176	0.215	-0.52	0.233	0.149	0.168	-0.077	0.1042	0.1285	0.3143	-0.184	0.0108	-0.21	0.3549	-0.042	-0.33	-0.97	-0.45	-0.69	-0.47	-0.08	-0.15	0.167	0.004	0.101485	0.405736	0.031282513	0.374453614
1012	O00468	AGRN	agrin	375790	ENSG00000188157	76	-0.51	-0.62	-0.5	-0.13	-0.58	-0.25	-0.05	0.175	-0.37	-0.343	0.2028	0.1723	-0.593	0.8852	-0.023	0.1749	-0.593	0.4063	-0.03	0.076	0.334	-0.45	-0.38	0.163	0.299	1E-04	-0.05	0.230663	-0.31188	-0.348456773	0.036575405
1000	O00299	CLIC1	chloride intracellular channel 1	1192	ENSG00000223639	19	0.21	0.288	-0.06	0.09	-0.09	-0.43	-0.02	-0.11	0.058	0.1109	-0.016	-0.044	0.1298	-0.204	0.0996	0.1854	0.1443	0.2407	-0.23	-1.09	-0.39	-0.45	-0.39	-0.03	-0.09	1E-04	0.169	0.125055	0.270708	-0.078950697	0.349658209

741	F8W022	CD63	CD63 molecule	967	ENSG00000135404	7	-0.42	0.387	0.139	-0.07	-0.28	-0.58	-0.02	-0.34	-0.05	0.1621	0.3665	-0.01	0.105	-0.526	-0.17	-0.05	0.4454	0.1359	-0.48	0.161	-0.6	-0.66	-0.57	0.035	-0.34	-0.25	0.076	0.207327	0.154487	-0.189468306	0.343955335
1328	P00441	SOD1	superoxide dismutase 1	6647	ENSG00000142168	17	0.424	0.445	0.096	-0.1	0.043	-0.66	0.094	-0.03	0.28	-0.224	-0.003	0.1068	-0.074	-0.025	-0.216	0.0472	0.3238	0.0349	-0.3	-1.21	-0.32	-1.02	-0.56	-0.25	-0.07	0.253	0.304	0.175984	0.418716	0.069378813	0.349337014
1463	P07195	LDHB	lactate dehydrogenase B	3945	ENSG00000111716	21	0.023	0.148	-0.21	-0.06	0.155	-0.48	0.325	0.148	-0.17	-0.331	-0.092	0.0298	-0.107	-0.076	0.0354	0.3067	0.3535	-0.249	-0.23	-0.83	-0.51	-0.77	-0.49	-0.09	0.224	0.173	0.423	0.42959	0.219357	6.91379E-05	0.219288357
1637	P16152	CBR1	carbonyl reductase 1	873	ENSG00000159228	23	0.097	0.315	-0.14	-0.13	-0.09	-0.68	-0.02	-0.12	0.221	0.0413	0.2638	0.2128	0.163	-0.154	0.0386	0.1129	0.5683	0.141	-0.14	-1	-0.18	-0.62	-0.81	-0.09	0.135	0.103	0.302	0.17315	0.194518	-0.215356392	0.409874413
711	F5H442	TSG101	tumor susceptibility 101	7251	ENSG00000074319	30	-0	0.494	-0.51	-0.1	0.004	-0.15	-0.08	0.272	-0.31	-0.428	0.247	0.4195	-0.118	-0.147	-0.058	0.2824	0.0512	0.5016	-0.19	-0.13	-0.42	-0.4	-0.25	-0.21	-0.19	0.059	0.647	0.530186	0.076089	-0.126598913	0.202687877
2475	Q14697	GANAB	glucosidase II alpha subunit	23193	ENSG00000089597	51	-0.2	-0.75	-0.12	-0.51	0.114	-0.27	-0.09	0.368	-0.13	0.1535	0.1002	-0.274	-0.627	0.3518	-0.358	0.0754	-0.368	0.1405	0.111	1.298	0.1	-0.23	-0.17	-0.25	0.424	-0.48	-0.22	0.595287	-0.24067	-0.086536047	-0.154130071
1717	P22314	UBA1	ubiquitin like modifier activating enzyme 1	7317	ENSG00000130985	57	0.123	0.275	0.022	0.011	0.047	-0.36	-0.08	-0.01	0.111	0.164	0.1095	0.0934	0.0875	-0.074	0.1042	0.0088	0.1913	0.0451	-0.19	-1.08	-0.24	-0.63	-0.3	0.08	-0.02	0.059	0.213	0.157831	0.249888	-0.065502239	0.315390059
1592	P13693	TPT1	tumor protein, translationally-controlled 1	7178	ENSG00000133112	13	-0.18	0.117	0.021	-0.31	-0.11	-0.33	0.138	-0.06	0.312	-0.011	-0.028	-0.057	-0.683	0.019	-0.442	-0.082	0.3659	0.1493	-0.36	-1.25	-0.09	-0.33	-0.22	-0.1	-0.17	-0.07	0.158	0.444257	0.226981	0.042122896	0.184857879
673	E9PLK3	NPEPPS	aminopeptidase puromycin sensitive	9520	ENSG00000141279	52	0.097	0.118	-0.13	0.018	0.117	-0.39	0.146	0.063	0.317	0.078	-0.094	0.0116	-0.041	-0.179	0.0687	0.2287	0.1914	0.1873	-0.26	-0.99	-0.36	-0.55	-0.38	-0.02	0.055	0.116	0.395	0.217135	0.260715	-0.010691608	0.271406973
236	B7WPD9	KIF26B	kinesin family member 26B	55083	ENSG00000162849	1	-0.1	-1.88	-0.57	-0.4	-0.92	0.622	0.155	-0.8	0.291	0.1262	-0.57	1.2162	-1.964	-1.379	-1.187	-0.557	-1.177	-0.419	-0.55	0.755	1.732	2.264	0.369	0.306	-0.16	-0.62	-1.94	0.327507	-0.63991	0.255935653	-0.895842233
3552	Q9NZM1	MYOF	myoferlin	26509	ENSG00000138119	114	-0.46	-0.06	-0.3	0.133	-0.14	-0.33	-0.07	-0.25	-0.18	0.041	0.2972	0.0487	0.3269	-0.22	0.2859	-0.058	0.3633	0.0522	-0.12	-0.25	-0.24	-0.41	-0.19	0.009	0.004	-0.09	0.198	0.063177	-0.06474	-0.310868181	0.246131041
1933	P43490	NAMPT	nicotinamide phosphoribosyltransferase	10135	ENSG00000105835	33	-0.02	0.508	0.12	-0.23	-0.05	-0.49	-0.07	0.112	0.09	0.0254	-0.021	-0.036	-0.039	-0.152	0.2437	-0.134	0.239	-0.108	-0.52	-0.98	-0.12	-0.43	-0.42	0.105	-0.03	-0.2	0.146	0.205263	0.267113	-0.007498805	0.274611569
3692	Q9UQB8	BAIAP2	BAR/IMD domain containing adaptor protein	10458	ENSG00000175866	43	0.009	0.465	-0.31	0.127	0.249	-0.06	-0.14	0.089	-0.13	-0.381	-0.154	0.2684	-0.018	-0.025	-0.063	0.0537	-0.01	0.3526	-0.17	0.126	-0.47	-0.26	-0.38	-0.5	-0.24	-0.04	0.413	0.358462	0.201303	0.029577282	0.171726102
1832	P32119	PRDX2	peroxiredoxin 2	7001	ENSG00000167815	17	-0.46	-0.14	-0.34	0.038	-0.31	-0.75	0.289	0.06	0.333	-0.34	-0.564	-0.256	-0.432	-0.298	0.1361	0.2438	0.23	0.1083	-0.3	-1.27	-0.43	-0.72	-0.67	-0.35	0.018	0.231	0.325	0.574362	0.208756	-0.011168139	0.219924131
577	D6RI10	SPINK2	serine peptidase inhibitor, Kazal type 2	6691	ENSG00000128040	7	0.444	-0.45	0.561	0.319	-0.1	0.278	-0.17	0.256	-0.17	-0.357	-0.099	0.1805	-0.352	-0.325	0.1271	0.2693	-0.807	-0.254	-0.21	0.723	-0.54	-0.1	-0.04	-0.5	-1.15	-0.37	-1.26	0.21575	0.490729	0.286981313	0.203747465
1556	P11216	PYGB	glycogen phosphorylase B	5834	ENSG00000100994	68	-0.04	-0.04	0.251	0.223	-0.14	-0.32	-0.18	-0.22	-0.07	-0.034	0.1343	0.2803	0.0917	-0.257	-0.009	0.1942	-0.037	0.0525	-0.16	-0.77	-0.25	-0.39	-0.23	-0.3	0.159	0.355	0.021	0.315815	0.114554	-0.106221176	0.220774987
1188	O75083	WDR1	WD repeat domain 1	9948	ENSG00000071127	35	0.086	0.144	0.027	-0.16	0.137	-0.5	0.007	0.147	0.185	0.0312	0.0291	0.1014	0.0033	-0.298	0.0575	-0.024	0.2406	0.1812	-0.29	-1.23	-0.26	-0.29	-0.28	0.192	-0.08	-0.05	0.258	0.282938	0.234196	-0.027478139	0.261673666
1618	P15121	AKR1B1	aldo-keto reductase family 1 member B	231	ENSG00000085662	22	0.77	-0.46	0.171	-0.76	-0.57	-0.23	-0.04	0.353	-0.3	-0.193	-0.028	0.4622	-0.458	-0.183	0.0548	-0.26	-0.139	0.6228	-1.01	-0.39	0.349	-0.4	0.757	-0	0.736	-0.11	-0.14	0.918138	-0.09373	-0.104204144	0.010477959
1423	P04792	HSPB1	heat shock protein family B (small) member	3315	ENSG00000106211	18	0.035	-0.1	-0.21	-0	-0.39	-0.31	0.198	-0.27	0.237	-0.035	-0.022	0.0204	0.1138	0.0704	0.0937	0.1635	0.3249	0.2983	-0.28	-1.08	-0.41	-0.86	-0.47	-0.14	0.052	0.025	0.291	0.122129	0.226389	-0.206201661	0.432591037
2304	Q02809	PLOD1	procollagen-lysine,2-oxoglutarate 5-dioxyger	5351	ENSG00000083444	43	-0.23	-0.64	-0.22	-0.56	-0.13	-0.31	0.029	0.075	0.217	0.077	0.0647	-0.229	-0.539	0.2258	-0.165	0.2623	-0.15	0.127	-0.12	0.826	0.201	0.016	-0.06	-0.15	0.524	-0.03	-0.04	0.219791	-0.32428	-0.159246473	-0.165033034
2815	Q86VP6	CAND1	cullin associated and neddylation dissociate	55832	ENSG00000111530	61	0.029	0.078	-0.07	0.326	0.478	-0.36	-0.08	0.104	0.176	0.1306	-0.114	-0.072	-0.105	-0.294	-0.097	0.0377	-0.008	0.1428	-0.26	-0.58	-0.17	-0.48	-0.42	-0.07	-0.01	0.034	0.062	0.140831	0.286098	0.11799235	0.168105224
2672	Q66K79	CPZ	carboxypeptidase Z	8532	ENSG00000109625	31	-0.37	-0.27	0.059	-0.74	-0.08	-0.16	-0.13	0.255	0.003	0.1827	-0.277	-0.477	-0.607	0.3023	-0.098	-0.944	-0.586	-0.942	0.057	1.249	0.928	-0.22	0.69	0.036	0.128	0.046	-0.39	0.108515	-0.44052	0.223277537	-0.663801122
1984	P49189	ALDH9A1	aldehyde dehydrogenase 9 family member A	223	ENSG00000143149	36	0.289	0.194	-0.08	-0.01	-0.17	-0.77	0.081	0.052	0.263	-0.061	0.0337	0.0031	0.144	-0.205	0.0046	0.3627	0.1795	0.1777	-0.3	-1.19	-0.22	-0.81	-0.5	-0.06	0.101	0.183	0.24	0.235634	0.267232	-0.087238847	0.354470752
1284	O95394	PGM3	phosphoglucomutase 3	5238	ENSG00000013375	34	-0.01	0.042	-0.11	-0.17	-0.1	-0.54	-0.18	-0.34	0.081	-0.072	0.0755	0.1791	-0.086	-0.071	0.1571	0.0888	0.0729	-0.102	-0.18	-1.02	-0.25	-0.63	-0.39	0.119	0.069	-0.26	0.176	0.207775	0.115416	-0.174903682	0.290319997
1560	P11279	LAMP1	lysosomal associated membrane protein 1	3916	ENSG00000185896	10	-0.49	0.455	0.498	-0.2	-0.47	-0.67	-0.32	-0.83	-0.21	0.4584	0.5149	-0.333	0.0602	-0.685	0.2617	-0.239	0.4401	-0.121	-0.23	-0.45	-0.56	-0.73	-0.53	0.238	-0.36	-0.23	-0.21	0.268945	0.090747	-0.288103979	0.378850552
3477	Q9NR99	MXRA5	matrix remodeling associated 5	25878	ENSG00000101825	77	-0.69	-0.87	-0.23	-0.5	-0.36	0.559	-0.02	-0.4	-0.61	-0.055	0.845	-0.177	-0.533	0.735	-0.104	-0.52	-0.401	-0.293	-0.79	-0.75	0.032	0.341	0.037	-0.13	1.126	-0.37	-0.38	0.580607	-0.24918	-0.290999543	0.041820177
2689	Q6IWH7	ANO7	anoctamin 7	50636	ENSG00000146205	31	0.089	0.348	0.009	-0.32	-0.56	-0.04	0.122	-0.3	0.275	0.1927	0.2957	-0.08	0.0426	-0.207	-0.124	-0.253	0.7173	-0.328	-0.07	-0.58	-0.1	-0.38	-0.12	0.417	-0.56	-0.13	0.012	0.565666	0.124358	-0.069827424	0.194185809
806	P0DP25	CALM1;CA	calmodulin 1;calmodulin 3;calmodulin 2	801;808;805	ENSG00000198668	13	0.726	0.514	0.279	0.106	0.205	-0.39	-1.47	-1.34	-0.77	0.2056	0.3908	0.3699	0.2428	-0.061	0.2209	-0.866	-0.406	-1.415	-0.04	-0.66	-0.14	-0.46	-0.27	0.054	-0.7	-0.44	-0.8	0.799916	0.145375	-0.091986298	0.237361001
3470	Q9NQX4	MYO5C	myosin VC	55930	ENSG00000128833	90	0.115	0.26	0.18	0.112	-0.44	-0.26	-0.06	-0.21	-0.02	0.0741	0.166	-0.022	0.2709	-0.23	0.0313	0.0367	0.3595	-0.102	-0.31	-0.72	0.034	-0.43	-0.34	0.241	-0.06	-0.29	0.052	0.211401	0.167207	-0.100721468	0.267928571
1460	P06865	HEXA	hexosaminidase subunit alpha	3073	ENSG00000213614	29	-0.03	-0.78	-0.42	-0.39	-0.23	-0.26	-0.2	0.022	0.069	0.2152	-0.045	0.0928	-0.459	0.4752	-0.258	-0.099	-0.546	-0.399	0.204	0.827	0.234	0.192	0.165	-0.22	0.345	-0.11	-0.48	0.202882	-0.37443	-0.13222272	-0.242207751
3245	Q9BR76	CORO1B	coronin 1B	57175	ENSG00000172725	29	0.012	-0.06	-0.02	-0.13	-0.15	-0.78	0.029	-0.03	0.313	0.0954	0.1589	-0.169	0.0021	-0.086	-0.088	0.1903	0.2871	0.3471	-0.12	-0.63	0.137	-0.84	-0.47	0.187	-0.05	0.119	0.387	0.451203	0.052465	-0.17213728	0.224602329
1324	P00338	LDHA	lactate dehydrogenase A	3939	ENSG00000134333	22	-0.18	-0.07	0.033	0.086	0.168	-0.67	0.439	0.05	0.09	0.2338	0.0226	0.2106	-0.309	-0.002	-0.373	0.0226	0.1642	-0.315	-0.3	-0.54	-0.03	-0.29	-0.12	0.053	0.174	0.036	-0.08	0.725193	0.116537	0.031381492	0.085155374
1207	O75369	FLNB	filamin B	2317	ENSG00000136068	99	0.071	0.138	-0.1	-0.08	0.028	-0.4	-0.1	-0.13	0.133	0.0951	-0.021	-0.086	0.0449	-0.161	0.1907	0.049	0.082	-0.002	-0.24	-0.68	-0.22	-0.45	-0.1	0.104	0.072	0.065	0.134	0.375657	0.096248	-0.070078156	0.166326127
1727	P23284	PPIB	peptidylprolyl isomerase B	5479	ENSG00000166794	22	0.438	-0.83	-0.24	-0.05	-0.43	0.256	0.157	0.169	0.008	0.3489	-0.226	-0.463	-0.801	0.3759	-0.435	0.4997	-0.518	-0.213	0.441	0.093	-0.01	0.439	0.258	-0.53	0.315	0.026	-0.58	0.684593	-0.10655	0.102571168	-0.209120273
1604	P14384	CPM	carboxypeptidase M	1368	ENSG00000135678	22	-0.03	0.307	-0.64	0.087	0.513	-0.61	-0.54	0.26	0.044	-0.423	0.8457	0.0113	-0.28	-0.454	0.2303	0.124	-0.312	0.4408	-0.42	0.181	-0.41	-0.92	-0.07	-0.26	0.222	0.059	0.069	0.729931	0.103957	-0.088139276	0.192096337
2088	P54289	CACNA2D	calcium voltage-gated channel auxiliary subu	781	ENSG00000153956	43	0.106	0.39	-0.28	-0.41	-0.03	-0.11	-0.38	0.431	0.024	0.3906	0.4569	-0.156	-0.178	-0.208	-0.138	0.1002	0.3522	0.4279	-0.55	-1.15	0.235	-0.86	-0.22	0.02	-0.29	-0.29	-0.63	0.100074	0.386151	-0.145143071	0.531293873
2063	P52788	SMS	spermine synthase	6611	ENSG00000102172	30	0.17	0.621	-0.09	0.043	-0.16	-0.28	-0.27	-0.07	0.277	0.3901	-0.019	0.1056	0.1766	-0.107	0.0035	0.0783	-0.175	-0.038	-0.24	-1.48	-0.57	-0.98	-0.29	0.325	-0.24	0.11	0.14	0.193518	0.385682	-0.019126256	0.40480857
1659	P17931	LGALS3	galectin 3	3958	ENSG00000131981	15	-0.03	0.218	-0.07	-0.01	-0.35	0.04	0.285	-0.01	0.405	-0.026	0.2932	0.0781	0.0606	0.1243	-0.018	0.3492	0.4988	0.4151	-0.45	-1.18	-0.23	-0.53	-0.37	-0	0.003	0.008	0.5	0.124627	0.304328	-0.144319108	0.448647037
1722	P22732	SLC2A5	solute carrier family 2 member 5	6518	ENSG00000142583	19	0.335	0.304	-1.87	0.307	-0.8	0.366	-0.63	0.308	-0.74	0.1678	-0.117	0.1667	-0.121	0.1014	0.6786	0.527	0.0058	-0.423	0.047	-1.16	-0.96	-0.56	0.27	-0.07	-1.85	-0.86	0.082	0.24189	0.295314	-0.377684188	0.672998405
2257	P78371	CCT2	chaperonin containing TCP1 subunit 2	10576	ENSG00000166226	45	0.063	-0.05	0.009	0.348	0.276	-0.23	0.231	0.235	0.247	-0.044	-0.058	-0.043	-0.047	-0.22	-0.04	0.2328	0.1566	0.0704	-0.28	-0.76	-0.15	-0.32	-0.26	0.008	0.181	0.023	0.15	0.149536	0.282176	0.124825863	0.157349933
3271	Q9BUD6	SPON2	spondin 2	10417	ENSG00000159674	16	1.022	0.642	-1.35	-1.01	-0.03	0.463	-0.93	0.285	-0.09	0.1942	-1.189	-0.526	-0.046	0.2072	0.1425	0.0331	0.687	0.0034	0.291	-0.28	-0.67	-1.4	-1.12	-0.57	-0.4	-0.94	0.503	0.463986	0.400054	-0.054605176	0.454659354
1992	P49368	CCT3	chaperonin containing TCP1 subunit 3	7203	ENSG00000163468	37	0.071	0.054	0.039	0.336	0.278	-0.33	0.201	0.226	0.208	-0.036	-0.11	-0.114	-0.143	-0.103	-0.028	0.2788	0.0585	0.1385	-0.22	-1.01	-0.22	-0.58	-0.3	-0.01	0.205	0.079	-0.06	0.127778	0.357212	0.126884491	0.230327612
1721	P22694	PRKACB	protein kinase cAMP-activated catalytic subu	5567	ENSG00000142875	26	0.209	0.28	-0.21	0.411	0.26	-0.32	-0.02	-0.06	0.038	-0.205	0.117	-0.251	0.1713	-0.403	-0.042	0.2727	0.1914	0.3539	-0.28	-0.76	-0.28	-0.64	-0.44	-0.12	-0.2	-0.24	0.119	0.092036	0.381361	0.041599632	0.339761857
3641	Q9UK41	VPS28	VPS28 subunit of ESCRT-I;VPS28, ESCRT	51160	ENSG00000160948	20	-0.05	0.471	-0.68	-0.06	-0.14	-0.31	-0.19	0.227	-0.5	-0.579	0.087	0.3519	-0.246	-0.202	-0.1	0.139	-0.104	0.3075	-0.21	-0.1	-0.77	-0.41	-0.49	-0.46	-0.47	-0.2	0.61	0.499739	0.144543	-0.096373984	0.240917448
3439	Q9HC84	MUC5B	mucin 5B, oligomeric mucus/gel-forming	727897	ENSG00000117983	76	-1.69	-1.47	-1.52	-1.24	-0.34	-0.99	-0.84	0.018	-1.04	-1.709	-1.933	-0.833	1.5117	0.1231	-0.909	-0.949	1.382	-1.325	-1.59	0.027	-1.2	0.839	1.428	-0.66	1.867	-1.24	-0.59	0.391528	-0.88576	-0.495261381	-0.390500481
1953	P46940	IQGAP1	IQ motif containing GTPase activating prote	8826	ENSG00000140575	69	0.058	0.145	-0.08	0.073	-0.04	-0.37	0.095	0.053	0.266	0.0211	0.0303	0.0855	0.019	-0.03	0.1016	0.145	0.031	0.0185	-0.29	-0.76	-0.16	-0.34	-0.34	-0.13	0.223	0.157	0.389	0.352832	0.160844	-0.025545345	0.186389441
2788	Q7Z4W1	DCXR	dicarbonyl and L-xylulose reductase	51181	ENSG00000169738	17	0.233	0.202	-0.05	-0.09	-0.02	-0.29	0.06	-0.01	0.32	-0.006	0.0409	0.0852	0.0077	0.1578	-0.106	0.1061	-0.087	0.1093	-0.33	-1.26	-0.35	-0.67	-0.51	-0.15	0.003	0.31	0.296	0.172654	0.335393	0.004580464	0.330812695
1997	P49588	AARS;AAF	alanyl-tRNA synthetase;alanyl-tRNA synthet	16	ENSG00000090861	57	-0.02	0.039	0.007	-0.04	-0	-0.48	-0.14	-0.09	0.047	0.0095	-0.061	0.0705	0.0539	-0.182	0.0687	-0.1	0.1379	0.0332	-0.21	-0.78	-0.17	-0.6	-0.29	0.053	0.127	0.007	0.1	0.313208	0.119747	-0.079833008	0.199580309
2356	Q10567	AP1B1	adaptor related protein complex 1 subunit be	162	ENSG00000100280	55	0.144	0.085	-0.05	-0.02	0.115	-0.36	-0.12	-0.12	0.126	0.0294	-0.044	-0.025	0.0371	-0.176	0.0343	0.0081	0.1224	0.1229	-0.17	-0.71	-0.24	-0.5	-0.22	0.108	-0.01	-0.03	0.123	0.225615	0.16298	-0.034218561	0.197198609
3106	Q96C24	SYTL4	synaptotagmin like 4	94121	ENSG00000102362	46	0.129	0.377	-0.15	-0.05	-0.23	-0.37	-0.12	-0.26	0.429	-0.094	0.0674	0.0263	0.0211	-0.143	0.3387	0.3745	0.2161	0.1916	-0.28	-0.57	-0.33	-0.64	-0.5	-0.1	-0.09	0.16	0.545	0.235298	0.172695	-0.138088551	0.310783556
2732	Q6UXI9	NPNT	nephronectin	255743	ENSG00000168743	30	0.471	-0.06	-0.05	-0.13	0.034	-0.24	-0.09	0.177	0.337	-0.105	-0.031	0.1402	-0.086	-0.005	-0.421	0.5655	0.5691	0.431	-0.53	-0.36	-0.17	-0.67	-1.07	-0.07	0.162	-0.32	0.093	0.120945	0.377738	-0.066723857	0.444461576
1470	P07384	CAPN1	calpain 1	823	ENSG00000014216	47	-0	0.17	-0.12	-0	-0.23	-0.5	0.07	-0.07	0.383	0.2048	0.0778	-0.008	0.055	-0.288	0.0726	0.1843	0.1945	-0.043	-0.16	-0.85	-0.25	-0.37	-0.38	-0.1	0.053	0.174	0.215	0.311487	0.151711	-0.084334625	0.236045879
3389	Q9H4A4	RNPEP	arginyl aminopeptidase	6051	ENSG00000176393	36	0.27	0.415	-0.04	-0.23	0.156	-0.35	-0.03	0.013	0.455	-0.005	-0.161	0.0774	0.3246	-0.26	0.3065	-0.142	0.1737	0.1735	-0.15	-1.07	-0.34	-0.55	-0.18	0.144	0.216	-0.05	0.186	0.286103	0.272803	0.01987617	0.252926432
1845	P34913	EPHX2	epoxide hydrolase 2	2053	ENSG00000120915	39	-0.08	0.408	-0	-0.33	0.019	-1.04	-0.19	0.375	0.068	0.3603	0.0746	-0.051	0.088	-0.473	0.3248	-0.237	0.2684	0.3185	0.137	-1.09	-0.39	-0.51	-0.42	0.093	0.037	-0.14	0.126	0.390502	0.153438	-0.162162283	0.315599821
3236	Q99985	SEMA3C	semaphorin 3C	10512	ENSG00000075223	33	0.123	-0.19	0.109	-0.31	0.031	-0.29	-0.28	0.301	0.423	-0.088	-0.4	-0.106	-0.351	-0.711	-0.619	0.6487	0.8655	0.6934	-0.06	0.307	0.038	-0.49	-1.05	-0	0.143	-0.15	0.226	0.898428	0.106637	-0.000840899	0.107478308
2303	Q02790	FKBP4	FK506 binding protein 4;FKBP prolyl isomer	2288	ENSG00000004478	31	0.205	0.184	0.045	-0.2	0.095	-0.45	0.043	-0.13	0.082	0.1115	0.0649	0.0956	0.1249	-0.056	-0.016	-0.076	0.2462	0.0998	-0.12	-1.1	-0.26	-0.75	-0.33	0.103	-0.04	-0.13	0.109	0.144484	0.267325	-0.079748877	0.347074207
866	H3BVD9	SPINT1	serine peptidase inhibitor, Kunitz type 1	6692	ENSG00000166145	22	-0.62	0.272	-0.45	-0.1	-0.51	-0.42	-0.02	0.068	0.147	0.2904	0.3558	0.4229	-0.348	-0.234	-0.033	-0.079	0.1735	0.6163	-0.66	-0.65	-0.38	-0.19	-0.4	-0.05	-0.2	0.186	0.31	0.194119	0.045193	-0.310505724	0.355698738
1708	P21399	ACO1	aconitase 1	48	ENSG00000122729	50	0.243	0.208	0.018	-0.16	-0.01	-0.46	0.119	0.129	0.189	-0.054	0.0289	0.0631	0.0419	-0.164	0.0368	0.1472	0.162	0.1051	-0.14	-1.11	-0.16	-0.44	-0.4	0.046	0.05	0.086	0.206	0.256356	0.237267	-0.010080611	0.247347121
1959	P47895	ALDH1A3	aldehyde dehydrogenase 1 family member A	220	ENSG00000184254	33	-0	0.219	-0.1	-0.07	0.13	-0.59	-0.03	-0.05	0.391	-0.044	-0.124	0.1543	0.1079	-0.249	-0.179	0.2219	0.0177	0.0575	-0.26	-1.16	-0.24	-0.26	-0.47	0.124	0.156	0.097	0.128	0.442253	0.198041	-0.007321616	0.205363057
1848	P34932	HSPA4	heat shock protein family A (Hsp70) membe	3308	ENSG00000170606	55	0.125	0.12	-0.07	0.012	0.188	-0.26	0.019	0.149	0.183	0.1475	0.061	-0.014	-0.005	-0.001	0.1186	0.1476	0.2341	0.4131	-0.03	-0.71	-0.19	-0.45	-0.35	-0.01	-0.07	-0.07	0.226	0.094851	0.234794	-0.070908911	0.305702554
1134	O43707	ACTN4	actinin alpha 4	81	ENSG00000130402	62	0.067	0.098	0.016	-0.16	0.074	-0.46	0.034	-0.17	0.084	0.2853	0.2346	-0.019	0.0816	-0.11	-0.012	0.0794	0.0431	-0.096	-0.16	-0.72	0.1	-0.25	-0.18	0.055	0.11	0.055	0.054	0.426538	0.058463	-0.10034644	0.158809078
2305	Q02818	NUCB1	nucleobindin 1	4924	ENSG00000104805	50	-0.25	-0.69	-0.42	-0.84	-0.08	-0.18	-0.09	0.062	0.182	0.4981	-0.019	-0.384	-0.583	0.7183	-0.111	-0.262	-0.807	-0.423	0.342	1.194	0.318	-0.55	0.172	-0.3	0.347	-0.24	-0.04	0.336661	-0.39451	-0.103023105	-0.291490222
2405	Q13438	OS9	OS9 endoplasmic reticulum lectin;OS9, end	10956	ENSG00000135506	45	0.025	-0.82	-0.32	-0.54	-0.31	-0.04	0.113	0.308	0.074	0.1949	-0.157	-0.286	-0.501	0.3931	-0.178	0.0799	-0.514	-0.269	0.565	0.937	0.432	-0.18	0.247	-0.18	0.593	-0.19	-0.08	0.172363	-0.40697	-0.032254392	-0.374714433
1977	P48643	CCT5	chaperonin containing TCP1 subunit 5	22948	ENSG00000150753	44	0.121	0.034	0.023	0.373	0.265	-0.37	0.075	0.091	0.127	-0.018	-0.027	-0.104	-0.164	-0.251	-0.023	0.1946	0.0882	-0.001	-0.23	-0.95	-0.14	-0.5	-0.37	-0.08	0.162	-0.1	-0.06	0.122333	0.333213	0.116003512	0.217209862
1683	P20073	ANXA7	annexin A7	310	ENSG00000138279	25	-0.45	-0.05	-0.39	0.04	-0.22	-0.42	0.297	0.143	0.106	-0.304	-0.108	-0.685	0.205	-0.43	0.1362	0.3863	0.0935	0.2624	-0.08	-0.05	-0.2	-0.4	-0.67	-0.19	0.166	0.013	0.382	0.928691	0.009647	-0.055352033	0.064999106
1476	P07686	HEXB	hexosaminidase subunit beta	3074	ENSG00000049860	29	-0.05	-0.49	1E-03	-0.12	-0.13	-0.28	-0.16	0.326	0.205	0.4319	-0.293	0.4276	-0.231	-0.243	-0.411	-0.088	-0.394	-0.464	0.167	0.567	-0.08	0.184	0.212	-0.26	0.105	0.194	-0.69	0.603824	-0.12127	0.063620714	-0.184888293
1551	P11021	HSPA5	heat shock protein family A (Hsp70) membe	3309	ENSG00000044574	45	0.018	-0.3	-0.17	0.25	0.465	-0.11	0.44	0.612	0.005	-0.183	-0.12	-0.213	-0.52	0.0246	-0.25	-0.155	-0.766	-0.568	-0.14	0.897	0.105	-0.14	-0.18	-0.45	0.364	-0.26	-0.37	0.149447	0.15407	0.439769554	-0.285699829
3282	Q9BV36	MLPH	melanophilin	79083	ENSG00000115648	43	0.057	0.295	0.056	-0.21	-0.13	-0.39	-0	-0.32	0.314	0.1191	-0.032	-0.125	-0.051	-0.267	-0.026	0.2494	-0.111	0.1474	-0.39	-0.35	-0.03	-0.62	-0.52	0.138	-0.16	-0.07	0.204	0.362953	0.162547	-0.026010308	0.188556989
1854	P35241	RDX	radixin	5962	ENSG00000137710	55	0.1	0.133	-0.31	0.04	0.247	-0.32	-0.01	0.018	0.017	-0.314	0.0515	0.3989	-0.083	-0.2	0.13	0.1756	-0.051	0.3848	-0.18	0.37	0.185	-0.29	-0.46	-0.44	-0.2	0.055	0.393	0.743799	0.05344	-0.063705833	0.117145712
2206	P62820	RAB1A	RAB1A, member RAS oncogene family	5861	ENSG00000138069	23	-0.11	0.325	-0.02	-0.01	-0.19	-0.38	0.193	0.106	0.202	0.1604	0.055	-0.116	0.174	-0.218	0.2077	0.0479	0.2794	0.1658	-0.37	-1.36	-0.39	-0.57	-0.36	0.165	-0.07	-0.02	0.428	0.2025	0.294093	-0.071346483	0.365439436
2090	P54578	USP14	ubiquitin specific peptidase 14	9097	ENSG00000101557	39	0.235	0.289	0.087	-0.11	0.214	-0.48	0.021	0.111	0.209	0.4042	0.2066	0.1724	0.0319	-0.023	0.246	-0.028	0.3354	0.1964	-0.1	-1.03	-0.4	-0.57	-0.31	0.122	-0.21	0.055	0.105	0.083685	0.325214	-0.107193405	0.432407706
802	H0Y4U4	PAEP	progestagen associated endometrial protein	5047	ENSG00000122133	14	0.471	-2.16	-0.98	0.255	-1.48	-0.74	0.13	0.56	0.328	0.8645	0.5056	-0.308	-0.489	-1.202	-0.137	1.0879	0.1375	0.3526	-0.84	1.203	-0.32	-0.91	0.245	-1.88	0.576	-1.06	-1.11	0.526855	0.052621	-0.491838858	0.544460058
1785	P29144	TPP2	tripeptidyl peptidase 2	7174	ENSG00000134900	49	-0.1	-0.03	-0.03	0.62	0.793	-0.35	0.239	0.231	0.092	0.0754	-0.185	-0.365	-0.2	-0.326	-0.33	0.4364	-0.276	0.2613	-0.73	-0.23	-0.15	-0.51	-0.3	-0.37	0.193	-0.23	-0.25	0.117492	0.450832	0.263941398	0.186890148
2458	Q14393	GAS6	growth arrest specific 6	2621	ENSG00000183087	28	-0.51	-0.56	-0.5	-0.58	-0.16	-0.43	0.358	0.106	0.404	-0.111	-0.104	-0.046	-0.561	0.5079	-0.188	0.0766	-0.481	-0.061	-0.14	0.014	-0.45	-0.46	-0.3	-0.33	0.78	0.099	-0.1	0.851566	-0.11063	-0.101493259	-0.009136536
2916	Q8N9U0	TC2N	tandem C2 domains, nuclear	123036	ENSG00000276776	29	0.16	0.251	-0.06	0.106	-0.31	-0.42	0.085	-0.24	0.103	0.1923	0.1496	0.0688	0.199	-0.156	-0.113	0.095	0.1976	-0.04	-0.37	-0.9	-0.26	-0.42	-0.54	0.338	-0.42	-0.24	0.326	0.165327	0.239022	-0.1024885	0.341510916
783	G3V5Z7	PSMA6	proteasome subunit alpha 6	5687	ENSG00000100902	20	-0.13	-0.23	0.011	-0.13	0.328	-0.67	0.202	-0.02	0.044	0.0619	-0.282	-0.097	-0.14	-0.179	-0.382	0.6159	0.2754	0.3948	-0.45	0.038	-0.3	0.078	-0.06	-0.22	0.643	0.164	-0.27	0.868067	-0.02437	-0.095497985	0.071130598
592	E5RJD8	TBCA	tubulin folding cofactor A	6902	ENSG00000171530	18	-0.07	0.17	-0.11	-0.23	-0.16	-0.67	0.484	0.118	0.452	0.0981	-0.038	-0.012	-0.091	-0.197	-0.069	0.4613	0.3486	0.1495	-0.21	-0.77	-0.35	-0.25	-0.37	-0.13	-0.03	0.099	0.31	0.338978	0.187222	-0.075181489	0.262403959
2032	P50995	ANXA11	annexin A11	311	ENSG00000122359	32	-0.47	0.189	-0.43	0.119	0.086	-0.21	0.216	0.186	0.123	-0.191	-0.141	0.1437	0.2227	-0.265	0.0234	0.6457	0.1832	0.6703	-0.03	-0.26	-0.73	-0.51	-0.65	-0.37	-0.12	-0.01	0.22	0.144717	0.25315	-0.163537022	0.416687445
3395	Q9H4G4	GLIPR2	GLI pathogenesis related 2	152007	ENSG00000122694	11	-0.87	0.756	-0.32	0.224	-0.14	-0.15	-0.16	-0.14	-0.46	-0.318	0.517	-0.149	-0.011	0.1062	0.1626	0.2461	0.4879	-0.006	-0.64	-0.55	-0.52	-0.51	-0.82	-0.39	-0.19	-0.1	0.388	0.146808	0.231247	-0.253256784	0.484503871

215	A0A0D9SG(	MYO6	myosin VI	4646	ENSG00000196586	66	0.026	0.302	-0.16	0.063	0.014	-0.28	0.033	-0.1	0.229	0.0808	-0.073	0.0756	-0.152	-0.365	0.1395	0.1464	0.202	-0.035	-0.13	-0.7	-0.17	-0.41	-0.27	0.042	0.037	0.001	0.274	0.404374	0.161222	0.0121734	0.149048642
1783	P28838	LAP3	leucine aminopeptidase 3	51056	ENSG00000002549	33	0.123	0.095	-0.06	-0.11	-0.07	-0.38	0.248	0.011	-0.01	0.2421	-0.038	0.2405	-0.011	-0.159	-0.094	0.3263	-0.067	-0.151	-0.19	-0.55	-0.07	-0.08	-0.01	-0.12	0.415	0.283	0.048	0.878916	0.013899	-0.048835943	0.06273495
3504	Q9NSK0	KLC4	kinesin light chain 4	89953	ENSG00000137171	46	0.071	0.232	-0.2	-0.02	-0.15	-0.42	0.135	-0.11	0.127	0.1569	0.0679	-0.006	-0.133	-0.078	0.047	0.0525	0.1847	-0.142	-0.19	-0.9	0.041	-0.31	-0.54	-0.09	0.107	-0.08	0.355	0.415782	0.142082	-0.053712385	0.195793885
3780	Q9Y5Z4	HEBP2	heme binding protein 2	23593	ENSG00000051620	18	0.286	0.605	0.091	0.025	-0.03	-0.3	0.247	-0.09	0.378	0.4416	0.2669	0.1311	0.0708	0.1019	0.1648	-0.296	0.3384	-0.496	0.027	-1.16	-0.26	-0.48	-0.35	0.005	0.034	0.053	0.165	0.205326	0.355074	0.055265894	0.299807731
2141	P60953	CDC42	cell division cycle 42	998	ENSG00000070831	17	0.247	0.534	0.226	0.219	-0.06	-0.36	0.256	-0.02	0.168	0.2291	0.3395	0.1178	0.2914	-0.262	0.1191	0.1593	0.3217	0.1568	-0.45	-0.93	-0.12	-0.58	-0.34	0.225	-0.18	-0.1	0.291	0.098103	0.376059	-0.029503543	0.405562807
1976	P48637	GSS	glutathione synthetase	2937	ENSG00000100983	33	0.221	0.122	0.074	-0.08	0.122	-0.53	0.263	0.23	0.289	0.0359	-0.05	0.1933	-0.031	-0.277	0.1353	0.1139	0.1535	0.1027	-0.2	-0.58	-0.12	-0.64	-0.2	-0.13	0.012	0.29	0.098	0.230277	0.242593	0.036867563	0.205725462
1562	P11717	IGF2R	insulin like growth factor 2 receptor	3482	ENSG00000197081	74	-0.52	-0.33	-0.45	-0.26	-0.23	-0.13	-0.15	0.076	0.136	0.5624	0.1644	-0.006	-0.449	0.0702	-0.301	0.8437	0.0095	0.3449	-0.31	0.134	-0.2	-0.11	-0.13	-0.21	0.398	0.332	-0.04	0.205251	-0.19184	-0.344704161	0.152861148
1545	P10599	TXN	thioredoxin	7295	ENSG00000136810	11	0.12	0.471	0.112	-0.06	-0.06	-0.6	0.546	0.176	0.518	0.2193	0.0746	0.0065	-0.024	-0.071	0.1061	0.5309	0.5713	0.3291	-0.07	-1.23	-0.19	-0.86	-0.4	0.036	-0.04	0.206	0.496	0.202883	0.365108	-0.05685815	0.4219663
2142	P60981	DSTN	destrin, actin depolymerizing factor	11034	ENSG00000125868	20	-0.1	0.038	-0.03	-0.04	-0.22	-0.51	0.162	0.013	0.35	0.1192	0.0347	-0.091	0.1326	-0.129	0.2173	0.095	0.3814	0.2109	-0.24	-0.67	-0.25	-0.49	-0.39	0.114	-0.14	0.082	0.248	0.170422	0.156393	-0.145173592	0.301566281
2168	P61586	RHOA	ras homolog family member A	387	ENSG00000067560	16	0.126	0.482	0.214	-0	-0.05	-0.32	-0.55	-0.68	-0.26	0.2454	0.315	0.1407	0.2577	-0.133	0.1511	-0.341	0.1517	-0.424	-0.26	-0.93	-0.38	-0.6	-0.34	0.184	-0.67	-0.27	-0.18	0.149335	0.264718	-0.157001003	0.421718847
1089	O15296	ALOX15B	arachidonate 15-lipoxygenase type B;arachi	247	ENSG00000179593	35	0.244	0.486	0.222	-0.78	-0.06	-0.62	-0.1	0.069	0.209	0.3982	-0.352	-0.514	0.3896	-0.306	-0.195	0.214	-0.324	0.8786	-0.51	-1.14	-0.23	-0.54	1E-03	0.332	0.207	-0.15	0.037	0.629281	0.183063	-0.05887939	0.241942513
3365	Q9H223	EHD4	EH domain containing 4	30844	ENSG00000103966	40	-0.14	0.144	-0.24	0.329	0.023	-0.32	0.096	-0.12	0.111	0.1333	0.0332	-0.071	0.0435	-0.268	0.12	0.197	0.1366	0.0968	-0.29	-0.5	-0.27	-0.19	-0.28	0.347	-0.1	-0.1	0.219	0.369404	0.11735	-0.059237719	0.176587231
2550	Q16348	SLC15A2	solute carrier family 15 member 2	6565	ENSG00000163406	21	-0.33	1.091	-0.42	-0.31	-0.07	-0.36	-0.47	-0.09	-0.47	-0.188	0.4553	-0.093	0.285	-0.736	0.192	0.3473	0.2952	0.1972	0.087	-0.21	-0.63	-0.71	-0.61	-0.44	-0.18	-0.11	-0.1	0.24671	0.163758	-0.241458131	0.405216368
1462	P07108	DBI	diazepam binding inhibitor, acyl-CoA binding	1622	ENSG00000155368	11	0.598	0.963	0.291	0.032	0.621	-0.6	-1.15	-1.31	-1.13	0.2955	0.4518	0.1447	0.5757	-0.003	0.1684	-0.058	-0.041	-1.16	-0.15	-1.24	-0.26	-0.64	-0.28	0.202	-1.23	-0.83	-0.57	0.315588	0.367884	-0.228553984	0.596438072
2893	Q8N335	GPD1L	glycerol-3-phosphate dehydrogenase 1 like	23171	ENSG00000152642	28	-0.02	0.2	-0.07	0.088	0.156	-0.48	0.18	0.06	0.18	0.3127	-0.146	-0.163	-0.061	-0.038	0.0726	0.285	0.3706	0.0891	-0.03	-1.15	-0.51	-0.54	-0.34	0.35	-0.25	0.16	0.603	0.392734	0.223122	-0.047708853	0.270831285
899	I3L0N3	NSF	N-ethylmaleimide sensitive factor, vesicle fu	4905	ENSG00000073969	48	0.132	0.095	-0.05	0.13	-0.05	-0.27	0.04	0.146	0.219	0.1418	-0.066	0.0998	0.1867	-0.241	-0.005	0.0539	0.0846	0.0089	-0.15	-0.74	-0.11	-0.46	-0.32	0.053	-0.05	-0.1	0.189	0.152413	0.229638	0.013622577	0.216015805
1123	O43581	SYT7	synaptotagmin 7	9066	ENSG00000011347	35	-0.09	0.275	-0.08	-0.11	-0.22	-0.19	-0.12	-0.06	0.135	0.0517	0.1214	-0.115	0.1883	-0.28	0.1137	0.1864	0.3124	0.0653	-0.15	-0.34	-0.21	-0.34	-0.32	0.134	-0.13	-0.12	0.251	0.204824	0.084165	-0.123503069	0.207667639
1605	P14410	SI	sucrase-isomaltase	6476	ENSG00000090402	72	0.543	0.237	-0.63	-0.07	0.493	-0.89	-0.6	-0.03	-0.51	0.4484	-0.772	0.3935	-0.039	-0.325	0.0788	-0.099	0.4771	0.9854	0.221	0.813	-0.01	-1.21	-0.21	-0.5	-0.74	-0.25	-0.06	0.517379	0.052833	-0.288772144	0.341605328
2137	P60842	EIF4A1	eukaryotic translation initiation factor 4A1	1973	ENSG00000161960	25	0.125	0.026	-0.1	-0.06	-0.06	-0.32	0.057	-0.03	0.188	-0.037	-0.011	0.077	-0.047	-0.126	0.047	-0.084	0.131	0.0689	-0.16	-0.88	-0.2	-0.52	-0.2	0.021	0.038	-0.03	0.255	0.303063	0.168732	-0.020822409	0.18955438
2179	P61981	YWHAG	tyrosine 3-monooxygenase/tryptophan 5-mo	7532	ENSG00000170027	20	0.384	0.327	-0.07	-0.08	0.137	-0.46	0.071	-0	0.201	0.2129	0.2502	0.1954	-0.139	-0.202	0.1155	0.0954	0.1786	0.1239	-0.01	-1.21	-0.29	-0.47	-0.43	-0.07	0.011	-0.06	0.307	0.17647	0.30288	-0.035563025	0.338443518
1660	P17987	TCP1	t-complex 1	6950	ENSG00000120438	32	0.069	0.038	-0.01	0.296	0.252	-0.4	0.262	0.222	0.199	-0.023	-0.065	-0.112	-0.157	-0.264	-0.101	0.2441	0.1244	0.1389	-0.15	-0.78	-0.12	-0.43	-0.36	-0.06	0.245	-0.06	0.02	0.167579	0.293292	0.127367326	0.165924586
3198	Q96TA1	FAM129B;	family with sequence similarity 129 member	64855	ENSG00000136830	35	-0.01	0.142	-0.06	0.008	-0.33	-0.46	0.005	-0.29	0.221	0.1441	0.1872	-0.004	0.1831	-0.088	0.2262	0.1279	0.2897	0.0266	-0.42	-1	-0.52	-0.76	-0.38	2E-04	-0.18	0.071	0.406	0.119624	0.222175	-0.207887246	0.430062034
2082	P53990	IST1	IST1, ESCRT-III associated factor;IST1 fac	9798	ENSG00000182149	13	0.024	0.173	-0.14	0.355	0.12	-0.27	0.061	-0.1	-0.07	-0.092	0.0083	0.1558	0.2808	-0.213	0.3367	0.1094	0.2074	0.1979	-0.32	-0.4	-0.36	-0.75	-0.64	-0.39	-0.24	-0.08	0.386	0.052876	0.327	-0.092438335	0.419438048
3753	Q9Y490	TLN1	talin 1	7094	ENSG00000137076	100	-0.12	0.028	-0.15	-0.12	-0.02	-0.31	0.065	-0.03	0.229	-0.057	-0.067	-0.081	-0.111	-0.137	0.0836	0.1073	0.1464	0.1399	-0.28	-0.65	-0.07	-0	-0.21	0.018	0.135	0.043	0.195	0.684377	0.044417	-0.050154588	0.094571128
2149	P61026	RAB10	RAB10, member RAS oncogene family	10890	ENSG00000084733	18	-0.08	0.289	0.032	0.08	-0.14	-0.39	0.011	-0.17	0.097	0.1262	0.1438	-0.041	0.1318	-0.354	0.1417	0.0506	0.3244	0.0505	-0.42	-1.01	-0.38	-0.73	-0.44	0.085	-0.26	-0.19	0.19	0.088242	0.319153	-0.094877552	0.414030465
1396	P02787	TF	transferrin	7018	ENSG00000091513	48	-0	-0.52	0.138	-0.08	-0.07	0.142	-0.18	0.489	-0.81	0.5066	-0.473	-0.03	-0.481	-0.759	-1.108	0.088	-0.302	-0.566	-0.79	0.062	0.435	1.134	0.509	-0.11	-0.15	0.647	-1.16	0.449205	-0.16296	0.248612335	-0.411569773
3438	Q9HC38	GLOD4	glyoxalase domain containing 4	51031	ENSG00000167699	25	0.24	-0.07	-0.21	-0.03	0.001	-0.47	0.184	0.029	0.412	-0.175	0.1283	-0.041	0.0681	-0.267	0.1083	0.3552	0.2823	0.2422	-0.08	-0.73	-0.16	-0.84	-0.48	-0.19	-0.01	0.126	0.416	0.263501	0.225381	-0.067635589	0.293016764
1173	O60701	UGDH	UDP-glucose 6-dehydrogenase	7358	ENSG00000109814	31	0.136	-0.1	-0.31	0.005	0.038	-0.59	0.06	-0.31	0.264	-0.1	-0.046	-0.082	0.0501	0.2869	0.1463	0.0739	0.0716	0.014	-0.19	-0.74	-0.11	-0.62	-0.5	-0.25	-0.04	0.07	0.303	0.234174	0.141315	-0.13511685	0.276431544
2100	P54920	NAPA	NSF attachment protein alpha	8775	ENSG00000105402	27	-0.05	0.291	-0.09	0.091	-0.17	-0.32	0.034	-0.02	0.077	0.1656	0.2608	0.0198	0.1694	-0.148	0.2459	0.1229	0.0844	-0.044	-0.25	-0.82	-0.32	-0.52	-0.24	0.122	-0.12	-0.01	0.295	0.14737	0.188863	-0.114712775	0.303576212
2154	P61106	RAB14	RAB14, member RAS oncogene family	51552	ENSG00000119396	18	-0.06	0.035	0.007	0.05	-0.05	-0.5	-0.03	9E-04	-0.07	0.1023	0.1779	0.0185	0.1879	-0.185	0.17	-0.131	0.1278	-0.097	-0.37	-0.76	-0.06	-0.66	-0.31	0.052	-0.08	-0.03	0.158	0.181833	0.161188	-0.108856481	0.270044253
1253	O94760	DDAH1	dimethylarginine dimethylaminohydrolase 1	23576	ENSG00000153904	29	0.039	0.251	0.019	-0.02	-0.02	-0.35	0.074	-0.04	0.252	0.0769	0.0313	0.0492	-0.036	-0.146	0.0035	0.0754	0.2107	-0.235	-0.14	-0.53	-0.07	-0.12	-0.4	-0.01	-0.03	0.078	0.235	0.442445	0.132083	0.018840618	0.113242462
414	B1AM27	UNC13B	unc-13 homolog B	10497	ENSG00000198722	71	0.031	0.32	-0.06	0.103	-0.07	-0.3	-0.19	-0.02	0.196	0.1165	0.012	0.0817	0.0946	-0.276	0.2264	0.4001	-0.017	0.0503	-0.06	-0.67	-0.26	-0.61	-0.47	-0.01	0.033	0.109	0.117	0.175863	0.20324	-0.075703958	0.278943663
1478	P07737	PFN1	profilin 1	5216	ENSG00000108518	19	-0.14	0.052	-0.18	0.002	-0.26	-0.43	0.218	-0.03	0.349	-0.098	-0.017	-0.334	-0.116	-0.207	-0.028	0.1574	0.2989	0.1454	-0.35	-1.19	-0.19	-0.43	-0.54	-0.13	-0.11	0.041	0.427	0.340529	0.23004	-0.024616039	0.254656352
2729	Q6UX06	OLFM4	olfactomedin 4	10562	ENSG00000102837	33	-1.73	-1.47	-0.3	-1.09	-1.13	-0.55	-1.25	-1.29	-1.16	-1.248	-1.126	-1.481	-0.213	-1.491	-1.337	-0.36	-0.845	-0.418	0.698	-0.11	1.194	2.91	0.261	1.13	0.238	-1.91	-1.92	0.100058	-1.38391	-0.160462545	-1.223447805
2226	P63000	RAC1	Rac family small GTPase 1	5879	ENSG00000136238	13	-0.04	0.404	0.081	0.162	-0.16	-0.42	0.278	-0.02	0.127	-0.066	0.0257	0.1066	0.0509	-0.253	0.1242	0.2038	0.1864	0.2371	-0.34	-0.37	-0.28	-0.33	-0.46	-0.05	-0.16	-0.04	0.147	0.111722	0.253136	-0.024228252	0.277363927
1657	P17858	PFKL	phosphofructokinase, liver type	5211	ENSG00000141959	32	0.035	0.088	-0.11	0.138	0.103	-0.4	-0.15	-0.21	-0.02	0.0372	0.0604	0.1462	0.1294	-0.169	-0.057	0.0094	0.0326	0.1118	-0.21	-0.74	-0.13	-0.31	-0.24	0.026	-0.08	-0.12	0.002	0.146483	0.142053	-0.091234952	0.233287832
3353	Q9H173	SIL1	SIL1 nucleotide exchange factor	64374	ENSG00000120725	30	-0.21	-0.67	-0.23	-0.54	-0.39	-0.18	-0.11	0.041	0.031	0.2731	0.1516	-0.078	-0.529	0.3087	-0.28	0.0336	-0.621	-0.194	0.115	0.685	0.087	0.014	0.134	0.008	0.551	0.065	-0.31	0.136295	-0.40047	-0.14679939	-0.253671463
2764	Q7L576	CYFIP1	cytoplasmic FMR1 interacting protein 1	23191	ENSG00000280618	53	0.235	0.215	0.036	-0.04	-0.09	-0.29	-0.12	-0.08	0.19	0.1225	-0.008	0.2275	0.0652	-0.216	-0.042	0.1006	0.0498	0.1137	-0.28	-0.84	-0.3	-0.29	-0.25	-0.04	0.008	-0.07	0.061	0.125334	0.226373	-0.040782477	0.267155016
2053	P52209	PGD	phosphogluconate dehydrogenase	5226	ENSG00000142657	31	0.164	0.082	-0.07	0.004	-0.02	-0.48	-0.07	-0.04	0.074	0.0461	-0.019	-0.051	-0.083	-0.168	-0.077	0.0127	0.1091	0.0275	-0.22	-1.2	-0.08	-0.05	-0.44	0.062	-0.09	-0.04	0.118	0.400779	0.174733	-0.0171771	0.191910091
1515	P09467	FBP1	fructose-bisphosphatase 1	2203	ENSG00000165140	25	-0.3	0.049	-0.01	-0.12	0.032	-0.47	0.286	0.074	0.08	0.4395	-0.236	0.1712	-0.18	0.0267	-0.15	-0.071	0.0142	-0.114	-0.2	-0.49	0.112	-0.42	-0.11	-0.03	0.084	0.019	-0.1	0.637753	0.084307	-0.0315914	0.11589815
3369	Q9H2G2	SLK	STE20 like kinase	9748	ENSG00000065613	52	0.119	0.119	-0.12	-0.14	-0.15	-0.12	-0.06	-0.22	0.236	0.0219	-0.028	0.0268	-0.177	-0.067	0.187	0.1348	0.12	0.0725	-0.15	-0.63	-0.24	-0.68	-0.33	-0.12	0.152	-0.06	0.059	0.148129	0.185213	-0.070012995	0.255226286
1679	P19801	AOC1	amine oxidase copper containing 1;amine o	26	ENSG00000002726	31	-0.06	-1.26	0.123	0.889	-0.34	0.784	-0.27	0.161	-0.23	-1.017	0.754	1.0475	-0.808	-0.817	0.367	0.4855	-0.203	0.6186	-0.47	-0.46	-0.62	-0.8	-0.57	-1.03	0.61	-0.18	0.309	0.53762	0.333519	-0.07061079	0.404129616
1667	P18669	PGAM1	phosphoglycerate mutase 1	5223	ENSG00000171314	20	0.221	0.197	-0.03	-0.03	0.101	-0.34	0.191	0.258	0.225	-0.006	-0.087	0.077	0.1098	-0.228	0.0551	0.1862	0.0866	0.0092	-0.21	-1.25	-0.38	-0.54	-0.36	0.058	0.012	0.14	0.222	0.170217	0.346174	0.066635954	0.279537889
1446	P06280	GLA	galactosidase alpha	2717	ENSG00000102393	20	0.242	-0.51	-0.21	-0.96	-0.52	-0.7	0.076	-0.31	0.216	-0.035	-0.263	-0.062	-0.327	0.1204	-0.193	0.2525	-0.13	-0.213	0.579	0.878	0.333	0.568	0.087	0.2	-0.02	-0.3	-0.03	0.088328	-0.55272	-0.203120151	-0.349601697
1957	P47756	CAPZB	capping actin protein of muscle Z-line subur	832	ENSG00000077549	26	0.201	0.312	0.19	0.015	0.057	-0.4	0.285	0.227	0.395	0.1294	0.1161	0.1253	0.1131	-0.094	0.0703	0.233	0.2301	0.2169	-0.17	-0.72	-0.05	-0.44	-0.25	0.086	0.15	0.093	0.193	0.154984	0.265293	0.016315817	0.248977428
1718	Q32Q12	NME1-NM	NME1-NME2 readthrough	654364	ENSG00000011052	19	0.145	0.09	-0.1	0.032	0.273	-0.49	0.308	0.132	0.32	0.0583	-0.138	0.0034	0.0439	-0.104	0.0576	0.0904	0.1028	0.0439	-0.14	-1.05	-0.14	-0.49	-0.25	0.089	0.082	0.234	0.17	0.328383	0.244746	0.061310148	0.183436283
1382	P02649	APOE	apolipoprotein E	348	ENSG00000130203	32	-0.39	-1.08	-0.39	-0.65	0.078	-0.11	-0.01	0.24	-0.47	0.0845	-0.24	-0.394	-0.409	0.1672	-0.61	0.7936	-0.933	-0.07	0.116	-0.33	0.343	1.33	0.147	0.136	-0.4	-0.25	-0.68	0.485515	-0.35606	-0.13097103	-0.22508944
2269	P84077	ARF1	ADP ribosylation factor 1	375	ENSG00000143761	19	0.281	0.42	0.187	0.122	-0.15	0.181	-0.09	-0.1	0.061	0.3522	0.4621	0.1148	0.1236	-0.125	0.0305	-0.022	0.2536	-0.03	-0.04	-0.49	-0.1	-0.07	-0.13	0.059	-0.2	-0.01	0.275	0.205517	0.180339	-0.027691105	0.208029769
3396	Q9H4M9	EHD1	EH domain containing 1	10938	ENSG00000110047	44	-0.17	0.163	-0.12	0.111	0.02	-0.29	0.16	0.045	0.25	0.0464	0.029	-0.025	-0.111	-0.181	0.101	0.137	0.1982	0.1938	-0.28	-0.51	-0.22	-0.18	-0.4	0.041	-0.02	0.025	0.192	0.20735	0.168229	-0.025007305	0.193236391
1381	P02647	APOA1	apolipoprotein A1	335	ENSG00000118137	35	-0.84	-0.65	-0.74	-0.91	-0.33	-0.1	-0.19	-0.12	-0.53	-0.198	-0.22	-0.592	-0.469	0.2555	-0.779	0.0442	-0.171	-0.337	-0.16	-0.28	1.307	1.733	-0.11	0.335	-0.06	-0.19	-0.78	0.146947	-0.69011	-0.216765939	-0.47333992
3230	Q99832	CCT7	chaperonin containing TCP1 subunit 7	10574	ENSG00000135624	34	0.058	-0.06	-0.07	0.245	0.229	-0.25	0.358	0.298	0.275	-0.1	-0.165	-0.141	-0.218	-0.141	-0.131	0.3308	0.0718	0.1388	-0.24	-0.72	-0.2	-0.39	-0.26	-0.03	0.349	0.043	0.146	0.219083	0.265409	0.160110491	0.105298394
1693	P20340	RAB6A	RAB6A, member RAS oncogene family	5870	ENSG00000175582	19	0.148	0.374	0.036	-0.03	-0.09	-0.25	-0.14	-0.2	0.042	0.2176	0.1425	0.0313	0.0892	-0.026	0.073	0.0206	0.2817	-0.099	-0.27	-0.82	-0.05	-0.39	-0.19	0.161	-0.36	-0.25	0.137	0.121235	0.213298	-0.09386566	0.307163415
1898	P40763	STAT3	signal transducer and activator of transcripti	6774	ENSG00000168610	37	0.291	0.065	-0.05	0.056	-0.15	-0.38	-0.13	-0.12	0.236	0.278	0.003	0.1279	0.045	-0.208	0.0106	0.007	0.1066	0.0664	-0.35	-0.73	-0.12	-0.42	-0.35	-0.01	0.222	0.028	0.206	0.297717	0.149203	-0.067823947	0.217026542
1520	P09525	ANXA4	annexin A4	307	ENSG00000196975	32	-0.09	0.256	-0.13	0.057	0.026	-0.48	0.33	-0.03	0.411	-0.152	-0.065	-0.009	0.2842	-0.05	-0.098	0.2176	0.3944	0.2078	-0.16	-0.63	-0.38	-0.42	-0.3	-0.14	0.14	-0.02	0.268	0.216341	0.221616	-0.0418502	0.263465992
149	P01857	IGHG1	immunoglobulin heavy constant gamma 1 (C	3500	ENSG00000211896	15	-1.47	-1.22	-0.46	-0.84	-0.69	-0.65	-0.56	-0.3	-0.79	-0.081	-0.746	0.1556	-0.314	-1.019	-1.196	0.0584	-0.443	-0.304	-1.13	-0.58	0.826	2.333	0.287	0.145	0.096	0.409	-1.18	0.156473	-0.90983	-0.344648255	-0.56517825
2335	Q07866	KLC1	kinesin light chain 1	3831	ENSG00000126214	50	0.125	0.429	0.009	-0.08	-0.49	-0.32	-0.04	-0.05	0.163	0.2699	0.2091	-0.007	-0.114	-0.13	0.2746	0.1419	0.2607	-0.243	-0.4	-1.1	-0.13	-0.76	-0.24	2E-04	-0.12	-0.1	0.436	0.226158	0.24079	-0.101499242	0.342289313
1723	P22748	CA4	carbonic anhydrase 4	762	ENSG00000167434	15	0.305	-0.07	0.161	-0.14	0.602	-0.33	0.176	0.046	0.438	-0.114	-0.1	-0.264	0.5079	-0.811	0.1079	-0.166	-0.179	-0.174	0.263	0.305	-0.15	0.297	-0.21	-0.77	-0.56	-0.45	-0.52	0.277993	0.330294	0.264006122	0.066288051
915	J3KPA1	CRISP3	cysteine rich secretory protein 3	10321	ENSG00000096006	10	-0.16	-1	-0.24	-0.11	-1	-0.42	1.292	0.282	1.948	-0.605	-1.106	0.1124	-1.436	-1.008	-1.66	0.2312	-1.637	-0.326	1.156	0.116	-0.56	0.443	0.877	-1.5	0.063	-0.15	-1.01	0.214531	0.128752	0.892047157	-0.763295464
1584	P13489	RNH1	ribonuclease/angiogenin inhibitor 1	6050	ENSG00000023191	32	0.175	0.285	-0.01	0.099	0.106	-0.52	-0.15	-0.01	0.046	0.1517	0.195	0.1289	0.1549	-0.132	0.1179	-0.009	0.2244	0.0089	-0.28	-1.37	-0.31	-0.64	-0.32	0.126	-0.06	-0	0.207	0.157922	0.29577	-0.09206494	0.38783474
3758	Q9Y4L1	HYOU1	hypoxia up-regulated 1	10525	ENSG00000149428	40	-0.02	-0.28	-0.26	-0.2	0.165	-0.09	0.047	0.183	0.033	-0.191	-0.132	-0.493	-0.489	0.1713	-0.21	0.0668	-0.267	0.0229	-0.14	0.445	0.166	-0.44	-0.34	-0.27	0.398	0.113	-0.15	0.566061	-0.02405	0.122066457	-0.14611607
2308	Q03167	TGFBR3	transforming growth factor beta receptor 3	7049	ENSG00000069702	30	-0.16	-0.37	-0.48	-0.68	-0.18	-0.19	0.616	-0.28	0.148	0.1975	-0.154	-0.181	-0.505	0.2228	-0.153	0.1208	-0.472	0.3182	0.298	1.089	-0.12	-0.15	0.027	-0.26	-0.5	-0.57	-0.01	0.791703	-0.15431	-0.107094992	-0.0472197
1830	P31948	STIP1	stress induced phosphoprotein 1	10963	ENSG00000168439	42	0.127	0.061	-0.04	0.049	0.172	-0.35	0.034	-0.08	0.259	-0.008	-0.031	-0.092	0.0182	-0.016	0.1455	0.1712	0.1156	0.0695	-0.09	-0.8	-0.22	-0.62	-0.36	0.003	0.106	-0	0.064	0.149344	0.237391	-0.015422202	0.252813142
1831	P31949	S100A11	S100 calcium binding protein A11	6282	ENSG00000163191	9	-0.18	0.004	-0.28	0.115	-0.17	-0.46	0.26	-0.19	0.188	-0.035	-0.066	-0.187	0.0246	-0.286	0.1252	0.3594	0.0725	-0.069	-0.28	-1.62	-0.37	-0.37	-0.3	0.007	0.089	0.088	0.396	0.498247	0.181712	-0.073732206	0.255443903
3694	Q9UQN3	CHMP2B	charged multivesicular body protein 2B	25978	ENSG00000083937	13	-0.11	0.238	-0.25	0.114	-0.1	-0.47	0.796	0.322	0.357	-0.311	-0.028	0.1008	-0.014	-0.046	0.1572	0.3869	0.0081	0.4784	-0.06	-0.26	-0.36	-0.9	-0.63	-0.24	0.115	0.001	0.642	0.344018	0.289115	0.019272402	0.269842508
2035	P51149	RAB7A	RAB7A, member RAS oncogene family	7879	ENSG00000075785	16	0.113	0.576	0.153	0.022	-0.03	-0.38	-0.02	-0.12	0.066	0.0253	0.0381	-0.027	0.0306	-0.27	0.1537	0.0462	0.2625	0.0363	-0.33	-0.74	-0.16	-0.61	-0.32	0.034	-0.19	0.012	0.218	0.16415	0.273146	0.010379665	0.262766624
1151	O60268	KIAA0513	KIAA0513	9764	ENSG00000135709	25	0.008	0.139	-0.18	-0.22	-0.08	-0.4	0.067	0.127	0.509	0.1202	0.0557	-0.095	0.3474	-0.194	-0.107	0.4986	0.2353	0.3653	-0.66	-0.43	-0.41	-0.55	-0.37	0.098	-0.04	0.088	0.114	0.129455	0.237814	-0.139870623	0.377684637
1003	O00339	MATN2	matrilin 2	4147	ENSG00000132561	48	-0.73	-0.92	0.132	-0.7	-0.25	-0.12	-0.41	-0.16	-0.18	-0.069	0.4731	-0.278	-0.842	0.8529	-0.34	-0.061	-0.215	-0.403	-0.3	0.916	0.331	-0.3	-0.41	0.045	0.687	0.541	-0.25	0.209509	-0.50964	-0.271909969	-0.237728179
29	P11940	PABPC1	poly(A) binding protein cytoplasmic 1	26986	ENSG00000070756	30	0.025	0.096	-0.05	-0.04	0.107	-0.33	0.121	0.049	0.3	0.0378	-0.068	-0.057	-0.056	-0.032	0.1379	0.2702	0.176	0.1174	-0.13	-0.6	-0.14	-0.41	-0.24	0.026	0.201	0.162	0.185	0.362791	0.134699	-0.028045094	0.162743956
1827	P31946	YWHAB	tyrosine 3-monooxygenase/tryptophan 5-mo	7529	ENSG00000166913	20	-0.03	-0	-0.23	-0.04	-0.16	-0.33	0.941	0.487	0.439	0.0608	0.0501	-0.071	-0.017	-0.045	0.3082	0.0243	-0.172	0.3199	-0.12	-0.44	-0.13	-0.54	-0.3	-0.03	0.262	0.231	1.125	0.883553	0.11346	0.068881952	0.044577729
2522	Q15435	PPP1R7	protein phosphatase 1 regulatory subunit 7	5510	ENSG00000115685	27	2E-04	0.211	-0.2	0.03	0.228	-0.48	0.141	0.377	0.162	-0.055	-0.065	-0.024	-0.028	0.0062	0.174	0.2542	0.2819	0.2313	-0.29	-0.63	-0.14	-0.43	-0.32	0.013	0.011	0.102	0.293	0.226834	0.206525	-0.034244709	0.240769486
1652	P17405	SMPD1	sphingomyelin phosphodiesterase 1	6609	ENSG00000166311	23	-0.15	-0.74	-0.36	-0.19	-0.22	-0.08	-0.15	-0.06	-0.14	0.4217	0.177	0.1299	-0.083	0.4022	-0.062	0.8802	-0.114	-0.064	-0.28	0.5	-0.14	-0.09	-0.11	-0.14	0.49	0.143	-0.26	0.118843	-0.24602	-0.420060202	0.174041265
2159	P61224	RAP1B	RAP1B, member of RAS oncogene family	5908	ENSG00000127314	20	-0.06	0.317	0.072	0.107	-0.12	-0.4	0.056	-0.11	0.046	0.1732	0.0184	0.0826	0.0394	-0.352	0.1875	0.1759	0.3398	0.0936	-0.33	-0.69	-0.44	-0.51	-0.41	0.065	-0.32	-0.07	0.359	0.12064	0.252394	-0.092758671	0.345152943
1789	P29373	CRABP2	cellular retinoic acid binding protein 2	1382	ENSG00000143320	9	-0.49	-0.73	-0.13	-0.34	-0.36	-0.87	1.088	-0.86	-0.33	0.3964	0.6861	0.1291	0.1235	-0.331	0.0872	-0.252	-0.463	-0.094	-0.44	-0.66	0.568	-0.2	0.121	0.652	-0.22	0.32	-0.03	0.357091	-0.34968	-0.36859069	0.018910145
3673	Q9UNF0	PACSIN2	protein kinase C and casein kinase substrat	11252	ENSG00000100266	35	-0.17	0.1	-0.21	0.013	0.19	-0.46	-0.08	-0.21	0.243	-0.039	-0.184	0.0157	0.0147	-0.262	0.0658	0.2006	0.1082	0.2131	-0.36	-0.2	-0.34	-0.56	-0.4	-0.1	-0.17	0.017	0.184	0.210772	0.150283	-0.079800352	0.230083632
1539	P10253	GAA	glucosidase alpha, acid	2548	ENSG00000171298	30	0.038	-0.54	0.18	0.244	-0.15	-0.13	0.113	0.479	0.21	0.1075	-0.282	0.2996	-0.208	0.1285	-0.089	0.1224	-0.251	-0.294	0.035	-0.02	0.03	-0.08	0.335	-0.26	0.302	0.226	-0.48	0.799943	0.039744	0.101295665	-0.061551765
3260	Q9BSW2	CRACR2A	calcium release activated channel regulator	84766	ENSG00000130038	56	0.016	0.053	-0.1	0.273	-0.16	-0.47	0.158	0.108	0.099	0.2618	0.1517	-0.033	0.1189	-0.341	0.1089	0.0173	0.4111	-0.143	-0.26	-0.75	0.002	-0.49	-0.76	0.131	-0.01	-0.17	0.425	0.295217	0.206528	-0.063677127	0.270204648
3182	Q96QK1	VPS35	VPS35 retromer complex component;VPS3	55737	ENSG00000069329	37	0.035	-0.01	-0.05	-0.02	-0.04	-0.28	-0.02	-0.16	0.088	0.1655	0.1109	-0.016	0.0054	0.0402	0.0298	-0.092	0.1279	0.0798	-0.19	-0.58	-0.03	-0.35	-0.3	0.079	-0.1	-0.05	0.093	0.131334	0.108332	-0.100965682	0.209297492
792	G5E9W0	PLA1A	phospholipase A1 member A	51365	ENSG00000144837	13	-0.08	-0.82	-0.43	-0.38	-0.28	-0.04	0.04	0.28	0.185	0.214	0.4721	0.7142	-0.323	0.7064	-0.119	-0.137	-0.743	0.4654	-0.02	0.033	-0.4	-0.07	-0.06	-0.34	0.654	-0.04	-0.29	0.422999	-0.10771	-0.306762416	0.199047622
1804	P30101	PDIA3	protein disulfide isomerase family A membe	2923	ENSG00000167004	33	0.112	-0.33	-0.19	0.249	0.525	-0.08	0.357	0.508	0.075	-0.112	-0.356	-0.303	-0.512	-0.011	-0.305	-0.209	-0.139	-0.201	-0.15	0.887	0.367	-0.24	0.002	-0.28	0.255	-0.16	-0.24	0.147321	0.085927	0.374413011	-0.288486429

2940	Q8NEU8	APPL2	adaptor protein, phosphotyrosine interacting	55198	ENSG00000136044	47	0.051	0.25	0.007	0.053	-0.08	-0.47	-0.06	0.06	0.151	0.1136	-0.348	0.0541	0.197	-0.169	0.3384	-0.18	0.1474	0.0238	-0.21	-1.03	-0.27	-0.5	-0.15	0.145	0.002	0.032	0.084	0.313565	0.207999	-0.023998174	0.231997045
1772	P27797	CALR	calreticulin	811	ENSG00000179218	21	-0	-0.32	-0.07	0.268	0.446	0.141	0.14	0.53	-0.08	-0.198	0.0439	-0.1	-0.404	0.1923	-0.218	-0.338	-0.515	-0.244	-0.34	0.372	0.213	-0.15	0.021	-0.24	0.478	-0.08	-0.57	0.212132	0.148424	0.315131415	-0.16670737
2317	Q05655	PRKCD	protein kinase C delta	5580	ENSG00000163932	43	0.036	0.176	-0.07	0.01	-0.13	-0.42	0.333	-0.01	0.288	0.1869	0.0705	0.0407	0.0716	-0.253	0.0227	0.1424	0.1143	0.1543	-0.21	-0.79	-0.16	-0.45	-0.26	0.141	-0.11	-0.03	0.312	0.246067	0.196407	-0.037864187	0.234270885
3249	Q9BRK5	SDF4	stromal cell derived factor 4	51150	ENSG00000078808	23	-0.26	-0.42	-0.18	-0.34	-0.28	-0.23	-0.27	0.054	-0.3	0.1102	0.1867	-0.29	-0.195	0.7755	-0.359	-0.069	-0.405	-0.364	0.112	0.943	0.199	0.027	-0.09	-0.18	0.422	-0.16	-0.32	0.22142	-0.35463	-0.18073539	-0.173895825
1936	P45974	USP5	ubiquitin specific peptidase 5	8078	ENSG00000111667	36	0.217	0.159	-0.04	-0.23	-0.09	-0.48	-0.05	-0.08	0.138	0.1248	0.1415	0.1359	0.0429	-0.173	0.0097	0.1201	0.109	0.1383	-0.12	-0.83	-0.21	0.137	-0.09	0.203	-0.01	-0.01	0.057	0.431081	0.045365	-0.122759707	0.168124403
1608	P14555	PLA2G2A	phospholipase A2 group IIA	5320	ENSG00000188257	13	-1.15	-0.61	-0.23	-1.57	-1.34	-0.86	-1.08	-0.3	0.451	0.2469	-0.549	0.0653	-0.303	-1.085	-0.23	-0.738	0.5322	-0.647	0.529	2.798	-0.3	-0.19	-1.8	0.607	0.259	-0.46	-0.61	0.293008	-0.83396	-0.439895298	-0.394067505
1818	P31150	GDI1	GDP dissociation inhibitor 1	2664	ENSG00000203879	37	-0.04	0.129	-0.09	0.16	-0.13	-0.19	0.086	5E-04	0.169	0.0954	-0.181	-0.073	0.0873	-0.231	0.0137	0.2249	0.1917	0.0027	-0.21	-1.16	-0.5	-0.5	-0.43	-0.03	-0.14	0.027	0.267	0.146224	0.30833	-0.003193624	0.311523291
2368	Q12913	PTPRJ	protein tyrosine phosphatase receptor type J	5795	ENSG00000149177	38	-0.17	0.175	-0.22	-0.31	4E-04	-0.2	-0.44	-0.21	-0.13	-0.305	0.0922	0.0471	-0.025	0.0644	-0.135	-0.052	-0.02	0.2102	-0.39	0.114	-0.21	-0.29	-0.27	-0.24	-0.17	-0.13	-0.11	0.178966	0.022008	-0.151542971	0.173550848
1128	O43633	CHMP2A	charged multivesicular body protein 2A	27243	ENSG00000130724	15	0.138	0.312	-0.16	0.125	-0.05	-0.46	0.194	-0.04	-0.06	0.0681	0.1244	0.2127	0.0367	-0.103	-0.068	0.1756	0.0034	0.1697	-0.35	-0.59	-0.38	-0.71	-0.45	-0.23	-0.13	-0.09	0.298	0.082123	0.291821	-0.068728568	0.360549648
1995	P49419	ALDH7A1	aldehyde dehydrogenase 7 family member A	501	ENSG00000164904	34	0.165	0.167	-0.05	-0.12	-0.09	-0.56	0.274	0.073	0.401	-0.045	-0.128	0.1087	0.1043	-0.129	-0.046	0.2436	0.003	0.0581	-0.19	-0.77	-0.25	-0.61	-0.33	0.014	0.192	0.257	0.339	0.498407	0.177538	0.009802951	0.167734678
2966	Q8TB96	ITFG1	integrin alpha FG-GAP repeat containing 1	81533	ENSG00000129636	14	-0.34	0.26	0.035	-0.22	-0.21	-0.4	-0.03	-0.21	0.088	0.4889	0.2951	-0.224	0.1077	-0.236	-0.177	0.1605	0.1544	0.1816	-0.34	-0.55	-0.09	-0.43	-0.35	0.229	0.052	-0.12	-0.19	0.177169	0.085757	-0.197122213	0.282879143
1340	P01009	SERPINA1	serpin family A member 1	5265	ENSG00000277377	30	-0.26	-1.07	-0.47	-0.84	-0.64	0.085	-0.58	0.071	-0.2	0.6261	0.4135	-0.005	-0.51	0.0936	-0.126	-0.049	-0.605	-0.524	0.088	0.069	0.803	0.532	0.418	0.116	-0.28	0.325	-0.34	0.09772	-0.62695	-0.358381342	-0.268573387
2030	P50990	CCT8	chaperonin containing TCP1 subunit 8	10694	ENSG00000156261	36	0.147	0.102	0.019	0.439	0.373	-0.31	0.234	0.209	0.231	0.0373	-0.002	-0.075	-0.132	-0.291	-0.03	0.2055	0.1106	0.1205	-0.23	-1.01	-0.19	-0.09	-0.37	-0.05	0.212	0.032	0.043	0.144944	0.344008	0.166236854	0.177770937
752	G3V500	EML1	EMAP like 1	2009	ENSG00000066629	2	-1.81	-2.93	-1.96	-0.33	-1.22	-1.2	1.353	0.908	0.953	-1.234	-2.737	-0.974	-0.962	0.5874	-0.221	1.1014	-0.538	0.2835	-0.69	0.59	-1.09	-0.28	-0.65	-0.95	0.888	0.542	0.283	0.722799	-0.54055	-0.170187482	-0.370366876
601	J3KMY5	NPC2	NPC intracellular cholesterol transporter 2	10577	ENSG00000119655	11	0.649	-0.61	0.359	0.621	0.268	0.356	-0.32	0.599	-0.24	0.6183	-0.32	1.1958	-0.328	-0.329	-0.451	-0.129	-0.651	-1.069	-0.6	-0.72	-0.78	-0.39	0.011	-0.9	-0.84	0.334	-1.82	0.131614	0.821687	0.350045365	0.471642127
1895	P40227	CCT6A	chaperonin containing TCP1 subunit 6A	908	ENSG00000146731	34	0.11	0.158	0.019	0.231	0.316	-0.34	0.121	0.11	0.271	-0.075	0.0011	-0.047	-3E-04	-0.14	0.0772	0.3074	0.1804	0.1617	-0.25	-0.69	-0.19	-0.54	-0.21	0.009	0.169	0.003	0.082	0.125884	0.291494	0.059139306	0.232354878
1753	P25789	PSMA4	proteasome subunit alpha 4	5685	ENSG00000041357	19	-0.18	-0.42	-0.05	-0.1	0.289	-0.6	0.28	-0.04	0.182	0.1395	-0.264	-0.078	-0.023	-0.208	-0.386	0.6931	0.2139	0.2822	-0.39	-0.06	-0.24	0.19	-0.02	-0.13	0.661	0.353	-0.23	0.828702	-0.08436	-0.110772234	0.026414247
2542	Q15836	VAMP3	vesicle associated membrane protein 3	9341	ENSG00000049245	11	-0.08	0.298	0.204	0.037	0.036	-0.19	0.509	0.366	0.615	0.2042	0.1112	-0.046	0.2105	-0.267	0.3298	0.3597	0.654	0.4279	-0.15	-0.89	-0.49	-0.45	-0.47	0.309	-0.06	0.355	0.587	0.208574	0.339552	-0.021622867	0.361174787
1791	P29401	TKT	transketolase	7086	ENSG00000163931	29	-0.01	0.295	-0.05	-0.01	-0.07	-0.34	0.456	0.026	0.152	0.1604	-0.036	-0.052	-0.141	-0.266	0.086	0.2135	0.2081	-0.073	-0.22	-0.96	0.134	0.389	-0.28	-0.07	0.133	0.048	0.046	0.682479	0.136202	0.037449091	0.098752957
941	K4DIA7	CD151	CD151 molecule (Raph blood group)	977	ENSG00000177697	11	-0.01	0.347	0.384	0.133	-0.81	-0.56	0.094	-0.14	-0.13	0.268	0.18	0.0312	0.0827	-0.347	0.1821	0.0378	0.1728	-0.085	-0.5	-0.63	-0.03	-0.72	-0.55	0.153	-0.05	-0.21	0.362	0.338344	0.164878	-0.135332116	0.300210055
593	E5RJR5	SKP1	S-phase kinase associated protein 1	6500	ENSG00000113558	14	-0.02	-0.03	-0.2	-0.04	0.1	-0.6	-0.02	0.006	0.126	-0.091	-0.039	-0.073	-0.027	-0.093	0.1507	-0.117	0.2437	-0.109	-0.23	-0.85	-0.27	-0.58	-0.47	-0.02	0.101	-0.17	0.074	0.214125	0.19194	-0.058512525	0.250452743
2674	Q687X5	STEAP4	STEAP4 metalloreductase	79689	ENSG00000127954	19	-0.23	0.793	0.142	-0.15	-0.29	-0.61	0.092	-0.16	-0.11	-0.053	0.2166	0.3572	0.1767	-0.41	-0.092	0.2253	0.033	0.0857	-0.15	0.099	-0.09	-0.03	-0.55	-0.38	-0.15	0.353	0.613	0.811712	-0.02615	-0.118365127	0.092212795
1205	O75363	BCAS1	breast carcinoma amplified sequence 1	8537	ENSG00000064787	35	-0.04	0.329	-0.11	-0.23	-0.2	-0.33	-0.09	0.124	0.384	0.0645	0.0836	-0.349	-0.182	-0.092	0.1396	0.1455	0.5771	-0.111	-0.2	-0.47	-0.19	-0.54	-0.67	-0.05	0.13	-0.18	0.04	0.226886	0.217554	-0.048915173	0.266469285
3450	Q9NP72	RAB18	RAB18, member RAS oncogene family	22931	ENSG00000099246	17	-0.03	0.232	0.052	0.037	-0.08	-0.22	0.149	0.014	0.293	0.2234	0.0457	-0.054	0.0899	0.0079	0.2721	0.1712	0.3054	0.1174	-0.16	-0.92	-0.32	-0.45	-0.32	0.087	-0.12	0.017	0.306	0.112894	0.258303	-0.081352967	0.339655755
1199	O75340	PDCD6	programmed cell death 6	10016	ENSG00000249915	10	-0.04	0.444	-0.29	0.083	-0.06	-0.26	-0.06	0.208	-0.48	-0.309	-0.013	0.4732	0.0048	-0.272	0.0481	0.237	0.0277	0.5995	-0.02	-0.08	-0.65	-0.46	-0.43	-0.34	-0.38	-0.32	0.459	0.218661	0.19661	-0.138279731	0.334889367
3733	Q9Y376	CAB39	calcium binding protein 39	51719	ENSG00000135932	22	0.081	0.171	-0.02	0.134	0.107	-0.38	-0.05	-0.14	0.071	-0.007	0.1123	0.1156	0.072	-0.212	-0.016	0.0861	0.1732	0.1304	-0.2	-0.4	-0.11	-0.24	-0.37	-0.1	-0.03	0.021	0.388	0.313606	0.113269	-0.053381757	0.16665108
3061	Q92973	TNPO1	transportin 1	3842	ENSG00000083312	18	0.173	0.264	0.09	-0.07	-0.18	-0.08	0.074	-0.09	0.085	0.1451	0.0538	-0.005	-0.056	-0.051	-0.082	0.1007	0.2631	-0.183	-0.28	-0.86	-0.18	-0.26	-0.2	-0.01	0.168	0.08	0.129	0.286135	0.18544	0.008144206	0.177295322
1465	P07237	P4HB	prolyl 4-hydroxylase subunit beta	5034	ENSG00000185624	34	-0.1	-0.6	-0.19	-0.29	0.046	0.004	-0.2	0.09	-0.11	0.0304	0.1929	-0.133	-0.36	0.3154	-0.167	-0.115	-0.166	0.0116	-0.2	0.338	0.356	-0.07	0.045	-0.15	0.533	-0	-0.26	0.321198	-0.2154	-0.1058532	-0.109542835
2022	P50502	ST13	ST13, Hsp70 interacting protein;ST13 Hsp7	6767	ENSG00000100380	21	0.184	0.047	-0.04	0.047	0.234	-0.33	0.014	-0.07	0.147	-0.15	0.1833	0.0327	0.0099	-0.126	0.156	0.1646	0.1158	0.4666	-0.22	-1.08	-0.32	-0.71	-0.3	-0.09	0.073	0.021	0.163	0.125753	0.298286	-0.07008479	0.368370911
1799	P30044	PRDX5	peroxiredoxin 5	25824	ENSG00000126432	16	0.134	0.152	-0.03	0.039	-0.14	-0.66	0.193	-0.07	0.39	0.2592	0.2527	0.0347	0.2036	-0.113	0.1522	0.2095	0.2593	0.1827	-0.25	-1.05	-0.2	-0.52	-0.56	-0.05	-0.12	0.102	0.288	0.123614	0.26401	-0.158853079	0.422862716
2213	P62873	GNB1	G protein subunit beta 1	2782	ENSG00000078369	23	-0.05	0.327	-0.29	0.12	-0.05	-0.44	-0.27	-0.29	-0.07	-0.231	-0.059	0.1511	-0.046	-0.399	0.3589	0.1558	0.1326	0.0835	-0.41	-0.19	-0.6	-0.57	-0.64	-0.32	-0.39	-0.04	0.227	0.137686	0.214126	-0.128558408	0.342684417
2173	P61769	B2M	beta-2-microglobulin	567	ENSG00000166710	11	-0.01	-0.76	-0.28	-0.65	-0.06	-0.14	-1.63	-1.28	-0.87	-0.371	-0.05	-0.187	-0.084	0.3124	-0.54	-0.056	-0.218	-0.367	-0.46	0.104	0.892	0.727	-0.1	0.25	-0.12	-0.28	-0.43	0.101279	-0.69666	-0.457684274	-0.23897535
2258	P78417	GSTO1	glutathione S-transferase omega 1	9446	ENSG00000148834	13	-0.49	0.375	-0.05	0.062	0.185	-0.33	-0.05	-0.17	0.126	0.0629	0.1954	0.2632	0.108	-0.075	0.1357	-0.027	0.2745	-0.021	-0.25	-1.01	-0.11	-0.72	-0.86	-0.04	-0.03	0.053	0.148	0.132188	0.274769	-0.140311047	0.415080146
2926	Q8NBZ7	UXS1	UDP-glucuronate decarboxylase 1	80146	ENSG00000115652	19	-0.22	-0.36	-0.46	-0.75	0.121	-0.3	0.19	0.093	-0.13	0.2677	-0.224	-0.36	-0.609	0.4729	-0.254	-0.302	-0.39	0.0876	0.606	0.383	0.221	-0.38	0.039	-0.08	0.262	0.346	-0.3	0.241159	-0.32344	-0.056192668	-0.267245184
2077	P53621	COPA	coatomer protein complex subunit alpha	1314	ENSG00000122218	50	0.047	0.067	-0.1	-0.08	-0.02	-0.34	-0.15	-0.03	0.077	0.0509	-0.079	0.0833	0.1376	-0.171	-0.089	0.0077	0.1156	0.1968	-0.25	-0.49	-0.07	-0.17	-0.32	0.09	0.206	-0.11	0.161	0.408989	0.047674	-0.086468187	0.134141691
2129	P60033	CD81	CD81 molecule	975	ENSG00000110651	6	-0.33	0.412	0.59	-0.4	-0.26	-0.01	-0.37	-0.23	-0.32	0.3987	0.0397	-0.056	0.043	-0.294	-0.166	-0.203	0.1811	-0.187	-0.12	0.595	-0.2	-0.26	-0.31	0.143	-0.31	-0.04	-0.12	0.911775	-0.03194	-0.074582593	0.042638809
2324	Q06210	GFPT1	glutamine--fructose-6-phosphate transamina	2673	ENSG00000198380	43	0.283	0.008	-0.11	-0.3	0.009	-0.4	-0.35	0.132	-0.09	0.0036	-0.099	0.0588	-0.107	-0.326	0.2241	-5E-04	0.1898	0.1212	-0.21	-0.8	-0.09	-0.65	-0.16	-0.01	0.087	-0.08	0.138	0.390078	0.108364	-0.096626497	0.204990141
2541	Q15833	STXBP2	syntaxin binding protein 2	6813	ENSG00000076944	42	-0.01	0.468	-0.23	0.159	-0.02	-0.39	-0.07	0.062	0.036	-0.206	0.0062	0.0661	0.157	-0.169	0.3347	0.4331	0.0383	0.3081	-0.46	-0.42	-0.42	-0.49	-0.45	-0.09	-0.1	0.102	0.315	0.146745	0.222935	-0.107877343	0.330812325
1709	P21589	NT5E	5'-nucleotidase ecto	4907	ENSG00000135318	25	-0.04	-0.33	-0.52	-0.11	-0.19	0.097	-0.34	0.319	-0.24	-0.342	0.4418	-0.192	-0.077	0.0536	-0.023	0.3095	0.3479	1.1159	-0.15	-0.47	-0.49	-1.22	-0.15	-0.37	-0.1	-0.4	0.069	0.111355	0.213014	-0.333416042	0.546430484
3114	Q96DG6	CMBL	carboxymethylenebutenolidase homolog	134147	ENSG00000164237	24	0.136	0.081	-0.48	-0.02	-0.03	-0.36	-0.06	-0.06	-0.02	-0.285	0.1614	0.5281	0.1885	-0.17	0.1735	-0.052	0.5128	-0.17	-0.62	-1.58	-0.82	-0.68	-0.26	0.339	0.056	0.014	0.275	0.211591	0.273188	-0.190543799	0.463731961
3619	Q9UHR4	BAIAP2L1	BAR/IMD domain containing adaptor protein	55971	ENSG00000006453	32	0.128	0.553	-0.39	-0.19	0.108	-0.43	-0.04	-0.03	-0.04	-0.259	-0.099	0.5308	-0.009	-0.261	0.036	0.1488	0.1009	0.2493	-0.28	-0.12	-0.34	-0.53	-0.54	-0.38	-0.05	0.123	0.334	0.341547	0.162702	-0.085479592	0.248181107
3712	Q9Y2A7	NCKAP1	NCK associated protein 1	10787	ENSG00000061676	47	0.255	0.152	0.046	-0.05	0.012	-0.36	-0.02	-0.09	0.19	0.0888	0.0411	0.1385	0.1598	-0.252	-0.076	0.085	0.1056	0.156	-0.28	-0.68	-0.19	-0.37	-0.27	0.044	-0.05	-0.08	0.055	0.121658	0.217506	-0.033945422	0.251450982
1888	P38606	ATP6V1A	ATPase H+ transporting V1 subunit A	523	ENSG00000114573	32	0.038	0.225	-0.07	-0.04	-0	-0.43	-0.05	-0.11	0.208	0.07	0.0051	0.1006	0.0378	-0.194	0.0699	0.1194	0.1024	0.0163	0.042	-0.7	-0.06	-0.57	-0.35	0.026	0.044	-0.06	0.113	0.277794	0.143672	-0.061773155	0.205444847
1876	P36871	PGM1	phosphoglucomutase 1	5236	ENSG00000079739	32	0.145	-0.1	-0.02	-0.05	-0.11	-0.56	-0.29	-0.11	-0.05	0.0537	0.0594	0.2665	0.1346	-0.471	0.0575	-0.061	0.0096	-0.176	-0.13	-0.93	-0.21	-0.42	-0.44	-0.09	-0.03	0.131	0.172	0.42812	0.086733	-0.115027395	0.20175995
1004	O00391	QSOX1	quiescin sulfhydryl oxidase 1	5768	ENSG00000116260	29	-0.2	-0.81	-0.27	-0.18	-0.26	-0.02	-0.01	-0.05	0.39	0.2082	0.0848	0.2144	-0.39	-0.098	-0.362	0.335	-0.313	-0.135	-0.3	0.108	0.142	0.502	0.369	-0.14	0.58	0.325	-0.45	0.338039	-0.28388	-0.107313799	-0.176566367
3768	Q9Y5K6	CD2AP	CD2 associated protein	23607	ENSG00000198087	42	0.059	0.203	-0.11	-0.08	-0.17	-0.41	-0.03	0.035	0.202	-0.292	-0.097	0.1541	0.0367	0.0078	-0.003	0.2672	0.2149	0.3559	-0.26	-0.72	-0.25	-0.89	-0.28	-0.23	-0.08	0.167	0.435	0.220617	0.201453	-0.105763259	0.307216095
1603	P14324	FDPS	farnesyl diphosphate synthase	2224	ENSG00000160752	14	-0	0.042	0.056	-0.03	-0.08	-0.5	0.068	-0.25	0.061	0.1411	-0.067	-0.139	-0.005	-0.118	0.0582	-0.233	0.331	-0.016	-0.08	-0.23	0.037	-0.47	-0.31	0.056	-0.15	-0.05	0.116	0.570866	0.048963	-0.065076423	0.114039647
1513	P09417	QDPR	quinoid dihydropteridine reductase	5860	ENSG00000151552	18	0.067	0.215	0.01	0.227	0.578	-0.45	0.21	0.087	0.048	-0.105	0.0697	0.0097	-0.042	-0.049	0.0112	0.1664	0.1959	0.0628	-0.44	-1.06	-0.18	-0.55	-0.58	-0.12	-0.12	0.29	0.179	0.125517	0.397046	0.075166975	0.321879465
1752	P25788	PSMA3	proteasome subunit alpha 3	5684	ENSG00000100567	21	-0.19	-0.28	-0.06	-0.15	0.306	-0.48	-0.08	-0.23	-0.04	0.0691	-0.236	-0.092	-0.099	-0.168	-0.436	0.3497	0.1752	-0.046	-0.45	-0.04	-0.32	0.026	-0.04	-0.17	0.415	0.129	-0.39	0.847298	-0.04292	-0.081812529	0.038891179
2007	P49773	HINT1	histidine triad nucleotide binding protein 1	3094	ENSG00000169567	13	0.112	0.264	0.001	-0.16	-0.15	-0.5	0.141	0.125	0.239	0.1209	0.0169	-0.135	-0.117	0.0496	0.2389	0.0261	0.0824	-0.117	-0.15	-1.02	-0.23	-0.68	-0.48	0.033	-0.08	0.129	0.143	0.207598	0.268192	-0.010462448	0.278654812
2759	Q7KZF4	SND1	staphylococcal nuclease and tudor domain c	27044	ENSG00000197157	48	0.014	0.178	-0.04	-0.07	-0.1	-0.41	0.137	0.127	0.346	0.0934	0.0313	0.0213	0.1116	-0.247	-7E-04	0.1612	0.1726	0.2133	-0.3	-0.89	-0.08	-0.28	-0.25	-0.02	0.074	0.073	0.227	0.28301	0.180992	-0.041358176	0.222349841
1166	O60568	PLOD3	procollagen-lysine,2-oxoglutarate 5-dioxyger	8985	ENSG00000106397	33	-0.28	-0.56	-0.07	-0.36	-0.11	-0	0.143	-0.15	-0.13	-0.208	0.4277	-0.056	-0.571	0.8917	-0.334	0.333	-0.326	0.0019	-0.05	0.797	-0.04	-0.23	-0.07	-0.21	0.526	-0.03	-0.33	0.562951	-0.20892	-0.18676542	-0.022150964
3572	Q9P2K2	TXNDC16	thioredoxin domain containing 16	57544	ENSG00000087301	30	-0.62	-0.12	-0.26	-0.39	-0.05	-0.41	-0.37	0.062	-0.04	0.2914	0.2075	0.017	0.0127	0.1953	-0.153	0.5209	0.223	0.3389	-0.21	0.44	-0	-0.59	-0.5	-0.25	0.263	0.179	0.085	0.092787	-0.17759	-0.426837322	0.249244601
3067	Q93050	ATP6V0A1	ATPase H+ transporting V0 subunit a1	535	ENSG00000033627	29	-0.16	-0.04	0.158	-0.1	-0.25	-0.47	0.021	0.187	0.132	0.1739	0.2191	0.0205	0.063	-0.122	0.1964	0.0538	0.3029	0.2258	-0.14	-0.54	-0.18	-0.5	-0.27	0.2	0.018	0.038	0.286	0.199723	0.063752	-0.184696495	0.24844835
1805	P30153	PPP2R1A	protein phosphatase 2 scaffold subunit Aalp	5518	ENSG00000105568	33	0.151	0.184	-0.07	-0.03	-0.02	-0.43	-0.23	-0.2	0.098	0.0075	-0.078	-0.013	0.0944	-0.05	0.0422	-0.067	0.0486	-0.113	-0.23	-1.09	-0.27	-0.55	-0.16	0.127	-0.09	-0.1	0.019	0.230042	0.200273	-0.046104269	0.246376822
3752	Q9Y485	DMXL1	Dmx like 1	1657	ENSG00000172869	83	0.002	0.149	-0.23	0.016	-0.06	-0.18	-0.12	-0.01	0.062	0.1359	-0.02	0.015	0.2155	-0.163	0.0542	0.2812	0.1118	0.142	-0.14	-0.35	-0.11	-0.38	-0.28	0.366	-0.02	4E-04	0.229	0.30358	0.034857	-0.127207896	0.162064474
2482	Q14914	PTGR1	prostaglandin reductase 1	22949	ENSG00000106853	18	-0.27	-0.72	-0.12	0.31	-0.26	-0.08	0.044	-0.7	0.24	0.0254	-0.42	0.3717	-0.73	-0.422	0.1764	0.2813	0.2479	-0.133	0.307	-1.32	-0.82	-0.7	-0.14	0.361	-0.24	0.397	0.807	0.927926	-0.02306	-0.106795851	0.083735555
2559	Q16651	PRSS8	serine protease 8	5652	ENSG00000052344	8	-0.07	0.441	-0.07	0.108	-0.15	-0.51	-0.25	-0.16	0.177	0.2834	0.496	0.4344	-0.084	-0.161	-0.15	-0.141	0.1916	0.6296	-0.47	-0.51	-0.1	-1.01	-0.58	-0.07	-0.65	0.141	0.509	0.14898	0.250036	-0.220808571	0.470844097
1802	P30085	CMPK1	cytidine/uridine monophosphate kinase 1	51727	ENSG00000162368	15	-0.14	-0.06	-0.38	-0.17	-0.2	-0.49	0.393	0.078	0.462	0.2196	-0.325	0.0346	-0.27	-0.092	0.2294	0.155	0.3626	0.2959	-0.11	-1.42	-0.64	-0.48	-0.12	0.21	0.137	-0.04	0.488	0.485176	0.16296	-0.124546842	0.287507271
3469	Q9NQW7	XPNPEP1	X-prolyl aminopeptidase 1	7511	ENSG00000108039	40	0.017	-0.14	-0.32	-0.02	0.038	-0.33	-0.2	0.027	0.098	-0.223	-0.097	-0.161	0.0574	-0.184	0.0642	0.124	0.1256	0.1807	-0.34	-0.92	-0.25	-0.46	-0.12	0.069	0.044	-0.05	0.211	0.408949	0.109806	-0.079127902	0.18893368
2153	P61088	UBE2N	ubiquitin conjugating enzyme E2 N	7334	ENSG00000177889	13	0.233	0.185	-0.15	-0.01	0.147	-0.51	-0.44	-0.32	-0.18	-0.018	0.0278	0.1343	0.1154	-0.136	0.054	-0.169	-0.073	-0.263	-0.2	-1.18	-0.25	-0.54	-0.34	0.069	0.096	-0.33	-0.01	0.311549	0.182452	-0.079434408	0.26188613
2708	Q6PCB0	VWA1	von Willebrand factor A domain containing 1	64856	ENSG00000179403	25	-0.38	-0.96	-0.3	-0.47	-0.13	0.311	-0.32	-0.16	-0.05	-0.024	0.4629	0.1204	-0.565	0.4772	-0.055	-0.025	-0.217	-0.173	-0.15	0.553	0.537	-0.23	-0.05	-0.2	0.654	-0.05	-0.4	0.258534	-0.34781	-0.273403964	-0.074409297
2872	Q8IZ41	RASEF	RAS and EF-hand domain containing	158158	ENSG00000165105	38	-0.33	-0.01	-0.29	-0.41	0.241	-0.51	0.179	-0.13	0.099	-0.243	0.1118	0.1661	-0.224	-0.286	0.2311	0.0648	0.0389	-0.023	0.018	-0.1	-0.16	-0.66	-0.49	0.047	-0.08	0.061	-0	0.603706	0.023028	-0.110459198	0.133487403
247	A0A0J9YYI4	SERINC5	serine incorporator 5	256987	ENSG00000164300	10	-0.71	0.314	0.106	-0.25	0.119	-0.39	-0.14	-0.11	-0.24	-0.035	0.3767	0.2514	0.0693	-0.111	-0.081	-0.137	0.1992	0.2579	-0.14	0.309	-0.49	-0.41	-0.27	-0.05	-0.29	-0.2	0.265	0.26365	-0.00283	-0.232576912	0.229748911
194	A0A0C4DGI	ZG16B	zymogen granule protein 16B	124220	ENSG00000162078	10	-1.16	-0.27	0.639	0.605	-0.33	-0.95	0.176	-0.09	0.874	0.2516	0.7213	0.0808	0.331	-0.085	-1.445	-0.341	0.6093	-0.793	-0.74	-0.66	-1.68	0.083	-0.62	0.013	-0.15	0.095	0.109	0.62547	0.337266	0.018203095	0.319063209
2155	P61158	ACTR3	actin related protein 3;ARP3 actin related pr	10096	ENSG00000115091	29	0.059	0.144	-0.03	-0.07	-0.01	-0.31	0.156	0.083	0.114	0.1732	-0.032	-0.005	-0.052	-0.207	0.0553	0.274	0.2446	0.288	-0.24	-0.72	0.182	0.648	-0.32	0.139	0.083	0.079	0.181	0.857649	0.012263	-0.066359025	0.07862182
614	E7EV01	CAPN5	calpain 5	726	ENSG00000149260	35	-0.03	0.34	-0.12	0.005	-0.06	-0.34	-0.22	0.034	-0.11	0.0473	0.3093	0.2528	0.1432	-0.021	-0.13	0.2153	-0.004	0.1711	-0.44	0.013	-0.5	-0.7	-0.03	0.073	-0.32	-0.07	0.108	0.122659	0.150954	-0.164931764	0.315885264
1890	P39023	RPL3	ribosomal protein L3	6122	ENSG00000100316	21	0.04	0.061	-0.13	0.072	0.468	-0.39	0.194	-0.17	0.091	-0.183	0.0081	-0.177	-0.194	-0.145	-0.08	-0.156	0.2596	0.2559	-0.26	-0.6	-0.13	-0.26	-0.1	0.06	0.149	-0.26	0.029	0.395711	0.178224	0.071874945	0.106349338
2495	Q15056	EIF4H	eukaryotic translation initiation factor 4H	7458	ENSG00000106682	18	-0.01	0.086	-0.09	-0.12	0.036	-0.27	-0.11	-0.3	0.115	-0.103	0.0378	-0.16	-0.022	-0.09	0.3572	-0.135	0.3143	-0.01	-0.29	-1.02	-0.4	-0.49	-0.11	0.04	-0.3	-0.04	0.142	0.173804	0.201014	-0.09432622	0.295340418
3476	Q9NR50	EIF2B3	eukaryotic translation initiation factor 2B sub	8891	ENSG00000070785	11	0.282	-1.74	-0.29	0.69	-0.95	0.833	0.018	-0.15	-0.34	-0.382	0.1413	0.3063	-0.831	1.0158	0.6842	-0.054	-1.147	-1.032	-0.36	-0.68	-0.45	-0.16	-1.32	-0.25	0.677	-0.33	-0.2	0.881231	0.159807	-0.038269982	0.198076496
2365	Q12841	FSTL1	follistatin like 1	11167	ENSG00000163430	19	-0.01	-0.12	-0.38	-0.18	0.046	-0.16	-0.37	0.096	0.1	0.5661	-0.177	0.0059	-0.185	-0.298	-0.179	0.6973	-0.062	0.2333	-0.34	-0.01	0.157	-0.18	-0.3	0.344	0.268	0.2	-0.31	0.568192	-0.08959	-0.175108095	0.085516791
2515	Q15365	PCBP1	poly(rC) binding protein 1	5093	ENSG00000169564	15	0.054	0.187	-0.04	-0.09	-0.15	-0.56	-0.13	-0.17	0.067	0.0797	0.0352	0.0918	-0.076	-0.306	-0.084	-0.04	0.2019	0.0707	-0.13	-0.92	-0.3	-0.7	-0.46	-0.09	-0.14	-0.05	0.166	0.188433	0.200468	-0.089454383	0.28992247
1247	O76054	SEC14L2	SEC14 like lipid binding 2	23541	ENSG00000100003	28	-0.41	0.048	-0.42	-0.02	-0.18	-0.42	0.042	0.147	0.294	0.2561	0.1445	-0.08	-0.133	-0.163	0.1641	0.1286	-8E-04	0.1529	-0.23	-0.42	0.288	-0.31	-0.18	0.25	-0.06	-0	0.429	0.565934	-0.07556	-0.154687715	0.079125793
2205	P62805	HIST1H4A	histone cluster 1 H4 family member a;histon	8359;55431;	ENSG00000278637	13	-1.22	-1.36	-1.21	-0.29	-0.33	0.906	-0.35	-0.89	-0.31	-2.114	-0.703	-1.639	-1.103	-0.892	-1.349	-0.956	-1.74	-1.999	-1.33	-0.98	2.684	1.973	-0.79	0.823	-1.39	-1.88	-2.23	0.290279	-0.21536	0.826699764	-1.042057759
2037	P51153	RAB13	RAB13, member RAS oncogene family	5872	ENSG00000143545	17	0.125	-0.5	0.188	-0.08	-0.14	-0.33	0.065	-0.02	0.132	0.1832	0.2027	0.1504	0.1332	-0.409	0.1755	0.1139	0.2479	0.0714	-0.42	-0.94	-0.31	-0.64	-0.41	0.073	-0.09	-0.16	0.033	0.091889	0.256097	-0.15860581	0.414702608
3468	Q9NQT8	KIF13B	kinesin family member 13B	23303	ENSG00000197892	66	-0.19	-0.08	-0.14	-0.12	-0.13	-0.33	0.155	-0.24	0.215	0.3317	-0.018	0.1362	-0.136	0.0458	0.1696	-0.042	-0.004	-0.119	-0.34	-0.7	-0.29	-0.64	-0.28	#####	-0.08	0.062	0.377	0.241075	0.114146	-0.136238657	0.25038514
2393	Q13237	PRKG2	protein kinase cGMP-dependent 2	5593	ENSG00000138669	40	-0.27	-0.12	-0.24	-0.1	-0.19	-0.9	-0.32	0.317	0.083	0.2163	0.1618	0.4328	0.1928	-0.2	0.0494	-0.253	0.3342	-0.14	0.365	-0.32	-0.81	-0.73	-1.05	-0.25	0.485	0.708	0.309	0.510785	-0.05046	-0.282450431	0.231990507
3070	Q93099	HGD	homogentisate 1,2-dioxygenase	3081	ENSG00000113924	23	0.247	0.109	0.013	0.046	0.064	-0.71	0.116	-0	0.178	0.1397	0.166	0.1868	-0.048	0.053	0.2423	0.2858	0.1123	-0.112	-0.32	-1.2	-0.23	-0.75	-0.33	-0.23	0.25	0.364	0.164	0.21502	0.258932	-0.1079029	0.366834941
2067	P53004	BLVRA	biliverdin reductase A	644	ENSG00000106605	15	0.183	0.306	0.13	-0.05	-0.07	-0.47	-0.09	-0.3	0.087	0.4353	0.155	0.3943	0.1403	0.0437	-0.027	0.0772	0.2188	0.0334	-0.28	-0.68	0.02	-0.34	-0.37	0.127	-0.19	0.016	-0.1	0.088	0.168136	-0.193705581	0.361841702

3284	Q9BV40	VAMP8	vesicle associated membrane protein 8	8673	ENSG00000118640	10	-0.41	0.163	0.016	0.013	-0.3	-0.33	0.141	-0.09	0.172	0.1841	0.1719	-0.019	0.1012	-0.267	0.1755	0.1894	0.0345	0.2133	-0.22	-0.5	-0.33	-0.49	-0.42	0.133	0.247	0.035	-0.08	0.187642	0.112434	-0.15603974	0.26847326
3432	Q9HB40	SCPEP1	serine carboxypeptidase 1	59342	ENSG00000121064	17	0.154	-0.48	-0.08	-0.49	-0.13	-0.25	-0.19	-0.3	0.148	0.0179	0.1667	-0.374	-0.458	0.2296	-0.506	-0.095	-0.613	-0.385	0.467	0.595	0.054	0.118	0.282	-0.01	0.396	0.03	-0.45	0.118484	-0.34519	0.043897099	-0.389082214
2450	Q14247	CTTN	cortactin	2017	ENSG00000085733	34	0.181	0.364	-0.14	-0.17	0.058	-0.5	0.023	-0.12	0.211	-0.078	-0.006	0.0819	0.0594	-0.011	0.166	0.0949	0.2972	0.1519	-0.52	-1.37	-0.35	-0.6	-0.33	-0.01	0.042	0.049	0.07	0.132428	0.323546	-0.094989783	0.418535494
1653	P17612	PRKACA	protein kinase cAMP-activated catalytic subı	5566	ENSG00000072062	25	0.143	0.155	-0.37	0.149	0.056	-0.23	0.51	0.289	0.339	-0.536	-0.133	-0.272	0.0303	0.1715	-0.221	0.6342	0.4565	0.7916	-0.5	-0.72	-0.34	-0.82	-0.1	-0.09	0.207	0.173	0.594	0.400562	0.291714	0.012745703	0.278968179
1630	P15529	CD46	CD46 molecule	4179	ENSG00000117335	9	-0.1	0.02	0.091	-0.27	-0.39	-0.37	0.117	-0.11	-0.05	0.0482	0.2177	-0.088	0.1711	-0.274	-0.184	0.224	0.3572	0.1161	-0.31	-0.42	-0.27	-0.54	-0.4	0.249	-0.15	-0.04	0.291	0.231011	0.057795	-0.184072521	0.241867346
1168	O60635	TSPAN1	tetraspanin 1	10103	ENSG00000117472	6	-0.93	0.12	-1.19	0.244	-0.41	-0.5	-0.54	0.072	0.256	-0.831	-1.039	0.068	-0.052	-0.693	-0.113	0.7003	0.228	1.0795	-0.6	-0.01	-0.71	-0.88	-0.74	-0.49	-0.08	0.632	1.128	0.804797	-0.12525	-0.247398794	0.122153034
1191	O75131	CPNE3	copine 3	8895	ENSG00000085719	27	-0.26	0.108	-0.14	-0.06	-0.18	-0.34	-0.05	-0.17	0.147	-0.006	-0.08	0.0052	-0.023	-0.32	-0.043	0.3951	0.276	-0.089	-0.07	-0.25	-0.14	-0.42	-0.14	-0.19	0.061	0.01	0.066	0.398882	0.014515	-0.117850563	0.132365775
1380	P02511	CRYAB	crystallin alpha B	1410	ENSG00000109846	17	-0.13	-0.26	-0.01	0.032	0.149	-0.62	0.349	-0.22	0.226	-0.433	1.2225	-0.156	-0.352	0.1854	0.1725	-0.196	0.4444	0.1666	-0.74	-0.85	-0.27	-0.92	-0.52	-0.42	0.218	-0.37	0.148	0.147939	0.360586	-0.170794993	0.53138146
1201	O75351	VPS4B	vacuolar protein sorting 4 homolog B	9525	ENSG00000119541	31	0.037	0.186	-0.03	0.073	0.084	-0.59	0.25	0.177	0.24	0.0014	0.147	0.2022	0.1451	-0.046	0.0481	0.3313	0.2977	0.3607	-0.19	-0.58	-0.19	-0.71	-0.34	-0.06	-0.04	0.071	0.55	0.175017	0.21321	-0.117749353	0.330959465
1558	P11234	RALB	RAS like proto-oncogene B	5899	ENSG00000144118	18	-0.16	0.502	-0.47	0.379	-0.08	-0.53	-0.14	-0.11	-0.1	-0.126	-0.003	-0.165	0.1587	-0.293	0.6701	0.3119	0.195	0.0434	-0.75	-0.69	-0.67	-0.95	-0.68	-0.07	-0.38	-0.09	0.281	0.10225	0.36504	-0.166719777	0.531759928
1327	P00390	GSR	glutathione-disulfide reductase	2936	ENSG00000104687	25	-0.11	0.378	0.142	-0.03	-0.07	-0.49	-0.07	-0.17	0.158	-7E-04	-0.02	-0.147	-0.032	-0.108	0.1736	-0.151	-0.074	-0.149	-0.01	-0.79	-0.19	-0.38	-0.24	0.15	0.045	0.165	0.167	0.804154	0.089542	0.026783405	0.062758769
1041	O14672	ADAM10	ADAM metallopeptidase domain 10	102	ENSG00000137845	20	-0.03	-0.13	-0.02	-0.56	-0.03	0.242	-0.34	-0.08	-0.33	0.108	0.3028	-0.018	-0.559	0.2516	-0.298	0.0319	0.0017	-0.242	-0.22	0.403	-0.01	-0.45	-0.38	-0.04	0.475	0.177	-0.43	0.826223	-0.08819	-0.093458711	0.005267625
3786	Q9Y678	COPG1	coatomer protein complex subunit gamma 1	22820	ENSG00000181789	37	0.063	0.061	-0.13	-0.18	-0.14	-0.09	-0.05	0.161	0.138	0.0108	-0.052	-0.008	-0.113	-0.067	-0.019	0.0918	0.1103	0.3204	-0.21	-0.39	-0.07	-0.27	-0.02	-0.07	0.153	0.135	0.222	0.637542	0.038618	-0.0492112	0.087829631
1593	P13716	ALAD	aminolevulinate dehydratase	210	ENSG00000148218	22	-0.17	-0.04	-0.14	-0.14	0.027	-0.48	0.047	0.098	0.026	0.5634	-0.329	-0.08	-0.136	-0.199	-0.012	0.3491	0.0624	0.0913	0.025	-0.21	-0.28	-0.03	-0.19	0.069	0.176	0.158	0.092	0.631441	-0.06347	-0.119783951	0.056313113
1338	P00966	ASS1	argininosuccinate synthase 1	445	ENSG00000130707	20	-0.42	-0.44	0.06	-0.6	0.091	-0.19	0.275	0.245	-0.4	-0.369	0.1324	-0.232	-0.581	-0.551	-0.029	-0.338	0.5938	0.4761	-0.87	-0.17	0.664	-0.65	0.067	-0.13	0.827	-0.09	-0.37	0.960269	-0.0713	-0.052139777	-0.019157745
1011	O00462	MANBA	mannosidase beta	4126	ENSG00000109323	38	-0.4	-0.92	-0.09	-0.37	-0.46	0.227	0.013	-0.06	0.348	0.2853	-0.169	0.1347	-0.582	0.5855	-0.142	0.1966	-0.119	-0.053	0.027	0.195	0.397	0.177	0.174	-0.1	0.53	0.299	0.025	0.183595	-0.3809	-0.204620284	-0.176277185
2024	P50570	DNM2	dynamin 2	1785	ENSG00000079805	39	0.115	0.088	-0.05	-0.19	0.068	-0.35	-0.06	-0.03	0.226	0.0017	-0.008	0.0808	-0.021	-0.073	-0.056	-0.206	0.0467	0.1941	-0.17	-0.3	0.023	-0.19	-0.2	-0.05	-0	-0.12	-0	0.363101	0.091441	-0.015731688	0.107172789
1668	P19075	TSPAN8	tetraspanin 8	7103	ENSG00000127324	9	-0.71	0.638	0.301	-0.13	-0.05	-0.81	-0.38	-1.15	-0.85	0.5805	0.5782	0.0559	0.3689	0.1679	0.1755	-0.395	0.0737	-0.079	-0.14	0.369	-0.54	-0.37	-0.72	0.13	-0.17	0.076	-0.01	0.181712	-0.19763	-0.519867164	0.322240938
2562	Q16851	UGP2	UDP-glucose pyrophosphorylase 2	7360	ENSG00000169764	29	-0.12	-0.04	-0.19	-0.04	0.316	-0.59	0.23	0.314	0.201	0.2287	-0.26	-0.003	-0.119	-0.161	-0.127	0.4774	0.1869	0.2375	-0.32	-0.49	-0.2	-0.16	-0.39	-0.02	0.499	0.306	0.151	0.777312	0.078286	-0.041529336	0.119815471
1779	P28074	PSMB5	proteasome subunit beta 5	5693	ENSG00000100804	14	0.083	-0.42	-0.26	-0.12	0.264	-0.41	0.163	0.016	0.172	0.2801	-0.204	-0.2	-0.206	-0.109	-0.129	0.413	0.0764	0.1523	0.135	0.373	-0.19	-0.46	-0.18	-0.19	0.521	0.286	-0.31	0.911948	-0.05631	-0.065204573	0.008894521
902	I3L397	EIF5A	eukaryotic translation initiation factor 5A	1984	ENSG00000132507	14	0.114	0.229	0.046	-0.04	-0.06	-0.41	-0.33	-0.35	-0.12	0.1717	0.1648	0.0822	0.0891	-0.068	0.1686	-0.2	0.2143	-0.189	-0.23	-1.15	-0.27	-0.64	-0.26	0.062	-0.34	-0.17	-0.01	0.122988	0.232385	-0.150835688	0.383220837
2326	Q06323	PSME1	proteasome activator subunit 1	5720	ENSG00000284916	17	0.012	0.176	0.06	-0.19	-0.04	-0.68	-0.33	-0.52	-0.3	-0.205	0.1168	0.0727	0.0867	0.0447	-0.076	-0.387	-0.279	-0.378	-0.02	-0.7	0.107	-0.38	-0.38	-0.04	-0.16	-0.27	-0.11	0.732909	0.01566	-0.090207457	0.105867499
707	F5H2F4	MTHFD1	methylenetetrahydrofolate dehydrogenase, c	4522	ENSG00000100714	40	-0.07	-0.03	-0.11	-0.06	-0.04	-0.41	0.497	-0.22	0.132	0.0548	0.0626	0.0406	0.0032	-0.129	0.0052	0.1721	0.0816	0.2031	-0.21	-0.67	-0.16	-0.34	-0.39	0.009	0.201	-0.09	0.384	0.4016	0.10575	-0.088898326	0.194648288
519	C9JIF9	APEH	acylaminoacyl-peptide hydrolase	327	ENSG00000164062	31	-0.03	-0.01	-0.1	-0.02	0.144	-0.57	0.199	0.105	0.195	0.2088	0.0094	0.0448	-0.065	-0.287	-0.139	0.3795	0.1265	0.1071	-0.3	-0.43	-0.13	-0.48	-0.25	-0.22	0.383	0.257	-0.07	0.44572	0.128576	-0.052734051	0.181310312
988	O00159	MYO1C	myosin IC	4641	ENSG00000197879	50	0.074	0.365	-0.45	0.332	0.191	-0.32	-0.23	-0.18	-0.11	-0.137	-0.081	0.3929	-0.058	-0.092	0.3949	0.3349	-0.104	0.2451	-0.38	-0.23	-0.54	-0.51	-0.47	-0.31	-0.08	0.092	0.425	0.208742	0.183762	-0.137480787	0.321242564
2782	Q7Z3U7	MON2	MON2 homolog, regulator of endosome-to-(	23041	ENSG00000061987	51	-0.09	0.203	-0.1	-0.08	-0.1	-0.25	-0.2	-0.04	0.146	0.2659	0.1538	0.2326	0.1346	-0.133	-0.219	0.3153	-0.104	0.211	-0.29	-0.67	-0.31	-0.55	-0.27	0.449	-0.13	0.011	0.075	0.195196	0.13109	-0.15289536	0.283985492
2031	P50991	CCT4	chaperonin containing TCP1 subunit 4	10575	ENSG00000115484	34	0.143	0.028	-0.01	0.286	0.266	-0.39	0.166	0.025	0.159	0.0437	-0.057	-0.019	0.0145	-0.241	-0.058	0.1947	-0.068	0.0486	-0.16	-0.8	-0.18	-0.54	-0.42	-0.03	0.164	-0.04	0.007	0.139046	0.296983	0.090359034	0.206624043
1223	O75695	RP2	RP2 activator of ARL3 GTPase;RP2, ARL3	6102	ENSG00000102218	15	-0.04	0.362	-0.1	0.015	-0.14	-0.31	-0.34	-0.19	-0.09	0.0985	0.0825	0.1034	-0.142	-0.329	-0.072	-0.077	0.1185	-0.07	-0.24	-0.31	-0.28	-0.31	-0.25	3E-04	-0.16	0.164	0.147	0.625678	0.043902	-0.060292952	0.104195063
1343	P01023	A2M	alpha-2-macroglobulin	2	ENSG00000175899	44	0.262	-1.04	1.272	0.118	0.343	-0.28	-0.02	0.361	0.046	-0.679	-0.425	0.1872	-0.588	-0.768	-0.601	-0.574	-0.253	-0.77	-1.21	-0.56	0.037	1.797	-0.62	-0.46	-0.45	-0.24	-1.01	0.28382	0.420832	0.615135043	-0.194303003
1978	P48723	HSPA13	heat shock protein family A (Hsp70) membe	6782	ENSG00000155304	23	-0.08	-0.71	-0.2	-0.51	-0.13	-0.17	-0.08	0.243	0.143	0.1554	0.0209	-0.326	-0.567	0.403	-0.104	0.0934	-0.23	-0.019	0.175	0.789	0.268	-0.2	-0.06	-0.18	0.641	0.13	-0.19	0.256188	-0.31846	-0.101850487	-0.216606112
2204	P62753	RPS6	ribosomal protein S6	6194	ENSG00000137154	17	-0.05	0.095	0.172	-0.34	0.551	-0.42	0.563	0.084	0.303	-0.19	-0.019	-0.119	-0.225	-0.251	-0.119	-0.105	0.6938	0.2321	-0.16	-0.06	0.008	-0.24	-0.29	-0.08	0.357	-0.17	0.375	0.701175	0.134759	0.117789879	0.016969421
1510	P09211	GSTP1	glutathione S-transferase pi 1	2950	ENSG00000084207	11	-0.07	0.015	-0.14	0.033	0.125	-0.38	0.323	0.01	0.372	-0.047	-0.397	-0.114	-0.232	-0.184	0.0612	-0.035	0.4667	-0.218	-0.76	-0.91	0.233	-0.9	-0.18	-0.17	0.282	0.134	0.069	0.401468	0.275589	0.109984163	0.16560526
3057	Q92896	GLG1	golgi glycoprotein 1	2734	ENSG00000090863	52	-0.26	-0.25	-0.19	-0.18	-0.25	-0	-0.1	-0.02	0.072	0.5195	-0.019	0.2978	-0.472	0.0066	-0.428	0.1784	-0.172	-0.1	-0.09	0.484	0.131	-0.2	-0.1	-0.28	0.483	0.275	-0.48	0.617498	-0.1574	-0.11096704	-0.046432785
1045	O14745	SLC9A3R1	SLC9A3 regulator 1	9368	ENSG00000109062	24	0.055	0.349	-0.02	0.021	0.162	-0.4	-0.23	-0.3	-0.04	-0.355	0.2197	0.1361	0.2828	-0.155	0.1975	-0.049	0.0194	0.3536	-0.29	-0.31	-0.4	-0.42	-0.37	-0.34	-0.2	-0	0.227	0.137957	0.18998	-0.116962716	0.30694242
1432	P05109	S100A8	S100 calcium binding protein A8	6279	ENSG00000143546	15	-2.46	-2.49	-1.16	-0.92	-2.21	2.257	0.269	-2.46	-2.18	-2.525	-0.169	-1.971	0.7128	-1.597	-1.56	-1.178	-2.78	-2.712	-2.02	-2.05	1.78	2.539	0.351	0.458	-2.06	-2.42	-2.94	0.680918	-0.55352	0.269821011	-0.823341906
2044	P51580	TPMT	thiopurine S-methyltransferase	7172	ENSG00000137364	13	0.209	0.293	-0.1	0.047	-0.07	-0.47	0.046	0.068	0.248	0.1778	-0.577	0.2007	0.3386	-0.167	0.1349	-0.216	0.3756	0.1451	-0.12	-0.53	-0.19	-0.7	-0.39	0.171	-0.05	-0.02	0.232	0.355327	0.207854	-0.016290591	0.224144757
1050	O14818	PSMA7	proteasome subunit alpha 7	5688	ENSG00000101182	21	-0.17	-0.17	-0.07	-0.28	0.165	-0.63	-0.15	-0.38	-0.17	0.1942	-0.146	-0.059	0.0712	-0.152	-0.327	0.3428	0.1094	0.0389	-0.37	-0.04	-0.22	0.153	0.004	-0.07	0.468	0.091	-0.42	0.301916	-0.16194	-0.213638438	0.051694604
1178	U3KQK0	HIST1H2B	histone cluster 1 H2B family member n	8341	ENSG00000233822	8	-1.09	-1.45	-1.09	-0.79	-0.52	1.034	-0.55	-0.84	-0.75	-1.508	-0.692	-1.092	-1.213	-0.874	-1.558	-0.975	-1.866	-1.87	-0.99	-0.46	2.538	2.285	-0.92	1.007	-1.53	-1.7	-2.45	0.315569	-0.42494	0.623778906	-1.048715568
3635	Q9UJC3	HOOK1	hook microtubule tethering protein 1	51361	ENSG00000134709	37	0.215	0.181	-0.08	-0.09	-0.06	-0.42	-0.01	-0.02	0.235	0.1486	0.0391	0.0604	-0.143	-0.096	0.0919	0.0207	0.3684	0.1821	-0.36	-1.31	-0.2	-0.71	-0.5	-0.17	-0.1	-0.08	0.324	0.121069	0.341065	-0.079468585	0.420533509
88	P09668	CTSH	cathepsin H	1512	ENSG00000103811	19	-0.12	-0.53	-0.26	0.041	-0.96	-0.25	-0.31	0.196	0.285	0.0261	-0.122	-0.502	-0.761	0.6862	-1.13	0.3799	-0.121	0.1924	0.468	-0.08	0.57	-0.06	-0.09	-0.01	0.969	0.361	-0.05	0.236652	-0.44273	-0.060935277	-0.381791113
351	A0MZ66	SHTN1	shootin 1	57698	ENSG00000187164	40	0.148	0.053	-0.18	0.017	0.089	-0.38	-0.07	-0.09	0.128	0.0169	-0.015	0.1377	0.0456	0.0099	0.1963	0.0627	0.0841	-0.02	-0.07	-0.51	-0.22	-0.65	-0.16	0.101	0.06	0.086	0.069	0.239604	0.112575	-0.090166581	0.202741227
1715	P22102	GART	phosphoribosylglycinamide formyltransferas	2618	ENSG00000262473	31	0.135	0.178	0.116	-0.01	-0.1	-0.33	-0.01	0.015	0.177	-0.015	-0.014	0.1434	-0.022	-0.087	0.1034	-0.054	0.1837	0.057	-0.09	-0.95	-0.15	-0.52	-0.27	0.031	-0.03	0.027	-0.04	0.146569	0.240725	-0.013572967	0.254297625
2951	Q8NFU3	TSTD1	thiosulfate sulfurtransferase like domain cor	100131187	ENSG00000215845	11	-0.18	0.353	-0.11	-0.03	0.063	-0.49	0.28	-0.02	0.23	0.1907	0.1353	0.0485	0.0227	0.0345	0.0547	0.2337	0.438	-0.064	-0.29	-1.56	-0.29	-0.62	-0.58	-0.02	-0.28	-0.01	0.437	0.137061	0.368066	-0.111040729	0.479106854
1716	P22234	PAICS	phosphoribosylaminoimidazole carboxylase	10606	ENSG00000128050	29	0.074	0.025	0.063	2E-04	0.229	-0.41	0.106	0.077	0.196	0.1322	-0.009	0.017	-0.039	-0.152	0.1193	0.2142	0.2431	0.0418	-0.1	-0.67	-0.11	-0.59	-0.29	-0.07	0.279	0.064	0.052	0.219291	0.198493	-0.023333731	0.221826541
2065	P52907	CAPZA1	capping actin protein of muscle Z-line subur	829	ENSG00000116489	16	0.109	0.16	0.008	-0.13	0.013	-0.43	0.252	0.08	0.312	0.0033	0.0117	######	0.0225	-0.156	-0.013	0.2186	0.2512	0.157	-0.26	-0.64	0.106	-0.44	-0.31	0.069	0.02	0.162	0.253	0.392913	0.158512	-0.013314156	0.171825676
2344	Q08722	CD47	CD47 molecule	961	ENSG00000196776	9	-0.07	0.11	0.092	-0.08	0.008	-0.45	-0.51	-0.39	-0.13	0.0118	0.4269	-0.243	0.1234	-0.174	0.3343	-0.04	0.4289	0.2238	-0.26	-0.37	-0.48	-0.55	-0.63	-0.07	-0.16	-0.06	0.142	0.088122	0.11431	-0.280114735	0.394425
2656	Q5VW32	BROX	BRO1 domain and CAAX motif containing	148362	ENSG00000162819	18	-0.6	-0.05	-0.49	0.152	-0.24	-0.29	-0.31	0.028	-0.29	-0.636	-0.583	0.0968	-0.16	-0.313	0.0171	0.0943	0.061	0.2108	-0.47	-0.36	-0.71	-0.58	-0.38	-0.3	-0.35	-0.04	0.425	0.611831	0.075008	-0.09785151	0.172859705
1313	O95834	EML2	EMAP like 2;echinoderm microtubule assoc	24139	ENSG00000125746	27	0.124	0.067	-0.07	-0.11	-0.12	-0.44	-0.04	-0.06	0.163	0.0587	0.0972	0.0415	0.0103	-0.107	0.0231	0.3743	0.1534	0.0908	-0.27	-1.01	-0.25	-0.52	-0.4	0.038	0.141	0.165	0.113	0.19362	0.168278	-0.136113317	0.304391696
1941	P46059	SLC15A1	solute carrier family 15 member 1	6564	ENSG00000088386	12	-0.59	0.06	0.503	-0.23	0.601	-0	-0.29	0.332	-0.37	-0.461	-0.262	-0.124	-0.231	0.1159	-0.204	0.4299	-0.061	0.7949	-0.81	-0.9	-0.1	-0.47	0.175	0.405	-0.82	-1.07	0.04	0.269628	0.394288	0.000960877	0.393327414
2445	Q14166	TTLL12	tubulin tyrosine ligase like 12	23170	ENSG00000100304	33	0.123	0.26	-0.03	-0.05	0.006	-0.42	-0.13	-0.18	0.085	-0.022	0.1393	0.2715	0.1701	-0.009	-0.023	0.1826	0.1338	0.3751	-0.09	-0.58	0.034	-0.42	-0.34	0.188	0.005	0.042	0.509	0.309757	0.034725	-0.172971773	0.207696478
1548	P10644	PRKAR1A	protein kinase cAMP-dependent type I regul	5573	ENSG00000108946	20	0.166	-0.17	0.055	0.361	0.571	-0.28	0.443	0.136	0.416	-0.312	0.1916	-0.187	-0.039	-0.294	-0.219	-0.05	0.2635	0.0937	-0.24	-0.68	-0.07	-0.37	-0.14	-0.1	0.269	-0.19	-0.03	0.122408	0.362312	0.251004354	0.111307587
1561	P11413	G6PD	glucose-6-phosphate dehydrogenase	2539	ENSG00000160211	32	0.055	0.013	-0.05	-0.2	0.098	-0.35	-0.12	-0.07	-0.08	0.0223	0.0128	-0.101	-0.11	-0.175	-0.039	0.0947	0.0427	-0.136	-0.27	-0.78	0.107	0.249	-0.4	0.125	0.064	-0.04	0.1	0.917279	0.016923	-0.034056587	0.050979205
3178	Q96PU5	NEDD4L	neural precursor cell expressed, developmei	23327	ENSG00000049759	43	0.022	0.226	-0.12	0.116	0.032	-0.31	-0.14	-0.16	0.093	-0.037	0.1573	0.2809	-0.015	-0.019	0.0426	-0.076	0.0201	0.0104	-0.29	-0.32	-0.21	-0.26	-0.23	-0.01	-0.01	5E-04	0.299	0.301943	0.089954	-0.066149687	0.156104093
1631	P15880	RPS2	ribosomal protein S2	6187	ENSG00000140988	14	-0.01	-0.21	-0.18	-0.15	0.19	-0.21	0.19	-0.07	0.216	-0.124	-0.143	-0.169	-0.289	0.0551	-0.118	0.0585	0.2196	0.3128	-0.15	-0.16	-0.09	-0.09	-0.12	-0.07	0.282	-0.28	0.073	0.883747	0.044555	-0.002581513	0.04713688
2026	P50591	TNFSF10	TNF superfamily member 10	8743	ENSG00000121858	17	-0.19	-0.2	-0.34	-0.54	0.153	-0.35	-0.12	0.34	-0.46	-0.062	-0.123	0.2608	-0.335	0.3146	-0.517	-0.212	0.073	0.4157	-0.17	0.187	0.352	-0.24	-0.69	-0.15	0.059	-0.56	0.232	0.662331	-0.08069	-0.169158575	0.088465578
1404	P04004	VTN	vitronectin	7448	ENSG00000109072	15	-0.64	-0.69	-0.33	-0.8	-0.21	-0.32	-0.28	0.321	-0.05	0.1843	0.1407	-0.045	-0.914	0.7944	-0.49	-0.094	-0.416	0.0018	-0.28	0.184	0.518	0.355	-0.11	-0.04	0.409	-0.13	-0.54	0.306226	-0.37304	-0.239072923	-0.133966932
422	H3BLV0	CD55	CD55 molecule (Cromer blood group)	1604	ENSG00000196352	18	-0.51	0.201	-0.1	0.015	0.622	-0.04	-0.1	-0.25	-0.24	-0.056	0.6132	0.1485	-0.064	-0.42	-0.138	-0.363	0.3325	-0.281	-0.82	0.113	-0.63	-0.37	-0.56	-0.28	0.515	-0.09	0.907	0.89572	0.089581	-0.020734931	0.110315868
2222	P62942	FKBP1A	FKBP prolyl isomerase 1A;FK506 binding p	2280	ENSG00000088832	8	-0.08	-0.04	-0.24	-0.05	0.081	-0.32	0.628	0.375	0.668	0.0747	0.0564	-0.056	-0.154	-0.08	0.1598	0.7241	0.6336	0.8197	-0.25	-0.72	-0.4	-0.46	-0.24	0.078	0.239	0.301	0.855	0.450374	0.181616	-0.128043933	0.309659692
183	A0A0C4DFY	GPRC5C	G protein-coupled receptor class C group 5	55890	ENSG00000170412	11	-0.03	0.488	-0.22	-0.35	-0.36	0.096	-0.01	0.314	0.149	-0.455	0.2114	0.1174	0.1003	-0.253	-0.018	0.4288	0.1357	0.6168	-0.29	0.162	-0.29	-0.32	-0.46	-0.65	-0.09	0.397	0.639	0.622289	0.10938	-0.090357959	0.199737659
2840	Q8IV36	HID1	HID1 domain containing	283987	ENSG00000167861	19	0.199	0.353	-0.01	-0.09	-0.21	-0.07	0.056	-0.08	0.351	0.2261	-0.062	0.1213	0.3105	-0.211	-0.106	0.1662	0.142	0.0511	-0.27	-1.1	-0.04	-0.71	-0.26	0.118	-0.16	-0.25	0.176	0.129205	0.331043	-0.016222791	0.347265444
2778	Q7Z304	MAMDC2	MAM domain containing 2	256691	ENSG00000165072	29	-0.41	-1	-0.63	-0.16	-0.04	0.091	-0.23	0.127	-0.34	0.0753	0.4552	-0.2	-0.277	0.6631	-0.304	0.1135	-0.198	-0.129	-0.26	0.527	0.431	-0.32	-0.36	-0.33	0.654	-0.49	-0.26	0.368663	-0.24553	-0.312690603	0.067156889
1685	P20151	KLK2	kallikrein related peptidase 2	3817	ENSG00000167751	13	0.341	0.393	-0.51	-0.41	0.192	-0.02	-0.82	-0.04	0.027	-0.02	-0.007	-0.026	-0.111	-0.154	-0.259	0.4029	0.2419	0.3381	-0.36	-0.13	0.266	0.067	-0.7	0.024	-0.04	-0.2	0.11	0.658878	0.014361	-0.13834848	0.152709689
1751	P25786	PSMA1	proteasome subunit alpha 1	5682	ENSG00000129084	21	-0.1	-0.17	-0.03	-0.14	0.273	-0.55	-0.01	-0.13	0.021	0.1901	-0.14	-0.085	-0.066	-0.217	-0.354	0.4675	0.1854	0.0726	-0.38	-0.07	-0.2	0.212	-0.05	-0.25	0.481	0.115	-0.36	0.791838	-0.03712	-0.099089319	0.061970192
3030	Q92485	SMPDL3B	sphingomyelin phosphodiesterase acid like ;	27293	ENSG00000130768	15	-0.31	-0.17	-0.4	0.068	-0.16	-0.18	-0.15	-0.19	0.213	-0.199	0.3313	0.2678	-0.273	0.0751	-0.049	-0.108	-0.168	0.159	-0.37	0.341	-0.18	-0.63	-0.29	-0.18	-0.2	-0.29	-0.03	0.303749	0.060247	-0.146337195	0.206584526
2361	Q12768	WASHC5	WASH complex subunit 5	9897	ENSG00000164961	40	-0.12	0.045	0.156	-0.09	-0.18	-0.48	0.039	0.178	0.149	0.0471	0.2134	-0.113	-0.023	-0.124	-0.105	0.2547	0.4764	0.4423	-0.21	-0.82	0.063	-0.31	-0.39	0.01	-0	-0.09	0.094	0.178489	0.150974	-0.151505835	0.302479561
2578	Q3LXA3	TKFC	triokinase and FMN cyclase	26007	ENSG00000149476	28	0.012	0.01	-0.03	0.254	-0.34	-0.22	0.164	0.081	0.424	0.1071	-0.076	0.0368	-0.481	-0.274	0.2375	0.0146	-0.038	-0.049	-0.14	-0.72	-0.15	-0.46	-0.32	0.013	0.012	0.049	0.208	0.383067	0.206529	0.096885537	0.109643648
3536	Q9NY33	DPP3	dipeptidyl peptidase 3	10072	ENSG00000254986	30	0.05	0.333	0.054	-0.07	0.156	-0.39	0.122	0.055	0.179	-0.051	-0.077	0.0089	0.0249	-0.153	-0.047	0.0002	0.2045	-0.05	-0.22	-0.77	-0.21	-0.47	-0.34	-0.09	0.038	-0	0.243	0.172362	0.257466	0.070257191	0.187209232
2111	P55259	GP2	glycoprotein 2	2813	ENSG00000169347	17	-0.38	-0.12	-0.35	-0.51	-0.25	-0.5	-0.49	-0.47	0.312	-0.277	0.351	-0.279	-0.323	-0.42	0.1098	0.2651	-0.856	0.1598	-0.92	-0.09	0.801	0.083	-0.37	1.565	-0.22	0.175	-0.61	0.51199	-0.35296	-0.165600483	-0.187355271
3225	Q99747	NAPG	NSF attachment protein gamma	8774	ENSG00000134265	24	0.011	0.086	-0.27	-0.23	-0.41	-0.18	-0.22	0.144	0.224	-0.023	0.1337	0.0193	0.0028	-0.407	0.5737	0.2446	0.091	0.1624	-0.37	-1.22	-0.47	-0.81	-0.25	-0.07	-0.15	-0.23	0.372	0.139529	0.259972	-0.183712856	0.443684481
2529	Q15691	MAPRE1	microtubule associated protein RP/EB famil	22919	ENSG00000101367	24	0.131	0.251	-0	-0.1	-0.11	-0.29	0.115	-0.08	0.186	-0.015	-0.144	-0.141	-0.089	-0.154	0.1937	0.285	0.1826	0.2763	-0.42	-0.87	-0.21	-0.38	-0.3	-0.04	-0.09	-0.1	0.511	0.235081	0.221545	-0.032925111	0.25447017
2134	P60660	MYL6	myosin light chain 6	4637	ENSG00000092841	12	-0.09	0.109	-0.12	-0.16	-0	-0.58	0.124	-0.16	0.219	0.1594	-0.06	-0.059	0.064	-0.171	0.1853	0.2275	0.2304	-0.082	-0.21	-1.05	-0.12	-0.07	-0.48	-0	0.083	0.008	0.137	0.328267	0.116401	-0.126954509	0.243355637
2504	Q15181	PPA1	pyrophosphatase (inorganic) 1;inorganic pyr	5464	ENSG00000180817	19	0.138	0.259	0.158	-0.13	0.092	-0.31	0.132	0.182	0.056	0.0626	0.0591	-0.035	0.0267	-0.285	0.1402	-0.017	0.1178	0.0711	-0.23	-0.7	0.178	-0.34	-0.19	0.123	0.037	-0.01	0.088	0.330074	0.17981	0.048736821	0.131072985
394	A8MTF8	FAM3B	family with sequence similarity 3 member B	54097	ENSG00000183844	13	0.015	-0.85	-0.41	0.035	-0.51	0.41	-0.28	-0.48	0.454	-0.137	0.0418	0.0488	-0.298	0.4486	-0.106	0.4978	-0.388	-0.132	0.008	-0	-0.24	0.166	-0.02	-0.12	0.113	0.31	-0.24	0.568946	-0.17717	-0.177804018	0.000630637
1567	P12036	NEFH	neurofilament heavy	4744	ENSG00000100285	34	0.3	0.217	-0.14	0.145	-0.1	-0.53	-0.33	0.395	-0.05	-0.882	0.1224	-0.405	0.0513	0.1598	0.051	0.4113	0.3831	0.1484	-0.19	-0.69	-0.38	-0.48	-0.11	-0.18	0.167	-0.35	-0.04	0.357801	0.239087	-0.01493718	0.254023794
3722	Q9Y2S2	CRYL1	crystallin lambda 1	51084	ENSG00000165475	20	8E-04	0.289	-0.17	-0.25	-0.01	-0.29	-0.27	-0.2	0.005	0.2212	0.0262	0.0873	-0.112	0.0267	0.1327	0.0584	0.0569	0.0783	-0.24	-1.2	-0.43	-0.89	-0.36	0.138	-0.19	-0.04	0.291	0.152085	0.225402	-0.162471192	0.387873519
2156	P61160	ACTR2	actin related protein 2;ARP2 actin related pr	10097	ENSG00000138071	18	-0.12	-0.2	-0.2	-0.03	-0.03	-0.35	-0.13	-0.11	-0.03	-0.154	-0.249	-0.296	0.1091	-0.053	0.0733	0.0596	0.1827	-0.028	-0.07	-0.12	0.256	0.073	-0.33	0.071	-0.05	-0.1	-0.04	0.463738	-0.09615	-0.092204416	-0.003948131
1929	P43034	PAFAH1B'	platelet activating factor acetylhydrolase 1b r	5048	ENSG00000007168	22	0.144	0.079	-0.07	0.021	0.106	-0.43	0.062	0.006	0.182	-0.039	-0.034	0.069	-0.041	-0.051	0.0672	-0.021	0.2	0.0945	-0.25	-0.98	-0.32	-0.48	-0.39	-0	0.08	0.067	0.222	0.198369	0.23878	-0.01585033	0.254630674
2242	P67936	TPM4	tropomyosin 4	7171	ENSG00000167460	30	0.013	0.143	-0.12	-0.11	0.173	-0.49	0.034	-0.11	0.058	0.0062	0.1313	-0.11	0.0324	0.0111	-0.009	-0.073	0.1565	-0.243	-0.32	-0.92	-0.12	-0.41	-0.27	0.106	0.05	0.035	-0.06	0.312022	0.166196	-0.0342392	0.200435186
1621	P15170	GSPT1	G1 to S phase transition 1	2935	ENSG00000103342	33	0.098	0.097	-0.13	-0.04	-0.05	-0.41	-0.05	-0.05	0.206	0.0522	-0.04	0.0241	0.0371	-0.035	0.0392	0.016	0.2348	0.0965	-0.24	-0.99	-0.16	-0.54	-0.33	0.069	0.02	0.036	0.206	0.221501	0.176465	-0.084878276	0.261343582

2498	Q15102	PAFAH1B:	platelet activating factor acetylhydrolase 1b (	5050	ENSG00000079462	15	0.013	0.187	-0.25	0.008	0.172	-0.34	0.337	-0.09	-0.16	0.1706	-0.069	-0.12	-0.083	0.075	0.204	0.1741	-0.25	-0.145	-0.15	-0.81	-0.18	-0.21	-0.4	-0.01	0.616	-0.23	0.372	0.78096	0.097894	-0.008408127	0.106301783
2810	Q86UW7	CADPS2	calcium dependent secretion activator 2	93664	ENSG00000081803	47	-0.05	0.289	0.073	0.067	-0.13	-0.45	-0.08	-0.09	0.228	0.0528	0.2401	0.1403	0.1114	-0.208	0.0365	0.2562	0.1991	0.2366	-0.19	-0.69	-0.26	-0.51	-0.41	0.051	-0.08	-0.07	0.183	0.106556	0.203111	-0.134151226	0.33726183
3346	Q9H0E2	TOLLIP	toll interacting protein	54472	ENSG00000078902	16	-0.33	0.166	0.017	0.019	-0.28	-0.71	0.048	0.042	-0.04	-0.131	0.3369	-0.097	0.1876	-0.042	0.0462	0.324	0.525	0.271	-0.36	-0.22	-0.22	-0.54	-0.46	-0.18	-0.12	0.239	0.35	0.149151	0.050805	-0.27506219	0.325867298
1861	P35573	AGL	amylo-alpha-1, 6-glucosidase, 4-alpha-gluca	178	ENSG00000162688	54	0.105	-0.07	-0.28	-0.16	0.074	-0.33	-0.11	-0.14	-0.08	0.0293	0.0849	0.2029	-0.106	-0.226	0.3247	-0.021	-0.073	-0.08	-0.16	-0.48	-0.16	-0.41	-0.16	-0.14	0.207	-0.22	-0.1	0.199625	0.070041	-0.124997908	0.195039085
3037	Q92597	NDRG1	N-myc downstream regulated 1	10397	ENSG00000104419	13	0.078	0.392	-0.03	-0.31	-0.05	-0.53	-0.42	0.028	-0.3	0.2274	0.4903	0.3347	-0.036	-0.037	-0.148	-0.151	-0.058	-0.052	-0.27	-0.7	-0.19	0.097	-0.22	0.076	-0.04	-0.09	0.065	0.343502	0.015568	-0.189593669	0.205162115
2592	Q53GD3	SLC44A4	solute carrier family 44 member 4	80736	ENSG00000232180	16	0.163	0.681	-0.05	-0.01	-0.04	-0.55	-1.09	-0.57	-0.61	-0.606	0.3287	0.3015	0.4008	0.0454	-0.008	0.4152	0.2561	0.0683	-0.27	-0.08	-0.22	-0.51	-1.25	-0.24	-0.46	0.151	0.412	0.27002	0.042943	-0.364210457	0.407153848
1826	P31939	ATIC	5-aminoimidazole-4-carboxamide ribonuclec	471	ENSG00000138363	36	0.124	0.058	0.291	0.157	0.176	-0.34	0.075	0.022	0.089	-0.017	-0.042	-0.081	-0.385	0.0035	0.1617	0.0587	0.0796	-0.015	-0.22	-0.81	-0.11	-0.37	-0.36	0.042	-0.15	-0.1	0.247	0.158253	0.275472	0.098332592	0.177139215
1120	O43520	ATP8B1	ATPase phospholipid transporting 8B1	5205	ENSG00000081923	32	-0.25	0.55	-0.25	0.006	-0.03	-0.39	-0.21	-0.1	-0.16	-0.42	-0.032	0.588	0.0881	-0.136	-0.088	-0.001	0.1292	0.5463	-0.3	-0	-0.35	-0.66	-0.55	-0.34	-0.16	0.244	0.325	0.342388	0.106482	-0.167994168	0.274475945
46	A0A0G2JMF	AC012314	transmembrane channel like 4	147798	ENSG00000277667	22	-0.22	0.396	-0.15	-0.01	-0.1	-0.4	-0.14	0.062	-0.35	-0.348	0.0571	0.2797	0.1415	-0.117	-0.039	0.0444	-0.068	0.239	-0.41	-0.06	-0.11	-0.08	-0.22	-0.22	-0.23	-0.18	0.176	0.358452	0.044036	-0.122614057	0.166650404
2391	Q13228	SELENBP	selenium binding protein 1	8991	ENSG00000143416	22	-0.05	0.355	-0.05	#####	-0.01	-0.28	0.279	0.033	0.275	0.3086	-0.137	0.2181	-0.17	-0.2	0.26	0.1625	0.1954	0.0589	0.206	-0.82	-0.09	-0.46	0.099	0.062	-0.15	-0.05	0.137	0.356849	0.181527	-0.014953592	0.196480773
1115	O43488	AKR7A2	aldo-keto reductase family 7 member A2	8574	ENSG00000053371	17	-0.02	0.007	-0.17	-0.1	-0.28	-0.45	0.186	0.076	0.307	0.0053	0.0777	-0.042	-0.302	0.2921	0.0417	0.1897	0.212	0.2536	-0.2	-0.73	-0.2	-0.51	-0.37	-0.14	0.045	-0.01	0.359	0.224969	0.145063	-0.130735715	0.275799036
197	A0A0C4DG(	CAPNS1	calpain small subunit 1	826	ENSG00000126247	16	0.213	0.173	-0.05	0.016	-0.01	-0.33	0.161	-0.02	0.192	0.17	0.0888	-0.116	-0.036	0.1885	0.0541	0.3853	0.3955	0.1238	-0.18	-0.59	-0.11	-0.42	-0.23	0.009	-0.02	0.071	0.381	0.178607	0.159164	-0.101660738	0.260824902
1972	P48506	GCLC	glutamate-cysteine ligase catalytic subunit	2729	ENSG00000001084	33	-0.15	0.292	-0.19	-0.08	0.132	-0.22	0.073	-0.03	0.003	0.0643	0.0093	-0.061	0.0839	0.0497	0.3087	0.2857	0.2822	-0.064	0.305	-0.58	0.104	-0.51	-0.21	0.017	-0.12	-0.22	0.332	0.328295	0.079332	-0.125608902	0.204940743
2481	Q14894	CRYM	crystallin mu	1428	ENSG00000103316	18	0.01	0.332	-0.58	-0.85	0.655	-0.18	0.291	0.076	0.386	-0.207	-0.283	-0.336	-0.681	-0.534	0.0541	0.2521	-0.086	0.004	0.124	-0.87	-0.02	0.035	-0.58	0.112	0.025	-0.09	0.593	0.656274	0.08912	0.21734858	-0.128228791
143	A0A0A0MRE	MADD	MAP kinase activating death domain	8567	ENSG00000110514	50	0.187	0.257	-0.01	-0.09	-0.17	-0.33	0.242	-0.02	0.324	-0.06	0.1385	0.0086	0.0276	-0.219	-0.066	0.268	0.1813	0.1461	-0.23	-0.59	0.017	-0.39	-0.45	0.013	-0.05	-0.13	0.248	0.203036	0.215532	-0.004946711	0.220478652
160	A0A0A0MT6	FKBP15	FKBP prolyl isomerase 15	23307	ENSG00000119321	28	-0.11	-0.19	0.385	0.029	-0.43	-0.53	0.073	0.114	0.115	-0.028	0.0835	-0.156	-0.199	0.019	-0.016	0.153	0.2709	0.1157	-0.17	-0.82	0.248	-0.28	-0.36	0.029	-0.18	-0.03	0.023	0.438923	0.109527	-0.088371446	0.19789841
3385	Q9H444	CHMP4B	charged multivesicular body protein 4B	128866	ENSG00000101421	13	-0.04	0.259	-0.11	0.135	-0.17	-0.58	0.239	0.057	0.141	-0.109	0.0964	0.0533	0.0587	0.0941	0.0091	0.242	0.1921	0.2447	-0.24	-0.58	-0.12	-0.65	-0.55	-0.22	-0.12	-0.07	0.335	0.121528	0.240073	-0.105006393	0.345079398
2625	Q5SPY9	NPDC1	neural proliferation, differentiation and contr	56654	ENSG00000107281	8	-0.25	0.339	-0.09	0.137	-0.27	0.019	0.728	0.327	0.659	0.0501	0.3165	-0.189	0.1201	-0.271	0.3137	1.1661	0.5326	0.7877	-0.58	-0.97	-0.25	-0.44	-0.28	0.209	0.166	0.393	0.597	0.267751	0.305253	-0.137177866	0.442430411
2307	Q02952	AKAP12	A-kinase anchoring protein 12	9590	ENSG00000131016	44	0.154	-0.35	-0.28	-0.07	-0.51	0.087	-0.07	-0.15	-0.51	-0.09	0.509	0.0506	-0.585	0.1681	0.3245	0.3332	-0.237	-0.593	-0.21	-0.76	-0.28	-0.98	-0.09	0.045	-0.15	0.109	0.156	0.523058	0.050693	-0.175485781	0.226178415
1430	P05067	APP	amyloid beta precursor protein	351	ENSG00000142192	26	-0.02	-0.59	-0.2	-0.5	-0.42	-0.19	-0.38	-0.17	-0.13	0.5035	0.5189	0.1117	-0.271	0.4145	-0.082	0.1693	-0.352	0.2242	0.036	0.422	0.404	0.125	0.11	-0.26	-0.09	-0.07	-0.16	0.060262	-0.3467	-0.428002017	0.081301202
2377	Q13093	PLA2G7	phospholipase A2 group VII	7941	ENSG00000146070	26	-0.04	-0.66	0.037	0.284	-0.28	-0.43	-0.43	-0.31	-0.49	-0.304	0.3867	0.1203	-1E-03	0.3743	0.0531	0.23	-0.322	-0.189	-0.59	-0.39	-0.07	-0.78	-0.48	0.089	-0.19	0.238	0.037	0.226741	-0.02038	-0.296533907	0.276152528
2225	P62993	GRB2	growth factor receptor bound protein 2	2885	ENSG00000177885	24	-0.2	0.583	-0.26	0.066	-0.05	-0.17	-0.32	-0.05	-0.16	-0.197	0.2789	0.1698	0.0882	0.0255	-2E-04	0.199	0.4018	-0.251	-0.09	-0.04	-0.14	-0.41	-0.43	-0.1	-0.4	0.092	0.217	0.305985	0.081183	-0.141757593	0.222940223
3129	Q96FJ2	DYNLL2	dynein light chain LC8-type 2	140735	ENSG00000264364	9	0.327	0.294	0.055	0.095	0.212	-0.34	-0.5	-0.23	-0.12	0.024	0.133	0.0191	0.1454	0.1231	0.1064	-0.395	0.1373	-0.377	-0.23	-0.91	-0.29	-0.53	-0.43	0.137	-0.22	-0.25	-0.06	0.169648	0.287171	-0.012956039	0.300126698
3038	Q92598	HSPH1	heat shock protein family H (Hsp110) memb	10808	ENSG00000120694	33	0.133	0.065	-0.04	-0.2	0.245	-0.39	0.086	-0.03	0.138	0.1633	0.2142	0.3155	0.0169	-0.096	-0.211	-0.046	0.107	0.1336	-0.31	-0.32	-0.29	-0.43	-0.41	0.138	0.08	-0.14	0.036	0.146743	0.184026	-0.065513966	0.249540171
1748	P25685	DNAJB1	DnaJ heat shock protein family (Hsp40) mei	3337	ENSG00000132002	25	0.056	-0.09	-0.15	-0.01	0.031	-0.36	-0.1	-0.22	0.132	-0.132	-0.034	0.0624	0.0487	0.0847	0.162	-0.121	0.1076	-0.099	0.184	0.287	0.073	-0.35	-0.31	0.076	-0.12	-0.18	-0.01	0.666548	-0.03964	-0.087071298	0.047428038
3229	Q99828	CIB1	calcium and integrin binding 1	10519	ENSG00000185043	15	-0.3	0.275	-0.34	0.025	0.078	-0.25	0.234	0.27	-0.19	-0.421	-0.019	0.1794	0.1874	-0.036	-0.031	0.2796	0.0771	0.6302	-0.29	0.194	-0.15	-0.07	-0.15	-0.16	-0.04	-0.08	0.532	0.674713	0.002266	-0.116098104	0.118363762
1665	P18206	VCL	vinculin	7414	ENSG00000035403	48	0.233	0.063	0.011	-0.09	0.123	-0.36	0.098	0.033	0.211	0.0225	-0.022	0.0181	0.0125	-0.143	0.098	0.1372	0.0172	-0.009	-0.08	-0.53	0.035	-0.16	-0.04	0.347	0.101	0.055	0.033	0.82658	0.063096	0.02187205	0.04122402
1820	P31153	MAT2A	methionine adenosyltransferase 2A	4144	ENSG00000168906	18	0.064	-0	-0.15	-0.04	0.111	-0.26	0.066	0.094	0.095	0.0122	-0.311	-0.049	0.2408	-0.144	0.0982	0.0009	0.0538	0.0738	-0.16	-1.02	-0.23	-0.4	-0.23	-0.11	0.205	0.048	0.122	0.310972	0.19552	0.000591069	0.1949288
2271	P84095	RHOG	ras homolog family member G	391	ENSG00000177105	14	-0.13	-0.08	-0.11	-0.21	-0.19	-0.21	0.042	-0.03	0.076	0.0911	0.1623	-0.122	-0.121	0.259	-0.042	0.0699	0.3456	0.3995	0.143	0.087	0.196	-0.05	-0.16	0.091	-0.15	-0.04	0.239	0.13239	-0.13452	-0.208963059	0.074439407
1759	P26639	TARS1;TA	threonyl-tRNA synthetase 1;threonyl-tRNA s	6897	ENSG00000113407	33	0.233	-0.01	-0.06	-0.07	0.013	-0.47	-0.05	-0.02	0.097	-0.021	-0.039	-0.048	0.077	-0.192	-0.101	0.044	0.1492	-0.007	-0.06	-0.42	0.147	-0.45	-0.31	-0.04	0.16	0.04	0.02	0.702588	0.063364	-0.022742858	0.08610717
2508	Q15257	PTPA	protein phosphatase 2 phosphatase activatc	5524	ENSG00000119383	15	0.345	0.379	-0.12	0.051	-0.06	-0.25	0.04	-0.18	0.09	0.1002	0.1719	0.1769	0.0248	-0.07	0.078	0.0903	0.2195	0.0035	-0.42	-1.3	-0.41	-0.76	-0.26	0.093	-0.1	-0.14	0.202	0.100659	0.376745	-0.055969305	0.432714684
1479	P07741	APRT	adenine phosphoribosyltransferase	353	ENSG00000198931	15	0.137	0.348	0.077	-0.03	0.12	-0.45	0.198	0.043	0.156	0.1706	0.1334	0.0157	0.1877	-0.051	0.1382	0.0604	0.0339	0.1207	0.014	-0.71	-0.14	-0.63	-0.23	0.043	0.04	0.087	0.257	0.226296	0.208235	-0.023051124	0.231286488
525	C9JK28	ADAM7	ADAM metallopeptidase domain 7	8756	ENSG00000069206	34	0.402	-0.52	1.299	0.949	-0.29	0.269	-0.15	1.213	-0.04	-0.741	0.4974	0.8124	0.4626	-1.189	0.3552	-0.467	0.1515	-0.861	-0.18	-0.71	-1.19	0.03	-1.13	-1.58	-1.01	-0.27	-1.39	0.056331	1.17456	0.456225471	0.718334214
990	O00161	SNAP23	synaptosome associated protein 23	8773	ENSG00000092531	26	-0.06	0.277	-0.08	0.213	-0	-0.22	-0.1	-0.02	0.007	-0.109	0.0472	0.1423	0.1784	-0.156	0.1462	0.2698	0.2484	0.4086	-0.15	0.096	-0.19	-0.43	-0.31	-0.21	-0.09	0.01	0.356	0.178887	0.104635	-0.129070234	0.23370545
1485	P07948	LYN	LYN proto-oncogene, Src family tyrosine kin	4067	ENSG00000254087	31	-0.2	0.24	-0.11	-0.23	0.036	0.006	-0.27	-0.3	-0.01	-0.144	0.0088	-0.168	0.2753	-0.014	-0.017	-0.063	0.1371	-0.028	-0.16	0.671	-0.01	-0.06	-0.08	0.058	-0.4	-0.05	0.046	0.676412	-0.09506	-0.091588941	-0.003475338
1347	P01034	CST3	cystatin C	1471	ENSG00000101439	9	0.193	-0.45	0.171	0.155	0.377	0.033	0.136	0.346	0.574	0.3384	-0.196	0.1766	-0.646	-0.256	-0.611	0.3258	-0.219	-0.071	-0.16	0.022	0.009	-0.24	-0.01	-0.3	0.196	0.2	-0.49	0.226824	0.256975	0.299418237	-0.042443222
3022	Q8WXA2	PATE1	prostate and testis expressed 1	160065	ENSG00000171053	9	-0.72	0.781	-0.7	-0.38	0.168	-0.18	0.254	0.431	1.024	0.5505	-1.017	0.3488	-0.456	-0.216	-0.184	1.352	-0.55	-0.125	-0.17	0.916	0.407	-0.23	-0.09	-0.34	-0.02	-0.49	0.089	0.949041	0.06728	0.108525155	-0.041244703
2544	Q15847	ADIRF	adipogenesis regulatory factor	10974	ENSG00000148671	8	-0.55	0.534	-0.04	-0.01	-0.19	-0.23	0.439	-0.03	0.367	0.2347	0.1167	-0.319	0.2117	-0.118	0.3892	0.6056	0.7054	-0.01	-0.35	-1.04	-0.52	-1.7	-0.66	0.017	-0.2	0.117	0.078	0.099555	0.504291	-0.169966236	0.674257023
3326	Q9BZV1	UBXN6	UBX domain protein 6	80700	ENSG00000167671	19	0.008	0.04	-0.05	-0.03	0.044	-0.36	0.259	0.119	0.207	-0.076	0.0193	0.0204	0.0179	-0.078	0.1747	0.2372	0.1204	0.1224	-0.28	-0.82	-0.38	-0.57	-0.28	-0.08	0.15	0.043	0.325	0.174305	0.236199	-0.036569281	0.272768362
1549	P10768	ESD	esterase D	2098	ENSG00000139684	16	0.08	0.024	-0.19	0.058	0.013	-0.27	0.067	0.221	0.396	0.0553	0.0712	-0.108	0.0366	-0.186	0.2031	0.2811	0.2001	0.1422	-0.3	-1.03	-0.29	-0.68	-0.34	-0.06	0.163	-0.1	0.703	0.269755	0.258834	-0.033568799	0.292403178
2408	Q13443	ADAM9	ADAM metallopeptidase domain 9	8754	ENSG00000168615	44	-0.25	0.118	-0.06	-0.25	-0.21	-0.43	-0.23	-0.01	0.02	-0.05	0.1078	0.1147	-0.199	-0.032	-0.177	0.2014	0.1486	0.3284	-0.17	0.074	-0.04	-0.37	-0.25	0.249	-0.09	0.096	-0.01	0.227922	-0.08922	-0.194234221	0.105009244
863	H3BTI0	CRISPLD2	cysteine rich secretory protein LCCL domair	83716	ENSG00000103196	17	-0.54	-0.47	-0.15	0.042	-0.54	-0.44	0.066	0.29	0.542	0.0402	0.1417	0.3582	-0.156	-0.014	-0.613	0.279	-0.573	-0.274	-0.39	-0.02	0.201	0.108	0.225	0.072	0.51	0.167	-0.13	0.520702	-0.21583	-0.042976908	-0.1728558
3	Q00341	HDLBP	high density lipoprotein binding protein	3069	ENSG00000115677	56	0.178	-0	-0.14	0.005	-0.07	-0.29	0.008	6E-04	0.254	0.0374	0.0777	0.0582	-0.083	0.0315	0.1703	0.105	0.1712	0.163	-0.2	-0.66	-0.15	-0.58	-0.26	0.039	0.103	0.177	0.32	0.279256	0.128044	-0.087993599	0.216037384
986	O00139	KIF2A	kinesin family member 2A	3796	ENSG00000068796	28	0.152	0.063	-0.34	-1.69	-1.9	-1.83	0.229	0.163	0.186	-0.018	-0.089	0.0382	-1.844	-1.335	-1.338	0.2106	0.0884	0.0712	-0.63	-1.07	-0.36	-2.12	-2.13	-1.8	-0	0.049	0.469	0.764117	0.291683	-0.083661089	0.375343686
3779	Q9Y5Z0	BACE2	beta-secretase 2	25825	ENSG00000182240	14	-0.15	0.627	-0.08	-0	-0.53	-0.67	0.058	-0.45	-0.28	0.6236	-0.023	0.0149	-0.251	0.1551	0.2176	-0.214	-0.346	0.0456	-0.42	-0.27	-0.31	-0.4	-0.61	0.516	0.711	-0.33	0.128	0.684757	-0.05743	-0.190441994	0.133013515
1455	P06737	PYGL	glycogen phosphorylase L	5836	ENSG00000100504	45	-0.42	0.023	-0.05	0.424	-0.24	-0.26	-0.03	-0.12	-0.01	0.0265	-0.114	0.4603	-0.02	-0.316	-0.053	0.0967	0.2766	-0.268	-0.33	-0.33	0.258	0.031	-0.38	-0.13	0.069	-0.01	-0.09	0.688661	0.026564	-0.085666188	0.112230607
3463	Q9NQ79	CRTAC1	cartilage acidic protein 1	55118	ENSG00000095713	26	-0.55	-0.68	-0.11	0.178	-0.41	0.78	-0.03	-0.17	-0.51	0.538	0.2682	0.6927	-0.421	0.394	-0.465	0.2966	-0.664	-0.453	-0.6	-0.83	0.075	0.128	0.08	-0.02	0.441	0.063	-0.1	0.781586	-0.08182	-0.187044539	0.105222549
114	A0A087X1Z:	PSME2	proteasome activator subunit 2	5721	ENSG00000100911	15	0.122	0.303	0.097	-0.19	0.036	-0.71	0.039	-0.19	0.131	-0.154	0.0317	0.1347	0.1829	-0.118	-0.097	0.018	0.0168	0.028	0.04	-0.92	0.142	-0.39	-0.27	0.03	0.255	0.027	0.114	0.786423	0.067279	-0.045299405	0.112578641
1823	P31689	DNAJA1	DnaJ heat shock protein family (Hsp40) mei	3301	ENSG00000086061	16	-0.28	0.299	-0.04	-0.1	0.026	-0.46	-0.1	-0.16	6E-04	-0.025	0.0275	0.0882	0.0491	-0.073	0.0194	0.0221	0.1322	0.0663	-0.17	-0.26	-0.17	-0.17	-0.31	0.004	-0.17	-0.06	0.074	0.177011	0.048163	-0.123600301	0.171763738
1407	P04066	FUCA1	alpha-L-fucosidase 1	2517	ENSG00000179163	17	-0.11	-0.66	-0.11	-0.15	-0.07	-0.16	-0.31	0.018	0.046	0.2972	-0.194	0.0927	-0.358	0.0783	-0.268	-0.015	-0.356	-0.542	-0.16	0.827	-0.16	0.45	0.061	-0.1	0.31	0.48	-0.42	0.209551	-0.31012	-0.02683392	-0.283282316
2553	Q16531	DDB1	damage specific DNA binding protein 1	1642	ENSG00000167986	41	0.021	0.076	0.002	-0	0.07	-0.4	0.1	0.036	0.258	-0.11	-0.155	-0.013	-0.124	-0.063	-0.212	0.2573	0.0893	-0.041	-0.14	-0.63	-0.23	-0.21	-0.05	-0	0.089	0.135	0.148	0.581379	0.118211	0.05891701	0.059294083
265	Q13510	ASAH1	N-acylsphingosine amidohydrolase 1	427	ENSG00000104763	24	-0.19	-0.21	-0.02	-0.1	-0.7	-0.37	0.057	0.058	0.391	0.5449	-0.129	0.2482	-0.372	0.2218	-0.518	0.4582	-0.148	0.1152	-0.15	-0.17	0.02	-0.37	0.09	-0.06	0.426	0.588	-0.07	0.618551	-0.15561	-0.167575605	0.011968283
2735	Q6UXY8	TMC5	transmembrane channel like 5	79838	ENSG00000103534	29	-0.25	0.359	-0.22	-0.04	-0.18	-0.42	0.106	0.222	-0.1	-0.384	0.0212	0.2732	0.0789	-0.017	-0.058	0.1631	0.0168	0.393	-0.46	0.113	-0.13	0.14	-0.27	-0.19	0.018	-0.03	0.411	0.686866	-0.01303	-0.111734281	0.0987011
3127	Q96EY5	MVB12A	multivesicular body subunit 12A	93343	ENSG00000141971	11	-0.23	0.1	-0.36	0.08	0.007	-0.27	-0.05	0.178	-0.03	-0.413	0.0159	0.3827	-0.294	-0.182	0.0299	0.2198	0.0955	0.4636	-0.23	-0.03	-0.43	-0.26	-0.42	-0.28	-0.39	0.117	0.586	0.535582	0.083196	-0.10027359	0.183469762
1033	O14494	PLPP1	phospholipid phosphatase 1	8611	ENSG00000067113	8	0.367	0.773	-0.01	0.088	0.081	-0.26	-1.08	-0.57	-0.96	-0.123	0.6384	0.3669	0.5742	-0.11	-0.122	-0.336	-0.093	-0.186	-0.49	-0.88	0.058	-0.53	0.124	0.224	-0.75	-0.42	-0.27	0.369821	0.152002	-0.242089045	0.394090644
3297	Q9BWD1	ACAT2	acetyl-CoA acetyltransferase 2	39	ENSG00000120437	21	0.423	0.353	-0.03	-0.03	-0.04	-0.61	-0.19	-0.11	0.053	0.1277	-0.024	0.196	0.0951	-0.252	-0.017	0.0112	0.0635	-0.023	-0.16	-1.04	-0.19	-0.73	-0.38	-0.02	-0.05	-0.05	-0.08	0.168195	0.282374	-0.040014616	0.322389109
1798	P30043	BLVRB	biliverdin reductase B	645	ENSG00000090013	13	0.053	0.235	0.003	7E-04	-0.14	-0.31	-0.07	-0.18	0.265	0.15	0.115	0.1459	0.1266	0.0776	0.0959	0.048	0.077	-0.015	-0.12	-0.71	-0.03	-0.37	-0.2	0.126	-0.11	-0.05	0.239	0.1997	0.120115	-0.107714658	0.227829651
2196	P62330	ARF6	ADP ribosylation factor 6	382	ENSG00000165527	12	-0.44	0.233	-0.05	0.158	-0.14	-0.46	0.096	0.079	0.036	-0.524	-0.352	0.0084	-0.019	-0.095	0.0593	0.2126	0.2012	0.2889	-0.23	-0.32	0.013	-0.54	-0.54	-0.23	-0.22	-0.04	0.347	0.512278	0.14104	-0.030318348	0.171357877
282	Q06HB9	BNC2	basonuclin 2	54796	ENSG00000173068	1	-0.01	-0.99	-0.18	-0.42	-0.19	-0.26	-1.05	-0.5	-0.63	0.3419	0.587	0.5946	-0.338	0.9132	-0.459	-0.988	-0.715	-0.574	-0.15	0.315	0.369	-0.03	0.23	-0.02	0.244	-0.31	-1.2	0.348193	-0.40856	-0.397721235	-0.010836527
2949	Q8NFT2	STEAP2	STEAP2 metalloreductase	261729	ENSG00000157214	18	-0.33	0.306	-0.25	-0.16	-0.26	-0.3	-0.17	-0.08	-0.21	-0.212	-0.127	0.3432	0.3813	-0.071	-0.167	0.2222	0.1638	0.3713	-0.43	-0.23	-0.33	-0.09	0.119	-0.03	-0.54	0.295	0.188	0.205195	-0.0439	-0.261278263	0.217374252
1579	P12931	SRC	SRC proto-oncogene, non-receptor tyrosine	6714	ENSG00000197122	24	0.104	0.185	-0.04	0.105	-0.05	-0.38	0.276	0.109	0.255	0.0664	0.0902	0.053	0.0767	-0.214	0.1326	0.2874	0.356	0.2708	-0.18	-1.13	-0.39	-0.6	-0.3	0.041	-0.05	-0.01	0.461	0.15293	0.302094	-0.062434104	0.364528531
1704	P21281	ATP6V1B2	ATPase H+ transporting V1 subunit B2	526	ENSG00000147416	26	0.066	0.136	-0	-0.05	7E-04	-0.33	-0.06	0.003	0.201	0.0888	0.1476	0.0905	0.0279	-0.057	0.0696	0.4207	0.0072	0.0759	0.02	-0.43	0.008	-0.4	-0.28	0.089	0.248	0.104	0.017	0.319439	0.065077	-0.100636722	0.165713319
237	A0A0J9YW3	STX3	syntaxin 3	6809	ENSG00000166900	15	-0.11	0.561	-0.19	0.203	-0.05	-0.36	-0.39	-0.12	-0.1	-0.217	0.0339	0.0159	0.1175	-0.16	0.502	0.2702	-0.051	0.1123	-0.49	-0.1	-0.53	-0.59	-0.38	-0.15	-0.23	0.343	0.016	0.214497	0.173984	-0.12983725	0.303821091
2760	Q7L1Q6	BZW1	basic leucine zipper and W2 domains 1	9689	ENSG00000082153	25	0.115	0.055	-0.08	-0.02	0.05	-0.42	-0.03	#####	0.069	0.2831	0.2037	0.0784	-0.038	-0.139	0.1957	0.0637	0.2963	0.1222	-0.24	-0.87	-0.36	-0.49	-0.27	0.102	0.018	-0.07	0.343	0.146978	0.174055	-0.148205708	0.322260596
2419	Q13561	DCTN2	dynactin subunit 2	10540	ENSG00000175203	26	-0	0.04	-0.14	-0.2	0.245	-0.64	0.4	-0.06	0.099	-0.117	-0.149	-0.03	0.2593	0.0967	0.0679	0.1385	0.041	0.0451	-0.13	-0.5	-0.14	-0.72	-0.47	0.224	0.167	-0.01	0.063	0.411353	0.140394	-0.067520837	0.207914994
1525	P09960	LTA4H	leukotriene A4 hydrolase	4048	ENSG00000111144	32	0.157	0.154	-0.06	-0.05	0.131	-0.47	0.034	-0.08	0.111	0.2153	0.2426	0.1844	0.0047	-0.199	0.0417	0.1108	0.1615	0.0387	-0.09	-0.91	0.101	-0.35	-0.39	0.182	-0.01	0.047	0.245	0.356743	0.121771	-0.097885199	0.219656087
497	C9J6P4	ZC3HAV1	zinc finger CCCH-type containing, antiviral 1	56829	ENSG00000105939	38	-0.01	0.162	-0.03	-0.03	-0.21	-0.39	-0.09	0.002	0.105	-0.01	-0.02	0.1776	-0.058	-0.16	0.0183	0.0007	0.1153	0.122	-0.32	-0.92	-0.3	-0.59	-0.44	0.026	-0.12	-0.08	0.06	0.102775	0.242113	-0.076054815	0.318167732
1712	P21980	TGM2	transglutaminase 2	7052	ENSG00000198959	25	0.061	-0.17	-0.27	0.177	0.41	-0.34	0.811	-0.44	-0.31	0.2906	-0.049	-0.327	0.3145	-0.134	-1.206	-0.441	-0.259	-0.823	-0.46	-0.93	1.088	0.2	-0.6	0.654	-0.4	-0.8	0.513	0.658071	0.072947	0.285061511	-0.21211457
3351	Q9H0U4	RAB1B	RAB1B, member RAS oncogene family	81876	ENSG00000174903	20	-0.25	0.406	-0.04	-0.14	-0.2	-0.54	0.161	0.052	0.225	0.097	-0.064	-0.19	0.1076	-0.395	0.1648	0.1046	0.2777	0.1866	-0.35	-1.17	-0.39	-0.59	-0.36	0.155	0.043	0.011	0.278	0.312994	0.225504	-0.068548035	0.294052358
3352	Q9H0W9	C11orf54	chromosome 11 open reading frame 54	28970	ENSG00000182919	16	0.214	0.231	0.083	-0.17	-0.44	-0.51	-0	-0.13	-0.11	0.1493	0.4365	-0.069	-0.108	-0.44	0.1682	-0.052	0.5302	-0.119	-0.22	-1.03	-0.18	-0.57	-0.34	-0.07	0.054	0.016	0.325	0.361789	0.129225	-0.149413467	0.278638774
437	B4DUC8	MTAP	methylthioadenosine phosphorylase	4507	ENSG00000099810	14	0.007	0.025	-0.11	0.195	0.1	-0.52	0.29	0.489	0.437	-0.183	0.0308	0.1377	0.0159	-0.06	0.1711	0.5044	0.2425	0.3582	-0.32	-0.95	-0.38	-0.39	-0.42	-0.1	0.363	0.293	0.312	0.255418	0.277663	-0.034438914	0.312101733
1518	P09496	CLTA	clathrin light chain A	1211	ENSG00000122705	15	0.584	-0.24	-0.21	0.469	0.185	-0.51	0.15	0.147	0.248	-0.379	-0.509	-0.358	-0.084	0.058	0.0873	-0.231	0.0713	-0.041	-0.5	-0.93	-0.47	-0.5	-0.3	-0.16	0.041	-0.08	0.036	0.13909	0.40916	0.24532117	0.163838409
2360	Q12765	SCRN1	secernin 1	9805	ENSG00000136193	18	0.219	0.282	-0.11	0.054	-0.03	-0.57	0.154	0.192	0.284	0.0363	0.1294	0.0792	-0.087	-0.058	0.2348	0.2305	0.3638	0.0547	-0.36	-1.21	-0.48	-1.03	-0.54	0.057	0.038	0.19	0.448	0.163581	0.373186	-0.056077316	0.429263117
2120	P56851	EDDM3B	epididymal protein 3B	64184	ENSG00000181552	10	0.282	-1.14	0.995	0.889	0.547	-0.1	-0.14	1.021	-0.15	-0.668	-0.108	0.6908	-3E-04	-0.847	0.6221	-0.447	-0.483	-1.289	-0.59	-0.82	-0.69	0.091	-0.55	-1.37	-0.74	0.576	-1.89	0.149033	0.910105	0.525878746	0.384226677
3448	Q9HD42	CHMP1A	charged multivesicular body protein 1A	5119	ENSG00000131165	11	0.028	0.268	0.015	-0.02	-0.17	-0.47	0.218	0.061	0.033	0.0256	0.058	0.1695	0.0489	-0.012	0.1271	0.0938	0.274	0.3208	-0.12	-0.59	-0.08	-0.89	-0.4	-0.18	-0.16	-0.11	0.292	0.102735	0.245404	-0.12658046	0.37198457
2394	Q13275	SEMA3F	semaphorin 3F	6405	ENSG00000001617	35	-0.27	-0.45	0.103	-0.44	-0.31	-0.34	-0.11	-0.09	0.061	0.1495	0.2524	0.0137	-0.48	0.2827	-0.315	0.0746	-0.369	0.0465	0.113	0.36	0.16	0.007	-0.21	-0.08	0.245	0.049	-0.29	0.234421	-0.24455	-0.166502279	-0.0780469
1874	P36543	ATP6V1E1	ATPase H+ transporting V1 subunit E1	529	ENSG00000131100	20	0.02	0.138	-0.09	-0.23	-0.08	-0.35	-0.08	-0.04	0.132	-0.049	0.0592	0.0197	-0.113	-0.091	0.0713	0.0695	0.1114	0.0233	-0.17	-0.72	-0.11	-0.62	-0.3	0.054	0.003	-0.06	0.122	0.230116	0.135838	-0.074409408	0.210247648
1850	P35052	GPC1	glypican 1	2817	ENSG00000063660	22	-0.16	-0.18	-0.27	0.052	-0.12	-0.05	-0.1	0.082	0.013	0.0919	0.3789	0.1921	-0.283	0.259	0.2715	0.1214	-0.175	0.2525	-0.21	-0.22	0.035	-0.49	-0.52	-0.13	0.18	0.169	0.144	0.176967	0.033961	-0.20551824	0.239479377
2183	P62140	PPP1CB	protein phosphatase 1 catalytic subunit beta	5500	ENSG00000213639	19	0.133	0.143	0.032	0.03	0.055	-0.51	-0.02	-0.06	0.053	0.0581	0.0944	0.2108	-0.028	-0.138	0.0103	-0.158	0.0077	-0.017	-0.31	-0.8	-0.27	-0.39	-0.45	-0.04	0.088	-0.14	0.138	0.159408	0.226162	-0.019248433	0.245410396

2399	Q13332	PTPRS	protein tyrosine phosphatase receptor type S	5802	ENSG00000105426	43	-0.26	-0.08	-0.05	-0.17	-0.1	-0.43	0.153	-0.16	0.079	0.1609	-0.011	0.0595	-0.033	-0.169	-0.172	0.3216	0.2288	0.459	-0.03	0.135	-0.09	-0.28	-0.24	-0.09	0.051	0.175	0.385	0.255502	-0.11411	-0.207144115	0.093036664
1177	O60784	TOM1	target of myb1 membrane trafficking protein	10043	ENSG00000100284	25	-0.12	0.126	0.005	0.129	-0.26	-0.55	0.117	0.106	0.362	-0.075	0.1908	-0.092	-0.029	-0.011	0.14	0.3165	0.3141	0.3851	-0.28	-0.38	-0.12	-0.47	-0.44	-0.06	0.198	0.191	0.254	0.275784	0.113454	-0.136069259	0.249523007
2001	P49720	PSMB3	proteasome subunit beta 3	5691	ENSG00000277791	16	-0.03	-0.23	-0.04	0.069	0.527	-0.62	0.302	0.177	0.258	0.32	-6E-04	0.0788	0.0604	-0.204	-0.566	0.555	0.235	0.3857	-0.45	-0.24	-0.2	0.241	0.082	-0.17	0.665	0.209	0.003	0.917523	0.029883	-0.050899482	0.080781996
1842	P34096	RNASE4	ribonuclease A family member 4	6038	ENSG00000258818	15	0.176	-0.34	-0.2	-0.16	-0.07	-0.63	-0.04	0.017	0.012	0.1438	0.084	-0.054	-0.225	0.1185	-0.381	0.5688	0.0443	0.1968	-0.21	-0.08	0.209	0.366	-0.55	0.015	0.353	0.137	-0.06	0.442614	-0.1571	-0.192065444	0.034962165
935	J3QRU1	YES1	YES proto-oncogene 1, Src family tyrosine k	7525	ENSG00000176105	25	-0.17	0.02	-0.19	-0.01	-0.22	-0.15	-0.01	-0.19	0.079	-0.213	-0.18	0.0892	-0.067	-0.028	-0.007	0.2348	0.2137	-0.015	-0.37	0.551	-0.41	-0.43	-0.24	-0.19	-0.29	0.04	0.122	0.537241	0.041133	-0.097712171	0.138845122
1268	O95025	SEMA3D	semaphorin 3D	223117	ENSG00000153993	32	-0.3	-0.98	0.994	-1	-0.35	-0.88	-0.09	0.237	0.004	0.1921	0.3208	-0.076	-0.903	-0.591	-0.714	0.5317	-0.375	0.9503	-0.09	1.546	0.116	-0.26	-0.47	-0.38	0.296	-0.52	-0.35	0.785148	-0.24965	-0.188790417	-0.06086251
1531	Q6ICJ4	GSTT2B	glutathione S-transferase theta 2B (gene/ps	653689	ENSG00000278695	14	-1.17	0.291	-0.23	0.539	-1.05	-0.71	-0.1	0.109	0.692	0.1761	-1.086	0.033	0.0556	-0.824	0.0852	0.418	0.5198	0.1912	-0.09	-0.18	0.082	-0.53	-0.42	-0.68	0.53	0.074	0.171	0.918662	-0.06258	-0.13187376	0.069291996
2161	P61247	RPS3A	ribosomal protein S3A	6189	ENSG00000145425	23	0.062	-0.09	-0.03	-0.22	0.273	-0.33	0.057	-0.18	0.133	-0.216	-0.167	-0.137	-0.234	-0.105	-0.053	-0.071	0.2539	0.1135	-0.11	-0.29	0.029	-0.19	0.002	-0.01	0.271	-0.27	-0.01	0.943129	0.027469	0.031925643	-0.004456278
2200	P62701	RPS4X	ribosomal protein S4 X-linked	6191	ENSG00000198034	18	-0.06	-0.16	-0.16	-0.21	0.145	-0.42	0.336	0.103	0.35	-0.269	-0.206	-0.187	-0.076	-0.153	-0.048	0.078	0.2901	0.4383	-0.16	-0.18	-0.02	-0.15	-0.14	0.077	0.265	-0.02	0.3	0.996322	-0.00515	0.005836895	-0.010986549
3487	Q9NRV9	HEBP1	heme binding protein 1	50865	ENSG00000013583	13	0.007	0.037	0.003	-0	0.006	-0.22	-0.15	-0.24	0.097	0.2386	0.1597	0.0018	0.1491	-0.161	0.3796	0.0838	0.2708	-0.002	-0.31	-1.17	-0.39	-0.75	-0.28	0.361	-0.17	-0.01	-0.4	0.072634	0.293384	-0.175830564	0.46921486
109	H0Y786	NEB	nebulin	4703	ENSG00000183091	3							0.555	0.663	0.538							0.7496	-0.177	0.3391							0.2	0.153	0.1	0.375346	0.43454	0.281549533	0.152990053
1851	P35080	PFN2	profilin 2	5217	ENSG00000070087	10	0.046	0.092	-0.17	-0	0.039	-0.59	0.158	0.135	0.451	0.013	-0.008	-0.079	0.046	-0.082	0.2732	0.0974	0.3049	0.3724	-0.42	-1.39	-0.44	-0.82	-0.43	0.016	0.077	0.113	0.328	0.165774	0.348094	-0.086724872	0.4348187
1999	P49591	SARS;SAF	seryl-tRNA synthetase;seryl-tRNA synthetas	6301	ENSG00000031698	29	0.032	0.133	-0.02	0.047	0.216	-0.44	0.022	0.014	0.102	0.0341	-0.033	0.0818	0.0121	-0.199	0.125	0.1102	0.2772	0.0647	-0.24	-0.85	-0.12	-0.45	-0.31	-0.01	0.173	0.038	0.169	0.235339	0.189332	-0.040868788	0.230200528
2002	P49721	PSMB2	proteasome subunit beta 2	5690	ENSG00000126067	20	-0.07	-0.1	-0.01	-0.01	0.299	-0.53	-0.21	-0.32	-0.17	0.292	-0.048	0.0593	0.042	-0.145	-0.328	0.3474	0.1463	-0.089	-0.3	-0.04	-0.03	0.069	-0.06	-0.11	0.525	0.092	-0.45	0.542884	-0.09215	-0.156137423	0.063986431
3676	Q9UNM6	PSMD13	proteasome 26S subunit, non-ATPase 13	5719	ENSG00000185627	19	0.064	-0	-0.11	-0.02	0.317	-0.39	-0.31	-0	-0.03	0.1322	-0.007	-0.043	0.0492	-0.189	0.0164	-0.228	-0.014	-0.184	0.019	-0.21	-0.03	-0.27	-0.26	0.009	-0.03	-0.01	-0.45	0.61498	0.084137	-0.001199977	0.085337225
1232	O75882	ATRN	attractin	8455	ENSG00000088812	44	-0.35	-0.14	-0.03	-0.16	-0.1	-0.27	-0.17	0.028	-0.31	0.3208	0.1567	0.3648	-0.045	-0.127	-0.566	0.1479	0.0195	0.0196	-0.29	0.187	-0.16	-0.47	0.013	-0.26	0.224	0.563	-0.14	0.41237	-0.13224	-0.199983344	0.06774006
2034	P51148	RAB5C	RAB5C, member RAS oncogene family	5878	ENSG00000108774	12	0.211	0.232	0.146	0.056	-0.02	-0.19	-0.31	-0.41	-0.09	0.2813	0.3776	0.0312	0.2776	-0.128	0.1386	-0.138	0.1497	-0.098	-0.02	-0.48	-0.07	-0.43	-0.29	0.116	-0.37	-0.22	-0.13	0.117378	0.167793	-0.14123291	0.309026037
1138	O43776	NARSRS1	asparaginyl-tRNA synthetase;asparaginyl-tF	4677	ENSG00000134440	30	0.065	0.265	0.027	-0.07	0.046	-0.58	-0.05	0.1	0.085	0.1348	0.0207	-0.015	0.1355	-0.281	0.1025	0.11	0.1469	0.1076	-0.23	-0.83	-0.26	-0.43	-0.34	0.116	0.193	0.193	0.137	0.367218	0.14995	-0.063443772	0.213393497
1924	P42702	LIFR	LIF receptor alpha;LIF receptor subunit alph	3977	ENSG00000113594	30	0.008	0.125	-0.27	-0.14	-0.05	-0.28	-0.07	0.017	0.094	0.0468	0.1075	0.0663	-0.058	-0.102	-0.131	0.5276	0.0316	0.5362	-0.44	-0.51	-0.01	-0.45	0.02	-0.29	0.268	0.197	-0.02	0.226877	0.073343	-0.177705916	0.251049038
1509	P09104	ENO2	enolase 2	2026	ENSG00000111674	6	-0.53	-0.08	0.733	-0.82	0.056	-0.48	-0.07	0.051	0.108	0.5398	0.1684	0.0012	-0.323	0.0576	-0.798	-0.15	0.1553	-0.002	-0.47	0.872	-0.27	0.176	-0.12	-0.16	0.518	0.55	-0.76	0.849553	-0.15349	-0.075896903	-0.077593468
2461	Q14507	EDDM3A	epididymal protein 3A	10876	ENSG00000181562	10	0.282	-0.77	-0.45	-0.21	-0.23	-0.37	0.358	0.093	0.33	0.2869	-0.622	0.3678	-0.13	0.1798	0.1462	0.3794	-1.123	-0.198	0.261	0.357	-0.3	-0.07	0.145	-0.34	0.234	0.33	-0.36	0.836636	-0.13644	-0.028756283	-0.10768378
2080	P53801	PTTG1IP	PTTG1 interacting protein	754	ENSG00000183255	7	-0.28	0.343	0.133	-0.15	0.205	-0.36	0.036	-0.08	0.089	-0.159	-0.043	0.0358	0.4926	-0.35	-0.021	0.3162	0.3659	0.3309	-0.51	0.077	-0.41	-0.45	0.01	0.143	-0.08	0.131	0.103	0.38881	0.103481	-0.114350612	0.217831412
1427	P04899	GNAI2	G protein subunit alpha i2	2771	ENSG00000114353	25	0.157	0.469	-0.25	0.397	0.107	-0.35	-0.56	-0.29	-0.19	-0.175	0.0838	0.2988	0.204	-0.336	0.4579	0.2125	-0.019	-0.04	-0.4	-0.19	-0.55	-0.41	-0.64	-0.4	-0.36	0.038	0.162	0.147602	0.248102	-0.133305488	0.381407426
2250	P68402	PAFAH1B	platelet activating factor acetylhydrolase 1b c	5049	ENSG00000168092	7	0.155	0.224	-0.01	-0.01	-0.02	-0.55	0.102	-0.04	0.115	0.1779	-0.082	0.023	-0.021	-0.128	0.1867	0.1007	0.2933	0.1034	-0.27	-1.47	-0.37	-0.71	-0.48	-0.01	-0.1	-0	0.324	0.146941	0.340179	-0.076226776	0.416406088
1175	O60749	SNX2	sorting nexin 2	6643	ENSG00000205302	20	0.105	0.125	-0.08	-0.08	-0.01	-0.42	0.172	0.058	0.357	0.0043	-0.123	0.0868	-0.045	-0.032	0.0932	0.0584	0.1194	0.087	-0.14	-0.72	-0.29	-0.52	-0.27	-0.05	0.076	1E-03	0.269	0.226532	0.205874	-0.003840013	0.209714365
1754	P26038	MSN	moesin	4478	ENSG00000147065	52	-0.19	0.05	-0.3	0.272	0.095	-0.3	-0.17	-0.02	0.067	-0.466	0.0474	0.1411	0.0076	-0.203	0.1212	0.0402	-0.122	0.2892	-0.45	-0.04	-0.04	0.144	-0.49	-0.12	-0.09	-0.05	0.135	0.75392	0.054334	-0.040070774	0.09440434
2114	P55327	TPD52	tumor protein D52	7163	ENSG00000076554	15	0.072	0.263	-0.08	-0.14	-0.08	-0.71	0.034	-0.1	0.319	-0.161	-0.066	-0.008	-0.146	-0.144	0.2588	0.0808	0.2307	0.2893	-0.18	-0.79	-0.29	-0.99	-0.68	-0.2	-0.16	0.036	0.418	0.205568	0.269504	-0.084100014	0.353603782
3424	Q9HAB8	PPCS	phosphopantothenoylcysteine synthetase	79717	ENSG00000127125	16	0.432	0.387	0.023	-0.03	-0.03	-0.31	0.051	-0.02	0.11	0.259	0.3634	0.197	0.0172	-0.011	0.085	-0.007	0.1366	-0.049	-0.12	-1.09	0.136	-0.2	-0.26	-0.01	-0.26	-0.01	0.227	0.205213	0.243238	-0.041272461	0.284509998
3202	Q99460	PSMD1	proteasome 26S subunit, non-ATPase 1	5707	ENSG00000173692	32	-0.04	-0.03	-0.09	0.023	0.438	-0.32	0.026	-0.05	0.142	-0.048	-0.132	-0.08	-0.104	0.0256	-0.005	-0.048	0.0964	0.056	-0.14	-0.55	-0.12	-0.24	-0.2	-0.01	0.176	-0.04	-0.02	0.377035	0.139257	0.037481905	0.101775483
1781	P28482	MAPK1	mitogen-activated protein kinase 1	5594	ENSG00000100030	18	0.038	0.158	-0.14	-0.01	-0.16	-0.47	0.012	-0.11	0.297	0.0496	0.0168	-0.03	0.0626	-0.184	0.0588	0.1913	0.2671	0.232	-0.27	-1	-0.3	-0.56	-0.56	-0	0.04	0.093	0.33	0.202277	0.205602	-0.115763043	0.321364818
1013	O00469	PLOD2	procollagen-lysine,2-oxoglutarate 5-dioxyger	5352	ENSG00000152952	23	-0.1	-0.63	-0.35	-0.53	-0.18	-0.14	0.177	0.11	-0.07	0.1804	0.2191	-0.173	-0.377	0.2972	-0.12	0.2109	-0.382	-0.017	-0.06	0.405	0.014	-0.2	-0.14	-0.25	0.701	-0.13	-0.05	0.390445	-0.22368	-0.172966929	-0.050714622
350	A0AVT1	UBA6	ubiquitin like modifier activating enzyme 6	55236	ENSG00000033178	32	0.042	0.155	-0.02	-0.06	-0.04	-0.29	-0.11	-0.02	0.01	0.0778	-0.014	0.0129	-0.08	0.0236	-0.127	0.0321	0.2535	0.0214	-0.24	-0.71	-0.21	-0.37	-0.3	-0.03	-0.07	-0.01	0.205	0.173179	0.154466	-0.058986561	0.213452226
1821	P31323	PRKAR2B	protein kinase cAMP-dependent type II regu	5577	ENSG00000284096	21	0.168	0.46	-0.3	-0.05	-0.14	-0.23	-0.02	0.144	-0.01	-0.152	0.0215	-0.099	-0.082	-0.239	0.0487	0.264	-0.142	0.1018	-0.15	-0.46	-0.05	0.231	-0.38	0.057	0.039	-0.08	-0.01	0.737377	0.092339	0.03334391	0.058995534
3732	Q9Y365	STARD10	StAR related lipid transfer domain containin	10809	ENSG00000214530	16	-0.16	-0.09	-0.21	-0.06	-0.2	-0.48	0.086	0.093	0.26	-0.188	-0.264	-0.037	0.0216	-0.087	-0.003	0.1899	0.2629	0.5782	-0.36	-0.72	-0.31	-0.54	-0.43	0.139	0.019	-0.04	0.416	0.338572	0.119252	-0.136273429	0.255525305
2014	P50135	HNMT	histamine N-methyltransferase	3176	ENSG00000150540	14	-0.12	0.108	-0.11	-0.36	-0.11	-0.47	-0.28	-0.12	0.224	0.2319	0.1875	0.024	-0.067	-0.234	0.0644	0.0636	0.3073	0.146	-0.31	-0.73	-0.19	-0.83	-0.44	-0.13	-0.19	0.213	0.479	0.208813	0.099722	-0.217851498	0.317573152
3617	Q9UHI8	ADAMTS1	ADAM metallopeptidase with thrombospond	9510	ENSG00000154734	26	-0.51	-0.57	-0.55	-0.24	-0.64	-0.44	0.162	0.073	-0.23	0.9618	-0.259	0.1719	-0.712	-0.273	-0.015	0.9858	0.1874	0.0989	-0.08	0.359	0.203	0.526	-0.23	0.01	0.143	0.093	-0.43	0.173044	-0.39296	-0.454831581	0.061874898
3654	Q9ULC4	MCTS1	MCTS1 re-initiation and release factor;MCT	28985	ENSG00000232119	13	0.006	0.145	-0.02	-0.05	-0.05	-0.35	0.226	0.137	0.404	-0.014	-0.182	0.0434	-0.063	-0.082	0.0614	0.2284	0.31	0.3004	-0.27	-0.69	-0.17	-0.39	-0.3	0.011	0.048	0.068	0.373	0.283921	0.195053	-0.017428225	0.21248123
1451	P06702	S100A9	S100 calcium binding protein A9	6280	ENSG00000163220	9	-2.07	-1.87	-0.98	-0.82	-1.54	2.106	0.329	-1.98	-1.64	-1.867	-0.145	-1.587	0.9205	-1.152	-1.287	-1.036	-2.018	-2.145	-1.89	-2.09	1.598	2.286	0.392	0.454	-1.97	-1.97	-2.02	0.784131	-0.36353	0.204427257	-0.567959595
2480	Q14847	LASP1	LIM and SH3 protein 1	3927	ENSG00000002834	12	0.052	0.198	0.032	-0.13	-0.01	-0.33	-0.08	-0.34	-0.02	-0.092	0.2449	0.0677	0.0123	-0.128	0.1976	-0.251	0.1043	-0.258	-0.25	-0.59	-0.09	-0.46	-0.3	-0.02	-0.05	-0.22	0.063	0.230406	0.14329	-0.056835927	0.200125636
2207	P62826	RAN	RAN, member RAS oncogene family	5901	ENSG00000132341	12	0.045	0.116	-0.02	0.027	0.154	-0.32	0.165	0.168	0.151	-0.022	-0.071	0.1413	-0.007	-0.12	0.1368	0.0244	0.1662	0.0275	-0.23	-0.64	-0.18	-0.29	-0.4	0.04	0.229	-0.09	0.199	0.203102	0.205901	0.023474096	0.182427278
3782	Q9Y617	PSAT1	phosphoserine aminotransferase 1	29968	ENSG00000135069	17	0.291	0.33	-0.3	0.22	0.217	-0.23	-0.16	0.011	0.227	-0.028	-0.136	0.706	0.0608	-0.218	0.0623	0.1599	-0.021	0.1369	-0.07	-1.01	-0.16	-0.58	-0.28	-0.03	-0.09	0.004	0.264	0.211908	0.284835	-0.01217859	0.297013371
3585	Q9UBQ7	GRHPR	glyoxylate and hydroxypyruvate reductase	9380	ENSG00000137106	16	0.139	0.203	-0.12	-0.03	0.029	-0.41	-0.03	-0.08	0.168	0.014	0.029	0.1394	0.1078	-0.115	0.135	0.0881	0.062	0.1313	-0.22	-0.95	-0.26	-0.68	-0.48	-0.05	-0.03	0.019	0.239	0.129828	0.250613	-0.08180187	0.332414676
1279	O95336	PGLS;AC	6-phosphogluconolactonase	25796	ENSG00000130313	13	0.025	0.279	-0.06	-0.03	0.153	-0.28	0.069	-0.07	0.213	0.1659	0.132	0.3103	0.0738	-0.07	0.159	0.1368	0.2373	0.0186	-0.15	-0.24	-0.07	-0.38	-0.26	0.117	-0.13	0.04	0.319	0.172794	0.115936	-0.097039019	0.212974635
446	B4E1Z4	AL645922.	novel complement component 2 (C2) and cc	NA	ENSG00000244255	4	-0.86	-0.88	-0.39	-0.55	-0.67	-0.22	-0.61	-0.64	-0.81	-0.762	-0.205	-0.255	-0.515	-0.333	-0.751	-0.361	-0.696	-0.497	-0.75	-0.3	1.939	2.028	-0.11	0.459	-0.2	-0.59	-0.81	0.147162	-0.80812	-0.138419782	-0.669703051
1135	O43747	AP1G1	adaptor related protein complex 1 subunit g	164	ENSG00000166747	22	0.14	0.14	-0.04	-0.03	-0.09	-0.45	-0.14	-0.1	0.098	0.1642	0.0145	0.101	0.0376	-0.136	-0.173	-0.051	0.0483	0.1959	-0.18	-0.69	-0.18	-0.38	-0.37	0.041	0.042	-0.06	0.027	0.205321	0.141147	-0.074733191	0.215879733
922	J3KR44	OTUB1	OTU deubiquitinase, ubiquitin aldehyde binc	55611	ENSG00000167770	18	0.105	0.056	-0.09	-0.15	0.055	-0.49	0.11	0.062	0.226	-0.112	0.0937	-0.008	0.0744	-0.143	0.0926	0.1069	0.2657	0.276	-0.2	-1.04	-0.3	-0.51	-0.22	0.129	0.028	0.08	0.371	0.315893	0.172373	-0.084072812	0.256446118
1481	P07858	CTSB	cathepsin B	1508	ENSG00000164733	15	-0.78	-0.84	-0.17	-1.02	-0.17	-0.1	-0.15	-0.45	-0.02	0.5422	0.1548	-0.066	-0.603	0.0459	-0.711	-0.269	-0.229	-0.214	-0.17	0.715	0.022	-0.23	0.242	-0.29	0.651	0.091	-0.79	0.23691	-0.43511	-0.259498534	-0.175607965
3465	Q9NQE9	HINT3	histidine triad nucleotide binding protein 3	135114	ENSG00000111911	10	0.157	0.212	0.063	-0.01	-0.13	-0.43	-0.08	-0.06	0.063	0.1389	0.1366	-0.018	0.2263	-0.372	0.1993	0.1587	0.2255	0.1213	-0.09	-1.12	-0.08	-0.8	-0.48	0.144	-0.16	-0.01	0.263	0.197534	0.235067	-0.114800307	0.349866846
1433	P05154	SERPINA5	serpin family A member 5	5104	ENSG00000188488	17	-0.37	-1.11	-0.2	0.038	-1.09	-0.39	-0.33	-0.21	-0.15	0.2238	0.7194	-0.331	-0.357	0.5265	-0.018	0.9447	-0.956	-0.9	-0.25	0.708	-0.4	0.255	0.086	-0.26	0.671	0.352	-0.43	0.261215	-0.50445	-0.406871021	-0.097579205
1967	P48147	PREP	prolyl endopeptidase	5550	ENSG00000085377	32	0.153	0.154	0.02	0.144	-0.01	-0.36	-0.01	0.018	0.324	0.0934	0.0096	0.1629	0.1534	-0.189	-0.027	0.1312	0.2041	0.178	-0.16	-0.48	-0.04	-0.31	-0.32	0.047	-0	0.166	0.302	0.328495	0.136994	-0.03128497	0.168278472
2878	Q8IZP9	ADGRG2	adhesion G protein-coupled receptor G2	10149	ENSG00000173698	18	-0.2	-0.45	1.672	0.948	0.616	-0.05	-0.2	0.512	-0.5	-0.795	-0.223	0.1921	0.0739	-0.715	-0.171	-0.66	-0.398	0.3818	-0.96	-0.41	-0.52	-0.51	-0.65	-0.86	-0.84	-0.31	-0.92	0.051259	0.92577	0.518016054	0.407753998
2144	P61006	RAB8A	RAB8A, member RAS oncogene family	4218	ENSG00000167461	17	-0.16	0.106	-0.23	0.133	-0.1	-0.41	0.046	-0.19	0.054	-0.024	0.0201	-0.074	0.104	-0.383	0.0656	0.0702	0.0224	-0.089	-0.35	-0.48	-0.28	-0.73	-0.54	0.007	-0.23	-0.09	0.158	0.170982	0.197895	-0.051873479	0.249768632
2755	Q709C8	VPS13C	vacuolar protein sorting 13 homolog C	54832	ENSG00000129003	64	-0.16	-0.1	-0.16	-0.17	-0.05	-0.41	-0.08	-0.09	0.063	0.0286	0.1327	-0.086	-0.074	-0.137	-0.033	0.0394	0.2344	0.1718	-0.18	-0.36	-0.05	-0.49	-0.24	-0.03	-0.26	0.023	0.126	0.145698	0.034855	-0.158761274	0.193616189
1856	P35270	SPR	sepiapterin reductase	6697	ENSG00000116096	13	-0.15	-0.1	-0.14	-0.05	0.018	-0.44	0.319	0.062	0.408	-0.164	-0.219	-0.161	0.0823	0.0144	-0.062	0.256	0.3686	0.2703	-0.35	-0.44	-0.53	-0.41	-0.43	0.156	-0.04	0.006	0.456	0.43568	0.135985	-0.081653259	0.217638619
2436	Q13938	CAPS	calcyphosine	828	ENSG00000105519	15	0.058	-0.21	-0.19	0.126	-0.12	-0.11	0.552	-0.07	0.272	-0.107	0.1324	-0.231	-0.366	-0.094	0.4471	0.4203	0.3308	-0.033	-0.06	-1.14	-0.33	-0.77	-0.35	0.368	0.067	-0.06	0.09	0.303474	0.278051	-0.020565648	0.29861695
2915	Q8N9N7	LRRC57	leucine rich repeat containing 57	255252	ENSG00000180979	17	0.044	0.155	-0.05	0.113	-0.14	-0.38	0.03	-0.16	0.161	0.3377	0.2133	0.0775	0.0863	-0.378	0.1637	0.1863	0.2933	0.1702	-0.34	-0.59	-0.24	-0.53	-0.42	0.019	-0.24	-0.1	0.354	0.100947	0.207469	-0.153466121	0.360935293
3771	Q9Y5P6	GMPPB	GDP-mannose pyrophosphorylase B	29925	ENSG00000173540	15	0.144	0.205	0.009	0.048	0.014	-0.35	-0.03	-0.23	0.073	0.2131	0.0303	0.0107	0.1884	0.1665	0.0545	0.0645	-0.011	0.0804	-0.31	-0.61	0.006	-0.18	-0.27	0.045	0.16	0.127	0.044	0.226741	0.098263	-0.100987212	0.199250507
155	A0A0A0MSE	PLEKHB2	pleckstrin homology domain containing B2	55041	ENSG00000115762	10	-0.32	0.068	-0.05	-0	-0.19	-0.51	-0	0.148	-0.06	-0.074	0.1278	0.0957	0.1231	-0.05	-0.224	0.5102	0.3766	0.5477	-0.45	-0.12	-0.34	-0.53	-0.4	-0.06	-0.18	0.117	0.321	0.129429	0.078265	-0.261679517	0.339944823
2899	Q8N3R9	MPP5	membrane palmitoylated protein 5	64398	ENSG00000072415	34	-0.23	-0.03	-0.19	0.231	0.027	0.004	-0.33	-0.25	-0.13	-0.13	0.1535	0.258	0.2043	-0.035	0.3167	0.1851	-0.014	0.1127	-0.36	-0.06	-0.18	-0.18	-0.01	-0.1	-0.12	-0.14	0.07	0.065896	0.019659	-0.216410635	0.236069368
2741	Q6XQN6	NAPRT	nicotinate phosphoribosyltransferase	93100	ENSG00000147813	19	-0.12	-0.98	0.627	-0.09	0.275	-1.08	-0.73	-0.54	-0.04	-0.319	-0.089	-0.039	0.0111	-0.808	0.0512	-0.169	0.163	0.1753	-0.27	-1.06	0.147	-0.37	0.156	0.16	0.198	0.085	0.196	0.672045	-0.21531	-0.185314901	-0.029991954
2773	Q7LBR1	CHMP1B	charged multivesicular body protein 1B	57132	ENSG00000255112	8	-0.02	0.233	-0.2	0.142	0.138	-0.55	-0.02	-0.07	-0.17	-0.111	0.1095	0.14	0.0977	0.0508	0.115	0.054	0.1126	0.1596	-0.22	-0.21	-0.29	-0.8	-0.54	-0.28	-0.23	-0.2	0.384	0.116422	0.2072	-0.137964216	0.345163875
3141	Q96HC4	PDLIM5	PDZ and LIM domain 5	10611	ENSG00000163110	28	0.14	0.423	0.026	-0.26	0.02	-0.57	0.018	-0.11	0.188	-0.053	-0.075	-0.043	-0.039	-0.068	0.1225	0.2129	0.0586	0.0724	-0.12	-0.65	-0.15	-0.64	-0.47	-0.03	-0.01	-0.04	0.216	0.264303	0.197411	-0.03444364	0.231854827
2050	P51888	PRELP	proline and arginine rich end leucine rich rep	5549	ENSG00000188783	18	-1.35	-1.52	-1.41	-1.36	-1.48	0.412	0.127	0.895	-0.96	-1.107	-0.64	-1.155	-1.287	0.7088	-1.282	-0.89	-0.881	-1.164	1.611	-0.71	2.575	-1.17	-1.05	0.715	-0.97	0.421	0.606	0.192799	-0.96507	0.116087563	-1.081153982
3742	Q9Y3E7	RNF103-C	RNF103-CHMP3 readthrough;charged mult	100526767;	ENSG00000249884	11	0.017	0.229	0.022	-0.02	-0.15	-0.32	0.279	0.264	0.313	0.0433	0.0265	0.0816	-0.02	0.2199	0.1838	0.2692	0.1748	0.2047	-0.15	-0.49	-0.17	-0.75	-0.34	-0.06	0.176	-0.03	0.407	0.172904	0.226155	-0.06107815	0.287233324
624	Q14203	DCTN1	dynactin subunit 1	1639	ENSG00000204843	44	0.101	0.205	-0.06	0.05	0.151	-0.2	-0.01	0.108	0.217	-0.079	0.0004	0.0476	-0.058	-0.025	0.2175	0.2949	0.0613	0.0401	-0.23	-0.75	-0.19	-0.66	-0.34	-0.09	0.208	0.123	0.176	0.152554	0.258713	0.007171694	0.251541462
1267	O94985	CLSTN1	calsyntenin 1	22883	ENSG00000171603	27	-0.05	-0.55	-0.32	0.077	-0.19	0.111	0.061	0.426	0.197	0.4307	0.1818	0.5413	-0.114	0.2818	-0.036	0.4159	-0.291	0.0206	-0.27	0.063	-0.26	-0.23	-0.24	-0.38	0.384	0.074	0.045	0.306503	0.063528	-0.186727933	0.250256313
1800	P30046	DDT;DDTL	D-dopachrome tautomerase;D-dopachrome	1652;10003	ENSG00000099977	9	-0.24	0.516	-0.07	0.155	0.009	-0.63	-0.06	-0.18	0.263	0.1099	0.2683	-0.059	0.1799	-0.302	0.0368	0.1916	0.4336	-0.015	-0.42	-0.94	-0.3	-0.92	-0.37	-0.07	-0.17	-0.11	0.129	0.11821	0.32562	-0.120175796	0.445795612
3517	Q9NV96	TMEM30A	transmembrane protein 30A	55754	ENSG00000112697	14	-0.21	0.138	0.039	-0.1	-0.34	-0.51	0.461	0.099	0.466	0.1483	0.1235	0.0674	-0.038	-0.31	0.0469	0.1451	0.2863	0.455	-0.38	-0.57	-0.39	-0.72	-0.45	-0.12	-0.01	0.079	0.282	0.172855	0.259403	-0.097613339	0.357016234
995	O00204	SULT2B1	sulfotransferase family 2B member 1	6820	ENSG00000088002	15	-0.02	0.297	-0.1	0.004	-0.11	-0.7	-0.05	-0.31	-0.04	0.1661	0.2917	0.1279	0.2309	-0.011	0.0996	-0.03	0.2355	0.0463	-0.41	-1.33	-0.41	-0.54	-0.53	0.146	-0.03	0.029	0.189	0.132029	0.208145	-0.241511341	0.449656404
69	A0A087WY	VTA1	vesicle trafficking 1	51534	ENSG00000009844	14	-0.04	0.348	-0.2	0.24	0.095	-0.47	0.004	-0.11	-0.04	-0.008	-0.022	0.2406	0.2199	-0.11	-0.08	-0.032	0.0591	0.0949	-0.2	-0.47	-0.29	-0.69	-0.42	-0.1	-0.08	-0.12	0.119	0.121536	0.230274	-0.060062021	0.290336373
1824	P31749	AKT1	AKT serine/threonine kinase 1	207	ENSG00000142208	22	-0.03	0.193	-0.08	-0.07	-0.04	-0.24	-0.3	-0.26	-0.14	0.1376	0.0337	0.0944	0.0148	-0.036	0.2929	-0.277	-0.3	-0.388	0.028	-0.81	-0.22	-0.31	-0.22	0.167	0.307	-0.26	-0.09	0.752221	0.048713	-0.059494225	0.108206952
1395	P02774	GC	GC vitamin D binding protein;GC, vitamin D	2638	ENSG00000145321	26	-0.71	-0.63	-0.25	-0.71	-0.16	0.042	0.157	0.222	-0.39	-0.2	0.0552	-0.138	-0.666	0.0738	-0.733	-0.143	0.0822	0.1033	-0.51	-0.39	1.242	1.172	-0.11	0.165	0.203	0.435	-1.03	0.405312	-0.40007	-0.095533574	-0.304540479
3556	Q9NZZ3	CHMP5	charged multivesicular body protein 5	51510	ENSG00000086065	10	-0.09	0.122	-0.24	0.121	0.059	-0.38	0.261	0.069	0.064	-0.44	0.0256	0.0564	0.0061	-0.092	0.1281	0.2797	0.3031	0.3378	-0.27	-0.59	-0.32	-0.95	-0.5	-0.2	-0.08	-0.01	0.576	0.211507	0.256917	-0.069173663	0.326090844
2121	P57735	RAB25	RAB25, member RAS oncogene family	57111	ENSG00000132698	13	0.006	0.507	0.083	-0	0.055	-0.62	0.308	0.066	0.188	0.1473	0.0979	0.1082	0.3415	-0.178	-0.135	-0.098	0.0779	-0.013	-0.17	-0.84	-0.36	-0.52	-0.37	0.303	-0.1	0.086	0.099	0.246018	0.271611	0.026580326	0.245031147
2337	Q07960	ARHGAP1	Rho GTPase activating protein 1	392	ENSG00000175220	18	0.197	0.2	-0.42	0.037	-0.24	-0.25	-0.1	-0.36	0.05	-0.029	0.2204	-0.084	-0.069	-0.098	0.0026	0.4953	0.1735	0.3898	-0.29	-0.74	-0.27	-0.71	-0.48	0.069	0.263	0.006	0.229	0.204536	0.114404	-0.210193836	0.324598132
286	A0A1W2PN	AC004922	novel protein, ARPC1A and ARPC1B readtl	10552	ENSG00000284292	22	0.132	0.187	0.02	-0.15	-0.09	-0.38	-0.12	-0.1	-0.03	0.2031	0.0574	-0.023	0.0269	-0.073	-0.042	0.0187	0.1604	0.0837	-0.1	-0.83	-0.05	0.041	-0.34	0.09	-0.05	-0.07	-0.01	0.285117	0.077351	-0.105551252	0.192902435
2350	Q09666	AHNAK	AHNAK nucleoprotein	79026	ENSG00000124942	52	0.197	0.142	0.157	-0.2	0.179	-0.34	-0.12	-0.14	0.127	0.1705	0.1476	0.0482	0.0554	0.091	0.1268	-0.119	0.1864	0.1191	-0.06	-0.17	-0.18	-0.29	-0.11	-0.07	0.062	0.003	0.158	0.221258	0.07299	-0.091727473	0.164717676
1075	O15144	ARPC2	actin related protein 2/3 complex subunit 2	10109	ENSG00000163466	17	0.138	0.177	-0.14	0.043	-0	-0.5	-0.13	-0.28	-0.17	0.0804	0.0452	0.0314	0.0016	-0.195	0.0726	-0.114	0.0651	-0.054	-0.2	-0.81	-0.03	-0.07	-0.46	0.002	-0.13	-0.07	-0.08	0.291097	0.109065	-0.088290487	0.197355855
3592	Q9UBX1	CTSF	cathepsin F	8722	ENSG00000174080	18	-0.35	-0.63	-0.05	-0.27	-0.04	-0.17	-0.28	0.23	0.174	0.2381	0.1326	0.0098	-0.326	0.2938	-0.169	0.0515	-0.413	-0.445	0.228	0.689	0.381	-0.43	0.047	-0	0.373	0.144	-0.31	0.309478	-0.27651	-0.082847924	-0.193660075

2511	Q15286	RAB35	RAB35, member RAS oncogene family	11021	ENSG00000111737	16	-0.21	0.29	-0.08	0.123	-0.31	-0.36	-0.24	-0.3	-0.07	0.0247	-0.067	-0.053	0.0077	-0.242	0.1023	0.0933	0.2231	0.0856	-0.39	-0.7	-0.45	-0.61	-0.55	-0.13	-0.35	-0.15	0.179	0.073617	0.221508	-0.149109158	0.370617388
2518	Q15382	RHEB	Ras homolog, mTORC1 binding	6009	ENSG00000106615	11	0.186	0.283	0.109	0.049	-0.09	-0.34	0.112	-0.03	0.048	0.4019	0.329	0.1471	0.1019	-0.234	0.2036	-0.031	0.2949	0.0237	-0.33	-0.97	-0.28	-0.61	-0.37	0.105	-0.26	-0.12	0.23	0.070454	0.326902	-0.100540845	0.427442448
514	P05090	APOD	apolipoprotein D	347	ENSG00000189058	9	-0.21	-0.04	-0.1	-0.31	-0.14	-0.57	-0.47	-0.12	0.334	-0.12	0.3286	0.0446	-0.46	0.2033	-0.618	0.2881	-0.557	-0.003	-0.56	-0.66	-0.18	-0.69	-0.34	1.524	0.231	0.438	-0.41	0.923299	-0.10807	-0.081128363	-0.026946524
1695	P20618	PSMB1	proteasome subunit beta 1	5689	ENSG00000008018	15	-0.11	-0.09	-0.01	-0.15	0.405	-0.35	-0.04	-0.07	0.017	0.1573	-0.184	-0.034	-0.023	-0.13	-0.323	0.4166	0.1321	0.1209	-0.22	0.115	-0.1	0.179	0.044	-0.15	0.48	0.238	-0.14	0.75751	-0.09363	-0.058813556	-0.034814342
1344	P01024	C3	complement C3	718	ENSG00000125730	76	-0.95	-0.97	0.075	-0.55	-0.64	-0.11	-0.46	-0.23	-0.61	-0.739	0.0233	-0.637	-0.425	-0.39	-0.626	-0.436	-0.813	-0.632	-0.72	-0.69	1.865	1.617	0.366	0.53	-0.13	-0.42	-1.14	0.205475	-0.63645	0.025656668	-0.662108529
2745	Q6ZMP0	THSD4	thrombospondin type 1 domain containing 4	79875	ENSG00000187720	21	-0.85	-0.52	0.353	-0.34	-0.46	-0.49	0.349	-0.01	0.545	0.1254	-0.021	-0.041	-0.585	0.0958	-0.286	0.2919	0.1046	0.0467	-0.5	0.169	0.169	0.14	-0.34	0.168	0.651	-0.07	0.049	0.633188	-0.20599	-0.12680753	-0.079180995
1143	O43865	AHCYL1	adenosylhomocysteinase like 1	10768	ENSG00000168710	23	0.21	0.17	-0.06	-0.22	-0.07	-0.6	-0.1	-0.08	0.29	0.1661	0.0796	0.1443	-0.062	-0.198	-0.063	0.1286	0.1068	0.1854	-0.14	-0.77	-0.1	-0.73	-0.49	0.021	0.121	-0.04	0.073	0.228248	0.175967	-0.104665795	0.280632448
3132	Q96G03	PGM2	phosphoglucomutase 2	55276	ENSG00000169299	21	0.095	0.02	0.078	0.065	0.032	-0.33	0.09	-0.02	0.089	0.0302	-0.033	0.2196	-0.013	-0.184	-0.04	-0.123	-0.035	-0.179	-0.33	-0.9	0.051	-0.43	-0.2	0.01	-0.02	-0.07	-0.01	0.209621	0.22362	0.053281419	0.170338859
3549	Q9NZJ9	NUDT4	nudix hydrolase 4	11163	ENSG00000173598	13	-0.15	0.37	-0.02	-0.18	-0.04	-0.52	0.218	0.066	0.151	0.2159	0.1327	0.3515	0.0635	-0.277	0.2344	-0.084	0.2083	-0.069	-0.35	-1.24	-0.18	-0.42	-0.3	0.205	0.022	0.101	0.184	0.289291	0.207581	-0.099107822	0.30668891
2549	Q16222	UAP1	UDP-N-acetylglucosamine pyrophosphoryla	6675	ENSG00000117143	25	0.122	0.432	0.246	-0.15	-0.08	-0.2	0.053	0.12	0.254	0.1635	0.0093	0.0136	0.0797	0.0831	-0.138	0.0037	0.19	0.2761	-0.32	-0.81	-0.09	-0.61	-0.2	0.062	-0.18	-0.14	0.094	0.081757	0.331386	0.011995883	0.319390297
1048	O14773	TPP1	tripeptidyl peptidase 1	1200	ENSG00000166340	11	-0.14	-0.62	-0.34	-0.23	-0.2	-0.24	0.061	0.007	0.218	0.0539	0.3677	0.2226	-0.254	0.562	-0.042	0.3675	-0.463	-0.079	-0.25	-0.02	-0.17	0.082	-0.22	-0.05	0.734	0.023	0.036	0.348456	-0.18406	-0.247203669	0.063141618
2089	P54577	YARS;YAF	tyrosyl-tRNA synthetase;tyrosyl-tRNA synth	8565	ENSG00000134684	25	0.181	0.108	0.031	0.116	0.111	-0.36	0.052	0.205	0.128	-0.09	-0.07	0.1769	-0.007	-0.211	0.0723	0.1115	0.1537	0.2476	-0.1	-0.79	0.155	-0.45	-0.27	-0	0.238	-0.02	0.139	0.349016	0.185829	0.020578723	0.165250416
280	A0A1B0GW	ABHD14A-	ABHD14A-ACY1 readthrough	100526760	ENSG00000114786	17	0.137	0.058	0.108	-0.08	-0.15	-0.28	0.285	0.303	0.112	0.2204	0.1395	0.165	0.0034	-0.197	0.0424	0.4737	0.2473	0.1124	-0.55	-0.84	-0.02	-0.51	0.136	0.013	-0.18	0.569	0.305	0.36416	0.173742	-0.079476836	0.253219074
1648	P17174	GOT1	glutamic-oxaloacetic transaminase 1	2805	ENSG00000120053	20	0.017	0.076	-0.12	0.181	0.514	-0.42	0.324	0.319	0.318	-0.076	-0.337	-0.022	-0.212	-0.288	-0.171	0.0791	-0.04	-0.049	-0.28	-0.37	-0.38	-0.57	-0.35	-0.44	0.029	0.126	0.008	0.081313	0.380231	0.257717758	0.122512907
2814	Q86VN1	VPS36	vacuolar protein sorting 36 homolog	51028	ENSG00000136100	24	0.057	0.151	-0.34	0.154	0.165	-0.3	-0.07	0.09	-0.12	-0.236	0.0418	0.3542	-0.162	-0.09	0.0385	0.1818	0.0812	0.2625	-0.16	-0.21	-0.19	-0.39	-0.29	-0.22	-0.06	0.083	0.512	0.506637	0.077453	-0.075689427	0.153142203
424	B4DG22	RPS6KA3	ribosomal protein S6 kinase A3	6197	ENSG00000177189	30	-0	0.103	-0.07	0.026	-0.16	-0.16	-0.22	-0.28	0.115	0.1977	-0.019	0.0659	0.135	0.1584	0.0275	-0.206	0.1003	-0.13	-0.24	-0.88	-0.15	-0.47	-0.31	0.197	-0.15	0.036	0.188	0.252748	0.124412	-0.109526647	0.233938491
2228	P63092	GNAS	GNAS complex locus	2778	ENSG00000087460	23	0.079	0.567	-0.11	0.198	0.065	-0.26	-0.26	-0.33	0.058	0.0093	0.1193	0.4337	0.1773	-0.329	0.3586	0.1151	0.1495	0.0576	-0.34	-0.45	-0.73	-0.65	-0.54	-0.1	-0.22	0.017	0.247	0.098449	0.307832	-0.120504056	0.428336426
116	A0A087X25	WASHC4	WASH complex subunit 4	23325	ENSG00000136051	27	-0.12	0.02	0.239	-0.07	-0.25	-0.58	-0.18	0.105	0.131	0.0963	0.1993	-0.073	0.034	0.012	-0.105	0.6083	0.38	0.4072	-0.22	-0.86	0.044	-0.45	-0.27	0.058	-0.06	-0.09	-0.04	0.118519	0.13252	-0.250696654	0.383216302
1795	P29992	GNA11	G protein subunit alpha 11	2767	ENSG00000088256	22	-0.07	0.141	-0.24	0.259	-0.08	0.24	-0.06	-0.26	-0.1	-0.191	0.0295	0.1544	0.0672	-0.096	0.3061	0.0613	0.0889	0.1826	-0.16	0.167	-0.24	-0.17	-0.37	-0.24	-0.17	-0.14	0.382	0.346297	0.084804	-0.086550996	0.171355492
3756	Q9Y4E6	WDR7	WD repeat domain 7	23335	ENSG00000091157	34	-0.04	0.071	-0.11	-0.05	-0.04	-0.21	-0.14	-0.07	0.105	-0.049	0.0746	-0.136	0.0701	-0.211	-0.025	0.2563	0.0273	0.0739	-0.23	-0.76	0.052	-0.47	-0.24	0.149	0.008	-0.07	0.163	0.393358	0.100571	-0.064959497	0.165530588
1577	P12814	ACTN1	actinin alpha 1	87	ENSG00000072110	40	-0.07	-0.01	-0.1	-0.18	0.094	-0.48	-0.28	-0.41	-0.09	0.1716	0.197	-0.045	0.1987	-0.023	-0.07	-0.105	0.1138	-0.375	-0.33	-0.79	0.298	0.109	-0.11	0.247	-0.02	0.108	-0.13	0.482085	-0.10142	-0.176841736	0.07542644
3039	Q92616	GCN1	GCN1 activator of EIF2AK4;GCN1, eIF2 alp	10985	ENSG00000089154	64	0.097	0.022	0.082	-0.16	0.039	-0.4	0.046	0.122	0.219	0.0885	0.1229	0.1627	0.033	-0.171	-0.107	-0.068	0.1188	0.2572	-0.21	-0.39	-0.16	-0.29	-0.1	0.152	0.174	-0.04	0.175	0.487647	0.08459	-0.041391609	0.125981344
1886	P37837	TALDO1	transaldolase 1	6888	ENSG00000177156	13	0.235	0.02	-0.18	-0.16	0.293	-0.37	0.217	0.085	0.092	-0.188	-0.155	0.0542	-0.103	-0.193	-0.019	-0.061	-0.046	-0.129	-0.34	-0.94	0.146	0.229	-0.33	0.027	-0.12	-0.1	0.145	0.503463	0.168388	0.119875588	0.048512132
2410	Q13451	FKBP5	FKBP prolyl isomerase 5;FK506 binding prc	2289	ENSG00000096060	26	0.169	0.299	0.272	-0.27	-0.2	-0.56	-0.31	-0.12	0.12	0.0921	-0.159	0.4007	0.0525	-0.04	-0.198	0.0089	-0.057	0.0098	-0.24	-0.93	-0.13	-0.32	-0.23	-0.1	0.001	-0.03	-0.11	0.291619	0.165424	-0.079271281	0.244695034
2551	Q16401	PSMD5	proteasome 26S subunit, non-ATPase 5	5711	ENSG00000095261	22	-0.08	0.436	-0.14	-0.02	-0.14	-0.38	0.063	0.14	0.245	0.1761	0.0082	0.1652	-0.121	-0.192	-0.079	0.1543	0.2827	0.3778	-0.1	-0.38	0.079	0.03	-0.26	-0.06	0.096	0.153	0.331	0.727466	0.02641	-0.071843655	0.098253991
182	G3V5T0	GSTZ1	glutathione S-transferase zeta 1	2954	ENSG00000100577	11	0.187	0.249	0.011	-0.25	0.117	-0.43	0.162	0.2	0.372	-0.037	0.0234	-0.263	-0.083	-0.078	-0.225	0.1451	0.2582	-0.188	-0.13	-1.12	0.026	-0.21	-0.18	-0.07	-0.27	0.047	0.229	0.338191	0.254613	0.11746756	0.137145935
2904	Q8N5I2	ARRDC1	arrestin domain containing 1	92714	ENSG00000197070	12	0.059	-0.08	-0.6	0.33	0.493	-0.34	-0.54	-0.2	-0.41	-0.586	-0.082	0.4264	-0.374	-0.264	-0.222	-0.019	-0.474	0.3635	-0.39	-0.04	-0.56	-0.34	-0.24	-0.59	-0.19	0.039	0.596	0.960793	0.048559	-0.006214199	0.054773355
2869	Q8IYS1	PM20D2	peptidase M20 domain containing 2	135293	ENSG00000146281	16	-0.07	0.192	-0.18	-0.09	-0.01	-0.37	0.01	0.018	-0.14	-0.084	0.1275	-0.066	-0.211	0.0264	0.0985	0.253	0.2536	-0.508	-0.26	-0.89	-0.09	0.085	-0.23	0.032	0.383	0.373	0.125	0.934897	-0.01753	-0.057879393	0.040352362
3144	Q96HU1	SGSM3	small G protein signaling modulator 3	27352	ENSG00000100359	24	0.14	0.13	-0.04	-0.08	-0.28	0.143	0.121	0.04	0.66	0.1737	-0.28	0.0251	0.3153	-0.028	-0.288	0.3044	0.1415	0.0132	-0.29	-0.88	-0.04	-0.52	-0.19	0.152	0.025	-0.11	0.387	0.315756	0.255407	0.050432893	0.204974218
318	A0A2R8Y80	CTNNB1	catenin beta 1	1499	ENSG00000168036	23	0.076	0.086	0.111	-0.06	-0.03	-0.52	-0.12	-0.13	-0.01	0.1445	0.1283	0.1843	0.2435	-0.148	-0.162	0.0364	0.148	0.0345	-0.1	-0.83	-0.18	-0.48	-0.27	0.037	0.048	-0.09	0.172	0.207566	0.12265	-0.133630102	0.256279919
1616	P14868	DARS;DAF	aspartyl-tRNA synthetase;aspartyl-tRNA syr	1615	ENSG00000115866	31	-0.04	0.1	-0.07	0.271	0.179	-0.02	0.089	-0.11	0.126	0.0162	-0.005	-0.025	-0.063	0.0976	0.0511	0.0016	0.2129	0.2315	-0.08	-0.26	0.076	-0.35	-0.2	-0.07	0.213	-0.04	0.062	0.229924	0.130736	0.000428536	0.130307578
3543	Q9NZ08	ERAP1	endoplasmic reticulum aminopeptidase 1	51752	ENSG00000164307	26	0.074	-0.58	-0.23	-0.03	0.014	-0.19	0.275	0.126	-0.12	-0.551	-0.069	-0.522	-0.297	0.2043	0.0673	0.4503	-0.252	-0.25	-0.11	0.258	0.167	0.181	0.093	-0.17	0.5	-0.05	-0.37	0.511231	-0.12888	0.061900852	-0.190777175
2657	Q5VWZ2	LYPLAL1	lysophospholipase like 1	127018	ENSG00000143353	10	-0.03	0.19	-0.2	-0.11	-0.17	-0.37	0.029	0.03	0.323	0.0055	-0.062	0.1336	0.0776	-0.029	0.061	0.1487	-0.015	0.0352	-0.11	-0.78	-0.28	-0.35	-0.23	0.246	-0.03	-0.09	0.252	0.356834	0.118866	-0.073348706	0.192214606
1926	P42785	PRCP	prolylcarboxypeptidase	5547	ENSG00000137509	18	-0.21	-0.5	-0.1	-0.18	-0.14	-0.16	-0.09	0.061	0.078	0.3788	0.21	0.1743	-0.315	0.2107	-0.115	0.099	-0.494	-0.09	-0.01	0.549	0.141	-0.01	0.108	-0.25	0.58	0.269	-0.23	0.252712	-0.26506	-0.144860222	-0.120199077
276	A0A1B0GV2	CTSD	cathepsin D	1509	ENSG00000117984	21	-0.5	-0.47	0.139	-0.54	0.062	0.121	0.057	0.025	0.06	0.7479	-0.02	0.1076	-0.328	-0.061	-0.647	0.0394	-0.226	-0.295	-0.21	0.297	-0.03	-0.1	0.235	-0.17	0.704	0.55	-0.42	0.563768	-0.21067	-0.039787586	-0.170886687
2484	Q14974	KPNB1	karyopherin subunit beta 1	3837	ENSG00000108424	29	-0.27	-0.11	-0.27	-0.09	0.172	-0.46	0.17	0.148	0.187	-0.054	-0.282	-0.196	0.0439	-0.209	0.0162	0.0976	0.033	-0.006	-0.31	-0.48	-0.26	-0.34	-0.35	0.102	0.342	-0.02	0.169	0.849771	0.068269	0.003424016	0.064844639
1916	P42224	STAT1	signal transducer and activator of transcripti	6772	ENSG00000115415	32	0.082	0.228	0.066	-0.43	-0.43	-0.54	-0.05	-0.07	-0.06	-0.106	-0.038	0.2023	0.0253	0.128	-0.168	-0.004	0.0942	0.1072	-0.05	-0.66	0.255	0.02	-0.22	0.058	0.022	-0.12	0.364	0.527031	-0.09684	-0.159636994	0.062798674
3298	Q9BWS9	CHID1	chitinase domain containing 1	66005	ENSG00000177830	18	0.024	-0.44	-0.13	-0.13	-0.15	0.123	-0.64	-0.24	-0.05	0.2912	0.2704	-0.07	-0.186	0.4302	-0.225	-0.332	-0.445	-0.383	0.206	0.448	0.254	-0.05	0.047	-0.07	-0.12	0.048	-0.43	0.415788	-0.21832	-0.11010891	-0.108210579
1564	P11908	PRPS2	phosphoribosyl pyrophosphate synthetase 2	5634	ENSG00000101911	17	0.082	-0.21	0.036	-0.71	-0.26	-0.46	-0.05	0.668	-0.1	-0.004	-0.389	-0.16	-0.328	-0.57	-0.218	-0.019	0.3549	0.258	-0.52	0.1	0.395	-0.92	-0.03	-0.06	0.322	-0.14	0.184	0.973591	-0.03766	0.007714947	-0.045371159
3290	Q9BVG4	PBDC1	polysaccharide biosynthesis domain contain	51260	ENSG00000102390	13	0.014	-0.04	0.015	0.134	-0.04	-0.21	0.146	0.075	0.241	0.2439	-0.017	0.2595	-0.05	-0.048	0.0414	0.1951	0.2226	0.212	-0.22	-0.95	-0.26	-0.5	-0.25	-0.14	-0.07	-0.05	0.15	0.051721	0.289041	-0.080033644	0.369075112
2387	Q13200	PSMD2	proteasome 26S subunit, non-ATPase 2	5708	ENSG00000175166	27	0.065	-0.04	-0.07	-0.07	0.352	-0.36	-0.28	-0.07	-0.05	0.1262	0.0549	-0.041	-0.049	-0.113	-0.011	-0.068	-0.092	-0.136	-0.07	-0.22	0.112	-0.39	-0.31	-0.02	0.169	-0.02	-0	0.887288	0.026399	-0.021503137	0.047902622
3211	Q99571	P2RX4	purinergic receptor P2X 4	5025	ENSG00000135124	12	-0.27	0.414	0.154	0.115	-0.45	-0.41	0.122	-0.17	0.247	0.1493	0.2743	-0.073	0.2553	-0.382	0.1336	0.2515	0.2634	0.3537	-0.38	-0.44	-0.22	-0.63	-0.34	0.331	-0.14	-0.13	0.281	0.214453	0.157689	-0.16436783	0.322056334
1816	P30711	GSTT1	glutathione S-transferase theta 1	2952	ENSG00000277656	14	-1.98	1.32	0.109	0.425	0.125	0.349	0.315	-1.38	-0.61	0.8898	-1.779	0.4828	-1.078	0.0767	0.3848	0.408	-0.917	0.4082	-1.15	-0.74	-0.09	-1.3	0.299	0.68	-1.08	-1.25	-1.33	0.525341	0.513245	-0.023947759	0.537193244
1500	P08263	GSTA1	glutathione S-transferase alpha 1	2938	ENSG00000243955	15	-0.39	-0.56	-0.66	-0.14	-0.58	-0.83	0.222	-0	0.058	0.2604	0.2501	0.1882	-0.513	-0.498	-0.002	-0.094	0.4111	-0.194	-0.49	-0.68	-1.05	0.279	0.698	0.278	-0.16	-0.05	0.638	0.474226	-0.26222	-0.299901797	0.03767769
2753	Q6ZVM7	TOM1L2	target of myb1 like 2 membrane trafficking p	146691	ENSG00000175662	30	0.063	0.303	-0.07	-0.05	-0.06	-0.62	0.02	0.156	0.123	0.0031	0.1548	0.0903	0.2417	-0.075	0.0913	0.2373	0.339	0.3386	-0.32	-0.75	-0.12	-0.78	-0.3	-0.03	-0.13	0.199	0.34	0.147857	0.194238	-0.172833089	0.367071497
366	A5D8V6	VPS37C	VPS37C subunit of ESCRT-I;VPS37C, ES(	55048	ENSG00000167987	8	-0.17	0.416	-0.64	-0.06	-0.08	-0.22	0.172	0.58	-0.22	-0.323	-0.16	0.0917	-0.222	-0.23	-0.02	0.5968	-0.084	0.7344	-0.18	0.246	-0.16	-0.35	-0.6	-0.42	-0.38	-0.06	0.919	0.809788	0.085697	-0.066171166	0.151868416
1052	O14841	OPLAH	5-oxoprolinase, ATP-hydrolysing	26873	ENSG00000178814	36	-0.3	-0.19	-0.26	-0.2	0.036	-0.48	0.034	0.027	0.154	-0.242	-0.307	0.0381	-0.058	-0.08	-0.004	0.1295	-0.193	0.2098	-0.3	-0.52	-0.1	-0.21	-0.14	0.011	0.249	0.044	0.155	0.826503	-0.04012	-0.074555805	0.034436924
1196	O75223	GGCT	gamma-glutamylcyclotransferase	79017	ENSG00000006625	17	0.138	-0.02	-0.05	-0.02	-0.16	-0.44	0.268	0.092	0.412	0.1055	0.0199	0.1428	-0.072	-0.254	0.1412	0.2518	-0.024	0.1243	-0.07	-0.74	-0.1	-0.64	-0.39	0.019	0.157	0.207	0.372	0.499926	0.154231	-0.025030065	0.179261445
1105	O43242	PSMD3	proteasome 26S subunit, non-ATPase 3	5709	ENSG00000108344	27	0.038	0.015	-0.03	-0.08	0.253	-0.3	-0.02	-0.1	0.09	0.028	-0.022	-0.018	-0.042	-0.127	-0.03	0.099	0.0303	0.0757	-0.16	-0.49	-0.06	-0.38	-0.23	-0.04	0.099	-0.05	0.078	0.277951	0.1204	-0.014735532	0.135135369
1729	P23381	WARS1;W	tryptophanyl-tRNA synthetase 1;tryptophany	7453	ENSG00000140105	23	0.17	0.21	0.151	0.168	0.255	-0.43	-0.09	0.204	0.196	-0.059	0.0327	0.0526	-0.048	-0.177	-0.183	-0.066	0.0279	0.0492	-0.1	-0.67	-0.12	-0.03	-0.45	0.086	0.11	0.119	0.207	0.343638	0.186377	0.133708554	0.052668081
3651	Q9UL25	RAB21	RAB21, member RAS oncogene family	23011	ENSG00000080371	17	0.045	0.319	0.115	0.05	-0.1	-0.42	0.256	0.086	0.213	-0.015	0.1738	-0.039	0.0414	-0.217	0.1731	0.3282	0.2543	0.2194	-0.38	-0.75	-0.13	-0.54	-0.27	0.019	0.131	0.011	0.175	0.14912	0.255262	-0.039607605	0.294869313
1993	P49407	ARRB1	arrestin beta 1	408	ENSG00000137486	14	0.142	0.108	-0.14	0.019	-0.08	-0.47	0.086	-0.07	0.235	0.0288	0.0128	0.0923	0.1371	0.0854	0.0105	0.1507	0.0451	0.0283	-0.02	-0.77	-0.19	-0.55	-0.32	0.105	-0.02	0.018	0.201	0.230048	0.15258	-0.084566279	0.237146023
1696	P20810	CAST	calpastatin	831	ENSG00000153113	21	-0.11	0.159	-0.1	-0.13	-0.03	-0.5	0.146	-0.03	0.202	-0.027	-0.053	-0.005	-0.146	-0.047	0.1955	0.1115	0.3043	-0.037	-0.09	-0.88	-0.25	-0.72	-0.49	-0.04	0.142	0.119	0.204	0.294599	0.177756	-0.077419824	0.255175944
613	Q04637	EIF4G1	eukaryotic translation initiation factor 4 gamr	1981	ENSG00000114867	38	0.045	0.136	-0.05	-0.25	0.058	-0.46	0.069	6E-04	0.267	-0.002	-0.027	-0.03	0.0484	-0.232	-0.033	0.0301	0.1193	0.1766	-0.16	-0.4	-0.15	-0.4	-0.24	0.074	0.102	-0.02	0.154	0.516349	0.094424	-0.026133285	0.120557416
2444	Q14152	EIF3A	eukaryotic translation initiation factor 3 subu	8661	ENSG00000107581	50	-0.02	-0.2	-0.17	-0.18	0.078	-0.27	-0.18	-0.15	0.082	-0.052	-0.085	-0.1	-0.006	-0.072	0.0406	-0.008	0.0604	0.1521	-0.11	-0.18	-0.06	-0.28	-0.1	0.012	0.122	-0.12	-0.01	0.294648	-0.03136	-0.103514839	0.072151933
25	A0A087X2I2	GPX4	glutathione peroxidase 4	2879	ENSG00000167468	16	-0.32	0.138	0.034	0.24	-0.01	-0.34	-0.3	-0.22	0.031	0.2255	0.1689	-0.239	0.1102	-0.288	0.2907	-0.374	0.0809	-0.259	-0.07	-0.54	-0.28	-0.63	-0.43	-0.03	-0.31	-0.19	-0.2	0.169612	0.216332	-0.050442917	0.266774739
1557	P11233	RALA	RAS like proto-oncogene A	5898	ENSG00000006451	18	-0.09	0.448	-0.29	0.278	0.239	-0.47	-0.24	-0.01	0.079	0.0598	0.189	0.0185	0.1131	-0.367	0.3744	0.4189	0.2219	0.5518	-0.04	-0.06	-0.05	-0.82	-0.42	-0.12	-0.14	0.177	0.333	0.268503	0.120727	-0.181030194	0.301757375
1869	P35998	PSMC2	proteasome 26S subunit, ATPase 2	5701	ENSG00000161057	25	-0.05	-0.07	-0.14	-0	0.278	-0.25	0.142	-0.05	0.045	-0.062	-0.013	-0.018	-0.073	-0.13	-0.049	0.0827	0.049	0.1745	-0.22	-0.49	-0.08	-0.2	-0.26	0.018	0.142	-0.03	-0.02	0.328587	0.11437	-0.00783708	0.122207113
3294	Q9BW04	C1orf116;S	chromosome 1 open reading frame 116	79098	ENSG00000182795	25	0.232	0.412	-0.29	-0	0.035	-0.46	0.098	0.151	0.08	-0.238	0.2028	0.0947	0.0166	0.0303	0.192	0.388	0.2426	0.2556	-0.29	-0.52	-0.3	-0.7	-0.44	-0.28	0.285	0.244	0.254	0.192892	0.222637	-0.103208055	0.325845354
1793	P29728	OAS2	2'-5'-oligoadenylate synthetase 2	4939	ENSG00000111335	24	-0.49	0.383	-0.14	-0.39	-0.77	-0.61	-0.18	-0.5	-0.22	-0.351	0.3162	0.3572	0.3759	0.309	-0.112	-0.238	0.0125	-0.23	-0.06	-0.55	0.426	-0.6	0.358	0.133	-0.17	-0.01	0.334	0.1928	-0.30913	-0.37299995	0.063869246
2587	Q4VX76	SYTL3	synaptotagmin like 3	94120	ENSG00000164674	30	-0.01	0.17	-0.09	-0.04	-0.19	-0.36	0.029	0.492	0.211	0.0957	0.006	0.1932	0.0831	-0.067	-0.05	0.0475	0.0945	0.1496	-0.08	-0.5	-0.21	-0.45	-0.48	0.134	0.211	0.061	0.509	0.575755	0.112432	-0.038394405	0.150826126
337	P49281	SLC11A2	solute carrier family 11 member 2	4891	ENSG00000110911	6	-0.26	0.48	-0.01	-0.14	-0.02	-0.51	0.421	0.193	0.122	-0.109	0.0586	0.002	0.015	-0.31	-0.2	0.5298	0.219	0.7674	-0.38	0.008	-0.44	-0.29	-0.38	-0.29	0.031	0.116	0.507	0.477879	0.15455	-0.077981692	0.232531892
1863	P35580	MYH10	myosin heavy chain 10	4628	ENSG00000133026	70	0.013	0.082	-0.15	-0.03	-0.03	-0.24	0.003	-0.15	0.189	0.078	-0.015	-0.033	0.1569	-0.033	0.195	0.2044	0.0475	0.0064	-0.03	-0.54	-0.21	-0.3	-0.23	0.058	0.338	0.136	0.031	0.362713	0.04785	-0.102646511	0.150496423
1988	P49257	LMAN1	lectin, mannose binding 1	3998	ENSG00000074695	14	0.111	-0.2	0.005	-0.31	0.125	-0.23	-0.06	-0	-0.04	0.0645	0.0473	-0.213	-0.125	0.4576	0.0633	0.0175	0.1821	0.1449	-0.15	-0.12	0.025	-0.52	-0.21	0.111	0.076	-0.15	0.35	0.418207	-0.00236	-0.138086871	0.135724477
1132	O43687	AKAP7	A-kinase anchoring protein 7	9465	ENSG00000118507	10	0.432	0.523	-0.04	0.214	0.373	-0.5	-0.29	-0.38	-0.19	-0.257	0.5703	-0.186	0.6711	-0.35	0.0748	0.026	0.17	0.0451	-0.18	-0.71	-0.19	-0.63	-0.13	-0.02	-0.11	-0.25	-0.09	0.224981	0.272143	-0.070003232	0.342146413
1076	O15145	ARPC3	actin related protein 2/3 complex subunit 3	10094	ENSG00000111229	8	0.034	0.227	0.027	-0.02	0.085	-0.42	0.502	0.368	0.418	0.1721	0.0147	-0.021	0.1015	-0.086	0.167	0.3031	0.2443	0.5577	-0.23	-0.73	0.098	0.022	-0.34	0.123	0.141	0.262	0.424	0.487726	0.16073	-0.026203107	0.186932943
2102	P55010	EIF5	eukaryotic translation initiation factor 5	1983	ENSG00000100664	16	0.131	0.134	-0.01	0.006	-0.07	-0.27	0.109	0.017	0.175	0.199	0.1476	0.0545	-0.035	-0.008	0.1149	0.1088	0.2283	0.3507	-0.26	-0.7	-0.1	-0.54	-0.19	0.028	0.166	0.017	0.086	0.116208	0.191706	-0.104844689	0.296550603
1200	O75348	ATP6V1G	ATPase H+ transporting V1 subunit G1	9550	ENSG00000136888	10	-0.07	0.117	-0.17	-0.14	-0.06	-0.47	0.16	-0.02	0.49	-0.062	0.0354	0.0126	-0.101	-0.279	-0.009	0.3325	0.2081	0.1944	-0.12	-0.78	-0.23	-0.57	-0.35	-0.02	0.075	0.016	0.214	0.322242	0.176311	-0.055853076	0.232163823
2742	Q6YHK3	CD109	CD109 molecule	135228	ENSG00000156535	26	-0.37	-0.3	-0.26	-0.02	-0.23	-0.34	0.155	0.399	0.123	0.1967	0.1826	0.2608	-0.11	-0.183	-0.331	-0.048	0.3527	-0.12	-0.69	-0.27	-0.2	-0.52	-0.02	0.047	0.613	0.515	-0.44	0.76427	0.013883	-0.115026075	0.128909089
1354	P01133	EGF	epidermal growth factor	1950	ENSG00000138798	18	-0.01	0.11	0.135	0.133	0.495	-0.29	-0.22	-0.13	-0.2	0.1696	0.1597	0.1909	-0.254	0.1305	-0.359	-0.437	-0.094	-0.029	-0.41	-0.41	0.546	-0.41	-0.65	0.085	-0.45	-0.35	-0	0.399926	0.23011	0.060392008	0.169717639
3083	Q96A49	SYAP1	synapse associated protein 1	94056	ENSG00000169895	12	0.235	0.155	0.009	-0.13	-0.01	-0.42	-0.32	-0.27	0.018	-0.019	0.1264	0.016	-0.05	-0.06	0.212	-0.157	0.2807	-0.178	-0.26	-0.9	-0.19	-0.81	-0.39	0.095	-0.11	-0.23	-0.03	0.138172	0.234614	-0.099502252	0.334115758
2385	Q13177	PAK2	p21 (RAC1) activated kinase 2	5062	ENSG00000180370	19	-0.02	0.242	-0	0.021	-0.06	-0.52	0.114	-0.04	0.254	0.1253	0.0504	0.0478	-0.043	-0.194	0.1314	0.2224	0.2302	0.1479	-0.23	-0.85	-0.22	-0.5	-0.48	-0.01	0.032	0.074	0.299	0.199547	0.207091	-0.081067905	0.288159019
1710	P21810	BGN	biglycan	633	ENSG00000182492	15	-0.32	-0.61	-0.67	-0.32	-0.42	-0.62	0.18	0.238	-0.41	-0.146	0.3107	0.4184	-0.175	0.5828	0.1006	-0.054	-0.594	-0.328	0.775	-0.09	0.903	0.021	0.13	-0.26	-0.77	-0.07	-0.57	0.330994	-0.3365	-0.341552438	0.005054509
1540	P10301	RRAS	RAS related	6237	ENSG00000126458	18	-0.16	0.111	-0.2	0.338	-0.2	-0.14	-0.01	-0.09	0.041	0.1304	0.3985	0.0476	0.1583	0.0965	0.142	0.3669	0.2919	-0.032	-0.54	-0.37	-0.27	-0.52	-0.4	-0.12	-0.04	0.118	0.406	0.086508	0.157061	-0.212874735	0.369935989
3800	Q9Y6W5	WASF2	WASP family member 2;WAS protein family	10163	ENSG00000158195	16	-0.38	-0.18	0.034	-0.28	-0.13	-0.3	0.096	-0.08	0.22	-0.113	-0.031	0.0241	-0.131	-0.128	0.0384	0.1563	0.2441	0.2921	-0.62	-0.87	-0.05	-0.38	-0.19	-0.14	0.099	0.042	0.181	0.25801	0.105004	-0.150202753	0.255206575
516	Q13098	GPS1	G protein pathway suppressor 1	2873	ENSG00000169727	19	-0.04	-0.05	-0.14	-0.05	-0.01	-0.45	0.471	-0.05	0.093	0.0201	-0.066	0.0064	0.0443	-0.188	0.1084	0.1316	0.0621	0.1603	-0.34	-0.65	-0.04	-0.4	-0.38	0.044	0.276	-0	0.099	0.390081	0.130917	-0.054768993	0.185686196
2046	P51665	PSMD7	proteasome 26S subunit, non-ATPase 7	5713	ENSG00000103035	12	-0.01	0.021	-0.06	-0.26	0.388	-0.31	0.038	0.125	0.04	-0.004	-0.024	-0.023	-0.116	-0.338	0.1929	0.0233	0.147	0.2888	-0.27	-0.59	-0.07	-0.27	-0.1	0.196	0.122	-0.05	0.132	0.609943	0.0979	-0.019309208	0.117209556
2439	Q14019	COTL1	coactosin like F-actin binding protein 1	23406	ENSG00000103187	14	0.171	0.474	-0	-0.98	-0.19	-0.56	0.028	0.226	-0.31	0.7331	-0.436	0.1559	-0.006	0.0085	-0.387	-0.479	0.2811	-0.282	0.744	-1.26	-0.27	-0.75	0.056	0.876	-0.4	0.127	-0.63	0.922667	0.039919	-0.080829139	0.1207477
3596	Q9UEF7	KL	klotho	9365	ENSG00000133116	17	0.149	0.383	-0.71	-0.8	0.023	0.42	0.33	0.12	0.18	-0.087	-0.797	0.4113	-0.193	0.6784	-0.5	-0.056	-0.384	0.6483	-0.3	0.554	-0.13	-0.32	-0.48	-0.35	-0.5	-0.88	-0.46	0.425163	0.32968	0.042002395	0.287677984

2086	P54136	RARS;RAI	arginyl-tRNA synthetase;arginyl-tRNA synth	5917	ENSG00000113643	28	0.084	0.15	0.048	-0.01	0.034	-0.43	-0.13	-0.07	0.123	-0.068	-0.046	-0.011	-0.045	-0.251	-0.065	-0.121	0.2643	0.1703	-0.23	-0.73	-0.03	-0.36	-0.28	0.05	0.061	-0.09	0.05	0.353852	0.151893	-0.002579673	0.154472939
2867	Q8IYI6	EXOC8	exocyst complex component 8	149371	ENSG00000116903	31	0.011	0.208	-0.26	0.031	0.007	-0.37	0.091	0.082	0.286	-0.102	0.0784	0.1814	0.0273	-0.043	0.1051	0.1634	0.1605	0.2939	-0.35	-0.87	0.317	-0.48	-0.38	-0.01	0.144	0.199	0.36	0.433991	0.130407	-0.086418353	0.216824905
903	I3L3P7	RPS15A	ribosomal protein S15a	6210	ENSG00000134419	9	0.23	-0.26	-0.24	-0.01	0.263	-0.31	0.084	-0.07	0.246	-0.111	-0.14	-0.223	0.0203	-0.144	-0.091	0.0362	0.0952	0.0262	-0.05	-0.25	-0.07	-0.19	-0.16	0.109	0.327	-0.24	0.128	0.891382	0.035741	0.050919271	-0.015178403
1873	P36507	MAP2K2	mitogen-activated protein kinase kinase 2	5605	ENSG00000126934	19	0.019	0.094	-0.08	-0.09	-0.2	-0.38	4E-04	0.015	0.242	0.1719	0.0764	0.0666	0.0073	-0.08	0.144	0.0972	0.1559	0.1487	-0.17	-0.72	-0.21	-0.49	-0.39	-0.03	-0.06	0.033	0.138	0.111018	0.1663	-0.130285438	0.296585589
1164	O60547	GMDS	GDP-mannose 4,6-dehydratase	2762	ENSG00000112699	21	0.088	-0.05	0.046	-0.12	-0.1	-0.49	0.113	-0.06	0.374	0.1642	-0.207	0.0388	0.0293	-0.046	-0.205	-3E-04	-0.082	0.1404	0.017	-0.51	0.183	-0.21	-0.19	0.246	0.125	-0.11	0.083	0.978802	0.018963	-0.003315977	0.022278527
2373	Q13045	FLII	FLII, actin remodeling protein;FLII actin rem	2314	ENSG00000284571	33	0.111	0.182	0.015	-0.04	0.08	-0.36	0.052	0.152	0.123	0.0147	0.1057	0.1355	0.1326	-0.231	0.1209	0.2522	0.1079	0.1273	-0.28	-0.74	-0.13	-0.4	-0.27	0.189	0.232	0.094	0.22	0.311291	0.154897	-0.050473985	0.205370609
3491	Q9NRX4	PHPT1	phosphohistidine phosphatase 1	29085	ENSG00000054148	9	0.168	0.215	0.226	-0.05	-0.17	-0.44	-0.23	-0.37	0.032	-0.176	-0.035	0.2382	0.0283	-0.137	0.2278	-0.276	0.0066	-0.126	-0.38	-1.31	-0.14	-1.09	-0.55	0.062	-0.13	-0.23	-0.2	0.126489	0.374524	-0.039442881	0.413967328
3248	Q9BRG1	VPS25	vacuolar protein sorting 25 homolog	84313	ENSG00000131475	14	0.192	0.361	-0.24	0.158	0.254	-0.27	-0.05	0.135	-0.2	-0.125	0.2099	0.4772	-0.011	-0.12	-0.033	0.0221	0.0037	0.192	-0.22	-0.33	-0.07	-0.04	-0.26	-0.08	-0.15	-0.06	0.402	0.427721	0.126744	-0.030713435	0.157457478
1664	P18124	RPL7	ribosomal protein L7	6129	ENSG00000147604	13	-0.05	0.026	-0.06	0.093	0.264	-0.1	0.258	-0.07	0.112	-0.121	-0.169	-0.141	-0.257	0.209	-0.095	0.1049	0.3314	0.2409	-0.25	-0.3	-0.13	-0.13	-0.02	0.14	0.258	-0.3	0.056	0.508839	0.126265	0.03993117	0.086333944
2340	Q08257	CRYZ	crystallin zeta	1429	ENSG00000116791	16	0.001	-0.01	0.152	-0	0.1	-0.35	0.32	0.13	0.334	-0.359	0.3026	0.0284	-0.499	-0.001	0.1755	0.087	0.1668	0.1881	-0.35	-0.55	0.152	-0.53	-0.48	-0.16	0.39	0.18	0.289	0.526443	0.192667	0.064955854	0.127711513
2191	P62277	RPS13	ribosomal protein S13	6207	ENSG00000110700	9	-0.05	-0.24	-0.37	-0.27	0.545	-0.43	0.567	0.154	0.36	-0.285	-0.45	-0.166	-0.373	-0.425	-0.146	0.2108	0.2214	0.3455	-0.47	-0.69	-0.24	-0.24	-0.23	-0.17	0.263	-0.01	0.299	0.595406	0.194798	0.14868908	0.046109319
27	G3V4F2	ACOT1	acyl-CoA thioesterase 1	641371	ENSG00000184227	4	-0.33	0.104	0.006	-0.31	-0.29	-0.73	0.242	0.007	0.105	0.9882	-0.093	0.1405	-0.246	-0.25	0.235	0.084	0.2739	0.2934	0.37	-0.04	-0.18	-1.35	-0.37	-0.16	0.112	-0.02	0.504	0.414145	-0.00584	-0.291210399	0.285366715
2010	P49902	NT5C2	5'-nucleotidase, cytosolic II	22978	ENSG00000076685	21	0.06	0.291	-0.03	-0.16	0.012	-0.44	-0.12	-0.14	0.028	0.3357	0.1894	0.1735	0.0925	-0.223	-0.065	0.1801	0.1483	-0.079	-0.34	-0.93	-0.24	-0.39	-0.3	0.059	0.206	-0.01	0.085	0.203116	0.150867	-0.138927951	0.289794627
3667	Q9UN37	VPS4A	vacuolar protein sorting 4 homolog A	27183	ENSG00000132612	30	0.124	0.306	0.034	0.012	-0.12	-0.37	-0.09	-0.08	-0.02	0.0542	0.1137	0.179	0.1005	0.1059	-0.109	0.119	0.0207	0.1142	-0.09	-0.53	-0.28	-0.71	-0.22	-0.1	-0.02	-0.07	0.279	0.145976	0.170674	-0.1008362	0.27150975
3516	Q9NV70	EXOC1	exocyst complex component 1	55763	ENSG00000090989	22	0.016	0.159	-0.13	-0.01	0.026	-0.41	-0.33	-0.26	-0.07	-0.046	0.0603	0.1938	-0.045	0.0414	0.0184	-0.14	0.0947	-0.019	-0.23	-0.8	-0.3	-0.5	-0.41	-0.12	0.239	0.181	0.113	0.305323	0.090622	-0.129719576	0.22034141
1214	O75531	BANF1	barrier to autointegration factor 1	8815	ENSG00000175334	10	-0.14	0.127	-0.05	-0.12	-0.03	-0.63	-0.15	-0.52	-0.23	-0.511	-0.395	-0.087	-0.033	-0.109	0.0293	-0.277	0.3059	0.0741	-0.25	-0.82	-0.56	-1.05	-0.39	0.332	-0.22	-0.28	-0.14	0.349977	0.181041	-0.083158516	0.264199313
1308	O95782	AP2A1	adaptor related protein complex 2 subunit al	160	ENSG00000196961	32	0.014	0.12	-0.11	0.153	0.118	-0.17	-0	-0.09	0.164	0.0729	-0.015	0.0315	0.016	-0.085	-0.011	0.1272	0.0854	0.2266	-0.17	-0.45	-0.19	-0.27	-0.18	-0.03	0.073	0.034	0.117	0.149208	0.141254	-0.027293516	0.168547564
2912	Q8N8N7	PTGR2	prostaglandin reductase 2	145482	ENSG00000140043	13	-0.01	-0.05	-0.33	-0.07	-0.12	-0.41	-0.04	-0.04	0.191	-0.104	0.42	0.5829	0.0952	-0.026	0.0069	-0.303	0.1573	-0.005	-0.13	-0.95	-0.13	-0.69	-0.31	0.008	-0.04	-0.25	-0.03	0.131091	0.182338	-0.190196941	0.372535046
2527	Q15599	SLC9A3R2	SLC9A3 regulator 2	9351	ENSG00000065054	18	-0.23	0.306	-0.44	0.159	-0.17	-0.15	-0.06	0.098	-0.07	-0.273	0.1864	0.2495	-0.131	0.185	0.1164	0.2782	-0.056	0.2179	-0.11	-0.21	-0.25	-0.31	-0.47	-0.34	0.041	-0.06	0.236	0.191034	0.101349	-0.14791905	0.24926824
1375	P01876	IGHA1	immunoglobulin heavy constant alpha 1	3493	ENSG00000211895	18	-1.86	-1.7	-1.04	-1.42	-1.74	-0.8	-1.94	-1.26	-1.99	-2.342	-0.944	-1.179	-1.518	-1.054	-1.641	-0.813	-1.52	-1.855	-1.03	-0.91	2.977	2.591	0.14	1.378	-0.61	-1.5	-1.94	0.082396	-1.64926	-0.098358254	-1.550901158
1864	P35606	COPB2	coatomer protein complex subunit beta 2	9276	ENSG00000184432	33	0.137	0.017	-0.02	-0.11	0.034	-0.35	-0.11	0.069	0.141	0.0653	-0.033	0.0642	0.0426	-0.158	0.0518	0.1486	0.1078	0.2013	-0.19	-0.68	-0.04	-0.17	-0.16	0.084	0.306	0.017	0.203	0.559685	0.048292	-0.076682887	0.124974646
721	F5H7S7	IQGAP2	IQ motif containing GTPase activating prote	10788	ENSG00000145703	39	0.115	0.448	0.008	0.192	0.375	-0.32	-0.15	0.081	0.038	-0.239	-0.138	0.003	-0.002	0.0484	0.0016	0.3099	-0.211	-0.121	-0.27	-0.39	-0.28	-0.35	-0.34	-0.22	0.019	0.184	-0.1	0.130552	0.281882	0.126115457	0.155766392
2215	P62879	GNB2	G protein subunit beta 2	2783	ENSG00000172354	22	0.011	0.406	-0.15	0.263	0.044	-0.36	-0.15	-0.28	0.033	-0.059	0.0347	0.1501	0.114	-0.403	0.4075	0.3127	0.1969	0.211	-0.3	-0.22	-0.49	-0.46	-0.5	-0.29	-0.27	-0.04	0.362	0.126493	0.225358	-0.127009912	0.35236751
3206	Q99519	NEU1	neuraminidase 1	4758	ENSG00000204386	16	-0.25	-0.57	0.005	-0.47	-0.08	-0.1	-0.29	0.139	-0.13	0.1151	0.2394	-0.284	-0.42	0.2175	-0.257	0.1462	-0.058	0.2494	-0.09	0.67	0.243	-0.35	-0.21	-0.14	0.48	-0.11	-0.29	0.396144	-0.21599	-0.188132974	-0.02785743
3419	Q9H9H4	VPS37B	VPS37B subunit of ESCRT-I;VPS37B, ESC	79720	ENSG00000139722	14	-0.01	0.258	-0.6	0.309	0.16	-0.31	-0.26	0.037	-0.32	-0.732	0.1556	0.4917	-0.161	-0.187	0.0981	0.1172	0.0575	0.2907	-0.23	0.061	-0.44	0.054	-0.38	-0.29	-0.31	0.01	0.555	0.788563	0.025518	-0.098170195	0.123688538
1948	P46778	RPL21	ribosomal protein L21	6144	ENSG00000122026	8	0.16	-0.09	-0.07	-0.15	0.442	-0.12	0.201	-0.07	0.213	-0.184	-0.116	-0.079	-0.256	-0.102	-0.057	-0.253	0.1892	0.1223	-0.07	-0.54	0.048	-0.21	-0.08	-0.06	0.131	-0.2	-0.02	0.286047	0.168764	0.140152036	0.02861207
352	C9J4Z0	SLC22A23	solute carrier family 22 member 23	63027	ENSG00000137266	11	-0.32	0.427	0.184	-0.06	2E-04	-0.56	-0.01	-0.05	-0.04	0.3406	0.0368	0.0201	0.3817	-0.51	0.431	0.1424	0.1666	0.2929	-0.44	-0.44	-0.09	-0.53	-0.39	0.431	-0.25	0.099	0.186	0.263843	0.10894	-0.192972357	0.301912574
1849	P34949	MPI	mannose phosphate isomerase	4351	ENSG00000178802	17	0.052	0.201	-0.04	-0.07	0.006	-0.55	-0.07	-0.14	0.075	0.2674	0.0142	0.2135	-0.097	-0.198	0.2252	0.0149	0.1762	-0.077	-0.14	-0.86	-0.16	-0.55	-0.51	-0.01	-0.15	-0.04	0.225	0.172283	0.183588	-0.121033403	0.304621761
3770	Q9Y5L3	ENTPD2	ectonucleoside triphosphate diphosphohydro	954	ENSG00000054179	12	-0.13	0.122	-0.14	0.081	-0.09	-0.08	-0.2	-0.13	0.15	-0.1	0.1776	0.1645	-0.092	0.076	-0.002	0.2078	0.0496	0.5066	-0.07	-0.05	0.039	-0.37	-0.22	-0.07	-0.07	-0.18	0.038	0.118269	0.061185	-0.155992706	0.217177349
3014	Q8WVQ1	CANT1	calcium activated nucleotidase 1	124583	ENSG00000171302	19	-0.42	-0.91	-0.55	-0.31	-0.35	0.063	-0.38	-0.31	-0.32	0.2543	0.4622	0.2946	-0.489	0.594	-0.33	-0.023	-0.549	-0.33	-0.38	-0.19	0.062	-0.34	-0.15	-0.28	0.54	0.175	-0.45	0.207521	-0.27664	-0.376265578	0.099628775
1947	P46777	RPL5	ribosomal protein L5	6125	ENSG00000122406	12	0.119	-0.09	-0.16	0.124	0.389	-0.09	0.112	-0.15	0.162	-0.236	-0.141	-0.155	-0.264	-0.038	-0.039	-0.122	0.2509	0.0881	-0.06	-0.41	0.049	-0.07	-0.14	-0.12	0.193	-0.22	-0.09	0.338251	0.143435	0.119599022	0.02383615
1875	P36578	RPL4	ribosomal protein L4	6124	ENSG00000174444	17	0.118	-0.01	-0.13	0.054	0.446	-0.41	0.129	-0.14	0.04	-0.157	-0.242	-0.136	-0.145	-0.169	0.1042	-0.037	0.1681	0.1741	-0.2	-0.47	-0.12	0.391	-0.24	-0	0.119	-0.38	-0.13	0.629111	0.125433	0.05892437	0.066508305
3675	Q9UNH7	SNX6	sorting nexin 6	58533	ENSG00000129515	22	0.005	0.096	-0.12	-0.22	0.004	-0.41	-0.09	-0.01	0.266	-0.134	-0.151	-0.075	0.0463	-0.038	0.0634	0.1689	0.249	0.1327	-0.21	-0.73	-0.14	-0.31	-0.11	-0	0.277	0.015	0.137	0.51767	0.065615	-0.082512628	0.148127881
1958	P47813	EIF1AX	eukaryotic translation initiation factor 1A X-li	1964	ENSG00000173674	7	0.035	0.03	-0.02	-0.3	-0.09	-0.42	-0.12	-0.71	0.087	-0.298	-0.143	-0.037	-0.219	-0.166	0.0739	-0.403	-0.325	-0.166	-0.2	-1	-0.31	-0.5	-0.31	0.017	-0.26	-0.57	-0.51	0.222812	0.238184	0.020124289	0.218059599
2505	Q15185	PTGES3	prostaglandin E synthase 3	10728	ENSG00000110958	8	-0.11	-0.02	-0.15	0.047	-0.15	-0.47	-0.15	0.176	0.048	-0.007	-0.089	-0.044	0.0862	-0.073	-0.078	-0.093	0.1012	0.052	-0.41	-0.91	-0.42	-0.55	-0.32	-0.02	0.063	-0.09	0.085	0.16381	0.200573	-0.070372484	0.270945143
921	J3KQA0	SYT1	synaptotagmin 1	6857	ENSG00000067715	23	-0.09	0.121	0.198	-0.42	-0.43	-0.69	-0.04	0.133	-0.53	0.8367	-0.343	0.1579	-0.058	0.142	-0.278	0.1667	0.1755	-0.209	-0.22	1.086	-0.13	-0.15	-0.01	0.167	-0.39	0.017	-0.53	0.519407	-0.17441	-0.25876533	0.084355962
1635	P16035	TIMP2	TIMP metallopeptidase inhibitor 2	7077	ENSG00000035862	13	-0.07	-0.25	0.178	-0.41	-0.31	0.054	-0.61	-0.22	-0.47	0.2154	0.1416	-0.543	-0.912	-0.179	-0.885	-0.477	-0.563	-0.79	0.28	1.037	0.833	0.293	0.123	0.002	0.511	0.157	-0.74	0.055915	-0.5135	0.207260881	-0.720764775
2793	Q7Z6L1	TECPR1	tectonin beta-propeller repeat containing 1	25851	ENSG00000205356	30	-0.21	0.103	-0.14	0.077	-0.21	-0.48	0.099	0.09	0.224	-0.054	-0.039	0.0751	0.1157	-0.238	0.0967	0.0743	0.1182	0.1831	-0.26	-0.7	-0.23	-0.52	-0.38	-0.02	0.134	0.101	0.005	0.217059	0.158826	-0.085626382	0.244452032
1569	P12109	COL6A1	collagen type VI alpha 1 chain	1291	ENSG00000142156	22	-0.34	-0.82	-0.28	-0.56	-0.19	0.258	-0.02	-0.27	0.903	-0.279	0.1929	-0.204	-0.767	-0.028	-0.505	-0.336	-0.479	-0.149	-0.36	0.41	1.041	-0.47	-0.25	-0.13	0.482	0.366	0.065	0.293709	-0.27589	0.136866261	-0.412760637
2277	Q00169	PITPNA	phosphatidylinositol transfer protein alpha	5306	ENSG00000174238	22	0.233	0.366	-0.02	0.121	0.026	-0.46	0.078	-0	0.186	-0.034	0.0234	0.0788	0.0039	-0.276	0.0732	0.1549	0.109	-0.111	-0.27	-0.81	-0.25	-0.66	-0.42	-0.02	-0.18	0.092	0.139	0.125214	0.324132	0.05601786	0.268114186
494	C9J5C3	PDCD10	programmed cell death 10	11235	ENSG00000114209	10	0.269	0.326	-0.17	0.149	0.119	-0.35	0.112	-0.14	0.111	-0.327	-0.01	0.2225	0.0205	-0.239	0.1166	0.1106	0.0735	0.2955	-0.34	-0.73	-0.37	-0.48	-0.26	-0.16	-0.14	-0.02	0.297	0.142221	0.292203	0.018657183	0.273545421
639	E9PEP6	NRP1	neuropilin 1	8829	ENSG00000099250	27	-0.49	-0.51	-0.23	-0.2	-0.03	0.216	0.191	-0.33	-0.1	-0.094	0.0728	-0.293	-0.431	0.3521	-0.119	-0.19	-0.268	-0.043	-0.04	0.441	0.185	0.137	0.053	-0.11	0.597	-0.25	-0.23	0.261691	-0.25196	-0.051348634	-0.200607164
502	C9J7K9	PLSCR1	phospholipid scramblase 1	5359	ENSG00000188313	11	-0.35	0.282	-0.34	-0.27	-0.43	-0.16	-0.37	0.152	-0.53	-0.272	0.0447	-0.022	0.3766	0.3361	-0.274	0.1509	-0.017	0.0489	-0.02	0.06	0.063	-0.17	0.353	0.008	-0.01	-0.11	0.273	0.138296	-0.27388	-0.265562389	-0.008317807
1971	P48449	LSS	lanosterol synthase	4047	ENSG00000160285	22	-0.19	0.419	0.095	-0.01	-0.21	-0.19	0.138	-0.18	-0.01	0.1157	-0.12	-0.056	0.0412	0.4083	0.0381	0.1245	0.4215	0.1276	-0.21	-0.53	-0.41	-0.44	-0.31	0.132	-0.17	0.077	0.166	0.128465	0.173476	-0.137228483	0.310704411
1705	P21283	ATP6V1C1	ATPase H+ transporting V1 subunit C1	528	ENSG00000155097	23	0.153	0.456	0.015	-0.05	0.024	-0.39	-0.16	-0.08	0.155	0.1996	0.1269	0.1486	0.0641	-0.192	0.0443	0.1022	0.148	-0.026	-0.05	-1.03	-0.19	-0.52	-0.32	0.089	0.067	-0.09	0.095	0.202973	0.229214	-0.055128472	0.284342256
2891	Q8N1G4	LRRC47	leucine rich repeat containing 47	57470	ENSG00000130764	26	-0.07	-0.05	-0.12	0.036	-0.01	-0.33	0.053	-0.22	0.076	0.0225	0.0141	-0.045	0.0639	-0.093	0.062	-0.134	0.0375	0.061	-0.16	-0.35	-0.14	-0.5	-0.15	0.069	0.081	-0.03	0.161	0.457894	0.040764	-0.070920285	0.111684267
1222	O75674	TOM1L1	target of myb1 like 1 membrane trafficking p	10040	ENSG00000141198	19	-0.34	-0.01	-0.06	0.247	-0.14	-0.67	0.133	0.056	0.016	-0.319	0.1548	-0.096	0.3019	-0.07	-0.039	0.2903	0.5263	0.1906	-0.38	0.086	0.038	-0.63	-0.49	-0.15	-0.19	0.258	0.267	0.355317	0.046332	-0.189495059	0.235826943
1956	P47755	CAPZA2	capping actin protein of muscle Z-line subur	830	ENSG00000198898	13	0.036	0.299	-0.1	-0.07	-0.02	-0.52	0.208	0.005	0.185	-0.1	-0.226	0.033	-0.029	-0.022	-0.041	0.1267	0.2242	0.124	-0.19	-0.59	-0.04	-0.42	-0.39	-0.08	0.143	0.092	0.177	0.43221	0.147322	-0.00733132	0.154653098
2302	Q02750	MAP2K1	mitogen-activated protein kinase kinase 1	5604	ENSG00000169032	17	-0.3	0.631	-0.36	-0.07	-0.09	-0.34	0.139	0.11	0.304	-0.043	-0.047	0.1354	0.0801	-0.204	0.1262	0.4876	0.2384	0.2552	-0.1	-0.28	-0.29	-0.47	-0.44	0.054	0.004	0.18	0.239	0.34833	0.12717	-0.110298059	0.237467563
1477	P07711	CTSL	cathepsin L	1514	ENSG00000135047	15	-0.05	-1.08	-0.22	-0.53	-0.61	0.281	0.108	0.334	0.204	0.1988	0.4333	0.046	-0.725	0.5212	-0.065	0.1315	-0.454	-0.16	-0.02	0.371	0.22	-0.22	-0.19	-0.34	0.624	-0.04	0.221	0.575969	-0.2449	-0.166616964	-0.078278286
1970	P48444	ARCN1	archain 1	372	ENSG00000095139	23	0.166	0.12	-0.01	-0.16	0.048	-0.42	-0.07	0.157	0.15	0.1264	-0.077	0.0805	0.0591	-0.103	-0.019	0.0723	0.149	0.2049	-0.11	-0.77	-0.05	-0.58	-0.29	0.031	0.04	-0.05	0.029	0.176322	0.191403	-0.056709234	0.248112125
165	A0A0A0MTJ	SPOCK3	SPARC (osteonectin), cwcv and kazal like d	50859	ENSG00000196104	13	-0.68	-0.9	-0.81	-0.4	-0.34	-0.37	-0.27	0.409	-0.52	0.5001	-0.213	0.6992	-0.154	0.605	-0.276	0.4009	-0.431	0.169	0.303	0.555	-0.29	-0.74	-0.72	-0.11	0.924	-0.25	-0.04	0.161533	-0.38935	-0.5748489	0.18549743
1507	P08754	GNAI3	G protein subunit alpha i3	2773	ENSG00000065135	21	0.046	0.288	-0.31	0.352	0.078	-0.31	-0.08	0.069	0.168	-0.246	-0.014	0.2579	0.1642	-0.285	0.3825	0.4366	0.1065	0.3359	-0.43	-0.29	-0.54	-0.56	-0.57	-0.38	-0.05	0.204	0.443	0.14958	0.276185	-0.092546054	0.368731527
301	A0A286YES	IGHG3	immunoglobulin heavy constant gamma 3 (C	3502	ENSG00000211897	11	-1.44	-1.23	-0.49	-1.43	-1.58	-1.38	-0.04	0.448	-0.33	-2.265	-1.959	-1.201	-1.193	-1.771	-1.314	-0.531	-1.261	-0.336	-2.2	-1.41	0.775	3.73	-3.26	0.841	0.365	-0.6	-0.36	0.397352	-0.59498	0.462350289	-1.057332612
302	A0A286YEY	IGHG2	immunoglobulin heavy constant gamma 2 (C	3501	ENSG00000211893	14	-0	-0.23	0.267	-0.75	-0.07	-0.22	-0.46	-0.01	-0.02	-0.022	-0.864	0.4534	-0.387	-0.872	-0.781	-0.508	0.3303	-0.373	-0.79	0.477	0.37	1.08	0.412	-0	0.212	0.61	-1.32	0.375693	-0.28399	0.168397349	-0.452384833
1289	O95460	MATN4	matrilin 4	8785	ENSG00000124159	24	-0.64	-0.29	-0.59	-0.29	-0.62	-0.44	-0.32	-0.14	0.136	0.0115	0.3668	-0.302	-0.818	0.552	-0.394	0.4593	-0.549	0.2846	-0.28	-0.24	-0.22	-0.15	-0.37	0.989	0.084	1.2	-0.38	0.314008	-0.42434	-0.310445812	-0.113893192
2418	Q13557	CAMK2D	calcium/calmodulin dependent protein kinas	817	ENSG00000145349	21	-0.06	-0.01	-0.23	-0.15	-0.07	-0.32	0.095	-0.06	0.008	0.1413	0.1226	-0.049	-0.303	-0.113	0.0833	-0.042	0.0535	0.0229	0.018	-0.6	-0.15	-0.64	-0.12	0.281	0.012	-0.01	0.274	0.734539	0.014508	-0.079098761	0.093607198
2192	P62280	RPS11	ribosomal protein S11	6205	ENSG00000142534	8	-0.01	-0.15	-0.11	-0.13	0.43	-0.26	0.251	-0.02	0.101	-0.196	-0.175	-0.164	-0.015	-0.242	-0.086	-0.006	0.0813	0.0502	-0.26	-0.6	0.078	-0.16	-0.06	0.051	0.369	-0.32	0.12	0.684251	0.097474	0.094043568	0.003430668
3420	Q9H9S4	CAB39L	calcium binding protein 39 like	81617	ENSG00000102547	21	-0.01	0.138	0.046	-0.07	-0.03	-0.45	-0.27	-0.28	-0.07	0.0068	0.1166	0.0359	0.0962	-0.288	-0.017	-0.121	0.0111	-0.052	-0.15	-0.61	-0.25	-0.48	-0.05	-0.07	0.14	-0.11	0.087	0.454818	0.05609	-0.087394228	0.143484311
701	F5GYQ1	ATP6V0D1	ATPase H+ transporting V0 subunit d1	9114	ENSG00000159720	17	-0.09	0.125	0.229	-0.1	-0.22	-0.59	0.128	0.159	0.177	0.1864	0.136	-0.02	0.0406	-0.017	0.0492	0.088	0.2507	0.1872	-0.17	-1.11	-0.26	-0.71	-0.3	0.276	0.009	0.084	0.198	0.239913	0.198272	-0.121094492	0.319366361
2539	Q15819	UBE2V2	ubiquitin conjugating enzyme E2 V2	7336	ENSG00000169139	10	-0	0.104	-0.17	0.189	0.129	-0.54	0.267	0.165	0.2	-0.189	-0.114	0.0269	0.1088	-0.22	0.0772	0.2918	0.1491	0.2197	-0.29	-0.49	0.033	-0.59	-0.47	-0.09	-0.08	0.064	0.352	0.295421	0.210933	-0.001658418	0.212591045
1442	P05783	KRT18	keratin 18	3875	ENSG00000111057	24	0.107	0.109	-0.05	-0.26	-0.03	-0.5	-0.22	-0.06	0.077	0.0657	-0.063	0.1962	-0.036	0.0569	0.0711	-0.118	0.1691	0.0391	0.121	-0.88	-0.09	-0.56	-0.13	0.112	0.172	0.099	0.064	0.496137	0.028744	-0.134280905	0.163024599
3656	Q9ULR3	PPM1H	protein phosphatase, Mg2+/Mn2+ depender	57460	ENSG00000111110	15	-0.02	0.32	-0.07	-0.09	-0.2	-0.4	-0.12	-0.13	0.208	0.0753	0.0596	0.0652	-0.134	-0.126	0.1673	0.1701	0.2028	0.1465	-0.28	-0.66	-0.29	-0.45	-0.3	-0.12	-0.07	0.116	0.106	0.131919	0.159152	-0.125238463	0.284390233
3200	Q99436	PSMB7	proteasome subunit beta 7	5695	ENSG00000136930	10	0.17	0.07	0.098	-0.05	0.47	-0.77	-0.3	-0.33	-0.18	0.3715	0.1053	-0.078	-0.116	-0.27	-0.406	0.2062	0.0703	-0.107	-0.31	-0.09	-0.27	-0.3	-0.15	-0.24	0.275	-0.01	-0.51	0.637874	0.080065	-0.073722231	0.153787427
2892	Q8N271	PROM2	prominin 2	150696	ENSG00000155066	28	-0.3	0.266	0.137	0.07	0.459	0.049	-0.1	0.17	-0.09	-0.526	0.2987	0.3952	-0.149	-0.408	0.0902	0.02	0.1331	0.0707	-0.13	0.119	-0.17	0.014	-0.2	-0.42	-0.46	-0.1	-0.7	0.190068	0.30311	0.082922497	0.220187328
2174	P61927	RPL37	ribosomal protein L37	6167	ENSG00000145592	6	0.064	-0.08	0.016	-0.58	0.719	-0.33	0.275	-0.15	0.24	-0.298	0.0866	-0.351	-0.393	-0.186	-0.025	-0.612	0.7151	-0.021	-0.07	-0.36	-0.07	-0.29	-0.17	-0.11	0.147	-0.38	-0.01	0.641804	0.166035	0.140069541	0.025965565
1651	P17342	NPR3	natriuretic peptide receptor 3	4883	ENSG00000113389	10	0.077	0.23	1.255	-1.44	0.122	-0.61	-0.37	0.414	0.104	0.3925	-0.809	0.9251	0.6008	-1.971	-0.749	-0.154	0.269	0.0298	-0.75	-0.16	0.067	-0.57	-1.57	-0.05	-0.61	-0.57	-0.68	0.464679	0.519639	0.138210823	0.381428246
1346	Q5H9A7	TIMP1	TIMP metallopeptidase inhibitor 1	7076	ENSG00000102265	7	0.261	-0.48	-0.33	-0.01	-0.05	0.308	-0.53	-0	-0.18	0.513	0.3777	0.2562	-0.336	0.1717	-0.391	0.0907	-0.209	-0.324	-0.61	0.16	0.459	-0.05	0.199	0.228	0.454	0.362	-0.78	0.734453	-0.16006	-0.129591638	-0.030463554
3574	Q9P2R3	ANKFY1	ankyrin repeat and FYVE domain containing	51479	ENSG00000185722	26	0.038	-0.07	-0.1	0.053	-0.04	-0.29	-0.03	-0.02	0.124	-0.041	0.1739	0.1754	0.2157	-0.083	0.0076	0.0799	-0.057	0.1687	-0.19	-0.4	-0.12	-0.45	-0.25	0.091	0.063	0.089	0.244	0.247565	0.064365	-0.108631888	0.172996562
1260	O94832	MYO1D	myosin ID	4642	ENSG00000176658	36	0.187	-0.28	-0.26	0.387	0.296	-0.17	-0.46	-0.18	-0.05	-0.168	0.084	0.4291	0.1597	-0.243	0.4039	0.178	-0.058	0.1043	-0.38	0.156	-0.23	-0.33	-0.5	-0.32	-0.3	-0.11	0.195	0.192951	0.143027	-0.157358829	0.300385966
1638	P16278	GLB1	galactosidase beta 1	2720	ENSG00000170266	15	-0.27	-0.62	-0.13	-0.48	-0.27	-0.41	0.126	0.224	0.217	0.1954	-0.035	-0.096	-0.467	0.2916	-0.3	0.2075	-0.315	0.057	0.192	0.775	0.223	0.027	0.011	-0.19	0.551	0.099	-0.05	0.173007	-0.36044	-0.127087355	-0.233354551
2274	P98095	FBLN2	fibulin 2	2199	ENSG00000163520	18	-0.12	-0.65	-1.29	-0.31	-0.1	-0.16	0.249	0.331	-0.56	-0.227	0.1882	-0.43	-0.473	1.0418	-0.455	-0.44	0.293	-0.391	0.124	-0.09	-0.22	0.064	0.29	-0.05	0.207	-0.78	-0.09	0.646993	-0.22926	-0.190868056	-0.038391504
2185	P62195	PSMC5	proteasome 26S subunit, ATPase 5	5705	ENSG00000087191	21	-0.04	-0.05	-0.06	-0.03	0.269	-0.4	0.272	0.096	0.26	0.0137	-0.055	0.0744	0.0117	-0.205	0.0372	0.1154	0.19	0.1952	-0.19	-0.54	-0.13	-0.14	-0.21	-0.06	0.204	-0	0.24	0.449778	0.127045	-0.005615557	0.132660829
586	E5RHG8	ELOC	elongin C	6921	ENSG00000154582	5	0.03	0.112	-0.13	0.03	0.022	-0.31	0.284	-0.18	0.161	-0.168	-0.114	-0.013	-0.078	-0.111	-0.056	-0.088	-0.136	-0.03	-0.32	-1.11	-0.34	-0.18	-0.23	0.018	0.405	0.03	-0.04	0.443147	0.200126	0.090822672	0.10930286
3565	Q9P270	SLAIN2	SLAIN motif family member 2	57606	ENSG00000109171	28	0.012	0.266	-0.24	-0.09	-0.24	-0.37	0.115	-0.09	0.133	0.0662	0.0277	0.0519	-0.086	-0.056	0.1144	0.3197	0.2158	-0.085	-0.36	-0.95	-0.27	-0.65	-0.52	-0.15	-0.01	-0.04	0.408	0.157664	0.224974	-0.118968766	0.343942735
1794	P29966	MARCKS	myristoylated alanine rich protein kinase C s	4082	ENSG00000277443	14	-0.29	-0.25	-0.24	0.287	-0.14	-0.37	0.192	-0.17	-0.14	0.259	-0.291	-0.063	-0.12	-0.408	0.3608	0.2751	0.2659	-0.164	-0.27	-1.04	-0.19	-0.22	-0.37	0.458	-0.09	0.103	0.198	0.625482	0.036275	-0.136312279	0.172587679
1212	O75487	GPC4	glypican 4	2239	ENSG00000076716	24	-0.39	-0.83	0.174	-0.15	-0.35	0.394	-0.6	0.17	-0.49	-0.327	0.5382	0.4259	-0.149	-0.062	0.6952	-0.263	0.2943	-0.419	-0.21	-0.53	0.133	-0.35	-0.67	-0.3	0.002	-0.5	-0.55	0.19955	0.101489	-0.311716091	0.413205011
1714	P22059	OSBP	oxysterol binding protein	5007	ENSG00000110048	25	-0.04	0.165	-0.08	-0.03	-0.05	-0.33	-0.06	-0.08	0.09	0.2045	0.0969	0.0768	0.0241	-0.156	0.235	0.3892	-0.017	-0.058	-0.09	-0.85	-0.26	-0.46	-0.24	0.038	0.238	0.142	0.091	0.2483	0.109638	-0.133580235	0.243218673
1865	P35625	TIMP3	TIMP metallopeptidase inhibitor 3	7078	ENSG00000100234	13	0.154	-0.34	-0.03	-0	-0.42	-0.04	-0.58	0.64	0.365	-0.198	0.1017	0.0216	-0.365	-0.377	-0.062	-0.051	-0.53	-0.33	0.111	0.637	-0.11	0.274	0.005	0.087	0.242	-0.01	-0.04	0.20328	-0.1622	0.169561673	-0.331766351

3068	Q93052	LPP	LIM domain containing preferred translocati	4026	ENSG00000145012	19	0.045	0.135	-0.18	-0.12	-0.13	-0.54	0.069	-0.31	0.009	0.068	-0.099	0.0021	-0.096	-0.078	0.0816	0.0154	-0.031	0.2277	-0.17	-0.65	-0.15	-0.7	-0.32	0.122	0.208	-0	0.077	0.40514	0.061509	-0.122993302	0.184501849
2623	Q5R3I4	TTC38	tetratricopeptide repeat domain 38	55020	ENSG00000075234	17	0.167	0.212	-0.14	-0.03	0.397	-0.5	0.165	-0.14	0.146	-0.084	0.087	0.2115	0.1327	-0.153	0.1107	0.1663	0.1509	0.0714	-0.23	-0.77	-0.23	-0.67	-0.25	0.04	-0.05	0.055	0.31	0.209813	0.228391	-0.046170801	0.274561617
3371	Q9H2K8	TAOK3	TAO kinase 3	51347	ENSG00000135090	36	0.018	0.188	0.061	-0.14	0.117	-0.55	0.025	-0.02	0.381	-0.139	0.0006	0.166	-0.121	-0.215	0.0885	0.1604	0.1412	0.1918	-0.13	-0.49	-0.43	-0.49	-0.38	-0.17	0.234	0.297	0.442	0.577318	0.133298	-0.021329235	0.154627218
58	Q9P2T1	GMPR2	guanosine monophosphate reductase 2	51292	ENSG00000100938	19	0.33	0.404	-0.16	0.03	0	-0.39	0.354	0.426	0.332	-0.111	0.0564	0.2089	-0.025	0.0308	0.2015	0.2737	0.1585	0.1711	-0.23	-1.18	-0.23	-0.61	-0.19	-0.13	0.169	0.214	0.319	0.184642	0.354547	0.04084644	0.313700106
2314	Q04917	YWHAH	tyrosine 3-monooxygenase/tryptophan 5-mo	7533	ENSG00000128245	21	0.051	0.175	-0.05	-0.09	-0.09	-0.37	0.226	-0.01	0.301	0.2179	0.0762	0.2091	-0.025	-0.044	0.1146	0.1686	0.1958	0.1898	-0.22	-1.04	-0.18	-0.45	-0.35	-0.02	0.181	-0.02	0.228	0.150372	0.222017	-0.107593459	0.329610183
1348	P01036	CST4	cystatin S	1472	ENSG00000101441	13	-0.2	-0.76	-0.33	-0.57	-0.9	-0.77	-1.33	0.284	0.307	0.377	-0.469	-0.547	-0.627	-0.523	0.3488	-0.944	-0.425	-1.186	-0.86	2.92	-0.16	-0.01	-0.54	-0.73	-0.99	-0.09	-0.71	0.729529	-0.34468	-0.031919407	-0.312764955
2180	P62070	RRAS2	RAS related 2	22800	ENSG00000133818	17	-0.03	0.359	-0.1	0.313	-0.19	-0.25	-0.07	-0.05	0.221	0.1218	0.1626	0.2148	0.0814	0.0528	0.0613	0.2117	0.2296	0.158	-0.32	-0.63	-0.39	-0.31	-0.43	-0.04	0.082	0.091	0.446	0.146559	0.189546	-0.121010158	0.310555733
1814	P30622	CLIP1	CAP-Gly domain containing linker protein 1	6249	ENSG00000130779	42	-0.04	0.033	-0.15	-0.1	-0.07	-0.44	-0.11	-0.17	-0.06	0.1908	-0.001	-0.012	0.0114	0.0866	0.3642	-0.077	-0.102	-0.197	0.005	-0.54	-0.01	-0.49	-0.36	0.011	-0.07	0.056	0.191	0.310838	0.010726	-0.152509187	0.163235426
3709	Q9Y287	ITM2B	integral membrane protein 2B	9445	ENSG00000136156	17	-0.1	0.223	0.031	-0.42	-0.04	-0.32	0.079	0.464	0.225	0.1468	0.3672	0.1648	-0.202	0.0234	-0.242	0.0002	0.0941	0.328	-0.27	0.064	-0.16	-0.31	-0.67	-0.1	-0.09	0.342	0.062	0.421484	0.139474	-0.060676706	0.200150509
3246	Q9BRA2	TXNDC17	thioredoxin domain containing 17	84817	ENSG00000129235	7	0.126	0.284	0.105	-0.16	-0.16	-0.47	-0.15	-0.12	0.004	0.2573	0.0213	0.1373	-0.015	-0.267	0.2297	-0.08	0.0946	0.0049	-0.05	-0.94	-0.21	-0.69	-0.36	0.424	-0.26	-0.13	0.079	0.269658	0.17634	-0.102964689	0.279304731
3803	R4GNH3	PSMC3	proteasome 26S subunit, ATPase 3	5702	ENSG00000165916	27	0.052	-0	-0.04	-0.02	0.363	-0.47	-0.1	-0.01	0.053	0.1641	-0.036	0.0585	-0.061	-0.182	0.0635	-0.062	0.0944	-0.062	-0.02	-0.3	-0.13	-0.34	-0.23	0.008	0.211	-0.04	-0.07	0.572481	0.082995	-0.015388551	0.098383467
2190	P62269	RPS18	ribosomal protein S18	6222	ENSG00000231500	10	0.105	-0.07	-0.23	-0.13	0.463	-0.11	-0.27	0.217	-0.16	0.0998	-0.044	-0.115	-0.237	-0.314	-0.108	-0.045	-0.504	-0.569	-0.08	0.028	0.201	-0.08	-0.14	-0.08	-0.26	0.064	-0.75	0.456751	0.100184	0.182670358	-0.082486654
3382	Q9H3U1	UNC45A	unc-45 myosin chaperone A	55898	ENSG00000140553	37	0.06	0.176	-0.4	-0.29	-0.33	-0.33	-0.17	-0.07	0.006	0.2077	0.0086	0.1693	-0.053	-0.027	-0.162	-0.033	-0.099	-0.009	-0.28	-0.77	-0.37	-0.42	-0.23	-0.06	0.132	-0.15	-0.1	0.172957	0.09753	-0.15053961	0.248069229
3796	Q9Y6Q5	AP1M2	adaptor related protein complex 1 subunit m	10053	ENSG00000129354	19	0.428	0.291	0.216	0.022	0.063	-0.35	-0.29	-0.25	-0.03	0.3414	0.0998	0.1096	0.0863	-0.072	0.0343	-0.249	0.0652	-0.078	0.083	-0.96	0.249	-0.59	-0.26	0.073	-0.1	-0.1	-0.1	0.352999	0.200777	-0.026046865	0.226823859
1116	O43490	PROM1	prominin 1	8842	ENSG00000007062	25	0.069	0.509	0.512	0.042	-0.53	0.374	-0.7	0.132	-0.05	-0.615	-0.133	-0.779	0.2272	-0.313	0.3329	-0.004	-0.181	0.1929	-0.42	-0.7	0.54	0.595	-0.48	0.053	-0.27	-0.01	0.022	0.755154	0.113686	0.181668973	-0.067983286
1527	P0C0L4	C4A;C4B	complement C4A (Rodgers blood group);co	720;110384	ENSG00000244731	51	-0.78	-1.12	-0.3	-0.54	-0.57	-0.47	-0.69	-0.39	-0.46	-0.511	-0.224	-0.141	-0.382	-0.426	-0.739	-0.536	-0.707	-0.702	-0.59	-0.38	1.693	2.086	-0.04	0.377	0.22	-0.34	-0.88	0.119875	-0.82889	-0.105736963	-0.723156047
51	A0A087WV	KCP	kielin cysteine rich BMP regulator	375616	ENSG00000135253	29	-0.58	-0.35	-0.79	-0.63	-0.04	-0.09	0.15	-0.03	0.107	0.0839	0.0427	-0.014	-0.348	0.7712	-0.04	0.3412	-0.26	0.1064	0.339	0.358	0.29	-0.19	0.04	-0.2	0.612	-0.1	-0.34	0.203218	-0.33938	-0.325026982	-0.014349558
2227	P63010	AP2B1	adaptor related protein complex 2 subunit be	163	ENSG00000006125	43	0.172	0.298	0.133	0.022	0.136	-0.38	-0.12	-0.09	0.071	0.1257	0.0855	0.0335	-0.01	-0.164	0.0215	0.0273	0.1092	0.0072	-0.14	-0.36	-0.06	-0.42	-0.3	-0.05	0.051	-0.02	0.012	0.178464	0.170224	0.000818253	0.169405288
3016	Q8WW22	DNAJA4	DnaJ heat shock protein family (Hsp40) me	55466	ENSG00000140403	16	-0.11	0.007	0.076	0.075	0.113	-0.38	0.211	0.193	0.254	0.3494	0.0021	0.1506	0.0877	-0.021	-0.094	-0.255	0.1002	0.2011	-0.05	-0.65	-0.39	-0.35	-0.24	0.02	-0.13	-0.13	0.114	0.122854	0.250621	-0.008739367	0.259360725
1040	O14657	TOR1B	torsin family 1 member B	27348	ENSG00000136816	12	-0.05	-0.28	-0.44	-0.81	0.138	-0.44	-0.31	-0.11	0.027	0.1794	-0.123	-0.215	-0.25	-0.003	-0.231	0.433	-0.101	-0.061	-0.04	0.594	0.138	-0.19	-0.1	-0.1	0.688	-0.02	-0.18	0.183145	-0.34028	-0.211315119	-0.128968286
438	C9JEN3	TMBIM1	transmembrane BAX inhibitor motif containi	64114	ENSG00000135926	3	-0.03	1.179	-0.11	-0	-0.15	0.029	-0.65	0.537	-0.98	0.2345	0.5209	0.5706	0.2302	-0.053	0.0597	-0.207	-0.27	0.0485	0.012	0.052	-0.39	-0.44	-0.31	-0.09	-0.29	-0.34	0.109	0.451091	0.168063	-0.145600996	0.313663753
1935	P45877	PPIC	peptidylprolyl isomerase C	5480	ENSG00000168938	9	0.049	-1.19	-0.36	0.041	-0.17	0.23	0.195	0.469	0.342	0.0959	0.2527	-0.025	-0.599	0.3721	-0.422	0.6473	-0.517	0.0442	-0.2	0.192	0.343	-0.05	-0.15	-0.16	0.729	0.363	-0.04	0.79024	-0.15734	-0.027332072	-0.130007388
2435	Q13907	IDI1	isopentenyl-diphosphate delta isomerase 1	3422	ENSG00000067064	11	0.349	0.14	0.043	-0	-0.06	-0.48	-0.1	0.208	-0.03	0.1286	0.0546	0.1827	0.009	-0.159	-0.152	0.0855	0.0211	0.0623	-0.24	-1.15	-0.2	-0.57	-0.18	-0.1	0.044	0.121	0.22	0.270099	0.236507	-0.017635096	0.254142391
1867	P35754	GLRX	glutaredoxin	2745	ENSG00000173221	6	-0.41	-0.16	-0.26	-0.16	0.151	-0.57	0.306	0.109	-0.02	0.2584	-0.403	-0.381	-0.414	0.0208	-0.143	0.1273	0.2446	0.1174	0.159	-0.38	0.159	-0.35	-0.16	0.543	-0.24	-0.34	0.304	0.905476	-0.07754	-0.048802289	-0.028741286
2076	P53618	COPB1	coatomer protein complex subunit beta 1	1315	ENSG00000129083	27	0.038	-0.03	-0.11	-0.08	-0.01	-0.29	-0.15	-0.06	0.044	0.1606	-2E-04	0.0887	0.0607	-0.2	-0.045	-0.009	-0.1	0.0943	-0.22	-0.37	-0.12	-0.23	-0.25	0.099	0.326	-0.07	-0.04	0.487387	0.026395	-0.076586533	0.102981816
1960	P47897	QARS1;Q	glutaminyl-tRNA synthetase 1;glutaminyl-tR	5859	ENSG00000172053	29	0.094	0.022	-0	0.181	0.076	-0.25	-0.03	-0.05	0.188	0.0904	0.0969	0.1657	0.1128	-0.253	-0.028	-0.081	0.1959	0.157	-0.19	-0.46	-0.13	0.018	-0.23	0.108	0.345	-0.03	0.105	0.594552	0.076525	-0.025452593	0.101977323
2240	P67775	PPP2CA	protein phosphatase 2 catalytic subunit alph	5515	ENSG00000113575	17	0.121	0.11	-0.1	0.08	0.032	-0.41	0.089	-0.03	0.222	0.0291	0.0288	-0.038	-0.021	-0.133	0.0629	0.2954	0.3165	0.2313	-0.28	-0.84	-0.25	-0.61	-0.41	0.077	-0	0.005	0.237	0.144747	0.242449	-0.072855653	0.315305032
2338	Q08209	PPP3CA	protein phosphatase 3 catalytic subunit alph	5530	ENSG00000138814	18	0.209	0.08	-0.02	0.049	-0	-0.41	0.024	-0.24	0.197	0.0091	0.0654	0.1178	-0.152	-0.244	0.1043	-0.04	0.0174	0.0339	-0.23	-0.57	-0.14	-0.1	-0.32	-0.09	0.202	0.056	-0.01	0.450762	0.120865	-0.003489369	0.124354366
2289	Q01469	FABP5	fatty acid binding protein 5	2171	ENSG00000164687	13	-0.69	-0.23	0.853	0.351	-0.45	-0.41	-0.62	-0.15	-0.6	0.3133	0.2005	0.6166	1.3097	-0.094	-0.339	-0.536	-0.284	-0.486	-0.26	-1.28	-0.57	-0.9	-0.99	0.776	-0.03	0.393	-0.07	0.504908	0.109144	-0.294037718	0.403181612
3124	Q96EK6	GNPNAT1	glucosamine-phosphate N-acetyltransferase	64841	ENSG00000100522	8	0.072	0.07	-0.22	0.153	-0.05	-0.49	-0.13	-0.2	0.244	0.1149	0.1212	-0.026	0.1954	-0.104	-0.034	0.0746	0.1137	0.0588	-0.31	-1.1	-0.39	-0.28	-0.36	0.225	-0.07	-0.05	0.147	0.209808	0.18039	-0.119182513	0.299572667
2446	Q14195	DPYSL3	dihydropyrimidinase like 3	1809	ENSG00000113657	26	0.387	0.369	-0.1	-0.09	0.088	-0.53	0.138	0.125	0.193	-0.051	-0.034	0.0271	-0.039	-0.051	0.0894	0.2066	0.16	0.0144	-0.28	-0.72	-0.09	-0.51	-0.46	-0.21	0.203	0.078	0.273	0.233934	0.253032	0.027972706	0.225059603
1373	P01834	IGKC	immunoglobulin kappa constant	3514	ENSG00000211592	10	-0.32	-0.58	-0.11	-0.65	-0.81	-0.6	-0.71	-0.35	-0.73	-0.18	-0.496	-0.009	-0.55	-0.789	-0.929	-0.193	-0.437	-0.564	-0.76	-0.31	1.673	2.386	0.281	0.611	-0.15	-0.14	-1.32	0.171861	-0.79385	-0.080256907	-0.713594586
1711	P21964	COMT	catechol-O-methyltransferase	1312	ENSG00000093010	20	0.457	0.312	0.009	0.17	-0.24	-0.14	-0.49	-0.33	0.12	0.2319	0.0884	0.1639	0.2211	-0.213	-0.322	-0.079	0.2154	-0.176	-0.11	-1.38	-0.2	-0.17	-0.24	0.1	-0.41	-0.64	-0.51	0.133266	0.382226	-0.029422655	0.411648177
918	J3KQ32	OLA1	Obg like ATPase 1	29789	ENSG00000138430	14	0.019	0.196	0.055	-0.16	-0.07	-0.42	0.114	0.043	0.26	0.0349	0.0714	0.0603	-0.029	-0.027	0.0453	0.1053	0.2201	0.0236	-0.22	-1	-0.23	-0.55	-0.3	0.056	-0.04	0.116	0.214	0.202978	0.220857	-0.052186111	0.273042743
267	O75787	ATP6AP2	ATPase H+ transporting accessory protein	10159	ENSG00000182220	10	-0.1	-0.41	-0.86	-0.43	0.069	-0.45	-0.16	0.144	0.151	-0.221	-0.403	-0.367	-0.243	0.2746	-0.196	0.4254	0.4074	0.2985	-0.34	0.081	0.322	-0.71	-0.81	-0.3	0.123	-0.13	0.184	0.549525	-0.05216	-0.223926856	0.171762558
2325	Q06278	AOX1	aldehyde oxidase 1	316	ENSG00000138356	29	0.221	0.417	0.246	-0.29	0.24	-0.54	0.203	0.313	-0.19	0.1522	-0.339	-0.226	-0.269	-0.286	-0.083	-0.232	0.3393	-0.139	-0.2	-0.19	0.181	-0.1	-0.16	0.276	0.169	-0.34	0.132	0.477547	0.096089	0.18974442	-0.093655061
1858	P35443	THBS4	thrombospondin 4	7060	ENSG00000113296	24	-0.6	-0.58	-0.15	-0.96	-0.25	-1.24	-0.58	0.322	0.413	0.4465	-0.461	-0.352	-1.165	0.103	-0.458	-0.507	1.2974	0.3012	-0.71	2.06	-0.28	-1.24	-1.08	-0.95	0.213	-0.38	-0.58	0.773016	-0.07667	-0.314475543	0.237801847
1613	P14735	IDE	insulin degrading enzyme	3416	ENSG00000119912	29	0.127	0.122	-0.03	0.01	0.135	-0.4	0.01	0.071	0.163	0.1461	-0.02	0.0338	0.0866	-0.164	0.045	0.1304	0.1934	0.1877	-0.19	-0.62	-0.05	-0.31	-0.25	0.054	0.167	0.156	0.155	0.342733	0.12089	-0.047753598	0.168643515
1176	O60763	USO1	USO1 vesicle transport factor	8615	ENSG00000138768	24	-0.06	0.066	-0.07	0.054	0.176	-0.26	0.19	0.224	0.299	0.0675	-0.162	0.0158	0.0242	-0.149	0.0069	0.1688	0.1433	0.2115	-0.3	-0.14	0.001	-0.09	-0.24	0.025	0.32	0.181	0.222	0.790339	0.070405	0.032225644	0.038178969
3562	Q9P206	KIAA1522	KIAA1522	57648	ENSG00000162522	19	0.298	0.171	0.228	-0.21	-0.03	-0.23	-0	-0.23	0.179	-0.056	0.0178	0.053	0.0824	-0.235	0.0106	0.1422	0.0795	0.1479	-0.27	-0.35	-0.07	-0.33	-0.21	-0.03	-0.09	-0.01	-0.01	0.169627	0.169364	-0.008933713	0.178297869
3033	Q92538	GBF1	golgi brefeldin A resistant guanine nucleotid	8729	ENSG00000107862	44	0.36	0.272	-0.07	-0.06	-0.08	-0.29	-0.16	0.111	0.046	0.0025	0.1918	0.0888	0.1273	-0.185	0.0177	0.0968	0.102	0.1458	-0.27	-0.94	-0.13	-0.33	-0.13	0.048	0.084	-0.11	0.119	0.204464	0.199537	-0.050788725	0.250325994
3161	Q96KP1	EXOC2	exocyst complex component 2	55770	ENSG00000112685	30	-0.03	0.192	-0.13	0.11	0.138	-0.35	0.013	0.016	0.179	-0.087	0.0355	0.1841	0.242	-0.031	0.0862	0.1019	0.2077	0.2682	-0.29	-0.68	-0.3	0.151	-0.3	-0.03	0.102	0.169	0.414	0.362942	0.0997	-0.097001664	0.196701413
1891	P39060	COL18A1	collagen type XVIII alpha 1 chain	80781	ENSG00000182871	18	0.04	-0.83	0.117	-0.1	-0.25	0.406	-0.55	0.632	-0.3	-0.572	0.4518	0.6615	-0.059	0.4913	0.3529	-0.07	-0.361	-0.403	-0.18	0.037	-0.1	-0.7	-0.42	-0.64	-0.23	-0.44	-0.73	0.224858	0.284933	-0.148208073	0.433141017
576	D6RHI9	RNASET2	ribonuclease T2	8635	ENSG00000026297	11	-0.12	-0.25	-0.25	-0.37	-0.28	0.289	0.148	0.251	0.487	0.6879	-0.437	0.4203	-0.365	-0.318	-0.73	0.4362	-0.326	-0.662	-0	-0.34	-0.07	0.02	0.438	-0.5	0.116	0.494	-0.9	0.873267	0.072826	0.133386945	-0.060560619
2162	P61254	RPL26	ribosomal protein L26	6154	ENSG00000161970	9	0.029	-0.33	-0.08	-0.04	0.348	-0.1	0.423	0.221	0.447	-0.259	-0.016	-0.096	-0.037	-0.151	0.1139	0.0321	0.34	0.2494	-0.24	-0.32	-0.03	-0.27	-0.15	-0.01	0.243	0.298	0.397	0.725364	0.1107	0.082439465	0.028260861
2423	Q13596	SNX1	sorting nexin 1	6642	ENSG00000028528	23	0.038	0.09	-0.15	-0.12	-0.03	-0.39	-0.08	-0	0.227	0.0619	-0.05	0.1179	-0.093	0.029	0.1609	0.1107	0.2291	0.1386	-0.15	-0.64	0.048	-0.38	-0.19	-0	0.097	0.062	0.141	0.258017	0.066752	-0.123496683	0.190248324
2287	Q01415	GALK2	galactokinase 2	2585	ENSG00000156958	16	0.17	0.073	-0.18	0.307	-0.15	-0.15	0.119	-0.12	0.107	0.263	0.0712	0.017	0.0963	0.1463	0.0601	0.2516	0.2189	0.2285	-0.26	-0.66	-0.09	-0.57	-0.39	-0.15	0.162	0.019	0.469	0.146765	0.180997	-0.131821502	0.31281892
2237	P63244	RACK1	receptor for activated C kinase 1	10399	ENSG00000204628	19	0.067	-0.18	-0.08	0.077	0.273	-0.23	0.017	-0.16	0.16	-0.205	0.0429	-0.158	-0.004	-0.126	-0.095	-0.082	0.1446	0.1813	-0.22	-0.85	-0.12	-0.39	-0.16	0.083	0.092	-0.24	0.072	0.301898	0.188266	0.028537483	0.159728974
7	A0A024RA5	PSMA2;AC	proteasome subunit alpha 2;novel PSMA2 a	5683	ENSG00000106588	15	-0.18	-0.05	-0.03	-0.18	0.332	-0.57	0.002	-0.24	0.026	0.1274	-0.158	-0.019	0.0245	-0.208	-0.425	0.3794	0.222	0.0025	-0.24	0.007	-0.07	0.081	-0.03	-0.1	0.415	0.097	-0.28	0.749987	-0.08447	-0.092517206	0.008050474
2556	Q16543	CDC37	cell division cycle 37	11140	ENSG00000105401	20	0.002	0.105	0.049	0.014	0.306	-0.43	0.062	0.074	0.236	-0.152	-0.021	-0.037	0.0063	-0.18	0.0909	0.1166	0.2934	0.2136	-0.29	-1.09	-0.24	-0.61	-0.35	0.047	0.053	-0.04	0.279	0.189862	0.294469	0.009546464	0.284922702
2457	Q14376	GALE	UDP-galactose-4-epimerase	2582	ENSG00000117308	17	0.07	0.271	-0.03	0.015	-0.05	-0.59	-0.09	-0.08	0.139	-0.054	-0.037	0.2004	-0.07	-0.17	-0.073	-0.087	0.1355	0.1291	-0.36	-0.81	-0.33	-0.63	-0.45	0.094	-0.03	0.131	0.233	0.293369	0.198882	-0.036554411	0.235436188
1266	O94973	AP2A2	adaptor related protein complex 2 subunit al	161	ENSG00000281385	30	-0	0.017	-0.1	0.112	0.199	-0.1	-0.07	-0.06	0.151	0.1348	-0.024	0.1651	0.0092	-0.192	-0.025	0.1228	0.1011	0.2018	-0.09	-0.44	-0.09	-0.33	-0.08	-0.12	0.05	0.022	0.166	0.209848	0.118866	-0.038759584	0.157625326
1209	O75410	TACC1	transforming acidic coiled-coil containing pr	6867	ENSG00000147526	27	0.318	0.442	-0.29	-0.05	-0.69	-0.58	0.083	-0.16	0.192	-0.196	0.1388	-0.109	-0.107	-0.338	-0.014	0.5748	0.1836	-0.08	-0.17	-1.12	0.127	-0.58	-0.37	0.063	-0.3	0.102	0.659	0.741246	0.092998	-0.087471301	0.180469521
2028	P50851	LRBA	LPS responsive beige-like anchor protein	987	ENSG00000198589	40	-0.17	0.07	-0.03	0.051	0.039	-0.32	0.077	-0.14	-0.05	0.4042	0.1852	0.1292	0.0007	-0.116	-0.031	-0.072	0.1687	-0.101	-0.07	-0.26	-0.18	-0.23	-0.21	0.222	0.102	0.041	0.121	0.396452	-0.00134	-0.116853457	0.115513296
2487	Q15008	PSMD6	proteasome 26S subunit, non-ATPase 6	9861	ENSG00000163636	21	0.09	-0	-0.07	0.106	0.362	-0.34	-0.18	-0.13	0.045	0.061	-0.04	-0.031	-0.024	-0.117	0.0846	-0.187	0.0779	-0.065	-0.1	-0.52	-0.07	-0.19	-0.27	0.027	0.219	-0.04	-0.06	0.575589	0.099352	0.013918098	0.085433976
1912	P41250	GARS;GA	glycyl-tRNA synthetase;glycyl-tRNA synthet	2617	ENSG00000106105	21	0.027	-0.16	-0.13	0.031	0.252	-0.34	-0.02	0.19	0.153	0.1393	-0.156	-0.126	-0.116	-0.156	0.0538	-0.019	-0.114	-0.046	-0.25	-0.6	-0.12	-0.29	-0.2	-0.13	0.019	-0.09	0.023	0.211231	0.182467	0.060012676	0.122454648
1769	X1WI28	RPL10	ribosomal protein L10	6134	ENSG00000147403	12	-0.1	-0.21	-0.25	0.125	0.397	-0.22	0.193	0.095	0.317	-0.377	-0.346	-0.298	0.0782	-0.182	-0.185	-0.063	0.0895	0.2233	-0.49	-0.77	-0.26	-0.02	0.047	0.082	0.249	-0.15	0.243	0.532459	0.157442	0.155627502	0.001814664
2164	P61313	RPL15	ribosomal protein L15	6138	ENSG00000174748	9	0.091	-0.05	-0.06	0.29	0.59	-0.11	-0.11	-0.23	-0.05	-0.037	-0.048	-0.117	-0.178	-0.059	-0.049	0.0379	0.1756	0.0628	-0.1	-0.56	0.052	-0.33	-0.28	-0.03	0.237	-0.3	-0.09	0.303595	0.195403	0.063873978	0.131528887
3576	Q9UBB4	ATXN10	ataxin 10	25814	ENSG00000130638	20	0.014	0.185	-0.03	-0.1	-0.19	-0.28	-0.37	-0.32	-0.08	0.0498	0.1307	0.0909	-0.006	-0.167	-0.07	-0.015	0.1697	0.0789	-0.27	-0.89	0.416	-0.29	-0.15	0.022	-0.25	-0.19	0.108	0.352932	0.035322	-0.160682393	0.196004733
583	E5RGN3	ATOX1	antioxidant 1 copper chaperone	475	ENSG00000177556	3	-0.1	-0.06	-0.2	-0.01	0.078	-0.18	0.607	0.064	0.37	-0.283	-0.346	-0.127	0.2977	-0.113	0.3379	0.4087	0.3436	0.0882	-0.29	-0.9	-0.28	-0.5	-0.35	0.341	-0.03	0.074	0.623	0.501302	0.20851	-0.00505296	0.213563075
2767	Q7L5Y1	ENOSF1	enolase superfamily member 1	55556	ENSG00000132199	17	-0.14	-0.73	0.595	0.409	0.259	-0.51	-0.3	0.12	0.092	0.1899	-0.582	0.4848	-0.188	0.1151	0.3198	0.3632	-0.12	-0.246	-0.07	-0.8	-0.57	-0.45	-0.4	-0.27	0.555	-0.19	0.35	0.574248	0.182924	-0.059210379	0.242134132
1746	P25325	MPST	mercaptopyruvate sulfurtransferase	4357	ENSG00000128309	17	0.102	0.321	0.035	-0.14	0.035	-0.42	0.141	-0.17	0.259	0.1695	0.0603	0.0275	-0.137	-0.134	0.1159	0.1369	0.3074	0.0601	-0.05	-0.98	-0.25	-0.76	-0.43	-0.13	0.146	0.053	0.259	0.205002	0.256467	-0.04961112	0.306077728
2186	P62241	RPS8	ribosomal protein S8	6202	ENSG00000142937	14	-0.06	-0.2	-0.03	0.114	0.636	-0.14	0.276	-0.15	0.11	-0.194	-0.122	-0.281	-0.127	0.0475	0.0179	-0.315	0.3857	-0.032	-0.07	-0.38	-0.11	-0.18	-0.03	0.01	0.24	-0.39	0.059	0.463875	0.157481	0.131335078	0.026145736
1780	P28161	GSTM2	glutathione S-transferase mu 2	2946	ENSG00000213366	22	-0.18	0.797	-0.14	-0.35	0.571	0.078	-0.14	-0.41	1.009	-0.135	0.0247	-0.308	-0.147	0.0308	-0.024	-0.699	0.3012	0.0339	0.221	-0.13	0.495	-0.9	-0.46	-0.07	0.01	-0.02	-0.63	0.450638	0.302347	0.239965251	0.06238192
2957	Q8NHG7	SVIP	small VCP interacting protein	258010	ENSG00000198168	5	-0.42	0.066	0.128	0.064	0.048	-0.57	-0.82	-0.51	-0.54	-0.161	-0.054	-0.146	0.2668	-0.333	0.0091	-0.308	-0.028	-0.253	-0.69	-0.71	-0.35	-0.29	-0.44	-0.12	-0.67	-0.2	-0.22	0.215118	0.126743	-0.171760533	0.298503922
1044	O14744	PRMT5	protein arginine methyltransferase 5	10419	ENSG00000100462	18	0.062	0.057	-0.03	0.068	0.37	-0.41	0.033	-0.06	0.09	0.171	-0.037	-0.015	-0.052	-0.173	-0.067	0.0303	-0.105	0.0352	-0.25	-0.61	-0.19	-0.1	-0.3	0.06	-0	-0.1	-0.07	0.19519	0.194126	0.044339098	0.149786953
1990	P49354	FNTA	farnesyltransferase, CAAX box, alpha	2339	ENSG00000168522	17	0.199	0.111	-0.07	-0.05	0.112	-0.47	-0.03	-0.02	0.11	-0.035	0.0413	0.0036	-0.001	-0.109	0.0254	0.0911	0.2312	0.1965	-0.12	-1.1	-0.21	-0.43	-0.36	-0.09	0.009	0.057	0.186	0.205476	0.215125	-0.062271956	0.277397036
277	Q7Z4S6	KIF21A	kinesin family member 21A	55605	ENSG00000139116	24	0.207	-0.04	-0.06	-0.14	-0.38	-0.12	0.071	-0.02	0.193	0.1352	0.1399	0.0098	-0.13	-0.162	0.2082	0.0422	0.1203	-0.079	-0.25	-0.74	-0.03	0.697	0.071	0.143	-0.08	-0.13	0.278	0.918519	-0.02669	-0.062546917	0.035856249
2	A0A024R44	DNPEP	aspartyl aminopeptidase	23549	ENSG00000123992	12	0.087	-0.2	-0.07	0.108	-0	-0.31	-0.11	-0.08	-0.1	-0.018	0.1136	0.1549	0.0473	0.0072	-0.152	0.0168	0.253	0.0728	-0.29	-0.34	-0.27	-0.26	-0.04	-0.05	0.012	0.072	0.046	0.145033	0.049059	-0.130735441	0.179794185
1255	X6R3P0	SLIT2	slit guidance ligand 2	9353	ENSG00000145147	35	-0.41	-0.31	-0.08	-0.45	-0.43	0.208	-0.04	-0.12	0.141	0.0462	0.4533	0.3284	-0.625	0.385	-0.198	-0.142	0.3054	0.2576	-0.34	-0.44	-0.32	-0.45	-0.02	-0.07	0.835	0.08	-0.08	0.434752	-0.07801	-0.256838399	0.178831042
2251	P68431	HIST1H3A	histone cluster 1 H3 family member a;H3 clu	8350;8358;8	ENSG00000275714	9	-1.84	-1.76	-1.6	-0.42	-0.57	0.864	0.29	-0.48	0.052	-3.667	-1.197	-2.449	-1.986	-1.239	-2.092	-0.752	-1.786	-2.212	-1.88	-1.53	2.902	2.395	-1.23	1.141	-1.57	-2.05	-2.69	0.209698	-0.10568	1.32503875	-1.430720131
791	G5E9Q6	PFN2	profilin 2	5217	ENSG00000070087	9	0.473	0.023	-0.15	0.031	-0.24	-0.66	-0.17	0.056	0.189	-0.297	-0.118	0.0237	-0.073	-0.257	0.2665	-0.188	0.241	0.2327	-0.55	-1.38	-0.42	-0.83	-0.23	-0.04	-0.31	-0.11	0.107	0.156382	0.368862	-0.031385948	0.400247902
3736	Q9Y3A5	SBDS	SBDS ribosome maturation factor;SBDS, ri	51119	ENSG00000126524	14	-0.01	0.14	-0.04	0.163	0.253	-0.3	-0.07	-0.16	0.018	0.0934	0.0648	0.0323	0.1004	-0.207	0.0159	-0.183	0.1126	-0.2	-0.3	-1.05	-0.35	-0.67	-0.43	-0.11	-0.25	-0.11	-0.02	0.053043	0.365619	0.018064301	0.347555186
2479	Q14764	MVP	major vault protein	9961	ENSG00000013364	31	-0.48	0.24	-0.13	-0.16	-0.02	-0.25	-0.07	-0.24	-0.12	-0.168	0.0466	-0.148	-0.026	-0.088	-0.203	0.0136	0.1063	0.0919	-0.16	0.474	0.572	0.832	-0.17	0.075	0.244	-0.13	-0.2	0.172853	-0.30824	-0.095695228	-0.212540312
2125	P58546	MTPN	myotrophin	136319	ENSG00000105887	9	-0.2	-0.17	-0.17	-0.12	-0.08	-0.54	0.188	0.169	0.365	-0.13	-0.602	-0.135	-0.096	-0.153	0.2817	0.2619	0.2525	0.384	-0.17	-0.87	-0.29	-0.63	-0.44	0.061	0.13	0.21	0.244	0.571799	0.133423	-0.069348767	0.202772107
650	E9PGT1	TSN	translin	7247	ENSG00000211460	11	0.185	-0.15	-0.01	0.558	0.779	-0.26	0.138	0.322	0.358	-0.024	-0.128	-0.168	-0.041	-0.276	-0.158	-0.126	-0.034	-0.112	-0.32	-0.53	-0.13	-0.3	-0.28	-0.17	0.327	-0.09	0.199	0.097889	0.356008	0.332135429	0.023872967
1901	P40879	SLC26A3	solute carrier family 26 member 3	1811	ENSG00000091138	18	-0.13	0.242	-0.2	-1.36	-0.51	0.676	-0.08	-0.12	0.277	0.0596	-0.558	-0.541	-0.422	-0.221	-0.009	0.0689	-0.033	0.2955	0.102	1.347	-0.26	0.239	0.196	-0.35	-0.23	-0.01	0.229	0.511358	-0.27339	0.017745317	-0.291130588
1170	O60664	PLIN3	perilipin 3	10226	ENSG00000105355	20	0.066	0.171	-0.12	-0.05	0.029	-0.52	0.087	-0	0.214	-0.102	0.0114	0.0143	-0.12	-0.234	0.2125	0.0626	0.3018	0.0879	-0.14	-1.21	-0.36	-0.69	-0.57	-0.15	-0.03	0.049	0.25	0.172751	0.30382	-0.039501093	0.343320731
3147	Q96IJ6	GMPPA	GDP-mannose pyrophosphorylase A	29926	ENSG00000144591	15	0.007	-0.11	-0.14	-0.08	-0.08	-0.24	-0.11	-0.04	0.099	0.0347	-0.068	-0.022	-0.03	-0.152	0.1467	0.301	0.1268	0.1669	-0.32	-0.63	-0.02	-0.15	-0.31	-0.2	0.275	0.168	0.122	0.303046	0.039918	-0.134354509	0.174272446

1776	P28066	PSMA5	proteasome subunit alpha 5	5686	ENSG00000143106	11	-0.17	0.005	-0.04	-0.19	-0.11	-0.26	-0.23	0.319	0.319	0.1627	-0.103	-0.031	-0.184	-0.025	-0.033	0.1961	0.0839	-0.13	-0.31	-0.12	-0.26	-0.23	-0.16	-0.21	0.704	0.087	0.189	0.968265	-0.00506	-0.032677871	0.0276193
635	E9PDL4	ICA1	islet cell autoantigen 1	3382	ENSG00000003147	22	0.267	0.051	0.058	-0.11	-0	-0.35	-0.04	-0.07	0.096	0.0167	0.2258	0.0675	-0.034	-0.044	0.2442	0.282	0.2097	0.1724	-0.07	-0.94	-0.25	-0.6	-0.34	-0.16	0.138	-0.07	0.219	0.118425	0.22135	-0.137158019	0.358507984
2792	Q7Z6K5	ARPIN	actin related protein 2/3 complex inhibitor	348110	ENSG00000242498	15	-0.07	0.081	-0.11	-0.13	-0.16	-0.16	0.137	0.236	0.358	0.0163	0.1456	-0.012	-0.105	0.0862	0.1141	0.2608	0.1009	0.0447	-0.19	-0.71	-0.26	-0.67	-0.4	0.057	0.3	0.199	0.169	0.270602	0.185984	-0.052902782	0.238886684
1628	P15374	UCHL3	ubiquitin C-terminal hydrolase L3	7347	ENSG00000118939	11	0.226	0.215	0.005	0.237	0.142	-0.23	0.174	0.142	0.159	0.1322	0.039	0.0058	0.0389	-0.073	0.0986	0.1091	0.1988	0.0936	-0.14	-1.13	-0.31	-0.69	-0.35	-0.01	-0.11	-0.06	0.308	0.083819	0.397672	0.047882154	0.349790249
3690	Q9UQ80	PA2G4	proliferation-associated 2G4	5036	ENSG00000170515	18	0.074	-0.07	-0	0.095	0.215	-0.23	0.147	0.2	0.323	0.1734	0.0536	-0.088	0.0599	-0.097	0.0454	-0.129	0.1668	0.0667	-0.2	-0.56	-0	-0.44	-0.23	0.121	0.094	1E-04	0.198	0.236616	0.195869	0.055156544	0.140712206
2106	P55060	CSE1L	chromosome segregation 1 like	1434	ENSG00000124207	21	-0.04	0.047	-0.07	0.138	0.153	-0.28	0.013	0.099	0.117	0.0437	-0.105	-0.142	0.0085	-0.198	-0.04	-0.029	0.0175	-0.009	-0.39	-0.56	5E-04	-0.13	-0.27	0.043	0.065	-0.03	0.078	0.309469	0.151093	0.069174348	0.081918376
1903	P41091	EIF2S3	eukaryotic translation initiation factor 2 subu	1968	ENSG00000130741	23	0.158	0.057	-0.06	-0.15	0.081	-0.36	0.017	-0	0.191	0.0109	-0.006	0.0569	0.0149	-0.019	-0.022	0.0505	0.0831	0.0704	-0.12	-0.75	-0.08	-0.28	-0.35	0.047	0.226	-0.02	-0.01	0.293429	0.140277	-0.0345832	0.174859707
1038	O14638	ENPP3	ectonucleotide pyrophosphatase/phosphodie	5169	ENSG00000154269	28	-0.19	-0	0.017	-0.7	0.122	0.664	0.194	0.092	0.063	-0.323	0.15	-0.242	-0.245	0.0303	-0.183	-0.16	-0.013	0.2194	-0.15	0.636	-0.04	-0.13	0.046	-0.23	0.39	-0.29	0.048	0.740319	-0.00236	0.113900163	-0.116255854
3008	Q8WUW1	BRK1	BRICK1 subunit of SCAR/WAVE actin nucl	55845	ENSG00000254999	9	0.093	0.155	0.076	-0.05	-0.07	-0.42	0.176	0.148	0.331	-0.118	0.0097	0.1568	-0.05	-0.04	0.1026	0.16	0.1129	0.1256	-0.24	-0.64	-0.09	-0.18	-0.33	-0.1	0.042	0.009	0.114	0.166021	0.206673	-0.001212718	0.207885599
3502	Q9NSB8	HOMER2	homer scaffold protein 2	9455	ENSG00000103942	19	0.335	0.432	-0.23	0.121	0.16	-0.52	-0.01	0.01	0.11	-0.148	0.3318	0.2518	0.0724	-0.037	0.1769	0.2574	0.2804	0.3536	0.072	-0.02	0.061	-0.76	-0.42	-0.54	0.025	0.118	0.41	0.27228	0.164123	-0.125103386	0.289225934
3034	Q92542	NCSTN	nicastrin	23385	ENSG00000162736	12	0.099	0.483	0.151	0.152	-0.11	-0.37	-0.35	-0.12	-0.13	0.1531	0.1913	0.2774	0.4244	-0.304	-0.006	0.06	0.2532	0.2456	-0.45	-0.65	-0.22	-0.57	-0.37	0.106	-0.35	-0.18	0.153	0.082125	0.259056	-0.165365959	0.424421962
3630	Q9UIK5	TMEFF2	transmembrane protein with EGF like and tv	23671	ENSG00000144339	9	-0	-1.13	-0.61	0.357	-0.01	0.642	-0.24	0.296	-0.03	0.0042	-0.001	1.263	-0.794	0.363	-0.849	-0.126	-0.639	-0.215	0.173	-0.49	-0.11	-1.5	-1.37	-0.47	-0.05	0.051	0.261	0.637729	0.308832	0.030520721	0.27831105
1471	Q5JP53	TUBB	tubulin beta class I	203068	ENSG00000183311	26	0.163	0.282	-0.19	0.101	-0.2	-0.43	0.511	0.342	0.436	0.1263	0.0221	0.1988	-0.143	-0.053	0.1852	0.2727	0.1857	0.5153	-0.4	-0.8	0.05	-0.11	-0.41	-0.11	-0.04	0.1	0.843	0.435141	0.209722	-0.032715616	0.242437711
1099	O15511	ARPC5	actin related protein 2/3 complex subunit 5	10092	ENSG00000162704	9	-0.04	-0.07	-0.17	-0.19	-0.2	-0.21	0.161	0.055	0.116	-0.105	-0.168	-0.086	-0.298	0.0879	0.0015	0.1148	-0.072	0.0198	-0.33	-0.76	0.183	0.169	-0.15	-0.13	0.147	0.078	0.133	0.988561	0.011719	-0.005967319	0.017686244
2784	Q7Z406	MYH14	myosin heavy chain 14	79784	ENSG00000105357	65	-0.19	0.031	-0.19	-0.13	0.067	-0.02	-0.1	-0.19	0.112	0.1702	-0.109	-0.013	0.1541	0.058	0.0347	-0.008	0.1782	-0.044	-0.16	-0.61	-0.06	-0.29	-0.2	0.168	0.257	-0.1	-0.02	0.302152	0.043984	-0.115317212	0.159301512
3551	Q9NZL9	MAT2B	methionine adenosyltransferase 2B	27430	ENSG00000038274	10	-0	-0.03	-0.17	-0.01	-0.07	-0.56	-0.19	-0.25	-0.15	-0.081	-0.095	-0.02	-0.035	-0.465	-0.37	0.323	0.0217	-0.056	-0.06	-0.53	-0.14	-0.62	-0.53	-0.13	0.26	0.158	-0.2	0.727332	0.04162	-0.070717526	0.112337625
2574	Q32MZ4	LRRFIP1	LRR binding FLII interacting protein 1	9208	ENSG00000124831	16	-0.01	0.101	0.009	-0.34	-0.18	-0.43	0.333	0.156	0.189	0.0235	-0.006	0.0465	-0.254	-0.059	0.1962	0.1567	0.2738	0.105	-0.26	-0.64	-0.23	-0.61	-0.3	-0.05	0.326	0.106	0.369	0.47231	0.124594	-0.07169927	0.196293113
2712	Q6PGP7	TTC37	tetratricopeptide repeat domain 37	9652	ENSG00000198677	36	0.044	-0.05	-0.07	-0.13	0.095	-0.55	-0.05	0.102	0.165	0.0147	0.0024	0.1606	0.077	-0.189	-0.011	0.0072	0.164	0.1636	-0.18	-0.56	-0.1	-0.32	-0.25	0.145	0.157	0.045	0.24	0.525811	0.043349	-0.092517202	0.135866177
3152	Q96J02	ITCH	itchy E3 ubiquitin protein ligase	83737	ENSG00000078747	34	0.137	0.235	-0.13	0.112	0.214	-0.34	-0.33	0.095	-0.18	-0.254	0.0424	0.0624	0.1143	-0.184	0.0522	0.0801	-0.063	0.2376	-0.43	-0.57	-0.31	-0.45	-0.26	-0.08	-0.01	0.054	0.267	0.270061	0.17784	-0.030510663	0.20835116
1234	O75884	RBBP9	RB binding protein 9, serine hydrolase	10741	ENSG00000089050	9	0.024	-0.2	0.206	-0.17	-0.19	-0.34	0.053	0.025	0.286	0.0467	0.1127	-0.089	-0.056	-0.322	0.3304	0.0256	0.2261	0.2065	0.277	-0.97	-0.08	-0.73	-0.45	0.017	0.015	-0.02	0.304	0.430599	0.147138	-0.08718727	0.234325311
2877	Q8IZJ3	CPAMD8	C3 and PZP like alpha-2-macroglobulin dom	27151	ENSG00000160111	32	-0.23	-0.16	0.185	-0.15	-0.69	-0.35	0.083	-0.1	0.089	-0.277	0.062	0.5905	-0.49	0.4272	-0.143	0.278	-0.132	0.0055	-0.4	-0.43	0.208	-0.17	-0.21	0.013	0.338	0.186	-0.21	0.581273	-0.07093	-0.181279392	0.110351739
1098	O15498	YKT6	YKT6 v-SNARE homolog	10652	ENSG00000106636	11	0.186	0.407	0.149	0.073	0.048	-0.37	-0.07	-0.1	0.087	0.2174	0.2534	0.1905	0.1793	-0.248	0.2236	0.0118	0.2823	0.0724	-0.36	-1.02	-0.14	-0.49	-0.27	0.201	-0.23	-0.07	0.176	0.118143	0.288576	-0.086075731	0.374651677
2064	P52888	AC006538	thimet oligopeptidase 1	7064	ENSG00000172009	19	0.064	0.095	-0.04	0.031	-0.04	-0.32	0.05	-0.02	0.032	0.1437	0.0294	0.0591	-0.086	-0.113	0.114	-0.08	0.0629	-0.042	-0.02	-0.69	-0.23	-0.29	-0.1	-0.05	0.001	0.024	0.017	0.242543	0.131406	-0.025972803	0.157378801
898	I3L0H8	DDX19A	DEAD-box helicase 19A	55308	ENSG00000168872	21	0.072	0.218	0.041	-0.04	-0.16	-0.37	0.075	-0.02	0.144	0.1041	0.0742	-0.011	0.1226	-0.178	0.1028	0.0669	0.2111	0.1602	-0.19	-0.78	-0.15	-0.43	-0.27	0.002	0.06	-0.04	0.263	0.198722	0.166265	-0.079031448	0.245296879
2218	P62906	RPL10A	ribosomal protein L10a	4736	ENSG00000198755	9	0.165	-0.16	-0.03	0.078	0.179	-0.17	0.273	0.257	0.196	-0.176	-0.097	-0.176	-0.17	-0.29	0.0721	-0.021	0.2488	0.3099	-0.12	-0.25	0.143	-0.14	-0.18	-0.03	0.35	-0.15	0.249	0.535579	0.100547	0.121431305	-0.020884427
1072	O15126	SCAMP1	secretory carrier membrane protein 1	9522	ENSG00000085365	12	-0.29	0.206	0.133	0.012	-0.31	-0.39	-0.03	-0.06	0.084	0.3376	0.073	-0.008	0.0286	-0.325	0.1771	0.0969	0.1864	0.0817	-0.15	-0.43	-0.4	-0.6	-0.45	0.076	0.046	0.04	0.034	0.172247	0.130839	-0.143776264	0.274615063
1528	Q99878	HIST1H2A	histone cluster 1 H2A family member j;H2A	8331	ENSG00000276368	7	-1.08	-1.52	-1.49	-0.02	-0.2	0.996	-0.8	-0.86	-0.98	-3.416	-1.004	-2.27	-1.945	-1.712	-2.503	-1.057	-2.18	-2.35	-1.85	-2	2.761	2.491	-1.86	1.071	-1.87	-2.26	-2.45	0.203407	0.003259	1.387904503	-1.384645489
2235	P63218	GNG5	G protein subunit gamma 5	2787	ENSG00000174021	6	-0.25	0.514	-0.33	0.208	0.108	-0.33	-0.23	-0.18	0.104	-0.079	0.1218	0.0274	-0.026	-0.08	0.4717	0.0756	0.0231	0.2973	-0.53	0.033	-0.47	-0.18	-0.6	-0.16	-0.3	0.23	0.224	0.235153	0.150404	-0.134616256	0.285020477
1892	P40121	CAPG	capping actin protein, gelsolin like	822	ENSG00000042493	15	-0.63	-0.47	-0.82	-0.02	-0.15	-0.32	0.04	-0.32	0.309	-0.543	-0.165	0.2052	0.2665	-0.01	0.7757	0.0055	0.0224	0.1453	-0.37	-1.01	-0.24	-0.47	-0.72	0.313	0.014	0.134	0.375	0.310971	-0.04491	-0.341651847	0.296739009
1393	P02763	ORM1	orosomucoid 1	5004	ENSG00000229314	8	0.162	-1.64	-0.02	0.118	-1.14	1.484	-0.48	-0.14	-1.67	-0.957	-0.627	-1.263	0.6606	-0.88	-1.361	-0.717	-1.736	0.0241	-1.65	-1.79	1.635	1.793	0.7	0.517	-0.95	-0.86	-2.33	0.773852	-0.04318	0.391883983	-0.435068787
1923	P42685	FRK	fyn related Src family tyrosine kinase	2444	ENSG00000111816	19	-0.12	0.274	-0.14	0.004	-0.12	-0.31	-0.11	-0.2	0.192	-0.075	-0.087	-0.035	-0.08	-0.148	0.0198	0.2277	0.3643	-0.27	-0.14	0.189	-0.25	-0.26	-0.26	-0.32	-0.24	-0.07	-0.12	0.348828	0.104991	-0.050218367	0.155209142
3311	Q9BY43	CHMP4A	charged multivesicular body protein 4A	29082	ENSG00000285302	10	-0.01	0.353	-0.09	0.028	-0.07	-0.51	0.129	-0.1	0.188	-0.053	0.0231	0.2727	-0.05	-0.023	-0.005	0.0619	0.0337	-0.059	-0.12	-0.37	-0.16	-0.77	-0.44	-0.1	-0.09	-0.01	0.211	0.217126	0.197317	-0.030752592	0.228069847
1897	P40429	RPL13A	ribosomal protein L13a	23521	ENSG00000142541	14	0.199	0.044	0.013	-0.17	0.35	-0.28	0.183	-0.14	-0.02	0.0829	-0.171	-0.152	-0.103	-0.149	-0.149	0.0758	0.1467	0.0904	-0.08	-0.39	-0.06	-0.03	-0.17	-0.08	0.289	-0.32	-0.09	0.505347	0.123159	0.055585033	0.06757392
3647	Q9UKS6	PACSIN3	protein kinase C and casein kinase substrat	29763	ENSG00000165912	21	-0.09	0.465	-0.3	0.236	0.139	-0.38	-0.33	0.031	0.246	-0.283	-0.071	0.3463	0.3178	-0.176	0.0554	0.0437	-0.01	0.0214	-0.43	-0.4	-0.33	-0.51	-0.49	-0.25	0.083	0.239	0.528	0.476856	0.173417	-0.026067583	0.199484302
1747	P25398	RPS12	ribosomal protein S12	6206	ENSG00000112306	13	0.27	-0.1	0.024	0.194	-0.17	-0.37	0.192	-0.11	0.357	-0.144	-0.045	-0.057	0.0565	0.2093	-0.027	-0.127	0.1684	0.1201	0.015	-0.45	0.051	-0.57	-0.2	-0.02	0.139	-0.07	0.064	0.436265	0.147577	0.015149594	0.132427411
1372	P01833	PIGR	polymeric immunoglobulin receptor	5284	ENSG00000162896	29	-1.49	-1.72	-0.97	-1.46	-1.52	-0.86	-2.32	-1.31	-2.26	-1.948	-0.687	-1.112	-1.07	-0.383	-1.432	-0.835	-1.641	-2.126	-1.05	-1.03	2.808	2.281	0.438	1.399	-0.71	-1.89	-2.33	0.12699	-1.53738	-0.298242639	-1.239136421
3363	Q9H1Y0	ATG5	autophagy related 5	9474	ENSG00000057663	13	-0.12	0.109	-0.07	-0.01	-0.04	-0.46	0.2	0.205	0.399	0.0027	-0.071	0.1103	0.1339	-0.173	0.1343	0.2403	0.3873	0.2899	-0.19	-0.82	-0.31	-0.56	-0.28	-0.04	-0.02	0.049	0.271	0.152162	0.233167	-0.09349325	0.326660589
2641	Q5T6V5	C9orf64	chromosome 9 open reading frame 64	84267	ENSG00000165118	14	-0.04	0.143	0.018	-0.03	-0.11	-0.48	0.163	-0.02	0.328	0.2059	-0.016	0.1499	0.0218	-0.244	-0.13	-0.276	0.0862	0.1065	-0.3	-0.93	-0.39	-0.52	-0.4	0.042	0.224	0.061	0.451	0.465041	0.193333	0.0076101	0.185722835
3082	Q96A23	CPNE4	copine 4	131034	ENSG00000196353	18	-0.08	0.327	-0.22	0.127	-0.02	-0.63	-0.45	-0.22	-0.02	0.008	-0.106	-0.002	0.1209	-0.247	0.2352	0.1625	0.2408	-0.028	-0.3	-0.44	-0.25	-0.57	-0.51	0.216	-0.16	-0.15	-0.03	0.183446	0.111745	-0.174238642	0.285984075
2245	P68036	UBE2L3	ubiquitin conjugating enzyme E2 L3	7332	ENSG00000185651	13	0.007	0.184	-0.08	-0.11	0.061	-0.45	-0.22	-0.22	0.052	0.0045	0.0675	0.0087	0.0877	0.0147	0.024	-0.002	0.1381	-0.144	-0.31	-1.11	-0.37	-0.64	-0.37	0.08	-0.07	-0.12	-0.02	0.120989	0.239229	-0.107284345	0.346513787
2639	Q5T5C0	STXBP5	syntaxin binding protein 5	134957	ENSG00000164506	18	0.093	0.14	-0.08	-0.03	0.153	-0.48	0.258	0.137	0.085	0.0585	-0.193	-0.009	0.1476	-0.062	-0.047	0.1598	0.1887	0.1538	-0.29	-0.7	-0.2	-0.12	-0.25	0.059	0.091	-0.08	0.229	0.297603	0.170874	-0.013690207	0.184564017
3747	Q9Y3P9	RABGAP1	RAB GTPase activating protein 1	23637;2844	ENSG00000011454	28	0.04	0.053	-0.22	0.013	0.035	-0.5	0.004	0.036	0.234	0.1419	0.0349	0.0506	0.1117	-0.052	0.1084	-0.062	0.2329	0.2089	0.021	-0.6	-0.23	-0.4	-0.29	0.007	0.174	0.139	0.253	0.342127	0.069951	-0.119889529	0.189840327
1868	P35813	PPM1A	protein phosphatase, Mg2+/Mn2+ dependen	5494	ENSG00000100614	18	0.024	0.209	-0.17	-0.12	-0.06	-0.38	-0.12	-0.02	0.092	0.008	-0.086	0.1412	-0.025	-0.03	0.1158	-0.113	0.0555	-0.038	-0.28	-0.53	-0.42	-0.48	-0.31	0.026	-0.14	-0.1	0.093	0.118352	0.176743	-0.062806954	0.23954998
3444	Q9HCR9	PDE11A	phosphodiesterase 11A	50940	ENSG00000128655	19	0.639	-0.41	0.059	-0.03	0.01	-0.86	-0.07	0.352	0.276	0.2662	0.1423	-0.226	0.2217	-0.833	0.4261	0.0578	0.5058	0.351	0.041	-1.14	-0.96	-0.55	-0.5	-0.24	0.03	0.091	-0.02	0.216473	0.358752	-0.104495479	0.46324773
2197	P62424	RPL7A	ribosomal protein L7a	6130	ENSG00000148303	14	0.084	-0.08	-0.27	0.021	0.349	-0.35	0.114	0.978	0.125	-0.088	-0.123	-0.064	-0.227	-0.136	0.0105	0.2351	-0.118	0.1939	-0.02	-0.3	-0.07	-0.32	-0.22	-0.06	0.583	0.216	0.157	0.678534	0.110717	0.143397886	-0.032680665
2148	P61020	RAB5B;AC	RAB5B, member RAS oncogene family	5869	ENSG00000111540	9	-0.04	0.463	0.121	0.022	-0.18	-0.26	-0.23	-0.36	-0.09	0.0679	0.1634	-0.006	0.1471	-0.316	0.224	-0.015	0.3967	-0.304	-0.26	-0.88	-0.32	-0.53	-0.42	0.007	-0.36	-0.23	-0.11	0.09264	0.281895	-0.102162023	0.384057457
3167	Q96N03	VSTM2L	V-set and transmembrane domain containin	128434	ENSG00000132821	8	-0.4	-0.71	-0.54	-0.67	-0.21	0.558	0.006	-0.36	0.045	-0.491	0.5773	-0.254	-0.536	0.5884	0.2829	-0.072	-0.516	-0.16	-0.4	0.049	0.092	-0.53	-0.05	-0.08	0.511	0.155	-0.24	0.620871	-0.20015	-0.190269393	-0.009877193
1014	O00471	EXOC5	exocyst complex component 5	10640	ENSG00000070367	24	0.127	0.302	-0.04	0.032	-0.09	-0.34	-0.18	-0.33	-0.05	-0.013	0.1329	0.3557	-0.022	-0.083	-0.111	-0.2	-0.297	-0.274	-0.25	-0.92	-0.38	-0.4	-0.34	-0.13	-0.33	-0.29	-0.2	0.091798	0.298307	-0.005176379	0.303483247
1777	P28070	PSMB4	proteasome subunit beta 4	5692	ENSG00000159377	9	-0.71	0.333	0.372	0.065	0.094	-0.45	0.355	0.255	0.349	-0.332	-0.656	0.0222	-0.212	-0.27	-0.358	-0.07	-0.478	-0.006	-0.85	0.045	0.143	0.077	-0.19	-0.4	0.882	0.505	-0.04	0.316568	0.054103	0.335631019	-0.281527751
2968	Q8TBF2	PRXL2B	peroxiredoxin like 2B	127281	ENSG00000157870	11	-0.05	-0.31	-0.48	-0.05	-0.29	-0.35	-0.02	-0.13	-0.14	-0.119	-0.103	-0.221	-0.017	0.0522	-0.037	-0.184	-0.116	0.103	-0.08	-0.29	0.265	-0.54	-0.12	0.059	0.127	-0.23	-0.19	0.460344	-0.08963	-0.129563295	0.039933401
1526	P09972	ALDOC	aldolase, fructose-bisphosphate C	230	ENSG00000109107	24	-0.06	0.098	-0.12	0.042	0.154	-0.38	0.133	-0.01	0.017	0.2336	0.1691	-0.101	0.0609	-0.049	0.0133	0.1212	0.0759	-0.088	-0.14	-0.39	-0.1	-0.35	-0.19	-0.07	0.242	0.148	0.062	0.40609	0.073097	-0.063416865	0.136513472
4	A0A024R4M	RPS9	ribosomal protein S9	6203	ENSG00000170889	19	0.155	-0.05	-0.18	0.064	0.508	-0.25	0.102	-0.02	0.046	-0.039	-0.084	-0.163	-0.025	-0.13	-0.152	-0.094	0.0731	0.082	-0.3	-0.47	-0.11	-0.16	-0.04	0.12	0.175	-0.25	0.084	0.39966	0.14672	0.100637779	0.046081737
124	A0A087X2I1	PSMC6	proteasome 26S subunit, ATPase 6	5706	ENSG00000100519	22	-0.11	0.011	-0.09	0.049	0.211	-0.14	0.119	0.124	0.067	-0.089	0.1309	-0.064	-0.015	0.0154	0.0828	-0.01	0.1872	0.1686	-0.31	0.019	-0.12	-0.11	-0.18	-0.01	0.304	-0.09	0.132	0.538037	0.067345	-0.019680901	0.087026196
1931	P43251	BTD	biotinidase	686	ENSG00000169814	13	-0.34	0.07	0.023	0.011	0.099	-0.24	-0.27	-0.19	0.087	0.348	0.4336	0.3185	-0.117	0.0261	-0.205	0.076	0.0067	-0.126	-0.17	-0.12	-0.05	-0.38	-0.35	-0.1	-0.1	0.102	0.144	0.226716	0.030371	-0.167232166	0.19760329
3401	Q9H6S3	EPS8L2	EPS8 like 2	64787	ENSG00000177106	26	0.022	0.255	-0.27	0.026	0.001	-0.37	-0.11	-0.01	0.058	-0.174	0.0368	0.1514	0.0615	0.0133	0.1535	0.0758	0.1616	0.1927	-0.3	-0.38	-0.31	-0.4	-0.25	-0.17	0.012	0.013	0.224	0.127521	0.129014	-0.119784278	0.248798336
3685	Q9UPT5	EXOC7	exocyst complex component 7	23265	ENSG00000182473	25	0.047	0.169	-0.16	0.127	0.196	-0.39	-0.27	-0.07	-0.05	-0.041	0.0223	0.2829	0.1858	-0.006	0.2629	0.036	0.0186	0.0731	-0.26	-0.74	-0.29	-0.6	-0.36	0.035	-0.16	0.031	0.079	0.092254	0.206918	-0.137676103	0.344593823
2140	P60903	S100A10	S100 calcium binding protein A10	6281	ENSG00000197747	6	-0.18	0.01	-0.07	-0.27	0.019	-0.39	0.463	-0.07	0.061	0.4008	-0.83	-0.38	-0.266	0.2433	0.2984	0.6137	0.3631	0.7529	-0.23	-0.24	-0.32	-0.54	-0.22	-0.1	-0.17	-0.35	0.856	0.49921	0.097095	-0.180074234	0.277168993
1801	P30050	RPL12	ribosomal protein L12	6136	ENSG00000197958	8	0.093	-0.22	-0.11	-0.01	0.119	-0.36	0.371	0.145	0.369	-0.375	-0.197	-0.234	-0.118	-0.064	-0.017	0.0835	0.2001	0.2852	-0.41	-0.74	0.002	-0.4	-0.2	-0.09	0.416	0.027	0.078	0.494819	0.191684	0.093218114	0.098465585
3086	Q96A65	EXOC4	exocyst complex component 4	60412	ENSG00000131558	36	-0.01	0.245	-0.02	0.049	-0.01	-0.46	-0.17	-0.08	0.087	-0.004	0.2179	0.115	-4E-04	-0.111	-0.008	-0.016	0.1135	0.1902	-0.15	-0.44	0.105	-0.47	-0.43	-0.07	-0.08	0.13	0.238	0.32164	0.088358	-0.096808199	0.18516648
1991	P49366	DHPS	deoxyhypusine synthase	1725	ENSG00000095059	9	-0.05	0.529	-0.08	-0.01	-0.05	-0.32	0.733	-0.27	0.135	0.2079	0.1005	0.3266	0.2408	-0.052	-0.332	0.245	-0.168	0.3121	-0.11	-0.5	-0.17	-0.07	-0.48	0.096	0.005	-0.02	0.105	0.343461	0.195784	-0.028853604	0.224637572
1443	P05976	MYL1	myosin light chain 1	4632	ENSG00000168530	1	-0.05	0.093	-0.19	0.037	0.042	-0.32	-0.03	-0.33	0.044	0.1709	-0.234	-0.161	0.0615	0.1473	0.2481	0.0236	0.1844	-0.183	-0.28	-1.41	-0.41	-0.26	-0.44	0.069	-0.03	-0.16	-0.01	0.172733	0.245585	-0.106888919	0.352473893
2881	Q8N0V5	GCNT2	glucosaminyl (N-acetyl) transferase 2 (I bloc	2651	ENSG00000111846	16	0.31	-0.17	-0.36	-0.37	0.255	-0.15	-0.12	0.035	0.007	-0.283	0.2393	-0.259	-0.075	-0.537	-0.322	0.6616	0.4867	0.4188	-0.35	-0.12	0.189	-0.64	-0.76	-0.19	0.079	-0.04	0.023	0.501163	0.135821	-0.09948657	0.235307092
3761	Q9Y570	PPME1	protein phosphatase methylesterase 1	51400	ENSG00000214517	16	0.01	0.174	0.054	-0	0.004	-0.39	0.111	-0.08	0.327	0.1395	0.1454	0.1678	0.0162	-0.147	-0.059	0.1292	0.2443	0.1405	-0.26	-1.15	-0.25	-0.52	-0.36	0.077	-0.13	0.006	0.325	0.156407	0.273667	-0.063983226	0.337649868
2645	Q5TC63	GRTP1	growth hormone regulated TBC protein 1	79774	ENSG00000139835	7	0.426	0.646	0.166	0.142	0.019	-0.48	0.084	0.158	0.095	0.1716	0.3296	0.5413	-0.037	-0.076	0.1178	0.2494	0.4689	0.1158	-0.21	0.457	-0.11	-0.47	-0.52	0.193	-0.36	0.05	0.68	0.435312	0.172676	-0.06922608	0.241902071
982	M0R117	RPL18A	ribosomal protein L18a	6142	ENSG00000105640	10	-0.05	-0.23	-0.14	0.182	0.322	-0.17	0.179	-0.14	0.039	0.2631	0.2556	-0.2	0.0748	-0.196	-0.097	0.1666	0.2007	0.0521	-0.32	-0.25	0.292	0.115	0.007	0.246	0.331	-0.22	0.218	0.881077	-0.04874	-0.0590853	0.010346509
1317	O95861	BPNT1	3'(2'), 5'-bisphosphate nucleotidase 1	10380	ENSG00000162813	15	0.081	-0	-0.11	0.119	8E-04	-0.23	0.049	-0.04	0.215	0.0719	0.0652	-0.09	-0.101	-0.264	0.0037	0.217	0.1195	-0.067	-0.07	-0.23	-0.06	0.07	-0.53	-0.1	-0.04	0.09	0.219	0.667579	0.082407	0.015324596	0.067082564
1383	V9GYG9	APOA2	apolipoprotein A2	336	ENSG00000158874	5	-1.09	-1	-0.45	-0.97	-0.67	0.001	-0.08	0.135	-0.37	-0.134	0.002	-0.403	-0.595	-0.019	-0.5	-0.001	0.1107	-0.138	-0.51	0.196	0.927	1.267	-0.24	-0.05	0.381	0.183	-0.55	0.121398	-0.67729	-0.312835052	-0.364457775
60	A0A087WW	TPM3	tropomyosin 3	7170	ENSG00000143549	30	0.174	-0	-0.06	-0.07	0.202	-0.35	0.078	0.038	0.071	-0.031	0.1443	-0.082	0.0722	-0.196	0.0041	-0.068	0.0948	-0.089	-0.23	-0.6	0.316	0.339	-0.28	0.017	0.048	-0.08	-0.14	0.809655	0.075983	0.025593638	0.050389396
3774	Q9Y5X1	SNX9	sorting nexin 9	51429	ENSG00000130340	25	0.082	-0.01	-0.08	-0.06	0.093	-0.25	-0.29	0.019	0.06	-0.106	-0.025	0.2715	-0.006	-0.005	0.127	-0.098	-0.124	-0.01	-0.32	-0.15	-0.35	-0.41	-0.18	-0.09	0.11	-0.16	-0.01	0.1699	0.124986	-0.052195283	0.177181081
2322	Q06124	PTPN11	protein tyrosine phosphatase non-receptor t	5781	ENSG00000179295	25	-0.12	-0.07	-0.14	-0.01	-0.09	-0.13	-0.11	-0.02	0.182	0.0041	0.0363	0.0096	-0.069	-0.085	0.0053	0.1465	0.0491	0.0819	-0.13	-0.46	-0.2	-0.36	-0.12	-0.02	0.101	0.089	0.277	0.455506	0.034155	-0.077291894	0.111447314
3773	Q9Y5S2	CDC42BPI	CDC42 binding protein kinase beta	9578	ENSG00000198752	24	0.01	-0.14	-0.26	-1.01	-0.08	0.864	-0.23	-0.18	-0.38	-0.191	-0.041	-0.283	-1.029	0.5782	0.0276	-0.63	-0.555	-0.469	0.254	0.31	0.519	-0.92	0.202	-0.44	-0.09	-0.56	0.079	0.736188	-0.08321	0.132478027	-0.215692086
1688	P20336	RAB3A	RAB3A, member RAS oncogene family	5864	ENSG00000105649	14	0.37	0.631	0.167	-0.04	-0.06	-0.62	-0.61	-0.44	-0.26	0.5917	0.5973	0.2384	0.5013	-0.303	-0.2	0.0655	0.1433	-0.102	-0.18	-0.95	-0.23	-0.94	-0.53	0.405	-0.47	-0.34	-0.12	0.142705	0.275741	-0.266984961	0.542726352
2184	P62191	PSMC1	proteasome 26S subunit, ATPase 1	5700	ENSG00000100764	22	0.066	0.196	-0.07	-0.03	0.197	-0.45	0.228	0.177	0.317	-0.035	-0.013	0.0185	-0.09	-0.232	-0.03	0.2038	-0.039	0.212	-0.25	-0.65	-0.12	-0.27	-0.25	-0.01	0.289	0.142	0.008	0.343774	0.195342	0.071784147	0.123557649
994	O00203	AP3B1	adaptor related protein complex 3 subunit be	8546	ENSG00000132842	25	0.062	-0.09	-0.16	-0.11	-0.03	-0.45	-0.13	-0.08	0.067	0.1159	0.0042	-0.042	-0.031	-0.102	0.0831	0.1104	0.0388	0.1894	-0	-0.22	0.114	-0.17	-0.23	-0.04	0.193	-0.1	0.169	0.261172	-0.06987	-0.142787576	0.072913208
2020	P50443	SLC26A2	solute carrier family 26 member 2	1836	ENSG00000155850	19	-0.42	0.309	-0.21	-0.1	-0.08	-0.28	-0.11	0.244	-0.2	0.2838	0.1626	-0.051	0.0885	-0.431	-0.009	0.3646	0.1876	0.461	-0.01	0.535	-0.23	-0.13	-0.23	-0.15	0.052	-0.1	0.396	0.397974	-0.10921	-0.211996521	0.102785246
3334	Q9GZP4	PITHD1	PITH domain containing 1	57095	ENSG00000057757	12	-0.09	0.066	-0.09	-0.03	-0.26	-0.61	-0	-0.16	0.263	-0.008	0.1328	-0.009	-0.108	-0.101	0.0917	0.0404	0.1462	-0.015	-0.23	-0.67	-0.32	-0.76	-0.61	-0.16	-0.14	0.078	0.154	0.147931	0.194265	-0.120099798	0.314365081
1594	P13726	F3	coagulation factor III, tissue factor	2152	ENSG00000117525	8	-0.41	0.171	-0.4	-0.75	-0.25	0.103	0.894	-0.64	-0.39	0.3453	0.41	-0.783	-0.397	0.3525	0.0289	-0.24	-0.392	-0.519	-0.43	-0.2	0.962	0.269	-0.53	0.419	-0.37	-0.05	-0.55	0.898208	-0.1321	-0.052349973	-0.079749652
3449	Q9NP61	ARFGAP3	ADP ribosylation factor GTPase activating p	26286	ENSG00000242247	17	0.199	0.078	-0.05	0.069	0.019	0.013	-0.06	-0.02	0.169	0.0848	0.0433	0.2577	0.1688	-0.057	0.1568	0.0658	0.1419	0.1318	-0.13	-0.77	-0.07	-0.69	-0.38	-0.02	0.109	-0.05	0.207	0.102908	0.245518	-0.063704085	0.30922233
3571	Q9P2J5	LARS1;LA	leucyl-tRNA synthetase 1;leucyl-tRNA synth	51520	ENSG00000133706	32	-0.09	0.031	-0.04	0.157	0.14	-0.3	0.19	0.01	0.315	-0.056	0.0163	0.0316	-0.042	-0.155	0.0072	0.035	0.2069	0.2834	-0.24	-0.48	-0.22	-0.38	-0.25	#####	0.246	0.193	0.345	0.503519	0.133407	0.010574474	0.122832882

1950	P46926	GNPDA1	glucosamine-6-phosphate deaminase 1	10007	ENSG00000113552	11	-0.23	-0.61	-0.5	0.156	0.14	-0.33	-0.02	0.056	0.127	-0.878	0.0146	-0.441	-0.075	-0.127	0.1839	0.1729	0.0838	0.0977	-0.4	-0.6	1.273	-0.55	-0.25	-0.21	0.159	0.175	0.192	0.899058	-0.11051	-0.026991338	-0.083520168
345	A0A2R8YGH	AP1S1	adaptor related protein complex 1 subunit si	1174	ENSG00000106367	5	0.062	0.084	-0.02	-0.17	0.09	-0.48	5E-04	0.09	0.246	0.0829	-0.132	-0.038	0.1388	-0.226	0.027	0.1196	0.1315	0.1397	-0.21	-1.05	-0.29	-0.51	-0.37	0.044	0.224	0.06	0.245	0.336986	0.195029	-0.03748483	0.23251336
1480	P07814	EPRS1;EP	glutamyl-prolyl-tRNA synthetase 1;glutamyl-	2058	ENSG00000136628	31	-0.09	-0.1	0.005	0.536	0.419	-0.09	-0.01	-0.2	0.158	-0.211	-0.15	-0.007	-0.125	-0.182	-0.035	-0.147	0.1733	0.0677	-0.35	-0.39	0.335	-0.27	-0.18	-0.07	0.289	-0.13	6E-04	0.440469	0.155273	0.138333673	0.016938914
2096	P54802	NAGLU	N-acetyl-alpha-glucosaminidase	4669	ENSG00000108784	27	-0.13	-0.29	0.134	-0.44	0.011	-0.43	-0.2	0.23	0.203	0.4177	-0.027	0.2725	-0.362	-0.1	-0.528	0.0552	-0.204	-0.04	-0.11	0.076	0.037	0.155	0.03	-0.09	0.297	0.333	-0.43	0.670724	-0.13422	-0.043388004	-0.090831458
1353	P01116	KRAS	KRAS proto-oncogene, GTPase	3845	ENSG00000133703	11	-0.13	0.303	-0.06	0.132	0.248	-0.34	0.122	0.052	0.174	-0.115	0.0139	0.1096	0.0743	-0.237	0.2543	0.3796	0.27	0.3125	-0.41	-0.53	-0.55	0.318	-0.36	0.175	-0.24	0.108	0.372	0.315934	0.179435	-0.062490092	0.241924954
3639	Q9UJU6	DBNL	drebrin like	28988	ENSG00000136279	18	-0.08	0.079	0.082	-0.06	-0.11	-0.36	0.033	0.066	0.323	-0.128	-0.154	-0.064	-0.144	-0.212	-0.033	0.0624	0.1451	0.2072	-0.12	-0.63	-0.19	-0.45	-0.24	-0.02	-0.1	0.074	0.159	0.342702	0.166235	0.033792758	0.132442729
5	A0A1B0GW	CLN5	CLN5 intracellular trafficking protein	1203	ENSG00000102805	11	-0.07	-0.44	-0.1	0.079	-0.16	-0.13	-0.14	0.137	0.215	0.2183	0.0842	0.3205	-0.285	0.2224	-0.302	-0.022	-0.285	-0.307	-0.26	-0.25	0.04	-0.19	-0.38	-0.16	0.185	0.365	-0.12	0.943389	0.017681	-0.027941547	0.045622238
1536	P0DOY2	IGLC2	immunoglobulin lambda constant 2	3538	ENSG00000211677	10	-1.14	-0.98	-0.41	-1.5	-1.45	-0.96	-1.56	-1.06	-1.44	-0.931	-0.482	-0.427	-1.127	-1.112	-1.673	-0.851	-0.968	-1.276	-0.86	-0.86	2.79	2.252	0.182	1.277	-0.53	-0.27	-1.99	0.101318	-1.38827	-0.18344999	-1.204815923
2172	P61758	VBP1	VHL binding protein 1	7411	ENSG00000155959	10	0.101	0.262	-0.07	0.021	0.163	-0.55	0.148	0.088	0.333	0.0923	-0.189	-0.036	-0.083	-0.183	0.1072	0.3275	0.244	0.118	-0.02	-0.85	-0.32	-0.57	-0.22	-0.1	0.092	0.097	0.212	0.264399	0.242311	0.010800057	0.231511247
3065	Q93009	USP7	ubiquitin specific peptidase 7	7874	ENSG00000187555	29	0.164	0.189	0.003	0.105	0.294	-0.31	0.041	0.12	0.214	0.0305	-0.035	0.0048	0.1107	-0.075	0.0056	0.0235	0.1794	0.1337	-0.21	-0.64	-0.25	-0.38	-0.2	0.089	0.007	0.06	0.159	0.137444	0.242653	0.048879891	0.193773183
1287	O95433	AHSA1	activator of HSP90 ATPase activity 1	10598	ENSG00000100591	13	0.063	-0.05	-0.12	0.09	0.022	-0.39	0.111	-0.01	0.314	0.0199	-0.21	0.065	0.0672	-0.161	0.0711	0.1838	0.0551	0.1335	-0.06	-0.9	-0.26	-0.61	-0.24	0.106	0.12	0.062	0.216	0.362348	0.177731	-0.020579658	0.198311142
3340	Q9GZZ9	UBA5	ubiquitin like modifier activating enzyme 5	79876	ENSG00000081307	13	0.174	0.218	-0.05	-0.12	-0.03	-0.53	0.004	0.034	0.226	0.0524	-0.002	0.0759	0.0803	-0.11	0.114	0.1008	0.2116	0.1348	-0.23	-1.07	-0.18	-0.59	-0.31	0.059	0.068	-0	0.119	0.179415	0.229288	-0.082567169	0.311855339
1374	P01871	IGHM	immunoglobulin heavy constant mu	3507	ENSG00000211899	28	-2.72	-3	-2.46	-1.12	-0.73	-0.63	-2.62	-2.53	-2.66	-3.196	-1.979	-2.614	-0.965	-0.549	-0.81	-1.865	-2.262	-2.572	-1.8	-1.88	1.181	3.325	-0.85	0.374	-1.62	-2.07	-2.41	0.20514	-1.41269	-0.183350417	-1.229336441
1113	O43396	TXNL1	thioredoxin like 1	9352	ENSG00000091164	16	0.054	0.26	0.026	-0.09	0.012	-0.47	0.244	0.037	0.314	0.0765	-0.102	-0.112	0.074	-0.196	0.0683	0.229	0.2284	0.1698	-0.23	-1.01	-0.16	-0.63	-0.4	0.12	-0.02	-0.06	0.354	0.226096	0.267877	-0.004940701	0.272817358
3058	Q92900	UPF1	UPF1 RNA helicase and ATPase;UPF1, RN	5976	ENSG00000005007	22	0.04	0.069	-0.09	-0.27	-0.17	-0.49	0.008	-0.06	0.242	0.0444	0.0582	0.1135	-0.144	-0.107	0.0246	0.0409	0.0872	0.1447	-0.24	-0.83	-0.29	-0.42	-0.41	0.016	0.107	0.056	0.35	0.356526	0.10465	-0.1086632	0.213312933
3743	Q9Y3F4	STRAP	serine/threonine kinase receptor associated	11171	ENSG00000023734	13	0.118	0.233	0.05	-0.07	0.026	-0.47	0.422	0.385	0.43	0.1097	0.0046	0.0167	0.0661	-0.183	0.0929	0.2324	0.3007	0.3533	-0.17	-0.8	-0.09	-0.34	-0.37	0.137	0.276	0.119	0.467	0.413799	0.211255	0.014310791	0.196944645
1325	P00352	ALDH1A1	aldehyde dehydrogenase 1 family member A	216	ENSG00000165092	22	0.068	-0.24	0.511	0.354	0.624	-0.02	-0.04	0.506	-0.01	-0.241	-0.083	0.4397	0.0561	-0.365	-0.227	-0.473	0.4003	-0.026	-0.57	-0.46	-0.27	-0.43	0.175	-0.66	0.305	-0.2	-0.99	0.10117	0.537956	0.252945998	0.285010408
1263	O94886	TMEM63A	transmembrane protein 63A	9725	ENSG00000196187	18	-0.3	0.086	0.185	-0.27	-0.28	-0.73	0.374	-0.2	0.314	0.2528	0.2998	-0.242	0.0334	-0.292	0.0612	0.1079	0.2943	0.3309	-0.27	-0.32	0.036	-0.18	-0.48	0.053	0.187	0.024	0.229	0.469985	-0.01041	-0.185221479	0.174816145
2097	P54803	GALC	galactosylceramidase	2581	ENSG00000054983	12	-0.16	-0.31	-0.02	-0.42	-0.06	-0.56	-0.17	-0.06	0.045	-0.267	0.0742	0.2586	-0.555	-0.012	-0.316	0.1582	-0.315	-0.025	0.167	0.441	-0.13	-0.31	-0.22	0.008	0.28	0.184	-0.22	0.353299	-0.21222	-0.079979779	-0.132238863
1281	O95372	LYPLA2	lysophospholipase 2;lysophospholipase II	11313	ENSG00000011009	9	0.026	0.139	-0.03	-0	-0.1	-0.39	-0.1	-0.05	0.184	0.1042	0.127	0.0605	0.0531	-0.119	0.164	0.1152	0.1561	0.0911	-0.33	-1.14	-0.34	-0.56	-0.33	0.132	-0.05	-0.11	0.151	0.118385	0.249959	-0.119625145	0.369584617
3209	Q99538	LGMN	legumain	5641	ENSG00000100600	16	-0.29	-0.69	-0.19	0.038	-0.46	0.038	-0.19	0.007	0.441	0.1902	-0.018	0.1127	-0.216	0.4484	-0.002	0.5474	-0.321	-0.279	-0.32	-0.2	-0.28	-0.22	-0.33	-0.4	0.786	0.516	-0.13	0.637511	-0.07987	-0.195494533	0.115621767
842	H3BLU2	LSAMP	limbic system associated membrane protein	4045	ENSG00000185565	15	0.211	0.257	-0.77	-0.32	0.501	-0.85	-0.06	-0.03	-0.04	0.6212	0.0893	0.1185	-0.356	-0.305	-0.458	0.474	-0.144	0.4959	0.146	-0.73	-0.1	-1.08	-0.03	-0.38	0.199	0.983	-0.74	0.673042	0.069467	-0.183026213	0.252493363
1757	P26572	MGAT1	mannosyl (alpha-1,3-)-glycoprotein beta-1,2	4245	ENSG00000131446	15	-0.26	-0.27	-0.41	-0.22	-0.11	-0.48	-0.13	#####	0.043	-0.057	0.2058	-0.114	-0.186	0.1767	-0.274	0.4553	-0.089	0.2308	-0.13	0.724	-0.01	-0.05	-0.53	-0.27	-0.04	0.094	-0.05	0.29174	-0.17476	-0.243431049	0.068671766
1104	O43237	DYNC1LI2	dynein cytoplasmic 1 light intermediate chai	1783	ENSG00000135720	16	0.093	0.105	-0.02	-0.1	-0.01	-0.45	0.07	-0.14	0.096	-0.056	0.1051	0.1114	0.0195	-0.174	0.1172	0.0574	0.1611	0.1083	-0.15	-0.91	-0.34	-0.51	-0.31	0.041	0.042	0.021	0.1	0.172033	0.184678	-0.089824659	0.274503056
693	F2Z2Y4	PDXK	pyridoxal kinase	105372824;8	ENSG00000160209	9	-0.13	0.036	0.083	-0.06	-0.16	0.12	0.267	0.127	0.273	-1E-04	0.0387	-0.119	-0.059	-0.083	0.0176	0.3473	0.2506	0.2711	-0.27	-0.38	-0.26	-0.23	-0.15	-0.06	0.156	0.16	0.418	0.433281	0.129929	-0.011974498	0.141903613
1787	P29350	PTPN6	protein tyrosine phosphatase non-receptor t	5777	ENSG00000111679	17	-0.05	0.017	0.106	-0.11	-0.17	-0.2	0.03	-0.08	0.002	-0.204	0.2007	0.6151	-0.031	-0.111	-0.034	0.4125	0.0796	0.2968	-0.54	-0.36	-0.25	-0.12	-0.4	-0.03	0.398	-0.14	-0.52	0.116176	0.167419	-0.185636106	0.353055126
255	A0A0U1RRC	SYTL2	synaptotagmin like 2	54843	ENSG00000137501	9	0.071	-0.32	0.172	-0.48	-0.16	-0.09	-0.44	-0.54	0.399	-0.039	0.1412	0.2175	-0.041	0.2849	-0.207	-0.864	-0.273	-0.118	-0.25	-0.01	-0	-0.18	-0.37	0.139	-0.06	0.642	-0.54	0.910309	-0.08438	-0.053388223	-0.030989712
2761	Q7L266	ASRGL1	asparaginase like 1;asparaginase and isoas	80150	ENSG00000162174	10	-0.04	-0.08	-0.08	0.498	0.63	-0.12	0.047	0.377	0.344	0.4953	-0.348	-0.592	0.0919	-0.28	-0.111	-0.076	-0.454	-0.045	-0.13	-0.37	0.14	0.084	-0.11	0.258	-0.13	-0.09	0.068	0.192018	0.204728	0.320146691	-0.115418344
2959	Q8NHP8	PLBD2	phospholipase B domain containing 2	196463	ENSG00000151176	11	-0.14	-0.69	-0.23	-0.39	-0.32	-0.35	-0.11	0.165	0.162	0.3002	-0.199	-0.118	-0.438	-0.104	-0.457	-0.041	-0.442	-0.414	0.051	0.599	0.066	0.315	0.043	-0.21	0.161	5E-04	-0.24	0.147389	-0.29935	0.000574694	-0.299925134
3383	Q9H3U7	SMOC2	SPARC related modular calcium binding 2	64094	ENSG00000112562	9	0.596	0.24	-0.47	-0.45	-0.61	0.412	0.947	0.591	0.848	-0.353	0.1694	-0.027	-0.563	-0.662	0.1121	-0.043	-0.067	-0.269	-0.77	-0.04	-0.48	-0.37	-0.75	-0.5	0.882	-0.08	-0.12	0.224258	0.483113	0.424128073	0.058985424
1538	P10155	RO60;TRC	Ro60, Y RNA binding protein;TROVE doma	6738	ENSG00000116747	17	0.165	0.111	-0.11	-0.05	-0	-0.4	-0.03	-0.1	0.178	0.1102	-0.041	0.1147	0.0516	-0.116	-0.066	0.1681	0.1723	-0.083	-0.12	-0.63	-0.16	-0	-0.3	0.069	0.236	-0.01	0.071	0.528633	0.065997	-0.061287741	0.127285215
3024	Q8WZ73	RFFL	ring finger and FYVE like domain containing	117584	ENSG00000092871	14	-0	0.269	-0.09	0.025	-0.26	-0.34	-0.16	-0.03	0.05	-0.296	-0.191	-0.11	-0.007	-0.18	0.0958	0.1963	0.1288	0.0265	-0.37	-0.77	-0.17	-0.43	-0.32	-0.01	0.059	-0.06	-0.08	0.235431	0.178961	-0.022470045	0.20143104
3276	Q9BUP3	HTATIP2	HIV-1 Tat interactive protein 2	10553	ENSG00000109854	16	0.399	0.022	-0.07	0.068	-0.21	-0.3	-0.1	-0.13	0.017	-0.017	0.0387	0.0082	0.1558	-0.156	0.2151	-0.012	0.1481	-0.043	-0.4	-0.76	-0.3	-0.46	-0.52	-0.15	0.102	-0.06	0.341	0.172402	0.210274	-0.072714025	0.282988003
1436	P05198	EIF2S1	eukaryotic translation initiation factor 2 subu	1965	ENSG00000134001	15	0.076	0.071	-0.1	0.108	0.158	-0.27	-0.16	-0.05	-0.03	-0.049	-0.121	-0.033	-0.115	0.0503	0.2416	-0.045	0.0902	-0.1	-0.12	-0.71	0.013	-0.23	-0.07	0.127	0.03	-0.08	-0	0.535157	0.094325	-0.012447701	0.106772742
1147	O60243	HS6ST1	heparan sulfate 6-O-sulfotransferase 1	9394	ENSG00000136720	13	-0.07	-0.62	-0.45	-0.25	-0.1	-0.28	-0.11	-0.53	-0.13	0.1929	0.4213	0.1595	-0.28	0.5458	-0.016	0.0661	-0.674	-0.136	0.44	-0.12	0.009	-0.08	-0.23	-0.09	0.568	0.041	-0.22	0.172932	-0.31842	-0.31329693	-0.005127876
2187	P62249	RPS16	ribosomal protein S16	6217	ENSG00000105193	14	0.072	-0.32	-0.11	0.039	0.294	-0.2	0.344	0.08	0.242	-0.247	-0.019	-0.17	-0.296	-0.155	-0.129	-0.092	0.1969	0.2251	-0.21	-0.45	-0.03	-0.32	-0.15	-0.13	0.246	-0.14	0.238	0.445595	0.154222	0.125007712	0.029214484
2991	Q8TE77	SSH3	slingshot protein phosphatase 3	54961	ENSG00000172830	13	-0.42	-0.21	-0.33	-0.17	-0.08	-0.6	0.212	-0.07	0.161	-0.019	-0.143	0.2282	0.3118	-0.009	-0.154	-0.011	0.1595	-0.03	-0.27	-0.53	-0.36	-0.26	-0.29	0.039	0.129	0.073	0.312	0.328077	-0.03989	-0.205628896	0.165741885
1501	P08294	SOD3	superoxide dismutase 3	6649	ENSG00000109610	12	-0.34	-0.55	0.101	-0.03	-0.58	-0.54	-0.28	-0.01	0.128	0.6506	-0.206	0.2748	-0.048	0.006	-0.56	0.4426	-0.179	-0.243	-0.46	0.098	0.022	-0.37	0.441	-0.36	0.18	0.409	-0.36	0.449144	-0.18936	-0.247931314	0.058569329
2404	Q13421	MSLN	mesothelin	10232	ENSG00000102854	18	-0.12	-0.07	-0.46	-0.45	0.233	-0.29	0.423	0.339	0.277	-0.079	-0.029	-0.489	-0.497	-0.173	-0.271	0.2715	-0.024	-0.297	0.113	0.086	0.65	-0.02	-0.52	-0.09	0.479	-0.13	1.402	0.283725	-0.23204	0.163898764	-0.395934166
2151	P61081	UBE2M	ubiquitin conjugating enzyme E2 M	9040	ENSG00000130725	12	0.036	0.221	0.021	-0.11	0.029	-0.31	0.059	0.079	0.174	0.2543	0.1313	0.0838	0.0266	-0.245	0.0124	0.1297	0.1734	0.1293	-0.36	-0.82	-0.24	-0.49	-0.3	0.043	-0.06	-0.04	0.077	0.084063	0.265556	-0.055303034	0.320859171
1318	O95865	DDAH2	dimethylarginine dimethylaminohydrolase 2	23564	ENSG00000213722	15	0.084	0.138	-0.09	-0.09	0.001	-0.39	0.213	-0.08	0.194	0.2487	0.0993	-0.156	-0.128	-0.027	0.1991	0.1307	0.23	0.1744	-0.07	-0.63	-0.24	-0.6	-0.29	0.034	-0.01	0.097	0.341	0.25381	0.14869	-0.088920296	0.237610037
1756	P26373	RPL13	ribosomal protein L13	6137	ENSG00000167526	10	0.187	0.235	-0.15	-0.24	0.777	-0.25	0.2	-0.14	0.034	-0.092	-0.21	-0.18	-0.221	-0.087	0.119	-0.238	0.1439	-0.035	-0.13	-0.26	0.126	-0.25	0.128	0.068	0.197	-0.36	-0.03	0.484981	0.129637	0.160338961	-0.030702162
1162	O60513	B4GALT4	beta-1,4-galactosyltransferase 4	8702	ENSG00000121578	14	-0.09	-0.5	-0.09	-0.78	-0.35	-0.13	-0.32	0.198	0.145	0.1682	-0.032	0.0938	-0.297	0.115	0.1583	-0.232	-0.265	-0.596	0.243	0.34	0.258	0.372	-0.15	0.027	0.354	-0.24	-0.12	0.173001	-0.33296	-0.113926474	-0.219029624
1139	O43813	LANCL1	LanC like 1	10314	ENSG00000115365	12	0.03	0.067	-0.08	0.123	0.097	-0.32	0.16	0.375	0.467	-0.054	-0.044	-0.166	0.0305	-0.208	0.1078	0.2064	0.0448	0.1714	-0.23	-0.61	-0.15	-0.45	-0.39	0.105	0.072	0.425	0.227	0.375717	0.2128	0.092303739	0.120495954
3627	Q9UIC8	LCMT1	leucine carboxyl methyltransferase 1	51451	ENSG00000205629	11	0.08	0.193	-0.22	0.258	0.548	-0.37	0.605	0.328	0.613	-0.092	-0.035	-0.163	0.3098	-0.252	0.0626	0.3013	0.0538	0.3768	-0.32	-1.2	-0.17	-0.58	-0.25	0.105	0.332	0.057	0.225	0.193273	0.42659	0.164465949	0.262124156
3705	Q9Y266	NUDC	nuclear distribution C, dynein complex regul	10726	ENSG00000090273	18	0.086	0.044	0.004	-0.12	0.21	-0.36	-0.11	0.059	0.186	-0.008	0.0072	-0.039	-0.127	0.017	0.1381	-0.094	0.1211	-0.022	-0.34	-1.01	-0.05	-0.51	-0.27	-0.1	0.095	-0.03	0.004	0.172484	0.245804	0.001099193	0.244705069
3653	Q9ULC3	RAB23	RAB23, member RAS oncogene family	51715	ENSG00000112210	10	0.094	0.316	-0.13	0.088	-0.15	-0.3	-0.02	-0.18	0.036	-0.031	0.1371	-0.01	0.0351	-0.324	0.3376	0.1491	0.2568	0.0876	-0.22	-1.13	-0.27	-0.74	-0.43	-0.04	-0.3	-0.05	0.537	0.177801	0.267351	-0.098654687	0.366005644
104	H3BU88	TMSB15B	thymosin beta 15B	286527	ENSG00000158427	1	-0.36	0.286	-0.07	0.142	-0.15	-0.65	0.754	-0.32	0.544	-0.161	-0.106	-0.35	0.0437	-0.29	-0.06	0.1905	0.2644	-0.054	-0.5	-0.95	0.299	-0.19	-0.29	0.164	-0.05	-0.14	0.424	0.773481	0.157825	0.0780507	0.079774184
34	A0A087WX1	CDH1	cadherin 1	999	ENSG00000039068	12	-0.08	-0.37	-0.14	-0.12	-0.17	0.005	-0.12	0.119	0.506	0.1447	0.046	0.3966	-0.171	-0.213	-0.035	0.6173	0.0945	0.4361	-0.24	-0.33	-0.09	0.486	-0.36	-0.1	0.654	0.292	0.08	0.57032	-0.08633	-0.188139501	0.101814496
1133	O43692	PI15	peptidase inhibitor 15	51050	ENSG00000137558	8	-0.47	-0.41	-0.39	-0.19	0.07	-0.28	0.214	-0.46	0.698	0.0165	-0.617	0.1659	-0.069	0.0141	-0.343	0.3755	-0.856	0.3003	-0.27	-0.01	-0.35	0.34	0.731	-0.13	0.785	0.606	-0.49	0.51421	-0.27027	-0.023572275	-0.246693848
2707	Q6P9B6	MEAK7	MTOR associated protein, eak-7 homolog	57707	ENSG00000140950	8	0.04	0.259	-0.04	0.218	-0.4	-0.36	0.343	-0.18	0.18	0.2634	0.3057	0.0272	-0.095	-0.334	0.1352	0.3024	0.1479	-0.063	-0.01	-0.52	-0.02	-0.8	-0.46	0.097	0.137	0.214	0.213	0.497696	0.132238	-0.070720285	0.202958745
2618	Q5M775	SPECC1	sperm antigen with calponin homology and c	92521	ENSG00000128487	15	0.007	0.145	-0	-0.12	0.275	0.156	-0.15	-0.1	0.191	-0.097	-0.009	0.3017	-0.094	-0.18	-0.3	0.1253	0.0988	0.015	-0.43	-0.81	-0.04	0.6	0.229	0.4	-0.03	-0.05	-0.06	0.909191	0.064609	0.05906691	0.005541963
3381	Q9H3S7	PTPN23	protein tyrosine phosphatase non-receptor t	25930	ENSG00000076201	26	-0.11	-0.07	-0.3	0.03	0.128	0.102	0.082	0.087	0.069	0.0261	0.1003	-0.074	0.0372	0.1649	0.0808	0.339	0.1444	0.3299	0.056	0.037	-0.19	-0.37	-0.02	-0.08	0.047	0.057	0.128	0.187996	0.038335	-0.12642405	0.164758837
2577	Q3KQV9	UAP1L1	UDP-N-acetylglucosamine pyrophosphoryla	91373	ENSG00000197355	19	-0.25	-0.04	-0.2	-0.04	0.021	-0.26	0.127	-0	-0.03	-0.271	0.2562	-0.315	-0.23	-0.178	0.1037	0.1355	0.2624	0.0927	-0.16	-0.77	-0.21	-0.02	-0.12	0.074	-0.03	0.214	0.473	0.917123	-0.01408	-0.058384656	0.044301381
2425	Q13619	CUL4A	cullin 4A	8451	ENSG00000139842	20	0.082	-0.08	-0.07	-0.07	-0.11	0.075	0.285	0.1	0.301	0.0334	-0.172	0.0657	-0.151	-0.037	0.0519	0.2814	0.0569	0.2193	-0.14	-0.71	-0.08	-0.08	-0.16	-0.07	0.165	0.057	0.311	0.48679	0.135282	0.017703523	0.1175785
3066	Q93034	CUL5	cullin 5	8065	ENSG00000166266	23	0.111	0.104	-0.07	-0.01	0.128	-0.19	-0.05	0.132	0.161	0.1458	-0.02	0.0459	-0.02	-0.106	0.1219	0.1856	0.0063	0.1084	-0.19	-0.61	-0.13	-0.43	-0.1	-0.08	0.173	0.131	0.147	0.23026	0.156337	-0.016327891	0.172664762
1983	P49006	MARCKSL	MARCKS like 1	65108	ENSG00000175130	10	-0.51	0.286	0.256	0.037	0.172	-0.59	0.12	-0.27	0.195	-0.042	0.0654	-0.015	0.0335	-0.21	0.3148	0.0404	0.108	-0.085	-0.17	-0.24	-0.38	-0.71	-0.35	-0.08	0.086	0.062	-0.01	0.341962	0.1647	-0.057259742	0.221959985
2517	Q15366	PCBP2	poly(rC) binding protein 2	5094	ENSG00000197111	12	-0	0.122	0.009	-0.06	0.169	-0.38	-0.1	-0.05	0.157	-0.007	-0.084	0.0921	0.0485	-0.169	0.1131	0.007	0.2595	0.1182	-0.23	-0.54	-0.28	-0.36	-0.24	0.05	-0.07	0.043	0.236	0.2214	0.139563	-0.05617792	0.195741011
997	O00232	PSMD12	proteasome 26S subunit, non-ATPase 12	5718	ENSG00000197170	18	0.013	0.098	-0.07	-0.02	0.241	-0.49	-0.13	-0.21	0.016	0.2049	-0.13	0.1261	0.1356	-0.207	0.006	-0.094	0.1704	0.1023	-0.17	-0.6	-0.1	-0.18	-0.31	0.068	0.071	-0.14	-0.13	0.232041	0.104495	-0.096834743	0.201329482
2970	Q8TBX8	PIP4K2C	phosphatidylinositol-5-phosphate 4-kinase t	79837	ENSG00000166908	18	0.191	0.208	0.071	0.035	0.163	-0.19	-0.11	-0.02	0.04	0.0842	-4E-04	0.1169	-0.014	-0.12	0.1634	-0.011	0.0287	-0.01	-0.17	-0.91	-0.32	-0.58	-0.3	0.019	-0.09	-0.06	0.088	0.084	0.301779	0.01667917	0.285100905
2008	P49788	RARRES1	retinoic acid receptor responder 1	5918	ENSG00000118849	11	-1.12	-1.02	-0.3	-0.9	-0.57	-0.69	-1.19	-0.99	-1.16	-0.993	0.5556	-0.676	-0.859	-0.177	-0.778	-1.156	-1	-0.528	-0.26	-0.3	2.165	1.787	-0.32	0.832	-0.31	-0.43	-1.63	0.128455	-1.05292	-0.258511035	-0.794408171
1836	P32929	CTH	cystathionine gamma-lyase	1491	ENSG00000116761	12	0.075	-0.05	-0.26	-0.06	0.318	-0.6	-0.01	-0.11	0.19	-0.087	-0.016	0.1734	0.1564	-0.128	-0.004	0.1809	0.1192	0.1676	-0.18	-0.97	-0.32	-0.6	-0.31	-0.09	0.109	0.051	0.258	0.239956	0.170298	-0.120416438	0.290714495
2370	Q12965	MYO1E	myosin IE	4643	ENSG00000157483	24	-0.3	0.163	-0.13	-0.32	-0.09	-0.37	-0.06	-0.01	-0.2	0.2127	-0.191	-0.011	0.017	-0.118	-0.232	-0.123	0.2309	-0.004	-0.38	-0.1	-0.09	-0.18	-0.28	0.135	-0.02	-0.17	0.055	0.444051	-0.03233	-0.12194329	0.089610408
651	Q15418	RPS6KA1	ribosomal protein S6 kinase A1	6195	ENSG00000117676	29	-0.01	0.005	0.006	-0.13	-0.02	-0.17	0.077	0.003	0.337	0.0407	-0.089	0.1366	-0.124	-0.133	0.0893	0.1537	0.2673	0.3055	-0.18	-0.59	-0.11	-0.48	-0.34	0.085	0.032	0.121	0.322	0.304045	0.137939	-0.060969747	0.198908651
3434	Q9HB71	CACYBP	calcyclin binding protein	27101	ENSG00000116161	13	0.16	0.121	0.074	-0.1	0.305	-0.37	0.125	-0.25	0.158	0.0985	-0.063	0.0446	0.1113	-0.13	-0.008	0.031	0.1175	0.2453	-0.12	-0.71	-0.18	-0.61	-0.36	0.117	0.066	-0.25	-0.02	0.126784	0.253977	-0.025056146	0.279033053
210	O00571	DDX3X	DEAD-box helicase 3 X-linked	1654	ENSG00000215301	19	0.262	0.176	0.13	-0.01	0.086	-0.26	0.14	0.165	0.3	0.0893	0.0171	0.0805	0.0598	-0.142	0.0089	0.1792	0.1009	0.187	-0.16	-0.51	-0.06	-0.23	-0.16	0.09	0.194	0.023	0.277	0.277611	0.170644	0.045563542	0.125080789
1304	O95749	GGPS1	geranylgeranyl diphosphate synthase 1	9453	ENSG00000152904	10	0.024	-0.17	-0.03	0.219	0.454	-0.26	-0.17	-0.19	-0.15	0.0043	-0.158	-0.132	0.047	-0.025	0.0229	-0.197	-0.037	-0.268	0.24	-0.43	-0.04	-0.21	-0.14	0.045	-0.1	-0.14	-0.21	0.756467	0.079638	0.051546441	0.028091425
3548	Q9NZH0	GPRC5B	G protein-coupled receptor class C group 5	51704	ENSG00000167191	4	-0.31	0.006	-0.15	-0.27	-0.35	0.336	-0.31	-0.41	-0.34	-0.262	0.3405	0.0812	-0.199	-0.216	-0.187	-0.287	-0.219	-0.231	-0.06	0.534	-0.03	1.34	0.185	0.304	-0.35	-0.23	-0.1	0.203116	-0.37781	-0.069686996	-0.308125779
1299	O95671	ASMTL	acetylserotonin O-methyltransferase like	8623	ENSG00000169093	21	0.457	0.243	0.086	0.006	-0.29	-0.03	-0.44	-0.01	0.226	-0.837	-0.276	0.2101	0.1941	-0.248	-0.193	-0.119	0.2834	0.3488	-0.17	-1.02	-0.54	-0.87	-0.14	-0.1	-0.14	0.144	0.524	0.463687	0.283113	0.098365611	0.184747153
80	A0A087WZC	AC008878	Rho/Rac guanine nucleotide exchange facto	23370	ENSG00000268861	2	-0.23	-0.89	-0.36	-0.51	-0.15	-0	0.004	0.087	-0.03	0.0393	0.3191	-0.226	-0.535	0.2741	0.0178	-0.412	-0.767	-0.543	0.037	1.037	0.495	0.168	0.163	-0.21	0.428	-0.33	-0.27	0.199638	-0.40038	-0.02815664	-0.372221852
2794	Q7Z6Z7	HUWE1	HECT, UBA and WWE domain containing E	10075	ENSG00000086758	44	-0.27	-0.07	-0.19	-0.02	0.186	-0.2	-0.08	-0.01	0.025	-0.06	-0.187	-0.116	0.0755	0.0134	-0.008	-0.024	0.2117	0.1292	-0.39	-0.63	-0.18	-0.35	-0.18	0.032	0.187	-0.05	0.03	0.294431	0.100047	-0.073933458	0.173980503
3652	Q9UL26	RAB22A	RAB22A, member RAS oncogene family	57403	ENSG00000124209	10	-0.18	0.068	-0.01	0.101	0.037	-0.41	0.169	0.193	0.177	0.0237	0.1261	0.0754	0.1766	-0.161	0.245	0.1567	0.1152	0.1108	-0.41	-0.47	-0.24	-0.61	-0.54	-0.12	-0.02	-0.04	0.331	0.099846	0.252872	-0.081040995	0.33391278
1228	O75828	CBR3	carbonyl reductase 3	874	ENSG00000159231	13	-0.07	0.178	-0.28	-0	-0.1	-0.49	0.265	0.184	-0.01	-0.085	0.1306	0.1292	0.206	-0.121	-0.076	-0.115	0.4238	0.2657	-0.3	-1.25	-0.16	-0.27	-0.7	0.109	0.118	0.3	0.248	0.353254	0.176325	-0.120063985	0.296389303
3466	Q9NQR4	NIT2	nitrilase family member 2	56954	ENSG00000114021	20	0.188	0.245	-0.01	-0.05	0.193	-0.28	0.055	-0.12	0.177	0.1303	0.1098	0.2411	-0.017	-0.107	0.1749	0.0271	0.1676	-0.075	-0.13	-0.99	-0.11	-0.51	-0.28	0.184	-0.14	0.094	0.308	0.240211	0.220598	-0.026737483	0.247335666
2654	Q5VST6	ABHD17B	abhydrolase domain containing 17B	51104	ENSG00000107362	13	-0.01	0.23	0.02	-0.02	-0.13	0.026	0.059	-0.11	0.149	0.2415	0.1897	0.0984	-0.073	-0.192	0.1464	0.1405	0.1728	0.152	-0.17	-0.86	-0.17	-0.44	-0.22	0.16	-0.08	0.023	0.322	0.193802	0.182693	-0.073771372	0.256464817
1568	P12081	HARS;HAF	histidyl-tRNA synthetase;histidyl-tRNA synth	3035	ENSG00000170445	17	0.15	0.193	0.041	-0.1	0.111	-0.44	0.178	0.072	0.073	0.1076	0.1456	0.1849	0.1106	-0.241	0.0255	0.2074	0.116	0.0298	-0.05	-0.64	0.171	-0.36	-0.26	0.05	0.121	0.114	0.117	0.440695	0.113207	-0.045050921	0.158257816
747	F8VXU5	VPS29	VPS29 retromer complex component;VPS2	51699	ENSG00000111237	11	0.086	0.022	-0.03	0.147	0.074	-0.43	0.163	0.065	0.258	0.0718	0.0484	-0.076	0.013	-0.048	0.0895	0.1745	0.1972	0.1995	-0.25	-1.04	-0.22	-0.39	-0.45	0.072	0.153	-0.09	0.082	0.134207	0.278229	-0.034413297	0.312641887
735	F8VU65	RPLP0	ribosomal protein lateral stalk subunit P0	6175	ENSG00000089157	8	0.106	-0.04	-0.07	0.022	0.243	-0.29	0.033	-0.08	0.21	-0.129	-0.137	-0.037	-0.194	-0.204	-0.03	-0.249	-0.07	0.0011	-0.23	-0.44	-0.02	-0.2	-0.07	-0.01	0.152	-0.02	0.129	0.346574	0.092898	0.130775494	-0.037877215
878	H7BYY1	TPM1	tropomyosin 1	7168	ENSG00000140416	20	0.224	-0.19	-0.31	-0.22	0.097	-0.31	-0	0.068	0.241	-0.2	0.1045	-0.089	0.0293	-0.153	0.1014	0.0245	0.1233	0.0245	-0.22	-0.28	-0.08	-0.7	-0.12	-0.24	0.088	-0.24	-0.11	0.198506	0.167939	-0.040224412	0.208163613

3483	Q9NRF8	CTPS2	CTP synthase 2	56474	ENSG00000047230	18	0.216	0.143	-0.14	-0.11	0.058	-0.41	-0.18	-0.21	0.032	0.0747	0.0795	0.0582	0.0827	-0.093	0.0957	0.0537	0.009	0.1128	-0.2	-0.86	-0.27	-0.57	-0.36	-0.02	0.172	-0.05	-0.04	0.138338	0.178134	-0.119629031	0.297762729
3626	Q9UIA9	XPO7	exportin 7	23039	ENSG00000130227	20	0.082	0.139	0.073	0.061	0.201	-0.22	0.083	-0.09	0.255	0.2073	-0.065	0.0224	0.0447	-0.064	-0.105	0.0258	0.0561	-0.054	-0.12	-0.36	-0.14	-0.19	-0.26	0.008	-0.09	-0.2	-0	0.050624	0.214704	0.057098925	0.157604908
769	F8WCF6	ARPC4-TT	ARPC4-TTLL3 readthrough	100526693	ENSG00000250151	10	0.203	0.147	-0.03	0.03	-0.04	-0.34	-0.16	-0.26	-0.11	0.1634	-0.11	-0.051	-0.031	-0.093	-0.122	-0.09	-0.093	-0.081	-0.24	-0.63	-0.12	0.154	-0.41	-0.04	-0.02	-0.09	-0.09	0.515927	0.101852	-0.005762191	0.107614165
1157	O60493	SNX3	sorting nexin 3	8724	ENSG00000112335	12	0.061	0.156	0.11	0.088	-0.23	-0.31	0.121	-0.13	0.148	-0.135	-0.097	0.2615	0.0315	0.1349	0.2642	-0.032	0.131	0.2407	-0.21	-0.53	-0.3	-0.13	-0.04	0.044	-0.12	-0.06	0.238	0.201989	0.125839	-0.087181432	0.213020922
1025	O00622	CCN1;CYF	cellular communication network factor 1;cys	3491	ENSG00000142871	14	-0.6	0.15	-0.56	-0.38	-0.46	-0.32	-0.65	-0.08	0.001	0.0269	-0.056	0.0556	-0.539	-0.05	0.0485	0.7021	0.132	0.5656	-0.03	-0.12	-0.05	-0.45	-0.71	0.262	0.366	0.017	-0.17	0.157476	-0.22381	-0.420125557	0.19631071
1663	P18085	ARF4	ADP ribosylation factor 4	378	ENSG00000168374	14	0.349	0.376	0.325	0.021	0.082	-0.27	-0.45	-0.31	-0.08	0.3804	0.398	0.1698	0.3194	-0.211	0.1888	-0.332	-0.068	-0.402	-0.25	-1.02	0.197	-0.57	-0.15	0.356	-0.35	-0.04	0.041	0.450325	0.203752	-0.043254565	0.247006881
2311	Q04446	GBE1	1,4-alpha-glucan branching enzyme 1	2632	ENSG00000114480	15	-0.25	-0.15	-0.32	-0.34	-0.38	-0.25	0.054	0.052	0.151	0.2713	-0.155	0.1427	-0.238	0.1734	-0.609	0.2219	0.151	0.2314	-0.32	-0.67	-0.02	0.442	0.083	-0.33	0.075	-0	0.216	0.559254	-0.09919	-0.179677192	0.080484702
3624	Q9UI12	ATP6V1H	ATPase H+ transporting V1 subunit H	51606	ENSG00000047249	21	0.08	0.192	-0.09	-0.02	-0.02	-0.29	-0.23	-0.19	0.167	0.1452	0.199	0.0758	0.0105	-0.234	0.0801	0.1488	0.0863	0.0634	-0.15	-0.94	-0.33	-0.51	-0.38	0.056	#####	-0.05	0.152	0.146511	0.194921	-0.10810239	0.303023497
2411	Q13464	ROCK1	Rho associated coiled-coil containing protei	6093	ENSG00000067900	42	0.141	0.17	-0.04	-0.07	0.04	-0.24	-0.15	-0.19	0.017	0.1101	0.0231	0.086	0.1249	-0.118	0.1273	0.0329	0.1637	-0.045	-0.25	-0.66	-0.26	-0.03	-0.19	0.096	0.022	-0.07	0.07	0.193421	0.106057	-0.092604104	0.198660888
3044	Q92696	RABGGTA	Rab geranylgeranyltransferase subunit alpha	5875	ENSG00000100949	27	0.051	0.083	-0.02	-0.05	-0.15	-0.23	-0.12	-0.02	0.037	0.1364	-0.003	0.0774	0.0806	-0.132	-0.066	-0.065	-0.106	0.0271	-0.14	-0.58	-0.08	-0.07	-0.13	0.044	0.106	-0.07	-0.08	0.433729	0.06364	-0.041755056	0.105394681
3785	Q9Y646	CPQ	carboxypeptidase Q	10404	ENSG00000104324	12	-0.2	-0.54	0.189	-0.54	-0.58	-0.18	-0.23	-0.16	0.002	0.0892	0.1208	-0.027	-0.362	0.619	-0.417	-0.661	-0.209	-0.246	-0.19	0.305	0.229	-0.24	-0.1	-0.1	0.516	0.222	-0.4	0.356445	-0.27496	-0.126095726	-0.148860106
2638	Q5T4S7	UBR4	ubiquitin protein ligase E3 component n-reco	23352	ENSG00000127481	42	0.116	0.138	-0.12	-0.05	0.179	-0.3	-0.02	0.042	0.141	0.0428	0.0214	0.0151	-0.056	-0.232	-0.113	0.0827	0.1653	0.2675	-0.11	-0.19	0.095	0.074	-0.3	-0.04	0.266	-0.06	0.163	0.933194	0.025448	-0.007812481	0.033260929
623	E7EX17	EIF4B	eukaryotic translation initiation factor 4B	1975	ENSG00000063046	22	0.081	0.159	0.012	-0.06	0.093	-0.09	0.167	0.079	0.35	-0.032	0.0471	0.0465	-0.039	-0.198	0.1587	0.2378	0.1435	0.021	-0.18	-0.34	-0.09	-0.34	-0.37	0.027	0.168	0.106	0.089	0.175127	0.190777	0.044858165	0.14591931
1949	P46779	RPL28	ribosomal protein L28	6158	ENSG00000108107	10	0.229	0.019	-0.16	-0.04	0.841	0.007	0.256	0.097	0.124	0.0271	-0.234	-0.15	-0.124	-0.111	-0.071	0.3533	-0.072	0.2138	-0.21	-0.62	-0.07	0.039	0.03	0.203	0.386	0.027	-0.07	0.412271	0.183574	0.17130573	0.012268387
2328	Q06828	FMOD	fibromodulin	2331	ENSG00000122176	11	0.12	-0.03	-0.09	-1.05	-0.06	-0.61	-0.54	0.373	-0.12	-0.099	0.0412	-0.037	-0.872	-7E-04	-0.968	-0.593	1.2405	0.1766	-0.13	-0.2	0.321	-0.05	-0.82	0.003	0.317	-0.21	-0.53	0.937613	-0.07728	-0.099204683	0.02192362
2157	P61163	ACTR1A	actin related protein 1A;ARP1 actin related p	10121	ENSG00000138107	14	0.214	0.181	-0	0.03	0.319	-0.24	0.102	-0	0.142	0.1715	0.1108	0.0321	0.0052	-0.169	0.1334	0.2236	-0.086	-0.07	-0.05	-0.73	-0.16	-0.39	-0.23	0.004	0.159	0.07	0.081	0.199751	0.222313	0.043715034	0.178597684
1975	R4GMR5	PSMD8	proteasome 26S subunit, non-ATPase 8	5714	ENSG00000099341	14	-0.02	-0.07	-0.13	-0.07	0.227	-0.45	-0.02	-0.08	0.169	0.0024	-0.044	-0.08	0.3449	-0.114	-0.176	0.0197	0.0538	0.1644	-0.14	-0.78	0.088	-0.23	-0.01	0.017	0.158	-0.09	0.23	0.72805	0.035269	-0.067709846	0.102978562
3207	Q99523	SORT1	sortilin 1	6272	ENSG00000134243	12	0.057	-0.03	0.712	0.268	0.272	0.107	0.121	0.629	0.085	0.3326	0.0346	0.3413	0.1293	-0.751	-0.38	-0.002	-0.076	-0.445	-0.52	0.733	-0.41	0.648	-0.49	-0.65	-0.05	-0.45	-0.52	0.205239	0.437361	0.337528482	0.099832792
1728	P23352	ANOS1	anosmin 1	3730	ENSG00000011201	13	-0.02	-0.03	0.303	-0.65	-0.34	-0.45	-0.45	0.146	0.033	0.1498	0.6398	0.0528	-0.632	0.1682	-0.562	0.2084	0.0576	0.1493	-0.01	0.786	0.252	-0.13	-0.51	0.02	0.639	-0.19	-0.41	0.597692	-0.21177	-0.187330898	-0.024435946
495	C9J5N1	PTGES3L-	PTGES3L-AARSD1 readthrough	100885850	ENSG00000108825	10	-0.14	6E-04	0.215	0.395	0.396	0.011	0.08	0.236	0.195	-0.192	-0.17	-0.279	-0.315	-0.215	-0.173	-0.246	0.1199	-0.02	0.52	-0.59	0.196	-0.19	-0.35	-0.13	-0.16	0.028	0.026	0.122443	0.226393	0.319337281	-0.092943817
339	A0A2R8YE6	EPS8	epidermal growth factor receptor pathway su	2059	ENSG00000151491	28	0.122	0.445	-0.29	-0.08	0.144	0.136	-0.15	0.046	-0.15	-0.249	-0.077	0.226	-0.071	-0.137	-0.022	0.0205	-0.15	0.221	0.169	0.465	-0.41	0.085	-0.32	-0.42	-0.11	-0.24	0.319	0.870552	0.07517	0.05039725	0.024772391
1882	P36959	GMPR	guanosine monophosphate reductase	2766	ENSG00000137198	15	0.068	0.05	-0.41	0.035	-0.14	-0.41	-0.09	0.012	0.088	-0.207	-0.261	-0.146	-0.161	0.0839	0.1012	0.1111	0.0373	-0.049	-0.3	-0.55	-0.29	-0.77	-0.34	-0.23	-0.18	0.126	0.022	0.203663	0.190999	-0.033378526	0.22437756
3131	Q96FZ7	CHMP6	charged multivesicular body protein 6	79643	ENSG00000176108	6	-0.03	0.255	0.005	0.164	-0.05	-0.2	0.394	0.322	0.542	0.0116	0.1599	0.0944	-0.095	-0.371	0.1453	0.5089	0.3637	0.521	-0.4	-0.73	-0.43	-0.52	-0.37	-0.09	0.238	0.221	0.588	0.218536	0.322073	0.007753103	0.31432
3496	Q9NS15	LTBP3	latent transforming growth factor beta bindin	4054	ENSG00000168056	23	-0.02	-0.71	-0.24	-0.23	-0.22	0.03	-0.2	-0.04	-0.17	0.3371	0.0768	0.2652	-0.551	0.4763	-0.128	-0.185	-0.74	-0.146	0.22	0.501	0.346	-0.08	-0.03	-0.08	0.64	-0.03	-0.42	0.286377	-0.31968	-0.134345271	-0.18533948
2851	Q8IWE2	FAM114A1	family with sequence similarity 114 member	92689	ENSG00000197712	14	0.268	0.047	-0.13	-0.18	0.143	-0.44	-0.27	0.004	0.07	0.1872	0.0694	0.019	0.0059	0.0447	0.3693	-0.086	0.122	0.1777	-0.1	-0.92	-0.31	-0.33	-0.21	0.028	-0.11	-0.03	0.393	0.219215	0.122692	-0.15560643	0.278298694
3148	Q96IU4	ABHD14B	abhydrolase domain containing 14B	84836	ENSG00000114779	8	0.086	0.229	0.116	-0.09	-0.16	-0.28	0.073	-0	0.136	0.1389	0.2254	0.1857	0.0088	-0.061	0.0898	0.0422	0.1881	0.031	-0.16	-0.67	-0.08	-0.17	-0.23	0.042	0.08	0.159	0.351	0.367338	0.087716	-0.0823431	0.170058808
2127	P59666	DEFA3	defensin alpha 3	1668	ENSG00000284835	2	-0.96	-1.05	-1.02	-1.07	-1.74	0.375	-1.51	-2.5	-3.36	-1.06	-0.272	-1.115	-0.079	-1.118	-1.368	-0.64	-3.282	-3.195	-0.78	-1.51	1.907	3.142	-0.71	1.025	-2.17	-2.65	-3.25	0.572014	-0.87122	-0.078748004	-0.792467021
3581	Q9UBI6	GNG12	G protein subunit gamma 12	55970	ENSG00000172380	7	-0.13	0.287	-0.22	-0.51	-1.2	0.948	-0.41	-0.17	0.127	-0.388	-0.115	0.1435	-0.755	0.0586	-0.657	0.0202	0.0596	0.1313	-0.55	-0.37	-0.5	-0.26	0.18	-0.51	0.041	-0.04	0.108	0.96043	0.069987	0.024714736	0.045271953
3064	Q93008	USP9X	ubiquitin specific peptidase 9 X-linked	8239	ENSG00000124486	37	0.013	0.029	-0.1	-0.01	0.027	-0.23	-0.12	-0.03	-0.04	0.115	-0.01	0.036	0.2134	-0.035	0.046	0.143	0.1266	0.1058	-0.14	-0.31	-0.05	-0.23	-0.15	0.194	0.145	0.033	-0.05	0.138595	0.012586	-0.133597902	0.146184044
554	D3YTB1	RPL32	ribosomal protein L32	6161	ENSG00000144713	5	0.012	-0.37	-0.21	0.106	1.1	-0.12	0.118	-0.28	-0.17	-0.173	-0.003	-0.215	0.1	-0.296	0.0845	-0.154	-0.218	-0.194	-0.12	-0.34	0.148	-0.01	0.098	0.219	0.275	-0.39	-0.22	0.734681	0.058605	0.138546381	-0.079940887
2364	Q12805	EFEMP1	EGF containing fibulin extracellular matrix pr	2202	ENSG00000115380	12	-0.05	-0.09	-0.67	0.361	-0.04	-0.67	-0.69	0.188	-0.23	-0.21	0.1503	0.0615	-0.206	-0.006	-0.133	0.5897	0.0551	0.4917	-0.65	-0.33	-0.26	-0.37	-0.17	0.049	0.318	0.095	-0.05	0.291332	-0.05716	-0.298039533	0.240875856
3634	Q9UJ70	NAGK	N-acetylglucosamine kinase	55577	ENSG00000124357	14	-0	0.19	0.029	-0.01	-0.45	-0.33	0.138	0.191	0.188	0.2806	0.0547	0.1132	-0.027	-0.08	0.0847	0.0292	0.2647	0.2897	-0.14	-0.52	-0.2	-0.36	-0.33	-0.02	-0.07	0.016	0.398	0.20867	0.131374	-0.118338218	0.249712405
3679	Q9UNS2	COPS3	COP9 signalosome subunit 3	8533	ENSG00000141030	14	0.02	0.064	-0.13	-0.21	0.295	-0.46	0.073	0.067	0.1	0.1108	0.0459	0.096	0.058	-0.039	0.115	0.2432	0.1759	0.2656	-0.18	-0.44	-0.25	-0.13	-0.3	0.111	0.286	-0.02	0.057	0.203289	0.076773	-0.139070469	0.215843329
3604	Q9UGV2	NDRG3	NDRG family member 3	57446	ENSG00000101079	11	-0.14	-0.64	-0.25	-0.11	-0.25	0.017	0.103	0.066	0.266	-0.472	0.0434	-0.266	-0.178	-0.132	0.1288	0.0843	-0.099	0.3623	0.5	0.544	0.285	-0.48	0.022	0.012	-0.07	-0.03	0.312	0.365223	-0.22526	-0.044426482	-0.180831438
1149	O60256	PRPSAP2	phosphoribosyl pyrophosphate synthetase a	5636	ENSG00000141127	13	0.061	-0.06	-0.11	-0.25	-0.07	-0.36	-0.12	-0.18	0.188	-0.064	0.1025	0.0786	0.0315	-0.1	0.3259	0.0002	-0.034	0.0643	-0.24	-0.25	-0.16	-0.45	-0.19	-0.03	0.133	0.05	0.267	0.305119	-0.00543	-0.145526441	0.140097637
2939	Q8NES3	LFNG	LFNG O-fucosylpeptide 3-beta-N-acetylgluc	3955	ENSG00000106003	13	-0.14	-0.1	0.326	-0.72	-0.01	-0.43	-0.08	0.042	0.55	-0.192	0.3368	-0.061	-0.169	-0.083	-0.508	0.1973	0.3816	0.2804	0.147	0.247	0.095	-0.27	-0.82	-0.14	0.304	0.236	0.275	0.911611	-0.06926	-0.082127534	0.012862908
346	A0A2U3TZH	EEF1A2	eukaryotic translation elongation factor 1 alp	1917	ENSG00000101210	19	0.128	0.08	-0.18	-0.53	0.325	-0.09	0.107	-0.06	-0.37	0.485	0.4033	0.2095	0.079	-0.128	0.2923	0.2777	0.0673	0.0529	-0.26	-0.62	0.042	-0.98	-0.77	-0.19	-0.12	0.132	-0.08	0.068348	0.2501	-0.259908561	0.510008897
3233	Q99961	SH3GL1	SH3 domain containing GRB2 like 1, endop	6455	ENSG00000141985	19	-0.05	0.164	-0.15	-0.09	0.224	-0.45	-0.13	-0.03	0.099	0.0196	0.0566	0.079	0.0484	-0.131	0.1695	0.0282	0.0995	0.0288	-0.25	-0.66	-0.31	-0.3	-0.26	-0.02	-0.17	0.113	0.113	0.159465	0.148106	-0.089817886	0.237923644
1682	P20042	EIF2S2	eukaryotic translation initiation factor 2 subu	8894	ENSG00000125977	19	-0.03	-0.02	-0.19	-0.21	-0.04	-0.33	0.23	0.152	0.312	-0.081	-0.24	-0.077	-0.028	-0.028	0.1709	0.2546	0.1755	0.2186	-0.31	-0.75	-0.29	-0.37	-0.03	0.046	0.277	0.149	0.403	0.665233	0.082221	-0.055545115	0.137765957
1885	X6RJP6	TAGLN2	transgelin 2	8407	ENSG00000158710	19	-0.04	0.04	-0.07	-0.05	-0.09	-0.47	-0.04	-0.26	0.059	0.2704	0.213	0.1865	-0.036	-0.055	0.2896	-0.006	0.3995	-0.159	-0.2	-0.72	0.253	-0.57	-0.73	-0.13	-0.13	-0.07	0.302	0.146966	0.118596	-0.225925766	0.344521614
1934	P43686	PSMC4	proteasome 26S subunit, ATPase 4	5704	ENSG00000281221	18	-0.23	-0.3	-0.26	0.057	0.384	-0.43	0.062	0.115	0.11	-0.208	-0.263	-0.099	0.053	-0.079	0.0183	0.0025	0.1196	0.1639	-0.25	-0.28	-0.14	-0.3	-0.31	-0.01	0.135	-0.08	0.153	0.745356	0.065067	-0.022716294	0.087783167
145	A0A0A0MR.	PCMT1	protein-L-isoaspartate (D-aspartate) O-meth	5110	ENSG00000120265	11	0.013	0.275	0.028	0.149	0.114	-0.38	0.024	-0.06	0.165	0.0441	0.1042	0.0304	-1E-04	-0.034	0.0752	0.0879	0.2931	0.0272	-0.2	-0.67	-0.18	-0.51	-0.51	-0.03	0.017	0.164	0.137	0.1384	0.233981	-0.033705368	0.267686829
3473	Q9NR19	ACSS2	acyl-CoA synthetase short chain family men	55902	ENSG00000131069	13	0.331	0.18	0.079	-0.21	-0.11	-0.81	-0.01	0.033	0.143	0.2352	-0.091	0.0732	0.0354	-0.235	-0.029	-0.06	0.286	0.0818	-0.26	-0.76	-0.14	-0.67	-0.07	0.009	-0.02	-0.16	0.183	0.340663	0.168876	-0.074332125	0.243207964
1730	P23396	RPS3	ribosomal protein S3	6188	ENSG00000149273	18	0.205	-0.16	-0.15	0.213	0.135	-0.14	0.106	-0.03	0.125	-0.103	-0.048	-0.029	-0.147	-0.086	-0.216	0.0458	0.0236	0.0623	-0.09	-0.43	0.029	-0.28	-0.08	-0.01	0.224	-0.21	-0.01	0.356898	0.129429	0.089229422	0.040199532
2291	Q01581	HMGCS1	3-hydroxy-3-methylglutaryl-CoA synthase 1	3157	ENSG00000112972	14	0.007	-0.07	-0.14	-0.29	-0.27	-0.52	0.191	0.272	0.315	0.1849	-0.237	-0.214	-0.126	-0.05	-0.102	0.091	0.2139	0.2642	-0.18	-0.66	0.032	-0.52	-0.14	-0.01	0.186	0.127	0.207	0.781201	0.04941	-0.05934344	0.108753146
1879	P36952	SERPINB5	serpin family B member 5	5268	ENSG00000206075	16	-0.11	-0.2	-0.7	0.696	-0.02	-0.16	0.289	-0.39	-0.28	-0.549	0.3407	0.1566	0.4026	-0.19	-0.113	0.4154	0.1841	-0.345	-0.2	-0.9	-0.38	-0.51	-0.39	0.374	-0.52	-0.18	0.17	0.380778	0.18455	-0.131076157	0.315625786
2359	Q12756	KIF1A	kinesin family member 1A	547	ENSG00000130294	22	-0.13	0.096	-0.03	0.289	-0.18	0.474	-0.01	-0.03	0.01	0.3386	0.0275	-0.051	0.0746	0.0464	0.1888	0.2649	0.1149	0.0582	0.148	0.198	0.202	-0.29	-0.43	-0.24	0.056	0.02	0.202	0.52474	0.070627	-0.062602365	0.133228958
465	B7ZC38	SH3GLB2	SH3 domain containing GRB2 like, endophil	56904	ENSG00000148341	14	0.052	0.198	-0.15	-0.06	-0.35	-0.49	0.121	-0.07	0.285	0.2386	0.0696	0.0606	0.0108	-0.058	0.3285	0.0191	0.0538	0.0235	-0.22	-1.1	-0.11	-0.76	-0.29	0.125	-0.08	0.02	0.148	0.202196	0.201501	-0.134304413	0.335805699
3737	Q9Y3B8	REXO2	RNA exonuclease 2	25996	ENSG00000076043	9	0.01	0.028	-0.12	0.017	0.306	-0.3	0.227	0.02	0.045	0.1236	0.0852	0.0147	0.2143	-0.163	0.2275	-0.104	0.0919	-0.192	-0.18	-0.05	0.129	-0.35	-0.26	0.195	-0.16	-0.1	0.164	0.560324	0.093263	-0.007114528	0.100377257
3481	Q9NRD5	PICK1	protein interacting with PRKCA 1	9463	ENSG00000100151	13	0.309	0.05	0.067	-0.11	-0.08	-0.21	0.127	-0.03	0.104	-0.045	0.1606	0.1245	-0.063	0.0285	-0.046	0.3338	0.097	0.211	-0.11	-0.96	-0.31	-0.43	-0.3	0.019	0.146	-0.06	0.282	0.181686	0.216	-0.063833449	0.279833388
3059	Q92905	COPS5	COP9 signalosome subunit 5	10987	ENSG00000121022	13	0.132	0.086	-0.03	-0.03	0.126	-0.42	-0.01	-0.06	0.09	0.2421	0.1724	0.0161	-8E-04	-0.149	0.0188	0.1769	0.11	0.1153	-0.1	-0.72	-0.25	-0.31	-0.25	0.085	0.196	0.034	0.029	0.21776	0.130273	-0.090994313	0.221267265
148	B6VEX4	ABI1	abl interactor 1	10006	ENSG00000136754	10	-0.07	-0.01	0.003	-0.24	0.051	-0.33	0.208	0.051	0.345	-0.062	-0.043	0.1441	0.0512	-0.229	0.2528	0.2453	0.0007	-0.022	-0.24	-0.47	0.019	-0.11	-0.2	-0.03	0.167	0.06	0.088	0.554124	0.082443	-0.035422977	0.117865495
1182	O60884	DNAJA2	DnaJ heat shock protein family (Hsp40) mer	10294	ENSG00000069345	15	0.201	0.433	0.064	0.05	0.19	-0.45	-0.06	-0.11	0.096	0.2153	0.2435	0.1743	0.1128	-0.168	0.0987	0.047	0.0974	0.1003	-0.21	-0.7	-0.3	-0.47	-0.34	-0.01	-0.1	-0.09	0.18	0.0949	0.271732	-0.056020479	0.327752382
1406	P04049	RAF1	Raf-1 proto-oncogene, serine/threonine kina	5894	ENSG00000132155	12	0.082	-0	-0.03	-0.06	-0.19	-0.23	-0.04	-0	0.132	0.0589	-0.032	0.1226	0.0139	-0.04	0.0405	-0.058	0.1097	0.0698	-0.12	-0.65	-0.28	0.021	-0.17	0.181	-0.07	-0.01	0.225	0.444027	0.060674	-0.068987632	0.129661715
925	J3KS94	MBP	myelin basic protein	4155	ENSG00000197971	4	-0.27	0.491	0.197	-0.24	-0.16	-0.29	0.063	-0.02	0.064	-0.318	-0.039	0.2442	0.076	-0.115	-0.093	0.1061	0.2905	0.1012	-0.46	-0.59	-0.23	-0.51	-0.62	-0.12	0.044	0.654	1.193	0.916677	0.053687	-0.045670282	0.099357535
305	A0A2C9F2P	PPT1	palmitoyl-protein thioesterase 1	5538	ENSG00000131238	8	0.01	-0.41	-0.11	-0.77	-0.34	-0.56	-0.02	-0.13	0.242	0.2008	0.0734	-0.309	-0.435	0.2118	-0.38	0.0167	-0.304	-0.214	0.617	0.457	0.166	-0.14	0.022	0.086	0.28	0.03	-0.1	0.121261	-0.38974	-0.105474962	-0.28426568
2403	Q13409	DYNC1I2	dynein cytoplasmic 1 intermediate chain 2	1781	ENSG00000077380	16	-0.02	0.012	-0.13	-0.24	0.143	-0.31	-0.39	-0.47	-0.25	0.0161	0.0878	-0.002	-0.092	-0.128	0.1441	-0.345	-0.023	-0.364	0.03	-0.54	-0.16	-0.45	-0.12	-0.08	-0.11	-0.15	-0.1	0.543068	0.003625	-0.104886383	0.108510931
3020	Q8WWI5	SLC44A1	solute carrier family 44 member 1	23446	ENSG00000070214	10	-0	0.32	0.964	0.004	0.134	0.196	-0.08	0.253	0.017	-0.258	0.473	0.4027	0.3766	-0.362	0.1069	-0.207	0.2401	-0.139	-0.64	0.332	-0.54	-0.01	-0.65	-0.74	0.023	-0.12	-0.36	0.103304	0.502007	0.130054836	0.371952304
3707	Q9Y281	CFL2	cofilin 2	1073	ENSG00000165410	13	0.221	0.246	-0.02	0.116	0.177	-0.68	0.253	0.198	0.246	-0.069	-0.266	-0.069	-0.144	0.121	0.1288	0.2483	0.4084	0.1983	-0.27	-0.79	-0.49	-0.79	-0.5	-0.08	-0.34	0.072	0.493	0.144992	0.384933	0.022750436	0.362182829
1158	O60502	OGA	O-GlcNAcase	10724	ENSG00000198408	15	-0.08	0.08	-0.08	-0.11	0.121	-0.38	0.024	-0.03	0.164	0.1758	-0.04	0.1743	0.0776	-0.059	0.2035	0.1367	0.2842	0.3903	-0.03	-0.44	-0.17	-0.34	-0.3	-0.07	0.128	0.206	0.205	0.14623	0.057694	-0.182990786	0.240684292
1060	O14966	RAB29	RAB29, member RAS oncogene family	8934	ENSG00000117280	12	-0.13	0.43	0.041	0.094	-0.09	-0.08	0.029	0.093	0.175	0.1485	0.071	0.0462	0.1408	0.0952	0.1953	-0.19	0.0227	-0.029	-0.15	-1.29	-0.57	-0.37	-0.31	0.259	-0.05	0.124	0.05	0.172082	0.319165	0.006886407	0.312278752
3784	Q9Y625	GPC6	glypican 6	10082	ENSG00000183098	14	-0.37	-0.19	-0.32	0.042	0.166	0.211	0.139	0.514	-0.28	0.4678	0.2877	-0.176	-0.357	-0.283	0.4728	0.3096	0.0891	0.5425	-0.01	-0.35	-0.18	-0.97	-0.91	0.331	0.037	-0.09	0.432	0.350536	0.179606	-0.160881528	0.340487188
934	J3QRS3	MYL12A	myosin light chain 12A	10627	ENSG00000101608	10	-0.06	0.08	-0.07	-0.36	0.29	-0.57	-0.47	-0.56	-0.31	0.21	0.1169	-0.24	0.1797	-0.117	0.3353	-0.168	-0.015	-0.597	-0.17	-1.14	0.091	0.011	-0.25	0.284	0.027	-0.16	-0.26	0.598875	-0.05252	-0.192667795	0.14014767
1141	O43852	CALU	calumenin	813	ENSG00000128595	7	0.236	-1.06	-0.23	-0.39	-0.08	-0.26	-0.15	0.159	0.149	0.2684	-0.348	-0.157	-0.288	0.1788	-0.2	0.1016	-0.703	-0.416	0.511	0.926	0.123	0.013	0.316	-0.17	0.293	-0.08	-0.28	0.204057	-0.36442	-0.007704707	-0.356711077
307	A0A2Q2TCH	LIPI	lipase I	149998	ENSG00000188992	12	0.075	-0.58	0.546	0.433	0.385	0.213	-0.14	0.683	-0.12	-0.502	0.2699	0.9882	0.5013	-1.222	0.6226	0.0684	-0.173	-0.667	-0.96	-0.86	0.387	-1.01	-1.46	-1.63	-0.7	0.165	-1.08	0.08191	0.962349	0.179727936	0.78262137
2920	Q8NBF2	NHLRC2	NHL repeat containing 2	374354	ENSG00000196865	15	0.21	0.131	0.018	-0.15	-0.09	-0.37	-0.38	-0.38	-0.03	0.1691	0.0571	0.0209	-0.044	0.0577	-0.023	-0.235	-0.113	-0.194	0.156	-0.77	-0.08	-0.36	-0.27	-0.01	-0.11	-0.11	-0.1	0.546307	0.057988	-0.081668782	0.139657064
996	O00231	PSMD11	proteasome 26S subunit, non-ATPase 11	5717	ENSG00000108671	19	-0.12	-0.04	-0	-0.1	0.347	-0.55	-0.06	-0.26	0.141	0.1137	-0.156	-0.01	-0.054	-0.215	0.0053	-0.053	0.1886	0.0909	-0.27	-0.29	-0.21	-0.5	-0.26	-0.09	0.15	-0.04	-0.04	0.388007	0.099783	-0.060967908	0.160750678
869	H7BXD5	GCA	grancalcin	25801	ENSG00000115271	8	-0.13	0.022	-0.03	0.051	-0	-0.44	-0	-0.35	0.082	0.1073	-0.119	-0.002	0.1095	-0.16	0.0863	0.173	0.0961	0.2194	-0.09	-0.39	0.259	0.027	-0.27	-0.07	-0.12	-0.1	0.021	0.286173	-0.00759	-0.145361294	0.13777479
468	B7ZKQ9	SCARB1	scavenger receptor class B member 1	949	ENSG00000073060	9	-0.25	0.651	-0.11	-0.22	-0.17	-0.59	-0.02	0.062	-0.23	0.0058	-0.117	-0.013	0.0823	-0.049	-0.017	0.1378	0.1789	0.3258	-0.2	-0.17	-0.25	-0.38	-0.47	-0.18	0.129	0.055	0.065	0.318653	0.058297	-0.156862591	0.215159651
2617	Q5KU26	COLEC12	collectin subfamily member 12	81035	ENSG00000158270	16	-0.97	0.09	-0.24	-0.32	0.421	-0.92	-0.14	-0.15	0.065	0.3805	-0.874	-0.078	0.5727	-0.118	-0.285	0.239	0.5491	-0.08	-0.21	0.291	-1.11	-0.38	-0.57	0.396	0.512	0.623	-0.31	0.638991	-0.15756	-0.275982794	0.118423774
2525	Q15477	SKIV2L	Ski2 like RNA helicase	6499	ENSG00000225737	22	-0.12	-0.06	-0.13	0.006	-0.15	-0.37	-0	-0.03	-0.2	-0.188	-0.185	0.1138	0.0474	0.0314	-0.098	-0.007	-0.07	0.04	-0.24	-0.47	-0.2	-0.32	-0.23	-0.05	-0.02	0.178	-0.45	0.226244	0.083149	-0.082157837	0.165306624
2379	Q13131	PRKAA1	protein kinase AMP-activated catalytic subu	5562	ENSG00000132356	16	0.028	0.085	-0.06	-0.03	0.082	-0.33	0.174	0.086	0.325	0.055	-0.079	0.1344	0.0615	-0.199	0.057	0.4508	0.2113	0.3341	-0.38	-0.91	-0.36	-0.15	-0.02	0.073	0.307	0.262	0.391	0.487337	0.126843	-0.074484949	0.201328382
3794	Q9Y6I3	EPN1	epsin 1	29924	ENSG00000063245	9	-0.13	0.024	-0.23	-0.21	0.136	-0.52	0.04	-0.23	-0.11	-0.055	-0.037	-0.143	-0.099	0.2265	0.0826	0.2695	-0.199	-0.097	-0.05	-0.22	-0.14	-0.56	-0.17	-0.01	0.366	-0.06	0.005	0.547682	-0.04161	-0.129335483	0.087727042
1154	O60343	TBC1D4	TBC1 domain family member 4	9882	ENSG00000136111	29	0.606	-0.02	-0.03	-0.08	0.092	-0.36	-0.21	-0.06	0.357	0.0735	-0.091	0.4217	0.2035	-0.198	-0.188	0.0948	-0.137	0.2491	-0.1	-0.8	-0.14	-0.33	-0.13	0.244	0.173	0.047	0.084	0.589637	0.13827	-0.013311539	0.151581846
1251	O76083	PDE9A	phosphodiesterase 9A	5152	ENSG00000160191	21	-0.1	0.058	0.175	0.054	0.13	-0.47	0.064	0.462	-0.19	-0.108	-0.031	0.0065	0.0092	-0.086	0.0816	0.3759	0.2869	0.0717	-0.25	0.11	0.094	-0.51	-0.3	-0.05	0.024	-0.02	0.131	0.480252	0.107031	-0.046075753	0.153106475
3593	Q9UBX7	KLK11	kallikrein related peptidase 11	11012	ENSG00000167757	9	-0.43	-0.47	-0.24	0.199	-0.42	-0.04	0.095	-0.22	0.191	0.4917	0.233	0.1525	-0.425	-0.144	-0.003	0.8204	-0.32	-0.344	-0.43	0.215	-0.27	-0.02	-0.12	-0.36	-0.06	0.836	-0.27	0.641553	-0.09505	-0.199898682	0.104853536
402	Q99829	CPNE1	copine 1	8904	ENSG00000214078	10	-0.06	-0.52	0.05	-0.2	0.264	-0.42	0.122	-0.22	0.196	0.0598	-0.538	-0.338	0.3348	-0.46	0.2032	0.3836	0.2564	0.1631	-0.19	-0.32	-0.18	-0.08	-0.08	0.208	0.187	0.062	0.04	0.847934	-0.04741	-0.094555803	0.047142687
726	F6TR53	HS1BP3	HCLS1 binding protein 3	64342	ENSG00000118960	9	0.032	0.333	-0.2	0.068	-0.03	-0.37	-0.34	-0.41	-0.12	-0.204	0.0475	0.0599	-0.127	0.1129	0.2301	-0.004	-0.039	-0.226	-0.37	-0.89	-0.61	-0.63	-0.3	-0.11	-0.17	-0.01	0.331	0.225751	0.190904	-0.0985836	0.289487387
1400	P02794	FTH1	ferritin heavy chain 1	2495	ENSG00000167996	7	-0.11	-0.32	0.373	0.485	0.55	-0.22	-0.04	0.612	-0.18	-0.019	-0.327	0.0742	0.0678	0.0612	-0.053	-0.42	0.0557	0.0025	-0.18	-0.6	-0.01	-0.32	-0.04	0.266	-0.11	0.145	0.366	0.481295	0.180797	0.188370115	-0.007573401

1409	P04080	CSTB	cystatin B	1476	ENSG00000160213	9	0.117	0.308	0.104	-0.03	-0.18	-0.16	0.089	-0.09	0.185	-0.164	0.0501	-0.177	0.2729	0.1474	0.2573	0.2069	0.1637	-0.082	-0.09	-0.62	-0.26	-0.72	-0.51	0.255	0.045	0.181	0.263	0.290201	0.199314	-0.036346593	0.235661085
703	F5GZS6	SLC3A2	solute carrier family 3 member 2	6520	ENSG00000168003	19	-0.26	-0.06	0.177	-0.09	-0.1	-0.31	-0.31	0.149	-0.09	0.033	-0.013	-0.167	0.0174	-0.023	0.007	0.0445	0.05	0.2889	-0.27	-0.04	-0.34	-0.19	-0.24	-0.09	-0.01	-0.06	0.069	0.208547	0.031431	-0.125573896	0.157004876
37	A0A087WU(	GPX1	glutathione peroxidase 1	2876	ENSG00000233276	10	-0.23	0.078	-0.04	0.08	-0.04	-0.35	0.108	0.022	0.111	0.15	0.0908	0.0367	-0.067	-0.216	0.1831	0.0314	0.4519	0.0054	-0.05	-0.9	-0.12	-0.48	-0.36	-0.09	-0.11	0.106	0.094	0.169408	0.185609	-0.101789576	0.287398803
1398	P02790	HPX	hemopexin	3263	ENSG00000110169	15	-0.19	0.06	-0.31	-0.36	0.056	0.149	-0.42	0.035	-0.33	0.7276	-0.017	0.4054	-0.406	-0.043	-0.599	-0.003	-0.197	0.03	-0.37	-0.28	0.269	0.033	0.263	-0.16	-0.13	0.287	-0.46	0.75949	-0.0837	-0.134323959	0.050626065
3296	Q9BW91	NUDT9	nudix hydrolase 9	53343	ENSG00000170502	10	0.002	0.173	-0.03	-0.19	-0.22	-0.46	0.03	0.034	0.189	0.117	0.0469	0.0081	-0.257	-0.063	0.0928	0.0484	0.0466	-0.011	0.081	-0.47	0.005	-0.72	-0.45	-0.14	0.076	0.064	0.168	0.499724	0.100751	-0.056747068	0.157497729
3257	Q9BS26	ERP44	endoplasmic reticulum protein 44	23071	ENSG00000023318	11	0.092	-0.25	-0.09	-0.06	0.21	-0.23	-0.21	-0.04	0.128	-0.016	-0.041	-0.061	-0.196	0.0025	0.047	-0.186	-0.281	-0.198	-0.11	-0.09	0.137	-0.31	-0.25	0.071	-0.01	0.19	-0.2	0.823266	0.013066	0.05376969	-0.040704135
3379	Q9H3K6	BOLA2;BC	bolA family member 2;bolA family member 2	552900;654	ENSG00000183336	6	-0.17	0.072	-0.23	-0.11	-0.3	-0.16	0.376	0.168	0.081	-0.005	-0.11	-0.189	-0.107	0.1492	-0.079	0.5074	-0.433	-0.069	-0.17	-0.25	-0.18	-0.24	-0.26	-0.08	0.333	0.202	0.04	0.958326	0.036876	0.006568476	0.030307594
595	Q7L5N1	COPS6	COP9 signalosome subunit 6	10980	ENSG00000168090	11	0.142	0.093	-0	0.127	0.267	-0.38	-0.33	-0.4	0.112	0.1535	0.0831	0.0762	0.0321	-0.11	0.0901	-0.304	0.0009	-0.26	-0.11	-0.82	-0.07	-0.28	-0.36	0.041	-0.06	-0.2	-0.36	0.2402	0.202567	-0.015864305	0.218431508
1219	O75608	LYPLA1	lysophospholipase 1;lysophospholipase I	10434	ENSG00000120992	10	-0.09	0.181	-0.05	-0.2	-0.08	-0.54	0.172	0.02	0.385	0.1693	0.0506	-0.008	-0.048	-0.213	0.0851	0.3445	0.2064	0.2253	-0.24	-1.1	-0.37	-0.69	-0.54	0.048	0.075	0.068	0.366	0.208619	0.242783	-0.113205371	0.355988313
2427	Q13630	TSTA3	tissue specific transplantation antigen P35B	7264	ENSG00000104522	12	0.021	0.274	0.209	-0.11	-0.06	-0.53	-0.08	-0.22	0.206	-0.051	0.1271	0.0491	0.0346	-0.197	0.1019	-0.089	0.1359	0.0386	-0.23	-0.93	0.194	-0.32	-0.23	0.188	-0.14	-0.12	0.101	0.469964	0.130907	-0.049115027	0.180022205
1778	P28072	PSMB6	proteasome subunit beta 6	5694	ENSG00000142507	8	0.11	-0.05	0.058	-0.18	0.333	-0.34	-0.17	-0.24	0.023	0.3976	-0.068	0.0466	-0.117	-0.177	-0.323	0.3163	0.3077	0.0271	-0.19	0.197	-0.21	-0.17	0.181	-0.06	0.506	0.214	-0.41	0.803748	-0.05553	-0.096033727	0.040505627
2288	Q01459	CTBS	chitobiase	1486	ENSG00000117151	13	-0.14	-0.64	-0.1	0.059	-0.04	-0.07	-0.07	0.269	-0.01	0.357	-0.094	0.2516	-0.039	-0.161	-0.217	0.2334	-0.245	-0.369	-0.33	0.208	0.014	-0.09	0.101	-0.3	0.287	0.611	-0.44	0.867975	-0.08833	-0.050502184	-0.037824808
1037	O14617	AP3D1	adaptor related protein complex 3 subunit de	8943	ENSG00000065000	24	0.118	0.033	0.146	-0.05	-0.1	-0.26	-0.02	-0.07	0.302	0.0058	-0.043	0.0628	0.1421	-0.154	0.0839	0.0163	0.2013	0.2658	-0.15	-0.77	-0.22	-0.3	-0.21	0.066	0.259	0.007	0.336	0.422778	0.120776	-0.052993668	0.173770047
2095	P54727	RAD23B	RAD23 homolog B, nucleotide excision repa	5887	ENSG00000119318	16	0.493	0.293	-0.01	0.034	0.39	-0.45	-0.73	-0.6	-0.28	0.0647	0.2179	0.2412	0.0936	-0.089	0.158	-0.693	-0.203	-0.666	-0	-0.69	0.004	-0.58	-0.15	0.094	-0.46	-0.34	-0.34	0.631086	0.179628	0.002026813	0.17760144
3586	Q9UBR2	CTSZ	cathepsin Z	1522	ENSG00000101160	10	0.057	-0.14	-0.18	-0.25	-0.23	-0.13	0.229	0.341	0.451	0.3725	0.0718	0.209	-0.508	-0.007	-0.457	0.3022	-0.1	0.1644	-0.3	0.057	0.175	-0.4	-0.67	-0.24	0.505	0.133	0.246	0.918412	0.071415	0.011136676	0.060278249
3513	Q9NUQ8	ABCF3	ATP binding cassette subfamily F member	55324	ENSG00000161204	20	0.279	0.388	0.004	-0.15	-0.03	-0.25	0.089	0.138	0.322	0.1817	0.1892	0.1657	-0.011	-0.201	-0.099	0.3062	0.1895	0.271	-0.18	-0.73	-0.02	0.036	-0.17	0.172	0.088	0.231	0.321	0.578305	0.116435	-0.022813124	0.139247753
675	E9PPJ5	MDK	midkine	4192	ENSG00000110492	11	-0.26	-0.35	-0.39	-0.66	-0.39	0.53	-0.56	-0.12	-0.68	-0.341	0.4909	0.2427	-0.658	0.1367	-0.159	-0.326	-0.414	-0.125	-0.14	0.047	0.46	0.044	-0.13	-0	0.149	0.576	0.243	0.131959	-0.45694	-0.190402303	-0.266537702
1030	O00743	PPP6C	protein phosphatase 6 catalytic subunit	5537	ENSG00000119414	13	0.143	0.187	0.017	-0.08	0.336	-0.5	0.019	0.014	0.143	0.1296	0.0376	0.0535	0.1028	-0.27	-0.031	0.0338	0.1645	0.1814	-0.26	-0.84	-0.26	-0.22	-0.18	0.111	5E-04	-0.05	0.169	0.248207	0.202419	-0.013443211	0.215862659
2931	Q8NCW5	NAXE	NAD(P)HX epimerase	128240	ENSG00000163382	11	0.208	0.205	0.005	0.003	0.023	-0.26	0.4	0.111	0.35	0.0072	-0.016	0.0905	-0.053	-0.147	-0.078	0.1412	-0.042	-0.028	-0.12	-0.6	0.153	-0.38	-0.14	0.037	0.045	0.211	0.205	0.32072	0.182551	0.130564585	0.051986057
1405	P04040	CAT	catalase	847	ENSG00000121691	16	-0.21	-0.25	-0.42	-0.26	-0.21	-0.24	-0.25	-0.34	0.047	-0.336	-0.172	-0.047	0.039	0.3416	-0.21	0.0836	-0.062	-0.016	-0.63	-0.88	0.467	0.886	0.209	0.058	0.081	0.11	-0.01	0.38994	-0.26812	-0.193296568	-0.074824645
3681	Q9UNW1	MINPP1	multiple inositol-polyphosphate phosphatase	9562	ENSG00000107789	10	0.186	-0.07	-0.17	-0.2	0.253	-0.43	-0.24	-0.07	-0.04	0.2441	0.0223	0.3411	-0.046	-0.089	-0.335	0.4913	0.0522	-0.023	-0.15	-0.1	0.038	-0.19	-0.23	0.016	0.269	0.118	-0.19	0.42447	-0.04036	-0.16095104	0.120593515
2955	Q8NG11	TSPAN14	tetraspanin 14	81619	ENSG00000108219	7	-0.26	-0.03	0.7	-0.17	-0.21	0.346	-0.22	0.131	-0.41	-0.082	0.1614	-0.238	-0.077	-0.207	-0.132	-0.53	0.1086	0.0447	-0.51	0.66	-0.46	-0	-0.18	-0.29	0.051	-0.19	-0.36	0.773742	0.12728	0.091968606	0.035310966
3408	Q9H7P6	MVB12B	multivesicular body subunit 12B	89853	ENSG00000196814	12	0.138	0.509	-0.33	-0.08	0.184	-0.36	-0.21	0.215	-0.44	-0.318	0.1677	0.2768	0.0166	-0.187	-0.004	0.36	-0.102	0.4974	-0.13	0.036	-0.25	-0.15	-0.27	-0.09	-0.46	0.043	0.495	0.60208	0.043113	-0.121181696	0.164294657
2494	Q15046	KARS;KAF	lysyl-tRNA synthetase;lysyl-tRNA synthetas	3735	ENSG00000065427	13	-0.02	-0.05	-0.17	0.331	0.267	-0.2	-0.4	-0.03	0.228	-0.028	0.0417	-0.011	-0.073	-0.209	0.0777	-0.014	0.0546	0.034	-0.23	-0.69	-0.08	-0.13	-0.24	-0.09	0.199	-0.05	0.004	0.44267	0.139558	0.007830795	0.1317275
67	A0A087WY(	MYO5A	myosin VA	4644	ENSG00000197535	33	-0.03	0.233	-0.14	0.048	0.181	-0.27	-0.4	-0.27	-0.16	0.0959	0.0528	0.035	0.0731	-0.214	0.0041	0.0443	-0.042	-0.182	-0.29	-0.57	-0.24	-0.18	-0.3	0.196	-0.04	-0.02	-0.09	0.389626	0.081018	-0.074890648	0.155908367
3790	Q9Y6D5	ARFGEF2	ADP ribosylation factor guanine nucleotide e	10564	ENSG00000124198	39	0.011	0.064	-0.08	0.086	0.002	-0.1	-0.06	-0.15	-0.07	0.1052	-0.139	0.3471	0.2246	-0.157	-0.105	0.1068	-0.094	-0.102	-0.21	-0.5	-0.24	-0.28	-0.02	0.039	0.071	-0.04	0.119	0.368831	0.084985	-0.053127443	0.138112176
3201	Q99447	PCYT2	phosphate cytidylyltransferase 2, ethanolam	5833	ENSG00000185813	18	0.274	0.108	0.021	0.201	0.406	-0.23	-0.12	0.107	0.011	0.1495	0.0828	-0.103	0.0456	-3E-04	-0.054	-0.111	0.1519	-0.078	-0.27	-1.07	-0.31	-0.32	-0.3	-0.06	-0.07	-0.08	0.048	0.088651	0.357187	0.077088287	0.280098304
640	E9PER6	PDPK1	3-phosphoinositide dependent protein kinas	5170	ENSG00000140992	12	-0.05	-0.13	-0.05	0.014	-0.01	-0.44	0.087	0.006	0.129	0.277	-0.027	0.0853	0.085	-0.071	-0.054	0.0259	0.1845	0.1952	0.184	-0.37	0.104	-0.43	-0.25	0.098	0.06	-0.05	0.247	0.432857	-0.00476	-0.127870199	0.123112426
2971	Q8TC07	TBC1D15	TBC1 domain family member 15	64786	ENSG00000121749	19	0.233	0.208	0.071	-0.12	0.014	-0.33	-0.17	-0.19	0.07	0.2563	0.1336	0.1774	0.2629	0.011	-0.028	0.0049	-0.128	-0.01	-0.01	-0.78	-0.01	-0.25	-0.25	0.212	-0.01	-0.01	#####	0.311521	0.099567	-0.100123064	0.199690549
3534	Q9NXL6	SIDT1	SID1 transmembrane family member 1	54847	ENSG00000072858	9	-0.12	-0.26	0.292	-0.07	-0.23	-0.46	-0.02	-0.12	0.072	0.4943	0.0708	0.0776	0.0033	-0.626	0.0635	0.344	0.1119	0.1484	-0.17	-0.39	-0.11	-0.16	-0.12	0.395	-0.08	-0.28	0.155	0.433923	-0.01742	-0.179512336	0.162094576
2027	P50747	HLCS	holocarboxylase synthetase	3141	ENSG00000159267	9	0.185	0.018	0.016	3.159	0.319	3.781	0.022	0.092	0.277	0.0137	-0.041	0.1577	0.0756	-0.119	0.2343	0.2428	0.1939	0.0546	-0.15	-0.57	-0.18	0.43	-0.18	0.109	0.255	0.13	0.226	0.22664	0.866297	0.784189542	0.082107433
2438	Q14012	CAMK1	calcium/calmodulin dependent protein kinas	8536	ENSG00000134072	15	-0.01	0.339	-0	0.035	-0.1	-0.33	-0.18	-0.1	0.032	0.2669	-0.125	0.2857	0.1726	-0.022	0.2297	-0.234	0.0785	-0.151	-0.26	-1.37	-0.61	-0.66	-0.39	-0.02	-0.15	0.262	0.202	0.175891	0.296448	-0.091710036	0.388158102
1434	P05164	MPO	myeloperoxidase	4353	ENSG00000005381	33	-1.35	-1.64	-1.37	-1	-1.17	1.137	-0.85	-1.49	-1.34	-1.111	-0.313	-0.952	-0.927	-0.81	-1.082	-1.027	-1.639	-1.45	-0.97	-0.89	2.536	2.1	-0.15	0.968	-1.19	-1.49	-1.48	0.237687	-0.94411	0.027401818	-0.971510253
3508	Q9NT62	ATG3	autophagy related 3	64422	ENSG00000144848	9	-0.1	0.054	-0.19	0.056	0.115	-0.39	0.379	0.263	0.222	-0.019	-0.118	0.0942	0.1135	-0.235	0.2002	0.3127	0.1671	0.3982	-0.43	-0.69	-0.38	-0.17	-0.2	0.124	0.299	0.099	0.241	0.351856	0.168292	-0.056280818	0.224572976
399	B0QY89	EIF3L	eukaryotic translation initiation factor 3 subu	51386	ENSG00000100129	15	0.163	-0.1	-0.1	-0.07	0.026	-0.47	-0.13	-0.15	0.126	0.0933	-0.019	-0.074	-0.125	-0.189	-0.185	0.2466	0.0128	0.1939	-0.17	-0.5	-0.02	-0.3	-0.37	-0.14	0.16	0.075	0.05	0.515126	0.056029	-0.074163911	0.130192741
319	A0A2R8Y54	PATJ	PATJ crumbs cell polarity complex compone	10207	ENSG00000132849	30	6E-04	0.154	-0.2	0.144	-0.19	-0.27	0.069	-0.17	0.052	-0.117	0.1448	0.179	0.1392	-0.37	0.3553	0.5477	0.6	0.1309	-0.26	-0.1	-0.27	-0.22	-0.31	-0.23	-0.1	-0.2	0.041	0.068328	0.137089	-0.223710121	0.360799128
1905	P41208	CETN2	centrin 2	1069	ENSG00000147400	8	0.31	0.135	0.191	-0.02	0.144	-0.12	0.002	-0.16	0.294	-0.231	0.1581	0.1719	-0.118	-0.163	0.0696	0.463	0.7002	0.2973	-0.11	-1.16	0.162	-0.48	-0.44	0.008	0.018	-0.29	0.125	0.14696	0.327599	-0.06312837	0.390727647
1889	P39019	RPS19	ribosomal protein S19	6223	ENSG00000105372	9	0.287	-0.17	-0.06	-0.35	0.234	-0.35	0.107	-0.22	0.192	-0.249	-0.068	-0.121	-0.36	0.1379	0.0186	-0.391	-0.157	-0.274	-0	-0.78	0.036	-0.62	-0.18	-0.15	0.095	-0.28	-0.06	0.464612	0.17933	0.124736874	0.054592912
1169	O60645	EXOC3	exocyst complex component 3	11336	ENSG00000180104	25	0.117	0.312	-0.02	0.064	-0	-0.28	-0.05	-0.05	0.138	0.0588	0.0813	0.2558	0.1178	-0.016	-0.025	0.047	0.1346	0.1626	-0.17	-0.76	-0.26	-0.47	-0.33	0.062	0.098	0.167	0.27	0.203639	0.181367	-0.064296619	0.245663395
3247	Q9BRF8	CPPED1	calcineurin like phosphoesterase domain co	55313	ENSG00000103381	11	-0.15	0.166	-0.15	0.048	0.233	-0.17	0.022	-0.09	-0.08	0.2519	0.0996	0.268	0.2949	-0.069	0.1715	0.036	-0.234	-0.146	-0.01	-0.74	-0.27	-0.34	-0.18	0.087	0.179	-0.01	0.173	0.338732	0.104081	-0.093958099	0.198038883
2232	P63151	PPP2R2A	protein phosphatase 2 regulatory subunit Ba	5520	ENSG00000221914	18	0.088	0.08	-0.03	0.015	0.148	-0.23	-0.13	-0.09	0.227	0.0449	0.0029	0.0507	0.0703	-0.053	0.1524	0.0754	0.3008	0.0566	-0.16	-0.75	-0.19	-0.39	-0.14	0.118	-0.12	-0.04	0.088	0.12572	0.182895	-0.070329743	0.25322473
3682	Q9UNZ2	NSFL1C	NSFL1 cofactor	55968	ENSG00000088833	16	0.287	-0.07	-0.1	-0.11	0.279	0.392	-0.22	0.016	-0.13	0.2978	0.1589	0.0466	0.0118	-0.131	0.1722	-0.103	-0.09	-0.133	-0.03	-0.47	-0.14	-0.53	-0.23	-0.03	0.438	0.085	-0.16	0.447685	0.157258	0.012244831	0.145013327
1837	P32969	RPL9;AC0	ribosomal protein L9;ribosomal protein L9 ps	6133	ENSG00000163682	8	0.066	-0.29	-0.26	-0.03	0.306	-0.13	0.18	0.225	0.27	0.3198	-0.066	-0.076	-0.196	-0.158	-0.074	-0.188	0.1306	0.0436	0.118	0.135	-0.12	-0.51	-0.09	-0.04	0.105	-0.32	-0.14	0.559154	0.131448	0.065671178	0.06577667
2828	Q86Y82	STX12	syntaxin 12	23673	ENSG00000117758	11	-0.05	0.039	0.078	0.059	-0.11	-0.36	-0.28	-0.09	0.316	0.2779	0.0887	-0.178	0.1556	-0.286	0.2802	0.0373	0.3439	0.0308	-0.14	-0.51	-0.14	-0.62	-0.37	0.216	-0.3	-0.19	0.07	0.14551	0.178367	-0.126216883	0.304584276
3775	Q9Y5X3	SNX5	sorting nexin 5	27131	ENSG00000089006	18	0.232	0.21	-0.01	-0.08	-0.06	-0.47	-0.36	-0.14	-0.12	-0.025	0.0985	0.0765	-0.069	-0.057	0.1823	-0.104	0.1061	0.0936	-0.06	-0.95	-0.14	-0.52	-0.39	-0.08	-0.32	-0.31	0.515	0.239861	0.163778	-0.121125784	0.284903705
2687	Q6IBS0	TWF2	twinfilin actin binding protein 2	11344	ENSG00000247596	14	-0.02	0.117	-0.07	-0.08	-0.11	-0.34	0.131	0.007	0.009	0.1411	0.0115	-0.019	-0.131	-0.086	0.1078	-0.025	0.3231	0.0755	-0.12	-0.6	-0.02	0.16	-0.22	0.021	-0.07	-0.09	0.235	0.499932	0.038515	-0.084547357	0.123062703
3717	Q9Y2H0	DLGAP4	DLG associated protein 4	22839	ENSG00000080845	11	0.061	0.349	-0.06	-0.03	-0.08	-0.59	-0.16	-0.01	-0.01	0.1753	0.0661	0.349	0.0032	0.035	0.1098	0.0074	0.2894	0.1514	-0.25	-1.07	-0.29	-0.62	-0.45	-0.01	-0.04	-0.01	0.321	0.128553	0.211775	-0.189635747	0.401410766
362	A2IBA6	LIPH	lipase H	200879	ENSG00000163898	9	-0.28	0.175	-0.11	-0.79	-0.63	-0.66	-0.38	-0.38	-0.39	0.0896	0.1916	-0.207	-0.736	-0.801	-0.844	-0.084	-0.195	0.0673	-0.17	0.509	0.23	-0.17	-1.11	-0.28	-0.18	-0.44	-0.44	0.780996	-0.15779	-0.103700029	-0.054092286
3041	Q92626	PXDN	peroxidasin	7837	ENSG00000130508	22	0.294	0.149	0.33	-0.59	-0.07	-0.74	-0.09	-0.17	0.055	0.0194	-0.033	-0.056	-0.411	-0.395	-0.197	0.1531	0.3493	0.3138	-0.36	0.107	0.253	-0.14	-0.28	-0.31	0.308	-0.02	-0.16	0.935096	-0.0245	-0.063828143	0.039329627
1215	O75534	CSDE1	cold shock domain containing E1	7812	ENSG00000009307	24	-0.02	0.025	-0.12	-0.18	-0.02	-0.43	-0.11	-0.05	0.132	-0.014	-0.094	0.0827	0.0377	-0.068	0.072	0.0147	0.1336	-0.015	-0.24	-0.73	-0.28	-0.39	-0.17	0.084	0.018	-0.06	0.027	0.199818	0.106997	-0.101529528	0.208526511
1136	O43760	SYNGR2	synaptogyrin 2	9144	ENSG00000108639	6	-0.1	-0.13	-0.16	0.027	-0.17	-0.12	-0.1	-0.08	0.229	-0.14	-0.154	-0.008	0.0046	0.0583	-0.022	0.0652	0.057	0.142	-0.28	-0.39	-0.34	-0.05	-0.01	0.088	0.215	-0.05	0.229	0.717397	-0.0023	-0.067903038	0.065606928
2003	P49755	TMED10	transmembrane p24 trafficking protein 10	10972	ENSG00000170348	6	0.121	0.137	-0.03	0.105	0.533	-0.36	0.259	0.334	0.491	-0.213	-0.39	-0.186	-0.027	-0.209	-0.108	-0.17	-0.077	0.1821	-0.33	-0.49	-0.28	-0.42	-0.24	-0.08	0.077	-0.02	0.222	0.082302	0.350143	0.309729463	0.040413934
2520	Q15389	ANGPT1	angiopoietin 1	284	ENSG00000154188	18	-0.21	-0.71	-0.22	-0.59	-0.06	-0.25	-0.54	0.005	-0.03	0.4673	0.4102	0.3423	-0.58	0.7872	-0.161	-0.352	-0.086	-0.066	-0.09	0.565	-0.27	-0.67	-0.38	-0.38	0.556	-0.29	-0.29	0.28921	-0.14983	-0.372963083	0.223137146
65	O43681	ASNA1;GE	arsA arsenite transporter, ATP-binding, hom	439	ENSG00000198356	5	-0.17	0.182	-0.04	-0.14	-0.1	-0.58	-0.03	-0.09	0.294	0.0018	-0.023	0.0106	0.087	-0.143	-0.084	-0.008	0.1134	0.1769	-0.1	-0.34	-0.15	-0.36	-0.42	0.014	0.104	-0.1	0.047	0.373218	0.067846	-0.0907209	0.158566555
3629	Q9UIJ5	ZDHHC2	zinc finger DHHC-type containing 2	51201	ENSG00000104219	12	-0.18	0.074	-0.03	-0.11	-0.29	-0.1	0.042	-0.11	0.019	0.0469	0.2495	0.0776	-0.032	-0.208	0.1812	0.3988	0.2233	0.2925	-0.34	-0.78	-0.2	-0.67	-0.29	-0.01	-0.14	-0.08	0.409	0.102176	0.160395	-0.210501622	0.370896504
1401	P02795	MT2A	metallothionein 2A	4502	ENSG00000125148	6	0.707	-0.3	-0.28	-0.14	-1.03	1.238	-0.42	0.806	-0.75	-0.338	-0.749	-0.568	0.4318	-1.183	-0.304	0.3403	-1.186	-1.614	0.16	-1.41	-0.13	0.724	0.227	0.079	0.091	-0.36	0.398	0.321246	0.004225	0.553703474	-0.54947835
1706	P21291	CSRP1	cysteine and glycine rich protein 1	1465	ENSG00000159176	8	-0.25	0.492	0.008	0.101	-0.09	-0.17	-0.13	-0.11	0.123	0.0795	0.007	-0.248	0.0056	-0.183	-0.007	-0.077	0.0618	-0.125	-0.25	-0.44	-0.1	-0.39	-0.29	0.042	-0.26	-0.18	0.08	0.202761	0.196741	0.051635518	0.145105856
2362	Q12792	TWF1	twinfilin actin binding protein 1	5756	ENSG00000151239	13	0.1	-0.07	0.078	-0.2	-0.09	-0.29	-0.03	0.014	-0	0.0352	0.0101	-0.103	0.0166	-0.05	0.1337	-0.15	-0.017	-0.056	-0.18	-0.82	0.041	-0.69	-0.14	-0.02	0.1	-0.1	0.064	0.355421	0.14065	-0.03349224	0.174142643
2561	Q16769	QPCT	glutaminyl-peptide cyclotransferase	25797	ENSG00000115828	9	0.081	0.088	0.482	0.116	-0.08	-0.3	0.224	0.02	-0.1	-0.192	0.0614	0.7712	0.2112	-0.168	-0.113	-0.018	-0.407	-0.167	-0.14	-0.48	0.359	0.053	-0.2	-0.22	0.32	-0.04	-0.17	0.773745	0.117541	0.061528822	0.056012151
223	A0A0G2JNZ	GBA	glucosylceramidase beta	2629	ENSG00000177628	14	-0.17	-0.48	-0.07	-0.27	-0.31	-0.18	-0.26	0.054	0.123	0.1104	0.1258	0.1064	-0.339	0.2847	-0.074	0.0639	-0.023	-0.14	0.199	0.454	0.29	-0.06	0.164	0.166	0.234	0.057	-0.29	0.098732	-0.30793	-0.186118484	-0.121810561
2858	Q8IXK2	GALNT12	polypeptide N-acetylgalactosaminyltransfera	79695	ENSG00000119514	17	-0.16	-0.7	-0.28	-0.17	-0.21	-0.35	0.248	0.321	0.173	-0.131	-0.113	-0.089	-0.013	0.0067	-0.093	-0.146	-0.125	-0.03	0.042	0.282	0.024	0.02	0.089	-0.05	0.507	-0.04	0.016	0.221515	-0.22515	-0.04420873	-0.18094584
1512	P09382	LGALS1	galectin 1	3956	ENSG00000100097	8	-0.74	0.275	0.189	0.473	-0.24	0.119	0.115	0.2	-0.43	0.0866	0.5254	-0.342	-0.311	0.1212	0.6573	0.1335	0.1126	-0.542	-0.69	-0.39	0.06	-0.13	-0.69	0.294	0.314	0.339	-0.01	0.808622	0.095391	-0.053515808	0.148906343
2449	Q14240	EIF4A2	eukaryotic translation initiation factor 4A2	1974	ENSG00000156976	24	0.034	-0.09	-0.06	0.063	0.126	-0.3	0.075	-0.02	0.275	-0.141	-0.052	-0.029	-0.033	-0.078	-0.025	0.0145	-0.055	0.0298	-0.21	-1.01	-0.17	-0.47	-0.24	0.045	0.207	-0.01	0.235	0.39346	0.193593	0.053314341	0.14027832
1813	P30520	ADSS2;AD	adenylosuccinate synthase 2;adenylosuccin	159	ENSG00000035687	18	0.009	0.132	-0.06	-0.14	0.013	-0.31	0.087	0.071	0.277	0.0196	-0.048	0.0742	-0.256	-0.175	-0.134	0.4669	0.1905	0.1922	-0.25	-0.69	-0.16	0.009	-0.16	-0.02	0.125	0.055	0.229	0.574324	0.104393	-0.028339377	0.132732053
2888	Q8N128	FAM177A1	family with sequence similarity 177 member	283635	ENSG00000151327	11	-0.12	0.311	-0.12	-0.24	-0.05	-0.51	-0.29	-0.52	-0.12	-0.054	0.391	-0.12	0.1024	0.0241	0.0725	-0.037	0.3945	-0.141	-0.46	-0.72	-0.3	-0.81	-0.52	-0.1	0.04	0.008	0.205	0.144754	0.109253	-0.255735304	0.364987829
2452	Q14258	TRIM25	tripartite motif containing 25	7706	ENSG00000121060	14	0.162	0.217	-0.1	0.037	-0.09	-0.47	-0.19	-0.23	0.102	0.0807	0.1172	0.1338	0.1612	-0.105	-0.033	-0.137	0.1139	-0.076	-0.27	-0.89	-0.2	-0.45	-0.19	0.18	-0.08	-0.03	0.142	0.293818	0.136527	-0.090376091	0.226903345
2239	P63313	TMSB10	thymosin beta 10	9168	ENSG00000034510	5	-0.8	0.368	-0.08	0.128	-0.11	-0.37	0.508	0.005	0.417	0.1395	0.2049	-0.087	-0.215	-0.045	0.2109	0.3556	0.8935	-0.088	-0.2	-0.69	-0.56	-0.8	-0.71	0.158	-0.04	0.136	0.033	0.183913	0.305765	-0.144351103	0.450115987
2834	Q8IUC4	RHPN2	rhophilin Rho GTPase binding protein 2	85415	ENSG00000131941	12	0.13	0.225	-0.17	0.034	-0.09	-0.46	-0.09	-0.09	0.125	0.044	0.0315	0.0283	0.1056	-0.333	0.0066	0.1125	0.0764	0.2224	-0.33	-0.93	-0.33	-0.41	-0.44	0.002	-0.02	-0.07	0.297	0.203131	0.204292	-0.075454441	0.2797467
604	E7EPK1	SEPT7;SE	septin 7	989	ENSG00000122545	12	0.063	-0.07	-0.28	0.041	0.104	-0.41	-0.09	-0.13	-0.09	0.053	-0.016	-0.195	0.151	0.0143	0.0774	-0.226	-0.244	-0.167	0.093	-0	0.087	-0.28	-0.19	-0.03	0.028	-0.13	-0.29	0.925381	-0.01795	-0.034967454	0.017020078
672	E9PL57	NEDD8-MI	NEDD8-MDP1 readthrough	100528064	ENSG00000255526	6	-0.05	0.128	-0.12	0.023	-0.12	-0.3	0.602	0.383	0.545	-0.136	-0.114	-0.155	-0.239	-0.275	0.0329	0.4345	0.3774	0.4191	-0.37	-0.9	-0.13	-0.53	-0.51	-0.25	0.411	0.196	0.533	0.401834	0.292116	0.082488741	0.209627355
3342	Q9H074	PAIP1	poly(A) binding protein interacting protein 1	10605	ENSG00000172239	9	0.225	0.141	-0.08	0.298	0.082	-0.41	0.168	0.162	0.338	-0.179	-9E-04	0.2172	0.0979	-0.135	0.0417	0.4069	0.003	0.1282	-0.23	-1.1	-0.2	-0.55	-0.33	0.065	0.322	0.321	0.151	0.302284	0.276014	0.038671626	0.237342488
2591	Q53FA7	TP53I3	tumor protein p53 inducible protein 3	9540	ENSG00000115129	8	0.073	0.311	-0.18	0.185	0.126	-0.43	0.299	0.179	0.239	-0.247	0.0426	0.2714	-0.009	-0.231	0.0066	-0.187	-0.041	0.0094	-0.35	-1.23	0.071	-0.24	-0.19	0.117	-0.1	-0.04	0.092	0.267841	0.296373	0.131828496	0.164544886
3801	Q9Y6X5	ENPP4	ectonucleotide pyrophosphatase/phosphodie	22875	ENSG00000001561	7	-0.5	-0.3	-0.12	-0.13	-0.3	-0.32	0.277	-0.11	-0.17	0.276	0.1302	0.0999	-0.167	-0.181	0.123	-0.184	0.1397	-0.129	-0.16	-0.45	-0.06	-0.3	-0.31	0.239	-0.06	-0.01	0.032	0.275976	-0.06495	-0.19697703	0.132022949
445	B4E1N1	ARMC6	armadillo repeat containing 6	93436	ENSG00000105676	7	0.745	-0.03	-0.12	0.15	-0.21	-0.22	0.519	0.481	0.587	-0.326	-0.368	-0.26	-0.082	-0.115	-0.109	0.3444	0.39	0.5614	-0.15	-0.71	-0.31	-0.3	-0.25	0.026	0.326	0.12	0.483	0.375651	0.29734	0.20811004	0.089229491
2801	Q86SR1	GALNT10	polypeptide N-acetylgalactosaminyltransfera	55568	ENSG00000164574	7	0.035	-0.25	-0.12	-0.04	0.06	-0.19	-0.08	-0.15	-0.07	0.0376	0.1601	0.2152	0.0747	0.1697	0.0267	0.0625	0.2252	0.2819	-0.45	-0.03	0.613	-0.31	-0.34	-0.08	0.157	-0.4	-0.22	0.145273	0.02773	-0.22924327	0.256973224
3055	Q92882	OSTF1	osteoclast stimulating factor 1	26578	ENSG00000134996	9	-0.04	0.355	-0.05	-0.15	-0.16	-0.47	-0.27	-0.17	-0.03	0.1883	0.1365	0.0939	0.0277	-0.179	0.2808	-0.008	0.2051	0.007	-0.23	-0.95	-0.04	-0.43	-0.45	0.146	-0.22	-0.08	0.147	0.161616	0.124053	-0.192426929	0.31648007
2104	P55039	DRG2	developmentally regulated GTP binding prot	1819	ENSG00000108591	14	0.022	0.24	-0.02	-0.04	-0.07	-0.49	0.129	0.197	0.328	0.1963	-0.096	0.0796	0.0736	0.0336	0.0517	0.1351	0.2302	0.2719	-0.27	-0.71	-0.21	-0.41	-0.2	-0.02	0	0.178	0.297	0.208731	0.182981	-0.075597145	0.258578193
1210	O75436	VPS26A	VPS26, retromer complex component A	9559	ENSG00000122958	14	0.003	0.137	0.068	-0	-0.05	-0.42	-0.13	-0.09	0.086	0.0921	0.1478	0.0659	0.0074	-0.179	0.0015	0.1824	0.2004	0.0135	-0.01	-0.65	0.037	-0.3	-0.28	0.046	0.119	0.023	0.048	0.338675	0.064284	-0.101483063	0.165767267
22	Q16881	TXNRD1	thioredoxin reductase 1	7296	ENSG00000198431	13	-0.05	-0.33	-0.36	-0.18	0.029	-0.27	-0.05	0.154	0.008	-0.309	-0.4	-0.02	-0.333	-0.137	-0.136	-0.022	0.2495	0.3322	-0.12	-0.3	0.871	-0.41	-0.19	-0.21	0.196	-0.02	0.294	0.720917	-0.13136	-0.031436393	-0.099926868

137	A0A0A0MQ)	SPOCK1	SPARC (osteonectin), cwcv and kazal like d	6695	ENSG00000152377	13	0.363	0.132	-0.5	0.014	-0.48	0.031	-0.95	-0.28	-0.42	-0.261	-0.036	-0.194	-0.014	-0.224	-0.562	0.1372	-0.781	-0.746	0.026	0.19	0.238	-0.1	-0.6	-0.31	-0.03	-0.11	-0.8	0.826602	-0.06524	0.065798693	-0.131042527
2295	Q01968	OCRL	OCRL inositol polyphosphate-5-phosphatas	4952	ENSG00000122126	22	0.158	0.184	0.006	0.027	-0.16	-0.34	-0.18	0.026	0.185	0.1135	-0.068	-0.022	-0.037	-0.241	0.0315	0.1198	0.0965	0.1434	-0.24	-0.65	-0.24	-0.32	-0.14	-0.16	-0.02	0.03	0.144	0.211185	0.164606	-0.026467938	0.191073506
3674	Q9UNF1	MAGED2	MAGE family member D2	10916	ENSG00000102316	18	0.053	-0.05	0.02	-0.08	-0.12	-0.39	-0.18	-0.13	0.069	0.0853	-0.17	0.0068	-0.024	-0.175	-0.033	0.1482	0.0973	0.0323	-0.27	-0.48	-0.18	-0.48	-0.21	-0.15	0.006	0.062	0.072	0.212004	0.092572	-0.086761534	0.179333911
2384	Q13162	PRDX4	peroxiredoxin 4	10549	ENSG00000123131	13	-0.22	-0.23	-0.02	-0.12	-0.03	0.088	0.193	1.059	0.016	0.4942	-0.249	-0.223	-0.678	0.0086	-0.317	-0.25	-0.012	-0.035	-0.02	1.353	-0.04	-0.23	0.256	-0.49	0.548	-0.06	-0.61	0.62586	0.003807	0.22280171	-0.21899446
1654	P17655	CAPN2	calpain 2	824	ENSG00000162909	19	0.045	-0.15	-0.11	0.285	0.325	-0.39	0.095	0.233	0.557	-0.031	-0.51	-0.536	0.0695	0.162	-0.177	0.4762	0.191	0.5171	-0.67	-0.99	-0.3	-0.22	-0.02	0.182	-0.08	0.01	0.244	0.356582	0.301384	0.079949479	0.221434611
2651	Q5TH69	ARFGEF3	ARFGEF family member 3	57221	ENSG00000112379	25	0.166	-0.17	0.042	-0.11	-0.27	-0.04	0.028	0.293	0.149	0.1571	-0.077	0.0594	0.142	-0.105	0.0716	0.2299	0.0129	0.0504	-0.27	-0.82	-0.23	-0.16	-0.13	0.202	0.196	0.156	0.169	0.485869	0.108476	-0.050932558	0.159408575
3263	Q9BT78	COPS4	COP9 signalosome subunit 4	51138	ENSG00000138663	17	-0.01	0.137	0.01	-0	0.124	-0.41	0.067	0.06	0.164	0.158	-0.041	0.0419	-0.02	-0.158	0.1343	0.4711	0.3697	0.1909	-0.18	-0.76	-0.14	-0.53	-0.39	-0.01	0.117	0.169	0.054	0.145944	0.200208	-0.112939843	0.313148115
836	J3KMZ9	LDLR	low density lipoprotein receptor	3949	ENSG00000130164	13	0.025	-0.12	-0.35	0.029	-0.8	-0.22	-0.12	0.265	0.095	0.2618	0.0032	0.1531	0.0734	-0.161	-0.349	0.4843	-0.27	0.2862	-0.07	-0.73	-0.21	0.106	0.032	-0.55	0.1	-0.18	0.207	0.464498	0.011231	-0.187209957	0.198440986
2586	Q4KMQ2	ANO6	anoctamin 6	196527	ENSG00000177119	15	-0.62	0.101	-0.39	-0.04	0.282	-0.35	0.037	-0	0.221	-0.478	-0.363	-0.31	0.1057	-0.124	-0.089	-0.051	-0.113	0.1237	-0.58	-0.36	-0.55	-0.32	-0.13	-0.03	-0.14	-0.14	-0.07	0.479518	0.172434	0.060181347	0.112253057
3638	Q9UJJ9	GNPTG	N-acetylglucosamine-1-phosphate transfera	84572	ENSG00000090581	5	-0.2	-0.65	-0.18	-0.3	-0.27	0.05	0.801	0.639	0.988	0.3481	0.2904	-0.099	-0.589	0.556	-0.247	0.4892	-0.346	0.2777	0.047	0.623	0.08	0.004	0.026	-0.08	0.82	0.335	0.594	0.716233	-0.17326	0.022876516	-0.196138001
2023	P50552	VASP	vasodilator stimulated phosphoprotein	7408	ENSG00000125753	9	0.181	0.16	-0.04	-0.09	-0.03	-0.26	0.046	0.072	0.237	0.0411	-0.077	0.1146	0.0233	-0.011	-0.042	-0.02	0.0291	0.0255	0.002	-1	0.018	-0.18	-0.25	0.004	0.024	-0.08	0.032	0.294018	0.187768	0.02113849	0.166629637
2560	Q16706	MAN2A1	mannosidase alpha class 2A member 1	4124	ENSG00000112893	26	0.064	-0.31	-0.3	-0.46	0.029	-0.13	-0.22	2E-04	0.147	0.4117	-0.119	0.3708	-0.335	0.388	-0.214	0.1214	-0.457	-0.112	-0.11	-0.08	0.134	-0.05	-0.08	-0.06	0.603	0.065	-0.15	0.53321	-0.16046	-0.137451987	-0.023010347
3350	Q9H0R4	HDHD2	haloacid dehalogenase like hydrolase domai	84064	ENSG00000167220	10	0.12	0.189	-0.11	-0.12	0.157	-0.47	-0.35	-0.31	-0.14	0.0196	0.1218	0.0601	-0.211	-0.279	0.1352	-0.196	-0.225	-0.415	-0.15	-0.9	-0.26	-0.47	-0.27	-0.05	-0.08	-0.07	-0.04	0.515916	0.140729	-0.003530006	0.144259298
1504	P08571	CD14	CD14 molecule	929	ENSG00000170458	9	-0.48	-0.32	-0.5	0.055	-0.25	-0.23	0.072	0.199	0.099	-0.143	0.1754	0.5739	0.0234	-0.207	-0.036	-0.13	-0.033	0.0312	-0.48	-0.37	-0.14	0.081	-0.35	-0.23	0.161	0.22	1.199	0.638767	-0.16143	-0.179349852	0.017921255
1009	O00442	RTCA	RNA 3'-terminal phosphate cyclase	8634	ENSG00000137996	13	0.103	0.275	-0.05	0.085	-0.13	-0.35	-0.08	-0.01	0.112	0.0981	0.0586	0.1393	0.1441	-0.23	-0.036	-0.012	0.0091	0.1127	-0.23	-0.86	-0.17	-0.05	-0.3	0.25	-0.16	-0.01	0.348	0.480932	0.125897	-0.036894938	0.162792302
2472	Q14689	DIP2A	disco interacting protein 2 homolog A	23181	ENSG00000160305	22	-0.07	-0.02	-0.2	-0.1	-0.18	-0.41	-0.09	0.016	0.015	-0.01	0.0817	0.1998	-0.14	-0.015	0.0062	0.0158	-0.043	-0.027	-0.26	-0.24	-0.02	-0.52	-0.69	-0.17	0.163	0.082	0.269	0.399544	0.039514	-0.121681807	0.161195576
3327	Q9C005	DPY30	dpy-30 histone methyltransferase complex r	84661	ENSG00000162961	5	0.155	0.106	-0.21	0.139	0.026	-0.25	0.213	-0.05	0.107	-0.061	-0.08	0.1017	-0.081	-0.174	0.0436	0.5185	0.0402	0.1149	-0.4	-0.61	0.017	-0.44	-0.08	-0.08	0.143	-0.03	0.205	0.309699	0.168126	-0.021126239	0.18925235
1744	P25098	GRK2	G protein-coupled receptor kinase 2	156	ENSG00000173020	16	-0.01	0.288	-0.01	-0.05	-0.07	-0.37	-0.02	0.005	0.034	0.3343	0.1702	0.1122	0.0524	-0.127	-0.001	0.1066	0.1527	0.0906	-0.2	-0.69	-0.21	-0.37	-0.36	0.109	0.019	-0.01	0.191	0.147438	0.145543	-0.121070538	0.266614025
3428	Q9HAT2	SIAE	sialic acid acetylesterase	54414	ENSG00000110013	13	-0.04	0.127	0.095	-0.29	-0.42	-0.16	-0.04	0.156	0.366	0.559	-0.002	0.4595	-0.475	-0.358	-0.841	-0.203	-0.138	-0.142	-0.14	-0.01	-0.05	-0.29	-0.05	-0.16	0.454	0.59	-0.48	0.830812	-0.00589	0.104585237	-0.110472439
1269	O95084	PRSS23	serine protease 23	11098	ENSG00000150687	12	0.369	0.635	0.277	-0.57	0.22	-0.6	0.308	0.509	0.032	0.2831	-0.054	0.1904	-0.526	-0.271	-0.456	0.335	0.3284	0.2253	0.369	0.556	-0.03	-0.58	-0.44	-0.34	0.427	-0.27	0.36	0.830965	0.126755	0.125291874	0.001463598
3518	Q9NVE7	PANK4	pantothenate kinase 4	55229	ENSG00000157881	19	0.18	-0.04	-0.09	-0.04	-0.07	-0.37	0.297	0.149	0.216	0.1963	0.1957	0.2444	0.0232	-0.022	0.0292	0.1897	0.2012	0.4857	-0.11	-0.27	0.049	-0.37	-0.25	-0.02	0.154	-0.01	0.479	0.257578	0.063919	-0.145943706	0.209862638
2500	Q15126	PMVK	phosphomevalonate kinase	10654	ENSG00000163344	16	-0.08	0.159	0.01	0.064	-0.23	-0.31	0.163	0.184	0.353	0.1448	0.0339	0.0062	0.0314	-0.127	0.1422	0.2252	0.1581	0.1739	-0.27	-0.84	-0.26	-0.53	-0.3	0.103	0.199	0.226	0.302	0.302395	0.185255	-0.053842497	0.239097744
923	Q9BTW9	TBCD	tubulin folding cofactor D	6904	ENSG00000141556	14	-0.12	-0.18	-0.24	-0.31	-0.11	-0.15	0.017	0.056	0.093	0.1712	0.0127	-0.117	-0.229	0.1645	-0.279	0.0075	0.1445	0.2583	-0.24	-0.13	0.039	-0.14	-0.01	0.071	0.159	0.172	0.09	0.39467	-0.1049	-0.119573749	0.014670875
1623	P15291	B4GALT1	beta-1,4-galactosyltransferase 1	2683	ENSG00000086062	11	0.007	-0.2	-0.24	-0.24	0.026	-0.21	-0.09	-0.01	0.235	0.1203	0.1738	0.2225	-0.381	0.0886	-0.226	0.5667	-0.108	0.1124	-0.21	-0.15	0.027	0.219	-0.41	-0	0.213	-0.13	-0.12	0.484647	-0.01832	-0.14377765	0.125461069
3697	Q9Y224	RTRAF	RNA transcription, translation and transport	51637	ENSG00000087302	13	0.076	0.093	0.06	-0.13	0.118	0.186	0.256	-0.06	0.314	0.2215	-0.012	-0.023	0.0487	0.0638	0.1717	-0.018	-0.249	-0.156	-0.1	-0.66	-0.12	0.068	0.015	0.201	0.339	-0.08	-0.24	0.40054	0.164385	0.095683039	0.068701692
3526	Q9NWV4	CZIB;C1or	CXXC motif containing zinc binding protein;(	54987	ENSG00000162384	9	0.118	0.111	-0.04	-0.01	0.303	-0.3	-0.03	0.017	0.085	0.1508	-0.113	0.0116	-0.147	-0.066	0.0333	-0.076	0.113	-0.037	-0.05	-0.62	0.107	-0.55	-0.15	0.105	-0.08	-0.03	0.061	0.343772	0.163628	0.043054333	0.120573449
1741	P24666	ACP1	acid phosphatase 1	52	ENSG00000143727	10	-0.23	0.188	-0.25	-0.01	-0.31	-0.19	-0.01	0.18	0.403	0.1205	0.1212	0.0287	-0.005	0.0141	0.277	0.3187	0.0433	0.3569	-0.15	-0.53	-0.09	-0.31	-0.21	-0.11	0.004	0.241	0.525	0.319356	0.044661	-0.165861586	0.210522892
2071	P53396	ACLY	ATP citrate lyase	47	ENSG00000131473	67	0.234	0.08	-0.12	-0.05	0.047	-0.5	-0.09	0.024	0.13	-0.213	-0.09	-7E-04	0.0089	-0.074	-0.063	0.1575	0.017	0.2423	-0.23	-0.75	-0.27	-0.66	-0.45	-0.2	0.183	0.049	0.141	0.220056	0.215151	-0.025603977	0.24075492
2219	P62913	RPL11	ribosomal protein L11	6135	ENSG00000142676	7	0.256	-0.04	-0.01	-0.07	0.176	-0.39	0.41	0.14	0.419	-0.284	0.009	-0.143	-0.114	-0.28	-0.051	0.0789	0.0196	0.107	-0.19	-0.77	-0.01	-0.52	-0.26	0.011	0.399	0.047	0.153	0.346647	0.225546	0.171793316	0.05375297
2158	P61201	COPS2	COP9 signalosome subunit 2	9318	ENSG00000166200	17	0.125	0.2	0.014	-0.26	0.049	-0.42	-0.15	0.076	0.08	0.2602	0.1925	0.2056	0.1401	-0.125	-0.058	0.2279	0.0922	0.2181	-0.1	-0.57	0.072	-0.41	-0.17	-0.03	0.06	-0.1	0.168	0.158887	0.0878	-0.159559127	0.247359335
2073	P53602	MVD	mevalonate diphosphate decarboxylase	4597	ENSG00000167508	12	0.19	0.113	-0.09	-0.11	0.152	-0.59	0.21	-0.08	0.155	0.3516	-0.009	0.0398	0.1443	-0.161	0.0656	0.0204	0.1508	0.2417	-0.13	-0.94	-0.14	-0.56	-0.2	0.115	-0.01	0.054	0.156	0.240297	0.177986	-0.099284837	0.277271224
3422	Q9HA64	FN3KRP	fructosamine 3 kinase related protein	79672	ENSG00000141560	14	0.248	-0.23	-0.3	0.025	0.045	-0.29	0.305	-0.04	0.14	0.0159	0.3241	-0.107	-0.115	0.1322	0.1639	0.0592	0.2437	0.0183	0.02	-0.42	-0.71	-0.42	-0.18	0.099	0.173	0.228	-0.01	0.335919	0.124857	-0.091805153	0.216662359
3060	Q92930	RAB8B	RAB8B, member RAS oncogene family	51762	ENSG00000166128	14	-0.15	0.264	-0.3	0.302	-0.11	-0.31	0.174	0.021	0.077	0.1049	0.0521	-0.046	0.3229	-0.123	0.1774	0.46	0.2583	0.2589	-0.37	-0.79	-0.42	-0.48	-0.4	-0.01	-0.23	0.048	0.438	0.106861	0.243017	-0.166433899	0.409451241
3105	Q96C23	GALM	galactose mutarotase	130589	ENSG00000143891	11	-0.04	0.18	0.242	0.152	0.444	-0.19	0.022	-0.12	0.156	-0.033	0.3307	0.0285	-0.01	-0.003	0.2844	-0.046	0.1889	0.0656	-0.42	-0.71	-0.07	-0.53	-0.23	0.027	0.281	0.111	0.312	0.241348	0.231963	0.005673819	0.226289608
3537	Q9NYL9	TMOD3	tropomodulin 3	29766	ENSG00000138594	12	0.094	0.033	-0.09	-0.07	0.08	-0.23	-0.11	-0.06	0.032	-0.006	-0.017	-0.042	0.0104	0.0004	0.2937	-0.11	0.1492	-0.073	-0.26	-0.78	-0.06	-0.2	-0.25	0.013	0.111	-0.03	0.056	0.273968	0.119166	-0.059185186	0.178351305
1103	O43182	ARHGAP6	Rho GTPase activating protein 6	395	ENSG00000047648	20	-0.03	-0.06	-0.28	0.019	0.545	-0.1	0.043	-0.05	0.066	-0.077	0.1784	0.0202	0.0754	-0.166	0.3227	0.2431	0.1278	0.1694	-0.37	-0.53	-0.08	-0.18	-0.07	0.101	0.114	-0.03	0.173	0.303193	0.112937	-0.082914708	0.195851525
612	E7EU05	CD36	CD36 molecule	948	ENSG00000135218	10	-0.19	-0.11	0.305	0.176	0.264	0.158	-0.17	0.561	-0.16	-0.161	-0.118	-0.332	0.3462	-0.077	0.1404	0.3414	-0.818	0.4414	-0.36	-0.29	0.479	0.063	-0.19	-0.31	-0.02	0.38	-0.28	0.711558	0.151281	0.118914777	0.032366305
1018	O00506	STK25	serine/threonine kinase 25	10494	ENSG00000115694	14	0.139	0.213	-0.16	0.084	0.073	-0.44	0.14	-0.24	-0.1	-0.042	0.0202	0.1772	0.068	-0.325	0.0937	0.0987	0.2534	0.3649	-0.27	-0.35	-0.52	-0.39	-0.39	-0.18	-0.12	0.034	0.711	0.339157	0.131585	-0.111489509	0.243074447
3138	Q96H20	SNF8	SNF8 subunit of ESCRT-II;SNF8, ESCRT-	11267	ENSG00000159210	11	0.005	0.127	-0.43	0.231	0.176	-0.46	0.17	0.36	-0.1	-0.404	-0.121	0.3908	-0.227	-0.178	0.0435	0.3575	0.2932	0.4813	-0.14	-0.1	-0.43	-0.49	-0.39	-0.35	-0.05	0.15	0.747	0.622625	0.125325	-0.061834607	0.187159506
1126	O43598	DNPH1	2'-deoxynucleoside 5'-phosphate N-hydrolas	10591	ENSG00000112667	7	0.324	0.511	0.164	-0.1	0.118	-0.78	0.04	0.103	0.311	-0.002	0.1793	0.3125	-0.078	-0.262	0.0819	-0.04	0.0369	0.0581	-0.07	-0.83	-0.08	-1.01	-0.6	0.112	0.064	0.015	-0.31	0.171888	0.378082	0.045053532	0.333028827
3564	Q9P265	DIP2B	disco interacting protein 2 homolog B	57609	ENSG00000066084	15	0.028	0.174	-0.07	0.111	-0.02	-0.18	0.097	0.071	0.102	0.1055	0.008	0.1713	0.3957	-0.117	-0.111	0.0961	0.0242	0.0698	-0.22	0.049	-0.26	-0.53	-0.21	0.131	-0.05	0.158	0.392	0.499951	0.094455	-0.03678674	0.131241788
1859	P35475	IDUA	iduronidase alpha-L-;iduronidase, alpha-L-;a	3425	ENSG00000127415	19	-0.38	-0.56	-0.19	-0.55	-0.34	-0.38	-0.31	-0.18	-0.05	0.4463	0.2383	0.5143	-0.357	0.1267	-0.51	-0.333	-0.434	-0.423	0.661	0.41	0.045	0.286	0.572	-0.17	0.257	0.089	-0.54	0.115028	-0.50471	-0.244256572	-0.260449314
3739	Q9Y3C8	UFC1	ubiquitin-fold modifier conjugating enzyme 1	51506	ENSG00000143222	7	-0.14	0.771	-0.13	0.313	-0.33	0.166	0.128	0.133	0.295	0.1813	-0.145	0.0036	-0.095	0.2521	0.1838	0.1259	0.3063	0.2131	-0.11	-0.58	-0.26	-0.01	-0.36	-0.08	-0.01	-0.05	0.225	0.176836	0.271058	0.0190744	0.25198371
2244	P68133	ACTA1	actin alpha 1, skeletal muscle;actin, alpha 1,	58	ENSG00000143632	22	0.31	-0.7	0.45	0.289	0.326	0.357	-0.71	-0.53	-0.2	-0.092	-0.553	0.0473	0.4559	-0.47	0.1072	-0.502	-0.965	-0.988	-0.25	-1.39	0.224	1.023	0.041	0.131	-0.11	-0.3	-0.63	0.669155	0.094833	0.283376163	-0.18854292
1194	O75165	DNAJC13	DnaJ heat shock protein family (Hsp40) mer	23317	ENSG00000138246	28	8E-04	0.102	-0.13	-0.04	0.031	-0.36	-0.22	0.037	0.126	0.0246	-0.075	0.2692	0.1022	-0.058	-0.197	0.5393	0.2446	0.2518	-0.22	-0.26	0.132	-0.03	-0.17	0.134	0.304	0.073	0.137	0.338209	-0.06263	-0.173253807	0.110619737
1598	P13807	GYS1	glycogen synthase 1	2997	ENSG00000104812	17	0.052	0.173	-0.09	-0.07	0.067	-0.51	-0.07	0.887	0.057	-0.044	-0.141	0.327	0.2194	-0.199	-0.006	0.091	-0.021	0.2895	-0.24	0.138	0.238	0.552	-0.28	0.225	0.47	0.155	0.445	0.668687	-0.13336	-0.001877454	-0.131484808
3769	Q9Y5K8	ATP6V1D	ATPase H+ transporting V1 subunit D	51382	ENSG00000100554	15	0.041	-0.04	-0.32	-0.16	0.061	-0.17	0.097	0.136	0.379	0.0167	-0.023	0.0478	-0.041	-0.031	0.0368	0.4424	-0.125	0.2641	-0.16	-0.75	-0.13	-0.5	-0.33	0.02	0.138	0.157	0.273	0.365485	0.145103	-0.063140692	0.208243557
3409	Q9H832	UBE2Z	ubiquitin conjugating enzyme E2 Z	65264	ENSG00000159202	13	0.149	0.052	-0.06	0.027	0.021	-0.33	0.046	0.123	0.215	0.0958	-0.071	0.008	0.1485	-0.037	0.1212	0.125	0.3604	0.2137	-0.11	-0.84	-0.21	-0.17	-0.27	0.059	0.083	-0.03	0.175	0.172737	0.173583	-0.079178054	0.252760823
476	B8ZZU8	ELOB	elongin B	6923	ENSG00000103363	7	0.21	0.236	0.03	0.256	0.351	-0.32	-0.14	-0.1	0.062	0.167	0.0579	0.0671	0.1533	0.028	0.1871	-0.065	0.1594	-0.193	-0.27	-0.9	-0.31	-0.69	-0.3	0.071	-0.11	-0.08	-0.01	0.070189	0.354836	0.001813657	0.353022791
1183	O60888	CUTA	cutA divalent cation tolerance homolog	51596	ENSG00000112514	6	-0.02	-0.08	-0.03	-0.16	0.12	-0.38	0.21	0.312	0.401	0.1048	-0.032	0.0739	-0.389	0.0166	0.0146	0.2943	-0.029	-0.018	-0.14	-0.25	-0.11	-0.56	-0.43	-0.25	0.147	0.43	0.086	0.526473	0.159184	0.037266282	0.121917639
1632	P15907	ST6GAL1	ST6 beta-galactoside alpha-2,6-sialyltransfe	6480	ENSG00000073849	8	-0.07	0.026	-0.23	0.13	-0.06	-0.05	-0.6	-0.32	0.207	0.0542	-0.228	0.4426	0.0181	-0.151	-0.215	0.0086	0.1925	-0.154	0.127	-0.18	0.35	0.084	-0.4	-0.12	0.044	0.052	0.123	0.633553	-0.11605	-0.10377957	-0.012267196
1852	P35221	CTNNA1	catenin alpha 1	1495	ENSG00000044115	20	-0.03	0.047	0.13	-0.31	-0.03	-0.2	-0.08	-0.06	0.22	0.2237	0.1999	0.011	-0.099	-0.016	0.0693	-0.027	0.2733	0.022	-0.17	-0.55	-0.1	-0.67	-0.24	-0.03	0.109	0.083	0.044	0.175927	0.135655	-0.109055019	0.244710037
801	P12268	IMPDH2	inosine monophosphate dehydrogenase 2	3615	ENSG00000178035	14	0.047	-0.13	-0.06	-0.06	0.163	-0.14	-0.13	-0.23	0.01	-0.146	-0.049	-0.084	0.0746	-0.072	0.2299	-0.318	0.1163	-0.069	-0.06	-0.57	-0.07	-0.11	-0.04	0.073	0.233	-0.1	-0.05	0.916671	0.017305	-0.023969322	0.041274512
1855	P35268	RPL22	ribosomal protein L22	6146	ENSG00000116251	6	0.053	-0.12	-0.23	-0.13	0.227	0.014	0.514	0.192	0.354	-0.018	-0.035	-0.088	-0.434	-0.213	-0.006	0.2551	0.0964	0.2377	-0.11	-0.35	-0.01	-0.4	-0.44	-0.07	0.497	-0.02	0.36	0.575911	0.157626	0.12007026	0.037555947
3417	Q9H993	ARMT1	acidic residue methyltransferase 1	79624	ENSG00000146476	9	0.053	0.051	-0.01	0.349	0.113	-0.34	-0.15	-0.03	0.176	0.2146	0.2585	0.0568	-0.115	-0.041	-0.037	-0.228	0.0683	0.022	-0.1	-0.86	-0.17	-0.39	-0.33	-0.01	-0.18	0.045	-0.07	0.138844	0.253296	0.001360457	0.251935837
1678	P19784	CSNK2A2	casein kinase 2 alpha 2	1459	ENSG00000070770	11	0.138	0.232	0.018	0.031	0.07	-0.51	0.218	0.05	0.336	0.1467	0.0324	-0.01	0.055	-0.277	-0.088	0.1071	0.2263	0.208	-0.35	-0.68	-0.12	-0.28	-0.39	0.183	0.167	0.028	0.262	0.356481	0.196026	0.020845867	0.175179698
2143	P60983	GMFB	glia maturation factor beta	2764	ENSG00000197045	8	0.145	0.303	0.028	0.023	-0.06	-0.41	0.037	0.017	0.127	0.062	-0.059	-0.041	-0.006	-0.21	0.0606	0.0194	0.2562	0.0429	-0.18	-0.74	0.155	-0.26	-0.37	-0.02	-0.17	-0.03	0.172	0.283014	0.183666	0.009371743	0.174294576
157	Q08345	DDR1	discoidin domain receptor tyrosine kinase 1	780	ENSG00000204580	6	-0.2	0.309	-0.38	-0.11	-0	0.308	-0.21	0.143	0.022	0.2281	-0.176	0.5261	-0.33	-0.301	-0.362	0.1723	-0.092	-0.179	-0.08	0.32	0.35	0.149	-0.38	0.159	0.049	-0.06	0.383	0.589584	-0.11216	0.042991247	-0.155155551
531	C9JQ42	GYG1	glycogenin 1	2992	ENSG00000163754	6	-0	-0.34	-0.2	-0.01	0.033	-0.33	0.029	-0.11	0.093	-0.086	-0.096	-0.062	-0.162	-0.086	0.0607	0.4681	-0.178	0.0876	-0.24	-0.41	0.223	0.167	-0.22	9E-04	0.161	0.244	-0.21	0.759594	-0.0602	-0.087168578	0.026966829
3623	Q9UHY7	ENOPH1	enolase-phosphatase 1	58478	ENSG00000145293	11	0.371	0.466	0.197	-0.3	-0.14	-0.45	0.035	0.074	0.256	0.2378	0.3206	0.3444	-0.221	0.1246	-0.036	0.0955	0.1057	0.2048	0.075	-1.04	-0.14	-0.39	-0.42	-0.05	-0.08	0.033	0.276	0.212215	0.249814	-0.073670658	0.323484302
2118	P56192	MARS;MAI	methionyl-tRNA synthetase;methionyl-tRNA	4141	ENSG00000166986	23	-0.04	-0.07	0.016	0.239	0.135	-0.21	0.066	0.016	0.228	-0.073	-0.128	0.1277	-0.059	-0.19	-0.171	0.0953	0.1201	0.2234	-0.2	-0.57	-0.03	-0.29	-0.21	-0.01	0.34	-0	0.134	0.496471	0.134713	0.047323533	0.087389738
3696	Q9Y223	GNE	glucosamine (UDP-N-acetyl)-2-epimerase/N	10020	ENSG00000159921	16	0.249	0.212	0.015	-0.09	0.068	-0.49	-0.35	-0.07	0.04	0.1225	0.0548	0.1935	0.3377	-0.102	0.0349	-0.101	0.1423	0.0836	-0.23	-0.57	-0.01	-0.57	-0.31	-0.01	-0.18	-0.05	0.024	0.126954	0.165905	-0.129919526	0.295824964
2952	Q8NFW9	MYRIP	myosin VIIA and Rab interacting protein	25924	ENSG00000170011	13	-0.87	-0.78	-0.83	-0.17	-0.08	-0.09	0.678	0.153	0.677	-0.588	-0.673	-0.603	0.0267	0.0941	-0.002	0.5393	0.0679	0.4929	-0.92	-0.48	-0.17	-0.49	-0.26	0.318	0.082	0.182	0.239	0.941414	0.021347	-0.073329972	0.094677345
461	B7Z613	GPM6B	glycoprotein M6B	2824	ENSG00000046653	5	-0.22	0.197	-0.11	-0.24	-0.08	0.449	-0.18	-0.25	-0.37	-0.38	0.4075	-0.102	-0.113	-0.382	0.0137	0.3091	0.4663	0.3382	-0.34	0.22	-0.14	-0.32	-0.16	-0.37	-0.41	-0.25	0.499	0.496089	0.053039	-0.150462498	0.20350186
1643	P16930	FAH	fumarylacetoacetate hydrolase	2184	ENSG00000103876	13	0.231	0.406	0.249	0.125	-0	-0.41	0.16	0.168	0.352	0.2179	-0.136	0.2114	0.0516	-0.045	0.1212	0.1719	-0.063	-0.096	-0.16	-0.99	-0.31	-0.68	-0.35	-0.06	0.113	0.237	-0.03	0.118765	0.390453	0.094478152	0.29597524
3590	Q9UBV8	PEF1	penta-EF-hand domain containing 1	553115	ENSG00000162517	8	0.062	0.508	0.122	0.202	0.076	-0.13	-0.32	0.002	-0.32	-6E-04	0.1242	0.3562	0.1692	-0.19	0.0209	0.0505	-0.003	0.1272	-0.05	-0.33	-0.52	-0.27	-0.33	-0.1	-0.19	-0.13	5E-04	0.103448	0.235288	-0.049662645	0.284950647
1435	P05186	ALPL	alkaline phosphatase, biomineralization assc	249	ENSG00000162551	17	-1.84	-0.66	-1.11	-0.08	-1.53	-0.99	-1.06	0.04	-1.32	1.0284	-0.28	-0.646	-0.749	-1.798	-0.116	0.535	0.6268	-0.987	-0.18	3.038	-1.05	-1.06	-0.73	-1.07	-0.42	-1.12	-1.11	0.478059	-0.54108	-0.686469931	0.145388631
1591	P13688	CEACAM1	CEA cell adhesion molecule 1;carcinoembry	634	ENSG00000079385	9	-0.83	0.187	-0.05	-0.17	-0.05	-0.72	-0.4	-0.51	-0.15	-0.204	-0.079	0.0527	-0.143	-0.005	-0.32	-0.113	-0.134	0.5056	-0.15	0.572	0.044	0.447	-0.41	0.058	0.245	-0.04	0.277	0.129935	-0.4133	-0.249750598	-0.163544738
288	A0A1W2PN'	STAT6	signal transducer and activator of transcripti	6778	ENSG00000166888	16	-0.1	-0.17	-0.28	0.109	0.099	-0.34	0.002	-0.08	0.133	-0.052	-0.047	0.0399	0.0704	0.0147	0.0291	0.1104	0.145	0.0761	-0.25	-0.59	-0.4	-0.4	-0.14	-0.03	0.09	0.013	0.307	0.256857	0.086749	-0.112709925	0.199458971
736	F8VXB1	COPZ1	coatomer protein complex subunit zeta 1	22818	ENSG00000111481	3	-0.09	0.911	0.464	-0.03	0.094	-0.35	0.345	-0.08	0.332	0.8193	-0.077	0.6265	0.1376	-0.363	0.008	0.0649	0.4526	-0.129	0.421	-0.06	0.458	-0.51	-0.16	0.08	0.332	0.06	-0.17	0.793582	0.126474	0.005872788	0.120601671
3013	Q8WVM8	SCFD1	sec1 family domain containing 1	23256	ENSG00000092108	19	-0.04	0.208	-0.03	0.134	0.007	-0.32	-0.18	-0	0.22	0.1075	-0.193	-0.097	0.1497	-0.287	0.094	0.0031	0.063	-5E-04	-0.09	-0.42	0.007	-0.26	-0.23	0.087	-0.18	-0.01	-0.04	0.375457	0.125772	0.017001275	0.108770646
2799	Q86SF2	GALNT7	polypeptide N-acetylgalactosaminyltransfera	51809	ENSG00000109586	18	0.043	0.359	-0.18	-0.12	0.059	-0.46	-0.37	-0.38	0.147	0.0023	-0.151	0.0423	0.1307	-0.053	-0.373	0.4773	0.1523	0.0823	-0.36	0.175	0.251	-0.07	-0.27	-0.09	-0.3	-0.16	0.04	0.579014	-0.01245	-0.134656022	0.122201453
716	F5H5D3	TUBA1C	tubulin alpha 1c	84790	ENSG00000167553	33	0.226	0.264	-0.1	0.073	-0.35	-0.23	0.142	0.094	0.172	0.3568	-0.045	0.2121	-0.475	-0.095	-0.249	-0.127	-0.021	-0.007	-0.13	-0.77	-0.09	0.624	-0.35	0.247	-0.25	-0.1	0.737	0.911906	0.042055	0.083455869	-0.041400933
2819	S4R3V8	LSR	lipolysis stimulated lipoprotein receptor	51599	ENSG00000105699	16	-0.17	-0.51	-0.15	-0.22	-0.06	0.027	-0.42	0.297	-0.12	0.1031	0.3403	-0.109	-0.521	0.6086	-0.197	0.1009	-0.555	0.03	-0.02	0.369	0.293	0.075	-0.07	-0.09	0.311	0.286	-0.36	0.411082	-0.2355	-0.125394625	-0.110101465
771	F8WE17	DPP4	dipeptidyl peptidase 4	1803	ENSG00000197635	10	-0.09	0.662	-0.14	0.223	-0.3	0.426	-0.29	-0.61	-0.42	-0.492	0.1825	0.6902	-0.163	-0.205	0.1051	-0.207	-0.457	0.0591	-0.08	0.309	-0.71	-0.39	-0.41	-0.33	-0.26	-0.19	0.048	0.660709	0.164029	-0.004757911	0.168787267
3618	Q9UHL4	DPP7	dipeptidyl peptidase 7	29952	ENSG00000176978	17	-0.22	-1.27	-0.74	0.16	-1.04	0.015	-0.37	-0.12	0.205	0.1239	-0.349	0.2853	-0.45	0.6399	-0.46	0.0716	-0.597	-0.223	-0.78	0.511	1.062	0.071	-0.21	-0.45	0.645	-0.06	-0.39	0.395301	-0.42004	-0.269425752	-0.150618434
2974	Q8TCD5	NT5C	5', 3'-nucleotidase, cytosolic	30833	ENSG00000125458	12	0.401	0.242	0.137	-0.02	0.138	-0.39	-0.04	-0.25	-0.01	0.1776	0.4388	0.2254	0.0994	-0.027	0.2621	0.1339	0.0791	-0.048	0.002	-0.89	-0.45	-0.68	-0.32	0.087	-0.07	0.03	0.015	0.102025	0.274249	-0.12621279	0.400462121
3384	Q9H3Z4	DNAJC5	DnaJ heat shock protein family (Hsp40) mer	80331	ENSG00000101152	6	0.082	0.406	0.251	-0.16	-0.17	-0.32	-0.92	-0.61	-0.53	0.2272	0.3807	0.0289	0.0029	-0.215	0.1906	-0.41	0.0505	-0.496	-0.19	-0.5	-0.2	-0.48	-0.29	0.2	-0.25	-0.43	-0.53	0.374333	0.075226	-0.193526976	0.268752799
2512	Q15293	RCN1;AL0	reticulocalbin 1;novel protein	5954	ENSG00000049449	8	-0.28	-0.82	-0.07	-0.37	-0.34	-0.44	0.103	0.288	-0.05	0.1839	-0.065	-0.059	-0.492	0.1766	-0.03	0.5693	-0.924	0.1082	0.208	0.819	0.252	-0.19	-0.06	-0.32	0.301	0.014	-0.52	0.489229	-0.27482	-0.159281434	-0.11553388

2829	Q86YS6	RAB43	RAB43, member RAS oncogene family	339122	ENSG00000172780	10	-0.13	0.254	-0.07	0.156	-0.36	-0.22	0.244	0.195	0.072	0.1168	0.168	-0.013	0.1731	-0.266	0.1661	0.1977	0.1032	-0.052	-0.23	-1.45	-0.42	-0.4	-0.35	0.221	0.089	0.122	0.397	0.347694	0.2413	-0.049457053	0.290757442
1053	O14863	SLC30A4	solute carrier family 30 member 4	7782	ENSG00000104154	9	0.269	0.083	-0.11	0.045	-0.02	-0.5	-0.47	-0.07	-0.22	0.1446	0.1226	0.1903	0.1491	-0.248	-0.015	0.1275	0.1922	0.1189	-0.26	-0.88	-0.36	-0.51	-0.15	0.01	-0.12	-0.07	0.08	0.128896	0.139829	-0.197383799	0.337212626
3295	Q9BW83	IFT27	intraflagellar transport 27	11020	ENSG00000100360	12	-0.03	0.167	0.063	0.105	0.076	-0.41	0.081	0.052	0.162	0.0233	0.1455	0.0368	0.0709	-0.095	0.0993	-0.035	0.117	-0.226	-0.27	-0.81	-0.1	-0.47	-0.29	0.001	0.139	0.137	0.068	0.241877	0.207467	0.014049123	0.193418085
3266	Q9BTY2	FUCA2	alpha-L-fucosidase 2	2519	ENSG00000001036	12	-0.42	-0.59	-0.01	0.014	-0.19	0.37	0.057	0.057	0.278	0.5151	0.0788	0.3036	-0.465	-0.138	-0.48	0.211	-0.374	-0.254	-0.08	0.114	-0.29	0.345	0.171	-0.25	0.456	0.759	-0.59	0.787708	-0.11687	0.019662326	-0.136529321
1919	P42345	MTOR	mechanistic target of rapamycin kinase	2475	ENSG00000198793	29	-0.03	0.343	-0.14	-0.14	0.013	-0.22	-0.04	-0.07	0.07	-0.093	0.0616	0.292	0.0275	-0.103	0.0002	0.0652	0.0711	0.08	-0.16	-0.35	-0.38	-0.14	-0.18	0.012	0.161	-0.07	0.076	0.258991	0.091135	-0.067323034	0.158458359
2763	Q7L2H7	EIF3M	eukaryotic translation initiation factor 3 subu	10480	ENSG00000149100	9	0.168	0.117	0.044	0.012	0.096	-0.33	-0.22	-0.43	0.01	0.0252	0.0921	0.0457	0.1251	-0.126	-0.043	-0.183	0.0798	0.1207	-0.16	-0.5	0.059	-0.13	-0.3	0.132	0.028	-0.16	0.068	0.525415	0.04712	-0.074452083	0.121572327
1913	P41252	IARS1;IAR	isoleucyl-tRNA synthetase 1;isoleucyl-tRNA	3376	ENSG00000196305	25	-0.03	0.045	0.024	0.231	0.062	-0.1	-0.07	-0.13	0.049	-0.016	-0.03	0.0653	0.0237	-0.132	0.1004	-0.146	-0.207	0.0202	-0.14	-0.25	0.115	-0.03	-0.2	0.016	0.257	-0.1	-0.03	0.769843	0.048684	0.043578883	0.005105418
2907	Q8N695	SLC5A8	solute carrier family 5 member 8	160728	ENSG00000256870	8	-1.09	0.794	-0.45	-0.78	-0.32	-0.08	-0.44	-0.05	-0.68	0.1897	0.8213	-0.12	-0.336	-0.079	-0.262	-0.083	-0.286	0.3905	0.913	1.433	-0.71	-0.78	-0.9	-0.77	-0.3	-0.2	-0.1	0.587076	-0.18631	-0.369080208	0.182770435
2989	Q8TE68	EPS8L1	EPS8 like 1	54869	ENSG00000131037	23	0.031	0.372	-0.44	0.118	-0.05	-0	-0.11	0.025	-0.06	-0.302	-0.151	0.312	-0.336	-0.31	-0.236	0.1494	-0.256	0.2651	-0.15	-0.27	-0.2	0.005	-0.59	-0.41	-0.05	0.047	0.56	0.780812	0.105719	0.083386902	0.022332091
1310	O95816	BAG2	BCL2 associated athanogene 2	9532	ENSG00000112208	10	0.385	0.322	-0.1	0.037	0.474	-0.46	-0.24	-0.32	0.047	0.1094	-0.027	-0.128	0.0526	-0.043	-0.069	-0.168	0.0451	-0.205	-0.12	-1.03	-0.2	-0.69	-0.41	-0.12	-0.02	-0.31	-0.06	0.147072	0.344953	0.063165478	0.281787152
3547	Q9NZD2	GLTP	glycolipid transfer protein	51228	ENSG00000139433	9	0.116	0.317	0.11	0.143	-0.09	-0.08	-0.11	-0.28	-0.2	0.2444	0.3113	0.2793	0.0026	-0.235	0.0062	-0.038	0.0163	-0.268	-0.24	-0.44	-0.3	-0.17	-0.39	0.019	0.12	-0.21	-0.21	0.147178	0.197025	-0.042917858	0.239943316
1580	P12955	PEPD	peptidase D	5184	ENSG00000124299	14	-0.06	-0.18	-0.25	-0.06	0.353	-0.62	0.027	-0.11	0.184	-0.079	-0.287	-0.111	-0.037	0.1719	0.1874	0.2963	0.1465	0.0591	-0.51	-0.52	-0.31	-0.35	-0.1	0.137	0.067	0.165	0.041	0.431707	0.073485	-0.118000362	0.191485005
1303	O95747	OXSR1	oxidative stress responsive kinase 1;oxidativ	9943	ENSG00000172939	17	0.106	0.113	-0.15	0.002	0.029	-0.34	-0.06	0.022	0.234	0.1176	-0.032	0.2116	0.0859	-0.192	0.0089	0.2564	0.1044	0.0909	-0.25	-0.94	-0.31	-0.63	-0.25	0.113	0.135	-0.04	0.179	0.177545	0.217079	-0.077283193	0.294361699
408	B1AKJ5	NRDC	nardilysin convertase	4898	ENSG00000078618	20	0.12	0.263	-0.07	0.064	0.184	-0.31	-0.48	-0.42	-0.22	0.2845	-0.026	-0.013	-0.003	-0.134	-0.1	-0.203	-0.097	-0.348	-0.27	-0.49	-0.2	-0.45	-0.29	-0.06	-0.15	-0.18	-0.18	0.291516	0.156219	-0.025868314	0.182087487
336	P68400	CSNK2A1	casein kinase 2 alpha 1	1457	ENSG00000101266	14	0.116	0.2	-0.05	-0.03	-0.12	-0.43	0.315	0.157	0.363	-0.016	-0.126	-0.084	-0.19	-0.262	-0.089	0.2679	0.2033	0.1847	-0.28	-0.79	-0.26	-0.25	-0.51	-0.13	0.204	0.13	0.371	0.386618	0.22417	0.069487401	0.154682682
185	C9J9K3	RPSA	ribosomal protein SA	3921	ENSG00000168028	7	0.014	-0.17	-0.15	-0.1	0.173	-0.31	-0.16	-0.19	-0.07	-0.177	0.0585	-0.119	-0.182	-0.399	0.0746	-0.257	0.0275	-0.076	-0.1	-0.09	0.053	-0.22	0.115	-0	0.078	-0.24	-0.04	0.680903	-0.05689	0.009538915	-0.066433077
3788	Q9Y696	CLIC4	chloride intracellular channel 4	25932	ENSG00000169504	11	0.215	0.136	-0.11	0.052	0.201	0.168	0.022	0.349	0.217	0.1296	0.1078	-0.013	0.2796	-0.194	0.0128	0.2665	0.1096	0.3645	-0.14	-0.39	-0.19	-0.16	-0.08	-0.16	-0.01	-0	0.17	0.061984	0.245721	0.020927666	0.224793202
1828	P31946	YWHAB	tyrosine 3-monooxygenase/tryptophan 5-mo	7529	ENSG00000166913	20	0.148	0.383	-0.05	-0.2	0.07	-0.6	0.248	0.204	0.424	0.0403	-0.081	-0.065	0.0057	-0.059	0.3981	0.2649	0.3818	0.314	-0.13	-1.24	-0.25	-0.84	-0.37	-0.02	-0.14	0.245	0.553	0.235627	0.313429	-0.063398133	0.376827568
2177	P61970	NUTF2	nuclear transport factor 2	10204	ENSG00000102898	5	0.439	0.267	0.085	0.08	0.175	-0.39	0.057	0.05	0.124	0.1002	0.3142	0.2036	-0.002	-0.154	-0.029	-0.095	0.069	-0.127	-0.26	-0.99	-0.12	-0.6	-0.46	-0.04	-0.2	-0.08	0.089	0.082399	0.393272	0.067939516	0.325332852
1337	P00918	CA2	carbonic anhydrase 2	760	ENSG00000104267	10	0.184	-0.38	-0.24	-0.03	0.361	-0.1	0.351	0.23	-0.21	-0.335	-0.28	0.3204	-0.21	-0.127	0.0474	-0.581	-0.287	-0.062	-0.74	-0.59	0.529	-0.62	-0.22	-0.36	0.512	-0.28	-0.28	0.450881	0.247116	0.188518649	0.058597296
1893	P40189	IL6ST	interleukin 6 signal transducer	3572	ENSG00000134352	13	-0.37	-0.43	-0.23	-0.04	-0.17	-0.17	-0.19	0.053	-0.14	0.4816	-0.049	0.2544	-0.387	0.0721	-0.478	0.2712	-0.222	-0.069	-0.19	0.095	-0.01	0.327	-0.02	-0.23	0.162	0.339	-0.39	0.359745	-0.19704	-0.174467349	-0.022574645
1439	P05387	RPLP2	ribosomal protein lateral stalk subunit P2	6181	ENSG00000177600	7	-0.04	0.233	-0.03	-0.09	0.068	-0.41	-0.03	-0.21	0.175	0.0986	0.058	0.1151	0.0002	-0.401	0.1494	-0.144	0.1883	0.0119	-0.22	-0.71	-0.33	-0.74	-0.42	0.102	0.189	-0.12	-0.08	0.198772	0.220333	-0.04519684	0.265529579
1319	O95967	EFEMP2	EGF containing fibulin extracellular matrix pı	30008	ENSG00000172638	15	-0.21	-0.37	-0.38	-0.07	-0.06	-0.14	-0.08	0.088	0.059	-0.051	0.2077	0.0117	-0.321	0.4567	-0.141	0.1813	0.0023	0.1013	-0.18	0.471	0.253	-0.01	-0.07	0.007	0.191	0.062	0.062	0.193598	-0.21677	-0.178623549	-0.038141599
1322	O96013	PAK4	p21 (RAC1) activated kinase 4	10298	ENSG00000130669	15	0.055	0.241	-0.26	-0.06	-0	-0.28	-0.21	-0.05	-0.13	-0.046	0.1156	0.1897	-0.063	0.007	0.1274	0.1044	-0.066	0.1147	-0.2	-0.24	-0.26	-0.3	-0.11	-0.11	-0.06	-0.02	0.121	0.129444	0.053475	-0.131096964	0.184571484
3160	Q96KN4	LRATD1;F	LRAT domain containing 1;family with sequ	151354	ENSG00000162981	11	-0.78	0.627	0.207	-0.03	0.094	-0.24	0.348	-0.09	0.573	0.3087	0.2133	0.0316	0.0997	-0.378	0.0288	-0.15	0.1866	-0.443	-0.39	-0.77	-0.63	-0.94	-0.88	0.333	-0.06	0.181	-0.18	0.189619	0.449533	0.089823362	0.359709583
3498	Q9NS86	LANCL2	LanC like 2	55915	ENSG00000132434	13	0.052	0.06	0.03	0.034	0.099	-0.24	-0.18	-0.16	0.193	0.1828	0.2025	0.1653	0.0929	-0.167	0.2476	0.0023	0.1519	0.0932	-0.11	-0.59	0.017	-0.38	-0.2	0.083	-0.04	-0.07	0.076	0.126162	0.121556	-0.120905009	0.242461258
2478	Q14749	GNMT	glycine N-methyltransferase	27232;1070	ENSG00000124713	11	0.001	0.57	-0.38	0.202	0.375	-0.78	-0.24	-0.24	0.527	0.1068	0.0436	0.1618	0.2879	-0.143	-0.316	0.5296	-0.052	0.1596	0.067	-0.84	-0.22	-0.75	0.291	-0.07	-0.13	0.072	0.299	0.582955	0.148774	-0.08123336	0.230007286
95	A0A087X0R	SNX12	sorting nexin 12	29934	ENSG00000147164	12	0.116	0.241	0.136	0.004	-0.2	-0.44	0.155	0.066	0.218	0.1109	0.1373	0.1632	0.1202	-0.143	0.3103	0.2326	0.2634	0.1581	-0.09	-0.73	-0.19	-0.59	-0.19	0.046	0.03	0.132	0.337	0.192906	0.171646	-0.11683572	0.288482168
2181	P62081	RPS7	ribosomal protein S7	6201	ENSG00000171863	10	0.253	-0.03	0.017	-0.26	0.252	-0.24	0.112	-0.12	0.173	-0.01	0.1217	0.0064	-0.19	-0.197	-0.059	-0.083	0.1006	-0.164	-0.03	-0.47	-0.04	-0.46	-0.04	0.014	0.14	-0.23	-0.15	0.343734	0.158984	0.071158254	0.087825555
406	B1AJY7	PSMD10	proteasome 26S subunit, non-ATPase 10	5716	ENSG00000101843	11	0.087	0.327	0.071	-0.03	-0.02	-0.41	0.081	0.1	0.28	0.3195	0.1861	0.2159	-0.099	-0.165	0.1034	0.2235	0.1467	0.1756	-0.09	-0.81	0.127	-0.43	-0.3	0.044	0.115	0.071	0.129	0.233396	0.182115	-0.069159646	0.251274693
2175	P61960	UFM1	ubiquitin fold modifier 1	51569	ENSG00000120686	4	0.048	-0.44	-0.34	-0.11	0.043	-0.28	-0.02	-0.14	0.086	-0.193	0.0673	-0.105	-0.097	-0.1	0.1	-0.043	-0.095	0.0529	-0.32	-0.02	-0.53	-0.44	-0.28	-0.19	0.024	-0.15	0.099	0.339341	0.07377	-0.08186582	0.155635598
797	P16070	CD44	CD44 molecule (Indian blood group)	960	ENSG00000026508	6	-0.27	-0.22	-0.04	-0.3	-0.24	-0.27	0.421	0.038	-0.26	0.2367	-0.198	-0.127	0.0649	0.0741	0.0269	0.0407	-0.44	-0.126	-0.26	0.1	-0.01	-0.07	-0.05	0.113	0.45	0.034	-0.11	0.499714	-0.14781	-0.076832053	-0.070975695
1782	P28799	GRN	granulin precursor	2896	ENSG00000030582	15	-0.2	-0.47	-0.04	-0.01	-0.15	-0.3	0.076	0.045	0.511	0.2506	-0.108	0.289	0.123	-0.003	-0.49	0.5081	-0.147	-0.002	-0.31	-0.05	-0.13	-0.14	-0.09	-0.16	0.397	0.448	-0.24	0.790407	-0.03087	-0.108131653	0.077259873
572	O94979	SEC31A	SEC31 homolog A, COPII coat complex cor	22872	ENSG00000138674	17	0.02	0.002	-0.09	-0.03	0.088	-0.28	0.217	0.343	0.214	0.0419	0.0294	0.0772	0.0299	-0.074	0.1281	0.2065	0.1103	0.394	-0.13	-0.53	-0.08	-0.31	-0.18	0.112	0.508	0.384	0.463	0.865713	0.02761	-0.050842053	0.078452534
551	D3DQ30	TTC39A	tetratricopeptide repeat domain 39A	22996	ENSG00000085831	14	0.118	0.159	0.011	-0.11	-0.09	-0.65	0.214	0.051	0.534	0.0538	0.0413	0.2494	-0.008	-0.418	-0.108	0.192	0.1111	0.2033	-0.26	-1.14	-0.18	-0.63	-0.2	-0.06	-0.28	0.084	0.274	0.23971	0.292139	-0.009643484	0.301782786
2310	Q03426	MVK	mevalonate kinase	4598	ENSG00000110921	13	0.38	0.249	0.271	-0.07	0.038	-0.44	-0.3	0.026	-0.05	0.2976	-0.041	0.2715	0.0851	-0.041	-0.034	-0.169	0.156	-0.018	-0.19	-1.48	-0.39	-0.33	-0.09	0.155	-0.12	-0.06	-0.1	0.205006	0.301861	-0.043741437	0.345602547
2953	Q8NFX7	STXBP6	syntaxin binding protein 6	29091	ENSG00000168952	5	-0	0.14	-0.26	0.051	-0.05	-0.29	-0.13	-0.18	-0.07	-0.054	0.1238	0.544	-0.003	0.0589	0.0376	0.0621	0.0483	-0.055	-0.28	0.122	-0.61	-0.21	-0.59	-0.27	0.057	0.194	0.369	0.293961	0.047434	-0.172009663	0.219443266
177	A0A0B4J23	IGLL5	immunoglobulin lambda like polypeptide 5	100423062	ENSG00000254709	9	-1.63	-1.65	-1.22	-1.54	-1.42	-1.03	-1.19	-1.32	-1.27	-1.147	-1.011	-0.658	-1.712	-0.946	-1.464	-0.783	-1.562	-1.395	-1.47	-0.29	2.756	2.341	-0.5	1.176	-0.32	-0.86	-1.8	0.081988	-1.48045	-0.178860499	-1.30158483
679	E9PN89	HSPA8	heat shock protein family A (Hsp70) membe	3312	ENSG00000109971	29				-0.11	-0.12	-0.76	0.526	0.172	0.389				-0.185	-0.002	0.3993	0.2415	0.1634	0.1453				-1.07	-0.17	0.278	0.286	0.141	0.363	0.878003	0.04303	-0.111388755	0.154419046
2327	Q06481	APLP2	amyloid beta precursor like protein 2	334	ENSG00000084234	23	#####	-0.4	-0.35	-0.04	-0.61	0.319	-0.11	0.178	0.49	0.6607	0.0285	0.5506	0.1108	0.3807	-0.318	1.0076	-0.296	0.573	-0.29	-0.54	-0.03	-0.24	-0.05	0.001	-0.03	0.314	-0.06	0.17443	0.044615	-0.357543604	0.402158572
2358	Q11201	ST3GAL1	ST3 beta-galactoside alpha-2,3-sialyltransfe	6482	ENSG00000008513	12	0.068	-0.28	-0.2	-0.53	-0.18	-0.36	0.4	-0.02	-0.24	0.2018	0.0983	-0.04	-0.366	0.1805	0.1925	-0.034	-0.026	-0.277	0.301	0.331	0.117	-0.28	0.079	-0.11	0.502	0.038	0.294	0.183786	-0.28977	-0.140634128	-0.149133099
1658	P17900	GM2A	GM2 ganglioside activator	2760	ENSG00000196743	6	-0.42	-0.05	0.029	0.046	-0.46	-0.02	-0.16	0.079	0.046	0.4094	-0.207	0.1188	-0.159	0.164	0.0128	0.2546	0.0238	-0.026	-0.21	-0.4	-0.2	-0.3	0.061	0.216	0.011	0.467	-0.07	0.452388	-0.05634	-0.167465997	0.111130753
552	H0Y3P2	EIF4G2	eukaryotic translation initiation factor 4 gamı	1982	ENSG00000110321	19	0.059	0.349	-0.24	-0.07	0.062	-0.37	-0.03	-0.05	0.15	0.1061	-0.127	-0.018	-0.034	-0.191	0.0355	0.1433	0.1449	0.1057	-0.32	-0.36	0.149	-0.43	-0.2	-0.03	0.078	0.045	0.232	0.621029	0.076627	-0.033469393	0.110096025
2797	Q7Z7H5	TMED4	transmembrane p24 trafficking protein 4	222068	ENSG00000158604	6	-0.02	-0.33	0.183	-0.16	0.252	-0.15	0.189	0.431	0.253	-0.002	0.1038	0.1562	-0.172	0.0452	0.1318	-0.139	-0.45	-0.1	-0.36	0.321	-0.17	-0.02	-0.13	-0.1	0.213	0.059	-0.1	0.589798	0.105681	0.120258079	-0.014577064
2043	P51570	GALK1	galactokinase 1	2584	ENSG00000108479	17	-0.31	0.144	-0.24	-0.09	-0.24	-0.22	0.2	0.154	0.239	-0.161	-0.023	-0.205	0.0443	0.0745	0.1927	0.2258	0.1355	0.19	-0.34	-0.57	-0.19	-0.29	-0.21	0.086	0.158	0.067	0.284	0.470112	0.069673	-0.093930607	0.163603564
2099	P54851	EMP2	epithelial membrane protein 2	2013	ENSG00000213853	2	-0.22	-0.06	-0.05				0.271	-0.15	0.202	-0.347	0.0191	0.1098				0.2564	0.4411	0.3316	-0.31	-0.14	-0.33				-0.06	0.041	0.063	0.31362	0.12225	-0.136341549	0.258591187
2720	Q6UWE0	LRSAM1	leucine rich repeat and sterile alpha motif cc	90678	ENSG00000148356	31	-0.04	0.185	-0.05	0.02	0.2	-0.03	-0.1	0.278	0.005	0.0848	0.1075	0.0147	0.0481	-0.135	0.1814	0.1077	0.0606	0.0713	-0.18	-0.56	-0.18	-0.36	-0.19	0.067	0.165	-0.03	0.229	0.20543	0.166519	-0.009207376	0.175726762
2210	P62851	RPS25	ribosomal protein S25	6230	ENSG00000118181	6	0.408	-0.06	0.17	-0.21	0.462	-0.05	0.11	-0.29	0.061	-0.379	-0.06	-0.22	-0.175	######	-0.033	-0.181	0.2032	-0.096	-0.17	-0.63	0.241	-0.19	0.025	0.088	0.201	-0.27	-0.25	0.37469	0.172948	0.172236507	0.000711755
1329	P00450	CP	ceruloplasmin	1356	ENSG00000047457	22	-0.82	-0.76	-0.36	-0.62	-0.75	0.784	-0.27	0.291	-0.66	-0.2	-0.15	-0.483	-0.241	-0.597	-0.714	0.1023	-0.62	-0.764	-0.82	-0.54	1.664	1.639	0.425	0.445	-0.05	-0.28	-0.81	0.275829	-0.53666	0.055924782	-0.592581147
2116	P55884	EIF3B	eukaryotic translation initiation factor 3 subu	8662	ENSG00000106263	19	0.038	0.017	-0.06	0.068	0.203	-0.35	-0.11	-0.17	-0.04	-0.042	0.0297	0.0541	0.1081	-0.034	0.0904	0.0372	0.0915	0.1693	-0.16	-0.4	-0.04	-0.25	-0.2	0.078	0.135	-0.05	0.059	0.230841	0.045447	-0.10255838	0.148005022
2132	P60228	EIF3E	eukaryotic translation initiation factor 3 subu	3646	ENSG00000104408	14	0.088	0.074	-0.07	-0.1	0.217	-0.34	-0.07	0.265	0.122	0.0432	0.0296	0.0779	0.2025	-0.158	-0.015	0.3428	0.0566	0.1117	-0.22	-0.49	0.033	-0.15	-0.12	0.147	0.365	-0.1	0.038	0.518481	0.075087	-0.056573233	0.131660028
2626	Q5SRE7	PHYHD1	phytanoyl-CoA dioxygenase domain containi	254295	ENSG00000175287	9	-0.07	0.483	0.188	-0.27	-0.17	-0.42	-0.23	0.216	0.69	0.6933	0.0286	0.5199	0.1595	-0.41	-1.051	-0.357	0.0912	0.1842	-0.08	-0.67	-0.29	-0.45	-0.03	0.077	-0.18	0.046	0.281	0.722169	0.18984	0.062684066	0.127155942
1998	P49589	CARS;CAF	cysteinyl-tRNA synthetase;cysteinyl-tRNA s	833	ENSG00000278191	16	0.135	0.197	0.009	0.28	0.226	-0.16	-0.07	-0.07	-0.06	0.1678	0.1006	0.1496	-0.004	-0.172	0.0039	-0.189	-0.279	-0.377	-0.18	-0.46	-0.02	-0.35	-0.2	0.045	0.257	-0.27	-0.09	0.266432	0.192841	0.119431796	0.073408916
1282	O95373	IPO7	importin 7	10527	ENSG00000205339	17	0.063	0.04	-0.03	-0.07	0.068	-0.25	-0.25	-0.18	0.011	0.2737	0.1076	0.0706	0.0214	-0.028	0.0396	0.1049	-0.026	0.0506	-0.18	-0.36	-0.23	-0.06	-0.1	0.101	0.113	-0.02	-0.05	0.146555	0.019806	-0.135768995	0.155575414
1758	P26583	HMGB2	high mobility group box 2	3148	ENSG00000164104	1							1.083	-0.08	-0.88							0.678	-0.27	0.1258							0.975	-0.51	-0.98	0.922768	0.209895	-0.138464277	0.348359535
3299	Q9BXI6	TBC1D10A	TBC1 domain family member 10A	83874	ENSG00000099992	12	-0.62	-0.13	-0.27	0.206	-0.05	0.084	0.057	0.208	-0.06	-0.173	0.1586	0.0089	-0.073	0.2502	-0.005	0.1404	0.0429	0.3404	-0.21	-0.05	-0.2	-0.05	-0.37	-0.24	-0.14	0.158	0.436	0.457216	0.010691	-0.140417273	0.151108523
2248	P68366	TUBA4A	tubulin alpha 4a	7277	ENSG00000127824	30	0.186	0.626	0.038	0.001	0.109	-0.74	-0.03	-0.15	-0.05	-0.175	0.0063	0.1337	-0.055	-0.076	0.3631	-0.122	0.2037	-0.335	-0.71	-1.99	-0.29	-0.8	-0.63	-0.24	-0.12	0.125	0.181	0.165532	0.494825	0.005228065	0.489596907
1130	O43653	PSCA	prostate stem cell antigen	8000	ENSG00000167653	3	-0.88	-4.64	-3.99	2.519	-3.13	-1.83	-1.42	1.925	0.902	-3.511	1.3828	-5.106	0.8865	0.5728	-3.161	-1.571	-1.661	-1.652	-5.26	0.135	-3.45	0.913	-2.71	-2.82	1.305	-1.06	-1.18	0.946842	0.39636	0.361865262	0.034494903
1090	O15305	PMM2	phosphomannomutase 2	5373	ENSG00000140650	14	0.078	0.209	0.037	-0.15	0.245	-0.34	-0.21	-0.03	0.087	0.2943	0.3366	0.0824	0.3636	-0.073	-0.021	-0.028	0.287	0.1213	-0.06	-0.23	-0.04	-0.46	-0.23	0.358	0.011	-0.21	0.375	0.28276	0.046159	-0.159565898	0.205724518
1204	O75356	ENTPD5	ectonucleoside triphosphate diphosphohydrı	957	ENSG00000187097	16	0.306	0.159	-0.25	-0.48	-0.08	-0.36	-0.58	0.002	0.193	-0.076	-0.1	0.0904	-0.209	-0.403	-0.017	0.2677	0.5259	0.1742	-0.01	-0.11	0.334	-0.53	-0.7	0.109	-0.11	-0.25	0.119	0.618326	0.005492	-0.150130158	0.155622032
135	A0A0A0MR8	WASHC2C	WASH complex subunit 2C	253725	ENSG00000172661	21	-0.16	-0.23	0.093	0.088	-0.14	-0.25	0.271	0.246	0.395	-0.268	-0.038	-0.269	0.0398	-0.259	-0.001	0.3873	0.4648	0.3924	-0.37	-0.5	0.253	-0.11	-0.34	0.133	0.09	0.029	0.292	0.773626	0.092424	-0.015134098	0.107558022
3286	Q9BV57	ADI1	acireductone dioxygenase 1	55256	ENSG00000182551	10	-0.22	0.421	0.103	-0.01	0.24	-0.34	-0.71	-0.57	-0.3	0.3116	0.0758	0.3118	0.1118	-0.137	0.2374	-0.81	-0.025	-0.651	0.062	-0.82	-0.1	-0.43	-0.06	-0.04	-0.31	-0.17	-0.25	0.717514	0.079169	-0.090039199	0.169208405
3173	Q96P70	IPO9	importin 9	55705	ENSG00000198700	16	-0.03	0.086	-0.16	-0.36	-0.53	0.278	0.011	-0.14	0.145	0.2662	-0.071	0.0887	-0.621	-0.785	-0.579	-0.001	-0.048	-0.025	-0.24	-0.64	-0.24	1.61	0.175	0.216	0.082	-0.11	-0.2	0.589324	-0.15102	0.119484806	-0.270500376
3467	Q9NQS7	INCENP	inner centromere protein	3619	ENSG00000149503	3	0.273	-1.19	-1.29	-0.44	-0.5	-0.85	0.491	0.341	0.68	0.2554	-0.55	-0.21	-0.246	0.4873	-0.111	0.6822	-0.833	0.0969	0.958	0.719	0.034	0.578	0.226	-0.3	0.674	-0	0.072	0.230025	-0.60558	-0.229000669	-0.376583434
77	A0A087WZ3	MAN1C1	mannosidase alpha class 1C member 1	57134	ENSG00000117643	7	0.073	0.196	-0.43	-0.47	0.352	-0.4	-0.28	-0.32	-0.38	-0.185	0.3567	0.0571	-0.447	0.2323	-0.349	0.4199	0.2727	-0.228	-0.4	0.529	0.025	-0.58	-0.62	-0.36	-0.23	-0.03	0.109	0.5303	-0.0119	-0.199441472	0.187546452
746	F8VXK3	CHST11	carbohydrate sulfotransferase 11	50515	ENSG00000171310	7	-0.16	-0.54	-0.33	-0.11	-0.22	-0.22	-1.01	-0.69	-1.11	-0.016	0.1125	-0.034	-0.272	0.2044	0.129	-1.196	-1.387	-0.954	0.394	0.618	0.19	-0.05	0.099	-0.02	-0.32	-1.11	-0.98	0.525259	-0.35448	-0.107282996	-0.247199109
1840	P33908	MAN1A1	mannosidase alpha class 1A member 1	4121	ENSG00000111885	14	-0.04	-0.6	0.305	-0.51	0.404	-0.91	-0.51	-0.23	0.545	0.0521	0.0208	0.5806	-0.105	-0.208	-0.711	-0.146	-0.049	-0.053	0.248	0.429	0.242	0.379	-0.75	0.036	0.132	-0.52	-0.29	0.801073	-0.16292	-0.103895322	-0.059024513
128	A0A088AWF	PRUNE2	prune homolog 2 with BCH domain;prune hc	158471	ENSG00000106772	18	0.058	0.438	-0.29	-0.06	-0.2	-0.22	-0.3	-0.04	-0.13	0.4438	-0.261	0.1632	-0.039	-0.077	0.0147	0.1173	0.1737	-0.034	0.068	-0.52	-0.2	-0.36	-0.15	-0.14	0.264	0.036	0.307	0.532264	-0.00497	-0.138204598	0.133238801
3339	Q9GZX9	TWSG1	twisted gastrulation BMP signaling modulatc	57045	ENSG00000128791	5	-0.06	-0.55	-0.32	-0.28	-0.54	-0.33	0.001	-0.04	0.647	0.2567	0.1269	0.2445	-0.594	0.0331	-0.47	0.4449	-0.651	-0.197	0.019	0.219	0.15	-0.19	-0.11	-0.36	0.244	0.366	-0.76	0.865858	-0.11807	-0.074645247	-0.043428102
354	A1IGU5	ARHGEF3	Rho guanine nucleotide exchange factor 37	389337	ENSG00000183111	17	-0.16	0.076	-0.09	-0.44	-0.02	-0.45	0.421	0.099	0.077	-0.074	0.0837	0.0854	-0.22	-0.448	-0.169	0.0686	-0.19	0.2131	-0.46	-0.45	-0.21	-0.32	0.086	-0.08	0.206	-0.01	0.165	0.902805	0.06351	0.016667717	0.04684212
2078	P53634	CTSC	cathepsin C	1075	ENSG00000109861	20	-0.29	-0.77	0.116	-0.64	0.08	0.059	-0.65	0.034	-0.27	0.4703	0.8906	0.0591	-0.875	0.46	-0.412	-0.071	-0.359	-0.163	0.213	-0.3	0.48	-0.06	-0.26	-0.14	1.061	-0.24	-0.29	0.477121	-0.31075	-0.260510647	-0.050239983
3793	Q9Y6G9	DYNC1LI1	dynein cytoplasmic 1 light intermediate chaiı	51143	ENSG00000144635	16	0.262	0.091	-0.12	-0.23	0.27	-0.21	-0.1	-0.06	0.026	0.0665	0.063	0.067	0.1283	-0.042	0.2515	0.1201	0.2819	0.1009	-0.1	-0.55	-0.02	0.246	-0.09	0.066	0.059	0.115	0.066	0.367329	0.015302	-0.12296038	0.138262085
122	F1T0B3	DDX1	DEAD-box helicase 1	1653	ENSG00000079785	18	-0.07	-0.07	-0.09	-0.15	-0.04	-0.27	-0.2	-0.16	-0.25	0.0946	0.1044	-0.057	0.1096	-0.122	0.1727	-0.241	-0.45	-0.512	-0.02	-0.42	-0.15	-0.29	-0.21	0.149	0.066	-0.16	-0.09	0.915569	-0.02043	-0.045648788	0.025217417
1555	P11172	UMPS	uridine monophosphate synthetase	7372	ENSG00000114491	15	0.06	0.222	0.018	0.111	0.006	-0.43	0.026	-0.01	0.247	0.1967	0.1564	0.1502	0.0863	-0.126	0.0554	-0.012	0.2578	0.0523	-0.12	-0.8	-0.28	-0.49	-0.45	0.206	0.016	-0.04	0.185	0.156437	0.225223	-0.062714453	0.287937838
3042	Q92673	SORL1	sortilin related receptor 1	6653	ENSG00000137642	25	-0.35	-0.3	-0.12	-0.23	-0.17	-0.27	-0.25	0.263	-0	0.1331	0.2463	0.0503	-0.487	0.0827	-0.35	0.2757	-0.285	0.0216	-0	0.291	0.035	-0.22	-0.12	-0.17	0.445	0.144	-0.1	0.374094	-0.19307	-0.124748565	-0.068321445
464	F8W118	NAP1L1	nucleosome assembly protein 1 like 1	4673	ENSG00000187109	7	0.149	-0.09	0.086	0.16	0.418	-0.32	0.113	0.03	0.429	-0.095	-0.265	-0.456	0.1508	0.0787	0.3522	0.3376	0.2855	0.3145	0.128	-0.25	-0.24	-0.49	-0.15	-0.12	0.003	0.106	0.392	0.479551	0.177502	0.030593524	0.146908397
537	C9JXX4	IGFBP5	insulin like growth factor binding protein 5	3488	ENSG00000115461	4	0.524	-1.46	-0.24	0.579	-0.49	-0.11	0.043	0.237	0.007	-0.514	-0.295	0.5712	-0.803	0.5981	-0.903	1.2235	-0.121	0.3412	-0.42	-0.82	-0.59	-0.61	-0.57	-0.29	0.526	0.606	0.225	0.826714	0.114841	-0.111957797	0.226798914
3224	Q99733	NAP1L4	nucleosome assembly protein 1 like 4	4676	ENSG00000273562	11	0.131	0.288	0.041	-0.15	0.066	-0.38	0.02	-0.05	0.243	0.1981	-0.04	0.0109	-0.011	-0.095	0.0987	0.2206	-0.047	-0.007	-0.22	-0.95	-0.27	-0.63	-0.28	-0.09	0.178	0.036	0.065	0.163983	0.263503	-0.014017216	0.277519778
164	A0A0A0MTH	ILK	integrin linked kinase	3611	ENSG00000166333	16	0.009	0.123	-0.12	0.048	0.073	-0.34	-0.07	0.032	0.233	-0.04	-0.069	0.0758	0.0769	-0.14	0.047	0.1282	0.2207	0.1423	-0.16	-0.49	-0.07	-0.24	-0.04	0.241	-0.06	-0.08	0.105	0.374506	0.088508	-0.050258034	0.138766525

2347	Q08AM6	VAC14	Vac14, PIKFYVE complex component;VAC	55697	ENSG00000103043	16	0.082	0.152	0.031	-0.15	0.447	-0.43	-0.25	-0.13	-0.01	0.1714	0.1156	0.1774	0.0814	-0.261	-0.042	0.0868	0.1258	0.1064	-0.04	-0.63	-0.12	-0.23	-0.17	-0.06	0.049	0.012	0.021	0.315738	0.100219	-0.091059038	0.191277819
529	C9JXB8	RPL24	ribosomal protein L24	6152	ENSG00000114391	10	0.25	-0.22	-0.05	-0.42	0.908	-0.38	-0.31	1.169	-0.24	-0.434	-0.309	-0.357	-0.097	-0.314	-0.002	-0.223	-0.195	0.396	-0.07	-0.53	0.089	-0.01	0.059	0.119	0.204	0.225	0.108	0.526362	0.057682	0.249958667	-0.192276864
56	J3KRB3	RPL17	ribosomal protein L17	6139	ENSG00000265681	10	0.026	-0.14	-0.2	0.015	0.673	-0.23	0.126	0.024	0.22	-0.139	-0.141	-0.243	0.0422	-0.323	0.0255	0.136	0.2586	0.2642	-0.07	-0.39	0.058	-0.12	0.012	0.102	0.232	-0.18	0.03	0.762107	0.094064	0.070772316	0.023291895
2283	Q00688	FKBP3	FK506 binding protein 3;FKBP prolyl isomer	2287	ENSG00000100442	14	0.255	0.276	0.134	-0	-0.02	-0.35	0.332	0.165	0.409	0.1311	0.0439	0.0849	-0.11	-0.122	0.2789	0.2483	0.3038	0.0949	-0.09	-0.82	-0.25	-0.73	-0.41	-0.05	0.19	0.139	0.221	0.144423	0.333191	0.027927485	0.30526323
1321	O95994	AGR2	anterior gradient 2, protein disulphide isome	10551	ENSG00000106541	6	-0.24	-0.24	-0.31	-0.47	-0.22	-0.43	-0.14	0.125	-0.11	0.0343	-0.244	0.3096	0.7613	-0.495	-0.225	-0.372	0.3847	0.09	-0.53	0.241	-0	0.204	0.34	-0.06	0.634	-0.25	-0.31	0.343571	-0.25617	-0.254014133	-0.002156684
1110	O43291	SPINT2	serine peptidase inhibitor, Kunitz type 2	10653	ENSG00000167642	7	-0.42	0.441	0.075	-0.16	-0.29	-0.04	-0.1	0.011	-0.07	0.0425	0.3165	0.3025	-0.153	-0.153	-0.217	-0.07	0.0518	0.3778	-0.24	-0.14	-0.17	-0.18	-0.3	-0.15	-0.25	-0.02	0.278	0.353155	0.071384	-0.116272925	0.187657022
436	B4DRN8	ZDHHC20	zinc finger DHHC-type containing 20	253832	ENSG00000180776	9	-0.28	-0.25	-0.25	0.296	-0.28	0.157	-0.1	-0.19	-0.11	0.3952	0.3723	-0.123	-0.132	0.0545	0.2824	0.1714	0.1986	-0.092	0.109	-0.44	-0.18	-0.4	-0.24	0.117	-0.01	0.001	0.414	0.225322	-0.04114	-0.236856008	0.195720863
1412	P04114	APOB	apolipoprotein B	338	ENSG00000084674	4							0.771	0.588	1.078							0.7689	0.6073	0.4286							0.463	0.566	0.406	0.302066	0.334081	0.210844338	0.123237004
510	C9JE82	CACNA2D	calcium voltage-gated channel auxiliary subu	9254	ENSG00000007402	13	-0.17	-0.29	0.004	-0.23	0.847	-0.38	-0.25	0.446	0.141	0.1269	-0.418	-0.051	-0.209	-0.211	-0.528	0.0145	0.0289	0.2893	-0.18	-0.23	0.084	-0.37	-0.06	0.201	0.044	-0.06	-0.14	0.769513	0.091098	0.12020811	-0.029110263
3454	Q9NPC4	A4GALT	alpha 1,4-galactosyltransferase (P blood grc	53947	ENSG00000128274	13	0.03	-0.09	-0.23	-0.26	-0.21	-0.38	0.026	-0.01	0.187	0.1638	0.105	0.0654	-0.116	0.1605	-0.187	0.2695	-0.156	0.1315	0.284	0.525	0.213	-0	-0.03	-0.07	0.205	0.117	0.109	0.122098	-0.25248	-0.151091515	-0.101383908
3750	Q9Y3U8	RPL36	ribosomal protein L36	25873	ENSG00000130255	4	-0.06	0.173	-0.05	-0.1	0.299	-0.19	-0.36	-0.48	-0.31	0.0463	0.0368	-0.061	-0.337	0.1162	0.2083	-0.201	-0.021	-0.448	-0.01	-0.07	-0.08	-0.28	-0.03	-0.1	-0.1	-0.47	-0.45	0.72181	0.055927	-0.046567107	0.102494549
697	F5GWQ8	CLUL1	clusterin like 1	27098	ENSG00000079101	13	0.067	0.475	-0.21	-0.64	0.206	-0.67	-0.33	0.202	0.069	-0.057	0.0546	-0.192	-0.524	-0.155	0.0596	0.4722	0.7713	-0.029	-0.56	0.61	-0.24	-0.65	-0.74	-0.12	0.152	0.169	-0.12	0.660614	0.075492	-0.137122171	0.21261396
2975	Q8TCT8	SPPL2A	signal peptide peptidase like 2A	84888	ENSG00000138600	6	-0.04	0.596	0.185	0.162	0.042	-0.3	-0.05	0.006	0.282	0.1217	0.1035	0.1662	0.2798	-0.225	0.0288	0.3265	0.3589	0.43	-0.32	0.039	-0.36	-0.67	-0.2	0.003	-0.21	-0.71	0.366	0.097798	0.32831	-0.077406194	0.405716278
1911	P41240	CSK	C-terminal Src kinase	1445	ENSG00000103653	15	-0.06	0.105	-0.13	-0.04	-0.08	-0.34	-0.08	0.046	0.114	-0.055	0.0229	-0.013	-0.009	-0.155	0.032	0.114	0.1061	0.0094	-0.29	-0.78	-0.24	-0.28	-0.24	0.042	-0.02	0.085	0.133	0.2812	0.122205	-0.058737882	0.180942769
3642	Q9UKA8	RCAN3	RCAN family member 3	11123	ENSG00000117602	6	0.048	-0.07	-0.29	0.092	0.115	-0.2	-0.31	0.165	-0.11	-0.13	-0.041	0.0512	0.0246	-0.179	0.1452	-0.034	0.1025	-0.008	-0.2	-0.9	-0.34	-0.38	-0.21	-0.22	0.219	0.061	0.137	0.338676	0.143862	-0.054436564	0.198298466
2823	Q86X76	NIT1	nitrilase 1	4817	ENSG00000158793	11	0.117	0.131	-0.13	0.208	0.224	-0.41	-0.14	0.026	0.241	0.0608	-0.018	-0.144	0.0697	-0.166	0.1363	0.2369	0.3043	0.084	-0.31	-1.36	-0.36	-0.58	-0.37	0.041	0.072	0.249	0.291	0.241171	0.287524	-0.033123558	0.32064798
2442	Q14118	DAG1	dystroglycan 1	1605	ENSG00000173402	5	-0.29	-0.56	-0.32	-0.16	-0.22	-0.07	-0.01	-0.2	0.186	-0.111	0.1712	0.2137	-0.295	0.2929	0.0025	0.2605	-0.174	-0.082	-0.18	0.08	-0.05	-0.21	-0.17	-0.21	0.306	-0.05	-0.15	0.229849	-0.1125	-0.214157641	0.101662133
3609	Q9UHC6	CNTNAP2	contactin associated protein like 2	26047	ENSG00000174469	12	0.101	-0.26	-0.33	-0.01	-0.47	0.38	0.112	0.114	0.114	0.7439	-0.036	0.7634	-0.345	-0.558	-0.407	0.5516	-0.513	-0.063	-0.12	-0.48	-0.15	-0.42	-0.01	-0.38	-0.09	0.414	-0.44	0.638202	0.159349	-0.041839832	0.201188441
847	H3BRH9	SYT17	synaptotagmin 17	51760	ENSG00000103528	12	0.046	0.068	0.165	-0.01	-0.24	-0.2	0.159	-0.23	0.335	0.2449	0.3183	0.2876	-0.126	-0.044	-0.167	0.0602	0.3988	0.1622	-0.01	-0.82	-0.27	-0.74	-0.63	-0.14	-0.22	-0.12	0.323	0.097933	0.301106	-0.116450061	0.417555757
1254	O94766	B3GAT3	beta-1,3-glucuronyltransferase 3	26229	ENSG00000149541	8	-0.16	-0.36	-0.16	-0.41	-0.06	-0.15	0.075	0.21	0.209	-7E-04	-0.019	-0.195	-0.412	0.1612	-0.012	0.3312	0.1774	0.2405	0.059	0.329	0.192	-0.32	-0.33	-0.21	0.433	0.338	0.134	0.53586	-0.15896	-0.119899844	-0.039063975
2392	Q13232	NME3	NME/NM23 nucleoside diphosphate kinase	4832	ENSG00000103024	12	-0.17	-0.37	-0.11	-0.4	-0.02	-0.28	-0.54	-0.03	-0.35	0.3867	0.4106	0.0544	-0.184	-0.119	0.0359	0.0984	-0.044	-0.034	-0.18	0.369	0.046	-0.39	-0.15	-0.08	0.635	0.064	-0.37	0.146321	-0.24498	-0.318582272	0.073601324
42	Q99417	MYCBP	MYC binding protein	26292	ENSG00000214114	4	0.398	-0.89	-0.68	-0.23	-0.18	0.046	-0.2	-0.12	-0.01	0.3037	-0.108	0.0007	-0.095	0.9711	0.0225	-0.131	0.1016	-0.329	0.795	0.488	0.293	-0.01	0.173	-0.03	0.094	-0.17	-0.05	0.215757	-0.382	-0.288322889	-0.093675357
1065	O15031	PLXNB2	plexin B2	23654	ENSG00000196576	21	0.22	0.229	0.2	-0.77	-0.49	-0.68	-0.2	-0.06	-0.05	0.1411	0.1841	0.0559	-0.643	-0.268	-0.674	-0.106	0.1994	0.1055	-0.07	-0.45	-0.12	-0.9	-0.63	-0.55	0.111	0.131	0.026	0.756593	0.094967	-0.065564518	0.160531143
455	B5MD23	TSPAN9	tetraspanin 9	10867	ENSG00000011105	5	-0.32	0.362	0.338	-1.16	0.01	0.732	0.121	0.432	0.074	0.1385	0.3533	-0.411	-0.268	-0.064	-0.583	0.2113	0.2486	0.4331	-0.13	1.244	-0.24	0.22	-0.11	-0.27	0.133	-0.08	0.293	0.918715	-0.05394	0.058504483	-0.112446551
3356	Q9H1C7	CYSTM1	cysteine rich transmembrane module contai	84418	ENSG00000120306	2	-0.11	0.183	-0.19	-0.67	-0.57	-0.05	0.061	0.096	-0.05	0.1461	0.1701	-0.117	-0.865	-0.125	-0.337	0.2367	0.125	0.2032	0.006	0.261	-0.02	-0.34	0.21	-0.72	0.204	-0.15	-0	0.883965	-0.08199	-0.081964344	-2.10686E-05
1160	O60506	SYNCRIP	synaptotagmin binding cytoplasmic RNA int	10492	ENSG00000135316	12	0.305	0.217	-0.08	-0.14	0.224	-0.23	0.063	-0.01	0.302	0.0399	0.0129	0.1402	-0.005	-0.226	0.0967	-0.026	0.1897	0.1422	0.015	-0.6	-0.06	-0.28	-0.12	0.055	0.16	0.059	0.249	0.517357	0.131265	0.032801191	0.098463369
1221	O75663	TIPRL	TOR signaling pathway regulator	261726	ENSG00000143155	8	0.154	0.411	0.032	0.136	-0.08	-0.47	-0.23	-0.33	0.139	0.2868	0.2468	0.161	0.1904	-0.098	0.2229	-0.133	0.1471	0.0017	-0.17	-0.94	-0.21	-0.62	-0.35	0.025	-0.21	-0.1	-0.02	0.097539	0.261444	-0.139748467	0.401192869
1054	O14896	IRF6	interferon regulatory factor 6	3664	ENSG00000117595	12	-0.09	0.315	0.027	0.076	-0.05	-0.61	0.181	-0.25	0.086	-0.064	0.0456	-0.126	-0.085	-0.113	######	-0.04	0.3072	0.2707	-0.38	-1.05	-0.48	-0.75	-0.33	0.174	0.082	0.19	0.234	0.324451	0.222247	-0.056323893	0.278571208
71	A0A087WY7	AP2M1	adaptor related protein complex 2 subunit m	1173	ENSG00000161203	15	-0.06	-0.08	-0.31	0.005	-0.07	-0.4	0.1	0.062	0.215	-0.027	-0.29	-0.037	-0.185	-0.125	-0.034	0.2092	0.2337	0.2765	-0.15	-0.01	-0.2	-0.42	-0.37	0.039	0.19	0.047	0.211	0.799334	0.01537	-0.061095263	0.076464942
426	B4E171	AF241726.	novel proline rich Gla (G-carboxyglutamic ac	NA	ENSG00000250349	4	-0.64	-0.23	0.31	-0.5	0.146	-0.32	-0.96	-0.14	-0.78	0.2378	0.2334	-0.418	-0.122	-0.484	-0.673	-0.589	0.3384	-0.112	0.082	1.245	0.219	0.016	0.174	0.518	0.55	0.072	-0.14	0.084989	-0.65056	-0.17000429	-0.480553992
3354	Q9H190	SDCBP2	syndecan binding protein 2	27111	ENSG00000125775	8	-0.73	-0.33	0.478	0.39	0.15	0.068	-0.09	0.32	-0.16	-0.048	-0.017	-0.166	-0.044	-0.17	-0.495	-0.002	-0.038	0.153	-0.74	0.962	-0.4	0.031	-0.38	-0.27	0.035	-0.28	0.097	0.846591	0.115106	0.102558845	0.012546988
2081	P53985	SLC16A1	solute carrier family 16 member 1	6566	ENSG00000155380	6	-0.16	-0.66	0.079	0.394	0.541	-0.11	0.144	0.488	-0.08	-0.061	-0.232	-0.183	0.1316	-0.206	0.0095	0.2697	0.21	0.456	-0.3	-0.15	-0.45	-0.99	-0.35	-0.31	0.281	0.25	0.02	0.309401	0.292576	0.027265717	0.26531038
1352	P01112	HRAS	HRas proto-oncogene, GTPase	3265	ENSG00000276536	11	-0.09	0.344	0.058	0.125	0.124	-0.35	-0.14	0.112	-0.17	0.4652	0.5424	2.2757	0.2617	-0.267	0.1236	0.1992	0.1067	0.1065	-0.01	1.418	-0.41	-0.68	-0.58	-0.09	-0.13	0.022	0.191	0.340498	0.031951	-0.421489293	0.453440337
1775	X5D2R7	PSMB8	proteasome subunit beta 8	5696	ENSG00000204264	11	-0.22	-0.29	0.084	-0.37	-0.26	-0.51	0.06	-0.46	-0.11	-0.057	-0.199	0.1782	-0.019	-0.033	-0.232	0.4696	0.1486	0.2104	-0.29	-0.22	0.036	0.454	-0	-0.04	0.476	0.028	-0.03	0.133929	-0.27514	-0.282691318	0.007552642
3509	Q9NTJ4	MAN2C1	mannosidase alpha class 2C member 1	4123	ENSG00000140400	15	0.171	0.117	-0.01	0.744	0.65	-0.37	0.345	0.631	0.581	-0.106	-0.153	-0.139	-0.081	-0.249	-0.121	0.3609	-0.281	-0.011	-0.27	-0.42	-0.31	-0.31	-0.22	-0.29	0.343	0.234	-0.07	0.060331	0.465046	0.405109579	0.059935956
2165	P61353	RPL27	ribosomal protein L27	6155	ENSG00000131469	6	-0.65	-1.01	-0.95	-0.92	-0.1	-0.57	0.147	-0.31	-0.01	-1.074	-0.69	-1.197	-0.943	-0.604	-0.195	-0.349	0.2813	-0.124	-0.87	-1.01	-0.44	-0.53	-0.65	-0.67	0.236	-0.41	-0.26	0.972592	0.027027	0.058735298	-0.031707986
2496	Q15075	EEA1	early endosome antigen 1	8411	ENSG00000102189	24	0.097	0.002	0.168	-0.07	-0.03	-0.21	-0.08	-0.37	-0.02	0.0226	-0.087	-0.017	0.3129	-0.145	0.199	-0.349	-0.239	-0.242	0.523	-0.45	0.072	-0.37	-0	0.125	0.153	-0.22	-0.42	0.997135	0.010029	0.005064142	0.004964917
2682	Q6GMV3	PTRHD1	peptidyl-tRNA hydrolase domain containing	391356	ENSG00000184924	8	0.243	0.484	0.052	-0.32	-0.13	-0.46	-0.04	0.142	0.56	0.3108	0.3625	0.2843	-0.111	0.0753	0.1848	0.137	0.5265	0.2986	-0.22	-1	-0.27	-0.85	-0.42	-0.01	-0.24	0.022	0.387	0.098335	0.348799	-0.170246858	0.519045928
1185	O60925	PFDN1	prefoldin subunit 1	5201	ENSG00000113068	7	-0.01	0.094	-0.05	-0.16	0.191	-0.52	0.011	0.06	0.131	-0.018	-0.301	-0.08	-0.063	-0.194	0.1309	0.2253	0.0619	-0.076	-0.08	-0.74	-0.2	-0.23	-0.05	0.075	-0.14	0.086	-0.05	0.55913	0.119258	0.006670607	0.112587043
1424	P04808	RLN2;RLN	relaxin 2;relaxin 1	6013	ENSG00000107014	3	0.643	-0.02	-0.78	-1.12	0.177	-0.72	-0.28	-1.39	-0.65	1.1409	-0.372	0.0705	0.9774	0.154	-0.642	-0.222	0.6954	-1.656	-0.4	0.58	-0.23	-0.72	-0.84	-0.55	-0.23	-1.72	-0.9	0.375069	0.095183	-0.477042678	0.572226015
1092	O15371	EIF3D	eukaryotic translation initiation factor 3 subu	8664	ENSG00000100353	14	0.101	-0	-0.18	-0.04	0.073	-0.43	-0.07	-0.09	0.123	-0.117	-0.091	0.0178	0.0406	-0.15	-0.039	0.1223	0.0834	0.0727	-0.22	-0.49	-0.08	-0.58	-0.22	0.036	0.231	0.021	0.152	0.571398	0.071931	-0.050068153	0.121998963
615	E7EVC7	ATG16L1	autophagy related 16 like 1	55054	ENSG00000281089	11	0.308	0.268	0.41	-0.06	-0.08	-0.56	0.231	0.035	0.222	0.1549	0.097	0.3403	0.0627	-0.156	0.1083	0.0441	0.42	0.2221	0.038	-0.43	-0.25	-0.59	-0.35	0.116	0.025	0.051	0.298	0.230689	0.20835	-0.056788708	0.265138758
279	A0A1B0GW	STXBP1	syntaxin binding protein 1	6812	ENSG00000136854	21	0.047	0.389	-0.75	0.345	-0.08	-0.49	-0.31	-0.19	-0.13	-0.184	0.0996	-0.039	-0.04	-0.139	0.6754	0.6157	-0.202	0.1576	-0.34	-0.14	-0.36	-0.88	-0.45	-0.38	-0.12	0.202	0.51	0.330236	0.086947	-0.235406227	0.322352867
486	D6RBD7	EEF1E1	eukaryotic translation elongation factor 1 eps	9521	ENSG00000124802	7	0.097	0.087	0.004	0.22	0.359	-0.29	0.347	-0.05	0.319	0.1087	0.3143	0.0463	0.0945	-0.119	-0.097	-0.052	0.1113	0.2285	-0.18	-0.48	0.071	-0.07	-0.21	0.025	0.252	-0.11	0.186	0.316665	0.177622	0.050803058	0.126819068
1516	P09471	GNAO1	G protein subunit alpha o1	2775	ENSG00000087258	12	-0.24	0.249	-0.1	-0.29	0.098	0.187	0.33	-0.01	0.179	-0.189	-0.149	-0.547	-0.085	-0.049	0.3574	-0.043	0.3018	0.2166	-0.28	1.151	-0.39	-0.37	-0.29	-0.25	0.207	-0.1	0.206	0.940207	0.056462	0.065863431	-0.009401247
218	H9KV28	DIAPH1	diaphanous related formin 1	1729	ENSG00000131504	23	-0.09	0.226	0.055	-0.15	0.059	-0.48	-0.26	-0.2	0.15	0.1487	-0.037	0.0701	0.1347	-0.052	-0.16	-0.274	0.214	-0.015	-0.12	-0.29	0.062	-0.07	-0.13	0.113	0.022	-0.17	-0.28	0.605484	0.019675	-0.079081596	0.098756301
1666	P18440	NAT1	N-acetyltransferase 1	9	ENSG00000171428	14	-0.04	0.41	-0.06	-0.1	-0.05	-0.56	0.138	-0.02	0.458	0.1055	-0.035	-0.063	0.2853	-0.33	-0.345	0.3037	0.1855	0.24	0.035	-0.78	-0.39	-0.39	-0.26	0.274	-0.08	0.12	0.401	0.638244	0.138996	-0.019125401	0.158121739
3418	Q9H9A6	LRRC40	leucine rich repeat containing 40	55631	ENSG00000066557	15	0.225	0.169	0.213	0.054	0.101	-0.31	-0.01	-0.1	0.151	0.187	0.1618	0.0343	0.0179	-1E-04	0.2114	0.0696	0.1844	0.0654	-0.24	-0.47	-0.03	-0.47	-0.15	0.09	0.037	0.012	0.199	0.157084	0.167808	-0.048625424	0.21643373
2737	Q6VEQ5	WASH2P	WASP family homolog 2, pseudogene	375260	ENSG00000146556	13	-0.1	-0.07	0.244	3E-04	-0.2	-0.56	0.082	0.159	0.206	-0.016	0.1634	-0.039	-0.049	-0.008	0.1214	0.1937	0.4861	0.3835	-0.23	-0.6	0.219	-0.34	-0.5	0.042	-0.1	-0.04	0.133	0.182657	0.129708	-0.165341941	0.295049871
1056	O14908	GIPC1	GIPC PDZ domain containing family membe	10755	ENSG00000123159	9	0.047	0.37	-0.21	0.194	-0.11	-0.36	-0.18	-0.11	0.005	-0.128	-0.068	0.1654	-0.075	-0.128	0.3744	0.016	0.1628	0.1278	-0.2	-0.34	0.052	-0.39	-0.32	-0.18	-0.05	0.143	0.259	0.430785	0.073215	-0.089469842	0.16268521
2545	Q15904	ATP6AP1	ATPase H+ transporting accessory protein	537	ENSG00000071553	7	-0.12	-0.03	0.05	-0.07	-0.18	-0.29	-0.27	0.038	-0.02	0.2173	0.0568	0.0163	0.1137	-0.026	-0.029	0.0705	0.2543	-0.014	-0.02	-0.36	0.024	-0.04	-0.28	0.293	0.072	0.119	0.087	0.202998	-0.0883	-0.172025598	0.083721946
3049	Q92791	P3H4	prolyl 3-hydroxylase family member 4 (non-e	10609	ENSG00000141696	12	0.03	-0.43	-0.26	-0.47	-0.13	-0.15	-0.18	0.095	-0.12	0.5111	-0.071	-0.254	-0.354	0.2199	-0.003	0.0126	-0.347	-0.264	0.374	0.981	0.107	-0.28	0.267	-0.2	0.429	-0.37	-0.23	0.311507	-0.30037	-0.119627479	-0.180742752
1943	P46108	CRK	CRK proto-oncogene, adaptor protein	1398	ENSG00000167193	19	0.05	0.078	-0.11	-0.27	-0.17	-0.07	-0.07	0.019	0.12	-0.027	-0.062	0.0967	-0.282	0.053	0.1738	0.1099	0.2677	0.0993	-0.13	-0.4	-0.13	-0.3	0.16	-0.35	-0.16	0.11	0.123	0.277838	0.071043	-0.095899548	0.166942239
2015	P50148	GNAQ	G protein subunit alpha q	2776	ENSG00000156052	19	0.221	0.579	-0.19	0.115	0.102	-0.32	0.143	0.08	0.366	-0.141	0.0209	0.3974	0.0999	-0.286	0.1992	0.4984	0.2446	0.4687	-0.18	-0.1	-0.45	-0.48	-0.43	-0.24	0.038	0.302	0.418	0.229883	0.244848	-0.045304084	0.290152505
2855	Q8IX04	UEVLD	UEV and lactate/malate dehyrogenase dom	55293	ENSG00000151116	10	-0.14	0.177	-0.14	-0.02	0.035	-0.18	-0.23	-0.33	-0.19	0.0168	-0.168	0.0188	0.3639	-0.057	0.0864	-0.297	-0.23	-0.269	-0.06	8E-04	-0.02	0.147	-0.05	-0	-0.09	-0.13	-0.27	0.786662	-0.06073	-0.053654892	-0.007076268
1570	P12110	COL6A2	collagen type VI alpha 2 chain	1292	ENSG00000142173	18	0.195	-1.01	-0.07	-0.87	0.075	0.061	-0.93	0.871	0.851	0.0187	-0.115	-0.557	-0.793	0.0808	-0.305	0.3992	-0.483	0.1984	-0.29	0.119	0.852	-1.34	-1.03	0.467	0.262	-0.1	0.593	0.944288	-0.04137	0.080234425	-0.121600566
2220	P62917	RPL8	ribosomal protein L8	6132	ENSG00000161016	9	0.107	0.103	-0.07	-0.34	0.684	-0.02	0.372	0.081	0.312	-0.355	-0.059	-0.169	-0.231	-0.065	-0.122	-0.168	0.3899	0.1826	-0.11	-0.52	-0.06	-0.56	-0.11	-0.24	0.117	-0.1	0.189	0.203112	0.290794	0.203029909	0.087763963
2152	P61086	UBE2K	ubiquitin conjugating enzyme E2 K	3093	ENSG00000078140	13	0.337	0.253	0.233	-0.06	0.072	-0.3	0.045	0.101	0.268	0.1738	0.2319	0.2428	-0.05	-0.165	0.094	0.0984	0.3046	0.1409	-0.15	-1.02	-0.06	-0.6	-0.26	0.062	-0.23	-0.01	0.218	0.114959	0.332135	-0.013853166	0.345988516
643	E9PFR3	PPP2R5D	protein phosphatase 2 regulatory subunit B'(	5528	ENSG00000112640	20	0.195	0.012	-0.13	0.361	-0.1	-0.43	-0.21	-0.07	0.027	0.0571	-0.023	0.0511	0.185	-0.091	0.0015	-0.004	0.0163	-0.048	-0.3	-0.5	-0.26	-0.15	-0.31	0.115	0.233	-0.05	0.091	0.48111	0.08575	-0.055427926	0.141177908
2648	Q5TDH0	DDI2	DNA damage inducible 1 homolog 2	84301	ENSG00000197312	16	0.088	0.22	-0.08	-0.13	0.044	-0.34	0.078	0.097	0.234	-0.031	0.0387	0.1249	-6E-04	-0.059	0.306	0.2566	0.2363	0.258	-0.04	-0.81	-0.23	-0.5	-0.15	0.024	0.007	0.168	0.422	0.26987	0.145856	-0.101881374	0.247737804
1402	P03950	ANG	angiogenin	283	ENSG00000214274	5	#####	0.17	-0.37	0.206	-0.32	-0.02	0.431	0.046	-0.02	0.1558	-0.068	0.2935	-0.514	-0.078	-0.214	0.2383	-0.044	-0.263	-0.35	-0.04	0.158	0.058	-0.25	-0.33	0.467	0.043	-0.4	0.845787	0.086042	0.068883779	0.017158233
1335	P00747	PLG	plasminogen	5340	ENSG00000122194	19	-0.65	-0.31	-0.4	-0.53	-0.21	0.184	-0.13	-0.05	-0.24	0.0934	-0.029	-0.19	-0.39	0.2518	-0.397	0.5462	-0.038	0.1595	-0.42	-0.05	0.681	1.153	0.049	0.148	0.44	0.04	-0.48	0.201531	-0.43396	-0.260435438	-0.173525519
1547	X6R5C5	CTSA	cathepsin A	5476	ENSG00000064601	10	0.007	-0.65	0.322	-0.04	-0.04	-0.16	-0.08	0.268	-0.02	0.2229	0.1745	-0.08	-0.373	0.2798	-0.179	-0.06	-0.329	-0.454	0.229	0.821	0.284	0.205	-0.17	-0.22	0.694	0.211	-0.4	0.338586	-0.22602	0.045459176	-0.271475524
2441	Q14117	DPYS	dihydropyrimidinase	1807	ENSG00000147647	9	0.148	-0.31	-0.2	-0.18	0.21	-0.14	-0.32	0.015	-0.03	-0.229	0.1111	0.025	-0.196	-0.52	0.0105	0.2979	-0.039	-0.245	-0.24	-0.64	0.107	0.063	-0	-0.26	0.281	0.017	0.324	0.919878	-0.05173	-0.002422539	-0.04931201
1496	P08240	SRPRA	SRP receptor subunit alpha	6734	ENSG00000182934	14	0.006	-0.31	-0.09	-0.49	0.004	-0.01	-0.15	-0.03	-0.01	0.1208	0.1291	-0.019	-0.416	0.3295	-0.129	0.217	-0.193	0.2122	-0.05	0.33	-0.09	0.031	0.198	-0.08	0.381	0.011	-0.08	0.270525	-0.19206	-0.147479365	-0.044577977
3111	Q96DA2	RAB39B	RAB39B, member RAS oncogene family	116442	ENSG00000155961	9	-0.01	0.319	0.113	-0.06	-0.1	-0.63	-0.5	0.15	0.293	-0.056	0.0211	-0.105	0.1121	-0.442	-0.028	0.5878	0.3206	-0.147	-0.14	-0.64	-0.32	-0.51	-0.66	-0.1	0.015	-0.23	-0.08	0.203128	0.247458	-0.077087235	0.324545226
3433	Q9HB63	NTN4	netrin 4	59277	ENSG00000074527	12	-0.55	-0.6	-0.55	-0.19	-0.5	-0.59	0.204	0.382	-0.03	-0.2	-0.011	0.0831	-0.407	0.4403	0.1719	0.2652	-0.604	0.2201	-0.61	0.085	-0.24	0.131	-0.4	-0.15	0.697	-0.27	-0.06	0.464718	-0.17915	-0.264540273	0.08538768
417	B1AMW1	CD58	CD58 molecule	965	ENSG00000116815	4	-0.11	0.126	-0.23	0.014	0.235	-0.51	-0.04	-0.11	0.408	-0.15	0.1145	0.539	-0.3	-0.155	-0.128	-0.005	0.1918	0.3934	-0.58	-0.65	-0.2	-0.47	-0.32	-0.03	-0.41	-0.01	0.471	0.254179	0.219971	-0.079706709	0.299677287
1691	P20339	RAB5A	RAB5A, member RAS oncogene family	5868	ENSG00000144566	9	0.035	-0.04	-0.25	-0.11	-0.25	-0.56	-0.08	0.028	0.2	0.1845	0.123	-0.019	0.1193	-0.214	0.035	0.3013	0.0139	-0.044	-0.19	-0.42	-0.27	-0.74	-0.41	0.058	0.33	-0.17	-0.04	0.210896	0.090907	-0.169916188	0.260822839
3137	Q96GY0	ZC2HC1A	zinc finger C2HC-type containing 1A	51101	ENSG00000104427	9	-0.43	-0.85	-0.94	0.275	-0.08	-0.14	0.075	0.265	0.16	0.8991	-0.516	-0.265	-0.275	-0.097	0.6311	0.4302	-0.13	0.0515	1.222	1.088	-0.09	-0.87	-0.64	-0.29	0.303	0.118	0.136	0.613832	-0.29297	-0.265088299	-0.027877446
3406	Q9H7C9	AAMDC	adipogenesis associated Mth938 domain co	28971	ENSG00000087884	7	-0.04	0.213	-0.57	-0.42	0.162	-0.56	0.341	-0.73	-0	-0.529	0.004	-0.485	0.0598	-0.017	0.1601	0.1217	0.0336	-0.512	-0.48	-0.45	-0.26	-0.92	-0.22	-0.03	0.197	0.031	-0.35	0.758408	0.098137	-0.048435563	0.146572829
3416	Q9H910	JPT2	Jupiter microtubule associated homolog 2	90861	ENSG00000206053	11	-0.17	0.082	-0.16	-0.11	-0.1	-0.61	0.156	0.108	0.327	0.0095	0.2481	0.0857	-0.049	-0.293	0.2714	0.3804	0.139	-0.119	0.031	-0	-0.04	-0.76	-0.62	-0.06	0.248	0.197	0.014	0.52486	0.056716	-0.128450815	0.185166781
2885	Q8N0X7	SPART	spartin	23111	ENSG00000133104	16	0.081	0.102	-0.13	-0.21	0.06	-0.4	0.012	-0.04	0.256	0.0359	0.0766	0.0856	0.0691	-0.184	-0.123	0.0215	0.2306	0.076	-0.11	-0.71	-0.15	-0.29	-0.39	-0.16	0.155	0.072	0.269	0.385044	0.117198	-0.061187147	0.178384713
3423	Q9HA65	TBC1D17	TBC1 domain family member 17	79735	ENSG00000104946	11	0.056	0.194	-0.08	-0.1	0.054	-0.3	-0	-0.06	0.266	0.1614	0.1119	0.0616	0.0699	-0.122	0.0833	-0.063	0.2098	0.1312	-0.28	-0.79	-0.2	-0.33	-0.26	0.098	0.079	0.038	0.111	0.176665	0.173688	-0.068753945	0.242441819
904	J3KN16	ECPAS	Ecm29 proteasome adaptor and scaffold	23392	ENSG00000136813	32	-0.07	0.03	-0.17	-0.09	-0.01	-0.22	-0.22	-0.22	0.047	0.1769	-0.057	0.0366	-0.116	-0.081	-0.043	-0.047	0.0132	0.0211	-0.27	-0.45	-0.06	-0.24	-0.08	0.102	0.004	0.018	0.234	0.52695	-0.02124	-0.092980823	0.071738049
1662	P18077	RPL35A	ribosomal protein L35a	6165	ENSG00000182899	9	0.202	-0.01	-0.16	-0.12	0.296	-0.27	-0.39	-0.44	-0.42	-0.069	-0.215	-0.142	-0.352	-0.075	-0.085	-0.514	-0.015	-0.395	-0.26	-0.46	0.054	-0.11	-0.07	0.067	-0.09	-0.36	-0.52	0.883798	0.048778	0.061931614	-0.013153408
1331	P00492	HPRT1	hypoxanthine phosphoribosyltransferase 1	3251	ENSG00000165704	10	-0.34	-0.18	-0.15	0.089	0.388	0.083	0.162	0.394	0.116	-0.435	-0.212	-0.697	-0.305	-0.124	-0.242	0.0047	-0.074	-0.244	-0.2	0.165	-0.1	0.402	0.171	-0.01	-0.06	0.048	-0.03	0.081859	0.021148	0.321819165	-0.300671404
3453	Q9NPA8	ENY2	ENY2 transcription and export complex 2 su	56943	ENSG00000120533	4	-0.05	0.039	-0.2				0.119	0.022	0.31	0.1426	-0.071	0.0605				0.0812	0.4245	0.1746	-0.13	-0.66	0.096				0.045	0.249	0.223	0.631952	0.069753	-0.09532501	0.165077749
3109	Q96CN7	ISOC1	isochorismatase domain containing 1	51015	ENSG00000066583	8	0.003	-0	-0.26	0.111	0.051	-0.41	0.262	0.004	0.424	-0.087	-0.187	0.0096	-0.065	-0.096	0.0181	-0.039	-0.133	-0.165	-0.27	-0.6	0.027	-0.63	-0.47	-0.07	0.026	0.004	0.034	0.230556	0.237384	0.102726373	0.134657967

1229	O75843	AP1G2	adaptor related protein complex 1 subunit ga	8906	ENSG00000213983	9	0.032	-0.04	-0.12	0.271	0.026	-0.09	-0.05	-0.12	0.056	0.1037	-0.157	0.0529	-0.004	-0.019	-0.011	-0.192	-0.021	0.0763	-0.1	-0.43	-0.03	-0.14	-0.21	0.116	-0.1	-0.03	0.101	0.487717	0.08813	0.015804027	0.072326207
795	O00429	DNM1L	dynamin 1 like	10059	ENSG00000087470	15	-0.05	-0.02	-0.03	-0.07	0.327	-0.28	0.048	-0.14	-0.02	0.0198	-0.002	0.0746	0.1076	-0.215	-0.018	-0.035	-0.148	0.0234	0.022	-0.02	-0.25	0.185	-0.15	0.086	-0.06	-0.18	0.141	0.997289	-0.00271	-0.005496556	0.002786701
2366	Q12904	AIMP1	aminoacyl tRNA synthetase complex interac	9255	ENSG00000164022	12	0.024	0.13	0.094	0.279	0.2	-0.13	-0.04	-0.09	0.225	-0.149	-0.132	-0.183	0.0154	0.0635	0.3047	0.006	0.2097	0.0807	-0.32	-0.48	-0.06	-0.24	-0.02	0.09	0.444	-0.15	0.063	0.440522	0.151498	0.053275008	0.098222595
2590	Q53EL6	PDCD4	programmed cell death 4	27250	ENSG00000150593	14	0.31	0.048	0.011	0.028	-0.01	-0.41	-0.1	-0.15	0.159	0.1346	0.1611	0.1442	0.1174	0.174	0.1561	-0.094	0.1052	0.3224	0.032	-0.96	-0.17	-0.39	-0.07	0.085	0.221	-0.05	0.352	0.311468	0.091686	-0.149122179	0.24080829
2543	S4R3E9	NEDD8-MI	NEDD8-MDP1 readthrough	100528064	ENSG00000255526	5							0.709	0.337	0.744							0.6704	0.6124	0.7061							0.069	0.272	0.745	0.495068	0.234607	-0.066233506	0.300840236
3587	Q9UBS3	DNAJB9	DnaJ heat shock protein family (Hsp40) mei	4189	ENSG00000128590	13	-0.03	-0.63	-0.3	-0.43	-0.61	0.189	-0.07	0.143	0.052	0.1695	0.0252	-0.195	-0.674	0.2126	0.2624	-0.15	-0.744	0.0338	0.519	1.226	0.429	-0.26	-0.02	-0.68	-0.02	-0.09	0.065	0.415616	-0.3182	-0.068902227	-0.249302543
3159	Q96KN1	LRATD2;F	LRAT domain containing 2;family with sequ	157638	ENSG00000168672	12	-0.03	0.117	0.029	0.005	-0.17	-0.37	-0.1	0.075	0.124	0.0575	-0.097	0.0154	0.1195	-0.145	0.0593	0.071	0.0303	0.1104	-0.35	-0.63	-0.16	-0.4	-0.33	0.033	-0.01	-0.02	0.171	0.183619	0.151759	-0.060681457	0.212440353
3391	Q9H4A6	GOLPH3	golgi phosphoprotein 3	64083	ENSG00000113384	16	0.261	0.438	0.15	-0.07	-0.07	-0.25	-0.19	-0.14	0.026	0.1082	0.2135	0.2447	0.1978	-0.154	0.1936	0.1489	-0.001	0.0871	-0.24	-0.51	-0.29	-0.28	-0.19	0.054	-0.11	-0.07	0.087	0.098475	0.189426	-0.099424397	0.288850167
353	A1A5D9	BICDL2	BICD family like cargo adaptor 2	146439	ENSG00000162069	17	0.251	-0.06	-0.12	-0.04	-0.18	-0.11	0.082	-0.08	0.017	-0.071	-0.029	-0.053	-0.053	0.0833	0.1454	0.1325	-0.048	-0.057	-0.07	0.137	0.001	-0.49	-0.17	-0.14	0.226	-0.03	0.023	0.774016	0.031801	-0.031037435	0.062838588
167	A0A0A0MTN	CUL2	cullin 2	8453	ENSG00000108094	11	0.006	-0.16	-0.06	0.07	0.107	-0.14	-0.17	-0.15	0.212	0.0521	0.1113	-0.078	-0.192	-0.142	-0.127	-0.195	0.0359	-0.349	-0.29	-0.19	-0.01	-0.27	-0.19	-0.07	-0.36	-0.26	-0.38	0.123154	0.193298	0.06693388	0.126364539
2703	Q6P4A8	PLBD1	phospholipase B domain containing 1	79887	ENSG00000121316	8	-0.43	-0.33	-0.17	-0.64	-0.01	0.016	-0.27	-0.03	-0.01	0.4494	0.169	0.1531	-0.237	0.6434	-0.465	0.186	-0.757	-0.12	0.307	0.294	0.361	0.301	0.179	0.005	0.545	0.455	-0.44	0.155109	-0.43106	-0.210180313	-0.220881968
2062	P52758	RIDA	reactive intermediate imine deaminase A ho	10247	ENSG00000132541	3	1.019	-0.42	0.538				0.35	-0.4	0.325	0.0094	-0.437	0.2138				0.1276	-0.044	0.0632	0.117	-0.73	-0.12				0.11	-0.3	0.163	0.463617	0.362816	0.246762852	0.116053537
3714	Q9Y2F5	ICE1	interactor of little elongation complex ELL su	23379	ENSG00000164151	1	-0.73	-1.53	-1.21	0.622	-1.16	-1.38	0.392	0.839	0.704	-1.168	-1.149	0.6136	-0.046	0.2157	-0.19	2.3718	-1.16	0.9286	-1.61	-1.33	-0.97	-1.45	-1.19	-0.42	0.415	1.457	-0.75	0.535271	0.266904	-0.431063046	0.697967545
1031	O00754	MAN2B1	mannosidase alpha class 2B member 1	4125	ENSG00000104774	18	-0.35	-0.33	0.079	-0.45	-0.03	-0.19	-0.1	-0.05	0.208	0.2682	-0.197	0.2972	-0.162	-0.288	-0.589	0.1911	-0.147	-0.132	-0.28	0.143	-0.26	0.109	0.069	-0.23	0.488	0.324	-0.6	0.809954	-0.10844	-0.050199138	-0.058240208
3620	Q9UHV9	PFDN2	prefoldin subunit 2	5202	ENSG00000143256	8	0.1	0.194	0.008	-0.12	0.072	-0.19	0.027	0.09	0.287	0.0363	-0.066	0.0849	-0.13	0.026	0.1646	0.2408	0.0975	0.135	-0.04	-0.95	-0.32	-0.6	-0.13	0.017	0.051	0.107	0.125	0.17378	0.246392	-0.013402441	0.259794844
38	E7ETK0	RPS24	ribosomal protein S24	6229	ENSG00000138326	5	0.148	-0.15	-0.19	-0.24	0.193	-0.49	0.246	-0.03	0.114	-0.092	0.0346	-0.1	-0.352	-0.276	-0.005	-0.241	0.1163	-0.023	-0.07	-0.65	0.117	-0.43	-0.22	0.009	0.201	-0.1	0.026	0.824886	0.080006	0.059555661	0.020449964
2209	P62834	RAP1A	RAP1A, member of RAS oncogene family	5906	ENSG00000116473	19	0.087	0.359	0.141	0.168	-0.03	-0.16	-0.09	-0.05	-0.05	0.3931	0.2351	-0.02	0.2654	-0.236	0.2627	0.2305	0.3888	0.0001	-0.21	-0.32	-0.24	-0.5	-0.31	0.15	-0.48	-0.07	0.179	0.059336	0.24073	-0.127591299	0.368321495
1119	O43505	B4GAT1	beta-1,4-glucuronyltransferase 1	11041	ENSG00000174684	5	-0.09	-0.47	-0.39	-0.2	0.127	-0.14	0.089	0.189	0.319	0.1969	-0.147	0.2789	0.1206	-0.308	-0.204	0.4493	-0.054	0.1789	-0.16	0.05	-0.11	0.13	-0.2	-0.15	0.36	0.563	-0.09	0.684352	-0.10768	-0.120017252	0.012334682
1606	P14543	NID1	nidogen 1	4811	ENSG00000116962	18	-0.04	-0.15	0.183	0.345	0.155	-0.35	-0.35	0.539	0.092	-0.088	-0.033	0.5989	-0.096	0.1156	0.2607	0.2595	-0.75	-0.063	-0.2	-0.4	0.045	-0.08	-0.17	-0.18	0.254	-0.16	-0	0.658784	0.146866	0.025544119	0.121321503
2208	P62829	RPL23	ribosomal protein L23	9349	ENSG00000125691	5	0.251	0.081	0.122	0.004	0.513	-0.19	0.092	-0.15	0.181	-0.019	0.0813	-0.155	-0.048	-0.11	0.0043	-0.14	0.2458	0.0665	-0.09	-0.45	0.055	-0.3	-0.12	0.007	0.117	-0.26	-0	0.178451	0.217028	0.109341738	0.107686426
728	F6WQW2	RANBP1	RAN binding protein 1	5902	ENSG00000099901	7	-0.08	0.252	-0.01	-0.08	0.1	-0.02	0.131	0.038	0.285	0.2696	0.1314	-2E-04	-0.047	-0.024	0.178	0.0816	0.1746	0.0719	-0.05	-1.04	-0.29	-0.59	-0.24	0.011	-0.05	0.008	0.162	0.112699	0.299313	-0.024820409	0.32413301
2676	Q68DQ2	CRYBG3	crystallin beta-gamma domain containing 3	131544	ENSG00000080200	29	0.083	0.303	0.088	-0.07	-0.27	-0.04	-0	0.056	0.392	0.05	-0.033	0.0693	-0.181	-0.214	-0.104	0.1142	0.0608	0.179	-0.21	-0.66	-0.09	-0.33	-0.2	0.088	0.183	-0.1	0.049	0.265376	0.200639	0.065467544	0.135171934
1150	O60262	GNG7	G protein subunit gamma 7	2788	ENSG00000176533	5	0.152	0.435	-0.11	0.028	0.041	-0.38	-0.1	-0.15	0.129	0.0107	0.1195	0.2113	0.1319	-0.184	0.36	0.1306	0.0328	0.2158	-0.39	-0.57	-0.29	-0.35	-0.48	-0.23	0.031	0.05	0.421	0.147073	0.20344	-0.111111087	0.314551471
2680	Q6FI81	CIAPIN1	cytokine induced apoptosis inhibitor 1	57019	ENSG00000005194	6	0.693	0.519	0.409	-0.01	0.161	-0.35	-0.4	-0.22	-0.03	0.6891	0.6813	0.2357	0.0869	-0.17	0.1086	-0.205	0.0574	-0.121	-0.08	-0.82	-0.14	-0.42	-0.11	0.203	-0.18	-0.13	-0.17	0.21095	0.292197	-0.065205365	0.357402378
900	I3L1H3	LITAF	lipopolysaccharide induced TNF factor	9516	ENSG00000189067	2	-0.06	0.58	0.012	-0.15	-0.55	-0.45	-0.37	-0.03	-0.41	-0.133	0.5273	-0.11	0.4194	0.0865	-0.054	-0.058	0.3298	0.1118	-0.4	-0.45	-0.19	-0.58	-0.22	-0.2	-0.33	-0.03	0.281	0.147437	0.075346	-0.282865306	0.358211491
600	J3QLD9	FLOT2	flotillin 2	2319	ENSG00000132589	14	-0.11	0.372	-0.21	0.115	-0.14	0.014	0.441	0.298	0.23	-0.243	0.09	0.0956	0.2396	0.0179	0.3676	0.9449	0.001	0.3933	-0.33	-0.3	-0.09	-0.25	-0.38	-0.27	0.12	0.086	-0.01	0.121857	0.271124	-0.09958929	0.370713357
2490	Q15019	SEPT2;SE	septin 2	4735	ENSG00000168385	13	0.166	0.329	0.095	-0.1	0.163	-0.24	0.655	0.119	0.368	0.0621	0.0976	0.0237	0.2703	0.0216	0.2207	0.2313	0.2475	0.4291	-0.06	-0.73	-0.15	-0.14	-0.17	0.199	0.025	0.011	0.357	0.194053	0.245629	-0.00558761	0.251216626
3644	Q9UKL6	PCTP	phosphatidylcholine transfer protein	58488	ENSG00000141179	8	0.054	0.019	-0	0.033	-0.23	-0.22	-0.51	-0.14	0.189	0.2364	0.1374	0.2178	0.1666	-0.251	-0.003	0.1559	-0.214	-0.004	-0.22	-0.45	-0.05	-0.34	-0.15	-0	-0.24	0.057	-0.01	0.217753	0.065306	-0.138772902	0.204079211
520	C9JIR6	PPM1B	protein phosphatase, Mg2+/Mn2+ depender	5495	ENSG00000138032	9	-0.2	-0.29	-0.24	0.35	-0.38	-0.17	0.057	-0.09	0.028	0.2546	0.0547	0.5095	-0.253	-0.009	0.135	0.0373	0.0785	0.0715	-0.6	-0.43	-0.05	-0.19	-0.94	-0.35	-0.06	-0	0.284	0.149813	0.155336	-0.202702818	0.358038518
41	A0A087WU2	SPTBN1	spectrin beta, non-erythrocytic 1	6711	ENSG00000115306	22	0.008	0.116	0.036	-0.35	-0.25	-0.27	-0.08	-0.04	0.08	0.3559	-0.082	0.0031	-0.314	-0.308	-0.134	-0.03	0.0355	-0.058	-0.06	-0.25	0.14	0.217	-0.28	-0.03	0.296	0.011	-0.09	0.76887	-0.07839	-0.02436107	-0.054031823
1094	O15400	STX7	syntaxin 7	8417	ENSG00000079950	9	-0.05	0.61	0.312	-0.09	-0.47	-0.61	0.128	0.202	0.265	0.1557	0.215	0.2597	-0.293	-0.479	-0.024	0.1156	0.0043	0.2943	-0.29	-0.56	-0.35	-0.47	-0.63	-0.47	-0.09	0.086	0.222	0.207749	0.317526	0.005870024	0.311656455
1249	O76076	CCN5;WIS	cellular communication network factor 5;WN	8839	ENSG00000064205	7	-0.41	-0.08	-0.43	-0.5	0.107	-0.55	0.621	0.225	0.472	-0.435	-0.163	-0.602	0.1715	-0.061	-0.58	-0.042	0.2282	-0.109	-0.01	0.51	0.744	-0.16	-0.19	0.128	0.277	0.211	0.246	0.23407	-0.2546	0.11677825	-0.371378077
1521	P09529	INHBB	inhibin subunit beta B	3625	ENSG00000163083	5	0.204	-0.17	-0.52	-0.52	0.344	-0.24	0.039	0.203	-0.07	-0.217	0.1066	0.0272	-0.18	-0.115	0.1179	0.2131	0.3492	0.3227	-0.49	-0.29	-0.25	-0.1	-0.64	-0.05	0.319	-0.26	0.431	0.45128	0.065467	-0.151946841	0.217413634
2171	P61626	LYZ	lysozyme	4069	ENSG00000090382	7	-1.18	-1.68	-0.87	-0.43	-0.38	0.282	-0.17	-0.38	-0.63	-0.098	-0.398	-0.777	-0.688	-0.826	-0.187	-0.267	-0.428	-0.373	-0.55	-0.69	1.345	1.976	-0.15	1.156	-0.27	-0.22	-0.62	0.15773	-0.82514	-0.155898508	-0.669241738
2195	P62328	TMSB4X	thymosin beta 4 X-linked	7114	ENSG00000205542	6	-0.24	0.68	0.412	-0.07	0.441	-0.28	-0.97	-0.67	-0.38	0.5438	0.1541	-0.03	-0.038	-0.042	0.0079	-0.622	0.2826	-0.526	-0.29	-0.26	-0.24	-0.42	-0.25	0.586	-0.76	-0.33	-0.7	0.580093	0.174427	-0.091217262	0.265644055
3238	Q9BPX5	ARPC5L	actin related protein 2/3 complex subunit 5 li	81873	ENSG00000136950	5	0.239	0.233	0.037	-0.09	0.159	-0.5	0.242	0.136	0.051	0.2576	-0.09	0.1472	0.0993	-0.091	0.163	0.2636	0.05	0.0702	0.073	-0.5	-0.01	-0.56	-0.28	-0.07	0.182	-0.06	0.193	0.266558	0.172044	-0.039811865	0.211855519
674	E9PLL6	RPL27A	ribosomal protein L27a	6157	ENSG00000166441	4	-0.08	-0.12	-0.16	-0.06	0.775	-0.18	0.231	-0.05	0.115	-0.246	-0.301	-0.31	0.1388	-0.15	0.0799	0.0217	0.4788	0.3457	-0.31	-0.55	-0.19	-0.15	-0.04	0.107	0.316	-0.14	0.058	0.637173	0.15148	0.045440577	0.106039023
3718	Q9Y2H1	STK38L	serine/threonine kinase 38 like	23012	ENSG00000211455	13	0.069	0.175	-0.02	-0.04	0.008	-0.45	-0.01	-0.16	0.148	0.2908	0.1471	0.1864	0.0632	-0.112	-0.045	-0.046	-0.035	-0.039	-0.15	-0.4	-0.14	-0.43	-0.37	0.11	0.088	-0.2	0.038	0.199807	0.131098	-0.07562249	0.206720449
1091	O15327	INPP4B	inositol polyphosphate-4-phosphatase type I	8821	ENSG00000109452	9	0.173	-0.06	0.043	0.05	-0.13	-0.04	-0.09	0.25	0.38	0.2948	0.1582	0.0825	0.1234	0.1044	0.1015	0.1092	0.1732	0.1486	0.094	-0.29	0.024	-0.27	-0.71	0.109	-0.11	0.195	0.02	0.149356	0.170068	-0.079176296	0.249244032
3798	Q9Y6U3	SCIN	scinderin	85477	ENSG00000006747	15	0.395	-0.59	0.291	-0.15	0.061	-0.26	0.246	0.498	0.684	-0.063	-0.309	0.003	0.2994	-3E-04	-0.33	0.1246	-0.65	-0.317	0.291	-0.58	0.351	-0.18	-0.23	0.084	0.457	0.149	-0.27	0.439538	0.122208	0.268300844	-0.146093052
2194	P62312	LSM6	LSM6 homolog, U6 small nuclear RNA and	11157	ENSG00000164167	2							0.157	-0.16	-0.09							-0.199	-0.498	-0.334							0.234	-0.06	-0	0.168563	-0.08926	0.313699228	-0.402959375
98	Q2TAM5	RELA	RELA proto-oncogene, NF-kB subunit	5970	ENSG00000173039	9	-0.09	0.012	-0.28	-0.1	0.083	-0.41	0.059	0.098	0.23	-0.093	-0.134	-0.004	0.0107	0.0399	0.0012	0.1043	0.1828	0.142	-0.26	-0.57	-0.09	-0.43	-0.23	0.135	0.184	0.047	0.204	0.524833	0.067827	-0.072246454	0.14007366
159	A0A0A0MT3	LIPA	lipase A, lysosomal acid type	3988	ENSG00000107798	5	-0.18	-0.63	0.495	-0.48	-0.37	-0.45	-0.86	-0.08	-0.46	0.3858	0.3371	0.2477	-0.131	0.0844	-0.38	-0.389	-1	-0.693	-0.43	0.306	0.345	0.071	0.148	0.114	0.241	0.132	-1.09	0.489878	-0.31728	-0.164301804	-0.152977677
2972	Q8TC12	RDH11	retinol dehydrogenase 11	51109	ENSG00000072042	8	-0.23	-0.26	-0.22	0.142	-0.14	-0.12	-0.04	-0.01	0.202	-0.078	-0.146	-0.104	0.0538	0.039	0.1578	0.0587	-0.214	-0.14	-0.63	-0.25	0.278	0.001	0.019	0.059	0.268	0.26	0.406	0.627403	-0.12168	-0.035166846	-0.086511255
3126	Q96EU7	C1GALT1(	C1GALT1 specific chaperone 1	29071	ENSG00000171155	9	0.055	-0.42	-0.05	-0.28	-0.58	-0.37	0.152	0.464	0.211	0.1768	-0.003	0.174	-0.71	0.141	-0.366	0.3018	-0.222	-0.099	0.053	0.234	0.141	-0.19	-0.31	-0.47	0.544	0.131	0.041	0.825595	-0.1114	-0.023474091	-0.087929022
2415	Q13526	PIN1	peptidylprolyl cis/trans isomerase, NIMA-inte	5300	ENSG00000127445	9	-0.1	0.29	0.069	0.03	-0.13	-0.29	0.032	-0.12	0.017	0.0593	0.0922	0.006	-0.028	-0.127	0.2351	0.0501	0.249	-0.019	-0.17	-0.57	-0.28	-0.47	-0.32	0.079	-0.21	-0.04	0.123	0.114928	0.183356	-0.079961224	0.263317147
3645	Q9UKM7	MAN1B1	mannosidase alpha class 1B member 1	11253	ENSG00000177239	9	-0.04	-0.34	-0.13	-0.16	0.133	-0.01	-0.39	-0.06	0.034	-0.181	0.097	-0.022	-0.081	-0.155	-0.153	0.2288	0.1977	0.3453	-0.17	-0	0.058	0.036	-0.59	-0.07	-0	0.025	0.164	0.482555	-0.04533	-0.138343184	0.093017153
2182	P62136	PPP1CA	protein phosphatase 1 catalytic subunit alph	5499	ENSG00000172531	17	-0.13	-0.17	-0.26	-0.03	-0.01	-0.24	-0.04	-0.09	0.034	-0.256	-0.159	-0.18	0.0319	-0.029	0.2213	0.0333	0.0363	-0.044	-0.14	-0.45	-0.13	-0.16	-0.38	-0	-0.04	-0.08	0.164	0.539862	0.030891	-0.065087148	0.095977654
1180	O60826	CCDC22	coiled-coil domain containing 22	28952	ENSG00000101997	12	-0.07	-0.01	0.049	-0.01	-0.18	-0.45	-0.04	-0.04	0.16	-0.049	0.026	-0.138	-0.236	-0.208	0.0979	-0.064	0.2932	0.1927	-0.17	-0.71	-0.06	-0.45	-0.45	-0.07	-0.09	0.089	0.141	0.346233	0.129972	-0.056958515	0.186930794
1420	P04424	ASL	argininosuccinate lyase	435	ENSG00000126522	19	0.336	0.246	0.128	0.015	0.138	-0.39	-0.11	-0.18	0.166	0.3523	-0.558	0.3201	0.1671	0.2079	-0.024	0.0762	0.0555	-0.026	0.02	-0.64	-0.35	-0.22	-0.07	0.08	0.199	0.161	-0.19	0.462312	0.152294	-0.024398432	0.176692211
868	O95166	GABARAP	GABA type A receptor-associated protein	11337	ENSG00000170296	3	0.055	0.166	0.019	0.247	0.265	-0.11	0.553	0.411	0.426	-0.061	0.168	-0.069	0.297	-0.305	0.0657	0.3635	0.2694	0.1988	-0.39	-0.94	-0.28	-0.32	-0.11	-0.07	0.016	0.003	0.105	0.056379	0.446654	0.122941923	0.323711661
39	A0A087WUT	EIF5B	eukaryotic translation initiation factor 5B	9669	ENSG00000158417	10	0.18	0.106	-0.15	-0	0.009	-0.18	-0.13	0.18	0.029	0.2215	0.042	-0.09	0.0187	-0.174	-0.006	0.0313	-0.042	0.1032	0.037	0.163	-0.15	0.223	-0.12	0.116	0.291	0.092	0.229	0.451276	-0.09216	-0.007004219	-0.085159364
3337	Q9GZT8	NIF3L1	NGG1 interacting factor 3 like 1	60491	ENSG00000196290	10	0.105	0.178	0.092	0.078	0.4	-0.4	0.295	0.206	0.168	0.2991	0.0191	0.2216	0.1445	-0.172	0.083	0.2132	0.1958	0.1898	-0.26	-0.69	-0.28	-0.36	-0.23	0.091	0.32	0.217	0.109	0.190299	0.244024	-0.007827582	0.251851866
2905	Q8N5J2	MINDY1	MINDY lysine 48 deubiquitinase 1	55793	ENSG00000143409	7	-0.21	-0.12	-0.12	0.023	-0.05	-0.55	0.337	0.306	0.413	-0.034	-0.062	-0.162	0.0811	-0.51	0.3276	0.41	-0.023	0.0226	-0.31	-1.21	-0.3	-0.86	-0.54	0.163	0.241	-0	0.206	0.331612	0.294134	-0.001829753	0.29596342
1939	P45985	MAP2K4	mitogen-activated protein kinase kinase 4	6416	ENSG00000065559	12	0.036	0.364	-0.13	-0.07	0.071	-0.36	-0.08	-0.07	0.183	-0.011	-0.069	0.0836	0.0372	-0.142	-0.128	0.0975	0.1632	0.0348	-0.25	-1.26	-0.24	-0.41	-0.19	0.1	-0.06	0.218	0.199	0.380701	0.204906	-0.013204105	0.218110074
2929	Q8NCH0	CHST14	carbohydrate sulfotransferase 14	113189	ENSG00000169105	11	0.157	-0.19	-0.34	-0.38	0.073	-0.31	-0.05	-0.08	-0.04	0.282	0.178	0.0883	-0.072	0.1445	-0.167	0.1459	-0.098	-4E-04	0.52	0.593	0.085	-0.26	-0.1	-0.02	0.31	0.018	0.174	0.149569	-0.27544	-0.184937698	-0.090499675
3471	Q9NQY0	BIN3	bridging integrator 3	55909	ENSG00000147439	10	0.369	0.148	-0.08	0.084	-0.14	-0.25	0.149	-0.1	0.023	0.0874	0.1589	-0.107	0.0711	0.2384	0.3154	0.0876	0.1064	0.2204	-0.26	-0.88	-0.14	-0.8	-0.4	-0.1	0.007	-0.08	0.135	0.063336	0.301281	-0.108811789	0.410092821
955	O60437	PPL	periplakin	5493	ENSG00000118898	3	0.14	0.246	-0.13				0.183	-0.12	0.105	-0.14	-0.07	0.1357				0.0625	0.3569	0.1086	-0.35	-1.15	-0.32				-0.2	-0.04	0.199	0.198926	0.381635	-0.005290057	0.386925486
2016	P50150	GNG4	G protein subunit gamma 4	2786	ENSG00000168243	8	0.237	0.231	-0.46	0.107	0.252	-0.66	0.205	-0.26	0.016	-0.188	-0.078	0.6078	0.5379	-0.219	0.5203	0.6463	0.636	0.3801	-0.36	-0.16	-0.78	-0.82	-0.77	-0.38	0.27	0.093	0.612	0.130752	0.219885	-0.352342061	0.57222729
3703	Q9Y263	PLAA	phospholipase A2 activating protein	9373	ENSG00000137055	19	-0.08	-0.09	-0.12	-0.27	-0.31	-0.45	-0.03	0.237	0.133	0.0216	-0.054	-0.049	-0.214	-0.297	-0.23	0.0788	0.1139	0.1253	-0.2	0.131	0.129	0.283	-0.32	0.044	0.122	0.032	0.108	0.427063	-0.14546	-0.052874443	-0.092590522
316	H3BV07	TBC1D24	TBC1 domain family member 24	57465	ENSG00000162065	9	0.197	0.171	0.226	0.064	0.125	-0.23	-0.02	0.175	0.202	0.2397	0.2683	0.1918	0.16	-0.195	0.0719	0.2221	0.138	0.1267	-0.2	-0.64	-0.14	-0.2	-0.24	0.01	0.18	0.04	0.004	0.091624	0.231843	-0.034916207	0.26675934
3184	Q96R05	RBP7	retinol binding protein 7	116362	ENSG00000162444	7	-0.11	-0.23	-0.04	-0.22	0.044	-0.04	-0.32	-0.04	0.021	-0.085	-0.024	-0.117	0.3182	0.0714	-0.082	-0.065	0.1173	-0.1	-0.22	-0.09	0.057	-0.27	-0.09	0.046	-0.06	-0.17	-0.04	0.310527	-0.01187	-0.109098925	0.097229534
3217	Q99613	EIF3C	eukaryotic translation initiation factor 3 subu	8663	ENSG00000184110	23	0.238	-0.05	0.012	0.047	0.034	-0.26	-0.19	-0.18	0.053	0.0512	0.1754	0.0211	-0.045	-0.14	-0.018	-0.028	0.0816	0.1217	-0.12	-0.48	0.047	-0.16	-0.25	-0.06	0.18	-0	0.046	0.464955	0.0572	-0.056603153	0.113803293
2036	P51151	RAB9A	RAB9A, member RAS oncogene family	9367	ENSG00000123595	6	-0.17	0.176	0.057	-0.05	-0.21	-0.32	0.122	-0.1	0.243	0.0355	0.1467	0.0525	0.1021	-0.339	0.0405	0.0555	0.4596	0.255	-0.23	-0.35	-0.15	-0.63	-0.35	-0.08	-0.06	0.018	0.268	0.187769	0.148035	-0.116431163	0.264466593
1125	O43592	XPOT	exportin for tRNA	11260	ENSG00000184575	13	0.278	0.284	0.035	0.082	0.784	-0.02	-0.18	0.161	0.041	0.3467	0.2155	0.1798	0.0672	0.0888	-0.116	0.0532	0.1202	0.0939	-0.19	-0.56	-0.11	-0.16	0.002	0.123	0.07	-0.05	0.135	0.177011	0.246073	0.045976058	0.200097081
1737	P23919	DTYMK	deoxythymidylate kinase	1841	ENSG00000168393	8				-0.38	-0.67	-0.93	-0.11	-0.16	-0.09				-0.989	-0.717	-0.518	-0.128	0.1084	-0.126				-0.84	-0.94	-1.16	-0.04	-0.2	0.173	0.924063	0.11098	0.004861186	0.10611876
3606	Q9UH65	SWAP70	switching B cell complex subunit SWAP70	23075	ENSG00000133789	15	0.205	0.229	0.092	0.125	0.011	-0.4	0.042	-0.27	0.318	0.2832	0.3117	0.1737	0.1275	-0.043	0.1132	0.0962	0.2385	-0.024	-0.13	-1.2	-0.35	-0.49	-0.26	0.013	0.093	0.087	0.31	0.177124	0.253031	-0.102779267	0.355810232
1069	O15068	MCF2L	MCF.2 cell line derived transforming sequer	23263	ENSG00000126217	11	-0.07	0.204	-0.08	-0.06	0.027	-0.16	0.009	-0.15	0.034	-0.049	0.3398	-0.011	-0.146	0.2545	0.0494	0.0297	0.2686	-0.049	-0.19	-0.63	-0.27	-0.34	-0.06	0.116	0.036	-0.2	0.12	0.157619	0.132361	-0.102153398	0.234514777
1749	P25686	DNAJB2	DnaJ heat shock protein family (Hsp40) mei	3300	ENSG00000135924	13	0.147	0.341	0.116	-0.13	0.035	-0.41	0.113	0.031	0.296	0.0985	0.0831	-0.111	-0.173	0.0581	0.3083	-0.04	0.0479	-0.149	-0.35	-0.84	-0.32	-0.41	-0.14	0.129	-0.09	-0.07	0.155	0.172383	0.274065	0.046497997	0.227567181
1529	P0C8F1	PATE4	prostate and testis expressed 4	399968	ENSG00000237353	11	1.683	-2.83	-1.88	-1.62	0.514	1.319	-1.2	-1.94	1.351	-2.985	-1.715	-2.063	-1.113	-1.334	-1.641	1.3235	-1.485	-1.505	-2.25	-2.33	1.797	-1.38	1.72	-0.06	-1.16	1.167	-1.86	0.528537	-0.02704	0.880368696	-0.907408485
2663	Q5W0U4	BSPRY	B-box and SPRY domain containing	54836	ENSG00000119411	13	0.221	-0.54	0.271	0.084	-0.07	-0.28	-0.52	-0.48	-0.19	-0.429	-0.132	-0.206	0.2793	-0.054	0.1684	-0.056	0.0289	-0.29	-0.76	-0.11	-0.47	-0.45	-0.37	0.16	-0	-0.07	-0.21	0.55786	0.086957	-0.089926958	0.176883727
2281	Q00577	PURA	purine rich element binding protein A	5813	ENSG00000185129	9	0.398	0.182	-0.08	-0.24	-0.03	-0.46	-0.11	-0.07	0.256	0.0786	0.1009	0.195	0.1057	0.0189	0.0362	0.0133	0.0024	-0.149	0.074	-0.59	-0.09	-0.31	-0.18	0.185	0.247	0.057	0.2	0.78528	0.029295	-0.061133624	0.090428583
1925	P42766	RPL35	ribosomal protein L35	11224	ENSG00000136942	5	0.207	-0.19	-0.21	-0.13	0.747	-0.16	0.351	-0.05	0.324	-0.276	-0.162	-0.17	-0.34	-0.27	-0.005	-0.113	0.0029	0.008	0.586	-0.82	0.136	-0.66	0	-0.14	0.117	-0.1	0.254	0.435918	0.167828	0.246221815	-0.078393824
393	P49840	GSK3A	glycogen synthase kinase 3 alpha	2931	ENSG00000105723	6	-0.02	0.254	-0.07	-0.1	-0.15	-0.39	-0.01	-0.18	0.215	0.1716	0.0042	-0.005	-0.141	-0.257	0.0376	-0.168	0.0308	0.2738	-0.3	-0.74	-0.38	-0.35	-0.14	0.015	0.075	-0.07	0.075	0.301123	0.151819	-0.04491342	0.19673208
3280	Q9BV20	MRI1	methylthioribose-1-phosphate isomerase 1	84245	ENSG00000037757	12	0.505	0.627	0.137	-0.36	0.028	-0.17	0.383	0.139	0.395	0.4313	0.325	0.1782	-0.424	0.1763	0.1211	-0.096	0.1828	0.0697	0.301	-0.19	0.458	-0.01	-0.27	0.193	0.416	0.193	0.557	0.870466	0.006143	0.080814916	-0.074671418
719	F5H6I3	NELL1	neural EGFL like 1	4745	ENSG00000165973	12	0.352	-0.21	-0.69	-0.5	0.104	-0.07	-0.17	0.394	-0.38	-0.043	0.2261	0.6507	-0.232	-0.329	-0.299	0.611	-0.141	0.7183	-0.3	-0.53	0.232	-0.63	0.039	-0.24	0.322	0.271	-0.18	0.4554	-0.01428	-0.256965968	0.242687673
452	P16422	EPCAM	epithelial cell adhesion molecule	4072	ENSG00000119888	7	-0.39	0.291	0.769	-0.67	-0.07	0.53	-0.13	0.348	-0.18	-0.413	0.5127	-0.414	-0.275	0.2146	0.0577	-0.378	0.0742	0.0931	-0.75	0.845	-0.28	-0.37	-0.42	-0.16	0.736	0.163	-0.16	0.898429	0.097716	0.1129052	-0.015188869
1981	P48745	CCN3;NOV	cellular communication network factor 3;nep	4856	ENSG00000136999	9	-0.77	-0.57	-0.4	-0.1	-0.54	-0.59	0.375	-0.37	0.808	0.0348	0.5636	0.801	-0.031	-0.145	-0.126	0.6195	0.4164	-0.169	-0.33	-0.22	-0.51	-0.79	-0.57	-0.32	0.772	0.421	-0.53	0.217096	-0.01014	-0.460355731	0.450210736
1690	P20338	RAB4A	RAB4A, member RAS oncogene family	5867	ENSG00000168118	10	-0.04	0.12	0.031	-0.29	0.077	-0.42	-0.05	0.188	0.035	0.0348	0.1017	0.0894	-0.18	-0.154	-0.168	0.1863	0.1176	0.007	-0.16	-0.26	-0.2	-0.39	-0.04	0.044	-0.01	0.154	0.245	0.791952	0.029234	-0.042755537	0.071989126

2012	P49914	MTHFS	methenyltetrahydrofolate synthetase	10588	ENSG00000136371	7	0.232	0.127	-0.04	0.329	0.046	-0.05	-0.11	-0.1	-0.29	-0.195	-0.023	0.0364	0.1857	-0.05	0.4456	0.1385	0.0398	-0.015	-0.23	-1.27	-0.37	-0.56	-0.02	0.18	0.048	-0.03	0.102	0.23561	0.253688	-0.046697155	0.300385448
621	E7EW84	EXOC6	exocyst complex component 6	54536	ENSG00000138190	15	0.037	0.128	-0.25	-0.08	-0.23	-0.2	-0.15	-0.08	0.101	-0.069	0.0244	0.1885	-0.02	0.0532	-0.145	0.0962	0.1256	0.2283	-0.31	-0.83	-0.21	-0.24	-0.31	0.076	-0.05	0.103	0.191	0.219945	0.094144	-0.134327687	0.228471201
295	A0A1W2PP	SCARB2	scavenger receptor class B member 2	950	ENSG00000138760	7	-0.12	0.059	0.214	-0.13	-0.34	-0.32	-0.14	0.142	0.349	0.2811	0.0997	-0.022	-0.071	-0.088	-0.133	-0.208	0.153	0.0685	-0.3	-0.47	-0.28	-0.38	-0.24	-0.2	0.139	0.169	0.445	0.625792	0.092251	-0.040931842	0.133182825
2567	Q27J81	INF2	inverted formin, FH2 and WH2 domain cont	64423	ENSG00000203485	13	0.115	0.234	1E-04	-0	-0.27	-0.46	0.157	-0.09	0.103	0.1579	0.229	0.1265	0.0075	-0.02	0.3518	0.1338	0.006	-0.159	-0.31	-1.09	-0.42	-0.48	-0.46	-0.04	-0.04	0.03	0.18	0.120362	0.268806	-0.116707164	0.38551358
374	K7EIJ0	WBP2	WW domain binding protein 2	23558	ENSG00000132471	6	-0.24	0.34	-0.1	-0.1	-0.01	-0.46	-0.14	-0.21	-0.26	-0.076	0.0912	0.072	0.0424	-0.003	0.1981	0.0747	0.3403	0.0409	-0.22	-0.09	-0.01	-0.49	-0.48	-0.25	-0.23	-0.22	0.033	0.065867	0.087632	-0.217568735	0.305200796
2430	Q13724	MOGS	mannosyl-oligosaccharide glucosidase	7841	ENSG00000115275	14	-0.16	-0.24	-0.23	-0.41	-0.08	-0.18	-0.79	-0.3	-0.48	0.2822	-0.171	0.0984	-0.136	0.1815	-0.259	0.027	-0.019	-0.403	-0.11	0.073	-0.11	-0.26	-0.11	-0.31	0.536	0.182	-0.31	0.146565	-0.27403	-0.276926487	0.002895216
184	A0A0C4DFZ	ARSA	arylsulfatase A	410	ENSG00000100299	9	-0.11	-0.58	0.077	-0.23	-0.14	-0.01	-0.38	0.076	-0.12	0.4056	-0.207	-0.093	-0.418	0.1165	-0.363	-0.133	-0.382	-0.505	0.041	0.787	0.091	0.26	0.157	-0.26	0.472	0.304	-0.65	0.222683	-0.2927	0.017092922	-0.309793687
3072	Q969E2	SCAMP4	secretory carrier membrane protein 4	113178	ENSG00000227500	4	-0.33	-0.03	0.075	0.081	0.408	7E-04	-0.29	0.102	-0.07	-0.246	0.1122	0.0033	0.2405	-0.184	0.2718	-0.138	-0.042	-0.028	-0.35	-0.61	-0.54	-0.69	-0.19	0.068	-0.33	-0.05	-0.08	0.102613	0.300595	-0.005400039	0.305995112
2241	Q5SRQ3	CSNK2B;C	casein kinase 2 beta;novel protein	1460	ENSG00000224774	5	0.296	0.181	-0.06	-0.1	0.144	-0.42	-0.09	-0.05	0.165	-0.036	-0.108	-0.067	-0.08	-0.149	0.1649	0.2029	0.2123	0.158	-0.21	-0.72	-0.19	-0.6	-0.48	-0.02	0.024	-0.18	0.15	0.138884	0.255015	-0.025503439	0.280518828
1240	O75935	DCTN3	dynactin subunit 3	11258	ENSG00000137100	8	0.28	0.311	-0.05	0.132	0.356	-0.31	0.053	-0.2	-0.07	0.1432	0.156	0.1354	0.009	0.0392	0.1872	0.2077	-0.391	-0.173	-0.14	-0.82	-0.14	-0.53	-0.27	-0.03	0.086	0.427	-0.29	0.27551	0.244243	0.020142442	0.224100635
343	G3V196	RAB15	RAB15, member RAS oncogene family	376267	ENSG00000139998	7				0.525	-0.02	-0.17	0.514	0.403	0.692				0.3047	-0.185	0.1717	0.7463	0.3096	0.767				-0.51	-0.14	0.082	0.256	0.252	0.544	0.524131	0.244003	-0.028839525	0.272843021
1524	P09936	UCHL1	ubiquitin C-terminal hydrolase L1	7345	ENSG00000154277	9	0.445	-0.02	-0.31	0.075	0.304	-0.31	0.062	0.573	0.019	0.0422	-0.095	-0.27	-0.268	-0.282	-0.093	0.3056	0.0537	0.5887	-0.1	-0.57	-0.28	-0.3	-0.01	-0.03	0.387	-0.19	0.084	0.497433	0.204022	0.094728231	0.109293328
2025	P50583	NUDT2	nudix hydrolase 2	318	ENSG00000164978	9	0.028	0.216	-0.24	-2.03	-2.64	-2.4	0.306	0.114	0.674	0.0656	0.1392	0.3252	-2.539	-1.917	-2.178	0.3441	0.3846	0.2567	-0.33	-0.64	-0.01	-2.61	-2.84	-2.19	0.034	0.313	0.437	0.918195	0.207863	-0.095540377	0.303403233
2055	P52306	RAP1GDS	Rap1 GTPase-GDP dissociation stimulator	5910	ENSG00000138698	14	0.171	0.188	0.1	0.057	-0.01	-0.35	-0.2	-0.05	-0.15	0.3903	0.2488	0.1777	-0.013	-0.148	0.0565	-0.061	0.0631	-0.257	-0.09	-0.66	-0.16	-0.45	-0.33	0.045	-0.11	-0.01	-0.09	0.15662	0.178741	-0.079176364	0.257917352
3582	Q9UBN7	HDAC6	histone deacetylase 6	10013	ENSG00000094631	7	0.247	0.3	0.123	0.36	0.214	-0.28	-0.3	-0.19	-0.09	0.1435	0.0994	0.1948	0.2771	-0.111	0.3672	-0.001	-0.051	-0.098	-0.21	-1.07	-0.3	-0.4	-0.06	0.144	0.004	-0.02	-0.08	0.17521	0.26486	-0.047379811	0.312239621
3744	Q9Y3I0	RTCB	RNA 2',3'-cyclic phosphate and 5'-OH ligas	51493	ENSG00000100220	11	-0.03	0.008	-0.07	-0.16	0.022	-0.32	0.048	-0.06	0.132	0.1101	-0.005	0.0059	-0.032	0.1046	0.1578	0.1116	0.0292	-0.043	-0.12	-0.69	-0.08	-0.19	-0.22	0.016	0.09	0.059	0.183	0.328179	0.059575	-0.095210589	0.15478556
3199	Q99426	TBCB	tubulin folding cofactor B	1155	ENSG00000105254	7	0.217	0.346	-0.09	-0.02	-0.14	-0.34	-0.19	-0.11	-0.17	0.2774	0.0491	0.0759	-0.14	-0.052	0.1971	-0.207	-0.009	-0.405	-0.05	-0.96	-0.31	-0.61	-0.32	0.012	-0.04	-0.07	-0.09	0.245386	0.214268	-0.031309389	0.245577222
2320	Q05DH4	FAM160A1	family with sequence similarity 160 member	729830	ENSG00000164142	10	0.155	0.309	-0.01	-0.03	-0.26	-0.35	-0.05	0.003	0.111	0.2591	0.087	0.1121	-0.056	0.0254	0.152	-0.081	0.3165	0.1146	-0.27	-0.8	-0.02	-0.59	-0.41	-0.07	-0.03	-0.09	0.328	0.145935	0.202202	-0.116952447	0.31915462
3546	Q9NZB2	FAM120A	family with sequence similarity 120A	23196	ENSG00000048828	12	0.197	-0.05	-0.08	-0.13	0.114	-0.33	0.056	0.039	0.172	-0.062	0.0175	0.0635	0.241	0.0275	0.0298	0.0618	-5E-04	0.1249	0.042	-0.4	-0.1	-0.29	-0.13	0.212	0.349	0.013	0.201	0.78071	0.011066	-0.057237055	0.068303427
263	A0A1B0GW	ADSL	adenylosuccinate lyase	158	ENSG00000239900	12	-0.02	0.052	-0.15	-0.13	0.088	-0.47	0.243	-0.09	0.147	0.1508	0.2812	0.1709	-0.057	-0.223	0.2004	0.2465	0.1163	0.2984	-0.11	-0.24	-0.05	-0.25	-0.26	-0.03	0.193	-0	0.128	0.193495	0.033062	-0.167240473	0.200302834
1985	P49207	RPL34	ribosomal protein L34	6164	ENSG00000109475	5	0.264	0.05	0.041	-0.08	0.255	0.036	0.372	0.04	0.334	-0.02	-0.119	-0.1	-0.186	-0.124	0.1792	0.172	-0.155	0.0614	-0.1	-0.65	0.182	0.033	-0.04	-0.03	0.482	-0.12	0.101	0.322263	0.16119	0.177897015	-0.016706835
3216	Q99598	TSNAX	translin associated factor X	7257	ENSG00000116918	8	0.042	-0.05	0.03				0.295	0.339	0.292	0.2275	-0.111	-0.065				0.2445	0.1146	0.1383	-0.17	-0.35	-0.16				0.305	0.113	0.015	0.356301	0.199399	0.067094744	0.132303797
2259	P78539	SRPX	sushi repeat containing protein X-linked	8406	ENSG00000101955	6	-0.49	-0.28	-0.33	0.184	0.131	-0.29	-0.04	-0.12	-0.06	0.1361	-0.016	0.216	-0.061	-0.517	-0.021	0.1134	-0.386	0.572	-0.6	0.073	0.3	0.344	-0.19	0.504	0.126	4E-04	0.442	0.373106	-0.25519	-0.148250137	-0.106942909
3194	Q96SU4	OSBPL9	oxysterol binding protein like 9	114883	ENSG00000117859	12	0.423	0.494	0.197	0.023	0.111	-0.19	-0.13	-0.12	0.053	0.2073	0.0295	-0.024	-0.043	0.0089	-0.141	-0.017	-0.048	-0.046	0.608	-0.18	0.084	-0.13	-0.04	-0.05	0.175	-0.06	0.089	0.678547	0.040812	0.103279081	-0.062467112
530	C9JP16	CRTAP	cartilage associated protein	10491	ENSG00000170275	9	-0.26	-0.26	-0.33	-0.5	-0	-0.29	0.076	0.067	0.185	0.236	-0.161	-0.351	-0.109	0.2402	0.156	-0.176	-0.221	-0.248	0.322	0.974	0.056	-0.51	0.002	-0.16	0.198	-0.39	-0.12	0.59752	-0.1867	-0.075618436	-0.111082554
1241	O75955	FLOT1	flotillin 1	10211	ENSG00000236271	16	-0.67	-0.43	-0.8	0.238	-0.06	0.05	-0.25	-0.01	-0.03	0.612	-0.465	-0.311	0.0725	0.0088	0.3776	0.3823	0.0003	0.0787	0.534	-0.13	-0.49	0.508	-0.27	-0.16	-0.05	0.03	0.117	0.325858	-0.22655	-0.301426836	0.074874073
2280	Q00535	CDK5	cyclin dependent kinase 5	1020	ENSG00000164885	7	0.279	0.325	0.289	-0.04	-0.07	-0.24	-0.06	-0.02	0.159	0.6544	0.2429	0.0416	0.0668	-0.145	0.1104	0.0438	-0.091	-0.085	0.824	0.586	0.238	-0.18	-0.28	-0.04	0.165	-0.07	0.084	0.885982	-0.07778	-0.023168814	-0.054607567
1585	P13497	BMP1	bone morphogenetic protein 1	649	ENSG00000168487	13	-0.05	-0.28	-0.41	-0.3	0.097	-0.29	-0.09	0.265	0.197	-0.077	0.246	0.1369	-0.27	-0.057	-0.12	0.4168	0.4644	0.4396	-0.09	0.06	0.1	-0.29	-0.64	-0.14	0.408	0.024	-0.19	0.306389	-0.01257	-0.227203873	0.214633754
297	A0A1W2PQ	RPS10-NU	RPS10-NUDT3 readthrough	100529239	ENSG00000270800	10	0.45	0.018	0.083	0.017	0.239	-0.03	-0.1	0.164	-0.02	0.0165	0.0361	0.2178	0.1171	0.0882	0.1102	0.239	-0.042	-0.068	0.01	-0.46	0.012	-0.07	-0.08	0.165	-0.17	-0.2	0.145	0.208596	0.162389	0.011327132	0.151061869
10	A0A075B6H	TMCO4	transmembrane and coiled-coil domains 4	255104	ENSG00000162542	10	0.395	0.385	-0.03	0.113	-0.16	-0.07	-0.02	-0.3	0.153	0.0548	0.3237	0.2652	0.0912	-0.229	-0.012	0.0537	0.0553	-0.112	-0.13	-1.3	-0.4	0.432	-0.49	0.177	-0.02	0.085	0.19	0.477213	0.213902	-0.002695209	0.216596774
3218	Q99614	TTC1	tetratricopeptide repeat domain 1	7265	ENSG00000113312	4	-0.07	0.168	0.012	0.039	-0.02	-0.16	0.001	-0.16	0.127	-0.063	0.0178	0.0665	-0.329	0.1339	-0.015	-0.07	-0.013	-0.334	0.077	-0.32	0.032	-0.27	-0.38	-0.19	0.047	-0.09	0.033	0.476926	0.111661	0.060119335	0.051541724
3560	Q9P0V3	SH3BP4	SH3 domain binding protein 4	23677	ENSG00000130147	17	0.176	0.442	-0.03	0.129	-0.28	-0.12	-0.26	-0.2	-0.09	-0.1	-0.011	0.2948	-0.088	0.2928	0.2649	0.1935	0.0262	0.3558	-0.17	0.029	-0.16	-0.24	-0.3	-0.22	-0.07	0.135	0.296	0.225892	0.05304	-0.16255115	0.215591567
2103	P55036	PSMD4	proteasome 26S subunit, non-ATPase 4	5710	ENSG00000159352	9	-0.18	-0.35	-0.4	-0.04	0.283	-0.09	0.073	0.106	0.218	-0.435	-0.432	-0.281	-0.074	-0.268	0.1347	0.0189	0.0647	-0.025	-0.24	-0.8	-0.53	-0.53	-0.13	-0.04	0.201	0.018	0.126	0.563591	0.169965	0.101227248	0.06873748
216	A0A0D9SGF	SPTAN1	spectrin alpha, non-erythrocytic 1	6709	ENSG00000197694	24	0.027	0.149	0.011	-0.25	0.124	-0.46	-0.12	0.068	0.296	0.2593	0.1638	0.0221	-0.101	-0.069	0.0551	0.1093	0.0202	-8E-04	-0.15	-0.22	-0.08	-0.19	-0.16	-0.02	0.249	0.16	-0.22	0.514424	0.052761	-0.067919036	0.120680104
2605	Q5GLZ8	HERC4	HECT and RLD domain containing E3 ubiqu	26091	ENSG00000148634	17	0.106	0.209	0.01	0.007	0.004	-0.34	0.06	0.067	0.259	0.1096	-0.095	0.1852	0.0209	-0.147	-0.027	0.1693	0.3105	0.2567	-0.17	-0.61	-0.24	-0.32	-0.19	0.035	0.097	0.209	0.328	0.346632	0.137382	-0.045092346	0.18247427
2612	Q5JTZ5	C9orf152	chromosome 9 open reading frame 152	401546	ENSG00000188959	10	0.338	0.273	0.175	-0.22	-0.5	-0.03	0.032	-0.17	0.489	0.0849	-0.12	0.1392	0.3259	-0.054	-0.332	0.1354	0.1483	0.0241	-0.05	-0.93	-0.35	-1.13	-0.42	0.222	-0.12	-0.11	0.268	0.220097	0.33374	0.003228986	0.33051107
3738	Q9Y3C6	PPIL1	peptidylprolyl isomerase like 1	51645	ENSG00000137168	7	-0.17	0.233	0.132	0.016	-0.01	-0.5	-0.04	0.014	0.26	0.1984	0.0897	-0.174	-0.217	-0.076	0.036	0.1978	0.0436	0.193	-0.18	-0.66	-0.12	-0.58	-0.43	0.044	-0.01	0.201	0.125	0.317347	0.17149	-0.039636523	0.211126774
2072	P53582	METAP1	methionyl aminopeptidase 1	23173	ENSG00000164024	9	-0.1	-0.01	-0.14	-0.27	-0.06	-0.11	0.49	0.444	0.187	0.1005	-0.016	0.0367	-0.049	-0.008	-0.068	1.8018	0.1688	0.2874	-0.29	-0.7	-0.06	-0.39	0.074	0.325	1.005	0.032	0.152	0.650218	0.030617	-0.20237656	0.232994028
1356	P01303	NPY	neuropeptide Y	4852	ENSG00000122585	4	0.664	0.778	-0.13	-0.57	-0.17	0.55	-0.58	0.361	0.842	-0.084	-0.505	0.0237	-0.96	-1.017	-0.537	0.2928	0.2371	-0.379	-0.67	0.962	-0.5	-1.09	-1.2	-0.49	-0.64	0.655	-0.68	0.227999	0.601452	0.520540159	0.080911851
1792	P29622	SERPINA4	serpin family A member 4	5267	ENSG00000100665	7	-0.19	-0.48	-0.22	0.176	-0.02	0.119	0.056	0.336	0.11	-0.045	0.1589	0.2039	-0.104	0.1029	-0.195	0.8838	-0.495	-0.241	-0.05	0.473	-0.13	0.252	0.094	0.125	-0.22	0.103	-0.21	0.934823	-0.06117	-0.042713579	-0.018456031
262	A0A182DWI	SELENOP	selenoprotein P	6414	ENSG00000250722	7	0.021	-0.3	-0.14	-0.08	-0.03	-0.08	-0.09	0.25	0.123	0.8645	0.267	0.5701	-0.151	0.2994	-0.179	0.3078	-0.359	0.1297	0.161	0.104	0.322	-0.07	0.174	-0.03	0.198	0.368	-0.21	0.343553	-0.1506	-0.230924812	0.080326192
3268	Q9BU02	THTPA	thiamine triphosphatase	79178	ENSG00000259431	6	-0.18	-0.17	0.112	0.035	0.217	-0.38	0.132	0.065	0.201	-0.347	-0.174	-0.26	-0.012	-0.088	0.2005	0.0283	0.0938	0.2402	-0.23	0.085	-0.11	-0.62	-0.46	0.055	0.136	0.159	0.195	0.804135	0.090833	0.038870166	0.051962829
364	A4D1S5	RAB19	RAB19, member RAS oncogene family	401409	ENSG00000146955	8	0.825	0.112	-0.36	0.776	0.136	-0.36	0.004	-0.28	0.035	-0.068	0.3744	-0.097	0.2575	-0.381	0.2292	0.0305	0.3109	-0.157	0.024	-0.96	1.488	-0.38	-0.44	1.45	-0.04	-0.2	0.249	0.972465	-0.0327	0.043943649	-0.076640759
2846	Q8IVN8	SBSPON	somatomedin B and thrombospondin type 1	157869	ENSG00000164764	9	-0.18	-0.3	-0.23	-0.61	0.107	-0.1	-0.26	-0	0.042	0.148	0.0098	-0.072	0.0462	0.2765	-0.064	0.253	-0.127	-0.017	-0.03	0.448	0.174	0.371	0.24	0.199	0.299	-0.26	-0.02	0.081497	-0.32904	-0.221191555	-0.107851195
3019	Q8WWB7	GLMP	glycosylated lysosomal membrane protein	112770	ENSG00000198715	4	-0.29	0.268	-0.21	0.091	-0.95	0.124	0.142	-0.17	0.357	0.2551	-0.18	-0.042	0.3183	-0.201	0.0401	0.2649	0.7119	0.18	0.138	-0.33	-0.71	-0.39	-0.61	0.24	-0.11	0.264	-0.08	0.303241	0.105189	-0.220441003	0.32562987
361	A2ACR1	PSMB9	proteasome subunit beta 9	5698	ENSG00000240065	8	-0.07	-0.29	0.041	-0.64	-0.32	-0.52	0.356	-0.3	0.053	0.009	-0.2	0.1281	0.021	-0.076	-0.304	0.5238	0.0206	0.1269	0.149	0.176	0.23	0.978	0.171	-0.06	0.355	-0.12	-0.01	0.145113	-0.39508	-0.214995547	-0.180081755
2400	Q13347	EIF3I	eukaryotic translation initiation factor 3 subu	8668	ENSG00000084623	11	0.146	0.09	-0.1	-0.06	0.158	-0.24	0.181	0.12	0.213	0.0082	0.0306	0.114	0.0819	-0.033	0.184	0.0689	0.1467	0.104	-0.14	-0.63	-0.12	-0.26	-0.19	0.015	0.093	-0.02	0.247	0.187725	0.168114	-0.023001652	0.191115409
1100	O15551	CLDN3	claudin 3	1365	ENSG00000165215	5	-0.24	-0.13	-0.47	-0.02	-0.46	0.036	-0.69	-0.94	-0.67	-0.094	0.0625	0.4286	-0.657	-0.537	-0.463	-0.894	-0.654	-0.44	-0.59	0.387	0.893	1.552	-0.49	0.087	-0.18	-0.71	-0.86	0.408755	-0.40691	-0.035717541	-0.371191503
3002	Q8WUD1	RAB2B	RAB2B, member RAS oncogene family	84932	ENSG00000129472	15	0.179	0.206	0.336	0.122	-0.25	-0.3	-0.03	0.037	0.005	0.3367	0.6075	0.1284	0.2355	-0.275	0.0975	0.1189	0.1779	-0.097	0.144	0.459	0.34	-0.35	-0.47	0.115	-0.04	0.062	0.083	0.682564	-0.00278	-0.113537164	0.110759274
3190	Q96S96	PEBP4	phosphatidylethanolamine binding protein 4	157310	ENSG00000134020	6	0.177	-0.22	-0.2	-0.03	-1.28	-1.33	-0.38	0.464	0.014	0.404	0.4962	0.9072	-0.789	-1.081	-0.702	0.46	0.2937	0.0089	-0.87	-1.27	-0.28	-1.15	0.191	-0.18	-0.15	0.672	-0.59	0.547853	0.093649	-0.308627397	0.402276217
557	D6R9I9	ABCE1	ATP binding cassette subfamily E member	6059	ENSG00000164163	11	0.004	-0.08	-0	-0.08	-0.22	-0.22	0.184	0.046	0.185	0.0123	-0.095	0.0689	-0.325	0.2091	-0.256	0.1196	0.1977	0.1589	-0.11	-0.62	-0.25	-0.54	-0.19	-0.04	0.187	-0.06	0.127	0.341818	0.146185	-0.03044163	0.176626215
2440	Q14055	COL9A2	collagen type IX alpha 2 chain	1298	ENSG00000049089	5	-0.22	-0.25	0.741	-0.96	-0.36	1.914	-0.11	-0.46	-0.02	-0.349	0.036	-0.485	-0.767	0.0005	-0.381	-0.642	-0.323	-0.077	0.343	0.149	0.522	-1.15	-0.07	-0.34	0.623	0.043	-0.15	0.525862	0.034733	0.362938567	-0.328205081
2579	Q3MIR4	TMEM30B	transmembrane protein 30B	161291	ENSG00000182107	5	0.541	0.626	0.35	0.118	-0.11	-0.36	0.131	0.286	0.184	0.3206	0.4237	0.5486	0.0915	-0.125	-0.163	0.2678	0.2414	0.4158	0.189	0.276	0.472	-0.63	-0.55	-0.25	-0.01	0.328	0.358	0.524828	0.17514	-0.029424551	0.20456452
3706	Q9Y275	TNFSF13	TNF superfamily member 13b	10673	ENSG00000102524	1							-0.42	-0.3	-0.12							-0.111	0.449	-0.422							-0.53	-0.13	-0.13	0.673029	-0.01594	-0.250966117	0.235028488
2376	Q13085	ACACA	acetyl-CoA carboxylase alpha	31	ENSG00000275176	19	0.089	0.028	-0.04	-0.27	0.212	-0.38	-0.01	-0.03	0.235	0.0682	-0.044	0.0551	0.1141	-0.022	-0.171	0.1593	0.2984	0.3453	-0.22	-0.31	-0.31	-0.01	0.179	0.069	0.088	0.069	0.245	0.540049	0.003055	-0.107951148	0.111006235
2874	Q8IZ83	ALDH16A1	aldehyde dehydrogenase 16 family member	126133	ENSG00000161618	9	0.085	-0.26	-0.02	-0.39	-0.08	0.256	-0.11	0.107	0.009	-0.033	0.1375	0.1078	-0.278	0.2578	0.1782	0.1644	-0.042	0.1518	-0.04	-0.15	0.135	-0.03	0.411	-0.25	0.394	0.068	-0.06	0.563811	-0.09703	-0.11629274	0.0192582
1964	P48059	LIMS1	LIM zinc finger domain containing 1	3987	ENSG00000169756	14	0.098	0.2	-0.08	-0.09	0.011	-0.38	-0.4	0.527	-0.12	0.1919	0.1292	0.0924	0.0517	0.3622	0.0688	-0.029	-0.037	-0.087	-0.07	-0.32	-0.03	-0.32	0.046	0.21	-0.02	-0.04	-0.05	0.465089	0.03963	-0.108158852	0.147788369
2115	P55735	SEC13	SEC13 homolog, nuclear pore and COPII c	6396	ENSG00000157020	8	0.03	0.063	0.004				-0.02	0.031	0.205	0.1409	0.1261	0.0367				0.0673	0.0856	0.2265	-0.3	-0.47	-0.14				0.241	-0.01	0.269	0.400327	0.119239	-0.061884673	0.181123741
3029	Q92484	SMPDL3A	sphingomyelin phosphodiesterase acid like	10924	ENSG00000172594	7	-0.17	-0.44	-0.07	-0.29	-0.9	-0.27	-0.49	0.201	0.081	0.6224	-0.022	0.2759	-0.504	-0.339	-0.895	0.1055	-0.25	-0.522	-0.7	-0.13	-0.23	-0.93	-0.26	-0.61	0.566	0.866	-0.48	0.94664	-0.04762	-0.090226167	0.042603683
138	F2Z357	RAP1GAP	RAP1 GTPase activating protein	5909	ENSG00000076864	5	-0.04	-0.18	-0.29	0.03	0.094	-0.17	0.138	-0.06	0.289	-0.001	-0.076	-0.069	0.2512	0.207	0.2053	-0.021	0.2052	0.2987	-0.25	-0.61	-0.2	0.092	0.01	0.134	-0.04	0.124	0.382	0.43878	0.018683	-0.132395372	0.151078422
2986	Q8TDX6	CSGALNA	chondroitin sulfate N-acetylgalactosaminyltra	55790	ENSG00000147408	11	-0.21	0.317	-0.27	-0.12	0.22	0.118	-0.07	0.117	-0.04	-0.085	0.1484	0.0912	0.0687	-0.009	-0.255	0.2162	-0.093	0.1941	-0.34	-0.33	-0.11	-0.38	-0.38	-0.11	0.571	-0.06	0.077	0.501112	0.124261	-0.025015324	0.149276193
2600	Q58FF8	HSP90AB	heat shock protein 90 alpha family class B m	391634	ENSG00000205940	18	0.159	-0.12	-0.08	-0.08	0.029	-0.39	-0.18	-0.16	0.041	0.1481	0.1109	-0.069	0.2011	-0.114	0.1564	-0.113	0.0726	-0.017	0.016	-0.27	-0.06	-0.65	-0.24	0.003	0.04	-0.18	0.057	0.227319	0.056811	-0.127245743	0.184057217
2775	Q7RTP6	MICAL3	microtubule associated monooxygenase, cal	57553	ENSG00000243156	1	0.255	-1.26	-0.41	0.355	-0.11	0.159	-0.17	0.931	0.625	-0.152	-0.455	1.1394	-0.498	-0.564	-0.393	0.123	-0.582	-0.464	-0.29	0.61	-0.41	-0.24	-0.2	-0.83	-0.69	-0.05	-0.83	0.520982	0.368417	0.247400048	0.121017389
2604	Q5GFL6	VWA2	von Willebrand factor A domain containing 2	340706	ENSG00000165816	11	-0.45	-0.5	0.405	-0.46	0.06	-0.18	-0.32	0.03	0.553	0.054	0.0029	-0.03	-0.302	0.1402	-0.238	-0.034	0.5056	-0.048	-0.25	0.131	0.371	-0.33	-0.4	0.097	0.156	-0.33	-0.31	0.806802	0.001691	-0.100863531	0.102554805
3081	Q96A22	C11orf52	chromosome 11 open reading frame 52	91894	ENSG00000149300	6	-0.23	-0.19	-0.2	0.4	-0.45	0.055	0.072	0.232	-0.14	0.0148	0.3352	0.1461	-0.12	0.2051	0.2466	0.1707	-0.126	0.1341	-0.25	0.319	0.026	-0.25	-0.07	-0.15	0.15	-0.07	0.186	0.414198	-0.03908	-0.161312962	0.122228045
2503	Q15172	PPP2R5A	protein phosphatase 2 regulatory subunit B'	5525	ENSG00000066027	12	0.152	0.18	-0.02	-0.05	-0.15	-0.31	0.054	0.003	0.125	-0.08	-0.002	-0.004	-0.075	-0.101	0.1732	-0.042	0.1796	0.0449	-0.3	-0.62	-0	-0.15	-0.22	-0.04	0.029	-0.09	0.153	0.30349	0.133101	-0.013367472	0.1464683
2233	P63167	DYNLL1	dynein light chain LC8-type 1	8655	ENSG00000088986	8	-0.01	-0.04	-0.08	0.39	0.502	-0.16	-0.48	0.009	-0.13	0.0184	0.2681	-0.263	-0.017	-0.119	0.3022	-0.513	0.2381	-0.591	-0.04	-0.19	0.169	-0.81	-0.34	0.064	-0.22	0.007	-0.15	0.638926	0.167171	0.075009074	0.092161892
3570	Q9P2G1	ANKIB1	ankyrin repeat and IBR domain containing 1	54467	ENSG00000001629	17	-0.03	0.171	0.026	-0.02	0.031	-0.46	-0.05	0.219	0.144	0.2278	0.1427	-0.167	0.1635	-0.17	-0.064	0.0316	0.0565	0.0113	-0.31	-0.9	-0.05	-0.6	-0.4	0.095	0.087	0.082	0.067	0.238191	0.218775	-0.022259377	0.241034265
1590	P13686	ACP5	acid phosphatase 5, tartrate resistant	54	ENSG00000102575	6	-0.08	-0.05	-0.14	-0.12	-0.18	-0.58	-0.11	0.197	0.039	0.5469	0.113	0.295	0.137	0.1233	-0.682	0.4532	0.2473	0.0807	-0.3	-0.01	-0.02	-0.98	-0.97	0.139	0.106	0.013	0.126	0.245769	0.096542	-0.260154853	0.356697193
1081	O15212	PFDN6	prefoldin subunit 6	10471	ENSG00000204220	10	0.113	0.221	-0.04	-0.04	0.029	-0.46	0.168	0.101	0.275	-0.018	-0.112	-0.022	-0.213	-0.077	0.1379	0.2428	0.2089	0.0658	-0.05	-0.23	-0.16	-0.4	-0.41	-0.07	0.04	0.098	0.145	0.347564	0.155257	0.017586847	0.137670376
3100	Q96BW5	PTER	phosphotriesterase related	9317	ENSG00000165983	11	0.372	0.129	0.295	-0.43	0.17	-0.54	0.078	0.23	0.28	0.5658	0.0526	0.4759	-0.204	0.0408	-0.103	0.1944	0.2608	0.262	0.247	-0.36	-0.15	-0.77	-0.43	0.048	0.005	0.065	0.047	0.234148	0.209463	-0.107032684	0.316495959
591	E5RJ40	SLC39A14	solute carrier family 39 member 14	23516	ENSG00000104635	1	-0.7	-0.57	-0.11	-0.68	-0.14	0.399	0.175	0.449	0.41	0.251	0.8989	-0.538	-0.478	0.0906	-0.083	0.0619	-0.121	-0.2	-0.18	0.794	0.803	-0.59	0.525	-0.02	0.871	0.146	-0.29	0.517565	-0.31478	-0.072218218	-0.242562472
633	E9PDF6	MYO1B	myosin IB	4430	ENSG00000128641	20	-0.13	0.651	-0.32	0.209	0.014	0.073	-0.31	0.141	0.276	-0.154	-0.22	0.1622	-0.02	-0.383	0.4214	0.4543	0.0372	0.3633	-0.53	-0.38	-0.28	-0.22	-0.09	-0.36	-0.12	0.04	0.196	0.225406	0.260855	-0.006424021	0.267278922
1790	P29400	COL4A5	collagen type IV alpha 5 chain	1287	ENSG00000188153	5	0.004	-0.38	-0.03	-0.48	-0.54	-0.1	0.148	-0.05	-0.02	-0.081	0.119	-0.135	-0.574	-0.163	-0.006	-0.336	-0.337	-0.183	0.297	0.01	0.498	-0.5	0.021	0.091	0.479	-0.14	0.214	0.163478	-0.26818	0.028454302	-0.296638138
974	M0QXB4	COPE	coatomer protein complex subunit epsilon	11316	ENSG00000105669	8	0.034	0.034	-0.13	-0.09	0.059	-0.37	-0.13	0.028	0.158	-0.034	-0.27	-0.016	0.1039	-0.12	-0.003	0.0866	0.1159	0.1476	-0.12	-0.61	-0.15	-0.38	-0.2	0.019	0.056	0.011	0.308	0.575486	0.072664	-0.046423836	0.119088046
329	Q9Y5P4	CERT1;CC	ceramide transporter 1;collagen type IV alph	10087	ENSG00000113163	13	0.224	-0.04	-0.34	-0.13	-0.16	-0.37	0.124	0.235	0.194	0.1556	-0.141	0.0385	-0.141	-0.023	0.0679	0.3932	0.0879	0.2823	0.148	-0.3	-0.11	-0.41	-0.27	-0.03	0.214	0.093	0.293	0.594623	0.012113	-0.109785612	0.121898554
2811	Q86V21	AACS	acetoacetyl-CoA synthetase	65985	ENSG00000081760	14	0.085	0.117	0.141	-0.24	-0.04	-0.2	-0.06	0.017	0.077	0.0528	-0.068	0.0929	-0.032	0.0387	-0.113	-0.05	-0.035	0.281	-0.21	-0.8	-0.13	-0.37	0.109	0.188	-0.21	-0.05	0.186	0.39266	0.131551	-0.030249049	0.161800382
517	C9JGI3	TYMP	thymidine phosphorylase	1890	ENSG00000025708	11	-0.21	-0.32	-0.18	0.023	-0.22	-0.22	0.27	0.097	0.062	-0.121	0.0266	-0.034	-0.049	0.0014	-0.356	0.249	0.2244	0.2993	-0.25	-0.4	0.932	0.025	-0.12	0.216	-0.03	0.36	0.039	0.603789	-0.1631	-0.104121301	-0.058978473
1878	P36915	GNL1	G protein nucleolar 1 (putative)	2794	ENSG00000204590	10	0.251	0.62	0.113	-0.11	-0.27	-0.32	-0.07	0.153	0.076	0.2053	0.2242	0.1126	-0.117	-0.117	-0.014	-0.008	-0.044	0.0507	-0.08	-0.54	-0.05	-0.34	-0.22	0.153	0.114	0.058	0.071	0.509231	0.14273	0.016685733	0.12604433

798	H0Y390	MACF1	microtubule actin crosslinking factor 1	643314;2349	ENSG00000127603	1							-0.2	0.107	0.044							0.0521	0.4037	0.0982							0.28	-0.09	-0.15	0.592859	-0.03012	-0.201904431	0.171782471
3300	Q9BXJ1	C1QTNF1	C1q and TNF related 1	114897	ENSG00000173918	8	-0.25	-0.33	-0.02	-0.84	-0.18	0.129	-0.38	-0.33	-0.3	0.1496	0.313	-0.148	-0.743	0.4737	-0.357	-0.718	-0.787	-0.501	0.161	0.22	0.451	-0.26	0.236	0.368	0.678	-0.1	-0.77	0.228018	-0.3882	-0.021323028	-0.366875931
688	E9PQY3	ACP2	acid phosphatase 2, lysosomal	53	ENSG00000134575	5	-0.39	0.414	0.076	-0.18	-0.22	-0.18	0.321	0.402	0.449	0.042	-0.33	0.1924	-0.153	0.1254	-0.177	0.4588	0.0575	0.2748	8E-04	0.644	0.208	-0.2	0.02	-0.11	0.386	0.287	0.307	0.75398	-0.09543	0.021770219	-0.117201228
3071	Q93100	PHKB	phosphorylase kinase regulatory subunit bet	5257	ENSG00000102893	17	0.059	0.002	-0.05	-0.12	0.089	-0.43	-0.16	-0.21	0.062	-0.128	-0.223	0.0551	0.0477	-0.136	0.0014	-0.061	0.1067	0.2166	-0.09	-0.6	0.012	-0.08	-0.2	0.105	0.254	-0.06	0.098	0.800823	-0.02297	-0.071246686	0.048275174
985	O00115	DNASE2	deoxyribonuclease 2, lysosomal	1777	ENSG00000105612	4	-0.22	-0.18	-0.1	-0.14	-0.14	-0.12	0.301	0.308	0.445	0.6649	-0.105	0.3878	-0.45	-0.051	-0.669	0.4819	-0.182	0.0974	-0.11	0.005	-0.11	-0.38	0.359	-0.17	0.439	0.626	-0.32	0.992897	-0.02078	-0.002080948	-0.018696247
1739	P24386	CHM	CHM, Rab escort protein 1;CHM Rab escor	1121	ENSG00000188419	12	0.14	0.107	-0.07	0.044	0.007	-0.18	-0.1	-0	0.045	0.0384	0.0716	0.0789	0.1908	-0.079	0.0983	0.1523	0.2926	0.1569	-0.23	-0.96	-0.24	-0.17	-0.13	0.052	0.059	-0.11	0.235	0.147972	0.166275	-0.111964155	0.27823946
1022	O00560	SDCBP	syndecan binding protein	6386	ENSG00000137575	25	-0.17	0.042	-0.01	0.055	0.274	-0.2	-0.55	-0.4	-0.26	0.3925	0.002	0.222	0.0793	-0.316	0.1557	-0.191	-0.019	-0.262	-0.44	-0.23	0.337	0.624	0.219	0.258	-0.36	-0.13	-0.25	0.640147	-0.13647	-0.141457156	0.004991038
1519	P09497	CLTB	clathrin light chain B	1212	ENSG00000175416	6	0.295	0.191	-0.03				-0.05	0.193	0.143	-0.035	-0.039	0.0336				0.1052	0.0796	-0.025	-0.11	-0.63	-0.08				0.159	0.064	0.068	0.334401	0.209869	0.102811944	0.107056801
2831	Q86Z20	CCDC125	coiled-coil domain containing 125	202243	ENSG00000183323	7	0.139	0.052	-0.13	-0.19	-0.3	0.128	0.072	-0.44	-0.24	0.465	-0.233	0.5128	-0.019	0.082	0.0377	0.171	-0.081	-0.046	-0.01	-0.97	-0.51	-0.76	0.03	-0.07	-0.03	-0.24	0.215	0.181461	0.159653	-0.199178474	0.358831456
931	J3QL71	SCRN2	secernin 2	90507	ENSG00000141295	9	-0.26	0.072	0.045	-0.06	-0.24	-0.18	-0.18	0.221	0.285	0.034	0.0176	0.1507	-0.155	-0.041	0.0704	-0.064	0.2136	0.0818	0.055	-0.73	-0.21	-0.43	-0.23	-0.06	0.212	0.052	0.228	0.495404	0.090736	-0.066912133	0.157648354
3778	Q9Y5Y6	ST14	suppression of tumorigenicity 14	6768	ENSG00000149418	7	-0.14	-0.3	-0.22	-0.13	-0.44	0.303	-0.11	-0.06	-0.02	0.2488	0.5622	0.1514	-0.406	0.404	0.1031	0.3124	-0.55	-0.159	-0.06	0.005	0.046	0.632	0.087	-0.03	0.552	0.073	-0.22	0.352976	-0.24391	-0.198425924	-0.045481454
573	D6RF35	GC	GC vitamin D binding protein	2638	ENSG00000145321	25	-0.53	0.44	0.226	-0.01	-2.21	1.045	0.297	-1.37	-1.23	0.1375	0.2831	0.145	-1.535	0.1437	-1.753	0.0067	0.1566	0.9596	-1.36	-0.01	1.795	-1.77	0.985	0.227	-1.19	0.497	-1.69	0.944538	-0.09211	-0.209440506	0.117334768
349	A0AV96	RBM47	RNA binding motif protein 47	54502	ENSG00000163694	8	0.415	-0.1	-0.24	-0.01	0.199	-0.33	-0.12	0.162	0.034	-0.189	0.0865	0.3364	0.1587	-0.036	0.0154	0.2624	-0.306	0.2831	-0.17	-0.86	-0.39	-0.57	0.019	0.051	0.484	0.172	0.215	0.590075	0.116135	-0.067311877	0.183446919
910	J3KNF4	CCS	copper chaperone for superoxide dismutase	9973	ENSG00000173992	8	0.1	0.296	-0.12	-0.05	0.158	-0.38	0.054	0.036	0.19	-0.108	-0.016	-0.047	-0.13	-0.241	0.0227	0.0519	0.2191	0.0123	-0.4	-1.46	-0.49	-0.72	-0.57	-0.07	0.019	-0.05	0.364	0.15015	0.405638	0.057623427	0.348014867
1909	P41226	UBA7	ubiquitin like modifier activating enzyme 7	7318	ENSG00000182179	17	-0.1	0.008	-0.21	-0.08	0.064	-0.29	-0.04	-0.29	0.331	-0.136	-0.059	0.1672	0.2033	0.1028	-0.111	-0.095	0.1041	0.1535	-0.29	-0.96	0.014	-0.07	0.172	0.231	0.071	-0.07	0.097	0.649033	0.022812	-0.103964568	0.126776725
3672	Q9UNE7	STUB1	STIP1 homology and U-box containing prote	10273	ENSG00000103266	10	0.01	0.186	0.084	-0.31	-0.26	-0.37	-0.1	-0.02	0.26	-0.024	-0.069	0.1335	0.0526	-0.353	0.1929	-0.084	0.0365	0.0221	-0.31	-0.43	-0.31	-0.53	-0.51	0.026	0.18	0.01	0.079	0.348029	0.141075	-0.048370405	0.18944532
3554	Q9NZP8	C1RL	complement C1r subcomponent like	51279	ENSG00000139178	7	-0.18	-0.07	-0.12	0.009	-0.08	-0.23	-0.23	0.3	0.037	-0.14	0.38	0.1219	-0.227	0.1287	-0.15	0.2925	0.2231	0.3411	-0.18	-0.1	0.155	-0.06	-0.15	-0.07	0.052	0.032	0.025	0.248513	-0.02707	-0.169230744	0.142164865
3012	Q8WVJ2	NUDCD2	NudC domain containing 2	134492	ENSG00000170584	6	0.019	0.118	-0.04	-0.14	-0.16	-0.27	0.14	0.239	0.306	0.2372	0.0189	0.0528	-0.131	-0.235	0.1479	0.3009	0.294	0.0883	0.038	-0.47	-0.23	-0.7	-0.15	-0.07	0.174	0.295	0.21	0.437461	0.123693	-0.062095811	0.185789071
2693	Q6MZW2	FSTL4	follistatin like 4	23105	ENSG00000053108	6	0.447	0.032	-0.73	-0.47	0.069	-0.08	-0.41	0.721	-0.04	0.3268	-0.184	-0.458	-0.711	-0.385	0.5514	0.0926	0.1262	0.3933	-0.26	0.233	0.618	0.56	0.002	0.73	-0.13	0.133	-0.52	0.686135	-0.20216	-0.023949746	-0.17820693
3520	Q9NVJ2	ARL8B	ADP ribosylation factor like GTPase 8B	55207	ENSG00000134108	5	-0.08	-0.26	0.036	-0.03	0.031	-0.12	-0.14	0.218	0.15	0.1576	-0.245	-0.029	0.055	0.0011	0.2703	0.2928	-0.036	0.4711	-0.48	-0.38	0.255	0.081	0.136	0.097	0.223	0.02	0.407	0.636919	-0.06179	-0.125814472	0.064026989
2203	P62750	RPL23A	ribosomal protein L23a	6147	ENSG00000198242	7	0.283	-0.06	0.043	-0.22	0.29	-0.29	-0.02	-0.38	-0	-0.23	0.097	-0.206	-0.279	-0.162	0.0326	-0.06	0.3166	-0.264	0.053	-0.45	0.129	-0.49	-0.11	-0.12	0.189	-0.15	-0.27	0.768796	0.095584	0.044112533	0.051471166
2306	Q02878	AC004086	ribosomal protein L6	6128	ENSG00000089009	9	0.053	-0.41	0.23	-0.18	0.393	-0.26	0.098	-0.08	0.005	-0.168	-0.092	-0.232	-0.159	-0.246	-0.03	-0.166	-0.019	-0.047	0.383	-0.21	0.367	-0.2	-0.23	-0.13	0.131	-0.22	-0.05	0.582744	-0.00071	0.110875314	-0.111582966
569	D6RD47	RPS23	ribosomal protein S23	6228	ENSG00000186468	5	-0	-0.15	0.147	-0.26	0.114	-0.14	-0.03	-0.33	0.126	0.5641	0.3018	-0.382	-0.338	0.0134	-0.038	-0.193	-0.072	-0.201	0.067	0.506	0.445	-0.2	-0.12	-0.22	0.055	-0.29	-0.06	0.87713	-0.07634	-0.019163892	-0.05717825
907	J3KN36	NOMO3	NODAL modulator 3	408050	ENSG00000103226	21	-0.11	-0.52	-0.26	-0.47	0.002	-0.1	-0.44	0.068	0.201	0.0318	0.1564	-0.254	-0.476	-0.078	-0.117	0.3377	0.048	0.2987	-0.15	0.323	0.113	-0.02	-0.15	-0.13	0.339	-0.16	-0.16	0.372311	-0.1821	-0.175068539	-0.007035567
3584	Q9UBQ6	EXTL2	exostosin like glycosyltransferase 2	2135	ENSG00000162694	5	-0.28	-0.25	-0	-0.26	0.004	-0.38	-0.17	0.057	0.158	0.3155	0.0544	0.1766	-0.05	-0.166	-0.069	0.1402	0.1099	0.0452	-0.25	0.116	0.161	-0.23	-0.17	-0.13	0.523	0.185	-0.22	0.315045	-0.1233	-0.186568264	0.063266184
2466	Q14623	IHH	Indian hedgehog signaling molecule;indian h	3549	ENSG00000163501	4	-0.09	-0.66	0.318	-0.32	-0.37	0.515	-0.05	-0.42	0.065	-0.34	-0.141	-0.756	-0.601	0.0546	-0.46	-0.199	0.1084	-0.233	1.067	0.039	0.57	-0.1	0.014	-0.28	0.265	-0.03	-0.51	0.2485	-0.22513	0.174151869	-0.399282186
3437	Q9HBL8	NMRAL1	NmrA like redox sensor 1	57407	ENSG00000274684	8	0.076	0.193	0.166	-0.1	-0.33	-0.39	0.079	-0.44	0.133	0.0216	0.1353	-0.133	0.0803	-0.364	0.2166	-0.225	0.2743	0.2857	-0.15	-1.08	-0.27	-0.43	-0.19	-0.04	-0.08	-0.27	0.333	0.309203	0.174567	-0.100516305	0.275082884
3799	Q9Y6W3	CAPN7	calpain 7	23473	ENSG00000131375	16	-0.27	0.115	0.065	0.095	-0.04	-0.32	0.062	0.065	0.02	0.1468	0.0001	0.1811	0.0819	-0.045	0.0051	0.0164	0.0341	0.0359	-0.14	-0.13	-0.08	-0.31	-0.25	-0.05	0.032	0.034	0.401	0.509288	0.031874	-0.073718847	0.105593098
107	A0A087X1H	ACAP2	ArfGAP with coiled-coil, ankyrin repeat and	23527	ENSG00000114331	11	0.161	0.225	0.021	-0.24	-0.05	-0.5	0.053	-0.03	0.17	0.084	0.3657	0.1008	-0.005	-0.238	0.0896	0.1735	0.1557	-0.044	-0.31	-0.97	-0	-0.46	-0.56	-0.03	0.04	0.14	0.277	0.259586	0.186556	-0.097297708	0.283854025
3116	Q96DT6	ATG4C	autophagy related 4C cysteine peptidase	84938	ENSG00000125703	5	0.019	0.423	-0.23				0.058	-0.01	0.244	-0.037	-0.019	0.1569				0.0167	0.2422	0.1204	-0.74	-0.6	-0.21				0.008	-0.11	0.209	0.203287	0.326008	0.005455983	0.320551577
1961	P47914	RPL29	ribosomal protein L29	6159	ENSG00000162244	4	-0	-1.02	0.061	-0.64	0.156	-0.07	1.265	0.717	0.854	-0.848	-0.23	-0.438	-0.334	-0.306	-0.528	0.4741	0.3577	0.6192	-0.11	0.007	0.239	-0.07	-0.01	-0.46	0.7	0.141	0.603	0.627513	0.031614	0.28338972	-0.251776185
3789	Q9Y6B6	SAR1B	secretion associated Ras related GTPase 1	51128	ENSG00000152700	7	-0.14	-0.06	-0.05	-0.07	0.062	-0.34	-0.2	-0.1	0.08	0.0248	-0.077	-0.18	0.1253	-0.382	-0.013	-0.09	0.102	-0.013	-0.04	-0.02	0.209	-0.44	-0.33	0.009	-0.04	-0.13	0.022	0.926414	-0.00481	-0.033648678	0.028838265
1302	O95721	SNAP29	synaptosome associated protein 29	9342	ENSG00000099940	11	-0.21	0.12	-0.05	-0.08	-0.22	-0.34	0.163	-0.09	0.104	-0.043	0.021	-0.15	-0.18	0.0359	0.3049	0.0357	0.1231	0.1341	-0.33	-0.62	-0.21	-0.48	-0.45	-0.11	-0.02	-0.12	0.002	0.092116	0.192202	-0.099207476	0.291409792
559	D6RA31	SNCA	synuclein alpha	6622	ENSG00000145335	5	-0.02	0.515	0.096	0.014	0.226	-0.02	0.11	-0.43	-0.2	0.2365	0.0021	0.123	0.1644	-0.038	0.0222	0.1572	0.3785	-0.345	-0.5	-0.29	-0.07	-0.59	-0.37	-0.19	-0.07	-0.04	0.072	0.126488	0.260782	-0.044983963	0.305765519
824	H0YDU8	PPP5C	protein phosphatase 5 catalytic subunit	5536	ENSG00000011485	13	0.157	0.08	0.001	0.003	0.004	-0.37	0	-0.01	0.242	-0.103	0.0447	0.0222	-0.083	-0.084	0.0482	0.1098	0.1073	0.0734	-0.27	-0.49	-0.07	-0.62	-0.35	-0.05	-0.02	0.124	0.208	0.226729	0.180725	-0.003754061	0.184479198
3310	Q9BY32	ITPA	inosine triphosphatase	3704	ENSG00000125877	7	0.089	-0.24	-0.45	-0.29	0	-0.47	-0.29	-0.05	0.287	0.2237	0.1956	-0.026	0.0917	-0.479	0.281	-0.013	0.0766	0.0146	0.041	-0.45	0.162	0.048	-0.69	0.312	0.166	0.145	0.217	0.450988	-0.15338	-0.19931691	0.045940788
2198	P62495	ETF1	eukaryotic translation termination factor 1	2107	ENSG00000120705	11	0.089	-0.05	-0.22	0.117	0.207	-0.38	-0.18	-0.13	-0.02	-0.024	-0.096	-0.11	0.0883	-0.173	0.0596	-0.172	0.0537	-0.144	-0.27	-0.56	-0.13	-0.37	-0.14	0.214	-0.15	-0.09	0.12	0.615597	0.088162	-0.007286549	0.095448408
654	E9PHY8	MROH1	maestro heat like repeat family member 1	727957	ENSG00000179832	14	0.191	0.051	-0.06	0.151	0.266	-0.38	0.142	-0.01	0.199	0.0173	0.0569	0.0896	0.0938	-0.261	0.0172	0.1259	0.0668	0.0956	-0.22	-0.52	-0.36	-0.47	-0.24	0.08	0.194	0.212	0.448	0.492604	0.157463	0.027893042	0.129569837
3657	Q9ULV0	MYO5B	myosin VB	4645	ENSG00000167306	27	0.057	0.095	-0.14	-0.1	0.055	-0.55	0.01	-0.14	0.101	0.1577	0.0381	0.2862	0.0368	-0.215	-0.046	0.0469	0.1839	0.2493	-0.17	-0.63	-0.27	-0.61	-0.21	0.007	-0.07	0.413	-0.03	0.226899	0.10771	-0.149865304	0.257575129
31	A3KFI3	NBL1	NBL1, DAN family BMP antagonist	4681;10053	ENSG00000158747	2	0.138	-0.04	0.33	0.319	-0.69	-0.04	-0.62	-0.43	0.129	0.4748	0.3324	0.195	0.032	-0.164	-0.532	1.0128	-0.508	-0.3	-0.36	-0.48	-0.25	-0.26	0.176	0.101	-0.41	0.46	-0.99	0.555804	0.1226	-0.161008346	0.283608199
2451	Q14257	RCN2	reticulocalbin 2	5955	ENSG00000117906	7	-0.18	-0.22	0.051	0.364	0.572	-0.03	0.271	0.325	-0.02	-0.054	-0.13	-0.166	-0.245	-0.115	-0.216	0.0105	-0.367	-0.112	-0.23	1.078	-0.07	-0.33	0.046	0.025	0.107	-0.17	-0.34	0.310317	0.113696	0.280881848	-0.167185798
1118	O43504	LAMTOR5	late endosomal/lysosomal adaptor, MAPK a	10542	ENSG00000134248	5	-0.02	0.491	0.007	-0.33	-0.3	-0.66	-0.19	-0.24	-0.16	-5E-04	0.3357	-0.068	0.1115	-0.2	0.179	-0.002	0.1846	-0.106	-0.22	-1.28	-0.27	-0.75	-0.38	0.06	-0.17	-0.03	0.036	0.193315	0.178069	-0.204086362	0.382155409
2202	P62745	RHOB	ras homolog family member B	388	ENSG00000143878	8	-0.15	0.319	0.061	-0.29	-0.05	0.033	0.035	-0.02	0.169	0.0029	-0.185	-0.024	-0.172	-0.048	0.1763	-0.032	0.1633	0.1178	-0.14	-0.03	-0.34	-0.1	-0	-0.11	0.115	-0.05	0.262	0.824276	0.055245	0.01169475	0.043550354
3637	Q9UJH8	METRN	meteorin, glial cell differentiation regulator	79006	ENSG00000103260	5	-0.21	-0.6	-0.33	-0.38	-0.16	0.336	0.142	-0.05	0.058	-0.137	-0.111	-0.342	-0.46	0.2396	-0.256	-0.274	-0.298	-0.164	0.181	0.292	0.098	-0.31	0.243	-0.1	0.625	0.007	-0.36	0.236436	-0.2071	0.067859725	-0.274957056
268	A0A1B0GTV	TJP2	tight junction protein 2	9414	ENSG00000119139	10	0.198	0.381	0.18	-0.12	-0.11	-0.54	0.11	0.196	0.315	0.079	-0.019	0.2157	-0.218	0.0136	0.2698	0.0351	0.2066	0.1582	-0.2	-0.6	-0.14	-0.71	-0.23	-0.06	0.205	0.131	0.191	0.267104	0.224746	-0.015440179	0.240186266
3376	Q9H336	CRISPLD1	cysteine rich secretory protein LCCL domain	83690	ENSG00000121005	8	-0.4	-0.5	0.015	-0.19	0.227	-0.4	0.159	0.293	-0.13	0.6846	-0.194	0.0253	-0.303	-0.055	-0.307	0.8942	-0.05	-0.176	-0.25	-0	-0.19	-0.23	-0.16	-0.05	0.7	0.543	-0.34	0.751947	-0.10494	-0.161984818	0.057041645
146	A0A0A0MRL	CASP7	caspase 7	840	ENSG00000165806	6	-0.1	0.117	-0.21	-0.17	-0.2	-0.36	0.383	0.066	0.238	0.1656	0.0189	-0.006	0.0844	0.0295	0.0832	0.2726	0.1178	0.182	-0.16	-0.21	0.104	-0.11	-0.12	-0.07	0.15	-0.03	0.242	0.362041	-0.00244	-0.130426253	0.127987191
2448	Q14232	EIF2B1	eukaryotic translation initiation factor 2B sub	1967	ENSG00000111361	6	0.136	0.222	0.019	0.069	0.077	-0.23	-0.03	-0.12	0.148	0.1917	0.12	0.0661	0.119	-0.129	-0.006	-0.007	0.2423	0.1504	-0.08	-0.66	-0.03	-0.27	-0.4	0.045	-0.09	-0.07	0.183	0.138213	0.184	-0.051107883	0.235107388
1305	O95750	FGF19	fibroblast growth factor 19	9965	ENSG00000162344	3							1.48	-0.43	0.105							1.3772	-0.75	-0.244							0.058	-0.15	-0.34	0.840486	0.530466	0.256584518	0.273881418
1127	O43617	TRAPPC3	trafficking protein particle complex 3	27095	ENSG00000054116	6	0.138	0.186	-0.02	-0.08	-0.1	-0.28	0.019	-0.13	0.054	-0.028	-0.129	-0.01	-0.153	-0.191	0.0266	-0.013	0.0866	0.0392	-0.21	-1.23	-0.01	-0.22	-0.3	0.093	-0.03	0.05	0.191	0.525704	0.162	0.018054596	0.143945347
2124	P58335	ANTXR2	ANTXR cell adhesion molecule 2	118429	ENSG00000163297	11	0.08	-0.1	0.082	0.042	0.06	-0.45	-0.33	-0.03	-0.08	-0.015	0.2158	0.0872	0.2922	-0.006	0.0092	0.0922	0.278	0.0749	-0.02	-0.5	-0.21	-0.52	-0.06	0.153	0.261	-0.12	0.091	0.195195	0.020073	-0.196181062	0.216253734
2852	Q8IWU2	LMTK2	lemur tyrosine kinase 2	22853	ENSG00000164715	19	-0.03	0.171	-0.19	-0.18	-0.03	-0.36	0.043	-0	0.052	-0.091	-0.047	0.0987	0.0542	-0.023	0.0985	0.3386	0.1385	0.409	-0.28	-0.2	-0.27	-0.55	-0.26	-0.17	-0.01	0.134	0.079	0.104034	0.112081	-0.166108262	0.278189581
1857	P35442	THBS2	thrombospondin 2	7058	ENSG00000186340	13	0.05	-0.78	1.839	0.602	0.7	0.313	0.468	1.448	0.125	-0.85	1.5764	-0.53	0.8768	0.1832	0.076	-0.018	-0.604	-0.847	-0.97	-0.43	-0.98	0.167	0.026	-0.18	-0.16	-0.26	-1.02	0.138661	0.953836	0.544859475	0.40897687
2094	P54725	RAD23A	RAD23 homolog A, nucleotide excision repa	5886	ENSG00000179262	10	0.094	0.248	-0.17	0.012	0.315	-0.24	-0.26	-0.08	-0.12	-0.024	0.0163	0.1386	0.0127	-0.03	0.1911	-0.253	-0.08	-0.377	0.325	-0.34	0.358	-0.52	-0.18	-0.02	-0.17	-0.17	-0.08	0.885639	0.06503	0.021993259	0.043037224
2057	P52564	MAP2K6	mitogen-activated protein kinase kinase 6	5608	ENSG00000108984	8	0.306	0.138	0.062	0.037	-0.24	-0.21	0.222	1.09	0.472	0.0674	0.0788	0.1332	-0.064	-0.201	-0.052	0.3653	0.1995	0.1553	-0.05	-0.89	-0.25	-0.23	-0.11	-0.16	0.501	0.499	0.392	0.524183	0.242633	0.133100366	0.109532561
1444	P05981	HPN	hepsin	3249	ENSG00000105707	4	-0.02	0.036	-0.12	-0.09	-0.09	-0.49	-0.49	-0.78	-0.19	-0.126	0.4322	-0.098	-0.089	0.1531	-0.102	-0.568	-0.417	-0.51	-0.33	0.359	0.292	-0.65	-0.38	-0.03	-0.82	-0.49	-0.58	0.759526	0.044486	-0.101018622	0.145504426
2736	Q6UY27	PATE2	prostate and testis expressed 2	399967	ENSG00000196844	6	0.419	-0.64	0.204	0.284	0.345	0.118	-0.08	0.531	0.562	-0.156	-0.139	0.1608	0.0515	-0.204	-0.015	0.0017	-0.644	-0.476	-0.11	0.592	-0.26	0.183	-0.06	-0.23	-0.27	-0.02	-0.69	0.208107	0.2906	0.35178728	-0.061187071
2483	Q14964	RAB39A	RAB39A, member RAS oncogene family	54734	ENSG00000179331	3	-0.34	0.212	0.024	0.313	-0.36	-0.54	0.48	0.211	0.437	-0.273	-0.681	0.0831	-0.088	-0.432	-0.246	0.5021	0.2789	0.2804	-0.51	-1.09	-0.48	-0.76	-0.88	-0.32	-0.05	0.044	0.494	0.226478	0.44231	0.111944576	0.330365498
2217	P62899	RPL31	ribosomal protein L31	6160	ENSG00000071082	7	0.316	-0.05	-0.07	-0.05	-0.37	0.133	0.194	-0.09	0.113	-0.068	0.0403	-0.094	-0.318	-0.034	0.3103	-0.137	0.0132	-0.105	-0.07	-0.74	0.112	-0.26	-0.09	-0.18	0.301	-0.19	-0.08	0.532475	0.147409	0.058460727	0.088947855
3608	Q9UHB9	SRP68	signal recognition particle 68	6730	ENSG00000167881	15	0.005	0.097	0.093	-0.12	0.169	0.054	0.062	-0.04	0.043	0.0778	-0.038	-0.034	0.1147	-0.011	0.231	0.2897	-0.087	0.0935	-0.13	-0.61	-0.04	0.049	0.068	0.117	0.376	-0.03	-0.01	0.639429	0.063843	-0.030497599	0.094340766
2150	P61077	UBE2D3	ubiquitin conjugating enzyme E2 D3	7323	ENSG00000109332	5	0.074	0.256	0.003	0.207	0.125	-0.67	-0.07	0.032	0.041	-0.052	-0.034	0.0699	-0.437	-0.228	-0.061	0.1391	0.1123	0.2632	-0.17	-0.61	-0.2	-0.91	-0.78	-0.44	-0.08	-0.12	0.194	0.146383	0.345198	0.024884013	0.320314087
3723	Q9Y2S6	TMA7	translation machinery associated 7 homolog	51372	ENSG00000232112	2	0.347	0.089	-0.25	0.012	0.247	-0.39	-0.45	-0.39	-0.18	-0.216	-0.181	-0.197	0.1364	-0.143	0.0749	-0.314	0.1813	-0.375	-0.46	-0.58	-0.61	-0.56	-0.42	-0.09	-0.43	-0.13	-0.13	0.153234	0.271256	0.008109546	0.26314627
384	A6NML8	DIAPH2	diaphanous related formin 2	1730	ENSG00000147202	10	0.062	-0.04	-0.13	-0.29	0.242	0.21	-0.16	-0.39	-0.07	0.0343	0.1965	0.1931	-0.012	0.3418	0.2461	0.1215	0.0668	0.0324	-0.34	-0.02	-0.37	-0.23	0.432	0.018	0.049	0.044	-0.01	0.217641	-0.01366	-0.19776536	0.184101146
449	F8WE86	TCN2	transcobalamin 2	6948	ENSG00000185339	10	-0.26	-0.58	-0.47	-0.44	0.03	-0.37	-0.37	0.294	0.083	0.0156	-0.003	-0.047	0.1013	-0.25	-0.081	0.1248	-0.261	0.4791	0.043	0.239	0.373	-0.05	-0.06	-0.23	0.335	0.678	0.329	0.102956	-0.41545	-0.239975844	-0.175475327
3610	Q9UHD1	CHORDC1	cysteine and histidine rich domain containing	26973	ENSG00000110172	6	0.374	0.229	0.077	0.106	0.368	-0.31	-0.02	-0.07	0.041	0.2646	0.3521	0.167	0.2224	0.0733	0.1845	-0.074	0.0796	-0.03	-0.03	-0.38	0.194	-0.28	-0.11	0.171	0.04	-0.12	0.062	0.249213	0.137162	-0.050048658	0.187210486
1190	O75116	ROCK2	Rho associated coiled-coil containing protei	9475	ENSG00000134318	21	-0.11	0.033	-0.2	-0.09	0.026	-0.46	0.181	0.005	0.277	-0.011	0.4419	-0.094	-0.061	-0.153	0.2565	-0.032	0.0547	-0.023	-0.18	-0.4	1.093	-0.52	-0.34	0.098	0.352	-0.01	-0.05	0.918737	-0.04106	-0.079532975	0.038471442
3146	Q96IG2	FBXL20	F-box and leucine rich repeat protein 20	84961	ENSG00000108306	14	0.102	0.361	0.119	0.186	0.013	-0.43	0.068	0.08	0.169	0.1068	0.1202	0.0816	0.1696	-0.142	0.1564	0.1615	0.1808	0.2123	-0.29	-0.99	-0.32	-0.55	-0.36	0.129	-0.08	0.067	0.257	0.119623	0.31102	-0.042478418	0.353498498
392	A8MQ02	AFDN	afadin, adherens junction formation factor	4301	ENSG00000130396	24	0.065	-0.12	-0.18	0.164	0.163	-0.22	-0.03	-0.1	0.292	-0.218	0.0209	0.102	-0.053	-0.338	-0.093	0.3447	0.0384	0.3815	-0.19	-0.16	0.216	-0.41	-0.24	-0.21	0.214	0.067	0.32	0.883394	0.046284	-0.018026834	0.064310805
1645	P17050	NAGA	alpha-N-acetylgalactosaminidase	4668	ENSG00000198951	8	-0.24	-0.73	-0.32	-0.04	-0.32	-0	0.074	0.635	0.182	0.3485	-0.037	0.1612	-0.176	0.0298	-0.028	0.4882	-0.612	-0.392	0.031	0.204	-0.16	0.042	-0.31	-0.3	0.812	0.451	-0.12	0.759398	-0.15848	-0.061657026	-0.096822642
1506	P08697	SERPINF2	serpin family F member 2	5345	ENSG00000276838	12	0.209	-1.35	-0.52	-0.47	-0.32	-0.19	-0.51	-0.52	-0.64	0.2717	-0.534	0.1327	-0.462	0.3922	-0.848	0.2436	-0.946	-0.925	-0.44	0.895	0.146	0.578	-0.01	0.149	-0.38	0.297	-0.46	0.189114	-0.56515	-0.182024509	-0.383121394
3331	Q9C0H2	TTYH3	tweety family member 3	80727	ENSG00000136295	10	-0.55	0.119	0.154	-0.42	-0.16	-0.74	0.304	-0.03	0.107	0.2212	0.2761	-0.153	0.1666	-0.37	-0.008	0.338	0.3259	0.3183	-0.25	0.08	-0.27	-0.18	-0.24	0.086	0.288	0.216	0.286	0.33904	-0.13484	-0.258214208	0.123370547
2621	Q5QPM7	PSMF1	proteasome inhibitor subunit 1	9491	ENSG00000125818	6	-0.18	-0.04	-0.07	-0.22	0.344	-0.62	0.461	0.233	0.489	-0.06	-0.173	-0.007	-0.044	-0.341	0.174	0.416	0.7606	0.5151	-0.17	-0.51	-0.07	-0.23	-0.24	0.067	0.125	0.126	0.365	0.596629	0.105904	-0.092588754	0.198492427
1763	P27169	PON1	paraoxonase 1	5444	ENSG00000005421	9	-1.37	-0.2	-0.49	-0.52	1.418	-0.13	-1.35	1.058	-1.2	-0.487	-0.726	-0.92	0.8592	-0.736	0.1761	1.173	1.4154	-1.358	-1.34	-0.32	-0.55	-0.44	0.047	-0.43	-0.8	-1.29	-1.2	0.464777	0.39255	-0.242562688	0.635112335
3460	Q9NQ36	SCUBE2	signal peptide, CUB domain and EGF like d	57758	ENSG00000175356	8	0.054	-0.9	-0.77	-0.45	0.167	-0.52	0.506	0.054	0.107	0.4775	-0.038	0.2618	-0.46	0.9164	-0.265	0.3691	-0.624	0.4656	0.153	0.465	-0.1	0.031	0.344	-0.22	0.183	0.162	0.029	0.358529	-0.31194	-0.318208225	0.006265747
3614	Q9UHE8	STEAP1	STEAP family member 1	26872	ENSG00000164647	7	-0.55	-0.02	-0.38	-0.35	-0.23	-0.14	-0.25	0.019	-0.35	-0.322	-0.078	0.1543	0.329	0.574	-0.623	0.1857	0.3297	0.2788	-0.59	0.097	-0.5	0.3	0.507	0.138	-0.26	0.211	0.131	0.220331	-0.25355	-0.341136381	0.087582194
2630	S4R347	FNBP1L	formin binding protein 1 like	54874	ENSG00000137942	13	-0.39	-0.16	-0.54	-0.1	0.201	-0.3	-0.1	-0.25	-0.02	-0.334	0.6149	-0.431	0.0489	-0.053	0.1033	-0.07	0.0251	-0.113	-0.71	-0.91	-0.52	-0.43	-0.21	0.066	-0.28	-0.12	0.045	0.219562	0.155706	-0.161700978	0.317407075
3313	Q9BY76	ANGPTL4	angiopoietin like 4	51129	ENSG00000167772	6	-0.05	-0.14	-0.53	-0.26	-0.18	-0.36	1.131	-0.07	0.268	0.0376	-0.302	-0.4	-0.26	-0.095	0.0548	0.2566	-0.336	-0.248	-0.02	1.443	0.184	0.054	-0.13	-0.13	-0.07	-0.23	-0.22	0.628618	-0.1207	0.122579729	-0.243283721
2942	Q8NEX5	WFDC9	WAP four-disulfide core domain 9	259240	ENSG00000180205	1	0.368	-0.79	0.215	0.445	0.424	0.132	-0.01	0.369	0.613	-1.352	0.2699	0.6123	0.8987	-0.348	0.431	0.1735	0.0571	0.1143	-2.01	-2.27	-2.16	-0.44	-0.61	-0.9	0.487	-0.22	-0.11	0.082325	1.110302	0.1003662	1.009935657
1193	O75155	CAND2	cullin associated and neddylation dissociate	23066	ENSG00000144712	6	-1.34	0.707	-1.59				-0.49	-0.46	-0.71	-1.175	-0.685	-0.372				0.6787		-0.429	-1.19	0.083	-1.21				-0.92	-0.22	0.712	0.899014	-0.18975	-0.251784295	0.06203843

3633	Q9UJ68	MSRA	methionine sulfoxide reductase A	4482	ENSG00000175806	9	-0.06	0.096	0.154	0.008	0.004	-0.26	-0.16	0.05	0.145	0.1581	0.0365	0.0718	0.1959	-0.175	0.3504	-0.057	0.062	-0.052	-0.06	-0.57	-0.03	-0.4	-0.32	0.06	-0.17	0.116	0.191	0.241008	0.12872	-0.068484739	0.197204607
3425	Q9HAC8	UBTD1	ubiquitin domain containing 1	80019	ENSG00000165886	4	0.15	0.386	0.017	0.305	0.494	-0.16	-0.13	0.238	-0.22	-0.71	-0.124	0.0579	0.1282	-0.494	0.0903	0.0172	-0.044	0.1027	-0.66	0.029	-0.69	-0.53	-0.41	-0.36	-0.36	-0.11	0.177	0.102306	0.442797	0.228143159	0.214654231
1264	O94903	PLPBP	pyridoxal phosphate binding protein	11212	ENSG00000147471	9	-0.17	0.132	-0.08	0.13	-0.02	-0.42	0.062	-0.02	0.163	0.1636	0.0652	-0.034	0.1854	0.0155	0.2642	0.1399	0.3677	0.1929	-0.23	-0.52	0.096	-0.48	-0.24	0.152	-0.02	-0	0.24	0.146693	0.085514	-0.177318006	0.262832498
435	B4DR80	STK24	serine/threonine kinase 24	8428	ENSG00000102572	13	0.185	0.286	-0.03	0.155	0.107	-0.39	-0.32	-0.36	-0.06	-0.08	0.193	0.2236	0.1467	-0.058	0.2668	-0.058	0.0323	0.0989	-0.25	-0.84	-0.31	-0.54	-0.34	-0.14	-0.09	-0.09	0.122	0.101611	0.229066	-0.130606445	0.359672152
2456	Q14353	GAMT	guanidinoacetate N-methyltransferase	2593	ENSG00000130005	9	-0.17	0.028	-0.45	0.242	0.146	0.135	0.023	0.111	0.215	0.068	-0.044	-0.122	0.2744	-0.047	0.1007	0.0461	0.1171	0.1381	-0.39	-0.5	-0.24	-0.32	-0.06	-0.01	-0.08	0.021	0.159	0.174365	0.187769	-0.028675834	0.216445091
1440	P05534	HLA-A	major histocompatibility complex, class I, A	3105	ENSG00000224320	11	-0.73	-0.33	0.353	-0.28	-0.3	-0.52	0.106	-0.29	0.237	-0.561	-0.225	0.6111	-0.275	-0.047	0.1025	-0.033	-0.385	-0.407	0.227	0.111	1.007	0.391	-0.54	0.004	-0.01	-0.38	0.386	0.340334	-0.32772	-0.059241286	-0.268481987
2537	Q15797	SMAD1	SMAD family member 1	4086	ENSG00000170365	7	0.206	-0.02	-0.08	0.117	-0.11	-0.19	0.451	0.34	-0.26	0.2742	0.1923	0.0877	-0.092	-0.199	0.1407	0.096	-0.497	-0.018	-0.05	0.101	-0.36	-0.47	-0.45	-0.39	-0.54	0.006	-0.32	0.122113	0.324405	0.051040779	0.273364622
2956	Q8NG35	DEFB105A	defensin beta 105A;defensin beta 105B	245908;504	ENSG00000186562	3	-0.14	-0.74	0.424				-0.23	-0.03	-0.65	-0.758	0.0286	1.843				-0.668	0.2516	-0.544	-1.53	-0.69	-0.66				-1.44	1.636	-0.93	0.631282	0.374669	-0.254023945	0.62869326
1980	P48739	PITPNB	phosphatidylinositol transfer protein beta	23760	ENSG00000180957	14	0.22	0.25	0.043	0.013	-0.02	-0.4	-0.28	-0.39	-0.15	-0.047	0.1686	0.0716	0.1743	-0.14	0.1665	-0.186	-0.019	-0.281	-0.23	-0.83	-0.25	-0.61	-0.35	0.07	-0.17	-0.28	-0.12	0.140558	0.229969	-0.068144853	0.298114231
2243	P67936	TPM4	tropomyosin 4	7171	ENSG00000167460	29	-0.54	0.102	-0.41	-0.47	-0.4	-0.66	0.913	0.517	0.65	-0.016	0.1848	-0.363	-0.586	-0.44	-0.316	0.5747	-0.023	0.4821	-0.54	-1.5	-0.6	-0.84	-0.61	-0.37	0.134	0.336	0.58	0.50359	0.34719	0.023467581	0.323722393
1841	P34059	GALNS	galactosamine (N-acetyl)-6-sulfatase	2588	ENSG00000141012	9	-0.05	-0.19	0.254	-0.11	-0.07	-0.38	-0.26	0.107	-0.13	0.7113	0.0479	0.5061	-0.098	-0.214	-0.063	0.1213	-0.271	-0.178	-0.37	-0.04	-0.15	-0.42	-0.3	-0.14	0.618	0.372	-0.76	0.574231	0.039686	-0.153406905	0.193093042
3646	Q9UKN1	MUC12	mucin 12, cell surface associated	10071	ENSG00000205277	8	-1.03	-0.06	-0.51	-0.39	-0.62	-0.13	-0.71	-0.86	0.601	-1.515	-1.778	-1.473	0.5174	-0.62	0.6718	-0.743	0.1156	-0.787	0.068	-0.09	-0.57	-0.8	-0.87	0.817	1.211	-0.01	0.142	0.356886	-0.40317	0.209488644	-0.612653762
3695	Q9Y217	MTMR6	myotubularin related protein 6	9107	ENSG00000139505	12	0.024	0.328	-0.08	0.697	0.621	0.377	-0.35	-0.2	0.01	0.0331	-0.09	0.0551	0.9961	0.5374	0.192	-0.044	-0.251	-0.027	-0.36	-0.89	-0.22	0.506	0.695	0.798	0.156	-0.01	-0.17	0.904293	0.101513	0.002489873	0.09902349
1248	O76071	CIAO1	cytosolic iron-sulfur assembly component 1	9391	ENSG00000144021	6	0.146	0.181	-0.13	-0.21	-0.21	-0.65	0.144	0.199	0.407	0.0938	0.1268	0.2649	-0.09	-0.211	-0.203	0.1682	0.2414	0.3049	-0.16	-1.12	-0.18	-0.39	-0.59	-0.14	0.32	0.18	0.18	0.355204	0.197006	-0.091621332	0.288627341
631	H0YA68	MAN2B2	mannosidase alpha class 2B member 2	23324	ENSG00000013288	10	0.004	0.245	0.332	-0.42	0.164	-0.37	0.168	0.31	-0.01	0.3255	0.1451	0.1915	-0.204	-0.082	-0.888	0.1456	0.1961	0.1901	-0.04	0.035	-0.04	0.008	-0.53	-0.27	0.073	0.192	0.028	0.826264	0.106128	0.044848108	0.06127986
2803	Q86TB3	ALPK2	alpha kinase 2	115701	ENSG00000198796	1	0.156	-1.63	-0.82	-1.17	-0.21	0.353	-0.8	-0.37	-0.46	0.7241	-0.428	-0.51	-0.704	0.4924	0.0739	-1.013	-0.773	-1.238	1.135	1.298	0.448	-0.33	0.859	-0.35	-0.5	-0.77	-0.81	0.303376	-0.65861	-0.175616007	-0.48299184
2528	Q15628	TRADD	TNFRSF1A associated via death domain	8717	ENSG00000102871	10	-0.18	0.133	0.021	-0.1	0.041	-0.15	0.008	0.037	0.068	0.155	0.0918	0.0818	-0.032	-0.06	-0.067	0.1459	0.106	0.0405	-0.19	-0.37	-0.3	-0.22	-0.19	0.003	0.072	0.044	0.12	0.15368	0.101661	-0.064777242	0.166438682
3135	Q96GW7	BCAN	brevican	63827	ENSG00000132692	17	0.027	-0.47	0.246	-0.2	-0.38	-0.12	-0.22	0.502	0.319	-0.238	0.0737	0.7235	-0.229	-0.094	-0.015	0.5908	-0.244	-0.234	-0.08	-0.2	0.46	-0.36	-0.12	-0.28	-0.3	0.257	-0.52	0.700608	0.095998	-0.06943664	0.165434415
533	P02765	AHSG	alpha 2-HS glycoprotein	197	ENSG00000145192	5	-0.71	-0.26	-0.94	-0.18	-0.15	0.266	0.164	-0.06	0.021	-0.102	-0.288	0.0746	-0.226	-0.021	-0.219	0.506	-0.386	0.3469	-0.55	-0.7	0.656	0.513	0.088	0.069	0.494	0.496	0.212	0.355134	-0.34769	-0.170801593	-0.176890519
1720	P22692	IGFBP4	insulin like growth factor binding protein 4	3487	ENSG00000141753	8	0.041	-0.4	-0.3	-0.08	0.049	-0.01	-0.1	0.049	-0.28	0.1996	0.1	-0.002	-0.294	-0.022	-0.393	0.284	-0.611	-0.286	-0.14	0.543	0.236	0.247	0.36	-0.12	0.255	0.395	-0.49	0.227367	-0.25687	0.000190723	-0.257062745
1073	O15127	SCAMP2	secretory carrier membrane protein 2	10066	ENSG00000140497	6	-0.45	0.211	0.047	-0.3	-0.43	-0.76	-0.02	0.022	0.194	0.2053	-0.221	-0.098	-8E-04	-0.435	0.3238	0.1265	0.3667	0.2729	-0.27	-0.89	-0.55	-0.77	-0.67	0.12	0.124	0.07	0.187	0.264501	0.129839	-0.225380873	0.355219961
2535	Q15785	TOMM34	translocase of outer mitochondrial membran	10953	ENSG00000025772	6	0.01	0.051	0.175	0.007	-0.04	0.1	0.25	0.164	0.072	-0.131	-0.266	-0.094	-0.186	-0.014	-0.068	-0.142	-0.086	-0.142	-0.32	-0.8	-0.43	0.328	0.208	-0.06	0.005	-0.13	-0.11	0.172523	0.232252	0.212682799	0.019569309
2499	Q15121	PEA15	proliferation and apoptosis adaptor protein 1	8682	ENSG00000162734	5	0.13	0.411	0.012	-0.09	-0.41	-0.43	-0.24	-0.39	-0.02	0.1866	0.2873	0.04	-0.11	0.0195	0.2935	-0.182	0.2008	-0.324	-0.3	-1.91	-0.46	-0.53	-0.47	-0.08	-0.2	-0.1	-0.05	0.14903	0.343551	-0.15883849	0.502389189
487	C9J1X3	TNK2	tyrosine kinase non receptor 2	10188	ENSG00000061938	8	-0.44	0.066	-0.22	0.065	-0.33	-0.63	0.305	0.465	0.12	-0.047	0.1094	-0.116	0.2645	-0.194	-0.066	0.4738	-0.043	0.0085	-0.31	-0.03	-0.18	-0.34	-0.2	-0.15	0.377	0.399	0.584	0.823097	-0.08326	-0.109417642	0.026161974
1644	P16949	STMN1	stathmin 1	3925	ENSG00000117632	4	-0.07	0.447	-0.18				0.242	-0.11	0.183	-0.001	-0.216	-0.136				-0.226	-0.239	-0.147	-0.24	-0.39	-0.35				-0	-0.21	-0.37	0.098172	0.347328	0.247062665	0.100265003
3767	Q9Y5K5	UCHL5	ubiquitin C-terminal hydrolase L5	51377	ENSG00000116750	12	0.059	0.178	-0.12	-0.56	0.208	-0.8	-0.14	-0.05	0.086	0.147	-0.032	0.1849	0.0676	-0.527	-0.083	-0.094	-0.121	-0.065	0.09	-0.35	0.088	1.645	-0.6	0.198	0.148	0.037	0.041	0.539816	-0.26962	-0.06777883	-0.201846057
865	H3BUP2	NOL3;AC0	nucleolar protein 3	8996	ENSG00000140939	6	0.036	0.167	0.213	0.033	-0.01	-0.33	0.296	0.264	0.224	0.1071	0.0846	-0.008	-0.175	-0.276	0.1012	0.0996	0.2822	0.2183	-0	-0.69	-0.13	-0.62	-0.35	-0.13	0.235	0.244	0.375	0.367163	0.218335	0.051403643	0.166930916
3119	Q96DZ1	ERLEC1	endoplasmic reticulum lectin 1	27248	ENSG00000068912	10	-0.24	-1.03	0.831	-0.25	-0.17	-0.01	-0.03	0.332	-0.2	-0.057	-0.038	-0.196	-0.196	0.371	0.1297	-0.288	-0.304	-0.323	-0.2	0.723	0.38	0.009	0.319	0.027	0.356	0.094	-0.12	0.356592	-0.26242	0.014868254	-0.277284802
2247	P68363	TUBA1B	tubulin alpha 1b	10376	ENSG00000123416	34	0.235	0.413	-0.15	0.059	0.021	-0.64	-0.06	-0.27	0.168	0.1542	-0.066	0.15	-0.017	0.0424	0.1499	-0.112	0.1292	0.0648	-0.6	-1.57	-0.42	-0.69	-0.51	-0.14	-0.05	0.025	0.396	0.15756	0.371041	-0.079714772	0.450755727
3591	Q9UBW8	COPS7A	COP9 signalosome subunit 7A	50813	ENSG00000111652	8	-0.03	0.24	0.022	-0.17	0.116	-0.32	-0.13	0.348	0.34	0.2083	-0.06	0.0516	0.095	-0.162	0.0592	0.3707	0.1387	0.2262	-0.04	-0.39	0.108	-0.46	-0.23	-0.05	0.315	0.242	0.144	0.561694	0.086419	-0.056516623	0.142935887
2061	P52735	VAV2	vav guanine nucleotide exchange factor 2	7410	ENSG00000160293	10	0.337	0.351	-0.06	-0.12	-0.2	-0.19	-0.18	0.032	0.042	0.0634	######	0.27	0.0587	-0.149	0.0297	0.0506	-0.019	0.0357	-0.36	-1.21	-0.3	-0.45	-0.05	0.111	0.1	-0.04	-0.07	0.205479	0.252079	-0.036906483	0.288985792
778	G3V3G9	AL139011.	novel protein	NA	ENSG00000258465	11	-0.2	-0.15	-0.12	-0.05	-0.14	-0.24	0.3	0.226	0.411	0.0894	-0.032	0.0519	-0.151	0.0419	0.122	0.5016	0.1931	0.2945	-0.15	-0.39	-0.02	-0.6	-0.36	0.012	0.258	0.221	0.28	0.401087	0.085997	-0.120853328	0.206850638
3336	Q9GZS3	WDR61	WD repeat domain 61	80349	ENSG00000140395	11	0.107	0.23	0.056	-0.11	0.262	-0.41	-0.06	0.018	0.147	0.1044	0.0269	0.1946	0.0616	-0.086	0.0713	0.0805	0.1804	0.0962	-0.14	-0.89	-0.24	-0.3	-0.16	0.168	0.18	-0.01	0.122	0.272774	0.166599	-0.055194638	0.221793602
991	O00170	AIP	aryl hydrocarbon receptor interacting protein	9049	ENSG00000110711	9	0.109	0.333	-0.11	0.031	0.065	-0.26	-0.01	0.394	0.112	0.1209	0.018	0.0625	0.065	-0.121	0.1321	0.2939	0.0837	0.0093	-0.27	-0.83	-0.22	-0.48	-0.23	-0.19	0.341	0.085	0.343	0.239861	0.233936	-0.000574109	0.23450995
3597	Q9UEW8	STK39	serine/threonine kinase 39	27347	ENSG00000198648	15	0.131	0.238	0.033	-0.17	0.244	-0.3	-0.12	0.683	0.305	0.2206	-0.107	0.3227	0.0357	-0.067	-0.006	0.2795	-0.23	-1E-03	-0.04	-0.69	0.263	-0.21	0.045	0.276	0.23	0.122	0.454	0.89652	0.067468	0.067563419	-9.56751E-05
2502	Q15149	PLEC	plectin	5339	ENSG00000178209	30	-0.06	-0.05	-0.2	-0.14	-0.12	-0.33	-0.02	0.09	0.153	-0.037	-0.019	-0.15	-0.256	-0.072	-0.013	0.0153	0.0742	-0.01	-0.22	-0.07	0.049	-0.3	-0.19	-0.14	0.187	0.031	0.083	0.960594	-0.01103	-0.022610799	0.0115806
3650	Q9UL15	BAG5	BCL2 associated athanogene 5	9529	ENSG00000166170	15	0.426	0.034	0.305	-0.6	-0.38	-0.41	-0.37	-0.24	-0.02	0.6219	0.7228	-0.222	-0.414	0.017	-0.266	-0.197	0.0304	-0.218	0.078	0.968	0.242	-0.31	-0.21	-0	-0.29	-0.02	0.06	0.650075	-0.19738	-0.148401003	-0.048974639
3781	Q9Y608	LRRFIP2	LRR binding FLII interacting protein 2	9209	ENSG00000093167	7	0.418	-0	0.186	-0.15	0.093	-0.3	-0.25	0.051	0.32	-0.09	-0.53	0.1241	-0.053	-0.125	0.1331	0.2121	0.0839	0.0741	0.183	-0.73	-0.23	-0.47	-0.28	-0.04	0.168	-0.04	-0.02	0.401549	0.204301	0.06000159	0.144299205
3777	Q9Y5Y2	NUBP2	nucleotide binding protein 2	10101	ENSG00000095906	10	0.071	0.194	0.037	-0.16	0.412	-0.15	0.001	0.173	0.026	0.0904	0.0822	0.0333	0.0838	0.0901	0.0395	0.1268	0.113	0.1292	-0.2	-0.53	-0.11	-0	-0.04	0.18	0.009	0.079	0.203	0.358528	0.11254	-0.02057059	0.133110866
1764	P27216	ANXA13	annexin A13	312	ENSG00000104537	9	-0.02	0.053	-0.32	-0.73	-0.12	0.456	0.151	0.405	0.266	0.2502	0.1109	-0.057	-0.231	-0.413	0.5375	1.0509	0.0599	0.1812	0.036	0.334	-0.17	-0.76	-0.5	-1.09	1.417	-0.04	-0.27	0.657696	0.133318	-0.149471221	0.282789059
723	F6QUN3	ERI3	ERI1 exoribonuclease family member 3	79033	ENSG00000117419	4	0.253	0.091	0.039	0.115	0.049	-0.37	-0.29	-0.1	-0.05	-0.019	0.1691	0.0447	-0.03	-0.303	-0.045	-0.037	0.0154	0.1948	-0.25	-0.64	-0.17	-0.33	-0.31	-0.09	-0.04	0.007	0.094	0.229213	0.163529	-0.02777484	0.191303852
641	E9PFK9	RABGEF1	RAB guanine nucleotide exchange factor 1	27342	ENSG00000154710	6	0.008	0.066	-0.14	0.009	-0.09	-0.38	-0.14	-0.05	-0.14	0.0521	-0.193	0.0415	-0.047	0.0769	-0.093	-0.026	-0.408	0.294	-0.05	-0.51	-0.14	-0.37	-0.28	0.011	0.168	-0.34	-0.06	0.439452	0.079292	-0.061336376	0.14062846
937	J9JIC5	C17orf75	chromosome 17 open reading frame 75	64149	ENSG00000108666	8	-0.01	0.16	-0.22	0.049	-0.15	-0.23	-0.03	0.092	0.457	0.1413	-0.027	0.004	0.1574	-0.071	-0.127	0.504	0.0583	0.1451	-0.44	-1.18	-0.48	-0.74	-0.34	0.137	0.174	0.184	0.465	0.267788	0.25887	-0.074108907	0.332978447
1137	O43765	SGTA	small glutamine rich tetratricopeptide repeat	6449	ENSG00000104969	11	0.099	0.025	-0.11	-0.02	-0.06	0.043	0.249	0.183	0.346	0.0004	0.0493	-0.022	-0.192	0.381	-0.09	0.2677	0.4392	0.4082	-0.23	-0.84	-0.21	-0.6	-0.19	-0.04	0.243	0.107	0.224	0.171959	0.25394	-0.054805873	0.308746154
2802	Q86SU0	ILDR1	immunoglobulin like domain containing rece	286676	ENSG00000145103	9	-0.58	0.365	-0.02	0.043	-0.25	-0.17	0.227	0.034	0.132	0.1392	-0.257	-1E-04	0.18	-0.298	0.0552	0.5726	0.1436	0.1792	-0.72	-0.28	-0.34	-0.41	-0.57	-0.18	0.155	0.296	0.58	0.465337	0.139374	-0.103651569	0.243025778
3107	Q96CD0	FBXL8	F-box and leucine rich repeat protein 8	55336	ENSG00000135722	7	-0	-0.41	-0.53	0.102	-0.33	0.583	0.035	0.236	0.313	-0.017	-0.154	0.1083	-0.077	0.3276	0.0866	0.0465	0.2443	-0.216	-0.15	-0.57	-0.09	0.317	-0.11	-0.17	0.132	-0.25	0.584	0.918361	0.033444	-0.039776498	0.073220959
3076	Q969Q5	RAB24	RAB24, member RAS oncogene family	53917	ENSG00000169228	7	0.003	0.685	0.049	-0.07	-0.03	-0.13	-0.12	-0.18	0.108	0.3962	-0.008	0.2246	0.1543	-0.088	0.1955	0.0695	0.2174	-0.005	-0.21	-0.81	-0.17	-0.59	-0.27	0.106	-0.2	0.034	-0.03	0.101335	0.272496	-0.09319846	0.365694237
2109	P55212	CASP6	caspase 6	839	ENSG00000138794	11	0.045	0.217	0.115	-0.03	-0.16	-0.37	-0.01	0.126	0.273	-0.159	-0.023	-0.141	0.0707	-0.12	0.0542	0.1845	0.1884	0.1155	-0.31	-0.84	-0.08	0.343	-0.45	0.256	0.191	-0	0.209	0.77354	0.098243	0.003951528	0.094291493
3026	Q8WZA0	LZIC	leucine zipper and CTNNBIP1 domain conta	84328	ENSG00000162441	4	0.364	0.398	0.443	0.256	0.233	-0.19	0.033	-0.16	-0.28	0.3635	0.4593	0.1833	0.2135	-0.34	0.4655	0.0568	0.3502	0.0498	-0.21	-0.86	-0.19	-0.23	-0.23	0.182	-0.19	0.007	0.114	0.126	0.300673	-0.078127246	0.378800297
3783	Q9Y624	F11R	F11 receptor	50848	ENSG00000158769	7	-0.32	0.259	0.567	-0.19	-0.08	0.681	-0.58	-0.1	-0.42	-0.018	0.419	0.2137	-0.365	0.3601	-0.158	-0.65	-0.226	-0.028	-0.64	0.34	-0.15	-0.13	-0.15	-0.15	0.494	0.101	-0.4	0.968109	0.056259	0.030765376	0.025494104
3721	Q9Y2Q5	LAMTOR2	late endosomal/lysosomal adaptor, MAPK a	28956	ENSG00000116586	6	-0.2	0.565	-0.13	0.044	0.001	-0.56	0.205	0.12	0.101	0.1047	0.018	0.0188	0.0461	-0.06	0.2549	0.1856	0.3734	0.1505	-0.13	-0.68	0.054	-0.69	-0.52	0.034	-0.13	0.084	0.452	0.275326	0.18626	-0.105642157	0.291901955
1124	O43583	DENR	density regulated re-initiation and release fa	8562	ENSG00000139726	9	0.133	0.269	0.152	-0.01	0.073	-0.46	0.037	0.244	0.292	0.1883	0.0764	0.0625	0.0847	-0.198	0.1909	0.1815	0.2352	0.0172	-0.16	-0.99	-0.32	-0.42	-0.3	0.106	0.004	0.114	0.1	0.147182	0.287892	-0.012692899	0.300585093
856	H3BRF9	ZFYVE19	zinc finger FYVE-type containing 19	84936	ENSG00000166140	5	-0.03	-0.17	-0.24				-0.07	-0.14	-0.06	-0.238	0.0716	-0.032				-0.111	-0.135	0.0581	-0.16	-0.28	-0.12				-0.03	-0.09	-0.11	0.591674	0.012865	-0.054810086	0.067674809
2685	Q6IA86	ELP2	elongator acetyltransferase complex subunit	55250	ENSG00000134759	13	-0.02	0.041	-0.09	0.022	0.145	-0.17	-0.16	0.586	-0.04	0.1985	0.1038	0.0146	0.1154	0.0035	-0.01	0.199	0.0368	0.1625	-0.03	-0.33	-0.25	-0.17	-0.16	0.027	0.272	0.209	0.05	0.47019	0.077372	-0.056863334	0.134234893
1298	O95631	NTN1	netrin 1	9423	ENSG00000065320	6	0.07	0.492	-0.14	-0.39	-0.28	-0.25	0.606	0.372	0.632	0.0031	0.2691	0.5853	-0.317	0.0022	0.3854	0.299	0.0552	0.6098	-0.22	0.195	-0.13	-0.21	-0.09	0.396	0.642	0.384	0.269	0.899101	-0.01316	-0.086753443	0.073593574
523	C9JJP5	TFG	TRK-fused gene	10342	ENSG00000114354	9	0.057	0.248	0.087	0.017	0.103	-0.52	-0.05	-0.11	-0.01	0.1934	0.0273	0.2463	0.3032	-0.073	0.0741	0.0465	-0.007	-0.123	-0.03	-0.36	-0.21	-0.41	-0.17	0.063	0.326	0.025	0.071	0.439337	0.056757	-0.095737074	0.15249418
1523	P09758	TACSTD2	tumor associated calcium signal transducer	4070	ENSG00000184292	8	-0.49	-0.2	0.038	-0.29	-0.36	-0.2	-0.07	0.143	-0.11	0.2012	0.4399	0.0206	-0.209	0.2147	-0.462	0.0001	-0.271	-0.155	-0.21	-0.15	-0.08	-0.19	-0.17	-0.06	0.66	-0.31	-0.13	0.60944	-0.10055	-0.147630371	0.047083828
730	F8VPD4	CAD	carbamoyl-phosphate synthetase 2, asparta	790	ENSG00000084774	20	-0.09	-0.03	-0.26	0.115	0.162	0.294	-0.01	0.883	0.093	0.0125	0.0032	0.041	0.0741	-0.115	0.0027	0.3373	0.0266	0.0539	-0.33	-0.71	-0.08	-0.01	-0.02	-0.02	0.578	0.416	0.302	0.798402	0.11557	0.079949594	0.035620062
3726	Q9Y2V2	CARHSP1	calcium regulated heat stable protein 1	23589	ENSG00000153048	6	-0.1	0.192	0.103	0.118	-0.01	-0.02	-0.04	-0.24	-0.04	0.0688	0.0438	0.1299	-0.044	0.0459	0.3606	0.0345	-0.026	-0.226	-0.04	-0.78	-0.21	-0.75	-0.45	-0.11	-0.11	-0.06	-0.19	0.056899	0.293224	-0.047614684	0.340838938
306	A0A286YFJ8	IGHG4	immunoglobulin heavy constant gamma 4 (	3503	ENSG00000211892	11	-0.6	-1.17	-0.5	0.031	-0.62	0.018	-0.39	0.849	-0.06	-0.229	-0.784	0.0433	0.1025	-0.571	-0.59	-0.091	-0.196	-0.253	0.079	-0.06	-0.18	0.312	0.859	-0.07	0.876	0.013	-0.52	0.233932	-0.41767	0.013832054	-0.431497505
3239	Q9BQ13	KCTD14	potassium channel tetramerization domain c	65987	ENSG00000151364	4	0.121	0.266	0.726	-0.03	-0.04	-0.16	-0.11	-0.09	0.013	0.1744	0.4244	0.5988	0.4195	0.0159	-0.493	0.0812	-0.004	-0.295	-0.05	-0.31	0.574	0.522	-0.31	0.33	0.14	-0.05	-0.01	0.991333	-0.01383	-0.023520096	0.009693481
2510	Q15276	RABEP1	rabaptin, RAB GTPase binding effector prot	9135	ENSG00000029725	19	0.203	0.449	-0.06	-0.12	-0.1	-0.67	-0.08	0.029	0.192	-0.034	0.3898	0.2092	0.0329	-0.159	0.0846	0.0858	0.2996	0.1917	-0.16	-0.45	-0.35	-0.66	-0.43	0.069	0.293	0.082	0.014	0.222776	0.158737	-0.13967004	0.29840688
2421	Q13572	ITPK1	inositol-tetrakisphosphate 1-kinase	3705	ENSG00000100605	6	-0.1	0.128	-0.21	-0.05	0.063	0.13	0.158	0.098	0.083	0.0894	-0.083	0.0097	-0.129	-0.074	0.1273	0.1288	0.1519	0.0574	-0.1	-0.51	-0.26	-0.49	-0.25	-0.03	0.176	0.134	0.3	0.348758	0.146886	0.002885737	0.144000037
1899	P40818	USP8	ubiquitin specific peptidase 8	9101	ENSG00000138592	6	-0.1	0.026	0.579	-0.27	-0.24	-0.48	-0.03	0.019	0.08	0.0863	0.0683	0.0568	0.0084	0.0554	0.0059	0.0929	0.3091	-0.045	-0.11	0.135	-0.32	-0.76	-0.23	0.1	-0.08	-0.1	-0.13	0.267615	0.120595	-0.117055309	0.23765067
235	A0A0G2JQ4	ABR	ABR activator of RhoGEF and GTPase	29	ENSG00000276016	6	0.04	0.334	-0.18	0.257	0.178	-0.2	0.075	0.053	0.108	0.4378	0.3262	0.2335	0.0454	-0.005	0.0778	0.0187	0.1408	0.1486	-0.16	-0.25	-0.42	0.137	0.028	0.297	0.11	0.11	0.278	0.462385	0.059441	-0.084361852	0.143803008
979	M0R0F0	RPS5	ribosomal protein S5	6193	ENSG00000083845	9	0.277	-0.03	-0.01	-0.01	0.369	-0.07	0.042	-0.09	0.274	-0.166	0.1009	-0.142	-0.143	-0.138	-0.081	-0.008	0.172	0.0142	-0.05	-0.65	0.159	-0.27	0.048	0.048	0.166	-0.09	0.075	0.402828	0.144649	0.125384235	0.019264776
2691	Q6J9G0	STYK1	serine/threonine/tyrosine kinase 1	55359	ENSG00000060140	9	0.146	0.152	0.039	-0.41	-0.09	-0.25	-0.08	-0.16	-0.01	-0.144	0.1484	0.5038	0.0747	0.0592	-2E-04	0.1739	0.0105	0.2485	-0.14	-0.31	-0.35	-0.39	0.067	-0.33	-0.01	0.179	0.216	0.173031	0.044965	-0.192418767	0.237383962
1016	O00499	BIN1	bridging integrator 1	274	ENSG00000136717	8	-0.05	-0.02	-0.02	-0.08	0.176	-0.43	-0.77	-0.59	-0.53	0.02	0.0421	0.0392	0.2223	-0.102	0.062	-0.769	-0.258	-0.64	-0.23	-0.39	0.039	-0.57	-0.24	0.07	-0.45	-0.45	-0.46	0.696506	0.042286	-0.103469638	0.145755427
3452	Q9NP97	DYNLRB1	dynein light chain roadblock-type 1	83658	ENSG00000125971	6	0.092	0.136	-0.1	-0.23	0.03	-0.59	0.317	0.042	0.175	0.0494	-0.007	-0.068	-0.196	-0.186	0.0962	0.1265	0.2748	0.0227	-0.03	-0.61	-0.09	-0.76	-0.43	-0.14	-0.11	0.187	0.199	0.355193	0.183504	-0.027509417	0.211013595
1494	P08237	PFKM	phosphofructokinase, muscle	5213	ENSG00000152556	16	-1	0.11	-0.78	0.188	0.354	-0.19	-0.1	-0.09	-0.09	-0.176	-0.874	0.095	0.025	-0.195	0.2253	0.004	0.0477	-0.068	-0.31	0.228	1.702	-0.21	-0.15	-0.09	0.135	-0.07	-0.05	0.517732	-0.30966	-0.075920473	-0.23373499
720	J3QS47	CYB561	cytochrome b561	1534	ENSG00000008283	2	-0.24	0.258	0.143	0.031	-0.1	-0.44	0.08	0.031	-0	0.0479	-0.224	-0.201	0.6193	-0.307	0.2628	0.2925	0.7808	0.3456	-0.34	-0.4	0.067	-0.24	-0.56	0.008	-0.12	-0.29	0.224	0.169605	0.156594	-0.206825462	0.363419906
970	K7ERS5	AD000671	novel protein	NA	ENSG00000188223	2	-0.36	0.265	-0.12				0.286	0.295	0.444	-0.249	-0.406	0.1555				0.5182	0.4506	0.7023	-0.4	-0.81	-0.48				-0.05	0.124	0.667	0.499694	0.292548	-0.060461298	0.353009256
52	A0A087WV	RRBP1	ribosome binding protein 1	6238	ENSG00000125844	10	-0.05	-0.49	-0.3	-0.11	-0.18	0.389	-0.2	0.292	0.543	0.2551	0.0017	0.1155	-0.313	0.444	0.1283	0.4005	0.652	0.3526	0.132	0.493	0.241	-0.12	0.475	-0.06	-0.4	0.328	0.53	0.418728	-0.19071	-0.237991735	0.047280689
3281	Q9BV36	MLPH	melanophilin	79083	ENSG00000115648	41							-0.03	-0.28	-0.12							0.2984	-0.046	0.3745							-0.65	-0.22	0.073	0.292936	0.117769	-0.356342976	0.474112111
338	A0A2R8YDC	AC068831	novel protein	NA	ENSG00000284946	11	0.193	0.296	0.143	0.146	-0.13	0.177	0.139	0.195	0.19	0.2569	0.1907	0.1235	0.1194	0.1137	0.1398	0.1446	0.0585	0.1863	-0.13	-0.69	-0.06	-0.11	-0.14	-0.11	0.066	-0.07	0.363	0.088114	0.248196	0.001739115	0.246457103
1179	O60825	PFKFB2	6-phosphofructo-2-kinase/fructose-2,6-biph	5208	ENSG00000123836	14	0.09	0.263	-0.04	-0.01	0.032	-0.33	0.017	0.154	0.177	0.222	0.0554	0.0809	0.0796	-0.262	0.0578	0.083	0.0291	0.0335	-0.02	-0.72	-0.13	-0.22	-0.27	0.134	0.111	0.311	0.213	0.628051	0.105908	-0.003118772	0.10902677
565	D6RBW1	EIF4E	eukaryotic translation initiation factor 4E	1977	ENSG00000151247	5	0.055	-0.41	-0.27	-0.01	0.057	-0.34	0.044	-0.06	0.222	-0.173	-0.093	-0.179	0.0695	-0.139	0.0253	-0.012	0.216	-0.001	-0.03	-0.09	0.009	-0.5	-0.28	0.049	0.096	-0.02	0.14	0.9013	-0.00817	-0.046667787	0.038493548
2807	Q86UD1	OAF	out at first homolog	220323	ENSG00000184232	8	-0.57	-0.69	0.028	-0.85	-0.47	-0.09	-0.47	0.012	-0.22	-0.214	0.4778	0.2522	-0.667	0.4479	-0.401	0.0894	-0.585	-0.125	0.08	0.418	0.269	-0.31	-0.06	-0.24	0.608	0.15	-0.3	0.172499	-0.43892	-0.289304322	-0.149620218
2776	Q7Z2W7	TRPM8	transient receptor potential cation channel s	79054	ENSG00000144481	14	-0.43	0.404	-0.19	-0.01	0.082	-0.19	-0.12	0.076	0.117	0.3138	0.1283	0.1163	0.0359	-0.1	-0.494	0.5404	0.0229	-0.164	-0.06	0.199	-0.08	-0.56	0.184	0.09	-0.17	-0.1	0.753	0.910103	-0.05577	-0.073190988	0.017425964

2880	Q8J025	APCDD1	APC down-regulated 1	147495	ENSG00000154856	5	-0.13	-0.64	-0.27	-0.18	0.11	0.14	-0.16	0.385	-0.15	0.0508	0.1365	-0.067	-0.079	-0.697	0.2335	-0.051	0.0977	0.1059	0.031	-0.24	1.435	0.089	-0.75	0.151	0.246	0.278	0.077	0.573995	-0.24428	-0.069031708	-0.175253232
2536	Q15796	SMAD2	SMAD family member 2	4087	ENSG00000175387	8	0.2	0.131	-0.03				-0.16	-0.1	-0.06	0.1911	0.089	0.1249				0.0739	-0.221	-0.147	-0.22	-0.49	-0.18				0.418	-0.12	0.197	0.865948	0.062868	-0.021648444	0.084516513
1394	P02766	TTR	transthyretin	7276	ENSG00000118271	5	-0.53	-0.25	0.165	-0.14	0.035	-0.15	-0.19	0.032	-0.14	-0.359	0.1803	0.038	-0.197	0.1082	-0.041	0.4078	-0.612	-0.357	-0.67	-0.38	0.861	0.443	-0.1	0.152	0.09	0.108	-0.72	0.884355	-0.10571	-0.037333397	-0.068377253
1245	O76003	GLRX3	glutaredoxin 3	10539	ENSG00000108010	12	0.078	0.176	-0.07	-0.03	0.063	-0.49	0.024	0.068	0.181	0.2833	0.1374	0.1505	0.0107	0.0857	-0.011	0.1364	0.0668	0.0962	-0.17	-0.44	-0.06	-0.74	-0.3	0.228	-0.01	0.099	0.139	0.214454	0.138343	-0.107612426	0.245954948
1387	P02748	C9	complement C9	735	ENSG00000113600	10	-0.2	-0.28	-0.41	-0.07	0.033	0.027	0.198	0.695	-0.04	-0.3	0.3993	0.4621	-0.042	0.2679	-0.114	0.0757	-0.325	-0.204	-0.54	-0.44	0.911	0.248	0.077	0.067	0.099	-0.01	-0.31	0.988932	-0.01667	-0.029226228	0.012556774
1600	P13866	SLC5A1	solute carrier family 5 member 1	6523	ENSG00000100170	6	0.376	0.15	0.628	-0.42	0.551	-0.03	-0.01	0.157	-0.01	-0.075	0.1995	-0.056	1.0134	-0.749	0.0318	-0.628	0.5142	-0.619	-0.43	0.495	-0.42	-0.05	-0.45	-0.87	-0.64	-0.8	-0.95	0.14471	0.612081	0.194971633	0.417109358
1489	P07998	RNASE1	ribonuclease A family member 1, pancreatic	6035	ENSG00000129538	5	-0.15	-0.81	-1.06	-0.62	0.045	0.182	0.275	-0.11	0.331	1.2309	0.2883	-0.505	-1.415	0.6364	-1.05	1.0236	-0.664	0.3747	0.37	-0.29	-0.6	-0.82	0.021	-0.71	0.683	1.407	-1.2	0.904316	-0.08674	-0.204643778	0.11790196
713	G3V1J9	TMED3	transmembrane p24 trafficking protein 3	23423	ENSG00000166557	2	0.034	0.032	-0.05	0.073	0.259	-0.1	-0.03	0.173	-0.13	-0.251	-0.022	0.1187	-0.142	0.1091	-0.053	0.0288	-0.158	0.1605	0.102	0.393	0.128	-0.19	-0.02	-0.22	0.237	-0.05	0.166	0.656076	-0.03161	0.052470229	-0.08407601
2017	P50151	GNG10	G protein subunit gamma 10	2790	ENSG00000242616	6	-0.29	0.612	-0.22	0.187	0.201	-0.25	-0.22	-0.02	0.075	0.148	-0.088	-0.036	-0.233	-0.546	0.5096	0.1735	0.0088	-0.034	-0.41	-0	0.024	-0.59	-0.49	-0.06	-0.1	0.193	0.174	0.635806	0.14955	0.019805028	0.129744561
2935	Q8NDZ4	DIPK2A	divergent protein kinase domain 2A	205428	ENSG00000181744	10	-0.06	-0.5	-0.56	-0.73	0.211	-0.26	-0.32	-0.29	-0.16	0.0174	-0.271	-0.093	-0.237	0.1405	-0.037	-0.053	-0.126	0.047	0.245	0.568	0.061	-0.08	-0.27	-0	-0.05	-0.54	-0.14	0.199662	-0.27666	-0.230534672	-0.046122945
930	J3QR09	RPL19	ribosomal protein L19	6143	ENSG00000108298	8	0.132	0.122	0.108	-0.48	0.291	-0.51	0.068	0.048	-0.02	-0.01	-0.046	-0.063	-0.117	-0.202	-0.032	-0.261	0.0876	0.0996	-0.16	-0.05	-0	-0.14	-0.25	-0.02	0.281	-0.18	-0.11	0.926128	0.042104	0.033913774	0.008189815
1077	O15160	POLR1C	RNA polymerase I and III subunit C	9533	ENSG00000171453	4	0.205	0.513	-0.25	0.098	0.523	-0.02	1.575	0.802	1.211	0.6588	0.378	0.4591	2.3842	0.0637	-0.007	0.779	-0.223	0.7401	0.061	-1.02	-0.22	3E-04	3.44	-0.05	0.375	0.025	0.897	0.934832	0.127297	-0.064614686	0.191911556
3214	Q99576	TSC22D3	TSC22 domain family member 3	1831	ENSG00000157514	5	0.244	0.041	-0.02	0.03	-0.01	-0.11	-0.05	0.049	0.378	-0.057	0.0644	0.1351	-0.046	0.169	0.1975	-0.052	0.2296	0.1399	-0.07	-0.66	-0.17	-0.36	-0.2	0.082	0.1	0.097	0.099	0.181252	0.182347	-0.024179571	0.206526916
696	F5GWI9	WASHC3	WASH complex subunit 3	51019	ENSG00000120860	5	-0.08	-0.09	0.563	0.11	-0.16	-0.65	-0.16	0.031	0.132	-0.261	0.0727	-0.24	0.136	-0.24	-0.036	0.221	0.5568	0.334	-0.07	-1.1	0.149	-0.34	-0.3	0.071	-0.28	-0.07	0.097	0.405395	0.169936	-0.094905734	0.264841366
2513	Q15311	RALBP1	ralA binding protein 1	10928	ENSG00000017797	7	0.225	0.348	-0.11	0.069	0.141	-0.36	0.029	0.043	0.137	0.0844	-0.026	0.1098	0.1093	-0.208	0.0607	0.1701	-0.018	0.4187	-0.2	-0.45	-0.13	-0.36	-0.18	0.148	0.333	-0.07	0.314	0.495563	0.124849	-0.019642735	0.144491597
2749	Q6ZS86	GK5	glycerol kinase 5	256356	ENSG00000175066	7	0.064	-0.49	0.364	0.378	0.091	-0.22	-0.32	-0.13	0.039	-0.613	0.1721	-0.449	0.1126	-0.418	0.313	-0.079	0.7275	0.3277	0.146	-0.96	-0.42	-0.53	0.029	-0.1	-0.02	0.201	0.35	0.773655	0.120217	-0.035316431	0.155533093
3486	Q9NRR3	CDC42SE:	CDC42 small effector 2	56990	ENSG00000158985	2	-0.22	0.25	-0.02	-0.18	-0.19	-0.27	-0.16	-0.09	0.086	-0.179	0.0154	-0.073	-0.002	-0.166	0.0218	0.3526	0.2594	0.3075	-0.27	-0.78	-0.42	-0.42	-0.27	-0.11	0.055	0.156	0.24	0.226703	0.112676	-0.146960298	0.259636321
2937	Q8NE71	ABCF1	ATP binding cassette subfamily F member '	23	ENSG00000225989	9	-0.05	0.162	-0.16	-0.07	-0.07	-0.16	0.35	0.011	0.481	-0.006	-0.113	0.0676	0.077	-0.065	0.1413	0.2158	-0.204	0.1084	-0.2	-0.43	-0.24	-0.24	-0.21	0.005	-0.01	0.155	0.039	0.249048	0.180454	0.030407866	0.150046047
2615	Q5JVZ5	ELMO2	engulfment and cell motility 2	63916	ENSG00000062598	13	-0.01	-0.05	-0.2	0.001	0.118	-0.37	-0	-0.02	0.178	0.0422	0.0377	-0.122	0.1552	-0.261	0.0675	0.1625	0.0902	0.16	-0.27	-0.58	-0.24	-0.1	-0.14	0.059	0.21	-0.04	0.144	0.441952	0.066925	-0.07700072	0.143925445
2902	Q8N573	OXR1	oxidation resistance 1	55074	ENSG00000164830	7	0.941	-1.13	0.824	-0.25	-0.28	-0.42	0.581	-0.6	-0.24	-1.028	-1.109	-1.206	-0.203	-0.805	-0.264	-0.512	0.0302	-0.151	-1.44	0.467	-1.36	-0.04	-0.41	-0.56	-0.2	-0.42	-0.04	0.33948	0.382487	0.520151085	-0.137663817
2131	P60201	PLP1	proteolipid protein 1	5354	ENSG00000123560	4	-1.24	0.185	-0.64	-0.32	-0.25	-0.23	-0.49	-0.04	-0.86	2.1058	0.5826	-0.197	0.3787	-0.151	-0.371	-0.29	-0.318	0.5931	-0.42	-0.31	-1.04	0.533	-0.66	-0.2	-0.85	-0.45	-0.16	0.147508	-0.03678	-0.690452879	0.653675528
1661	P18065	IGFBP2	insulin like growth factor binding protein 2	3485	ENSG00000115457	8	0.046	-0.04	0.089	-0.35	-0.19	-0.17	-0.19	-0.19	-0.23	0.3712	-0.46	0.3111	-0.215	-0.025	-0.264	0.1517	-0.166	-0.133	-0.09	0.356	-0.09	-0.2	0.614	-0.39	0.156	0.176	-0.34	0.591707	-0.15817	-0.089109796	-0.069057994
1172	O60684	KPNA6	karyopherin subunit alpha 6	23633	ENSG00000025800	8	0.025	-0.04	-0.07	0.005	-0.05	-0.33	-0	0.012	0.159	0.0425	-0.165	0.37	0.0213	-0.023	-0.073	0.1935	0.0227	0.138	-0.25	-0.53	-0.39	-0.23	-0.21	0.127	-0.04	0.024	0.07	0.168189	0.126827	-0.090276023	0.217103173
1915	P42025	ACTR1B	actin related protein 1B;ARP1 actin related ¡	10120	ENSG00000115073	12	1.016	0.189	0.052	0.152	0.321	-0.32	0.169	-0.13	0.081	0.0906	0.0988	0.2194	-0.085	-0.178	0.0727	-0.047	-0.041	0.047	-0.11	-0.54	0.825	-0.61	-0.32	-0.09	0.177	0.035	0.22	0.541131	0.215684	0.150833205	0.064850359
1061	O14974	PPP1R12/	protein phosphatase 1 regulatory subunit 12	4659	ENSG00000058272	13	0.026	0.152	-0.1	-0.09	0.074	-0.23	0.157	0.006	0.276	-0.208	0.0819	0.1091	-0.056	-0.103	-0.08	0.2132	0.3055	0.1046	-0.31	-0.28	-0.23	-0.37	0.092	0.064	-0	0.138	0.222	0.497683	0.106278	-0.010551925	0.116829453
1015	O00487	PSMD14	proteasome 26S subunit, non-ATPase 14	10213	ENSG00000115233	7	0.103	0.024	-0.03	0.095	0.441	-0.28	0.138	0.28	0.255	-0.132	-0.281	-0.02	-0.106	-0.263	0.167	0.2546	0.1582	0.0673	-0.08	-0.53	-0.06	-0.34	-0.24	0.042	0.479	0.196	0.301	0.56373	0.139562	0.130705694	0.008856304
2455	Q14344	GNA13	G protein subunit alpha 13	10672	ENSG00000120063	14	-0.19	0.102	-0.07	-0.03	-0.06	0.211	0.092	0.209	0.237	0.0489	0.0453	0.0525	-0.053	-0.148	0.2169	0.2963	0.0031	0.2684	-0.09	0.056	-0.12	-0.33	-0.3	-0.35	0.492	0.102	0.417	0.741759	0.070263	-0.02477549	0.095038644
1068	O15067	PFAS	phosphoribosylformylglycinamidine synthase	5198	ENSG00000178921	18	0.326	0.086	0.898	0.002	0.282	-0.32	0.134	0.106	0.236	0.1501	0.0604	0.0788	0.183	-0.41	0.0787	0.1546	0.066	-0.002	-0.09	-0.29	0.151	-0.09	-0.11	0.132	0.313	0.67	0.028	0.602853	0.114161	0.154145009	-0.039983701
1355	P01137	TGFB1	transforming growth factor beta 1	7040	ENSG00000105329	7	-0.19	-0.49	-0.24	-0.29	0.149	0.063	0.693	0.354	0.587	0.4854	0.1667	0.0976	-0.769	0.3812	-0.625	0.5838	-0.076	0.1903	-0.02	0.717	0.211	0.411	0.268	-0.08	0.521	0.341	0.009	0.589366	-0.19445	0.021415232	-0.215864098
2467	Q14642	INPP5A	inositol polyphosphate-5-phosphatase A	3632	ENSG00000068383	9	-0.16	0.061	-0.11	-0.67	1.014	-0.23	0.04	9E-04	0.053	-0.02	-0.029	0.0442	-0.077	-1.083	-0.893	0.3821	0.7273	0.0862	-0.27	-0.06	-0.42	-2.28	-0.91	0.547	-0.28	1.721	0.142	0.902008	0.200411	0.094904244	0.105506435
969	K7ERP1	ROGDI	rogdi atypical leucine zipper	79641	ENSG00000067836	4				-0.35	-0.31	-0.56	0.287	0.053	0.334				0.0203	0.0668	-0.16	0.0591	-0.191	0.1872				-0.64	-0.46	0.158	0.037	-0.01	-0.04	0.762461	0.06622	-0.089266057	0.155485883
3237	Q99988	GDF15	growth differentiation factor 15	9518	ENSG00000130513	9	-0.18	-0.38	0.049	0.055	-0.22	-0.65	-0.13	-0.25	1.104	0.4236	-0.721	0.7945	0.0959	-0.396	-0.833	1.2258	-0.766	-0.336	-0.19	0.593	-0.52	0.409	-0.41	0.196	0.684	0.102	-0.56	0.946685	-0.09982	-0.009101236	-0.090720149
1484	P07902	GALT	galactose-1-phosphate uridylyltransferase	2592	ENSG00000213930	8	0.066	0.029	-0.32	0.077	-0.06	-0.15	0.036	0.119	0.126	0.0853	-0.211	-0.214	0.212	-0.1	0.0894	0.182	0.1131	0.2616	0.218	0.331	-0.13	-0.59	-0.33	-0.08	0.211	0.305	0.09	0.908131	-0.01176	-0.055179052	0.043420439
3312	Q9BY44	EIF2A	eukaryotic translation initiation factor 2A	83939	ENSG00000144895	8	-0.02	0.03	-0.12	-0.2	-0.15	-0.53	-0.06	0.023	5E-04	-0.101	-0.282	0.1374	0.1211	-0.089	-0.07	0.027	0.0607	0.2243	-0.48	-0.87	-0.63	-0.18	-0.33	0.086	0.072	-0.04	0.039	0.224629	0.145162	-0.11766877	0.262830649
1493	P08236	GUSB	glucuronidase beta	2990	ENSG00000169919	5	0.194	-0.93	-0.41	-0.58	-0.4	0.274	-0.11	-0.08	0.078	0.3021	0.1339	-0.272	-0.58	0.2738	-0.298	-0.106	-0.412	-0.025	0.102	0.556	0.214	0.057	0.143	-0.24	0.251	0.627	-0.01	0.157602	-0.40611	-0.107647212	-0.298457828
2547	Q16186	ADRM1	adhesion regulating molecule 1	11047	ENSG00000130706	8	0.135	0.153	-0.1	-0.11	0.4	-0.3	-0.12	0.279	0.23	-0.19	-0.112	0.2205	-0.065	0.0172	0.0943	-0.018	-0.4	0.1123	0.017	-0.56	-0.16	-0.06	-0.26	0.015	0.081	0.599	0.072	0.739797	0.092706	0.101905135	-0.009198647
2074	P53609	PGGT1B	protein geranylgeranyltransferase type I sub	5229	ENSG00000164219	10	0.327	-0.02	-0.22	-0.06	0.019	-0.11	-0.11	0.069	0.107	0.3385	0.2344	0.2497	0.0842	0.066	-0.221	0.1954	-0.036	0.063	-0.04	-0.46	-0.14	0.272	0.018	0.201	0.171	0.128	0.215	0.615162	-0.03987	-0.108586367	0.06871218
1844	P34896	SHMT1	serine hydroxymethyltransferase 1	6470	ENSG00000176974	10	-0.01	-0.04	0.259	0.23	0.086	-0.2	0.109	0.297	0.18	0.0722	0.2008	0.3165	0.0703	-0.249	0.0135	-0.199	-0.067	-0.024	-0.17	-0.38	0.139	-0.33	-0.23	-0.04	0.41	0.018	-0.14	0.327011	0.182279	0.086797671	0.09548134
3078	Q969X5	ERGIC1	endoplasmic reticulum-golgi intermediate co	57222	ENSG00000113719	5	0.531	1.179	0.008	-0.02	0.272	-0.5	-0.11	0.331	0.475	0.3206	-0.089	0.1121	0.3242	-0.301	-0.005	-0.181	-0.157	0.1727	-0.03	-0.86	0.013	-0.53	-0.06	0.187	0.01	0.037	0.432	0.343641	0.328607	0.218940405	0.109666804
1062	O14976	GAK	cyclin G associated kinase	2580	ENSG00000178950	5	0.353	0.408	0.125	-0.33	-0.08	-0.08	0.439	0.113	0.163	0.1538	0.0498	0.1669	-0.002	0.1794	0.2333	0.4723	-0.127	0.229	0.029	-0.54	-0.22	0.128	0.078	-0.14	0.186	0.081	-0.1	0.26128	0.179869	-0.026608631	0.206477234
1043	O14737	PDCD5	programmed cell death 5	9141	ENSG00000105185	7	-0.31	-0.04	0.007	-0.02	0.025	-0.37	0.07	0.008	0.122	0.082	-0.325	-0.247	0.0374	-0.229	0.0817	0.171	0.1496	-0.142	-0.18	1.056	-0.76	-0.7	-0.34	-0.06	-0.04	-0.12	-0.08	0.890778	0.076833	-0.010354064	0.087187347
3101	Q96BX8	MOB3A	MOB kinase activator 3A	126308	ENSG00000172081	3				0.202	0.469	-0.37	0.489	0.79	0.212				0.1846	-0.396	-0.158	0.1485	0.2464	1.0103				-0.31	-0.38	0.212	0.061	-0.03	0.372	0.559696	0.310798	0.126226075	0.184571791
3158	Q96K76	USP47	ubiquitin specific peptidase 47	55031	ENSG00000170242	20	0.05	-0.63	-0.21	-0.15	-0.12	-0.2	-0.16	0.015	0.203	-0.194	0.3014	-0.183	-0.027	-0.078	-0.001	0.0198	0.1886	0.1279	0.543	0.47	0.943	-0.3	-0.05	0.045	0.006	0.238	0.194	0.142032	-0.36504	-0.15031181	-0.214725859
57	A0A2R8Y69	IQSEC1	IQ motif and Sec7 domain 1	9922	ENSG00000144711	7	0.024	0.28	-0.11	-0.18	-0	-0.44	-0.06	-0.04	0.217	0.0139	-0.294	0.1844	0.0841	0.0085	0.6023	0.0846	-0.03	0.0304	-0.51	-0.59	-0.4	-0.11	-0.27	-0.25	0.12	0.017	0.061	0.173168	0.178606	-0.112086839	0.29069263
2224	P62987	UBA52	ubiquitin A-52 residue ribosomal protein fusi	7311	ENSG00000221983	11	0.206	0.039	0.012				0.137	-0.41	0.283	-0.256	-0.211	-0.281				-0.155	0.5826	-0.018	-0.08	-0.83	-0.07				0.239	-0.31	-0.06	0.601405	0.23029	0.10138521	0.128904898
1701	P21217	FUT3	fucosyltransferase 3 (Lewis blood group)	2525	ENSG00000171124	14	-0.32	-1.01	-0.23	-0.26	-0.45	0.1	-1.23	0.172	-0.32	0.3369	0.5736	0.3644	-0.451	0.6079	-0.272	0.4717	-0.385	-0.46	-0.48	0.199	0.324	-0.21	0.098	0.044	0.384	0.347	-0.98	0.226202	-0.36253	-0.48156386	0.119036985
3662	Q9UM54	MYO6	myosin VI	4646	ENSG00000196586	66							1.076	1.624	1.266							1.2522	-0.089	0.4373							1.507	0.818	1.239	0.319221	0.133825	0.788330915	-0.654506328
2667	Q5XXA6	ANO1	anoctamin 1	55107	ENSG00000131620	21	-0.37	-0.01	0.015	-0.71	-0.31	0.102	-0.16	0.138	-0.14	-0.065	-0.068	-0.387	-0.204	0.0011	-0.346	-0.229	0.1131	-0.032	0.201	1.138	0.154	0.05	-0.04	-0.29	0.119	-0.15	-0.18	0.277449	-0.2739	-0.027306155	-0.246589747
2216	P62888	RPL30	ribosomal protein L30	6156	ENSG00000156482	5	0.26	-0.3	-0.07	0.024	0.101	-0.2	0.209	0.138	0.328	-0.261	-0.305	-0.203	-0.112	-0.339	-0.054	0.1661	-0.107	0.0862	-0.14	-0.56	-0.02	-0.06	-0.18	-0.1	0.39	0.124	0.217	0.404533	0.090678	0.179724973	-0.089046752
2963	Q8TAT6	NPLOC4	NPL4 homolog, ubiquitin recognition factor	55666	ENSG00000182446	5	0.261	0.055	0.004				-0.24	-0.13	-0.14	0.1883	-0.162	0.0065				-0.065	-0.213	-0.039	-0.14	-0.56	-0.2				0.044	-0.28	-0.21	0.291063	0.192215	0.01483318	0.177381998
724	H7C3P4	GNS	glucosamine (N-acetyl)-6-sulfatase	2799	ENSG00000135677	7	-0.16	-0.23	0.016	-0.18	-0.22	-0.5	-0.33	-0.2	-0.13	0.7912	-0.102	0.3675	-0.398	-0.288	-0.512	0.0199	-0.475	-0.436	-0.39	0.234	-0.27	-0.36	-0.08	-0.24	0.282	0.772	-0.82	0.850695	-0.11846	-0.100157811	-0.01830392
411	B1AL69	CDC37L1	cell division cycle 37 like 1	55664	ENSG00000106993	2							1.394	0.801	0.877							0.9413	0.3617	0.6841							0.205	0.302	1.024	0.433889	0.513739	0.36151429	0.152225172
1914	P41743	PRKCI	protein kinase C iota	5584	ENSG00000163558	9	0.101	0.175	-0.22	0.094	-0.12	-0.32	-0.2	-0.13	-0.03	0.0139	0.1619	-0.006	0.1851	-0.238	0.0082	0.248	0.1367	0.1061	-0.4	-0.87	-0.36	-0.35	-0.1	0.067	-0.17	-0.12	0.069	0.116564	0.174777	-0.141146563	0.315923392
2375	Q13057	COASY	Coenzyme A synthase	80347	ENSG00000068120	13	1.017	0.101	-0.14	0.021	-0.29	-0.32	0.272	0.216	0.256	0.0249	0.0371	-0.017	0.0811	-0.127	-0.192	0.3082	-0.047	0.1221	0.136	-0.58	0.85	-0.43	-0.2	0.138	0.174	0.154	0.371	0.881015	0.056359	0.104359957	-0.04800145
608	E7ER68	FAM91A1	family with sequence similarity 91 member /	157769	ENSG00000176853	12	0.034	0.13	-0.06	0.218	-0.09	-0.1	-0.03	-0.02	0.381	0.2842	0.1099	0.1386	0.2806	-0.011	-0.101	0.2484	0.1874	0.209	-0.28	-0.76	-0.1	-0.32	-0.2	0.132	0.17	-0.01	0.275	0.170245	0.171898	-0.098722212	0.270620045
321	P49815	TSC2	TSC complex subunit 2	7249	ENSG00000103197	11	0.071	0.66	-0.28	-0.04	-0.19	-0.3	0.28	0.185	0.326	0.013	-0.051	0.1497	-0.08	-0.002	-0.23	0.1464	-0.041	0.1538	-0.13	-0.53	-0.01	-0.31	-0.24	0.047	0.383	0.252	0.291	0.781173	0.106486	0.073285703	0.033200253
2965	Q8TB45	DEPTOR	DEP domain containing MTOR interacting ¡	64798	ENSG00000155792	5	0.005	-0.3	-0.2				-0.05	0.04	0.14	0.0372	0.1285	-0.001				-0.051	-0.047	-0.147	-0.14	0.112	0.059				0.163	-0.02	-0.05	0.669377	-0.08053	-0.046541328	-0.033986377
3258	Q9BS40	LXN	latexin	56925	ENSG00000079257	1	0.18	0.166	0.204	0.116	0.094	-0.53	0.245	0.085	0.278	0.3271	-0.158	0.0719	0.4819	-0.141	0.2395	0.1061	0.0996	-0.105	-0.35	-1.62	-0.25	-0.01	0.123	0.183	0.01	-0.17	0.003	0.256449	0.323244	-0.009839279	0.333083177
64	P50895	BCAM	basal cell adhesion molecule (Lutheran bloo	4059	ENSG00000187244	14	-0.43	-0.35	-0.21	-0.24	-0.35	0.034	-0.23	0.043	-0.13	0.0141	0.0747	0.0466	-0.361	0.0785	0.116	0.3953	-0.328	0.2276	-0.03	-0.31	0.068	-0.35	-0.2	-0.04	0.321	0.011	-0.49	0.233778	-0.09281	-0.234994681	0.14218417
1702	P21246	PTN	pleiotrophin	5764	ENSG00000105894	6	-0.33	-0.01	-0.41	-0.01	-0.85	0.082	-0.2	-0.26	0.01	######	0.2179	0.1808	-0.552	-0.17	0.3111	0.1942	0.1023	-0.039	-0.29	-0.4	0.148	-0.42	-0.78	-0.08	1.196	0.523	-0.08	0.558846	-0.19925	-0.246443491	0.04718951
2521	Q15404	RSU1	Ras suppressor protein 1	6251	ENSG00000148484	13	0.129	0.198	0.004	-0.14	0.065	-0.34	0.216	0.186	0.278	0.0758	0.1051	0.159	-0.034	0.0334	0.0768	0.3823	0.2057	0.3226	-0.03	-0.48	-0.06	-0.33	-0.29	0.079	0.117	0.005	0.28	0.202974	0.144382	-0.081258645	0.225640937
2146	P61018	RAB4B	RAB4B, member RAS oncogene family	53916	ENSG00000167578	7	-0.12	0.265	0.04	-0.22	0.026	-0.52	0.102	0.128	0.142	-0.015	-0.226	0.088	0.1531	-0.176	-0.313	0.1039	0.0305	0.1538	-0.1	-0.15	-0.09	-0.05	-0.1	-0.22	-0.07	0.093	0.185	0.924137	0.038973	0.0054834	0.033489376
2119	P56537	EIF6	eukaryotic translation initiation factor 6	3692	ENSG00000242372	5	0.071	0.291	0.189	0.134	0.214	-0.29	0.079	0.178	0.45	0.1149	0.0934	0.0156	-0.022	0.0966	0.2205	0.1986	0.0865	0.0038	-0.17	-0.78	-0.03	-0.23	-0.27	0.147	0.253	0.058	0.091	0.187932	0.248689	0.056015127	0.192673676
685	H0YCZ2	KNL1	kinetochore scaffold 1	57082	ENSG00000137812	2	-0.5	-1.95	-0.53	-0.77	-0.95	0.585	-0.7	-0.26	-0.38	-0.671	-0.915	-0.587	-1.952	-0.169	-0.564	-0.963	-0.266	-0.343		0.073	-0.27	-1.06	1.235	0.25	0.781	-0.5	-0.95	0.268646	-0.55148	0.107959411	-0.659434882
1235	O75886	STAM2	signal transducing adaptor molecule 2	10254	ENSG00000115145	12	0.1	0.297	0.008	-0.03	0.502	-0.38	-0.12	-0.07	0.01	-0.06	0.0479	0.1517	0.021	0.0744	0.2287	-0.007	0.1139	0.0546	-0.13	-0.48	-0.25	-0.2	-0.11	0.005	-0.09	-0.02	0.079	0.187825	0.167908	-0.033192958	0.201100473
1063	O14980	XPO1	exportin 1	7514	ENSG00000082898	12	0.089	0.154	0.098	0.035	0.034	0.059	-0.11	0.204	0.165	0.0879	-0.07	-0.063	-0.086	-0.067	0.0395	0.1187	0.0753	0.1654	-0.5	-1.4	-0.25	0.072	-0.18	0.139	0.061	0.109	0.1	0.265997	0.285848	0.058342071	0.227505654
1566	P12004	PCNA	proliferating cell nuclear antigen	5111	ENSG00000132646	5	-0.13	0.734	-0.3	-0.19	-0.18	-0.37	0.203	-0.33	0.127	0.144	-0.15	0.1212	-0.039	-0.149	-0.026	-0.159	-0.751	-0.095	0.037	-0.35	0.546	1.158	-0.41	-0.03	0.125	-0.34	-0.84	0.903376	-0.03628	0.074209484	-0.110485795
767	J3QRU4	VAMP2	vesicle associated membrane protein 2	6844	ENSG00000220205	10	-0.18	0.094	-0.1	-0.28	-0.06	-0.32	-0.36	-0.05	-0	0.1085	-0.061	0.0674	-0.015	-0.213	0.3344	-0.263	-0.359	-0.457	-0.12	-0.45	-0.14	-0.2	-0.4	-0.01	-0.1	-0.26	-0.14	0.622725	0.064447	-0.043470367	0.107917182
3377	Q9H3G5	CPVL	carboxypeptidase vitellogenic like;carboxype	54504	ENSG00000106066	7	0.09	-0.4	0.318	0.181	-0.11	0.314	-0.24	0.772	0.331	-0.39	-0.068	0.7542	-0.4	-0.463	-0.157	-0.468	0.3853	-0.73	-0.74	-0.74	-0.44	0.003	0.181	-0.32	-0.25	-0.04	-0.95	0.169437	0.504972	0.310212475	0.194759585
1039	O14656	TOR1A	torsin family 1 member A	1861	ENSG00000136827	8	-0.28	-0.44	-0.22	-0.5	0.034	-0.07	0.029	0.292	0.371	0.0716	-0.041	-0.225	-0.346	-0.182	-0.325	0.4494	0.0637	0.4232	-0.11	0.341	0.168	-0.06	-0.38	-0.28	0.331	0.399	-0.14	0.785267	-0.11873	-0.075239249	-0.043486074
3338	Q9GZU7	CTDSP1	CTD small phosphatase 1	58190	ENSG00000144579	5	0.123	0.595	0.01	0.304	-0.09	-0.3	0.037	-0.17	-0.21	0.0749	0.1659	0.3241	0.1954	-0.182	0.1404	0.5366	-0.215	0.5596	-0.27	-0.46	-0.4	-0.45	-0.32	-0.12	0.107	-0.01	0.208	0.132323	0.223597	-0.144768324	0.368364897
774	G3V1A6	GSDMD	gasdermin D	79792	ENSG00000278718	7	0.388	0.433	0.237	0.115	0.307	-0.12	-0.29	-0.58	-0.29	0.4752	0.4441	-0.135	0.5849	0.1844	0.1013	-0.311	-0.093	-0.375	-0.09	-0.83	-0.1	-0.39	-0.43	0.422	-0.05	-0.09	-0.14	0.374997	0.210697	-0.075217852	0.28591469
1256	O94817	ATG12	autophagy related 12	9140	ENSG00000145782	3	-0.19	-0.13	-0.15	-0.06	0.028	-0.55	0.073	0.027	0.284	-0.179	0.0178	0.0246	0.0734	-0.258	0.3028	-0.027	0.3687	0.1972	-0.54	-1.13	-0.12	-0.79	-0.43	-0.11	-0.18	-0.07	0.099	0.117975	0.289496	-0.132061172	0.421556935
3278	Q9BUT1	BDH2	3-hydroxybutyrate dehydrogenase 2	56898	ENSG00000164039	5	-0.23	-0.58	-0.45	0.072	-0.07	-0.07	0.046	-0	0.436	0.5416	-0.235	-0.196	-0.582	-0.532	0.4601	-0.131	-0.012	0.1752	0.397	-0.52	-0.24	-0.42	-0.53	-0.26	0.578	-0.42	0.693	0.9861	-0.01389	-0.037426826	0.023537191
3702	Q9Y259	CHKB	choline kinase beta	1120	ENSG00000100288	7	0.13	-0.16	-0.13	-0.15	0.198	-0.47	-0.1	-0.03	0.177	-0.056	-0.089	-0.098	-0.256	-0.197	0.034	0.1047	0.0956	0.1103	0.014	-0.05	0.07	-0.22	-0.25	-0.09	0.02	0.037	0.204	0.948215	-0.0302	-0.020929123	-0.009273394
2382	Q13155	AIMP2	aminoacyl tRNA synthetase complex interac	7965	ENSG00000106305	8	-0.17	-0.1	-0.11	0.36	0.303	-0.17	0.114	0.011	0.247	-0.124	-0.099	-0.057	0.0706	-0.288	0.0048	0.0846	0.1576	0.1826	-0.18	-0.5	-0.12	-0.16	-0.21	-0.07	0.538	-0.1	-0.01	0.535355	0.142994	0.060912394	0.082081852
913	Q9NPR2	SEMA4B	semaphorin 4B	10509	ENSG00000185033	7	-0.11	-0.44	-0.53	0.115	-0.15	0.298	-0.06	0.21	0.086	0.1189	-0.402	0.2049	-0.487	-0.063	-0.075	0.0575	0.1448	0.3056	0.414	0.47	0.044	-0.72	-0.83	-0.27	-0.33	0.067	0.905	0.981073	-0.03502	-0.041167943	0.006146846
2679	Q6DKJ4	NXN	nucleoredoxin	64359	ENSG00000167693	9	0.113	0.142	0.022	0.12	-0.07	-0.31	0.005	-0.18	0.305	0.2818	0.2126	-0.109	0.0381	-0.312	0.0195	0.026	0.1945	0.11	-0.05	-0.47	-0.14	-0.4	-0.17	-0.11	-0.01	0.278	0.418	0.618973	0.089726	-0.033819341	0.123545378
1954	P47224	RABIF	RAB interacting factor	5877	ENSG00000183155	2				0.118	-0.2	-0.43	0.535	-0.09	0.186				0.1566	-0.171	-0.014	0.049	0.1635	0.1529				0.373	-0.39	-0.2	-0.16	-0.06	0.353	0.934934	0.036073	-0.035407779	0.071481243

3343	Q9H082	RAB33B	RAB33B, member RAS oncogene family	83452	ENSG00000172007	5	-0.17	0.231	0.013	-0.15	-0.1	-0.31	-0.46	-0.28	-0.1	0.2851	0.1934	-0.003	-0.109	-0.127	0.2619	-0.014	-0.157	-0.363	-0.16	-0.55	-0.34	-0.32	-0.38	0.151	-0.02	0.183	-0.13	0.392221	0.02346	-0.145796797	0.169256606
892	H7C5G1	IAH1	isoamyl acetate hydrolyzing esterase 1 (puta	285148	ENSG00000134330	9	0.13	0.128	-0.25	0.102	0.047	-0.35	0.248	0.285	0.381	-0.073	-0.105	0.067	-0.099	-0.01	0.1112	0.3792	0.144	0.1009	-0.22	-0.84	-0.21	-0.7	-0.46	-0.09	0.001	0.079	0.107	0.118773	0.338951	0.023180487	0.315770914
1822	P31431	SDC4	syndecan 4	6385	ENSG00000124145	2				-0.03	-0.42	-0.02	-0.23	-0.02	-0.16				0.034	0.4152	0.1429	-0.022	0.3591	-0.171				-0.19	-0.18	0.119	0.119	0.167	0.065	0.197894	-0.16359	-0.272288827	0.108700916
3108	Q96CF2	CHMP4C	charged multivesicular body protein 4C	92421	ENSG00000164695	10	0.039	0.085	-0.06	0.024	0.029	-0.28	-0.39	-0.07	-0.19	0.0854	-0.126	-0.009	0.0818	0.0091	-0.041	0.0536	-0.108	0.2129	-0.01	-0.09	-0.25	-0.48	-0.34	-0.13	-0.11	-0.07	0.36	0.380653	0.034121	-0.107828531	0.141949459
1074	O15143	ARPC1B	actin related protein 2/3 complex subunit 1B	10095	ENSG00000130429	16	-0.01	0.233	-0.18	-0.34	0.012	-0.7	-0.49	-0.29	-0.27	0.0298	-0.124	-0.057	-0.071	0.0185	0.0778	-0.227	0.118	-0.262	-0.13	-0.31	0.184	0.272	-0.11	0.027	-0.15	-0.02	-0.18	0.291174	-0.17894	-0.170605026	-0.008336287
2583	Q3ZM63	ETDA	embryonic testis differentiation homolog A	101928677	ENSG00000238210	1	-0.16	0.002	-0.22	-0.02	0.158	-0.44	-0.32	-0.32	-0.27	-0.164	-0.035	-0.128	0.1257	-0.222	0.0509	0.3147	0.0393	-0.13	-0.24	-0.8	-0.22	-0.54	-0.44	-0.01	0.464	0.079	-0.24	0.395148	0.040867	-0.160794533	0.201661792
2818	Q86X10	RALGAPB	Ral GTPase activating protein non-catalytic	57148	ENSG00000170471	21	0.127	0.127	-0.22	0.039	0.075	-0.09	-0.01	0.053	0.144	0.1155	0.0791	0.1628	0.2443	-0.064	0.0387	0.255	0.0648	0.2147	-0.28	-0.47	-0.27	-0.16	-0.15	0.037	0.101	0.105	0.209	0.131899	0.125627	-0.094758969	0.220386279
618	E7EVJ5	CYFIP2	cytoplasmic FMR1 interacting protein 2	26999	ENSG00000055163	23	0.522	0.277	0.107	0.09	-0.01	-0.25	-0.01	-0.02	0.25	-0.057	-0.188	0.1977	0.0593	-0.212	-0.121	-0.026	0.1479	0.2362	-0.28	-0.89	-0.18	-0.23	-0.26	0.037	-0.15	-0.14	0.118	0.122199	0.324214	0.102496102	0.221717489
3484	Q9NRN7	AASDHPP	aminoadipate-semialdehyde dehydrogenase	60496	ENSG00000149313	5	0.104	0.25	0.007	0.044	0.213	-0.26	-0.13	-0.02	0.004	0.1416	0.0806	0.1286	0.0666	0.0973	0.1664	-0.137	-0.082	-0.081	-0.25	-0.61	-0.09	-0.22	-0.11	0.044	0.278	-0.17	0.094	0.309265	0.139173	-0.01908549	0.158258958
3367	Q9H255	OR51E2	olfactory receptor family 51 subfamily E mer	81285	ENSG00000167332	2	0.081	0.104	0.216	-0.43	-0.26	-1.35	-1.32	-1.06	-0.13	0.2889	0.1307	0.4525	0.2979	-0.499	-0.92	-0.746	0.0521	0.5995	0.56	-0.12	-0.07	-0.91	-1.39	-0.12	-0.35	0.209	0.392	0.489431	-0.25975	-0.422545294	0.162800086
82	A0A087WZF	NID2	nidogen 2	22795	ENSG00000087303	10	0.008	-0.98	0.113	0.302	0.145	-0.58	-0.17	0.197	0.016	-0.673	0.1815	-0.248	-0.02	0.3623	0.226	0.2566	-0.033	0.2153	-0.07	-0.22	0.142	1.227	-0.19	0.196	0.619	-0.04	-0	0.485715	-0.291	-0.135696046	-0.155301362
1546	P10600	TGFB3	transforming growth factor beta 3	7043	ENSG00000119699	4	0.1	-0.26	-0.03	0.278	0.473	-0.11	-0.45	-0.11	0.344	-0.704	-0.018	0.3771	0.8183	-1.482	-0.088	0.6408	-0.23	0.4263	-0.3	-0.21	-0.47	-1.02	-0.86	-0.66	0.816	0.526	-0.06	0.685937	0.273092	0.053594296	0.219497824
2354	Q10469	MGAT2	mannosyl (alpha-1,6-)-glycoprotein beta-1,2	4247	ENSG00000168282	3	0.076	0.218	-0.17				0.211	-0.25	-0.07	0.0545	-0.076	0.0389				0.0656	0.0723	0.2196	-0.37	0.002	-0.2				-0.08	-0.41	-0.04	0.174995	0.182454	-0.060241151	0.242694779
998	O00264	PGRMC1	progesterone receptor membrane componer	10857	ENSG00000101856	5	-0.12	-0.43	-0.3	-0.2	-0.23	-0.15	-0.2	-0.13	0.084	0.1817	0.1711	-0.182	-0.229	0.1251	0.0764	0.0766	-0.071	-0.068	-0.04	0.932	0.102	-0	-0.22	-0.05	0.049	-0.11	0.13	0.171836	-0.2728	-0.194981152	-0.077817444
199	A0A0C4DG	CUL1	cullin 1	8454	ENSG00000055130	14	-0.03	-0.03	-0.15	0.049	-0.04	-0.17	-0.11	-0.12	-0.01	-0.101	-0.073	0.0139	0.0187	0.0803	0.1519	0.026	0.1153	-0.09	-0.23	-0.8	-0.23	-0.35	-0.22	0.008	0.012	-0.02	0.107	0.18148	0.122919	-0.084844595	0.207763429
121	A0A087X2D	SPAG9	sperm associated antigen 9	9043	ENSG00000008294	11	0.08	0.092	-0.06	-0.21	0.115	-0.12	-0.08	0.087	0.111	0.0724	-0.005	0.1345	-0.31	-0.127	0.0644	0.1714	0.1525	0.1086	-0.18	-0.68	-0.18	-0.57	-0.04	0.052	0.153	0.026	0.081	0.313548	0.149785	-0.027185771	0.176971033
706	F5H1S8	MLEC	malectin	9761	ENSG00000110917	4				-0.24	0.129	-0.21	-0.27	-0.06	0.134				-0.03	0.1402	-0.163	-0.102	-3E-04	-0.043				-0.42	-0.25	-0.13	-0.1	0.029	-0.02	0.595486	0.061014	-0.052985475	0.113999503
540	C9JZY6	UBE2H	ubiquitin conjugating enzyme E2 H	7328	ENSG00000186591	4	0.191	0.417	0.164	-0.28	-0.4	0.209	-0.1	0.08	0.078	0.3322	0.1135	0.22	-0.329	0.1522	0.0029	0.1926	0.1	0.3573	0.101	-0.53	-0.16	-0.46	-0.35	-0.18	-0.04	0.218	0.295	0.270647	0.163677	-0.086483287	0.250160151
3366	Q9H246	C1orf21	chromosome 1 open reading frame 21	81563	ENSG00000116667	6	-0.11	0.357	-0.25	0.027	-0.35	-0.58	-0.03	0.083	0.179	-0.536	0.123	-0.11	-0.186	0.182	-0.004	0.1269	-0.17	0.038	-0.67	-0.21	-0.35	-0.51	-0.68	-0.52	0.336	0.068	0.161	0.450215	0.190342	-0.014806888	0.205148486
418	C9JVB2	KIAA0319L	KIAA0319 like	79932	ENSG00000142687	3	-0.27	-0.46	-0.3	-0.26	-0.37	-0.22	0.116	-0	0.192	0.065	0.2663	-0.022	-0.42	0.3121	-0.134	0.0461	-0.614	-0.301	0.09	0.237	0.085	-0.34	-0.09	-0.15	0.554	0.223	0.194	0.267767	-0.26436	-0.086163011	-0.178195998
404	B0YJC4	VIM	vimentin	7431	ENSG00000026025	14	-0.15	-0.5	-0.63	-0.54	-0.63	-0.52	-0.18	0.104	-0.13	-0.614	-0.364	-0.443	-0.658	-0.396	-0.608	0.5689	-0.4	-0.214	-0.51	-0.84	1.42	2.169	-0.73	0.401	-0.04	-0.08	-0.31	0.32126	-0.51674	-0.005423602	-0.511318724
2485	Q14997	PSME4	proteasome activator subunit 4	23198	ENSG00000068878	11	0.263	-0.01	-0.07	0.247	0.351	0.343	0.167	0.605	0.263	-0.269	-0.528	-0.206	-0.126	-0.056	-0.202	0.0137	-0.012	0.1748	-0.76	-0.56	-0.4	0.88	0.468	0.402	-0.06	-0.3	-0.24	0.209539	0.302444	0.374605759	-0.072161404
2211	P62854	RPS26	ribosomal protein S26	6231;10192	ENSG00000197728	3	0.066	-0.1	-0.16	-0.17	0.568	-0.35	0.29	-0.06	0.199	-0.152	-0.095	-0.245	-0.141	-0.239	-0.031	0.0995	0.3151	0.2056	-0.28	-0.65	-0.08	-0.23	-0.03	0.058	0.359	-0.21	0.013	0.613217	0.148134	0.062306923	0.085826882
1517	P09488	GSTM1	glutathione S-transferase mu 1	2944	ENSG00000134184	17	-2.5	1.77	-2.06	-2.05	1.528	1.171	-1.88	-2.34	2.217	-1.792	-1.456	-1.927	-1.76	-1.653	-1.134	-2.05	-2.187	-2.256	0.882	0.909	1.644	-0.41	-1.06	-0.09	-1.96	-1.05	-2.18	0.211413	-0.0922	1.341419273	-1.433614924
3671	Q9UNE2	RPH3AL	rabphilin 3A like (without C2 domains)	9501	ENSG00000181031	7	0.237	0.377	-0.19	-0.07	-0.43	-0.35	1.167	0.318	0.377	-0.054	0.1149	-0.121	-0.026	-0.21	-0.121	0.1567	0.365	0.3536	-0.17	-0.35	0.099	-0.28	-0.43	0.03	-0.06	0.191	1.367	0.881222	0.116643	0.109061909	0.00758139
271	A0A1B0GU	KIF1BP	KIF1 binding protein	26128	ENSG00000198954	10	0.076	0.171	0.031	-0.02	0.047	-0.29	0.112	0.237	0.44	0.1011	-0.018	-0.073	0.0854	-0.18	0.103	0.1231	0.0785	-0.094	0.026	-0.71	-0.11	-0.42	-0.17	0.033	0.322	0.092	0.33	0.532687	0.155942	0.074816334	0.081125487
1239	O75923	DYSF	dysferlin	8291	ENSG00000135636	4	-0.21	-0.93	-0.38	0.655	-0.54	0.469	-0.15	0.122	0.259	-0.243	-0.129	-0.137	-0.365	0.7012	0.0723	0.6099	-0.004	-0.484	-0.74	-0.03	-0.19	-0.09	-0.4	-0.26	0.355	0.216	-0.41	0.780694	0.092347	-0.081532321	0.173879264
2941	Q8NEV8	EXPH5	exophilin 5	23086	ENSG00000110723	8	0.048	0.067	0.203	-0.15	-0.2	0.33	-0.06	0.192	0.086	0.1226	0.1986	0.1858	-0.209	0.3033	-0.095	-0.201	-0.635	-0.428	-0.1	0.027	0.101	0.181	0.334	-0.04	0.812	-0.54	-0.08	0.603673	-0.02051	0.14083185	-0.16134575
2041	P51452	DUSP3	dual specificity phosphatase 3	1845	ENSG00000108861	7	-0	0.042	0.1	0.071	-0.01	-0.37	-0.02	-0.11	0.241	0.0927	0.1229	0.2385	0.0623	-0.235	0.2877	-4E-04	-0.167	-0.101	-0.04	-0.98	-0.1	-0.48	-0.2	0.131	-0.06	0.025	0.068	0.310443	0.177696	-0.039094832	0.216790383
3095	Q96BJ8	ELMO3	engulfment and cell motility 3	79767	ENSG00000102890	9	-0.02	0.192	0.075	-0.19	0.024	-0.24	0.012	-0.12	0.23	0.0726	0.0829	0.1988	0.0221	0.0064	0.0619	0.1631	0.2452	0.3182	-0.27	-0.65	0.04	-0.46	-0.32	0.012	0.09	0.078	0.163	0.1344	0.142474	-0.134077306	0.276551573
2997	Q8TF74	WIPF2	WAS/WASL interacting protein family meml	147179	ENSG00000171475	6	0.033	0.598	-0.15	-0.04	0.134	-0.1	-0.21	-0.27	0.113	-0.088	0.1146	0.3955	-0.026	0.0461	0.1547	0.053	0.081	0.3634	-0.16	0.104	-0.06	-0.35	-0.13	-0.19	0.109	0.048	0.166	0.356622	0.064483	-0.110152514	0.174635384
47	P15941	MUC1	mucin 1, cell surface associated	4582	ENSG00000185499	4	-0.77	-0.5	-0.27	-1.11	-0.72	-0.4	-0.62	-0.55	-0.36	-0.604	-0.015	-0.283	0.5725	-0.967	-0.144	-0.642	-0.003	-0.296	-0.91	0.327	0.442	1.955	0.385	0.913	0.387	-0.46	-0.61	0.116694	-0.86018	-0.325720303	-0.534455045
1342	P01019	AGT	angiotensinogen	183	ENSG00000135744	7	-0.57	-0.34	-0.51	-0.71	-0.13	-1.13	-0.45	0.092	0.302	0.1921	-0.44	0.7051	-0.601	-0.806	-0.676	0.6675	-0.36	-0.154	-0.48	0.494	-0.19	-0.09	-0.87	-0.01	0.415	1.13	0.004	0.411561	-0.42582	-0.218606675	-0.20721774
309	Q5SWX3	CAMK2G	calcium/calmodulin dependent protein kinas	818	ENSG00000148660	13	-0.16	-0.22	-0.27	0.073	-0.01	-0.24	-0.27	-0.12	-0.14	0.1571	-0.176	-0.094	0.1286	-0.086	-0.027	-0.059	-0.171	-0.289	-0.13	-0.21	-0.21	-0.19	-0.15	0.145	0.236	-0.05	-0.16	0.566145	-0.07236	-0.081817993	0.0094567
917	J3KPH2	ALOXE3	arachidonate lipoxygenase 3	59344	ENSG00000179148	1	0.471	0.557	0.33	-1.1	-0.14	-0.5	-0.18	-0.32	-0.44	0.6159	0.0345	-0.23	0.4637	-0.38	-0.268	-0.766	-0.627	-0.192	-0.35	-0.49	-0.04	-0.84	-0.08	0.353	0.588	-0.38	-0.65	0.969124	0.063234	0.003616149	0.05961756
447	H0Y9V7	ATP2C1	ATPase secretory pathway Ca2+ transportir	27032	ENSG00000017260	10	-0.4	0.686	-0.07	-0.1	-0.59	0.119	-0.28	0.248	0.004	0.5275	-0.204	0.17	0.2497	-0.054	-0.252	0.3443	-0.518	-0.078	-0.43	-0.12	-0.44	-0.57	-0.17	0.142	-0.11	0.788	0.149	0.891367	0.042103	-0.063097117	0.105200425
1699	P20933	AGA	aspartylglucosaminidase	175	ENSG00000038002	4	-0.26	-0.58	-0.06	-0.3	-0.34	-0.15	-0.21	0.081	0.141	0.5668	0.0585	0.16	-0.246	-0.424	-0.891	0.1024	-0.226	-0.306	-0.18	0.084	0.148	-0.52	0.304	-0.48	0.061	0.738	-0.53	0.788876	-0.14622	-0.053172954	-0.093044288
687	E9PQY2	PFDN4	prefoldin subunit 4	5203	ENSG00000101132	2	0.153	0.338	-0.05	0.084	0.241	-0.57	0.277	0.077	0.178	0.0859	-0.073	-0.06	0.0088	-0.039	0.1935	0.2878	0.2937	0.1439	-0.1	-1.07	-0.27	-0.53	-0.26	-0.03	-0.15	0.084	0.172	0.142865	0.321143	-0.011887457	0.33303093
789	G5E9C8	SOS1	SOS Ras/Rac guanine nucleotide exchange	6654	ENSG00000115904	2							0.552	-0.04	0.53							0.4834	0.317	0.3952							0.187	0.292	0.218	0.722641	0.115608	-0.050801472	0.166409194
1206	O75368	SH3BGRL	SH3 domain binding glutamate rich protein l	6451	ENSG00000131171	9	0.188	0.223	0.08	-0.14	0.163	-0.59	-0.56	-0.13	0.049	0.1532	0.2584	0.0035	0.0575	-0.212	0.125	-0.299	0.2769	-0.356	-0.18	-0.69	-0.11	-0.77	-0.58	0.004	-0.18	-0.07	-0.24	0.199166	0.233569	-0.080574729	0.314144169
2253	P78310	CXADR	CXADR Ig-like cell adhesion molecule;CXAI	1525	ENSG00000154639	3	-0.34	0.084	0.714	-0.59	-0.15	0.444	0.301	0.508	0.248	-0.233	0.7114	-0.097	-0.13	0.2487	-0.087	-0.05	0.0269	0.2676	-0.43	0.756	0.073	-0.49	-0.45	-0.44	0.569	0.101	0.12	0.801046	0.155023	0.061632663	0.093389899
3097	Q96BN8	OTULIN	OTU deubiquitinase with linear linkage spec	90268	ENSG00000154124	8	0.169	0.128	-0.07	-0.03	-0.07	-0.34	-0.38	-0.23	0.031	0.0629	0.1078	0.2295	0.0989	0.026	0.0955	-0.143	0.133	-0.092	-0.27	-0.44	-0.26	-0.63	-0.29	0.053	-0.13	-0.04	0.085	0.121271	0.127352	-0.145373517	0.272725644
992	O00178	GTPBP1	GTP binding protein 1	9567	ENSG00000100226	11	0.102	0.178	-0.03	-0.17	-0.32	-0.41	0.362	0.09	0.399	0.2721	0.0575	0.0608	-0.131	-0.038	-0.066	0.353	0.2089	0.245	-0.13	-0.78	-0.22	-0.31	-0.29	-0.18	0.377	0.321	0.239	0.447313	0.130705	-0.085633628	0.216338806
1414	P04217	A1BG	alpha-1-B glycoprotein	1	ENSG00000121410	6	-0.65	-0.21	-0.29	-0.05	0.169	-0.1	0.086	0.501	-0.11	-0.23	-0.489	-0.337	-0.37	-0.678	-0.158	-0.22	-0.077	-0.185	-0.25	-0.38	-0.06	0.833	0.107	0.135	0.602	-0.07	-0.6	0.256771	-0.10815	0.232017795	-0.340165508
2380	Q13144	EIF2B5	eukaryotic translation initiation factor 2B sub	8893	ENSG00000145191	8	0.166	0.211	-0.13	-0.03	0.031	-0.23	0.242	0.19	0.122	0.189	-0.064	0.0887	0.1164	-0.051	0.0976	0.0299	0.0915	0.1259	-0.25	-0.79	-0.12	-0.35	-0.22	-0.01	0.262	0.02	0.213	0.217134	0.201652	-0.005777424	0.207429619
85	A0A087X0C	ABO	ABO, alpha 1-3-N-acetylgalactosaminyltrans	28	ENSG00000175164	9	-0.19	0.102	-0.24	-1.04	0.947	0.389	0.804	-0.89	-0.79	0.0515	0.3147	0.9655	1.1854	-0.538	-0.684	1.3165	-1.124	-0.861	-0.89	0.212	-0.32	-0.57	-1.23	-1.15	0.391	0.618	-0.53	0.609392	0.282923	-0.171243215	0.45416621
2960	Q8NI22	MCFD2	multiple coagulation factor deficiency 2	90411	ENSG00000180398	1	0.226	-1.28	0.13	-0.68	-0.01	0.073	-1.07	-0.76	-0.97	0.3157	0.6041	0.2192	-0.224	0.2714	-0.154	-0.965	-0.639	-0.966	-0.23	0.399	0.619	-0.52	0.476	0.136	0.549	-0.58	-1.02	0.409646	-0.46147	-0.310500852	-0.150967384
967	K7ERF1	EIF3K	eukaryotic translation initiation factor 3 subu	27335	ENSG00000282986	6	0.102	0.132	0.002	-0.03	0.308	-0.26	0.191	0.207	0.301	0.1166	0.1312	0.0165	0.2829	-0.41	0.1064	0.2007	0.1481	0.309	-0.1	-0.42	-0.06	0.111	-0.02	0.158	0.501	0.194	0.308	0.967586	0.031108	0.005385892	0.025722391
2913	Q8N8Y2	ATP6V0D2	ATPase H+ transporting V0 subunit d2	245972	ENSG00000147614	1	-0.4	-0.05	0.134				0.326	0.211	0.327	-0.109	-0.12	0.0279				0.0861	0.2676	0.3225	-0.37	-0.92	-0.37				0.044	0.066	0.315	0.374819	0.298195	0.012232858	0.28596214
2497	Q15084	PDIA6	protein disulfide isomerase family A membe	10130	ENSG00000143870	7	0.177	-0.5	-0.1	0.212	0.367	0.095	0.075	0.157	-0.2	0.1151	-0.137	-0.071	-0.249	-0.025	0.0077	-0.196	-0.343	-0.314	-0.16	0.07	0.276	-0.12	-0.03	-0.26	0.404	-0.35	-0.57	0.511457	0.115744	0.16681627	-0.051072348
775	G3V203	RPL18	ribosomal protein L18	6141	ENSG00000063177	7	-0.1	-0.2	-0.08	0.016	0.627	-0.23	-0.16	-0.23	-0.21	-0.046	-0.077	-0.267	-0.151	-0.178	-0.114	-0.078	-0.015	-0.138	-0.25	-0.26	0.039	-0.16	-0.05	-0.02	0.078	-0.36	-0.3	0.763419	0.078944	0.05438262	0.024561489
373	A6NFX8	NUDT5	nudix hydrolase 5	11164	ENSG00000165609	5	-0.12	-0.4	-0.31	0.069	-0.12	-0.57	0.046	0.181	0.072	-0.218	0.0105	0.0067	-0.214	-0.182	-0.068	0.2024	0.2092	0.1181	-0.24	-0.49	-0.13	0.363	-0.45	-0.13	-0.02	-0.07	0.267	0.704126	-0.02759	-0.112632937	0.085042418
3532	Q9NX62	IMPAD1	inositol monophosphatase domain containin	54928	ENSG00000104331	1							-0.2	0.041	0.143							0.1406	0.0858	0.2984							0.102	-0.15	0.328	0.645498	-0.09862	-0.181588636	0.082964872
3724	Q9Y2T2	AP3M1	adaptor related protein complex 3 subunit m	26985	ENSG00000185009	8	0.121	0.243	-0.01	-0.06	-0.01	-0.31	0.115	0.128	0.245	0.0319	-0.134	-0.019	0.1037	-0.15	0.1018	0.0853	0.0743	0.1356	-0.22	-1.18	-0.27	-0.44	-0.3	-0.04	0.349	0.001	0.298	0.299495	0.2508	0.025088716	0.225711621
2488	Q15012	LAPTM4A	lysosomal protein transmembrane 4 alpha	9741	ENSG00000068697	2	-0.15	-0.03	-0.05	0.089	0.212	0.374	0.227	0.536	0.175	-0.132	0.1666	0.0799	-0.004	0.1633	0.3164	-0.087	0.4412	0.3838	-0.38	-0.51	-0.2	-0.53	-0.09	0.386	0.284	0.259	0.66	0.565241	0.167691	0.006131149	0.161559834
2005	P49768	PSEN1	presenilin 1	5663	ENSG00000080815	9	-0.03	0.34	0.01	-0.13	0.027	-0.3	-0.43	-0.23	-0.24	0.1012	0.2362	0.1532	0.1852	-0.093	0.0335	0.166	0.1368	0.0115	-0.26	-0.24	-0.2	-0.32	-0.37	-0.07	0.143	-0.18	-0.03	0.081402	0.059824	-0.212835006	0.272658967
3451	Q9NP94	SLC39A2	solute carrier family 39 member 2	29986	ENSG00000165794	3	-0.78	0.708	-0.57	-0.56	0.062	0.191	-0.67	-0.15	-0.48	0.5105	0.2488	-0.183	-0.105	-0.191	-0.399	-0.642	-0.388	0.0216	0.444	0.503	-0.1	0.414	0.146	0.145	-0.27	-0.85	-0.32	0.592684	-0.26092	-0.124032262	-0.136883464
3196	Q96T51	RUFY1	RUN and FYVE domain containing 1	80230	ENSG00000176783	6	0.115	0.18	0.032	0.312	-0.05	-0.11	0.171	0.123	0.234	0.0822	0.0414	0.0792	0.0437	0.0166	0.2716	0.2924	0.1774	0.1706	-0.18	-0.81	-0.35	-0.4	-0.12	0.709	0.235	0.295	0.516	0.674277	0.123357	-0.018096393	0.141453414
1357	P01591	JCHAIN	joining chain of multimeric IgA and IgM	3512	ENSG00000132465	7	-1.26	-1.39	-0.81	-1.1	-1.2	-0.66	-1.95	-1.42	-2.16	-1.363	-0.527	-1.006	-1.113	-0.59	-0.961	-0.932	-1.727	-2.056	-0.73	-0.52	2.562	2.355	0.098	1.337	-0.73	-1.76	-2.09	0.124559	-1.3863	-0.186456786	-1.199838376
2649	Q5TEC6	HIST2H3P	histone cluster 2 H3 pseudogene 2	440686	ENSG00000273213	6	-0.7	-0.71	-0.31	0.421	0.305	1.226	0.575	-0.29	0.73	-1.346	-0.641	-1.947	-1.417	-1.594	-2.13	-1.067	-0.973	-2.207	-1.26	-0.5	2.369	2.015	-0.68	0.892	-1.35	-1.96	-2.41	0.114073	0.458328	1.61890926	-1.160581181
763	F8WBA3	PRKD1	protein kinase D1	5587	ENSG00000184304	9	0.212	0.081	-0.25	0.043	0.101	-0.35	0.07	-0.24	0.037	0.0211	0.1258	0.2483	0.1275	-0.053	0.2236	-0.001	0.0898	0.1122	-0.12	-0.78	-0.39	2E-04	0.09	0.209	0.127	0.072	0.291	0.481106	0.02395	-0.131290933	0.155241186
1968	P48163	ME1	malic enzyme 1	4199	ENSG00000065833	6	0.109	0.066	0.085	-0.24	0.257	-0.27	-0.06	-0.06	-0.05	0.0515	-0.213	0.215	-0.277	-0.197	-0.061	0.0679	-0.135	-0.107	-0.34	-0.4	-0.16	-0.39	-0.01	-0.24	0.571	0.004	0.11	0.828843	0.077974	0.055594372	0.022379841
3358	Q9H1H9	KIF13A	kinesin family member 13A	63971	ENSG00000137177	14	-0.13	0.208	-0.09	-0.12	-0.35	-0.33	0.19	-0.15	0.349	0.3546	0.0787	0.1477	-0.016	0.4474	-0.153	-0.028	0.2188	-0.076	-0.33	-0.9	-0.26	0.215	-0.25	-0.2	-0.05	0.158	0.218	0.29156	0.108226	-0.156396787	0.264622967
2234	P63172	DYNLT1	dynein light chain Tctex-type 1	6993	ENSG00000146425	3	-0.14	-0.44	-0.33				0.039	-0	0.069	-0.165	0.157	0.018				0.6811	0.082	-0.115	-0.36	-0.57	-0.35				-0.07	0.342	-0.04	0.358164	0.040337	-0.243614553	0.283951713
2270	P84085	ARF5	ADP ribosylation factor 5	381	ENSG00000004059	11	-0.06	0.283	0.087	-0.04	0.788	-0.29	-0.48	-0.48	-0.25	0.1972	0.0252	0.034	0.1804	-0.157	-0.01	-0.369	-0.085	-0.488	-0.35	-1.01	-0.35	-0.51	-0.1	0.084	-0.46	-0.34	-0.32	0.211304	0.323842	0.026414097	0.297427825
3092	Q96AT9	RPE	ribulose-5-phosphate-3-epimerase	6120	ENSG00000197713	4	-0.04	0.223	-0.02	0.009	0.132	-0.37	0.433	0.298	0.223	-0.178	-0.043	0.1221	-0.083	-0.166	-0.058	0.0868	0.1269	0.0103	-0.27	-1.26	-0.25	-0.55	-0.27	-0.1	-0.04	0.014	0.11	0.1289	0.388138	0.119493706	0.268644241
2345	Q08752	PPID	peptidylprolyl isomerase D	5481	ENSG00000171497	10	-0.02	-0.05	-0.02	-0.05	0.26	-0.28	-0.2	-0.11	-0.03	-0.061	-0.06	0.0096	0.3241	0.1458	0.2294	-0.032	-0.013	-0.031	-0.22	-0.64	-0.12	-0.41	0.108	0.2	0.06	-0.11	2E-04	0.303365	0.071245	-0.112741076	0.183986529
17	H0YN88	RPS17	ribosomal protein S17	6218	ENSG00000182774	6	0.065	-0.18	-0.04	-0.07	-0	0.096	0.126	-0.12	0.209	-0.082	-0.03	-0.229	-0.358	-0.04	-0.013	-0.197	0.1857	0.162	-0.16	-0.48	0.223	0.508	0.029	-0	0.187	-0.19	0.054	0.738195	-0.00889	0.076488723	-0.08538364
1589	P13667	PDIA4	protein disulfide isomerase family A membe	9601	ENSG00000155660	12	0.117	-0.24	-0.03	-0.15	0.86	-0.22	-0.16	0.089	-0.16	0.1723	0.148	0.0676	-0.097	-0.033	-0.372	-0.317	-0.633	-0.644	-0.21	0.027	0.08	0.396	0.367	-0.05	0.458	-0.11	-0.57	0.444163	-0.03106	0.201570842	-0.232627031
3580	R4GMW6	COMMD3-	COMMD3-BMI1 readthrough	100532731	ENSG00000269897	8	-0.05	-0.09	0.088	-0.09	-0.1	-0.45	-0.06	0.051	0.162	0.004	0.0981	0.012	0.0495	-0.113	0.0003	-0.037	0.174	0.2836	-0.14	-0.63	0.009	-0.05	-0.2	0.079	-0.1	-0.07	0.169	0.339859	0.042589	-0.112441978	0.155031427
3666	Q9UN36	NDRG2	NDRG family member 2	57447	ENSG00000165795	10	-0.17	-0.13	-0.18	-0.16	0.166	-0.25	-0.02	-0.08	-0.07	0.3986	0.1347	0.0315	0.097	0.0079	0.1809	-0.038	0.2552	0.0473	-0.14	-0.68	-0.14	-0.38	-0.06	0.489	-0.17	-0.01	0.317	0.220204	-0.01252	-0.222181076	0.209663156
1766	P27361	MAPK3	mitogen-activated protein kinase 3	5595	ENSG00000102882	11	0.015	0.004	-0.2	-0.1	0.172	-0.43	-0.45	0.375	-0.21	0.2191	-0.082	-0.082	0.0941	-0.146	0.1298	-0.129	0.3532	-0.002	-0.12	-0.64	-0.04	-0.46	-0.29	0.12	-0.03	0.076	0.172	0.462334	0.043811	-0.130385392	0.174196843
733	F8VR84	C12orf10	chromosome 12 open reading frame 10	60314	ENSG00000139637	4	0.104	0.027	0.013	-0.11	0.127	-0.28	0.142	0.273	0.096	-0.135	-0.126	-0.002	-0.014	-0.261	-0.225	-0.082	0.0824	-0.11	-0.31	-0.93	-0.43	-0.37	-0.39	0.094	0.081	0.013	-0	0.148833	0.293437	0.139870214	0.153566671
3461	Q9NQ40	SLC52A3	solute carrier family 52 member 3	113278	ENSG00000101276	6	-0.36	-0.04	-0.69	-0.12	-0.24	-0.27	0.019	-0.56	-0.31	0.1578	0.1547	0.6042	-0.944	-0.466	0.7922	0.8173	0.0283	0.4681	-0.01	0.491	-0.57	-0.36	-1.01	0.27	0.188	0.149	0.255	0.266178	-0.21932	-0.464802236	0.245480583
2006	P49770	EIF2B2	eukaryotic translation initiation factor 2B sub	8892	ENSG00000119718	6	0.407	0.327	0.208				-0.09	-0.17	0.011	0.3692	0.0632	0.2918				-0.05	-0.069	-0.02	-0.13	-0.81	-0.04				0.098	-0.03	0.029	0.336853	0.261888	0.016598442	0.245289673
2319	Q05940	SLC18A2	solute carrier family 18 member A2	6571	ENSG00000165646	4	-0.37	-0.43	-0.1	0.918	0.295	-0.32	-0.32	-0.2	0.31	-0.396	0.6209	0.6527	-0.265	0.569	-0.563	0.3695	0.6672	0.0291	-0.58	-1.69	-0.84	-1.46	-0.04	0.067	-0.11	0.337	0.406	0.228599	0.409674	-0.211604334	0.621278489
3048	Q92783	STAM	signal transducing adaptor molecule	8027	ENSG00000136738	9	0.128	-0.1	-0.07	0.076	0.341	-0.35	-0.27	-0.01	0.024	-0.359	-0.016	0.1536	0.2802	0.2262	0.1471	0.0672	0.2829	0.0704	-0.33	-0.48	-0.31	-0.65	-0.32	-0.1	-0.07	0.139	0.27	0.160809	0.180938	-0.119607674	0.300545426
3568	Q9P2D3	HEATR5B	HEAT repeat containing 5B	54497	ENSG00000008869	7	0.407	1.665	0.773	0.102	0.211	0.283	0.008	0.099	0.11	0.8882	0.2287	0.6746	0.3569	-0.462	-0.031	0.2189	-0.032	0.0591	0.845	0.52	0.46	-0.88	-0.11	-0.06	0.323	-0.17	0.112	0.57706	0.290728	0.195275004	0.095453209
2692	Q6L8Q7	PDE12	phosphodiesterase 12	201626	ENSG00000174840	7	0.191	0.394	-0.04	-0.09	0.088	-0.35	0.061	0.217	0.231	0.372	0.3246	0.2078	0.2588	-0.105	-0.096	0.0791	0.0915	0.0828	0.028	-0.69	-0.19	0.187	-0.08	-0.07	0.076	0.11	0.26	0.422723	0.119415	-0.056896492	0.176311331

2606	Q5H8C1	FREM1	FRAS1 related extracellular matrix 1	158326	ENSG00000164946	17	0.046	-0.01	-0.01	-0	-0.17	0.101	-0.3	0.04	-0.12	-0.142	0.4316	0.2619	-0.224	0.1444	-0.032	-0.015	-0.235	-0.032	-0.02	0.057	0.52	-0.29	-0.22	-0.14	0.301	0.067	-0.18	0.849014	-0.0586	-0.064932978	0.006333152
3708	Q9Y285	FARSA	phenylalanyl-tRNA synthetase subunit alpha	2193	ENSG00000179115	7	-0.43	-0.29	-0.09	0.386	0.475	-0.24	0.422	0.321	0.312	-0.443	-0.156	-0.586	-0.021	-0.08	-0.022	0.1831	-0.048	0.052	-0.05	0.604	0.18	-0.23	-0.31	0.083	0.367	0.004	0.044	0.396065	0.019555	0.220772519	-0.201217211
3304	Q9BXS5	AP1M1	adaptor related protein complex 1 subunit m	8907	ENSG00000072958	13	0.274	0.032	-0.15	0.247	0.252	-0.07	0.227	0.248	0.511	0.1542	0.0425	0.1228	0.1658	0.1548	0.0709	0.2342	0.1943	0.2724	0.16	-0.04	0.017	0.181	0.077	0.027	0.124	0.216	0.351	0.825515	0.050849	0.017885339	0.032964142
1441	P05556	ITGB1	integrin subunit beta 1	3688	ENSG00000150093	5	-0.36	-0.04	0.263	-0.22	-0.25	-0.21	0.325	0.232	-0.24	0.1829	0.2002	-0.342	-0.07	-0.16	0.1016	-0.555	0.2378	-0.114	-0.05	0.517	0.386	0.007	-0.12	-0.16	0.139	-0	-0.05	0.602196	-0.12966	0.002203398	-0.131861698
1910	P41236	PPP1R2	protein phosphatase 1 regulatory inhibitor su	5504	ENSG00000184203	3	-0.18	0.009	-0.32				0.213	0.087	0.307	-0.355	-0.084	-0.305				0.2488	0.3931	0.2092	-0.22	-0.56	-0.26				-0.05	0.166	0.486	0.891831	0.093282	0.00160189	0.09168029
3028	Q8WZA9	IRGQ	immunity related GTPase Q	126298	ENSG00000167378	9	-0.07	0.278	-0.06	0.046	0.233	-0.43	0.052	0.154	0.305	0.1526	-0.129	-0.062	0.0083	-0.168	0.1452	0.3368	-0.12	0.0839	-0.18	-0.77	-0.16	-0.07	-0.07	0.16	0.29	0.048	-0.01	0.56758	0.140632	0.028434158	0.112197781
3372	Q9H2M9	RAB3GAP	RAB3 GTPase activating non-catalytic prote	25782	ENSG00000118873	3				0.093	-0.04	-0.22	-0.63	0.253	-0.54				0.1085	0.0223	0.0439	-0.363	-0.526	-0.63				-0.29	-0.02	0.223	-0.32	-0.18	-0.57	0.978164	0.012318	0.044380318	-0.032062041
2911	Q8N7R7	CCNYL1	cyclin Y like 1	151195	ENSG00000163249	6	-0.05	0.005	-0.09				0.5	0.254	0.401	-0.056	0.036	0.0328				0.1931	0.2301	0.7434	-0.19	0.044	-0.18				-0.1	-0.19	0.92	0.809945	0.120188	-0.025591687	0.145779954
3459	Q9NPQ8	RIC8A	RIC8 guanine nucleotide exchange factor A	60626	ENSG00000177963	8	-0.06	0.115	-0.07	0.115	0.028	-0.23	0.336	-0.09	0.116	0.0767	-0.051	0.0703	0.7226	-0.113	0.1705	0.0812	-0.237	0.055	-0.26	-0.44	-0.26	-0.02	-0.15	0.141	0.182	-0.16	-0.1	0.284626	0.14687	-0.058564823	0.205435112
2718	Q6S8J3	POTEE	POTE ankyrin domain family member E	445582	ENSG00000188219	11	0.321	0.213	-0.25				-0.18	0.088	0.027	0.2206	0.0657	0.0282				-0.275	0.4246	-0.425	-0.59	-1.26	-0.19				-0.01	0.248	0.356	0.595307	0.279889	0.030918258	0.248970324
2029	P50914	RPL14	ribosomal protein L14	9045	ENSG00000188846	6	0.152	-0.07	-0.06	0.09	0.735	-0.26	0.321	-0.11	0.293	-0.061	-0.092	-0.091	0.0824	-0.153	-0.084	-0.189	0.2772	0.1415	-0.19	-0.42	0.067	-0.11	-0.11	0.175	0.185	-0.27	0.287	0.464958	0.163421	0.138746621	0.024673985
2420	Q13564	NAE1	NEDD8 activating enzyme E1 subunit 1	8883	ENSG00000159593	7	0.021	-0.26	-0.04	0.093	0.399	-0.38	-0.15	-0.01	0.05	0.1811	-0.034	0.135	0.1387	-0.22	-0.123	-0.063	0.1298	0.0038	-0.02	0.143	0.324	-0.23	-0.2	0.067	-0	0.103	0.067	0.847444	-0.05791	-0.046939255	-0.010970807
1106	O43246	SLC7A4	solute carrier family 7 member 4	6545	ENSG00000099960	5	0.082	-0.05	-0.4	-0.01	-0.18	-0.19	-0.03	-0.03	0.035	-0.074	-0.003	-0.094	0.0356	0.1732	0.3103	0.0325	-0.411	-0.056	0.169	0.345	0.189	-0.21	-0.23	0.064	0.259	-0.05	0.466	0.277923	-0.19589	-0.075436385	-0.120456785
2719	Q6UW15	REG3G	regenerating family member 3 gamma	130120	ENSG00000143954	4	0.141	-1.28	-0.68	0.155	-0.23	-0.21	0.414	0.637	0.401	-1.269	-0.925	1.1231	0.14	-0.192	-0.067	-0.136	-0.476	-0.471	-0.73	-0.19	-0.6	-0.14	-0.23	-0.3	-0.59	0.136	-0.25	0.72554	0.249716	0.180987739	0.068728757
9	P46379	BAG6	BCL2 associated athanogene 6	7917	ENSG00000204463	9	0.065	0.05	-0.06	-0.23	0.061	0.043	-0.01	0.168	0.232	0.2075	0.1795	0.0875	-0.191	0.1945	-0.23	0.013	0.1179	0.0532	-0.18	-0.38	0.284	-0.52	-0.4	0.176	0.075	0.01	-0.01	0.375891	0.14038	-0.012124334	0.152504126
1896	P40306	PSMB10	proteasome subunit beta 10	5699	ENSG00000205220	5	-0.17	-0.5	0.078	-0.91	-0.43	-0.52	-0.04	-0.55	-0.37	-0.282	-0.362	-0.009	0.132	0.0575	-0.496	0.5706	0.1507	-0.016	-0.26	-0.01	0.289	1.002	0.312	-0.02	0.603	-0.03	-0.15	0.082459	-0.57106	-0.350956172	-0.220106023
627	E9PB18	KIAA1324	KIAA1324	57535	ENSG00000116299	15	-0.21	0.021	0.146	0.094	-0.03	-0.44	0.086	0.032	0.254	0.0278	-0.168	0.1049	0.1371	0.0972	-0.139	0.2165	-0.048	0.1324	-0.33	-0.23	-0.28	-0.46	0.047	0.048	0.132	0.21	0.167	0.609512	0.071585	-0.045414246	0.11699959
2838	Q8IUZ5	PHYKPL	5-phosphohydroxy-L-lysine phospho-lyase	85007	ENSG00000175309	7	0.297	0.234	-0.42				0.019	0.12	0.236	0.3569	-0.646	0.4124				0.0093	0.0839	0.1664	-0.11	-0.66	0.018				0.105	0.167	0.205	0.844313	0.127019	0.017744758	0.109274066
1740	P24534	EEF1B2	eukaryotic translation elongation factor 1 bet	1933	ENSG00000283391	4	0.119	-0.02	0.059	0.139	0.227	-0	0.292	0.183	0.437	-0.222	-0.465	0.0456	0.0971	-0.139	0.1043	0.0043	0.22	0.0923	-0.1	-0.93	-0.15	-0.2	-0.16	-0.03	0.055	0.066	0.203	0.165957	0.297916	0.18842574	0.109489829
3254	Q9BRT3	MIEN1	migration and invasion enhancer 1	84299	ENSG00000141741	6	0.185	0.475	0.121	-0.01	0.053	-0.36	-0.35	-0.26	-0.12	0.3447	0.0177	-0.056	0.0594	-0.103	0.0791	0.2772	0.0134	-0.27	-0.16	-0.78	-0.2	-0.63	-0.33	0.035	-0.19	0.013	-0.24	0.146811	0.246316	-0.069961517	0.316277298
965	K7EP59	STK11	serine/threonine kinase 11	6794	ENSG00000118046	4				0.152	0.901	-0.38	0.123	0.226	0.592				0.4111	-0.598	-0.128	0.0924	0.0502	0.0195				-0.35	-0.48	0.144	-0	0.174	0.123	0.400461	0.332097	0.293433477	0.038663953
1213	O75493	CA11	carbonic anhydrase 11	770	ENSG00000063180	3	-0.19	-0.71	-0.17				0.21	0.061	0.05	-0.026	0.4145	0.0904				0.2501	-0.323	0.0462	-0.5	0.51	0.068				0.467	0.184	-0.25	0.618831	-0.20558	-0.200898812	-0.004682794
2599	Q53TN4	CYBRD1	cytochrome b reductase 1	79901	ENSG00000071967	2	-0.77	0.119	-0.42				0.266	0.112	-0.17	-0.347	0.0144	-0.079				0.129	0.246	0.5778	-0.17	0.054	0.09				-0.12	-0.29	-0.14	0.53596	-0.04828	-0.234627359	0.186351278
2714	Q6PK18	OGFOD3	2-oxoglutarate and iron dependent oxygenas	79701	ENSG00000181396	4	-0.3	-0.24	-0.4	-0.24	-0.73	0.115	-0.14	0.023	0.279	0.0153	-0.185	0.1192	-0.274	0.3869	-0.121	0.139	-0.226	0.0219	-0.34	0.31	0.019	0.531	-0.01	-0.02	0.262	0.328	-0.24	0.253652	-0.27463	-0.167364845	-0.10726073
532	C9JQD4	PPIH	peptidylprolyl isomerase H	10465	ENSG00000171960	4	-0.14	0.195	-0.05	0.193	0.007	-0.11	0.073	-0.01	0.24	0.0541	-0.005	-0.036	0.1959	0.0103	0.4378	0.1246	0.2458	0.062	-0.2	-0.72	-0.07	-0.58	-0.18	0.046	0.01	-0.06	0.179	0.117045	0.220284	-0.076369956	0.296653546
1681	P19838	NFKB1	nuclear factor kappa B subunit 1	4790	ENSG00000109320	6	-0.11	-0.45	-0.4	-0.15	0.055	-0.57	-0.1	-0.03	0.134	0.0385	0.0102	0.1534	0.1756	-0.315	0.0694	0.0207	-0.019	-0.057	-0.19	0.104	-0.34	-0.08	-0.13	0.065	0.358	-0.15	0.054	0.28274	-0.1452	-0.188617536	0.043416088
914	J3KNQ4	PARVA	parvin alpha	55742	ENSG00000197702	11	0.118	0.303	0.04	-0.06	-0.09	-0.4	-0.36	0.229	0.369	0.1303	0.1017	0.05	-0.122	-0.236	0.0441	-0.158	0.2659	0.0635	-0.22	-0.83	-0.25	-0.24	-0.37	0.022	-0.38	-0.04	0.068	0.16174	0.265634	0.001578201	0.264055714
2688	Q6ICL3	TANGO2	transport and golgi organization 2 homolog	128989	ENSG00000183597	7	-0.15	0.307	0.116	-0.31	0.077	-0.32	-0.22	-0.15	0.074	0.0531	0.211	0.0023	-0.262	0.1222	0.1659	0.1142	0.0391	-0.034	0.135	-0.86	-0.13	-0.22	-0.13	0.078	-0.07	-0.03	0.135	0.464861	0.058082	-0.10898508	0.167067416
375	A6NGJ0	DYNLT3	dynein light chain Tctex-type 3	6990	ENSG00000165169	2	-0.04	-0.16	-0.13	-0.24	-0.03	-0.31	0.556	0.155	0.366	0.4087	0.1612	-0.099	-0.183	-0.071	0.2078	0.2648	-0.158	0.1056	0.028	-0.33	-0.19	-0.27	-0.29	-0.07	0.286	0.106	0.456	0.799623	0.048768	-0.052440503	0.101208421
1057	O14920	IKBKB	inhibitor of nuclear factor kappa B kinase su	3551	ENSG00000104365	5				-0.03	-0.14	-0.45	0.036	-0.05	0.097				0.095	-0.008	-0.055	0.0306	0.0974	0.2417				-0.43	-0.31	0.11	0.041	0.026	0.339	0.577113	-0.0515	-0.156100997	0.104603254
1514	P09466	PAEP	progestagen associated endometrial protein	5047	ENSG00000122133	7	-0.3	0.347	-0.13	0.402	-0.06	-0.11	0.266	-0.01	1.32	-0.152	0.635	0.3976	0.0272	-0.351	0.2294	0.7818	0.3665	1.0673	0.232	-0.39	-0.55	0.046	0.476	-0.23	1.093	0.435	-0.21	0.711725	0.090152	-0.142852726	0.233005085
298	A0A1W2PR	SLC7A2	solute carrier family 7 member 2	6542	ENSG00000003989	9	-0.17	0.77	0.31	0.025	-0.06	-0.2	-0.25	0.174	0.057	0.3223	-0.264	-0.262	0.1745	-0.108	0.1344	-0.211	0.1308	-0.154	-0.02	-0.05	-0.62	0.023	-0.31	-0.08	-0.28	0.142	0.1	0.464496	0.195089	0.099752726	0.095335796
252	A0A0U1RQ	DIP2C	disco interacting protein 2 homolog C	22982	ENSG00000151240	13	0.112	0.298	-0.15	0.191	0.23	-0.38	0.198	-0.04	0.021	-0.109	0.1364	0.3002	-0.006	-0.364	-0.002	0.3345	0.2195	0.2168	-0.28	-0.21	-0.41	-0.49	-0.42	-0.14	-0.04	0.062	0.442	0.225923	0.217977	-0.027341342	0.245318091
1697	P20827	EFNA1	ephrin A1	1942	ENSG00000169242	3	-0.45	-0.73	-0.57	0.016	-0.03	-0.12	-0.04	-0.03	0.22	0.3058	0.28	0.4341	0.1057	-0.157	-0.178	0.1658	-0.024	-0.081	-0.52	-0.65	-0.08	-0.44	-0.15	-0.24	-0.01	0.269	-0.75	0.142871	0.091817	-0.286980204	0.378797171
1285	O95396	MOCS3	molybdenum cofactor synthesis 3	27304	ENSG00000124217	7	-0.1	-0.26	-0.05	-0.05	-0.25	-0.12	0.132	0.147	0.174	-0.003	-0.025	0.0439	0.1015	0.2264	-0.167	0.2197	-0.015	0.2248	-0.2	-0.12	-0.21	-0.27	-0.25	-0.09	0.222	0.098	0.224	0.373179	0.023094	-0.110643818	0.133737394
848	H3BND4	LOC10272	pyridoxal-dependent decarboxylase domain-	102724985;	ENSG00000179889	11	0.254	0.253	0.133	-0.11	0.289	-0.28	-0.12	0.131	0.085	0.2066	0.2212	0.1706	0.102	-0.139	0.071	0.0538	0.02	0.5172	0.064	-0.62	-0.17	-0.08	-0	0.016	0.131	-0.06	0.132	0.266473	0.137933	-0.064596509	0.202529281
102	Q92599	SEPTIN8;	septin 8	23176	ENSG00000164402	6	0.295	0.545	0.217	0.62	0.616	-0.46	0.014	-0.16	0.078	0.3545	0.4047	0.1543	-0.364	-0.348	-0.45	0.1018	0.2267	0.0732	0.051	-0.41	-0.13	-0.68	-0.62	-0.51	0.125	0.001	0.21	0.184671	0.413361	0.179080457	0.234280886
948	K7EJ78	RPS15	ribosomal protein S15	6209	ENSG00000115268	3	-0.69	0.077	-0.17				0.305	-0.05	0.446	-0.526	-0.318	-0.353				0.0341	0.363	0.136	-0.32	-0.82	-0.46				0.382	-0.03	0.3	0.881239	0.145992	0.097149638	0.048842582
3355	Q9H1B5	XYLT2	xylosyltransferase 2	64132	ENSG00000015532	10	-0.05	0.05	-0.2	-0.33	-0.18	-0.43	-0.08	-0.1	-0.02	0.1854	0.1523	0.1132	-0.102	0.1419	-0.24	0.0253	-0.259	0.1493	0.158	-0.02	0.131	-0.3	-0.41	-0.24	0.074	0.002	-0.06	0.306078	-0.07515	-0.167097027	0.091947082
958	K7ELL7	PRKCSH	protein kinase C substrate 80K-H	5589	ENSG00000130175	9	-0.05	-0.47	-0.11	0.053	0.327	0.114	0.469	0.648	0.293	0.2246	-0.146	0.0351	-0.58	0.034	-0.326	-0.093	-0.085	-0.119	-0.04	0.865	0.158	0.201	0.704	-0.48	0.158	-0.15	-0.1	0.340143	-0.00452	0.259277533	-0.263799363
2948	Q8NFQ8	TOR1AIP2	torsin 1A interacting protein 2	163590	ENSG00000169905	3	-0.12	-0.7	-0.4	-0.37	-0.37	-0.12	-0.4	-0.15	-0.14	0.2284	0.3255	-0.084	-0.49	0.5162	-0.122	0.4728	-0.235	-0.084	-0	0.466	0.177	-0.06	-0.02	-0.22	0.473	0.089	-0.39	0.118243	-0.3634	-0.365001404	0.001600719
2660	Q7L523	RRAGA	Ras related GTP binding A	10670	ENSG00000155876	9	-0.04	0.185	-0.08	0.135	-0.2	-0.28	-0.2	-0.12	-0.03	0.4732	0.2827	-0.107	0.1133	0.0532	0.2509	0.0613	0.2914	-0.012	-0.31	-0.76	-0.09	-0.31	-0.24	0.091	0.033	-0.17	0.197	0.099772	0.104376	-0.225443031	0.329818907
3728	Q9Y2Z0	SUGT1	SGT1 homolog, MIS12 kinetochore complex	10910	ENSG00000165416	9	0.234	0.022	0.043	0.09	0.02	0.15	0.097	0.124	0.271	0.0002	0.0036	0.1478	-0.031	0.0156	0.0977	0.0722	0.2912	-0.03	-0.19	-0.37	-0.37	-0.05	-0.28	0.025	0.141	0.056	0.142	0.101778	0.215671	0.053949226	0.161721775
2049	P51692	STAT5B	signal transducer and activator of transcripti	6777	ENSG00000173757	11	-0.61	-0.61	-0.78	-0.18	-0.35	-0.07	-0.12	-0.08	0.138	-0.689	-0.695	-0.327	-0.33	0.4776	-0.154	0.0461	-0.023	-0.157	-0.93	-1.53	-0.42	-0.28	0.136	-0.03	0.128	0.056	0.095	0.908965	0.014285	-0.088694006	0.102978845
3668	Q9UN76	SLC6A14	solute carrier family 6 member 14	11254	ENSG00000268104	5	-0.53	-0.57	-0.46	-0.72	-1.12	-0.21	-0.61	-0.21	-0.2	-0.692	-0.806	-0.257	0.0632	-0.488	0.3501	0.2203	-0.586	-0.417	-0.28	0.332	-0.24	0.381	-0.11	-0.13	-0.33	-0.14	-0.04	0.137603	-0.45157	-0.223368683	-0.228197361
3688	Q9UPY8	MAPRE3	microtubule associated protein RP/EB family	22924	ENSG00000084764	13	0.32	0.569	0.036	-0.1	-0.01	-0.15	-0.19	-0.2	0.114	0.3831	0.2324	-0.009	0.1179	-0.06	0.4729	0.1321	0.1449	-0.001	-0.24	-1.31	-0.15	-0.5	-0.26	0.173	-0.17	-0.15	0.32	0.149914	0.295373	-0.114452839	0.409826327
3600	Q9UGI8	TES	testin LIM domain protein	26136	ENSG00000135269	12	0.081	0.099	-0.22	-0.08	-0.1	-0.58	-0.29	0.014	0.054	0.0874	0.2274	0.0821	-0.166	-0.337	0.0078	-0.098	-0.078	0.0204	-0.24	-0.67	-0.18	-0.52	-0.34	-0.28	-0.03	0.06	-0.03	0.240038	0.13517	-0.084678857	0.219848693
264	A0A1B0GTL	RAB11FIP	RAB11 family interacting protein 5	26056	ENSG00000135631	5				0.268	0.546	-0.61	-0.02	0.26	0.219				0.1155	-0.249	-0.185	0.2792	0.0195	0.1885				-0.61	-0.5	-0.51	0.214	0.462	0.07	0.618319	0.256416	0.082372277	0.174043296
2519	Q15386	UBE3C	ubiquitin protein ligase E3C	9690	ENSG00000009335	7	-0.02	0.014	-0.14	0.094	0.174	-0.1	-0.05	0.08	0.212	0.1461	-0.223	0.0132	0.2654	-0.107	0.0852	0.1274	0.1782	0.086	-0.22	-0.7	-0.21	0.025	0.001	0.294	0.001	0.057	0.194	0.55254	0.091527	-0.034094417	0.125621589
2406	Q13438	OS9	OS9 endoplasmic reticulum lectin;OS9, end	10956	ENSG00000135506	39	0.141	-1.23	-0.36	-0.63	-0.38	0.228	-0.17	0.101	0.11	0.3993	-0.378	0.0264	-0.777	0.209	0.0222	0.0311	-0.891	-0.426	0.454	0.68	0.492	-0.35	0.375	-0.39	0.72	0.009	-0.4	0.269901	-0.42047	-0.044840893	-0.375628525
2334	Q07812	BAX	BCL2 associated X, apoptosis regulator	581	ENSG00000087088	7	0.078	0.149	0.2	0.007	-0.19	-0.19	0.151	0.087	0.244	0.4458	0.3451	0.3047	-0.12	-0.166	0.1003	0.3776	-0.175	0.2105	0.117	-0.39	0.055	-0.44	-0.21	-0.02	0.161	-0.04	-0.12	0.189143	0.158748	-0.086985694	0.245734056
2629	Q5T0F9	CC2D1B	coiled-coil and C2 domain containing 1B	200014	ENSG00000154222	10	0.104	0.113	-0.04				-0.06	-0.09	0.049	0.2132	-0.043	-0.09				0.1046	0.13	0.1259	-0.26	-0.5	-0.04				-0.19	-0.03	0.015	0.129633	0.181752	-0.061132217	0.242884455
2743	Q6ZMB5	TMEM184	transmembrane protein 184A	202915	ENSG00000164855	5	-0.08	0.366	-0.03	0.155	0.058	-0.15	0.244	0.188	0.099	0.1664	0.1989	-0.148	0.1398	-0.21	0.0594	0.299	0.2709	0.274	-0.01	-0.54	0.116	0.099	-0.12	0.064	0.162	0.009	0.307	0.623384	0.085117	-0.022299535	0.107416129
2727	Q6UWW0	LCN15	lipocalin 15	389812	ENSG00000177984	8	-1.13	-0.76	-0.23	-0.72	0.598	-0.14	-0.9	-0.23	-0.17	-1.386	0.9476	-1.407	-0.182	-0.544	-0.474	0.3065	0.792	-0.367	-1.19	-0.67	0.512	-1.43	-0.61	0.296	-0.18	1.989	-0.46	0.902171	-0.21462	-0.151177921	-0.063442932
2554	Q16537	PPP2R5E	protein phosphatase 2 regulatory subunit B'e	5529	ENSG00000154001	5	0.307	0.271	0.169	0.093	-0.08	-0.41	-0.09	0.003	0.086	0.2131	-0.063	0.0437	-0.198	-0.261	-0.038	-0.169	0.5874	0.0501	-0.07	-0.49	-0.19	-0.6	-0.54	0.015	0.151	0.148	-0.09	0.29562	0.224111	0.0205899	0.203521308
3274	Q9BUH6	PAXX	PAXX non-homologous end joining factor;P	286257	ENSG00000148362	5	0.363	0.33	0.039	-0.1	0.161	-0.31	-0.15	-0.15	-0.08	0.4902	0.2678	0.3508	0.0085	0.0363	0.1427	-0.114	0.0351	-0.169	0.094	-0.58	0.232	-0.15	-0.15	-0.05	-0.12	-0.04	-0.03	0.326957	0.099039	-0.10593205	0.204970797
2214	P62877	RBX1	ring-box 1	9978	ENSG00000100387	3	0.471	-0.26	0.111				0.656	0.472	0.451	0.1156	0.1813	0.3014				0.4094	0.244	0.238	0.077	-0.19	0.054				0.29	0.114	0.451	0.561612	0.185459	0.069253293	0.116205297
2644	Q5TAA0	TTC22	tetratricopeptide repeat domain 22	55001	ENSG00000006555	11	0.016	0.249	-0.04	0.081	-0.13	-0.34	0.732	-0.03	0.068	0.1161	0.1034	0.1295	0.1975	-0.061	0.0938	0.0662	0.1157	-0.097	-0.28	-0.66	-0	-0.63	-0.14	0.101	0.23	-0.05	0.111	0.286365	0.215202	-0.005982667	0.221184707
1700	P20962	PTMS	parathymosin	5763	ENSG00000159335	2	-0.02	0.033	-0.02	0.185	-0.04	-0.5	0.321	0.09	0.362	0.043	0.0407	0.062	0.0019	0.1412	-0.019	0.2274	0.2268	0.122	-0.24	-0.53	-0.22	-0.76	-0.31	-0.23	0.102	0.295	0.146	0.172718	0.238736	-0.04898221	0.287718334
132	A0A096LNZ	ISG15	ISG15 ubiquitin like modifier	9636	ENSG00000187608	3				-0.69	-0.41	-0.55	-0.13	-0.2	-0.01				-0.324	0.4211	-0.231	0.0392	-0.001	0.0272				-0.31	-0.17	0.022	-0.13	-0.1	0.121	0.208707	-0.2362	-0.319156924	0.082955496
1677	P19652	ORM2	orosomucoid 2	5005	ENSG00000228278	7	1.573	-0.43	0.665	0.172	-0.65	-0.25	-0.63	0.674	-0.17	0.8748	-0.028	-0.339	0.3378	-0.343	-1.27	0.4884	-0.48	1.3245	-0.39	-0.48	-0.05	0.276	-0.02	-0.21	-0.27	0.28	-1.48	0.618412	0.366188	0.04267869	0.323509522
977	M0R0N4	AP2S1	adaptor related protein complex 2 subunit si	1175	ENSG00000042753	6	0.025	0.127	0.062	-0.01	0.12	-0.29	-0.09	-0.09	0.005	0.1401	0.114	-0.08	0.0424	-0.15	-0.172	-0.025	0.2009	0.1685	-0.13	-0.17	-0	0.021	-0.11	0.079	-0.02	-0.03	0.018	0.637362	0.023867	-0.041870739	0.065737406
2160	P61225	RAP2B	RAP2B, member of RAS oncogene family	5912	ENSG00000181467	6	0.03	0.41	0.168	-0.12	-0.05	-0.17	0.192	0.132	-0.06	-0.093	0.273	0.0957	0.0463	-0.29	0.0642	0.276	0.1842	0.3793	-0.28	-0.35	-0.29	0.31	-0.17	0.086	-0.33	-0.25	0.189	0.219482	0.17972	-0.045018382	0.224738394
1904	P41181	AQP2	aquaporin 2	359	ENSG00000167580	2	-0.2	0.06	-0.17	-1.32	-0.24	0.168	-0.45	-0.5	-0.22	-0.233	-0.334	-0.599	-0.347	-0.456	-0.038	-0.442	-0.229	-0.006	0.261	1.635	-0.03	0.638	-0.01	-0.33	0.021	0.073	-0.49	0.157747	-0.51472	-0.020064129	-0.494651246
1930	P43234	CTSO	cathepsin O	1519	ENSG00000256043	5	-0.29	-0.64	-0.38	-0.05	-0.72	-0.06	-0.02	-0.18	0.158	0.0997	-0.043	-0.042	-0.422	0.0831	-0.357	0.3544	-0.021	0.0542	-0.42	-0	0.038	0.047	-0.04	-0.23	0.394	0.16	-0.21	0.302297	-0.21519	-0.211636446	-0.003553436
1871	P36405	ARL3	ADP ribosylation factor like GTPase 3	403	ENSG00000138175	9	0.274	0.346	0.171	0.246	0.294	-0.3	0.052	0.102	0.148	0.2062	0.1343	0.2857	0.136	-0.014	0.2816	0.0335	0.0616	-0.026	-0.14	-0.64	-0.28	-0.35	-0.33	-0.02	0.118	0.032	0.249	0.093537	0.29962	0.026587952	0.273031594
2454	Q14289	PTK2B	protein tyrosine kinase 2 beta	2185	ENSG00000120899	13	-0.14	0.057	-0.12	-0.21	-0.3	-0.58	-0.26	-0.03	-0.05	0.3641	0.0297	0.0519	0.0487	-0.292	0.0258	-0.183	0.0652	-0.075	-0.16	-0.59	-0.08	0.317	0.036	0.369	0.071	-0.06	0.066	0.310512	-0.17704	-0.184243097	0.007202509
2768	Q7L7V1	DHX32	DEAH-box helicase 32 (putative)	55760	ENSG00000089876	5	0.199	0.403	0.097	-0.01	-0.17	-0.17	-0.06	0.098	0.2	0.3045	-3E-04	0.114	0.1939	-0.06	0.0275	0.007	0.1388	-0.226	-0.06	-1.02	-0.09	-0.46	-0.16	0.159	-0.09	-0.06	0.269	0.262577	0.234566	0.010185279	0.2243812
2998	Q8WTS6	SETD7	SET domain containing lysine methyltransfe	80854	ENSG00000145391	8	0.095	0.092	-0.11	0.117	-0.07	-0.11	-0.24	-0.23	0.182	0.0214	0.0033	-0.034	0.0397	-0.156	0.1669	0.1312	0.2662	0.039	-0.22	-0.89	-0.16	-0.43	-0.36	-0.11	-0.18	0.014	0.448	0.209625	0.178609	-0.083392269	0.262001496
1922	P42574	CASP3	caspase 3	836	ENSG00000164305	4							-0.08	-0.01	0.06							-0.017	-0.041	0.0092							0.027	-0.13	0.224	0.863526	-0.05061	0.006765909	-0.057375162
3594	Q9UBY5	LPAR3	lysophosphatidic acid receptor 3	23566	ENSG00000171517	7	-0.23	0.131	-0.19	-0.07	-0.23	-0.04	-0.08	0.41	-0.24	0.0241	0.3993	0.5313	-0.023	-0.067	-0.682	0.4699	0.0469	-0.003	-0.39	0.218	-0.24	-0.43	-0.5	-0.09	-0.08	0.353	0.071	0.522369	0.060772	-0.137637543	0.198409246
427	B8ZZ56	ZFAND2B	zinc finger AN1-type containing 2B	130617	ENSG00000158552	3	0.066	0.009	0.141	-0.08	-0.07	-0.26	0.189	0.227	0.358	-0.058	0.082	-0.054	-0.055	-0.262	0.2654	0.271	0.402	0.3988	-0.34	-0.49	0.065	-0.29	-0.36	0.05	0.148	0.235	0.44	0.503544	0.124767	-0.045425101	0.170192193
704	F5H039	GPHN	gephyrin	10243	ENSG00000171723	7	0.167	0.112	-0.04	-0.19	-0.02	-0.29	-0.03	-0.05	0.162	-0.218	-0.241	-0.012	-0.081	-0.046	0.054	0.1184	0.3435	0.1579	-0.21	-0.76	-0.02	-0.45	0.009	-0.01	0.245	0.089	0.107	0.642274	0.091369	-0.028389954	0.119758643
3341	Q9H008	LHPP	phospholysine phosphohistidine inorganic p	64077	ENSG00000107902	7	0.413	0.27	-0	0.322	0.04	-0.26	-0.1	-0.03	-0.29	0.0393	0.3915	0.319	0.1151	-0.109	0.2529	-0.356	-0.465	-0.502	-0.24	-0.76	-0.15	-0.28	-0.19	-0.15	0.473	-0.33	0.216	0.567734	0.196502	0.074717475	0.121784083
1220	O75610	LEFTY1	left-right determination factor 1	10637	ENSG00000243709	13	-0.81	-0.42	0.644	0.491	-0.54	-0.14	-0.49	0.736	0.047	-0.851	-0.048	0.5246	0.6902	-0.331	-0.149	-0.007	0.0835	-0.532	0.253	-0.44	0.677	0.088	-0.03	-0.11	-0.45	-0.38	-0.91	0.944693	0.091959	0.016010386	0.075948962
334	A0A2R8YCX	SEMA3E	semaphorin 3E	9723	ENSG00000170381	7	-0.73	0.28	-0.11	-0.28	0.05	-0.63	-0.42	0.31	-0.02	0.5154	-0.345	-0.08	-0.019	-0.323	-0.186	0.2051	0.635	-0.269	0.055	1.076	-0.19	-0.71	-0.53	-0.08	-0.32	-0.09	-0.45	0.722618	-0.03366	-0.187734623	0.154074589
1095	O15431	SLC31A1	solute carrier family 31 member 1	1317	ENSG00000136868	3	-0.63	-0.11	-0.66	-0.41	-0.52	-0.3	-0.61	0.069	-0.85	0.1697	-0.045	-0.034	-0.09	-0.344	-0.407	0.2573	-0.793	0.3199	-0.12	0.548	-0.44	-0.05	-0.83	-0.28	-0.43	-0.3	-0.04	0.269968	-0.2298	-0.338518438	0.108713691
2631	Q5T0Z8	C6orf132	chromosome 6 open reading frame 132	647024	ENSG00000188112	7	-0.05	-2.2	-0.93	-0.32	-0.23	2.065	-0.77	0.077	-0.53	-2.688	-0.678	-1.025	-1.228	-0.632	-1.92	-0.853	-1.249	-0.81	-0.31	-0.32	2.165	1.391	0.269	0.084	-0.71	-0.59	-0.95	0.125639	-0.4356	0.910734703	-1.34633148
1544	P10586	PTPRF	protein tyrosine phosphatase receptor type	5792	ENSG00000142949	23	-0.31	-0.4	-0.1	-0.34	-0.29	-0.02	-0.15	-0.13	-0.01	0.3778	-0.009	0.0219	-0.549	0.2599	-0.189	0.1068	-0.267	0.1791	-0.08	0.438	0.313	-0.25	-0.31	-0.5	0.6	0.352	-0.31	0.40773	-0.22259	-0.186109069	-0.036477191

1024	O00592	PODXL	podocalyxin like	5420	ENSG00000128567	5	-0.27	0.519	0.306	-0.69	0.098	0.613	-0.46	-0.36	-0.61	-0.017	0.5213	-0.169	-0.201	0.3517	-0.281	-0.794	0.1855	-0.228	-0.41	-0.06	-0.23	-0.44	0.076	-0.2	0.435	-0.09	-0.38	0.946711	0.048737	-0.024911886	0.073648484
3221	Q99653	CHP1	calcineurin like EF-hand protein 1	11261	ENSG00000187446	6	-0.22	0.007	-0.2	0.111	0.242	-0.38	-0.46	-0.25	-0.04	-0.095	-0.444	-0.124	0.0411	-0.081	0.3319	-0.072	0.1412	-0.367	-0.1	0.138	-0.18	-0.39	-0.38	-0.07	-0.34	0.086	-0.11	0.833779	0.016677	-0.057684668	0.074362015
2201	P62714	PPP2CB	protein phosphatase 2 catalytic subunit beta	5516	ENSG00000104695	17	0.196	0.234	-0.08	-0.07	-0.08	-0.76	-0.26	-0.04	0.179	0.0478	-0.02	-0.057	-0.019	0.0173	0.0242	0.0182	0.3127	0.1892	-0.19	-1.1	-0.34	-0.87	-0.44	-0.03	-0.14	-0.03	0.238	0.172948	0.247927	-0.131886973	0.37981353
2069	P53384	NUBP1	nucleotide binding protein 1	4682	ENSG00000103274	5	0.05	0.131	-0.07	0.217	-0.09	-0.32	0.181	0.139	0.424	0.0301	-0.045	0.1232	-0.012	-0.443	0.1621	0.2383	0.205	0.233	-0.27	-1.18	-0.26	-0.47	-0.23	-0.05	0.042	0.055	0.22	0.193097	0.311299	0.018709304	0.292589613
2982	Q8TDL5	BPIFB1	BPI fold containing family B member 1	92747	ENSG00000125999	7	-1.79	-1.09	-1.87	-1.75	-0.44	-1.4	-1.69	-0.02	-1.94	-2.177	-1.989	-0.852	1.6371	0.0438	-1.315	-1.749	1.6665	-2.365	-2.22	-0.81	-1.07	0.693	1.883	-0.35	1.322	-1.55	-1.15	0.441367	-0.97279	-0.544813386	-0.427978203
2236	Q8WVC2	RPS21	ribosomal protein S21	6227	ENSG00000171858	4	1.675	-0.3	0.896	-0.1	0.08	-0.49	0.17	-0.03	0.203	0.4183	-0.025	0.3797	0.0068	-0.274	-0.048	0.4806	-0.021	0.0482	0.542	-0.54	-0.1	-0.5	-0.18	0.025	0.263	0.109	0.036	0.599583	0.27212	0.126423758	0.145695856
1349	P01037	CST1	cystatin SN	1469	ENSG00000170373	5	-1.43	-1.6	-0.66				0.13	-0.8	-0.11	-0.553	-1.359	-1.422				1.2465	1.6015	0.0871	-0.08	0.751	-0.88				-0.52	-0.02	0.05	0.524179	-0.62993	-0.678731721	0.048803469
293	J3QT87	CRBN	cereblon	51185	ENSG00000113851	8	0.127	0.354	-0.05	0.061	0.142	-0.53	0.1	-0.06	0.146	0.3747	0.1271	0.1341	0.3812	-0.015	-0.143	0.1664	0.0456	0.192	-0.14	-0.96	-0.35	-0.19	-0.32	0.08	0.024	0.167	-0.15	0.137509	0.23811	-0.107366407	0.34547629
3333	Q9GZM7	TINAGL1	tubulointerstitial nephritis antigen like 1	64129	ENSG00000142910	11	-0.16	-0.87	-0.2	-0.49	0.012	-0.63	-0.38	0.456	-0.34	-0.083	1.6726	-0.424	-0.128	0.0891	-0.289	0.3839	0.0327	-0.009	0.501	1.072	-0.07	-0.5	-0.33	-0	0.253	-0.47	-0.16	0.357934	-0.32158	-0.427198234	0.105614065
2772	Q9H8S9	MOB1A	MOB kinase activator 1A	55233	ENSG00000114978	4	0.073	0.283	-0.05	0.22	0.182	-0.23	-0.71	-0.75	-0.34	0.1145	0.1973	0.1172	0.2176	-0.289	0.185	0.1327	0.8561	1.2467	-0.1	-0.6	-0.27	-0.49	-0.28	0.165	-0.63	-0.14	0.306	0.121828	0.081051	-0.455562349	0.536613184
376	A6NIH7	UNC119B	unc-119 lipid binding chaperone B	84747	ENSG00000175970	5	0.14	0.302	0.09	-0.18	-0.11	-0.56	0.102	0.009	0.111	0.3025	0.1367	0.1321	0.1211	-0.332	0.1714	0.0675	0.2958	0.0927	-0.08	-0.97	-0.1	-0.6	-0.35	0.269	0.023	-0.03	0.218	0.270048	0.171119	-0.119632967	0.290751802
2178	Q5T6W2	HNRNPK	heterogeneous nuclear ribonucleoprotein K	3190	ENSG00000165119	7	0.119	-0	-0.09	-0.08	0.038	0.004	-0.24	0.096	0.016	0.2841	0.0745	0.0116	0.0339	0.2712	0.0037	-0.068	-0.044	0.0123	0.138	-0.17	0.004	-0.03	0.014	0.294	0.044	-0.13	-0.11	0.548048	-0.02145	-0.080425993	0.058972895
249	F8VSC5	SCYL2	SCY1 like pseudokinase 2	55681	ENSG00000136021	4							0.076	0.098	0.131							0.1319	0.0098	0.0313							0.056	-0.04	0.214	0.881035	0.023574	0.043803504	-0.020229786
2739	Q6WKZ4	RAB11FIP	RAB11 family interacting protein 1	80223	ENSG00000156675	11	-0.07	0.563	-0.3	0.217	0.296	-0.53	0.031	-0.13	0.224	-0.178	0.2816	0.4999	0.1593	0.0937	-0.007	-0.004	0.3598	0.5775	-0.26	-0.21	-0.17	-0.06	-0.34	-0.09	0.502	0.507	0.592	0.634449	-0.01882	-0.164948583	0.146124685
1283	O95379	TNFAIP8	TNF alpha induced protein 8	25816	ENSG00000145779	7	0.142	-0.09	-0.08	-0.32	0.046	-0.45	-0.19	-0.33	0.162	0.0561	0.1442	0.0937	-0.044	-0.329	0.0778	-0.007	0.1391	0.3859	-0.18	-0.48	0.011	0.296	-0.48	0.148	-0.15	-0.22	0.047	0.33805	-0.01303	-0.182414679	0.169380924
2138	P60866	RPS20	ribosomal protein S20	6224	ENSG00000008988	3	0.33	-0	0.131	0.098	0.343	-0.26	0.072	-0.14	0.102	-0.416	-0.086	-0.007	-0.163	-0.245	0.0333	-0.219	0.1071	-0.013	-0.23	-0.94	0.061	-0.45	0.112	0.145	0.236	-0.21	-0.26	0.277928	0.245658	0.187009215	0.058649081
2738	Q6VY07	PACS1	phosphofurin acidic cluster sorting protein 1	55690	ENSG00000175115	9	0.211	0.116	0.064	0.016	-0.05	-0.2	-0.24	-0.12	0.171	0.0728	-0.017	0.2472	0.0403	0.1028	0.149	0.0746	0.1408	0.2029	-0.05	-0.51	-0.27	-0.4	-0.34	-0.02	-0.17	-0.12	0.286	0.082232	0.173146	-0.115994771	0.28914071
108	A0A087X1J7	GPX3	glutathione peroxidase 3	2878	ENSG00000211445	8	-0.57	-0.41	0.201	-0.17	-0.8	-1.24	-0.47	0.11	-0.72	-0.625	-0.045	0.3155	-0.194	-0.747	-0.803	0.0181	-0.394	0.0391	-0.09	0.457	0.647	0.22	-0.71	0.31	1.447	0.342	-0.98	0.183712	-0.63465	-0.18208365	-0.452564186
2489	Q15018	ABRAXAS	abraxas 2, BRISC complex subunit	23172	ENSG00000165660	4	-0.06	0.108	-0.22	-0.5	-0.17	-0.54	-0.05	0.07	0.058	-0.013	-0.146	-0.145	0.7589	0.0887	-0.28	0.0111	0.1018	0.0006	-0.19	-0.05	-0.25	0.175	0.738	-0.12	0.18	-0.02	0.069	0.400238	-0.20499	-0.186924101	-0.018063842
93	H0YCT6	ARHGEF4	Rho guanine nucleotide exchange factor 4	50649	ENSG00000136002	1							1.25	1.104	1.402							1.0778	-0.165	1.2454							0.778	0.953	0.801	0.535558	0.407723	0.532580917	-0.124858086
1066	O15037	KHNYN	KH and NYN domain containing	23351	ENSG00000100441	8	0.179	0.273	-0.11	0.133	0.13	-0.35	0.201	-0.02	0.205	-0.021	0.0926	0.2808	0.0987	-0.088	0.2293	0.0073	0.2073	0.2173	-0.25	-0.53	-0.2	-0.27	-0.15	0.035	-0.14	0.062	0.45	0.207316	0.180856	-0.042519585	0.223375786
2671	Q66K14	TBC1D9B	TBC1 domain family member 9B	23061	ENSG00000284400	13	0.089	0.232	-0.02	-0.1	-0.08	-0.29	-0.13	0.038	0.031	0.219	0.0218	0.0578	0.0289	0.2558	0.0535	0.2406	-0.219	0.1321	-0.13	-0.4	-0.23	-0.37	-0.21	0.257	0.245	-0.12	0.05	0.253512	0.076642	-0.112105112	0.188747117
2075	Q5T4U8	RABGGTE	Rab geranylgeranyltransferase subunit beta	5876	ENSG00000137955	5				0.043	-0.19	-0.15	0.039	0.13	0.416				0.0415	-0.06	-0.024	0.7029	0.1273	0.1846				-0.26	-0.27	0.093	0.277	0.407	0.041	0.780894	-0.00037	-0.11479112	0.114416269
300	A0A1X7SBT	TRPV6	transient receptor potential cation channel su	55503	ENSG00000165125	7	-0.04	0.205	0.157				0.175	-0.13	-0.08	0.3251	-0.038	0.2262				0.3703	0.1522	0.4314	-0.09	0.298	0.052				0.076	0.232	0.418	0.313616	-0.116	-0.196542288	0.080537728
2636	Q5T447	HECTD3	HECT domain E3 ubiquitin protein ligase 3	79654	ENSG00000126107	10	-0.12	0.073	-0.06	-0.24	-0.24	-0.25	0.074	0.2	0.175	0.2324	0.031	0.1049	0.0326	-0.01	-0.332	0.2644	0.0531	0.1607	-0.19	0.335	0.035	-0.12	-0.16	-0.15	0.654	0.068	0.109	0.625665	-0.10955	-0.10316756	-0.006384822
3219	Q99615	DNAJC7	DnaJ heat shock protein family (Hsp40) mer	7266	ENSG00000168259	13	0.127	0.17	0.02	-0.14	-0.4	-0.26	0.086	0.121	0.237	0.0653	0.1416	0.1068	-0.24	-0.05	0.0521	0.197	0.2204	0.171	-0.28	-0.74	-0.23	-0.44	-0.42	-0.22	0.032	0.051	0.194	0.13246	0.222163	-0.078615372	0.300778043
3407	Q9H7D0	DOCK5	dedicator of cytokinesis 5	80005	ENSG00000147459	14	-0.04	0.164	-0.03	-0.13	-0.01	-0.31	0.224	0.258	0.235	0.2425	0.2084	-0.011	-0.009	-0.146	0.0329	0.2349	0.0588	0.2057	-0.26	-0.36	-0.2	-0.2	-0.17	-0.03	0.349	0.151	0.42	0.605041	0.074331	-0.04903601	0.123366788
3588	Q9UBS8	RNF14	ring finger protein 14	9604	ENSG00000013561	5	0.016	0.239	-0.12	-0.16	0.165	-0.34	0.033	0.405	0.246	0.0721	-0.242	0.1519	0.2545	0.031	0.1217	0.1214	-0.108	0.1575	-0.07	-0.71	-0.18	-0.56	0.31	0.299	0.047	0.285	0.218	0.775361	0.094285	-0.008402199	0.102687502
1921	P42566	EPS15	epidermal growth factor receptor pathway su	2060	ENSG00000085832	18	0.03	-0.02	-0.33	-0.11	0.058	-0.37	-0.22	-0.23	0.141	-0.162	-0.164	-0.038	0.0451	-0.164	0.0829	0.0733	0.0158	-0.01	-0.27	-0.45	-0.25	-0.58	-0.23	0.063	0.254	0.006	0.031	0.559713	0.041022	-0.081559902	0.122582352
3323	Q9BZH6	WDR11	WD repeat domain 11	55717	ENSG00000120008	17	0.191	0.258	0.04	-0.03	0.095	-0.32	-0.25	-0.11	0.293	0.3817	0.3996	0.1554	0.1858	-0.042	-0.007	0.0509	0.152	-0.012	-0.27	-0.81	-0.09	-0.25	-0.21	0.237	0.006	-0.09	0.213	0.193101	0.159957	-0.122525586	0.282482812
1202	O75354	ENTPD6	ectonucleoside triphosphate diphosphohydro	955	ENSG00000197586	8	0.405	0.281	0.262	-0.25	0.323	-0.13	-0.29	-0.18	0.037	0.5497	0.6219	0.3178	0.0205	-0.01	-0.376	0.3188	-0.111	-0.104	-0.54	-0.13	0.273	-0.19	-0.33	0.203	0.093	0.057	-0.21	0.442141	0.13675	-0.084759022	0.221509166
2548	Q16204	CCDC6	coiled-coil domain containing 6	8030	ENSG00000108091	11	0.183	0.176	-0.13	0.033	0.052	-0.11	-0.01	-0.06	-0.02	0.1251	0.0792	0.0672	0.036	0.0684	0.2393	0.2517	0.1402	0.0591	-0.03	-0.66	-0.15	-0.14	0.101	0.144	0.095	0.163	0.267	0.395834	0.036534	-0.103992455	0.140526444
961	K7EM18	EIF1	eukaryotic translation initiation factor 1	10209	ENSG00000173812	2	-0	-0.08	-0.14	-0.08	-0.12	-0.48	0.051	0.178	0.196	0.2179	-0.052	-0.057	-0.123	-0.046	0.4104	0.225	0.2373	0.1256	0.079	-0.13	-0.19	-0.53	-0.39	-0.45	0.271	0.029	0.277	0.297008	0.061322	-0.156914605	0.218237105
3569	Q9P2D6	FAM135A	family with sequence similarity 135 member	57579	ENSG00000082269	12	0.224	-0.3	-0.17	-0.19	-0.1	-0.08	0.421	0.308	0.256	0.0601	0.1251	-0.15	-0.023	-0.105	0.2497	0.5591	0.3729	0.259	0.028	-0.25	-0.08	-0.54	-0.08	0.177	0.116	0.394	0.565	0.736551	0.003851	-0.10858643	0.112437847
2523	Q15437	SEC23B	SEC23 homolog B, coat complex II compone	10483	ENSG00000101310	13	-0.06	-0.03	-0.06	-0.01	-0.06	-0.18	-0.13	-0.05	-0.06	0.1818	0.0685	0.0546	0.0882	-0.067	-0.023	0.1611	-0.001	0.2009	-0.11	0.068	0.235	-0.06	-0.25	-0.02	0.088	-0.09	0.035	0.125329	-0.05923	-0.144431133	0.085199993
2582	Q3ZCW2	LGALSL	galectin like	29094	ENSG00000119862	5	0.093	0.204	-0	0.065	-0.08	-0.09	-0.06	0.117	0.012	0.3378	0.1418	0.0299	0.0749	0.198	0.0469	0.1115	0.273	0.1834	0.091	-0.47	0.014	-0.42	-0.17	0.184	-0.16	0.008	0.228	0.129839	0.10534	-0.127114499	0.232454585
106	A0A087X1G	SELENOF	selenoprotein F	9403	ENSG00000183291	5	-0.49	0.116	0.773				-0.2	0.478	-0.18	0.1413	0.3741	0.4982				-0.035	0.0273	-0.693	0.013	0.362	0.141				0.227	0.215	-0.01	0.91979	-0.0742	0.031804883	-0.10600115
181	S4R460	IGHV3OR1	immunoglobulin heavy variable 3/OR16-9 (n	28307	ENSG00000270472	3	-0.12	-0.41	-0.14	-0.12	-0.25	-0	-0.19	-0.22	-0.1	-0.312	-0.138	-0.124	-0.213	0.0476	-0.12	0.071	-0.394	-0.369	0.037	-0.03	0.981	1.11	-0	0.253	0.141	-0.03	-0.24	0.091812	-0.41976	-0.000840656	-0.418914743
3121	Q96ED9	HOOK2	hook microtubule tethering protein 2	29911	ENSG00000095066	13	-0.07	0.078	-0.02	-0.31	0.055	-0.16	0.229	0.067	0.378	0.2786	0.0014	0.0569	-0.23	0.0533	0.381	0.2686	0.0908	0.1832	-0.15	-0.51	0.164	-0.32	0.139	-0.05	0.202	0.073	0.406	0.623563	0.031225	-0.093399116	0.124623975
976	M0QYT9	IRF3	interferon regulatory factor 3	3661	ENSG00000126456	6	0.458	0.473	0.342				-0.05	0.042	0.026	0.4233	0.298	0.1318				0.0196	0.2484	0.2623	0.262	0.121	0.354				0.023	-0.02	0.163	0.806124	0.063333	-0.016019431	0.079352048
2789	Q7Z5G4	GOLGA7	golgin A7	51125	ENSG00000147533	7	-0.1	-0.04	0.367	0.153	-0.09	-0.47	-0.14	0.149	-0.03	-0.301	0.0142	-0.166	0.159	-0.301	0.0016	0.237	0.015	0.2329	0.842	-0.37	0.378	-0.64	-0.46	-0.27	-0.04	0.103	0.057	0.983333	0.023183	-0.009621222	0.032804017
1008	O00418	EEF2K	eukaryotic elongation factor 2 kinase	29904;1019;	ENSG00000103319	12	0.076	-0.18	-0.49	0.036	-0	-0.28	-0.1	-0.16	0.421	0.292	-0.226	-0.21	0.1944	-0.02	0.0331	0.0319	0.193	0.0735	0.026	0.036	-0.21	-0.59	-0.29	-0.05	0.16	0.078	0.285	0.653465	-0.01323	-0.115601358	0.102375284
880	H7BZ14	PPIL3	peptidylprolyl isomerase like 3	53938	ENSG00000240344	5	0.18	0.212	0.133	-0.08	-0.02	-0.72	0.301	0.042	0.322	0.1027	0.0045	-0.065	-0.131	-0.215	0.2182	0.1289	0.1279	0.0502	-0.16	-1.28	-0.31	-0.4	-0.32	0.077	0.146	-0.01	0.196	0.3159	0.271892	0.017008276	0.254884125
3332	Q9C0I1	MTMR12	myotubularin related protein 12	54545	ENSG00000150712	10	0.201	0.058	-0.06	0.297	0.974	0.46	-0.13	-0.1	0.155	-0.031	-0.077	0.1145	0.3567	0.1515	0.4641	0.066	0.3783	0.1886	-0.27	-1	-0.4	-0.21	0.495	0.233	-0.11	-0.02	0.266	0.248531	0.320107	0.027715415	0.292391675
3719	Q9Y2I8	WDR37	WD repeat domain 37	22884	ENSG00000047056	4	-0	0.171	-0.16	-0.05	-0.16	-0.32	0.226	0.102	0.143	0.028	0.1432	0.0607	0.3927	-0.112	0.5478	0.195	-0.067	0.266	-0.34	-0.46	-0.51	-0.52	-0.54	-0.1	0.308	-0.06	0.313	0.121134	0.207545	-0.167092608	0.37463789
2598	Q53S08	RAB6D	RAB6D, member RAS oncogene family	150786	ENSG00000233087	5	-0.01	0.48	-0.06	0.275	-0.33	-0.06	-0.04	0.143	0.049	0.1112	0.3698	0.059	0.0397	0.104	0.007	0.2309	0.5423	0.1348	-0.66	-1.6	-0.39	-0.05	-0.28	0.017	-0.61	-0.26	0.154	0.060367	0.456816	-0.12835983	0.585176061
2847	Q8IW45	NAXD	NAD(P)HX dehydratase	55739	ENSG00000213995	10	-0.34	-0.5	-0.45	-0.42	-0.07	-0.54	0.062	0.088	0.157	-0.215	-0.505	-0.351	-0.101	0.0308	0.0129	0.0387	0.0034	0.1125	-0.28	-0.12	-0.25	-0.47	-0.28	-0.01	0.143	0.092	0.218	0.637556	-0.11715	-0.1156752	-0.001476723
1774	P27986	PIK3R1	phosphoinositide-3-kinase regulatory subuni	5295	ENSG00000145675	11	-0.02	0.089	-0.14	0.139	-0	-0.35	-0.4	-0.24	-0.05	0.2267	0.0605	0.0911	0.0061	-0.074	0.2962	0.3713	-0.284	-0.121	-0.28	-0.34	-0.18	-0.1	-0.26	-0.04	0.259	-0.08	-0.17	0.219341	0.023184	-0.171413125	0.194597503
1674	P19525	EIF2AK2	eukaryotic translation initiation factor 2 alpha	5610	ENSG00000055332	3				0.187	0.06	-0.19	-0.09	0.026	0.101				0.3202	0.1799	0.2291	0.018	0.1	0.1348				-0.04	0.024	0.19	-0.06	0.022	0.266	0.299167	-0.05239	-0.148726064	0.096332211
2984	Q8TDQ7	GNPDA2	glucosamine-6-phosphate deaminase 2	132789	ENSG00000163281	8	0.319	0.091	0.139	0.074	0.188	-0.37	-0.09	-0.01	0.106	0.2229	-0.018	0.0227	-0.248	-0.238	-0.049	0.1638	0.0079	-0.233	-0.22	-0.78	-0.08	-0.35	-0.26	-0.03	0.167	0.095	0.043	0.318622	0.207501	0.089625935	0.117875462
2433	Q13867	BLMH	bleomycin hydrolase	642	ENSG00000108578	5	0.261	-0.35	-0.1	0.473	-0.03	0.077	-0.07	0.047	0.203	0.059	0.0329	-0.165	-0.444	0.1655	0.0428	0.3097	-0.069	0.1153	-0.26	-0.37	-0.12	0.242	0.098	-0.33	0.252	0.272	-0.23	0.755025	0.106019	0.05132613	0.054693041
1532	P0CG37	CFC1	cripto, FRL-1, cryptic family 1	55997	ENSG00000136698	5	-0.44	-0.99	-0.47	-0.1	0.368	0.322	-0.23	0.221	-0.02	0.4426	-0.293	0.0529	0.651	-0.547	0.0198	0.4421	-0.129	-0.114	-0.89	0.284	-1.02	-0.5	-0	0.234	0.065	0.282	0.022	0.631308	0.019971	-0.207095873	0.227067201
3387	Q9H479	FN3K	fructosamine 3 kinase	64122	ENSG00000167363	6	-1.92	-1.27	-1.9	0.099	0.045	-0.32	0.443	-0.13	0.173	-1.57	-1.709	-1.183	0.0401	0.1792	0.3439	0.1007	0.4311	0.2178	-1.46	-0.46	-1.87	-0.46	-0.19	0.085	0.248	0.056	0.088	0.934853	-0.09087	-0.181215284	0.090347039
1624	P15309	ACPP	acid phosphatase, prostate	55	ENSG00000014257	33	-0.49	-0.3	-0.62	-0.43	0.081	-0.69	-0.24	0.221	-0.28	1.0358	-0.785	0.396	-0.357	-0.161	-1.156	0.5942	0.1067	0.4269	-0.45	-0.02	0.687	-0.1	0.208	0.44	-0.1	0.819	-0.25	0.336567	-0.44237	-0.31737244	-0.124995661
3050	Q92820	GGH	gamma-glutamyl hydrolase	8836	ENSG00000137563	3	0.17	-0.68	-0.34				0.33	0.532	0.339	0.0206	-0.042	0.3441				0.4574	-0.423	-0.042	-0.39	-0.09	0.034				0.412	0.493	-0.05	0.99799	-0.01018	0.005002402	-0.015187389
2276	P98164	LRP2	LDL receptor related protein 2	4036	ENSG00000081479	6	-0.02	0.413	-0.45				0.727	1.783	0.179	-0.092	0.437	0.1667				0.0308	-0.12	-0.676	-0.53	-0.19	-0.22				0.332	-0.01	-0.38	0.290728	0.605736	0.480686404	0.125049662
3669	Q9UN86	G3BP2	G3BP stress granule assembly factor 2	9908	ENSG00000138757	3				-0.36	-0.25	-0.44	0.127	-0.06	0.451				-0.23	-0.179	-0.007	-0.004	0.1524	0.1115				-0.82	-0.45	-0.22	0.248	0.153	0.282	0.900747	0.046343	-0.063764269	0.110107752
3179	Q96Q11	TRNT1	tRNA nucleotidyl transferase 1	51095	ENSG00000072756	8	0.21	0.303	-0.15	0.035	0.107	-0.17	-0.11	-0.06	0.129	0.1992	0.2361	0.108	0.0159	-0.25	0.3159	0.3217	0.2062	0.1534	-0.19	-0.54	-0.28	-0.03	-0.09	0.096	-0	0.212	0.076	0.189813	0.116941	-0.111800662	0.228741841
2040	P51397	DAP	death associated protein	1611	ENSG00000112977	2	-0.12	0.235	-0.14	-0.26	-0.32	-0.28	-0.45	-0.37	-0.21	-0.177	0.1103	-0.127	-0.087	-0.143	0.2686	-0.147	-0.096	-0.345	-0.29	-0.75	-0.28	0.22	-0.23	0.19	-0	-0.13	-0.06	0.628009	-0.06324	-0.12923759	0.065997461
3123	Q96EF6	FBXO17	F-box protein 17	115290	ENSG00000269190	7	-0.05	-0.22	-0.05	0.292	-0.13	-0.25	-0.13	0.262	0.061	0.2931	0.3497	-0.032	0.0922	-0.032	0.2601	0.1435	0.15	0.2688	-0.28	-0.79	-0.1	-0.31	-0.05	0.064	-0.12	-0.04	0.4	0.148011	0.113915	-0.189284556	0.303199935
1032	O00757	FBP2	fructose-bisphosphatase 2	8789	ENSG00000130957	4	-0.01	0.416	0.07				0.373	0.052	0.217	0.9466	-0.168	0.2981				0.1114	0.0617	-0.304	0.032	-0.91	-0.24				0.275	-0.08	-0.63	0.241191	0.445156	0.028732208	0.4164234
3700	Q9Y237	PIN4	peptidylprolyl cis/trans isomerase, NIMA-inte	5303	ENSG00000102309	4	-0.04	0.311	0.074	0.185	-0.07	-0.31	0.02	-0.38	-0.07	0.1198	0.1938	0.0915	0.0306	0.2097	0.0238	-0.115	-0.185	0.0569	-0.01	-0.15	-0.05	-0.21	-0.36	-0.2	0.25	-0.2	-0.29	0.263318	0.104128	-0.07845887	0.182586685
3687	Q9UPX8	SHANK2	SH3 and multiple ankyrin repeat domains 2	22941	ENSG00000162105	6	-0.41	0.668	-0.63	0.02	0.066	-0.33	0.336	0.383	0.068	-0.248	-0.048	0.2234	0.2909	-0.238	-0.039	0.3899	-0.12	0.2283	-0.61	-0.54	-1.11	-0.11	-0.25	0.069	0.55	0.035	0.425	0.614061	0.189874	-0.030090952	0.219965089
3759	Q9Y4P1	ATG4B	autophagy related 4B cysteine peptidase	23192	ENSG00000168397	6	0.174	0.108	0.026	-0.02	-0.4	-0.37	0.393	3E-04	0.141	0.3417	0.1993	0.204	0.2108	0.1297	-0.07	0.1501	0.0241	0.1706	-0.15	-0.64	-0.01	-0.29	-0.32	0.059	-0.06	0.15	0.312	0.216442	0.110041	-0.145868928	0.255910321
2145	P61011	SRP54	signal recognition particle 54	6729	ENSG00000100883	11	0.047	0.159	-0.15	-0	0.033	-0.4	0.403	-0.41	0.418	0.0311	-0.16	-0.144	0.0614	-0.191	0.1206	-0.463	-0.21	0.1404	-0.19	-0.66	0.103	-0.26	-0.26	0.17	0.78	0.336	-0.01	0.834704	0.010154	0.10114917	-0.090995304
1979	P48729	CSNK1A1	casein kinase 1 alpha 1	1452	ENSG00000113712	10	0.095	0.242	-0.06	-0.05	-0.04	-0.29	-0.19	-0.32	0.044	-0.162	-0.016	0.0147	0.1379	0.2182	-0.034	-0.074	0.0201	0.0354	-0.3	-0.26	0.06	0.026	-0.05	-0.06	-0.03	-0.15	0.182	0.595598	0.000366	-0.078525884	0.078891897
3636	Q9UJC5	SH3BGRL	SH3 domain binding glutamate rich protein l	83699	ENSG00000198478	3	0.217	0.231	0.128	-0.08	0.088	-0.43	-0.14	-0.31	0.032	0.0614	0.3504	0.2017	0.1568	-0.078	0.2688	-0.214	0.3553	-0.158	-0.31	-1.36	-0.44	-0.53	-0.37	0.034	-0.44	0.101	0.102	0.117848	0.32642	-0.135439774	0.461860173
2386	Q13188	STK3	serine/threonine kinase 3	6788	ENSG00000104375	8	-0.05	0.106	-0.19	-0.11	-0.21	-0.4	-0.05	-0.43	0.061	0.1223	0.2343	-0.065	-0.146	-0.233	0.1185	-0.342	-0.137	-0.041	-0.09	-0.29	0.009	-0.64	-0.44	-0.11	0.029	0.013	-0.09	0.566821	0.03486	-0.088442299	0.123302428
1736	P23743	DGKA	diacylglycerol kinase alpha	1606	ENSG00000065357	11	-0.16	0.042	-0.08	0.148	0.096	-0.27	-0.06	0.229	0.062	0.1557	-0.061	0.0483	0.3347	-0.235	-0.096	0.143	0.1567	-0.108	-0.2	-0.53	0.248	-0.13	-0.28	0.076	0.037	-0	0.157	0.631525	0.071779	-0.036535511	0.108314123
727	H0YB70	HYI	hydroxypyruvate isomerase (putative)	81888	ENSG00000178922	5				-0.34	-0.13	-0.25	0.106	-0.08	0.169				-0.043	-0.336	-0.325	0.0142	0.1743	0.0821				-0.2	-0.23	-0.15	-0.09	0.001	0.282	0.985458	-0.02317	-0.016026579	-0.007144363
2923	Q8NBR6	MINDY2	MINDY lysine 48 deubiquitinase 2	54629	ENSG00000128923	6	0.19	-0.01	-0.19	0.055	0.082	-0.37	-0.06	0.017	0.127	0.0719	0.3682	0.0077	-0.054	-0.096	0.1716	0.0722	0.034	-0.063	-0.24	-1.03	-0.28	-0.86	-0.56	0.095	0.042	0.081	0.168	0.165554	0.268661	-0.075368586	0.344029265
626	E9PAV3	NACA	nascent polypeptide associated complex sul	4666	ENSG00000196531	6	0.189	0.136	-0.09	-0.05	0.086	-0.01	0.219	0.041	0.075	0.0555	0.0297	0.1509	-0.247	-0.317	-0.009	-0.058	-0.734	-0.619	-0.15	-0.98	-0.25	0.932	0.182	0.154	0.166	-0.39	-0.45	0.479703	0.152765	0.260736642	-0.10797133
2493	Q15036	SNX17	sorting nexin 17	9784	ENSG00000115234	7	0.144	0.41	0.097	0.009	-0.03	-0.36	0.156	-0.02	0.103	0.0886	0.0123	0.1287	0.1256	-0.165	0.0796	0.1594	0.0438	0.1702	-0.27	-0.48	-0.29	-0.34	-0.33	0.046	-0.07	0.043	0.163	0.11956	0.227113	-0.015469543	0.242582317
1326	P00387	CYB5R3	cytochrome b5 reductase 3	1727	ENSG00000100243	4	0.326	0.303	0.096	-0.05	0.305	-0.33	-0.11	0.159	-0.02	0.1802	0.1655	0.3535	0.0559	0.1285	-0.376	0.0089	0.0253	0.0521	-0.22	0.048	0.46	-0.31	-0.11	0.12	0.232	0.018	-0.05	0.905596	0.053542	0.009552002	0.043989688
2060	P52630	STAT2	signal transducer and activator of transcripti	6773	ENSG00000170581	6	0.087	0.353	0.151	-0.18	-0.27	-0.74	0.273	0.254	0.394	0.2475	0.1401	0.221	0.2257	-0.011	-0.188	0.1773	0.038	0.2554	-0.16	-0.7	0.375	-0.32	-0.31	0.228	0.433	0.071	0.625	0.870571	0.007474	-0.087669012	0.095142654
2409	Q13444	ADAM15	ADAM metallopeptidase domain 15	8751	ENSG00000143537	5	-0	0.029	-0.07	-0.68	-0.13	0.069	-0.07	0.175	0.143	-0.074	-0.015	0.2136	-0.46	-0.027	-0.282	-0.01	0.0397	0.1009	-0.23	0.118	0.051	0.256	-0.08	0.042	0.265	0.067	0.286	0.427933	-0.14487	-0.002145768	-0.142728138
3134	Q96GS6	ABHD17A	abhydrolase domain containing 17A	81926	ENSG00000129968	4				-0.04	-0.08	-0.47	0.445	0.723	-0.1				0.118	-0.673	0.2686	-0.303	-0.47	0.0804				-0.74	-0.56	0.213	-0.15	-0.31	-0.42	0.361673	0.408171	0.243061842	0.165109638
1788	P29353	SHC1	SHC adaptor protein 1	6464	ENSG00000160691	8	0.328	0.369	0.261	0.122	-0.04	-0.28	-0.32	0.07	0.059	0.1481	0.3002	0.0093	0.0694	-0.063	0.1126	0.039	0.1619	-0.056	-0.23	-0.3	-0.22	-0.49	-0.15	-0.05	-0.14	0.196	0.126	0.173275	0.202683	-0.016800207	0.219482962
2861	Q8IXU6	SLC35F2	solute carrier family 35 member F2	54733	ENSG00000110660	4	0.035	0.51	-0.25	0.005	-0.11	-0.31	-1.13	-1.04	-0.29	-0.485	0.8496	0.68	0.4428	-0.06	-0.138	0.1334	-0.55	0.5267	-0.27	-0.75	0.02	-0.7	0.047	0.027	-0.75	-0.53	0.313	0.233826	0.002807	-0.441732529	0.444539906

3128	Q96F10	SAT2	spermidine/spermine N1-acetyltransferase f	112483	ENSG00000141504	6	0.117	0.162	-0.63	0.203	-0.22	-0.06	-0.17	0.191	0.605	-1.006	-0.057	-0.791	0.0384	-0.031	0.1075	0.2286	0.0479	0.3617	0.021	-0.2	-0.09	-0.27	-0.2	0.226	0.205	0.256	0.449	0.697311	-0.02291	0.143401326	-0.166315424
2624	Q5SGD2	PPM1L	protein phosphatase, Mg2+/Mn2+ dependen	151742	ENSG00000163590	5	0.255	0.256	-0.24	-0.04	0.589	-0.05	-0.38	-0.19	-0.09	-0.143	0.0575	-0.198	0.1428	-0.155	0.095	0.4093	0.4551	0.3263	-0.22	0.206	0.088	-0.32	-0.38	0.106	0.283	-0.16	0.094	0.637312	0.045615	-0.09768755	0.143302687
2922	Q8NBP0	TTC13	tetratricopeptide repeat domain 13	79573	ENSG00000143643	11	-0.07	-0.39	-0.17	-0.31	0.063	-0.12	-0.13	-0.05	-0.15	0.2786	-0.283	-0.268	-0.212	0.1045	-0.146	0.0564	-0.321	-0.176	0.38	0.915	0.242	0.088	0.166	-0.1	0.34	0.094	-0.16	0.073291	-0.36395	-0.039025733	-0.324922164
1944	P46109	CRKL	CRK like proto-oncogene, adaptor protein	1399	ENSG00000099942	6	0.215	0.184	0.027	-0.42	-0.17	-0.46	-0.18	-0.31	-0	0.1158	0.197	0.1272	-0.405	-0.276	-0.467	0.6592	-0.125	-0.108	-0.07	-0.82	-0.26	1.652	0.526	1.257	0.163	0.203	-0.06	0.367158	-0.41236	-0.0925725	-0.319783485
2052	P51993	FUT6	fucosyltransferase 6	2528	ENSG00000156413	5							0.687	0.365	0.382							0.8493	-0.196	0.0939							0.91	0.283	0.298	0.789751	-0.0187	0.229042972	-0.247738813
605	E7EPS8	PTPRM	protein tyrosine phosphatase receptor type M	5797	ENSG00000173482	9	-0.07	-0.1	0.038	-0.19	-0.21	-0.21	-0.6	-0.35	-0.43	0.2858	0.1825	-0.006	0.0798	0.0091	0.1056	-0.389	-0.165	-0.441	0.087	0.173	0.108	-0.27	-0.25	-0.13	-0.11	-0.48	-0.21	0.33393	-0.11538	-0.19792311	0.08254406
3632	Q9UJ14	GGT7	gamma-glutamyltransferase 7	2686	ENSG00000131067	7	0.055	-0.31	0.433	-0.17	0.141	-0.56	0.811	0.506	0.15	0.1427	0.7613	0.008	0.1307	-0.278	-0.041	0.34	0.0795	0.41	-0.29	0.111	0.482	-0.37	-0.39	0.029	0.518	0.28	0.382	0.918618	0.032561	-0.055561335	0.08812222
2465	Q14558	PRPSAP1	phosphoribosyl pyrophosphate synthetase a	5635	ENSG00000161542	7	-0.09	-0.18	-0.2	-0.28	-0	-0.39	-0.05	-0.16	0.088	-0.206	-0.314	-0.188	0.1645	0.017	-0.035	0.186	0.1832	0.2356	-0.35	-0.41	-0.21	-0.29	-0.14	0.006	0.304	0.018	0.146	0.450984	-0.03677	-0.145132401	0.108361216
1646	P17066	HSPA6	heat shock protein family A (Hsp70) membe	3310	ENSG00000173110	18	0.679	0.566	0.292	-0.02	0.346	-0.6	-0.95	-0.82	-0.61	0.4702	0.7621	0.185	0.3263	-0.164	0.0244	-0.911	-0.239	-0.89	-0.19	-0.98	-0.2	-1.04	-0.4	0.218	-0.6	-0.41	-0.68	0.382123	0.350359	-0.076794127	0.427153198
2402	Q13404	UBE2V1;T	ubiquitin conjugating enzyme E2 V1;TMEM	7335;38752	ENSG00000244687	10	-0.21	-0.04	-0.46	0.121	-0.2	-0.45	0.438	0.378	0.429	-0.619	-0.716	-0.094	-0.37	-0.125	-0.063	0.42	0.2668	0.1707	-0.44	-0.64	-0.32	-0.58	-0.53	-0.48	0.227	0.323	0.544	0.65977	0.211265	0.126846689	0.084418558
3402	Q9H6U6	BCAS3	BCAS3 microtubule associated cell migratio	54828	ENSG00000141376	4							0.548	0.067	0.308							0.1395	-0.446	-0.149							0.277	-0.31	0.231	0.385237	0.240502	0.459468978	-0.218966713
3104	Q96C19	EFHD2	EF-hand domain family member D2	79180	ENSG00000142634	9	0.062	-0.18	0.143	-0.14	0.035	-0.27	-0.59	-0.43	-0.4	0.17	0.3267	0.193	0.2237	-0.025	0.2957	-0.406	0.4552	-0.564	-0.17	-0.88	-0.13	-0.51	-0.47	0.202	-0.41	-0.43	-0.36	0.125886	0.155639	-0.270297328	0.425936809
178	A0A0B4J26	AC092143	novel protein (MC1R-TUBB3 readthrough)	NA	ENSG00000198211	15	-0.06	0.487	-0.21				0.708	0.592	0.297	0.2877	-0.021	0.3487				0.4837	0.2401	0.3349	0.001	5E-04	-0.11				0.246	0.923	0.045	0.866778	0.118127	0.022650651	0.095475852
258	A0A0U1RRM	ENAH	ENAH actin regulator	55740	ENSG00000154380	6	0.083	-0.24	-0.14	-0.05	0.161	-0.13	0.046	0.049	0.245	-0.077	-0.259	-0.004	0.011	-0.159	0.0346	0.1467	-0.211	-0.02	-0.07	-0.53	-0.03	-0.65	0.1	0.24	0.174	0.109	0.015	0.815601	0.074194	0.062776159	0.011417993
3348	Q9H0M0	WWP1	WW domain containing E3 ubiquitin protein	11059	ENSG00000123124	17	0.341	0.413	-0.21	0.232	0.335	-0.33	-0.16	-0.1	-0.24	-0.429	0.0639	0.3818	0.1047	-0.033	0.1012	0.2414	-0.175	0.2653	-0.19	0.106	-0.39	-0.41	-0.17	0.048	-0.2	0.015	0.514	0.681036	0.106592	-0.026726092	0.133318368
401	Q9UJY5	GGA1	golgi associated, gamma adaptin ear contai	26088	ENSG00000100083	5	0.341	0.419	0.072				-0.16	-0	-0.01	0.0705	0.2236	0.1786				-0.087	-0.031	0.0036	-0.23	-0.49	-0.09				0.006	-0.06	0.13	0.269802	0.232514	0.050974339	0.181539637
3046	Q92743	HTRA1	HtrA serine peptidase 1	5654	ENSG00000166033	11	0.748	-0.11	0.256	-0.14	0.26	0.229	-0.33	0.522	0.245	-0.59	0.3125	1.0518	0.2533	-0.425	0.0693	-0.063	-0.292	-0.174	-0.91	-0.6	-0.26	-0.29	-0.39	-0.39	0.061	0.045	-0.43	0.12575	0.540556	0.170853496	0.369702395
2416	Q13530	SERINC3	serine incorporator 3	10955	ENSG00000132824	7	-0.13	0.297	0.47	0.087	-0.15	-0.36	-0.32	0.086	-0.28	0.1469	0.0597	-0.044	0.1101	0.1278	-0.192	-0.152	0.1206	0.1632	-0.48	-0.48	-0.07	-0.36	-0.15	0.001	-0.1	-0.21	-0.28	0.143028	0.20265	-0.071093835	0.273743482
3181	Q96QG7	MTMR9	myotubularin related protein 9	66036	ENSG00000104643	9	-0.01	0.068	-0.1	-0.26	-0.45	0.066	-0.24	-0.05	0.128	0.1232	0.015	-0.051	-0.206	0.2111	-0.147	0.0555	0.107	-0.074	-0.28	-0.42	0.07	-0.37	0.255	-0.06	0.01	-0.13	0.115	0.611653	-0.00597	-0.098102366	0.092130152
1102	O43149	ZZEF1	zinc finger ZZ-type and EF-hand domain co	23140	ENSG00000074755	22	-0.11	0.098	0.006	0.012	-0.01	-0.26	-0.07	-0.14	-0.07	0.064	0.0664	-0.021	0.1926	-0.109	-0.186	-0.104	-0.037	0.0995	-0.23	-0.33	0.11	-0.08	-0.19	0.139	0.346	-0.19	-0.11	0.777068	3.81E-05	-0.055621526	0.055659662
3088	Q96AB6	NTAN1	N-terminal asparagine amidase	123803	ENSG00000157045	6	0.036	0.097	-0.37	0.006	-0.06	-0.11	-0.16	0.026	0.133	-0.088	0.0494	0.0802	0.1141	-0.195	0.0561	0.0308	0.098	-0.026	-0.38	-0.98	-0.13	-0.02	-0.15	-0.12	0.014	0.043	0.087	0.324394	0.1364	-0.058680717	0.195081096
246	A0A0J9YYF	DAAM2	dishevelled associated activator of morphog	23500	ENSG00000146122	2							-0.05	-0.2	0.215							-0.072	0.3438	0.0921							-0.25	-0.14	0.358	0.846622	-0.00262	-0.132823045	0.130208008
943	K7EM02	KATNAL2	katanin catalytic subunit A1 like 2	83473	ENSG00000167216	1	0.149	0.137	-0	-0.15	0.043	-0.47	0.169	-0.02	0.098	-0.065	-0.008	-0.078	-0.064	-0.156	-0.089	0.2925	0.4763	0.1013	-0.17	-0.46	0.017	-0.19	-0.25	-0.04	0.364	-0.02	-0.04	0.563239	0.082626	-0.050642732	0.13326923
2369	Q12933	TRAF2	TNF receptor associated factor 2	7186	ENSG00000127191	5	-0.06	-0.43	-0.43				0.047	-0.21	-0.01	0.1173	-0.083	0.3786				0.0067	-0.2	0.1522	-0.27	0.321	1.118				0.201	-0.06	-0.1	0.315774	-0.38379	-0.244363559	-0.139421846
1280	O95352	ATG7	autophagy related 7	10533	ENSG00000197548	8	0.222	0.165	0.149	-0.04	-0.25	-0.55	-0.15	-0.09	-0.06	0.3461	0.1693	0.2344	-0.276	-0.58	-0.364	-0.027	0.1833	-0.013	0.124	-0.18	0.046	-0.49	-0.67	-0.32	-0.05	-0.09	0.07	0.673104	0.105275	-0.031629575	0.136904115
3011	Q8WVD5	RNF141	ring finger protein 141	50862	ENSG00000110315	4	0.338	0.022	0.024	-0.05	-0.07	-0.28	0.29	0.041	0.128	0.1356	-0.102	0.2582	-0.191	0.0392	0.2056	0.0192	-0.015	-0.18	-0.25	-1.23	-0.46	-0.43	-0.15	0.043	0.008	-0.04	0.285	0.213178	0.295711	0.029618799	0.266092557
1853	P35240	NF2	neurofibromin 2	4771	ENSG00000186575	14	-0.47	-0.07	-0.54	0.153	0.028	-0.08	-0.22	-0.14	-0.16	0.0425	0.041	-0.06	0.3134	-0.195	0.1012	0.7124	0.2134	-0.019	-0.2	0.07	-0.6	-0.18	0.306	0.003	-0.04	-0.04	0.22	0.193791	-0.11453	-0.293542147	0.179008774
3730	Q9Y315	DERA	deoxyribose-phosphate aldolase	51071	ENSG00000023697	6	0.271	0.07	0.097	-0.01	0.324	-0.24	0.033	-0.14	-0.01	0.0942	-0.047	-0.097	-0.105	0.0118	-0.236	0.1194	0.0841	0.146	-0.18	-0.34	0.362	0.544	-0.07	-0.2	0.555	0.05	-0.21	0.901972	-0.01338	0.046718901	-0.060094873
2958	Q8NHG8	ZNRF2	zinc and ring finger 2	223082	ENSG00000180233	4	0.14	0.61	0.046	0.06	-0.1	-0.13	-0.54	-0.15	-0.15	0.1651	0.0584	0.1333	0.1259	-0.194	0.3414	-0.201	-0.17	-0.174	0.139	0.023	0.294	-0.35	-0.08	0.002	-0.05	-0.12	0.016	0.969452	-0.00822	-0.032756638	0.024536237
1227	O75822	EIF3J	eukaryotic translation initiation factor 3 subu	8669	ENSG00000104131	6	0.359	0.26	0.139	-0.22	0.143	-0.32	-0.43	-0.37	-0.08	0.0649	0.205	0.1723	0.0259	0.1119	0.1471	-0.37	-0.098	-0.52	-0.01	-0.82	-0.02	-0.54	-0.11	0.086	0.014	-0.25	-0.19	0.535758	0.145406	-0.029083889	0.174489726
2462	Q14508	WFDC2	WAP four-disulfide core domain 2	10406	ENSG00000101443	5	0.526	-0.88	-0.06	0.358	-0.52	-0.35	-0.19	0.583	0.473	0.4236	-0.131	1.0516	-0.201	-0.251	-0.836	0.3022	-0.593	-0.662	0.24	-1.2	0.369	0.385	0.642	-0.3	-0.61	0.336	-1.27	0.918481	0.149167	0.091976821	0.057190239
3283	Q9BV36	MLPH	melanophilin	79083	ENSG00000115648	40	0.124	0.631	0.461	0.139	-0.03	-0.38	-0.22	-0.12	0.227	0.268	0.1524	-0.112	0.0719	0.09	0.1221	0.1887	0.0141	0.1148	-0.32	-0.7	-0.46	-0.34	-0.39	-0.01	-0.15	-0.14	0.224	0.094204	0.344802	-0.010034708	0.354836249
3386	Q9H446	RWDD1	RWD domain containing 1	51389	ENSG00000111832	7	0.05	0.153	-0.33	0.037	0.164	-0.29	0.02	0.057	0.164	0.1458	-0.129	-0.276	0.1702	0.1739	0.1942	0.0662	0.3659	0.0287	-0.19	-0.62	-0.26	-0.7	-0.09	0.186	0.049	-0.01	0.199	0.26107	0.163043	-0.078552464	0.241595274
1341	P01011	SERPINA3	serpin family A member 3;novel protein	12	ENSG00000196136	9	-0.53	-0.39	-0.51	-0.19	-0.29	-0.24	-0.51	0.327	-0.48	0.4115	0.1799	-0.359	-0.54	-0.247	-0.405	1.4267	-0.347	-0.127	0.375	0.038	0.689	1.046	0.261	0.078	0.059	-0.09	-0.64	0.226853	-0.51548	-0.31284701	-0.202628204
2857	Q8IXJ6	SIRT2	sirtuin 2	22933	ENSG00000068903	5	0.035	0.259	-0.08	-0.03	0.064	-0.37	0.044	-0.1	0.239	0.2432	0.069	0.228	-0.064	-0.132	0.3104	-0.012	0.2715	0.2492	-0.29	-0.82	-0.19	-0.31	-0.27	-0.02	-0.01	0.151	0.354	0.191948	0.16317	-0.122787496	0.285957056
1487	P07954	FH	fumarate hydratase	2271	ENSG00000091483	8	0.458	0.376	0.402	0.503	0.83	0.136	-0.36	0.137	-0.06	-0.062	-0.119	-0.122	0.2902	-0.076	0.0558	-0.311	-0.361	-0.264	-0.24	0.211	-0.32	-0.27	0.148	0.4	-0.2	-0.1	0.091	0.121148	0.301598	0.377136911	-0.075539069
3204	Q99487	PAFAH2	platelet activating factor acetylhydrolase 2	5051	ENSG00000158006	8	0.041	-0.03	0.276	0.321	0.082	-0.05	0.157	0.246	0.239	0.0212	0.0609	0.0505	0.0266	-0.297	-0.006	0.0564	0.0806	-0.221	-0.09	-0.12	0.125	-0.36	0.006	0.299	0.068	-0.01	0.044	0.186451	0.146777	0.168274384	-0.021496921
3447	Q9HD40	SEPSECS	Sep (O-phosphoserine) tRNA:Sec (selenoc	51091	ENSG00000109618	4	0.315	0.343	0.106				-0.03	-0.13	0.065	-0.484	0.0259	0.1924				0.0406	-0.102	0.0369	-1.96	-1.23	-0.24				0.285	-0.08	-0.03	0.261023	0.651631	0.158869303	0.492761304
501	C9J7I0	UMAD1	UBAP1-MVB12-associated (UMA) domain c	729852	ENSG00000219545	4	-0.25	0.497	-0.32	-0.13	-0.13	-0.26	-0.4	0.109	-0.47	-0.182	0.0529	0.227	-0.098	-0.147	-0.108	0.1369	-0.08	0.2689	-0.12	0.003	-0.5	-0.39	-0.59	-0.39	-0.34	-0.07	0.634	0.467502	0.045846	-0.158318965	0.204165342
2083	P53992	SEC24C	SEC24 homolog C, COPII coat complex cor	9632	ENSG00000176986	8	-0.03	-0.04	-0.13	0.098	0.183	-0.31	-0.27	0.068	0.107	-0.104	-0.212	0.0236	0.336	-0.044	0.0243	0.0103	0.1504	0.1015	-0.2	-0.61	-0.28	-0.07	-0.22	0.088	0.237	0.094	0.224	0.645621	0.045456	-0.067412696	0.112868903
680	E9PNK6	TPD52L1	TPD52 like 1	7164	ENSG00000111907	3	-0.18	0.327	-0.15	-0.28	-0.09	-0.33	-0.15	0.125	0.189	0.0134	-0.509	0.1296	0.2108	-0.07	0.1081	0.2594	-0.079	0.1675	-0.11	-0.41	-0.16	-0.37	-0.02	0.073	0.003	0.124	0.398	0.788656	-0.00761	-0.085780244	0.078170133
1252	O76094	SRP72	signal recognition particle 72	6731	ENSG00000174780	8	0.135	-0.05	0.169	-0.12	-0.04	-0.4	-0.17	0.074	0.483	0.0692	0.373	0.4195	-0.033	-0.053	0.0778	0.6015	0.5245	0.4533	-0.25	-0.8	-0.23	-0.38	-0.12	0.149	-0.22	0.111	-0.13	0.051464	0.21689	-0.261654547	0.47854453
1243	O75976	CPD	carboxypeptidase D	1362	ENSG00000108582	16	-0.12	-0.04	-0.19	-0.12	0.01	0.165	-0.09	0.182	0.156	0.2852	-0.314	-0.164	-0.288	-0.084	-0.597	0.3049	0.016	0.0683	-0.01	-0.07	-0.08	0.063	-0	-0.11	0.042	0.048	-0.07	0.747435	0.016411	0.081008773	-0.064598204
3505	Q9NSY0	NRBP2	nuclear receptor binding protein 2	340371	ENSG00000185189	5	0.065	-0	-0.23	0.294	0.346	-0.38	-0.22	-0.24	-0.1	-0.131	0.1636	0.0662	0.3271	0.0172	0.0826	-0.025	-0.2	-0.069	-0.41	-0.57	-0.09	-0.45	-0.25	0.042	0.091	0.023	-0	0.316453	0.127625	-0.076806271	0.20443125
3489	Q9NRW3	APOBEC3	apolipoprotein B mRNA editing enzyme cata	27350	ENSG00000244509	4				-0.07	-0.89	-0.14	-0.03	0.011	0.196				-0.475	-0.355	0.1117	0.2189	-0.071	0.2881				0.116	0.161	0.335	0.099	0.05	0.219	0.342037	-0.31645	-0.106040368	-0.21040612
1438	P05386	RPLP1	ribosomal protein lateral stalk subunit P1	6176	ENSG00000137818	3				-0.03	0.443	-0.42	0.63	0.457	0.782				-0.295	-0.192	-0.143	0.3929	-0.119	0.0187				-0.43	-0.17	-0.02	0.566	0.232	0.344	0.401342	0.222793	0.365777638	-0.142985115
466	F8W9W0	EPHA5	EPH receptor A5	2044	ENSG00000145242	4	-0.03	0.153	-0.18	-0.05	0.142	-0.34	-0.4	-0.11	-0.13	-0.233	0.1593	0.3302	-0.169	0.2736	0.0173	0.0358	0.0596	0.1351	-0.39	-0.32	-0.46	-0.75	-0.55	-0.04	-0.44	0.137	-0.05	0.069779	0.212786	-0.172168203	0.384954303
2730	Q6UX72	B3GNT9	UDP-GlcNAc:betaGal beta-1,3-N-acetylgluc	84752	ENSG00000237172	7	-0.06	-0.43	-0.06	-0.43	-0.45	-0.2	-0.27	0.207	0.13	0.1154	0.3097	0.1777	-0.672	0.4241	-0.339	0.1672	-0.074	0.0852	0.059	0.563	0.334	0.038	-0.18	-0.12	0.559	0.398	-0.14	0.204732	-0.34079	-0.194394542	-0.146392971
3640	Q9UJW0	DCTN4	dynactin subunit 4	51164	ENSG00000132912	7	-0.12	0.07	-0.38	-0.05	0.082	-0.19	-0.05	-0.21	0.041	-0.046	0.3561	-0.032	-0.084	-0.101	0.2127	-0.071	-0.163	-0.219	-0.27	0.017	0.041	-0.1	-0.13	0.084	-0.08	-0.15	0.014	0.698314	-0.02683	-0.074448213	0.047620362
3203	Q99471	PFDN5	prefoldin subunit 5	5204	ENSG00000123349	7	0.104	0.211	-0.15	-0.02	0.088	-0.59	0.132	0.203	0.31	0.0035	-0.048	-0.012	-0.125	-0.099	0.1913	0.3116	0.2482	0.0636	-0.21	-1.1	-0.31	-0.7	-0.31	-0.03	0.117	0.232	0.251	0.267606	0.261218	-0.026876532	0.288094189
3163	Q96L92	SNX27	sorting nexin family member 27;sorting nexi	81609	ENSG00000143376	6	0.153	0.24	-0.11	6E-04	-0.02	-0.49	0.008	0.039	0.198	0.0232	0.014	0.325	-0.018	-0.139	0.1074	0.1548	0.0209	0.2331	-0.19	-0.31	-0	-0.37	-0.37	0.083	0.074	0.122	0.271	0.435562	0.079318	-0.077704662	0.157023065
2407	Q13442	PDAP1	PDGFA associated protein 1	11333	ENSG00000106244	8	0.062	0.202	0.127	0.058	0.202	-0.36	-0.12	-0.29	4E-04	-0.029	0.0944	0.1658	0.1338	-0.144	0.2746	0.009	0.2649	-0.207	-0.25	-0.78	-0.43	-0.54	-0.33	0.011	-0.06	0.008	-0.1	0.092448	0.262421	-0.075728058	0.338148792
1203	O75355	ENTPD3	ectonucleoside triphosphate diphosphohydr	956	ENSG00000168032	9	-0.31	-0.09	-0.06	0.144	-0.01	-0.35	0.202	0.396	-0.06	-0.174	-0.009	0.1062	0.5715	-0.428	-0.188	-0.291	0.3752	0.2392	-0.29	0.254	-0.45	0.073	-0.44	-0.6	-0.3	-0.3	-0.27	0.226807	0.24201	-0.037950222	0.279960008
1167	O60603	TLR2	toll like receptor 2	7097	ENSG00000137462	10	-0.16	-0.18	-0.15	-0.65	-0.38	-0.25	-0.14	0.104	0.098	-0.15	-0.1	-0.26	-0.527	-0.253	-0.304	-0.055	-0.157	-0.043	-0.18	0.861	0.094	0.961	-0.01	-0.28	0.255	-0.15	0.108	0.120659	-0.37473	0.015069388	-0.389801667
2176	P61962	DCAF7	DDB1 and CUL4 associated factor 7	10238	ENSG00000136485	4	-0.11	-0.02	-0.11	0.043	0.066	-0.24	-0.13	-0.14	0.06	-0.01	-0.145	-0.003	0.4949	-0.179	-0.204	0.0125	0.081	0.0006	-0.22	-0.43	-0.01	0.052	-0.07	0.219	-0.04	0.023	-0.01	0.749221	-0.01381	-0.071918649	0.058113329
874	H7BY83	WDR44	WD repeat domain 44	54521	ENSG00000131725	8	0.201	0.173	-0.18	-0.06	0.1	-0.28	-0.02	0.061	0.165	0.2946	-0.085	0.1785	0.0567	-0.076	0.1725	0.0833	0.1773	0.1088	-0.2	-0.48	-0.29	-0.43	-0.27	-0.03	0.272	0.085	0.197	0.198518	0.14496	-0.083433589	0.228393646
1771	P27701	CD82	CD82 molecule	3732	ENSG00000085117	7	-0.45	0.559	0.392	-0.71	0.003	0.322	-1.01	-0.51	-0.84	0.0651	0.648	-0.093	0.2353	-0.088	-0.252	-0.911	-0.115	-0.563	-0.35	0.691	-0.22	-0.23	-0.21	-0.11	-0.27	-0.32	-0.45	0.88901	-0.08603	-0.131019799	0.044994455
3612	Q9UHD8	SEPTIN9;S	septin 9	10801	ENSG00000184640	6	-0.19	0.076	-0.15	-0.27	0.042	-0.56	0.105	0.119	0.141	0.1682	0.0473	0.2174	-0.004	-0.231	0.0199	-0.027	0.2132	0.1585	-0.24	-0.26	0.152	0.222	-0.1	-0.03	0.217	-0.08	0.217	0.48589	-0.08773	-0.13873303	0.051004496
2780	Q7Z3J2	VPS35L	VPS35 endosomal protein sorting factor like	57020	ENSG00000103544	9	-0.16	-0.14	-0.06	0.133	-0.23	-0.19	0.003	0.065	0.197	0.1793	0.3135	0.0089	0.0615	0.1179	0.0515	0.0037	0.1415	0.3041	-0.39	-0.62	0.046	-0.07	-0.16	0.146	-0.23	-0.01	0.172	0.129143	0.084274	-0.172780362	0.257054518
1351	P01111	NRAS	NRAS proto-oncogene, GTPase	4893	ENSG00000213281	10	-0.11	-0.29	-0.07	0.29	0.148	0.07	-0.05	0.008	0.02	-0.084	-0.186	0.0893	0.1714	-0.091	0.1668	0.0913	0.3184	0.1199	-0.13	-0.42	-0.3	0.31	-0.03	0.145	-0.14	0.03	0.237	0.665979	0.034256	-0.065126291	0.099382355
3510	Q9NTX5	ECHDC1	ethylmalonyl-CoA decarboxylase 1	55862	ENSG00000093144	8	0.332	0.245	0.014	-0.16	-0.17	-0.55	0.133	0.12	0.351	0.0547	-0.045	0.1939	-0.438	-0.166	-0.105	0.3168	0.1499	0.2137	-0.13	-0.71	-0.23	-0.68	-0.21	-0.44	0.13	0.2	0.005	0.252773	0.264599	0.015266401	0.249332195
1174	O60704	TPST2	tyrosylprotein sulfotransferase 2	8459	ENSG00000128294	6	-0.09	-0.6	0.021	-0.48	-0.38	0.061	-0.03	0.238	0.043	0.2752	-0.13	-0.057	-0.584	0.3176	0.2122	-0.194	-0.709	-0.435	-0.05	0.707	0.251	-0.16	0.071	-0.27	0.281	0.008	-0.12	0.421525	-0.21518	0.010033738	-0.225214868
2374	Q13049	TRIM32	tripartite motif containing 32	22954	ENSG00000119401	3	-0.36	0.089	0.052	0.084	0.732	-0.81	0.143	0.044	0.009	0.0864	-0.044	-0.085	0.6138	-0.102	-0.59	0.3176	0.158	0.1699	-0.19	-0.07	0.207	-0.69	-0.28	0.239	-0.01	0.119	0.201	0.867088	0.051682	-0.05978305	0.11146529
191	A0A0C4DGE	ARHGAP2	Rho GTPase activating protein 27	201176	ENSG00000159314	4	-0.28	-0.18	-0.44	0.106	-0.1	0.046	0.309	0.005	-0.03	-0.033	-0.407	-0.284	0.0082	-0.176	-0.254	1.2821	-0.097	0.147	-0.12	-0.09	0.208	0.195	0.124	0.085	0.015	0.017	0.075	0.808688	-0.11985	-0.0844414	-0.035405647
1497	P08243	ASNS	asparagine synthetase (glutamine-hydrolyzi	440	ENSG00000070669	5	-0.02	-0.02	0.225				-0.22	0.105	-0.07	-0.475	-0.141	-0.007				-0.064	0.0163	0.0876	-0.41	-0.54	-0.26				0.129	-0.23	-0.09	0.305999	0.236063	0.099157422	0.136905901
3031	Q92520	FAM3C	family with sequence similarity 3 member C	10447	ENSG00000196937	6	0.035	-0.95	-0.5	-0.04	-0.22	-0.24	-0.51	-0.35	-0.26	0.3473	0.5456	0.1126	-0.333	0.1238	0.1952	0.0045	-0.564	-0.549	0.09	0.411	0.088	0.507	-0.04	-0.14	0.31	-0.13	-0.52	0.164729	-0.40124	-0.323889832	-0.077350156
2976	Q8TD19	NEK9	NIMA related kinase 9	91754	ENSG00000119638	11	0.188	0.142	0.038	-0	-0.01	-0.43	0.112	0.032	0.261	0.2491	0.1349	0.1758	0.0597	-0.11	-0.035	0.1321	0.1998	0.1058	-0.06	-0.53	0.135	-0.34	-0.23	0.097	0.142	0.046	0.093	0.328057	0.109056	-0.063964099	0.173019908
642	E9PFN5	GSTK1	glutathione S-transferase kappa 1	373156	ENSG00000197448	4	0.368	-0.18	-0.18	-0.52	0.153	0.076	0.298	0.275	-0.2	0.0081	0.2806	0.0609	-0.268	0.452	0.2745	0.2175	0.3094	0.1008	0.204	0.624	0.797	0.12	0.336	-0.25	-0.11	-0.3	-0.53	0.738828	-0.08999	-0.149929009	0.059943665
1582	P13284	IFI30	IFI30, lysosomal thiol reductase;IFI30 lysos	10437	ENSG00000216490	2	0.163	-0.5	-0.28	-0.12	-0.14	-0.16	0.162	-0.17	0.239	-0.134	0.0187	0.0778	-0.005	0.3018	-0.059	0.1976	0.3232	-0.053	0.196	0.287	0.282	0.79	0.308	0.196	0.038	-0.15	-0.03	0.146906	-0.30302	-0.164200238	-0.138823845
313	A0A2R8Y48	SPAST	spastin	6683	ENSG00000021574	7	0.042	0.236	-0.49	-0.15	0.071	-0.67	-0.04	0.018	0.009	-0.443	-0.184	0.1747	-0.306	-0.166	-0.108	0.1537	0.119	0.1464	-0.17	-0.31	-0.38	-0.65	-0.47	0.139	0.045	0.045	0.454	0.907101	0.035702	-0.040264354	0.075966678
2530	Q15714	TSC22D1	TSC22 domain family member 1	8848	ENSG00000102804	5	0.186	0.118	-0.13	0.072	0.238	-0.02	0.336	0.175	0.57	0.1201	0.0398	0.0646	-0.174	-0.166	0.2397	0.3094	0.2589	0.208	-0.24	-0.67	0.211	0.95	-0.01	0.169	0.261	0.433	0.592	0.880176	-0.01778	0.071280348	-0.089056607
1647	P17081	RHOQ	ras homolog family member Q	23433	ENSG00000119729	7	-0.14	0.194	-0.14	0.002	-0.31	-0.48	-0.34	-0.12	-0.35	0.0649	-0.038	-0.093	-0.066	-0.031	0.1983	0.1529	0.1788	0.0948	-0.38	-0.7	-0.27	-0.73	-0.41	0.163	-0.13	-0.08	0.136	0.125429	0.07881	-0.238942982	0.317753429
755	H7C106	RTN4	reticulon 4	57142	ENSG00000115310	1	-0.02	-0.1	-0.18	-0.04	0.308	-0.63	-0.22	-0.28	0.004	-0.419	-0.332	-0.152	-0.106	-0.487	-0.298	-0.137	-0.052	0.0875	-0.73	-0.8	-0.2	0.935	-0.21	0.053	0.029	-0.06	-0.12	0.887083	-0.00607	0.082326755	-0.088392541
1378	P02144	MB	myoglobin	4151	ENSG00000198125	7	-0.11	-0.17	0.225	-0.33	-0.47	0.139	0.238	-0.46	0.438	0.2501	0.5341	0.0613	-0.227	-0.006	0.2718	-0.215	0.2016	0.1386	0.041	-0.3	0.159	-0.41	-0.29	0.087	-0.01	-0.07	0.154	0.446944	0.015128	-0.167850534	0.182978221
978	M0QZR4	ARHGEF1	Rho guanine nucleotide exchange factor 1	9138	ENSG00000076928	7	0.191	0.249	-0.03	-0.4	-0.3	-0.33	0.074	-0.01	0.196	0.1239	0.1774	0.1691	-0.37	-0.045	-0.395	0.1653	0.055	0.1536	-0.33	-0.95	0.028	-0.1	-0.13	-0.13	0.212	-0.01	0.162	0.65977	0.101573	-0.042978031	0.144550665
30	A0A096LNJ	ALG13	ALG13 UDP-N-acetylglucosaminyltransfera	79868	ENSG00000101901	3	0.025	0.189	0.203	0.05	-0.07	-0.43	0.158	0.457	0.286	0.0357	-0.034	-0.198	0.2057	-0.16	0.1424	0.3635	0.2796	0.1653	-0.45	-0.59	-0.28	-0.51	-0.19	0.01	0.534	0.136	0.485	0.485885	0.190834	0.0069187	0.183914839
1101	O43147	SGSM2	small G protein signaling modulator 2	9905	ENSG00000141258	12	0.027	-0.64	-0.28	-0.31	-0.07	-0.3	-0.2	-0.15	0.107	-0.397	-0.209	-0.275	-0.244	-0.095	-0.231	0.4216	0.4598	0.2585	0.343	-0.13	0.61	-0.44	-0.15	-0.07	-0.04	-0.24	0.144	0.463813	-0.20342	-0.166768492	-0.036655212
3085	Q96A59	MARVELD	MARVEL domain containing 3	91862	ENSG00000140832	8	-0.43	0.337	-0.47	-0.05	0.049	-0.41	-0.43	-0.15	-0.31	-0.482	0.0469	0.3287	0.1345	-0.142	-0.02	-0.011	-0.513	0.037	-0.56	-0.2	-0.41	-0.59	-0.53	-0.28	-0	0.023	-0.11	0.361847	0.087069	-0.138339781	0.225408561
2199	P62633	CNBP	CCHC-type zinc finger nucleic acid binding	7555	ENSG00000169714	6	0.405	0.479	0.267	-0.04	-0.09	-0.03	-0.3	-0.09	-0.02	0.2782	0.2608	0.1879	0.0316	0.0627	0.0315	-0.046	0.0359	-0.163	0.093	-0.52	0.044	-0.04	-0.16	0.216	-0.05	-0.15	0.135	0.549402	0.113537	-0.009906548	0.123443683

2230	P63096	GNAI1	G protein subunit alpha i1	2770	ENSG00000127955	16	-0.05	0.72	-0.28	0.46	0.092	-0.19	-0.29	-0.39	-0.08	-0.068	-0.286	0.1649	-0.026	0.2082	0.7139	0.2645	-0.39	-0.31	-0.47	-0.43	-0.61	-0.52	-0.69	-0.43	-0.22	0.345	0.095	0.219158	0.322775	-0.033350959	0.356126423
2584	Q4G0F5	VPS26B	VPS26, retromer complex component B	112936	ENSG00000151502	8	0.119	0.189	0.187	0.365	-0.39	0.013	-0.05	-0.11	0.189	0.3036	-0.007	0.0087	-0.122	0.4082	-0.007	0.0544	0.1212	0.0822	-0.21	-0.56	-0.1	-0.08	-0.43	-0.15	-0	-0.04	0.064	0.125642	0.224743	-0.036397865	0.261140401
2756	Q711Q0	C10orf71;C	chromosome 10 open reading frame 71	118461	ENSG00000177354	3	0.667	-1.04	-1.17	-0.78	-0.14	0.091	-0.7	-0.16	-0.42	2.1163	-0.202	-0.202	0.1986	0.2696	0.3236	-0.056	-0.947	-0.485	1.556	0.746	-0.01	0.239	0.649	-0.49	0.71	-0.54	-0.56	0.303491	-0.66093	-0.518237175	-0.142691163
3601	Q9UGK3	STAP2	signal transducing adaptor family member 2	55620	ENSG00000178078	7	0.05	-0.1	0.067	-0.09	0.056	-0.26	0.117	0.324	0.106	-0.036	0.2666	0.2481	0.3009	-0.22	-0.057	0.1956	0.0566	0.0431	-0.34	-0.58	-0.3	0.275	-0.19	-0.04	0.041	0.159	0.316	0.472727	0.102658	-0.058778308	0.161436441
2777	Q7Z2Z2	EFL1	elongation factor like GTPase 1	79631	ENSG00000140598	6	0.019	0.012	0.224	0.005	-0.12	-0.62	0.33	0.28	0.302	0.3616	0.0893	0.1861	-0.072	-0.076	0.002	0.3107	0.1194	0.2888	0.011	-0.12	-0.06	-0.41	-0.4	0.082	0.24	0.163	0.49	0.667443	0.04736	-0.086157549	0.133517333
211	A0A1B0GVH	TBL1XR1	transducin beta like 1 X-linked receptor 1	79718	ENSG00000177565	3	0.079	0.548	0.6	0.12	0.491	-0.09	-0.49	-0.02	-0.23	-0.048	0.0314	-0.011	0.5053	-0.361	-0.072	-0.004	0.0632	-0.064	-0.19	-0.89	-0.32	-0.14	-0.37	0.264	-0.2	-0.05	-0.14	0.21126	0.339064	0.107686937	0.23137719
1309	O95786	DDX58	DExD/H-box helicase 58	23586	ENSG00000107201	13	-0.17	0.152	-0.33	-0.58	-0.56	-1.08	-0.22	-0.1	0.033	0.0385	-0.004	0.2115	-0.319	-0.338	-0.455	0.259	0.009	-0.019	-0.02	-0.54	-0.13	-0.49	-0.55	0.435	0.26	0.25	-0.04	0.401255	-0.22646	-0.248561614	0.022098077
3740	Q9Y3D6	FIS1	fission, mitochondrial 1	51024	ENSG00000214253	4	-0.21	-0.21	-0.39	-0	0.079	-0.31	0.139	-0.03	0.184	-0.353	-0.306	-0.084	0.0291	-0.277	0.0029	0.0814	0.1551	-0.033	-0.42	-0.51	-0.63	-0.5	-0.39	-0.06	-0.09	0.07	0.145	0.328007	0.181511	0.003290414	0.178220293
1938	P45984	MAPK9	mitogen-activated protein kinase 9	5601	ENSG00000050748	9	-0.02	-0.22	-0.33	-0.02	0.186	-0.17	-0.3	-0.04	0.159	0.389	-0.271	-0.024	0.2565	-0.03	0.2295	-0.023	-0.001	-0.094	0.432	0.258	0.271	-0.18	0.073	0.289	-0.01	0.006	0.141	0.195221	-0.2264	-0.131922914	-0.09447702
3649	Q9UKZ4	TENM1	teneurin transmembrane protein 1	10178	ENSG00000009694	10				0.659	0.249	-0.35	-0.56	0.116	0.028				0.092	-0.054	-0.113	0.455	-0.074	-0.002				-0.39	-0.47	-0.06	0.45	0.118	-0.19	0.833561	0.112773	-0.027813947	0.140586908
2378	Q13107	USP4	ubiquitin specific peptidase 4	7375;10798(	ENSG00000114316	7	0.147	0.377	-0.06	0.256	-0.02	-0.33	-0.13	0.047	0.097	0.2114	0.0176	0.0794	0.2851	-0.154	-0.007	0.0526	0.2245	0.1497	0.029	-0.25	-0.11	-0.38	-0.29	-0.05	0.004	0.132	0.179	0.269766	0.126268	-0.052264562	0.178532534
912	J3KNN3	PHKG2	phosphorylase kinase catalytic subunit gamı	5261	ENSG00000156873	8	0.018	-0.03	-0.04	0.307	0.831	-0.16	0.108	-0.07	0.084	0.0642	0.0145	0.1456	-0.012	-0.336	-0.194	-0.141	0.2428	0.3999	-0.07	-0.23	-0.09	-0.34	-0.41	-0.13	0.113	-0.07	0.196	0.30413	0.231352	0.096488204	0.134864022
732	F8VRR3	METAP2	methionyl aminopeptidase 2	10988	ENSG00000111142	4	-0.42	0.751	-0.68	0.117	-0.13	-0.1	0.041	0.013	0.005	-0.263	-0.228	0.2526	-0.168	-0.067	-0.094	-0.116	0.0294	0.1797	-0.65	-0.46	-0.6	-0.13	-0.18	-0.06	0.195	-0.08	-0.09	0.503696	0.182662	0.008478011	0.174184111
81	A0A087WZH	EIF3H	eukaryotic translation initiation factor 3 subu	8667	ENSG00000147677	15	0.05	0.149	-0.09	-0.08	0.311	-0.27	-0.03	-0.3	0.011	-0.042	-0.011	0.0558	-0.076	-0.158	0.1892	-0.026	0.0834	0.1916	-0.01	-0.47	0.164	-0.39	-0.15	-0.01	0.282	-0.33	-0.2	0.451108	0.096104	-0.049356379	0.145460613
3529	Q9NX46	ADPRHL2	ADP-ribosylhydrolase like 2	54936	ENSG00000116863	2				0.206	-0.25	-0.24	0.016	0.226	-0.02				0.1875	0.0757	-0.059	1.3065	-0.074	0.2374				-0.49	-0.3	-0.18	0.095	0.324	-0.06	0.355498	0.091432	-0.288561902	0.379994234
2726	Q6UWU2	GLB1L	galactosidase beta 1 like	79411	ENSG00000163521	6	0.013	-0.28	0.055	0.205	0.169	-0.44	-0.12	0.853	0.116	-0.065	-0.053	0.2564	-0.195	-0.078	-0.022	-0.238	-0.746	-0.319	-0.02	0.002	-0.03	0.211	-0.22	0.139	0.408	0.096	0.11	0.303515	-0.01395	0.225777634	-0.239726661
2921	Q8NBJ4	GOLM1	golgi membrane protein 1	51280	ENSG00000135052	3				-0.16	-0.11	-0.7	-0.29	-0.1	0.41				-0.371	-0.412	-0.73	0.5233	0.2926	-0.262				-0.01	-0.59	0.219	-0.15	-0.06	-0.54	0.991686	0.029872	0.001843474	0.02802812
1927	P42858	HTT	huntingtin	3064	ENSG00000197386	8	-0.32	-0.21	-0.21	-0.13	-0.12	-0.52	-0.07	-0.03	0.055	0.0591	-0.381	-0.151	0.0376	-0.183	0.0999	0.0463	-0.018	0.0493	-0.31	-0.65	-0.3	-0.34	-0.29	-0.09	0.313	-0.04	-0.02	0.450761	0.018556	-0.124402711	0.14295833
2474	Q14696	MESD	mesoderm development LRP chaperone	23184	ENSG00000117899	2				-0.11	0.057	-0.13	0.151	0.219	-0.01				-0.557	0.1608	0.0541	0.3535	-0.033	0.1567				-0.14	-0.22	-0.32	0.364	0.009	0.081	0.916317	0.06661	0.006513879	0.060096083
3678	Q9UNQ0	ABCG2	ATP binding cassette subfamily G member	9429	ENSG00000118777	8	-0.47	-0.29	0.057	-1.77	-0.22	0.508	-0.7	-0.29	0.082	0.0451	0.3465	-0.661	-0.665	-0.347	-0.18	-0.953	0.3317	0.0043	-0	1.972	-0.43	-0	-0.24	-1.01	0.38	-0.31	0.114	0.572355	-0.39834	-0.114309722	-0.284028265
63	B7Z879	VPS11	VPS11 core subunit of CORVET and HOPS	55823	ENSG00000160695	9	-0.33	-0.06	-0.2	-0.15	-0.04	-0.21	0.006	0.168	0.157	0.148	-0.443	0.0398	-0.065	-0.226	-0.097	0.3663	0.1083	0.2484	0.112	-0.45	0.071	-0.37	-0.16	-0.03	0.368	0.055	0.216	0.826087	-0.05377	-0.082212757	0.028444032
2477	Q14703	MBTPS1	membrane bound transcription factor peptid	8720	ENSG00000140943	11	-0.08	0.026	-0.13	-0.2	-0.16	-0.17	-0.36	0.102	-0.13	0.0062	0.0853	0.0798	-0.332	-0.003	0.034	0.3074	-0.32	-0.294	-0.07	0.126	0.055	-0.5	-0.4	-0.35	0.622	-0.13	-0.23	0.871872	-0.02654	-0.075265356	0.048729772
3607	Q9UHA4	LAMTOR3	late endosomal/lysosomal adaptor, MAPK aı	8649	ENSG00000109270	2	0.108	0.345	0.121	0.087	0.133	-0.27	0.118	-0.3	0.237	0.1781	0.3652	0.072	0.1747	0.0468	0.4265	-0.026	0.1607	0.0701	-0.2	-0.62	-0	-0.54	-0.38	0.067	-0.32	0.121	0.275	0.116906	0.240777	-0.09910185	0.339878693
3441	Q9HCM3	KIAA1549	KIAA1549	57670	ENSG00000122778	5	-0.4	0.301	0.009	-0.28	-0.23	-0.52	0.354	-0.21	0.056	0.1334	0.0498	-0.014	-0.222	-0.299	-0.198	-0.133	0.0346	-0.064	0.01	-0.01	-0.2	-0.53	-0.34	-0.1	0.089	-0.3	-0.11	0.790323	0.063561	-0.022943979	0.086505133
238	A0A0J9YW8	KCNN2	potassium calcium-activated channel subfar	3781	ENSG00000080709	6				-0.01	0.128	-0.34	-0.04	0.245	-0.06				-0.064	-0.03	0.1246	0.9072	-0.042	0.56				-0.63	0.106	-0.17	0.025	0.122	0.202	0.392475	0.044845	-0.255377427	0.300222109
281	A0A1B0GW	PRRT1B	proline rich transmembrane protein 1B	642515	ENSG00000283526	5	-0.03	0.084	0.06	-0.17	-0.52	0.392	-0.43	-0.13	-0.59	0.5493	0.3944	-0.092	0.0857	0.2814	0.0861	-0.014	-0.189	-0.339	-0.28	-0.31	-0.26	-0.3	-0.01	-0.11	0.204	0.054	-0.1	0.302159	-0.02506	-0.23347389	0.208417078
1966	P48066	SLC6A11	solute carrier family 6 member 11	6538	ENSG00000132164	3	-0.08	0.528	-0.42	-0.02	-0.29	-0.14	-0.49	0.158	-0.56	-0.248	-0.081	0.009	0.841	-0.301	0.3615	0.6876	-0.046	0.174	-0.43	-0.29	-0.38	-0.97	-0.39	-0.37	-0.33	-0.23	0.62	0.17824	0.161423	-0.302256718	0.463680161
2875	Q8IZF0	NALCN	sodium leak channel, non-selective	259232	ENSG00000102452	1	-0.61	0.477	-0.13	-0.28	-0.19	-0.88	-0.17	0.226	0.196	-0.255	0.0722	0.1547	0.0533	-0.016	-0.9	0.1826	0.3587	0.4274	-0.45	0.169	-0.35	-1.27	-0.48	0.045	-0.37	0.005	0.12	0.501366	0.133824	-0.16087504	0.294698752
3368	Q9H269	VPS16	VPS16, CORVET/HOPS core subunit;VPS	64601	ENSG00000215305	6	0.105	0.544	0.055	0.001	0.112	-0.33	-0.02	0.01	0.092	0.4406	-0.05	0.107	0.0826	-0.226	-0.072	0.2599	-0.028	0.1774	-0.1	0.005	0.463	0.308	-0.33	0.253	0.247	-0.04	0.231	0.918963	-0.05177	-0.013333297	-0.03843363
562	D6RAP6	ANAPC4	anaphase promoting complex subunit 4	29945	ENSG00000053900	2							0.088	-0.13	0.164							0.806	-0.023	0.3356							0.284	0.314	0.1	0.5184	-0.19163	-0.331916802	0.140284449
3503	Q9NSD9	FARSB	phenylalanyl-tRNA synthetase subunit beta	10056	ENSG00000116120	10	0.037	-0.3	-0.11	0.159	0.104	0.198	0.163	0.079	0.154	0.0045	-0.09	0.0292	-0.228	-0.199	-0.519	-0.183	-0.193	-0.188	-0.17	-0.1	0.315	0.17	0.234	0.057	0.211	-0.12	-0.2	0.118346	0.011519	0.228391903	-0.216873271
2397	Q13308	PTK7	protein tyrosine kinase 7 (inactive)	5754	ENSG00000112655	11	-0.06	0.105	0.33	0.036	-0.05	-0.08	0.262	-0.08	0.025	0.1381	-0.072	0.1099	-0.049	-0.137	-0.143	0.1567	0.2002	-0.018	-0.38	-0.18	0.202	-0.39	-0.1	-0.02	0.103	-0	0.178	0.483096	0.119807	0.03335105	0.086456226
606	E7EQ69	NAA50	N(alpha)-acetyltransferase 50, NatE catalyti	80218	ENSG00000121579	5				-0.24	-0.81	-0.02	0.17	-0.13	0.309				-0.033	0.3473	-0.25	0.0701	0.195	0.2755				-0.04	-0.02	0.782	-0.03	-0.01	0.1	0.517745	-0.24771	-0.219959209	-0.027755454
1064	O15020	SPTBN2	spectrin beta, non-erythrocytic 2	6712	ENSG00000173898	10	-0.26	-1.3	-0.22	0.017	0.189	-0.38	-0.27	0.048	-0.02	0.1784	0.8398	-0.347	-0.497	-0.335	-0.335	0.8477	-1.119	-0.646	-0.27	0.555	-0.52	0.449	-0.02	-0.17	0.693	0.444	-0.38	0.547532	-0.33128	-0.087755279	-0.243529673
984	M0R2A0	EMC10	ER membrane protein complex subunit 10	284361	ENSG00000161671	3	-0.4	-0.05	-0.32	-0.96	-0.24	0.022	-0.39	-0.19	-0.17	0.0646	-0.269	-0.271	-0.474	-0.021	0.1209	-0.061	-0.549	-0.128	-0.11	0.305	0.129	-0.35	-0.21	0.173	0.606	-0.26	-0.55	0.331797	-0.27006	-0.123329757	-0.146727628
256	A0A0U1RRE	EXOC6B	exocyst complex component 6B	23233	ENSG00000144036	8	0.275	0.275	0.184	0.441	0.14	-0.16	-0.1	-0.07	0.217	0.3391	0.477	0.5423	0.2028	0.0712	0.2971	0.1313	0.1511	0.14	-0.25	-0.37	-0.07	-0.32	-0.02	0.083	-0.03	0.303	0.302	0.122077	0.174607	-0.128006303	0.302613662
503	C9J813	CALD1	caldesmon 1	800	ENSG00000122786	3							-0.45	-0.25	0.068							-5E-04	0.666	-0.021							-0.53	0.041	-0.06	0.43244	-0.03258	-0.428035878	0.395455941
385	O43768	ENSA	endosulfine alpha	2029	ENSG00000143420	6	0.112	0.54	0.225	-0.16	0.041	-0.34	-0.05	-0.17	0.127	0.067	0.2354	0.1512	-0.02	-0.116	0.134	0.0682	0.3314	-0.108	-0.17	-0.48	-0.24	-0.49	-0.3	0.071	0.048	-0.08	-0.06	0.136104	0.224171	-0.046386673	0.270557294
433	F5H2D0	C1R	complement C1r	715	ENSG00000159403	8	-0.11	-0.2	-0.04	-0.16	-0.26	0.056	-0.27	0.067	-0.17	-0.081	0.1434	0.2162	-0.513	0.2185	-0.404	-0.011	-0.552	-0.3	-0.13	-0.15	0.936	1.441	0.02	0.15	0.437	-0.09	-0.49	0.235805	-0.35751	0.021383988	-0.378896815
344	B1ALK7	ARHGEF7	Rho guanine nucleotide exchange factor 7	8874	ENSG00000102606	10	0.14	0.115	9E-04	0.255	-0.28	-0.07	0.232	-0.06	0.345	0.0593	0.0333	0.0803	-0.231	0.1049	0.2685	0.3633	0.0781	0.1846	-0.05	-0.46	-0.1	-0.53	-0.46	-0.36	0.643	0.105	0.189	0.343843	0.189976	-0.029232157	0.219207723
1872	P36406	TRIM23	tripartite motif containing 23	373	ENSG00000113595	1	-0.04	0.489	0.026	0.088	0.065	-0.35	-0.72	-0.66	-0.69	-0.119	0.1237	0.2044	0.3394	-0.086	-0.011	-0.482	-0.085	-0.388	-0.18	-0.71	-0.35	-0.52	-0.47	0.101	-0.74	-0.17	-0.3	0.293712	0.17521	-0.142660864	0.317870391
939	K4DI92	RWDD4	RWD domain containing 4	201965	ENSG00000182552	3	0.27	0.286	0.027	0.404	-0.09	0.133	-0.17	-0.18	0.117	0.1041	0.2551	0.1283	0.0393	0.0747	0.1942	-0.096	0.022	-0.127	-0.07	-0.9	-0.4	-0.1	-0.02	0.09	0.038	0.021	0.108	0.234238	0.227084	0.023564104	0.203520126
3285	Q9BV44	THUMPD3	THUMP domain containing 3	25917	ENSG00000134077	6	0.108	0.028	0.094	-0.13	-0.39	0.534	0.154	0.219	0.249	0.0561	-0.214	0.0485	-0.184	0.0306	-0.028	-0.033	0.0931	0.2168	0.03	-0.49	-0.24	-0.11	0.082	-0.09	-0.04	0.072	0.368	0.524676	0.143521	0.097244869	0.046276012
169	A0A0A0MTS	TTN	titin	7273	ENSG00000155657	3	0.1	0.031	-0.07	0.549	-0.69	1.753	0.161	0.387	-0.17	0.0183	-0.188	0.1874	-0.399	0.2801	0.7283	0.2782	-0.112	0.5525	-0.12	-0.74	-0.4	0.605	-0.16	-0.44	-0.1	-0.15	0.218	0.419524	0.371775	0.078798593	0.292976489
2779	Q7Z392	TRAPPC1	trafficking protein particle complex 11	60684	ENSG00000168538	5	-0.01	0.039	-0.21				-0.15	-0.13	0.123	0.0093	-0.055	0.0296				-0.069	0.1428	0.1586	-0.16	-0.62	-0.28				0.159	0.012	-0.05	0.380355	0.096931	-0.093390413	0.190321398
2646	Q5TCJ5	SEMA4A	semaphorin 4A	64218	ENSG00000196189	5	-0.05	0.095	-0.25	-0.2	-0.43	-0.45	-0.11	0.257	0.255	-0.238	-0.186	0.0855	-0.182	-0.471	-0.045	0.3222	0.2528	0.4248	-0.49	-0.37	-0.18	-0.31	-0.78	-0.37	-0.11	0.124	0.447	0.478812	0.129255	-0.093114279	0.222369281
1530	P0CG12	DERPC;CI	DERPC proline and glycine rich nuclear pro	113455421;!	ENSG00000286140	4	-0.3	0.43	-0.27	-0.35	-0.32	0.026	0.269	0.638	0.29	0.1515	0.065	-0.063	-0.233	-0.191	-0.05	0.4769	-0.007	0.651	-0.05	-0.08	-0.32	0.32	-0.34	-0.1	0.133	0.254	0.713	0.973441	-0.01324	-0.042809831	0.029569135
3242	Q9BQA1	WDR77	WD repeat domain 77	79084	ENSG00000116455	6	0.234	0.291	-0.04	0.13	0.162	-0.13	0.191	0.141	0.087	0.0237	0.0915	0.1439	0.4172	0.2558	-0.144	0.1936	-0.093	-0.022	-0.11	-0.58	-0.02	-0.24	0.041	0.131	0.374	0.072	0.026	0.395221	0.151813	0.021604388	0.130208658
2774	Q7LG56	RRM2B	ribonucleotide reductase regulatory TP53 in	50484	ENSG00000048392	9	0.412	0.431	0.128	0.014	0.306	0.004	-0.15	-0.2	-0.01	0.4466	0.4011	0.2136	0.0903	0.0116	0.1783	-0.082	0.0165	-0.236	-0	-0.58	-0.16	-0.3	-0.14	0.256	-0.06	-0.1	0.015	0.202635	0.222739	-0.011197339	0.233936342
1184	O60890	OPHN1	oligophrenin 1	4983	ENSG00000079482	7	0.072	0.108	0.062	0.119	0.292	-0.3	0.329	0.354	0.491	-0.088	-0.012	0.0729	0.2097	0.1269	0.4173	0.3781	0.1233	0.0724	-0.38	-0.54	-0.07	-0.22	-0.29	-0.13	0.576	0.313	0.542	0.470222	0.192799	0.025550097	0.167249324
3631	Q9UIQ6	LNPEP	leucyl and cystinyl aminopeptidase	4012	ENSG00000113441	10	-0.41	-0.24	-0.16	-0.2	-0.04	-0.37	-0.06	0.014	-0.05	0.6746	-0.013	0.4044	0.0082	-0.045	-0.251	0.0106	0.086	0.0043	-0.41	-0.08	-0.36	-0.25	0.004	-0.06	0.169	0.69	-0.23	0.268359	-0.11036	-0.265909163	0.155550915
3075	Q969Q0	RPL36AL	ribosomal protein L36a like	6166	ENSG00000165502	6	0.151	-0.19	-0.14	0.011	0.619	-0.07	-0.5	-0.42	-0.25	-0.197	-0.144	-0.332	0.0771	0.1104	-0.017	-0.502	0.129	-0.157	0.045	-0.36	-0.11	-0.05	0.14	0.252	-0.15	-0.41	0.125	0.931617	-0.0303	0.027249374	-0.05754924
3025	Q8WZ82	OVCA2	OVCA2 serine hydrolase domain containing	124641	ENSG00000262664	3	-0.14	0.256	-0.15				-0.13	0.041	0.197	0.3114	0.1358	0.1612				0.0271	0.1375	0.0527	-0.08	-0.29	-0.02				-0.23	0.089	0.248	0.328459	0.061813	-0.124972916	0.186785523
1216	O75556	SCGB2A1	secretoglobin family 2A member 1	4246	ENSG00000124939	3	-0.31	-0.71	0.83	0.084	-0.92	-0.58	-0.42	0.527	-0.12	0.1276	0.5396	0.26	-0.1	0.3225	0.0454	0.7536	-0.412	-0.003	-1.25	-0.77	0.52	-1.02	-0.69	-0.65	-1.3	0.754	-0.76	0.152329	0.395648	-0.349963852	0.745611377
2817	Q86WV7	CCDC43	coiled-coil domain containing 43	124808	ENSG00000180329	5	-0.23	0.056	-0.05	-0.03	0.022	-0.39	0.248	-0.27	0.151	-0.097	0.2189	-0.092	0.0654	0.0066	0.2947	0.1983	-0.131	-0.202	-0.4	-1	-0.57	-0.45	-0.17	0.105	0.248	0.495	0.212	0.565854	0.115217	-0.083818667	0.199035723
610	Q05397	PTK2	protein tyrosine kinase 2	5747	ENSG00000169398	7				-0.13	-0.26	-0.23	0.002	0.666	-0.09				-0.279	-0.13	-0.021	0.3079	-0.072	0.1933				-0.35	-0.45	-0.03	0.506	0.077	0.315	0.997217	-0.01602	-0.00491609	-0.01110302
644	E9PG46	AAK1	AP2 associated kinase 1	22848	ENSG00000115977	7	-0.05	0.102	-0.16	-0.14	0.004	-0.43	0.021	0.209	0.339	0.0171	0.0508	0.2119	-0.109	-0.026	-0.029	0.4031	0.1037	0.5581	-0.28	-0.62	-0.15	-0.47	-0.44	-0.02	0.409	0.097	0.161	0.249733	0.133673	-0.142586901	0.276259855
380	A6NJX0	ST6GALN/	ST6 N-acetylgalactosaminide alpha-2,6-sial	27090	ENSG00000136840	4	-0.12	0.656	-0.51	-0.26	-0.43	0.358	-0.59	-0.36	-0.42	-0.097	0.1049	0.3624	-0.387	0.5727	-0.273	0.0617	-0.176	0.0866	-0.18	-0.24	-0.38	0.158	-0.04	-0.31	-0.05	-0.2	-0.33	0.446504	-0.0136	-0.215635493	0.202030973
2967	Q8TBC4	UBA3	ubiquitin like modifier activating enzyme 3	9039	ENSG00000144744	9	0.257	0.234	0.063	0.057	1E-04	-0.54	0.181	0.056	0.201	0.3049	0.1519	0.0961	-0.063	-0.272	-0.049	0.2024	0.1304	0.2655	-0.14	-0.81	-0.22	-0.41	-0.48	-0.01	0.036	0.105	0.129	0.173051	0.255873	-0.028475601	0.284348523
3303	Q9BXS4	TMEM59	transmembrane protein 59	9528	ENSG00000116209	6	-0.35	0.216	-0	-0.31	-0.24	-0.28	-0.19	0.61	-0.24	-0.044	-0.056	-0.026	-0.145	0.0707	-0.122	-0.085	-0.031	0.1083	-0.31	0.1	0.055	-0.46	-0.07	-0.06	0.468	-0.03	0.148	0.884479	-0.06892	-0.049820925	-0.019094105
36	A0A087WUI	HNRNPA2	heterogeneous nuclear ribonucleoprotein A2	3181	ENSG00000122566	4	0.024	0.044	-0.32	0.287	0.401	-0.16	-0.11	0.138	0.068	-0.165	-0.116	-0.31	0.0509	-0.304	-0.134	0.0083	-0.073	-0.123	-0.23	-0.61	0.283	0.245	-0.19	0.166	-0.04	-0.13	0.035	0.42741	0.093207	0.170647935	-0.077441405
189	E7ENN3	SYNE1	spectrin repeat containing nuclear envelope	23345	ENSG00000131018	2	0.449	-1.36	-0.44	-0.15	-0.74	0.625	0.087	-0.57	0.057	-0.045	0.3656	0.7255	-0.96	-0.186	-0.506	-0.395	-1.264	-0.665	-0.22	-0.48	0.331	2.167	0.15	0.439	-0.09	-0.39	-1.49	0.686047	-0.27267	0.099205267	-0.371875008
1883	P37108	SRP14	signal recognition particle 14	6727	ENSG00000140319	3	0.07	0.048	-0.15	0.275	0.297	-0.12	0.207	0.296	0.291	-0.172	-0.202	-0.085	-0.045	0.0674	-0.125	0.2419	0.1371	0.0949	-0.36	-0.94	-0.32	-0.13	-0.19	-0.02	0.106	0.261	0.34	0.246599	0.273268	0.144795026	0.128472591
963	K7ENA8	EIF3G	eukaryotic translation initiation factor 3 subu	8666	ENSG00000130811	4	0.378	0.487	0.126	-0.2	0.358	-0.35	0.125	0.088	0.213	0.1888	0.1806	0.1367	0.1232	-0.254	0.0362	0.0682	0.4032	0.1151	0.069	-0.78	0.091	-0.5	-0.05	0.06	-0	0.261	0.214	0.389359	0.20718	0.024975491	0.182204992
3035	Q92567	FAM168A	family with sequence similarity 168 member	23201	ENSG00000054965	6	-0.41	0.145	-0.57	-0.19	-0.19	0.058	0.054	0.319	-0.09	-0.137	-0.031	-0.162	-0.027	-0.028	0.1461	0.4406	0.0939	0.5306	-0.11	-0.21	-0.28	-0	-0.33	-0.16	-0.04	-0.08	0.509	0.405153	-0.0192	-0.188829124	0.169624774
1918	P42338	PIK3CB	phosphatidylinositol-4,5-bisphosphate 3-kina	5291	ENSG00000051382	12	0.318	0.247	0.067	-0.24	-0.31	-0.43	-0.06	0.027	0.361	0.2911	0.113	0.1774	-0.027	-0.107	-0.085	0.2388	0.1662	0.3012	-0.03	-0.33	0.222	-0.27	-0.25	0.029	0.171	0.143	0.256	0.574418	0.003135	-0.121517569	0.124652845
129	A0A096LNH	DOCK1	dedicator of cytokinesis 1	1793	ENSG00000150760	8	-0.01	0.051	-0.13				-0.28	-0.11	0.023	-0.248	0.0009	-0.1				-0.097	0.0355	-0.055	-0.29	-0.64	0.22				0.047	-0.1	0.144	0.976998	0.027307	0.001249233	0.026057504
2128	P59861	DEFB131A	defensin beta 131A	644414	ENSG00000186146	3	0.212	-0.3	-0.13	0.062	-0.09	-0.09	0.067	0.353	0.195	0.1858	0.1581	0.1001	-0.354	0.0623	0.3056	0.3414	-0.635	-0.348	0.488	0.61	-0.08	-0.13	-0.12	-0.5	0.506	-0.17	-0.28	0.945528	-0.00524	0.051190667	-0.056432997
232	A0A0G2JNJ	MUC6	mucin 6, oligomeric mucus/gel-forming	4588	ENSG00000184956	70	-1.22	-2.05	0.49	-0.11	-0.32	-0.62	0.666	0.025	0.425	-1.124	1.0384	-1.105	-0.365	-0.481	-0.663	-0.793	-0.481	0.0361	-1.94	-1.21	-0.03	-0.47	-0.63	-0.62	0.609	-0.52	-0.1	0.86582	0.243268	0.13561232	0.107656059
2601	Q58FG1	HSP90AA4	heat shock protein 90 alpha family class A n	3323	ENSG00000205100	3	0.296	-0	0.049				-0.85	-0.68	-0.6	0.0716	0.2887	0.1011				-0.857	-0.132	-0.786	-0.13	-0.46	-0.24				-0.35	-0.34	-0.79	0.857388	0.084931	-0.081249494	0.16618027
3170	Q96NT5	SLC46A1	solute carrier family 46 member 1	113235	ENSG00000076351	3	-0.28	-0.08	-0.04	0.286	0.554	0.131	0.011	0.231	0.212	0.1783	0.0181	0.1557	-0.035	-0.193	-0.052	0.192	0.0838	0.2677	-0.28	-0.17	-0.61	-0.08	-0.09	-0.15	-0.03	0.201	0.073	0.183599	0.238661	0.044816935	0.193843865
1055	O14907	TAX1BP3	Tax1 binding protein 3	30851	ENSG00000213977	3				0.013	0.139	-0.05	0.119	-0.3	-0.16				0.2722	-0.17	0.2173	0.8632	-0.285	0.1967				-0.34	-0.11	0.237	0.324	0.02	-0.23	0.532491	-0.02347	-0.22105365	0.197583096
3421	Q9H9T3	ELP3	elongator acetyltransferase complex subunit	55140	ENSG00000134014	7							-0.08	-0.14	0.055							0.1205	0.0173	0.1858							0.078	0.097	0.171	0.205422	-0.17036	-0.162835992	-0.007522413
843	H3BM14	NUB1	negative regulator of ubiquitin like proteins 1	51667	ENSG00000013374	6	-0.44	-0.48	-0.64	0.09	-0.44	-0.18	-0.24	-0.17	-0.1	-0.264	-0.176	-0.193	-0.227	0.1253	0.2619	-0.347	0.1392	-0.209	-0.37	-0.15	-0.17	-0.54	-0.47	-0.16	-0.49	-0.24	-0.1	0.225769	0.007168	-0.191006695	0.198174275
3056	Q92890	UFD1	ubiquitin recognition factor in ER associated	7353	ENSG00000070010	5	0.197	0.129	0.107	0.025	0.237	-0.41	0.091	0.132	0.225	0.0281	-0.064	0.0562	-0.036	-0.262	-0.153	0.111	0.2607	0.1629	-0.1	-0.68	-0.13	-0.53	-0.17	0.04	0.201	0.229	0.174	0.402955	0.189841	0.070139311	0.119701662
3493	Q9NRY4	ARHGAP3	Rho GTPase activating protein 35	2909	ENSG00000160007	10	-0.13	0.053	-0	-0.26	-0.45	-0.29	0.338	0.381	0.72	-0.043	-0.064	0.0853	0.0311	0.3657	-0.074	0.5014	0.0243	0.3867	-0.4	-0.51	-0.37	-0.54	-0.15	-0.08	0.213	0.414	0.336	0.432098	0.15934	-0.09550307	0.2548427
3139	Q96H79	ZC3HAV1L	zinc finger CCCH-type containing, antiviral 1	92092	ENSG00000146858	6	-0.29	-0.03	-0.15	0.411	0.228	-0.18	-0.91	-0.55	-0.23	-0.095	-0.125	0.108	-0.183	-0.052	-0.166	-0.54	-0.367	-0.631	-0.54	-0.5	-0.35	-0.58	-0.54	-0.02	-0.4	-0.38	-0.58	0.324671	0.242648	0.037505275	0.205142987
940	K4DI93	CUL4B	cullin 4B	8450	ENSG00000158290	10	-0.04	-0.52	-0.41	-1.02	-0.64	0.036	-0.16	0.07	-0.01	0.0367	-0.144	-0.328	-0.906	0.5915	-0.034	-0.044	-0.339	-0.373	0.52	0.655	0.478	-0.36	0.263	-0.17	0.242	-0.13	0.024	0.156947	-0.46934	-0.129013729	-0.340331072
2890	Q8N1B4	VPS52	VPS52 subunit of GARP complex;VPS52, C	6293	ENSG00000223618	5	0.486	0.452	0.34	-0.08	0.087	-0.51	-0.23	0.027	0.013	0.6079	0.5332	0.1012	0.3006	-0.309	0.0485	0.1305	0.1786	0.0846	-0.16	-1.08	0.096	-0.12	-0.39	0.114	0.142	-0.15	0.085	0.236091	0.225662	-0.121516601	0.34717825

1186	O60927	PPP1R11	protein phosphatase 1 regulatory inhibitor su	6992	ENSG00000236560	2	0.323	0.176	0.085				0.468	0.387	-0.01	0.1969	-0.16	-0.108				0.2611	0.2729	-0.189	-0.03	-0.85	-0.19				0.534	0.087	-0.08	0.369697	0.326677	0.193461403	0.133215723
3485	Q9NRQ2	PLSCR4	phospholipid scramblase 4	57088	ENSG00000114698	6	-0.15	-0.23	-0.12	-0.31	-0.25	-0.23	-0.19	0.286	-0.26	-0.082	-0.036	-0.009	0.2638	0.0462	-0.002	0.4399	0.1318	0.061	-0.26	-0.34	-0.18	0.017	-0.28	-0.31	0.002	0.277	0.411	0.17966	-0.08699	-0.251949025	0.164959356
3183	Q96QR8	PURB	purine rich element binding protein B	5814	ENSG00000146676	7	0.109	-0.1	-0.15	0.051	0.062	-0.31	0.054	0.221	0.32	-0.082	0.0068	0.0924	0.0448	-0.032	0.1272	0.2572	-0.103	-0.006	-0.02	-0.65	0.051	-0.56	-0.02	-0.05	0.352	0.071	0.347	0.78658	0.080329	-0.006176974	0.086505499
3472	Q9NR09	BIRC6	baculoviral IAP repeat containing 6	57448	ENSG00000115760	15	0.148	-0.02	0.145	-0.17	-0.1	-0.38	-0.12	-0.19	-0.03	0.0315	0.1054	0.0929	0.0574	-0.161	-0.158	0.0298	0.1564	0.0718	-0.13	-0.32	0.004	-0.27	-0.25	-0.12	0.278	0.027	0.01	0.427799	0.005513	-0.10529006	0.110803256
2388	Q13214	SEMA3B	semaphorin 3B	7869	ENSG00000012171	16	-0.24	-0.72	-0.39	-0.52	0.112	-0.36	-0.39	0.282	-0.08	0.1901	-0.184	-0.097	-0.288	0.2658	0.178	0.1519	-0.554	-0.027	0.355	1.333	0.246	0.062	-0.18	-0.03	0.753	-0.1	-0.29	0.155366	-0.49502	-0.215862266	-0.279160836
3169	Q96NT3	GUCD1	guanylyl cyclase domain containing 1	83606	ENSG00000138867	6	0.333	0.503	-0.24	-0.01	-0.21	0.136	-0.27	0.069	-0.01	-0.007	0.4709	0.4784	-0.149	0.0594	0.1636	0.3266	-0.104	0.3351	-0.38	-0.4	-0.23	-0.34	-0.07	-0.22	-0.34	0.223	0.536	0.211521	0.168023	-0.142317639	0.310340165
285	A0A1C7CYZ	MAPK15	mitogen-activated protein kinase 15	225689	ENSG00000274205	1	0.393	0.179	-0.16	0.06	-0.11	-0.5	-0.02	-0.25	0.133	0.2037	-0.085	0.0022	0.1748	-0.147	0.1123	0.026	0.1692	0.1827	-0.25	-1.69	-0.62	-0.62	-0.54	0.165	-0.16	-0.06	0.282	0.163869	0.355156	-0.102120105	0.457276549
1542	Q96II5	ARAF	A-Raf proto-oncogene, serine/threonine kina	369	ENSG00000078061	10	0.069	0.147	-0.14	0.029	0.242	-0.45	-0	0.33	0.179	0.0584	-0.047	0.1894	0.266	-0.178	0.1235	0.1125	0.1943	0.0992	-0.28	-0.84	-0.42	-0.15	-0.17	0.209	0.02	0.031	0.215	0.241211	0.199595	-0.046289583	0.245884711
2381	Q13153	PAK1	p21 (RAC1) activated kinase 1	5058	ENSG00000149269	8	-0.02	0.263	-0.47	0.134	0.132	-0.2	-0.39	-0.2	0.001	-0.082	0.1161	0.2221	0.3382	0.0023	0.1518	0.076	0.1376	-0.12	-0.41	-1.12	-0.57	-0.64	-0.14	0.065	-0.09	-0.14	0.019	0.103282	0.251846	-0.176977495	0.428823967
2946	Q8NFH8	REPS2	RALBP1 associated Eps domain containing	9185	ENSG00000169891	6	0.133	0.267	-0.05	-0.12	0.059	-0.35	-0.31	0.06	0.173	0.015	-0.12	0.1303	0.0327	-0.222	0.0377	0.0579	-0.107	0.0394	0.084	-0.37	-0.21	-0.37	-0.2	0.192	0.248	-0.03	0.087	0.885102	0.048763	3.60612E-05	0.048727045
3314	Q9BY89	KIAA1671	KIAA1671	85379	ENSG00000197077	7	0.161	0.779	-0.01	0.368	0.459	0.17	0.135	0.064	0.423	-0.021	-0.623	-0.243	0.0086	-0.086	-0.626	0.5554	-0.237	0.2572	-0.45	-0.49	0.182	-0.36	-0.06	0.125	0.329	-0.12	0.546	0.173097	0.315803	0.395257119	-0.079453902
2254	P78318	IGBP1	immunoglobulin binding protein 1	3476	ENSG00000089289	6	0.135	0.322	0.039	0.103	0.46	-0.34	-0.06	0.174	0.135	0.0503	0.0432	0.108	0.3458	-0.047	0.0025	0.0688	0.3329	0.2036	-0.2	-0.84	-0.29	-0.06	-0.23	0.319	0.058	-0.03	0.042	0.181575	0.244389	-0.015079805	0.259468552
1042	O14733	MAP2K7	mitogen-activated protein kinase kinase 7	5609	ENSG00000076984	2	0.033	0.468	-0.07	-0.05	-0.16	-0.4	-0.19	-0.07	0.035	0.2629	0.1687	0.2294	0.0167	-0.193	-0.008	0.0832	0.0212	0.1351	-0.22	-1.15	-0.42	-0.56	-0.32	0.114	0.074	-0.11	0.082	0.153014	0.234396	-0.124290777	0.3586868
2945	Q8NFA0	USP32	ubiquitin specific peptidase 32	84669	ENSG00000170832	12	0.11	0.14	-0.08	0.066	-0.03	-0.12	0.213	0.335	0.264	0.2189	0.1357	0.0229	0.0307	-0.159	0.1571	0.2757	0.3162	0.3247	-0.16	-0.48	-0.11	-0.44	-0.02	0.025	0.089	0.084	0.316	0.183891	0.177041	-0.047340359	0.224381807
1020	O00534	VWA5A	von Willebrand factor A domain containing 5	4013	ENSG00000110002	6	-0.25	0.484	0.242	-0.13	0.289	-0.51	0.007	0.042	0.056	-0.297	-0.151	-0.158	-0.279	-0.431	-0.166	0.1681	0.2802	0.14	0.336	0.247	-0.32	0.352	-0.04	0.075	-0.1	-0.08	0.198	0.503844	-0.04885	0.125089402	-0.173939493
647	Q5TG12	PTPRK	protein tyrosine phosphatase, receptor type	5796	ENSG00000152894	5				-0.07	-0.06	-0.2	0.027	0.608	0.015				-0.379	0.1982	-0.243	0.2854	-0.141	-0.216				0.336	-0.07	0.125	0.615	0.393	-0.08	0.346368	-0.16994	0.134080203	-0.30402073
1724	P23142	FBLN1	fibulin 1	2192	ENSG00000077942	9	-0.21	-0.33	-0.01	-0.08	-0.19	-0.32	-0.14	0.336	0.12	0.1255	-0.019	0.34	-0.14	-0.009	-0.289	0.2084	0.0802	-0.1	-0.07	0.055	0.56	0.342	0.32	0.033	0.011	0.071	-0.28	0.306481	-0.20796	-0.112940318	-0.095022736
2804	Q86TP1	PRUNE1	prune exopolyphosphatase 1	58497	ENSG00000143363	5	0.107	-0.07	-0.11	0.013	1.195	-0.55	-0.02	0.092	0.026	0.2474	0.146	-0.009	0.2016	-0.383	0.0004	0.0522	-0.062	0.0162	0.013	-0.52	-0.22	-0.18	0.184	0.39	0.016	0.046	0.222	0.910561	0.082411	0.053725229	0.028686069
323	A0A2R8Y6S	TSC1	TSC complex subunit 1	7248	ENSG00000165699	5	-0.05	-0.67	-0.35	-0.23	-0.17	-0.31	-0.35	-0.41	-0	0.1563	0.0051	-0.309	-0.186	-0.127	-0.051	0.0827	-0.142	-0.313	0.423	0.591	0.099	-0.49	-0.03	-0.06	0.077	-0.06	-0.17	0.136784	-0.32517	-0.184477823	-0.140690227
3400	Q9H6Q4	CIAO3	cytosolic iron-sulfur assembly component 3	64428	ENSG00000103245	5	-0.11	0.519	-0.21	0.086	-0.1	0.033	0.222	0.5	0.338	0.2239	-0.204	0.1959	0.3021	-0.023	0.1993	0.4434	0.008	0.3293	-0.49	-0.74	-0.52	-0.39	-0.09	0.17	0.456	0.229	0.397	0.309501	0.249685	-0.022646497	0.272331062
72	F6UYG0	WNK1	WNK lysine deficient protein kinase 1	65125	ENSG00000060237	2	-0.06	-0.22	-0.12	-0.12	-0.07	-0.26	-0.02	-0.03	0.013	0.1257	0.1347	-0.047	0.65	-0.05	-0.094	-0.019	0.0446	0.0938	0.038	-0.61	-0.36	-0.32	-0.07	-0.1	-0.07	-0.1	0.043	0.119347	0.074443	-0.191736669	0.266179475
942	K7EII4	USP32	ubiquitin specific peptidase 32	84669	ENSG00000170832	2	0.295	0.052	-0.07				0.435	0.237	0.322	0.0866	0.2625	0.0415				0.118	-0.187	-0.055	-0.12	-0.56	-0.02				0.352	-0.07	0.451	0.501231	0.205998	0.167022362	0.038975941
1426	P04844	RPN2	ribophorin II	6185	ENSG00000118705	3							0.029	0.162	0.07							0.1772	-0.108	0.1655							0.333	0.08	0.097	0.759328	-0.08276	0.008892082	-0.091650106
3349	Q9H0Q0	FAM49A	family with sequence similarity 49 member A	81553	ENSG00000197872	7	0.243	0.078	-0.07	-0.05	-0.06	-0.26	0.042	0.043	0.076	0.2348	0.3176	0.154	0.0331	-0.081	-1E-03	0.3563	0.142	0.007	-0.02	-0.25	-0.32	-0.37	-0.1	-0.03	0.145	0.111	0.3	0.225727	0.06287	-0.125756909	0.188626973
3523	Q9NVZ3	NECAP2	NECAP endocytosis associated 2	55707	ENSG00000157191	6				-0.1	-0.15	-0.32	0.286	-0.07	0.407				-0.122	-0.141	0.1907	0.242	0.277	0.4586				-0.48	-0.3	0.006	0.012	0.124	0.328	0.575865	0.059602	-0.141556042	0.201157916
1429	P05060	CHGB	chromogranin B	1114	ENSG00000089199	14	0.228	0.005	-0.14	-0.69	-0.5	0.005	-0.28	0.116	-0.04	0.3764	0.7361	-0.202	-0.528	1.1458	-0.362	-0.493	-0.429	0.4309	0.301	0.478	-0.14	-0.98	-0.44	-0.63	-0.09	-0.03	0.292	0.673633	-0.00973	-0.220161248	0.210428799
2572	Q30KQ8	DEFB112	defensin beta 112	245915	ENSG00000180872	2				0.792	-0.21	0.131	0.145	0.651	0.199				0.6081	-0.689	0.191	0.0713	-0.089	-0.617				-0.38	-0.54	-0.96	-0.29	-0.13	-1.05	0.101522	0.842935	0.372013873	0.47092154
3541	Q9NYU2	UGGT1	UDP-glucose glycoprotein glucosyltransfera	56886	ENSG00000136731	12	0.01	-0.15	-0.03	-0.08	-0.07	-0.38	-0.04	0.238	-0.19	0.0628	0.0352	0.0239	-0.257	0.0843	-0.129	0.2202	-0.085	0.0052	-0.03	0.34	-0.05	-0.2	-0.25	-0.18	0.407	-0.02	-0.28	0.793006	-0.04798	-0.074237483	0.026254391
2634	Q5T2E6	ARMH3	armadillo like helical domain containing 3;arr	79591	ENSG00000120029	5	-0.24	-0.16	-0.53	0.141	0.069	-0.15	0.132	-0	0.132	-0.328	-0.187	-0.502	0.367	-0.02	0.1649	0.1651	0.1698	0.0419	-0.48	-0.91	1.001	-0.31	0.083	-0.17	-0.06	-0.08	0.336	0.96114	-0.00243	-0.053396101	0.05096323
3390	Q9H4A5	GOLPH3L	golgi phosphoprotein 3 like	55204	ENSG00000143457	4	0.093	0.34	0.139	0.123	-0.13	-0.21	-0.18	0.13	0.023	-0.038	0.2487	0.0721	0.0038	-0.038	0.3064	-0.004	0.1213	-0.067	-0.17	-0.7	-0.17	-0.79	-0.39	-0.05	-0.04	-0	0.044	0.095938	0.287929	-0.031132849	0.319061478
2695	Q8TCE6	DENND10	DENN domain containing 10;family with seq	404636	ENSG00000119979	5	0.042	-0.16	-0.05	-0.06	-0.52	-0.5	-0.11	-0.02	0.092	-0.166	0.2475	0.0327	0.1734	0.0191	0.0055	0.0771	0.3541	0.3687	0.064	-0.17	-0.12	-0.55	-0.44	-0.09	-0.11	-0.03	0.096	0.112196	0.008271	-0.265731648	0.27400229
665	E9PS17	SCYL1	SCY1 like pseudokinase 1	57410	ENSG00000142186	8	0.103	0.208	-0	0.013	0.022	-0.39	0.419	0.335	0.487	-0.007	0.0195	0.0326	0.0182	-0.197	-0.077	0.3941	-0.007	0.4674	-0.23	-0.56	0.007	0.308	-0.27	0.034	0.231	0.162	0.511	0.787873	0.111298	0.060909905	0.050387858
500	E9PFH4	TNPO3	transportin 3	23534	ENSG00000064419	8				-0.1	-0.17	-0.2	-0.35	-0.33	0.172				0.0009	-0.13	-0.043	0.3922	0.5558	0.4957				-0.14	-0.13	0.099	-0.13	0.427	0.379	0.199511	-0.24504	-0.373047009	0.128009423
2850	Q8IWB7	WDFY1	WD repeat and FYVE domain containing 1	57590	ENSG00000085449	12	0.135	0.019	-0.05	-0.1	-0.26	-0.26	-0.78	-0.23	-0.62	0.1995	0.0764	0.0467	-0.069	0.3037	0.0112	-0.301	-0.769	-0.613	0.077	-0.32	0.155	-0.5	-0.03	0.091	-0.29	-0.17	-0.5	0.808779	-0.07483	-0.115509456	0.040676538
1907	P41221	WNT5A	Wnt family member 5A	7474	ENSG00000114251	12	-0.09	-0.58	0.088	-0.41	0.076	-0.04	0.275	0.705	0.174	0.0441	0.0615	-0.364	-0.428	0.2071	0.0166	0.1827	0.2962	0.3674	-0.07	0.177	0.322	-0.26	-0.08	0.035	0.927	0.006	0.054	0.866287	-0.1007	-0.020311525	-0.080389825
1502	P08311	CTSG	cathepsin G	1511	ENSG00000100448	8	-1.73	-1.53	-1.66	-0.98	-1.15	0.523	-1.06	-1.2	-1.16	-2.234	-0.316	-1.228	-0.811	-0.879	-1.022	-0.728	-1.805	-1.53	-1.03	-1.25	2.497	2.769	-0.63	1.103	-0.51	-1	-1.38	0.155534	-1.16876	0.067491651	-1.236251016
3563	Q9P253	VPS18	VPS18 core subunit of CORVET and HOPS	57617	ENSG00000104142	5	0.024	0.284	-0.04	0.29	0.336	-0.25	0.056	-0.07	0.181	-0.027	-0.129	-0.018	0.5471	0.1981	0.2703	0.1904	-0.101	0.0393	-0.04	-0.35	-0.12	0.298	0.013	0.154	0.283	0.073	0.044	0.844407	0.049882	-0.018080328	0.067962157
498	C9J715	NISCH	nischarin	11188	ENSG00000010322	2	-0.43	-0.13	-0.43	0.063	-0.11	-0.27	-0.3	-0.17	-0.16	-0.449	-0.324	-0.351	0.1873	-0.019	0.1112	-0.245	-0.243	-0.367	-0.57	-1.5	2.017	-0.42	-0.31	0.072	0.229	-0.1	0.613	0.777213	-0.22007	-0.027029047	-0.193038895
28	A0A087WT/	EIF2B4	eukaryotic translation initiation factor 2B sub	8890	ENSG00000115211	8	0.063	0.077	-0.24	-0	-0.05	-0.34	0.021	-0.07	0.105	0.1542	-0.16	0.0638	0.2351	-0.341	-0.287	0.062	0.0429	0.1316	-0.14	-0.49	-0.17	-0.01	-0.3	0.053	0.121	-0	-0.02	0.672005	0.057235	-0.037647481	0.094882609
240	A0A0J9YWL	CRYBG1	crystallin beta-gamma domain containing 1	202	ENSG00000112297	2							0.191	0.401	0.521							0.6869	-0.059	0.572							0.624	0.512	0.535	0.753097	-0.18583	-0.028963874	-0.156866948
3288	Q9BVC4	MLST8	MTOR associated protein, LST8 homolog	64223	ENSG00000167965	6	-0.15	-0.12	-0.27	-0.4	0.258	-0.43	-0.19	-0.21	0.039	-0.136	-0.241	-0.046	-0.043	0.0263	-0.209	-0.015	0.1189	0.1539	-0.12	-0.51	-0.19	0.231	-0.21	0.321	0.131	-0.18	0.013	0.561564	-0.1073	-0.121034942	0.013739607
66	P54252	ATXN3	ataxin 3	4287	ENSG00000066427	6	0.168	-0.04	-0.09	-0.13	-0.12	-0.7	0.01	0.023	0.509	0.0448	0.1321	0.2539	0.0945	0.0179	0.2775	0.8285	0.1081	0.3147	-0.06	-0.67	-0.29	-0.55	-0.48	0.097	0.238	0.596	0.364	0.282771	0.041146	-0.271856465	0.313002031
3455	Q9NPF0	CD320	CD320 molecule	51293	ENSG00000167775	4	-0.41	0.358	0.386	0.011	-0.31	0.07	0.139	0.177	-0.14	-0.046	0.149	-0.194	-0.213	0.3841	-0.261	0.2152	0.2023	0.2952	-0.14	-0.46	-0.47	0.318	0.201	0.148	-0.11	-0.15	0.336	0.834041	0.066509	-0.029006921	0.09551565
538	C9JYN0	SYPL1	synaptophysin like 1	6856	ENSG00000008282	3	-0.1	0.033	-0.1	0.532	0.409	-0.34	-0.14	0.035	0.268	-0.196	-0.187	-0.172	0.1811	-0.44	-0.069	-0.295	-0.179	0.0356	-0.38	-0.83	-0.42	-0.41	-0.35	-0.18	0.034	-0.13	0.054	0.124987	0.355392	0.212686041	0.142706095
973	M0QXB0	LSM4	LSM4 homolog, U6 small nuclear RNA and	25804	ENSG00000130520	2	0.063	0.075	-0.02	0.044	0.188	-0.36	0.043	0.172	0.11	-0.152	-0.083	-0.029	0.0011	0.0746	0.0047	0.4264	-0.255	0.1276	-0.07	-0.44	-0.05	-0.04	-0.32	0.058	0.179	0.202	-0.15	0.612761	0.105554	0.02294931	0.082604569
667	H7BXH2	PPP6R3	protein phosphatase 6 regulatory subunit 3	55291	ENSG00000110075	11	0.833	-0.45	0.501	-0.03	0.041	-0.39	0.32	0.199	0.223	0.1633	-0.176	0.3074	0.0175	-0.021	0.0257	0.2253	0.122	0.4915	0.174	-0.39	-0.17	-0.23	-0.31	0.013	0.314	-0.17	0.244	0.467342	0.196113	0.01018402	0.185929362
3760	Q9Y4X5	ARIH1	ariadne RBR E3 ubiquitin protein ligase 1	25820	ENSG00000166233	7	-0.07	0.016	0.054	-0.03	0.261	0.054	-0.09	-0.07	0.096	0.1007	-0.026	0.0311	0.0266	-0.051	-0.048	-0.104	0.2303	0.0461	0.016	-0.29	-0.14	-0.28	0.318	0.032	-0.1	-0.06	0.046	0.566519	0.075931	0.00176151	0.074169852
3373	Q9H2P9	DPH5	diphthamide biosynthesis 5	51611	ENSG00000117543	2	-0.01	0.275	-2.79	0.369	-0.59	0.455	0.164	0.79	-0.1	-5E-04	-0.354	0.0428	-0.065	0.0547	0.7027	0.8427	0.2799	0.247	-0.09	-1.47	-1.29	-0.77	0.339	-0.06	-0.82	-0.28	0.578	0.401508	0.268436	-0.353498068	0.621934151
2463	Q14515	SPARCL1	SPARC like 1	8404	ENSG00000152583	8	-0.46	-0.58	-0.77	-0.03	-0.16	0.054	0.21	0.123	-0.19	-0.242	-0.438	-0.141	-0.613	0.7549	-0.183	-0.002	-0.313	-0.393	-0.69	-0.06	0.118	-0.62	-0.32	-0.38	0.327	-0.08	-0.52	0.940659	0.045903	-0.025637377	0.071540825
275	A0A1B0GUZ	EFR3A	EFR3 homolog A	23167	ENSG00000132294	10	0.035	0.248	-0.44	0.117	0.283	-0.24	-0.25	0.055	0.034	-0.294	-0.279	0.1325	0.0194	-0.162	0.2471	0.2816	-0.181	0.0293	-0.35	-0.29	-0.4	-0.31	-0.36	-0.23	0.317	0.15	0.015	0.512153	0.146328	0.006251875	0.140076143
2507	Q15208	STK38	serine/threonine kinase 38	11329	ENSG00000112079	6	-0.04	0.194	-0.03	0.022	-0.06	-0.22	-0.07	-0.06	0.076	0.1031	0.063	0.1647	-0.079	-0.304	-0.032	0.0222	0.2309	-0.058	-0.2	-0.29	0.165	-0.48	-0.37	0.203	-0.04	-0.18	0.141	0.465311	0.096309	-0.033031864	0.129340787
3727	Q9Y2V7	COG6	component of oligomeric golgi complex 6	57511	ENSG00000133103	3	-0.02	0.165	-0.14				-0.03	-0.11	0.217	-0.202	-0.319	-0.088				0.0903	0.0348	0.1626	-0.15	-1	-0.4				0.02	0.137	0.25	0.625515	0.204595	0.066678237	0.137916379
2993	Q8TEB1	DCAF11	DDB1 and CUL4 associated factor 11	80344	ENSG00000100897	6	-0.17	0.025	-0.18				-0.03	0.392	0.151	0.2842	-0.123	-0.006				-0.096	-0.04	-0.229	0.06	0.12	-0.01				0.232	-0.02	0.16	0.596197	-0.05941	0.066284574	-0.125697972
1368	P0DP02	IGHV3-33;	immunoglobulin heavy variable 3-33;immunc	57290	ENSG00000282642	4	-1.59	-1.48	-0.99	-1.61	-1.5	-1.42	-1.99	-1.61	-1.9	-1.788	-0.879	-0.733	-1.492	-1.384	-1.756	-1.321	-1.207	-1.82	-1.12	-0.8	2.66	2.818	-1.23	1.414	-0.71	-1.07	-2.49	0.121066	-1.50679	-0.190264523	-1.316525843
3393	Q9H4D0	CLSTN2	calsyntenin 2	64084	ENSG00000158258	7	-0.41	-0.14	0.114	-0.33	-0.07	-0.08	-0.45	-0.41	-0.72	0.5319	0.1603	-0.139	-0.44	-0.238	-0.291	0.1079	0.0638	-0.429	0.009	0.13	-0.02	-0.01	-0.15	0.563	-0.43	-0.26	-0.69	0.487545	-0.18248	-0.202723471	0.020243943
1211	O75462	CRLF1	cytokine receptor like factor 1	9244	ENSG00000006016	3							-0.25	0.524	-0.07							0.7033	-0.235	-0.423							-0.35	0.18	-0.57	0.791059	0.313127	0.052528103	0.260599201
2853	Q8IWV7	UBR1	ubiquitin protein ligase E3 component n-reco	197131	ENSG00000159459	12	0.095	-0.01	0.042	-0.02	0.007	-0.25	-0.15	0.23	0.033	0.011	0.1087	0.2775	0.1531	-0.4	-0.092	0.2389	0.0446	0.0182	-0.14	-0.38	0.177	0.152	-0.18	0.117	0.257	0.205	-0.03	0.926614	-0.0212	-0.042408892	0.02120658
2091	P54619	PRKAG1	protein kinase AMP-activated non-catalytic s	5571	ENSG00000181929	10	0.168	0.303	0.011	0.029	0.043	-0.25	-0.35	-0.22	0.056	0.3685	0.2133	0.2986	0.0627	-0.039	0.0295	-0.021	0.1608	0.094	-0	-0.51	-0.04	-0.3	-0.22	0.067	-0.11	-0.1	0.087	0.132916	0.099735	-0.152751774	0.252486428
2098	P54826	GAS1	growth arrest specific 1	2619	ENSG00000180447	5	-0.22	0.247	0.199	-0.23	-0.12	-0.53	0.124	0.2	-0.05	0.4541	-0.088	0.8317	0.1473	0.0337	-0.113	0.205	-0.53	0.0637	-0.18	-0.9	-0.62	-0.52	-0.46	-0.09	0.397	0.137	0.444	0.396436	0.158149	-0.153370644	0.311519353
99	A0A087X0Z7	DHRS7	dehydrogenase/reductase 7	51635	ENSG00000100612	6	-0.15	0.696	-0.3	-0.09	-0.39	-0.43	-0.17	0.254	0.244	0.6164	-0.392	0.2736	-0.095	0.1754	0.1075	-0.291	-0.434	-0.342	-0.1	0.422	0.588	-0.22	0.076	0.004	0.671	0.177	-0.03	0.487458	-0.21407	0.004043171	-0.218108823
3188	Q96RR4	CAMKK2	calcium/calmodulin dependent protein kinas	10645	ENSG00000110931	6	0.324	0.207	-0.04	-0.08	-0.06	-0.29	-0.29	-0.1	0.572	0.1004	0.1208	0.3885	0.0468	-0.14	-0.084	0.275	0.1227	0.0929	-0.17	-1.2	-0.29	-0.32	-0.48	-0.1	0.222	-0.02	0.087	0.178318	0.278418	-0.07550967	0.353927456
127	A0A088AWN	DOCK9	dedicator of cytokinesis 9	23348	ENSG00000088387	7	0.687	0.335	-0.23	0.44	0.532	-0.34	-0.37	-0.18	-0.19	0.0786	0.5339	0.6913	0.043	0.12	0.4599	0.0662	-0.047	0.0439	-0.51	-0.24	-0.35	-0.63	-0.36	-0.05	-0.01	-0.09	0.275	0.139337	0.294191	-0.145639939	0.439830638
2555	Q16539	MAPK14	mitogen-activated protein kinase 14	1432	ENSG00000112062	6	-0.11	-0.04	-0.14	-0	-0.24	-0.54	0.176	-0.1	0.381	-0.193	-0.098	0.1059	-0.039	-0.229	0.1055	0.095	0.2546	-0.029	-0.31	-1.09	-0.12	-0.17	-0.29	0.056	-0.09	0.054	0.285	0.542447	0.116952	-0.066280491	0.183232279
3018	Q8WWA0	ITLN1	intelectin 1	55600	ENSG00000179914	9	-1.32	0.771	0.576	-2.35	-0.51	-2.4	-1.05	-1.53	0.604	0.292	-0.75	1.527	-1.72	1.8463	-1.781	-0.617	-1.277	0.703	-0.53	-0.48	-0.92	-2.48	0.887	-1.63	-0.84	-1.12	-1.62	0.517691	0.168989	-0.604643547	0.773632463
1080	O15211	RGL2	ral guanine nucleotide dissociation stimulatc	5863	ENSG00000237441	5	-0.06	0.091	-0.02	-0.06	-0.15	-0.48	0.077	0.192	0.205	-0.068	0.0884	0.1703	-0.006	-0.121	0.0484	0.205	-9E-04	0.2428	-0.37	-0.09	-0.3	-0.72	-0.32	-0.01	0.234	0.113	0.2	0.347939	0.116006	-0.084880304	0.200886404
3398	Q9H5N1	RABEP2	rabaptin, RAB GTPase binding effector prot	79874	ENSG00000177548	9	-0.08	-0.14	-0.14	-0.03	-0.09	-0.2	0.303	0.097	0.3	0.0199	-0.023	-0.023	0.1229	0.0901	0.103	0.4534	0.1569	0.4947	-0.09	-0.38	0.011	-0.19	-0.09	0.088	0.282	0.115	0.314	0.362594	-0.00298	-0.151950046	0.148968225
2809	Q86UP2	KTN1	kinectin 1	3895	ENSG00000126777	4							-0.2	-0.43	-0.33							-0.136	0.0132	-0.244							0.13	-0.3	-0.37	0.595058	-0.14005	-0.196680008	0.056625613
193	A0A0C4DGL	HP	haptoglobin	3240	ENSG00000257017	6	-0.97	-1.12	-1.25	-0.61	-0.83	0.103	-0.78	0.213	-1.52	-0.633	-0.751	-0.947	-0.39	-0.354	-0.694	-1.041	-1.372	-1.697	-1.25	-0.91	1.47	2.077	0.291	0.377	-0.09	-0.19	-1.52	0.181942	-0.78058	0.12224146	-0.902823442
1296	Q5TIH2	SFT2D2	SFT2 domain containing 2	375035	ENSG00000213064	2				5E-04	-0.15	-0.16	-0.76	-0.69	-0.5				-0.037	-0.446	0.1	-0.273	-0.071	-0.614				-0.79	-0.42	-0.27	-0.67	-0.4	-0.5	0.362187	0.131441	-0.153685147	0.28512638
3185	Q96RF0	SNX18	sorting nexin 18	112574	ENSG00000178996	11	0.04	0.134	-0.36	0.014	0.733	-0.2	-0.23	-0.05	0.022	-0.187	-0.109	0.4781	-0.255	-0.329	-0.031	0.0285	-0.024	0.4225	-0.2	0.489	-0.26	-0.2	-0.2	-0.06	0.017	0.024	0.091	0.958942	0.045064	0.012087343	0.032976229
434	B4DR12	MGRN1	mahogunin ring finger 1	23295	ENSG00000102858	5	-0.24	0.442	-0.13	0.208	-0.04	-0.1	-0.02	0.031	0.026	-0.096	0.1875	0.37	0.3313	-0.201	0.1094	0.3911	0.0923	0.3178	-0.22	-0.15	-0.23	-0.16	-0.14	0.025	-0.01	0.171	-0.01	0.146558	0.097314	-0.148007169	0.245320999
509	C9JD84	LTBP1	latent transforming growth factor beta bindin	4052	ENSG00000049323	3	0.14	-0.26	-0.02	-0.12	0.345	-0.05	-0.22	0.006	0.272	0.0014	-0.132	-0.459	0.3055	-0.124	-0.54	0.0035	-0.327	-0.284	-0.47	-0.38	0.05	-0.29	0.157	0.436	0.661	0.028	0.002	0.450838	-0.0092	0.184350085	-0.193547429
3415	Q9H8Y8	GORASP2	golgi reassembly stacking protein 2	26003	ENSG00000115806	4	0.312	0.141	0.235	-0.08	-0.1	-0.28	0.021	0.243	0.168	0.1493	0.1294	0.1026	-0.189	0.0779	0.1122	-0.145	0.1136	0.2578	-0.23	-0.78	-0.13	-0.53	-0.25	0.127	0.11	-0.08	0.425	0.270156	0.220628	0.004485532	0.216142558
3670	Q9UNA0	ADAMTS5	ADAM metallopeptidase with thrombospond	11096	ENSG00000154736	6	-0.89	-0.32	-0.6	-0.67	0.135	0.21	0.192	-0.15	0.234	-0.39	0.0237	-0.218	-0.384	0.4637	-0.292	0.3619	-0.115	0.6313	0.286	-0.37	-0.24	-0.18	-0.09	-0.02	1.237	0.575	-0.42	0.532369	-0.2925	-0.215773126	-0.076727256
649	E9PGN7	SERPING1	serpin family G member 1	710	ENSG00000149131	6	-0.17	-0.82	-0.11	-0.28	-0.28	-0.14	-0.44	0.02	-0.14	0.1745	0.0024	0.3048	-0.043	0.1075	-0.395	-0.536	-0.798	-0.641	-0.01	-0.43	1.052	0.623	0.045	-0.02	0.234	0.049	-0.36	0.21414	-0.39437	-0.060180528	-0.334189013
781	G3V4K3	VIPAS39	VPS33B interacting protein, apical-basolater	63894	ENSG00000151445	4	0.339	0.518	0.423	-0.15	0.1	-0.52	0.125	0.126	0.26	0.663	0.4677	0.5244	0.3406	-0.265	0.2263	0.2839	-0.001	0.1453	0.069	-0.54	-0.09	-0.37	-0.24	0.246	0.454	0.114	0.304	0.355432	0.142273	-0.128659809	0.27093317
3010	Q8WV41	SNX33	sorting nexin 33	257364	ENSG00000173548	9	0.167	0.351	-0.32	0.501	0.711	-0.41	0.126	0.091	0.09	-0.25	0.0865	0.3716	0.0664	-0.285	-0.105	0.262	0.0144	0.7568	-0.26	0.036	-0.07	-0.08	-0.25	-0.36	0.218	0.331	0.655	0.822466	0.122278	0.043485873	0.078791724
3622	Q9UHY1	NRBP1	nuclear receptor binding protein 1	29959	ENSG00000115216	6	0.198	0.172	0.117	0.066	-0.04	-0.37	-0.26	-0.21	0.149	0.2762	-0.047	0.133	0.0038	-0.445	-0.051	-0.018	-0.013	0.1693	-0.16	-0.86	-0.15	-0.2	-0.19	0.062	-0.04	-0.1	-0.07	0.32375	0.16958	-0.020387625	0.189967852
3269	Q9BU68	PRR15L	proline rich 15 like	79170	ENSG00000167183	2	0.04	0.526	-0.03	-0.21	-0.24	-0.7	0.124	0.02	0.147	0.4153	0.0537	0.0176	-0.073	-0.3	-0.072	0.2438	0.3777	-0.048	-0.1	-1.37	-0.58	-0.98	-0.49	-0.19	-0.11	-0.07	-0.04	0.11806	0.400705	-0.10369427	0.50439944

1023	O00567	NOP56	NOP56 ribonucleoprotein	10528	ENSG00000101361	6	-0.04	-0.08	0.094	-0.73	-0.15	-0.52	-0.14	0.206	0.084	0.1	-0.016	0.2476	-0.145	-0.271	-0.159	0.145	0.28	0.0677	-0.42	0.546	0.113	0.8	-0.19	0.065	0.399	-0.14	-0.02	0.338822	-0.27039	-0.1700163	-0.100374791
117	K7ES69	CNN2	calponin 2	1265	ENSG00000064666	3	-0.12	0.48	-0.03	-0.11	0.106	-0.35	0.688	0.306	0.627	0.067	-0.09	0.0462	0.0831	-0.157	-0.011	0.5126	0.6844	0.6403	-0.04	-0.53	-0.11	-0.21	-0.22	-0.02	0.311	0.431	0.559	0.631335	0.158571	-0.019067082	0.177638028
597	E7EM93	NUDT12	nudix hydrolase 12	83594	ENSG00000112874	4	0.044	-0.09	-0	-0.07	0.218	-0.26	-0.4	-0.2	0.039	0.173	0.0386	0.1253	0.1862	0.0186	0.1797	0.1672	0.0649	0.4193	-0.23	-0.38	0.127	-0.6	0.4	0.129	0.214	0.13	0.046	0.230287	-0.06248	-0.233073165	0.170592156
2336	Q07954	LRP1	LDL receptor related protein 1	4035	ENSG00000123384	6	-0.22	-0.69	-0.26	-0.07	0.261	-0.26	0.167	0.593	0.161	-0.298	-0.181	-0.077	-0.572	-0.094	-0.204	-0.005	0.067	-0.076	-0.22	0.434	-0.17	0.014	-0.22	-0.18	0.596	-0.12	-0.16	0.639082	-0.03151	0.12489769	-0.156403314
1937	P45983	MAPK8	mitogen-activated protein kinase 8	5599	ENSG00000107643	5	0.254	0.381	0.024				-0.77	-0.46	-0.32	-0.049	0.1378	0.1886				-0.402	0.032	-0.224	-0.18	-0.65	-0.29				-0.51	-0.28	-0.05	0.493508	0.176478	-0.096465346	0.272943645
518	C9JI98	TMEM238	transmembrane protein 238	388564	ENSG00000233493	4	0.024	0.743	-0.29	0.319	-0.5	0.327	-0.12	0.089	-0.05	-0.291	0.0807	0.6944	0.0163	-0.054	0.5361	0.0739	0.0306	0.4782	-0.24	0.006	-0.17	0.112	-0.37	-0.03	0.052	0.233	0.226	0.56277	0.080385	-0.11364221	0.194026981
2731	Q6UXI7	VIT	vitrin	5212	ENSG00000205221	1							1.681	0.889	1.279							1.0072	0.6216	1.3118							0.445	0.244	1.236	0.422808	0.641169	0.302753925	0.338414629
472	B8ZZF5	EVA1A	eva-1 homolog A, regulator of programmed (	84141	ENSG00000115363	1	-0.57	-0.37	-0.41				-0.39	-0.06	0.235	-0.159	0.0421	-0.635				-0.109	-0.057	-0.155	0.316	1.137	0.036				0.027	-0.38	-0.56	0.471487	-0.35543	-0.081041895	-0.274391014
3677	Q9UNN5	FAF1	Fas associated factor 1	11124	ENSG00000185104	9	0.053	0.004	-0.19	0.392	0.454	-0.28	0.196	0.402	0.322	-0.075	-0.148	-0.101	-0.01	-0.102	0.017	0.2455	0.1502	0.2929	-0.23	-0.9	-0.36	-0.45	-0.42	-0.07	0.203	0.13	0.249	0.161073	0.354689	0.119678493	0.235010166
101	Q9BRT8	CBWD1	COBW domain containing 1	55871	ENSG00000172785	4	0.047	-0.11	0.18	-0.07	0.101	-0.3	0.156	0.129	0.253	-0.08	-0.211	0.1756	-0.034	-0.138	0.0992	0.1872	0.2858	0.3623	-0.45	-0.9	-0.5	-0.24	-0.06	-0.02	0.048	-0.01	0.136	0.149245	0.264196	-0.02891595	0.293112217
1028	O00629	KPNA4	karyopherin subunit alpha 4	3840	ENSG00000186432	6	0.256	0.487	0.031	-0.08	0.343	-0.07	-0.03	-0.39	-0.11	0.187	0.0715	0.231	0.426	0.0624	0.1773	-0.226	0.3228	-0.348	-0.38	-1.06	-0.23	-0.6	-0.31	0.353	-0.05	-0.09	0.149	0.19962	0.294771	-0.052833444	0.347604642
2596	Q53LP3	SOWAHC	sosondowah ankyrin repeat domain family m	65124	ENSG00000198142	2							-1.14	-0.91	-0.23							1.1641	0.2405	-0.005							-0.29	0.176	-0.44	0.187924	-0.57639	-1.22678852	0.650401517
3197	Q96T76	MMS19	MMS19 homolog, cytosolic iron-sulfur asser	64210	ENSG00000155229	10	0.316	0.445	0.03	-0.04	0.033	0.145	-0.14	1.318	0.058	-0.022	0.0642	0.1066	0.1462	-0.076	0.0545	-0.025	-0.386	-0.118	-0.13	-0.67	-0.21	-0.25	-0.04	-0.03	0.1	0.047	-0.12	0.149888	0.38374	0.268644529	0.115095334
3735	S4R3N1	HSPE1-M(	HSPE1-MOB4 readthrough	100529241	ENSG00000270757	4	0.138	0.162	-0.04	5E-04	0.001	0.046	0.077	-0.04	0.112	0.2571	0.04	0.1062	0.1029	0.2088	0.2552	0.0225	-0.166	-0.071	0.074	0.011	0.132	-0.03	0.06	-0.27	0.373	0.136	0.116	0.916451	-0.01635	-0.033706289	0.017354848
3611	Q9UHD2	TBK1	TANK binding kinase 1	29110	ENSG00000183735	5	-0.01	-0.04	-0.05	-0.07	-0.02	-0.29	-0.43	0.074	0.054	0.2896	-0.121	0.4528	0.0704	0.0172	-0.008	0.0083	-0.229	0.0353	0.004	0.085	0.261	-0.05	-0.15	-0.02	0.097	-0.08	-0.48	0.450568	-0.04998	-0.143513636	0.093538111
378	A6NIT2	PHKA1	phosphorylase kinase regulatory subunit alp	5255	ENSG00000067177	7	0.209	0.293	0.142	-0.28	-0.28	-0.36	0.211	-0.18	0.116	0.1598	0.1638	0.3501	-0.226	-0.301	-0.137	0.2004	-0.197	0.3069	-0.12	-0.7	-0.03	0.554	-0.01	0.315	0.492	-0.06	0.016	0.922902	-0.06663	-0.050189421	-0.016440092
111	A0A087X1P!	COPS7B	COP9 signalosome subunit 7B	64708	ENSG00000144524	4				-0.06	-0.45	-0.33	0.185	0.118	0.012				-0.391	-0.042	0.0276	0.098	-0.002	0.0311				-0.29	-0.31	-0.05	0.197	-0.04	0.031	0.958919	-0.01062	-0.041837449	0.031215155
3436	Q9HBH5	RDH14	retinol dehydrogenase 14	57665	ENSG00000240857	9	-0.1	0.308	-0.09	-0.04	-0.16	-0.21	-0.03	0.213	-0.06	0.2986	0.5454	0.0493	-0.07	-0.011	-0.064	0.3458	0.1417	0.4097	-0.06	-0.46	-0.17	-0.25	-0.16	0.017	-0.03	-0.13	0.463	0.157328	0.066734	-0.201632819	0.268366945
691	F2Z2T0	RABL6	RAB, member RAS oncogene family like 6	55684	ENSG00000196642	11	0.118	0.068	0.046	-0.1	0.06	-0.33	-0.02	0.027	0.085	0.0983	-0.061	-0.077	0.0341	-0.118	0.191	0.0291	0.1594	0.148	-0.01	-1.38	-0.34	-0.41	-0.23	0.026	0.249	0.201	0.249	0.445622	0.177601	-0.050820169	0.228421091
2670	Q641Q3	METRNL	meteorin like, glial cell differentiation regulat(	284207	ENSG00000176845	5	-0.17	-0.02	0.069				-0.16	0.123	0.146	0.2742	0.3745	0.0095				-0.073	0.0851	0.1262	0.123	0.627	0.136				0.28	0.139	-0.22	0.455463	-0.18171	-0.133487194	-0.048227554
507	C9JBI3	PSPH	phosphoserine phosphatase	5723	ENSG00000146733	4	-0.34	0.584	-0.18				-0.16	0.031	-0.17	-0.016	-0.305	0.2691				0.0265	-0.234	-0.104	-0.15	-0.36	0.042				0.142	-0.09	0.111	0.990052	0.013984	0.023631574	-0.009647829
2593	Q53GD3	SLC44A4	solute carrier family 44 member 4	80736	ENSG00000232180	11	0.132	0.66	-0.15	0.018	0.189	-0.47	-0.87	-1.04	-0.92	-0.084	0.4473	0.2228	0.5363	0.0523	-0.063	-0.166	0.1961	-0.211	-0.42	-0.06	-0.26	-0.51	-0.5	-0.1	-0.44	-0.09	-0.1	0.211238	0.005219	-0.375236037	0.380455376
1005	O00399	DCTN6	dynactin subunit 6	10671	ENSG00000104671	2	0.161	0.248	-0.04	-0.11	0.329	-0.38	0.189	-0	0.116	0.0962	-0.006	0.1922	-0.293	-0.429	0.2075	0.2269	0.1321	0.0498	0.103	-0.95	-0.33	-0.73	-0.29	0.032	0.109	0.135	0.103	0.310331	0.25844	0.036302588	0.222137006
2798	Q7Z7M0	MEGF8	multiple EGF like domains 8	1954	ENSG00000105429	10	-0.24	-0.44	0.047	-0.09	0.144	0.107	0.098	0.21	0.17	0.4641	0.0299	0.1861	-0.138	0.2733	-0.261	0.2351	0.0321	0.295	-0.25	0.772	0.349	0.006	0.152	-0.1	0.388	0.474	-0.21	0.531767	-0.17544	-0.12371581	-0.051723088
2944	Q8NEZ2	VPS37A	VPS37A subunit of ESCRT-I;VPS37A, ES(	137492	ENSG00000155975	4	-0.02	0.231	-0.47	-0	-0.09	-0.19	-0.11	0.266	0.004	-0.591	-0.119	0.2471	-0.209	-0.147	0.0186	0.0346	-0.008	0.0739	-0.18	-0.53	-0.36	-0.37	-0.3	-0.26	-0.04	-0.08	0.398	0.558516	0.148447	0.034540688	0.113905902
230	A0A0G2JN5	SPAG11B	sperm associated antigen 11B	10407	ENSG00000164871	3	-0.12	-0.15	-0.25	0.748	0.017	-0.91	0.172	1.866	0.315	-0.205	0.7322	1.3212	-0.216	0.0399	0.1403	-0.357	-0.609	-0.629	-0.16	-0.82	-0.06	-0.26	-0.54	-0.32	-0.19	-0.33	-0.09	0.374284	0.496171	0.163426168	0.332744452
451	B5MC20	JMJD7	jumonji domain containing 7	100137047	ENSG00000243789	4	-0.05	-0.04	-0.23				-0.13	0.123	0.094	-0.022	-0.087	-0.092				0.4942	0.1956	-0.22	-0.3	-0.63	-0.26				0.257	0.104	0.068	0.641544	0.088395	-0.083836766	0.172232219
2900	Q8N490	PNKD	PNKD metallo-beta-lactamase domain conta	25953	ENSG00000127838	8	0.332	0.164	-0.48	0.121	0.005	0.072	-0.32	-0.05	-0.22	-0.254	0.1623	0.1821	0.324	-0.17	-0.34	0.4006	-0.052	-0.01	-0.45	-0.49	-0.2	-0.46	-0.1	-0.19	-0.02	0.036	0.369	0.463778	0.125651	-0.068935389	0.194586301
1026	O00625	PIR	pirin	8544	ENSG00000087842	3	0.204	0.184	0.184	-0.32	0.081	-0.15	-0.15	0.023	-0.26	0.0022	-0.096	-0.14	0.3831	0.036	0.1419	-0.141	0.366	-0.144	0.116	-0.89	0.071	0.225	0.072	-0.04	-0.05	-0.01	-0	0.781225	0.033513	-0.068126111	0.101638925
3293	Q9BVM4	GGACT	gamma-glutamylamine cyclotransferase	87769	ENSG00000134864	4	-0.09	0.857	0.259	0.035	-0.33	-0.49	-0.41	0.434	-0.23	0.5501	0.28	0.0714	0.1092	-0.024	0.1782	-0.066	-0.155	0.4016	0.003	-0.81	0.024	-0.47	-0.61	-0.17	-0.15	0.308	0.191	0.309624	0.192664	-0.145539277	0.338203376
3544	Q9NZ09	UBAP1	ubiquitin associated protein 1	51271	ENSG00000165006	6	0.228	0.307	-0.09	0.068	-0.15	-0.37	-0.05	0.092	-0.03	-0.148	0.0927	0.2774	-0.157	-0.108	0.0687	0.0531	0.1317	0.3013	-0.12	-0.47	-0.2	-0.43	-0.48	-0.09	0.053	0.03	0.405	0.325009	0.145625	-0.055358409	0.200983603
770	Q15166	PON3	paraoxonase 3	5446	ENSG00000105852	3	-0.25	-0.85	-0.86	-0.12	-0.09	-0.09	0.287	0.063	-0.06	0.0431	-0.287	0.4284	-0.106	0.0446	0.0908	0.2795	-0.282	-0.206	-0.28	0.79	-0.23	-0.07	-0.2	-0.25	0.413	-0.19	-0.03	0.470391	-0.21287	-0.219217658	0.006350677
2822	Q86X55	CARM1	coactivator associated arginine methyltransf	10498	ENSG00000142453	7	-0.01	-0.01	-0.14	-0.04	0.062	-0.25	0.305	0.178	0.455	0.1599	0.0889	-0.055	0.0569	0.0034	0.0167	0.4453	0.2404	0.3726	0.264	0.11	0.332	-0.27	-0.17	0.199	0.172	0.236	0.352	0.73832	-0.07459	-0.086174211	0.011583129
40	Q9NXU5	ARL15	ADP ribosylation factor like GTPase 15	54622	ENSG00000185305	5	0.025	0.631	0.105	0.177	0.024	-0.53	0.09	-0.11	-0.02	0.1172	-0.002	-0.043	0.1652	-0.209	0.1202	0.2705	0.2379	0.0668	-0.27	-0.33	-0.41	-0.57	-0.24	0.035	-0.13	0.038	0.361	0.236712	0.211451	-0.037349104	0.248799666
2470	Q14677	CLINT1	clathrin interactor 1	9685	ENSG00000113282	4	-0.06	0.111	-0.19	-1.24	2.172	-1.1	-0.73	-0.39	-0.33	-0.213	-0.07	-0.109	0.0426	-1.072	0.0406	-0.644	-0.345	-0.635	-0.17	-0.82	-0.19	1.062	0.234	0.581	-0.16	-0.3	-0.39	0.739064	-0.1781	0.139212315	-0.317313721
1884	P37235	HPCAL1	hippocalcin like 1	3241	ENSG00000115756	7	0.018	0.284	0.277	0.105	-0.15	-0.18	-0.33	-0.51	-0.46	0.3646	0.0965	0.1273	0.0825	-0.092	0.3362	-0.189	-0.086	-0.654	-0.11	-0.45	-0.08	-0.23	-0.18	0.27	-0.49	-0.1	-0.24	0.562399	0.072985	-0.103076438	0.176061155
3567	Q9P2B2	PTGFRN	prostaglandin F2 receptor inhibitor	5738	ENSG00000134247	5	-0.28	0.03	0.313				-0.16	0.212	-0.29	0.2946	0.4059	0.0556				-0.347	-0.146	-0.329	-0.23	-0.17	-0.06				0.098	-0.12	-0.35	0.749121	0.109353	-0.018533782	0.127886775
619	E7EW39	SLC36A1	solute carrier family 36 member 1	206358	ENSG00000123643	5	0.269	0.307	-0.38	-0.34	-0.27	0.056	-0.27	-0.76	-0.26	0.476	0.4053	-0.135	-0.048	-0.305	-0.149	-0.111	-0.128	-0.386	0.202	-0.67	0.085	-0.49	-0.31	0.412	-0.6	-0.58	-0.22	0.602949	0.05628	-0.141098574	0.19737815
3553	Q9NZN3	EHD3	EH domain containing 3	30845	ENSG00000013016	22	-0.22	0.191	0.14	-0.1	-0.04	-0.17	-0.03	0.243	0.081	0.0514	-0.057	-0.305	-0.169	-0.485	0.5545	0.511	-0.067	-0.41	-0.02	-0.19	-0.31	-0.57	-0.38	0.093	-0.1	-0.06	0.128	0.561466	0.166668	0.051545117	0.115123278
504	C9J8E1	MAPKAPK	mitogen-activated protein kinase-activated p	7867	ENSG00000114738	4	-0.06	-0.03	-0.09	0.097	-0.11	-0.24	-0.38	-0.44	-0.01	0.3514	-0.005	0.2651	0.0763	-0.133	0.1775	-0.176	0.0519	-0.087	-0.38	-0.66	0.081	-0.36	-0.25	0.057	-0.05	-0.07	0.405	0.290152	-0.0059	-0.199188819	0.193286214
2054	P52294	KPNA1	karyopherin subunit alpha 1	3836	ENSG00000114030	6	-0.19	-0.27	-0.1	-0.57	-0.35	-0.16	0.129	0.038	0.27	0.0145	-0.141	0.0107	-0.357	0.3531	-0.208	0.1621	0.1668	0.3526	-0.34	-0.41	-0.42	-0.26	0.075	0.315	0.081	0.17	0.181	0.532368	-0.06717	-0.174116156	0.106948311
3212	Q99574	SERPINI1	serpin family I member 1	5274	ENSG00000163536	4	-0.17	-0.14	-0.18	0.233	0.238	-0.1	0.012	-0.06	0.219	0.7046	0.3599	0.115	-0.246	-0.023	-0.044	0.1198	0.3543	0.3488	0.144	0.362	0.579	0.606	-0.01	0.022	0.22	0.395	0.317	0.168946	-0.28807	-0.182636485	-0.105431461
3495	Q9NRY6	PLSCR3	phospholipid scramblase 3	57048	ENSG00000187838	5	-0.21	-0.15	-0.08	0.614	0.53	0.263	0.061	0.254	-0.16	-0.189	0.0071	-0.136	0.4209	-0.27	0.1518	0.1837	-0.25	0.2051	-0.24	-0.36	-0.26	-0.3	-0.43	-0.28	-0.21	-0.32	-0.09	0.059694	0.402821	0.111026762	0.291794451
1034	O14494	PLPP1	phospholipid phosphatase 1	8611	ENSG00000067113	7	0.151	0.58	-0.1	-0.05	-0.17	-0.33	-0.37	0.164	-0.43	-0.024	0.2302	0.2251	0.3612	-0.126	0.1266	0.1619	0.0549	0.1625	-0.43	-0.86	-0.19	-0.23	-0.22	-0.12	-0.41	-0	0.312	0.14687	0.176483	-0.191027559	0.36751035
758	Q567U6	CCDC93	coiled-coil domain containing 93	54520	ENSG00000125633	8	-0.13	-0.27	-0.04	-0.17	0.03	-0.68	0.011	0.299	0.291	0.0609	0.1256	-0.124	-0.05	-0.084	-0.006	0.2379	0.3987	0.3704	0.214	-0.08	0.118	-0.44	-0.31	0.08	0.152	-0.04	0.097	0.450892	-0.05064	-0.177399267	0.126762276
671	E9PL40	TSPAN18	tetraspanin 18	90139	ENSG00000157570	3	-0.22	0.067	-0.07	-0.96	0.053	0.378	0.05	0.14	0.467	-0.026	-0.059	-0.355	-0.137	0.1108	0.0012	0.2214	0.4353	0.4859	0.19	0.34	0.15	0.221	0.021	-0.06	0.039	0.172	0.091	0.717474	-0.13905	-0.085074841	-0.053978536
1762	P27105	STOM	stomatin	2040	ENSG00000148175	6	-0.28	-0.39	0.212	0.379	0.107	-0.02	-0.29	0.341	-0.3	-0.306	-0.35	-0.064	0.1217	0.3768	0.159	-0.57	-0.24	-0.235	-0.31	-0.19	-0.12	0.291	-0.41	-0.11	-0.13	0.002	-0.76	0.622602	0.168135	0.096698593	0.07143683
740	F8VWE0	TSPAN31	tetraspanin 31	6302	ENSG00000135452	4	-0.32	-0.82	-0.12	-0.37	-0.47	-0.3	-0.23	-0.25	-0.19	0.1922	-0.14	-0.072	0.2249	-0.241	-0.159	-0.131	0.1044	-0.337	-0.42	-0.27	-0.32	-0.44	-0.19	0.468	-0.15	0.066	-0.1	0.17265	-0.19006	-0.279629986	0.08957233
929	J3KT73	RPL38	ribosomal protein L38	6169	ENSG00000172809	2	0.454	0.23	0.071	-0.2	0.235	-0.4	0.394	-0.16	0.427	-0.043	0.1463	0.1805	-0.026	-0.344	0.1393	0.2968	0.2884	0.0431	-0	-0.87	0.047	-0.61	-0.05	0.027	0.032	-0.08	0.186	0.295156	0.263125	0.039918083	0.223207023
574	D6RF76	CCDC112	coiled-coil domain containing 112	153733	ENSG00000164221	3	-0.11	-0.45	-0.19	-0.87	-0.13	-0.46	0.042	-0.06	0.114	-0.054	-0.26	-0.235	-0.565	0.2051	-0.184	0.0245	-0.034	-0.151	0.07	0.881	0.034	-0.31	-0.23	-0.16	0.472	-0.05	-0.16	0.289307	-0.29663	-0.095805587	-0.200829118
3287	Q9BV86	NTMT1	N-terminal Xaa-Pro-Lys N-methyltransferase	28989	ENSG00000148335	5							0.312	0.002	0.221							0.1561	0.3362	0.188							-0.01	0.17	0.386	0.94371	-0.00341	-0.048330351	0.044916512
3330	Q9C0C9	UBE2O	ubiquitin conjugating enzyme E2 O	63893	ENSG00000175931	7	-0.25	0.151	0.038	-0.08	0.044	-0.53	-0.01	0.01	0.2	0.2076	0.0715	0.0226	0.0867	-0.252	-0.192	0.043	0.0506	-0.023	-0.26	-0.36	0.07	-0.69	-0.08	0.118	0.131	-0	0.075	0.676238	0.064228	-0.049879846	0.114108172
2934	Q8NDH3	NPEPL1	aminopeptidase like 1	79716	ENSG00000215440	5	-0.13	0.214	-0.04	0.063	-0.04	-0.42	0.291	0.087	0.087	0.2491	0.1257	0.0292	0.3534	-0.063	0.2253	0.3375	0.2625	0.1237	-0.39	-0.63	-0.14	-0.14	-0.19	0.045	0.081	0.18	0.118	0.125319	0.129294	-0.171540567	0.300834153
3528	Q9NX08	COMMD8	COMM domain containing 8	54951	ENSG00000169019	4	-0.08	-0.03	0.11	-0.02	-0.16	-0.55	0.214	0.232	0.232	-0.084	-0.048	-0.144	-0.125	-0.14	-0.116	0.2337	0.3428	0.4785	-0.32	0.013	-0.09	-0.19	-0.23	0.006	-0.05	0.058	0.122	0.636645	0.071383	-0.048816674	0.120200037
2748	Q6ZS30	NBEAL1	neurobeachin like 1	65065	ENSG00000144426	13	-0.22	-0.06	-0.17	0.105	0.268	-0.16	-0.12	-0.22	0.056	0.2782	-0.085	-0.036	0.2297	-0.094	0.0812	0.1273	0.1066	0.268	-0.29	-0.27	-0.1	-0.2	-0.04	0.168	0.514	0.071	0.382	0.45515	-0.08564	-0.155935052	0.070295569
987	O00151	PDLIM1	PDZ and LIM domain 1	9124	ENSG00000107438	3				-0.24	-0.11	-0.22	0.033	-0.09	-0.2				-0.14	0.3553	0.1154	-0.214	0.017	-0.183				-0.41	-0.46	-0.15	0.02	-0.07	0.055	0.472241	0.030966	-0.130231286	0.161197119
213	A0A0X1KG7	COBLL1	cordon-bleu WH2 repeat protein like 1	22837	ENSG00000082438	3				0.193	0.603	-0.15	-0.65	-0.03	-0.38				0.1406	0.0051	0.0261	-0.018	-0.118	-0.103				-0.27	-0.3	0.232	-0.14	-0.2	0.07	0.908694	0.0323	-0.057088223	0.089388489
358	A2A2V1	PRNP	prion protein	5621	ENSG00000171867	3	-0.21	-0.1	0.463	0.025	0.195	0.563	0.121	0.466	0.167	0.2318	-0.141	-0.24	-0.55	-0.254	-0.497	-0.623	-0.05	0.0326	-0.22	1.056	-0.5	0.064	-0.23	-0.48	-0.49	-0.41	-0.36	0.17283	0.359896	0.419204925	-0.059308562
1942	P46060	RANGAP1	Ran GTPase activating protein 1	5905	ENSG00000100401	5	0.269	0.423	0.283	-0.08	0.164	-0.07	0.069	-0.01	0.214	0.4154	0.0898	0.3196	0.1132	0.1613	0.0589	-0.005	0.0522	0.0041	0.179	-0.44	-0.05	-0.41	-0.15	0.045	0.113	0.051	0.195	0.203082	0.192743	0.00604058	0.186702715
3474	Q9NR31	SAR1A	secretion associated Ras related GTPase 1.	56681	ENSG00000079332	6							0.194	0.192	0.219							0.1271	0.485	0.3772							0.024	0.098	0.321	0.48098	0.053972	-0.128165354	0.182137168
35	A0A087WU!	OPRK1	opioid receptor kappa 1	4986	ENSG00000082556	2				0.593	0.028	0.174	0.232	0.255	0.168				0.4569	0.3231	0.0286	0.6393	-0.321	-0.022				0.211	0.227	0.113	0.053	0.244	0.27	0.927474	0.055266	0.057538679	-0.002272685
3149	Q96IV0	NGLY1	N-glycanase 1	55768	ENSG00000151092	13	0.028	-0.07	0.195	0.235	0.201	-0.19	0.109	0.217	0.335	-0.118	0.1055	0.1836	-0.004	-0.124	-0.066	0.1177	0.2078	0.2121	-0.31	-0.82	-0.08	0.206	-0.23	0.177	0.235	0.22	0.406	0.638936	0.139972	0.060143826	0.079828264
3755	Q9Y4D1	DAAM1	dishevelled associated activator of morphog(	23002	ENSG00000100592	9	0.104	0.22	-0.11	-0.03	-0.07	-0.18	-0.06	0.044	0.217	-0.231	-0.015	0.0506	0.1142	-0.086	0.3107	0.4607	0.1864	0.3408	-0.21	-0.2	-0.27	-0.63	-0.13	-0.37	1E-04	0.237	0.319	0.199868	0.154341	-0.110491805	0.264832561
1543	P10412	H1-4;HIST	H1.4 linker histone, cluster member;histone	3008	ENSG00000168298	8	-0.77	-1.37	-0.82	-1.06	-1.16	-0.46	-1.45	-1.69	-1.33	-1.382	0.331	-1.009	-1.015	-0.481	-0.978	-0.839	-0.973	-1.732	-0.21	-0.53	2.929	0.129	-0.37	1.2	-0.55	-1.17	-1.33	0.131902	-1.13377	-0.225310408	-0.908464402
3686	Q9UPU5	USP24	ubiquitin specific peptidase 24	23358	ENSG00000162402	9	0.032	0.114	0.016	0.044	0.112	-0.11	-0.04	-0.2	0.059	0.4076	0.1601	0.1408	0.0785	-0.04	0.3445	0.1198	0.0067	-0.021	-0.06	-0.41	-0.06	-0.12	-0.37	0.121	0.265	0.08	0.092	0.214227	0.055077	-0.129154418	0.184231811
1900	Q5QNY5	PEX19	peroxisomal biogenesis factor 19	5824	ENSG00000162735	7	0.129	0.239	0.285	-0.24	0.152	-0.07	-0.14	-0	0.082	0.4312	0.1252	0.1678	0.0287	0.1398	-0.031	0.019	-0.042	-0.188	0.396	0.138	0.279	-0.58	0.006	0.055	0.099	0.085	-0.12	0.962776	0.008491	-0.024276572	0.032767766
14	A0A087X0Q	IGKV2D-4(	immunoglobulin kappa variable 2D-40;immu	28878;2891(	ENSG00000251039	3	-2.29	-1.19	-0.81	-1.79	-1.31	-0.69	-1.26	-0.67	-1.04	-1.136	-1.445	-0.658	-1.19	-1.351	-1.791	-0.848	-0.964	-1.221	-1.64	-0.86	2.586	2.666	-0.17	1.266	-0.45	-0.44	-2.1	0.127109	-1.32252	-0.049112817	-1.273406569
3220	Q99622	C12orf57	chromosome 12 open reading frame 57	113246	ENSG00000111678	6	0.157	0.274	-0.24	-0.14	-0.39	-0.47	-0.21	-0.07	-0.05	0.1912	0.2211	0.1409	-0.145	-0.499	0.0178	0.1003	0.0968	-0.08	-0.04	-0.75	-0.09	-0.5	-0.68	-0.66	0.188	-0.01	0.099	0.294764	0.145127	-0.131307183	0.276433899
174	A0A0A6YYL	CORO7-P/	CORO7-PAM16 readthrough	100529144	ENSG00000103426	4				0.237	-0.18	-0	0.11	0.115	0.167				-0.015	0.0196	0.1645	0.1273	-0.144	-0.059				0.474	-0.34	0.146	0.257	0.072	0.162	0.711047	-0.05286	0.059914367	-0.11277158
872	K7ERG2	NKIRAS2	NFKB inhibitor interacting Ras like 2	28511	ENSG00000168256	3	0.09	-0.04	-0.16				0.084	0.507	0.347	0.799	0.6642	0.2161				0.0669	-0.178	-0.035	0.295	-0.03	0.006				0.416	0.037	0.41	0.856183	-0.05164	-0.118719634	0.067080275
3291	Q9BVK6	TMED9	transmembrane p24 trafficking protein 9	54732	ENSG00000184840	4	0.216	0.209	0.148	0.151	0.619	-0.25	-0.03	0.311	0.425	0.0198	0.0885	0.0098	0.3577	-0.16	0.0369	-0.288	0.1098	0.1138	-0.31	-0.46	-0.4	-0.18	-0.11	-0.15	-0.06	0.118	0.461	0.146358	0.32168	0.167608399	0.154071174
1111	O43318	MAP3K7	mitogen-activated protein kinase kinase kina	6885	ENSG00000135341	3	0.232	0.143	0.138	0.129	0.041	0.572	0.261	0.375	0.248	0.4241	0.1698	0.4634	0.5306	0.1219	0.2761	-0.074	-0.209	-0.034	0.042	-0.47	-0.13	0.287	0.098	0.367	0.148	-0.11	0.262	0.404973	0.181305	0.052164986	0.129140057
3715	Q9Y2G3	ATP11B	ATPase phospholipid transporting 11B (puta	23200	ENSG00000058063	8	-0.03	0.559	-0.24	0.195	-0.23	-0.45	-0.55	0.003	-0.11	-0.144	-0.009	0.2467	0.372	-0.299	0.0606	-0.032	0.1336	0.0212	-0.33	-0.45	-0.35	-0.63	-0.21	-0.11	-0.21	0.051	0.099	0.241618	0.142414	-0.134473008	0.276887286
2711	Q6PD74	AAGAB	alpha and gamma adaptin binding protein	79719	ENSG00000103591	4	0.216	0.029	-0.22	-0.1	0.14	-0.37	-0.07	0.462	0.261	0.0596	0.203	0.3373	0.3425	0.0573	0.1285	0.358	0.318	0.5339	-0.09	-0.65	-0.08	-0.36	-0.01	0.345	-0.06	0.252	0.115	0.147734	0.099241	-0.220576315	0.319817028
355	A1KXE4	FAM168B	family with sequence similarity 168 member	130074	ENSG00000152102	2	-0.33	0.086	-0.23	-0.3	-0.03	-0.25	-0.02	0.362	-0.12	0.0571	0.2626	-0.151	-0.205	-0.195	-0.193	0.4066	-0.071	0.4681	-0.29	-0.37	-0.08	-0.29	-0.4	-0.12	0.087	0.127	0.403	0.562377	0.01285	-0.133145447	0.145995384
2897	Q8N3F0	MTURN	maturin, neural progenitor differentiation reg	222166	ENSG00000180354	4	0.031	0.374	0.162	0.349	-0.06	-0.17	-0.25	-0.27	-0.1	0.3816	0.3852	0.1414	-0.098	0.076	0.4887	-0.237	0.0312	-0.331	-0.2	-1.11	-0.38	-0.63	-0.24	0.087	-0.52	-0.19	0.079	0.111194	0.353131	-0.085957144	0.439088145
432	B4DNG0	OLFML3	olfactomedin like 3	56944	ENSG00000116774	3	-0.13	-0.26	-0.13	-0.03	0.164	-0.28	0.081	0.905	0.284	-0.091	-0.275	-0.206	-0.139	-0.092	0	0.2964	0.2229	0.8077	-0.14	0.089	0.322	-0.09	-0.26	0.048	0.723	-0.07	0.304	0.970274	-0.03649	0.008352326	-0.044842202
1996	P49458	SRP9	signal recognition particle 9	6726	ENSG00000143742	5	0.35	0.152	0.016				0.114	0.154	0.138	0.1615	0.2394	0.0347				0.1364	0.1045	-0.009	-0.18	-0.58	0.036				0.214	0.158	0.098	0.356582	0.196578	0.042591951	0.153985845
32	A0A087WTZ	UBXN1	UBX domain protein 1	51035	ENSG00000162191	3				-0.07	-0.68	0.531	0.195	0.057	0.245				-0.39	0.9877	0.0639	0.018	0.3425	0.226				-0.16	-0.04	0.029	0.065	0.158	0.293	0.788597	-0.01043	-0.161715158	0.151286367

1413	P04155	TFF1	trefoil factor 1	7031	ENSG00000160182	2	-0.71	-1.21	-0.63	-0.06	-0.09	-0.66	-0.11	-0.36	-0.73	1.4408	-0.697	0.1762	-0.579	-0.453	-0.957	0.0647	0.1533	-0.033	-0.34	0.532	-0.31	1.262	-0.37	0.547	0.179	-0.31	-1.01	0.328506	-0.52705	-0.408389218	-0.118664953
3153	Q96JB2	COG3	component of oligomeric golgi complex 3	83548	ENSG00000136152	8	0.014	0.045	0.195	0.046	-0.02	-0.28	0.08	0.045	0.12	0.0728	-0.028	0.1996	0.0935	-0.131	-0.16	0.0988	0.0346	0.0987	-0.25	-0.35	-0.15	-0.02	-0.2	0.125	0.15	0.106	0.168	0.614757	0.074758	-0.003027299	0.077785345
932	J3QQJ5	TRAPPC8	trafficking protein particle complex 8	22878	ENSG00000153339	9	0.199	0.131	0.076	-0.54	0.027	-0.71	-0.17	0.037	0.031	0.028	0.1295	-0.094	-0.171	-0.728	-0.684	0.1223	-0.142	-0.12	-0.28	-0.55	-0.28	-0.59	0.165	-0.01	0.207	0.064	-0.03	0.904364	0.043333	0.081708791	-0.038376236
1087	O15264	MAPK13	mitogen-activated protein kinase 13	5603	ENSG00000156711	3	-0.02	0.141	-0.16	-0.06	-0.06	-0.07	0.209	-0.47	0.085	0.074	0.0563	0.0417	0.295	0.2475	0.1104	-0.161	0.3007	0.0291	-0.22	-0.24	-0.2	-0.48	-0.34	-0.15	-0.18	-0.2	0.303	0.094843	0.144705	-0.156015628	0.300720675
738	F8VUW5	DAZAP2	DAZ associated protein 2	9802	ENSG00000183283	2	-0.19	0.613	-0.31	-0.42	-0.19	-0.43	-0.17	0.216	-0.27	-0.654	0.206	0.0437	0.0228	-0.158	-0.103	0.3303	-0.19	0.5667	-0.12	-0.32	-0.02	0.217	-0.1	-0.06	-0.01	-0.11	0.413	0.713503	-0.11689	-0.136017937	0.019123955
1946	P46736	BRCC3	BRCA1/BRCA2-containing complex subunit	79184	ENSG00000185515	4	0.077	0.009	-0.09	-0.11	0.023	-0.25	-0.04	-0.23	0.098	-0.009	-0.095	-0.041	0.0334	0.0582	-0.013	0.1432	0.1636	0.1714	-0.07	-0.85	-0.23	0.002	-0.13	-0.08	0.088	-0.02	-0.04	0.246774	0.089514	-0.103394049	0.192908149
3240	Q9BQ24	ZFYVE21	zinc finger FYVE-type containing 21	79038	ENSG00000100711	5				0.49	0.069	-0.23	0.589	0.763	0.505				0.1764	-0.071	0.3047	0.2611	0.2828	0.589				0.025	-0.2	-0.09	0.358	0.269	0.682	0.694838	0.190726	0.107091126	0.083634878
2884	Q8N0W3	FCSK;FUK	fucose kinase;fucokinase	197258	ENSG00000157353	13	0.049	0.125	-0.06	0.126	0.27	0.009	-0.35	-0.09	0.236	0.1271	0.0586	0.0529	0.2407	-0.246	0.1853	0.1918	0.2637	0.278	-0.11	-0.5	-0.12	0.314	0.024	0.285	0.248	0.046	0.144	0.67847	0.000242	-0.091710557	0.091952476
3689	Q9UQ49	NEU3	neuraminidase 3	10825	ENSG00000162139	3				-0.41	-0.54	-0.26	0.294	0.584	0.69				-0.808	0.5285	-0.073	0.259	-0.023	0.7122				0.026	-0.14	-0.6	0.437	0.467	0.363	0.991971	-0.03207	-0.039670148	0.007596083
2895	Q8N392	ARHGAP1	Rho GTPase activating protein 18	93663	ENSG00000146376	8	-0.01	0.017	-0.11	-0.22	0.185	-0.57	-0.05	-0.06	-0.04	0.8823	0.1862	0.3311	0.1861	-0.086	-0.075	0.179	0.2152	0.1957	0.329	0.104	0.152	-0.14	-0.13	0.223	-0.04	0.51	-0.07	0.144741	-0.19951	-0.319923908	0.120417244
175	A0A0B4J1W	NAA15	N(alpha)-acetyltransferase 15, NatA auxiliar	80155	ENSG00000164134	10	0.124	0.023	0.018	0.118	0.081	-0.22	-0.02	0.178	0.228	0.1824	0.0767	0.1059	-0.011	-0.082	-0.006	0.1486	0.1769	0.083	0.079	-0.06	-0.17	0.017	-0.11	0.047	0.275	-0.01	-0	0.603849	0.0507	-0.016224764	0.066924292
566	D6RC51	SPINK2	serine peptidase inhibitor, Kazal type 2	6691	ENSG00000128040	7	0.449	-0.59	-0.76				-0.07	0.366	0.464	0.4174	-0.657	-0.143				0.2107	-0.233	0.2802	0.311	1.382	-0.14				-0.35	-0.76	0.044	0.958315	-0.10589	-0.002912745	-0.102978553
3251	Q9BRP4	PAAF1	proteasomal ATPase associated factor 1	80227	ENSG00000175575	4	0.084	-0.21	-0.1				-0.29	-0.22	-0.35	0.2777	0.0908	0.0648				-0.153	0.1326	0.032	-0.21	-0.17	0.364				-0.44	-0.13	0.019	0.246567	-0.08587	-0.254490786	0.168621843
2301	Q02742	GCNT1	glucosaminyl (N-acetyl) transferase 1;glucos	2650	ENSG00000187210	9	-0.31	0.13	0.21	0.044	0.196	-0.75	-0.6	-0.87	-0.6	0.359	-0.187	-0.045	-0.091	0.052	-0.336	-0.054	0.4477	0.2982	-0.35	0.78	0.154	-0.01	-0.66	0.055	-0.03	-0.37	-0.49	0.358708	-0.18165	-0.332614037	0.150963983
1670	P19174	PLCG1	phospholipase C gamma 1	5335	ENSG00000124181	3							0.038	-0.07	0.113							0.2421	0.3137	0.2293							0.465	-0.03	0.075	0.432202	-0.1445	-0.234559876	0.090059254
579	K7EMY9	CIRBP	cold inducible RNA binding protein	1153	ENSG00000099622	3	0.463	0.379	0.201	-0.01	-0.12	-0.4	0.195	0.051	0.135	0.1505	0.5365	0.4025	-0.085	-0.257	0.14	0.0647	-0.003	-0.031	0.096	-0.26	-0.34	-0.26	-0.34	-0.14	0.115	-0.06	0.39	0.352841	0.189961	-0.001796081	0.191757187
1270	O95155	UBE4B	ubiquitination factor E4B	10277	ENSG00000130939	5	-0.02	0.124	-0.22	-0.03	-0.04	-0.13	-0.19	-0.17	-0.01	-0.274	-0.015	0.0835	0.1634	-0.084	0.1792	0.0381	0.1421	0.0089	-0.34	-0.94	-0.46	-0.27	-0.2	0.072	0.077	-0.03	0.083	0.207247	0.145157	-0.103587381	0.248744803
340	A0A2R8YF2	PANK2	pantothenate kinase 2	80025	ENSG00000125779	3				0.117	0.278	-0.03	-0.13	-0.06	0.101				0.4303	-0.106	-0.04	0.0407	0.3369	0.1125				-0.21	-0.36	0.588	0.03	-0.1	0.154	0.804185	0.026923	-0.08374488	0.110667769
2996	Q8TEX9	IPO4	importin 4	79711	ENSG00000196497	8	-0.16	0.046	-0.1	0.284	0.551	-0.25	-0.08	-0.15	-0.18	-0.011	0.0216	-0.049	0.2117	-0.272	-0.159	-0.493	-0.23	-0.032	0.014	-0.19	0.204	-0.09	-0.38	0.198	-0.04	-0.45	-0.06	0.697854	0.085754	0.108576556	-0.022822174
2255	Q5TFQ8	SIRPB1	signal regulatory protein beta 1	10326	ENSG00000101307	2	-0.04	-0.16	0.28	0.042	0.178	0.491	-0.1	0.088	0.098	-0.395	0.106	0.3442	0.4931	-0.583	0.5076	-0.1	0.0851	0.0274	-0.64	-0.2	-0.27	-0.38	-0.42	-0.7	-0.19	0.165	-0.23	0.099457	0.413028	0.042349775	0.370677783
3561	Q9P1F3	ABRACL	ABRA C-terminal like	58527	ENSG00000146386	3	0.46	0.302	0.342	0.302	0.43	-0.19	-0.6	-0.53	-0.31	0.5115	0.2961	0.4193	0.2415	-0.01	0.3659	-0.414	0.2651	-0.393	0.124	-1.13	-0.21	-0.68	-0.38	0.286	-0.78	-0.27	-0.08	0.172989	0.370051	-0.118279184	0.488329997
3725	Q9Y2T7	YBX2	Y-box binding protein 2	51087	ENSG00000006047	2							2.077	0.701	0.948							-0.888	-1.6	-1.046							-0.17	-1.13	-1.1	0.06575	2.041397	2.41990296	-0.378505846
3306	Q9BXW6	OSBPL1A	oxysterol binding protein like 1A	114876	ENSG00000141447	3	-0.05	0.041	-0.32	0.071	-0.12	-0.39	0.015	0.249	0.27	-0.168	-0.13	-0.09	-0.026	-0.211	0.0133	0.4442	0.0409	0.3469	-0.36	-0.79	-0.35	-0.54	-0.45	-0.04	0.359	0.216	0.212	0.440493	0.1652	-0.051950717	0.217150259
677	E9PMG1	REPS1	RALBP1 associated Eps domain containing	85021	ENSG00000135597	5	-0.33	0.267	-0.33	0.015	0.33	-0.34	0.137	0.142	0.313	0.1022	-0.277	-0.191	-0.021	-0.438	0.1418	0.331	0.2263	0.3471	-0.26	0.034	-0.3	-0.55	-0.38	-0.23	0.364	0.065	0.507	0.789898	0.105134	-0.002437889	0.107571551
2709	Q6PCB6	ABHD17C	abhydrolase domain containing 17C	58489	ENSG00000136379	5	-0.23	0.553	-0.11	0.028	-0.38	-0.73	-0.32	-0.11	-0.22	-0.219	0.0668	0.1785	0.3097	-0.441	-0.198	0.1902	0.0793	0.1121	-0.31	-0.2	-0.31	-0.56	-0.44	-0.18	-0.33	0.074	0.112	0.319943	0.069012	-0.176906745	0.245918601
1311	O95817	BAG3	BCL2 associated athanogene 3	9531	ENSG00000151929	6	0.255	0.017	-0.12	-0.1	0.13	-0.4	0.156	0.05	0.014	0.0264	0.1411	-0.177	0.2452	-0.155	0.1298	0.2499	0.1278	0.1696	-0.44	-0.6	0.064	-0.46	-0.33	0.013	0.165	0.061	0.13	0.227853	0.155339	-0.083442424	0.238781665
652	E9PGT6	COPS8	COP9 signalosome subunit 8	10920	ENSG00000198612	4	-0.05	-0.02	0.024	-0.12	0.146	-0.55	0.06	0.154	0.144	0.1984	-0.032	-0.128	-0.104	-0.091	0.1306	0.279	0.2681	0.1607	-0.2	-0.7	-0.03	-0.51	-0.28	-0.03	-0.06	0.013	-0.19	0.124987	0.198185	-0.099725906	0.29791058
3648	Q9UKT5	FBXO4	F-box protein 4	26272	ENSG00000151876	4	0.252	0.702	0.009	-0.09	0.037	-0.34	0.313	-0.12	0.389	0.3152	0.2954	0.4037	0.1289	-0.236	0.1474	0.4441	0.4196	0.8842	0.003	-0.75	-0.28	-0.4	-0.34	0.042	0.261	0.23	0.498	0.203021	0.208065	-0.18382036	0.391885041
999	O00273	DFFA	DNA fragmentation factor subunit alpha	1676	ENSG00000160049	4	-0.02	-0.16	-0.11				0.021	0.319	0.246	0.2065	0.3505	0.0239				0.2274	-0.218	-0.201	-0	0.214	0.312				0.175	-0.21	-0.07	0.988681	-0.02044	-0.015350633	-0.005084947
3595	Q9UDY8	MALT1	MALT1 paracaspase	10892	ENSG00000172175	9	-0.13	-0.31	-0.24	-0.12	0.1	-0.41	-0.09	-0.37	0.011	-0.19	-0.183	0.1004	0.0573	-0.157	-0.139	-0.218	-0.09	-0.42	-0.56	-0.44	0.322	-0.58	-0.27	0.141	-0.12	-0.09	-0.05	0.935174	0.008355	-0.036130461	0.04448536
3099	Q96BS2	TESC	tescalcin	54997	ENSG00000088992	5	0.05	0.213	0.42	-0.14	0.226	0.021	0.096	0.23	-0.01	-0.095	0.056	-0.011	0.0879	-0.404	0.1168	0.1088	0.0469	0.0157	-0.49	-0.57	-0.42	0.065	-0.22	-0.11	0.022	-0.14	0.168	0.103032	0.312024	0.131619252	0.180404929
3535	Q9NXR7	BABAM2	BRISC and BRCA1 A complex member 2	9577	ENSG00000158019	3							-0.18	-0.08	-0.05							0.0441	-0.29	-0.066							0.371	-0.02	-0.19	0.671898	-0.1589	0.000677753	-0.159573659
367	A5PLN9	TRAPPC1:	trafficking protein particle complex 13	80006	ENSG00000113597	3	-0.2	-0.1	-0.08	-0.08	0.057	-0.31	0.121	0.3	0.127	0.0839	0.193	0.0906	0.1761	0.0116	0.167	0.2082	0.2	0.0849	-0.13	-0.32	-0.11	-0.27	-0.17	0.063	0.392	0.063	0.211	0.246477	0.011701	-0.153779877	0.165480649
3579	Q9UBF6	RNF7	ring finger protein 7	9616	ENSG00000114125	1	0.058	0.188	-0.11	0.233	-0.17	-0.32	0.02	0.083	0.242	0.0009	-0.103	0.0324	-0.071	-0.092	0.1662	0.1697	0.0856	-0.038	-0.43	-1.26	-0.37	-0.44	-0.34	-0.08	0.073	-0.02	0.225	0.156123	0.318048	0.007975549	0.310072402
3378	Q9H3H3	C11orf68	chromosome 11 open reading frame 68	83638	ENSG00000175573	4	0.308	0.257	0.289	-0.18	0.179	-0.29	0.174	0.136	0.368	0.447	0.528	0.3417	0.1685	0.0935	0.09	0.1813	0.1782	0.2717	0.131	-1	-0.13	-0.31	-0.06	0.025	0.234	-0.08	0.339	0.157468	0.232282	-0.117710713	0.349992509
1224	O75751	SLC22A3	solute carrier family 22 member 3	6581	ENSG00000146477	2	0.131	0.305	0.151				-0.48	0.028	-0.09	0.1052	-0.14	-0.18				0.1747	0.6154	0.1501	-0.39	-1.04	0.191				-0.29	0.419	0.201	0.618535	0.158803	-0.113185856	0.271988767
26	A0A087WW	PLCB1	phospholipase C beta 1	23236	ENSG00000182621	8	0.16	0.272	-0.16	-0.3	-0.33	-0.5	0.674	0.171	0.813	0.1378	0.0747	-0.095	-0.438	-0.217	0.1784	0.7677	0.0204	0.1902	-0.09	-0.27	-0.25	-0.27	-0.59	-0.26	0.181	0.228	0.541	0.687572	0.175746	0.020524214	0.155221828
2348	Q09028	RBBP4	RB binding protein 4, chromatin remodeling	5928	ENSG00000162521	4	-0.03	0.009	-0.05	0.354	0.18	-0.18	-0.14	0.987	-0.01	0.2153	0.1342	0.1285	0.4741	-0.231	0.1692	0.2708	-0.146	0.4986	-0.2	-0.13	0.109	-0.2	-0.25	0.106	0.383	-0.01	0.148	0.556079	0.129358	-0.043797112	0.173155534
2987	Q8TDX7	NEK7	NIMA related kinase 7	140609	ENSG00000151414	5	0.222	-0.02	0.021				0.004	0.163	0.292	0.2533	0.1984	0.012				0.1864	-0.045	0.1457	-0.01	-0.19	-0				0.085	0.111	0.242	0.623146	0.073463	-0.012151966	0.085615313
2860	Q8IXQ6	PARP9	poly(ADP-ribose) polymerase family membe	83666	ENSG00000138496	4	0.267	0.604	0.027				-0.22	-0.3	-0.06	0.3172	0.2818	0.3514				-0.011	-0.203	-0.036	0.285	-0.39	0.233				0.071	-0.15	0.021	0.867846	0.042754	-0.063652752	0.106406447
2898	Q8N3F8	MICALL1	MICAL like 1	85377	ENSG00000100139	3	0.26	0.529	0.241	0.021	-0.07	-0.34	0.083	0.122	0.41	-0.027	0.091	0.1519	-0.236	-0.22	-0.016	0.0804	0.1931	0.2948	-0.53	-0.84	0.06	-0.52	0.08	-0.07	-0.15	0.17	0.085	0.173692	0.329901	0.105079623	0.224821283
3566	Q9P287	BCCIP	BRCA2 and CDKN1A interacting protein	56647	ENSG00000107949	4	-0.02	0.226	0.039				-0.02	-0.05	-0.01	0.0088	0.2473	0.1327				0.0768	0.306	-0.044	-0.05	-0.57	0.221				-0.01	0.087	-0.1	0.385186	0.096478	-0.095595523	0.192073807
2262	P80297	MT1X	metallothionein 1X	4501	ENSG00000187193	4	0.953	0.231	-0.16	0.038	-0.43	0.72	-0.75	0.825	-0.64	-0.305	-0.622	-0.059	0.4957	-0.789	0.1018	0.438	-0.721	-1.069	-0.18	-1.08	-0.11	-0.2	0.393	0.522	0.313	-0.04	0.285	0.524462	0.097517	0.368457658	-0.270940443
2698	Q6P1N0	CC2D1A	coiled-coil and C2 domain containing 1A	54862	ENSG00000132024	9	-0.31	0.303	-0.21	0.053	0.2	-0.54	0.633	0.242	-0.09	-0.034	0.1286	0.1343	-0.027	-0.245	0.0479	0.1581	0.0268	0.2805	-0.19	-0.29	-0.21	-0.5	-0.4	-0.1	0.136	-0.11	0.574	0.54579	0.152704	-0.020818622	0.173522973
3394	Q9H4E7	DEF6	DEF6 guanine nucleotide exchange factor;D	50619	ENSG00000023892	4				0.008	0.092	-0.4	-0.03	-0.2	0.25				0.092	0.1517	0.1073	0.446	0.386	0.3931				0.01	-0.19	0.203	-0.25	0.038	0.445	0.20526	-0.08878	-0.309127533	0.220347048
2021	P50479	PDLIM4	PDZ and LIM domain 4	8572	ENSG00000131435	2	-0.06	-0.31	-0.07				-0.01	-0.06	-0.09	-0.4	0.1536	-0.429				-0.456	0.027	-0.282	-0.27	-0.03	-0.31				-0.13	-0.22	0.061	0.603699	0.048832	0.131603012	-0.082771457
638	G5E9R3	RPL37A	ribosomal protein L37a	6168	ENSG00000197756	2	0.23	-0.6	-0.03				0.409	-0.16	0.347	-0.145	-0.275	-0.307				-0.038	-0.201	-0.137	0.037	-0.71	0.095				0.341	-0.1	0.112	0.614577	0.069181	0.215625644	-0.146445101
1261	O94855	SEC24D	SEC24 homolog D, COPII coat complex cor	9871	ENSG00000150961	8	0.056	-0.12	-0.1				-0.36	-0.09	-0.03	-0.039	0.0043	-0.023				-0.031	0.0907	0.0299	-0.31	-0.49	0.01				0.154	-0.05	0.135	0.624551	-0.01523	-0.110899442	0.095671912
749	F8VY02	ERP29	endoplasmic reticulum protein 29	10961	ENSG00000089248	2							0.553	0.384	0.749							0.3595	1.5829	0.2572							-0.29	-0.14	-0.22	0.214661	0.779806	-0.171193798	0.950999881
1140	O43815	STRN	striatin	6801	ENSG00000115808	2							0.133	-0.01	0.13							0.0723	0.0373	0.2481							0.347	0.061	0.247	0.547692	-0.13373	-0.034507936	-0.099224232
489	C9J2Y9	POLR2B	RNA polymerase II subunit B	5431	ENSG00000047315	8				-0.04	-0.11	-0.41	0.212	0.27	0.357				0.2123	-0.149	-0.117	0.3909	-0.087	0.3384				0.234	-0.1	0.252	0.148	0.112	0.234	0.835812	-0.09987	-0.051007199	-0.048862794
2193	P62310	LSM3	LSM3 homolog, U6 small nuclear RNA and	27258	ENSG00000170860	2	-0	0.206	0.119	0.004	0.203	-0.26	-0.65	-0.28	-0.4	0.085	0.1433	0.1605	-0.071	0.0762	0.1578	-0.134	0.3878	0.3332	-0.11	-0.4	0.053	-0.31	-0.11	0.062	-0.53	-0.41	-0.16	0.114618	0.091984	-0.245449915	0.337433579
3322	Q9BZE9	ASPSCR1	ASPSCR1 tether for SLC2A4, UBX domain	79058	ENSG00000169696	6	0.042	0.266	0.221	-0.31	-0.42	-0.19	0.352	0.316	0.557	0.1755	0.1325	-0.024	-0.253	-0.104	-0.1	0.4793	0.3394	0.433	0.02	-0.57	-0.1	-0.11	-0.25	-0.36	0.156	0.28	0.54	0.636796	0.135031	-0.027339516	0.16237006
887	H7C1J4	UHRF1BP	UHRF1 binding protein 1	54887	ENSG00000065060	6	0.125	0.241	-0	-0.09	-0.07	-0.32	-0.21	-0.17	0.188	0.1867	0.236	0.5955	-0.004	-0.153	0.0149	0.1454	-0.086	-0.062	0.242	-0.6	-0.18	-0.37	-0.17	0.199	-0.05	-0.03	-0.02	0.327957	0.074645	-0.131597067	0.206242208
3693	Q9UQE7	SMC3	structural maintece of chromosomes 3	9126	ENSG00000108055	2	0.22	-0.36	0.136				-0.19	-0.36	-0.33	0.6516	0.1957	0.4028				-0.175	-0.272	-0.544	-0.42	-0.22	0.627				0.135	-0.54	0.014	0.789314	-0.08113	-0.190824697	0.109689718
3329	Q9C0C2	TNKS1BP	tankyrase 1 binding protein 1	85456	ENSG00000149115	10	0.145	0.125	0.058	-0.27	-0.17	-0.42	-0.03	-0.14	0.058	0.0359	0.1238	0.0417	-0.041	-0.056	-0.065	0.1783	0.0805	0.2116	-0.18	-0.65	-0.22	-0.6	-0.38	-0.18	0.341	-0.03	-0.16	0.146298	0.157405	-0.128209609	0.285614943
2048	P51674	GPM6A	glycoprotein M6A	2823	ENSG00000150625	3	-0.18	0.044	-0.04	-0.03	0.67	0.551	-0.32	-0.32	-0.39	-0.212	0.2326	0.0576	0.0775	0.0028	-0.171	0.3381	0.1916	0.4931	-0.4	-0.28	-0.2	-0.29	0.235	-0.35	-0.5	-0.06	0.622	0.450631	0.132645	-0.115241104	0.247886157
1085	O15232	MATN3	matrilin 3	4148	ENSG00000132031	7	-0.64	-1.21	0.421	-0.19	-0.33	0.052	0.015	0.329	-0.34	0.0644	0.8899	-0.284	-0.254	0.5421	-0.154	0.0045	-0.02	-0.028	-0.2	1.036	0.237	0.246	-0	-0.1	0.686	-0.16	0.105	0.286474	-0.41708	-0.295890333	-0.1211892
412	B1ALA9	PRPS1	phosphoribosyl pyrophosphate synthetase 1	5631	ENSG00000147224	14	0.099	0.281	-0.04	-0.14	0.095	-0.48	-0.06	0.146	0.339	0.34	0.0077	0.096	0.1942	-0.095	-0.042	0.367	0.2112	0.3485	0.214	-0.33	-0.14	-0.36	-0.45	0.15	0.234	0.225	0.376	0.497568	0.035282	-0.131799612	0.167081598
3558	Q9P0J7	KCMF1	potassium channel modulatory factor 1	56888	ENSG00000176407	3	0.02	0.13	-0.04	-0.58	-0.32	-0.33	0.11	-0.01	0.185	-0.035	0.0712	-0.008	-0.99	-0.415	-0.455	0.2235	0.068	0.1771	-0.18	-0.57	0.65	-0.25	0.06	-0.35	0.176	0.044	0.105	0.848736	-0.05735	0.058088158	-0.115441952
372	A6NDU8	C5orf51	chromosome 5 open reading frame 51	285636	ENSG00000205765	5	0.082	0.241	0.232	-0.52	-0.51	-0.53	-0.5	-0.26	-0.11	0.2441	-0.128	0.2135	-0.096	-0.022	-0.253	-0.078	0.249	0.153	-0.12	-0.22	0.116	-0.11	-0.26	-0.17	-0.13	-0.15	0.12	0.252808	-0.10449	-0.239739446	0.135248902
3575	Q9P2X3	IMPACT	impact RWD domain protein	55364	ENSG00000154059	4	0.195	0.04	-0.06				0.058	0.197	0.393	0.2346	-0.027	0.1788				0.1099	0.1229	0.2781	-0.13	-0.94	-0.27				-0.14	0.139	0.508	0.35366	0.276895	-0.011993113	0.288887618
2123	P57772	EEFSEC	eukaryotic elongation factor, selenocysteine-	60678	ENSG00000132394	8	0.145	0.319	0.057	0.053	-0.03	-0.19	0.183	0.083	0.291	0.2052	0.1522	0.215	-0.061	-0.184	0.2463	0.0324	0.3076	0.0482	-0.11	-0.41	0.004	-0.29	-0.01	0.29	-0.09	0.168	0.064	0.310824	0.143813	-0.006058175	0.149871138
3555	Q9NZV1	CRIM1	cysteine rich transmembrane BMP regulator	51232	ENSG00000150938	6	-0.81	-0.72	-0.37	-0.55	0.025	-0.39	0.177	-0.05	0.089	0.3786	-0.086	0.6895	0.2247	0.1129	-0.008	0.4321	0.0521	0.1174	-0.5	0.425	-0.24	-0.04	-0.17	0.111	0.515	0.341	-0.45	0.112853	-0.28837	-0.50215355	0.213785696
3515	Q9NV35	NUDT15	nudix hydrolase 15	55270	ENSG00000136159	2	0.302	0.286	0.12	0.141	-0.18	-0.17	-0.45	-0.22	-0.2	0.4063	0.3898	0.4294	-0.198	-0.072	0.1669	-0.228	0.0461	-0.185	-0.3	0.111	0.338	-0.27	-0.23	0.211	-0.29	-0.01	-0.02	0.609924	0.009825	-0.125392598	0.135218012
190	A0A0C4DGE	SCOC	short coiled-coil protein	60592	ENSG00000153130	2	-0.07	0.224	-0.12				0.087	0.071	0.174	-0.043	-0.384	0.1751				0.0839	0.3229	0.073	-0.24	-0.45	-0.08				0.091	0.104	0.159	0.649424	0.132146	0.023375231	0.108770405
2531	Q15746	MYLK	myosin light chain kinase	4638	ENSG00000065534	2	0.219	0.392	0.136	0.101	0.081	-0.38	-0.06	-0.08	0.11	0.3479	0.1509	0.1695	0.1622	-0.185	0.1502	0.0235	0.1576	0.136	-0.2	-1.03	-0.14	-0.44	-0.26	0.065	-0.21	-0.07	0.266	0.126099	0.281633	-0.065982907	0.347615549
2769	Q7L7X3	TAOK1	TAO kinase 1	57551	ENSG00000160551	14	0.034	0.433	0.024	0.188	-0.07	-0.19	-0.21	-0.13	-0.13	-0.143	-0.049	0.1795	-0.077	-0.079	0.2197	0.3431	-0.038	0.2359	-0.11	0.026	-0.31	-0.4	-0.34	0.165	0.209	0.003	0.058	0.499364	0.072059	-0.07101405	0.143072566
443	Q8NBY1	STK26	serine/threonine kinase 26	51765	ENSG00000134602	13	-0.01	0.143	-0.1	0.184	-0.43	-0.28	0.121	-0.03	0.13	0.2408	0.1238	0.0779	0.4695	0.1041	-0.055	0.2268	0.0787	0.0997	-0.4	0.168	0.083	-0.27	-0.22	-0	0.239	0.003	-0.01	0.205085	0.015332	-0.182245345	0.197576943
3175	Q96PD5	PGLYRP2	peptidoglycan recognition protein 2	114770	ENSG00000161031	3	-0.21	-0.5	-0.24	0.081	-0.04	-0.14	-0.15	0.152	-0.1	-0.237	-0.025	-0.145	-0.202	0.0222	-0.324	0.3902	-0.441	-0.225	-0.8	-0.63	-0.21	-0.36	-0.3	0.065	0.345	0.186	-0.3	0.805331	0.094167	0.003128181	0.091038792
3492	Q9NRX5	SERINC1	serine incorporator 1	57515	ENSG00000111897	5	-0.19	0.109	0.104	-0.16	-0.32	-0.23	-0.32	-0.34	-0.25	0.5142	0.1359	-0.253	0.274	0.0227	-0.076	-0.094	0.1725	-0.2	-0.39	-1.16	-0.5	-0.19	-0.12	0.069	-0.41	-0.2	#####	0.132313	0.143953	-0.233320271	0.377273631
2565	Q17RN3	FAM98C	family with sequence similarity 98 member C	147965	ENSG00000130244	4	-0.18	-0.46	-0.27	0.102	0.007	0.07	0.648	0.15	0.42	0.0189	-0.083	-0.065	0.1596	0.0562	0.1973	0.6351	-0.019	0.3352	-0.18	-0.31	-0.02	-0.49	-0.37	0.252	0.318	0.127	0.565	0.721383	0.065159	-0.083327184	0.148486274
2995	Q8TEV9	SMCR8	Smith-Magenis syndrome chromosome regi	140775	ENSG00000283741	6				-0.1	0.142	-0.35	1.087	0.133	0.185				0.0859	-0.064	-0.108	-0.071	0.3203	0.3762				-0.21	-0.21	0.027	-0.03	-0.22	0.076	0.486543	0.276542	0.093367218	0.183174417
2697	Q6NZY7	CDC42EP:	CDC42 effector protein 5	148170	ENSG00000167617	5	0.007	0.241	-0.37	0.256	-0.21	-0.18	0.172	0.311	0.482	-0.16	0.0249	0.0572	0.0992	0.0723	0.1796	0.6386	0.0111	0.2965	-0.53	-0.33	-0.3	-0.47	-0.43	-0.09	0.217	0.334	0.322	0.270585	0.218233	-0.057663933	0.275897318
284	A0A1C7CYX	DPYSL2	dihydropyrimidinase like 2	1808	ENSG00000092964	6	0.139	0.312	0.017				0.357	0.091	0.002	0.2023	-0.069	0.1005				0.5265	0.3753	0.1688	-0.17	-1	-0.09				0.629	0.197	0.1	0.532401	0.209077	-0.064311529	0.273388855
2908	Q8N6G6	ADAMTSL	ADAMTS like 1	92949	ENSG00000178031	3	-0.29	-0.08	-0.56				0.398	0.605	0.321	0.4845	-0.699	-0.009				0.4258	-0.337	0.4705	0.341	0.116	0.091				0.467	0.154	0.286	0.762425	-0.17734	0.009063265	-0.186404857
3514	Q9NUQ9	FAM49B	family with sequence similarity 49 member E	51571	ENSG00000153310	10	-0.11	-0.02	-0.04	0.011	-0.26	-0.21	0.268	0.124	0.378	0.1668	-0.192	0.0446	-0.128	0.011	-0.039	0.3335	0.1981	0.1402	-0.23	-0.52	-0.12	-0.03	-0.38	-0.17	-0.02	0.118	0.361	0.404178	0.125931	-0.04348704	0.169417971
960	K7ELV2	SEH1L	SEH1 like nucleoporin	81929	ENSG00000085415	3	0.072	0.643	0.07	-0.08	0.032	-0.3	0.252	0.332	0.199	0.184	0.101	0.2436	0.104	-0.485	-0.175	0.1864	0.0523	0.2469	0.051	-0.97	-0.06	0.086	-0.35	-0.04	0.345	0.159	0.417	0.63863	0.175223	0.084325679	0.09089763
410	B1AL00	BMP7	bone morphogenetic protein 7	655	ENSG00000101144	4	-0.12	-0.04	-0.52	-0.46	-0.11	-0.13	-0.33	-0.21	0.226	-0.132	-0.163	-0.16	-0.054	-0.305	0.0777	0.0082	-0.07	-0.196	0.815	1.033	0.885	0.257	-0.18	0.186	-0.21	0.28	-0.15	0.063364	-0.5138	-0.078750964	-0.435048103

2901	Q8N4F0	BPIFB2	BPI fold containing family B member 2	80341	ENSG00000078898	5				-1.19	-0.2	-0.94	-0.24	0.502	-0.26				0.8738	0.3214	-0.924	-0.028	0.4304	-0.616				1.176	1.451	-0.88	1.12	-0.53	-0.51	0.501433	-0.69166	-0.39665972	-0.295004268
3427	Q9HAS3	SLC28A3	solute carrier family 28 member 3	64078	ENSG00000197506	7	0.689	0.945	0.23	-0.07	0.268	-0.03	0.32	0.089	0.532	0.6404	0.5286	0.0073	0.1431	0.1827	0.0616	0.2597	0.4003	0.3112	0.435	0.435	0.123	-0.19	0.188	0.087	0.581	0.451	0.416	0.936097	0.049136	0.048632452	0.000503142
3069	Q93077	HIST1H2A	histone cluster 1 H2A family member c;H2A	8334	ENSG00000180573	7	-2.63	-2.15	-2.28	-0.3	-0.67	0.651	-1.24	-0.98	-1.59		-1.221	-2.341	-0.49	-0.399	-0.735	-0.176	-1.502	-1.852	-1.83	-1.7	3.107	1.875	-0.38	0.594	-1.4	-1.85	-2.11	0.537762	-0.83437	-0.155072218	-0.679295855
1963	P48029	SLC6A8	solute carrier family 6 member 8	6535	ENSG00000130821	3	-0.27	0.107	-0.2				5E-04	0.263	-0.08	-0.043	-0.063	0.1424				0.2128	-0.065	0.4028	0.103	-0.01	-0.05				-0.01	0.091	0.466	0.575594	-0.1284	-0.128037916	-0.000364701
3228	Q99805	TM9SF2	transmembrane 9 superfamily member 2	9375	ENSG00000125304	4				0.155	0.114	-0.33	-0.07	0.063	0.139				-0.004	-0.168	-0.148	0.1323	0.2531	0.2437				-0.51	-0.32	-0.06	0.068	0.206	0.022	0.613559	0.110896	-0.039295744	0.150191601
782	G3XA98	FBLN5	fibulin 5	10516	ENSG00000140092	3	-0.36	-0.41	-0.12	-0.09	0.182	0.16	-0.15	0.435	-0.17	0.4034	0.3826	0.2568	-0.399	0.28	-0.186	-0.099	-0.384	-0.027	-0.18	0.69	0.539	0.496	0.04	-0.02	0.748	-0.18	-0.52	0.52095	-0.23758	-0.083698352	-0.153883695
3791	Q9Y6D6	ARFGEF1	ADP ribosylation factor guanine nucleotide e	10565	ENSG00000066777	28	0.175	0.17	-0	0.022	-0.09	0.066	-0.17	-0.1	-0.16	0.409	0.049	0.3882	0.007	0.281	-0.16	0.1087	-0.095	-0.14	-0.04	-0.44	-0.28	-0.41	0.026	-0.14	0.166	-0.06	0.127	0.227941	0.108451	-0.102118929	0.210570253
450	B5MBZ0	EML4	EMAP like 4;echinoderm microtubule assoc	27436	ENSG00000143924	3	-0.24	0.017	-0.05				0.026	1.177	0.097	0.0771	-0.199	0.0564				-0.119	-0.269	0.028	-0.19	0.046	0.059				0.73	0.212	0.344	0.524799	-0.0281	0.242130037	-0.27023004
1473	P07602	PSAP	prosaposin	5660	ENSG00000197746	40	-0.83	-0.49	-0.63	-0.82	-0.11	-0.32	-0.15	0.074	0.417	0.1891	-0.356	-0.39	-0.612	-0.208	-0.748	0.289	0.1619	0.374	-0.7	-0.35	-0.25	-0.2	0.537	-0.04	0.895	0.07	-0.54	0.618833	-0.25303	-0.172640305	-0.080391411
112	A0A087X1V	OVCH2	ovochymase 2 (gene/pseudogene)	341277	ENSG00000183378	8	-0.13	-0.88	0.866	1.044	0.488	-0.27	0.286	0.907	0.115	-0.456	-0.66	0.7564	-0.417	-0.314	-0.99	-0.29	-0.717	-0.597	0.205	0.142	-0.26	0.397	-0.33	-0.72	-0.47	-0.18	-0.51	0.138709	0.463316	0.67973521	-0.216418806
23	A0A087WSY	CPB2	carboxypeptidase B2	1361	ENSG00000080618	2	0.144	-0.85	-0.36	-0.26	-0.3	-0.27	0.014	0.212	-0.12	-0.094	0.0787	-0.24	-0.477	-0.416	-0.077	0.2436	-0.138	-0.153	-0.5	0.109	0.355	0.253	-0.44	-0.24	0.337	0.007	-0.17	0.613649	-0.16668	-0.056862656	-0.109818036
248	E3W994	CLASP2	cytoplasmic linker associated protein 2	23122	ENSG00000163539	9	0.006	0.244	-0.06	-0.14	-0.13	-0.06	0.058	-0.07	0.259	0.2324	-0.051	0.2023	-0.092	-0.117	0.1116	0.159	-0.015	-0.042	-0.07	-0.46	-0.06	-0.17	-0.2	-0.08	0.213	0.126	0.197	0.595593	0.067668	-0.031843511	0.099511214
636	E9PDQ8	SUCLG2	succinate-CoA ligase GDP-forming beta sul	8801	ENSG00000172340	2	-0.09	0.31	-0.34	0.125	0.218	-0.41	0.051	0.222	0.084	-0.336	0.0916	0.1058	0.1009	-0.033	-0.078	0.2796	0.3091	0.2344	-0.26	-0.25	-0.25	-0.97	-0.55	-0.18	-0.08	0.047	0.441	0.219324	0.248138	-0.056114001	0.304252434
3661	Q9UM22	EPDR1	ependymin related 1	54749	ENSG00000086289	4	0.032	0.516	0.079	-0.29	-0.24	-0.7	-0.41	0.14	-0.27	0.3462	-0.15	0.0936	-0.16	0.0838	-0.376	0.0496	-0.078	-0.078	0.03	-0.17	0.022	-0.48	0.019	-0.32	0.25	0.42	-0.3	0.846405	-0.0677	-0.096219975	0.028518694
2988	Q8TDZ2	MICAL1	microtubule associated monooxygenase, cal	64780	ENSG00000135596	7	-0.14	0.25	-0.2	0.157	-0.42	-0.13	-0.06	0.211	-0.09	0.2205	-0.041	-0.112	0.0783	0.071	-0.498	-0.132	-0.7	-0.025	-0.1	-0.2	0.227	-0.49	0.059	0.103	0.381	-0.45	-0.27	0.884178	0.036331	0.079997295	-0.043666114
188	X6REF7	SFTPA2	surfactant protein A2	729238	ENSG00000185303	3	-0.71	-0.94	0.325	-0.53	-1.01	0.438	-0.14	-0.24	0.615	-0.343	-0.131	-0.447	0.1048	-0.273	-0.811	0.1166	0.9733	0.0662	1.539	-0.41	0.094	-0.29	0.068	0.741	0.341	-0.28	-0.11	0.451068	-0.4311	-0.16052343	-0.270572969
2686	Q6IAA8	LAMTOR1	late endosomal/lysosomal adaptor, MAPK ar	55004	ENSG00000149357	4	0.109	0.461	0.081	0.219	0.041	-0.18	0.127	-0.23	0.154	0.2712	0.2953	-0.083	0.0652	-0.099	0.5009	0.4384	0.424	0.3998	-0.17	-0.48	-0.11	-0.63	-0.45	0.134	-0.2	0.107	0.38	0.101617	0.244651	-0.158551836	0.403202676
3506	Q9NSY2	STARD5	StAR related lipid transfer domain containing	80765	ENSG00000172345	2	-0.37	0.322	-0.04	-0.14	-0.03	-0.18	-0.14	-0.02	0.32	-0.116	-0.184	-0.2	0.1082	-0.379	0.1082	-0.146	-0.024	0.2163	-0.35	-1.1	-0.48	-0.73	-0.47	0.052	-0.52	-0.35	0.248	0.119562	0.380807	0.038086469	0.342720149
75	A0A087WYF	TPD52L2	TPD52 like 2	7165	ENSG00000101150	7	-0.11	0.028	-0.2	-0.02	0.144	-0.26	-0.23	-0.08	0.031	0.5506	-0.043	0.1406	0.0332	-0.048	-0.175	-0.024	0.0429	-0.263	-0.27	-0.18	-0.26	-0.07	0.029	-0.03	0.082	0.066	-0.08	0.522544	0.000787	-0.101036412	0.101823524
3559	Q9P0R6	GSKIP	GSK3B interacting protein	51527	ENSG00000100744	2	0.363	0.391	0.105	0.132	0.173	-0.22	0.522	0.369	0.466	0.5757	0.3373	0.3182	0.548	0.2202	0.2429	0.5438	0.1801	0.3438	0.018	-1.13	0.205	0.073	0.009	0.428	0.266	0.235	0.66	0.361986	0.171257	-0.112164466	0.283421323
331	P62263	RPS14	ribosomal protein S14	6208	ENSG00000164587	5	0.102	-0.1	0.029	-0.08	0.06	-0.26	0.337	-0.05	0.469	-0.061	0.0802	-0.033	-0.138	-0.041	0.1078	0.0258	0.14	0.1962	-0.01	-0.44	0.127	-0.34	-0.14	0.021	0.272	-0.04	0.366	0.818837	0.076415	0.025633141	0.050781544
2434	Q13885	TUBB2A	tubulin beta 2A class IIa	7280	ENSG00000137267	22	0.139	0.109	-0.1	0.11	-0.08	-0.31	-0.29	0.027	0.195	0.3941	-0.269	0.1858	0.2148	0.023	-0.004	-0.27	0.134	-0.325	-0.05	-0.62	-0.62	-0.59	-0.32	-0.07	0.122	0.094	0.073	0.297135	0.19857	-0.031584575	0.23015497
3023	Q8WXG6	MADD	MAP kinase activating death domain	8567	ENSG00000110514	50							0.486	0.499	0.786							0.8137	0.0212	0.2973							0.328	0.409	0.576	0.726928	0.152923	0.213164151	-0.06024159
2706	Q6P9A2	GALNT18	polypeptide N-acetylgalactosaminyltransfera	374378	ENSG00000110328	6	0.095	-0.7	-0.14	-0.64	0.998	-0.36	-0.13	0.196	-0.19	0.0468	0.5318	0.1936	-0.024	-0.152	0.1003	0.1477	0.0393	-0.072	-1.07	0.577	0.548	0.403	-0.09	0.441	0.576	0.193	-0.01	0.565801	-0.27011	-0.18628709	-0.083826713
2824	Q86X83	COMMD2	COMM domain containing 2	51122	ENSG00000114744	2	0.092	0.114	0.17				0.08	0.173	0.192	0.3837	0.2216	0.09				0.1049	0.1867	0.2715	-0.02	-0.79	0.01				-0.11	0.034	0.042	0.124675	0.277244	-0.07285853	0.350102936
954	K7EKI0	EVPL	envoplakin	2125	ENSG00000167880	3	-0.23	-0.31	-0.22	0.017	0.336	-0.68	0.255	0.258	0.443	-0.183	0.0262	-0.023	-0.268	-0.128	0.1837	1.5608	0.1478	0.4511	-0.31	-0.48	0.145	-1.13	-0.71	-0.3	0.261	0.384	0.182	0.36624	0.203702	-0.21124775	0.414949742
2563	Q16864	ATP6V1F	ATPase H+ transporting V1 subunit F	9296	ENSG00000128524	4	0.128	0.324	0.024	-0.3	0.015	-0.16	0.006	-0.2	0.287	0.0508	0.0818	0.0395	0.015	0.0087	0.0094	0.1331	0.1932	0.0189	-0.14	-0.92	-0.19	-0.49	-0.27	0.097	-0.15	-0.06	0.073	0.131819	0.24073	-0.048046231	0.288776401
777	G3V2U7	ACYP1	acylphosphatase 1	97	ENSG00000119640	2	0.072	-0.17	0.014				0.189	0.29	0.072	-0.324	-0.318	0.1703				0.0866	-0.037	-0.007	-0.35	-0.65	-0.05				0.212	-0.09	-0.08	0.356912	0.246104	0.148541901	0.097562367
3426	Q9HAD4	WDR41	WD repeat domain 41	55255	ENSG00000164253	4	0.12	0.457	0.194	0.15	-0.04	-0.6	0.043	0.145	0.13	0.4811	0.2688	-0.163	0.0279	-0.017	-0.186	-0.017	0.1002	0.008	0.106	-0.13	0.068	-0.48	-0.56	0.075	-0.02	0.088	0.139	0.559594	0.145825	0.010037258	0.135788096
2791	Q7Z6J0	SH3RF1	SH3 domain containing ring finger 1	57630	ENSG00000154447	4	-0.03	0.517	-0.02	0.137	0.383	-0.41	-0.33	0.069	-0.1	0.0449	0.3453	0.3014	0.0986	0.124	0.258	-0.393	-0.014	-0.135	-0.05	0.172	-0.04	-0.55	-0.32	0.137	0.071	-0.03	-0.05	0.618597	0.098217	-0.046070216	0.144286723
2557	Q16568	CARTPT	CART prepropeptide	9607	ENSG00000164326	1	-0.86	2.633	-0.07	-0.57	-0.96	-0.09	-1.13	0.477	0.272	0.9781	-1.57	0.2754	-0.033	0.6943	-1.706	-0.071	-0.546	-0.118	-0.71	-0.85	-0.85	-0.45	-0.3	-0.99	-0.73	-0.18	-1.21	0.427568	0.665212	0.201036178	0.464175407
2721	Q6UWH4	GASK1B;F	golgi associated kinase 1B;family with seque	51313	ENSG00000164125	4	0.008	-0.62	-0.03	-0.11	-0.47	-0.12	0.061	0.35	0.04	-0.186	-0.156	0.2661	-0.153	0.0318	0.0212	0.5003	-0.029	-0.114	0.233	0.384	0.127	-0.02	-0.2	-0.23	0.416	-0.01	-0.28	0.603021	-0.14472	-0.117694431	-0.027023452
1096	O15439	ABCC4	ATP binding cassette subfamily C member	10257	ENSG00000125257	2							0.722	0.605	0.543							0.4183	-0.259	0.0639							2.488	0.264	0.498	0.468742	-0.45973	0.549081186	-1.008809894
3754	Q9Y4C2	TCAF1	TRPM8 channel associated factor 1	9747	ENSG00000198420	8	-0.35	0.857	-0.27	0.004	0.018	-0.2	-0.16	-0.04	-0.08	-0.156	-0.431	-0.105	0.0747	0.1271	-0.147	0.1573	0.1049	-0.051	0.039	-0.39	-0.15	-0.37	-0.19	0.08	-0.06	-0.13	-0.02	0.748615	0.106213	0.022614762	0.083597793
3540	Q9NYU1	UGGT2	UDP-glucose glycoprotein glucosyltransfera	55757	ENSG00000102595	5	-0.01	0.005	-0.09				-0.25	-0.01	-0.22	0.2088	0.0504	-0.075				0.0107	0.0497	-0.031	0.065	0.418	0.171				0.247	-0.14	-0.12	0.233689	-0.2027	-0.131824783	-0.070878095
482	C9IZ46	SHISA5	shisa family member 5	51246	ENSG00000164054	2	-0.25	-0.49	-0.02	0.413	0.054	0.173	-0.33	-0.11	-0.18	0.6118	-0.093	0.0831	0.1768	0.3362	-0.343	0.1558	-0.379	-0.261	-0.32	-0.66	0.918	0.043	0.285	0.272	0.109	0.294	-0.69	0.853897	-0.10914	-0.112862908	0.003726326
2928	Q8NCC3	PLA2G15	phospholipase A2 group XV	23659	ENSG00000103066	3	0.132	-0.23	0.742				0.444	0.614	0.725	-0.218	-0.039	-0.68				0.591	0.222	0.7612	1.51	-0.33	0.645				0.506	0.361	0.083	0.603479	-0.05833	0.29748457	-0.355817891
2906	Q8N687	DEFB125	defensin beta 125	245938	ENSG00000178591	1	-0.2	-0.54	0.584	0.587	-0.36	-0.22	-0.11	1.267	0.988	-0.644	0.148	0.293	0.8269	-1.431	0.2452	0.0677	0.0046	-1.07	-0.51	-1.49	-0.73	-0.66	-0.16	-0.88	-0.46	-0.57	-1.54	0.092122	0.999997	0.396180392	0.603816734
50	A0A087WVS	MOB2	MOB kinase activator 2	81532	ENSG00000182208	3	0.065	0.428	0.055	-0.04	-0.24	-0.65	0.062	0.028	0.149	0.1393	-0.038	0.3509	0.0593	-0.385	-0.163	0.1705	0.1448	0.1824	-0.42	-1.25	-0.48	-0.85	-0.47	0.048	-0.06	0.104	0.139	0.160997	0.344181	-0.067005328	0.411185991
2414	Q13501	SQSTM1;A	sequestosome 1	8878	ENSG00000284099	4	-0.05	0.079	-0.09				0.745	0.68	0.633	0.0333	-0.265	-0.074				0.8201	-0.207	0.5985	-0.09	-0.01	0.008				0.355	0.587	0.091	0.745362	0.178206	0.182971967	-0.004765548
74	A0A087WYN	DHX29	DExH-box helicase 29	54505	ENSG00000067248	9	-0.21	-0.25	-0.18	-0.37	-0.39	-0.4	-0.12	0.364	0.218	0.2436	-0.101	0.2806	-0.324	-0.293	-0.065	0.1509	0.0596	0.2715	-0.48	-0.36	0.219	-0.46	-0.1	0.106	0.074	0.255	0.154	0.559048	-0.07988	-0.171294017	0.091418238
2268	P83436	COG7	component of oligomeric golgi complex 7	91949	ENSG00000168434	8	0.292	0.212	0.032	0.11	0.328	-0.16	-0.03	0.057	0.375	0.1868	0.1187	0.2004	-0.02	0.0809	0.0635	0.0882	0.0708	0.2638	0.044	-0.68	-0.08	-0.16	-0.14	0.303	-0.18	0.193	0.331	0.32863	0.176294	0.017905373	0.158389043
853	H3BQK0	DDX19B	DEAD-box helicase 19B	11269	ENSG00000157349	19				0.01	0.043	-0.56	0.081	-0.01	0.065				0.0914	-0.124	0.2237	-0.023	0.3631	0.2377				-0.45	-0.33	0.118	-0.02	-0.03	0.045	0.324428	0.047782	-0.19126396	0.239045747
850	H3BPK3	HAGH	hydroxyacylglutathione hydrolase	3029	ENSG00000063854	8	-0.32	-0.1	0.096	0.236	0.234	-0.3	0.019	0.064	0.214	0.512	0.1047	0.0161	0.1374	-0.205	-0.127	0.1788	-0.221	-0.219	-0.18	-0.32	0.102	-0.44	-0.29	0.101	0.13	0.199	-0.13	0.642118	0.107633	-0.00393232	0.111565731
1198	O75326	SEMA7A	semaphorin 7A (John Milton Hagen blood gr	8482	ENSG00000138623	5	-0.39	0.353	-0.13	-0.19	0.316	0.109	0.885	0.091	0.228	0.223	0.4223	0.0024	0.0163	0.0988	0.0044	-0.262	-0.634	0.0719	-0.06	-0.21	0.038	0.221	0.063	0.048	0.369	0.402	-0.11	0.697209	0.057397	0.147924157	-0.090527442
666	E9PKC5	AMPD3	adenosine monophosphate deaminase 3	272	ENSG00000133805	2							0.457	0.287	0.291							0.3273	-0.221	0.1601							0.537	0.19	0.116	0.52707	0.063904	0.256206522	-0.192302711
2524	Q15438	CYTH1	cytohesin 1	9267	ENSG00000108669	3							0.202	0.153	0.24							0.4655	-0.117	0.0943							0.209	-0.01	0.254	0.9583	0.047303	0.05062444	-0.003320999
2854	Q8IWV8	UBR2	ubiquitin protein ligase E3 component n-rec	23304	ENSG00000024048	7	-0.08	-0.08	-0.04				0.102	0.084	0.142	0.075	-0.075	0.1267				0.1277	0.0091	0.1662	-0.08	-0.41	-0.2				0.224	0.078	0.164	0.637376	0.058917	-0.050682693	0.109600085
163	A0A0A0MTF	RNF213;A	ring finger protein 213	57674	ENSG00000173821	13	0.048	-0.13	-0.2	-0.27	-0.27	0.052	-0.81	0.03	-0.07	0.0867	0.0342	0.2339	-0.037	-0.029	-0.087	-0.71	-0.776	-0.375	-0.09	-0.02	0.218	-0.08	0.081	0.171	-0.38	-0.41	-0.53	0.911915	-0.0626	0.006020869	-0.068616167
3501	Q9NSA3	CTNNBIP1	catenin beta interacting protein 1	56998	ENSG00000178585	3	0.031	0.238	0.035	-0.1	0.033	-0.64	0.189	-0.06	0.11	-0.03	-0.16	0.1574	0.0317	-0.057	0.0063	0.3121	0.3693	0.0616	-0.04	-1.14	-0.03	-0.81	-0.28	-0	-0.03	0.179	0.315	0.356568	0.185351	-0.095018479	0.28036938
700	F5GX88	ENG	endoglin	2022	ENSG00000106991	3	-0.5	-0.22	-0	-0.07	-0.12	0.389	-0.5	-0.23	-0.51	-0.272	0.2728	-0.428	0.022	0.0257	0.0435	-0.515	0.1293	-0.527	-0.24	1.16	0.451	0.006	0.09	0.13	0.028	-0.25	-0.67	0.450465	-0.27409	-0.056733509	-0.217356861
582	G5EA19	TATDN1	TatD DNase domain containing 1	83940	ENSG00000147687	5	-0.08	0.152	-0.15	0.088	0.019	-0.26	0.075	0.192	0.25	0.0221	-0.149	0.2373	0.2923	-0.015	-0.367	0.3571	0.2117	-0.042	-0.34	-1.33	-0.4	-0.43	-0.28	-0.03	0.077	0.336	0.301	0.296806	0.264519	-0.028430042	0.292949299
1236	O75891	ALDH1L1	aldehyde dehydrogenase 1 family member L	10840	ENSG00000144908	3	0.037	0.328	-0.45	-0.11	0.097	0.265	0.589	0.424	-0.21	0.027	0.341	0.1324	-0.322	0.334	-0.051	-0.304	0.4779	0.261	-0.01	0.028	0.267	0.149	0.209	-0.04	-0.05	-0.35	0.037	0.854085	0.082077	0.00793082	0.074146487
2534	Q15773	MLF2	myeloid leukemia factor 2	8079	ENSG00000089693	7	0.435	-0.22	-0.14	-0.12	-0	-0.16	0.286	0.127	0.265	-0.048	0.6015	0.3378	-0.057	0.0354	0.5042	0.1705	0.0167	0.1625	-0.28	-0.3	-0.3	-0.24	-0.06	0.373	0.189	0.169	0.32	0.388615	0.066674	-0.13885442	0.205527929
750	F8VYE8	PPP1CC	protein phosphatase 1 catalytic subunit gam	5501	ENSG00000186298	16	0.183	0.269	-0.04	0.379	0.717	-0.29	0.023	0.358	0.146	-0.238	-0.07	-0.049	0.2055	-0.081	0.3786	0.1	0.1141	0.1754	-0.19	-0.22	-0.33	-0.02	-0.22	-0.16	-0.16	0.024	0.242	0.132031	0.308208	0.134366576	0.173840948
3192	Q96SA4	SERINC2	serine incorporator 2	347735	ENSG00000168528	2	-0.52	0.039	0.021	-0.34	0.652	-0.45	-0.76	-0.5	-1.11	-0.452	-0.004	-0.121	0.2217	-0.004	-0.551	-0.092	-0.294	-0.236	-0.2	0.227	-0.18	-0.12	0.191	0.231	-0.5	-0.63	-0.27	0.64216	-0.19056	-0.159955572	-0.030603742
788	I3L4X3	NFKBIB	NFKB inhibitor beta	4793	ENSG00000104825	2				0.237	-0.04	-0.05	0.852	0.773	0.938				0.2421	-0.289	0.1298	0.3467	-0.081	0.5043				0.281	-0.36	0.332	0.115	0.362	0.329	0.432995	0.275465	0.310036351	-0.034571746
947	K7EJ46	SMIM22	small integral membrane protein 22	440335	ENSG00000267795	2	-0.22	0.303	0.221	0.047	0.284	0.131	-0.32	-0.74	0.231	0.089	0.1921	0.0482	0.2011	-0.248	-0.443	0.4219	0.1696	0.276	-0.17	0.258	-0.02	0.327	0.023	0.2	-0.53	0.032	-0.47	0.799174	0.031819	-0.08523503	0.117054186
1161	O60512	B4GALT3	beta-1,4-galactosyltransferase 3	8703	ENSG00000158850	4	0.232	0.349	0.187				-0.36	0.129	0.186	-0.096	0.1423	0.0066				0.3298	0.451	0.3888	-0.37	0.208	0.102				0.057	0.084	0.042	0.535469	0.100093	-0.083484134	0.183577501
396	A8MUM1	EIPR1	EARP complex and GARP complex interact	7260	ENSG00000032389	4	-0.64	-0.6	0.302				-0.02	0.067	0.216	0.2922	-0.968	-0.859				0.0064	0.161	0.11	-0.6	-0.45	-0.83				0.215	0.081	0.095	0.918644	0.134166	0.09618769	0.03797785
382	P26599	PTBP1	polypyrimidine tract binding protein 1	5725	ENSG00000011304	4	-0.02	0.146	-0.02	0.035	0.123	-0.32	-0.02	-0.25	0.03	0.1964	-0.063	-0.049	-0.071	-0.089	0.1301	-0.075	-0.147	-0.196	0.073	0.592	0.142	-0.5	-0.39	-0.05	-0.01	-0.21	-0.09	0.991476	0.015807	0.007028225	0.008779207
2571	Q2PPJ7	RALGAPA	Ral GTPase activating protein catalytic alph	57186	ENSG00000188559	10	0.164	0.27	-0.17	-0.02	0.11	-0.34	-0.06	-0.12	0.125	0.0702	-0.044	0.0655	0.2262	-0.184	0.2178	0.0762	0.0728	0.2691	-0.3	-1.23	-0.38	-0.31	-0.13	0.079	0.155	0.208	0.234	0.317353	0.181917	-0.089013971	0.270931157
304	Q9UK22	FBXO2	F-box protein 2	26232	ENSG00000116661	2	0.104	-0.3	0.398	-0.21	0.26	-0.37	0.181	0.992	0.148	-0.013	-0.011	0.5158	-0.197	-0.069	-0.33	0.1618	0.362	0.4522	-0.17	-0.2	-0.08	0.407	-0.29	0.103	1.587	0.87	0.173	0.804039	-0.1323	0.036710654	-0.169008504
320	A0A2R8Y56	RELCH	RAB11 binding and LisH domain, coiled-coi	57614	ENSG00000134444	3	-0.01	-0.03	-0.15	-0.18	-0.04	-0.13	-0.09	0.104	0.307	-0.114	-0.096	0.0382	-0.094	0.3732	0.2153	0.0087	0.218	0.0354	-0.13	-0.39	-0.15	-0.49	-0.1	-0.13	0.317	0.183	0.3	0.561729	0.04169	-0.089404951	0.131094964
2917	Q8N9V3	WDSUB1	WD repeat, sterile alpha motif and U-box do	151525	ENSG00000196151	3	0.054	-0.26	-0.2				-0.01	0.129	0.308	0.0484	-0.071	-0.093				0.2605	0.2405	0.1991	-0.36	-1.23	-0.47				0.044	-0.02	0.334	0.337699	0.285107	-0.094275019	0.379382442
423	B1APY4	ROR2	receptor tyrosine kinase like orphan receptor	4920	ENSG00000169071	3	0.113	-0.03	-0.11				0.082	0.164	0.201	-0.162	0.1071	-0.769				0.3784	0.4628	0.3254	-0.45	-0.88	-0.51				0.075	0.187	0.482	0.627461	0.251603	0.012307952	0.239294843
1965	P48061	CXCL12	C-X-C motif chemokine ligand 12	6387	ENSG00000107562	3	0.307	0.402	-0.35	-0.29	0.647	-0.09	-0.39	0.063	-0.34	-0.074	0.5419	0.0507	0.3985	-0.501	0.2213	0.1228	0.3325	0.2357	-0.6	-0.69	0.613	-0.21	0.204	0.12	-0.23	0.261	-0.19	0.574304	0.075719	-0.15118235	0.226901209
2163	P61278	SST	somatostatin	6750	ENSG00000157005	3	-0.2	-0.14	-0.57	-1.58	-1	-2.22	-0.34	0.501	2.204	0.9454	0.2146	-0.076	-2.497	-0.135	-1.108	-2.041	-1.994	-2.407	0.358	0.172	-0.65	-2.51	-2.04	-1.41	0.423	-0.95	-1.81	0.621693	0.562863	0.638050499	-0.075187742
3009	Q8WUX9	CHMP7	charged multivesicular body protein 7	91782	ENSG00000147457	2				0.076	0.146	-0.38	0.736	0.206	0.246				0.0756	-0.179	0.0931	0.4524	0.1482	0.0551				-0.45	-0.34	-0.08	0.725	0.245	0.59	0.965747	0.055625	0.063721105	-0.008096052
140	A0A0A0MR1	FAM189A2	family with sequence similarity 189 member	9413	ENSG00000135063	3	-0.03	0.544	0.436	0.115	-0.09	-0.05	-0.35	-0.12	-0.33	0.2169	0.2516	-0.07	0.3771	0.306	0.2435	-0.256	0.0609	-0.346	-0.32	-0.1	0.063	-0.14	0.111	0.089	-0.64	-0.11	-0.03	0.435369	0.131994	-0.073738409	0.205732766
308	A0A2Q2TTZ	IGKV1D-3	immunoglobulin kappa variable 1D-33;immu	28896;2893	ENSG00000239975	2	0.094	-0.27	-0.21	-0.07	-0.37	-0.3	0.109	0.023	0.006	-0.282	0.0026	-0.158	0.034	0.0444	-0.042	-0.074	-0.221	0.0098	-0.09	-0.07	0.606	0.539	0.034	0.149	0.287	-0.04	0.12	0.102536	-0.27892	-0.033179016	-0.245737342
3232	Q99943	AGPAT1	1-acylglycerol-3-phosphate O-acyltransferas	10554	ENSG00000204310	2							0.274	0.227	0.122							-0.031	-0.201	0.1325							0.74	0.244	-0.15	0.561706	-0.0695	0.240986693	-0.310488031
3494	Q9NRY5	FAM114A2	family with sequence similarity 114 member	10827	ENSG00000055147	4	0.145	0.101	-0.06	-0.09	-0.12	-0.26	-0.09	0.04	0.132	-0.041	0.0075	0.0542	0.1341	0.0171	0.063	-0.036	0.1915	0.0602	-0.27	-1.08	-0.27	-0.46	-0.23	-0.03	0.199	0.067	0.111	0.20585	0.194423	-0.072873094	0.267296129
3573	Q9P2K8	EIF2AK4	eukaryotic translation initiation factor 2 alpha	440275	ENSG00000128829	5	0.165	0.244	0.028	-0.04	-0.19	0.151	0.061	0.1	0.27	0.0405	-0.108	0.2268	-0.111	-0.123	0.0301	0.1454	0.175	0.2273	0.025	-0.86	-0.13	-0.33	-0.09	0.169	0.086	0.343	0.286	0.578813	0.142808	0.032075881	0.11073165
3074	Q969P0	IGSF8	immunoglobulin superfamily member 8	93185	ENSG00000162729	9	-0.33	-0.12	0.992	0.218	-0.05	0.35	-0.6	0.246	-0.43	0.1114	-0.062	0.0375	0.0833	0.2259	0.0577	-0.413	-0.496	-0.683	-0.19	0.291	-0.09	0.218	-0.29	-0.09	0.1	-0.58	-0.52	0.710935	0.159846	0.157673459	0.002172227
3017	Q8WW43	APH1B	aph-1 homolog B, gamma-secretase subuni	83464	ENSG00000138613	2				0.306	0.093	-0.35	-0.13	-0.05	0.087				0.42	-0.014	0.0629	0.1466	0.1583	-0.004				-0.47	-0.31	0.104	0.032	-0.09	0.255	0.445705	0.073271	-0.135579054	0.208849874
2640	Q5T6J7	IDNK	IDNK gluconokinase;IDNK, gluconokinase	414328	ENSG00000148057	5	-0.12	0.2	0.17	0.093	-0.13	-0.28	-0.1	0.067	0.168	0.0187	0.2401	0.1179	0.0628	-0.002	0.2985	0.1704	0.276	0.1674	-0.13	-0.97	-0.1	-0.3	-0.19	0.221	0.181	0.274	0.196	0.303375	0.0965	-0.143493481	0.239993781
1084	O15231	ZNF185	zinc finger protein 185 with LIM domain	7739	ENSG00000147394	2	0.137	-0.03	0.43	-0.77	0.339	0.058	-0.12	0.132	-0.03	-0.707	0.233	0.1641	-0.134	-0.418	-0.473	-0.537	-0.174	0.0557	-0.83	-0.37	-0.22	-1.28	-0.78	-0.98	-0.13	-0.36	-0.53	0.059024	0.626157	0.236778625	0.389377941
2766	Q7L5D6	GET4	golgi to ER traffic protein 4;guided entry of t	51608	ENSG00000239857	5	-0.17	-0.35	-0.37	-0.06	-0.41	0.804	-0.23	0.015	0.097	-0.05	-0.013	-0.328	-0.02	-0.124	-0.328	0.1168	-0.34	-0.481	-0.1	0.181	-0.12	-0.38	0.344	-0.18	0.46	-0.21	-0.06	0.619357	-0.06676	0.100127388	-0.166889728

172	A0A0A6YYA	TMED7-TI	TMED7-TICAM2 readthrough	100302736	ENSG00000251201	4	-0.21	-0.04	-0.16	0.091	0.222	-0.14	-0.02	0.823	0.128	-0.601	-0.884	-0.278	-0.205	-0.114	-0.148	-0.005	-0.411	-0.182	-0.51	-0.1	-0.1	0.081	0.008	-0.03	0.345	0.294	0.09	0.119523	0.068582	0.390305806	-0.321724164
3045	X6RAB3	USP6NL	USP6 N-terminal like	9712	ENSG00000148429	2				0.594	0.624	-0.07	0.141	-0.02	0.17				0.2441	-0.382	-0.058	0.2939	0.1012	0.0569				0.652	-0.27	-0.06	0.03	0.913	1.269	0.472742	-0.1832	0.196874515	-0.380077825
1181	O60869	EDF1	endothelial differentiation related factor 1	8721	ENSG00000107223	4	0.368	0.531	0.073				0.054	-0.07	0.125	0.1742	0.2706	0.2088				0.2517	0.2962	0.2563	-0.04	-0.36	-0.11				0.079	0.037	0.213	0.156265	0.210653	-0.062249418	0.272902872
3701	Q9Y257	KCNK6	potassium two pore domain channel subfam	9424	ENSG00000099337	3	0.063	0.28	-0.02	0.235	-0.22	-0.25	-0.09	-0.76	-0.04	0.151	0.3372	-0.007	0.1134	-0.275	0.1531	0.1087	0.3324	0.2047	-0.36	-0.55	0.104	-0.52	-0.04	0.105	-0.09	-0.4	0.275	0.220017	0.077589	-0.211730911	0.28932015
1112	O43353	RIPK2	receptor interacting serine/threonine kinase	8767	ENSG00000104312	3	0.205	0.831	-0.33	0.031	0.073	-0.63	-0.33	-0	-0.25	0.0478	0.2139	0.3588	0.2251	-0.275	-0.109	-0.25	-0.348	-0.206	-0.23	-0.54	-0.51	-0.46	-0.55	-0.28	-0.15	-0.25	-0.03	0.218347	0.288463	-0.005659366	0.294122628
2866	Q8IYD1	GSPT2	G1 to S phase transition 2	23708	ENSG00000189369	18	-0.04	-0.12	-0.2				-0.01	-0.06	0.028	0.2536	-0.246	0.4159				-0.164	0.2438	0.1045	0.282	0.89	-0.45				-0.11	0.093	0.183	0.586992	-0.21613	-0.168356215	-0.047770948
3168	Q96N67	DOCK7	dedicator of cytokinesis 7	85440	ENSG00000116641	2	0.073	-0.37	-0.34	0.345	-0.09	-0.43	1.335	-0.28	-0	0.2121	0.1136	0.123	0.5187	-0.14	-0.492	-0.16	-0.68	0.6121	-0.31	-0.31	0.124	-0.09	0.6	-0.12	0.343	-0.35	1	0.947064	-0.07145	0.014784872	-0.086233208
793	G5E9W2	CHPF2	chondroitin polymerizing factor 2	54480	ENSG00000033100	6	0.093	-0	-0.28	-0.19	0.186	-0.16	-0.32	0.143	-0.06	0.5431	-0.156	-0.008	0.1075	0.0321	-0.115	-0.03	-0.129	-0.2	0.143	0.78	0.068	-0.11	-0.02	-0.12	0.158	-0.08	-0.12	0.591417	-0.14223	-0.070207542	-0.072019409
460	B7Z524	HBS1L	HBS1 like translational GTPase	10767	ENSG00000112339	4	0.183	0.148	-0.12	0.033	-0.13	-0.07	0.013	0.157	0.09	0.4378	0.2358	0.1831	-0.123	-0.102	-0.054	0.0319	0.1332	0.1965	-0.33	-0.37	0.042	0.552	-0.15	0.158	0.286	-0.09	0.193	0.803895	0.000832	-0.071256545	0.072089043
3096	Q96BM9	ARL8A	ADP ribosylation factor like GTPase 8A	127829	ENSG00000143862	4							0.033	0.035	0.326							0.0118	0.2625	0.0233							-0.13	0.112	0.536	0.949712	-0.04171	0.032263567	-0.073975561
1952	P46937	YAP1	Yes associated protein 1	10413	ENSG00000137693	2							-0.32	-0.41	-0.22							-0.228	-0.322	-0.427							-0.16	-0.49	-0.43	0.940761	0.043412	0.011594966	0.031817252
485	C9J164	RAPH1	Ras association (RalGDS/AF-6) and plecks	65059	ENSG00000173166	3							0.44	0.37	0.472							0.9738	-0.047	0.4985							0.547	-0	-0.17	0.62466	0.302177	-0.047338256	0.349515179
2282	Q00653	NFKB2	nuclear factor kappa B subunit 2	4791	ENSG00000077150	3	-0.08	0.085	-0.11				-0.25	0.015	0.105	0.1134	0.0791	-0.035				0.1836	0.1396	-0.02	-0.28	-0.59	-0.05				0.438	0.18	0.137	0.745201	-0.01166	-0.116681129	0.105023628
799	H0Y3Q9	FAM214A	family with sequence similarity 214 member	56204	ENSG00000047346	1	0.237	0.18	0.132	-0.07	-0.04	-0.13	-0.83	-0.64	-0.45	0.3476	0.2497	-0.069	0.1798	0.0535	0.3715	-0.712	0.1872	-0.421	-0.08	-2.14	-0.43	-0.81	-0.12	0.382	-0.48	-0.18	-0.21	0.317837	0.272626	-0.199650103	0.47227639
2267	P83105	HTRA4	HtrA serine peptidase 4	203100	ENSG00000169495	4	-0.67	-0.38	0.533	-0.91	-1.03	-0.5	0.01	-0.43	-0.25	-0.298	0.1355	-0.354	-0.878	-0.956	-0.164	-0.16	-0.018	-0.285	-0.5	-0.03	0.221	-0.45	-0.48	0.114	0.293	0.287	-0.28	0.398935	-0.31176	-0.073036037	-0.238720505
3664	Q9UMX0	UBQLN1	ubiquilin 1	29979	ENSG00000135018	5	-0.17	-0.01	-0.26	-0.08	-0.05	-0.31	-0.08	-0.15	-0.07	-0.124	0.0874	0.0135	-0.109	-0.133	-0.025	0.1469	-0.289	-0.125	-0.08	0.323	0.207	0.258	-0.16	-0.09	0.048	-0.18	-0.07	0.226968	-0.15827	-0.069073376	-0.089200556
3234	Q99962	SH3GL2	SH3 domain containing GRB2 like 2, endop	6456	ENSG00000107295	10	0.444	0.335	-0.27				-0.81	-0.38	-0.62	-0.183	0.1472	-0.046				-0.345	0.2395	0.3569	-0.25	-1.11	-0.2				-0.32	-0.17	-0.08	0.428638	0.138604	-0.243946114	0.382549629
2694	Q6N063	OGFOD2	2-oxoglutarate and iron dependent oxygenas	79676	ENSG00000111325	3	0.032	0.372	-0.37				-0.38	-0.15	-0.07	-0.505	-0.145	0.2416				-0.18	-0.019	0.0969	-0.04	-0.8	-0.39				-0.04	-0.17	0.144	0.788113	0.121687	-0.008783265	0.130469863
870	H7BXE3	SLTM	SAFB like transcription modulator	79811	ENSG00000137776	1	-0.5	-1.31	-0.46	-1.86	0.04	0.226	-0.74	-0.42	-1.67	-0.466	0.3345	-0.704	-0.493	-0.153	0.0804	-1.232	-1.666	-1.05	1.372	0.92	0.759	-0.07	0.581	-0.42	0.972	-1.44	-1.95	0.267777	-0.82404	-0.148844419	-0.675192118
705	F5H1M8	PCMTD1	protein-L-isoaspartate (D-aspartate) O-meth	115294	ENSG00000168300	3	-0.01	0.166	-0.48	0.205	-0.18	-0.37	0.433	0.243	0.344	0.1734	0.2324	0.0798	0.3027	-0.236	0.0725	0.4351	0.3517	0.3218	-0.07	-0.32	-0.42	-0.97	-0.43	-0.15	0.239	0.26	0.473	0.255239	0.192289	-0.154242463	0.346531285
668	E9PKT9	MOCS2	molybdenum cofactor synthesis 2	4338	ENSG00000164172	1	0.357	0.002	0.2	0.159	-0.15	-0.33	-0.11	-0.05	0.203	0.2094	0.1374	0.0147	0.1655	-0.05	0.0224	0.1516	-0.153	-0.161	0.156	-0.6	-0.13	-0.49	-0.17	-0.09	0.119	0.032	0.317	0.535059	0.127184	-0.005143612	0.132327783
1627	P15328	FOLR1	folate receptor 1;folate receptor alpha	2348	ENSG00000110195	6	-0.51	-0.2	0.178	-0.08	-0.64	-0.44	-0.37	-0.03	0.268	-0.139	0.1546	0.5453	-0.205	-0.135	-0.182	-0.113	0.4047	0.2114	-0.48	-0.63	0.201	0.102	0.135	-0.14	-0.02	-0.2	-0.3	0.307369	-0.05253	-0.262210426	0.209675726
829	H0YJ75	PPP2R5C	protein phosphatase 2 regulatory subunit B'	5527	ENSG00000078304	8							-0.19	0.035	0.098							0.0317	0.1001	0.0745							0.062	-0.09	0.128	0.744039	-0.05365	-0.087685406	0.034035639
153	A0A0A0MSA	LAMA3	laminin subunit alpha 3	3909	ENSG00000053747	4	-1.41	-1.43	-0.83	-1.45	-1.53	-0.9	-2.36	-1.76	-2.29	-1.554	-0.271	-0.547	-1.029	-1.342	-1.891	-1.448	-1.457	-1.97	-1.29	-1.19	2.816	2.304	0.72	1.508	-0.75	-0.87	-2.66	0.117205	-1.61722	-0.273107817	-1.344116413
808	H0Y8D0	TMEM222	transmembrane protein 222	84065	ENSG00000186501	1	0.084	0.593	0.249	-0.1	0.048	-0.63	-0.44	-0.02	-0.35	0.2359	-0.167	-0.144	-0.213	-0.409	0.0473	0.0198	0.2594	0.0973	-0.24	-0.18	0.025	-0.53	-0.53	-0.22	-0.4	0.13	0.218	0.614178	0.130207	-0.031657328	0.161864234
151	A0A0A0MS6	IZUMO4	IZUMO family member 4	113177	ENSG00000099840	3	-0.31	0.053	-0.39	0.556	0.431	-0.06	0.433	1.294	0.384	-0.304	0.3778	-0.224	-0.517	-0.221	-0.314	0.0078	-0.286	-0.25	0.075	0.706	1.683	-0.44	-0.15	-0.33	0.137	-0.19	-0.19	0.315704	0.12321	0.457988558	-0.33477853
1292	R4GMU1	H6PD	hexose-6-phosphate dehydrogenase/glucos	9563	ENSG00000049239	13	-0.12	-0.2	-0.22	-0.26	-0.21	0.091	0.131	0.161	0.079	0.0572	-0.024	-0.015	-0.591	0.0976	0.0135	0.1607	-0.551	-0.32	0.043	0.444	0.034	-0.02	0.103	-0.31	0.529	0.228	-0.1	0.277457	-0.16628	0.069849895	-0.236132671
292	A0A1W2PQ	GOSR2	golgi SNAP receptor complex member 2	9570	ENSG00000108433	1	-0.1	-0.81	-0.22				0.601	-0.02	-0.24	-0.108	0.1822	-0.061				0.1808	-0.851	-0.173	-0.52	-0	-0.02				0.675	-0.2	-0.36	0.969389	-0.06167	0.005358509	-0.067032467
365	A4D1U4	KIAA1147;	KIAA1147;DENN domain containing 11	57189	ENSG00000257093	6	0.176	0.058	0.158	-0.05	-0.02	-0.51	-0.02	-0.11	0.139	0.3141	0.0994	0.1783	-0.172	-0.376	0.2603	-0.04	-0.008	0.0077	-0.08	-0.24	-0.09	-0.47	-0.44	0.325	0.153	-0.23	0.027	0.554691	0.09717	-0.048406738	0.145576872
669	E9PKV8	TTC9C	tetratricopeptide repeat domain 9C	283237	ENSG00000162222	2	0.191	0.06	-0.07	0.12	0.375	-0.04	-0	-0.15	0.135	0.1154	-0.048	-0.05	0.1921	-0.286	0.431	0.0312	-0.062	0.0658	-0.32	-0.35	-0.01	-0.28	0.044	-0.09	0.035	-0.06	0.111	0.248419	0.171399	0.025342059	0.146056691
1001	O00303	EIF3F	eukaryotic translation initiation factor 3 subu	8665	ENSG00000175390	6	0.14	-0.35	-0.38	-0.02	0.044	-0.54	0.001	0.151	0.103	0.0303	-0.075	-0.171	-0.067	-0.284	-0.054	0.2367	0.2751	0.2406	0.132	-0.28	0.144	-0.7	-0.47	-0.11	0.502	-0.06	0.174	0.781364	-0.02027	-0.109713947	0.089440702
3411	Q9H8M7	MINDY3	MINDY lysine 48 deubiquitinase 3	80013	ENSG00000148481	4	0.295	0.307	-0.05	0.028	0.239	-0.4	-0.04	0.123	0.077	-0.009	-0.062	0.202	0.1595	-0.123	0.0051	-0.042	0.0743	-0.022	-0.26	-1.09	-0.2	-0.51	0.004	0.363	-0.14	-0.13	0.151	0.264389	0.264355	0.043925275	0.220429825
2260	P78560	CRADD	CASP2 and RIPK1 domain containing adap	8738	ENSG00000169372	3	-0.94	-1.53	-1.12				0.034	0.354	0.056	-0.771	0.0814	-1.369				0.2736	-0.043	-0.053	-0.68	0.548	0.799				0.335	#####	0.117	0.336429	-0.71054	-0.210853434	-0.49968877
3136	Q96GX9	APIP	APAF1 interacting protein	51074	ENSG00000149089	2							0.345	0.058	0.238							0.4378	-0.034	0.0348							0.065	0.325	0.129	0.936219	0.040607	0.067414214	-0.026806978
745	R4GNG3	GIT2	GIT ArfGAP 2	9815	ENSG00000139436	3	-0.05	-0.13	-0.02				0.126	-0.1	0.391	-0.185	-0.447	-0.023				0.2069	0.1054	0.2354	-0.31	0.046	-0.35				0.088	0.098	0.098	0.849519	0.092807	0.055670817	0.037136445
430	B4DL54	CHURC1-F	CHURC1-FNTB readthrough;farnesyltransf	100529261	ENSG00000125954	5	-0.24	-0.36	-0.48	-0.22	-0.05	-0.38	-0.31	-0.23	0.031	-0.49	-0.547	-0.106	-0.128	-0.215	-0.205	-0.135	-0.115	-0.113	-0.36	-0.11	-0.11	-0.06	-0.28	-0.2	-0.06	-0.13	-0.17	0.586969	-0.08579	-0.021110034	-0.064675071
1159	O60504	SORBS3	sorbin and SH3 domain containing 3	10174	ENSG00000120896	2	0.039	0.178	-0.14	0.181	0.138	-0.35	-0.54	-0.39	-0.22	0.0541	0.2407	0.1744	-0.073	-0.016	0.0869	-0.271	0.1046	-0.36	-0.07	-0.77	0.009	-0.49	-0.32	-0.08	-0.28	-0.17	-0.05	0.277216	0.123732	-0.117298381	0.24103062
2506	Q15198	PDGFRL	platelet derived growth factor receptor like	5157	ENSG00000104213	1	-0.12	-0.05	-0.42				-0.48	0.01	-0.1	0.0238	0.2014	0.1287				-0.418	-0.446	-0.657	-0.42	-0.53	0.214				0.174	-0.21	-0.39	0.99972	-0.00091	0.00049914	-0.001404783
686	E9PQE8	TRAPPC4	trafficking protein particle complex 4	51399	ENSG00000196655	3	0.013	0.037	-0.2	-0.17	-0.08	-0.19	0.036	-0.08	0.111	0.1477	0.1077	0.0494	0.0255	0.0458	-0.031	0.0816	0.0643	0.0874	-0.11	0.273	-0.07	0.102	-0.09	-0.04	0.005	-0.04	0.079	0.175729	-0.07113	-0.122183528	0.051058394
413	B1ALM5	TMEM9	transmembrane protein 9	252839	ENSG00000116857	2	-0.35	0.457	0.292	-0.31	-0.24	-0.1	0.481	0.511	-0.15	0.2939	0.1216	0.1653	0.4009	-0.387	0.1842	-0.294	-0.193	0.9501	-0.2	-0.15	-0.21	-0.14	-0.15	0.278	-1.01	0.832	-1.13	0.42567	0.276204	-0.071147191	0.347351564
483	Q969M7	UBE2F	ubiquitin conjugating enzyme E2 F (putative	140739	ENSG00000184182	2	0.019	-0.27	-0.51	-0.25	-0.02	0.224	0.939	0.346	0.494	0.1152	-0.008	-0.066	-0.066	-0.165	-0.015	0.2739	-0.312	-0.025	0.047	0.032	0.142	0.225	-0.1	0.131	0.286	0.056	1.494	0.468903	-0.14854	0.137692141	-0.28623213
729	F8VNQ3	TENM2	teneurin transmembrane protein 2	57451	ENSG00000145934	7	0.112	-0.46	-0.29	-0.25	-0.04	-0.6	0.001	0.448	-0.05	0.0238	-0.118	-0.263	-0.607	-0.25	-0.417	0.527	-0.086	0.0578	-0.4	-0.14	-0.09	-0.7	-0.57	-0.07	0.446	0.346	0.083	0.99923	-0.0057	-0.000411007	-0.005291722
3164	Q96LM1	LINC0061	long intergenic non-protein coding RNA 615	439916	ENSG00000196243	1							-0.21	-0.1	-0.21							0.219	-0.192	-0.039							-0.29	-0.18	0.366	0.764037	-0.14091	-0.17042248	0.029508121
1107	O43252	PAPSS1	3'-phosphoadenosine 5'-phosphosulfate syn	9061	ENSG00000138801	2				-0.06	-0.1	-0.51	-0.13	-0.14	0.209				-0.128	0.2076	-0.029	-0.185	0.2815	-0.037				0.018	-0.1	-0.24	-0.07	0.108	0.316	0.577181	-0.12827	-0.140673211	0.012400902
2039	P51178	PLCD1	phospholipase C delta 1	5333	ENSG00000187091	3				0.48	0.117	-0.1	-0.17	0.162	-0.33				-0.074	-0.109	0.3724	0.1315	0.4808	0.1138				0.064	-0.35	-0.29	0.075	0.008	0.356	0.636829	0.049239	-0.125231357	0.174470618
3497	Q9NS68	TNFRSF1	TNF receptor superfamily member 19	55504	ENSG00000127863	1							-0.42	0.323	-0.32							0.245	0.0359	0.2455							0.012	0.411	0.071	0.498043	-0.3034	-0.314220502	0.010824488
3317	Q9BYG5	PARD6B	par-6 family cell polarity regulator beta	84612	ENSG00000124171	3	-0.13	-0.17	-0.25	0.01	-0.26	-0.29	0.045	0.916	0.143	-0.199	0.107	-0.069	-0.022	-0.184	-0.105	-0.483	0.3133	-0.724	-0.18	-0.19	0.115	-0.47	0.152	-0.04	-1.4	-0.23	-0.63	0.400363	0.3209	0.152450786	0.168449449
699	H0YGE9	M6PR	mannose-6-phosphate receptor, cation depe	4074	ENSG00000003056	2				0.323	-0.11	0.088	-0.54	-0.42	-0.7				-0.142	0.0381	-0.252	-0.868	-0.861	-0.743				0.379	-0.19	0.076	-0.1	-0.33	-0.07	0.288794	-0.18715	0.245173821	-0.432319799
2256	P78356	PIP4K2B	phosphatidylinositol-5-phosphate 4-kinase ty	8396	ENSG00000276293	6	0.058	0.197	-0.07	0.273	0.168	-0.4	0.078	0.037	0.359	0.0053	0.0182	0.0816	0.1998	-0.147	0.2525	0.1361	0.2707	0.2396	-0.26	-0.89	-0.37	-0.66	-0.42	0.214	-0.11	0.037	0.235	0.118503	0.324871	-0.038718143	0.363589327
2716	Q6R327	RICTOR	RPTOR independent companion of MTOR	253260	ENSG00000164327	8	0.014	0.108	0.085	-0	0.011	-0.16	0.092	-0.15	0.112	-2E-04	-0.1	0.0432	0.1569	-0.075	0.0136	0.0613	-0.308	0.1983	-0.01	-0.29	-0.2	-0.32	-0.25	-0.04	0.107	-0.03	0.018	0.277895	0.124415	0.012835438	0.111579688
458	B7Z4T2	PXYLP1	2-phosphoxylose phosphatase 1	92370	ENSG00000155893	3	-0.34	-1.05	-0.62				0.485	0.257	0.154	-0.452	-0.22	0.0092				0.2949	-0.677	-0.373	-0.63	0.131	-0.46				0.382	-0.16	0.155	0.911533	-0.08904	0.050442901	-0.139485694
294	A0A1W2PPI	HLA-B	major histocompatibility complex, class I, B	3106	ENSG00000234745	8	0.436	-0.82	-0.7	-0.89	-1.27	-1.13	-1.22	-0.7	-1.29	0.8977	-0.406	0.6144	-1.132	-0.812	0.2198	-0.489	0.3477	0.7704	1.015	-0.24	1.311	0.327	-0.74	0.669	-0.7	-0.31	-0.23	0.102323	-0.96538	-0.843665265	-0.121718658
933	J3QQZ9	PNPO	pyridoxamine 5'-phosphate oxidase	55163	ENSG00000108439	4	0.091	0.051	0.309				-0.02	0.34	-0.06	-0.351	-0.123	0.4722				-0.309	-0.072	-0.224	-0.05	-0.8	-0.11				0.029	-0	-0.2	0.308981	0.306743	0.220282238	0.086460774
3691	Q9UQB8	BAIAP2	BAR/IMD domain containing adaptor protein	10458	ENSG00000175866	43							0.23	-0.02	0.087							0.0459	-0.602	-0.431							0.013	-0.07	0.428	0.275147	-0.02558	0.428388258	-0.453970819
2683	Q6IA17	SIGIRR	single Ig and TIR domain containing	59307	ENSG00000185187	3				-0.06	-0.2	-0.12	0.065	0.131	0.469				0.1624	-0.071	0.5058	-0.314	0.5411	-0.1				-0.34	-0.13	0.207	-0.26	-0.2	0.234	0.607921	0.128675	-0.072892165	0.201567072
3043	Q92692	NECTIN2	nectin cell adhesion molecule 2	5819	ENSG00000130202	3	-0.09	-0.33	-0.07	0.139	-0.18	0.2	-0.38	0.136	0.066	0.0111	0.1673	-0.169	-0.109	0.5838	-0.23	0.2937	-0.195	-0.167	-0.54	0.571	-0.4	0.144	0.032	-0.07	0.447	0.027	-0.31	0.903621	-0.04577	-0.077408052	0.031640095
894	P18084	ITGB5	integrin subunit beta 5	3693	ENSG00000082781	4	-0.3	0.327	0.041	-0.27	-0.35	-0.26	0.379	-0.11	0.369	0.3703	0.324	0.0354	0.0489	-0.212	0.1288	-0.109	-0.098	-0.025	0.189	-0.11	0.178	-0.31	-0.21	0.532	0.332	-0.01	-0.36	0.90571	-0.04504	-0.070841438	0.025798135
173	A0A0A6YYG	STON1-GT	STON1-GTF2A1L readthrough	286749	ENSG00000068781	7	-0.09	0.443	-0.14	0.039	-0.08	-0.34	0.1	-0.08	0.136	0.0553	0.1171	0.2248	-0.028	0.0957	0.1617	0.3404	0.1971	0.2254	0.065	-0.69	-0.12	-0.31	-0.09	-0.15	-0.13	0.025	0.36	0.170257	0.115437	-0.154526192	0.269963132
227	J3QTJ5	LRRC37A	leucine rich repeat containing 37 member A	374819	ENSG00000176809	3	0.339	-1.09	0.128				0.554	1.664	0.705	0.0138	-0.751	0.3452				-1.11	-1.238	-1.295	1.489	0.026	-0.71				-0.11	-0.76	-1.23	0.284111	0.600063	1.055383151	-0.455320292
834	H0YLX6	AP3S2	adaptor related protein complex 3 subunit si	10239	ENSG00000157823	1							0.614	0.014	-0.01							-0.198	0.1477	-0.004							0.193	-0.14	5E-04	0.656635	0.185743	0.222804254	-0.037061402
1707	P21359	NF1	neurofibromin 1	4763	ENSG00000196712	8	0.156	0.015	-0.03	-0.1	-0.08	0.335	0.02	0.011	0.203	0.1628	-0.044	0.0589	-0.003	-0.111	0.0047	0.4181	0.4166	0.1524	-0.2	-0.44	-0.05	0.079	-0.02	0.021	0.204	0.056	0.246	0.496331	0.07083	-0.057925464	0.128755457
2568	Q2I0M4	LRRC26	leucine rich repeat containing 26	389816	ENSG00000184709	4	-0.62	0.773	-0.14	-0.12	-0.09	-0.36	-0.71	-0.5	-0.4	0.3107	-0.011	0.0415	0.2008	0.1906	-0.175	-0.003	0.1108	0.3221	-0.01	-0.12	-0.05	0.196	-0.14	0.216	-0.28	-0.19	-0.09	0.17846	-0.18833	-0.350323783	0.161996577
1315	O95848	NUDT14	nudix hydrolase 14	256281	ENSG00000183828	2				-0.07	-0.03	-0.21	0.846	0.162	0.736				-0.023	0.2973	0.0496	0.7988	0.0865	0.3884				0.039	0.195	-0.05	0.635	0.289	0.658	0.973585	-0.0569	-0.028498791	-0.02839877
1035	O14545	TRAFD1	TRAF-type zinc finger domain containing 1	10906	ENSG00000135148	5	0.203	0.17	0.136	-0.62	-0.92	-0.56	0.154	0.246	0.3	0.1418	-0.007	0.148	-0.847	0.1183	0.1664	0.4112	-0.068	0.1784	-0.22	-0.62	-0.15	-0.44	-0.48	0.578	0.22	0.396	0.103	0.866306	-0.03054	-0.126759651	0.096217755
2509	Q15274	QPRT	quinolinate phosphoribosyltransferase	23475	ENSG00000103485	2	-0.03	0.045	0.257				0.025	0.438	0.304	-0.353	0.0332	-0.256				-0.408	-0.193	-0.428	-0.41	-0.56	-0.53				0.67	0.164	-0.49	0.205075	0.365969	0.440950823	-0.07498221
3320	Q9BYT8	NLN	neurolysin	57486	ENSG00000123213	5	0.117	0.124	-0.08	0.112	0.023	-0.09	0.447	0.04	0.318	0.0603	0.4683	0.1127	-0.102	0.069	0.4241	0.4704	0.3113	-0.126	-0.03	-0.66	-0.03	-0.25	0.164	-0.02	0.26	0.24	0.357	0.462552	0.10872	-0.075029548	0.183749328
1350	P01042	KNG1	kininogen 1	3827	ENSG00000113889	2	-0.73	-1.03	0.285				-0.21	-0.06	-0.33	1.1799	0.8412	0.327				0.2109	-0.327	-0.299	0.076	-0.08	1.704				-0.02	0.389	-1.01	0.389013	-0.52149	-0.666850656	0.145358598
2398	Q13325	IFIT5	interferon induced protein with tetratricopept	24138	ENSG00000152778	3	-0.12	0.208	0.138	0.129	-0.26	-0.39	-0.14	0.028	0.225	-0.11	-0.024	0.1671	0.0062	0.0512	-0.007	0.026	-0.101	-0.132	-0.25	-0.77	-0.15	-0.23	-0	0.172	-0.23	0.094	-0.09	0.418211	0.140718	-0.007297173	0.148014793
1738	P23921	RRM1	ribonucleotide reductase catalytic subunit M	6240	ENSG00000167325	4	0.097	0.34	-0.16	0.232	0.305	-0	-0.12	-0.09	0.161	0.0561	-0.223	0.063	0.0493	0.0348	0.5454	0.0649	0.2459	-0.175	0.269	-0.16	-0.16	-0.28	-0.19	0.197	0.193	0.239	0.059	0.849685	0.06608	0.010815019	0.055265232
3748	Q9Y3Q8	TSC22D4	TSC22 domain family member 4	81628	ENSG00000166925	6	0.633	0.579	0.299	-0.09	0.174	-0.34	0.018	0.381	0.144	0.4419	0.6261	0.3093	0.2047	0.0881	0.1054	0.1388	-0.021	0.0134	-0.05	-1.02	-0.1	-0.46	-0.11	0.108	0.09	0.186	0.129	0.164173	0.335637	-0.011806833	0.347443751
1448	P06312	IGKV4-1	immunoglobulin kappa variable 4-1	28908	ENSG00000211598	3	-0.83	-0.94	-0.36	-0.34	-0.45	-0.26	-1.07	-0.2	-0.87	-0.631	-0.321	-0.482	-0.249	-0.393	-0.252	-0.275	-0.738	-0.745	-1	0.267	2.234	1.453	0.042	0.588	-0.15	-0.15	-1.15	0.14675	-0.82919	-0.136490615	-0.69269521
2101	P54922	ADPRH	ADP-ribosylarginine hydrolase	141	ENSG00000144843	2	-0.1	-0.1	-0				0.633	0.328	0.298	0.2052	-0.071	-0.125				0.5416	-0.045	0.0679	-0.25	-0.67	0.035				0.368	0.265	0.204	0.720782	0.18494	0.080995012	0.103944857
807	O60476	MAN1A2	mannosidase alpha class 1A member 2	10905	ENSG00000198162	4	0.202	0.046	-0.16				-0.22	0.287	0.214	0.1735	0.1045	-0.076				0.619	0.1844	0.3535	-0.12	0.478	0.032				0.025	0.001	0.16	0.559059	-0.03524	-0.165164599	0.129922794
1655	P17707	AMD1	adenosylmethionine decarboxylase 1	262	ENSG00000123505	8	-0.32	0.161	0.177	-0.66	0.09	-0.49	-0.02	0.34	0.043	0.4462	-0.153	-0.014	-0.145	0.3604	0.0885	-0.556	-0.309	0.1216	-0.09	-0.5	-0.1	-0.08	0.166	0.559	0.031	0.223	-0.04	0.879156	-0.09441	-0.058338094	-0.036073648
141	A0A0A0MR	GSTM4	glutathione S-transferase mu 4	2948	ENSG00000168765	18	-1.57	1.694	-1.25	-1.26	1.588	1.268	-1.24	-1.33	2.738	-1.059	-0.938	-1.19	-1.164	-1.399	-1.52	-1.434	-1.853	-1.42	0.791	0.616	1.337	-0.7	-1.07	0.05	-1.45	-0.22	-1.51	0.164208	0.312085	1.402015097	-1.089929991
2613	Q5JU69	TOR2A	torsin family 2 member A	27433	ENSG00000160404	4	-0.07	-0.16	-0.25	-0.44	-0.05	-0.49	0.003	0.084	0.075	0.1795	0.1546	0.2525	-0.333	0.286	-0.26	0.2285	0.1139	0.4456	-0.02	0.132	-0.05	0.075	0.053	-0.08	0.221	0.089	0.281	0.133512	-0.22083	-0.26198363	0.041152362
3047	Q92747	ARPC1A	actin related protein 2/3 complex subunit 1A	10552	ENSG00000241685	13	0.075	0.378	-0.1	-0.05	0.112	-0.19	0.229	-0.1	0.366	0.1533	0.0117	0.18	0.0514	-0.121	0.1947	0.3556	0.2555	0.1792	-0.17	-0.7	-0.22	-0.27	-0.28	-0.13	-0.05	0.197	0.156	0.111336	0.243302	-0.060525354	0.303826969
609	E7ERK6	CLU	clusterin	1191	ENSG00000120885	22	-1.07	-0.78	-1.04	0.363	0.142	-0.76	-0.92	1.044	0.482	-1.035	-1.474	-0.268	-0.646	0.0059	-0.753	-0.669	-0.823	-0.818	-0.83	-0.39	-0.22	-0.14	-0.52	-0.59	0.252	-0.38	-1.34	0.450185	0.179771	0.437498732	-0.25772741
3711	Q9Y295	DRG1	developmentally regulated GTP binding prot	4733	ENSG00000185721	4	0.419	0.679	0.388	-0.13	0.163	-0.09	5E-04	-0.16	-0.07	0.5473	0.5547	0.4072	0.2342	0.0041	0.0054	0.1794	-0.23	-0.105	0.462	-0.18	0.262	1.308	0.208	0.648	0.058	0.018	-0.08	0.713108	-0.16527	-0.042677953	-0.122587467

1687	P20160	AZU1	azurocidin 1	566	ENSG00000172232	3	-0.14	-0.58	0.729	0.312	0.181	0.612	-0.98	-1.26	-1.65	-0.949	-0.034	-0.329	-0.227	-1.62	-1.064	-0.947	-2.553	-1.646	-0.38	-0.24	2.892	1.56	-1.08	0.254	-1.52	-1.58	-2.51	0.478997	-0.01883	0.733545405	-0.752375658
1486	P07948	LYN	LYN proto-oncogene, Src family tyrosine kin	4067	ENSG00000254087	30							0.103	0.27	-0.07							0.1196	-0.495	-0.228							0.559	-0.18	-0.01	0.541985	-0.02292	0.302935913	-0.325857312
3125	Q96EP0	RNF31	ring finger protein 31	55072	ENSG00000092098	6	0.144	0.275	-0.23				-0	-0.17	0.005	0.1492	-0.038	0.374				-0.105	-0.272	0.0548	0.078	0.116	0.147				0.124	-0.18	-0.2	0.983493	-0.01124	-0.02403738	0.012794572
2297	Q02083	NAAA	N-acylethanolamine acid amidase	27163	ENSG00000138744	7	-0.04	-0.17	0.097	-0.05	-0.28	-0.54	-0.17	-0.04	0.282	0.0069	-0.003	0.3796	0.1087	-0.007	-0.343	0.2411	-0.547	0.1619	-0.22	-0.03	0.142	-0.22	-0.49	-0.22	0.554	0.038	-0.75	0.739011	0.031709	-0.10103031	0.13273955
3140	Q96HA7	TONSL	tonsoku like, DNA repair protein	4796	ENSG00000160949	1				-0.14	-0.06	-0.23	0.363	-0.31	0.029				-0.141	-0.267	0.2374	-0.511	0.0462	-0.126				-0.77	-0.2	0.071	-0.14	-0.21	-0.03	0.722829	0.153464	0.069021753	0.084442154
3410	Q9H841	NIPAL2	NIPA like domain containing 2	79815	ENSG00000104361	2	-0.2	0.062	-0.21	-0.54	-0.32	-0.47	-0.52	-0.56	0.039	-0.073	0.0851	-0.081	0.2615	-0.369	0.1966	0.279	0.1189	-0.149	-0.43	-0.53	-0.4	-0.47	-0.12	0.158	-0.03	-0.17	-0.11	0.111161	-0.06786	-0.331536332	0.263672032
656	E9PIX6	PRMT1	protein arginine methyltransferase 1	3276	ENSG00000126457	4	0.158	0.304	0.088	-0.07	0.024	-0.54	-0.43	-0.32	-0.17	0.2086	-0.029	0.1241	0.1424	-0.293	-0.093	-0.409	0.1204	-0.269	-0.09	0.291	0.119	-0.33	-0.42	0.084	-0.06	-0.29	-0.28	0.918825	0.002001	-0.051365564	0.053366312
454	B5MCT7	PPM1F	protein phosphatase, Mg2+/Mn2+ depender	9647	ENSG00000100034	2							0.01	0.238	0.114							0.228	0.0671	0.2371							0.154	-0.03	0.059	0.570833	0.058319	-0.056781999	0.115101038
2684	Q6IA69	NADSYN1	NAD synthetase 1	55191	ENSG00000172890	4	0.018	0.211	-0.23	0.132	1.276	-0.51	0.241	0.24	0.198	0.4801	-0.2	0.1056	0.0324	-0.471	-0.506	0.0719	-0.045	0	-0.19	-0.34	-0.24	-0.02	-0.7	-0.02	0.281	-0.08	-0.02	0.328027	0.323316	0.234120312	0.08919612
553	F6U1T9	PPP3R1	protein phosphatase 3 regulatory subunit B,	5534	ENSG00000221823	4	0.572	0.285	0.135	-0.14	0.512	-0.25	-0.32	-0.09	-0.31	0.4239	0.6084	0.0663	0.6249	-0.186	0.1937	-0.194	-0.082	-0.251	-0.4	-0.58	-0.27	-0.65	0.002	0.206	-0.05	-0.08	-0.11	0.221427	0.256909	-0.089848541	0.34675755
1445	P06132	UROD	uroporphyrinogen decarboxylase	7389	ENSG00000126088	4	0.188	0.177	-0.04				-0.28	0.099	0.255	0.0582	0.2258	0.1511				0.6154	0.3173	0.2015	-0.1	-0.59	-0.12				-0.05	0.065	-0.02	0.117982	0.203967	-0.194143012	0.39811005
3324	Q9BZL1	UBL5	ubiquitin like 5	59286	ENSG00000198258	2	-0.03	0.081	-0.08	-0.09	-0.15	-0.3	0.095	0.056	0.055	0.0958	0.0702	-0.055	-0.062	0.0585	-0.034	0.1207	0.0488	0.0944	-0.12	-0.31	-0.07	-0.48	-0.27	-0.02	-0.11	0.089	0.532	0.537831	0.043368	-0.077939489	0.121307445
123	G3V4V5	HECTD1	HECT domain E3 ubiquitin protein ligase 1	25831	ENSG00000092148	2							-0.14	-0.02	0.089							0.1839	0.2905	0.2774							0.17	0.123	0.051	0.122925	-0.13838	-0.274330273	0.135945421
1278	O95297	MPZL1	myelin protein zero like 1	9019	ENSG00000197965	1				0.38	-0.2	0.158	-0.12	0.249	-0.13				-0.05	-0.339	0.1716	0.049	0.0174	-0.061				0.011	-0.15	0.129	-0.16	-0.08	0.091	0.748646	0.08245	0.091706897	-0.009256397
3625	Q9UI30	TRMT112	tRNA methyltransferase subunit 11-2	51504	ENSG00000173113	3	0.022	0.419	0.1	-0.17	-0.27	-0.21	0.068	-0.01	0.202	0.2033	-0.018	-0.01	-0.162	-0.424	-0.06	0.0269	-0.061	0.1411	-0.01	-0.32	-0.16	0.34	-0.27	0.109	0.122	-0.07	0.111	0.888445	0.033695	0.058014283	-0.02431903
2576	Q32P28	P3H1	prolyl 3-hydroxylase 1	64175	ENSG00000117385	3	0.08	-0.27	-0.04				-0.2	0.191	0.129	0.1498	0.2562	-0.225				0.364	0.2132	0.1856	0.573	1.087	0.762				0.302	0.021	-0	0.161754	-0.47552	-0.175496481	-0.300020137
2570	Q2M3M2	SLC5A9	solute carrier family 5 member 9	200010	ENSG00000117834	3	0.055	0.044	0.444	0.5	0.613	0.139	0.062	0.086	0.226	0.9872	0.4802	0.3888	0.5405	-0.531	-0.22	0.0158	0.1589	0.3048	0.104	0.245	0.099	-0.18	-0.39	-0.33	0.289	0.254	0.2	0.469834	0.209163	0.004784477	0.204378708
1114	O43427	FIBP	FGF1 intracellular binding protein	9158	ENSG00000172500	3							-0.11	-0.28	0.119							-0.099	0.0025	-0.041							-0.14	-0.36	-0.05	0.673312	0.093571	-0.04352938	0.13710054
3073	Q969L2	MAL2	mal, T cell differentiation protein 2 (gene/pse	114569	ENSG00000147676	2	-0.1	0.281	-0.13	0.058	-0.1	-0.56	-0.26	-0.15	-0.02	0.1975	-0.083	0.1903	0.1238	-0.17	-4E-04	-0.035	-0.144	-0.075	0.007	0.135	-0.1	-0.19	-0.26	-0.33	-0.18	0.194	0.079	0.60392	-0.03635	-0.108607286	0.07225403
1920	P42356	PI4KA	phosphatidylinositol 4-kinase alpha	5297	ENSG00000241973	14	0.233	0.483	-0.52	0.191	-0.06	-0.14	-0.45	-0.19	-0.01	-0.339	-0.026	0.3702	0.0289	-0.121	0.2815	0.3543	-0.217	0.2514	-0.41	-0.65	-0.36	-0.47	-0.27	-0.25	-0.04	-0.09	0.241	0.207167	0.202871	-0.116125921	0.318997086
170	P48200	IREB2	iron responsive element binding protein 2	3658	ENSG00000136381	3				-0.02	-0.09	-0.32	-0.1	-0.03	0.019				0.2369	-0.015	0.0641	-0.057	-0.174	-0.09				0.048	-0	-0.01	0.224	-0.12	0.017	0.435728	-0.11788	-0.084214696	-0.033663294
828	H0YJ34	FERMT2	fermitin family member 2	10979	ENSG00000073712	9	0.138	0.71	-0.13	-0.23	-0.23	-0.39	-0.16	0.011	0.041	0.0502	-0.388	-0.085	-0.17	-0.233	0.147	0.1501	-0.017	0.0794	0.113	-0.11	0.252	-0.33	-0.25	-0.25	-0.01	0.053	0.212	0.983687	0.006898	0.024466756	-0.017568758
2839	Q8IV08	PLD3	phospholipase D family member 3	23646	ENSG00000105223	3	-0.09	0.165	0.135	-0.26	-0.19	-0.15	-0.15	-0.04	-0.11	0.1505	-0.078	-0.026	-0.232	-0.172	-0.246	0.0552	-0.003	0.1041	-0.16	-0.11	-0.15	0.051	-0.26	-0.14	0.213	0.013	-0.04	0.946476	-0.01156	-0.027009363	0.01545124
326	A0A2R8Y6H	DYNC1H1	dynein cytoplasmic 1 heavy chain 1	1778	ENSG00000197102	84	0.092	0.172	0.036	-0.21	-0.02	-0.22	-0.83	-0.65	-0.61	0.3132	0.4434	0.2222	-0.301	0.4653	-0.088	-0.806	0.0304	-0.523	-0.19	-0.61	-0.12	-0.39	-0.07	0.029	-0.47	-0.21	-0.38	0.450373	0.017118	-0.222231342	0.23934974
1452	R4GN98	S100A6	S100 calcium binding protein A6	6277	ENSG00000197956	3	-0.69	-0.6	-0.3	-0.5	-0.31	-0.26	-0.2	-0.28	-0.57	-0.887	0.3611	-0.523	-0.295	-0.244	-0.033	-0.202	-0.204	-0.748	-0.56	-0.7	1.686	1.31	0.127	0.533	-0.07	-0.06	-0.37	0.170597	-0.62233	-0.104438265	-0.517892806
822	H0YC40	CD47	CD47 molecule	961	ENSG00000196776	2	-0.04	-0.87	-0.15	0.126	-0.64	-0.14	0.007	-0.18	-0.07	-0.34	-0.322	0.0323	-0.613	-0.141	0.2765	0.4695	-0.354	-0.028	-0.38	-0.64	-0.55	-0.24	-1.01	-0.42	0.054	0.185	-0.06	0.543138	0.122122	-0.104408645	0.226530347
1121	O43524	FOXO3	forkhead box O3	2309	ENSG00000118689	2				0.066	0.019	-0.16	0.046	0.202	0.125				0.0364	-0.036	0.4088	0.1349	0.1396	0.189				-0.3	-0.11	0.22	0.287	0.239	0.152	0.745242	-0.03291	-0.095631862	0.062725782
2980	Q8TDB6	DTX3L	deltex E3 ubiquitin ligase 3L	151636	ENSG00000163840	5	0.09	0.025	-0.09	0.171	-0.36	-0.29	0.164	0.041	0.181	0.3219	0.3962	0.0453	0.1315	0.2993	-0.199	0.2608	0.2485	0.2575	0.042	-0.07	0.271	0.08	0.142	0.389	0.006	0.281	0.284	0.183069	-0.16612	-0.203271465	0.03714676
1974	P48553	TRAPPC1	trafficking protein particle complex 10	7109	ENSG00000160218	4	0.372	0.152	-0.01	-0.38	-0.15	-0.41	0.069	0.261	0.367	0.0104	0.0371	-0.03	-0.241	-0.041	-0.38	0.026	0.5751	0.3444	-0.5	-0.56	-0.35	-0.15	-0.39	-0.1	0.405	0.056	0.329	0.561576	0.169239	-0.003963089	0.173202403
171	K7EJ28	TNFSF13	TNF superfamily member 13	8741	ENSG00000161955	2	-0.3	-0.78	-0.19	-0.02	-0.36	-0.02	0.135	0.43	0.272	-0.021	0.0452	0.2737	-0.127	0.4228	0.2077	0.3247	-0.024	0.0068	-0.28	0.245	0.328	-0.12	-0.18	-0.26	0.777	0.552	-0.08	0.457286	-0.20281	-0.21574022	0.012926184
3446	Q9HD15	SRA1	steroid receptor RNA activator 1	10011	ENSG00000213523	2	-2.01	-1.35	-1.47				0.058	0.655	-0	-1.178	-1.772	-2.192				1.1325	0.7432	1.2675	-1.98	0.214	0.583				0.676	0.664	0.207	0.704333	-0.74621	-0.352845665	-0.393366598
908	J3KN75	TBC1D8B	TBC1 domain family member 8B	54885	ENSG00000133138	6	0.446	0.33	0.155	-0.04	-0.02	-0.42	-0.4	-0.05	-0.13	0.2586	0.2232	0.2263	0.1509	-0.416	-0.072	-0.157	0.2597	-0.215	-0.2	-0.88	-0.14	-0.42	-0.21	0.126	-0.09	-0.57	0.126	0.252636	0.237647	-0.042638613	0.28028529
2659	Q5VZK9	CARMIL1	capping protein regulator and myosin 1 linke	55604	ENSG00000079691	6	0.248	0.081	0.039	-0.28	0.499	-0.33	-0.2	0.556	0.343	-0.011	0.0686	0.2266	0.1932	-0.108	0.093	0.1445	0.0007	-0.004	-0.17	-0.6	-0.12	-0.01	0.122	0.013	0.458	0.083	0.312	0.82075	0.097486	0.039531887	0.057953994
3142	Q96HE7	ERO1A	endoplasmic reticulum oxidoreductase 1 alp	30001	ENSG00000197930	5	0.111	-0.59	-0.12	-0.19	-0.38	-0.48	-0.44	0.064	-0.26	0.0965	-0.007	-0.263	-0.865	-0.225	-0.1	-0.176	-0.276	-0.406	0.105	0.933	0.315	-0.31	-0.42	-0.57	0.448	-0.15	-0.46	0.44589	-0.24108	-0.007011638	-0.234071278
2795	Q7Z7B7	DEFB132	defensin beta 132	400830	ENSG00000186458	2	0.033	-0.71	-0.47	0.128	-0.59	0.397	0.499	1.882	-0.44	-0.496	0.9143	-0.255	0.5552	-0.827	0.7513	-0.462	-0.632	-1.675	0.336	0.912	-0.92	-1.92	-1.52	-1.78	-0.8	-0.01	-1.93	0.247684	0.929343	0.317148003	0.612195108
196	Q86Y91	KIF18B	kinesin family member 18B	146909	ENSG00000186185	2	0.474	0.694	0.084				-0.84	-0.82	-0.91	0.2066	0.0333	-0.022				-0.168	-0.876	0.0873	-0.5	-1.44	-0.27				-1.39	0.302	-1.01	0.390559	0.499633	-0.095804659	0.595438041
3316	Q9BYF1	ACE2	angiotensin I converting enzyme 2	59272	ENSG00000130234	12	-0.17	-0.64	0.531	0.146	-0.09	-0.18	-0.56	-0.47	-0.39	-0.587	-0.132	-0.574	0.3071	0.0086	-0.037	-0.289	-0.07	-0.308	0.762	-0	0.627	-0.02	0.293	0.467	-0.57	-0.04	-0.21	0.234208	-0.34678	-0.015590401	-0.331190175
739	Q16527	CSRP2	cysteine and glycine rich protein 2	1466	ENSG00000175183	3	-0.19	-2.18	-1.18	-0.13	-0.42	0.207	-0.03	0.092	-0.02	-0.929	-0.024	-0.882	-0.574	0.2259	0.2393	0.3408	-0.106	0.1788	-0.7	-0.35	-0.38	-0.45	-0.14	-0.06	0.045	0.01	0.496	0.670921	-0.256	-0.256805468	0.00080113
2665	Q5W111	SPRYD7	SPRY domain containing 7	57213	ENSG00000123178	5	-0.06	-0.02	0.34	-0.13	-0.28	-0.05	-0.53	-0.42	-0.16	0.2365	0.1845	-0.017	0.1888	-0.05	0.0226	0.0247	-0.31	-0.203	0.067	-0.13	0.04	-0.32	-0.2	0.16	-0.03	-0.26	-0.57	0.430331	-0.00739	-0.155104084	0.147714
535	C9JVH1	VOPP1	VOPP1 WW domain binding protein;VOPP	81552	ENSG00000154978	2	-0.02	-0.22	-0.18	0.038	0.029	-0.69	-0	-0.25	0.158	0.2438	0.2626	0.0908	0.3079	-0.511	-0.118	0.0821	0.2115	0.4238	-0.42	0.479	0.159	-0.79	-0.59	-0.24	-0.12	0.107	0.116	0.343562	0.017275	-0.237178951	0.2544544
2677	Q68EM7	ARHGAP1	Rho GTPase activating protein 17	55114	ENSG00000140750	3	-0.01	0.076	-0.17	0.061	0.107	-0.38	-0.07	-0.07	0.189	0.0065	-0.332	0.3235	0.2758	-0.12	0.1838	0.0922	0.0401	0.3726	-0.34	-0.42	-0.35	-0.38	-0.19	-0.06	-0.07	0.048	0.367	0.20924	0.127332	-0.121826758	0.249158323
2417	Q13546	RIPK1	receptor interacting serine/threonine kinase	8737	ENSG00000137275	10	-0.17	-0.06	-0.2	-0.08	-0.29	-0.27	-0.15	-0.13	0.075	0.1258	-0.16	-0.041	-0.114	-0.219	-0.069	-0.08	0.1628	0.193	-0.28	-0.57	-0.22	0.355	-0.08	0.083	-0.12	-0.04	0.101	0.561591	-0.0546	-0.118368679	0.063765147
1320	O95969	SCGB1D2	secretoglobin family 1D member 2	10647	ENSG00000124935	2	-0.48	-0.9	0.393				0.084	0.515	0.333	0.1537	0.241	0.1448				0.5918	0.1436	0.447	-0.69	-0.42	0.12				-0.23	0.723	0.171	0.532106	0.043069	-0.296656786	0.339726215
2589	Q50LG9	LRRC24	leucine rich repeat containing 24	441381	ENSG00000254402	1							0.254	0.051	0.297							0.4397	0.1339	0.3859							-0.14	-0.13	0.447	0.574127	0.140154	-0.119316371	0.259470557
3683	Q9UP83	COG5	component of oligomeric golgi complex 5	10466	ENSG00000164597	6	0.286	0.111	-0.06				-0.01	0.205	0.402	0.2298	0.0698	0.1655				0.191	-0.049	-0.153	-0.08	-0.42	-0.02				0.12	0.184	-0.11	0.319396	0.209519	0.079282011	0.130236538
1602	P14209	CD99	CD99 molecule (Xg blood group)	4267	ENSG00000002586	3				-0.21	-0.64	0.822	0.339	0.185	0.285				-0.224	-0.823	-0.351	0.3818	0.459	-0.031				0.48	-0.43	-0.34	0.304	0.004	0.201	0.78077	0.093978	0.228314379	-0.134336414
694	F2Z3E0	PSMG4	proteasome assembly chaperone 4	389362	ENSG00000180822	1	0.16	0.125	-0.1				0.031	-0.06	0.162	-0.247	0.0434	-0.103				-0.243	0.1495	-0.07	-0.37	-1.14	-0.36				-0.12	-0.16	0.193	0.230603	0.378331	0.131143485	0.247187278
3482	Q9NRD9	DUOX1	dual oxidase 1	53905	ENSG00000137857	13	-0.35	0.089	0.033	0.213	0.133	-0.28	0.367	0.727	0.377	-0.199	0.0639	0.1688	0.3432	-0.02	-0.096	0.578	0.3559	0.1851	-0.5	-0.32	-0.28	-0.07	-0.35	-0.26	0.55	0.457	0.224	0.455337	0.20721	-0.008067759	0.215277586
3262	Q9BT73	PSMG3	proteasome assembly chaperone 3	84262	ENSG00000157778	2							0.125	0.51	0.485							0.3487	0.2006	0.2695							0.309	0.245	0.414	0.788547	0.050584	0.100232358	-0.049648672
2994	Q8TED4	SLC37A2	solute carrier family 37 member 2	219855	ENSG00000134955	3	-0.35	0.7	0.372	-0.32	-0.31	-0.79	-0.28	-0.47	-0.12	0.6886	0.148	0.0893	-0.299	0.3424	-0.047	0.343	0.074	0.0928	-0.42	0.75	-0.51	-0.52	-0.55	-0.49	-0.26	0.344	0.268	0.333818	-0.01821	-0.332581015	0.314371083
2662	Q5W041	ARMC3	armadillo repeat containing 3	219681	ENSG00000165309	1							0.176	0.012	0.458							0.2955	0.128	0.2208							0.609	0.227	0.335	0.579895	-0.17471	0.000536458	-0.175243561
534	C9JVE2	DCUN1D1	defective in cullin neddylation 1 domain cont	54165	ENSG00000043093	4				0.345	0.228	-0.01	0.159	0.368	0.209				-0.032	0.2959	0.1382	0.2445	0.3263	0.1172				-0.25	-0.26	0.169	0.387	0.217	0.435	0.790348	0.100797	0.034929846	0.065867598
180	A0A0B4J2A	SHPK;AC	sedoheptulokinase;novel readthrough SHPK	23729	ENSG00000197417	5	-0.99	-0.94	-0.63				-0.03	0.186	0.096	-0.732	-0.635	-0.804				0.3666	0.1346	0.094	-0.81	-0.69	-0.34				0.371	0.14	0.507	0.812283	-0.24793	-0.122683764	-0.125250734
2412	Q13492	PICALM	phosphatidylinositol binding clathrin assemb	8301	ENSG00000073921	4	0.082	0.214	-0.02	0.125	-0.11	-0.67	0.197	0.072	0.57	-0.303	-0.074	0.06	0.0115	-0.105	-0.024	0.6975	0.0984	0.2795	-0.25	-0.75	-0.03	-0.73	-0.26	-0.22	0.426	0.267	0.514	0.635434	0.165174	-0.020687711	0.185861647
2785	Q7Z419	RNF144B	ring finger protein 144B	255488	ENSG00000137393	3	0.075	-0.17	0.16	0.012	-0.42	0.055	-0.22	0.023	-0.05	0.1275	0.4699	0.103	-0.344	0.3542	-0.045	-0.06	-0.045	-0.349	-0.1	-0.7	0.221	-0.4	-0.02	-0.2	0.015	-0.13	-0.23	0.411046	0.112015	-0.083327826	0.195342372
257	F2Z2V1	FGGY	FGGY carbohydrate kinase domain containi	55277	ENSG00000172456	8	0.196	0.164	0.195	0.051	-0.06	-0.18	-0.1	-0.09	0.248	0.2917	0.2543	0.1415	0.0315	-0.012	0.0556	0.2334	0.3104	0.1655	-0.07	-0.74	-0.23	0.126	-0.12	0.027	0.126	0.027	0.174	0.184094	0.123439	-0.115884291	0.239322892
3655	Q9ULE0	WWC3	WWC family member 3	55841	ENSG00000047644	2				-0.05	0.046	-0.13	-1.71	-1.31	-1.03				-0.096	-0.233	0.1182	-1.178	-0.048	-1.171				-0.84	0.021	-0.08	-1.24	-0.7	-0.97	0.828945	-0.06362	-0.262975341	0.199352795
176	A0A0B4J1Y	IGHV3-72	immunoglobulin heavy variable 3-72	28410	ENSG00000225698	4	-1.58	-1.32	-0.76	-0.85	-1.16	-0.69	-2.41	-1.62	-2.27	-1.127	-0.944	-0.392	-0.845	-0.74	-1.063	-0.995	-1.053	-1.911	-1.04	-0.2	2.031	2.77	0.144	0.799	-0.44	-0.18	-3.24	0.132262	-1.47889	-0.399302191	-1.079585887
2643	Q5TA58	AGO1	argonaute RISC component 1;argonaute 1,	26523	ENSG00000092847	3	-1.14	-1.46	-1.43	0.791	-0.29	0.475	-0.36	-0.22	-0.34	-0.812	-0.845	-0.122	0.1248	0.7605	0.2713	0.119	-0.25	0.1054	-1.33	-0.93	0.059	0.445	0.066	-0.09	0.348	-0.37	-0.33	0.61479	-0.20539	-0.369180768	0.163790779
3577	Q9UBB6	NCDN	neurochondrin	23154	ENSG00000020129	5	-0.25	-0.01	0.071	-0.11	0.12	0.251	0.246	0.153	0.181	0.137	-0.226	0.1302	0.0571	-0.329	0.1486	0.1675	0.1617	0.0873	-0.23	-0.41	-0.23	-0.35	0.062	0.229	0.236	-0.01	0.069	0.469285	0.141292	0.035183651	0.106108832
2047	P51668	UBE2D1	ubiquitin conjugating enzyme E2 D1	7321	ENSG00000072401	3				0.223	0.097	-0.21	-0	0.066	0.203				0.0492	-0.247	0.1855	0.419	-0.11	0.1778				-0.2	-0.04	0.006	0.075	0.235	0.224	0.975128	0.012984	-0.016395495	0.02937933
849	Q9Y3D0	CIAO2B	cytosolic iron-sulfur assembly component 2	51647	ENSG00000166595	3	0.269	0.033	-0.2	0.474	1.009	0.605	0.086	0.045	0.132	0.0981	-0.021	0.2432	0.1653	-0.047	0.1783	-0.067	0.1772	0.2566	-0.35	-1.32	-0.39	0.449	0.488	0.146	-0.03	-0.07	0.229	0.310944	0.36641	0.162835454	0.203575039
3658	Q9ULV4	CORO1C	coronin 1C	23603	ENSG00000110880	5	0.082	-0.17	-0.19				0.048	0.085	0.293	0.0662	-0.094	-0.346				0.233	0.0046	0.1724	-0.16	-1.19	0.066				0.145	-0.01	0.126	0.673538	0.194949	0.018012064	0.176937331
1928	P43005	SLC1A1	solute carrier family 1 member 1	6505	ENSG00000106688	3	0.005	-0.06	1.846	1.259	0.366	0.243	-0.27	1.372	-0.21	-0.125	-0.508	-0.277	0.4436	-0.391	-0.278	0.5096	-0.79	0.0639	0.239	-0.26	-0.16	-0.47	-0.25	-0.64	-0.17	-0.42	-0.68	0.094807	0.817698	0.656548478	0.161149948
84	D6RBU4	PPIP5K2	diphosphoinositol pentakisphosphate kinase	23262	ENSG00000145725	4	0.117	0.064	-0.15	0.182	-0.04	-0.16	0.116	0.063	0.311	0.3266	-0.031	-0.023	0.1592	-0.128	-0.218	0.216	0.2875	0.2757	-0	-0.19	-0.08	-0.11	-0.32	-0.1	0.242	0.095	0.315	0.587294	0.071751	-0.04081353	0.112564783
139	A0A0A0MR	PTPN9	protein tyrosine phosphatase non-receptor t	5780	ENSG00000169410	5	0.454	0.659	-0.06	-0.01	0.171	-0.05	-0.64	-0.5	-0.44	0.2659	0.4523	0.1681	0.343	-0.133	0.1644	-0.145	-0.212	-0.339	-0.06	-0.48	-0.08	-0.75	-0.55	0.443	-0.15	-0.3	-0.09	0.40828	0.17832	-0.107872859	0.286192766
2936	Q8NE01	CNNM3	cyclin and CBS domain divalent metal catior	26505	ENSG00000168763	4	0.228	0.573	0.218	-0.41	-0.03	-0.07	-0.18	0.228	-0.07	-0.61	-0.156	0.2895	-0.377	-0.038	-0.154	-0.076	-0.229	-0.113	-0.62	-0.15	-0.21	-0.54	-0.17	-0.14	-0.01	0.022	-0.05	0.221069	0.262411	0.216317142	0.046094113
2979	Q8TD55	PLEKHO2	pleckstrin homology domain containing O2	80301	ENSG00000241839	3	0.039	0.258	0.054				0.282	0.361	0.344	-0.944	0.4584	-0.106				0.415	0.3123	0.2246	-0.06	-1.04	-0.21				0.243	0.21	0.309	0.607609	0.313083	0.163169816	0.149913509
2316	Q05513	PRKCZ	protein kinase C zeta	5590	ENSG00000067606	7	0.08	0.43	-0.28	0.05	-0.21	-0.12	-0.11	0.017	-0	0.2018	0.0874	0.0701	0.1076	-0.523	0.1342	-0.022	-0.041	0.0241	-0.46	-0.63	-0.34	0.481	-0.03	0.041	-0.02	0.003	0.054	0.76262	0.083719	-0.020860204	0.104579553
1295	X6R5A3	TDP2	tyrosyl-DNA phosphodiesterase 2	51567	ENSG00000111802	1							-0.13	-0.03	0.044							0.0224	-0.384	-0.161							0.138	-0.08	0.069	0.408374	-0.08011	0.136363792	-0.21646979
2616	Q5TA12	DOP1A	DOP1 leucine zipper like protein A	23033	ENSG00000083097	8	0.135	0.409	0.057	0.203	0.197	-0.21	0.022	0.112	0.353	0.3651	0.2067	0.2941	0.0245	-0.195	-0.214	0.4954	-0.125	0.4943	-0.31	-0.83	-0.07	-0.11	-0.19	-0.31	0.108	0.033	0.243	0.147903	0.301693	-0.007765598	0.30945889
1917	P42336	PIK3CA	phosphatidylinositol-4,5-bisphosphate 3-kin	5290	ENSG00000121879	2							-0.18	0.026	-0.31							-0.335	-0.644	-0.457							0.01	-0.28	-0.53	0.389087	0.113908	0.323043591	-0.209135551
1425	P04843	RPN1	ribophorin I	6184	ENSG00000163902	3				0.326	0.946	0.301	0.448	0.377	0.109				0.2159	-0.771	-0.121	-0.107	-0.5	-0.374				0.222	0.051	-0.74	0.196	-0.2	-0.43	0.084514	0.567317	0.694103775	-0.126787161
1195	O75170	PPP6R2	protein phosphatase 6 regulatory subunit 2	9701	ENSG00000100239	6	-0.06	-0.13	-0.14	0.082	0.353	-0.38	-0.39	-0.37	-0.15	0.2703	-0.015	-3E-04	0.4138	-0.122	-0.074	-0.284	0.4679	0.1113	-0.3	-0.31	0.052	-0.41	-0.12	0.189	-0.24	-0.18	-0.16	0.195048	0.03198	-0.216488574	0.248468234
2353	Q0VDG4	SCRN3	secernin 3	79634	ENSG00000144306	5	0.186	-0.07	0.155	0.08	0.025	-0.26	-0.17	-0.1	-0.2	0.1789	-0.085	-0.043	0.0759	-0.121	0.2403	-0.047	-0.229	-0.036	-0.16	-0.65	-0.3	-0.53	-0.15	0.064	-0.01	0.008	-0.1	0.221156	0.162563	-0.031989556	0.194552932
875	H7BYD9	PAM	peptidylglycine alpha-amidating monooxyger	5066	ENSG00000145730	2	0.114	-0.09	0.389				-0.02	0.187	0.07	0.6809	0.3138	0.7917				0.1676	-0.223	-0.303	-0.19	-0.53	0.143				0.49	0.061	-0.16	0.562602	0.141093	-0.12854039	0.269632935
3235	Q99969	RARRES2	retinoic acid receptor responder 2	5919	ENSG00000106538	2	-0.88	-1.04	-0.88	0.453	-1.13	-0.5	0.167	0.676	0.783	-0.503	-0.515	-0.043	-0.27	-1.003	-0.353	0.1389	-0.904	-0.179	-1.02	-0.49	-0.53	-0.79	-0.51	-0.57	0.416	0.689	0.728	0.883581	-0.03047	0.142653169	-0.173122292
884	O75312	ZPR1	ZPR1 zinc finger	8882	ENSG00000109917	4	0.078	0.185	0.016	-0.17	-0.41	-0.64	0.05	0.152	0.187	0.4817	0.0194	0.0806	-0.276	-0.253	-0.302	0.0428	0.0967	0.0165	0.122	-0.51	-0	-0.48	-0.39	-0.35	0.228	-0.13	0.091	0.645672	0.095456	-0.051533193	0.146988887
1109	O43286	B4GALT5	beta-1,4-galactosyltransferase 5	9334	ENSG00000158470	2	-0.26	-1.01	-0.27	-0.47	-0.3	-0.11	0.009	0.116	0.58	-0.07	-0.221	0.0273	-0.426	-0.091	-0.334	0.5947	0.0437	0.2555	-0.27	-0.24	0.217	0.442	-0.48	0.117	0.217	-0	0.261	0.550214	-0.22014	-0.166539981	-0.053598033

867	H3BVG0	NUP93	nucleoporin 93	9688	ENSG00000102900	9	0.215	0.335	0.303	0.018	0.029	-0.18	-0.5	0.002	0.091	0.0197	0.0208	0.0805	0.0271	-0.021	0.0907	0.0383	0.0127	0.0052	0.026	-0.71	-0.28	-0.26	-0.21	0.044	-0.05	-0.13	0.059	0.209723	0.202855	0.004691463	0.198164018
3191	Q96S99	PLEKHF1	pleckstrin homology and FYVE domain cont	79156	ENSG00000166289	2							0.064	0.183	0.254							0.1398	0.0006	0.1867							0.033	-0.06	0.112	0.425253	0.138091	0.057725786	0.080365411
299	F5GYH1	NECAP1	NECAP endocytosis associated 1	25977	ENSG00000089818	4				-0.08	-0.05	-0.43	0.369	0.254	0.661				0.1721	-0.079	0.1482	0.5937	0.5368	0.6383				-0.74	-0.38	-0.01	0.025	0.224	0.616	0.432195	0.163414	-0.215817641	0.379231743
2538	Q15814	TBCC	tubulin folding cofactor C	6903	ENSG00000124659	7	-0.06	0.229	0.09	-0.04	0.13	-0.3	0.016	-0.01	0.293	0.0225	-0.103	-0.115	0.0075	-0.288	0.1196	0.2562	0.184	0.1953	-0.3	-0.9	-0.44	-0.54	-0.3	-0.07	0.191	-0.12	0.126	0.131155	0.29945	0.007437613	0.292012153
1807	P30419	NMT1	N-myristoyltransferase 1	4836	ENSG00000136448	6	0.321	0.523	-0	-0.06	0.151	-0.01	-0.28	0.146	0.143	0.2228	0.1555	-0.005	0.2849	-0.038	0.0216	0.0489	0.3779	0.0624	-0.28	-1.04	-0.05	-0.05	-0.16	0.207	-0.08	0.05	0.289	0.261205	0.22895	-0.021697036	0.250646621
3186	Q96RL1	UIMC1	ubiquitin interaction motif containing 1	51720	ENSG00000087206	1							-1.63	1.979	-1.41							0.1699	-1.371	0.33							1.103	0.819	0.257	0.678457	-1.0817	-0.065095494	-1.016603122
2464	Q14554	PDIA5	protein disulfide isomerase family A membe	10954	ENSG00000065485	4				0.038	0.094	0.169	-0.13	-0.09	0.079				-0.008	-0.156	-0.214	-0.248	-0.354	-0.068				2.233	0.29	0.257	0.175	0.098	0.249	0.18347	-0.52358	0.201541811	-0.725126556
212	Q8N6H7	ARFGAP2	ADP ribosylation factor GTPase activating p	84364	ENSG00000149182	5	0.258	0.441	-0.03	0.02	0.61	0.012	-0.47	-0.27	-0.02	0.247	0.3	0.0765	0.3347	-0.265	0.1318	-0.183	0.1608	-0.022	-0.12	-0.94	-0.21	0.103	0.412	0.091	-0.2	-0.2	-0.2	0.415683	0.201082	-0.025160967	0.226242711
983	M0R165	EPS15L1	epidermal growth factor receptor pathway su	58513	ENSG00000127527	4				-0.06	-0.02	-0.24	0.204	0.058	0.093				0.1977	0.0739	0.372	0.2193	0.0287	0.1462				-0.11	-0.36	0.015	0.085	0.026	0.302	0.305885	0.013451	-0.16676994	0.180220659
467	B7ZKJ8	ITIH4	inter-alpha-trypsin inhibitor heavy chain 4;int	3700	ENSG00000055955	8	-0.76	-0.2	-0.68	-0.35	-0.16	0.123	0.07	0.3	-0.33	0.1205	-0.001	-0.226	-0.458	0.0423	-0.467	0.1627	-0.536	-0.39	-0.22	0.34	1.084	0.979	0.14	0.135	0.534	0.019	-0.46	0.125818	-0.50441	-0.025482289	-0.478928556
166	A0A0A0MTL	RIPOR1	RHO family interacting cell polarization regu	79567	ENSG00000039523	3	-0.12	0.198	-0.32				0.063	0.186	0.216	0.0374	0.0749	0.1156				0.6353	0.0708	0.2507	-0.24	-0.21	-0.47				0.152	0.138	0.069	0.274122	0.130744	-0.159706727	0.290451206
2133	P60484	PTEN	phosphatase and tensin homolog	5728	ENSG00000171862	3	0.3	0.237	0.046				0.269	0.072	0.536	-4E-04	0.1178	0.1873				0.2952	-0.141	0.0196	-0.23	-1.02	0.015				0.082	0.221	0.248	0.316026	0.356595	0.163475527	0.193118995
1257	O94819	KBTBD11	kelch repeat and BTB domain containing 11	9920	ENSG00000273645	6	-0.04	0.076	0.018	-0	0.238	-0.52	0.448	0.089	0.578	-0.063	0.1367	0.1412	0.4414	0.1853	0.2962	0.4297	0.4832	0.2555	-0.09	-0.2	-0.13	-0.25	-0.01	0.113	0.382	0.448	-0.12	0.294379	0.083237	-0.158304142	0.241541467
76	A0A087WYU	METTL26	methyltransferase like 26	84326	ENSG00000130731	4	0.122	0.022	0.04				0.25	0.241	0.283	0.2123	0.1409	-0.015				0.366	0.0329	0.0615	0.222	0.08	-0.07				0.228	-0.05	0.277	0.900959	0.044483	0.026549353	0.017933989
3557	Q9P000	COMMD9	COMM domain containing 9	29099	ENSG00000110442	7	-0.14	0.076	-0.02	0.023	-0.02	-0.47	-0.14	0.125	-0.04	-0.124	0.0764	-0.072	0.0078	-0.072	0.1683	-0.032	0.1759	0.2002	-0.1	-0.67	0.015	-0.28	-0.33	-0.06	0.028	-0.07	0.035	0.23027	0.091155	-0.104054445	0.195209243
3259	Q9BST9	RTKN	rhotekin	6242	ENSG00000114993	2	-0.43	-0.05	-0.2	-0.54	0.169	-0.7	-0.13	-0.25	-0	-0.013	-0.42	0.048	-0.488	-0.199	-0.42	0.2679	-0.033	0.0365	-0.49	-0.92	-0.6	-0.3	-0.38	-0.16	-0.04	-0.05	-0.16	0.445822	0.110422	-0.099903702	0.210325749
2859	Q8IXL6	FAM20C	FAM20C golgi associated secretory pathwa	56975	ENSG00000177706	9	0.026	-0.11	-0.33	-0.39	0.918	-0.24	0.128	0.191	0.044	-0.083	-0.477	-0.138	-0.267	-0.124	0.3535	0.4477	0.4705	0.1527	-0.34	-0.06	0.169	0.164	0.162	0.255	0.453	0.198	0.081	0.865527	-0.09499	-0.011746362	-0.083245164
110	A0A087X1P	PCDHGC3	protocadherin gamma subfamily C, 3	5098	ENSG00000240184	1	0.007	-0.13	-0	0.071	-0	0.224	-0.28	0.147	-0.33	-0.207	0.3875	0.46	-0.283	-0.151	-0.265	0.199	-0.067	0.4379	-0.25	-0.27	0.28	-0.56	-0.72	-0.15	-0.09	-0.12	0.283	0.390015	0.144938	-0.0893138	0.234251497
324	A0A2R8Y6C	TBCE	tubulin folding cofactor E	6905	ENSG00000284770	4				0.07	0.062	0.051	0.028	-0.03	-0.03				-0.012	-0.251	-0.061	0.0679	-0.023	-0.089				-0.13	0.155	0.209	0.11	0.208	-0.05	0.227404	-0.05638	0.088002484	-0.14438747
832	H0YLB9	MAN2A2	mannosidase alpha class 2A member 2	4122	ENSG00000196547	5	0.15	-0.11	0.011				-0.88	-0.26	-0.44	0.1705	-0.048	0.2564				-0.225	-1.069	-0.76	-0.05	0.415	0.142				-0.12	-0.17	-0.53	0.700636	-0.20544	0.023849869	-0.229292778
1810	P30464	HLA-B	major histocompatibility complex, class I, B	3106	ENSG00000232126	9	0.014	0.678	0.933	-1.6	-0.87	-0.88	-0.62	-0.43	-0.89	-0.417	-0.614	0.9066	-0.132	0.3835	-0.22	-0.473	0.0301	-0.531	0.07	-0.45	0.403	0.199	0.391	-0.32	0.57	-0.11	-0.24	0.414091	-0.46566	-0.289180697	-0.176477017
512	C9JG97	AAMP	angio associated migratory cell protein	14	ENSG00000127837	5	-0.11	-0.03	0.107	0.105	0.02	-0.34	0.719	0.314	0.35	0.0057	-0.258	-0.091	0.0712	0.0302	0.2159	0.2395	0.3393	0.3643	-0.26	-0.27	0.013	-0.45	-0.07	0.089	0.11	-0	0.508	0.541794	0.161724	0.023334076	0.138390287
21	A0A075B7D	AC135068	novel gene identicle to IGHV1OR15-1;immu	388077	ENSG00000281179	1	-1.1	-0.61	-0.47				-0.13	-0.53	-0.68	-0.592	-0.746	0.0313				0.1496	-0.707	-0.651	-0.76	-0.87	1.636				0.429	-0.21	-1.28	0.702936	-0.4104	-0.166715998	-0.243679893
3545	Q9NZ32	ACTR10	actin related protein 10;actin related protein	55860	ENSG00000131966	7	-0.06	-0.11	-0.12	0.05	0.227	-0.13	0.277	0.295	0.491	0.0715	-0.01	0.0024	0.3394	-0.154	0.1692	0.4123	0.2311	0.3317	-0.24	-0.57	-0.22	0.025	-0.08	0.273	0.446	0.347	0.383	0.7623	0.060622	-0.053598713	0.114221087
757	F8W9W8	SLCO2A1	solute carrier organic anion transporter famil	6578;80111	ENSG00000174640	3	-0.67	-0.26	0.295	-0.68	-0.18	0.662	0.1	0.505	-0.08	-0.16	0.376	-0.342	-0.912	0.4667	-0.449	-0.415	0.343	0.4224	-0.26	0.301	-0.16	-0.43	-0.1	-0.36	0.907	-0.26	-0.15	0.987724	0.023544	0.040903854	-0.017359678
469	B7ZL14	FNBP1	formin binding protein 1	23048	ENSG00000187239	2	0.184	-0.11	-0.52	0.32	0.31	-0.18	0.557	0.56	0.389	0.027	0.0417	-0.324	0.2467	0.458	0.32	0.6212	0.2729	0.447	-0.32	-0.84	-0.08	0.324	0.155	0.223	0.454	0.307	0.659	0.825096	0.068868	-0.067620282	0.136488427
3255	Q9BRX2	PELO	pelota mRNA surveillance and ribosome res	53918	ENSG00000152684	4	0.018	0.108	0.075	0.301	-0.15	-0.23	0.129	0.178	0.215	0.2044	-0.361	0.124	0.0242	0.0045	0.2513	0.0739	0.1761	0.274	-0.43	-0.82	-0.31	-0.37	-0.28	0.007	-0.17	0.049	0.206	0.101013	0.306986	-0.014161727	0.321148224
48	A0A087WVF	FAT1	FAT atypical cadherin 1	2195	ENSG00000083857	10	-0.36	0.302	0.036	-0.11	0.3	-0.44	-0.35	0.294	-0.56	0.5268	0.2898	-0.034	0.3327	-0.161	0.0227	0.1466	0.3141	0.0553	-0.34	-0.17	-0.14	-0.43	-0.36	-0.14	0.228	-0.09	-0.53	0.120949	0.11871	-0.26459841	0.383308346
330	H3BS66	SMIM1	small integral membrane protein 1 (Vel bloo	388588	ENSG00000235169	3	-0.24	0.646	0.563				0.336	0.164	0.392	-0.133	0.179	0.2141				0.5295	0.1221	0.2169	-0.47	-0.39	-0.34				0.481	0.424	0.263	0.449361	0.316059	0.122039288	0.194019852
3318	Q9BYM8	RBCK1	RANBP2-type and C3HC4-type zinc finger	10616	ENSG00000125826	6				-0.09	0.077	-0.37	0.016	-0.04	-0.02				0.3766	-0.112	0.1232	0.0638	-0.267	-0.19				0.013	0.206	0.142	0.359	-0.17	-0.07	0.582828	-0.14994	-0.068548557	-0.081395113
479	B9A049	WDR54	WD repeat domain 54	84058	ENSG00000005448	3	0.393	0.427	-0.2				0.002	0.11	0.137	-0.034	0.078	0.3863				0.1406	0.0364	0.1251	-0.42	-0.71	-0.09				0.166	0.068	0.105	0.269828	0.291734	0.022861605	0.26887282
371	A6NDG6	PGP	phosphoglycolate phosphatase	283871	ENSG00000184207	5				0.242	0.253	0.131	0.63	0.788	0.597				-0.273	0.2156	0.0007	0.4656	-0.654	0.213				-0.17	-0.05	-0.06	0.555	0.32	0.125	0.209929	0.320784	0.44540966	-0.124625654
1894	P40222	TXLNA	taxilin alpha	200081	ENSG00000084652	3	-0.23	-1.71	-0.44	0.038	-0.64	-0.34	0.585	0.306	0.479	-0.337	0.3784	-0.566	-0.404	0.4442	0.3457	0.0844	0.1167	0.051	-0.39	0.185	0.105	-0.25	-0.27	-0.06	0.756	-0.03	0.262	0.635123	-0.25174	-0.230155616	-0.021582803
3531	Q9NX57	RAB20	RAB20, member RAS oncogene family	55647	ENSG00000139832	4	0.016	-0.06	0.138				0.328	-0.12	0.308	0.0508	0.0283	-0.039				0.0878	0.0067	0.0094	-0.07	-0.71	0.425				-0.1	-0.13	0.076	0.566825	0.187166	0.077601539	0.109564497
317	A0A2R8Y53	ANO4	anoctamin 4	121601	ENSG00000151572	2	-0.23	0.366	0.319				-0.81	-0.23	-0.5	0.1941	0.0876	-0.29				-0.747	0.2327	-0.455	0.329	1.468	-0.13				-0.13	-0.1	-0.67	0.678528	-0.30915	-0.018015012	-0.291133324
1002	O00308	WWP2	WW domain containing E3 ubiquitin protein	11060	ENSG00000198373	8	0.185	0.673	-0				-0.05	0.259	0.211	0.1054	0.0816	0.4103				0.1026	0.1108	0.1402	-0.59	-0.63	-0.24				-0.01	0.084	0.427	0.223315	0.370599	0.054056314	0.316543123
3273	Q9BUF7	CRB3	crumbs cell polarity complex component 3;c	92359	ENSG00000130545	4	-0.27	0.068	-0.38	0.233	-0.05	-0.31	-0.33	-0.08	-0.41	-0.129	-0.009	0.1875	0.1583	0.0589	0.0646	0.2856	-0.052	0.3164	-0.08	0.355	-0.21	-0.46	-0.23	-0.14	-0.43	-0.15	0.334	0.173573	-0.05566	-0.26606054	0.21040142
877	H7BYW6	PDGFA	platelet derived growth factor subunit A	5154	ENSG00000197461	3	-0.45	-0.31	-0.43	0.179	-0.46	-0.29	0.029	-0.05	0.085	0.3289	-0.235	0.1372	-0.156	-0.021	-0.533	0.6271	0.1432	0.0573	-0.15	0.382	-0.01	-0.27	-0.24	-0.04	0.231	0.287	-0.4	0.399987	-0.1657	-0.227571521	0.061871708
3087	Q96A83	COL26A1	collagen type XXVI alpha 1 chain	136227	ENSG00000160963	2	0.129	0.048	0.063				-0.17	0.459	0.443	-0.19	-0.097	-0.305				-0.141	0.1001	0.7405	0.902	-0.15	0.289				0.652	0.174	0.69	0.301159	-0.26509	0.143294702	-0.408381004
2349	Q09328	MGAT5	alpha-1,6-mannosylglycoprotein 6-beta-N-ac	4249	ENSG00000152127	4	0.427	0.106	-0.12	0.157	0.097	-0.52	0.679	1.569	0.237	0.3928	0.2104	0.1519	0.0514	-0.15	-0.206	1.5907	0.6087	0.6287	0.086	0.237	0.214	0.517	-0.03	-0.01	-1.03	-0.15	1.181	0.743689	0.180016	-0.071613147	0.251628847
817	H0YBG8	ADAM28	ADAM metallopeptidase domain 28	10863	ENSG00000042980	1	-0.25	-0.8	1.058	0.629	0.159	-0.87	0.345	0.566	-0.44	0.5746	-0.418	0.6049	0.1049	0.0072	-0.522	-0.029	-0.126	-0.781	-0.28	-0.19	0.157	-0.22	-0.13	0.184	0.072	0.232	-0.18	0.919605	0.084187	0.109927984	-0.025740958
1719	P22612	PRKACG	protein kinase cAMP-activated catalytic sub	5568	ENSG00000165059	5	0.082	0.198	-0.15	0.425	0.078	-0.46	0.537	0.133	0.376	-0.548	-0.218	-0.182	-0.216	-0.62	-0.254	0.0994	0.2532	0.3509	-0.35	-1	-0.29	-0.72	-0.67	-0.34	0.088	-0.11	0.083	0.118153	0.503311	0.283419546	0.219891376
765	F8WBU7	PNCK	pregcy up-regulated nonubiquitous CaM kin	139728	ENSG00000130822	1							0.451	0.257	0.358							0.2163	0.3964	0.2811							1.265	0.357	-0.92	0.981403	0.122356	0.057549914	0.064806322
754	F8W7W8	HFE	homeostatic iron regulator	3077	ENSG00000010704	4							-0.22	0.001	0.211							-0.021	-0.118	-0.177							0.088	0.289	-0.01	0.451276	-0.12585	0.10150031	-0.227355257
1692	P20340	RAB6A	RAB6A, member RAS oncogene family	5870	ENSG00000175582	19	-0.25	0.785	-0.14				-0.05	-0.13	0.016	0.1775	-0.137	0.0069				-0.228	0.2663	-0.195	-0.24	-1.18	-0.18				-0.47	-0.12	0.075	0.309053	0.390413	0.056423125	0.333990095
630	E9PC41	IL1RL1	interleukin 1 receptor like 1	9173	ENSG00000115602	9	-1.21	-1.42	-0.34	-0.11	-0.93	-0.09	-0.54	0.961	0.317	-0.193	0.9342	0.2427	-0.394	0.381	-0.243	0.4311	-0.682	-0.227	0.331	0.094	0.427	0.111	0.056	-0.06	1.185	-0.13	-0.2	0.261099	-0.57443	-0.400595579	-0.17383397
1376	P01877	IGHA2	immunoglobulin heavy constant alpha 2 (A2	3494	ENSG00000211890	15	-1.3	-1.31	-1.45	-0.54	-1.49	-0.64	-2.25	-2.36	-2.04	-1.431	-0.291	-1.009	-1.134	-0.729	-0.682	-2.157	-1.834	-2.536	-1.26	-0.58	2.854	2.347	1.042	1.616	-0.77	-2.44	-2.04	0.137255	-1.57518	-0.177277012	-1.397900406
61	A0A087WW	BCL10	BCL10 immune signaling adaptor	8915	ENSG00000142867	3	0.111	0.405	-0.07	-0.26	0.073	-0.56	0.13	0.195	0.126	0.3161	-0.301	0.1449	0.0876	0.0478	0.1928	0.1799	0.2446	0.0502	-0.05	-0.83	-0.15	-0.42	-0.33	0.221	0.298	0.044	0.385	0.523144	0.110051	-0.090658266	0.200709621
369	H0Y984	BST1	bone marrow stromal cell antigen 1	683	ENSG00000109743	3	-0.02	0.2	0.138				-0.4	0.022	-0.15	-0.027	0.2663	-0.17				0.2076	0.035	-0.038	-0.2	0.123	0.207				0.084	0.191	0.066	0.658079	-0.11436	-0.081065911	-0.033293169
3110	Q96CV9	OPTN	optineurin	10133	ENSG00000123240	3	-0.28	0.169	-0.21	-0.08	-0.15	-0.29	-0.62	1.353	-0.26	0.1324	-0.003	-0.006	-0.105	-0.058	0.2235	0.042	-0.222	0.0563	-0.16	-0.51	-0.07	-0.35	-0.19	-0.24	0.392	0.258	0.373	0.958165	0.016124	-0.046514885	0.062639321
3040	Q92625	ANKS1A	ankyrin repeat and sterile alpha motif domai	23294	ENSG00000064999	4	0.009	0.089	-0.02				-0.49	-0.36	-0.21	-0.136	-0.13	0.1105				-0.13	-0.607	-0.593	-0.43	-0.54	-0.47				0.419	-0.39	-0.3	0.844171	0.121878	0.08443159	0.03744669
78	A0A087WZ4	MAP7	microtubule associated protein 7	9053	ENSG00000135525	4				-0.28	-0.1	-0.45	0.514	0.23	0.4				-0.355	0.1089	-0.031	0.0768	0.1693	0.133				-0.7	-0.19	-0.35	0.311	0.448	0.511	0.983066	0.047276	0.034521929	0.012753639
3315	Q9BYC5	FUT8	fucosyltransferase 8	2530	ENSG00000033170	7	-1.04	2.324	-1.03	-0.24	0.255	-0.64	-0.25	-0.46	-0.41	0.5114	-1.065	0.488	-0.14	0.2315	-0.246	-0.521	-0.619	-0.182	1.212	-0.2	0.429	-0.36	-0.23	0.016	0.313	0.127	-0.76	0.827209	-0.22767	0.004824478	-0.232496898
3458	Q9NPP4	NLRC4	NLR family CARD domain containing 4	58484	ENSG00000091106	1	0.332	0.29	0.172	-0.09	0.017	-0.54	-0.42	-0.36	-0.09	0.4354	0.3964	0.0665	0.2635	-0.11	-0.051	-0.256	0.2036	######	-0.2	-1.26	-0.14	-0.33	-0.43	0.164	-0.4	-0.25	-0.33	0.12021	0.276836	-0.182392776	0.459228565
1071	O15111	CHUK	component of inhibitor of nuclear factor kap	1147	ENSG00000213341	3							0.01	0.117	0.068							-0.132	0.1953	0.0269							0.118	0.145	0.137	0.616682	-0.06825	0.034841846	-0.10308975
2637	Q5T4F7	SFRP5	secreted frizzled related protein 5	6425	ENSG00000120057	4	0.094	-0.21	-0.22	-1.16	-0.64	-0.31	-0.54	0.034	-0.21	0.7353	-0.307	0.0312	-0.074	-0.556	-0.28	0.7444	-0.855	-0.289	0.115	0.728	0.19	0.56	0.499	0.511	-0.57	-0.08	-0.51	0.225853	-0.51157	-0.256402165	-0.255166258
419	B1ANH5	GUK1	guanylate kinase 1	2987	ENSG00000143774	4	0.118	0.185	0.036	-0.13	-0.21	-0.24	-0.06	-0.13	-0.08	0.281	0.0255	-0.003	0.0109	0.1525	0.1717	0.1436	0.2366	-0.292	-0.19	-0.94	-0.19	-0.26	-0.3	-0	-0.17	-0.01	-0.32	0.082124	0.208152	-0.13745004	0.34560166
18	A0A075B73	EPPK1	epiplakin 1	83481	ENSG00000261150	9	-0.62	-0.94	-1.04	0.207	0.163	-0.21	-0.18	-0.52	0.024	-0.957	-0.495	-0.783	-0.378	-0.565	0.0771	-0.05	-0.11	-0.155	-1.26	-0.71	-0.74	-0.74	-0.22	-0.11	-0.11	-0.15	-0.24	0.855098	0.129589	0.034005397	0.095583734
1192	O75146	HIP1R	huntingtin interacting protein 1 related	9026	ENSG00000130787	2	0.162	0.439	-0.25	-0.06	0.41	-0.21	-0.14	0.319	0.127	-0.123	-0.013	0.1649	-0.326	-0.285	-0.193	0.125	0.1736	0.0423	-0.15	-0.7	-0.13	-0.53	-0.36	-0.41	0.346	0.055	0.125	0.250945	0.282686	0.135163877	0.14752198
2675	Q68D91	MBLAC2	metallo-beta-lactamase domain containing 2	153364	ENSG00000176055	4				0.059	0.121	-0.32	-0.05	-0.08	-0.27				0.0629	-0.587	0.2659	0.087	0.0489	-0.142				-0.7	-0.32	0.062	0.021	0.021	0.194	0.926185	0.030936	-0.04517472	0.076110519
453	B5MCE7	BZW2	basic leucine zipper and W2 domains 2	28969	ENSG00000136261	5	0.132	0.253	-0.02	0.315	0.223	-0.06	-0.34	-0.14	-0.35	0.4951	0.4633	0.1521	0.3136	-0.061	0.3041	-0.327	-0.057	-0.419	-0.19	-0.36	-0.22	0.047	-0.23	0.346	0.062	-0.23	-0.15	0.467508	0.104893	-0.09398713	0.198880243
2627	Q5SW96	LDLRAP1	low density lipoprotein receptor adaptor prot	26119	ENSG00000157978	2							-0.04	0.026	0.19							0.2407	0.0742	-0.157							0.121	0.246	0.107	0.727278	-0.09965	0.005676984	-0.105329176
478	F8WCJ3	TXNDC9	thioredoxin domain containing 9	10190	ENSG00000115514	2	0.342	0.241	-0.06				-0.79	-0.33	-0.59	0.2031	0.2904	0.0466				-0.628	-0.119	-0.05	-0.02	-1.09	-0.28				-0.41	0.127	-0.14	0.6733	0.105349	-0.153226495	0.258575889
3243	Q9BQS8	FYCO1	FYVE and coiled-coil domain containing 1	79443	ENSG00000163820	6				-0.01	-0.05	-0.13	0.121	0.087	0.277				-0.035	0.0497	0.107	0.0064	0.1831	0.3372				-0.22	-0.15	0.021	0.339	-0.02	0.635	0.918636	-0.05358	-0.059148787	0.005570439
949	K7EJH8	ACTN4	actinin alpha 4	81	ENSG00000130402	11	0.177	0.034	-0.04				0.431	0.169	0.509	0.2831	-0.014	-0.135				0.6562	0.1487	0.2616	-0.17	-1.07	-0.38				0.444	-0.1	0.203	0.290906	0.392726	0.013830243	0.37889532
751	F8VYN9	ARL1	ADP ribosylation factor like GTPase 1	400	ENSG00000120805	7	-0.08	0.019	-0.2	-0.02	0.162	-0.5	0.125	0.382	0.351	0.2761	-0.078	0.037	0.2181	-0.027	0.1502	0.313	0.1287	0.4448	0.334	0.192	-0.16	-0.26	-0.16	0.154	-0	0.122	0.302	0.56229	-0.03059	-0.135842686	0.105252765
3684	Q9UPN7	PPP6R1	protein phosphatase 6 regulatory subunit 1	22870	ENSG00000105063	2				-0.1	-0.25	-0.81	0.064	-0.34	-0.11				-0.138	-0.306	-0.072	-0.201	-0.026	0.155				-0.25	-0.04	-0.39	0.07	-0.2	0.031	0.581199	-0.12756	-0.160454564	0.032895661
2569	Q2M3G4	SHROOM1	shroom family member 1	134549	ENSG00000164403	5	0.184	-0.26	-0.06	-0.34	-0.09	-0.6	0.075	-0.39	-0.38	0.8855	0.1081	0.1551	0.0126	-0.317	-0.17	-0.051	0.1688	-0.145	-0.33	-0.82	-0.19	-0.13	-0.18	0.309	-0.2	-0.21	-0.07	0.2342	-0.00295	-0.278068381	0.275118088
2661	Q5VZU9	TPP2	tripeptidyl peptidase 2	7174	ENSG00000134900	48							0.152	0.251	0.232							0.1808	-0.089	0.1578							0.227	-0.1	0.152	0.611705	0.117158	0.128312642	-0.011154438
1673	P19474	TRIM21	tripartite motif containing 21	6737	ENSG00000132109	5	-0.17	-0.02	-0.07	-0.02	-0.01	-0.15	0.079	0.197	0.26	-0.171	-0.28	0.1608	0.14	0.2419	0.1892	0.1076	0.5235	0.2856	-0.1	-0.49	-0.21	-0.04	-0.07	0.105	0.348	0.11	0.466	0.591893	-0.00354	-0.122688146	0.119152336
3277	Q9BUQ8	DDX23	DEAD-box helicase 23	9416	ENSG00000174243	4	-0.32	-0.27	-0.28	-0.45	0.027	-0.39	-0	0.152	0.497	0.1722	0.1573	-0.208	-0.234	0.1936	-0.324	0.5669	0.9395	0.7729	0.082	0.485	0.272	0.005	-0.01	-0.07	0.093	0.568	0.599	0.212718	-0.3398	-0.341257644	0.001455221
513	C9JEX3	MTMR2	myotubularin related protein 2	8898	ENSG00000087053	6	0.242	0.384	0.11	-0	0.08	-0.33	-0.3	-0.07	0.05	0.3934	-0.011	0.3314	0.0868	-0.186	0.1824	0.2211	-0.023	0.0119	-0.13	-0.19	0.023	0.437	-0.24	0.089	0.16	-0.02	0.127	0.698347	-0.00933	-0.093896505	0.084566715
3213	Q99576	TSC22D3	TSC22 domain family member 3	1831	ENSG00000157514	4							-0.08	0.384	0.59							0.0099	0.5343	0.5493							0.294	0.256	0.394	0.967789	-0.01776	-0.067841381	0.050081415
103	A0A087X1A	STAU1	staufen double-stranded RNA binding prote	6780	ENSG00000124214	2	-0.05	0.184	-0.27				0.109	0.118	0.286	-0.13	0.2067	-0.007				-0.055	-0.457	-0.188	0.22	0.028	0.071				0.762	-0.21	-0.02	0.427545	-0.07968	0.167155287	-0.246838503
2820	Q86X29	LSR	lipolysis stimulated lipoprotein receptor	51599	ENSG00000105699	16							-0.25	0.289	-0.12							0.0646	-0.697	-0.233							0.567	0.28	-0.2	0.450942	-0.24525	0.258868821	-0.504122317
928	J3KST3	CPTP	ceramide-1-phosphate transfer protein	80772	ENSG00000224051	3	-0	0.216	-0.05	-0.74	-0.62	-0.33	-0.16	-0.26	0.266	-0.001	0.2306	0.1753	-0.145	-0.117	-0.169	0.3012	0.084	0.1617	-0.06	-0.5	-0.29	-0.19	-0.41	-0.02	-0.05	0.11	0.241	0.287191	-0.05595	-0.244284145	0.188331884
3244	Q9BQT9	CLSTN3	calsyntenin 3	9746	ENSG00000139182	4	-0.07	-0.22	-0.11	0.23	0.078	-0.09	-0.05	0.348	0.063	0.6419	0.1952	0.4631	-0.008	-0.108	-0.499	0.3555	-0.606	-0.149	0.096	0.695	0.535	0.236	0.057	-0.07	0.544	0.259	-0.42	0.544819	-0.19423	-0.011992051	-0.182233899
810	H0Y8B3	AHCYL2	adenosylhomocysteinase like 2	23382	ENSG00000158467	15	0.251	0.338	0.072				0.08	0.454	0.414	0.1934	0.0017	0.1362				0.2366	0.0281	0.2469	-0.01	-0.49	-0.17				0.274	0.129	0.311	0.274387	0.260321	0.127662166	0.132659322
470	C9JEL3	EIF4E2	eukaryotic translation initiation factor 4E fam	9470	ENSG00000135930	2				0.148	0.188	-0.09	0.267	0.393	0.361				0.2013	-0.518	0.3152	0.4967	-0.548	-0.077				-0.03	0.178	0.108	0.529	0.321	0.324	0.432547	-0.02656	0.233412181	-0.259972781

1750	P25774	CTSS	cathepsin S	1520	ENSG00000163131	5	-0.11	-0.46	-0.01	0.031	-1.1	-0.12	-0.2	0.353	0.107	0.6139	0.3524	0.258	-0.364	0.3962	-0.199	0.6017	-0.247	-0.386	-0.12	0.036	0.156	0.665	0.072	-0.21	0.935	0.535	-0.28	0.321556	-0.36538	-0.28080924	-0.084569251
1029	O00635	TRIM38	tripartite motif containing 38	10475	ENSG00000112343	4	-0	0.069	-0.22	0.131	0.635	-0.69	0.074	0.043	0.176	0.0678	0.1355	0.0391	0.2224	-0.095	-0.216	0.1002	0.0775	-0.119	-0.01	-0.28	0.088	-0.64	-0.64	0.031	0.025	0.003	0.166	0.52534	0.164264	0.000717171	0.163547122
664	E9PK54	HSPA8	heat shock protein family A (Hsp70) membe	3312	ENSG00000109971	18				-0.19	-0.22	-0.22	-0.09	0.114	0.229				-0.179	0.0548	0.019	0.1734	0.0807	0.2857				-0.11	-0.23	-0.29	0.093	0.018	0.209	0.530509	-0.00927	-0.134536684	0.125263465
1511	P09237	MMP7	matrix metallopeptidase 7	4316	ENSG00000137673	7	-0.96	-1.31	-0.61	-0.46	-0.87	-0.87	-1.19	-0.33	-0.41	-1.618	-0.705	-0.214	-0.647	-0.985	-0.72	-0.347	-0.934	-0.591	-0.05	-0.95	2.227	2.595	-0.29	0.483	-0.19	-0.44	-0.75	0.107164	-1.07274	-0.028469594	-1.044270914
3052	Q92845	KIFAP3	kinesin associated protein 3	22920	ENSG00000075945	3	0.334	0.269	0.125				-0.46	-0.22	-0.17	0.1953	0.0859	-0.191				-0.156	-0.053	-0.237	-0.02	-0.52	-0.03				-0	0.161	-0.09	0.934617	0.063098	0.039772196	0.023326288
3659	Q9ULW5	RAB26	RAB26, member RAS oncogene family	25837	ENSG00000167964	3				-0.49	0.262	-0.4	0.12	0.563	-0.11				0.582	0.1239	-0.174	0.3073	0.0146	0.0324				-0.37	-0.2	0.229	0.159	-0.02	0.066	0.693	0.013021	-0.157227839	0.170248689
1973	P48507	GCLM	glutamate-cysteine ligase modifier subunit	2730	ENSG00000023909	6	0.355	0.4	0.181	0.111	-0.15	-0.33	-0.29	-0	0.052	0.4555	0.2725	0.1247	0.0135	-0.097	0.0613	-0.15	-0.168	-0.107	-0.03	-0.08	0.01	-0.25	-0.26	0.033	0.342	0.309	0.15	0.987406	0.012829	-0.0075727	0.020401457
269	A0A1B0GTZ	DCLK3	doublecortin like kinase 3	85443	ENSG00000163673	1	-0.51	0.468	-0.44	-0.08	0.019	-1.13	0.14	-0.01	0.057	-0.543	0.0027	-0.098	-0.331	-0.192	0.0808	0.2131	1.0534	0.7896	-0.59	0.047	-0.65	-1.35	-0.96	-0.43	-0.6	0.072	0.542	0.248392	0.26932	-0.274182885	0.543503341
3795	Q9Y6M4	CSNK1G3	casein kinase 1 gamma 3	1456	ENSG00000151292	8	-0.02	-0.46	-0.05	0.217	0.523	-0.24	-0.5	0.948	-0.26	-0.264	-0.182	-0.08	0.0434	-0.351	-0.03	-0.101	-0.524	0.038	-0.36	-0.27	-0.23	-0.33	-0.5	-0.34	-0.29	0.12	0.364	0.499595	0.220734	0.179027077	0.041707155
2585	Q4J6C6	PREPL	prolyl endopeptidase like	9581	ENSG00000138078	4	0.189	0.326	0.082	-0.16	0.103	-0.29	-0.04	0.072	-0.04	0.2823	0.0739	0.0964	0.2196	-0.279	0.131	0.2441	-0.072	0.1414	-0.07	-0.38	-0.15	-0.5	-0.09	0.205	-0.2	0.032	0.537	0.496109	0.095922	-0.065856362	0.161778613
3289	Q9BVC6	TMEM109	transmembrane protein 109	79073	ENSG00000110108	1				0.457	0.125	-0.26	-0.47	-0.09	0.318				0.2352	0.1313	-0.066	0.7105	-0.03	0.2145				-0.55	-0.73	-0.06	-0.2	0.192	-0.24	0.207342	0.277011	-0.187484764	0.464495378
2068	P53367	ARFIP1	ADP ribosylation factor interacting protein 1	27236	ENSG00000164144	5	0.092	0.062	-0.06	0.049	0.157	-0.34	0.145	0.113	0.358	0.0064	0.1155	-0.043	-0.02	-0.199	0.1698	0.127	0.2106	0.0597	-0.06	-0.71	0.56	-0.7	-0.25	-0.11	0.191	0.056	0.237	0.591927	0.152022	0.017321176	0.134701243
242	C9J2C4	DNAJB6	DnaJ heat shock protein family (Hsp40) mer	10049	ENSG00000105993	3	-0.02	0.013	-0	0.114	0.513	0.032	0.352	0.264	0.247	-0.167	0.1182	-0.092	0.2604	-0.066	0.4195	-8E-04	0.0747	0.1155	-0.2	-0.33	-0.26	0.068	0.404	0.452	-0.08	-0.08	0.15	0.499871	0.153406	0.094551848	0.0588537
1316	O95857	TSPAN13	tetraspanin 13	27075	ENSG00000106537	2	-0.61	0.273	0.012				0.228	0.084	0.08	0.345	-0.115	-0.099				0.1722	0.2981	-0.193	-0.27	-0.11	0.53				-0.01	-0.06	-0.03	0.940636	0.004699	-0.0574397	0.06213838
3488	Q9NRW1	RAB6B	RAB6B, member RAS oncogene family	51560	ENSG00000154917	12	-0.4	0.179	-0.02	0.141	-0.25	-0.39	0.511	0.138	0.308	0.267	0.1508	0.1213	-0.047	-0.136	0.2468	0.3043	0.2326	-0.054	0.1	-0.51	-0.34	-0.84	-0.2	0.248	0.164	0.212	0.018	0.380547	0.150675	-0.097344568	0.248019878
800	H0Y449	YBX1	Y-box binding protein 1	4904	ENSG00000065978	3	-0.17	-0.15	0.103	-0.39	-0.1	-0.58	-0.26	-0.39	-0.16	-0.181	0.388	-0.086	0.0016	0.5592	-0.288	0.4051	-0.099	-0.317	0.152	-0.19	-0.31	-0.18	-0.36	-0.29	0.841	-0.17	-0.38	0.356424	-0.13216	-0.273577141	0.141417431
3145	Q96HY6	DDRGK1	DDRGK domain containing 1	65992	ENSG00000198171	2	-0.15	-0.7	-0.54	-0.38	0.124	0.002	-0.65	-0.22	-0.16	-0.058	0.1554	-0.219	-0.16	0.4642	0.1154	0.0176	-0.674	-0.423	-0.22	0.361	-0.03	0.142	0.296	0.043	0.393	-0.15	-0.67	0.271279	-0.31639	-0.21069306	-0.105695648
975	M0QXF7	MYDGF	myeloid derived growth factor	56005	ENSG00000074842	3	0.183	-0.16	-0.13	0.148	0.479	-0.22	0.32	0.581	0.224	-0.097	-0.306	0.1336	-0.253	-0.166	-0.153	0.1234	-0.434	0.1665	-0.54	-0.39	0.166	-0.17	-0.21	-0.13	0.258	0.027	-0.25	0.152311	0.296956	0.268323862	0.028631869
2432	Q13835	PKP1	plakophilin 1	5317	ENSG00000081277	4	-0.16	0.125	-0.17	0.03	-0.03	-0.3	0.12	0.88	0.07	-0.055	-0.146	0.1941	0.0013	0.147	-0.043	0.4124	-0.248	0.2784	-0.16	-0.31	-0.24	-0.23	-0.08	-0.09	0.718	0.233	0.414	0.973948	0.035851	0.001805332	0.034045684
3583	Q9UBP9	GULP1	GULP PTB domain containing engulfment a	51454	ENSG00000144366	2	-0.06	0.103	0.261				0.22	0.192	0.449	0.3072	0.2835	0.5174				0.1394	0.2115	0.1846	0.078	-0.04	0.182				0.151	0.052	0.35	0.418159	0.065556	-0.079823143	0.145378945
134	G5E9D8	ITIH5	inter-alpha-trypsin inhibitor heavy chain 5;int	80760	ENSG00000123243	3	-0.02	-0.45	-0.09				-0.11	0.055	-0.06	-0.459	0.3934	0.7837				0.0129	-0.089	-0.3	0.261	-0.59	0.113				0.164	-0.03	-0.13	0.780812	-0.07812	-0.169418402	0.091300037
3279	Q9BV19	C1orf50	chromosome 1 open reading frame 50	79078	ENSG00000164008	4	0.356	0.237	0.223	-0.01	0.603	-0.24	-0.13	-0.04	0.244	0.18	0.0994	0.0501	0.3172	0.1183	0.4529	0.1424	0.3118	0.1181	8E-04	-0.58	-0.16	-0.23	0.009	0.333	0.106	0.179	0.088	0.261892	0.166072	-0.060963695	0.227036063
222	A0A0G2JL6	C2	complement C2	717	ENSG00000166278	15	-0.19	-0.18	-0	0.151	0.197	-0.37	-0.6	-0.5	-0.16	-0.023	0.0874	0.0685	-0.096	-0.234	-0.568	0.319	0.0063	0.4331	-0.2	0.313	0.124	-0.23	-0.66	-0.1	-0.16	-0.07	-0.62	0.495472	-0.00667	-0.183172321	0.176499216
3464	Q9NQ88	TIGAR	TP53 induced glycolysis regulatory phospha	57103	ENSG00000078237	2	0.025	-0.22	-0.17				-0.14	0.061	0.059	0.3337	0.2222	0.0996				0.3654	-0.147	0.09	0.197	-0.39	-0.17				-0.11	-0.13	0.096	0.183999	0.023052	-0.223394379	0.246446807
3729	Q9Y303	AMDHD2	amidohydrolase domain containing 2	51005	ENSG00000162066	5	0.394	0.206	0.284				-0.24	0.029	-0.11	0.221	0.0016	-0.047				0.0532	-0.234	-0.335	-0.36	-0.31	-0.85				0.167	-0.04	-0.07	0.282891	0.339528	0.150559242	0.188968783
1595	P13747	HLA-E	major histocompatibility complex, class I, E	3133	ENSG00000233904	5	0.206	-0.03	0.501	-0.68	-0.37	-0.82	-0.41	-0.31	-0.3	-0.233	-0.389	0.14	-0.432	-0.045	-0.163	0.4061	-0.009	0.2659	0.487	0.428	0.718	1.258	0.094	-0.1	0.23	0.063	0.507	0.059472	-0.65433	-0.194213017	-0.460114967
2930	Q8NCI6	GLB1L3	galactosidase beta 1 like 3	112937	ENSG00000166105	2	0.399	0.793	-0.1	0.099	0.022	-0.14	0.186	0.363	-0.71	-0.332	0.3063	0.3761	-0.504	-0.293	-0.457	0.4477	0.3556	0.6701	-0.13	-1.06	-0.05	-0.79	-0.69	-0.14	-0.11	-0.1	0.366	0.268799	0.402135	0.038155478	0.363979104
3036	Q92572	AP3S1	adaptor related protein complex 3 subunit si	1176	ENSG00000177879	3	1.513	0.009	0.156	0.476	1.012	-0.19	0.122	0.491	0.345	0.2743	0.1002	0.1401	-0.28	-0.783	-0.704	-0.03	-0.018	-0.137	-0.07	-0.17	0.231	-0.42	-0.64	-0.43	0.427	-0.41	-0.2	0.082964	0.625356	0.597476547	0.02787939
2532	Q15750	TAB1	TGF-beta activated kinase 1 (MAP3K7) binc	10454	ENSG00000100324	4	0.002	0.033	-0.28	0.035	0.175	-0.51	0.09	0.211	0.373	-0.201	-0.294	-0.049	0.1913	-0.184	0.1974	0.2778	0.263	0.1324	-0.46	-1.85	-0.61	-0.9	-0.12	-0.05	0.071	0.273	0.256	0.240133	0.391223	-0.023042774	0.414265713
3490	Q9NRW4	DUSP22	dual specificity phosphatase 22	56940	ENSG00000112679	2	0.037	0.281	0.042	0.773	0.244	-0.28	-0.07	-0.33	-0.02	0.3212	0.3632	0.075	0.1021	-0.057	0.8196	0.0764	0.0279	-0.09	-0.66	-0.76	-0.26	-0.46	0.379	0.097	-0.33	-0.09	0.112	0.172994	0.293933	-0.106587404	0.400520689
1743	P24855	DNASE1	deoxyribonuclease 1	1773	ENSG00000213918	2							-0.16	-0.03	0.117							0.0598	-0.327	-0.034							-0	0.321	-0.26	0.870446	-0.04419	0.075273892	-0.119465688
1450	P06396	GSN	gelsolin	2934	ENSG00000148180	25	-0.06	-0.08	-0.25	-0.07	-0.21	-0.51	-0.14	-0.04	-0.42	-0.429	0.0351	-0.005	-0.03	-0.379	-0.156	0.0079	0.1986	-0.377	-0.12	-1.18	0.837	0.766	-0.53	-0.01	0.115	-0.45	8E-04	0.848668	-0.13422	-0.071320165	-0.062901037
1498	P08246	ELANE	elastase, neutrophil expressed	1991	ENSG00000277571	4	-1.2	-1.13	-0.89	-0.61	-1.04	-0.05	-2.08	-2.85	-3.23	-0.672	-0.316	-0.822	-0.928	-0.842	-0.162	-1.674	-3.198	-3.43	-0.55	-0.55	1.869	1.445	-1.09	-0.35	-2.01	-2.58	-3.19	0.655269	-0.67337	-0.113973526	-0.559394553
459	F8W1R0	FGD4	FYVE, RhoGEF and PH domain containing	121512	ENSG00000139132	4	-0.07	-0.08	-0.12	0.351	-0.23	-0.15	-0.3	0.229	-0.03	-0.243	-0.157	-0.24	-0.164	0.0567	-0.087	-0.01	0.2815	0.0666	-0.45	-0.24	-0.12	-0.25	-0.28	-0.15	-0.2	0.32	0.192	0.740534	0.087305	0.010733813	0.076571516
589	E5RI90	C1orf198	chromosome 1 open reading frame 198	84886	ENSG00000119280	1				0.245	0.25	-0.28	0.226	0.036	0.328				0.064	0.1161	0.6607	0.2847	0.2167	0.2109				-0.68	-0.17	-0.08	0.211	0.032	0.16	0.230149	0.222348	-0.124274903	0.346623397
416	B1AMS2	SEPT6;SE	septin 6	23157	ENSG00000125354	5	0.173	0.201	0.079				-0.11	-0.11	0.287	-0.133	0.0544	-0.036				0.2476	0.1853	0.2529	-0.21	-0.59	0.093				0.085	-0	0.004	0.343432	0.189263	-0.007634509	0.196897954
2973	Q8TCB6	OR51E1	olfactory receptor family 51 subfamily E mer	143503	ENSG00000180785	3	-0.26	-0.15	-0.24	-0.06	0.099	-0.39	-0.46	-0.1	-0.04	-0.048	-0.051	-0.059	0.1649	0.2806	-0.215	0.0016	0.0026	-0.172	-0.39	0.249	-0.42	-0.33	-0.3	-0.13	-0.19	-0.16	-0.18	0.193699	0.026722	-0.168276027	0.194997816
3413	Q9H8W4	PLEKHF2	pleckstrin homology and FYVE domain cont	79666	ENSG00000175895	1							0.322	0.223	0.233							0.3272	-0.193	0.1752							0.02	0.068	0.289	0.666701	0.133619	0.156164	-0.022544914
2710	Q6PCE3	PGM2L1	phosphoglucomutase 2 like 1	283209	ENSG00000165434	3							0.182	0.242	0.411							-0.241	-0.191	0.2574							-0.13	-0.12	0.214	0.329893	0.28997	0.336469496	-0.046499225
2887	Q8N122	RPTOR	regulatory associated protein of MTOR com	57521	ENSG00000141564	2							-0.07	0.206	0.127							0.011	-0.031	-0.079							0.286	0.122	0.128	0.255315	-0.09219	0.119635833	-0.211823069
290	A0A1W2PPI	ELP4	elongator acetyltransferase complex subunit	26610	ENSG00000109911	1	-0.06	0.42	-0.17				0.035	0.736	0.009	0.0582	-0.074	-0.049				0.2832	-0.046	0.052	0.227	0.493	0.762				0.557	0.377	0.111	0.178818	-0.25984	0.124059038	-0.383898783
2614	Q5JVF3	PCID2	PCI domain containing 2	55795	ENSG00000126226	5	-0.07	0.216	0.059	0.037	0.241	0.649	0.107	0.115	0.189	0.3235	0.0716	0.1106	0.3717	0.5086	0.3562	0.3318	-0.256	0.0355	-0.07	-0.18	-0.01	0.548	0.437	0.311	-0.12	0.082	0.278	0.898451	0.029732	-0.034558139	0.064289836
2962	Q8TAF3	WDR48	WD repeat domain 48	57599	ENSG00000114742	7	0.015	-0.08	-0.2	0.102	0.05	-0.21	-0.43	-0.26	-0.15	0.0677	-0.048	0.0149	0.5449	-0.176	0.0994	-0.018	0.0143	-0.187	0.015	-0.45	-0.15	-0.42	-0.22	0.125	-0.01	-0.17	-0.16	0.227077	0.031972	-0.16410668	0.196078722
861	H3BSE1	TBC1D10E	TBC1 domain family member 10B	26000	ENSG00000169221	1							0.287	0.161	0.375							0.1788	-0.256	0.3053							0.284	-0.04	0.317	0.677868	0.088578	0.198103701	-0.10952614
952	K7EJV8	GALNT1	polypeptide N-acetylgalactosaminyltransfera	2589	ENSG00000141429	2	-0.16	0.645	-0.06				0.756	0.873	0.903	-0.213	-0.151	0.1932				0.99	0.2807	1.2617	0.016	-0.08	0.238				0.277	0.341	0.493	0.697381	0.278442	0.098579705	0.179862575
3113	Q96DE0	NUDT16	nudix hydrolase 16	131870	ENSG00000198585	6	-0.06	0.116	-0.28	0.115	-0.12	-0.62	-0.02	0.154	-0.01	-0.053	-0.034	-0.058	-0.367	-0.288	-0.33	0.0559	-0.119	-0.186	-0.65	-0.61	-0.05	-0.6	-0.28	-0.46	0.206	0.103	0.005	0.492437	0.179079	0.072979037	0.106100414
3307	Q9BXX0	EMILIN2	elastin microfibril interfacer 2	84034	ENSG00000132205	2	-0.01	0.212	-0.42				0.034	0.609	0.501	0.3395	-0.659	0.2087				0.4832	0.2153	0.1848	0.057	0.889	-0.19				0.136	-0.3	-0.32	0.927539	0.108147	0.025227424	0.082919885
2533	Q15758	SLC1A5	solute carrier family 1 member 5	6510	ENSG00000105281	4	-0.78	0.934	0.621	-0.31	-0.25	-0.21	-0.7	-0.02	-0.12	0.6558	-0.091	-0.639	0.2353	-0.344	0.1421	-0.69	0.1592	-0.227	-0.46	-0.7	-0.62	-0.46	0.153	-0.11	0.063	0.19	0.122	0.891443	0.110659	-0.003382574	0.114041264
2170	P61601	NCALD	neurocalcin delta	83988	ENSG00000104490	4	-0.69	-0.31	-0.47	0.488	-0.31	-0.18	-0.19	-0.13	-0.15	0.1204	0.1008	-0.491	-0.217	0.0962	0.5512	0.1556	-0.074	-0.221	0.225	0.064	0.051	-0.55	-0.23	0.331	0.135	-0	-0.09	0.384266	-0.20743	-0.218104319	0.010677822
1880	P36954	POLR2I	RNA polymerase II subunit I	5438	ENSG00000105258	2	-0.57	-0.5	-0.26	0.203	-0.52	-0.05	0.324	0.235	0.398	-0.588	-0.333	-0.845	0.0114	0.4566	0.0528	0.5263	0.5537	0.5628	-0.06	0.432	0.042	-0.57	-0.32	0.079	0.276	0.419	0.475	0.79115	-0.16609	-0.124896559	-0.041195761
398	H0Y645	CSNK1E	casein kinase 1 epsilon	1454	ENSG00000213923	2	0.281	0.289	-0.09				0.011	0.049	-0.02	0.1447	-0.015	0.3442				-0.001	-0.13	-0.127	-0.27	-0.89	-0.37				-0.03	-0.15	0.171	0.202929	0.341904	0.04980666	0.292097801
905	J3KN29	PSMD9	proteasome 26S subunit, non-ATPase 9	5715	ENSG00000110801	6	-0.02	0.216	0.274	0.086	0.189	-0.42	0.319	0.253	0.341	0.0704	0.0502	-0.127	-0.047	-0.133	0.1451	0.2976	0.3361	0.2921	0.194	-0.64	0.082	-0.37	-0.26	-0.08	0.166	0.187	0.398	0.496786	0.1736	0.039501613	0.134098599
714	F5H4R1	MAP3K4	mitogen-activated protein kinase kinase kina	4216	ENSG00000085511	2							0.448	0.362	0.559							0.5354	0.3094	0.3789							0.419	0.472	0.13	0.721585	0.116397	0.048566868	0.067829947
322	A0A2R8Y5U	C9orf72	C9orf72-SMCR8 complex subunit	203228	ENSG00000147894	3							-0.2	-0.34	-0.28							-0.464	-0.413	-0.345							-0	-0.15	-0.16	0.084743	-0.17162	0.13304322	-0.304659099
1447	P06310	IGKV2-30	immunoglobulin kappa variable 2-30	28919	ENSG00000281933	2				-1.16	-1.29	-0.81	-1.53	-1.48	-1.5				-0.931	-0.606	-0.904	-1.07	-1.029	-1.335				2.02	0.428	1.19	-0.44	-0.17	-2.01	0.125439	-1.46523	-0.316228509	-1.149000781
158	K7ENP6	DDX39A	DExD-box helicase 39A	10212	ENSG00000123136	1				0.315	0.428	-0.01	0.065	0.893	-0.06				0.0277	-0.458	-0.44	0.1732	0.1778	0.3201				1.213	-0.22	0.457	0.292	-0.09	0.18	0.47168	-0.03309	0.304643532	-0.337736201
179	A0A0B4J29	TRAPPC2I	trafficking protein particle complex 2 like	51693	ENSG00000167515	4	0.308	0.482	0.07	-0.2	0.004	-0.45	0.215	0.248	0.248	0.16	0.2152	-0.075	-0.022	-0.135	0.5183	0.4648	-0.019	0.2784	-0.07	-0.97	-0.09	-0.48	-0.29	0.069	0.286	0.177	0.323	0.340586	0.219037	-0.051230681	0.270268024
964	V9GZ55	PSMG2	proteasome assembly chaperone 2	56984	ENSG00000128789	1	0.537	0.223	0.145				0.067	-0.04	0.324	0.033	-0.005	0.1638				0.1258	-0.014	-0.154	-0.16	-0.71	-0.08				0.033	0.049	0.15	0.199896	0.328462	0.184971513	0.143490786
2471	Q5W0W3	KANK1	KN motif and ankyrin repeat domains 1	23189	ENSG00000107104	3	0.138	-0.21	0.136				0.468	0.406	0.394	0.1098	0.1423	-0.471				0.1262	0.1443	0.2383	0.213	-0.6	0.054				0.068	0.191	0.639	0.725409	0.128207	0.174452169	-0.046245086
325	A0A2R8Y63	ATP13A3	ATPase 13A3	79572	ENSG00000133657	6	0.285	0.132	0.017	-0.11	-0.03	-0.22	-0.26	-0.14	-0.14	0.1209	0.1939	0.2074	0.3496	-0.215	0.0225	0.0386	0.1927	0.2728	-0.29	-0.96	-0.39	-0.54	-0.29	0.306	-0.03	-0.08	-0.04	0.103152	0.204221	-0.183995116	0.388216336
226	A0A0G2JME	IGHA2	immunoglobulin heavy constant alpha 2 (A2	3494	ENSG00000276173	16	-1.17	-1.16	-0.85	-1.83	-2.14	-1.35	-3.89	-2.57	-4.42	-0.707	-0.124	-0.71	-1.7	-1.097	-2.156	-2.149	-2.774	-4.239	-0.72	-0.7	3.009	1.854	0.171	1.679	-1.21	-2.92	-3.8	0.193749	-1.86015	-0.414020639	-1.446133685
2136	P60763	RAC3	Rac family small GTPase 3	5881	ENSG00000169750	8				0.194	-0.02	-0.24	0.125	0.01	0.247				0.2847	0.0377	0.0405	0.0851	0.3185	0.1816				-0.16	-0.27	-0.11	-0.08	0.005	0.273	0.255414	0.106894	-0.105414968	0.212309342
1046	O14772	FPGT	fucose-1-phosphate guanylyltransferase	8790	ENSG00000254685	7	-0.02	0.34	-0	-0.02	0.102	-0.61	0.129	-0.07	0.217	0.2078	0.0983	0.1049	0.3257	-0.272	-0.123	0.1847	0.2998	0.168	-0.17	-0.51	0.059	-0.33	-0.24	0.262	0.129	0.129	0.282	0.571784	0.048801	-0.103770301	0.152571205
2514	Q15363	TMED2	transmembrane p24 trafficking protein 2	10959	ENSG00000086598	3	0.12	0.208	0.46	-0.54	0.311	-0.04	-0.04	0.404	0.235	-0.28	-0.158	0.2031	-0.456	-0.056	-0.178	-0.017	-0.642	-0.338	-0.42	-0.58	-0.13	-0.42	0.027	0.283	0.652	-0.19	-0.08	0.230049	0.220121	0.337800674	-0.117680154
2740	Q6X4U4	SOSTDC1	sclerostin domain containing 1	25928	ENSG00000171243	3	-0.4	0.071	-0.45	-0.57	-0.89	0.258	0.002	-0.09	-0.17	0.1141	0.2014	-0.084	-0.378	0.595	-0.355	0.2887	0.0154	0.0313	-0.37	-0.44	0.459	-0.18	0.429	0.183	0.569	0.172	0.325	0.207891	-0.37519	-0.29604811	-0.079139723
1129	O43639	NCK2	NCK adaptor protein 2	8440	ENSG00000071051	7	0.089	0.405	0.13	0.089	-0.08	-0.42	-0.39	-0.19	0.026	0.0673	0.1329	0.1606	0.1883	-0.08	0.3164	-0.084	0.1002	-0.096	-0.16	-0.54	-0.15	-0.56	-0.21	0.064	-0.15	0.125	0.144	0.247811	0.121514	-0.115999952	0.237513638
3210	Q99570	PIK3R4	phosphoinositide-3-kinase regulatory subuni	30849	ENSG00000196455	2							0.188	-0.27	0.109							0.2305	0.0091	0.1644							0.472	-0.03	0.148	0.69556	-0.18705	-0.124310648	-0.06273585
3435	Q9HB90	RRAGC	Ras related GTP binding C	64121	ENSG00000116954	3	-0.58	0.01	0.083				-0.22	-0.39	-0.22	0.4632	-0.085	-0.011				-0.076	0.1879	-0.12	0.344	0.889	0.053				-0.54	-0.38	0.19	0.43962	-0.31029	-0.278404345	-0.031887948
499	E5RH64	NT5C3B	5'-nucleotidase, cytosolic IIIB	115024	ENSG00000141698	1							-0.08	0.041	0.35							-0.021	0.31	0.4957							0.023	-0.19	0.407	0.773804	0.021392	-0.159330299	0.180722702
2112	P55263	ADK	adenosine kinase	132	ENSG00000156110	10	-0.18	-0.1	-0.04	-0.17	0.043	-0.32	0.051	0.189	0.278	-0.003	0.4814	-0.158	0.1326	-0.116	0.1508	0.1094	0.0903	0.0731	-0.22	-0.02	0.487	-0.23	-0.21	0.104	-0.06	0.044	0.359	0.662295	-0.0566	-0.112379924	0.05577553
3388	Q9H497	TOR3A	torsin family 3 member A	64222	ENSG00000186283	1	0.371	0.188	-0.51	0.032	0.52	-0.15	-0.46	-0.02	0.056	0.1794	-0.158	-0.17	-0.081	-0.105	0.0083	0.4696	-0.104	0.1472	-0.41	-0.15	0.195	-0.01	-0.33	0.105	-0.09	0.172	0.402	0.977307	0.016861	-0.016650454	0.033511158
1887	P38117	ETFB	electron transfer flavoprotein subunit beta	2109	ENSG00000105379	3	0.145	0.335	-0.19				0.253	0.152	0.249	0.1037	0.0377	0.0518				0.2022	0.1888	0.1055	-0.34	-0.47	-0.12				0.097	-0.01	0.455	0.372347	0.222505	0.041887232	0.180617897
3241	Q9BQ69	MACROD1	mono-ADP ribosylhydrolase 1;MACRO dom	28992	ENSG00000133315	6	-0.13	-0.02	-0.05	-0.04	-0.21	-0.3	-0.87	-0.7	-0.43	-0.003	0.111	-0.092	-0.285	-0.294	0.0294	-0.692	-0.025	-0.466	-0.12	-0.26	-0.13	-0.51	-0.36	-0.28	-0.51	-0.29	-0.3	0.655853	0.000644	-0.114466401	0.115110751
632	P31943	HNRNPH1	heterogeneous nuclear ribonucleoprotein H1	3187	ENSG00000169045	3	0.066	0.136	-0.24	-0.05	-0.53	-0.69	0.199	0.5	0.046	-0.154	-0.154	0.0739	-0.27	-0.391	-0.36	0.1028	0.2255	0.1453	-0.3	-1.05	0.354	-0.23	-0.46	-0.3	0.043	-0.18	0.159	0.705502	0.155738	0.024560041	0.13117786
488	C9J2C3	GALNT3	polypeptide N-acetylgalactosaminyltransfera	2591	ENSG00000115339	1	-0.09	0.092	-0.07	-0.25	-0.19	-0.28	-0.14	-0.12	0.163	0.2314	0.0108	-0.084	-0.439	-0.094	-0.512	0.1873	-0.223	-0.14	-0.5	0.193	-0.49	-0.33	-0.37	-0.44	0.131	-0.15	-0.18	0.525402	0.14022	0.020265167	0.119954388
2390	Q13219	PAPPA	pappalysin 1	5069	ENSG00000182752	2	-0.11	-0.33	0.315				-0.06	-0.13	0.174	0.1334	0.2843	-0.126				0.105	0.0383	0.2543	0.182	0.122	0.108				0.329	0.427	0.109	0.236591	-0.23689	-0.138982682	-0.097911711
2977	Q8TD20	SLC2A12	solute carrier family 2 member 12	154091	ENSG00000146411	3							-0.59	-0.21	0.007							-0.131	0.1544	-0.092							-0.07	-0.12	-0.48	0.586423	-0.04257	-0.243412348	0.20083944
2733	Q6UXI9	NPNT	nephronectin	255743	ENSG00000168743	30							-0.01	0.08	0.38							0.2628	0.04	-0.017							-0.05	0.205	0.175	0.941487	0.040601	0.055908513	-0.015307936
1951	P46934	NEDD4	neural precursor cell expressed, developme	4734	ENSG00000069869	11	-0.06	0.322	0.017	0.203	-0.36	-0.34	-0.29	0.15	0.202	-0.019	-0.143	0.0943	-0.088	-0.492	-0.097	0.3398	0.1729	0.1066	-0.34	-0.61	-0.1	-0.13	0.249	-0.06	-0.05	0.015	0.208	0.844379	0.073149	-0.004979176	0.078127754

3403	Q9H772	GREM2	gremlin 2, DAN family BMP antagonist	64388	ENSG00000180875	2	-1.21	0.705	-0.67	0.238	0.48	-0.31	-0.75	0.383	0.213	0.5425	-0.836	-0.228	0.1492	0.3077	-0.002	-0.095	0.7173	-0.012	-1.26	-0.31	-0.36	-0.02	0.205	0.266	-0.43	0.455	-1.36	0.563847	0.21037	-0.16329661	0.373666261
1293	O95486	SEC24A	SEC24 homolog A, COPII coat complex cor	10802	ENSG00000113615	2							-0.15	-0.04	-0.28							0.3289	0.0718	0.1298							0.18	0.641	-0.3	0.492701	-0.33021	-0.333371639	0.003164637
2286	Q01085	TIAL1	TIA1 cytotoxic granule associated RNA binc	7073	ENSG00000151923	2				-0.32	-0.06	-0.24	0.094	0.283	0.067				-0.163	-0.019	-0.018	0.0427	-0.008	0.3391				-0.06	0.177	-0.16	0.019	0.008	0.422	0.791592	-0.09787	-0.059123623	-0.038746296
3762	Q9Y5A9	YTHDF2	YTH N6-methyladenosine RNA binding prot	51441	ENSG00000198492	3				-0.14	0.056	-0.2	-0.08	0.063	0.231				-0.063	-0.072	0.0772	0.065	0.0823	-0.046				-0.04	0.024	0.056	-0.02	-0.08	0.061	0.969878	-0.01129	-0.018504718	0.007216692
2042	P51511	MMP15	matrix metallopeptidase 15	4324	ENSG00000102996	2	-0.37	0.127	-0.47	0.257	-0.01	0.034	0.256	0.314	0.32	0.4913	0.429	0.1927	0.1868	-0.037	0.2194	0.4684	0.0143	0.4084	-0.11	-0.21	0.397	-0	-0.06	0.078	0.407	0.268	0.473	0.369667	-0.08703	-0.213133326	0.12610268
3098	Q96BQ1	FAM3D	family with sequence similarity 3 member D	131177	ENSG00000198643	9	-0.62	-0.68	-0.45	-0.51	-0.74	-0.08	-0.59	-0.08	-0.35	0.8586	0.6313	0.0735	-0.168	-0.037	0.2935	-0.245	-0.464	-0.548	-0.14	0.234	0.301	0.07	-0.24	-0.07	0.541	0.773	-0.22	0.071396	-0.59482	-0.499598643	-0.095219792
3530	Q9NX55	HYPK	huntingtin interacting protein K	25764	ENSG00000242028	2	0.391	0.295	0.735				-0.21	-0.1	-0.05	0.8958	0.8831	0.6386				0.0822	0.1405	-0.092	-1.54	-0.64	0.235				0.437	0.102	-0.26	0.236768	0.455634	-0.247993664	0.70362757
787	G5E931	BLOC1S5	biogenesis of lysosomal organelles complex	63915	ENSG00000188428	2	-0.03	0.149	0.079				0.147	-0.01	0.239	0.2135	-0.005	0.2439				0.1592	-0.017	0.0313	0.022	-0.53	0.075				0.362	0.122	0.206	0.898252	0.052612	-0.008291585	0.060903499
539	O60716	CTNND1	catenin delta 1	1500	ENSG00000198561	2	-0.35	-0.07	-0.14				0.064	-0.16	0.135	-0.153	-0.199	-0.143				0.1947	0.1193	0.323	-0.35	-0.33	0.735				-0.13	-0.06	0.117	0.857885	-0.08207	-0.109098997	0.027033708
1755	P26196	DDX6	DEAD-box helicase 6	1656	ENSG00000110367	6	0.155	0.028	-0.08	0.023	0.093	-0.31	-0.15	0.295	0.262	-0.003	0.0428	0.1048	0.0992	-0.004	0.0411	-0.082	-0.169	-0.341	-0.2	-0.66	-0.25	0.08	-0.03	0.192	0.268	0.057	-0.15	0.663799	0.111573	0.069709363	0.041863418
2396	Q13283	G3BP1	G3BP stress granule assembly factor 1	10146	ENSG00000145907	4	0.211	0.025	-0.09				0.09	0.282	0.258	0.1679	0.1823	0.1343				0.1713	0.2347	0.1813	-0.02	-0.38	0.176				0.124	0.107	0.403	0.673048	0.061628	-0.049503854	0.111131949
695	F5GWG3	GPRC5A	G protein-coupled receptor class C group 5	9052	ENSG00000013588	2							0.382	0.064	0.571							0.3668	1.146	1.208							0.408	0.433	0.498	0.284246	-0.10741	-0.567961236	0.46055003
1288	O95436	SLC34A2	solute carrier family 34 member 2	10568	ENSG00000157765	5	-0.34	0.453	-0.01	0.013	-0.11	0.243	0.726	0.605	0.664	-0.538	0.1464	-0.386	0.5426	-0.046	-0.258	0.4022	0.1002	0.8794	-0.58	0.304	0.073	0.889	-0.09	-0.24	0.856	0.198	0.547	0.821374	0.032491	0.156029555	-0.123539025
1587	P13640	MT1G	metallothionein 1G	4495	ENSG00000125144	4	1.099	-0.24	-0.44	0.011	-0.19	0.926	-0.67	1.412	-0.51	-0.073	-0.326	-1.429	0.4008	0.3829	-0.307	-1.297	-0.95	-1.39	0.318	-0.96	0.195	-0.56	-0.82	-0.06	-1.18	-0.04	0.74	0.281997	0.419466	0.709527599	-0.290061312
2918	Q8NB37	GATD1	glutamine amidotransferase like class 1 don	347862	ENSG00000177225	3	-0.3	-0.02	-0.04				0.054	0.198	0.522	-0.023	-0.244	0.067				0.1926	0.3095	0.2208	-0.16	-0.25	0.048				0.413	0.235	0.459	0.955853	-0.05416	-0.016684683	-0.037475365
779	P25963	NFKBIA	NFKB inhibitor alpha	4792	ENSG00000100906	2				-0.13	-0.24	-0.34	0.074	0.233	0.248				0.0221	-0.13	-0.077	0.1658	0.0023	0.0676				-0.27	-0.34	0.06	0.215	0.048	0.141	0.967835	-0.00131	-0.032877017	0.031563971
3171	Q96NY7	CLIC6	chloride intracellular channel 6	54102	ENSG00000159212	3	-0.01	0.092	-0.03	-0.02	-0.3	-0.1	0.141	0.334	0.17	-0.163	0.0091	-0.036	-0.147	0.3205	0.2291	0.0278	0.2566	0.0121	-0.05	-0.57	-0.35	-0.07	-0.21	0.162	0.11	0.004	0.301	0.540566	0.105571	-0.025858367	0.131429126
816	H0YAZ5	C8orf58	chromosome 8 open reading frame 58	541565	ENSG00000241852	1	-0.22	-1.04	-0.08	0.043	-0.38	-0.52	-0.78	-0.46	0.292	1.3182	-0.565	0.4759	0.3602	-0.301	-0.764	0.5419	-0.57	-0.402	0.209	-0.35	-0.31	-0.45	-0.58	-0.17	0.124	0.594	-0.61	0.510932	-0.17723	-0.360347398	0.183120257
1208	O75396	SEC22B	SEC22 homolog B, vesicle trafficking protei	9554	ENSG00000265808	5	0.245	-0.04	-0.1	0.229	0.102	-0.18	0.031	0.255	0.417	-0.093	-0.138	-0.111	0.0531	-0.079	0.0728	-0.34	-0.154	0.0817	-0.32	-0.63	0.017	-0	-0.03	-0.02	0.212	0.052	0.347	0.327325	0.147501	0.185311143	-0.037810339
3305	Q9BXV9	GON7	GON7 subunit of KEOPS complex;GON7, I	84520	ENSG00000170270	1				0.179	0.352	-0.35	0.022	0.125	0.376				0.2086	-0.07	0.3434	0.3163	0.4033	0.1191				-0.45	-0.33	0.162	0.091	0.107	0.159	0.35259	0.161563	-0.103282092	0.264844902
2122	P57739	CLDN2	claudin 2	9075	ENSG00000165376	1	0.18	0.337	-0.09	-0.01	0.134	0.056	0.193	-0.03	0.152	0.4911	-0.127	0.2645	0.148	-0.207	0.1249	-0.001	-0.078	0.2633	0.28	0.883	-0.06	-0	-0.21	0.002	0.266	-0.12	0.202	0.947455	-0.03694	0.004295398	-0.041233374
1421	P04732	MT1E	metallothionein 1E	4493	ENSG00000169715	4	1.067	0.019	-0.1	-0.04	-1.55	1.33	-0.47	0.716	-0.87	-0.191	-0.851	-0.561	0.3601	-2.41	-0.713	0.5837	-1.149	-1.164	0.506	-1.46	-0.1	1.466	0.351	-0.37	-0.09	-0.15	0.007	0.321612	-0.00714	0.689186694	-0.69632507
2166	P61457	PCBD1	pterin-4 alpha-carbinolamine dehydratase 1	5092	ENSG00000166228	4	0.182	0.161	0.139	0.007	-0.04	-0.26	-0.26	-0.44	-0.37	0.1942	0.1741	0.1579	0.2138	0.0877	0.2718	-0.275	-0.025	-0.478	-0.28	-0.56	-0.18	-0.07	-0.07	0.074	-0.27	-0.45	-0.42	0.18151	0.149625	-0.133581977	0.283206552
2910	Q8N6R0	EEF1AKNI	eEF1A lysine and N-terminal methyltransfer	51603	ENSG00000010165	2	-0.02	0.187	0.028	0.33	0.093	0.286	0.003	-0.09	0.16	-0.133	-0.162	0.0568	-0.029	0.173	0.181	0.2995	0.2269	0.6433	-0.28	-0.73	-0.33	-0.07	-0.2	0.036	0.247	0.09	0.418	0.291359	0.199545	-0.031209038	0.230753988
1686	P20151	KLK2	kallikrein related peptidase 2	3817	ENSG00000167751	7	-0.53	0.35	0.317	-0.23	0.339	0.035	-0.57	-0.65	-0.03	-0.271	0.1154	0.0363	0.0181	-0.129	-0.487	0.0905	0.2678	0.5552	-0.03	0.21	0.133	0.142	-0.02	0.147	-0.5	-0.54	-0.27	0.786516	-0.0251	-0.128118111	0.103021632
1633	P15924	DSP	desmoplakin	1832	ENSG00000096696	6	0.001	0.135	0.004	-0	0.285	-0.32	0.496	0.095	0.246	0.3056	-0.116	0.1033	0.0158	-0.201	-0.289	0.3835	-0.371	0.4431	-0.01	-0.48	0.084	0.574	-0.01	0.473	0.23	0.111	-0.05	0.880722	0.002532	0.07428921	-0.071756883
1389	P02750	LRG1	leucine rich alpha-2-glycoprotein 1	116844	ENSG00000171236	4	-0.32	-0.43	-0.48	-0.1	0.035	-0.2	-0.41	-0.12	0.004	0.4257	0.1025	0.4679	0.019	0.1994	-0.366	0.3138	-0.518	-0.397	0.101	0.145	0.287	0.436	0.424	0.071	0.195	0.56	-0.5	0.131711	-0.41556	-0.251935725	-0.163621783
980	M0R3F4	BABAM1	BRISC and BRCA1 A complex member 1	29086	ENSG00000105393	4	0.222	0.236	-0.01	-0.34	-0.08	-0.4	0.038	0.013	0.267	0.0872	0.0287	0.0342	-0.083	-0.117	-0.007	0.2152	0.2041	0.1653	-0.21	-0.64	-0.18	-0.91	0.065	0.048	0.397	-0.12	-0.01	0.35653	0.166221	-0.06551568	0.231736269
3156	Q96JZ2	HSH2D	hematopoietic SH2 domain containing	84941	ENSG00000196684	6	-0.65	-0.06	-0.6	0.228	0.124	-0.4	-0.46	-0.45	-0.26	-0.128	0.0612	-0.012	0.3465	0.0924	0.0223	0.2478	0.3936	-0.426	-0.36	0.248	-0.22	-0.14	-0.08	-0.04	-0.44	0.302	0.207	0.164439	-0.22315	-0.348798297	0.125649496
725	F6SYF8	DKK3	dickkopf WNT signaling pathway inhibitor 3	27122	ENSG00000050165	2	-0.33	-0.65	-0.47	0.182	-0.33	-0.17	-0.15	0.355	0.12	-0.173	0.0547	0.3369	-0.518	0.3656	-0.083	-0.076	-0.3	0.6301	0.059	-1.15	0.144	-0.5	0.257	-0.06	0.555	0.168	-0.15	0.731539	-0.08367	-0.186643311	0.102972903
3189	Q96S53	TESK2	testis associated actin remodelling kinase 2	10420	ENSG00000070759	3	-0.36	0.397	-0.15	0.229	0.298	-0.11	-0.16	-0.33	-0.31	0.6544	0.0239	0.2527	0.2309	0.0685	0.2503	-0.171	0.1054	-0.373	-0.25	-0.54	-0.4	-0.08	-0.04	0.385	-0.23	0.057	-0	0.358585	0.067813	-0.170770927	0.238584289
2459	Q14439	GPR176	G protein-coupled receptor 176	11245	ENSG00000166073	3	-0.46	0.029	-0.42				-0.71	-0.17	-0.65	-0.349	-0.523	0.9678				-0.15	-0.707	-0.119	-0.35	-0.68	0.319				0.349	-0.06	0.433	0.493319	-0.39864	-0.250120477	-0.148516037
2401	Q13363	CTBP1	C-terminal binding protein 1	1487	ENSG00000159692	4	0.362	0.161	0.071	-0.01	0.269	-0.14	0.116	0.058	0.241	0.215	-0.314	0.1812	0.0486	-0.248	-0.011	0.2274	0.2045	0.1749	0.132	-0.63	-0.07	0.141	-0.05	0.006	0.108	0.026	0.008	0.408465	0.162494	0.071559488	0.090934912
2722	Q6UWJ8	CD164L2	CD164 molecule like 2	388611	ENSG00000174950	1	-0.03	-0.07	0.06				-0.48	-0.1	0.085	0.1029	0.0838	0.0532				-0.442	0.1197	-0.386	-0.05	-0.01	-0.05				0.232	-0.27	-0.14	0.961829	-0.04073	-0.010523825	-0.030203256
2837	Q8IUR0	TRAPPC5	trafficking protein particle complex 5	126003	ENSG00000181029	2				-0.17	-0.01	-0.49	-0.03	0.021	0.252				-0.031	-0.197	0.0703	-0.006	0.2138	0.15				-0.38	-0.39	-0.03	0.146	-0.04	0.254	0.758682	0.003165	-0.103350537	0.106515464
283	C9J3Q3	FAM107B	family with sequence similarity 107 member	83641	ENSG00000065809	2							0.502	-0.51	0.304							0.0295	0.382	0.1752							0.063	0.226	0.356	0.934582	-0.11723	-0.097711647	-0.019514601
3302	Q9BXR0	QTRT1	queuine tRNA-ribosyltransferase catalytic su	81890	ENSG00000213339	2	0.099	0.141	-0.1	-0.13	0.128	-0.37	0.176	0.152	0.265	0.1805	0.0197	0.003	0.1217	0.0954	0.1351	0.0549	0.2207	0.0728	-0.13	-0.62	-0.13	-0.13	-0.07	0.054	0.115	0.037	0.286	0.34733	0.105551	-0.059764797	0.165316269
2843	Q8IVI9	NOSTRIN	nitric oxide synthase trafficking	115677	ENSG00000163072	2	-0.49	0.119	0.203				0.135	0.271	0.242	-0.148	-0.055	0.2726				0.3857	-0.091	0.1697	-0.25	0.775	-0.06				0.283	0.276	0.035	0.883609	-0.09644	-0.009384289	-0.087059649
3223	Q99707	MTR	5-methyltetrahydrofolate-homocysteine meth	4548	ENSG00000116984	3							-0.08	0.361	0.367							0.4922	0.1491	0.0505							-0.01	0.382	0.404	0.983214	-0.04253	-0.013684551	-0.028849151
598	O43572	AKAP10	A-kinase anchoring protein 10	11216	ENSG00000108599	2	-0.07	-0.09	-0.23	-0.03	-0.04	-0.08	0.047	-0.19	-0.07	-0.02	-0.284	0.1505	0.0783	0.1284	-0.018	0.5159	0.0727	0.0987	-0.1	-0.57	-0.35	-0.44	-0.16	-0.02	0.217	0.125	0.09	0.219157	0.050777	-0.164594105	0.215371339
2594	Q53H82	LACTB2	lactamase beta 2	51110	ENSG00000147592	2							0.273	0.457	0.271							0.4273	0.138	0.2542							-0.15	0.196	0.485	0.773542	0.155396	0.060284391	0.095111127
2668	Q63HM1	AFMID	arylformamidase	125061	ENSG00000183077	1	-0.08	0.262	0.133	0.028	0.197	-0.41	-0.12	0.073	0.39	0.1269	0.1497	0.0567	0.3059	-0.148	-0.032	-0.077	0.3894	0.1249	-0.03	-0.85	-0.11	-0.49	-0.29	-0.25	-0.34	-0.04	-0.05	0.063707	0.32522	-0.047381709	0.372601341
2664	Q5W0V3	FAM160B1	family with sequence similarity 160 member	57700	ENSG00000151553	3							-0.01	0.004	0.188							0.0355	-0.068	0.0121							0.099	0.123	0.126	0.346455	-0.05578	0.066933226	-0.122709855
1388	P02749	APOH	apolipoprotein H	350	ENSG00000091583	4	-0.16	-0.66	0.063	-0.35	-0.26	0.08	-0.73	-0.48	-0.69	0.9427	-0.289	0.1919	-0.443	-0.324	-0.825	-0.235	-0.49	-0.444	-0.54	-0.58	0.822	0.99	0.413	0.071	-0.2	0.366	-1.85	0.700545	-0.29911	-0.14150896	-0.157598178
2699	Q96GK7	FAHD2A	fumarylacetoacetate hydrolase domain conta	51011	ENSG00000115042	2							0.313	0.355	0.395							0.9212	0.3997	0.4937							0.222	0.345	0.494	0.380263	0.000575	-0.250592043	0.251167541
3328	Q9C037	TRIM4	tripartite motif containing 4	89122	ENSG00000146833	3	-0.76	0.973	-0.45	-0.23	0.154	-0.42	-0.36	1.906	-0.4	-0.025	-0.443	0.3034	0.3631	-0.205	0.2443	0.4709	-0.41	0.8005	-0.11	0.03	-0.06	-0.09	-0.15	-0.07	0.528	0.84	1.4	0.850761	-0.21082	-0.076188613	-0.134627787
1365	P01718	IGLV3-27	immunoglobulin lambda variable 3-27	28791	ENSG00000211658	2	-1.19	-1.67	-1.45	-1.75	-1.85	-1.27	-2.82	-3.08	-2.96	-1.928	-0.423	-1.196	-1.949	-0.547	-1.181	-3.262	-3.068	-3.25	-0.98	-1.2	3.394	-0.17	-1.09	1.743	-2.63	-2.78	-2.47	0.267073	-1.31773	-0.137518691	-1.18020681
1049	O14787	TNPO2	transportin 2	30000	ENSG00000105576	15	-0.08	0.354	-0.18	-0.04	-0.03	-0.33	-0.08	0.029	0.206	-0.1	0.1155	0.0927	-0.059	-0.131	-0.061	0.0552	0.2217	-7E-04	-0.05	-0.41	-0.1	-0.09	-0.28	0.11	0.096	-0.06	0.133	0.675638	0.055237	-0.03173674	0.086974185
278	A0A1B0GVL	UBE3A	ubiquitin protein ligase E3A	7337	ENSG00000114062	4	-0.42	-0.28	-0.32				-0.31	-0.11	0.055	-0.197	-0.426	-0.149				-0.028	0.041	0.1857	-0.38	-0.45	-0.31				-0.16	-0.17	0.169	0.602983	-0.0169	-0.136676346	0.119773035
3227	Q99801	NKX3-1	NK3 homeobox 1	4824	ENSG00000167034	2	0.187	0.214	-0.06	-0.07	-0.12	-0.4	-0.08	0.036	0.165	-0.076	0.1283	0.0386	-0.106	-0.274	0.1779	0.114	0.0084	-0.058	-0.35	-1.03	-0.06	-0.59	-0.19	-0.04	0.018	-0.11	-0.28	0.137942	0.277185	-0.010309959	0.287495303
563	D6RBR6	EPN3	epsin 3	55040	ENSG00000049283	2							-0.16	-0.02	0.255							0.1706	0.3628	0.3708							-0.1	0.266	0.412	0.47653	-0.16829	-0.27604043	0.107749927
528	C9JMV9	ABHD14A-	ABHD14A-ACY1 readthrough	100526760	ENSG00000114786	17	-0.06	-0.01	-0.27	-0.16	-0.14	-0.06	0.464	0.452	0.824	0.2254	0.0972	0.0356	-0.204	-0.166	-0.221	0.5349	0.4967	0.8566	-0.09	-0.05	0.406	-0.21	-0.23	-0.17	0.21	0.205	0.756	0.904111	0.023158	-0.068508765	0.091667025
1418	P04350	TUBB4A	tubulin beta 4A class IVa	10382	ENSG00000104833	19							-0.02	0.327	0.052							0.313	0.3971	0.0298							0.417	0.278	-0.26	0.880514	-0.02551	-0.128326967	0.102817191
830	H0YKV4	CIAO2A	cytosolic iron-sulfur assembly component 2/	84191	ENSG00000166797	1	0.012	0.002	-0.3	0.094	0.03	-0.53	0.022	-0.2	0.16	0.2547	0.0134	0.2073	-0.119	-0.177	-0.066	-0.088	0.2585	0.1995	-0.26	-0.57	-0.36	-0.38	-0.43	-0.13	0.132	0.014	0.083	0.178462	0.131554	-0.133680827	0.265235118
1806	P30154	PPP2R1B	protein phosphatase 2 scaffold subunit Abet	5519	ENSG00000137713	13	0.386	0.497	0.008				-0.36	-0.09	-0.12	0.3356	0.1416	0.2718				-0.12	-0.119	-0.194	0.197	-0.55	-0.08				-0.16	-0.16	-0.15	0.496245	0.203352	0.001640121	0.201712012
1265	O94955	RHOBTB3	Rho related BTB domain containing 3	22836	ENSG00000164292	2	0.215	0.567	0.299				0.127	0.1	0.129	0.719	0.768	0.4084				0.3312	-0.217	0.3778	0.374	0.332	0.501				0.238	0.071	0.154	0.645466	-0.03895	-0.158440097	0.119491318
1358	P01602	IGKV1-5	immunoglobulin kappa variable 1-5	28299	ENSG00000243466	1	-0.97	-1.1	-0.51	-0.23	-0.45	-0.06	-1.05	-0.55	-0.84	-0.847	-1.148	-0.9	-0.58	0.051	-0.46	-0.436	-1.644	-1.278	-0.94	-0.75	1.192	1.846	0.436	0.164	0.419	-0.44	-1.56	0.172364	-0.68117	0.164108148	-0.845282114
3699	Q9Y233	PDE10A	phosphodiesterase 10A	10846;9063	ENSG00000112541	3	-0.2	0.274	0.138				-0.39	0.583	-0.15	-0.271	-0.112	0.2739				0.0806	-0.551	0.2519	-0.47	-0.12	-0.48				0.246	-0.05	-0.34	0.572288	0.24674	0.098964855	0.14777525
505	C9JA99	PCDHA13	protocadherin alpha 13	56136	ENSG00000239389	1							0.093	0.402	0.022							0.6761	0.5173	0.337							0.244	0.225	0.1	0.203898	-0.01717	-0.337664002	0.320489913
186	F8WAL6	ABI2	abl interactor 2	10152	ENSG00000138443	5	0.064	0.239	-0.02	-0.08	0.249	-0.28	-0.09	-0.02	0.099	-0.012	-0.146	0.0473	0.0208	-0.141	-0.008	0.1875	0.1031	0.0442	-0.26	-0.84	-0.35	-0.1	-0.34	-0.05	-0.02	-0.04	-0.01	0.125573	0.243275	0.007336575	0.235938039
1218	O75592	MYCBP2	MYC binding protein 2;MYC binding protein	23077	ENSG00000005810	5				-0.05	-0.09	-0.25	0.044	0.304	-0.14				0.1524	0.0558	0.0363	0.0344	0.1878	0.2533				8E-04	0.103	0.079	0.59	-0.01	-0.44	0.65399	-0.08306	-0.148992604	0.065934401
3660	Q9ULZ3	PYCARD	PYD and CARD domain containing	29108	ENSG00000103490	2	-0.25	0.405	0.141				-0.03	-0.48	-0.43	-0.862	0.4553	0.0893				-0.619	-0.018	-0.537	0.288	-0.52	0.24				-0.21	-0.31	-0.33	0.879015	0.033162	0.141951027	-0.108789256
645	E9PGA7	FRMD4B	FERM domain containing 4B	23150	ENSG00000114541	1	-1.31	1.149	0.197	-0.39	-0.06	-0.66	-0.08	-0.22	0.095	0.5827	0.2742	0.1022	-0.089	0.2239	-0.586	-0.303	0.3166	0.4786	-0.18	-0	-0.12	-0.69	-0.86	0.055	-0.6	-0.16	0.344	0.465402	0.105187	-0.251800537	0.356987305
2863	Q8IY63	AMOTL1	angiomotin like 1	154810	ENSG00000166025	4	0.449	0.417	0.255	0.168	-0.5	0.155	-0.3	-0.38	-0.29	0.4869	0.4666	0.1856	0.0922	-0.14	0.1349	0.1611	-0.445	-0.284	0.216	-0.4	-0.05	-0.12	6E-04	-0.33	0.09	-0.41	0.297	0.716421	0.075271	-0.076232505	0.151503193
94	A0A087X0Q	SELENOO	selenoprotein O	83642	ENSG00000073169	4				-0.32	-0.07	-0.44	-0.05	0.557	0.163				0.0759	-0.07	0.2053	0.2397	0.1311	0.1374				-0.47	-0.38	0.085	0.209	-0.04	0.254	0.641495	0.031038	-0.145799365	0.176837375
1742	P24666	ACP1	acid phosphatase 1	52	ENSG00000143727	8	0.242	-0.29	-0.16	-0.18	-0.4	-0.55	-0.27	0.295	0.098	0.0601	0.3197	0.3525	0.1123	-0.453	0.1097	0.0423	-0.029	0.1791	0.264	0.108	0.202	-0.71	-0.37	-0.07	-0.02	0.089	-0.44	0.413836	-0.03289	-0.213401301	0.18050748
2333	X6R3S7	TFF3	trefoil factor 3	7033	ENSG00000160180	2	0.264	0.194	0.185	-0.28	-0.13	-0.99	-0.98	-0.23	0.256	0.8313	0.0636	0.5729	0.311	-0.211	-1.451	0.328	-0.032	-0.229	0.177	-0.71	-0.18	-1.14	-0.87	-0.22	0.085	0.392	-0.43	0.577889	0.13168	-0.210648391	0.342328458
890	H7C3Q6	MITD1	microtubule interacting and trafficking doma	129531	ENSG00000158411	3				-0.3	-0.09	-0.14	0.383	0.235	0.131				0.0596	0.2981	0.0173	0.5979	0.442	0.5777				-0.26	-0.18	0.3	0.519	0.169	0.757	0.43755	-0.17899	-0.294420154	0.115425266
972	L7P8G6	MTHFR	methylenetetrahydrofolate reductase	4524	ENSG00000177000	5	-0.43	-0.38	-0.7				-0.51	0.308	0.015	-0.293	-0.951	-0.213				-0.343	-0.13	-0.532	-0.36	-0.67	-0.69				0.188	-0.31	-0.19	0.873172	0.056391	0.126536901	-0.070145915
526	C9JLU1	POLR2H	RNA polymerase II subunit H	5437	ENSG00000163882	4	-0.32	0.002	-0.29				0.447	0.458	0.153	0.113	-0.03	0.1321				0.6378	0.0909	0.4085	-0.18	-0.44	0.165				0.473	0.324	0.12	0.745573	-0.00226	-0.151020576	0.148757708
2783	Q7Z3V4	UBE3B	ubiquitin protein ligase E3B	89910	ENSG00000151148	7	0.112	0.199	0.089	0.047	-0.26	-0.4	-0.17	-0.1	-0	0.1367	0.2457	0.1273	-0.087	-0.167	0.0268	0.2496	0.1653	-0.059	-0.28	-0.59	-0.33	-0.3	-0.32	0.014	-0.32	0.139	0.074	0.125997	0.157157	-0.125175723	0.282333123
407	B1AKD8	CROCC	ciliary rootlet coiled-coil, rootletin	9696	ENSG00000058453	1	-0.82	-2.48	-0.57	0.409	-0.73	0.513	0.874	0.473	0.891	-0.974	-2.44	-0.753	0.0342	-1.927	-0.13	0.9241	-1.333	-0.924	-0.28	-1.06	-0.68	1.442	-0.14	-0.94	0.402	0.575	0.685	0.3418	-0.16113	0.675329949	-0.836462106
2862	Q8IY21	DDX60	DExD/H-box helicase 60	55601	ENSG00000137628	2							-0.24	-0.4	-0.07							-0.266	-0.084	-0.08							-0.07	0.104	0.173	0.211888	-0.3045	-0.092917273	-0.211587069
1230	O75864	PPP1R37	protein phosphatase 1 regulatory subunit 37	284352	ENSG00000104866	4	0.061	0.467	0.274	-0.02	0.389	-0.05	0.047	-0.13	0.148	0.2741	0.2179	0.1923	-7E-04	-0.433	-0.255	0.0909	0.2085	0.1053	-0.18	-1.09	-0.29	0.558	-0.01	0.178	-0.17	-0.05	0.044	0.426576	0.244025	0.087800241	0.156224312
1940	P46019	PHKA2	phosphorylase kinase regulatory subunit alp	5256	ENSG00000044446	3				0.212	0.448	0.226	0.261	0.19	0.464				0.192	-0.3	-0.251	0.4787	-0.409	0.2736				0.278	0.409	-0.02	0.691	0.365	0.392	0.199743	-0.05187	0.303077087	-0.354944751
2705	Q6P6B1	ERICH5	glutamate rich 5	203111	ENSG00000177459	3	-0.26	-0.55	-0.12				0.488	0.327	0.196	0.0712	0.0505	-0.372				0.2879	0.2683	-0.09	-0.55	0.147	-0.03				0.389	0.371	0.231	0.94344	-0.0786	-0.022382187	-0.056217054
2573	Q32M84	BTBD16	BTB domain containing 16	118663	ENSG00000138152	2				-0.12	0.35	-0.91	-0.65	-0.21	-0.46				0.2949	0.3345	0.1812	-0.357	0.5297	0.1355				-1	-0.01	0.36	-0.34	-0.3	-0.15	0.226483	-0.09541	-0.521852868	0.426442747
357	A2A2Q9	AAR2	AAR2 splicing factor	25980	ENSG00000131043	1							-0.6	-0.32	-0.37							-0.117	-0.258	-0.159							-0.04	-0.67	-0.37	0.515105	-0.06915	-0.251399294	0.182246863
1955	P47712	PLA2G4A	phospholipase A2 group IVA	5321	ENSG00000116711	4	-0.2	0.212	0.083	-0.32	0.021	0.006	-0.03	0.195	0.127	0.0463	-0.06	0.1032	-0.073	0.5562	-0.11	-0.046	0.3482	-0.007	-0.48	0.153	0.442	-0.41	0.303	0.027	-0.04	0.021	0.021	0.813022	0.007432	-0.073731558	0.081163062

2871	Q8IZ21	PHACTR4	phosphatase and actin regulator 4	65979	ENSG00000204138	4	0.339	0.497	-0.32	0.063	-0.12	-0.12	-0.15	0.1	0.094	-0.261	0.155	0.1328	0.0254	-0.097	-0.149	0.4497	0.069	0.4025	-0.16	-0.44	-0.24	-0.45	0.034	-0.13	0.257	0.165	0.131	0.478946	0.134558	-0.038161177	0.172719237
2879	Q8IZQ1	WDFY3	WD repeat and FYVE domain containing 3	23001	ENSG00000163625	4	0.089	0.052	-0.23	-0.34	-0.62	-0.36	0.152	0.28	0.38	1.0647	0.1572	-0.115	-0.303	-0.407	-0.422	0.52	0.3654	0.3918	0.441	-0.21	-0.46	-0.8	-0.56	-0.27	0.345	0.278	0.343	0.606052	0.034109	-0.20414504	0.238253597
2085	P54107	CRISP1	cysteine rich secretory protein 1	167	ENSG00000124812	12	0.283	-0.84	0.109	0.253	0.544	-0.31	0.1	0.471	0.475	-0.11	-0.143	0.0964	0.12	-0.303	0.0969	0.1677	-0.534	-0.604	0.55	0.803	0.056	0.245	-0.14	-0.01	0.078	-0.09	-0.66	0.485123	0.027847	0.255363632	-0.227516324
1634	Q5TEJ0	RPA2	replication protein A2	6118	ENSG00000117748	2				-0.03	0.182	-0.04	-0.29	-0.19	-0.26				-0.065	-0.26	0.3092	-0.085	-0.011	-0.199				-0.25	-0.27	0.208	-0.01	-0.27	0.038	0.919527	-0.01311	-0.053994032	0.040885882
3193	Q96SM3	CPXM1	carboxypeptidase X, M14 family member 1	56265	ENSG00000088882	1							0.275	0.434	0.04							0.2073	0.1443	0.0979							0.514	-0.16	0.227	0.913396	0.055492	0.099719632	-0.044227354
3716	Q9Y2G5	POFUT2	protein O-fucosyltransferase 2	23275	ENSG00000186866	5	0.088	-0.08	-0.22	-0.06	0.092	-0.15	-0.48	0.055	-0.26	0.3158	0.204	0.0663	-0.131	0.4639	0.0298	0.0307	-0.273	-0.545	-0.02	0.522	0.091	-0.33	-0.09	-0	0.242	-0.12	-0.31	0.652701	-0.10883	-0.129616601	0.020785169
508	C9JEU5	FGG	fibrinogen gamma chain	2266	ENSG00000171557	11	-1.44	-1.42	-1.53	-0.95	-0.93	-0.64	-0.63	-0.06	-0.97	-1.107	-0.895	-1.259	-0.674	-0.929	-0.767	-0.547	-0.854	-1.009	-1.2	-1.09	1.192	3.378	-0.77	0.432	-0.17	-0.77	-0.72	0.176741	-0.98394	-0.057693188	-0.926246978
1259	O94830	DDHD2	DDHD domain containing 2	23259	ENSG00000085788	2	-0.21	0.47	-0.39				0.374	0.36	0.143	-0.09	-0.071	0.0678				0.5901	0.0791	0.2051	-0.13	-0.16	0.066				0.331	-0.07	0.421	0.960006	0.047134	-0.005970646	0.053105029
2647	Q5TDF0	NTPCR	nucleoside-triphosphatase, cancer-related	84284	ENSG00000135778	4	-0.11	0.144	-0.09	0.109	-0.13	-0.48	0.446	0.2	0.552	0.1973	0.2508	0.0918	0.143	0.0603	0.0396	0.4614	0.0984	0.1003	-0.06	-0.36	-0.38	-0.22	-0.26	0.194	0.024	0.036	0.183	0.223236	0.165471	-0.088503388	0.253974059
2673	Q66LE6	PPP2R2D	protein phosphatase 2 regulatory subunit Bo	55844	ENSG00000175470	8	0.018	0.363	0.043	0.08	-0.1	-0.26	0.319	0.085	0.248	0.1794	-0.151	0.0012	0.2166	0.1501	0.068	0.2091	-0.021	0.0893	0.083	-1.05	-0.29	-0.05	-0.13	0.127	0.547	0.027	0.333	0.678706	0.132784	0.005759176	0.127024763
3524	Q9NWM3	CUEDC1	CUE domain containing 1	404093	ENSG00000180891	1							0.783	0.964	0.746							1.0077	0.2862	0.3988							1.016	0.785	0.296	0.722846	0.132407	0.266991973	-0.134585132
266	F5GZ78	PXN	paxillin	5829	ENSG00000089159	4	0.29	0.359	0.224	-0.12	0.019	-0.23	-0.79	-0.35	-0.06	0.0403	-0.035	0.1667	0.2378	-0.022	0.0325	-0.605	-0.074	-0.156	-0.28	-1.19	0.012	0.037	0.124	0.283	-0.28	-0.28	-0.51	0.625717	0.159971	-0.026800312	0.186771723
154	A0A0A0MS/	PVR	PVR cell adhesion molecule;poliovirus recep	5817	ENSG00000073008	1							-0.08	0.179	-0.16							0.3591	-0.128	-0.237							0.475	0.136	-0.31	0.91873	-0.12142	-0.01899635	-0.102425009
3757	Q9Y4H2	IRS2	insulin receptor substrate 2	8660	ENSG00000185950	1							-0.27	-0.02	-0.12							0.1306	-0.048	-0.041							0.194	-0.13	0.003	0.479203	-0.1599	-0.152618358	-0.007278872
2751	Q6ZT12	UBR3	ubiquitin protein ligase E3 component n-rec	130507	ENSG00000144357	3	-0.4	-0.43	-0.58				0.007	0.046	0.098	0.6113	0.1054	-0.328				0.1106	-0.053	-0.039	0.352	0.276	0.089				0.37	-0	0.098	0.172509	-0.40593	-0.27682644	-0.129106239
2011	P49903	SEPHS1	selenophosphate synthetase 1	22929	ENSG00000086475	2							-0.01	0.163	0.103							0.2451	0.015	-0.014							0.309	0.241	0.202	0.29455	-0.16562	0.002956071	-0.16857222
1614	P14778	IL1R1	interleukin 1 receptor type 1	3554	ENSG00000115594	5	-0.21	0.387	0.386	0.044	0.129	-0.15	-0.59	0.022	-0.23	-0.082	0.1974	0.0273	0.2117	-0.234	-0.032	-0.298	0.0464	-0.676	-0.13	0.102	-0.37	0.446	0.174	0.084	-0.17	-0.09	-0.15	0.868232	-0.0127	0.068881301	-0.081581713
1811	P30479	HLA-B	major histocompatibility complex, class I, B	3106	ENSG00000224608	8	-0.39	-2	-1.79	0.313	-0.4	-1.14	0.002	0.209	-0.17	0.6839	-0.601	1.1785	-0.27	-0.017	-1.463	0.0916	-1.018	-0.865	0.75	-1.2	0.896	2.405	-1.22	0.366	0.525	-1.06	-0.8	0.525175	-0.66948	-0.342311567	-0.327172844
1636	Q5TD07	NQO2	N-ribosyldihydronicotinamide:quinone reduc	4835	ENSG00000124588	5	0.702	-0.3	-0.68	-0.03	0.226	-0.25	-0.54	-0.22	0.083	0.2685	-0.487	-0.396	-0.285	-0.239	-0.247	0.3174	0.0856	0.5023	-0.34	-0.26	0.045	0.124	-0.15	0.022	0.016	-0.17	-0.33	0.93496	0.006143	-0.058164368	0.064306976
1246	O76038	SCGN	secretagogin, EF-hand calcium binding prot	10590	ENSG00000079689	5	0.498	0.377	-0.31				-0.87	-1.17	0.385	0.9085	-0.005	1.0991				-0.855	-0.514	-0.761	-0.52	-0.21	0.016				5E-04	-0.32	0.566	0.943801	-0.10315	-0.160676796	0.057530141
3431	Q9HAV0	GNB4	G protein subunit beta 4	59345	ENSG00000114450	11	0.328	0.438	-0.77	0.043	0.103	-0.29	-0.6	0.018	-0.37	-0.242	-0.123	0.0004	0.1344	-0.185	0.6543	0.8046	-0.278	0.2514	-0.38	-0.78	-0.31	-0.2	-0.52	-0.23	-0.03	-0.31	0.103	0.207211	0.173628	-0.235301953	0.408929999
2315	Q04941	PLP2	proteolipid protein 2	5355	ENSG00000102007	1				-0.25	0.117	-0.28	-0.92	-0.82	-0.69				-0.019	-0.133	-0.068	-0.56	-0.445	-0.739				0.021	-0.22	-0.1	-0.11	-0.61	-0.52	0.634777	-0.21843	-0.147171057	-0.071254365
1835	P32856	STX2	syntaxin 2	2054	ENSG00000111450	1							0.086	0.285	0.366							0.4771	-0.037	0.3934							-0.3	0.258	0.375	0.82441	0.135008	-0.032395416	0.167403128
55	A0A087WW	KIF1B	kinesin family member 1B	23095	ENSG00000054523	11	-0.22	-0.15	-0.25				-0.29	-0.7	0.662	-0.053	-0.19	-0.141				-0.676	0.2833	-0.072	-0.3	-0.21	0.19				-0.5	0.53	0.179	0.85807	-0.14069	-0.016056761	-0.124631864
561	D6RJE5	ABCC3	ATP binding cassette subfamily C member	8714	ENSG00000108846	1				-0.99	-0.26	0.102	0.535	0.52	0.168				-0.948	-0.37	-0.27	-0.236	-0.014	0.0479				-0.72	0.513	-0.15	1.022	-0.36	-1	0.762206	0.126791	0.311174719	-0.184383671
776	G3V279	ERH	ERH mRNA splicing and mitosis factor	2079	ENSG00000100632	1							0.32	0.083	0.214							0.1819	0.2068	0.0226							-0.19	0.037	0.157	0.404968	0.203886	0.068790841	0.135095134
1770	P27658	COL8A1	collagen type VIII alpha 1 chain	1295	ENSG00000144810	3	-0.04	-0.28	0.243	-0.81	-0.26	0.807	-0.02	-0.53	-0.14	0.1962	-0.175	-0.072	-1.014	0.1819	0.6032	-0.087	-0.382	0.1317	0.78	-0.13	0.135	-0.88	0.113	-0.17	0.529	0.343	-0.44	0.867939	-0.1464	-0.046707151	-0.099692478
1155	O60487	MPZL2	myelin protein zero like 2	10205	ENSG00000149573	3	-0.5	0.158	0.313	-0.25	0.126	0.268	-0.69	0.076	-0.38	-0.199	-0.112	-0.272	0.0188	0.1222	-0.35	-0.551	0.0291	-0.307	-0.35	0.905	-0.25	-0.21	-0.4	-0.07	0.431	-0.23	-0.35	0.834029	-0.03826	0.083575841	-0.12183816
239	A0A0J9YWk	COBL	cordon-bleu WH2 repeat protein	23242	ENSG00000106078	2	-0.13	0.487	0.005				-0.35	-0.41	-0.17	-0.053	0.1377	0.2456				0.3379	-0.41	-0.332	-0.14	-0.17	-0.13				-0.11	-0.13	-0.14	0.790153	0.044377	-0.080525431	0.124902115
3628	Q9UID3	VPS51	VPS51 subunit of GARP complex;VPS51, (	738	ENSG00000149823	2	-0.39	0.057	0.233				-0.11	0.154	0.515	0.1035	-0.051	0.1758				0.2467	-0.3	0.0684	-0.04	-0.43	0.018				-0.05	0.114	0.218	0.833965	0.105569	0.036439015	0.069129574
2848	Q8IW52	SLITRK4	SLIT and NTRK like family member 4	139065	ENSG00000179542	1	0.531	-0.51	0.238				-0.27	0.552	-0.42	0.3223	0.363	0.4789				0.657	0.3602	-0.165	-0.26	-0.34	0.05				-0.38	-0.17	-0.03	0.172637	0.208299	-0.316182586	0.524481761
1640	P16401	HIST1H1B	histone cluster 1 H1 family member b;H1.5 l	3009	ENSG00000184357	5	-0.3	-0.37	-0.54	-0.1	0.036	-0.41	-0.68	-0.68	-0.74	-0.051	0.0255	-0.236	-0.52	0.1018	-0.461	-0.069	-1.13	-1.109	0.397	0.36	1.963	0.166	-0.24	0.719	-0.03	-0.28	-0.43	0.10848	-0.71177	-0.037644815	-0.67412473
2476	Q14697	GANAB	glucosidase II alpha subunit	23193	ENSG00000089597	51	-0.38	-0.5	-0.18	-0.72	0.388	-0.51	-0.48	-0.27	-0.6	0.6284	0.7486	-0.31	-0.708	0.7359	-0.739	-1.007	-0.553	-0.207	0.294	0.773	0.104	-0.41	-0.04	-0.38	0.341	-0.67	-0.51	0.595139	-0.30532	-0.204264258	-0.101052692
1333	P00558	PGK1	phosphoglycerate kinase 1	5230	ENSG00000102144	37	0.325	0.119	-0.11	0.263	-0.59	-0.46	-0.09	-0.11	-0.16	-0.025	0.4372	0.0635	0.375	0.0768	0.0418	0.3052	-0.236	-0.613	-0.25	-0.2	0.078	-0.46	-0.48	0.272	0.25	0.172	-0.29	0.65744	0.010616	-0.137369265	0.147985681
2473	Q14694	USP10	ubiquitin specific peptidase 10	9100	ENSG00000103194	2	0.731	0.074	-0.1				-0.51	-0.31	-0.28	-0.007	0.1168	0.3631				0.0375	-0.007	-0.144	0.002	-0.93	-0.22				0.134	-0.06	-0.08	0.609949	0.127733	-0.124514554	0.252247497
1079	O15195	VILL	villin like	50853	ENSG00000136059	3	-0.09	-0.41	-0.45				-1.39	-0.61	-0.98	0.6495	-0.193	-0.124				-0.255	-0.928	-0.228	0.143	0.395	-0.37				-0.23	-0.92	0.66	0.286497	-0.60016	-0.474043753	-0.126114693
3157	Q96K17	BTF3L4	basic transcription factor 3 like 4	91408	ENSG00000134717	1	0.099	0.132	0.079				-0.03	-0.26	0.285	-0.217	-0.057	-0.22				0.0049	0.0633	0.0343	-0.07	-0.73	-0.2				0.08	-0.16	-0.01	0.352934	0.229719	0.115899313	0.113819669
444	B4E107	ATE1	arginyltransferase 1	11101	ENSG00000107669	4	0.322	0.287	0.178	0.082	-0	-0.35	-0.5	-0.28	-0.13	0.3901	0.2578	0.1039	0.1377	-0.184	0.019	-0.358	0.0538	-0.361	-0.17	-0.82	-0.48	-0.51	-0.18	0.192	-0.23	0.014	-0.15	0.279215	0.216174	-0.049808289	0.26598278
311	A0A2R8Y44	DYRK1A	dual specificity tyrosine phosphorylation regu	1859	ENSG00000157540	2							0.231	0.531	0.302							0.0892	0.2083	0.1743							0.171	0.307	0.368	0.331572	0.072846	0.197436146	-0.124590484
332	H7C560	BRAF	B-Raf proto-oncogene, serine/threonine kina	673	ENSG00000157764	7	-0.2	-0.08	-0.06				-0.22	-0.09	-0	0.2353	-0.184	-0.023				-0.112	-0.015	-0.249	-0.49	-1.19	-0.17				-0.06	-0.07	0.129	0.484842	0.200888	-0.050085161	0.250972724
1650	P17252	PRKCA	protein kinase C alpha	5578	ENSG00000154229	2	0.23	-0.14	-0.21				0.067	0.002	0.049	-1.252	0.0173	-0.002				0.3347	-0.022	0.1578	-0.13	-0.2	4.452				-0.06	0.199	0.288	0.501271	-0.75939	0.12734328	-0.88673246
481	C9IYN7	ELP6	elongator acetyltransferase complex subunit	54859	ENSG00000163832	4							-0.55	-0.11	-0.38							-0.196	-0.366	-0.368							0.095	-0.34	-0.35	0.764167	-0.14878	-0.036737546	-0.11204394
2821	Q86X52	CHSY1	chondroitin sulfate synthase 1	22856	ENSG00000131873	4	-0.02	0.188	-0.19	0.385	-0.14	0.176	0.383	0.345	0.289	0.0455	0.0806	0.1166	-0.117	-0.088	0.213	0.5704	0.1522	0.5884	-0.33	0.03	0.087	-0.3	-0.16	0.06	0.355	0.069	0.364	0.50008	0.138314	-0.016675291	0.15498956
3397	Q9H4P4	RNF41	ring finger protein 41	10193	ENSG00000181852	2							-0.06	0.094	0.087							0.1899	-0.084	0.0844							0.062	0.018	0.148	0.936345	-0.03666	-0.024090347	-0.012572519
881	H7BZ18	MCFD2	multiple coagulation factor deficiency 2	90411	ENSG00000180398	1	-0.28	-0.85	-0.22	0.373	-0.07	0.394	-0.63	-0.55	-0.21	0.0493	0.7427	0.2323	0.1338	0.2782	-0.068	0.1053	-0.632	-0.475	-0.76	-0.35	0.023	-0.31	0.375	0.394	0.269	0.078	-0.67	0.565471	-0.12001	-0.266555412	0.146545
634	E9PDH4	PIKFYVE	phosphoinositide kinase, FYVE-type zinc fin	200576	ENSG00000115020	2				-1.96	-0.87	-0.36	-0.2	-0.16	-0.06				-1.157	1.7005	-0.81	-0.392	-0.1	0.3098				-2.36	-0.97	-0.84	-0.09	0.118	0.291	0.634612	0.041095	-0.526557549	0.567652757
681	E9PNU4	STX5	syntaxin 5	6811	ENSG00000162236	2	0.25	0.232	-0.16				0.24	0.193	0.359	0.1289	-0.071	-0.006				0.4764	-0.05	0.3725	0.071	-0.66	-0.13				0.328	0.239	0.19	0.634908	0.178519	0.043812956	0.134706266
2486	Q14CX7	NAA25	N(alpha)-acetyltransferase 25, NatB auxiliar	80018	ENSG00000111300	3	0.208	-0.05	-0.21				-0.09	0.076	0.252	0.0887	0.3217	0.2149				0.0081	-0.107	-0.143	-0.34	-0.23	-0.01				0.035	-0.07	0.043	0.431797	0.126368	-0.033382631	0.159750151
2865	Q8IYB7	DIS3L2	DIS3 like 3'-5' exoribonuclease 2	129563	ENSG00000144535	5	0.104	0.059	-0.01	-0.24	-0.16	-0.62	-0.16	-0.19	0.074	0.2753	0.0477	0.1385	0.0989	-0.133	-0.307	-0.226	-0.135	-0.292	-0.22	-0.69	-0.08	-0.31	-0.5	-0.05	-0.09	-0	-0.05	0.477357	0.093872	-0.067670268	0.161542045
2300	Q02487	DSC2	desmocollin 2	1824	ENSG00000134755	2	-0.2	0.049	0.016	-0.62	-0.86	-0.19	-0.09	-0.39	0.062	0.7328	-0.129	0.3236	-0.068	0.4668	-0.169	0.0469	0.3388	0.081	-0.16	-0.17	0.032	-0.68	-0.3	0.223	-0.69	-0.06	-0.15	0.103261	-0.02972	-0.427880827	0.398162572
3364	Q9H1Z4	WDR13	WD repeat domain 13	64743	ENSG00000101940	2							0.036	0.123	0.224							0.7337	-0.298	0.2415							0.351	0.439	0.078	0.887331	-0.16132	-0.097873626	-0.063443184
1384	P02671	FGA	fibrinogen alpha chain	2243	ENSG00000171560	9	-1.88	-1.88	-1.64	-0.68	-0.67	-0.43	-1.87	-0.58	-2.05	-1.464	-1.241	-1.701	-0.642	-0.547	-0.661	-1.754	-1.126	-1.79	-1.5	-0.75	1.265	3.048	-1.05	0.143	-1.1	-1.54	-1.7	0.268827	-0.94583	-0.084117995	-0.861714342
564	D6RBR7	ZNF330	zinc finger protein 330	27309	ENSG00000109445	3	0.042	0.012	-0.13	-0.13	-0.22	-0.22	0.019	-0.1	0.283	-0.015	0.0817	0.0686	-0.217	-0.219	-0.049	-0.129	0.2545	0.1964	0.066	-0.81	-0.12	-0.73	-0.18	-0.11	0.033	0.105	0.121	0.450812	0.13092	-0.046324218	0.177243721
1272	O95164	UBL3	ubiquitin like 3	5412	ENSG00000122042	4	-0.17	0.122	-0.18	0.043	0.047	-0.18	-0.35	0.327	0.045	-0.123	0.2385	0.1274	0.2593	-0.033	0.0405	0.2739	0.3494	-0.029	-0.36	-0.33	0.286	-0.65	-0.35	-0.16	-0.4	0.34	0.514	0.34034	0.089927	-0.155779338	0.245706053
1932	P43405	SYK	spleen associated tyrosine kinase	6850	ENSG00000165025	1							0.209	0.43	0.094							0.307	-0.087	0.1334							0.114	-0.23	0.567	0.899106	0.093835	0.126544555	-0.032709725
846	H3BMF2	JMJD8	jumonji domain containing 8	339123	ENSG00000161999	2	-0.39	-1.4	-0.36	-0.21	-0.37	-0.16	0.137	0.226	0.502	-0.203	-0.331	-0.258	-0.584	-0.039	0.1611	-0.006	-0.287	-0.205	-0.12	1.108	0.242	0.109	0.005	-0.26	0.351	0.163	-0.04	0.224767	-0.39808	-0.028739385	-0.369335884
2702	Q6P499	NIPAL3	NIPA like domain containing 3	57185	ENSG00000001461	2	0.235	0.313	0.281				-0.02	0.023	-0.15	0.1849	0.227	0.3436				-0.044	-0.112	-0.053	-0.18	-0.39	-0.02				0.13	0.109	-0.11	0.348719	0.191739	0.023050689	0.168688772
2453	Q14264	ERV3-1	endogenous retrovirus group 3 member 1, e	2086	ENSG00000213462	2	1.141	-0.98	0.62	0.703	0.06	1.044	0.455	0.432	0.712	0.2235	0.2399	0.6428	-1.126	-0.38	-1.354	-0.783	-0.937	-1.056	0.69	-0.04	-0.39	-1.21	-1.35	-1.49	-0.92	0.91	0.691	0.146629	0.810393	0.969045164	-0.158652327
3519	Q9NVG8	TBC1D13	TBC1 domain family member 13	54662	ENSG00000107021	7	-0.11	0.15	-0.08	-0.11	-0.22	-0.15	-0.19	-0.07	0.44	0.2814	0.2228	0.0579	-0.073	0.0128	0.0604	0.488	0.0337	0.0508	-0.1	-0.75	0.013	-0.24	-0.16	-0.09	0.246	0.063	0.262	0.311123	0.045551	-0.163970058	0.20952134
1271	O95163	ELP1	elongator complex protein 1	8518	ENSG00000070061	10	-0.2	-0.08	-0.14	-0.04	0.187	0.282	0.02	0.273	0.19	0.456	-0.04	-0.029	0.1819	0.1479	0.0576	0.0905	-0.037	-0.092	-0.05	-0.18	-0.1	-0.03	0.253	0.228	0.154	-0.05	0.16	0.920046	0.011934	-0.027039263	0.038972982
1017	O00505	KPNA3	karyopherin subunit alpha 3	3839	ENSG00000102753	4	0.184	0.501	0.216	0.476	0.445	-0.32	-0.14	0.11	0.064	0.383	0.1807	0.1355	0.1259	-0.176	-0.083	-0.556	0.121	-0.11	-0.09	-0.44	-0.17	0.031	-0.26	0.334	-0.27	-0.1	-0.24	0.196525	0.303162	0.168803591	0.13435874
147	A0A0A0MRN	OGFR	opioid growth factor receptor	11054	ENSG00000060491	2				0.199	0.064	0.072	-0.08	-0.1	-0.27				0.2084	0.4707	0.1611	0.0627	-0.12	0.2466				-0.01	0.084	0.033	0.351	-0.43	-0.27	0.365462	0.02007	-0.191846949	0.21191691
53	A0A0G2JQH	CYFIP1	cytoplasmic FMR1 interacting protein 1	23191	ENSG00000280618	8							0.31	0.184	0.304							0.1426	0.496	0.321							0.22	0.126	0.217	0.562316	0.078353	-0.054011256	0.132363876
73	A0A087WYL	SEZ6L2	seizure related 6 homolog like 2	26470	ENSG00000174938	6	-0.44	-0.41	-0.73	-0.14	-0.19	0.459	-0.57	0.174	0.013	0.6904	-0.332	0.2886	-0.393	0.3467	-0.44	0.3006	-0.228	0.0191	-0.25	-0.49	0.045	-0.07	0.503	-0	4E-04	0.726	-0.63	0.589895	-0.18403	-0.231206077	0.047179793
1238	O75915	ARL6IP5	ADP ribosylation factor like GTPase 6 intera	10550	ENSG00000144746	2	0.286	0.275	0.177	-0.21	0.114	-0.55	-0.55	-0.36	-0.36	0.3295	0.1709	0.2975	0.1781	-0.128	0.0297	-0.43	-0.233	-0.523	-0.04	-0.31	0.527	1.188	-0.29	0.109	0.445	0.155	-0.09	0.373127	-0.31836	-0.097242587	-0.22112214
2595	Q53H96	PYCR3	pyrroline-5-carboxylate reductase 3	65263	ENSG00000276657	5	-0.22	0.02	-0.34	-0.02	0.265	-0.23	0.043	0.104	0.174	-0.043	-0.066	-0.1	-0.259	-0.274	-0.055	-0.144	0.2796	0.1921	-0.18	-0.14	0.05	0.266	-0.08	0.207	0.223	0.045	0.041	0.624813	-0.07311	0.028157488	-0.101267959
1339	P01008	SERPINC1	serpin family C member 1	462	ENSG00000117601	5	-0.45	-0.44	-0.22	-0.4	0.173	0.23	-0.56	-0.37	-0.85	0.5336	0.2306	0.3472	-0.405	-0.232	-0.589	-0.477	-0.34	-0.651	-0.22	-0.3	1.149	1.104	0.392	0.158	-0.07	0.57	-1.05	0.259536	-0.51303	-0.144524942	-0.368502575
1385	P02675	FGB	fibrinogen beta chain	2244	ENSG00000171564	16	-1.46	-1.13	-1.34	-0.48	-0.81	-0.14	0.628	0.203	-0.2	-1.047	-0.913	-1.164	-0.66	-0.347	-0.361	0.1229	-0.143	0.1346	-1.17	-1.23	1.001	2.348	-0.55	0.124	0.159	0.033	0.185	0.358629	-0.62542	-0.039407191	-0.58601775
152	A0A0A0MSI	MYD88	MYD88 innate immune signal transduction a	4615	ENSG00000172936	5	0.437	0.904	0.195	0.385	0.507	0.256	-0.17	-0.24	-0.17	0.4609	0.2059	0.4885	0.6983	-0.666	0.6105	-0.028	-0.157	-0.18	0.167	-0.3	-0.03	-0.94	-0.92	-0.88	-0.06	-0.33	0.052	0.112848	0.591711	0.074069257	0.517641736
584	E5RGR6	GFRA2	GDNF family receptor alpha 2	2675	ENSG00000168546	4	0.001	-0.1	-0.45				0.015	-0.22	0.359	-0.106	0.0968	-0.173				0.2539	0.4532	-0.287	-0.34	-0.24	0.126				-0.04	0.638	-0.34	0.891266	-0.03641	-0.106598878	0.07018558
3122	Q96EF0	MTMR8	myotubularin related protein 8	55613	ENSG00000102043	1							0.262	0.334	0.376							0.5458	0.4681	0.5225							0.188	0.287	0.424	0.147476	0.02415	-0.188151727	0.212301731
827	H0YIE9	FBXO21	F-box protein 21	23014	ENSG00000135108	3	0.249	0.151	0.059	0.065	0.454	-0.59	0.104	-0.24	0.033	-0.058	0.0773	0.0149	-0.131	-0.236	0.0102	-0.007	0.0274	0.1759	-0.26	-0.78	-0.27	-0.69	-0.5	-0.1	0.215	0.293	0.345	0.430365	0.22492	0.045843166	0.17907727
2833	Q8IUB5	WFDC13	WAP four-disulfide core domain 13	164237	ENSG00000168634	1				1.091	0.484	0.287	-0.55	0.145	0.101				0.3364	-1.214	0.1993	-0.808	-0.332	-0.866				-0.53	-0.81	-0.66	-0.86	-1.04	-1.36	0.070289	1.138536	0.707897993	0.430637814
882	H7BZJ3	PDIA3	protein disulfide isomerase family A membe	2923	ENSG00000167004	8	0.079	-0.06	-0.25				0.204	0.45	0.089	-0.17	-0.27	0.051				-0.338	-0.174	-0.021	-0.1	0.082	-0.12				0.184	-0.26	0.438	0.316538	0.048566	0.238884136	-0.190318572
2357	Q10589	BST2	bone marrow stromal cell antigen 2	684	ENSG00000130303	1	0.04	0.07	-0.5	-0.15	0.236	-0.12	-0.91	0.478	-0.89	-0.309	-0.483	-0.099	-0.358	0.3995	-0.533	-0.023	-0.165	-0.198	-0.11	-0.41	0.026	1.396	0.037	-0.06	-0.62	0.393	-0.92	0.805087	-0.16465	0.003017665	-0.167671703
841	H0YNX7	GCHFR	GTP cyclohydrolase I feedback regulator	2644	ENSG00000137880	3	0.452	0.273	0.198				0.184	0.481	0.455	0.1393	0.2882	-0.02				0.4073	-0.13	0.2698	-0.13	-0.59	-0.13				0.165	0.319	0.212	0.183487	0.366497	0.181399825	0.185096795
3443	Q9HCN4	GPN1	GPN-loop GTPase 1	11321	ENSG00000198522	2							-0.15	0.252	0.387							-0.037	-0.127	-0.065							1.303	0.126	0.684	0.242364	-0.5399	0.240482669	-0.780383315
2933	Q8NDC0	MAPK1IP1	mitogen-activated protein kinase 1 interactin	93487	ENSG00000168175	1	-0.18	0.492	-0.45	-0.04	0.106	-0.08	-0.12	0.074	-0.01	-0.184	-0.166	-0.029	0.0792	-0.088	0.0856	0.2929	0.1584	0.4532	-0.12	0.184	0.278	-0.37	-0.33	-0.23	0.058	0.15	0.219	0.773395	-0.00591	-0.089528847	0.083623748
2581	Q3ZCM7	TUBB8;TU	tubulin beta 8 class VIII;tubulin beta 7 pseu	347688	ENSG00000261456	9	0.155	0.564	-0.08	-0.2	-0.03	-0.52	0.028	0.244	0.197	0.0263	-0.073	0.0679	-0.487	-0.145	-0.167	-0.118	0.2862	0.05	-0.34	-1.57	-0.39	-0.73	-0.51	-0.26	0.045	0.33	0.454	0.311569	0.371894	0.102835136	0.26905921

2856	Q8IXA5	SPACA3	sperm acrosome associated 3	124912	ENSG00000141316	2							0.636	1.494	0.653							-0.178	-0.233	-0.281							0.054	0.092	-0.21	0.085711	0.947707	1.157976116	-0.210269254
1675	P19623	SRM	spermidine synthase	6723	ENSG00000116649	3	0.01	0.254	-0.1	-0.41	-0.12	-0.51	-0.33	-0	0.017	0.256	0.0724	-0.044	-0.264	-0.129	-0.002	-0.315	-0.153	-0.298	-0.16	-0.57	0.074	-0.3	-0.29	-0.09	0.25	-0.08	-0.37	0.868062	0.035652	-0.036028275	0.071680601
3851	U3KQP0	BASP1	brain abundant membrane attached signal p	10409	ENSG00000176788	10							0.249	0.329	0.299							0.7362	0.3082	0.1623							-0.14	0.177	-0.07	0.237309	0.301241	-0.109893903	0.411134683
1070	O15084	ANKRD28	ankyrin repeat domain 28	23243	ENSG00000206560	1	0.355	0.104	0.084	0.335	0.618	-0.1	0.149	0.368	0.208	0.4646	-0.113	0.2217	0.0702	0.0823	0.1907	0.1522	0.0898	-0.175	-0.43	-0.05	0.411	-0.46	0.062	0.054	0.282	-0.04	0.071	0.236456	0.245883	0.126642339	0.119240237
3522	Q9NVQ4	FAIM	Fas apoptotic inhibitory molecule	55179	ENSG00000158234	3	0.142	0.227	0.085	-0.15	-0.27	-0.15	0.021	-0.11	0.162	0.0781	0.1856	0.1209	-0.064	0.0686	0.1306	0.0802	-0.114	-0.101	-0.2	-0.57	-0.19	-0.28	-0.09	-0.02	0.128	-0.11	0.164	0.256201	0.125881	-0.047540718	0.173421896
661	E9PJM3	FBXO3	F-box protein 3	26273	ENSG00000110429	2							-0.12	0.077	0.173							0.1065	0.0025	0.052							0.421	0.121	0.276	0.261103	-0.23059	-0.0119217	-0.218664412
858	H3BRQ0	PPCDC	phosphopantothenoylcysteine decarboxylase	60490	ENSG00000138621	2	-0.38	-0.42	-0.55				0.142	-0.01	0.095	-0.284	-0.313	-0.289				0.3421	0.1292	0.1805	-0.4	-0.2	-0.75				-0.13	0.079	-0.18	0.559821	0.076043	-0.150139044	0.22618214
2896	Q8N398	VWA5B2	von Willebrand factor A domain containing 5	90113	ENSG00000145198	3	-0.29	0.345	-0.05	0.355	0.097	-0.25	-0.39	0.203	-0.31	0.2428	0.3227	0.1711	0.0277	-0.459	0.2053	0.1333	-0.036	-0.166	-0.28	0.207	0.176	-0.25	-0.7	-0.31	0.026	-0.09	0.112	0.575718	0.089668	-0.08192075	0.171588502
3412	Q9H8T0	AKTIP	AKT interacting protein	64400	ENSG00000166971	3	0.178	0.391	0.241				-0.46	-0.31	-0.26	0.3687	0.2381	0.134				-0.356	-0.163	-0.326	0.06	-0.2	0.09				-0.14	-0.1	-0.03	0.981958	0.01626	-0.020465538	0.036725971
3177	Q96PM5	RCHY1	ring finger and CHY zinc finger domain cont	25898	ENSG00000163743	3	-0.04	-0.19	-0.14	0.013	-0.02	-0.16	-0.29	-0.41	-0.32	-0.248	-0.026	-0.063	0.1394	0.0379	0.1235	-0.338	-0.068	-0.261	-0.14	-0.32	-0.09	-0.33	-0.07	0.228	0.032	-0.09	-0.23	0.613054	-0.05979	-0.094676177	0.034881792
3062	Q92990	GLMN	glomulin, FKBP associated protein	11146	ENSG00000174842	2	-0.01	0.273	-0.24				0.402	0.444	0.424	0.3564	-0.244	-0.177				0.6888	0.4092	0.151	-0.41	-0.73	0.536				0.407	0.509	0.366	0.931429	0.103319	0.019010545	0.084308311
3751	Q9Y478	PRKAB1	protein kinase AMP-activated non-catalytic s	5564	ENSG00000111725	4	0.385	0.38	0.071	0.046	-0.13	-0.52	-0.5	-0.27	-0.21	0.3825	-0.041	0.3993	0.0901	-0.096	-0.021	-0.307	-0.039	-0.16	-0.19	-0.77	0.028	-0.52	-0.16	-0.06	-0.11	-0.23	-0.31	0.255432	0.176203	-0.104645387	0.280848765
2469	Q14669	TRIP12	thyroid hormone receptor interactor 12	9320	ENSG00000153827	4							-0.44	-0.46	-0.28							-0.31	-0.119	-0.504							-0.39	-0.14	-0.3	0.751485	-0.1107	-0.079602162	-0.031094427
3150	Q96IZ0	PAWR	pro-apoptotic WT1 regulator	5074	ENSG00000177425	4	0.155	0.222	-0.08	0.017	0.255	-0.33	0.291	0.307	0.356	0.009	0.0302	-0.055	-0.038	0.0241	0.0401	0.8832	0.311	0.1358	-0.13	-0.66	-0.12	-0.56	-0.28	0.191	0.861	0.162	0.319	0.637156	0.157609	-0.015902026	0.173511408
3003	Q8WUF5	PPP1R13L	protein phosphatase 1 regulatory subunit 13	10848	ENSG00000104881	4	0.012	-0.13	-0.32	0.014	0.113	-0.23	0.317	-0.03	0.348	-0.047	-0.069	-0.093	-0.072	0.1353	0.2646	0.1297	0.2456	0.0504	0.402	0.83	0.099	-0.44	-0.1	-0.04	0.032	0.024	0.071	0.842573	-0.08766	-0.05054055	-0.037120603
3359	Q9H1K6	TLNRD1	talin rod domain containing 1	59274	ENSG00000140406	2							0.269	0.47	0.401							0.3531	0.1213	0.1412							0.768	0.286	0.377	0.394993	-0.09693	0.174802621	-0.2717339
2999	Q8WU10	PYROXD1	pyridine nucleotide-disulphide oxidoreductas	79912	ENSG00000121350	1							0.073	0.287	0.175							0.0878	0.1922	0.0683							0.157	0.195	0.11	0.738521	0.024261	0.062224666	-0.037963713
1226	O75781	PALM	paralemmin	5064	ENSG00000099864	3	0.318	0.945	-0.01	0.183	0.544	-0.29	0.319	0.36	0.371	0.0444	0.2131	0.2741	0.2929	0.2642	0.4423	0.3467	0.2031	0.5607	-0.21	0.197	0.123	-0.89	-0.48	0.166	0.539	0.177	0.475	0.291305	0.29368	0.01056292	0.283116921
596	E7EM83	PTPRN2	protein tyrosine phosphatase receptor type N	5799	ENSG00000155093	4	0.619	-0.5	0.461	-0.09	-0.41	0.091	-0.25	-0.4	-0.01	-0.033	0.566	0.1777	-0.455	-0.348	-0.51	0.0819	0.1527	0.0663	-0.11	-0.95	0.299	-1	-0.53	0.138	0.031	-0.01	-0.38	0.525691	0.22553	-0.020329151	0.245859427
906	J3KN34	PTPN3	protein tyrosine phosphatase non-receptor ty	5774	ENSG00000070159	2	-0.32	-0.11	-0.29	-0.17	-0.3	-0.23	-0.52	-0.44	-0.35	-0.325	0.0927	0.2116	-0.052	0.7028	-0.132	-0.445	-0.383	-0.568	-0.11	-0.68	0.207	-0.53	-0.33	0.001	-0.33	0.225	-0.47	0.526368	-0.0786	-0.202885627	0.124284341
1163	O60518	RANBP6	RAN binding protein 6	26953	ENSG00000137040	8	0.096	0.256	0.371	0.046	0.117	0.184	0.091	0.078	0.231	0.5261	0.1859	0.2005	0.1384	0.0336	0.0957	0.2617	0.3323	0.2553	0.023	-0.32	0.127	-0.4	-0.34	0.356	-0.19	0.151	0.327	0.146285	0.192417	-0.06221429	0.254631714
1846	P34913	EPHX2	epoxide hydrolase 2	2053	ENSG00000120915	34							-0.24	-0.02	-0.19							-0.408	0.0882	0.0213							-0.15	-0.29	-0.3	0.714305	0.098995	-0.050360804	0.149355796
1588	P13640	MT1G	metallothionein 1G	4495	ENSG00000125144	4	0.109	0.704	-1.18	-0.45	-0.24	0.631	-1.06	0.311	-0.99	-0.805	-1.234	1.1213	0.1956	-0.776	0.3629	1.3446	-0.471	-1.161	-0.72	-1.67	-0.61	-0.49	1.175	0.621	1.171	-0.66	-0	0.973315	-0.11079	-0.083468255	-0.027317753
3001	Q8WUA8	TSKU	tsukushi, small leucine rich proteoglycan	25987	ENSG00000182704	3	-0.1	0.124	-0.24				0.11	0.12	0.351	-0.442	-0.452	0.5687				0.4288	0.1169	0.2164	-0.14	0.032	-0.06				0.463	0.32	0.243	0.920012	-0.08301	-0.012357039	-0.070649065
1217	O75569	PRKRA	protein activator of interferon induced protein	8575	ENSG00000180228	3	0.555	0.128	0.08	0.39	0.388	-0.02	0.012	-0.32	-0.01	-0.044	-0.019	0.1611	0.0655	0.0991	0.0345	-0.256	-0.083	-0.299	0.142	-0.74	-0.18	-0.33	-0.02	0.065	-0	-0.09	-0.4	0.15925	0.306461	0.170956489	0.135504634
219	F6UB75	AL645941.	novel protein	NA	ENSG00000248993	1	-0.73	-0.67	0.313				-0.3	-0.4	-0.13	-0.615	-0.338	-0.417				0.1916	-0.306	0.0044	-0.7	-0.34	1.535				-0.15	-0.43	-0.08	0.738736	-0.2919	-0.073808923	-0.218086417
3264	Q9BTE1	DCTN5	dynactin subunit 5	84516	ENSG00000166847	6	0.334	0.378	0.191	-0.05	0.118	-0.46	-0.68	-0.41	-0.38	0.2212	0.2035	0.1068	0.0536	0.0472	-0.043	-0.292	-0.049	-0.47	-0.11	-0.89	-0.2	-0.2	-0.4	-0.07	-0.34	-0.16	-0.27	0.313648	0.187178	-0.081995929	0.269173483
3000	Q8WU90	ZC3H15	zinc finger CCCH-type containing 15	55854	ENSG00000065548	4	0.437	0.067	0.015	5E-04	0.416	0.109	0.212	-0.01	0.248	0.3368	-0.074	0.1917	0.3138	0.211	-0.121	-0.019	0.2733	-0.016	0.073	-0.75	-0.13	0.14	0.319	0.455	0.209	0.012	0.176	0.744227	0.110708	0.044580032	0.066128457
2873	Q8IZ52	CHPF	chondroitin polymerizing factor	79586	ENSG00000123989	2							-0.03	0.214	0.164							0.3975	0.0445	0.0301							0.236	-0.09	-0.06	0.781553	0.084118	-0.041538658	0.12565698
1554	P11166	SLC2A1	solute carrier family 2 member 1	6513	ENSG00000117394	2				9E-04	-0.05	-0.22	-0.15	-0.25	-0.07				0.1138	-0.006	0.0681	-0.399	0.3167	-0.122				-0.21	-0.26	0.155	0.041	0.143	0.118	0.591972	-0.12102	-0.118110225	-0.002905326
1649	P17181	IFNAR1	interferon alpha and beta receptor subunit 1	3454	ENSG00000142166	1	-0.27	-0.27	-0.4				0.303	0.344	0.245	0.1597	-0.012	-0.12				0.2269	-0.112	0.4238	-0.03	0.153	0.307				0.337	-0.18	0.441	0.648188	-0.18035	-0.102997738	-0.077357054
291	A0A1W2PP2	HLA-B	major histocompatibility complex, class I, B	3106	ENSG00000234745	2				-0.9	-0.44	-1.24	-0.38	-0.2	-0.24				-0.5	0.1691	-0.07	-0.592	-0.044	-0.133				-0.33	-0.29	0.107	-0.14	-0.62	0.21	0.263505	-0.3875	-0.371203612	-0.016294697
2323	Q06203	PPAT	phosphoribosyl pyrophosphate amidotransfe	5471	ENSG00000128059	2							0.156	0.258	0.101							0.4997	0.2306	0.0671							0.324	0.21	0.334	0.697579	-0.11756	-0.093944612	-0.023620129
1725	Q9NRH3	TUBG2	tubulin gamma 2	27175	ENSG00000037042	3	0.082	-0.05	-0.09	0.296	0.783	-0.15	0.013	0.372	0.116	-0.007	-0.262	-0.183	0.3572	-0.029	0.1386	0.0845	-0.033	0.264	-0.24	-0.07	-0.57	0.041	0.053	0.232	0.215	0.047	0.311	0.586867	0.150234	0.1148876	0.035346297
3117	Q96DU7	ITPKC	inositol-trisphosphate 3-kinase C	80271	ENSG00000086544	1							-0.32	1.114	0.016							0.6037	-0.482	0.337							0.171	-0.5	-0.02	0.799357	0.39008	0.118536729	0.271543747
1153	O60331	PIP5K1C	phosphatidylinositol-4-phosphate 5-kinase ty	23396	ENSG00000186111	3	-0.14	0.232	-0.12	0.326	0.573	-0.38	-0.11	0.155	-0.01	0.1236	-0.028	0.2311	0.3432	-0.123	0.2595	0.5154	-0.16	0.1114	-0.37	-0.37	-0.35	-0.38	-0.35	0.037	0.268	0.317	-0.08	0.226844	0.201009	-0.08160717	0.282616441
555	F8WAB6	IRAK1	interleukin 1 receptor associated kinase 1	3654	ENSG00000184216	2				0.063	-0.13	-0.53	-0.28	-0.13	-0.15				0.282	-0.277	-0.131	-0.083	-0.088	-0.284				-0.26	-0.29	-0.07	0.05	-0.05	-0.26	0.768006	-0.04686	-0.09699255	0.050130462
2812	Q86V87	FAM160B2	family with sequence similarity 160 member	64760	ENSG00000158863	5				-0.11	0.366	-0.08	0.273	0.29	0.189				-0.039	-0.058	0.5841	0.497	0.4159	0.1871				-0.52	0.09	0.083	0.084	0.068	0.821	0.773101	0.051152	-0.109359453	0.16051189
2607	Q5H9B4	TIMP1	TIMP metallopeptidase inhibitor 1	7076	ENSG00000102265	2							-0.78	-0.15	0.044							-0.144	0.1647	-0.154							-0.43	-0.11	-0.25	0.664666	-0.03372	-0.249884235	0.216166228
924	J3KRN4	MAP3K3	mitogen-activated protein kinase kinase kina	4215	ENSG00000198909	1							0.074	0.561	-0.01							0.6871	-0.046	0.1603							0.674	0.146	-0.48	0.939651	0.095943	-0.058131129	0.154074532
3054	Q92871	PMM1	phosphomannomutase 1	5372	ENSG00000100417	3	0.282	0.697	0.226	-0.15	-0.05	-0.3	-0.13	-0.02	0.054	-0.03	0.1757	0.3059	0.5507	0.0545	0.154	-0.332	0.1093	-0.085	0.077	-0.82	-0.08	-0.68	-0.12	0.338	0.351	-0.08	-0.38	0.365097	0.223985	-0.031981377	0.255965922
3538	Q9NYQ8	FAT2	FAT atypical cadherin 2	2196	ENSG00000086570	8	-0.25	0.751	-0.44	0.591	0.657	-0.51	-0.7	-0.17	-0.36	-0.286	-0.104	0.2512	0.1408	-0.282	-0.363	0.0959	-0.601	-0.259	-0.37	-0.14	-0.04	-0.54	-0.68	-0.32	0.294	-0.23	-0.38	0.634996	0.221193	0.108598876	0.112594022
1834	P32455	GBP1	guanylate binding protein 1	2633	ENSG00000117228	3	0.639	0.335	0.938				-0.19	-0.24	0.046	0.3235	0.1985	0.8413				0.3245	0.1056	0.0972	-0	-0.48	0.91				0.062	-0.02	0.408	0.853698	0.106846	-0.0608725	0.167718718
2816	Q86VS8	HOOK3	hook microtubule tethering protein 3	84376	ENSG00000168172	2							0.084	0.327	0.314							0.7037	-0.216	0.3163							0.424	0.158	0.205	0.994866	-0.02063	-0.026232449	0.005601928
844	H3BQD2	METTL9	methyltransferase like 9	51108	ENSG00000197006	1							-0.18	-0.3	0.317							0.5353	0.069	0.1602							0.45	0.151	-0.03	0.557236	-0.24208	-0.307214183	0.065129604
1300	O95685	PPP1R3D	protein phosphatase 1 regulatory subunit 3D	5509	ENSG00000132825	7				0.019	-0.01	-0.17	-0.16	0.073	0.375				0.1146	-0.051	-0.078	0.335	-0.454	0.0244				0.187	-0.07	0.074	0.31	0.312	0.287	0.398771	-0.16303	0.038622759	-0.201649956
441	E9PFF5	FXR1	FMR1 autosomal homolog 1	8087	ENSG00000114416	3	0.569	0.542	0.331				0.311	0.219	0.327	0.2635	0.4258	0.163				0.2767	0.2717	0.4707	0.1	-0.33	0.237				0.006	0.093	0.385	0.13019	0.300609	0.071389357	0.229219201
1866	P35626	GRK3	G protein-coupled receptor kinase 3	157	ENSG00000100077	6	0.115	0.448	-0.11				-0.4	-0.17	-0.09	0.6706	0.1594	-0.227				-0.053	0.2577	-0.303	-0.38	-0.95	-0.4				0.311	0.107	0.055	0.565779	0.172969	-0.118637721	0.291606237
314	A0A2R8Y4E	AC022414	phosphodiesterase 8B	8622	ENSG00000284762	2				0.411	0.647	-0.2	-0.07	0.119	0.352				0.4166	-0.189	0.0363	-0.021	-0.212	-0.036				-0.15	0.31	0.25	0.402	0.021	0.834	0.432654	-0.06769	0.210622175	-0.278314426
44	A0A087WVI	AS3MT	arsenite methyltransferase	57412	ENSG00000214435	3	0.514	0.355	-0.56	0.023	0.284	0.201	-0.14	0.373	0.278	-0.483	0.0257	0.844	0.2202	0.0049	0.15	0.4708	-0.538	0.2939	-0.48	-0.19	-0.92	-0.44	-0.17	0.512	0.362	0.279	-0.02	0.499718	0.266732	0.037633697	0.229098494
475	E7EUS6	ITM2C	integral membrane protein 2C	81618	ENSG00000135916	2	-0.37	0.405	-0.21	0.362	-0.15	-0.14	-0.69	0.018	0.166	-0.101	-0.429	-0.013	-0.034	0.0397	0.0105	-0.05	0.0801	0.0531	-0.24	0.849	-0.16	-0.18	-0.63	-0.61	-0.1	0.065	-0.17	0.909125	0.063576	-0.017944522	0.081520124
100	A6NC89	CNTNAP3	contactin associated protein like 3	79937	ENSG00000106714	3	0.347	0.269	-0.12				-0.42	0.328	-0.38	0.2758	0.132	0.2688				-0.207	-0.567	-0.271	-0.08	-1.23	-0.19				-0.04	0.434	0.176	0.880271	0.159965	0.066057102	0.093907978
560	D6RA77	PAIP2	poly(A) binding protein interacting protein 2	51247	ENSG00000120727	1							0.225	-0.01	-0.5							0.5559	0.652	0.3804							0.347	0.311	0.866	0.19981	-0.60288	-0.624302388	0.021426459
1250	O76082	SLC22A5	solute carrier family 22 member 5	6584	ENSG00000197375	5	-0.38	-0.05	-0.21	-0.38	-0.18	-0.28	0.025	0.185	-0.05	0.2128	0.1407	-0.232	-0.066	-0.11	-0.024	-0.028	0.0066	0.517	-0.21	-0.22	0.215	-0.11	0.244	0.031	0.292	0.049	-0.12	0.25559	-0.16575	-0.193123522	0.027375667
359	A2A2V4	VEGFA	vascular endothelial growth factor A	7422	ENSG00000112715	3	-0.31	-0.78	-0.39	-0.26	0.062	0.018	0.066	0.063	0.201	-0.004	-0.676	-0.474	-0.469	-0.155	-0.434	0.1192	-0.669	-0.346	-0.12	0.326	0.454	0.251	0.371	0.11	0.997	0.089	-0.61	0.095469	-0.35448	0.197691941	-0.552168209
2765	Q7L576	CYFIP1	cytoplasmic FMR1 interacting protein 1	23191	ENSG00000280618	29	0.139	0.192	0.087	-0.23	0.096	-0.51	0.112	0.259	0.35	0.2021	0.1499	0.2295	0.2537	-0.068	-0.013	0.259	0.1181	-0.016	-0.13	-0.21	-0.19	-0.41	-0.04	0.115	0.259	0.168	0.009	0.399803	0.100087	-0.069348434	0.16943529
201	A0A0C4DH2	IGHV1-3	immunoglobulin heavy variable 1-3	28473	ENSG00000211935	2	-1.64	-1.27	-0.9	-1.57	-1.07	-0.76	-1.64	-0.85	-1.41	-0.724	-1.068	-0.846	-1.435	-1.391	-1.625	-0.553	-1.235	-1.417	-1.06	-0.81	1.917	3.202	-0.83	0.722	-0.3	-0.62	-1.71	0.118172	-1.29251	-0.091514919	-1.200996147
341	A0A2R8YF4	ARHGEF5	Rho guanine nucleotide exchange factor 5	7984	ENSG00000050327	3	0.058	0.192	0.408				-0.06	0.015	0.057	0.1523	-0.2	0.2265				0.3139	0.1695	0.0654	-0.37	-0.66	-0.16				0.517	0.196	0.227	0.67308	0.155361	-0.009271054	0.164632123
2992	Q8TE82	SH3TC1	SH3 domain and tetratricopeptide repeats 1	54436	ENSG00000125089	3	-0.41	0.337	-0.32				-0.37	-0.26	-0.33	0.1118	-0.027	0.2008				-0.399	-0.065	-0.572	-0.31	-0.37	-0.26				0.042	-0.4	-0.21	0.769763	0.026347	-0.099680774	0.126027871
785	G3XAL9	SLC12A2	solute carrier family 12 member 2	6558	ENSG00000064651	4	-0.32	-0.13	-0.09				-0.19	0.006	0.048	0.0236	-0.019	-0.298				0.0717	-0.049	-0.256	-0.16	0.36	-0.08				0.062	0.044	0.026	0.363291	-0.1559	-0.025062633	-0.130836003
1639	P16333	NCK1	NCK adaptor protein 1	4690	ENSG00000158092	4	0.113	0.021	0.057	0.301	0.15	-0.27	-0.2	-0.04	-0.1	0.0777	0.3179	0.3826	0.3691	0.0035	0.3479	-0.014	-0.136	-0.221	-0.25	-0.25	0.057	-0.4	-0.02	0.431	0.083	-0.06	-0.05	0.39935	0.05313	-0.121803052	0.174933461
3745	Q9Y3I1	FBXO7	F-box protein 7	25793	ENSG00000100225	2	0.042	0.06	-0.11				0.281	0.002	0.246	0.1564	-0.01	-0.02				0.3097	-0.135	0.6629	-0.08	-0.22	-0.14				0.262	-0.06	0.189	0.575699	0.093285	-0.074266606	0.167551784
1458	P06756	ITGAV	integrin subunit alpha V	3685	ENSG00000138448	10	-0.21	0.104	0.172	-0.48	-0.19	-0.22	0.138	0.014	-0.03	0.294	0.1345	-0.101	-0.259	0.0177	-0.009	-0.011	0.0783	0.0837	-0.07	-0.56	0.374	-0.13	-0.34	0.246	-0.06	-0.19	-0.15	0.598139	0.020658	-0.102857589	0.123515093
3507	Q9NT22	EMILIN3	elastin microfibril interfacer 3	90187	ENSG00000183798	7	-0.15	-0.44	-0.06	-0.47	0.029	0.934	-0.23	0.28	0.176	0.1696	-0.009	-0.003	0.2222	-0.123	0.2955	0.191	-0.406	0.1505	0.611	0.339	0.304	1.176	0.667	-0.02	-0.2	0.531	-0.22	0.284142	-0.34609	-0.04619599	-0.29989172
3004	Q8WUH2	TGFBRAP	transforming growth factor beta receptor ass	9392	ENSG00000135966	3	-0.12	-0.46	-0.58				-0.25	0.152	0.022	1.0464	0.3453	0.2047				0.0613	0.1801	0.323	1.376	0.844	0.205				0.244	0.143	0.049	0.126285	-0.68436	-0.567541478	-0.116818855
1006	O00401	WASL	WASP like actin nucleation promoting factor	8976	ENSG00000106299	4	0.142	0.301	0.394	0.013	0.247	-0.46	-0.35	-0.07	-0.07	0.0797	0.4522	0.0028	-0.028	-0.286	0.2906	-0.142	-0.14	-0.008	-0.08	0.103	0.008	-0.47	-0.44	-0.07	-0.27	0.081	-0.24	0.397822	0.170058	-0.008414419	0.178471994
2363	Q12797	ASPH	aspartate beta-hydroxylase	444	ENSG00000198363	4	-0.05	-0.53	-0.18	-0.02	-0.35	0.683	-0.07	-0.14	0.326	0.013	0.0981	0.088	-0.265	0.4282	0.144	-0.313	-0.25	0.1407	0.074	0.532	0.161	0.034	-0	-0.43	0.433	0.047	0.273	0.636802	-0.16075	-0.045854062	-0.114895298
527	C9JLV4	APAF1	apoptotic peptidase activating factor 1	317	ENSG00000120868	1	-0.14	-0.07	-0.33				0.161	-0.22	0.322	-0.075	-0.435	-0.015				0.2391	0.3749	0.0996	-0.55	-1.32	-0.14				0.223	0.171	0.272	0.685972	0.176056	-0.078335246	0.254390834
709	J3QLN0	RFNG	RFNG O-fucosylpeptide 3-beta-N-acetylgluc	5986	ENSG00000169733	3	0.02	-0.06	-0.03				-0.25	0.168	-0.35	0.3332	0.1822	0.2985				-0.297	-0.68	-0.256	-0.11	-0.03	0.324				-0.17	-0.18	-0.07	0.973667	-0.04451	-0.014209404	-0.030299562
383	A6NMA8	CRYZL1	crystallin zeta like 1	9946	ENSG00000205758	2				-0.05	0.265	-0.51	0.371	0.415	0.458				0.2184	-0.083	0.3617	0.3155	0.3744	0.2684				-0.54	0.012	0.272	0.155	0.124	0.497	0.783643	0.071961	-0.084523841	0.156484607
253	A0A0U1RQ\	ATP7B	ATPase copper transporting beta	540	ENSG00000123191	1	0.717	0.067	0.237				-0.92	-0.95	-0.58	0.6067	0.9384	1.1876				-0.401	-0.504	-0.751	-0.14	-0.26	-0.25				-0.22	-0.54	-1.54	0.427257	0.255219	-0.41654045	0.67175896
809	H0Y873	AGAP3	ArfGAP with GTPase domain, ankyrin repea	116988	ENSG00000133612	1							-0.06	0.286	0.285							0.0797	-0.334	-0.298							0.698	0.267	-0.02	0.302201	-0.14441	0.354012211	-0.498419302
3480	Q9NRB3	CHST12	carbohydrate sulfotransferase 12	55501	ENSG00000136213	1							-0.65	-0.13	-0.25							-0.086	-0.029	-0.235							0.369	0.016	-0.13	0.29279	-0.4288	-0.227575286	-0.2012244
24	D6RHE0	PDE4D	phosphodiesterase 4D	5144	ENSG00000113448	3	0.35	0.532	0.186	-0.07	0.448	-0.87	0.229	0.137	0.467	0.0344	0.2006	0.0727	0.1678	0.1068	0.1318	0.3611	0.2684	0.244	-0.18	-0.48	-0.14	-0.74	-0.23	0.224	0.112	0.034	-0.07	0.172774	0.320662	-0.019312731	0.339974682
2669	Q641Q2	WASHC2A	WASH complex subunit 2A	387680	ENSG00000099290	21				-0.3	-0.6	0.072	0.114	0.462	0.428				-0.248	-0.272	-0.183	0.3818	0.6789	0.4385				-0.48	-0.1	0.216	-0.04	0.225	-0.19	0.766347	0.092882	-0.103243607	0.196126
342	A0A2R8YF8	VPS33A	VPS33A core subunit of CORVET and HOP	65082	ENSG00000139719	5	-0.61	0.63	-0.72	-0.29	1.287	-0.62	-0.35	-0.86	-0.18	0.1648	-0.081	0.0106	0.1576	-0.337	0.085	0.0228	-0.799	0.156	-0.23	-0.34	-0.66	0.038	0.166	0.28	-0.13	-0.37	-0.25	0.899491	-0.02406	-0.122218715	0.098158635
2882	Q8N0V5	GCNT2	glucosaminyl (N-acetyl) transferase 2 (I bloc	2651	ENSG00000111846	7	0.136	-0.4	-0.37	-0.04	0.282	-0.55	-0.61	-0.41	-0.17	-0.341	-0.439	-0.433	0.1218	0.4529	0.026	0.6769	0.1543	0.4952	-0.36	0.273	-0.28	-0.79	-0.65	-0.78	-0.16	0.145	0.57	0.351573	-0.01244	-0.317478071	0.305035579
1225	O75752	B3GALNT1	beta-1,3-N-acetylgalactosaminyltransferase	8706	ENSG00000169255	3				0.075	0.061	-0.32	0.024	-0.19	0.423				0.1774	-0.084	-0.069	0.3621	-0.257	-0.326				-0.16	-0.26	0.069	0.223	0.512	-0.41	0.973782	0.01915	0.046090262	-0.026940055
3275	Q9BUN1	C1orf56;MI	chromosome 1 open reading frame 56	54964	ENSG00000143443	3				-0.26	-0.16	-0.32	-0.23	-0.19	-0.76				-0.607	0.2072	-0.161	-0.337	-0.667	-0.224				-0.29	-0.21	-0.27	0.135	-0.11	-0.39	0.73811	-0.13074	-0.020962005	-0.109781077

1364	P01717	IGLV3-25	immunoglobulin lambda variable 3-25	28793	ENSG00000211659	2	-2.51	-2.39	-2.06	-2.24	-2.11	-2.25	-2.1	-2.02	-2.34		-1.401	-2.017	-2.604	-1.933	-2.484	-1.388	-2.361	-2.122	-1.53	-1.91	3.765	1.691	-1.2	2.019	-1.37	-1.45	-2.35	0.088179	-1.96443	-0.185045876	-1.779380628
1276	O95274	LYPD3	LY6/PLAUR domain containing 3	27076	ENSG00000124466	1							-0.11	0.364	0.014							0.4304	0.4398	-0.286							-0.41	0.751	-0.64	0.854004	0.190505	-0.104656989	0.29516234
2903	Q8N584	TTC39C	tetratricopeptide repeat domain 39C	125488	ENSG00000168234	4	0.015	0.078	-0.02	-0.06	-0.11	-0.4	-0.12	-0.31	0.155	-0.007	-0.043	0.202	-0.269	-0.111	-0.146	-0.283	-0.088	0.1265	0.067	-0.22	0.16	-0.48	-0.4	-0.09	0.143	-0.22	0.016	0.919831	0.027987	-0.01726921	0.045256133
3090	Q96AQ2	TMEM125	transmembrane protein 125	128218	ENSG00000179178	2				-0.64	-0.03	-0.28	0.068	0.362	0.056				-0.22	0.2234	0.4411	0.3378	-0.195	0.1375				-0.37	0.088	-0.09	0.634	0.258	0.006	0.654849	-0.16575	-0.198082946	0.032337385
1767	P27482	CALML3	calmodulin like 3	810	ENSG00000178363	4	-0.4	0.416	0.325				-0.57	0.551	-0.45	0.0788	-0.101	0.7169				-0.026	-0.325	-0.219	0.053	0.144	0.103				0.233	0.581	-0.11	0.775373	-0.18916	-0.042902359	-0.146253755
3741	Q9Y3E2	BOLA1	bolA family member 1	51027	ENSG00000178096	1	-0.15	0.114	-0.04	0.027	0.251	-0.43	-0.03	-0.2	0.156	0.1409	-0.236	0.351	0.0541	-0.228	0.06	0.1219	0.2979	0.2518	-0.22	-0.85	-0.24	-0.6	-0.55	-0.08	0.024	-0.19	0.278	0.131689	0.235852	-0.124020476	0.359872091
3404	Q9H773	DCTPP1	dCTP pyrophosphatase 1	79077	ENSG00000179958	3	0.31	0.12	0.224	-0.06	0.377	-0.24	-0.31	-0.22	-0.16	0.0441	0.3208	-0.021	-0.099	0.1027	0.4	0.1168	0.0056	-0.47	0.322	-0.22	0.167	-0.59	-0.08	0.126	0.689	0.076	-0.18	0.970873	-0.02934	-0.039740502	0.010397492
2757	Q712K3	UBE2R2	ubiquitin conjugating enzyme E2 R2	54926	ENSG00000107341	3	0.247	0.242	0.121	-0.2	0.009	-0.18	-0.46	-0.31	-0.14	0.2732	0.3263	0.2338	-0.137	0.043	-0.094	-0.25	0.2174	-0.227	0.187	-0.25	0.178	-0.21	0.562	-0.03	-0.17	-0.07	-0.12	0.697115	-0.08361	-0.117771758	0.034158009
1156	O60488	ACSL4	acyl-CoA synthetase long chain family mem	2182	ENSG00000068366	2	0.107	0.538	0.27	0.322	0.236	-0.33	0.124	-0.11	-0.26	0.1838	0.0492	0.1244	0.0569	0.1244	0.3079	-0.429	-0.106	0.2121	-0.1	-0.37	-0.19	-0.18	0.168	0.343	0.219	-0.25	0.259	0.759517	0.110148	0.041034615	0.069113467
70	H0Y3Q0	PCSK6	proprotein convertase subtilisin/kexin type 6	5046	ENSG00000140479	3				-0.53	0.152	-0.17	-0.75	0.06	-0.21				-0.214	-0.325	0.4768	0.3359	-0.241	0.2031				0.145	-0.43	0.123	0.019	0.018	-0.18	0.461144	-0.18845	-0.278370201	0.089920024
1474	P07602	PSAP	prosaposin	5660	ENSG00000197746	40							-0.26	-0.09	0.315							0.2362	-0.03	0.1604							0.15	0.049	0.231	0.711687	-0.15667	-0.135639664	-0.021029778
3143	Q96HL8	SH3YL1	SH3 and SYLF domain containing 1	26751	ENSG00000035115	3	-0.53	0.475	-0.79	0.181	-0.48	-0.04	-0.36	0.127	0.101	0.3571	0.3789	0.4133	0.3219	-0.035	0.1788	0.1638	-0.909	-0.067	-0.05	-0.18	0.067	0.057	0.069	-0.43	-0.37	0.264	0.289	0.559699	-0.11314	-0.235538544	0.122398062
420	E9PJ77	LGALS8	galectin 8	3964	ENSG00000116977	1	0.303	0.334	0.082	0.339	0.68	0.157	0.218	0.297	0.541	-0.04	0.1836	0.1571	0.701	0.253	0.7131	0.2383	0.1135	0.0351	0.071	-0.09	-0.09	0.392	0.79	0.379	0.35	0.268	0.437	0.895794	0.049947	0.066291629	-0.016344905
3525	Q9NWU2	GID8	GID complex subunit 8 homolog	54994	ENSG00000101193	3	0.05	-0.07	-0.12	0.006	-0.13	-0.23	-0.2	-0.04	0.207	-0.026	-0.131	-0.073	-0.457	-0.208	0.0802	0.0344	0.0396	0.3476	-0.28	-0.37	-0.15	-0.39	-0.61	-0.49	0.214	-0.07	0.237	0.397327	0.153767	-0.014669147	0.168436271
3265	Q9BTN0	LRFN3	leucine rich repeat and fibronectin type III dc	79414	ENSG00000126243	1							0.256	0.276	0.151							0.3428	-0.272	0.1322							0.589	0.351	0.372	0.306073	-0.20949	0.160299251	-0.369784926
3665	Q9UMZ2	SYNRG	synergin gamma	11276	ENSG00000275066	2				-0.14	0.053	-0.23	-0.03	0.383	0.359				0.2155	0.4642	-0.026	0.7684	-0.186	-0.061				-0.06	-0.12	0.331	0.278	2.534	-0.2	0.696099	-0.39287	-0.12920126	-0.263664241
2552	Q16513	PKN2	protein kinase N2	5586	ENSG00000065243	5				0.103	0.178	-0.32	-0.25	-0.12	-0.21				0.1328	0.0672	-0.062	-0.153	-0.137	-0.335				-0.12	-0.24	-0.03	0.177	0.123	-0.34	0.973598	-0.03013	-0.020905549	-0.009226648
731	F8VVA0	ATG101	autophagy related 101	60673	ENSG00000123395	1							-0.07	0.149	0.072							0.3731	0.2666	0.0726							0.323	-0.03	0.336	0.525643	-0.15833	-0.185868919	0.027535185
379	I3L3G9	NDE1	nudE neurodevelopment protein 1	54820	ENSG00000275911	1							0.142	-0.01	0.214							0.2788	0.2359	-0.093							0.242	-0.03	0.074	0.958961	0.019146	-0.026680408	0.045826603
2666	Q5XUX1	FBXW9	F-box and WD repeat domain containing 9	84261	ENSG00000132004	3	0.176	0.191	0.258	0.227	0.202	-0.45	-0.1	0.38	0.265	0.0261	0.0195	0.3329	-0.093	-0.118	-0.015	0.0201	-0.09	0.1759	-0.16	-0.78	3E-04	0.05	-0.4	-0.04	0.07	-0.06	0.08	0.207203	0.264808	0.098734683	0.166073314
1145	O43924	PDE6D	phosphodiesterase 6D	5147	ENSG00000156973	1	0.069	0.122	-0.08	0.294	0.18	-0.1	0.301	0.021	0.181	-0.276	0.0318	-0.117	0.2183	0.113	0.2479	0.3311	0.1068	0.336	-0.19	-0.88	-0.4	-0.34	0.023	0.094	0.439	0.141	0.162	0.319496	0.215832	-0.000105716	0.215937667
2830	Q86YS7	C2CD5	C2 calcium dependent domain containing 5	9847	ENSG00000111731	2				0.182	0.052	-0.39	-0.21	-0.05	0.078				0.0664	-0.135	0.0862	0.1676	-0.415	-0.141				-0.21	-0.25	0.057	0.319	0.204	-0.07	0.887964	-0.06408	0.004772725	-0.068851044
655	E9PIT3	F2	coagulation factor II, thrombin	2147	ENSG00000180210	5	-0.81	-0.22	-0.24	-0.41	-0.04	0.246	-0.09	0.483	-0.22	0.0007	0.3647	-0.172	-0.49	0.3728	-0.563	-0.022	-0.098	-0.238	-0.27	-0.38	1.128	0.97	-0.08	0.163	0.831	-0.3	-0.86	0.592253	-0.27798	-0.050960726	-0.227016227
205	P0DP08	AC233755	immunoglobulin heavy variable 4-38-2-like;ir	102723407;	ENSG00000275063	3	-0.89	-1.55	-0.27	-0.89	-0.61	-0.38	-0.56	-0.27	-0.16	-0.852	-1.054	-0.871	-0.589	-0.704	-0.572	-0.079	-0.221	-0.214	-1.22	-0.99	1.802	2.646	-0.24	0.67	0.024	0.013	-0.24	0.163684	-0.89463	-0.047663435	-0.846971061
2126	P59282	TPPP2	tubulin polymerization promoting protein fam	122664	ENSG00000179636	1							0.925	1.925	0.947							-1.048	0.0292	-1.048							-0.37	-0.65	-1.14	0.082385	1.98481	1.954416592	0.030393751
130	A0A096LNV	RNF114	ring finger protein 114	55905	ENSG00000124226	2	-0.39	-0.11	-0.17				0.07	0.314	0.172	0.0909	-0.395	-0.422				0.6431	0.2164	0.2338	-0.09	-0.58	-0.47				0.301	0.243	0.076	0.844838	0.068006	-0.079340356	0.147346678
3267	Q9BTY7	HGH1	HGH1 homolog	51236	ENSG00000235173	3	-0.39	-0.59	-0.22				0.114	0.437	0.389	0.3669	-0.057	0.2934				0.6815	0.0485	0.8485	-0.47	-0.32	0.587				0.151	0.443	0.674	0.435277	-0.22002	-0.406299492	0.18627908
2264	P80511	S100A12	S100 calcium binding protein A12	6283	ENSG00000163221	5	-4.26	-4.3	-3.79	-1.04	-2.18	1.944	-2.85	-2.53	-3.27	-5.742	-1.792	-3.354	-0.92	-1.175	-1.613	-2.331	-2.506	-2.929	-2.72	-3.48	2.516	3.289	-0.59	0.593	-2.56	-2.53	-3.18	0.365095	-1.51157	0.010171771	-1.521743304
1537	P10114	RAP2A	RAP2A, member of RAS oncogene family	5911	ENSG00000125249	5	-0.87	2.073	-0.59				0.12	0.036	0.181	0.0817	-0.924	0.303				0.471	-0.043	0.4126	0.478	-0.56	0.337				0.002	-0	0.305	0.97443	0.065078	0.108597115	-0.043519156
2318	Q05823	RNASEL	ribonuclease L	6041	ENSG00000135828	2							-0.22	0.415	0.426							-0.078	0.3586	0.1938							-0.17	0.108	0.35	0.934432	0.1087	0.047844912	0.060854939
3550	Q9NZL4	HSPBP1	HSPA (Hsp70) binding protein 1	23640	ENSG00000133265	2				0.526	0.368	-0.02	0.143	0.171	0.239				0.1092	0.1618	-0.009	0.2737	0.3556	0.0085				0.177	-0	0.193	0.138	0.384	0.486	0.733618	0.009246	0.088663703	-0.079417905
2813	Q86V88	MDP1	magnesium dependent phosphatase 1	145553	ENSG00000285200	4	-0.13	-0.83	-0.81				-0.3	-0.18	-0.12	0.4588	-0.272	-0.063				0.2614	-0.137	-0.165	-0.19	-0.16	-0.96				-0.01	0.102	-0.25	0.28108	-0.15122	-0.409743879	0.258519962
1422	P04733	MT1F	metallothionein 1F	4494	ENSG00000198417	3	0.569	0.239	-0.18	-0.24	-0.59	-0.01	-0.62	0.465	-0.45	-0.135	-0.191	-0.049	-0.451	0.7393	-0.12	0.1928	-0.445	-0.711	0.194	-0.34	0.093	-0.35	-0.19	-0.2	0.215	-0.32	0.197	0.969614	-0.0127	0.039337407	-0.052039889
3115	Q96DM3	RMC1	regulator of MON1-CCZ1	29919	ENSG00000141452	3							-0.35	-0.27	0.366							0.3323	0.3572	-0.049							0.331	-0.01	-0.15	0.638416	-0.14261	-0.298864599	0.156254092
133	A0A096LP2	AAK1	AP2 associated kinase 1	22848	ENSG00000115977	1							-0.13	0.275	0.398							0.7297	0.1727	0.2166							0.231	0.285	-0.08	0.678633	0.035859	-0.191419616	0.227278809
815	H0YAE2	FCHO2	FCH domain only 2	115548	ENSG00000157107	1	0.152	0.009	-0.14				-0.51	-0.43	-0.33	0.0124	0.0383	0.1098				-0.284	-0.076	-0.58	-0.26	-0.77	-0.39				-0.06	0.098	-0.3	0.74988	0.071478	-0.078205289	0.149682993
2167	P61550	LOC10537	endogenous retrovirus group S71 member 1	105372315;	NA	2	-0.61	-0.49	0.437	-0.57	-0.01	-0.67	-0.03	0.299	0.12	-0.418	0.1283	0.0241	0.3642	0.5807	-0.511	0.9397	0.0936	0.2501	-0.56	-0.04	0.049	-0.25	0.127	0.271	0.71	0.396	-0.23	0.411244	-0.22234	-0.331012549	0.108671778
480	C9IY88	HAAO	3-hydroxyanthranilate 3,4-dioxygenase	23498	ENSG00000162882	3	-0.14	0.125	-0.25	-0.15	0.18	-0.61	-0.46	-0.32	-0.58	-0.074	0.2105	-0.476	-0.078	-0.05	-0.045	-0.229	0.2934	-0.397	-0.23	-0.59	0.145	0.122	-0.35	0.407	-0.09	-0.28	0.16	0.547902	-0.16668	-0.150845984	-0.015831352
1097	O15496	PLA2G10	phospholipase A2 group X	8399	ENSG00000069764	4	-0.37	-0.29	-0.19	0.062	-0.24	0.349	0.051	0.177	-0.12	0.0305	-0.181	-0.044	-0.324	0.3717	-0.082	0.2832	-0.044	0.1478	-0.13	0.052	0.024	0.203	0.004	0.084	0.407	0.435	0.193	0.302222	-0.20503	-0.081225771	-0.123800031
946	K7EIX2	HEATR6	HEAT repeat containing 6	63897	ENSG00000068097	2				-0.19	0.11	0.515	-0.17	0.034	0.162				-0.147	-0.049	0.1114	0.4261	0.0065	0.1213				0.343	0.24	0.342	0.404	0.212	-0.05	0.450279	-0.17205	-0.00098611	-0.171062203
1067	O15066	KIF3B	kinesin family member 3B	9371	ENSG00000101350	3	-0.51	-0.22	-0.44	0.16	0.259	0.121	0.03	0.392	0.189	-0.099	0.0915	-0.101	-0.149	-0.099	0.1811	0.0607	-0.078	0.1492	-0.1	0.037	0.002	-0.38	-0.28	-0.03	0.598	0.056	0.26	0.983543	-0.02109	0.003050876	-0.024145284
1078	O15194	CTDSPL	CTD small phosphatase like	10217	ENSG00000144677	2							0.115	0.078	0.334							0.2374	-0.09	0.4662							-0.05	-0.09	0.177	0.618771	0.163778	-0.028645965	0.192423612
845	H3BM58	VPS9D1	VPS9 domain containing 1	9605	ENSG00000075399	2							-0.01	0.121	-0.17							0.2047	-0.273	-0.177							0.733	0.173	0.209	0.274172	-0.38972	0.063414487	-0.453135351
462	B7Z637	ARMC8	armadillo repeat containing 8	25852	ENSG00000114098	1							0.149	0.154	0.075							0.1204	0.3761	0.5815							0.388	0.189	0.266	0.379706	-0.15494	-0.233356905	0.07841296
2770	Q7L804	RAB11FIP	RAB11 family interacting protein 2	22841	ENSG00000107560	2	0.045	0.483	-0.24	0.085	0.423	-0.34	-0.62	0.785	-0.04	-0.651	0.0967	0.6771	0.4006	-0.13	0.3509	-0.126	0.0866	0.3884	-0.49	-0.43	-0.28	-0.14	-0.33	-0.08	0.113	0.115	0.192	0.442212	0.213087	-0.056247839	0.269334416
3457	Q9NPH2	ISYNA1	inositol-3-phosphate synthase 1	51477	ENSG00000105655	5	-0.15	0.142	0.404	0.166	0.722	-0.14	-0.03	0.006	-0.12	-0.244	0.0836	-0.038	-0.068	-0.194	-0.196	-0.603	0.239	-0.027	-0.37	-0.23	0.131	-0.48	-0.5	-0.04	0.15	-0.09	-0.11	0.202828	0.280297	0.227285663	0.053011413
3089	Q96AE7	TTC17	tetratricopeptide repeat domain 17	55761	ENSG00000052841	7	-0.01	-0.45	-0.23	-0.48	-0.03	-0.07	-0.67	0.103	0.046	0.2669	-0.256	-0.361	-0.354	0.1444	0.0145	-0.251	-0.236	-0.353	-0	0.919	0.051	0.018	0.274	-0.24	0.181	-0.17	-0.09	0.207509	-0.3044	-0.045721497	-0.258681646
3511	Q9NU53	GINM1	glycoprotein integral membrane 1	116254	ENSG00000055211	2	0.166	-0.4	-0.13				-0.53	0.091	-0.17	0.319	-0.296	-0.048				-0.084	-0.069	-0.046	0.114	-0.3	0.19				0.026	0.014	0.201	0.464415	-0.20207	-0.124414255	-0.077659152
2744	Q6ZMI0	PPP1R21	protein phosphatase 1 regulatory subunit 21	129285	ENSG00000162869	3							-0.07	0.12	0.12							0.2756	0.058	0.1225							0.191	0.068	-0.06	0.687479	-0.00872	-0.095501427	0.086786192
83	H0YAZ9	CDK16	cyclin dependent kinase 16	5127	ENSG00000102225	1	-0.14	0.436	0.486	0.019	-0.42	-0.78	0.464	0.521	0.508	0.2279	0.1882	0.0987	-0.418	-0.742	-0.418	0.5772	0.6588	1.1276	0.098	-0.51	-0.32	-0.63	-0.68	-0.4	0.203	0.196	0.609	0.535928	0.281468	-0.023059236	0.304526762
3802	Q9Y6Y8	SEC23IP	SEC23 interacting protein	11196	ENSG00000107651	4				-0.58	-0.49	-0.67	-0.3	-0.23	-0.18				0.2311	0.5407	-0.636	-0.094	0.1013	0.0686				0.502	0.091	-0.85	-0.02	0.044	-0.23	0.266733	-0.3316	-0.445296284	0.113699045
653	E9PHH3	SDC1	syndecan 1	6382	ENSG00000115884	1	-0.37	-0.17	-0.37	-0.06	0.167	0.267	0.036	0.045	-0.35	0.3242	0.2041	-0.009	0.0247	0.4323	0.0524	-0.04	-0.305	-0.535	0.086	-0.02	0.489	0.211	0.55	0.228	0.017	-0.39	-0.13	0.467382	-0.20566	-0.105985877	-0.099670932
616	E7EVH9	PUDP	pseudouridine 5'-phosphatase	8226	ENSG00000130021	3	0.239	0.151	-0.13	-1.14	-0.08	-0.24	-0.31	-0.18	0.05	0.2383	0.1953	0.1638	-0.052	0.1419	0.3225	-0.177	0.2674	0.0397	-0.15	-0.18	-0.09	-0.69	-0.21	-0.08	0.163	0.142	-0.19	0.199996	-0.04066	-0.309461437	0.268803398
2658	Q5VZ89	DENND4C	DENN domain containing 4C	55667	ENSG00000137145	6	0.137	0.458	0.263	0.135	0.1	-0.27	-0.64	-0.16	-0.39	0.2396	0.0869	0.0091	0.122	-0.089	0.2431	0.3346	-0.325	-0.28	-0.58	-0.34	-0.18	-0.47	-0.05	0.093	-0.22	-0.19	-0.15	0.255698	0.190949	-0.07869279	0.269641914
570	D6RDY5	TBCK	TBC1 domain containing kinase	93627	ENSG00000145348	1	0.064	0.029	-0.23				-0.18	0.063	0.297	0.2388	0.4447	0.2966				0.1859	-0.344	-0.042	-0.07	-0.28	0.07				0.242	0.187	0.209	0.757287	-0.05311	-0.123041635	0.06992962
889	H7C2Y5	AC068631	novel protein;DnaJ heat shock protein family	51726	ENSG00000283149	3	0.389	0.471	-0.05				-0.81	-0.13	-0.18	0.5365	-0.37	-0.277				-0.031	-0.334	0.1961	-0.11	1.5	0.806				-0.12	-0.35	-0.24	0.680996	-0.30051	-0.005890899	-0.294614783
2273	P86791	CCZ1;CCZ	CCZ1 homolog, vacuolar protein trafficking a	51622;2219	ENSG00000122674	3							-0.17	-0.04	0.411							0.2421	0.0988	0.3045							-0.04	-0.01	0.326	0.786143	-0.02537	-0.148501076	0.123132877
1314	O95837	GNA14	G protein subunit alpha 14	9630	ENSG00000156049	7							-0.33	0.6	0.174							0.2785	0.1073	-0.067							1.049	0.112	0.455	0.54328	-0.39187	0.040596396	-0.43246609
3787	Q9Y689	ARL5A	ADP ribosylation factor like GTPase 5A	26225	ENSG00000162980	2	0.247	0.464	0.162	-0.23	0.194	-0.37	-0.4	0.103	0.085	0.2495	0.384	0.5773	0.1909	-0.389	-0.153	-0.209	-0.046	0.1781	-0.18	-0.97	-0.24	-0.45	-0.19	-0.1	-0.52	0.135	-0.13	0.146205	0.322513	-0.058575475	0.381088124
1345	P01031	C5	complement C5	727	ENSG00000106804	3	-0.72	-0.37	-0.41	-0.63	-0.25	0.271	-0.45	0.119	-0.26	-0.204	-0.116	0.0604	-0.412	-0.311	-0.649	0.1629	-0.247	-0.153	0.176	0.431	1.195	1.675	0.279	0.558	0.02	0.115	-0.56	0.059408	-0.73277	-0.092572065	-0.640195372
3602	Q9UGM3	DMBT1	deleted in maligt brain tumors 1	1755	ENSG00000187908	5	-0.51	0.357	-0	-0.56	-0.4	-0.12	-1	-0.34	-1.07	-0.133	-0.346	-0.08	-0.005	-0.521	-0.404	-0.563	1.1365	-0.514	-0.32	0.34	0.232	0.514	0.489	-0.42	0.078	-0.36	-0.93	0.452883	-0.36339	-0.24625686	-0.117133467
3578	Q9UBF2	COPG2	coatomer protein complex subunit gamma 2	26958	ENSG00000158623	5							-0.18	-0.06	0.218							-0.064	0.1173	0.1823							0.278	0.006	0.004	0.816663	-0.10169	-0.08440182	-0.017284653
1843	P34820	BMP8B	bone morphogenetic protein 8b	656	ENSG00000116985	4	-0.2	0.587	-0.58	-0.34	-0.28	-0.11	-0.44	-0.11	-0.16	-0.079	-0.34	0.3063	-0.296	-0.193	-0.512	0.3573	0.0884	0.448	-0.18	0.261	-0.36	-0.75	-0.81	-0.77	0.27	0.265	0.138	0.66623	0.032884	-0.157434668	0.190318994
415	B1AMG5	PCSK5	proprotein convertase subtilisin/kexin type 5	5125	ENSG00000099139	3	0.235	0.142	0.193	0.682	0.235	0.242	-0.44	0.235	0.097	-0.545	0.2326	0.8023	0.0719	-0.33	0.2375	0.0976	-0.002	-0.131	-0.07	0.329	-0.12	-0.63	-0.37	-0.6	0.02	0.249	-0.51	0.228161	0.368478	0.131558497	0.236919386
92	A0A087X0N	LEPROT	leptin receptor overlapping transcript	54741	ENSG00000213625	1				0.063	-0.15	-0.17	-0.32	-0.24	0.029				0.3487	-0.079	0.3233	-0.064	0.3316	0.0023				-0.65	-0.06	0.351	-0.43	-0.2	0.169	0.287298	0.005286	-0.275767062	0.281052956
1669	P19086	GNAZ	G protein subunit alpha z	2781	ENSG00000128266	4	0.15	-1.49	-0.18	0.021	-0.48	0.249	-0.47	-0.26	-0.13	0.0376	0.7447	0.2104	-0.77	0.0214	0.3559	-0.202	-0.119	0.0276	-0.54	0.334	0.198	0.108	-0.25	-0.38	-0.06	-0.02	0.339	0.405904	-0.25754	-0.322510479	0.064972956
2588	Q4ZG55	GREB1	growth regulating estrogen receptor binding	9687	ENSG00000196208	1							0.016	0.223	0.008							0.3507	0.3229	0.1888							0.313	0.152	-0.2	0.484506	-0.00533	-0.204995488	0.199666555
3764	Q9Y5E6	PCDHB3	protocadherin beta 3	56132	ENSG00000113205	2							-0.04	0.032	-0.16							0.3993	-0.502	0.6674							-0.25	-0.29	-0.22	0.528887	0.199139	-0.243159062	0.44229795
1258	O94822	LTN1	listerin E3 ubiquitin protein ligase 1	26046	ENSG00000198862	3				0.234	0.586	-0.07	-0.1	1.6	0.273				-0.031	-0.225	-0.442	0.2074	0.2181	0.2679				-0.12	-0.11	0.238	0.213	-0.07	0.321	0.362868	0.343284	0.42129314	-0.078009534
794	G8JLD3	ERC1	ELKS/RAB6-interacting/CAST family memb	23085	ENSG00000082805	3				0.059	0.329	-0.26	-0.21	-0.06	0.428				0.4877	0.2014	0.2427	-0.292	0.1709	0.3432				0.63	0.257	0.892	0.736	0.253	-0.1	0.252945	-0.39818	-0.145283646	-0.252896243
1294	O95497	VNN1	vanin 1	8876	ENSG00000112299	2				0.395	-0.02	-0.27	-0.9	-0.96	0.03				-0.022	0.3978	-0.326	0.4249	0.3894	0.8194				-0.94	-0.76	0.379	-0.04	1.396	-0.62	0.476936	-0.19078	-0.568567611	0.377791056
2805	Q86TV6	TTC7B	tetratricopeptide repeat domain 7B	145567	ENSG00000165914	4	0.647	0.726	0.168	-0.1	0.026	0.181	-0.83	-0.41	-0.34	0.2002	0.7941	0.5903	-0.714	-0.4	0.6722	0.0807	-0.367	-0.233	0.199	0.216	-0.11	-0.32	-0.36	-0.82	-0.11	0.013	-0.09	0.692135	0.162331	-0.061166934	0.223497995
395	A8MTY9	VPS26C	VPS26 endosomal protein sorting factor C	10311	ENSG00000157538	3	-0.26	-0.34	-0.15				-0.18	-0.28	-0.21	-0.141	-0.215	-0.201				-0.857	-0.318	-0.358	-0.19	-0.22	-0.02				-0.43	-0.26	-0.37	0.635169	0.010963	0.111298747	-0.100335894
1731	P23443	RPS6KB1	ribosomal protein S6 kinase B1	6198	ENSG00000108443	3	0.609	-0.04	0.214				0.016	0.378	0.213	-0.207	0.2761	0.3644				0.416	0.5796	0.3291	0.043	-0.61	-0.14				0.23	0.069	0.174	0.257961	0.269119	-0.062148834	0.331267387
2781	Q7Z3T8	ZFYVE16	zinc finger FYVE-type containing 16	9765	ENSG00000039319	1							-0.29	-0.27	0.178							-0.024	0.0959	0.225							-0.2	0.067	-0	0.532427	-0.08363	-0.226024556	0.142396745
2602	Q5FVE4	ACSBG2	acyl-CoA synthetase bubblegum family mem	81616	ENSG00000130377	4				0.33	1.669	-0.15	-0.43	0.59	0.198				-0.347	-0.363	-0.124	-0.165	-0.184	-0.008				-0.2	-0.54	-0.2	-0.05	-0.05	-0.46	0.183513	0.618855	0.56516792	0.053687252
234	A0A0G2JPR	C4A	complement C4A (Rodgers blood group)	720;110384	ENSG00000244731	51	-0.18	-0.29	0.277				0.499	0.669	0.761	-0.083	0.5233	0.5786				0.7566	0.045	0.0308	0.004	0.371	1.358				0.757	0.609	0.471	0.535101	-0.30668	-0.020482088	-0.286195237
897	H9KV81	TTC5	tetratricopeptide repeat domain 5	91875	ENSG00000136319	1							0.173	0.31	0.076							0.4035	0.2936	0.3215							0.123	0.067	0.372	0.43391	-0.00068	-0.153034215	0.152353001
1010	O00443	PIK3C2A	phosphatidylinositol-4-phosphate 3-kinase c	5286	ENSG00000011405	4	-0.06	-0.16	-0.08	0.154	0.021	-0.49	-0.06	0.184	0.079	-0.078	-0.265	-0.337	0.0275	0.0108	-0.043	0.041	0.0665	0.2094	-0.37	-0.78	-0.28	0.08	-0.13	0.129	0.328	-0.01	0.049	0.865297	0.065339	-0.004253866	0.069592543

692	F2Z2X4	XPO4	exportin 4	64328	ENSG00000132953	3	0.112	0.009	-0.23				-0.37	0.047	-0.04	0.2648	-0.254	0.1684				-0.252	-0.203	-0.089	-0.21	-0.65	-0.25				-0.19	-0.07	0.245	0.727939	0.10845	-0.017206691	0.125656279
717	H0YAM1	GRSF1	G-rich RNA sequence binding factor 1	2926	ENSG00000132463	1							-0.15	-0.19	-0.33							-0.282	-0.031	-0.18							0.209	-0.38	-0.81	0.880793	0.101822	-0.058265366	0.160087503
3347	Q9H0J9	PARP12	poly(ADP-ribose) polymerase family membe	64761	ENSG00000059378	3	0.194	0.001	-0.03	0.11	0.279	-0.48	0.29	0.491	0.422	0.0444	0.3892	0.2689	0.1655	0.055	-0.21	0.9886	0.4833	0.6783	-0.4	-0.38	-0.04	-0.44	-0.03	0.437	0.36	0.367	0.451	0.397455	0.105235	-0.17704324	0.282278586
86	A0A087X06:	ZNF2	zinc finger protein 2	7549	ENSG00000275111	1	-0.65	1.156	-0.43	-0.66	0.721	-0.07	-0.43	0.767	-0.16	-0.146	-0.694	0.1919	-0.365	-0.039	-0.37	0.0826	-0.615	0.1649	-0.15	0.107	0.592	0.392	-0.11	-0.32	-0.06	0.066	0.49	0.521779	-0.08373	0.226463088	-0.310192194
1468	P07332	FES	FES proto-oncogene, tyrosine kinase	2242	ENSG00000182511	2				-0.17	0.293	-0.47	0.105	0.136	0.027				0.1303	-0.081	-0.367	-0.06	0.0358	-0.237				-0.57	-0.44	0.064	-0.07	0.107	0.305	0.884331	0.085449	0.08300049	0.002448232
813	H0YA20	DGKQ	diacylglycerol kinase theta	1609	ENSG00000145214	1							-0.07	0.115	-0.03							0.1404	0.2078	0.1382							-0.18	-0.21	0.14	0.283736	0.087998	-0.157940106	0.245938535
2724	Q6UWN0	LYPD4	LY6/PLAUR domain containing 4	147719	ENSG00000273111	3	0.388	0.045	0.29	0.136	0.592	-0.11	0.231	0.77	0.462	0.0448	-0.025	-0.117	0.0897	-0.129	-0.077	0.0687	0.064	0.0752	-0.34	-1.16	-0.57	0.081	-0.14	0.033	0.34	-0.13	0.099	0.081627	0.510654	0.312177794	0.198476038
981	M0R300	MYO9B	myosin IXB	4650	ENSG00000099331	2	0.209	0.016	0.189				-0.64	-0.22	-0.08	-0.042	-0.092	0.525				-0.394	-0.09	-0.022	-0.42	-0.74	-0.17				-0.01	-0.24	-0.18	0.417977	0.209805	-0.066685374	0.276490505
833	H0YLF3	B2M	beta-2-microglobulin	567	ENSG00000166710	8							-0.82	-0.62	-0.27							-0.173	0.1736	-0.007							-0.28	-0.08	-0.35	0.158822	-0.33453	-0.569229043	0.234698051
1399	P02792	FTL	ferritin light chain	2512	ENSG00000087086	2	-0.28	0.734	-0.04				-0.24	0.098	-0.18	0.3836	-0.178	-0.096				-0.25	-0.352	-0.391	0.152	3.498	0.077				-0	0.182	-0.19	0.453366	-0.60518	0.161170945	-0.766354173
962	K7EM87	EPG5	ectopic P-granules autophagy protein 5 hor	57724	ENSG00000152223	1							-0.55	0.211	-0.28							0.4653	0.1772	0.3606							-0.09	0.047	0.04	0.221446	-0.20484	-0.538637126	0.33379652
2932	Q8ND76	CCNY	cyclin Y	219771	ENSG00000108100	4	0.152	0.286	-0.01	-0.07	0.069	-0.16	0.019	0.182	-0.24	0.2371	-0.068	0.3688	0.086	-0.408	0.2173	0.2402	-0.045	0.3784	-0.05	0.188	0.333	0.406	-0.07	0.103	0.382	0.095	0.129	0.492226	-0.14271	-0.087056552	-0.05565051
1809	P30453	HLA-A	major histocompatibility complex, class I, A	3105	ENSG00000206505	9	-0.3	0.494	-0.1				-1.58	0.174	-0.47	0.2619	0.2935	0.3187				-1.02	-0.051	-0.201	0.849	-0.74	0.513				-0.16	-0.64	-0.61	0.879045	-0.16481	-0.2303713	0.065561341
442	B4DY09	ILF2	interleukin enhancer binding factor 2	3608	ENSG00000143621	2	-0.11	-0.02	-0.15				-0.18	-0.22	-0.24	-0.288	-0.083	0.008				-0.513	-0.436	-0.175	-0.33	-0.36	0.002				0.304	-0.19	-0.84	0.861949	0.082025	0.093395134	-0.011370166
2610	Q5JR04	MOV10	Mov10 RISC complex RNA helicase	4343	ENSG00000155363	2							0.082	0.03	0.168							0.2866	0.1328	0.1734							0.195	0.11	0.148	0.389218	-0.05775	-0.104356322	0.046608508
3374	Q9H2U1	DHX36	DEAH-box helicase 36	170506	ENSG00000174953	4				-0.14	0.133	-0.44	-0.78	-0.29	0.383				0.0746	-0.362	-0.104	-0.299	-0.09	0.0288				-0.16	-0.2	-0.07	-0.21	-0.17	-0.01	0.93454	-0.05293	-0.064597883	0.011663618
368	A5YKK6	CNOT1	CCR4-NOT transcription complex subunit 1	23019	ENSG00000125107	4	0.288	0.238	0.205	0.281	0.116	0.024	-0.06	0.081	-0.01	0.2514	0.1853	0.2426	-0.07	-0.11	-0.085	-0.111	-0.005	0.1746	0.016	-0.49	-0.02	0.13	0.057	-0.04	-0.14	-0	0.254	0.316073	0.156147	0.07682047	0.079326942
16	A0A0C4DH6	IGKV1-8	immunoglobulin kappa variable 1-8	28942	ENSG00000240671	1				-0.97	0.054	-0.75	-0.66	0.014	-0.65				-0.004	-1.046	-0.589	-0.269	-0.433	-0.349				1.772	0.591	0.467	-0.29	0.259	0.061	0.092485	-0.9701	-0.044878383	-0.92522373
762	F8WB95	ANKMY2	ankyrin repeat and MYND domain containing	57037	ENSG00000106524	1				-0.25	-0.26	-0.3	-0.39	-0.17	0.187				-0.409	-0.046	0.2222	0.1765	0.0108	-0.039				-0.66	-0.32	-0.1	-0.02	0.017	0.251	0.595397	-0.05789	-0.182373857	0.124483115
2806	Q86U28	ISCA2	iron-sulfur cluster assembly 2	122961	ENSG00000165898	2							0.535	0.298	0.778							0.6931	0.4359	0.5808							0.512	0.595	0.338	0.885686	0.055344	-0.032839954	0.088183999
473	B8ZZF8	LSM5	LSM5 homolog, U6 small nuclear RNA and	23658	ENSG00000106355	2				0.116	0.374	-0.29	1.078	0.888	0.727				0.1248	-0.042	0.2181	0.8913	-0.2	0.6105				-0.22	-0.13	0.15	0.621	0.453	0.683	0.732675	0.222217	0.214114441	0.008102415
2346	Q08AG7	MZT1	mitotic spindle organizing protein 1	440145	ENSG00000204899	1	0.2	0.505	0.245				-0.12	-0.08	0.234	0.3233	-0.043	0.1985				0.2061	0.0256	-0.383	0.2	-0.46	0.06				0.124	0.17	-0.05	0.654661	0.157263	0.109760672	0.047502593
244	C9IZ13	ARL6	ADP ribosylation factor like GTPase 6	84100	ENSG00000113966	1							0.265	0.818	0.308							0.5695	0.3733	0.5417							0.631	0.286	0.17	0.849609	0.101157	-0.031345884	0.132502442
1019	O00522	KRIT1	KRIT1 ankyrin repeat containing;KRIT1, anl	889	ENSG00000285953	2				0.078	-0.44	0.119	-0.46	0.089	0.3				-0.313	0.1646	0.3996	0.1398	-0.113	0.3721				-0.34	-0.71	-0.22	0.04	-0.04	0.123	0.418086	0.1376	-0.16059835	0.298198523
1571	P12110	COL6A2	collagen type VI alpha 2 chain	1292	ENSG00000142173	14				-0.22	0.327	-0.18	-0.47	0.596	0.511				0.1025	-0.33	-0.378	-0.111	-0.328	-0.328				1.108	0.621	0.221	0.11	-0	0.164	0.161327	-0.27662	0.322293287	-0.598910436
524	C9JK14	USP40	ubiquitin specific peptidase 40	55230	ENSG00000085982	1							-0.02	0.399	0.501							0.2134	0.428	0.532							-0.02	0.068	0.385	0.583521	0.148735	-0.098045269	0.246780035
2800	Q86SJ2	AMIGO2	adhesion molecule with Ig like domain 2	347902	ENSG00000139211	3	0.322	0.699	-0.12				-0.14	0.008	0.07	0.4552	0.3438	0.6428				-0.101	-0.166	0.0022	0.162	-0.08	-0.05				-0.14	-0.15	0.428	0.727082	0.112117	-0.055807422	0.167924199
659	E9PMS5	KCTD21	potassium channel tetramerization domain c	283219	ENSG00000188997	2	-0.02	-0.07	-0.01				-2.17	-1.36	-1.84	0.1564	0.0472	0.3				-1.566	-1.026	-1.261	-0.51	-0.56	-0.38				-0.94	-1.26	-1.88	0.781143	0.009558	-0.353158282	0.362716454
1275	O95248	SBF1	SET binding factor 1	6305	ENSG00000100241	4	-0.05	0.293	-0.17	-0.1	0.268	-0.32	0.189	-0.36	0.535	0.3087	0.1535	0.1114	-0.011	0.0067	-0.145	0.104	0.292	0.348	-0.49	-0.52	-0.25	-0.11	-0.41	-0.13	-0.38	0.122	0.683	0.269642	0.19456	-0.099162128	0.293722121
3256	Q9BRX8	PRXL2A	peroxiredoxin like 2A	84293	ENSG00000122378	1							-0.11	0.754	0.098							0.0356	-0.381	-0.18							0.103	0.201	-0.11	0.454278	0.182016	0.422645352	-0.240629413
893	H7C5R8	PARP14	poly(ADP-ribose) polymerase family membe	54625	ENSG00000173193	2	-0.15	-0.11	-0.45	-0.11	-0.07	-0.3	0.08	0.167	0.037	-0.158	-0.558	-0.076	0.0808	-0.078	0.0645	0.7412	-0.125	0.2721	-0.35	-2.28	-0.53	-0.3	0.146	0.409	0.19	0.243	0.337	0.717452	0.138408	-0.117917168	0.256325151
3103	Q96C10	DHX58	DExH-box helicase 58	79132	ENSG00000108771	2	-0.17	0.306	0.305				-0.25	0.765	0.095	0.063	-0.053	-0.51				0.0851	-0.106	-0.303	0.418	-0.53	0.209				0.418	-0.16	0.066	0.435585	0.104916	0.312494146	-0.207578098
759	H0Y5T1	CLASP1	cytoplasmic linker associated protein 1	23332	ENSG00000074054	7				-0.38	-0.83	-0.3	-0.06	0.172	0.178				-0.465	-0.793	0.6892	0.0649	0.1996	-0.054				-0.88	-0.4	-0.5	-0.32	0.136	0.165	0.748691	0.097148	-0.142930552	0.240078109
1262	O94885	SASH1	SAM and SH3 domain containing 1	23328	ENSG00000111961	2	-0.1	0.256	-0.09				-0.02	0.413	0.664	0.3289	0.1057	0.3767				0.4104	0.4354	0.3334	0.126	0.062	0.284				0.164	0.755	0.322	0.697346	-0.09791	-0.144094103	0.046184684
821	H0YC35	CLU	clusterin	1191	ENSG00000120885	27	-0.36	0.241	-0.14				-0.04	-0.15	-0.12	0.5655	-0.295	0.4605				0.2624	0.4591	0.2971	-0.08	-0.27	-0.09				0.6	0.367	-0.13	0.235184	-0.16119	-0.386057356	0.224865908
456	E7EW69	SEPT10;S	septin 10	151011	ENSG00000186522	3							-0.21	-0.32	-0.07							-0.291	-0.11	0.2839							-0.2	-0.22	0.108	0.777138	-0.09049	-0.157223706	0.066733092
3231	Q99941	ATF6B	activating transcription factor 6 beta	1388	ENSG00000213676	1							0.175	-0.04	0.091							0.376	-0.042	-0.182							0.136	0.539	-0.15	0.891434	-0.09918	0.026289797	-0.125474093
3344	Q9H0A8	COMMD4	COMM domain containing 4	54939	ENSG00000140365	5	0.125	-0.03	-0.03	-0.01	-0.47	-0.76	-0.04	0.363	0.487	0.1206	0.1924	0.2628	-0.183	0.4412	-0.08	0.5122	0.6101	0.7209	-0.02	-0.7	-0.09	-0.12	-0.03	0.124	0.453	0.404	0.404	0.294624	-0.08717	-0.328840176	0.241672825
270	C9JVA8	SERPINB8	serpin family B member 8	5271	ENSG00000166401	2							-0.38	-0.53	-0.07							0.1847	0.0067	-0.194							-0.04	-0.21	-0.3	0.340843	-0.14373	-0.32789394	0.184165748
2713	Q6PHR2	ULK3	unc-51 like kinase 3	25989	ENSG00000140474	2	0.104	0.487	-0.02				-0.36	0.126	-0.09	0.018	0.1592	0.5099				0.2876	0.2504	0.5997	-0.1	-0.5	-0.27				0.211	-0.1	0.353	0.219408	0.107235	-0.263390428	0.370625668
2796	Q7Z7E8	UBE2Q1	ubiquitin conjugating enzyme E2 Q1	55585	ENSG00000160714	1	-0.04	0.364	0.15				-0.41	-0.24	0.141	0.1602	0.1912	0.2164				-0.071	0.1586	-0.344	-0.46	-0.58	-0.33				-0.27	-0.06	-0.29	0.131829	0.326771	-0.057039671	0.383810516
3222	Q99698	LYST	lysosomal trafficking regulator	1130	ENSG00000143669	2	0.145	0.139	0.18				-0.44	-0.21	0.125	0.0322	0.0592	-0.061				0.1505	-0.242	-0.452	0.27	-0.32	-0.23				0.605	0.207	0.236	0.566767	-0.13739	0.076911653	-0.214304815
1171	O60678	PRMT3	protein arginine methyltransferase 3	10196	ENSG00000185238	2							0.006	0.643	0.12							0.4565	-0.348	-0.01							0.173	-0.07	0.124	0.769108	0.181281	0.223300207	-0.04201873
3335	Q9GZQ3	COMMD5	COMM domain containing 5	28991	ENSG00000170619	3	0.388	0.152	0.194	-0.05	-0.07	-0.72	0.139	0.586	0.523	0.1626	0.2146	0.0626	-0.226	-0.145	-0.018	0.7951	0.7509	0.3993	0.107	-0.77	0.264	-0.6	-0.42	-0.02	0.981	0.633	0.599	0.880056	0.041057	-0.094695907	0.135752471
2919	Q8NB46	ANKRD52	ankyrin repeat domain 52	283373	ENSG00000139645	1							-0.09	0.548	0.231							0.4968	0.2413	0.3725							0.318	0.117	0.043	0.638449	0.070915	-0.139911907	0.210827169
471	B8ZZA2	FAM126A	family with sequence similarity 126 member	84668	ENSG00000122591	2	0.162	0.722	-0.5				-0.96	-0.33	-0.12	-0.235	-0.138	0.4253				0.3374	-0.323	0.2394	-0.14	-0.39	-0.51				-0.17	-0.25	0.132	0.615795	0.049235	-0.22111326	0.270347876
97	A0A087X0T	CADM1	cell adhesion molecule 1	23705	ENSG00000182985	1							-0.13	0.883	0.277							-0.02	0.2331	-0.118							-0.41	-0.05	-0.49	0.285145	0.660763	0.313092973	0.347669533
1982	P48960	ADGRE5;(	adhesion G protein-coupled receptor E5	976	ENSG00000123146	3	0.316	0.423	-0.24	0.277	0.595	-0.25	0.601	0.743	0.608	0.4335	0.419	0.5257	0.2689	0.159	0.3037	0.547	0.318	0.8464	0.258	0.088	0.029	0.317	0.214	0.417	0.627	0.844	0.099	0.807913	0.020145	-0.082941019	0.103086217
3763	Q9Y5E2	PCDHB7	protocadherin beta 7	56129	ENSG00000113212	2							-0.02	-0.05	-0.33							0.0922	-0.015	-0.034							0.375	-0.67	-0.42	0.764267	0.107351	-0.146507295	0.253857835
823	H0YCR7	RNH1	ribonuclease/angiogenin inhibitor 1	6050	ENSG00000023191	17							0.258	0.312	0.079							-0.445	-0.328	-0.253							0.302	0.574	-0.1	0.132578	-0.04339	0.558536296	-0.601926791
629	E9PBG3	TRIM36	tripartite motif containing 36	55521	ENSG00000152503	1	0.058	-0.39	0.234				0.311	0.086	0.386	-0.1	0.1866	0.6255				0.2166	0.0268	0.5746	-0.15	-0.26	0.242				-0.01	0.394	-0.15	0.485845	0.103374	-0.14064085	0.244014925
8	A0A0G2JH7	PPT2	palmitoyl-protein thioesterase 2	9374	ENSG00000227600	2	-0.24	-0.11	0.003				-0.2	-0.2	-0.03	0.3446	0.3326	0.2446				0.1644	0.4364	0.3496	-0.39	0.734	0.252				0.345	0.432	0.634	0.088703	-0.4639	-0.441608759	-0.022293734
838	H0YN26	ANP32A	acidic nuclear phosphoprotein 32 family mer	8125	ENSG00000140350	2	-0.2	-0.02	-0.32				-0.85	-0.28	-0.26	-0.301	0.1008	-0.206				0.1962	-0.204	-0.372	-0.32	-0.43	0.832				-0.09	-0.2	-0.21	0.574293	-0.24867	-0.188919245	-0.059752411
587	E5RHT6	ARFGAP1	ADP ribosylation factor GTPase activating p	55738	ENSG00000101199	3	0.241	0.281	-0.01	-0.02	-0.06	-0.34	0.319	0.036	0.459	0.009	-0.002	0.1874	-0.05	-0.221	0.1142	0.3115	0.6166	-0.163	-0.15	-1.1	-0.12	-0.53	-0.19	-0.06	0.71	0.087	0.304	0.517682	0.217097	0.012038444	0.205058112
702	F5GZG1	RAP1B	RAP1B, member of RAS oncogene family	5908	ENSG00000127314	13							-0.3	-0.01	-0.21							0.2828	-0.007	-0.148							-0.27	0.241	0.228	0.561586	-0.24055	-0.216141923	-0.024410036
2169	P61599	NAA20	N(alpha)-acetyltransferase 20, NatB catalyti	51126	ENSG00000173418	2				-0.03	0.161	0.096	0.297	0.271	0.078				0.0943	0.0974	0.1144	0.2134	0.5931	0.1309				0.008	-0.2	-0.08	0.235	0.203	0.356	0.652766	0.058965	-0.061034295	0.119999278
2786	Q7Z4G1	COMMD6	COMM domain containing 6	170622	ENSG00000188243	2	0.068	0.019	0.045	-0.26	-0.24	-0.89	-0.71	-0.57	-0.22	0.0467	0.1343	0.0303	-0.181	-0.222	0.524	-0.236	-0.319	-0.09	-0.22	-0.85	-0.07	-0.49	-0.57	-0.06	0.187	-0.14	-0.55	0.269428	0.003308	-0.270750544	0.27405807
938	J9JIG6	ARSG	arylsulfatase G	22901	ENSG00000141337	1							-0.16	-0.24	0.6							0.3376	-0.179	0.1468							0.339	0.804	-0.27	0.870664	-0.22263	-0.03423582	-0.188396831
241	A0A0J9YX5	TUBGCP3	tubulin gamma complex associated protein 3	10426	ENSG00000126216	1							0.443	0.326	0.31							0.2836	0.1091	0.2534							0.358	0.099	0.173	0.365345	0.149295	0.144142715	0.005151836
2339	Q08211	DHX9	DExH-box helicase 9	1660	ENSG00000135829	1	0.4	0.374	0.382				0.381	0.213	0.558	0.3831	0.3501	0.23				0.1972	0.1948	-0.196	-0.2	0.214	0.573				0.036	0.494	-0.23	0.378687	0.235695	0.19142917	0.044265635
2653	Q5VSL9	STRIP1	striatin interacting protein 1	85369	ENSG00000143093	1							0.41	0.525	0.487							0.3026	0.0788	0.6593							0.736	0.625	0.768	0.25558	-0.23519	0.127247877	-0.362436272
3599	Q9UGC6	RGS17	regulator of G protein signaling 17	26575	ENSG00000091844	1							0.681	0.602	0.652							0.587	0.4931	0.216							0.572	0.637	1.17	0.36724	-0.14794	0.212883125	-0.360819687
1058	O14933	UBE2L6	ubiquitin conjugating enzyme E2 L6	9246	ENSG00000156587	4	0.044	0.536	0.085	0.181	0.048	-0.34	-1.02	-0.64	-1.17	0.0395	0.2124	0.1246	0.3027	0.252	0.0424	-0.782	-0.473	-0.408	0.128	-0.44	0.498	-0.16	-0.16	0.315	-0.53	-0.27	-0.47	0.783886	-0.13374	-0.177666184	0.043931142
2580	Q3SYG4	BBS9	Bardet-Biedl syndrome 9	27241	ENSG00000122507	3							-0.15	-0.11	-0.32							0.1151	-0.151	0.0041							-0.03	-0.07	-0.24	0.422938	-0.07978	-0.180352038	0.100570628
2842	Q8IVD9	NUDCD3	NudC domain containing 3	23386	ENSG00000015676	5	-0.21	-0.1	-0.32	-0.32	-0.07	-0.62	0.091	-0.29	0.009	-0.007	-0.312	-0.163	0.155	0.0958	0.0523	-0.204	0.2235	-0.141	-0.15	-1.15	-0.42	-0.28	-0.48	-0.39	-0.46	-0.28	0.694	0.324549	0.120831	-0.170493588	0.291325068
2105	P55058	PLTP	phospholipid transfer protein	5360	ENSG00000100979	2	-0.81	-0.56	-1.44	-0.07	-0.16	-0.6	-0.49	-0.39	0.139	-0.241	0.3523	-0.805	-0.273	-0.184	-0.399	1.2057	-0.409	0.169	-0.64	0.032	-0.31	-0.01	-0.44	-0.16	-0	0.61	-0.75	0.347379	-0.30101	-0.421093355	0.12008508
2728	Q6UWX4	HHIPL2	HHIP like 2	79802	ENSG00000143512	2				-0.49	0.319	-0.68	-0.56	0.372	0.19				0.1861	0.5215	-0.642	0.0012	-0.052	0.07				-0.95	-0.41	0.616	-0.27	0.23	-0.2	0.853215	0.022414	-0.155183877	0.177597454
2372	Q13043	STK4	serine/threonine kinase 4	6789	ENSG00000101109	5	-0.02	0.019	-0.1				0.035	0.338	0.491	-0.089	-0.026	-0.116				0.2528	-0.434	-0.246	-0.4	-0.7	0.087				0.345	0.333	0.102	0.562257	0.165588	0.236398728	-0.070811089
2383	Q13158	FADD	Fas associated via death domain	8772	ENSG00000168040	1	-1.11	-0.47	-1.41				0.117	-0.37	-0.24	0.2847	-1.173	0.6427				0.1937	0.0461	0.0353	-0.93	-0.96	-0.86				0.278	0.008	0.344	0.431744	-0.2263	-0.585984328	0.359679396
1391	P02751	FN1	fibronectin 1	2335	ENSG00000115414	161	0.441	0.66	0.937	0.071	0.071	0.394	-0.18	-1.33	-0.12	0.4541	0.6331	0.5927	-0.008	-0.05	0.1741	-0.497	-0.267	-0.674	1.308	0.475	0.44	0.059	-0.03	0.042	0.039	-0.22	-0.41	0.898446	-0.08391	0.065365022	-0.149276386
3713	Q9Y2A9	B3GNT3	UDP-GlcNAc:betaGal beta-1,3-N-acetylgluc	10331	ENSG00000179913	3	-1.68	-1.56	-0.86	-0.58	-0.39	-0.24	-0.51	-0.68	-0.91	-0.182	0.7517	-0.34	0.3713	0.4649	-0.059	-0.476	-0.259	-0.957	-1.07	0.377	-0.22	0.439	0.092	-0.09	-0.16	-0.45	-0.81	0.102249	-0.61329	-0.747373246	0.134084078
1237	O75896	TUSC2	tumor suppressor 2, mitochondrial calcium r	11334	ENSG00000114383	2	-0.06	0.647	-0.15	-0.69	0.107	-0.87	0.451	0.82	0.611	0.335	-0.09	0.1564	-0.808	-0.144	-0.382	0.578	0.6055	0.803	-0.06	-0.35	-0.35	-0.78	-0.51	-0.35	0.319	0.046	0.724	0.637654	0.243251	-0.020860446	0.264111715
936	J3QTA2	BAG1	BCL2 associated athanogene 1	573	ENSG00000107262	1	-0.29	-0.2	-0.19				0.116	-0.15	0.597	-0.285	-0.219	-0.193				-0.091	0.4211	0.1043	-0.15	-0.94	-0.36				0.627	0.406	-0.05	0.977557	0.059097	0.024448777	0.034648073
522	C9JJN9	VPS8	VPS8 subunit of CORVET complex	23355	ENSG00000156931	3	-0.09	0.177	0.638	-0.19	0.527	-0.36	0.63	1.061	0.644	0.3679	0.5588	0.2101	0.0419	-0.562	-0.104	1.3048	0.5659	0.8121	-0.3	-0.01	-0.29	-0.18	-0.17	0.104	0.709	0.458	0.377	0.528108	0.261955	-0.017496362	0.279451271
1306	O95755	RAB36	RAB36, member RAS oncogene family	9609	ENSG00000100228	2	-0.61	-0.45	-0.16				0.318	-0.46	0.098	-0.576	-0.447	-0.229				0.4281	0.1129	0.1043	-0.57	-1.17	-0.42				0.045	0.328	0.16	0.867868	0.060125	-0.109496514	0.16962156
3621	Q9UHW5	GPN3	GPN-loop GTPase 3	51184	ENSG00000111231	2	0.345	0.241	0.486				0.585	1.185	1.112	0.401	-0.03	0.3249				1.1887	0.6615	0.4919	-0.24	0.149	-0.15				1.086	0.927	0.624	0.736283	0.260401	0.152581725	0.10781903
3172	Q96P48	ARAP1	ArfGAP with RhoGAP domain, ankyrin repe	116985	ENSG00000186635	2							0.41	0.25	0.673							-0.225	0.1487	0.153							-0.18	0.27	0.39	0.341866	0.285068	0.418803647	-0.133736109
125	A0A087X2I5	LSM7	LSM7 homolog, U6 small nuclear RNA and	51690	ENSG00000130332	2	-0.22	-0.58	-0.05	-0.08	-0.01	-0.35	-0.33	0.113	-0.73	-0.017	-0.407	-0.191	-0.05	-0.228	0.0829	-0.246	-0.054	0.1092	-0.56	-0.05	-0.81	-0.46	-0.13	0.059	0.117	-0.2	-0.4	0.529598	0.020937	-0.138190116	0.159127194
3456	Q9NPH0	ACP6	acid phosphatase 6, lysophosphatidic	51205	ENSG00000162836	2							-0.03	0.239	-1.16							-0.542	-0.021	-0.439							0.042	0.041	0.056	0.672043	-0.36338	0.016849481	-0.380226361

883	H7BZK6	USP46	ubiquitin specific peptidase 46	64854	ENSG00000109189	4	-0.2	0.483	-0.08				-0.96	-0.23	-0.05	0.2697	0.1542	0.2726				-0.62	0.1344	-0.084	0.048	0.674	0.158				0.002	-0.47	-0.36	0.762297	-0.18167	-0.194455342	0.012786118
3093	Q96BH1	RNF25	ring finger protein 25	64320	ENSG00000163481	1				0.421	0.38	-0.19	-0.84	-0.45	0.053				0.7215	-0.158	0.473	-0.217	0.2037	-0.234				-0.92	0.188	0.496	0.19	0.211	-0.14	0.781186	-0.1072	-0.234608604	0.127412175
742	Q9Y4D8	HECTD4	HECT domain E3 ubiquitin protein ligase 4	283450	ENSG00000173064	1							0.93	1.278	0.557							1.2574	0.3048	0.6218							0.561	0.595	0.81	0.762524	0.266847	0.193989654	0.072857718
2747	Q6ZNW5	GDPGP1	GDP-D-glucose phosphorylase 1	390637	ENSG00000183208	2	-1.01	0.465	-0.81	-1.28	2.342	-1.54	-1.6	2.431	-0.81	0.2581	-0.679	0.2006	-0.217	-1.87	0.0895	0.7737	-0.43	0.8983	0.403	1.504	-0.1	0.157	-0.11	0.116	1.396	0.305	1.002	0.487513	-0.72074	-0.093127074	-0.627615854
3118	Q96DX4	RSPRY1	ring finger and SPRY domain containing 1	89970	ENSG00000159579	4	0.133	0.443	0.095	0.187	0.116	-0.31	-0.62	-0.04	0.08	0.0863	-0.047	0.5159	0.1527	-0.462	-0.127	0.5339	-0.493	0.3893	-0.05	0.467	0.348	-0.21	-0.4	-0.4	0.727	-0.6	0.536	0.972485	-0.037	-0.052519404	0.015520887
3180	Q96QF0	RAB3IP	RAB3A interacting protein	117177	ENSG00000127328	4	0.299	0.992	-0.19	-0.01	0.172	-0.45	0.502	1.246	1.096	0.6552	-0.054	0.2003	0.0322	-0.271	-0.024	1.5824	1.2641	1.0646	0.267	0.221	-0.45	-0.5	-0.3	0.222	1.177	1.533	1.509	0.969516	-0.00284	-0.088651593	0.08581563
1457	P06748	NPM1	nucleophosmin 1	4869	ENSG00000181163	1							-1.36	-0.04	-0.18							-0.563	-0.493	-1.214							-1.14	-0.6	-0.16	0.919539	0.107812	0.229985119	-0.122173369
3792	Q9Y6G5	COMMD1(	COMM domain containing 10	51397	ENSG00000145781	2	-0.2	0.322	0.031	-0.29	-0.35	-0.78	-1.24	0.351	0.097	0.8073	0.475	0.0606	-0.695	-0.508	-0.954	-0.066	-0.108	-0.066	-1.02	0.2	0.632	-0.84	-0.01	-0.11	-0.01	0.648	-0.14	0.883965	-0.15573	-0.111793454	-0.043931969
698	F5GX12	GABARAP	GABA type A receptor associated protein lik	23710	ENSG00000139112	1	-0.42	-0.21	-0.28	0.065	-0.12	0.064				-0.312	-0.26	-0.068	-0.315	0.1537	-0.043				-0.12	0.019	-0.07	0.112	-0.05	-0.25				0.721814	-0.09105	-0.009806407	-0.08124313
3539	Q9NYT6	ZNF226	zinc finger protein 226	7769	ENSG00000167380	1	-0.05	-2.05	-1.13	-0.41	-0.85	1.325				-0.653	-0.412	-1.361	-1.13	1.4407	0.03				0.912	0.867	1.094	-0.36	-0.02	-0.48				0.450736	-0.86082	-0.178018127	-0.682800801
207	A0A0C4DH9	IGKV3OR2	immunoglobulin kappa variable 3/OR2-268 (	3523	ENSG00000233999	2	-0.37	-0.09	-0.53	-0.81	-0.68	-0.78				-0.345	-0.241	0.0885	-0.787	-0.676	-0.58				-0.42	-0.44	0.399	2.641	-0.91	0.129				0.358339	-0.77726	-0.120981138	-0.656279868
796	G8JLG1	SMC1A	structural maintece of chromosomes 1A	8243	ENSG00000072501	1				-0.96	-0.74	-0.14							-1.451	-0.175	-0.577							-0.46	0.296	-1.05				0.868172	-0.20535	0.121513834	-0.326860776
2252	P68871	HBB	hemoglobin subunit beta	3043	ENSG00000244734	6				-0.59	-0.34	-0.88							-0.439	-0.339	-0.271							2.649	-0.57	-0.1				0.496119	-1.26339	-0.255651683	-1.007738493
273	A0A1B0GUL	AC011005	novel protein similar to mitogen-activated pr(	NA	ENSG00000230626	10				-0.23	-0.18	-0.52							-0.08	-0.226	0.0587							-0.73	-0.55	-0.11				0.356931	0.154773	-0.224347812	0.379120444
2635	Q5T2L0	VTCN1	V-set domain containing T cell activation inh	79679	ENSG00000134258	4	-0.22	0.023	0.815	0.501	0.037	-0.3				-0.445	0.0255	0.852	0.1169	-0.76	-0.078				-0.12	-0.43	0.544	0.984	-0.24	0.039				0.846653	0.012202	0.190357341	-0.178155735
386	E7ESG1	DNASE1L'	deoxyribonuclease 1 like 1	1774	ENSG00000013563	2	-0.12	-0.52	-0.33	0.09	-0.28	0.238				0.1791	0.447	0.0721	-0.412	0.0573	0.117				-0.48	-0.5	0.134	0.029	-0.28	-0.11				0.358109	0.047323	-0.231539731	0.278862988
734	F8VU56	PTPRB	protein tyrosine phosphatase receptor type E	5787	ENSG00000127329	1				0.068	-0.33	-0.46							0.509	-0.168	0.0008							-0.62	-0.47	-0.41				0.211784	0.2585	-0.357262896	0.615763005
1817	P31146	CORO1A	coronin 1A	11151	ENSG00000102879	7	-1.25	-1.09	-1.24	-0.77	-0.77	0.215				-0.624	-0.905	-0.914	-0.555	-0.638	-0.799				-1.04	-0.4	2.195	2.686	-0.89	0.603				0.18156	-1.3444	-0.079046619	-1.265356873
3215	Q99584	S100A13	S100 calcium binding protein A13	6284	ENSG00000189171	4	-0.15	0.045	-0.36	0.121	0.121	-0.6				-0.763	-0.449	0.4435	-0.067	-0.496	0.6481				-0.14	-0.53	-0.26	-0.05	-0.11	-0.04				0.962474	0.050627	-0.02233839	0.072964944
2212	P62857	RPS28	ribosomal protein S28	6234	ENSG00000233927	3	0.105	0.046	-0.15	-0.11	0.344	0.107				-0.325	-0.183	-0.171	-0.139	-0.011	0.0845				-0.01	-0.53	-0.21	-0.63	-0.05	-0.01				0.205638	0.298955	0.181066416	0.117888489
1461	Q16778	H2BC21;H	H2B clustered histone 21;histone cluster 2 I	8349	ENSG00000184678	8	-1.7	-1.75	-1.62	-1.12	-1.54	1.015				-2.004	-0.557	-1.378	-1.298	-0.698	-1.248				-1.04	-0.65	2.665	2.153	-0.41	1.081				0.127086	-1.75339	0.077080031	-1.830469603
1815	P30679	GNA15	G protein subunit alpha 15	2769	ENSG00000060558	4				-0.03	-0.14	-0.38							0.007	-0.281	0.1038							0.168	-0.28	0.123				0.693012	-0.18504	-0.124402729	-0.060634876
2045	P51654	GPC3	glypican 3	2719	ENSG00000147257	4				0.259	-0.26	-0.27							-0.077	0.4515	-0.103							0.055	-0.05	0.158				0.775067	-0.14601	-0.180809709	0.034800153
1377	Q30167	HLA-DRB1	major histocompatibility complex, class II, D	3123	ENSG00000228080	3				0.008	0.307	-0.11							-0.042	-0.024	-0.619							0.343	0.854	-0.21				0.439458	-0.26186	0.296026212	-0.55788416
457	H0Y4Z2	ATP13A4	ATPase 13A4	84239	ENSG00000127249	3				-0.32	-0.01	-0.23							-0.125	-0.305	-0.031							0.069	-0.58	-0.15				0.961239	0.027889	-0.035917379	0.063806467
2925	Q8NBX0	SCCPDH	saccharopine dehydrogenase (putative)	51097	ENSG00000143653	2				1.097	1.383	-0.04							-0.365	-0.508	-0.577							-0.62	-0.45	-0.66				0.116878	1.392413	1.297796976	0.094616363
803	H0Y512	APMAP	adipocyte plasma membrane associated prc	57136	ENSG00000101474	3				0.029	0.302	0.199							0.0026	0.1406	-0.08							-0.03	0.022	-0.18				0.264556	0.236666	0.155620526	0.081045409
1036	O14556	GAPDHS	glyceraldehyde-3-phosphate dehydrogenase	26330	ENSG00000105679	4				1.069	0.389	-0.19							-0.067	0.557	-0.691							-1.11	-1.26	0.638				0.519715	1.002509	0.490683583	0.511825033
876	H7BYJ3	CIT	citron rho-interacting serine/threonine kinase	11113	ENSG00000122966	2	-0.1	-0.89	-0.29	0.128	0.022	-0.26				-0.234	0.5177	-0.157	0.1721	0.3049	-0.243				-0.11	0.816	0.531	0.106	-0.05	0.245				0.212062	-0.488	-0.290903155	-0.197091936
3616	Q9UHI5	SLC7A8	solute carrier family 7 member 8	23428	ENSG00000092068	2	-0.28	-0.25	-0.22	-0.75	0.056	-0.17				0.6031	0.4107	0.0461	-0.494	-0.168	-0.115				0.099	0.494	0.499	-0.17	0.221	0.025				0.198431	-0.46212	-0.314391651	-0.147726099
1833	P32189	GK	glycerol kinase	2710	ENSG00000198814	2	0.051	0.266	0.116	0.497	-0.04	-0.44				0.4428	0.3507	0.4769	0.2235	0.1607	-0.219				-0.09	-0.72	0.121	-0.05	-0.14	0.072				0.26447	0.210024	-0.163399316	0.373423044
637	E9PE72	ENPP1	ectonucleotide pyrophosphatase/phosphodie	5167	ENSG00000197594	1	-0.97	-1.69	-1.33	-1.13	-0.71	-0.33				0.4694	-0.323	0.8984	-1.412	-0.99	-0.886				-0.26	0.62	0.771	2.645	-0.68	-0.27				0.156478	-1.49985	-0.65379035	-0.846059198
3772	Q9Y5R8	TRAPPC1	trafficking protein particle complex 1	58485	ENSG00000170043	4				-0.06	0.043	-0.27							0.0655	-0.121	-0.054							-0.52	-0.3	-0.01				0.46503	0.182721	-0.059129787	0.241851063
2845	Q8IVM0	CCDC50	coiled-coil domain containing 50	152137	ENSG00000152492	8	0.181	0.116	-0.04	0.035	-0	-0.32				-0.428	-0.069	0.0995	-0.151	-0.097	0.0897				-0.21	-0.34	-0.01	-0.45	-0.12	-0.02				0.38685	0.188773	0.087374624	0.101398372
3112	Q96DD7	SHISA4	shisa family member 4	149345	ENSG00000198892	2	0.095	0.625	0.067	-0.07	0.129	-0.36				-0.165	0.3104	0.1534	0.2385	-0.121	-0.19				-0.49	0.593	-0.26	-0.32	-0.4	-0.21				0.495673	0.261755	0.042484693	0.219270599
1576	P12724	RNASE3	ribonuclease A family member 3	6037	ENSG00000169397	3				-0.21	0.928	0.663							0.1906	0.227	-0.45							0.929	0.417	-0.17				0.645421	0.071762	0.472898016	-0.40113609
1369	P01780	IGHV3-7	immunoglobulin heavy variable 3-7	28452	ENSG00000282211	4	-0.95	-0.54	-0.3	-0.4	-0.47	-0.39				-0.357	-0.524	-0.431	-0.39	-0.186	-0.535				-0.97	-0.18	2.195	2.045	0.301	0.662				0.13452	-1.18298	-0.104201923	-1.078782586
3414	Q9H8Y1	VRTN	vertebrae development associated	55237	ENSG00000133980	1	-0.03	0.606	-0.55	0.368	0.583	-0.24				-0.029	-0.033	0.2301	0.3035	-0.161	-0.26				0.171	0.155	-0.02	-0.23	-0.39	-0.31				0.638303	0.226983	0.112948491	0.114035004
715	F5H4Z8	THBS3	thrombospondin 3	7059	ENSG00000169231	5	-0.82	-1.25	-0.64	0.105	-0.58	-0.16				-0.09	1.1774	-0.111	-0.557	0.7086	-0.1				-0.79	-0.11	-0.4	-0.18	-0.61	0.168				0.202378	-0.23795	-0.73035453	0.492406858
1472	P07585	DCN	decorin	1634	ENSG00000011465	4	-0.02	-0.76	-0.8	0.1	0.393	-0.09				0.0047	-0.14	-0.707	-0.415	0.631	-0.561				0.46	0.291	1.152	0.366	0.568	0.212				0.12543	-0.70415	0.001764323	-0.705918212
2443	Q14145	KEAP1	kelch like ECH associated protein 1	9817	ENSG00000079999	3				0.04	0.295	-0.08							0.1411	-0.123	-0.059							-0.41	-0.24	-0.12				0.234016	0.338282	0.097750588	0.240531373
2914	Q8N9I0	SYT2	synaptotagmin 2	127833	ENSG00000143858	8				-0.35	-0.42	-1.68							-0.01	-0.068	-0.407							-0.36	-0.88	0.678				0.565758	-0.62933	-0.655699864	0.026367401
3663	Q9UMS4	PRPF19	pre-mRNA processing factor 19	27339	ENSG00000110107	2	0.023	0.098	-0.33	-0.31	-0.2	-0.5				-0.148	-0.122	0.1106	-0.157	0.1214	-0.14				-0.35	-0.89	-0.29	-0.08	-0.27	0.098				0.415651	0.096379	-0.146168758	0.242548096
1575	P12532	CKMT1B;(	creatine kinase, mitochondrial 1B;creatine ki	1159;54859(	ENSG00000237289	1	-0.29	-0.24	-0.46	-0.17	-0.22	-0.51				-0.06	-0.528	-0.188	-0.158	-0.327	-0.229				-0.42	-0.39	-0.23	0.135	-0.39	0.073				0.722686	-0.10955	-0.064989322	-0.044557569
1286	Q5H8X8	UTS2	urotensin 2	10911	ENSG00000049247	1	0.005	-0.26	0.031	0.536	0.931	0.011				0.0669	-0.205	-0.223	-0.494	-0.169	-0.397				-0.12	0.619	-0.02	-0.16	-0.25	-0.3				0.245062	0.247471	0.446447728	-0.198976443
13	A0A075B6K	IGLV3-9	immunoglobulin lambda variable 3-9 (gene/p	28804	ENSG00000211670	3	-1.47	-1.38	-0.71	-0.3	-0.67	-0.58				-0.914	-1.001	-0.505	-0.444	-0.164	-0.317				-0.71	-0.44	2.315	1.226	-0.35	0.478				0.137524	-1.2719	-0.293532046	-0.978372298
2540	Q15828	CST6	cystatin E/M	1474	ENSG00000175315	2				0.391	-0.74	1.224							-0.336	0.1255	-0.062							-0.08	0.614	-0.29				0.851059	0.210804	0.380801654	-0.169997656
204	A0A0C4DH3	IGHV5-51	immunoglobulin heavy variable 5-51	28388	ENSG00000277574	3	-0.39	-0.66	-0.03	-0.15	0.323	-0.14				-0.249	-0.116	-0.343	0.1509	-0.121	-0.244				-0.4	-0.66	1.346	1.836	0.147	0.411				0.316496	-0.62048	-0.021089709	-0.59938964
310	H0YJW4	COCH	cochlin	1690	ENSG00000100473	2				-0.27	0.06	-0.58							-0.814	-0.304	-0.349							-0.55	-0.31	0.087				0.716293	-0.00495	0.22641091	-0.231359318
1082	Q5JW88	GSTA4	glutathione S-transferase alpha 4	2941	ENSG00000170899	2	0.299	0.203	-0.05	-0.2	-0.08	-0.38				0.1868	0.1859	0.2044	0.3611	-0.325	0.091				0.03	-0.51	-0.09	0.655	0.289	0.368				0.722216	-0.15838	-0.152367496	-0.006009706
2652	Q5VIR6	VPS53	VPS53, GARP complex subunit;VPS53 sub	55275	ENSG00000141252	3	0.097	0.07	-0.03	0.069	0.005	-0.1				0.1244	0.1596	0.1291	0.1232	-0.141	0.1338				-0.34	-0.46	-0.24	-0.46	-0.14	0.194				0.09139	0.258625	-0.071026313	0.329651367
1615	P14780	MMP9	matrix metallopeptidase 9	4318	ENSG00000100985	5	-0.18	-0.16	-0.3	-0.96	-1.24	-0.56				-0.328	-0.084	0.0424	-0.947	-0.993	-0.895				-0.63	-1.39	0.819	3.173	-0.9	0.921				0.427336	-0.89762	-0.033067515	-0.864550918
820	H0YBZ2	CD74	CD74 molecule	972	ENSG00000019582	3	-0.45	0.948	-0.61	-0.56	-0.26	-0.43				0.1931	-0.127	0.4768	-0.272	0.8819	-0.328				0.846	1.276	0.92	1.311	0.041	0.316				0.116596	-1.01245	-0.364733227	-0.647714421
192	A0A0C4DGI	NRXN3	neurexin 3	9369	ENSG00000021645	3	-0.3	-0.62	-0.47	-0.32	0.174	-0.06				0.3368	0.4114	0.4589	-0.412	0.8432	0.2282				0.055	-0.18	-0.02	-0.03	-0.29	-0.17				0.106229	-0.16174	-0.579131418	0.417391786
737	F8VUJ3	POC1B-G/	POC1B-GALNT4 readthrough	100528030	ENSG00000259075	3	0.545	0.179	-0.01	-0.14	0.071	-0.34				0.9321	0.3095	0.4352	0.0326	-0.255	-0.127				0.276	0.923	0.054	-0.2	-0.31	-0.1				0.836768	-0.05484	-0.169175075	0.114336419
712	I3L3M4	AC003688	novel protein	NA	ENSG00000262302	1				-0.86	-0.09	0.589							-0.536	0.1556	-0.442							-0.28	-0.57	-0.46				0.815598	0.316652	0.15376012	0.162891597
2827	Q86Y38	XYLT1	xylosyltransferase 1	64131	ENSG00000103489	3	-0.41	-0.61	-0.2	0.02	0.221	-0.08				0.4095	0.1828	0.2613	0.2081	0.254	-0.138				0.097	-0.06	-0.13	0.108	0.195	0.24				0.143069	-0.25212	-0.372420243	0.120298399
1962	P47989	XDH	xanthine dehydrogenase	7498	ENSG00000158125	2				-0.29	-0.06	-0.35							-0.43	-0.196	0.02							-0.23	-0.17	0.159				0.738889	-0.15325	-0.031637402	-0.121612605
429	B4DM51	MSI2	musashi RNA binding protein 2	124540	ENSG00000153944	2	0.486	0.035	0.083	-0.15	0.32	-0.14				-0.175	0.0363	0.2758	0.1982	0.0377	0.1535				0.055	-0.75	-0.07	-0.38	0.127	0.156				0.390678	0.249334	0.01693584	0.232398218
162	C9JUH3	CCM2	CCM2 scaffold protein	83605	ENSG00000136280	2	0.326	0.574	-0.19	-0.27	-0.42	-0.43				0.0644	0.165	0.545	0.0313	-0.578	1.7				-0.11	0.688	-0.02	0.894	-0.87	0.042				0.684236	-0.17123	-0.388649858	0.217417748
968	K7ERI9	APOC1	apolipoprotein C1	341	ENSG00000130208	2	-0.77	-1.06	-0.02	0.077	0.264	0.114				-0.919	-0.324	-0.046	0.1472	-0.197	-0.014				-0.39	-0.3	0.523	1.679	0.035	0.221				0.384659	-0.52753	-0.006343378	-0.521190089
2261	X6R8F3	LCN2	lipocalin 2	3934	ENSG00000148346	5	-0.95	-1.3	-0.99	-1.04	-0.93	-0.02				-1.049	-0.426	-0.985	-0.564	-1.048	-1.384				-0.82	-0.92	2.705	2.138	0.493	1.201				0.093108	-1.67038	0.037662706	-1.708038275
3710	Q9Y289	SLC5A6	solute carrier family 5 member 6	8884	ENSG00000138074	3	0.189	0.047	0.072	0.652	0.587	0.2				-7E-04	0.0589	0.0937	0.7642	-0.402	0.3751				0.129	-0.33	0.035	0.318	-0.35	-0.17				0.336641	0.351511	0.142971218	0.208539922
3133	Q96G61	NUDT11	nudix hydrolase 11	55190	ENSG00000196368	7	-0.29	0.184	-0.33	-0.04	-0.07	-0.22				0.4743	-0.311	0.4693	0.0196	0.0903	0.131				-0.16	-0.32	-0.48	-0.35	-0.21	-0.02				0.126044	0.129055	-0.273733709	0.402788216
1906	P41218	MNDA	myeloid cell nuclear differentiation antigen	4332	ENSG00000163563	3	-0.69	-0.45	-0.67	-0.47	-0.42	-0.11				-0.333	-0.236	-0.406	-0.38	-0.591	-0.337				-0.25	-0.67	1.422	1.847	-0.28	0.383				0.17096	-0.8752	-0.087654628	-0.78754389
2013	P49915	GMPS	guanine monophosphate synthase	8833	ENSG00000163655	3	-0.15	-0.35	-0.23	-0.02	0.119	-0.18				-0.173	-0.265	0.2264	-0.1	-0.249	0.2523				-0.68	-0.45	-0.25	-0.09	-0.23	0.136				0.46903	0.125159	-0.084639681	0.209798967
1362	P01704	IGLV2-14	immunoglobulin lambda variable 2-14	28815	ENSG00000211666	1				-0.98	-0.88	-0.72							-0.77	-0.616	-0.446							2.798	-1.17	0.069				0.502282	-1.42966	-0.25383994	-1.175817604
2292	Q01628	IFITM3	interferon induced transmembrane protein 3	10410	ENSG00000142089	2	-0.17	0.198	0.209	-0.31	-0.63	1.299				-0.651	-0.067	0.3965	0.1928	-0.408	-0.395				-0.59	-0.42	-0.22	-0.54	2.033	-0.65				0.892362	0.165997	0.254885537	-0.088888178
254	H0Y329	WDR45	WD repeat domain 45	11152	ENSG00000196998	2	0.053	0.059	-0.11	0.036	-0.21	-0.41				-0.048	-0.022	0.0022	0.0712	-0.375	0.1025				-0.2	-0.4	-0.12	-0.76	-0.61	0.051				0.229963	0.241409	-0.052265749	0.293674879
3094	Q96BI3	APH1A	aph-1 homolog A, gamma-secretase subuni	51107	ENSG00000117362	2	0.261	0.091	0.07	-0.09	-0.52	0.042				-0.265	-0.058	0.0809	-0.037	-0.06	0.6024				-0.44	-0.33	-0.58	-0.12	-0.39	-0.38				0.128719	0.348764	-0.068538933	0.417303244
1620	P15153	RAC2	Rac family small GTPase 2	5880	ENSG00000128340	8	-1.14	-1.64	-1.47	-0.79	-0.52	-0.16				-0.774	-0.599	-1.006	-0.681	-0.342	-0.613				-0.84	-0.98	2.126	2.163	-0.33	0.384				0.162479	-1.37308	-0.283438834	-1.089638253
1392	Q5VY30	RBP4	retinol binding protein 4	5950	ENSG00000138207	3				-0.25	-0.3	0.317							-0.802	-0.109	-0.489							1.753	-0.23	-0.01				0.451235	-0.58032	0.389797074	-0.970117827
335	E7ER27	HSD17B4	hydroxysteroid 17-beta dehydrogenase 4	3295	ENSG00000133835	3				-0.27	-0.55	1.679							-0.404	0.0932	-0.508							0.121	0.12	-0.06				0.761929	0.226585	0.559364684	-0.332779821
2771	Q7L8C5	SYT13	synaptotagmin 13	57586	ENSG00000019505	3	-0.23	-0.12	-0.06	-0.03	0.11	-0.44				0.3668	-0.047	0.1051	0.0423	-0.265	0.2101				-0.16	-0.16	-0.04	-0.02	-0.21	0.401				0.450526	-0.09919	-0.19917966	0.099991983
2715	Q6PKG0	LARP1	La ribonucleoprotein domain family member	23367	ENSG00000155506	7	0.049	-0.28	-0.05	0.092	-0.04	-0.42				-0.022	0.1328	0.0543	-0.194	-0.058	0.3324				-0.18	-0.59	-0.13	-0.63	-0.19	-0.19				0.138813	0.20645	-0.150125138	0.356575491
187	A0A0C4DG3	AGFG2	ArfGAP with FG repeats 2	3268	ENSG00000106351	2	-0.41	-0.25	-0.37	-0.37	-0.24	-0.62				-0.23	0.0822	-0.091	-0.046	0.0891	-0.167				-0.3	-0.48	-0.25	-0.23	-0.27	0.042				0.082467	-0.13223	-0.318545716	0.186311632
2188	P62253	UBE2G1	ubiquitin conjugating enzyme E2 G1	7326	ENSG00000132388	3	0.428	0.358	0.222	0.158	0.098	-0.2				0.1806	0.1558	0.2357	0.1564	-0.091	0.0322				-0.17	-1.17	-0.09	-0.11	-0.27	0.147				0.161319	0.454758	0.066285582	0.388472874
568	E7EPK0	LIMCH1	LIM and calponin homology domains 1	22998	ENSG00000064042	3	-0.3	-0.09	-0.42	-0.08	-0.16	-0.14				0.1191	0.0301	-0.325	-0.161	-0.005	0.3677				-0.23	0.345	-0.35	-0.13	0.054	0.172				0.405081	-0.17603	-0.202620418	0.026590197
3361	Q9H1M4	DEFB127	defensin beta 127	140850	ENSG00000088782	2				1.424	0.745	0.291							-0.06	-0.816	0.5655							-0.34	-0.78	-0.8				0.163518	1.462517	0.923826667	0.538690356

1360	P01700	IGLV1-47	immunoglobulin lambda variable 1-47	28822	ENSG00000211648	2	-1.09	-1.01	-0.51	-0.83	-0.67	-0.24	-0.711	-0.826	-0.723	-0.645	-0.273	-0.584	-0.89	-0.58	2.589	1.948	0.15	0.661	0.125208	-1.37145	-0.097127698	-1.274318129
431	B4DLR8	NQO1	NAD(P)H quinone dehydrogenase 1	1728	ENSG00000181019	3	0.747	1.506	1.187	0.314	0.881	-1.02	-1.38	-2.075	-1.281	-1.098	-0.167	0.5947	0.556	-1.65	1.654	-1.29	0.6	-0.19	0.207573	0.657298	1.504336891	-0.847038751
136	A0A096LPE:	SAA2-SAA	SAA2-SAA4 readthrough	100528017	ENSG00000255071	3				-1.39	-1.29	0.242				-1.243	-1.03	-0.975				2.94	-1.3	0.968	0.389926	-1.68408	0.268563474	-1.95264703
1877	P36894	BMPR1A	bone morphogenetic protein receptor type 1/	657	ENSG00000107779	3	-0.35	-0.05	0.035	0.243	-0.04	-0.06	0.1562	0.1216	0.0636	0.2485	-0.133	0.2367	-0.17	-0.37	-0.29	-0.25	-0.25	0.186	0.144225	0.154374	-0.153206282	0.307579807
764	F8WBH7	PSMG1	proteasome assembly chaperone 1	8624	ENSG00000183527	2	0.497	0.689	0.467	0.387	0.267	0.853	0.3087	0.1356	-0.132	0.041	0.3174	0.555	-0.19	-0.47	-0.47	0.127	0.447	0.238	0.103103	0.579086	0.322457219	0.256628422
2876	Q8IZH2	XRN1	5'-3' exoribonuclease 1	54464	ENSG00000114127	2	-0.54	-0.79	-0.68	0.146	-0.34	1.312	-1E-03	-0.469	0.1867	-0.159	0.0214	-0.075	0.273	1.035	-0.49	-0.27	0.174	-0.08	0.808935	-0.25349	-0.063713986	-0.18977724
327	A0A2R8Y79	ACTB	actin beta	60	ENSG00000075624	27				0.972	-0.58	0.5				-0.352	0.1363	0.3322				1.078	-0.36	0.023	0.919695	0.047808	0.256830096	-0.209021938
2395	Q13275	SEMA3F	semaphorin 3F	6405	ENSG00000001617	33				-0.71	-0.36	-0.14				-0.531	0.6482	-0.451				-0.1	0.12	-0.04	0.65099	-0.3954	-0.291138932	-0.104256485
477	B8ZZW5	AVL9	AVL9 cell migration associated	23080	ENSG00000105778	3	0.042	0.28	0.166	0.047	-0.19	-0.17	0.3522	0.0611	0.0338	0.0349	-0.13	0.201	-0.17	-0.4	0.119	-0.27	-0.13	0.131	0.326532	0.146715	-0.064174912	0.210890304
3445	Q9HCS5	EPB41L4A	erythrocyte membrane protein band 4.1 like	64097	ENSG00000129595	1				0.223	-0.21	-0.09				0.2217	-0.643	0.4253				-0.49	-0.56	-0.5	0.363923	0.489523	-0.028345602	0.517868623
1819	P31151	S100A7	S100 calcium binding protein A7	6278	ENSG00000143556	2				-0.14	0.082	-0.57				-0.187	-0.299	-0.717				2.196	0.471	-0.06	0.299218	-1.07776	0.193387764	-1.271144621
2298	Q02108	GUCY1A1	guanylate cyclase 1 soluble subunit alpha 1	2982	ENSG00000164116	2				0.09	-0.21	0.315				-0.03	0.1325	0.156				0.181	0.025	0.258	0.877195	-0.09146	-0.022577869	-0.068877494
2938	Q8NEG4	FAM83F	family with sequence similarity 83 member F	113828	ENSG00000133477	2	0.269	0.308	-0.02	0.201	-0.52	-0.03	-0.209	0.0139	0.0566	-0.21	-0.297	0.0813	-0.2	-0.41	-0.26	-0.3	-0.26	-0.31	0.16816	0.326007	0.1285391	0.197468118
599	E7EMF1	ITGA2	integrin subunit alpha 2	3673	ENSG00000164171	2	-0.44	-0.1	-0.13	-0.07	-0.48	-0.08	0.2361	0.1432	-0.21	-0.185	0.4431	0.0053	-0.14	0.029	0.158	0.207	0.053	-0.02	0.162015	-0.26414	-0.288795988	0.024651495
852	H3BQJ8	LY6L	lymphocyte antigen 6 family member L	101928108	ENSG00000261667	1				0.768	0.449	-0.09				0.445	-0.116	0.0601				0.08	-0.04	-0.03	0.519405	0.37339	0.24738234	0.126007892
208	A0A0C4DH7	IGKV1-12	immunoglobulin kappa variable 1-12	28940	ENSG00000243290	1	-0.38	-0.35	-0.07	-0.06	0.039	-0.66	0.2688	-0.011	0.1509	0.1459	-0.261	-0.28	0.133	-0	-0.14	-0.07	0.05	0.095	0.219066	-0.25918	-0.249419681	-0.009758883
2717	Q6RW13	AGTRAP	angiotensin II receptor associated protein	57085	ENSG00000177674	1	0.047	0.042	-0.06	0.092	0.283	-0.38	0.2135	-0.044	0.2029	0.4945	-0.145	0.0841	-0.24	-0.32	-0	-0.65	-0.15	0.184	0.230194	0.19991	-0.131249957	0.331160123
567	D6RCP9	DCK	deoxycytidine kinase	1633	ENSG00000156136	2	0.025	-0.01	8E-04	0.313	-0.13	-0.83	0.0763	-0.023	-0.052	0.1859	-0.042	-0.042	-0.23	-0.42	-0.42	-0.12	-0.22	0.298	0.593831	0.081333	-0.122493207	0.203826552
2622	Q5R3F8	ELFN2	extracellular leucine rich repeat and fibronec	114794	ENSG00000166897	1				-0.15	-0.22	-0.09				0.1718	-0.624	0.3425				-0.42	-0.08	-0.63	0.632936	0.225475	-0.114238138	0.339713112
3166	Q96MX6	WDR92	WD repeat domain 92	116143	ENSG00000243667	1				-0.09	0.09	-0.29				0.0113	-0.06	-0.122				0.204	-0.3	-0.05	0.96375	-0.04732	-0.039334841	-0.007987495
879	H7C1R9	SNED1	sushi, nidogen and EGF like domains 1	25992	ENSG00000162804	1	0.025	-0.19	0.092	-0.22	-0.28	0.182	-0.057	0.2443	0.0764	-0.406	0.0317	-0.066	-0.1	-0.12	0.016	-0.35	-0.08	-0.24	0.653951	0.081583	-0.0363788	0.117961715
784	G3XAD9	ST6GALN/	ST6 N-acetylgalactosaminide alpha-2,6-sial	55808	ENSG00000070526	1	-0.22	-0	0.105	0.016	0.168	-0.24	0.2707	0.2543	-0.079	0.1542	-0.009	-0.432	-0.39	0.112	0.6	-0.04	-0.39	-0.06	0.949945	-0.00116	-0.054880201	0.053715242
360	A2A492	BNIPL	BCL2 interacting protein like	149428	ENSG00000163141	3	-0.05	0.635	-0.22	0.145	-0.78	-0.49	-0.355	0.4226	-0.041	0.5974	-0.089	-0.079	-0.48	-1.73	-0.29	-0.54	-0.26	0.57	0.434953	0.329759	-0.20232365	0.532083146
33	E9PE15	CLCN3	chloride voltage-gated channel 3	1182	ENSG00000109572	2	0.071	-0.17	0.217	-0.06	-0.29	-0.08	0.0838	0.2228	0.1038	-0.086	-0.148	-0.051	0.016	-0.09	-0.12	-0.3	-0.05	0.083	0.642397	0.024222	-0.074446685	0.098668574
2428	Q13637	RAB32	RAB32, member RAS oncogene family	10981	ENSG00000118508	4	-0.01	0.331	0.451	-0.13	-0.41	-0.17	0.1077	0.1931	-0.082	-0.172	-0.022	-0.098	-0.36	-1	-0.32	-0.38	-0.13	0.147	0.251039	0.348742	0.021890717	0.326851026
221	A0A0G2JIW	TRIM26	tripartite motif containing 26	7726	ENSG00000230230	2				1.202	-0.41	-0.08				0.1427	-0.11	-0.391				0.364	-0.25	-0.14	0.80996	0.246879	0.358066303	-0.111187095
662	E9PJP2	SOGA3	SOGA family member 3	387104	ENSG00000214338	1				-0.84	-0.32	0.385				-0.647	0.5371	0.6432				-0.24	0.667	-0.41	0.801353	-0.26392	-0.435997665	0.172075287
2468	Q14651	PLS1	plastin 1	5357	ENSG00000120756	5	0.486	0.114	0.314	0.141	-0.17	-0.13	0.3124	0.5242	0.1865	-0.09	0.2772	0.0161	-0.07	0.136	0.087	0.042	-0.26	0.002	0.392672	0.135479	-0.079206761	0.21468611
2294	Q01955	COL4A3	collagen type IV alpha 3 chain	1285	ENSG00000169031	1				-0.3	-0.51	0.043				-0.472	0.1151	-0.251				-0.12	-0.02	0.053	0.648837	-0.22621	-0.054142351	-0.172072416
59	A0A087WW	TMEM237	transmembrane protein 237	65062	ENSG00000155755	2				0.171	0.14	-0.28				-0.048	-0.089	-0.1				-0.42	-0.09	-0.12	0.530419	0.220324	0.089679553	0.130643987
2681	Q6GMV2	SMYD5	SMYD family member 5	10322	ENSG00000135632	2				0.105	-0.04	-0.23				0.1784	0.0373	0.0411				-0.3	-0.15	0.087	0.48102	0.066259	-0.140655285	0.206913916
3195	Q96SZ5	ADO	2-aminoethanethiol dioxygenase	84890	ENSG00000181915	2	-0.16	0.298	-0.29	-0.17	-0.11	-0.44	-0.112	-0.204	-9E-04	-0.154	-0.202	-0.122	-0.49	-0.84	-0.22	-0.6	-0.35	-0.04	0.199583	0.276783	-0.014874322	0.291657161
90	A0A087X0J	ZNF445	zinc finger protein 445	353274	ENSG00000185219	1				-0.18	-0.72	-0.19				-0.434	0.4273	0.0869				0.02	-0.4	-0.27	0.552564	-0.14686	-0.389313996	0.242457382
1870	V9GYD0	ARL2-SNX	ARL2-SNX15 readthrough (NMD candidate	100528018	ENSG00000273003	2				0.627	-0.14	-0.33				-0.152	-0.367	0.4704				0.082	-0.84	-0.08	0.780952	0.33173	0.068311279	0.263419102
228	A0A0G2JMZ	OCLN	occludin	100506658	ENSG00000197822	1				0.13	-0.08	0.007				0.3646	-0.242	-0.055				-0.31	-0.37	-0.38	0.219668	0.373368	-0.002836624	0.376204176
3319	Q9BYN0	SRXN1	sulfiredoxin 1	140809	ENSG00000271303	1				0.004	0.724	-0.24				0.2485	-0.006	0.1349				-0.09	0.116	0.233	0.969614	0.077771	0.038540122	0.039231211
1187	O75054	IGSF3	immunoglobulin superfamily member 3	3321	ENSG00000143061	3	-0.81	0.104	0.026	-0.29	-0.17	-0.4	0.3518	-0.167	-0.165	0.2028	-2E-04	0.2763	-0.3	-0.5	-0.4	-0.11	0.105	0.068	0.246199	-0.06664	-0.339197295	0.272552817
272	A0A1B0GUI	RALGAPA	Ral GTPase activating protein catalytic alph	253959	ENSG00000174373	5	0.113	-0.2	-0.33	-0.27	-0.16	-0.47	-0.089	0.022	-0.077	-0.231	0.1758	-0.099	-0.11	-0.46	-0.41	-0.42	-0.48	0.109	0.259385	0.079603	-0.167728792	0.247332171
901	I3L2N2	DVL2	dishevelled segment polarity protein 2	1856	ENSG00000004975	2	0.167	-0.02	-0.23	0.01	0.134	-0.3	-0.014	0.1258	-0.254	0.2356	-0.013	0.0074	-0.63	-0.99	0.164	-0.5	0.063	0.21	0.427744	0.239949	-0.055629165	0.295578219
198	A0A0C4DG	DDOST	dolichyl-diphosphooligosaccharide--protein g	1650	ENSG00000244038	2				0.503	1.087	0.264				-0.517	-0.711	-0.806				-0.38	-0.24	-0.66	0.056783	1.041343	1.295388649	-0.254045819
2051	P51991	HNRNPA3	heterogeneous nuclear ribonucleoprotein A3	220988	ENSG00000170144	1	0.084	0.097	0.245	0.074	9E-04	-0.26	-0.063	-0.056	-0.098	-0.087	-0.178	-0.019	0.08	-0.18	0.028	0.214	-0.11	0.206	0.401386	0.000919	0.122835116	-0.121915822
2229	P63092	GNAS	GNAS complex locus	2778	ENSG00000087460	23	0.343	0.75	0.066	-0.22	0.471	-0.43	0.2755	0.1728	0.4568	0.111	-0.33	0.2377	-0.19	-0.18	-0.24	-0.39	-0.37	0.101	0.229794	0.376268	0.010106655	0.366161219
3852	X6R3G6	MFGE8	milk fat globule-EGF factor 8 protein	4240	ENSG00000140545	16				-0.17	-0.39	0.322				0.3771	-0.722	0.2046				0.57	-0.26	-0.3	0.984835	-0.08367	-0.034489204	-0.049176464
287	J3QKU4	STRADA	STE20 related adaptor alpha	92335	ENSG00000266173	1	-0.35	0.128	0.038	0.066	0.342	-0.22	-0.207	-0.279	-0.093	-0.035	-0.34	-0.103	-0.39	-0.76	-0.24	-0.52	-0.17	0.018	0.18786	0.342111	0.175856972	0.166253833
2954	Q8NFZ8	CADM4	cell adhesion molecule 4	199731	ENSG00000105767	2	0.361	0.197	0.138	0.107	0.095	-0.16	0.6981	0.1893	0.1726	0.172	0.0061	-0.383	0.318	0.085	0.309	-0.2	0.238	0.08	0.992411	-0.01497	-0.019613588	0.004647315
2139	P60880	SNAP25	synaptosome associated protein 25	6616	ENSG00000132639	2				-0.75	-0.24	-0.03				-0.071	-0.264	-0.073				0.252	-0.19	-0.36	0.696283	-0.23934	-0.202543892	-0.036792854
2272	P84101	SERF2	small EDRK-rich factor 2	10169	ENSG00000140264	2				-0.01	-0.03	-0.4				-0.109	-0.075	0.0261				-0.44	-0.26	0.03	0.695702	0.075662	-0.095229269	0.170891123
403	B0YIW2	APOC3	apolipoprotein C3	345	ENSG00000110245	1				-0.39	-0.54	0.673				-0.432	0.2345	0.3396				0.053	0.17	-0.16	0.955064	-0.10685	-0.134188543	0.027339754
2864	Q8IYB5	SMAP1	small ArfGAP 1	60682	ENSG00000112305	2	-0.13	0.054	-0.15	-0.09	-0.39	-0.36	0.0179	-0.154	-0.295	-0.256	-0.215	0.1123	-0.27	-0.96	-0.28	-0.54	-0.53	-0.2	0.146665	0.285631	-0.045116692	0.330748187
1656	P17812	CTPS1	CTP synthase 1	1503	ENSG00000171793	5				-0.2	0.535	-0.18				-0.136	-0.438	-0.043				0.133	-0.12	0.289	0.582809	-0.0513	0.256297763	-0.307601495
1370	P0DP04	IGHV3-43D	immunoglobulin heavy variable 3-43D	103106901	NA	3				-0.57	-0.47	-1.46				-0.836	-1.094	-0.456				3.577	-0.81	0.136	0.42687	-1.7996	-0.038115333	-1.761488269
2978	Q8TD43	TRPM4	transient receptor potential cation channel su	54795	ENSG00000130529	2	-0.23	-0.33	-0.13	-0.48	-0.6	-0.25	0.004	0.1359	-0.026	-0.357	-0.192	0.064	-0.06	0.823	0.535	-0.46	-0.59	0.282	0.291539	-0.42465	-0.273186683	-0.151465451
3015	Q8WVT3	TRAPPC1:	trafficking protein particle complex 12	51112	ENSG00000274891	2	-0.04	0.111	0.148	-0.26	0.067	-0.33	-0.181	-0.232	-0.145	0.0176	-0.454	0.1591	-0.25	-0.54	0.032	-0.4	-0.23	0.131	0.618805	0.158341	0.089034829	0.069306456
3007	Q8WUN7	UBTD2	ubiquitin domain containing 2	92181	ENSG00000168246	3				-0.59	-0.55	-1.01				-0.374	0.5793	-0.068				-1.25	-0.03	-0.08	0.411244	-0.26173	-0.760764586	0.499031123
1428	P05023	ATP1A1	ATPase Na+/K+ transporting subunit alpha	476	ENSG00000163399	5	0.231	0.11	0.69	0.404	0.735	0.013	-0.236	-0.312	0.1523	0.0993	-0.238	-0.24	-0.52	0.147	-0.16	-0.16	-0.06	0.154	0.084401	0.465059	0.493171998	-0.028113068
131	A0A096LNY	TMEM114	transmembrane protein 114	283953	ENSG00000232258	1				-0.85	-0.08	-0				-0.363	0.0807	-0.477				-0.46	0.327	-0.03	0.806172	-0.25335	-0.057182796	-0.196163805
2620	Q5QJ74	TBCEL	tubulin folding cofactor E like	219899	ENSG00000154114	2				0.019	0.051	-0.4				0.0402	-0.085	0.0316				-0.7	-0.23	-0.06	0.47322	0.219958	-0.106858771	0.326817135
860	H3BRS6	ADPGK	ADP dependent glucokinase	83440	ENSG00000159322	1				0.834	0.343	0.202				-0.031	0.1424	-0.168				-0.23	-0.25	-0.22	0.121364	0.692367	0.478848238	0.213518883
79	A0A087WZ5	SPPL2B	signal peptide peptidase like 2B	56928	ENSG00000005206	1				0.145	0.618	-0.35				0.528	-0.363	-0.306				-0.25	-0.41	-0.14	0.645513	0.403832	0.183437193	0.220394601
594	E5RK69	ANXA6	annexin A6	309	ENSG00000197043	36	0.3	0.02	-0.09	-0.3	-0.01	-0.54	-0.011	0.7078	0.0957	0.4934	0.1394	0.1035	-0.15	-0.9	-0.2	-0.69	0.023	-0.16	0.116662	0.241809	-0.357994159	0.599803312
2655	Q5VV41	ARHGEF1	Rho guanine nucleotide exchange factor 16	27237	ENSG00000130762	2				0.166	0.116	0.058				-0.062	0.2815	0.0846				0.01	-0.09	-0.03	0.404988	0.150227	0.012095424	0.138131504
3440	Q9HCC8	GDPD2	glycerophosphodiester phosphodiesterase d	54857	ENSG00000130055	2				0.364	0.913	0.106				0.5523	-0.732	0.1177				0.267	-0.29	-0.59	0.482937	0.66527	0.481487284	0.183783211
206	A0A0C4DH4	IGHV2-70D	immunoglobulin heavy variable 2-70D	102723168	ENSG00000211974	2	-1.18	-0.71	-2.24	-0.55	-0.6	-0.68	-1.517	-1.17	-0.271	-0.641	-0.934	-0.912	-1.73	-1.11	2.463	2.654	-1.15	1.577	0.250949	-1.44291	-0.084482546	-1.358426695
1581	P13010	XRCC5	X-ray repair cross complementing 5	7520	ENSG00000079246	2	0.05	0.023	-0.12	-0.1	0.244	-0.39	-0.019	-0.348	-0.22	0.2124	-0.466	-0.176	-0.38	-0.54	0.377	0.868	-0.25	0.24	0.698458	-0.09941	0.120623075	-0.22003733
3084	Q96A59	MARVELD	MARVEL domain containing 3	91862	ENSG00000140832	7				-0.13	0.396	-0.22				0.0423	0.0151	-0.098				-0.15	-0.4	0.069	0.745115	0.179501	0.031688291	0.147812964
3308	S4R3Y2	SLC46A2	solute carrier family 46 member 2	57864	ENSG00000119457	1				0.27	0.28	0.436				0.6335	-0.639	0.2098				-0.03	-0.3	-0.65	0.394844	0.65333	0.260427778	0.392901784
1361	P01701	IGLV1-51	immunoglobulin lambda variable 1-51	28820	ENSG00000211644	2				-0.69	-0.64	-0.47				-0.662	-0.227	-0.854				1.393	-0.02	1.056	0.119463	-1.40926	-0.019958888	-1.389305701
2296	Q01970	PLCB3	phospholipase C beta 3	5331	ENSG00000149782	6	-0.31	-0.17	-0.66	-0.01	0.037	-0.27	-0.25	-0.2	-0.252	0.1477	0.1091	-0.011	-0.68	-0.37	-0.76	-0.14	-0.04	-0.07	0.375811	0.113153	-0.154187647	0.267340958
710	F5H3P3	ARHGDIB	Rho GDP dissociation inhibitor beta	397	ENSG00000111348	3	-0.6	0.519	-0.43	-1.58	-0.96	-0.35	-0.144	0.0122	-0.146	-0.931	-0.468	-1.407	-0.14	-0.56	2.342	2.64	-2.11	0.834	0.39083	-1.07023	-0.054555246	-1.015675422
2841	Q8IV50	LYSMD2	LysM domain containing 2	256586	ENSG00000140280	1				-0.31	-0.07	-0.48				-0.034	0.1486	0.2836				-0.17	-0.05	0.075	0.187884	-0.23652	-0.416835651	0.18031744
760	F8WA39	MTMR1	myotubularin related protein 1	8776	ENSG00000063601	4	0.025	0.519	-0.04	-0.21	0.223	-0.11	0.3785	0.0364	0.2164	0.2694	-0.555	0.1051	-0.05	0.011	-0.18	-0.7	-0.46	0.235	0.432066	0.260209	-0.005337211	0.265546144
156	A0A0A0MS\	C1QB	complement C1q B chain	713	ENSG00000173369	1				-0.27	-0.44	0.387				-0.355	0.1374	-0.124				0.195	-0.02	-0.16	0.91187	-0.11543	0.004106479	-0.119537947
3527	Q9NWZ3	IRAK4	interleukin 1 receptor associated kinase 4	51135	ENSG00000198001	4	-0.09	-0.18	0.043	-0.35	-0.17	-0.3	0.2998	-0.082	-0.21	-0.135	-0.029	0.0011	-0.1	-0.62	0.524	-0.53	-0.24	-0.2	0.637522	0.019534	-0.148952314	0.168486234
3272	Q9BUF5	TUBB6	tubulin beta 6 class V	84617	ENSG00000176014	9				0.227	0.465	0.109				-0.172	0.1267	0.2195				-0.13	0.25	-0.1	0.465377	0.261638	0.208764267	0.05287352
315	A0A2R8Y4C	TLR5	toll like receptor 5	7100	ENSG00000187554	3	-0.8	-0.4	-0.29	0.253	0.201	-0.39	-0.124	-0.435	-0.394	-0.041	0.0803	-0.229	-0.26	-0.24	0.177	-0.07	-0.01	0.006	0.668738	-0.1726	-0.047542113	-0.125056934
864	H3BTK5	SENP8	SUMO peptidase family member, NEDD8 s	123228	ENSG00000166192	1				-0.01	-0.2	-0.69				-0.089	-0.32	-0.065				-0.37	-0.59	-0.09	0.764284	0.05022	-0.142543766	0.192763353
115	F8WCZ6	C1S	complement C1s	716	ENSG00000182326	3				-0.63	-0.67	-0.53				-0.706	-0.495	-0.626				0.896	-0.58	-0.13	0.338524	-0.66979	-0.000318547	-0.669473027
2422	Q13591	SEMA5A	semaphorin 5A	9037	ENSG00000112902	2				-0.15	-0.38	-0.35				-0.189	0.0336	-0.241				-0.03	0.572	-0.1	0.313494	-0.43542	-0.15865529	-0.276764575
768	F8WCE5	LMLN	leishmanolysin like peptidase	89782	ENSG00000185621	3	0.035	0.139	0.285	-0.08	-0.55	-0.26	0.1297	0.4866	0.5133	-0.388	-0.308	-0.094	-0.02	0.018	0.071	-0.66	-0.59	-0.17	0.526459	0.153278	-0.127574112	0.280852363

683	E9PP13	POLR2M	RNA polymerase II subunit M	81488	ENSG00000255529	1				0.243	0.438	-0.29				0.327	-0.359	-0.035				-0.41	-0.4	0.011	0.530927	0.396074	0.153549114	0.24252478
3187	Q96RQ9	IL4I1	interleukin 4 induced 1	259307	ENSG00000104951	2	1.294	-0.47	-0.37	0.59	0.78	-0.45	-0.751	-0.635	-0.591	-0.337	-0.689	-0.553	-0.27	-0.1	1.155	1.185	-0.1	0.091	0.146641	-0.09718	0.822045704	-0.919229009
296	D6RHC4	ANKHD1	ankyrin repeat and KH domain containing 1	54882	ENSG00000131503	1				-0.12	0.456	-0.15				0.5111	0.5101	0.0137				0.347	0.672	0.081	0.592881	-0.30445	-0.282436214	-0.022013126
217	A0A1W2PP7	HLA-DQA1	major histocompatibility complex, class II, D	3117	ENSG00000236418	1	-1.16	-0.72	-0.36	-0.63	-0.15	0.248	-0.956	-0.746	-0.785	0.0497	-1.358	-1.496	-0.97	-0.8	1.287	3.146	0.454	1.179	0.147134	-1.17893	0.419840492	-1.598774393
1808	P30447	HLA-A	major histocompatibility complex, class I, A	3105	ENSG00000223980	9				0.069	0.354	-0.14				0.2406	-0.037	0.1073				0.03	-0.27	-0.13	0.466583	0.217541	-0.010174241	0.227715606
19	A0A087WT4	GRIPAP1	GRIP1 associated protein 1	56850	ENSG00000068400	2				0.07	0.217	-0.11				0.1664	0.1622	0.1256				0.142	0.16	0.169	0.568067	-0.09916	-0.093924026	-0.005237465
3399	Q9H5V8	CDCP1	CUB domain containing protein 1	64866	ENSG00000163814	1	-0.39	-0.1	0.038	0.097	6E-04	0.041	0.0277	-0.073	-0.383	0.0553	-0.207	-0.278	-0.57	-0.41	-0.13	0.054	-0.23	0.103	0.637625	0.142183	0.089509412	0.052673679
2059	P52597	HNRNPF	heterogeneous nuclear ribonucleoprotein F	3185	ENSG00000169813	4	0.062	0.358	0.019	-0.07	0.143	-0.16	0.0961	0.0663	0.1015	0.2191	-0.103	-0.127	-0.14	-0.64	0.087	0.411	-0.09	0.374	0.946387	0.058274	0.016405232	0.04186909
3542	Q9NYY1	IL20	interleukin 20	50604	ENSG00000162891	1				0.225	-0.55	0.231				-0.474	0.4187	0.5997				0.182	0.242	0.006	0.880089	-0.17326	-0.211263532	0.037999968
3500	Q9NSA0	SLC22A11	solute carrier family 22 member 11	55867	ENSG00000168065	2	-0.15	0.032	0.714	-0.32	0.457	0.016	0.0313	0.3424	0.323	-0.189	1.0931	-0.487	-0.18	0.195	-0.04	-0.4	0.053	0.092	0.711249	0.17263	-0.060855148	0.233485226
682	E9PNV3	RDX	radixin	5962	ENSG00000137710	13				-0.36	-0.31	-0.57				0.1345	-0.18	-0.034				-0.29	-0.39	-0.17	0.150681	-0.13188	-0.387825052	0.255942692
2924	Q8NBS9	TXNDC5	thioredoxin domain containing 5	81567	ENSG00000239264	2	-0.12	-0.52	-0.31	-0.32	0.166	-0.31	0.2738	-0.157	0.0064	0.4517	-0.549	-0.57	-0.18	0.553	0.246	-0.14	-0.23	-0.5	0.734484	-0.19286	-0.145297873	-0.047566721
20	A0A075B7B	IGHV3OR1	immunoglobulin heavy variable 3/OR16-12 (	28304	ENSG00000270467	2	-1.21	-0.92	-0.76	-1.04	-1.11	-0.63	-0.627	-1.212	-0.142	-0.505	-0.828	-1.258	-1.04	-0.33	2.138	2.33	0.444	0.941	0.073815	-1.69249	-0.183807274	-1.508679743
1812	P30481	HLA-B	major histocompatibility complex, class I, B	3106	ENSG00000224608	8				-0.09	-0.15	-0.33				-0.135	-0.018	0.0288				1.8	-0.48	0.117	0.631429	-0.66827	-0.147558461	-0.520714266
951	K7EPA6	TXNL4A	thioredoxin like 4A	10907	ENSG00000141759	2	-0.08	0.219	0.061	-0.36	-0.06	-0.27	0.097	0.1174	0.1956	0.0086	-0.229	-0.251	-0.04	-0.12	-0.28	-0.34	-0.37	-0.13	0.336862	0.131148	-0.070093162	0.201240666
387	A6ZKI3	RTL8C	retrotransposon Gag like 8C	8933	ENSG00000134590	2	0.057	0.341	-0.61	-0.1	-0.04	0.014	0.2276	-0.218	0.0147	-0.131	-0.035	0.2048	-0.12	-0.54	-0.41	0.054	-0.27	-0.21	0.338908	0.193691	-0.066015905	0.259706491
926	J3KSH8	JPT1	Jupiter microtubule associated homolog 1	51155	ENSG00000189159	3	0.15	-0.15	-0.14	-0.47	-0.6	0.187	0.4624	0.0293	0.1079	-0.896	-0.906	-0.906	-0.07	-0.17	0.254	3.257	0.237	0.118	0.30927	-0.77498	0.180739618	-0.955715224
854	H3BQX5	SULT1A1	sulfotransferase family 1A member 1	6817	ENSG00000196502	2	-0.52	0.188	0.326	-0.4	-0.5	0.589	0.0114	-0.205	0.0097	-0.251	0.0051	0.1496	-0.55	0.083	0.716	0.416	-0.5	0.004	0.953	-0.08016	-0.005237723	-0.07492002
2883	Q8N0V5	GCNT2	glucosaminyl (N-acetyl) transferase 2 (I bloc	2651	ENSG00000111846	6	1.99	-0.43	-0.52	-0.42	0.266	-0.17	-0.219	0.5526	0.2926	-0.047	0.5918	-0.811	-0.5	-0.66	-0.49	-0.67	-0.26	-0.58	0.340687	0.648621	0.060843531	0.587777725
895	H9KV50	SELENON	selenoprotein N	57190	ENSG00000162430	1	0.03	0.031	-0.1	-0.25	0.535	-0.14	0.1892	-0.082	0.1974	0.0065	0.1075	0.2234	0.015	0.259	0.216	0.406	0.47	0.228	0.266191	-0.24831	-0.089773823	-0.158537649
3766	Q9Y5J5	PHLDA3	pleckstrin homology like domain family A me	23612	ENSG00000174307	1				0.337	0.181	0.047				0.0869	0.003	0.4186				-0.24	-0.09	-0.08	0.219778	0.325578	0.018848311	0.306729689
3021	Q8WWY7	WFDC12	WAP four-disulfide core domain 12	128488	ENSG00000168703	1				-0.49	-0.54	0.238				-0.761	0.4969	-0.12				0.097	0.008	0.118	0.762463	-0.34024	-0.137810633	-0.202432752
511	D6RF88	CPE	carboxypeptidase E	1363	ENSG00000109472	13	0.075	-0.22	-0.02	0.168	0.063	0.198	0.032	0.0705	0.1174	0.093	0.1448	-0.008	-0.39	0.187	0.011	-0.18	-0.07	0.375	0.799365	0.055892	-0.030061541	0.08595328
1431	P05107	ITGB2	integrin subunit beta 2	3689	ENSG00000160255	4				-0.59	-0.39	0.639				0.0896	-0.69	-0.775				1.983	0.332	0.649	0.230189	-1.10053	0.346307111	-1.446832937
957	K7ELG9	LSM12	LSM12 homolog	124801	ENSG00000161654	1	0.134	0.039	-0.15	0.162	0.181	-0.21	-0.13	0.0749	-0.036	0.2218	0.0089	0.2373	-0.17	-0.71	-0.3	-0.49	-0.07	0.047	0.126592	0.306429	-0.036776208	0.343205326
3253	Q9BRQ8	AIFM2	apoptosis inducing factor mitochondria assc	84883	ENSG00000042286	1				0.416	-0.12	-0.7				0.0073	-0.584	-0.445				-0.61	-0.52	-0.48	0.59838	0.403412	0.20458975	0.198822371
2352	Q0VDF9	HSPA14	heat shock protein family A (Hsp70) membe	51182	ENSG00000187522	1				0.195	0.31	0.178				0.0254	-0.224	0.2111				-0.52	-0.08	0.188	0.421608	0.366592	0.223486607	0.143105467
678	E9PMR6	ARHGEF1	Rho guanine nucleotide exchange factor 12	23365	ENSG00000196914	2	0.112	0.205	0.054	-0.29	-0.09	-0.46	0.1771	0.3345	0.3406	-0.232	-0.244	-0.023	-0	-0.53	0.15	-0.79	-0.38	-0.15	0.311842	0.203834	-0.137459838	0.341293583
611	E7ETY4	MARK2	microtubule affinity regulating kinase 2	2011	ENSG00000072518	1	-0.02	0.226	0.125	0.089	0.06	-0.18	0.4934	0.2142	0.2072	0.258	0.1083	0.0641	0.182	-0.24	-0.05	-0.26	-0.09	0.187	0.149956	0.096537	-0.174173676	0.270710317
2597	Q53RD9	FBLN7	fibulin 7	129804	ENSG00000144152	3	0.007	-0.25	-0.19	-0.12	-0.03	-0.05	0.0929	0.0482	0.0825	-0.451	0.1559	-0.148	0.058	-0.19	-0.03	-0.04	0.351	-0.16	0.725701	-0.10371	-0.07017378	-0.033538765
1312	O95833	CLIC3	chloride intracellular channel 3	9022	ENSG00000169583	1				0.544	0.332	0.272				1.016	-0.277	0.0791				-0.3	-0.42	-0.34	0.269599	0.735741	0.109979923	0.625760918
857	H3BRL3	UBFD1	ubiquitin family domain containing 1	56061	ENSG00000103353	2				-0.14	-0.01	-0.21				0.1943	-0.081	0.1247				0.632	0.296	0.405	0.089526	-0.56477	-0.199336611	-0.365431036
646	E9PGC0	RASA1	RAS p21 protein activator 1	5921	ENSG00000145715	2				0.293	0.619	-0.05				0.277	0.263	-0.011				0.334	0.302	0.472	0.702413	-0.08078	0.11216807	-0.192950286
1535	P0DJI9	SAA2	serum amyloid A2	6289	ENSG00000134339	3				-0.29	0.691	-0.08				-0.064	0.2326	-0.011				0.821	0.104	0.484	0.540344	-0.36178	0.055387218	-0.417171579
790	G5E9F8	PROS1	protein S	5627	ENSG00000184500	1				0.157	0.633	-0.11				0.2419	-0.145	-0.043				-0.1	0.46	0.409	0.721512	-0.02731	0.209353125	-0.236663517
862	H3BTA2	PPP4C	protein phosphatase 4 catalytic subunit	5531	ENSG00000149923	4	-0.05	0.301	0.023	0.112	0.246	-0.19	0.1862	0.1127	0.0727	0.2866	0.0896	0.1076	-0.13	-0.06	-0.04	0.032	-0.12	-0.06	0.146637	0.134918	-0.069344506	0.204262779
1291	O95473	SYNGR4	synaptogyrin 4	23546	ENSG00000105467	1				1.14	1.194	0.278				-0.06	0.4247	0.1703				-0.03	-0.15	-0.08	0.14465	0.957275	0.692251028	0.265024132
3613	Q9UHD9	UBQLN2	ubiquilin 2	29978	ENSG00000188021	2				-0.31	0.14	-0.39				0.1546	-0.269	-0.2				0.243	-0.09	-0.11	0.727627	-0.19954	-0.08178399	-0.11775931
2564	Q66S35	PFKFB4	6-phosphofructo-2-kinase/fructose-2,6-biph	5210	ENSG00000114268	3				0.069	0.541	-0.35				0.1106	-0.228	-0.042				-0.53	0.026	0.126	0.83076	0.212888	0.139332499	0.073555461
1437	P05362	ICAM1	intercellular adhesion molecule 1	3383	ENSG00000090339	2				-0.73	-0.29	-0.35				-0.329	-0.698	-0.491				2.33	-0.3	0.529	0.286204	-1.30968	0.050474922	-1.360150735
126	A0A088AW	WDR20	WD repeat domain 20	91833	ENSG00000140153	2	0.039	0.363	-0.11	-0	0.049	-0.47	-0.022	0.1495	-0.101	0.0908	-0.327	0.2749	-0.12	-0.59	-0	-0.62	-0.34	-0.08	0.244624	0.270122	-0.032879793	0.303001778
3053	Q92859	NEO1	neogenin 1	4756	ENSG00000067141	1				-0.02	0.237	0.088				-0.46	0.6183	0.0455				0.685	0.221	0.206	0.674633	-0.26859	0.03418213	-0.302770248
1559	P11274	BCR	BCR activator of RhoGEF and GTPase;BCI	613	ENSG00000186716	1				-0.07	-0.03	0.052				-0.193	-0.123	-0.01				0.004	-0.23	-0.13	0.525914	0.105239	0.094288109	0.010950442
2734	Q6UXI9	NPNT	nephronectin	255743	ENSG00000168743	30	0.253	0.424	-0.36	0.254	0.579	-0.39	-0.385	-0.382	-0.243	0.031	-0.161	-0.19	-0.49	-0.48	-0.21	-0.61	-0.82	-0.14	0.103631	0.584237	0.347325262	0.236911941
225	A0A0G2JRC	MUC4	mucin 4, cell surface associated	4585	ENSG00000278468	2	-0.67	-0.22	-0.27	-0.61	-0.43	-0.1	-0.292	-0.645	-0.288	0.0216	-0.317	-0.417	-0.58	-0.17	0.742	0.944	-0.12	1.155	0.139292	-0.70999	-0.060130682	-0.649856912
773	G3V171	TPTE2	transmembrane phosphoinositide 3-phospha	93492	ENSG00000132958	1				0.466	2.146	-0.46				-0.514	-1.198	-1.099				-0.28	-0.63	-0.4	0.241332	1.155247	1.655698111	-0.500451416
871	H7BXI7	LYPD6B	LY6/PLAUR domain containing 6B	130576	ENSG00000150556	1	-0.21	-0.15	0.126	-0.07	-0.87	0.374	0.3501	0.06	0.123	-0.371	0.706	-0.095	0.046	0.152	0.258	0.24	-0.34	-0.19	0.587118	-0.16213	-0.263833094	0.101703006
3533	Q9NXC5	MIOS	meiosis regulator for oocyte development	54468	ENSG00000164654	3				-0.12	0.233	-0.18				-0.031	-0.453	-0.163				-0.13	0.008	-0.11	0.606752	0.056202	0.192513745	-0.136311865
588	E5RJB8	SDC2	syndecan 2	6383	ENSG00000169439	2	0.095	-0.45	-0.19	-0.82	0.082	0.145	0.2604	0.4238	-0.192	-0.343	0.6992	-0.379	0.211	0.155	0.339	-0.28	0.328	0.252	0.401548	-0.35686	-0.267998002	-0.088862819
105	A0A087X1E	ARFIP2	ADP ribosylation factor interacting protein 2	23647	ENSG00000132254	2	0.05	-0.43	-0.14	0.342	0.065	-0.28	-0.144	-0.146	-0.396	0.3279	-0.205	0.229	-0.02	-0.3	-0.13	-0.83	-0.54	0.042	0.470261	0.229471	-0.010324125	0.239794934
214	A0A0D9SG8	CFH	complement factor H	3075	ENSG00000000971	2	-0.23	-0.03	-0.35	-0.46	-0.23	0.03	0.0249	-0.074	-0.01	-0.366	0.1188	-0.154	-0.3	-0.18	0.682	1.83	0.237	0.152	0.228211	-0.61658	-0.136643195	-0.479940206
1386	P02747	C1QC	complement C1q C chain	714	ENSG00000159189	1				-0.58	0.099	-0.09				-0.336	-0.262	-0.606				2.143	0.039	0.129	0.34231	-0.96158	0.210363102	-1.171944983
772	G3V153	CAPRIN1	cell cycle associated protein 1	4076	ENSG00000135387	3	0.502	0.128	0.084	-0.06	0.252	0.012	0.1692	0.1582	0.14	0.1747	-0.004	0.3129	-0.01	-0.59	-0.01	-0.23	0.036	0.079	0.161905	0.272104	-0.004966526	0.277070564
251	A0A0U1RQ	PRSS22	serine protease 22	64063	ENSG00000282937	3	-0.04	0.354	-0.1	-0.19	-0.29	-0.05	0.1254	0.4425	0.2884	-0.337	-0.075	-0.077	0.185	-0.54	0.136	0.656	0.07	-0.06	0.809704	-0.12895	-0.115972452	-0.012979509
840	H0YNP1	GLCE	glucuronic acid epimerase	26035	ENSG00000138604	1	-0.13	0.095	-0.23	-0.36	0.427	-0.4	0.8517	0.4196	0.3033	-0.127	0.2483	-0.032	-0.08	0.87	0.141	-0.13	-0.01	-0.07	0.356369	-0.2205	-0.377005112	0.156501997
1027	O00628	PEX7	peroxisomal biogenesis factor 7	5191	ENSG00000112357	2	-0.21	0.379	-0.46	-0.21	0.397	-0.56	-0.066	-0.399	0.1905	-0.201	0.0056	-0.02	0.009	-0.39	-0.12	0.177	-0.15	-0.01	0.986143	-0.02818	-0.029082178	0.000898036
2754	Q6ZWL3	CYP4V2	cytochrome P450 family 4 subfamily V mem	285440	ENSG00000145476	1	-0.26	-1.28	-0.88	-0.91	-1.1	0.583	-0.535	-0.169	0.9345	-2.552	-1.271	-1.565	-0.84	0.649	1.348	2.495	0.488	0.391	0.147596	-1.39632	0.218910798	-1.615235776
628	E9PB39	ARHGEF1	Rho guanine nucleotide exchange factor 10	9639	ENSG00000104728	1				0.624	0.484	-0.06				0.4425	0.21	-0.316				-0.44	-0.01	0.288	0.598865	0.403457	0.235911878	0.167544695
3252	Q9BRP8	PYM1	PYM homolog 1, exon junction complex ass	84305	ENSG00000170473	1				-0.12	0.148	-0.24				-0.071	0.014	0.0711				-0.65	-0.04	0.094	0.746577	0.13	-0.075994555	0.205994583
2750	Q6ZSS7	MFSD6	major facilitator superfamily domain containi	54842	ENSG00000151690	3	0.02	0.449	0.296	-0.38	0.296	-0.37	0.0846	0.1165	0.2793	0.4068	-0.258	0.0338	0.302	-0.23	0.236	-0.12	-0.18	0.025	0.873961	0.045657	-0.058662673	0.104319766
989	O00160	MYO1F	myosin IF	4542	ENSG00000142347	9				-0.31	-0.86	-0.29				-0.145	-0.33	-0.584				2.464	-0.19	0.467	0.30521	-1.40017	-0.132978871	-1.267186905
2033	P51116	FXR2	FMR1 autosomal homolog 2	9513	ENSG00000129245	3	0.501	0.256	-0	-0.08	-0.02	-0.17	-0.05	0.2204	0.1611	-0.441	-0.124	-0.176	0.152	-0.93	-0.14	-0.12	-0.24	-0.05	0.390607	0.303233	0.1480129	0.155219653
1411	P04090	RLN2	relaxin 2	6019;6013	ENSG00000107014	1				-1.19	-1.4	3.086				-0.637	-0.374	1.8553				-1.36	0.804	-1	0.898318	0.679516	-0.118561566	0.798077663
3063	Q92995	USP13	ubiquitin specific peptidase 13	8975	ENSG00000058056	3				0.129	-0.07	0.06				0.1891	-0.456	0.2109				0.607	0.28	0.534	0.233463	-0.43234	0.059768189	-0.492111375
1698	P20849	COL9A1	collagen type IX alpha 1 chain	1297	ENSG00000112280	2	0.53	-1.04	-0.58	0.082	0.435	-0.29	-0.294	-0.606	-0.72	0.067	-0.206	0.6059	-0.34	-0.46	-0.38	-0.68	-0.22	0.055	0.833796	0.196337	0.049017182	0.147320141
1945	P46734	MAP2K3	mitogen-activated protein kinase kinase 3	5606	ENSG00000034152	6				-0.45	0.059	-0.4				0.0195	-0.063	0.0392				-0.14	-0.01	0.292	0.380651	-0.31137	-0.262860583	-0.048510702
397	C9JN83	NAA10	N(alpha)-acetyltransferase 10, NatA catalyti	8260	ENSG00000102030	2				-0.24	-0.11	-0.53				-0.202	-0.447	-0.11				-0.3	-0.27	0.059	0.831208	-0.12207	-0.041814208	-0.080255672
506	C9JBG0	RAB34	RAB34, member RAS oncogene family	83871	ENSG00000109113	4	-0.09	-0.12	-0.17	-0.23	-0.6	-0.31	-0.24	-0.101	-0.598	-0.572	-0.54	-0.15	-0.53	-0.55	0.407	0.163	0.041	-0.74	0.763007	-0.05092	0.114257673	-0.165175443
2087	P54289	CACNA2D	calcium voltage-gated channel auxiliary subu	781	ENSG00000153956	43	0.382	0.354	-0.35	-0.86	0.31	0.223	0.397	0.257	0.0066	0.1271	-0.266	-0.213	-0.41	-0.76	-0	-0.76	0.425	0.405	0.745822	0.194172	-0.040652274	0.234824185
3362	Q9H1U4	MEGF9	multiple EGF like domains 9	1955	ENSG00000106780	2	0.066	-0.2	-0.11	0.067	0.631	-0.24	-0.11	-0.032	-0.047	0.6808	0.0778	0.2132	-0.4	-0	-0.25	-0.23	-0.02	0.357	0.579727	0.127789	-0.095023093	0.222811821
118	A0A087X29	WDR6	WD repeat domain 6	11180	ENSG00000178252	3	-0.04	0.445	0.354	0.059	-0.31	-0.52	0.2514	-0.055	0.3545	0.072	-0.282	-0.174	-0.07	-0.17	-0.23	-0.05	-0.15	-0.08	0.687691	0.124139	-0.028307655	0.152446652
250	Q9HCP0	CSNK1G1	casein kinase 1 gamma 1	53944	ENSG00000169118	7	-0.86	-1.04	-0.64	0.167	-0.06	-0.13	-0.789	-0.731	-0.269	-0.895	-0.533	-0.204	-0.67	0.104	-0.34	-0.56	-0.86	-0.53	0.867219	0.050266	0.144597058	-0.094330816
663	E9PK26	SNX15	sorting nexin 15	29907	ENSG00000110025	1				-0.14	-0.28	-0.41				0.189	0.0101	0.1339				-0.15	-0.15	0.18	0.170611	-0.23553	-0.387257167	0.151731231
2575	Q32MZ4	LRRFIP1	LRR binding FLII interacting protein 1	9208	ENSG00000124831	5				-0.26	0.424	-0.04				0.1435	-0.306	0.3098				0.096	0.173	0.229	0.886682	-0.12518	-0.008292605	-0.116890227
203	A0A0C4DH3	IGHV3-35	immunoglobulin heavy variable 3-35 (non-fu	28432	ENSG00000211957	1				-0.36	-0.53	-0.47				-0.632	-0.571	-0.884				1.626	-0.06	0.4	0.157061	-1.10928	0.242319852	-1.351598229
2355	Q10471	GALNT2	polypeptide N-acetylgalactosaminyltransfera	2590	ENSG00000143641	4	0.024	-0.29	-0.23	-0.15	-0.49	0.465	0.2979	0.0046	0.3172	-0.703	0.1929	0.0257	-0.21	0.719	0.023	0.5	0.293	-0.31	0.586328	-0.2808	-0.133382088	-0.147416199
3615	Q9UHG2	PCSK1N	proprotein convertase subtilisin/kexin type 1	27344	ENSG00000102109	2				-0.3	-0.01	-0.17				0.146	0.3477	-0.237				-0.07	-0.36	-0.22	0.405381	0.057145	-0.244613267	0.301758237
684	E9PPG9	RAE1	ribonucleic acid export 1	8480	ENSG00000101146	2	0.417	0.181	0.146	0.363	0.345	-0.24	-0.088	0.009	0.2832	0.2559	-0.023	-0.269	0.118	0.043	-0.08	-0.27	0.005	0.037	0.311277	0.227626	0.173684374	0.053942
1732	P23468	PTPRD	protein tyrosine phosphatase, receptor type	5789	ENSG00000153707	17				-0.62	-0.78	-0.34				-0.399	-0.423	-0.161				0.025	0.226	-0.29	0.171742	-0.56771	-0.252162829	-0.315546306

142	A0A0A0MRE	FHIT	fragile histidine triad	2272	ENSG00000189283	1	-0.06	0.222	0.219	-0.37	0.098	-0.52	0.041	0.029	0.0243	0.1202	-0.673	0.0022	0.262	-0.75	-0.29	-0.69	-0.7	-0.3	0.313685	0.343411	0.008005508	0.335405087
690	F2Z2S2	FRYL	FRY like transcription coactivator	285527	ENSG00000075539	2	-0.28	-0.22	-0.28	-0.47	0.171	-0.23	-0.312	0.1636	-0.023	0.1511	-0.448	-0.385	-0.7	-0.07	-0.04	-0.6	0.012	0.537	0.935289	-0.07231	-0.074948125	0.002641683
3130	Q96FQ6	S100A16	S100 calcium binding protein A16	140576	ENSG00000188643	2				-0.05	-0.26	-0.65				-0.216	-0.353	0.4766				-0	-0.35	-0.11	0.697482	-0.16568	-0.289710362	0.1240285
259	A0A140T9G	HLA-DPA1	major histocompatibility complex, class II, D	3113	ENSG00000206291	1				-0.22	-0.39	0.079				0.2163	0.0476	-0.463				1.629	0.145	0.365	0.309172	-0.88814	-0.108552451	-0.77959234
260	A0A140T93	HLA-C	major histocompatibility complex, class I, C	3107	ENSG00000237022	8	0.078	0.134	-0.15	-0.66	0.044	-0.48	1.1279	-0.245	0.9915	0.036	-0.662	-0.511	0.734	-0.06	0.123	0.476	0.306	-0.3	0.577101	-0.38501	-0.29457117	-0.090437167
1694	P20594	NPR2	natriuretic peptide receptor 2	4882	ENSG00000159899	1				0.168	0.168	-0.45				0.0501	-0.092	0.0517				-0.24	-0.92	-0.06	0.480202	0.369459	-0.040133925	0.409592935
1047	O14772	FPGT	fucose-1-phosphate guanylyltransferase	8790	ENSG00000254685	6	-0.07	-0.33	-0.35	-0.15	-0.13	-0.07	-0.095	-0.108	-0.186	0.3473	0.335	0.27	-0.22	0.442	-0.2	0.378	0.15	0.039	0.214616	-0.28198	-0.277960548	-0.004021816
804	H0Y650	DNAAF5	dynein axonemal assembly factor 5	54919	ENSG00000164818	3	9E-04	0.077	-0.28	-0.04	-0.09	-0.24	-0.022	0.1586	0.1821	0.3327	-0.53	-0.089	-0.44	-0.89	-0.25	-0.15	-0.09	-0.03	0.297613	0.21406	-0.100048356	0.314107997
3027	Q8WZA1	POMGNT1	protein O-linked mannose N-acetylglucosam	55624	ENSG00000085998	1				-0.23	-0.11	-0.07				0.3299	0.0552	0.2109				-0.17	-0.08	0.076	0.146775	-0.077	-0.335233176	0.258235806
2330	Q07065	CKAP4	cytoskeleton associated protein 4	10970	ENSG00000136026	3	-0.04	-0.47	-0.22	-0.73	0.196	0.071	-0.007	0.0725	-0.156	-0.552	0.2939	0.2324	0.277	0.981	0.06	0.084	0.553	0.076	0.170593	-0.53599	-0.178098446	-0.357888694
3051	Q92828	CORO2A	coronin 2A	7464	ENSG00000106789	1				0.15	0.337	-0.2				0.4819	-0.492	-0.052				0.135	-0.48	0.077	0.891148	0.18427	0.117339342	0.066931142
1148	O60245	PCDH7	protocadherin 7	5099	ENSG00000169851	2	0.288	-0.25	0.46	-0.27	0.123	0.675	0.2987	0.1164	0.1124	-0.106	0.5451	0.2878	0.133	0.233	0.074	-0.07	0.487	-0.27	0.870437	0.073641	-0.037185473	0.110826857
2331	Q07075	ENPEP	glutamyl aminopeptidase	2028	ENSG00000138792	2				0.757	-0.25	0.604				1.1912	-0.394	0.6497				-1.03	-0.08	-0.5	0.324792	0.906763	-0.112411201	1.019174079
1676	P19634	SLC9A1	solute carrier family 9 member A1	6548	ENSG00000090020	1				-0.34	0.36	-0.43				0.2799	-0.194	0.1084				0.016	-0.29	-0.31	0.692929	0.059926	-0.201482214	0.261408587
920	J3KQ72	FBXO6	F-box protein 6	26270	ENSG00000116663	2				0.204	0.055	-1.23				-0.576	-0.425	-0.348				-0.88	-0.27	0.23	0.962269	-0.01647	0.126446631	-0.142921165
1	A0A024R0K	CEACAM5	CEA cell adhesion molecule 5;carcinoembry	1048	ENSG00000105388	3				-1.05	-0.68	-0.5				-0.021	-0.468	-0.609				2.942	0.992	0.402	0.147537	-2.1886	-0.377732561	-1.810867463
2279	Q00534	CDK6	cyclin dependent kinase 6	1021	ENSG00000105810	2				-0.07	-0.27	-0.1				-0.102	-0.017	-0.038				0.05	-0.07	-0.2	0.64917	-0.07194	-0.09476376	0.022821459
1144	O43866	CD5L	CD5 molecule like	922	ENSG00000073754	2				-0.72	-0.43	-0.79				-0.641	-0.595	-0.734				2.683	-0.14	0.442	0.230221	-1.64243	0.009157469	-1.651588683
657	E9PMI6	CLNS1A	chloride nucleotide-sensitive channel 1A	1207	ENSG00000074201	1				0.696	0.018	-0.05				0.2401	0.2324	0.2079				-0.02	-0.09	0.068	0.574281	0.236531	-0.005055933	0.24158713
3380	Q9H3R2	MUC13	mucin 13, cell surface associated	56667	ENSG00000173702	2	-0.45	0.754	0.795	0.409	-0.42	-1.28	-0.844	-0.09	0.7667	-0.041	-0.089	-0.245	-0.04	0.493	-0.14	0.792	0.63	-0.6	0.816491	-0.21914	0.059124491	-0.278267469
2746	Q6ZNJ1	NBEAL2	neurobeachin like 2	23218	ENSG00000160796	3	0.307	0.548	0.104	0.382	0.619	0.238	0.2641	0.0593	0.2839	0.6676	-0.079	0.1787	-0.21	-0.35	-0.2	0.388	0.492	0.498	0.480901	0.263454	0.137214736	0.126239533
405	B1AHC9	XRCC6	X-ray repair cross complementing 6	2547	ENSG00000196419	2	-0.04	-0.15	-0.01	-0.18	0.102	0.061	-0.022	-0.279	-0.271	-0.499	-0.163	-0.32	-0.23	-0.56	1.001	1.568	-0.22	0.303	0.310794	-0.34861	0.222128479	-0.570737693
580	E2QRK7	TJAP1	tight junction associated protein 1	93643	ENSG00000137221	1				-0.09	-0.28	-0.85				0.2119	-0.673	0.1826				-0.72	-0.34	0.04	0.762502	-0.06497	-0.313386785	0.248419446
753	Q5VU81	UBAP2L	ubiquitin associated protein 2 like	9898	ENSG00000143569	1				-0.43	-0.18	-0.57				-0.133	-0.244	0.1258				-0.56	-0.23	-0.01	0.468022	-0.12645	-0.309094563	0.182646096
812	H0Y9R3	WDR26	WD repeat domain 26	80232	ENSG00000162923	1				-0.33	-0.04	-0.06				-0.026	-0.588	-0.301				-0.17	-0.01	-0.07	0.548177	-0.06106	0.161751832	-0.222816802
12	A0A075B6I1	IGLV4-60	immunoglobulin lambda variable 4-60	28785	ENSG00000211639	1				-1.17	-1.61	-0.51				-0.946	-0.258	-1.128				0.897	-1.01	1.035	0.301973	-1.40273	-0.318839351	-1.083889008
2835	Q8IUE6	HIST2H2A	histone cluster 2 H2A family member b;H2A	317772	ENSG00000184270	4				-1.21	-1.06	1.095				-0.689	-0.105	-0.583				1.553	0.389	0.404	0.373085	-1.17528	0.065273866	-1.240552142
3405	Q9H7B4	SMYD3	SET and MYND domain containing 3	64754	ENSG00000185420	3	-0.13	-0.22	-0.3	-0.04	-0.05	-0.17	0.1206	0.1792	-0.128	0.1348	0.3675	-0.914	-0.15	-0.24	-0.08	-0.33	0.414	0.113	0.854075	-0.10666	-0.11372067	0.007061407
1505	P08572	COL4A2	collagen type IV alpha 2 chain	1284	ENSG00000134871	3	0.013	-0.38	0.224	-0.8	-0.65	0.416	-0.06	0.2633	0.1021	-0.88	-0.004	-0.673	-0.5	-0.31	-0.02	1.359	-0.73	0.638	0.763794	-0.26796	0.013068847	-0.281027514
289	A0A1W2PN	FLT1	fms related tyrosine kinase 1	2321	ENSG00000102755	1	-0.42	-0.22	-0.27				-0.487	-0.279	-0.274				-0.54	-0.71	0.344				0.988264	-8.6E-05	0.04418617	-0.044272304
2351	Q09MP3	RAD51AP2	RAD51 associated protein 2	729475	ENSG00000214842	2	0.257	-0.95	-0.67				0.4605	-0.159	-0.191				0.678	0.588	0.045				0.291575	-0.89204	-0.491821816	-0.400218529
819	H0YBT3	CNOT7	CCR4-NOT transcription complex subunit 7	29883	ENSG00000198791	1	0.517	-0	-0.28				0.3933	0.317	0.5288				-0.24	0.018	0.253				0.404946	0.068644	-0.333361967	0.402006061
1969	P48307	TFPI2	tissue factor pathway inhibitor 2	7980	ENSG00000105825	3	-0.31	-0.49	-0.24				0.0082	-0.026	-0.006				-0.54	0.774	-0.34				0.688894	-0.31084	-0.338721418	0.02788563
3375	Q9H2U9	ADAM7	ADAM metallopeptidase domain 7	8756	ENSG00000069206	34	0.057	-0.29	1.014				-0.222	0.4347	0.1162				0.449	-0.48	0.089				0.898499	0.24149	0.151915222	0.089575209
1825	P31751	AKT2	AKT serine/threonine kinase 2	208	ENSG00000105221	7	-0.06	0.077	-0				-0.022	-0.053	0.0173				-0.27	-0.64	-0.11				0.205341	0.34659	0.022879609	0.32371013
837	H0YMV8	RPS27L	ribosomal protein S27 like	51065	ENSG00000185088	2	0.086	0.246	-0.04				-0.107	0.0449	-0.044				-0.06	-0.42	-0.15				0.23552	0.308546	0.132060686	0.176484971
2611	Q5JRA6	MIA3	MIA SH3 domain ER export factor 3	375056	ENSG00000154305	2	0.034	-0.41	-0.06				1.1282	0.438	0.2765				-0.14	0.082	0.717				0.283006	-0.36847	-0.761548152	0.393080751
3357	Q9H1F0	WFDC10A	WAP four-disulfide core domain 10A	140832	ENSG00000180305	2	1.365	-0.99	1.4				-1.028	-0.877	-0.638				-1.13	-0.25	-0.85				0.294703	1.333585	1.437868873	-0.104284346
1274	O95239	KIF4A	kinesin family member 4A	24137	ENSG00000090889	2	-0.07	1.366	-0.08				0.0375	0.9035	0.3036				0.282	-0.36	-0.51				0.559725	0.601312	-0.009755526	0.61106744
440	B4DXW1	ACTR3	actin related protein 3;ARP3 actin related pr	10096	ENSG00000115091	25	0.351	0.435	0.025				0.1473	-0.064	-0.017				-0.38	-0.91	0.427				0.479543	0.556198	0.248430159	0.307768152
87	A0A087X0A	COL12A1	collagen type XII alpha 1 chain	1303	ENSG00000111799	4	-0.1	-0.34	0.949				0.2516	-0.386	-0.081				0.077	0.158	0.523				0.780954	-0.08486	0.239594154	-0.32445539
2284	Q00765	REEP5	receptor accessory protein 5	7905	ENSG00000129625	3	-0.01	0.182	0.247				-0.005	-0.372	-0.309				-0.42	-0.34	-0.15				0.143996	0.441447	0.36966521	0.071781719
944	K7EIL3	ICAM5	intercellular adhesion molecule 5	7087	ENSG00000105376	2	-0.03	-0.01	-0				0.0468	0.1141	-0.068				-0.27	-1.08	-0.11				0.301278	0.472122	-0.045560583	0.517682205
1796	P30039	PBLD	phenazine biosynthesis like protein domain c	64081	ENSG00000108187	2	0.134	-0.24	0.007				0.0406	-0.006	0.2183				0.024	-0.81	-0.07				0.505141	0.250711	-0.118282211	0.368993037
3479	Q9NRA2	SLC17A5	solute carrier family 17 member 5	26503	ENSG00000119899	2	-0	0.426	-0.03				-0.089	0.1734	0.3556				-0.26	-0.25	-0.36				0.192464	0.423388	-0.014236375	0.437624584
2619	Q5MNZ6	WDR45B	WD repeat domain 45B	56270	ENSG00000141580	2	-0	-0.2	-0.2				-0.09	0.0706	0.0344				-0.25	-0.52	-0.2				0.17827	0.191328	-0.139671898	0.330999681
1534	P0DJI8	SAA1	serum amyloid A1	6288	ENSG00000173432	3	-0.15	-0.76	-0.06				-0.21	0.0731	-0.226				-0.07	0.429	0.714				0.244926	-0.68035	-0.200950305	-0.479396748
3598	Q9UG63	ABCF2;LO	ATP binding cassette subfamily F member 2	10061;11448	ENSG00000033050	2	-0.01	0.169	0.055				0.0626	-0.017	0.2221				-0.13	-0.42	-0.27				0.121509	0.346167	-0.017869978	0.364036626
3603	Q9UGT4	SUSD2	sushi domain containing 2	56241	ENSG00000099994	2	-0.2	0.059	-0.5				-0.002	0.0064	-0.076				0.277	0.373	0.593				0.119332	-0.62801	-0.189443125	-0.43856711
119	A0A087X2B	NRCAM	neuronal cell adhesion molecule	4897	ENSG00000091129	2	-0.1	-0.64	-0.23				0.1713	-0.087	0.046				-0.24	0.324	-0.32				0.460071	-0.24892	-0.368332023	0.119408585
2413	Q13496	MTM1	myotubularin 1	4534	ENSG00000171100	2	-0.36	-0.14	-0.33				-0.068	-0.366	-0.22				-0.58	0.077	-0.24				0.965895	-0.0283	-0.058196209	0.029899831
855	H3BR56	BMERB1	bMERB domain containing 1	89927	ENSG00000166780	2	0.561	-0.37	-0.39				-0.18	-0.075	0.2576				-0.65	-0.75	-0.17				0.431942	0.45358	-0.069215255	0.52279495
1165	O60565	GREM1	gremlin 1, DAN family BMP antagonist	26585	ENSG00000282046	2	-0.86	-1.69	0.047				-0.935	-1.247	1.9822				0.327	-0.05	-1.3				0.833299	-0.49581	-0.769279443	0.273473103
3250	Q9BRK5	SDF4	stromal cell derived factor 4	51150	ENSG00000078808	22	-0.07	-0.6	-0.16				-0.103	0.059	-0.332				0.117	0.937	0.153				0.239842	-0.67964	-0.152114616	-0.527525501
1565	P11926	ODC1	ornithine decarboxylase 1	4953	ENSG00000115758	2	0.003	0.085	-0.05				0.1611	0.2256	-0.125				-0.05	-0.47	-0.1				0.338548	0.220745	-0.073182718	0.293928186
1629	P15529	CD46	CD46 molecule	4179	ENSG00000117335	6	0.379	0.538	0.385				-0.109	0.2629	0.253				0.101	-0.85	-0.08				0.226995	0.711145	0.298348444	0.412796249
2985	Q8TDW5	SYTL5	synaptotagmin like 5	94122	ENSG00000147041	4	-0.71	-0.5	-0.01				0.7234	0.0981	-0.042				0.057	-0.29	-0.4				0.270192	-0.19393	-0.666435419	0.472503598
2808	Q86UN2	RTN4RL1	reticulon 4 receptor like 1	146760	ENSG00000185924	2	0.108	-0.07	-0.33				0.2019	0.2796	0.3574				-0.12	-0.17	0.409				0.359251	-0.13647	-0.37675341	0.240283924
2826	Q86XT2	VPS37D	VPS37D, ESCRT-I subunit;VPS37D subun	155382	ENSG00000176428	3	-0.22	-0.12	-0.39				-0.566	-0.06	0.0746				-0.53	0.112	-0.3				0.972571	-0.00342	-0.05917493	0.055755171
3731	Q9Y333	LSM2	LSM2 homolog, U6 small nuclear RNA and	57819	ENSG00000204392	1	-0.62	-0.34	-0.58				-0.522	-0.704	-0.344				-0.55	-0.62	-0.41				0.993393	0.014887	0.01265795	0.002229104
780	G3V4D9	NEMF	nuclear export mediator factor	9147	ENSG00000165525	2	0.261	-0.07	0.295				0.1149	0.2576	0.1883				-0.05	-0.21	-0.21				0.152747	0.318503	-0.025378677	0.343881637
400	H3BPC4	UBE2I	ubiquitin conjugating enzyme E2 I	7329	ENSG00000103275	1	-0	0.35	0.075				0.1584	0.1036	-0.044				0.088	0.11	0.071				0.86441	0.050456	0.067371193	-0.016915372
1146	O43933	PEX1	peroxisomal biogenesis factor 1	5189	ENSG00000127980	4	-0.25	0.159	-0.12				-0.043	-0.41	-0.327				-0.44	-1.01	-0.48				0.205396	0.572959	0.188659406	0.38429916
2889	Q8N1A6	C4orf33	chromosome 4 open reading frame 33	132321	ENSG00000151470	1	0.6	-0.03	0.031				0.5848	-0.636	0.7755				0.387	-0.38	-0.02				0.891639	0.205014	-0.040928586	0.245942995
873	H7BY56	SYNJ2	synaptojanin 2	8871	ENSG00000078269	1	0.095	-0.17	-0.18				0.2619	-0.226	0.0615				-0.26	-0.33	-0.17				0.373997	0.163065	-0.119503014	0.282567548
718	F5H619	HEATR5A	HEAT repeat containing 5A	25938	ENSG00000129493	3	0.132	0.205	-0.12				0.1356	0.0002	0.3088				-0.35	-0.64	-0.32				0.102354	0.50638	-0.075258181	0.581638468
2526	Q15555	MAPRE2	microtubule associated protein RP/EB famil	10982	ENSG00000166974	4	0.383	0.111	-0.1				-0.227	0.0036	-0.192				-0.16	-0.53	-0.2				0.228064	0.427348	0.269131643	0.158216829
1363	P01714	IGLV3-19	immunoglobulin lambda variable 3-19	28797	ENSG00000211663	1	-1.12	-1.62	-1.08				-1.115	-0.584	-0.746				-0.7	-0.37	2.584				0.343366	-1.77607	-0.457118491	-1.318948895
3746	Q9Y3L5	RAP2C	RAP2C, member of RAS oncogene family	57826	ENSG00000123728	4	0.035	0.646	0.007				-0.103	-0.046	0.2197				-0.16	0.058	-0.09				0.520805	0.293302	0.205628316	0.087673736
1367	P01766	IGHV3-13	immunoglobulin heavy variable 3-13	28449	ENSG00000282286	3	-0.49	-1.02	-0.54				0.0631	-0.217	-0.334				-0.15	6E-04	1.854				0.306014	-1.25207	-0.520471883	-0.73159538
2516	Q15366	PCBP2	poly(rC) binding protein 2	5094	ENSG00000197111	12	-1.03	-0.83	-1.06				-0.92	-1.1	-0.725				-1.15	-1.42	-0.97				0.415543	0.209147	-0.055509667	0.264656981
3605	Q9UH62	ARMCX3	armadillo repeat containing X-linked 3	51566	ENSG00000102401	2	-0.26	0.133	-0.42				0.3976	0.3176	0.0675				0.104	0.382	0.602				0.202734	-0.54372	-0.441755831	-0.101965902
835	H0YNP4	IRF9	interferon regulatory factor 9	10379	ENSG00000213928	1	0.208	-0.61	-0.31				-0.275	-0.249	-0.387				-0.52	-0.42	-0.7				0.50673	0.311815	0.066519956	0.24529473
911	J3KNI1	COG4	component of oligomeric golgi complex 4	25839	ENSG00000103051	2	0.249	-0.09	0.13				-0.004	0.1021	0.2846				-0.27	-0.09	-0.02				0.309175	0.221889	-0.030618546	0.252507633
209	A0A0D9SG7	UBE2A	ubiquitin conjugating enzyme E2 A	7319	ENSG00000077721	1	0.209	0.355	0.063				0.3225	0.1604	0.2577				0.071	-0.24	-0.05				0.172191	0.284407	-0.037581161	0.321988517
1051	O14828	SCAMP3	secretory carrier membrane protein 3	10067	ENSG00000116521	3	-0.17	-0.24	-0.01				-0.251	-0.122	-0.126				-0.38	-0.17	0.007				0.948352	0.043893	0.0293805	0.014512057
409	B1AKT3	TMED5	transmembrane p24 trafficking protein 5	50999	ENSG00000117500	1	0.604	-0.11	0.201				0.2213	0.4901	-0.451				0.111	0.555	0.428				0.773696	-0.13208	0.145701441	-0.277779683
748	H0YIB8	AC004551	2'-5'-oligoadenylate synthetase 1	4938	ENSG00000089127	1	0.402	-0.37	-0.13				-0.486	0.6245	0.0124				-0.54	-0.44	-0.11				0.617402	0.327972	-0.084657245	0.41262875
491	C9J3D7	CROT	carnitine O-octanoyltransferase	54677	ENSG00000005469	2	-0.14	-0.17	-0.77				0.2852	-0.461	-0.143				0.495	0.591	0.312				0.163361	-0.82828	-0.255791828	-0.572487086

1323	P00167	CYB5A	cytochrome b5 type A	1528	ENSG00000166347	1	-0.36	-0.52	-0.41	-0.291	-0.437	0.5703	-0.74	-1.13	-0.2	0.407774	0.257974	-0.377741257	0.635715107
493	C9J494	MKRN2	makorin ring finger protein 2	23609	ENSG00000075975	3	0.032	-0.06	-0.18	0.043	0.2248	0.0839	-0.05	-0.46	-0.18	0.209406	0.15761	-0.187908371	0.345518244
2278	Q00526	CDK3	cyclin dependent kinase 3	1018	ENSG00000250506	2	-0.14	-0.12	-0.29	0.2529	0.1163	0.0272	-0.11	-0.54	-0.18	0.163984	0.093572	-0.315610843	0.409182864
3165	Q96M27	PRRC1	proline rich coiled-coil 1	133619	ENSG00000164244	1	0.106	0.272	0.01	0.0131	-0.034	0.076	-0.1	-0.81	-0.3	0.192874	0.534186	0.110880995	0.423305263
1088	O15294	OGT	O-linked N-acetylglucosamine (GlcNAc) tra	8473	ENSG00000147162	1	-0.03	-0.25	-0.2	-0.008	0.0606	0.0726	-0.38	-0.44	-0.19	0.113053	0.177601	-0.201069825	0.378670901
660	E9PJJ9	TRIM68	tripartite motif containing 68	55128	ENSG00000167333	1	-0.16	0.075	-0.1	0.0364	-0.329	0.0667	-0.18	-0.67	-0.3	0.338659	0.319154	0.011676401	0.307477596
2079	P53675	CLTCL1	clathrin heavy chain like 1	8218	ENSG00000070371	24	-0.15	0.411	-0.11	-0.174	-0.158	0.0461	-0.39	-0.97	-0.32	0.220053	0.609965	0.145985791	0.463979539
2825	Q86XF0	DHFR2	dihydrofolate reductase 2	200895	ENSG00000178700	1	-0.38	0.672	-0.37	0.3308	-0.188	0.0686	0.226	0.897	-0.12	0.763895	-0.35732	-0.094608779	-0.262710912
1277	O95295	SNAPIN	SNAP associated protein	23557	ENSG00000143553	1	0.016	-0.05	-0.31	0.2021	-1E-03	0.0972	-0.23	-0.59	0.053	0.373852	0.141308	-0.21465964	0.355967603
805	H0Y6A0	ARFGAP3	ADP ribosylation factor GTPase activating p	26286	ENSG00000242247	7	0.131	0.006	-0.53	-0.339	-0.092	0.1666	-0.15	-0.47	-0.75	0.487355	0.325365	-0.042845739	0.368210934
45	A0A087WVI	AK6	adenylate kinase 6	102157402	ENSG00000085231	1	-0.18	0.231	-0.05	0.0696	0.0194	0.1185	0.05	-0.48	-0.03	0.597188	0.151965	-0.069522476	0.221487287
2004	P49765	VEGFB	vascular endothelial growth factor B	7423	ENSG00000173511	1	-0.41	-0.27	-0.58	0.0427	-0.009	-0.016	0.052	0.384	-0.05	0.111695	-0.54796	-0.42490549	-0.123053116
2460	Q14457	BECN1	beclin 1	8678	ENSG00000126581	2	0.114	0.345	0.044	0.2556	0.0261	0.109	-0.04	-0.51	-0.38	0.146939	0.47526	0.037251413	0.438008811
2343	Q08462	ADCY2	adenylate cyclase 2	108	ENSG00000078295	3	-0.19	0.032	0.245	0.1069	0.4282	0.0676	0.228	0.199	-0.16	0.734156	-0.05857	-0.17045324	0.111882143
2056	P52435	POLR2J	RNA polymerase II subunit J	5439	ENSG00000005075	1	0.095	0.432	0.221	-0.21	-0.018	0.265	-0.17	-0.59	-0.26	0.157539	0.590552	0.237012809	0.353538734
3499	Q9NS98	SEMA3G	semaphorin 3G	56920	ENSG00000010319	1	0.104	-0.25	-0.37	-0.375	-0.159	-0.123	-0.51	-0	-0.08	0.977321	0.027062	0.047124408	-0.020062322
195	E7EU35	PITPNC1	phosphatidylinositol transfer protein cytoplas	26207	ENSG00000154217	2	0.508	0.074	0.097	0.1996	-0.318	-0.223	-0.02	-0.51	0.307	0.563166	0.300848	0.340052284	-0.03920457
3155	Q96JT2	SLC45A3	solute carrier family 45 member 3	85414	ENSG00000158715	1	-0.53	0.074	-0.17	0.2533	-0.034	0.0065	-0.36	-0.16	-0.36	0.305238	0.085756	-0.284337643	0.3700935
370	A6NCS6	C2orf72	chromosome 2 open reading frame 72	257407	ENSG00000204128	3	-0.14	1.004	-0.28	0.3222	-0.27	0.1781	-0.37	-0.22	-0.58	0.479334	0.582606	0.117347921	0.465257774
2321	Q06124	PTPN11	protein tyrosine phosphatase non-receptor ty	5781	ENSG00000179295	25	-0.24	-0.04	-0.34	0.2761	-0.116	-0.001	-0.06	-0.07	-0.07	0.328243	-0.14317	-0.258936021	0.115766966
363	A2RUU4	CLPSL1	colipase like 1	340204	ENSG00000204140	1	-0.67	-0.23	0.635	-0.266	-0.469	-0.275	-0.68	0.735	-0.59	0.918678	0.091912	0.24894009	-0.157028327
3734	Q9Y397	ZDHHC9	zinc finger DHHC-type containing 9	51114	ENSG00000188706	1	0.101	0.622	0.35	0.2812	0.3083	0.5998	0.265	0.377	0.323	0.923138	0.036084	-0.038573061	0.074657381
3765	Q9Y5E9	PCDHB14	protocadherin beta 14	56122	ENSG00000120327	1	-0.45	0.293	0.183	-0.409	0.2291	0.8667	-0.31	-0.28	-0.06	0.634894	0.227452	-0.219145298	0.446597335
617	E7EVJ3	NDST1	N-deacetylase and N-sulfotransferase 1	3340	ENSG00000070614	2	0.291	-0.25	-0.5	0.4378	0.1628	0.2312	0.063	0.125	-0.16	0.361863	-0.1619	-0.430390482	0.268490459
2266	P81605	DCD	dermcidin	117159	ENSG00000161634	1	0.443	-1.03	0.34	0.1322	-0.754	-0.022	-0.36	0.26	-0.45	0.973857	0.099385	0.130844582	-0.031459947
2633	Q5T196	FLAD1	flavin adenine dinucleotide synthetase 1	80308	ENSG00000160688	2	0.157	0.226	-0.03	-0.117	0.0123	-0.109	0.137	-0.4	0.175	0.653113	0.149403	0.190005057	-0.040601794
1713	P22033	MMUT;MU	methylmalonyl-CoA mutase	4594	ENSG00000146085	1	-0.19	-0.67	-1.34	1.0238	-0.439	-0.202	1.315	1.016	-0.42	0.327649	-1.3672	-0.859491522	-0.507711803
3174	U3KQF5	NECAB3	N-terminal EF-hand calcium binding protein	63941	ENSG00000125967	1	-0.37	0.139	-0.37	-0.289	-0.466	-0.083	-0.06	-0.15	-0.31	0.88722	-0.0258	0.078494887	-0.104290992
826	H0YIA2	SDSL	serine dehydratase like	113675	ENSG00000139410	1	-0.27	-0.23	1.375	-0.287	-0.432	0.3925	-0.47	-0.63	0.242	0.690771	0.577975	0.399164389	0.178810855
439	B4E372	RCBTB2	RCC1 and BTB domain containing protein 2	1102	ENSG00000136161	1	0.159	0.12	0.021	-0.044	-0.081	0.2339	-0.39	-0.7	-0.22	0.125695	0.533654	0.063731153	0.469922371
959	K7EM09	TMEM205	transmembrane protein 205	374882	ENSG00000105518	1	-0.54	0.104	-0.2	-0.075	-0.726	-0.176	-0.43	-0.55	-0.31	0.762468	0.219053	0.11320281	0.105850324
2964	Q8TB03	CXorf38	chromosome X open reading frame 38	159013	ENSG00000185753	2	-0.01	0.535	0.017	0.5383	0.2884	0.1878	-0.1	-0.25	0.084	0.297089	0.26808	-0.157686306	0.425766394
490	C9K0R9	IMPDH1	inosine monophosphate dehydrogenase 1	3614	ENSG00000106348	5	-0.37	-0.02	-0.22	0.0256	-0.048	-0.026	-0.03	-0.46	0.108	0.673796	-0.07455	-0.18590047	0.111352926
571	D6REB0	CALCOCC	calcium binding and coiled-coil domain 2	10241	ENSG00000136436	3	0.226	0.163	0.097	0.6716	0.3924	0.5745	-0.5	-0.31	0.244	0.138421	0.349437	-0.38395118	0.73338832
1366	P01743	IGHV1-46	immunoglobulin heavy variable 1-46	28465	ENSG00000282131	2	-0.38	-0.01	-0.01	-0.006	0.1581	0.0374	-0.3	-0.17	0.647	0.800088	-0.19209	-0.196946153	0.00485476
814	H0YAF9	FST	follistatin	10468	ENSG00000134363	1	-0.55	-0.26	0.853	0.0835	1.0706	0.4477	-0.23	0.436	0.198	0.645567	-0.11819	-0.51838841	0.400197486
1371	P01817	IGHV2-5	immunoglobulin heavy variable 2-5	28457	ENSG00000211937	2	-1.66	-1.11	-1.18	-0.897	-0.117	-0.729	-1.01	-1.04	2.965	0.552518	-1.61601	-0.732051666	-0.883959914
3680	Q9UNT1	RABL2B	RAB, member of RAS oncogene family like	11158	ENSG00000079974	4	-0.34	-0.05	-0.3	0.1156	0.0399	0.0556	-0.4	-0.3	-0.08	0.164102	0.026495	-0.302866772	0.329361301
3102	Q96BY7	ATG2B	autophagy related 2B	55102	ENSG00000066739	3	-0.14	0.335	-0.28	0.339	-0.027	0.0705	0.249	0.102	-0.01	0.773464	-0.13986	-0.154683216	0.01482022
3120	Q96EC8	YIPF6	Yip1 domain family member 6	286451	ENSG00000181704	1	0.179	0.354	0.222	0.3554	-0.232	-0.103	-0.27	-0.61	-0.28	0.14607	0.638607	0.244955905	0.393651543
243	J3QT77	PON2	paraoxonase 2	5445	ENSG00000105854	1	0.357	0.226	0.303	0.1989	0.1859	0.5826	-0.14	-0.1	0.024	0.138769	0.368241	-0.027148828	0.395390303
448	H0YMH9	DUOXA1	dual oxidase maturation factor 1	90527	ENSG00000140254	1	-0.63	-0.07	0.226	-0.415	0.2849	-0.034	-0.4	-0.08	-0.73	0.662346	0.243383	-0.104299578	0.347682975
1994	P49411	TUFM	Tu translation elongation factor, mitochondri	7284	ENSG00000178952	1	0.185	-0.4	0.336	0.0667	-0.048	0.09	0.278	0.616	0.32	0.356532	-0.36541	0.003128247	-0.368534507
11	A0A075B6H	IGLV4-69	immunoglobulin lambda variable 4-69	28784	ENSG00000211637	1	-0.29	-1.29	-0.29	-0.319	-0.071	-0.395	-0.63	-0.16	2.151	0.55262	-1.08	-0.362493902	-0.717501968
2790	Q7Z5L0	VMO1	vitelline membrane outer layer 1 homolog	284013	ENSG00000182853	1	-0.57	-1.34	-0.36	0.4187	-0.333	-0.005	-0.61	0.074	0.282	0.30526	-0.674	-0.785072659	0.111074351
722	F6QUH3	AFAP1L2	actin filament associated protein 1 like 2	84632	ENSG00000169129	1	-0.35	0.612	-0.25	-0.043	0.2389	0.2918	-0.19	-0.17	0.203	0.824374	0.057344	-0.159081088	0.216425023
927	J3KSS7	GGA3	golgi associated, gamma adaptin ear contair	23163	ENSG00000125447	2	0.102	0.355	-0.07	0.0405	-0.098	0.0159	0.163	-0.47	0.04	0.667771	0.216512	0.142603292	0.073909101
1467	P07305	H1-0;H1F0	H1.0 linker histone;H1 histone family memb	3005	ENSG00000189060	1	-0.5	-0.95	-0.81	-0.979	0.5523	-0.844	-0.02	-0.1	2.091	0.356617	-1.4108	-0.33077834	-1.080026496
825	H0YF61	SCP2	sterol carrier protein 2	6342	ENSG00000116171	1	0.702	0.21	0.37	-0.152	-0.59	-0.354	0.037	-0.73	-0.36	0.137322	0.780555	0.792696675	-0.012141642
348	H0YBR2	ESRP1	epithelial splicing regulatory protein 1	54845	ENSG00000104413	1	-0.07	-0.29	-0.23	0.0187	-0.042	-0.163	-0.31	-0.63	-0.01	0.503562	0.119975	-0.133141546	0.253116245
2752	Q6ZTN6	ANKRD13I	ankyrin repeat domain 13D	338692	ENSG00000172932	2	0.084	0.145	0.382	0.507	0.2835	-0.086	0.008	0.841	0.339	0.812998	-0.19238	-0.030929928	-0.161449571
886	H7C1G0	DNAJC10	DnaJ heat shock protein family (Hsp40) mer	54431	ENSG00000077232	1	0.103	-0.53	-0.13	0.0682	-0.072	-0.177	0.099	0.627	0.091	0.327561	-0.4567	-0.123825826	-0.332870672
622	E7EWP0	NDUFB5	NADH:ubiquinone oxidoreductase subunit E	4711	ENSG00000136521	1	-0.25	-0.26	-0.16	-0.092	-0.29	-0.045	0.065	0.36	-0.28	0.464978	-0.27218	-0.081207717	-0.190969112
670	E9PKW0	IFT46	intraflagellar transport 46	56912	ENSG00000118096	1	-0.06	-0.13	-0.2	0.1946	-0.313	-0.261	-0.34	-0.22	-0.09	0.866041	0.085469	-0.004244333	0.089713094
224	A0A0G2JPB	NLRP2	NLR family pyrin domain containing 2	55655	ENSG00000277060	1	0.158	0.439	0.483	-0.33	0.8526	0.0253	-0.2	-0.36	0.353	0.634655	0.42726	0.177314384	0.249945176
2870	Q8IYU2	HACE1	HECT domain and ankyrin repeat containing	57531	ENSG00000085382	2	0.024	0.615	-0.21	0.2857	-0.172	0.2759	0.166	-0.36	-0.39	0.578036	0.339293	0.013500928	0.32579191
3005	Q8WUM0	NUP133	nucleoporin 133	55746	ENSG00000069248	2	0.1	0.195	-0.23	0.0376	-0.129	0.4106	-0.31	-0.56	-0.07	0.31939	0.33935	-0.083410631	0.422760956
3370	Q9H2J4	PDCL3	phosducin like 3	79031	ENSG00000115539	2	-0.16	0.347	-0.37	0.1148	0.1126	0.1094	-0.22	-0.27	-0.37	0.309108	0.228407	-0.172139193	0.40054667
1611	U3KQL2	ACYP2	acylphosphatase 2	98	ENSG00000170634	2	1.297	-0.42	1.303	0.0125	1.2846	0.7473	0.27	-1.45	0.815	0.642225	0.850172	0.046615524	0.803556012
2309	Q03393	PTS	6-pyruvoyltetrahydropterin synthase	5805	ENSG00000150787	3	-0.23	-0.06	-0.14	0.1776	-0.444	0.387	-0.26	-0.04	0.008	0.788752	-0.04595	-0.183538442	0.137592793
839	H0YNA2	SPPL2A	signal peptide peptidase like 2A	84888	ENSG00000138600	4	0.331	0.082	0.01	0.1801	0.3015	-0.454	0.128	0.375	0.082	0.790161	-0.054	0.131816013	-0.185816415
2628	Q5SZ59	OTUD7B	OTU deubiquitinase 7B	56957	ENSG00000264522	1	-0.21	0.451	0.472	0.5077	0.125	0.0219	0.45	-0.44	0.524	0.988249	0.059895	0.018219475	0.041675563
2426	Q13625	TP53BP2	tumor protein p53 binding protein 2	7159	ENSG00000143514	1	0.141	0.565	-0.23	0.2934	-0.414	0.1561	-0.62	-0.18	0.218	0.696194	0.353396	0.146437322	0.206958314
620	E7EW73	FUT2	fucosyltransferase 2	2524	ENSG00000176920	1	-0.43	0.894	-0.96	0.4077	-0.169	-0.662	-0.46	0.135	-0.2	0.998318	0.006504	-0.024911329	0.031415045
658	E9PRS1	KAT5	lysine acetyltransferase 5	10524	ENSG00000172977	1	0.251	-1.8	-0.45	0.3529	-0.008	-0.202	-0.01	0.764	-0.88	0.642127	-0.62593	-0.714031936	0.088100762
2429	Q13641	TPBG	trophoblast glycoprotein	7162	ENSG00000146242	1	-0.72	-0.05	-0.31	0.0606	0.0096	0.0753	-0.03	0.318	-0.47	0.460838	-0.29693	-0.407086992	0.110154288
3261	Q9BT67	NDFIP1	Nedd4 family interacting protein 1	80762	ENSG00000131507	1	0.247	1.238	0.046	0.7964	0.3786	0.8684	0.076	0.185	0.171	0.470007	0.366404	-0.170856223	0.53726018
474	B8ZZI9	AFTPH	aftiphilin	54812	ENSG00000119844	1	0.06	0.307	-0.66	-0.321	0.2473	0.4109	0.849	-1.8	0.361	0.940671	0.098664	-0.209315978	0.307980329
6	A0A024R7W	YTHDF3	YTH N6-methyladenosine RNA binding prot	253943	ENSG00000185728	3										1	#DIV/0!	#DIV/0!	#DIV/0!
62	A0A087WX9	BCL2L13	BCL2 like 13	23786	ENSG00000099968	1										1	#DIV/0!	#DIV/0!	#DIV/0!
96	A0A087X0T2	CEACAM2	carcinoembryonic antigen related cell adhes	125931	ENSG00000273777	1										1	#DIV/0!	#DIV/0!	#DIV/0!
168	A0A0A0MTF	INPPL1	inositol polyphosphate phosphatase like 1	3636	ENSG00000165458	2										1	#DIV/0!	#DIV/0!	#DIV/0!
200	A0A0C4DH2	IGKV3D-2(	immunoglobulin kappa variable 3D-20	28874	ENSG00000211625	3										1	#DIV/0!	#DIV/0!	#DIV/0!
144	A0A1W2PN.	KCNMA1	potassium calcium-activated channel subfar	3778	ENSG00000156113	1										1	#DIV/0!	#DIV/0!	#DIV/0!
333	A0A2R8YCL	GRB10	growth factor receptor bound protein 10	2887	ENSG00000106070	1										1	#DIV/0!	#DIV/0!	#DIV/0!
356	A2A283	SYNGR1	synaptogyrin 1	9145	ENSG00000100321	1										1	#DIV/0!	#DIV/0!	#DIV/0!
15	A2NJV5	IGKV2-29	immunoglobulin kappa variable 2-29 (gene/p	28920	ENSG00000253998	2										1	#DIV/0!	#DIV/0!	#DIV/0!
381	A6NKD9	CCDC85C	coiled-coil domain containing 85C	317762	ENSG00000205476	1										1	#DIV/0!	#DIV/0!	#DIV/0!
389	A7E2Y1	MYH7B	myosin heavy chain 7B	57644	ENSG00000078814	1										1	#DIV/0!	#DIV/0!	#DIV/0!

391	A8K878	MANF	mesencephalic astrocyte derived neurotroph	7873	ENSG00000145050	1	1	#DIV/0!	#DIV/0!	#DIV/0!
421	B1ANR0	PABPC4	poly(A) binding protein cytoplasmic 4	8761	ENSG00000090621	10	1	#DIV/0!	#DIV/0!	#DIV/0!
428	B4DGQ7	ERBB3	erb-b2 receptor tyrosine kinase 3	2065	ENSG00000065361	1	1	#DIV/0!	#DIV/0!	#DIV/0!
150	C9JB29	CASP8	caspase 8	841	ENSG00000064012	2	1	#DIV/0!	#DIV/0!	#DIV/0!
91	C9JH37	CHL1	cell adhesion molecule L1 like	10752	ENSG00000134121	1	1	#DIV/0!	#DIV/0!	#DIV/0!
521	C9JIS7	ZPBP	zona pellucida binding protein	11055	ENSG00000042813	2	1	#DIV/0!	#DIV/0!	#DIV/0!
492	C9JNZ3	BTN3A3	butyrophilin subfamily 3 member A3	10384	ENSG00000111801	1	1	#DIV/0!	#DIV/0!	#DIV/0!
556	D4Q8H0	MAP3K20;	mitogen-activated protein kinase kinase kina	51776	ENSG00000091436	1	1	#DIV/0!	#DIV/0!	#DIV/0!
558	D6RAD7	DCTD	dCMP deaminase	1635	ENSG00000129187	1	1	#DIV/0!	#DIV/0!	#DIV/0!
575	D6RFW1	SGTB	small glutamine rich tetratricopeptide repeat	54557	ENSG00000197860	1	1	#DIV/0!	#DIV/0!	#DIV/0!
578	D6RIF6	SLAIN2	SLAIN motif family member 2	57606	ENSG00000109171	13	1	#DIV/0!	#DIV/0!	#DIV/0!
581	E5RFG2	SEMA4G	semaphorin 4G	57715	ENSG00000095539	1	1	#DIV/0!	#DIV/0!	#DIV/0!
585	E5RH18	LSM1	LSM1 homolog, mRNA degradation associa	27257	ENSG00000175324	1	1	#DIV/0!	#DIV/0!	#DIV/0!
590	E5RIH5	TTI2	TELO2 interacting protein 2	80185	ENSG00000129696	1	1	#DIV/0!	#DIV/0!	#DIV/0!
233	E7ER82	ADAM32	ADAM metallopeptidase domain 32	203102	ENSG00000197140	1	1	#DIV/0!	#DIV/0!	#DIV/0!
625	E9PAM4	AL355315.	novel protein	NA	ENSG00000249967	1	1	#DIV/0!	#DIV/0!	#DIV/0!
648	E9PGE5	TBC1D23	TBC1 domain family member 23	55773	ENSG00000036054	1	1	#DIV/0!	#DIV/0!	#DIV/0!
676	E9PM31	CD81	CD81 molecule	975	ENSG00000110651	2	1	#DIV/0!	#DIV/0!	#DIV/0!
689	E9PR22	KLK12	kallikrein related peptidase 12	43849	ENSG00000186474	1	1	#DIV/0!	#DIV/0!	#DIV/0!
743	F8VWW7	SPRYD3	SPRY domain containing 3	84926	ENSG00000167778	1	1	#DIV/0!	#DIV/0!	#DIV/0!
744	F8VX55	TFCP2	transcription factor CP2	7024	ENSG00000135457	2	1	#DIV/0!	#DIV/0!	#DIV/0!
756	F8W9S7	GAPVD1	GTPase activating protein and VPS9 domai	26130	ENSG00000165219	1	1	#DIV/0!	#DIV/0!	#DIV/0!
766	F8WBZ6	CNOT9	CCR4-NOT transcription complex subunit 9	9125	ENSG00000144580	1	1	#DIV/0!	#DIV/0!	#DIV/0!
811	H0Y8L3	TGFBI	transforming growth factor beta induced	7045	ENSG00000120708	1	1	#DIV/0!	#DIV/0!	#DIV/0!
818	H0YBR0	TRAPPC9	trafficking protein particle complex 9	83696	ENSG00000167632	1	1	#DIV/0!	#DIV/0!	#DIV/0!
831	H0YL68	GMPR2	guanosine monophosphate reductase 2	51292	ENSG00000100938	2	1	#DIV/0!	#DIV/0!	#DIV/0!
859	H3BRR2	POLR2C	RNA polymerase II subunit C	5432	ENSG00000102978	1	1	#DIV/0!	#DIV/0!	#DIV/0!
851	H3BSS3	TCF25	transcription factor 25	22980	ENSG00000141002	1	1	#DIV/0!	#DIV/0!	#DIV/0!
536	H7C0G7	NHEJ1	non-homologous end joining factor 1	79840	ENSG00000187736	1	1	#DIV/0!	#DIV/0!	#DIV/0!
515	H7C0S2	OSCP1	organic solute carrier partner 1	127700	ENSG00000116885	2	1	#DIV/0!	#DIV/0!	#DIV/0!
888	H7C2K2	NCKIPSD	NCK interacting protein with SH3 domain	51517	ENSG00000213672	1	1	#DIV/0!	#DIV/0!	#DIV/0!
891	H7C557	MEGF6	multiple EGF like domains 6	1953	ENSG00000162591	1	1	#DIV/0!	#DIV/0!	#DIV/0!
916	J3KPG9	CSAD	cysteine sulfinic acid decarboxylase	51380	ENSG00000139631	1	1	#DIV/0!	#DIV/0!	#DIV/0!
919	J3KQ40	TBC1D8	TBC1 domain family member 8	11138	ENSG00000204634	1	1	#DIV/0!	#DIV/0!	#DIV/0!
896	J3QRX6	COPRS	coordinator of PRMT5 and differentiation sti	55352	ENSG00000172301	1	1	#DIV/0!	#DIV/0!	#DIV/0!
463	K7EIJ4	RANBP3	RAN binding protein 3	8498	ENSG00000031823	2	1	#DIV/0!	#DIV/0!	#DIV/0!
950	K7EJS4	SPINT2	serine peptidase inhibitor, Kunitz type 2	10653	ENSG00000167642	1	1	#DIV/0!	#DIV/0!	#DIV/0!
956	K7ELC2	RPS15	ribosomal protein S15	6209	ENSG00000115268	3	1	#DIV/0!	#DIV/0!	#DIV/0!
966	K7EPM3	ABCA5	ATP binding cassette subfamily A member 5	23461	ENSG00000154265	2	1	#DIV/0!	#DIV/0!	#DIV/0!
1086	O15258	RER1	retention in endoplasmic reticulum sorting re	11079	ENSG00000157916	2	1	#DIV/0!	#DIV/0!	#DIV/0!
1108	O43278	SPINT1	serine peptidase inhibitor, Kunitz type 1	6692	ENSG00000166145	22	1	#DIV/0!	#DIV/0!	#DIV/0!
1117	O43493	TGOLN2	trans-golgi network protein 2	10618	ENSG00000152291	1	1	#DIV/0!	#DIV/0!	#DIV/0!
1122	O43581	SYT7	synaptotagmin 7	9066	ENSG00000011347	28	1	#DIV/0!	#DIV/0!	#DIV/0!
1233	O75882	ATRN	attractin	8455	ENSG00000088812	42	1	#DIV/0!	#DIV/0!	#DIV/0!
1244	O75995	SASH3	SAM and SH3 domain containing 3	54440	ENSG00000122122	1	1	#DIV/0!	#DIV/0!	#DIV/0!
1273	O95219	SNX4	sorting nexin 4	8723	ENSG00000114520	3	1	#DIV/0!	#DIV/0!	#DIV/0!
1290	O95461	LARGE1	LARGE xylosyl- and glucuronyltransferase 1	9215	ENSG00000133424	2	1	#DIV/0!	#DIV/0!	#DIV/0!
1379	P02462	COL4A1	collagen type IV alpha 1 chain	1282	ENSG00000187498	3	1	#DIV/0!	#DIV/0!	#DIV/0!
1533	P0DI82	TRAPPC2I	trafficking protein particle complex 2B;traffic	10597;6399	ENSG00000256060	2	1	#DIV/0!	#DIV/0!	#DIV/0!
2000	P49662	CASP4	caspase 4	837	ENSG00000196954	1	1	#DIV/0!	#DIV/0!	#DIV/0!
2110	P55259	GP2	glycoprotein 2	2813	ENSG00000169347	16	1	#DIV/0!	#DIV/0!	#DIV/0!
2117	P55957	BID	BH3 interacting domain death agonist	637	ENSG00000015475	1	1	#DIV/0!	#DIV/0!	#DIV/0!
2491	Q15029	EFTUD2	elongation factor Tu GTP binding domain co	9343	ENSG00000108883	3	1	#DIV/0!	#DIV/0!	#DIV/0!
2492	Q15034	HERC3;AC	HECT and RLD domain containing E3 ubiqu	8916	ENSG00000138641	2	1	#DIV/0!	#DIV/0!	#DIV/0!
2690	Q6IWH7	ANO7	anoctamin 7	50636	ENSG00000146205	30	1	#DIV/0!	#DIV/0!	#DIV/0!
2696	Q6NXR6	SETD3	SET domain containing 3, actin histidine me	84193	ENSG00000183576	1	1	#DIV/0!	#DIV/0!	#DIV/0!
2700	Q6P3S1	DENND1B	DENN domain containing 1B	163486	ENSG00000213047	1	1	#DIV/0!	#DIV/0!	#DIV/0!
2701	Q6P3X3	TTC27	tetratricopeptide repeat domain 27	55622	ENSG00000018699	1	1	#DIV/0!	#DIV/0!	#DIV/0!
2762	Q7L273	KCTD9	potassium channel tetramerization domain c	54793	ENSG00000104756	1	1	#DIV/0!	#DIV/0!	#DIV/0!
2836	Q8IUI8	CRLF3	cytokine receptor like factor 3	51379	ENSG00000176390	1	1	#DIV/0!	#DIV/0!	#DIV/0!
2894	Q8N357	SLC35F6	solute carrier family 35 member F6	54978	ENSG00000213699	1	1	#DIV/0!	#DIV/0!	#DIV/0!
2927	Q8NC51	SERBP1	SERPINE1 mRNA binding protein 1	26135	ENSG00000142864	1	1	#DIV/0!	#DIV/0!	#DIV/0!
2947	Q8NFL0	B3GNT7	UDP-GlcNAc:betaGal beta-1,3-N-acetylgluc	93010	ENSG00000156966	1	1	#DIV/0!	#DIV/0!	#DIV/0!
2950	Q8NFT8	DNER	delta/notch like EGF repeat containing	92737	ENSG00000187957	1	1	#DIV/0!	#DIV/0!	#DIV/0!
2969	Q8TBP5	FAM174A	family with sequence similarity 174 member	345757	ENSG00000174132	1	1	#DIV/0!	#DIV/0!	#DIV/0!
2990	Q8TE73	DNAH5	dynein axonemal heavy chain 5	1767	ENSG00000039139	1	1	#DIV/0!	#DIV/0!	#DIV/0!
3077	Q969S3	ZNF622	zinc finger protein 622	90441	ENSG00000173545	1	1	#DIV/0!	#DIV/0!	#DIV/0!
3079	Q96A05	ATP6V1E2	ATPase H+ transporting V1 subunit E2	90423	ENSG00000250565	11	1	#DIV/0!	#DIV/0!	#DIV/0!
3151	Q96J01	THOC3	THO complex 3	84321	ENSG00000051596	2	1	#DIV/0!	#DIV/0!	#DIV/0!
3154	Q96JC1	VPS39	VPS39 subunit of HOPS complex;VPS39, H	23339	ENSG00000166887	1	1	#DIV/0!	#DIV/0!	#DIV/0!
3226	Q99747	NAPG	NSF attachment protein gamma	8774	ENSG00000134265	18	1	#DIV/0!	#DIV/0!	#DIV/0!
3270	Q9BU89	DOHH	deoxyhypusine hydroxylase	83475	ENSG00000129932	1	1	#DIV/0!	#DIV/0!	#DIV/0!
3429	Q9HAU4	SMURF2	SMAD specific E3 ubiquitin protein ligase 2	64750	ENSG00000108854	2	1	#DIV/0!	#DIV/0!	#DIV/0!
3430	Q9HAU5	UPF2	UPF2 regulator of nonsense mediated mRN	26019	ENSG00000151461	1	1	#DIV/0!	#DIV/0!	#DIV/0!
3521	Q9NVM1	EVA1B	eva-1 homolog B	55194	ENSG00000142694	1	1	#DIV/0!	#DIV/0!	#DIV/0!
3301	Q9NY87	SPANXC	SPANX family member C	64663	ENSG00000198573	1	1	#DIV/0!	#DIV/0!	#DIV/0!
3589	Q9UBV2	SEL1L	SEL1L adaptor subunit of ERAD E3 ubiquiti	6400	ENSG00000071537	1	1	#DIV/0!	#DIV/0!	#DIV/0!
388	S4R3B3	ZSWIM8	zinc finger SWIM-type containing 8	23053	ENSG00000214655	1	1	#DIV/0!	#DIV/0!	#DIV/0!
761	V9GY95	MAP4K3	mitogen-activated protein kinase kinase kina	8491	ENSG00000011566	1	1	#DIV/0!	#DIV/0!	#DIV/0!
