# Supplementary material for: Quantitative proteomic biomarkers from extracellular vesicles of human seminal plasma in the differential diagnosis of azoospermia
Source: Clin Transl Med. 2021 May 28;11(5):e423. doi: 10.1002/ctm2.423 (PMC8161617; doi:10.1002/ctm2.423)
Supplement: Supplementary file 5 — Supporting Information [file CTM2-11-e423-s005.pdf]

Supplementary Table 3. Quantitative phosphoproteome of spEV from NS, NOA and OA patients.

id	UniprotID	Gene Symbol	Gene name	ENTREZID	ENSEMBL gene	Sequence window	Amino acid	Localization prob	Position	NS-1	NS-2	NS-3	NOA-1	NOA-2	NOA-3	OA-1	OA-2	OA-3	ANOVA FDR-q	Fold change (NS/OA)	Fold change (NS/NOA)	Fold change (NOA/OA)	ANOVA Significant	Differential proteins	Significant pairs
587	P04279	SEMG1	semenogelin 1	6406	ENSG00000124233	KAHRGTQNPSQDQGNSPSGKGISSQYSNTEE	S	0.996581	149	21.17968	20.82058	20.9369	21.03564	21.63462	21.32926	20.45352	20.84728	20.6808	1.47075997	0.318514506	-0.354124069	0.672638575	+	Yes	NOA_OA; NOA_OA; OA_NS
29	E9PK06	EEF1D	eukaryotic translation elongation factor 1 delta	1936	ENSG00000104529	SSPGHRATAPQTQHVSPMRQVEPPAKKPATP	S	0.999806	114	19.78437	19.83353	19.53285	18.45599	18.35341	18.33582	18.6562	18.33237	18.14564	3.865834312	1.338841756	1.335171382	0.003670375	+	Yes	NS_OA; NS_NOA
608	P04554	PRM2	protamine 2	5620	ENSG00000122304	LSPEHVEVYERTHGQSHYRRRHCSRRRLHRI	S	0.999787	51	19.41376	18.79734	18.09692	16.25494	16.07021	15.77123	16.55989	16.32473	15.7948	3.216964254	2.542871157	2.737215996	-0.194344838	+	Yes	NS_NOA; NS_OA
982	Q92526	CCT6B	chaperonin containing TCP1 subunit 6B	10693	ENSG00000132141	_______MAAIKAVNSKAEVARARAALAVNI	S	1	9	18.30555	17.08535	16.57363	15.57155	15.33609	16.03075	15.41989	15.9348	15.34228	1.769764581	1.75585111	1.675379753	0.080471357	+	Yes	NS_OA; NS_NOA
340	F5H5K1	LRRC37B	leucine rich repeat containing 37B	114659	ENSG00000185158	VNSHKRASEKYKDNPSISGA___________	S	0.825745	861	18.23458	17.22873	16.9194	14.97316	15.24492	14.96551	15.21071	15.10603	15.00013	3.248570936	2.355278333	2.399704297	-0.044425964	+	Yes	NS_NOA; NS_OA
662	P27824	CANX	calnexin	821	ENSG00000283777	EDRKPKAEEDEILNRSPRNRKPRRE______	S	1	475	17.75974	18.29818	17.24998	16.73754	16.63566	16.46423	17.00668	16.57283	16.47233	1.920425677	1.085356394	1.156827291	-0.071470896	+	Yes	NS_NOA; NS_OA
1467	Q9HBV2	SPACA1	sperm acrosome associated 1	81833	ENSG00000118434	INWAAVKAFWGAKASTPEVQSEQSSVRYKDS	T	0.987867	257	17.59244	17.87778	17.08421	16.80763	16.55853	16.56662	16.33542	16.18743	16.3961	2.549083619	1.211821874	0.873881658	0.337940216	+	Yes	NS_OA; NS_NOA
13	A0A087WWA1	P3R3URF;LOC11011749	PIK3R3 upstream reading frame;uncharacterized LOC110117498	110117498	ENSG00000250719	CSRRRYQQGLRGRTASSAAINPATRAMGINN	S	0.893905	52	17.31743	16.58144	16.75016	15.91354	15.95515	15.66256	15.93397	15.83716	15.54424	2.418166539	1.111219088	1.039258003	0.071961085	+	Yes	NS_OA; NS_NOA
1165	Q9Y2Q0	ATP8A1	ATPase phospholipid transporting 8A1	10396	ENSG00000124406	RAEGYEKTDDVSEKTSLADQEEVRTIFINQP	S	0.913551	29	17.23613	17.4677	17.45024	18.01683	17.94106	17.54465	17.09177	16.89829	17.36016	1.781960092	0.267950058	-0.449488322	0.71743838	+	Yes	NOA_OA
208	C9J020	SLCO6A1	solute carrier organic anion transporter family member 6A1	133482	ENSG00000205359	_____MFVGVARHSGSQDEVSRGVEPLEAAR	S	0.996742	11	17.18824	18.1684	16.795	13.1536	13.78484	12.41569	13.75144	12.96394	12.96967	3.620155593	4.155527751	4.265837351	-0.110309601	+	Yes	NS_NOA; NS_OA
602	P04279	SEMG1	semenogelin 1	6406	ENSG00000124233	EEKAQGKSQKQITIPSQEQEHSQKANKISYQ	S	0.999885	334	17.09569	16.88286	16.17629	17.06371	17.57703	17.12333	18.24813	17.43705	17.81052	1.412978048	-1.113623937	-0.536413193	-0.577210744	+	Yes	NOA_OA; NOA_OA; OA_NS
578	O96013	PAK4	p21 (RAC1) activated kinase 4	10298	ENSG00000130669	RRAGPEKRPKSSREGSGGPQESSRDKRPLSG	S	0.9994	167	16.77892	16.70144	17.1972	16.26893	16.50409	16.4496	15.99925	16.20201	16.27928	2.066405503	0.732340177	0.484982808	0.247357368	+	Yes	NS_OA; NS_NOA
1511	F5GYU8	TMC5	transmembrane channel like 5	79838	ENSG00000103534	RSLSNPDYSGTRSNAYSAASRTSPDHPTSLP	Y	0.988395	91	16.69112	17.34143	16.83812	17.77095	17.70442	17.54215	17.50556	17.43624	17.5524	1.846953332	-0.541177114	-0.715616226	0.174439112	+	Yes	OA_NS; NOA_NS
151	A6NDB9	PALM3	paralemmin 3	342979	ENSG00000187867	GLEGPEVAGRERGDESPLGAEGAKTGGGEET	S	1	375	16.60875	16.47765	16.49765	16.33321	16.30184	16.3016	15.75968	15.83654	15.88753	4.862264713	0.70009613	0.21579806	0.48429807	+	Yes	NOA_OA; NS_OA; NS_NOA
582	Q8TCE1	SERPINC1	serpin family C member 1	462	ENSG00000117601	CIYRSPEKKATEDEGSEQKIPEATNRRVWEL	S	0.999989	68	16.4126	16.52	16.5699	17.07515	16.97992	16.54037	17.64892	17.16457	17.20428	1.903695475	-0.838423411	-0.364308039	-0.474115372	+	Yes	OA_NS
147	D6RJH4	ARHGEF37	Rho guanine nucleotide exchange factor 37	389337	ENSG00000183111	_____MAKHGADEPSSRSGSPDREGRASEDR	S	0.806488	11	16.3653	16.49952	16.21904	17.16457	16.90395	16.86339	16.56689	16.43809	16.69927	2.255487356	-0.206800461	-0.616020203	0.409219742	+	Yes	NOA_OA; NOA_NS
308	E9PRA5	ATP1A4	ATPase Na+/K+ transporting subunit alpha 4	480	ENSG00000132681	MGLWGKKGTVAPHDQSPRRRPKKGLIKKKMV	S	0.999458	16	16.02918	15.86513	15.84154	15.27103	14.81438	14.47212	15.31154	14.92203	14.78923	2.100088563	0.904352506	1.059439977	-0.155087471	+	Yes	NS_NOA; NS_OA
268	E7EQB2	LTF	lactotransferrin	4057	ENSG00000012223	LCIGDEQGENKCVPNSNERYYGYTGAFRCLA	S	1	527	15.95185	15.37473	15.82382	16.03142	16.19762	16.01674	18.21504	16.27633	17.3356	1.391488688	-1.558854103	-0.365127246	-1.193726858	+	Yes	OA_NS
95	Q08648	SPAG11B;SPAG11A	sperm associated antigen 11B;sperm associated antigen 11A	10407;653423	ENSG00000164871	ICHMQQGICRLFFCHSGEKKRDICSDPWNRC	S	1	44	15.95167	16.27556	16.02881	15.13723	15.02341	16.09069	14.02367	14.03273	13.45417	2.905187448	2.248490334	0.668238322	1.580252012	+	Yes	NOA_OA; NS_OA
247	E5RJV7	SGK3	serum/glucocorticoid regulated kinase family member 3	23678;100533	ENSG00000104205	PDVRAFLQMDSPKHQSDPSEDEDERSSQKLH	S	0.945363	59	15.9402	15.98477	15.84955	15.89539	16.21031	16.09783	15.30916	15.40932	15.33497	3.448596114	0.57369105	-0.142998695	0.716689746	+	Yes	NS_OA; NOA_OA
897	Q75WM6	H1-7;H1FNT	H1.7 linker histone;H1 histone family member N, testis specific	341567	ENSG00000187166	AMAEAPGPSGESRGHSATQLPAEKTVGGPSR	S	0.759231	36	15.72547	15.05723	14.68863	14.58531	14.49891	14.36277	13.83901	14.22558	14.38093	1.498991027	1.008603414	0.674781164	0.33382225	+	Yes	NS_OA
1237	B3KVN2	FNTA	farnesyltransferase, CAAX box, alpha	2339	ENSG00000168522	GRSLQSKHSTENDSPTNVQQ___________	T	0.87725	224	15.46298	14.88784	15.22619	15.82483	16.08042	15.90619	16.17915	16.06232	15.60386	1.779017049	-0.756107648	-0.744811694	-0.011295954	+	Yes	NOA_NS; OA_NS
1099	Q9NRD9	DUOX1	dual oxidase 1	53905	ENSG00000137857	LRMRNFKRLQGQDRQSIVSEKLVGGMEALEW	S	0.983596	280	15.28526	15.34301	15.11431	15.68129	15.61778	15.22457	14.71414	15.02072	14.40714	1.772940619	0.5335296	-0.26035436	0.793883959	+	Yes	NOA_OA
658	P25686	DNAJB2	DnaJ heat shock protein family (Hsp40) member B2	3300	ENSG00000135924	DRYGREGLTGTGTGPSRAEAGSGGPGFTFTF	S	0.925037	81	15.27081	15.7628	15.04729	15.67868	15.5055	15.78645	15.09593	15.04354	15.03566	1.309226782	0.30192248	-0.296578089	0.59850057	+	Yes	NOA_OA
1203	A0A0A0MR22	ATP9A	ATPase phospholipid transporting 9A (putative)	10079	ENSG00000054793	IRRDSKIPGTVVRSSTIPEQLGRISYLLTDK	T	0.984722	241	15.22038	15.20197	15.15513	15.71408	15.24273	15.247	14.57205	14.71671	14.84422	2.10718384	0.481502215	-0.208776474	0.690278689	+	Yes	NS_OA; NOA_OA
944	Q8IZP9	ADGRG2	adhesion G protein-coupled receptor G2	10149	ENSG00000173698	NGKGRMALRRTSKRGSLHFIEQM________	S	1	891	15.07456	15.09379	15.51021	14.70455	14.63226	15.07656	14.17063	14.76332	14.61039	1.346158925	0.711406708	0.421727816	0.289678891	+	Yes	NS_OA
434	I3L4Z8	PCTP	phosphatidylcholine transfer protein	58488	ENSG00000141179	DMEGRKIHVILARSTSMPQLGERSGVIRVKQ	S	0.911264	22	14.8914	15.12541	14.63146	15.28443	14.94522	15.13743	14.29211	14.29333	14.62799	1.852326379	0.478285154	-0.239597956	0.71788311	+	Yes	NOA_OA
1217	H0YN01	TLN2	talin 2	83660	ENSG00000171914	MVDAIAEAMSKLDEGTPPEPKGTFVDYQTTV	T	1	236	14.88617	14.66606	14.48243	14.95851	15.07844	15.0011	14.28164	14.50457	14.12807	2.262991106	0.373458227	-0.334463437	0.707921664	+	Yes	NOA_OA
808	Q14764	MVP	major vault protein	9961	ENSG00000013364	GQDPLADRGEKDTAKSLQPLAPRNKTRVVSY	S	1	445	14.67673	15.43577	15.3444	15.12791	15.36191	15.15648	16.13917	15.51237	16.20917	1.330359084	-0.801270803	-0.063134829	-0.738135974	+	Yes	OA _ NS
888	Q6P597	KLC3	kinesin light chain 3	147700	ENSG00000104892	RGEAAAGAAGMKRAMSLNTLNVDAPRAPGTQ	S	0.999931	466	14.58114	14.51638	14.26993	14.22897	14.3683	14.6297	13.86467	13.71789	14.01507	1.978989693	0.589940707	0.046826998	0.543113708	+	Yes	NOA_OA; NS_OA
1117	Q9P206	KIAA1522	KIAA1522	57648	ENSG00000162522	DTIVSDGSTLSSKGGSEGQPESSTASNSVVP	S	0.991226	390	14.42653	14.60403	14.25805	14.92254	14.78862	14.44708	13.16806	13.58461	12.9855	3.114101761	1.183478038	-0.289875984	1.473354022	+	Yes	NS_OA; NOA_OA
704	P51787	KCNQ1	potassium voltage-gated channel subfamily Q member 1	3784	ENSG00000282076	__________MAAASSPPRAERKRWGWGRLP	S	0.874026	6	14.2735	14.23736	14.65788	13.92258	14.53382	14.04157	14.75149	14.74468	14.87224	1.350663657	-0.399889946	0.223588626	-0.623478572	+	Yes	OA_NOA
371	F8WCG7	PHYHD1	phytanoyl-CoA dioxygenase domain containing 1	254295	ENSG00000175287	___________MACLSPSQLQKQPILIALCW	S	0.99454	5		12.24404		11.1905	12.4425	11.82544				1.493579384	#DIV/0!	0.424555779	#DIV/0!	+	Yes	NOA_OA
1133	Q9P2G1	ANKIB1	ankyrin repeat and IBR domain containing 1	54467	ENSG00000001629	QEFLASVARGVAPADSPEAPRRSFAGGTWDW	S	1	737	21.93247	21.5757	21.60246	21.50833	21.45704	21.61108	20.9549	21.03329	21.46229	1.427452315	0.553386688	0.178058624	0.375328064	+		
586	P04279	SEMG1	semenogelin 1	6406	ENSG00000124233	IHHKGGKAHRGTQNPSQDQGNSPSGKGISSQ	S	0.999932	143	20.61342	20.05933	20.47179	20.655	20.9022	20.67211	20.18725	20.31937	20.14221	1.419476351	0.165240606	-0.361584981	0.526825587	+		
44	B6VEX4	ABI1	abl interactor 1	10006	ENSG00000136754	TGTLSRTNPPTQKPPSPPMSGRGTLGRNTPY	S	0.994999	183	19.95546	19.98036	19.97183	20.12389	19.99092	20.11082	19.73289	19.55345	19.93014	1.500133019	0.230391184	-0.105993907	0.336385091	+		
192	C9JRC4	PPP1R7	protein phosphatase 1 regulatory subunit 7	5510	ENSG00000115685	SQEMMEVDRRVESEESGDEEGKKHSSGIVAD	S	0.997464	27	19.51468	19.48487	19.18403	19.4194	19.60132	19.51079	19.11146	19.0855	19.19964	1.627443079	0.262325287	-0.115975698	0.378300985	+		
837	Q4VX76	SYTL3	synaptotagmin like 3	94120	ENSG00000164674	TGPRQCVGQTERRSQSDTAVNVTTRKVSAPD	S	0.973523	231	19.14619	19.11301	19.12659	19.36273	19.21627	19.21665	18.97118	18.94804	19.17653	1.329604477	0.096681595	-0.136620839	0.233302434	+		
828	Q2M2I8	AAK1	AP2 associated kinase 1	22848	ENSG00000115977	ASLNKSKSATTTPSGSPRTSQQNVYNPSEGS	S	0.997671	678	18.92555	18.91992	18.97309	19.13904	18.97846	19.03392	18.73618	18.76098	18.87894	2.039631844	0.147487005	-0.11095047	0.258437475	+		
72	A0A0C4DFY5	GPRC5C	G protein-coupled receptor class C group 5 member C	55890	ENSG00000170412	AQSHQAATPPKDGKNSQVFRNPYVWD_____	S	1	476	18.92381	19.36068	18.92776	19.48776	19.51355	19.17177	18.6789	18.97508	18.89436	1.347338184	0.221302668	-0.320276896	0.541579564	+		
702	P51397	DAP	death associated protein	1611	ENSG00000112977	_____________MSSPPEGKLETKAGHPP	S	0.5	2	18.78972	18.89655	18.71843	18.88923	18.97992	18.99339	18.61036	18.6824	18.72241	2.133958252	0.129847844	-0.1526076	0.282455444	+		
703	P51397	DAP	death associated protein	1611	ENSG00000112977	____________MSSPPEGKLETKAGHPPA	S	0.5	3	18.78972	18.89655	18.71843	18.88923	18.97992	18.99339	18.61036	18.6824	18.72241	2.133958252	0.129847844	-0.1526076	0.282455444	+		
1497	A0A0C4DFY5	GPRC5C	G protein-coupled receptor class C group 5 member C	55890	ENSG00000170412	IPEVSQVTKSSPEQSYQGDMYPTRGVGYETI	Y	0.998982	357	18.39636	18.19724	18.4132	18.70127	18.8252	18.58883	18.58097	18.78788	18.45378	1.402920659	-0.271942774	-0.369496028	0.097553253	+		
648	P18206	VCL	vinculin	7414	ENSG00000035403	QMTDQVADLRARGQGSSPVAMQKAQQVSQGL	S	0.632623	345	18.34728	18.37802	18.27476	18.25898	18.38136	18.33508	18.06697	18.21262	18.18471	1.601761776	0.178585688	0.008216222	0.170369466	+		
224	C9JZY6	UBE2H	ubiquitin conjugating enzyme E2 H	7328	ENSG00000186591	____________MSSPSPGKRRMDTDVVKL	S	0.499983	3	18.30479	18.49001	18.38313	18.73675	18.88774	18.61425	17.87712	18.01944	18.55195	1.320207272	0.243143082	-0.353602727	0.596745809	+		
223	C9JZY6	UBE2H	ubiquitin conjugating enzyme E2 H	7328	ENSG00000186591	_____________MSSPSPGKRRMDTDVVK	S	0.499983	2	18.30479	18.49001	18.38313	18.73675	18.88774	18.61425	17.87712	18.01944	18.55195	1.320207272	0.243143082	-0.353602727	0.596745809	+		
1235	H0Y9T8	PRR5-ARHGAP8	PRR5-ARHGAP8 readthrough	553158	ENSG00000248405	GLRTEGLFRRSASVQTVREIQRLYNQGKPVN	T	0.630334	293	18.16384	18.09672	18.05796	18.18839	18.21058	18.13149	17.94261	18.06781	17.85307	1.592271995	0.151676178	-0.070648829	0.222325007	+		
330	Q92934	BAD	BCL2 associated agonist of cell death	572	ENSG00000002330	DEFVDSFKKGLPRPKSAGTATQMRQSSSWTR	S	0.999743	134	18.12065	18.37054	18.04666	18.10871	18.21238	18.13841	17.83559	17.87484	17.98799	1.382800656	0.279811223	0.026116053	0.25369517	+		
148	D6RJH4	ARHGEF37	Rho guanine nucleotide exchange factor 37	389337	ENSG00000183111	_MAKHGADEPSSRSGSPDREGRASEDRSLLH	S	0.999027	15	18.03489	18.09032	17.90982	18.18103	18.15551	18.10488	17.69608	17.54909	17.92503	1.824969059	0.288271586	-0.135461807	0.423733393	+		
380	H0Y332	STXBP5	syntaxin binding protein 5	134957	ENSG00000164506	DLDVKDNSFSRSRSSSVTSIDKESREAISAL	S	0.91503	108	17.9522	17.97029	17.7282	18.10651	17.90748	17.82215	17.44662	17.25322	17.69988	1.543479825	0.416987101	-0.061819077	0.478806178	+		
35	F2Z357	RAP1GAP	RAP1 GTPase activating protein	5909	ENSG00000076864	HVSQEPKSENSSTQSSPEMPTTKNRAETAAQ	S	0.790921	542	17.76338	17.86689	17.6757	17.96115	18.08171	17.80593	17.73866	17.57799	17.61913	1.42910131	0.12339592	-0.180944443	0.304340363	+		
338	F5H4I7	CAMKK2	calcium/calmodulin dependent protein kinase kinase 2	10645	ENSG00000110931	TSGSQARPHLSGRKLSLQERSQGGLAAGGSL	S	1	100	17.51945	17.55487	17.55367	17.89741	17.66101	17.7924	17.49271	17.04953	17.50238	1.319936226	0.194458644	-0.240938822	0.435397466	+		
91	A0A0G2JIW1	HSPA1B	heat shock protein family A (Hsp70) member 1B	3304	ENSG00000232804	LETAGGVMTALIKRNSTIPTKQTQIFTTYSD	S	0.981622	419	17.35621	17.25599	17.45234	17.23098	17.07064	17.10656	17.05304	16.80209	17.05346	1.795749719	0.385317485	0.218783061	0.166534424	+		
798	Q13835	PKP1	plakophilin 1	5317	ENSG00000081277	TGAGSDICFMQKIKASRSEPDLYCDPRGTLR	S	0.607948	153	17.34897	17.20705	17.25654	17.49451	17.55817	17.51059	17.14619	17.27821	17.34265	2.118904833	0.015170415	-0.250236511	0.265406926	+		
1080	Q9H4A6	GOLPH3	golgi phosphoprotein 3	64083	ENSG00000113384	NAADKERAAGGGAGSSEDDAQSRRDEQDDDD	S	0.979135	36	17.20524	17.15837	17.16967	17.30706	17.22327	17.34655	17.18544	17.09589	17.20686	1.376740787	0.015031179	-0.114529928	0.129561106	+		
132	A0A2R8Y549	PATJ	PATJ crumbs cell polarity complex component	10207	ENSG00000132849	NAGQVVHLTLVRRKTSSSTSPLEPPSDRGTV	S	0.622328	455	17.19393	16.71156	17.09135	18.94638	17.52451	17.83461	16.59006	16.88608	17.05325	1.46630144	0.155818303	-1.102885564	1.258703868	+		
277	E7EX17	EIF4B	eukaryotic translation initiation factor 4B	1975	ENSG00000063046	AWVKRSSNPPARSQSSDTEQQSPTSGGGKVA	S	0.812579	503	17.09672	17.05346	17.26362	17.30519	17.44354	17.4777	17.28996	17.16595	17.2822	1.510786901	-0.108106613	-0.270877838	0.162771225	+		
555	O94819	KBTBD11	kelch repeat and BTB domain containing 11	9920	ENSG00000273645	AALGPAGERAGSRPQSPSGDADARGDAAVYC	S	0.986849	314	17.02896	17.04516	17.03757	17.30635	17.34733	17.18901	16.90689	17.15837	17.07546	1.616247675	-0.00967598	-0.243665059	0.23398908	+		
191	C9JRC4	PPP1R7	protein phosphatase 1 regulatory subunit 7	5510	ENSG00000115685	QQQSQEMMEVDRRVESEESGDEEGKKHSSGI	S	0.999543	24	17.01574	16.96387	16.70428	17.014	17.01105	16.99037	16.59405	16.52795	16.75238	1.732408561	0.269837697	-0.110509872	0.38034757	+		
997	Q96C24	SYTL4	synaptotagmin like 4	94121	ENSG00000102362	SDRTCARCQESLGRLSPKTNTCRGCNHLVCR	S	0.999999	74	16.97332	17.27712	17.08794	17.50347	17.35819	17.36854	16.92937	17.11105	17.06634	1.806794817	0.077203751	-0.297271729	0.374475479	+		
415	H3BUH7	ALDOA	aldolase, fructose-bisphosphate A	226	ENSG00000149925	LAADESTGSIAKRLQSIGTENTEENRRFYRQ	S	0.997636	46	16.9686	17.04676	16.84169	17.41782	17.26334	17.0441	16.96623	16.87604	16.79943	1.462005365	0.071781158	-0.28940328	0.361184438	+		
509	O43182	ARHGAP6	Rho GTPase activating protein 6	395	ENSG00000047648	FMLVGHLSSSKSRESSPGPRLGKDLSEEPFD	S	0.948642	515	16.95276	16.81591	16.7563	17.17485	17.04538	16.92405	16.38303	16.68702	16.66442	1.822546443	0.263500214	-0.20643425	0.469934464	+		
1308	O15127	SCAMP2	secretory carrier membrane protein 2	10066	ENSG00000140497	RKAAELERKERELQNTVANLHVRQNNWPPLP	T	1	109	16.93825	16.89877	16.60216	17.00009	16.97354	16.88584	16.31292	16.49512	16.72813	1.382652394	0.301002502	-0.140096664	0.441099167	+		
444	I3L4J1	AC026464.4	novel protein	NA	ENSG00000260914	KKPVKENQSEGKGSDSDSEGDNPEKKKLQEQ	S	0.586091	121	16.93261	17.02399	16.56556	17.1201	17.0351	16.97242	16.39607	16.31831	16.74018	1.467395226	0.355868022	-0.201817195	0.557685216	+		
375	G3V1S3	DENND5B	DENN domain containing 5B	160518	ENSG00000170456	DEDLVKQCRTPPQQKSPTTARRLSITSLTGK	S	0.899828	1103	16.83096	16.59425	16.74176	16.75903	16.69615	16.90124	16.48285	16.50386	16.51379	1.681033073	0.222155889	-0.063151677	0.285307566	+		
49	D3DNG8	AGTR1	angiotensin II receptor type 1	185	ENSG00000144891	LLKYIPPKAKSHSNLSTKMSTLSYRPSDNVS	S	0.75943	366	16.80461	16.92254	16.90677	16.91882	17.04067	16.97724	17.25755	17.59281	17.11136	1.574400436	-0.44260025	-0.100935618	-0.341664632	+		
1154	Q9UPX8	SHANK2	SH3 and multiple ankyrin repeat domains 2	22941	ENSG00000162105	AAKTIGSYVPGPRSRSPSLNRLGGAGEDGKR	S	0.794253	475	16.74767	16.81253	16.78775	16.53051	16.7889	16.83059	16.45031	16.40068	16.41726	2.074482808	0.359900157	0.065982819	0.293917338	+		
149	A2A2F0	RALGAPB	Ral GTPase activating protein non-catalytic beta subunit	57148	ENSG00000170471	CLVDAFLGISRPRSDSAPPTPVNRLSMPQSA	S	0.961021	187	16.74123	16.67411	16.61209	16.63708	16.69615	16.88107	16.47161	16.49376	16.31794	1.717492658	0.248039246	-0.062287649	0.310326894	+		
1414	Q7L1Q6	BZW1	basic leucine zipper and W2 domains 1	9689	ENSG00000082153	______MNNQKQQKPTLSGQRFKTRKRDEKE	T	0.995863	10	16.69112	16.644	16.71237	16.8758	16.68934	17.03896	16.41904	16.41561	16.63197	1.549701105	0.19361941	-0.185540517	0.379159927	+		
566	O95671	ASMTL	acetylserotonin O-methyltransferase like	8623	ENSG00000169093	VIGKLLHKRVVLASASPRRQEILSNAGLRFE	S	0.999581	21	16.63282	16.71612	16.75838	16.67024	16.41512	16.68332	16.38315	16.20843	16.4207	1.68110656	0.365017573	0.112882614	0.252134959	+		
160	A6PW57	PIP5K1A	phosphatidylinositol-4-phosphate 5-kinase type 1 alpha	8394	ENSG00000143398	IPLKPSPSKKFRSGSSFSRRAGSSGNSCITY	S	0.555089	456	16.61569	16.31157	16.41528	16.62385	16.74609	16.68278	15.94137	16.31865	16.4025	1.35583813	0.22667408	-0.236728668	0.463402748	+		
408	H3BMH2	RAB11A	RAB11A, member RAS oncogene family	8766	ENSG00000103769	QIWDTAGLERYRAITSAYYRGAVGALLVFDL;QIWI	S	0.965703	78	16.588	16.80133	16.63183	16.74399	16.625	16.51206	16.24295	16.14376	16.47834	1.587997354	0.38536644	0.046701431	0.338665009	+		
1007	Q96KN4	LRATD1;FAM84A	LRAT domain containing 1;family with sequence similarity 84 member A	151354	ENSG00000162981	KFGVKAPPGCTPCPESPSRHHHHLLHQLVLN	S	0.759172	67	16.57968	16.8734	16.70685	17.06118	16.88858	16.78072	16.57933	16.57645	16.52722	1.496442995	0.15897433	-0.190184275	0.349158605	+		
821	Q15773	MLF2	myeloid leukemia factor 2	8079	ENSG00000089693	EGPPRLAIQGPEDSPSRQSRRYDW_______	S	0.503606	240	16.57092	16.4372	16.54326	16.87123	16.5694	16.7546	16.92833	16.84341	16.81466	1.76553307	-0.345005671	-0.214615504	-0.130390167	+		
119	A0A1B0GUI1	RALGAPA1	Ral GTPase activating protein catalytic alpha subunit 1	253959	ENSG00000174373	PGQAPMRQRSATTTGSPGTEKARSIVRQKTV	S	0.996872	740	16.54028	16.42031	16.25849	16.33335	16.40718	16.4764	15.62751	15.97163	16.11975	1.742453553	0.500062307	0.000715256	0.499347051	+		
377	G3V530	FUT8	fucosyltransferase 8	2530	ENSG00000033170	IGGHLVRDNDHPDHSSRELSKI_________	S	0.786387	40	16.53842	16.6368	16.3074	16.8919	17.22289	16.89024	17.14171	17.10298	16.79475	1.667952024	-0.518942515	-0.507474899	-0.011467616	+		
660	P26373	RPL13	ribosomal protein L13	6137	ENSG00000167526	SLEELRVAGIHKKGDSSAEELKLATQLTGPV	S	0.5	92	16.52781	18.00064	16.46493	14.62628	14.56611	14.00589	15.42141	14.67441	14.19591	2.275707438	2.233882904	2.598366102	-0.364483198	+		
661	P26373	RPL13	ribosomal protein L13	6137	ENSG00000167526	LEELRVAGIHKKGDSSAEELKLATQLTGPVM	S	0.5	93	16.52781	18.00064	16.46493	14.62628	14.56611	14.00589	15.42141	14.67441	14.19591	2.275707438	2.233882904	2.598366102	-0.364483198	+		
58	A0A0A0MT60	FKBP15	FKBP prolyl isomerase 15	23307	ENSG00000119321	RYVEQSNLMMEKRNNSLQTATENTQARVLHA	S	0.999436	644	16.48223	16.57272	16.47822	16.42983	16.51524	16.33597	16.3559	16.32418	16.27894	1.526630868	0.191378276	0.084040324	0.107337952	+		
1077	Q9H2G2	SLK	STE20 like kinase	9748	ENSG00000065613	NLSISSFLSKTKDSGSISLQETRRQKKTLKK	S	0.99525	779	16.47785	16.73384	16.71143	16.93664	16.58113	16.8114	16.3051	16.44121	16.36924	1.524419999	0.269189199	-0.135349274	0.404538472	+		

93	A0A0G2JMR5	TRPV6	transient receptor potential cation channel subfamily V member 6	55503	ENSG00000276971	LCLWSKFCRWFQRRESWAQSRDEQNLLQQKR	S	0.999578	28	16.30553	15.99667	16.09488	16.50823	16.64976	16.32476	16.69044	16.51297	16.82824	1.811936805	-0.544854482	-0.361890157	-0.182964325	+
628	P11137	MAP2	microtubule associated protein 2	4133	ENSG00000078018	GAEIITQSPGRSSVASPRRLSNVSSSGSINL	S	0.999998	434	16.24574	16.57055	16.17294	16.46432	16.27696	16.35409	16.00626	15.87246	16.09842	1.390380569	0.337361972	-0.035383224	0.372745196	+
483	O00418	EEF2K	eukaryotic elongation factor 2 kinase	29904;101930	ENSG00000103319	KWNLLNSSRLHLPRASAVALEVQRLNALDLE	S	1	500	16.17851	15.79607	15.892	16.13071	15.97296	15.94903	15.30003	15.62062	15.6301	1.683269267	0.438607852	-0.062041918	0.50064977	+
1216	H0Y9Z7	KIF13A	kinesin family member 13A	63971	ENSG00000137177	SSQSIPEKNSKSLCRTGSCSELDACPSKISQ	T	0.536431	700	16.16791	16.26153	16.03476	16.51322	16.49674	16.43086	16.37541	16.0754	16.05439	1.498196753	-0.013669968	-0.325544357	0.31187439	+
270	E7EQR4	EZR	ezrin	7430	ENSG00000092820	KFGDYNKEVHKSGYLSSERLIPQRVMDQHKL	S	0.969128	148	16.16092	16.33534	16.0102	16.44713	16.39369	16.3554	15.96233	16.06388	16.09881	1.780638185	0.127143542	-0.229923884	0.357067426	+
4	A0A087WU14	SCAMP1	secretory carrier membrane protein 1	9522	ENSG00000085365	__XELDRREREMQNLSQHGRKNNWPPLPSNF	S	1	14	16.09988	16.20302	16.17215	16.48955	16.44729	16.4072	16.27354	16.49742	16.53263	1.902507757	-0.276175817	-0.289662043	0.013486226	+
1263	F8VV64	TNS2	tensin 2	23371	ENSG00000111077	TSACQALPPVELRRNTAPVRRIEHLGSTKSL	T	1	91	16.08294	16.18373	15.98946	16.00924	16.12507	16.13144	15.65027	15.78167	15.88815	1.950097657	0.312017123	-0.003203392	0.315220515	+
1406	Q5T0Z8	C6orf132	chromosome 6 open reading frame 132	647024	ENSG00000188112	KWKSDVALNGRQAEATRASPPRSPAEPKGSA	T	0.59408	272	16.07031	15.8209	15.91583	16.24096	15.98749	15.97654	15.6977	15.55294	15.76409	1.614653378	0.264103572	-0.132647196	0.396750768	+
785	Q13131	PRKAA1	protein kinase AMP-activated catalytic subunit alpha 1	5562	ENSG00000132356	PQRSGSVSNYRSCQRSDSDAEAQGKSSEVSL	S	0.576961	506	16.00264	16.06211	16.05569	16.04587	16.15893	16.28311	16.39836	16.27134	16.22366	1.490085229	-0.257640839	-0.122488022	-0.135152817	+
445	I6L9I8	EPN3	epsin 3	55040	ENSG00000049283	GFSRRYGEDYSRSRGSPSSYNSSSSSPRYTS	S	0.752341	182	15.98453	16.03114	15.85475	16.30841	16.30464	16.40926	16.27134	16.32726	16.1441	2.500686364	-0.290757815	-0.383962313	0.093204498	+
1113	Q9NYF3	FAM53C	family with sequence similarity 53 member C	51307	ENSG00000120709	PELPWRPRGLRNLPRSRSQPCDLDARKTGVK	S	0.501714	271	15.96335	15.86733	15.89354	16.07414	16.05698	15.86781	16.25965	16.22935	16.18532	2.266685997	-0.316701253	-0.091571172	-0.225130081	+
900	Q7L7X3	TAOK1	TAO kinase 1	57551	ENSG00000160551	YREEGDPRTRASDPQSPPQVSRHKSHYRNRE	S	0.999969	421	15.95474	15.82068	15.79898	15.89964	15.83158	15.98007	15.52983	15.64374	15.72973	1.720746774	0.223699888	-0.045631727	0.269331614	+
1423	Q86YL5	TDRP	testis development related protein	157695	ENSG00000180190	AGRLVSIRRQSKGHLTDSPEEAE________	T	0.579067	178	15.93724	16.12549	15.91024	15.88546	15.84884	15.84016	15.64555	15.60293	15.83997	1.498718217	0.294843674	0.132836978	0.162006696	+
1161	Q9UQN3	CHMP2B	charged multivesicular body protein 2B	25978	ENSG00000083937	SGKMAKAPSAARSLPSASTSKATISDEEIER	S	0.868748	149	15.88694	15.95042	15.89938	15.52142	15.82078	15.81476	15.49866	15.32203	15.61448	1.71167518	0.433853149	0.193261464	0.240591685	+
480	O00299	CLIC1	chloride intracellular channel 1	1192	ENSG00000213719	GFTIPEAFRGVHRYLSNAYAREEFASTCPDD	S	0.999241	211	15.8063	16.09338	15.93921	16.4084	16.24945	16.13164	15.97262	15.72494	15.80055	1.691872516	0.113593102	-0.316864014	0.430457115	+
179	B1AMA6	MAPKAP1	mitogen-activated protein kinase associated protein 1	79109	ENSG00000119487	HKFGFSTLALVEKYSSPGLTSKESLFVRINA	S	0.965174	78	15.79464	15.988	15.77012	15.83291	15.62933	15.81766	15.41709	15.38495	15.69702	1.32387466	0.351231575	0.090954145	0.26027743	+
115	A0A1B0GTW1	TJP2	tight junction protein 2	9414	ENSG00000119139	SEIEDISEIESNRSFSPEERRHQYSDYDYHS	S	0.998402	474	15.58197	15.53561	15.55315	15.84679	15.64036	15.75455	15.39396	15.47969	15.64219	1.339193443	0.051628431	-0.190323512	0.241951942	+
1299	K7EMD6	SGTA	small glutamine rich tetratricopeptide repeat containing alpha	6449	ENSG00000104969	ATGKEMPQDLRSPARTPPSEEDSAEAERLKT	T	0.997755	81	15.56254	15.61249	15.46894	15.87951	16	15.70393	15.51215	15.29444	15.3865	2.161600742	0.150293668	-0.313154856	0.463448524	+
402	H0YNP1	GLCE	glucuronic acid epimerase	26035	ENSG00000138604	_______MNHVAKQQSEEAFPQEQQKAPPVV	S	1	9	15.43603	15.38812	15.55165	16.10993	16.05036	15.6139	15.768	15.60075	15.72118	1.366851019	-0.2380476	-0.466134071	0.228086472	+
975	Q8TEA8	DTD1	D-aminoacyl-tRNA deacylase 1;D-tyrosyl-tRNA deacylase 1	92675	ENSG00000125821	KEDRSASSGAEGDVSSEREP___________	S	0.763672	205	15.33525	15.16353	15.342	15.57787	15.4829	15.67808	15.37907	15.31667	15.41548	1.863352404	-0.090145111	-0.299354871	0.20920976	+
870	X6R5W5	DENND4C	DENN domain containing 4C	55667	ENSG00000137145	FKQQTPSRTHKERSTSLSALVRSSPHGSLGS	S	0.920905	112	15.3342	15.4198	15.17676	15.62262	15.61053	15.64445	15.17863	15.15956	15.47754	1.591801174	0.038345973	-0.315615972	0.353961945	+
403	H3BSP0	SYT17	synaptotagmin 17	51760	ENSG00000103528	SDTSKSTYSLTRRISSLESRRPSSPLIDIKP	S	0.62752	50	15.30128	15.27641	15.61039	15.80713	15.6228	15.62317	15.29185	15.34253	15.20449	1.655867683	0.116404215	-0.288334529	0.404738744	+
918	S4R3V8	LSR	lipolysis stimulated lipoprotein receptor	51599	ENSG00000105699	GPALTPIRDEEWGGHSPRSPRGWDQEPAREQ	S	1	444	15.23167	15.46512	15.27449	15.81117	15.5527	15.67165	15.47332	15.36977	15.4641	1.733083946	-0.111970901	-0.354748726	0.242777824	+
534	O60784	TOM1	target of myb1 membrane trafficking protein	10043	ENSG00000100284	GRLEDEFDMFALTRGSSLADQRKEVKYEAPQ	S	0.5	330	15.21694	15.65679	15.50609	15.29476	15.4467	15.3402	14.97298	15.10456	15.06811	1.547124007	0.411389669	0.099385262	0.312004407	+
470	K7EQ12	UBE2O	ubiquitin conjugating enzyme E2 O	63893	ENSG00000175931	VKRLLKKQVVRIMSCSPDTQCSRDHSMEDPD	S	0.833074	161	15.03493	14.96366	15.17758	15.17664	14.90153	15.01898	14.69125	14.57406	14.75999	1.949385353	0.383620262	0.026339213	0.357281049	+
508	O43182	ARHGAP6	Rho GTPase activating protein 6	395	ENSG00000047648	QFMLVGHLSSSKSRESSPGPRLGKDLSEEPF	S	0.625052	514	14.95083	14.73651	14.4373	14.71043	14.70169	14.93682	13.92694	14.23751	14.30228	1.749123151	0.55263265	-0.074768066	0.627400716	+
1412	Q6ZVM7	TOM1L2	target of myb1 like 2 membrane trafficking protein	146691	ENSG00000175662	IHTPQRSVPEVDPAATMPRSQSQQRTSAGSY	T	0.99998	127	14.76575	14.32158	14.29935	14.82367	14.78325	14.73783	14.20633	14.05002	14.36646	1.662423251	0.254624685	-0.319356283	0.573980967	+
1208	A0A0C4DFY5	GPRC5C	G protein-coupled receptor class C group 5 member C	55890	ENSG00000170412	KVPSEGAYDIILPRATANSQVMGSANSTLRA	T	0.541056	440	14.66761	14.83289	14.54067	15.21394	15.08804	15.05189	14.46614	14.99691	14.67783	1.331435672	-0.033237139	-0.437566121	0.404328982	+
166	Q5TIG5	AFDN	afadin, adherens junction formation factor	4301	ENSG00000130396	LPGDDRLMKNRADHRSSPNVANQPPSPGGKS	S	0.49999	1172	14.63691	14.57317	14.83546	14.8358	14.80019	15.07652	14.42167	14.27401	14.55991	1.763570259	0.263313611	-0.222326597	0.485640208	+
167	Q5TIG5	AFDN	afadin, adherens junction formation factor	4301	ENSG00000130396	PGDDRLMKNRADHRSSPNVANQPPSPGGKSA	S	0.49999	1173	14.63691	14.57317	14.83546	14.8358	14.80019	15.07652	14.42167	14.27401	14.55991	1.763570259	0.263313611	-0.222326597	0.485640208	+
14	A0A0U1RQL3	KIF1B	kinesin family member 1B	23095	ENSG00000054523	TGIYELSLCKMSDTGSPGMQRRRRKILDTSV	S	0.864605	136	14.59269	15.05142	14.75081	15.25492	15.09482	15.24299	14.76243	14.65015	14.92453	1.457352064	0.019267082	-0.399267515	0.418534597	+
1284	H7C1E7	BCAS1	breast carcinoma amplified sequence 1	8537	ENSG00000064787	KDSSCQTSDSTEKTITPPEPEPTGAPQKGKE	T	0.78025	145	14.24652	14.90928	14.82128	15.32179	15.22128	15.11744	14.81738	14.7121	14.88049	1.308535818	-0.144298553	-0.561144829	0.416846275	+
847	Q5M775	SPECC1	sperm antigen with calponin homology and coiled-coil domains 1	92521	ENSG00000128487	SRERSVPRGPSNPRKSVSSPTSSNTPTPTKH	S	0.782408	50	14.18627	14.30855	14.44113	14.71086	14.02367	14.45828	13.84068	13.77211	13.91036	1.38695149	0.470933914	-0.085618655	0.556552569	+
487	O14494	PLPP1	phospholipid phosphatase 1	8611	ENSG00000067113	HTTLHETPTTGNHYPSNHQP___________	S	0.535104	280	13.98549	13.79563	14.14633	14.55393	14.36427	14.24852	14.08356	13.78433	14.13531	1.330971449	-0.025249799	-0.413090388	0.387840589	+
931	Q8IXS8	FAM126B	family with sequence similarity 126 member B	285172	ENSG00000155744	SAKDKETASAIKSSESPRDSVVRKQYVQQPT	S	0.983798	398	13.9591	14.1317	14.09853	14.57288	14.24667	14.59199	14.47833	14.46218	14.24101	1.519339808	-0.330735524	-0.407405217	0.076669693	+
82	A0A0C4DGH5	CAND1	cullin associated and neddylation dissociated 1	55832	ENSG00000111530	SNPELAAIFESIQKDSSSTNLESMDTS____	S	0.518902	759	13.67364	13.42901	13.14509	12.60652	13.00571	12.33271	13.52332	13.71306	13.11203	1.519070664	-0.033556938	0.767600377	-0.801157316	+
732	P80723	BASP1	brain abundant membrane attached signal protein 1	10409	ENSG00000176788	AAPSSKETPAATEAPSSTPKAQGPAASAEEP	S	0.683065	194	13.47725	13.3655	13.4818	13.55962	13.59526	13.80453	13.15741	13.21616	13.37694	1.96144748	0.191349665	-0.211618106	0.402967771	+
590	P04279	SEMG1	semenogelin 1	6406	ENSG00000124233	QQHGRKANKISYQSSSTEERRLHYGENGVQK	S	0.992934	292	24.71282	23.29292	22.61446	23.44101	23.94708	23.68688	25.24804	24.75817	23.51881	0.443825906	-0.96827507	-0.151594798	-0.816680272	
738	Q02383	SEMG2	semenogelin 2	6407	ENSG00000124157	QEHGHKENKISYQSSSTEERHLNCGEKGIQK	S	0.992934	352	24.41319	23.0127	22.24389	23.1149	23.69487	23.41584	25.00659	24.4954	23.26356	0.468800758	-1.031922658	-0.185276031	-0.846646627	
1259	F5H442	TSG101	tumor susceptibility 101	7251	ENSG00000074319	PSQPPVTTVGPSRDGTISEDTIRASLISAVS	T	0.999022	220	23.467	23.65709	22.96381	23.05087	23.41545	23.88148	23.27938	23.45287	23.31188	0.037194901	0.014590581	-0.086633682	0.101224264	
597	P04279	SEMG1	semenogelin 1	6406	ENSG00000124233	NPSQDQGNSPSGKGISSQYSNTEERLWVHGL	S	0.929067	156	23.41235	22.6058	21.68239	22.45666	24.13305	23.07547	23.11074	23.33974	22.71282	0.260582287	-0.487586975	-0.654876073	0.167289098	
596	P04279	SEMG1	semenogelin 1	6406	ENSG00000124233	NPKQEPWHGENAKGESGQSTNREQDLLSHEQ	S	0.997932	407	23.39153	21.99079	21.52712	22.21886	24.14701	22.55532	22.67663	22.97694	22.39822	0.196312999	-0.380788803	-0.670588811	0.289800008	
598	P04279	SEMG1	semenogelin 1	6406	ENSG00000124233	PSQDQGNSPSGKGISSQYSNTEERLWVHGLS	S	0.890228	157	23.38943	22.58699	21.64619	22.44213	24.10366	23.05676	23.08505	23.32237	22.69273	0.264581496	-0.492516836	-0.659979502	0.167462667	
263	E7EMZ9	TACC2	transforming acidic coiled-coil containing protein 2	10579	ENSG00000138162	EGVVPPASGGGRVQNSPPVGRKTLPLTTAPE	S	1	2181	23.21028	23.33347	22.97486	23.29109	23.3317	23.2157	22.54555	22.29348	23.14875	1.15103997	0.510278702	-0.106623967	0.616902669	
594	P04279	SEMG1	semenogelin 1	6406	ENSG00000124233	KGESGQSTNREQDLLSHEQKGRHQHGSHGGL	S	1	419	22.88826	22.07225	21.39807	22.21919	23.47184	22.51152	22.36054	22.53929	22.25272	0.324801149	-0.264659246	-0.614660263	0.350001017	
443	I3L4C2	BAIAP2	BAI1 associated protein 2;BAR/IMD domain containing adaptor protein 2	10458	ENSG00000175866	NSYATTENKTLPRSSSMAAGLERNGRMRVKA	S	0.997492	399	22.72519	23.42675	22.48136	22.61014	22.77121	23.12874	22.64555	22.71704	22.59049	0.16938614	0.22674497	0.041074753	0.185670217	
298	E9PLL0	MVB12A	multivesicular body subunit 12A	93343	ENSG00000141971	AASQPSKGGLLERTASRLGSRASTLRRNDSI	S	0.999975	96	22.11633	22.1557	21.6479	21.61468	21.80057	22.36589	21.44723	21.86661	22.0211	0.120616992	0.194993973	0.04626147	0.148732503	
301	E9PP50	CFL1	cofilin 1	1072	ENSG00000172757	_____________MASGVAVSDGVIKVFNDM	S	1	3	22.08018	22.1437	21.91554	22.13518	21.97488	22.05474	21.46044	21.52176	22.04297	0.997323729	0.371416092	-0.008459091	0.379875183	
853	Q5SPY9	NPDC1	neural proliferation, differentiation and control 1	56654	ENSG00000107281	YATAKAPGSPAAPRISPGDQRLAQSAEMYHY	S	1	314	22.07411	22.20443	22.0462	22.55541	22.51612	22.08258	21.26201	21.59355	22.24386	0.963177245	0.408442179	-0.27645874	0.68490092	
753	Q02383	SEMG2	semenogelin 2	6407	ENSG00000124157	QEYGHKENKISYQSSSTEERRLNSGEKDVQK	S	0.984772	412	22.0482	20.64315	20.24109	20.93955	21.0879	20.84957	22.4949	22.08877	20.75849	0.419256791	-0.803239822	0.018472672	-0.821712494	
139	H7C560	BRAF	B-Raf proto-oncogene, serine/threonine kinase	673	ENSG00000157764	IELLARSLPKIHRSASEPSLNRAGFQTEDFS	S	0.996661	729	21.47289	21.57828	21.43252	21.46543	21.37055	21.49033	21.03926	20.89844	21.5418	0.754136714	0.334732691	0.052460353	0.282272339	
754	Q02383	SEMG2	semenogelin 2	6407	ENSG00000124157	KISYQSSSTEERRLNSGEKDVQKGVSKGSIS	S	1	420	21.46658	20.66023	19.98843	21.66227	21.62685	20.81222	22.08932	21.71745	21.58628	0.949667466	-1.092606862	-0.662034988	-0.430571874	
577	O96013	PAK4	p21 (RAC1) activated kinase 4	10298	ENSG00000130669	FENMSVTRSNSLRRDSPPPPARARQENGMPE	S	1	104	21.38611	21.50824	21.17585	21.4867	21.42547	21.56946	21.147	21.26859	21.28042	1.097384574	0.124725342	-0.137144089	0.261869431	
600	P04279	SEMG1	semenogelin 1	6406	ENSG00000124233	SEFSQFPHGQKGQHYSGQKGKQQTESKGSFS	S	0.999993	49	21.19544	19.86325	20.34397	20.59714	21.32641	20.71339	20.8247	20.49228	20.09905	0.265586442	-0.004456202	-0.411426544	0.406970342	
245	D6REA1	SIL1	SIL1 nucleotide exchange factor	64374	ENSG00000120725	KSALAKFKEGAEMESSKEDKARQAEVKRLFR	S	0.972351	154	21.13031	20.2012	19.63277	20.71632	21.73863	20.81002	21.18961	21.37761	20.69038	0.593036609	-0.764438629	-0.766896566	0.002457937	
422	H7C155	RAF1	Raf-1 proto-oncogene, serine/threonine kinase	5894	ENSG00000132155	IELLQHSLPKINRSASEPSLHRAAHTEDINA	S	0.994431	500	21.04367	21.21549	21.14532	21.08161	21.32707	21.20759	21.26928	21.44662	21.3098	0.945860776	-0.207075755	-0.070595423	-0.136480331	
482	O00391	QSOX1	quiescin sulfhydryl oxidase 1	5768	ENSG00000116260	FHFLTVQAARQNVDHSQEAAKAKEVLPAIRG	S	1	426	21.0157	20.20896	20.33658	20.93467	21.30073	20.96423	21.41302	21.45884	20.59049	0.733262667	-0.633703868	-0.546129862	-0.087574005	
999	Q96C24	SYTL4	synaptotagmin like 4	94121	ENSG00000102362	KNELLEIKRKGAKRGSQHYSDRTCARCQESL	S	0.999996	55	20.93589	21.24795	20.98231	21.12805	20.97708	21.35842	20.63236	20.97841	20.99238	0.608984954	0.187666575	-0.099135081	0.286801656	
759	Q02383	SEMG2	semenogelin 2	6407	ENSG00000124157	KGKSGQSADSKQDLLSHEQKGRYKQESSESH	S	1	539	20.84384	20.02093	19.31431	20.57126	20.72559	20.07783	20.73595	21.07176	20.06671	0.304092995	-0.565113068	-0.398535411	-0.166577657	
1084	Q9H6S3	EPS8L2	EPS8 like 2	64787	ENSG00000177106	GPQVPLSEPGFRRRESQEEPRAVLAQKIEKE	S	1	240	20.84253	21.19135	20.31296	20.62934	20.74419	21.13402	20.51999	20.56598	20.58545	0.286578263	0.225142797	-0.053567886	0.278710683	
757	Q02383	SEMG2	semenogelin 2	6407	ENSG00000124157	QEHGHKENKMSYQSSSTEERRLNYGGKSTQK	S	0.993707	472	20.81621	19.58186	18.8898	19.83653	19.89736	19.88626	21.33145	20.85087	20.08861	0.661754624	-0.994358699	-0.110766093	-0.883592606	
790	Q13443	ADAM9	ADAM metallopeptidase domain 9	8754	ENSG00000168615	QANPSRQPGSVPRHVSPVTPPREVPIYANRF	S	0.999749	758	20.64059	19.93387	20.35853	20.48207	20.52554	20.53212	20.58003	20.16681	20.21608	0.257722233	-0.009974162	-0.202246984	0.192272822	
611	P04792	HSPB1	heat shock protein family B (small) member 1	3315	ENSG00000106211	PAYSRALSRQLSSGVSEIRHTADRWRVSLDV	S	0.98563	86	20.61504	20.13971	20.28308	20.33734	20.4714	20.40748	20.75735	20.42665	20.8768	0.795309871	-0.340990702	-0.059463501	-0.281527201	
679	P41181	AQP2	aquaporin 2	359	ENSG00000167580	WEEREVRRRQSVELHSPQSLPRGTKA_____	S	0.999936	261	20.57782	20.01059	19.77647	20.5435	20.83854	20.20538	21.11133	20.8042	20.52621	0.916544391	-0.692289352	-0.407512029	-0.284777323	
940	Q8IYJ3	SYTL1	synaptotagmin like 1	84958	ENSG00000142765	GSEPTWLPLQPRVPPSPDDLPSRGLLALSLK	S	1	392	20.45754	20.50533	20.39655	20.54821	20.30819	20.45643	19.92036	19.91654	20.46463	0.914332328	0.352627436	0.015527725	0.337099711	
740	Q02383	SEMG2	semenogelin 2	6407	ENSG00000124157	IHHKGGQAHHGTQNPSQDQGNSPSGKGLSSQ	S	0.999984	143	20.43553	20.14917	20.4867	20.47546	20.55777	20.30519	20.39425	20.46513	20.09275	0.178567916	0.039756775	-0.089008331	0.128765106	
1130	Q9P270	SLAIN2	SLAIN motif family member 2	57606	ENSG00000109171	PRNSPRPSPKQSPRNSPRSRSPARGIEYSRV	S	0.999922	361	20.42706	20.25373	20.14669	20.25072	20.23116	20.54236	19.76124	19.50789	20.44192	0.603630051	0.37214152	-0.065592448	0.437733968	
1368	Q02383	SEMG2	semenogelin 2	6407	ENSG00000124157	GNSPSGKGLSSQCSNTEKRLWVHGLSKEQAS	T	0.999969	162	20.41967	19.91392	20.20586	20.37103	20.34039	20.3721	20.09841	20.25315	20.12502	0.544144895	0.020953496	-0.181355794	0.202309291	
1159	Q9UQN3	CHMP2B	charged multivesicular body protein 2B	25978	ENSG00000083937	AARSLPSASTSKATISDEEIERQLKALGVD_	S	0.99724	158	20.2841	20.63777	20.06421	20.43614	20.21288	20.68569	20.03376	20.18665	20.49297	0.200488075	0.0909036	-0.116208394	0.207111994	
1544	Q53GD3	SLC44A4	solute carrier family 44 member 4	80736	ENSG00000204385	___MGGKQRDEDDEAYGKPVKYDPSFRGPIK	Y	1	13	20.28127	20.56765	20.19051	20.53896	20.76668	20.45	20.36965	20.24841	20.56987	0.54463148	-0.049500148	-0.238737742	0.189237595	
707	P53396	ACLY	ATP citrate lyase	47	ENSG00000131473	NASGSTSTPAPSRTASFSESRADEVAPAKKA	S	0.988857	194	20.26534	20.70109	20.22823	20.7032	20.47388	20.08822	20.78852	20.1561	20.69515	0.085197692	-0.148372014	-0.023550034	-0.124821981	
697	P50443	SLC26A2	solute carrier family 26 member 2	1836	ENSG00000155850	DSYPSGIHLELQRESSTDFKQFETNDQCRPY	S	0.951245	36	20.23491	20.70176	20.60693	21.13915	21.11965	20.60513	20.58454	20.974	20.31462	0.612106884	-0.109851837	-0.44010671	0.330254873	
1343	P29966	MARCKS	myristoylated alanine rich protein kinase C substrate	4082	ENSG00000277443	SPKAEDGATPSPSNETPKKKKKRFSFKKSFK	T	0.999978	150	20.2061	20.19856	19.99935	20.40759	20.42019	20.27515	19.94006	19.88876	20.54793	0.405584665	0.009090424	-0.232970556	0.242060979	
1094	Q9NQT8	KIF13B	kinesin family member 13B	23303	ENSG00000197892	KEQLTGKGKLSRRSISSPNVNRLSGSRQDLI	S	0.976846	1381	20.16681	19.93039	19.97393	20.36624	20.07966	20.02621	19.56666	19.48609	20.16214	0.722104011	0.285410563	-0.133660634	0.419071198	
518	O43581	SYT7	synaptotagmin 7	9066	ENSG00000011347	LCHWCQRKLGKRYKNSLETVGTPDSGRGRSE	S	0.988993	52	20.14868	20.61827	20.22223	20.59814	20.4247	20.38701	19.89267	20.29412	20.2433	0.650911938	0.18635877	-0.140225728	0.326584498	
325	F5GYU8	TMC5	transmembrane channel like 5	79838	ENSG00000103534	PLNNPDYPGTRSNPYSVASRTRPDYPGSLAE	S	0.702908	56	20.0625	20.36965	19.5367	19.98357	20.13796	20.54623	19.98245	19.97771	20.23771	0.180917375	-0.076342265	-0.23296992	0.156627655	
178	B1AM27	UNC13B	unc-13 homolog B	10497	ENSG00000198722	QMADILGQVRGTGNASPDARASAAQDADSVL	S	0.999992	1263	20.05046	20.04966	19.97407	20.06026	19.90144	20.1881	19.77364	19.37237	20.08148	0.601456716	0.282235463	-0.025198619	0.307434082	
327	F5GYU8	TMC5	transmembrane channel like 5	79838	ENSG00000103534	SETPKMTRGVLSRTSSIQPSFRHRSDDPVGS	S	0.935037	270	20.03416	20.30619	19.48909	19.97785	20.07117	20.28275	19.88002	19.8365	20.07457	0.173046109	0.012782415	-0.16744105	0.180223465	
39	A0A0A0MRB5	MADD	MAP kinase activating death domain	8567	ENSG00000110514	SSVIKHSPTVKREPPSPQGRSSNSSENQQFL	S	1	910	20.01563	20.05788	20.03604	20.17998	20.04847	20.06933	19.66811	19.59749	20.04113	1.184921526	0.267606735	-0.062742869	0.330349604	
958	Q8ND76	CCNY	cyclin Y	219771	ENSG00000108100	DKYKDLRRSARKRSASADNLTLPRWSPAIIS	S	0.929281	272	19.95773	20.12452	19.81309	19.85038	19.65405	20.22199	19.37437	19.5162	19.72787	0.998909097	0.425634384	0.056305567	0.369328817	
725	P63092	GNAS	GNAS complex locus	2778	ENSG00000087460	AKYFIRDEFLRISTASGDGRHYCYPHFTCAV	S	0.999995	337	19.90482	20.2756	19.8615	20.10827	20.07705	20.36698	19.69739	20.03322	19.80204	0.77468544	0.169755936	-0.170130412	0.339886347	
1500	A8MT37	GSK3A	glycogen synthase kinase 3 alpha	2931	ENSG00000105723	FGSAKQLVRGEPNVSYICSRYYRAPELIFGA	Y	0.999152	197	19.786	19.82737	19.67822	19.67356	19.65122	19.76132	19.46228	19.31396	19.72531	0.943148235	0.263348262	0.068497976	0.194850286	
603	P04279	SEMG1	semenogelin 1	6406	ENSG00000124233	LHYGENGVQKDVSQRSIYSQTEKLVAGKSQI	S	0.999872	373	19.72161	18.0882	18.65204	18.72534	20.42675	18.21148	19.25926	19.44567	19.02662	0.086322092	-0.423230489	-0.300575256	-0.122655233	
303	E9PP50	CFL1	cofilin 1	1072	ENSG00000172757	DGVIKVFNDMKVRKSSTPEEVKKRKKAVLFC	S	0.991605	24	19.71497	19.87631	19.92119	19.84811	19.73488	19.86744	19.47374	19.39866	19.7914	1.048479538	0.282888412	0.020679474	0.262208939	
43	B6VEX4	ABI1	abl interactor 1	10006	ENSG00000136754	PNDYMTSPARLGSQHSPGRTASLNQRPRTHS	S	0.999999	225	19.70126	19.75807	19.72722	19.86349	19.63171	19.80958	19.63694	19.46276	19.82073	0.313045549	0.08870697	-0.039411545	0.128118515	
1177	Q9Y3R5	DOP1B	DOP1 leucine zipper like protein B	9980	ENSG00000142197	PFKTKSSESPSSSPSSPARKNGGEWDVEKVV	S	0.894964	721	19.6986	19.94664	19.69457	20.37369	19.92129	19.98371	19.40543	19.2786	20.00662	0.846545374	0.216386159	-0.312959035	0.529345194	

741	Q02383	SEMG2	semenogelin 2	6407	ENSG00000124157	QAHHGTQNPSQDQGNSPSGKGLSSQCSNTEK	S	0.997612	149	19.66929	19.4706	19.81717	19.79081	19.82344	19.71087	19.81497	19.82149	19.5745	0.260688529	-0.084631602	-0.122681936	0.038050334
1207	A0A0C4DFY5	GPRC5C	G protein-coupled receptor class C group 5 member C	55890	ENSG00000170412	LRAEDMYSAQSHQAATPPKDGKNSQVFRNPY	T	0.99972	468	19.58804	20.13546	20.2543	20.14793	20.38227	20.26178	20.02025	20.28828	19.86917	0.365919889	-0.066632589	-0.271392822	0.204760234
344	J3QS47	CYB561	cytochrome b561	1534	ENSG00000008283	EQALSMDFKTLTEGDSPGSQ__________	S	0.999974	218	19.52514	19.4342	19.37105	19.7083	19.34588	19.58253	19.23545	19.10736	19.3906	1.01104458	0.198998133	-0.10210228	0.301100413
176	B1AM27	UNC13B	unc-13 homolog B	10497	ENSG00000198722	SMQSYDLDYPERRAISPTSSSRYGSSCNVSQ	S	0.995952	266	19.49835	19.41912	19.34228	19.44552	19.27073	19.71074	18.86967	19.11403	19.45425	0.660952812	0.273934682	-0.055746078	0.329680761
1321	P04279	SEMG1	semenogelin 1	6406	ENSG00000124233	HRVVIHHKGGKAHRGTQNPSQDQGNSPSGKG	T	0.995403	139	19.49213	18.75551	19.05973	19.59803	19.703	19.38581	19.36461	19.22178	18.90777	0.806671006	-0.062264125	-0.459822337	0.397558212
404	H3BSP0	SYT17	synaptotagmin 17	51760	ENSG00000103528	SVHTASEVPLTPRTNSPDGRRSSSDTSKSTY	S	0.986898	27	19.47044	18.94306	19.52499	19.29298	19.401	19.62876	18.94701	18.78126	19.30466	0.70339152	0.301856995	-0.128077189	0.429934184
321	F5GYU8	TMC5	transmembrane channel like 5	79838	ENSG00000103534	HLGEHDGSLDLRSRRSVQEGNPRA_______	S	1	946	19.43194	19.57458	19.16769	19.33486	19.52961	19.78762	19.40241	19.5011	19.60773	0.228709366	-0.112344106	-0.159294764	0.046950658
857	Q5T0Z8	C6orf132	chromosome 6 open reading frame 132	647024	ENSG00000188112	SDVALNGRQAEATRASPPRSPAEPKGSALGP	S	1	275	19.39899	19.77835	18.88244	19.15887	19.38404	19.60382	18.85875	19.23769	19.04011	0.403644333	0.307742437	-0.028981527	0.336723963
750	Q02383	SEMG2	semenogelin 2	6407	ENSG00000124157	QDQEHGRKAHKISYPSSRTEERQLHHGEKSV	S	0.692559	290	19.27292	18.77037	19.21954	18.94598	19.14979	18.52168	19.04855	19.34852	18.67525	0.153730369	0.063503901	0.21512413	-0.151620229
306	H0YCZ2	KNL1	kinetochore scaffold 1	57082	ENSG00000137812	HLETQPVSSKDSGIGSVAGKLNLSPSQYINE	S	0.894144	743	19.2513	18.42062	18.32834	18.69666	19.32356	18.72067	19.19068	18.96203	18.94292	0.299038712	-0.365122477	-0.246876399	-0.118246078
441	I3L4C2	BAIAP2	BAI1 associated protein 2;BAR/IMD domain containing adaptor protein 2	10458	ENSG00000175866	YSPWADRKAAQPKSLSPPQSQSKLSDSYSNT	S	0.985001	358	19.24864	19.54221	19.463	19.56121	19.56514	19.64054	19.2934	19.6395	19.32852	0.50171672	-0.002523422	-0.171012878	0.168489456
883	Q6IWH7	ANO7	anoctamin 7	50636	ENSG00000146205	ASEPGGQQAAACRAGSPAKPRIDFVLVWEED	S	1	103	19.24602	18.97525	19.40304	19.16197	19.23613	18.93134	18.38856	18.64417	19.32069	0.515193468	0.42362531	0.098291397	0.325333913
1523	J3QRU1	YES1	YES proto-oncogene 1, Src family tyrosine kinase	7525	ENSG00000176105	KIADFGLARLIEDNEYTARQGAKFPIKWTAP	Y	0.999992	431	19.15665	19.36841	19.40198	19.55433	19.61698	19.11375	19.15049	20.27367	19.32942	0.153791481	-0.27551206	-0.119342804	-0.156169256
678	P41181	AQP2	aquaporin 2	359	ENSG00000167580	EPDTDWEEREVRRRQSVELHSPQSLPRGTKA	S	0.999926	256	19.11187	19.53794	19.41819	19.67173	19.36438	19.46695	19.78208	20.38058	19.60964	0.969938161	-0.568099976	-0.145018895	-0.42308108
254	E5RJY1	NDRG1	N-myc downstream regulated 1	10397	ENSG00000104419	ASMTRLMRSRTASGSSVTSLDGTRSRSHTSE	S	0.93305	42	19.06231	19.1124	18.94304	19.46573	18.56015	19.03058	19.3398	18.86203	19.30753	0.089197301	-0.130533218	0.020432154	-0.150965373
765	Q02952	AKAP12	A-kinase anchoring protein 12	9590	ENSG00000131016	KDDALELQSHAKSPPSPVEREMVVQVEREKT	S	0.991055	1226	19.06071	18.81822	18.82286	18.96691	18.96899	19.24121	18.37356	18.582	19.1726	0.462278899	0.19120725	-0.158441544	0.349648794
214	C9JI98	TMEM238	transmembrane protein 238	388564	ENSG00000233493	RARRAARAPPPPAAGSRRVRLQLATLEAGPG	S	1	154	18.99562	19.57989	19.00878	19.3865	19.48363	19.77443	18.87574	19.39546	19.28613	0.582978655	0.008986791	-0.353422165	0.362408956
1269	G5E9C7	MAP2K2	mitogen-activated protein kinase kinase 2	5605	ENSG00000126934	FAGWLCKTLRLNQPGTPTRTAV_________	T	0.999874	297	18.99455	18.95988	18.93304	19.31268	19.03674	19.00662	19.00536	18.55925	19.0979	0.411496014	0.07498614	-0.156188965	0.231175105
744	Q02383	SEMG2	semenogelin 2	6407	ENSG00000124157	DQGNSPSGKGLSSQCSNTEKRLWVHGLSKEQ	S	0.958541	160	18.96454	18.56536	18.79446	18.99849	19.20386	18.98696	18.8712	18.93905	18.72674	0.912196507	-0.070873896	-0.288312912	0.217439016
249	E5RJY1	NDRG1	N-myc downstream regulated 1	10397	ENSG00000104419	ASGSSVTSLDGTRSRSHTSEGTRSRSHTSEG	S	0.985401	53	18.95222	19.23713	19.03316	18.98011	18.93114	19.28857	19.17555	18.70691	19.50343	0.020245238	-0.05445989	0.007567724	-0.062027613
710	P57735	RAB25	RAB25, member RAS oncogene family	57111	ENSG00000132698	VLKEIFAKVSKQRQNSIRTNAITLGSAQAGQ	S	1	184	18.93894	19.35688	19.10188	19.449	19.08918	19.2116	18.91739	18.83	19.14594	0.60527485	0.168120066	-0.117361069	0.285481135
1085	Q9H7P6	MVB12B	multivesicular body subunit 12B	89853	ENSG00000196814	YSFRTEQSAAARLPPSPTRCQQIPQS_____	S	0.985726	309	18.90348	19.28107	18.43965	18.82445	18.68051	19.06076	18.51098	18.75401	18.84846	0.131741873	0.170250575	0.019494375	0.1507562
743	Q02383	SEMG2	semenogelin 2	6407	ENSG00000124157	PSQDQGNSPSGKGLSSQCSNTEKRLWVHGLS	S	0.902664	157	18.89626	18.10626	18.36427	18.7271	18.99739	18.69741	18.64899	18.65617	18.3276	0.480934885	-0.088656108	-0.351705551	0.263049444
1312	O43581	SYT7	synaptotagmin 7	9066	ENSG00000011347	RKLGKRYKNSLETVGTPDSGRGRSEKKAING	T	1	58	18.8822	18.96601	18.79839	19.33042	19.00638	18.94795	18.83476	18.62756	19.00418	0.709986455	0.060031891	-0.212715785	0.272747676
38	G8JL96	PTPRS	protein tyrosine phosphatase receptor type S	5802	ENSG00000105426	AILLYKKKPHSKRKDSEPRTKCLLNNADLAP	S	1	886	18.86593	18.70039	18.77201	18.73678	18.60758	18.9356	18.31562	18.62046	18.77053	0.494857001	0.210575104	0.019454956	0.191120148
763	Q02952	AKAP12	A-kinase anchoring protein 12	9590	ENSG00000131016	ICVGSSKKRARRGSSSDEEGGPKAMGGDHQK	S	0.969606	593	18.8639	18.73029	18.75401	18.7292	18.60552	18.94635	18.13576	18.45419	18.72141	0.855886474	0.345617294	0.022378286	0.323239009
1292	J3KSM2	MARVELD3	MARVEL domain containing 3	91862	ENSG00000140832	ERERERDPDRGPRRDTHRDAGPRAGEHGVWE	T	1	93	18.86218	19.02583	18.51303	18.26096	18.39578	19.37893	18.19528	18.9465	19.23006	0.024399798	0.0097332	0.12179184	-0.11205864
742	Q02383	SEMG2	semenogelin 2	6407	ENSG00000124157	NPSQDQGNSPSGKGLSSQCSNTEKRLWVHGL	S	0.946174	156	18.85614	18.33001	18.41823	18.83214	19.1756	18.66553	18.69135	18.86946	18.3656	0.503744146	-0.107343038	-0.356297811	0.248954773
639	P13861	PRKAR2A	protein kinase cAMP-dependent type II regulatory subunit alpha	5576	ENSG00000114302	ARAPASVLPAATPRQSLGHPPPEPGPDRVAD	S	1	58	18.82926	18.47437	18.21694	18.51272	18.50928	18.35814	18.23827	18.35703	18.37216	0.296807894	0.184368134	0.046807607	0.137560527
1112	Q9NXL2	ARHGEF38	Rho guanine nucleotide exchange factor 38	54848	ENSG00000236699	SSVSGDHSGTLRRSQSDRTEYNQKLQEKMTP	S	0.966694	55	18.80625	18.83384	18.91579	18.65515	18.99275	18.79649	18.76244	18.5785	18.91395	0.157148379	0.100326538	0.037158966	0.063167572
102	A0A0J9YWK3	COBL	cordon-bleu WH2 repeat protein	23242	ENSG00000106078	QSEPSLSCQETFRKSSLGNDETDKEKKKFLG	S	0.854822	250	18.79741	19.29681	18.74265	18.73783	18.83818	18.98962	18.49931	18.4831	18.94935	0.439330962	0.301703771	0.090409597	0.211294174
705	P51815	ZNF75D	zinc finger protein 75D	7626	ENSG00000186376	PVYERAVHDQQMLALSEQKRIKHWKMASKLI	S	1	212	18.79341	17.59274	16.70212	18.67428	18.79484	18.71196	17.95333	17.87478	17.52168	0.799212397	-0.087173462	-1.030939738	0.943766276
836	Q4VX76	SYTL3	synaptotagmin like 3	94120	ENSG00000164674	NLFLSLATHVKKLSKSQNDMTSEKHLLATGP	S	0.998832	203	18.7652	18.96305	18.83901	19.06513	18.95228	18.9575	18.53142	18.35565	18.95711	0.900796685	0.241025925	-0.13588651	0.376912435
1040	Q9BW04	C1orf116;SARG	chromosome 1 open reading frame 116	79098	ENSG00000182795	HPPEPQGLGLRSGSYSLPRNIHIARSQNFRK	S	0.743732	133	18.76367	18.97771	18.04265	18.67342	18.87204	18.95932	18.20605	18.27435	18.45956	0.716957461	0.281358083	-0.240247726	0.52160581
331	Q92934	BAD	BCL2 associated agonist of cell death	572	ENSG00000002330	EGMGEEPSPFRGRSRSAPPNLWAAQRYGREL	S	0.981877	99	18.75444	18.91418	18.47279	18.76481	18.60429	18.8278	18.34884	18.32193	18.70867	0.639055158	0.253989538	-0.018498739	0.272488276
659	P25686	DNAJB2	DnaJ heat shock protein family (Hsp40) member B2	3300	ENSG00000135924	GTQEGARGEATKRSPSPEEKASRCLIL____	S	0.999095	313	18.74836	19.11698	18.9235	18.76442	18.80747	18.91389	18.59412	18.54446	18.8681	0.719739399	0.260717392	0.101016363	0.159701029
1068	Q9H0B6	KLC2	kinesin light chain 2	64837	ENSG00000174996	ELLKDGSGRRGDRRSSRDMAGGAGPRSESDL	S	0.925731	508	18.73754	18.86967	18.77475	18.44883	18.30701	18.75701	17.91123	18.26687	18.84846	0.578077028	0.451799393	0.289697647	0.162101746
1465	Q9H2G2	SLK	STE20 like kinase	9748	ENSG00000065613	DIKLADFGVSAKNTRTIQRRDSFIGTPYWMA	T	0.999634	183	18.72161	18.71183	18.54725	18.98123	18.50172	18.99358	18.461	18.26137	18.76956	0.549993663	0.162918091	-0.165281296	0.328199387
0	H0Y394	HDLBP	high density lipoprotein binding protein	3069	ENSG00000115677	DEAGEGREAKDCDPGSPRRCDIIISGRKEK	S	1	753	18.72094	18.55873	18.39515	18.71706	18.67377	18.74616	18.53001	18.30278	18.73271	0.451202534	0.036437988	-0.154056549	0.190494537
595	P04279	SEMG1	semenogelin 1	6406	ENSG00000124233	ERLWVHGLSKEQTSVSGAQKGRKQGGSQSSY	S	0.995653	179	18.7083	17.6245	18.17952	18.20848	18.48231	17.99645	18.31478	18.43041	17.78635	0.008459055	-0.006408056	-0.058311462	0.051903407
259	E7EMZ9	TACC2	transforming acidic coiled-coil containing protein 2	10579	ENSG00000138162	GNSQNIKRKQQDTPGSPDHRDASSIGSVGLG	S	0.999964	41	18.67325	18.83864	18.61945	18.84307	18.86701	18.88429	18.62934	18.37186	18.92239	0.474983299	0.069248835	-0.154343287	0.223592122
852	Q5SPY9	NPDC1	neural proliferation, differentiation and control 1	56654	ENSG00000107281	RLTQKADYATAKAPGSPAAPRISPGDQRLAQ	S	1	307	18.67325	18.1749	18.66553	19.00314	18.67514	18.39912	18.06561	18.0675	18.36359	0.939778266	0.33899498	-0.187909444	0.526904424
1035	Q9BV36	MLPH	melanophilin	79083	ENSG00000115648	HVMASHHSKRRGRASSESQGLGAGVRTEADV	S	0.93686	337	18.65965	19.04022	17.80146	18.13771	18.07629	16.89865	17.41369	18.15581	18.89995	0.37316884	0.343961716	0.79622523	-0.452263514
612	P05060	CHGB	chromogranin B	1114	ENSG00000089199	PTKADTEKWAEGGGHSRERADEPQWSLYPSD	S	1	130	18.65207	18.25266	18.4435	18.88914	19.83444	18.42152	18.90695	19.31179	18.40541	0.412833077	-0.425301234	-0.598952611	0.173651377
140	A0A2R8YCL1	GRB10	growth factor receptor bound protein 10	2887	ENSG00000106070	QPRASGPPRSIQPQVSPRQRVQRSQPVHILA	S	1	142	18.64523	18.68979	17.81697	18.36196	18.97749	18.59602	18.18107	18.43461	18.08114	0.393256234	0.151720047	-0.261159261	0.412879308
599	P04279	SEMG1	semenogelin 1	6406	ENSG00000124233	DQGNSPSGKGISSQYSNTEERLWVHGLSKEQ	S	0.994886	160	18.63778	18.03106	18.51318	18.31814	19.42743	18.30528	18.89119	18.80603	18.85221	0.349098984	-0.455799739	-0.289604187	-0.166195552
649	P18206	VCL	vinculin	7414	ENSG00000035403	MTDQVADLRARGQGSSPVAMQKAQQVSQGLD	S	0.869172	346	18.62432	18.71358	18.50265	18.57533	18.73694	18.66244	18.43123	18.56272	18.58384	0.573186496	0.087586085	-0.044723511	0.132309596
1322	P04279	SEMG1	semenogelin 1	6406	ENSG00000124233	QHGRKANKISYQSSSTEERRLHYGENGVQKD	T	0.949036	293	18.61025	17.0963	16.55481	17.54631	17.32457	17.34525	18.42546	18.01313	16.98504	0.114482139	-0.387419383	0.015082677	-0.40250206
592	P04279	SEMG1	semenogelin 1	6406	ENSG00000124233	LHYGENGVQKDVSQSSIYSQTEEKAQGKSQK	S	0.902554	313	18.6031	17.55802	17.27621	18.52658	19.54353	17.93796	18.84316	19.09738	18.20758	0.588997579	-0.903596878	-0.856912613	-0.046684265
126	A0A286YFF8	MON2	MON2 homolog, regulator of endosome-to-Golgi trafficking	23041	ENSG00000061987	IGPISGMSRPFVRTDSIGEKLGRYSSSEPPI	S	0.919055	1215	18.59798	18.89166	18.69292	19.24795	18.76153	18.90783	18.53351	18.47156	18.87918	0.697857619	0.099438985	-0.244913101	0.344352086
548	O75923	DYSF	dysferlin	8291	ENSG00000135636	RGRLLLSLETKLVEHSEQKVEDLPADDILRV	S	1	554	18.58791	18.2336	17.13521	19.16288	20.18095	18.63863	18.34594	18.81997	18.06713	0.940280315	-0.425439835	-1.341915766	0.916475932
1323	P04279	SEMG1	semenogelin 1	6406	ENSG00000124233	NTEERLWVHGLSKEQTSVSGAQKGRKQGGSQ	T	0.988518	176	18.54121	17.58204	18.07064	18.16845	18.83053	17.93024	18.79468	18.87742	17.99507	0.305263572	-0.491092046	-0.245107651	-0.245984395
357	F8W8V9	KIF1A	kinesin family member 1A	547	ENSG00000130294	QSESCPVVGMSRSGTSQEELRIVEGQGQGAD	S	0.994867	1038	18.47647	18.78893	18.03843	19.05038	18.70198	18.69462	18.36457	18.10631	18.63775	0.680345008	0.065064748	-0.38105456	0.446119308
571	O95816	BAG2	BCL2 associated athanogene 2	9532	ENSG00000112208	KINAKANEGRFCRSSSMADRSSRLLESLDQL	S	0.992457	20	18.46064	18.66035	18.0146	18.65707	18.3719	18.59346	18.84953	18.23888	18.5834	0.159075918	-0.178738912	-0.162284215	-0.016454697
519	O43581	SYT7	synaptotagmin 7	9066	ENSG00000011347	GKRYKNSLETVGTPDSGRGRSEKKAINGTLL	S	0.998974	61	18.4475	18.44904	18.27107	18.50858	18.52352	18.2017	18.32426	18.2018	18.29648	0.38289197	0.115024567	-0.022064209	0.137088776
36	A0A0A0MR12	FAM189A2	family with sequence similarity 189 member A2	9413	ENSG00000135063	RPIRSRRALPPLRTRSKSDPVLHPSEERAAP	S	0.647092	426	18.42653	18.23617	17.83485	18.58138	18.29992	18.32874	18.52638	18.61981	18.05208	0.308254543	-0.233573914	-0.237493515	0.003919601
934	Q8IY63	AMOTL1	angiomotin like 1	154810	ENSG00000166025	KVLQQRSRKDAGKTDSSSLRPARSVPSIAAA	S	0.705951	735	18.40877	18.44581	18.28021	18.87298	18.45571	18.44209	18.52852	18.83161	18.81978	0.872067859	-0.348375956	-0.211996714	-0.136379242
1046	Q9BZL6	PRKD2	protein kinase D2	25865	ENSG00000105287	LMRVVQSVRHTTRKSSTTLREGWVVHYSNKD	S	0.933901	239	18.38368	18.31531	18.37297	18.44516	18.48093	18.6168	17.94923	17.89131	18.56967	0.702720803	0.220580419	-0.156979243	0.377559662
1424	Q8IUC4	RHPN2	rhophilin Rho GTPase binding protein 2	85415	ENSG00000131941	ENDGYFRKGCNPLAQTGRSKLQNQRAALNQQ	T	1	31	18.35754	18.94455	17.97029	18.59157	18.27799	18.75971	18.54185	18.75649	18.47132	0.085644551	-0.16576004	-0.118961334	-0.046798706
1251	E7EX73	EIF4G1	eukaryotic translation initiation factor 4 gamma 1	1981	ENSG00000114867	ERPSQPEGLRKAASLTEDRDRGRDAVKREAA	T	0.965485	1048	18.31871	18.20986	18.25336	18.43835	18.17685	18.31145	18.43766	18.27025	18.36402	0.299238027	-0.0966657	-0.048240662	-0.048425039
589	P04279	SEMG1	semenogelin 1	6406	ENSG00000124233	DQQHGRKANKISYQSSSTEERRLHYGENGVQ	S	0.802382	291	18.31212	17.51276	17.42086	18.14604	18.2219	17.90965	18.63513	18.63158	17.927	0.714837064	-0.649324417	-0.343949	-0.305375417
1510	F5GYU8	TMC5	transmembrane channel like 5	79838	ENSG00000103534	SLAEPNYPRSLSNPDYSGTRSNAYSAASRTS	Y	0.747212	83	18.27435	18.1865	18.13636	18.71485	18.47619	18.12757	18.27139	18.09816	18.10942	0.674642126	0.039414088	-0.240467072	0.279881159
971	Q8TE68	EPS8L1	EPS8 like 1	54869	ENSG00000131037	NILTPYPGPRLHHSQSPARSLNSTPPPPPAP	S	0.992593	545	18.27166	18.55585	18.11396	18.1564	18.00963	18.572	17.97528	18.10144	18.29634	0.20600622	0.189468384	0.067810694	0.121657689
1360	P62937	PPIA	peptidylprolyl isomerase A	5478	ENSG00000196262	ADKVPKTAENFRALSTGEKGFGYKGSCFHRI	T	0.5	41	18.2671	18.41848	18.1564	18.47128	18.43147	18.43787	17.91316	17.97029	18.43909	0.805026912	0.173148473	-0.166214625	0.339363098
723	P62937	PPIA	peptidylprolyl isomerase A	5478	ENSG00000196262	FADKVPKTAENFRALSTGEKGFGYKGSCFHR	S	0.5	40	18.2671	18.41848	18.1564	18.47128	18.43147	18.43787	17.91316	17.97029	18.43909	0.805026912	0.173148473	-0.166214625	0.339363098
1065	Q9H0B6	KLC2	kinesin light chain 2	64837	ENSG00000174996	AEWNGDGSGSLRRSGSFGKLRDALRRSSEML	S	0.991747	545	18.25811	18.40869	18.31571	18.458	18.329	17.98454	17.788	17.63091	18.36995	0.630646212	0.397879918	0.070320129	0.327559789
1340	P17509	HOXB6	homeobox B6	3216	ENSG00000108511	PRKSDCAQDKSVFGETEEQKCSTPVYPWMQR	T	0.545594	118	18.25525	17.66996	17.32659	17.83399	18.71585	18.19186	17.88292	17.98588	17.75734	0.497837313	-0.12477684	-0.496634801	0.371857961
1032	Q9BV36	MLPH	melanophilin	79083	ENSG00000115648	GKDDDSFDRKSVYRGSLTQRNPNARKGMASH	S	0.99727	546	18.22675	18.23865	18.13736	18.21371	18.08949	18.26421	18.003	17.77114	18.17733	0.800701333	0.217095057	0.011780421	0.205314636
739	Q02383	SEMG2	semenogelin 2	6407	ENSG00000124157	TEKRLWVHGLSKEQASASGAQKGRTQGGSQS	S	0.998596	177	18.22553	17.12646	17.24312	17.40923	17.10697	17.41485	17.39507	17.11533	17.27421	0.181851306	0.270167033	0.221353531	0.048813502
1023	Q9BSW2	CRACR2A	calcium release activated channel regulator 2A	84766	ENSG00000130038	AKANTAASRASWKKRSGSVIGKYVDSRGILR	S	0.5	408	18.21831	17.90071	17.91532	18.33921	18.17699	18.05563	17.89983	17.26829	18.31588	0.340109111	0.183446884	-0.179165522	0.362612406
1024	Q9BSW2	CRACR2A	calcium release activated channel regulator 2A	84766	ENSG00000130038	ANTAASRASWKKRSGSVIGKYVDSRGILRSQ	S	0.5	410	18.21831	17.90071	17.91532	18.33921	18.17699	18.05563	17.89983	17.26829	18.31588	0.340109111	0.183446884	-0.179165522	0.362612406
1518	H0YD57	CD82	CD82 molecule	3732	ENSG00000085117	MVLSICLCRHVHSEDYSKVPKY_________	Y	0.999985	51	18.21746	18.29661	19.00865	18.9631	18.42788	18.21461	18.59058	19.90522	18.49334	0.275540235	-0.488806407	-0.027626038	-0.461180369
1121	Q9P206	KIAA1522	KIAA1522	57648	ENSG00000162522	LRKLKRPPPPPRRTHSLHQRGLAVPDGPLGL	S	0.992646	458	18.19205	17.98532	17.9624	18.35806	18.14614	18.46989	18.06897	18.27817	18.07012	0.932503015	-0.0924956	-0.278106054	0.185610453
653	P20337	RAB3B	RAB3B, member RAS oncogene family	5865	ENSG00000169213	_____________MASVTDGKTGVKDASDQN	S	0.991749	3	18.17709	18.60964	18.58435	18.5716	18.42123	18.31057	17.80744	17.81428	18.63764	0.462302382	0.370570501	0.02256012	0.348010381
1225	E9PS67	ZNF707	zinc finger protein 707	286075	ENSG00000274352	GQLPRAAPERTDAKPTAFPCQVLTQRCGRRP	T	0.951874	110	18.15965	18.52482	17.96798	18.36986	18.50974	18.74849	17.81265	18.38751	18.27125	0.626284233	0.06034406	-0.32521375	0.38555781
1172	Q9Y3R5	DOP1B	DOP1 leucine zipper like protein B	9980	ENSG00000142197	LFTARGSPFKTKSSESPSSSPSSPARKNGGE	S	0.977434	714	18.14833	18.31013	18.06645	18.73433	18.27034	18.39678	17.68168	17.7204	18.36705	0.906920651	0.251923243	-0.292185465	0.544108709
1039	Q9BW04	C1orf116;SARG	chromosome 1 open reading frame 116	79098	ENSG00000182795	GKLADLEQEQSSKRLSYQGQSRDKLPRPPCV	S	0.999929	560	18.12571	18.45057	17.97908	18.0963	18.29688	18.42562	18.06865	18.18988	18.16044	0.185952009	0.04545784	-0.087819417	0.133277257
956	Q8ND56	LSM14A	LSM14A mRNA processing body assembly factor;LSM14A, mRNA proce	26065	ENSG00000257103	TASAHLPAPAAVGRRSPVSTRPLPSASQKAG	S	0.98505	175	18.09903	18.25428	18.15482	18.10799	18.20953	18.33865	18.05272	17.79785	18.13245	0.789243136	0.175036748	-0.049348195	0.224384944
1354	P51159	RAB27A	RAB27A, member RAS oncogene family	5873	ENSG00000069974	REKRVVYRASGPDGATGRGQRIHLQLWDTAG	T	1	62	18.09836	17.92347	18.08602	18.27239	17.87676	17.95623	17.78322	17.81834	18.04942	0.352502188	0.152289073	0.00082461	0.151464462
1289	I3L4C2	BAIAP2	BAI1 associated protein 2;BAR/IMD domain containing adaptor protein 2	10458	ENSG00000175866	TLPVRKSVTPKNSYATTENKTLPRSSSMAAG	T	0.967629	388	18.07582	18.3363	17.84537	18.13054	18.07676	18.25709	17.92085	17.93905	17.85355	0.696741073	0.18134435	-0.068969091	0.250313441
440	I3L4C2	BAIAP2	BAI1 associated protein 2;BAR/IMD domain containing adaptor protein 2	10458	ENSG00000175866	EDYSPWADRKAAQPKSLSPPQSQSKLSDSYS	S	0.897917	356	18.07368	18.2803	17.97673	17.87027	18.11502	18.26673	18.08135	18.29778	17.80744	0.018054169	0.048048655	0.026230494	0.021818161
861	Q5T0Z8	C6orf132	chromosome 6 open reading frame 132	647024	ENSG00000188112	PEPPAPALQYLGRQSSPPRNNYSDLRQLPNA	S	0.980583	1011	18.07269	18.5068	17.50858	18.08535	18.36277	18.61619	17.56904	18.10605	17.60552	0.650021859	0.269152959	-0.32541275	0.594565709
1114	Q9NZH0	GPRC5B	G protein-coupled receptor class C group 5 member B	51704	ENSG00000167191	EDVQLPRAYMENKAFSMDEHNAALRTAGFPN	S	1	337	18.06613	18.26174	18.54057	18.41328	18.53012	18.32861	18.30497	18.35784	18.15753	0.30463715	0.016030629	-0.134525299	0.150555929
1036	Q9BVG4	PBDC1	polysaccharide biosynthesis domain containing 1	51260	ENSG00000102390	EGEKGVNNGGEKRADSGEEENTKNGGEKGAD	S	1	181	18.05822	18.32065	18.12787	18.36432	18.38064	18.28861	18.22199	17.9406	18.41316	0.393973937	-0.023000081	-0.17560641	0.152606328
201	H7BZ50	MZT2B	mitotic spindle organizing protein 2B	80097	ENSG00000152082	QPSATRLPKGGGPGKSPTRGST_________	S	0.785494	115	18.05754	17.89149	17.99015	18.0752	17.84751	18.16359	17.51745	17.76824	18.0002	0.659280447	0.217761358	-0.049041748	0.266803106
37	A0A0A0MR12	FAM189A2	family with sequence similarity 189 member A2	9413	ENSG00000135063	IRSRRALPPLRTRSKSDPVLHPSEERAAPVL	S	0.758232	428	18.05011	18.98601	18.73816	18.63084	18.8559	18.75831	18.14549	18.67366	18.8171	0.113543353	0.046013514	-0.156918208	0.202931722

520	O43581	SYT7	synaptotagmin 7	9066	ENSG00000011347	PPPGEDALRSGGAAPSEPGSGGKAGRGRWRT	S	0.984333	118	18.04276	18.37488	18.30086	18.47789	18.51872	18.12156	17.97668	18.2268	18.2984	0.347313338	0.072207769	-0.133225759	0.205433528
1480	Q9ULR3	PPM1H	protein phosphatase, Mg2+/Mn2+ dependent 1H	57460	ENSG00000111110	CEVLTVKKKAGAVTSTPNRNSSKRRSSLPNG	T	0.993562	113	18.02686	17.90177	17.9506	18.19748	17.98187	17.95407	17.88703	17.62464	17.97846	0.637298759	0.129700979	-0.084730148	0.214431127
782	Q12929	EPS8	epidermal growth factor receptor pathway substrate 8	2059	ENSG00000151491	VSKVPANITRQNSSSSDSGGSIVRDSQRHKQ	S	0.979476	662	18.01373	18.48271	17.82588	18.06203	17.97023	18.28424	18.17869	17.96882	17.94238	0.047625376	0.077471415	0.001939138	0.075532277
531	O60664	PLIN3	perilipin 3	10226	ENSG00000105355	VQQQRQEQSYFVRLGSLSERLRQHAYEHSLG	S	0.989062	58	18.00152	17.96093	17.7576	17.79227	17.86575	17.9201	17.59215	17.23341	18.08701	0.344183011	0.269158681	0.047311783	0.221846898
314	F5GYU8	TMC5	transmembrane channel like 5	79838	ENSG00000103534	NSDHPGPRANLNHPGSRKNLEHTSFRINPYA	S	1	184	17.98593	18.2616	17.55869	17.83214	17.97936	18.41691	17.76357	17.93238	17.99026	0.143713494	0.040002823	-0.140729268	0.180732091
1264	F8W1J5	DDX23	DEAD-box helicase 23	9416	ENSG00000174243	DRDASPSKEERKRSRTPDRERDRDRDRKSSP	T	0.94625	25	17.9727	18.15244	17.95146	17.28906	18.43969	16.90348	17.33717	19.56003	18.89705	0.463843751	-0.572552363	0.481452942	-1.054005305
911	Q86SU0	ILDR1	immunoglobulin like domain containing receptor 1	286676	ENSG00000145103	VERRIIHLPPLIRDLSSSRRTSDSLHQQWLT	S	0.721466	251	17.92787	18.03053	17.93387	18.03472	17.77385	18.17402	17.67673	17.88965	17.94003	0.374049565	0.128622691	-0.030106227	0.158728917
983	Q92538	GBF1	golgi brefeldin A resistant guanine nucleotide exchange factor 1	8729	ENSG00000107862	PSYHQNDVSLDRGYTSDSEVYTDHGRPGKIH	S	0.8801	1318	17.90959	18.31823	18.11974	18.18384	18.24632	18.13766	18.0099	18.1983	18.12338	0.1337774	0.005321503	-0.07342275	0.078744253
1443	Q96A22	C11orf52	chromosome 11 open reading frame 52	91894	ENSG00000149300	PQPQQLQQNLPKGHETTGHTYERVLQQQGSQ	T	0.995792	48	17.90201	18.00766	17.56733	17.77166	17.92091	18.39134	17.15403	17.74307	17.91876	0.458499561	0.220382055	-0.202301025	0.42268308
1528	P04279	SEMG1	semenogelin 1	6406	ENSG00000124233	QDQGNSPSGKGISSQYSNTEERLWVHGLSKE	Y	0.999764	159	17.89386	17.51391	18.3173	17.93641	19.03923	18.24284	18.4216	18.31265	18.12303	0.436567691	-0.377406438	-0.49780337	0.120396932
1033	Q9BV36	MLPH	melanophilin	79083	ENSG00000115648	EAKAMAVPYLLRRKFSNSLKSQGKDDDSFDR	S	0.998178	524	17.8922	18.38368	18.35999	18.00152	17.94324	17.94083	17.70826	17.57504	18.2061	0.611625209	0.382156372	0.250094096	0.132062276
7	A0A087WUZ3	SPTBN1	spectrin beta, non-erythrocytic 1	6711	ENSG00000115306	VRRQQEEEERKRRPPSPEPSTKVSEEAESQQ	S	0.999373	2104	17.88566	18.29365	18.07629	18.1895	18.00974	18.145	18.24734	17.82991	18.07394	0.042255008	0.034804026	-0.029542923	0.064346949
771	Q08495	DMTN	dematin actin binding protein	2039	ENSG00000158856	TPFHTSLHQGTSKSSSLPAYGRTTLSRLQST	S	0.998685	264	17.86659	17.94049	17.5278	17.77758	17.55045	18.01051	17.47722	17.18166	17.56822	0.878180189	0.369256973	-0.00121816	0.370475133
343	F5H6E2	MYO1C	myosin IC	4641	ENSG00000197879	KTLFATEDALEVRRQSLATKIQAAWRGFHWR	S	0.998487	712	17.86556	17.88572	17.28609	17.76785	17.60009	18.16069	17.60632	17.71827	17.47255	0.255509368	0.080080668	-0.163750331	0.243830999
1253	E9PFK9	RABGEF1	RAB guanine nucleotide exchange factor 1	27342	ENSG00000154710	EIQEAKAPSPSINRQTSIETDRVSKEFIEFL	T	0.654954	144	17.85757	17.97376	17.81359	17.97779	18.06529	17.95991	18.0228	17.98103	17.85258	0.615200606	-0.07049497	-0.119356791	0.048861821
1281	H3BQF7	IST1	IST1 factor associated with ESCRT-III	9798	ENSG00000182149	YSKEYGKLCRTNQIGTVNDRLMHKLSVEAPP	T	0.999993	143	17.85678	17.76798	17.08752	17.57777	17.56153	17.82277	17.26673	17.34247	17.38886	0.449833393	0.238071442	-0.083262126	0.321333567
605	P04279	SEMG1	semenogelin 1	6406	ENSG00000124233	QRSIYSQTEKLVAGKSQIQAPNPKQEPWHGE	S	1	386	17.82861	17.80007	17.85447	17.96392	18.30341	17.49037	17.80228	18.56993	17.98382	0.239901243	-0.290960948	-0.091517766	-0.199443181
1436	R4GNF9	TSTD1	thiosulfate sulfurtransferase like domain containing 1	100131187	ENSG00000215845	HLVFFCQMGKRGLQATQLARSLGYTGARNYA	T	1	36	17.82034	17.8853	17.93341	18.02388	17.77243	18.02788	16.7432	17.05165	17.85416	1.124914396	0.663345973	-0.061712901	0.725058873
945	Q8N1G4	LRRC47	leucine rich repeat containing 47	57470	ENSG00000130764	VGGRGGGKGKGRAEGSEKEESRRKRRERKQR	S	0.998488	275	17.81535	17.63297	17.52955	17.6435	17.47009	18.15891	17.30981	17.16781	17.99794	0.189131762	0.167433421	-0.098213196	0.265646617
369	F8WBA3	PRKD1	protein kinase D1	5587	ENSG00000184304	LMRVVQSVKHTKRKSSTVMKEGWMVHYTSKD	S	0.970887	429	17.80713	17.68065	17.55772	17.68716	17.79398	18.10077	17.28834	17.42045	17.89469	0.519542214	0.14733696	-0.178806305	0.326143265
255	E5RJY1	NDRG1	N-myc downstream regulated 1	10397	ENSG00000104419	TRLMRSRTASGSSVTSLDGTRSRSHTSEGTR	S	0.997015	45	17.79456	17.9673	17.55502	18.25197	17.59827	18.16943	18.19748	17.58945	18.1516	0.20829449	-0.207216899	-0.234261831	0.027044932
1290	I3L4C2	BAIAP2	BAI1 associated protein 2;BAR/IMD domain containing adaptor protein 2	10458	ENSG00000175866	KSVTPKNSYATTENKTLPRSSSMAAGLERNG	T	0.99999	393	17.79081	18.16663	17.44443	17.81196	17.6287	18.01367	17.57422	17.58776	17.6282	0.311376416	0.203893661	-0.01748848	0.221382141
758	Q02383	SEMG2	semenogelin 2	6407	ENSG00000124157	ELLVYNKNQHQTKNLSQDQEHGRKAHKISYP	S	1	274	17.7788	16.83553	16.66331	17.48982	18.11482	17.11217	18.11319	17.92178	17.48856	0.612107051	-0.748634338	-0.47972552	-0.268908819
1489	Q9Y5K6	CD2AP	CD2 associated protein	23607	ENSG00000198087	PTPPTKASNLLRSSGTVYPKRPEKPVPPPPP	T	0.638188	407	17.75414	17.9581	17.49303	17.76403	17.67204	17.98332	17.0395	17.40532	17.73648	0.709965116	0.341322581	-0.071371078	0.412693659
1513	H0Y3C5	HCK	HCK proto-oncogene, Src family tyrosine kinase	3055	ENSG00000101336	KIADFGLARVIEDNEYTAREGAKFPIKWTAP	Y	0.998029	410	17.75245	18.44803	18.3557	18.21471	18.3905	18.1922	18.04543	19.02975	18.4448	0.24817114	-0.321267446	-0.080411275	-0.240856171
1286	H7C504	DHX34	DExH-box helicase 34	9704	ENSG00000134815	DAFNSSRKDSDQIFHTQAKQGAVLHPTCVFA	T	0.5	220	17.74714	17.84009	17.92202	17.87694	17.82724	18.23173	18.05431	17.623	18.2754	0.155218243	-0.14781634	-0.142218908	-0.005597432
429	H7C504	DHX34	DExH-box helicase 34	9704	ENSG00000134815	PQLAVPDAFNSSRKDSDQIFHTQAKQGAVLH	S	0.5	214	17.74714	17.84009	17.92202	17.87694	17.82724	18.23173	18.05431	17.623	18.2754	0.155218243	-0.14781634	-0.142218908	-0.005597432
1300	Q8TE68	EPS8L1	EPS8 like 1	54869	ENSG00000131037	TPPLQRRPSVRAVISTVERGAGRGRPQAKPI	T	0.999113	202	17.74162	17.80675	17.33908	17.39465	17.26911	17.92126	17.38852	17.40549	17.58674	0.136614705	0.168903987	0.100815455	0.068088531
73	A0A0C4DFY5	GPRC5C	G protein-coupled receptor class C group 5 member C	55890	ENSG00000170412	RGVGYETILKEQKGQSMFVENKAFSMDEPVA	S	1	380	17.74103	18.23524	18.11436	17.99899	17.90536	17.97824	17.52688	18.08005	17.53958	0.516759365	0.314706802	0.069347382	0.245359421
924	Q86YD1	PTOV1	PTOV1 extended AT-hook containing adaptor protein;prostate tumor ove	53635	ENSG00000104960	PASPRGPQPPRIRARSAPPMEGARVFGALGP	S	1	53	17.73542	17.80889	17.64562	17.91514	17.82389	17.79144	17.60682	17.56473	17.76584	1.107099679	0.084177653	-0.113515218	0.197692871
69	A0A0C4DFY5	GPRC5C	G protein-coupled receptor class C group 5 member C	55890	ENSG00000170412	SEGAYDIILPRATANSQVMGSANSTLRAEDM	S	0.996992	443	17.73357	18.0058	17.87963	17.81741	17.98526	17.99629	17.61978	17.91339	17.78238	0.434658727	0.101150513	-0.059984843	0.161135356
485	O00499	BIN1	bridging integrator 1	274	ENSG00000136717	VKAQPSDNAPAKGNKSPSPPDGSPAATPEIR	S	0.837866	265	17.73351	18.10288	17.58152	18.29558	17.99004	18.2523	18.37037	18.17031	17.83584	0.698783248	-0.319537481	-0.37333552	0.05379804
1366	Q02383	SEMG2	semenogelin 2	6407	ENSG00000124157	EHGHKENKISYQSSSTEERHLNCGEKGIQKG	T	0.949036	353	17.72793	16.33896	15.79766	16.99689	16.90018	16.66122	18.16251	17.66407	16.52307	0.359025258	-0.828365008	-0.231245995	-0.597119013
159	A6PVI4	MID2	midline 2	11043	ENSG00000080561	NIIDRFQKASVSGPNSPSESRRERTYRPTTA	S	0.99296	96	17.72381	17.556	17.59157	17.88172	17.47191	17.89693	17.71304	17.73622	17.58593	0.200420808	-0.054604212	-0.126399358	0.071795146
996	Q96C24	SYTL4	synaptotagmin like 4	94121	ENSG00000102362	GSQHYSDRTCARCQESLGRLSPKTNTCRGCN	S	1	69	17.71699	17.58138	17.43363	17.74583	17.60407	17.82768	17.30742	17.28184	17.74103	0.613960336	0.133906047	-0.14852651	0.282432556
1548	Q9BUF7	CRB3	crumbs cell polarity complex component 3;crumbs 3, cell polarity comple	92359	ENSG00000130545	LLVRKLREKRQTEGTYRPSSEEQVGARVPPT	Y	0.998035	93	17.71089	18.402	17.71069	18.02842	17.99236	17.80562	17.76169	18.07363	17.92428	0.003128053	0.021322886	-0.000944773	0.02226766
729	P63218	GNG5	G protein subunit gamma 5	2787	ENSG00000174021	__________MSGSSSVAAMKKVVQQLRLEA	S	0.895098	6	17.70712	17.93635	17.47872	17.66934	17.61884	17.80461	17.22581	17.58241	17.40424	0.872802453	0.303243001	0.009797414	0.293445587
349	F6PQP6	EPN2	epsin 2	22905	ENSG00000072134	LSTSHSEQEYGKAGGSPASYHGSTSPRVSSE	S	0.992903	192	17.69724	17.16428	17.51784	17.57696	17.56956	17.75421	17.48754	17.5203	17.56733	0.309968568	-0.065270742	-0.173789342	0.1085186
969	Q8TDW5	SYTL5	synaptotagmin like 5	94122	ENSG00000147041	QSQEQTRQDAEKSDTSPVAGKKASHDGPKRK	S	0.871907	169	17.68085	17.6499	18.34624	18.61788	18.2865	18.14365	18.01759	17.8119	18.10682	0.726616272	-0.08643659	-0.45700709	0.370570501
260	E7EMZ9	TACC2	transforming acidic coiled-coil containing protein 2	10579	ENSG00000138162	PKMKKTPEKLDNTPASPPRSPAEPNDIPIAK	S	0.999576	2272	17.67749	17.48164	17.50525	17.8646	17.61705	17.49021	17.46866	17.24107	17.63013	0.426822404	0.108175913	-0.102492015	0.210667928
1152	Q9UPU5	USP24	ubiquitin specific peptidase 24	23358	ENSG00000162402	EVGENGRSVDQGGGGSPRKKVALTENYELVG	S	1	1943	17.66477	17.77385	17.72174	17.70077	17.73112	17.561	17.95997	17.85063	17.77751	1.244401774	-0.142583847	0.055821101	-0.198404948
486	O00499	BIN1	bridging integrator 1	274	ENSG00000136717	AQPSDNAPAKGNKSPSPPDGSPAATPEIRVN	S	0.499996	267	17.65746	18.0308	17.48809	18.23009	17.91351	18.18297	18.30274	18.1085	17.75987	0.701683929	-0.331590017	-0.383405685	0.051815669
1245	E7ERH2	SKP1	S-phase kinase associated protein 1	6500	ENSG00000113558	LDVTCKTVANMIKGKTPEEIRKTFNIK____	T	1	131	17.64315	17.88334	17.7198	17.72054	17.86707	17.78647	17.56852	17.50673	17.85751	0.351535773	0.104512533	-0.042598724	0.147111257
1057	Q9C0H2	TTYH3	tweety family member 3	80727	ENSG00000136295	AKYLATSQPRPDSSGSH______________	S	0.999409	351	17.64266	17.877	17.83238	18.20877	18.1169	17.84806	18.32109	18.15926	17.84806	0.799198432	-0.325457255	-0.273896535	-0.05156072
446	J3KNN7	BRAP	BRCA1 associated protein	8315	ENSG00000089234	DELKTTVEERKSSEASPTAQRSKDHSKECIN	S	0.894398	67	17.64047	17.64857	17.39331	17.33158	17.4758	17.5524	17.22081	17.11878	17.5226	0.715817426	0.273386002	0.107522964	0.165863037
1341	P19634	SLC9A1	solute carrier family 9 member A1	6548	ENSG00000090020	INNYLTVPAHKLDSPTMSRARIGSDPLAYEP	T	0.695773	695	17.63389	18.03682	17.69622	17.97606	17.9518	17.67776	17.62342	17.66525	17.7032	0.456308631	0.12501653	-0.079563777	0.204580307
236	F8W6W8	RIMS2	regulating synaptic membrane exocytosis 2	9699	ENSG00000176406	GAKMVAIVGLSRKSRSASQLSQTEAGGKKLR	S	0.995775	1111	17.62271	17.76124	17.9398	17.99573	17.8675	18.1252	17.36504	17.16349	17.88649	0.996338121	0.302906036	-0.221562068	0.524468104
953	Q8N9U0	TC2N	tandem C2 domains, nuclear	123036	ENSG00000165929	KNSQGSNRSLDTITLSGDERDFGRLNVKLFY	S	0.999973	218	17.61906	17.65081	17.49701	17.94872	17.65277	17.32659	17.05632	17.03467	17.83874	0.34746122	0.279052099	-0.053733826	0.332785924
946	Q8N1G4	LRRC47	leucine rich repeat containing 47	57470	ENSG00000130764	LQLEAEEQRKQKKRQSVSGLHRYLHLLDGNE	S	0.997978	431	17.61173	17.34975	17.59696	17.4681	17.7378	17.54019	17.89285	17.71371	17.48927	0.344027728	-0.179128647	-0.062549591	-0.116579056
896	Q6ZVM7	TOM1L2	target of myb1 like 2 membrane trafficking protein	146691	ENSG00000175662	NPRDGFDMFAQTRGNSLAEQRKTVTYEDPQA	S	1	344	17.60942	17.8586	17.42669	17.81516	17.73503	17.92381	17.43339	17.50618	17.74642	0.668843495	0.06957372	-0.193094889	0.26266861
1111	Q9NXL2	ARHGEF38	Rho guanine nucleotide exchange factor 38	54848	ENSG00000236699	VESSVSGDHSGTLRRSQSDRTEYNQKLQEKM	S	0.589535	53	17.60321	17.21608	17.44799	17.4634	17.51268	17.4735	17.23697	17.12374	17.26472	1.147646657	0.213951747	-0.060765584	0.274717331
217	C9JIG9	OXSR1	oxidative stress responsive kinase 1	9943	ENSG00000172939	TISERAKKVRRVPGSSGRLHKTEDGGWEWSD	S	0.629136	325	17.60125	17.39933	17.74064	17.63941	17.73608	17.22901	17.61115	17.84904	17.8278	0.405961437	-0.182260513	0.045570374	-0.227830887
1302	Q8TE68	EPS8L1	EPS8 like 1	54869	ENSG00000131037	KQLQHERRRRQVTQATQQGRGWEVRGRGRSA	T	0.999997	439	17.59332	17.67473	17.06792	17.09558	17.00108	17.68661	16.93802	17.13731	17.45716	0.214284741	0.267826716	0.184232076	0.08359464
535	O60784	TOM1	target of myb1 membrane trafficking protein	10043	ENSG00000100284	RLEDEFDMFALTRGSSLADQRKEVKYEAPQA	S	0.92105	331	17.59091	17.63467	17.5533	17.57208	17.5625	17.5509	17.19576	17.25994	17.56993	1.109948941	0.251081467	0.031134288	0.219947179
1067	Q9H0B6	KLC2	kinesin light chain 2	64837	ENSG00000174996	RSGSFGKLRDALRRSSEMLVKKLQGGTPQEP	S	0.963374	557	17.58762	17.78635	17.56458	17.81428	17.64744	17.30144	17.19306	16.94638	17.869	0.302553523	0.310031891	0.058458328	0.251573563
273	E7ERA6	RNF223	ring finger protein 223	401934	ENSG00000237330	QQVWHTAVPPPRRSSSIASMPRSPSSAGSPR	S	0.971794	20	17.58732	17.40108	17.53731	17.37823	18.25668	17.33752	17.36521	17.7341	17.77475	0.07058299	-0.11611557	-0.148903529	0.032787959
5	A0A087WUV0	AC092835.1	novel C2H2 type zinc finger protein	NA	ENSG00000233757	KPYECHECGKAFSHRSALIRHHIIHTGEKPY	S	0.999909	290	17.58688	16.80864	17.45619	17.23444	17.23248	17.53161	17.59602	17.33787	17.16005	0.025471037	-0.080738703	-0.048938751	-0.031799952
1533	P46059	SLC15A1	solute carrier family 15 member 1	6564	ENSG00000088386	FDEDEKKNRLEKSNPYFMSGANSQKQM____	Y	0.99838	697	17.57393	17.95612	17.66553	17.89113	17.74255	17.85927	17.50649	17.69343	17.79722	0.348689852	0.066144307	-0.099123001	0.165267309
951	Q8N9U0	TC2N	tandem C2 domains, nuclear	123036	ENSG00000165929	LTNSSQRFIQRHDSLSSVPSSSSSRKNSQGS	S	0.742544	193	17.57163	17.43453	17.59776	17.64082	17.56503	17.49436	17.27385	17.2616	17.51129	1.063661703	0.185729345	-0.032093684	0.217823029
809	Q15036	SNX17	sorting nexin 17	9784	ENSG00000115234	KMLRRRVGGTLRRSDSQQAVKSPPLLESPDA	S	0.988704	384	17.56518	17.52932	17.50688	17.64294	17.56115	17.83312	17.23472	17.51345	17.48377	1.049272667	0.123146057	-0.145278931	0.268424988
1347	P46937	YAP1	Yes associated protein 1	10413	ENSG00000137693	FFKPPEPKSHSRQASTDAGTAGALTPQHVRA	T	0.585411	110	17.56503	17.07085	17.09311	17.50974	17.85172	17.17582	17.2291	17.19672	17.47215	0.323771661	-0.056327184	-0.269434611	0.213107427
1272	H0Y572	DOCK9	dedicator of cytokinesis 9	23348	ENSG00000088387	NQEGLGPIVHDRKSQTLPVSRNRTGMMHARL	T	0.975112	44	17.56473	17.45619	16.4812	17.15224	17.2061	17.5414	16.95106	16.98671	17.06076	0.202823424	0.167866389	-0.132539113	0.300405502
500	O15127	SCAMP2	secretory carrier membrane protein 2	10066	ENSG00000140497	FSQGIFSSRTFHRAASSAAQGAFQGN_____	S	0.876017	319	17.55525	17.44152	17.46921	17.48589	17.24405	17.47017	17.02128	16.75929	17.49872	0.762943365	0.395559311	0.088623683	0.306935628
71	A0A0C4DFY5	GPRC5C	G protein-coupled receptor class C group 5 member C	55890	ENSG00000170412	LPRATANSQVMGSANSTLRAEDMYSAQSHQA	S	0.958776	451	17.5539	18.04628	17.78469	18.07577	18.04634	18.06181	17.32703	17.92816	17.60038	0.89260804	0.176434199	-0.266351064	0.442785263
1083	Q9H6S3	EPS8L2	EPS8 like 2	64787	ENSG00000177106	PIEEVSPVSRQSIRNSQKHSPTSEPTPPGDA	S	0.999999	459	17.5536	17.58659	17.16889	17.24042	17.42882	17.81265	17.40956	16.97779	17.30626	0.343329859	0.205155691	-0.057605108	0.262760798
1120	Q9P206	KIAA1522	KIAA1522	57648	ENSG00000162522	____MGNSHHKRKAPSGPRVRSFWRFGRSAK	S	1	12	17.53556	17.62778	17.28564	17.39138	17.26068	17.71384	17.20438	17.18505	17.29302	0.662078436	0.255508423	0.027691523	0.2278169
350	F8VW41	PACS2	phosphofurin acidic cluster sorting protein 2	23241	ENSG00000179364	LKERQAARPQNERANSLDNERCPDARSQLQI	S	1	423	17.51907	17.35699	17.2702	17.28003	17.49763	17.47326	17.39046	17.36675	17.37169	0.060957252	0.005787532	-0.03488795	0.040675481
293	E9PIU8	ECHDC1	ethylmalonyl-CoA decarboxylase 1	55862	ENSG00000093144	______MAKSLLKTASLSGRTKLLHQTGLSL	S	0.660672	10	17.51807	17.34126	17.12364	17.3739	17.04399	17.19075	17.15145	16.92068	17.10452	0.666678853	0.268771489	0.124774297	0.143997192
645	P16455	MGMT	O-6-methylguanine-DNA methyltransferase	4255	ENSG00000170430	GLAGAWLKGAGATSGSPPAGRN_________	S	0.995691	201	17.51121	17.48738	17.46124	17.3833	17.25082	17.4681	16.97062	17.04761	17.45884	0.910716522	0.327586492	0.119206746	0.208379745
97	A0A0J9YVR0	PHACTR4	phosphatase and actin regulator 4	65979	ENSG00000204138	LKNGHTTPIGNARSSSPVQVEEEPVRLASLR	S	0.901104	126	17.50812	17.83381	17.15403	17.58681	17.5226	17.7055	16.85355	16.91625	17.33368	1.159814782	0.464155197	-0.106318156	0.570473353
252	E5RJY1	NDRG1	N-myc downstream regulated 1	10397	ENSG00000104419	MPSASMTRLMRSRTASGSSVTSLDGTRSRSH	S	0.955208	39	17.50207	17.76991	17.51945	17.81134	17.43738	17.67749	17.58344	17.19748	17.83065	0.065859187	0.059954325	-0.044921875	0.1048762
315	F5GYU8	TMC5	transmembrane channel like 5	79838	ENSG00000103534	KLIKLQDMEKKANPSSLVLERREVEQQGFLH	S	0.970723	916	17.49927	18.04772	17.28437	17.50944	17.66823	17.74176	17.48282	17.42972	17.6466	0.081647276	0.090739568	-0.029354731	0.120094299
399	H0YJD9	ACTR10	actin related protein 10	55860	ENSG00000131966	FDVGKTQPPLMKRAFSTEK____________	S	0.958921	146	17.48322	17.46961	17.4298	17.58373	17.17991	17.37679	17.52826	17.29374	17.44127	0.109797178	0.039784749	0.080733617	-0.040948868
955	Q8N9U0	TC2N	tandem C2 domains, nuclear	123036	ENSG00000165929	LYGSVCDLRTNKLPGSPGLSKSMFDLTNSSQ	S	0.936967	168	17.47738	17.25211	17.27485	17.66525	17.4677	17.51453	17.08493	16.79817	17.61676	0.575537852	0.168158849	-0.214380264	0.382539113
1547	Q96A22	C11orf52	chromosome 11 open reading frame 52	91894	ENSG00000149300	LQQNLPKGHETTGHTYERVLQQQGSQERSPG	Y	0.805475	53	17.47405	17.42169	17.50121	17.66726	17.83016	17.531	17.67521	17.8764	17.48613	0.709216409	-0.213593801	-0.210488637	-0.003105164
346	F5H858	PHACTR2	phosphatase and actin regulator 2	9749	ENSG00000112419	GSSHSKKTTGSKASASPSTSSTSSRPKASKE	S	0.701267	112	17.46921	17.6615	17.29563	17.51853	17.47722	17.69853	17.19354	17.21589	17.52986	0.604560752	0.162347158	-0.089314143	0.251661301
762	Q02952	AKAP12	A-kinase anchoring protein 12	9590	ENSG00000131016	ALICVGSSKKRARRGSSSDEEGGPKAMGGDH	S	0.997068	591	17.46579	17.32166	17.3902	17.40574	17.34629	17.56971	16.83923	17.19335	17.50835	0.475518616	0.212242126	-0.048026403	0.260268529
381	H0Y4U4	PAEP	progestagen associated endometrial protein	5047	ENSG00000122133	QLSWKPQGPRTDPASSTARVLVEDDEIMQGF	S	0.814948	90	17.45772	17.15877	17.09857	17.12	17.1325	17.206	17.42119	17.21181	16.60931	0.1076089	0.157578786	0.085517883	0.072060903
677	P40189	IL6ST	interleukin 6 signal transducer	3572	ENSG00000134352	WPNVPDPSKSHIAQWSPHTPPRHNFNSKDQM	S	0.996224	606	17.45395	17.73185	17.54714	17.92584	17.72407	17.79671	17.56428	17.72274	17.62385	1.055507072	-0.059315364	-0.237899144	0.178583781
683	P42858	HTT	huntingtin	3064	ENSG00000197386	DYRWWAEVQQTPKRHSLSSTKLLSPQMSGEE	S	0.999605	1862	17.45105	17.50323	17.5329	17.53966	17.53184	17.54819	17.42054	17.32887	17.58835	0.388316493	0.049812317	-0.044165929	0.093978246
186	F8WCJ4	ACSS2	acyl-CoA synthetase short chain family member 2	55902	ENSG00000131069	RGQEEAGAGGRARSWSPPPEVSRSAHVPSLQ	S	0.625008	30	17.4496	17.64646	17.03843	17.50028	17.18979	17.1866	17.02497	16.92718	17.50425	0.209286975	0.226032257	0.085939407	0.14009285
1441	Q8TDW5	SYTL5	synaptotagmin like 5	94122	ENSG00000147041	VQSQEQTRQDAEKSDTSPVAGKKASHDGPKR	T	0.708853	168	17.44888	17.23921	17.71873	18.09398	17.66185	17.71129	17.56793	17.22825	17.69798	0.667535688	-0.029116948	-0.353436152	0.324319204
1496	A0A0C4DFY5	GPRC5C	G protein-coupled receptor class C group 5 member C	55890	ENSG00000170412	QVMGSANSTLRAEDMYSAQSHQAATPPKDGK	Y	0.978982	459	17.44459	17.89741	17.83109	17.95106	18.03036	17.78641	17.72314	18.14445	17.62164	0.231013406	-0.105380376	-0.198248545	0.092868169
604	P04279	SEMG1	semenogelin 1	6406	ENSG00000124233	GENGVQKDVSQRSIYSQTEKLVAGKSQIQAP	S	0.97233	376	17.43722	16.55171	17.3229	16.81378	17.94552	16.12963	16.74754	16.99281	16.53442	0.100899356	0.345684687	0.140963236	0.204721451
131	Q9Y3X0	CCDC9	coiled-coil domain containing 9	26093	ENSG00000105321	NVAVESEKNLGPSRRSPGTPRPPGASKGGRT	S	0.996292	80	17.42686	17.39171	17.29643	17.33412	17.73476	17.60371	17.27385	17.15343	17.41856	0.854966334	0.089719772	-0.185863495	0.275583267
384	H0Y6A0	ARFGAP3	ADP ribosylation factor GTPase activating protein 3	26286	ENSG00000242247	KKPNQAKKGLGAKKGSLGAQKLANTCFNEIE	S	1	78	17.42366	17.43437	17.49911	17.53898	17.40433	17.58373	17.41138	17.3641	17.50827	0.370360459	0.024462382	-0.056631088	0.08109347

933	Q8IY63	AMOTL1	angiomotin like 1	154810	ENSG00000166025	QKSRTEGRPTVNRANSGQAHKDEALKELKQG	S	1	191	17.41254	17.59827	17.52099	17.90124	17.80738	17.58505	17.32887	17.91876	17.71478	0.428771929	-0.143538157	-0.253960292	0.110422134
172	B0QYA6	EIF3D	eukaryotic translation initiation factor 3 subunit D	8664	ENSG00000100353	VRQKWDQKSQKPRDSSVEVRSDWEVKEE___	S	0.600828	161	17.41229	17.32122	16.9288	17.18679	17.11837	17.12938	16.80725	17.0654	17.23444	0.268100728	0.185071945	0.075920741	0.109151204
206	B9A041	MDH1	malate dehydrogenase 1	4190	ENSG00000014641	TVQQRGAAVIKARKLSSAMSAAKAICDHVRD	S	0.842647	117	17.40133	17.49732	17.48361	17.60168	17.29518	17.48093	17.24516	17.02248	17.37424	0.929054986	0.246794383	0.001489639	0.245304743
891	Q6PKG0	LARP1	La ribonucleoprotein domain family member 1	23367	ENSG00000155506	CPEFVPRQHYQKETESAPGSPRAVTPVPTKT	S	0.968334	440	17.40058	17.21352	17.46905	17.63275	17.31999	17.70793	17.17816	16.77623	17.37611	0.881850702	0.250886281	-0.192504883	0.443391164
1486	Q9Y3Q8	TSC22D4	TSC22 domain family member 4	81628	ENSG00000166925	PSLRVEAEAGGSGARTPPLSRRKAVDMRLRM	T	0.997618	229	17.39381	17.42603	17.14041	17.55749	17.55772	17.27667	17.20572	17.01998	17.47128	0.439628764	0.087759018	-0.143875758	0.231634776
1089	Q9HCS5	EPB41L4A	erythrocyte membrane protein band 4.1 like 4A	64097	ENSG00000129595	APSPVKSFKKAKNENSPDTQRSKSHAPWEEN	S	0.999999	402	17.39222	17.55996	17.29347	17.88131	17.4614	17.68414	17.06245	17.28446	17.44508	1.091401021	0.151220322	-0.260400136	0.411620458
1298	K7EM11	GIPC1	GIPC PDZ domain containing family member 1	10755	ENSG00000123159	QRSAGGRPGSGPQLGTGRGTLRLRSRGPATV	T	0.999993	33	17.39171	17.48707	16.97902	17.39582	17.1442	17.45627	17.14947	17.19912	17.12616	0.279448285	0.127687454	-0.046161652	0.173849106
373	G3V133	TBC1D9B	TBC1 domain family member 9B	23061	ENSG00000197226	EGSGSEERGEEKGTSSPDYRHYLRMWAKEKE	S	0.913949	154	17.3844	16.71666	16.97679	17.09538	16.52864	16.74819	16.6297	16.33303	16.70482	0.69865883	0.470096588	0.23521169	0.234884898
465	K7EJV0	SEPT9;SEPTIN9	septin 9	10801	ENSG00000184640	NSEPSARHVDSLSQRSPKASLRRVELSGPKA	S	0.984868	70	17.37543	17.4403	17.23341	17.556	17.45876	17.43094	17.24051	17.20762	17.36683	1.194407073	0.078058243	-0.132184982	0.210243225
488	O14545	TRAFD1	TRAF-type zinc finger domain containing 1	10906	ENSG00000135148	CNPSRALPSLNTGSSSPRGVEEPDVIFQNFL	S	0.996193	327	17.35501	17.59609	17.24423	17.28591	17.37899	17.42612	16.72294	16.87939	17.6905	0.305722416	0.300831477	0.034772237	0.26605924
182	H0Y5J4	DBNL	drebrin like	28988	ENSG00000136279	AVHPREIFKQKERAMSTTSISSPQPGKLRSP	S	0.815102	198	17.35225	17.19287	16.95072	17.27239	17.4614	17.30857	17.52673	17.41609	17.39607	0.960856302	-0.281016032	-0.182174683	-0.098841349
737	Q02383	SEMG2	semenogelin 2	6407	ENSG00000124157	DQEHGHKENKISYQSSSTEERHLNCGEKGIQ	S	0.802382	351	17.34187	16.48651	15.77082	16.59674	16.65243	16.93848	17.99629	17.57097	17.18515	0.995541466	-1.051069895	-0.196151733	-0.854918162
672	P31949	S100A11	S100 calcium binding protein A11	6282	ENSG00000163191	__________MAKISSPTETERCIESLIAVF	S	0.935499	6	17.33717	17.26664	17.09156	17.20371	17.37271	17.17933	16.94352	16.87879	17.35707	0.395130361	0.171993891	-0.020126343	0.192120234
629	P11229	CHRM1	cholinergic receptor muscarinic 1	1128	ENSG00000168539	VDPEAQAPTKQPPRSSPNTVKRPTKKGRDRA	S	0.814085	322	17.33569	17.66428	17.12252	17.57548	17.65166	17.58908	17.12878	17.1685	17.29572	1.192749988	0.17650032	-0.231241226	0.407741547
1551	Q9H361	PABPC3	poly(A) binding protein cytoplasmic 3	5042	ENSG00000151846	AAAAAATPAVRTVPRYKYAAGVRNPQQHRNA	Y	0.957251	506	17.32887	15.11175	16.50909	17.2121	17.32316	17.03789	17.50036	16.71559	15.9488	0.347578183	-0.405013084	-0.874479612	0.469466527
516	O43520	ATP8B1	ATPase phospholipid transporting 8B1	5205	ENSG00000081923	QRRQQVFRRGVSTRRSAYAFSHQRGYADLIS	S	0.999979	1207	17.3236	17.66907	17.34802	17.46443	17.70584	17.58042	17.06224	17.41245	17.52145	0.470671443	0.114849091	-0.136668523	0.251517614
783	Q12929	EPS8	epidermal growth factor receptor pathway substrate 8	2059	ENSG00000151491	ANITRQNSSSSDSGGSIVRDSQRHKQLPVDR	S	0.999997	667	17.32158	17.91146	17.10728	17.62863	17.43876	17.78864	17.52405	17.31796	17.21788	0.278061404	0.09347407	-0.171904882	0.265378952
1295	K7ENG1	SMAD4	SMAD family member 4	4089	ENSG00000141646	IVHSLMCHRQGGESETFAKRAIESLVKKLKE	T	0.987318	34	17.3192	17.22043	17.14768	17.13501	17.33132	17.14897	17.04612	16.9514	17.09949	1.130263748	0.196767171	0.024003983	0.172763189
1003	Q96JZ2	HSH2D	hematopoietic SH2 domain containing	84941	ENSG00000196684	NRITTKEATSSCPPKSPLGETRQKLWRSLKM	S	0.883305	137	17.31796	17.40831	17.04974	17.60292	17.71706	17.27121	17.02594	17.47603	17.46698	0.430213329	-0.064313889	-0.271722794	0.207408905
286	E9PAK5	DENND4B	DENN domain containing 4B	9909	ENSG00000198837	PGALPVPGPSRSAPSSPAPRRTKQEMKVAQR	S	0.996256	747	17.30581	17.01389	17.24637	17.16595	17.06055	17.19844	17.05441	16.9036	17.19364	0.337523572	0.138139725	0.047044118	0.091095606
469	Q8TE68	EPS8L1	EPS8 like 1	54869	ENSG00000131037	AAALRATQEELQRDRSPAAETPPLQRRPSVR	S	1	182	17.29948	17.463	17.26325	17.27057	17.10983	17.49826	17.21817	17.36333	17.12606	0.162263435	0.106056213	0.049022675	0.057033539
987	X6RAB3	USP6NL	USP6 N-terminal like	9712	ENSG00000148429	RSVGRPSPLASGRRESGAPHRRHEHSPHPQS	S	1	428	17.29769	17.26151	17.10626	17.41905	17.33699	17.48511	17.39297	17.30082	17.38	1.242013521	-0.136114756	-0.191901525	0.055786769
901	Q7L7X3	TAOK1	TAO kinase 1	57551	ENSG00000160551	PMQGVPRGSSMGVRNSPQALRRTASGGRTEQ	S	1	817	17.27785	17.62549	17.16113	17.42095	17.33508	17.48259	17.30421	17.30403	17.12797	0.33422573	0.109422048	-0.058044434	0.167466482
187	B4DNK4	PKM	pyruvate kinase M1/2	5315	ENSG00000067225	EQDVDMVFASFIRKASDVHEVRKVLGEKGKN	S	1	175	17.27621	17.38102	17.14231	17.22835	17.04857	17.0241	17.1083	16.88262	17.25645	0.525595166	0.184057872	0.166172663	0.017885208
1153	Q9UPU5	USP24	ubiquitin specific peptidase 24	23358	ENSG00000162402	WNAYMLFYQRVSDQNSPVLPKKSRVSVVRQE	S	0.999926	2047	17.26545	17.16113	17.14838	17.3756	17.15135	17.17251	16.99092	16.90124	17.23051	0.639791	0.150761286	-0.041500092	0.192261378
3	C9JFU4	TMC4	transmembrane channel like 4	147798	ENSG00000167608	___MQARRAHRQRNASRDQVVYGSGTKTDRW	S	1	13	17.25331	17.60393	17.14838	17.78513	17.29033	17.43852	17.41212	17.41559	17.4685	0.212491496	-0.0968647	-0.169452031	0.072587331
46	E7EMV7	TNIP1	TNFAIP3 interacting protein 1	10318	ENSG00000145901	LMSNGNKEGASGRPGSPKMEGTGKKAVAGQQ	S	0.999615	213	17.25054	17.39548	16.94249	17.6235	17.286	17.28852	17.40225	17.10134	17.45499	0.28689218	-0.123353958	-0.203166962	0.079813004
644	P16152	CBR1	carbonyl reductase 1	873	ENSG00000159228	VNVSSIMSVRALKSCSPELQQKFRSETITEE	S	0.99709	151	17.24693	17.401	17.2703	17.4537	17.57467	17.39012	17.36307	17.00843	17.56317	0.326288094	-0.005483627	-0.166757584	0.161273956
1537	Q02383	SEMG2	semenogelin 2	6407	ENSG00000124157	PSGSSQFPHGQKGQHYFGQKDQQHTKSKGSF	Y	1	48	17.24088	16.80272	17.2702	17.36999	17.82792	17.1836	17.27894	17.03424	16.75942	0.658256738	0.080402374	-0.355901082	0.436303457
718	P61278	SST	somatostatin	6750	ENSG00000157005	AEQDEMRLELQRSANSNPAMAPRERKAGCKN	S	0.720145	92	17.23986	17.89196	17.56317	18.29109	18.2029	17.47104	17.8853	18.1322	17.16457	0.284057912	-0.162363052	-0.423347473	0.260984421
1250	E7EQR4	EZR	ezrin	7430	ENSG00000092820	QLERQQLETEKKRRETVEREKEQMMREKEEL	T	1	332	17.23818	17.58725	16.77944	17.29069	17.36931	17.35216	16.85063	17.13119	17.05357	0.465504507	0.189827601	-0.135763168	0.325590769
442	I3L4C2	BAIAP2	BAI1 associated protein 2;BAR/IMD domain containing adaptor protein 2	10458	ENSG00000175866	AVPAFSQGLDDYGARSMSSADVEVARF____	S	0.890474	542	17.20428	17.36487	16.69152	17.2851	17.09104	17.03134	16.91485	16.62827	16.51844	0.897889553	0.399704615	-0.048931758	0.448636373
283	E7EX40	RAB11FIP1	RAB11 family interacting protein 1	80223	ENSG00000156675	EAKGEIKDSSPSSSPSPKGFRKKHLFSSTEN	S	0.987705	197	17.20228	17.62078	17.3229	17.34187	17.67169	17.58578	17.10329	17.36033	17.44209	0.425977508	0.08008194	-0.151130676	0.231212616
204	B8ZZI9	AFTPH	aftiphilin	54812	ENSG00000119844	HLCMDSVKTSDDEVGSPKEESRKFTNFQSPN	S	0.998656	37	17.1893	17.07848	17.12343	17.33926	17.18389	17.10881	17.23088	16.7516	17.26206	0.158183442	0.048894246	-0.080245336	0.129139582
1201	H0YBE9	SPAG9	sperm associated antigen 9	9043	ENSG00000008294	ELDMDKDLSGYKGSSTPTKGIENKAFDRNTE	T	0.801136	35	17.1893	17.28501	17.257	17.13551	17.12687	17.19537	17.26224	16.9959	17.21267	0.373921786	0.086833954	0.091187795	-0.004353841
979	Q8WUM4	PDCD6IP	programmed cell death 6 interacting protein	10015	ENSG00000170248	ARKTERDELLKDLQQSIAREPSAPSIPTPAY	S	1	712	17.18795	17.51792	17.07148	17.32992	17.60531	17.57408	17.26856	17.43526	16.99336	0.553373612	0.026723226	-0.243983587	0.270706813
850	Q5R3F8	ELFN2	extracellular leucine rich repeat and fibronectin type III domain containing	114794	ENSG00000166897	DAAVTRKTCSVSSSGSIKSAKVFSLDVPDHP	S	0.91899	636	17.18756	17.46052	17.17475	17.22901	17.33717	17.31115	16.83947	17.49436	17.50261	0.002005316	-0.004536947	-0.018162409	0.013625463
253	E5RJY1	NDRG1	N-myc downstream regulated 1	10397	ENSG00000104419	SASMTRLMRSRTASGSSVTSLDGTRSRSHTS	S	0.945203	41	17.18486	17.163	16.71021	17.00207	16.89083	16.97511	16.88095	16.60436	17.2984	0.042777011	0.091452281	0.063353221	0.02809906
767	Q06210	GFPT1	glutamine--fructose-6-phosphate transaminase 1	2673	ENSG00000198380	DHIPILYRTGKDKKGSCNLSRVDSTTCLFPV	S	0.963322	235	17.17641	17.14071	16.62013	17.05579	16.76344	16.98326	16.65522	16.49659	16.87724	0.537804702	0.302735011	0.044918696	0.257816315
177	B1AM27	UNC13B	unc-13 homolog B	10497	ENSG00000198722	YDLDYPERRAISPTSSSRYGSSCNVSQGSSQ	S	0.683271	270	17.17407	17.4165	17.04623	17.3881	17.06739	17.35836	17.16231	17.00064	17.28942	0.152471836	0.061472575	-0.059019089	0.120491664
89	A0A0G2JIW1	HSPA1B	heat shock protein family A (Hsp70) member 1B	3304	ENSG00000232804	NHFVEEFKRKHKKDISQNKRAVRRLRTACER	S	1	255	17.17348	16.95128	17.02085	16.89143	16.96623	17.60646	16.82028	16.67687	17.15254	0.288066863	0.165309906	-0.106169383	0.271479289
418	H3BRH8	SH3GL3	SH3 domain containing GRB2 like 3, endophilin A3	6457	ENSG00000140600	______________MSVAGLKKQFHKASQLF	S	1	2	17.16575	17.5367	16.43468	16.95481	17.08452	17.3342	16.64695	16.91614	16.88869	0.242448465	0.228449504	-0.078800201	0.307249705
1246	E5RJY1	NDRG1	N-myc downstream regulated 1	10397	ENSG00000104419	GYMPSASMTRLMRSRTASGSSVTSLDGTRSR	T	0.993229	37	17.16565	17.54193	17.01748	17.48038	17.28663	17.23641	17.41444	17.04548	17.4735	0.061954344	-0.06945165	-0.09278361	0.02333196
1116	Q9P206	KIAA1522	KIAA1522	57648	ENSG00000162522	RCSLRTLSRCSLHSASPASVRSLGRFSSVSS	S	0.752279	338	17.16516	17.04942	17.23463	17.36948	16.98638	17.31849	17.21514	16.96229	17.11908	0.21150947	0.050903956	-0.075042089	0.125946045
515	O43520	ATP8B1	ATPase phospholipid transporting 8B1	5205	ENSG00000081923	AYAFSHQRGYADLISSGRSIRKKRSPLDAIV	S	0.998447	1223	17.16084	17.60574	17.02637	17.16752	17.63432	17.44548	16.86206	17.1922	17.1947	0.487145348	0.181332906	-0.151455561	0.332788467
473	M0R300	MYO9B	myosin IXB	4650	ENSG00000099331	KSKPCGSPRVQEKPDSPGGSTQIQRYLDAER	S	0.946401	1290	17.16064	17.10185	17.40516	17.47849	17.25967	17.66491	17.31354	17.3018	17.18427	0.699364153	-0.043987274	-0.245136897	0.201149623
820	Q15678	PTPN14	protein tyrosine phosphatase non-receptor type 14;protein tyrosine phos|	5784	ENSG00000152104	HKFYKQNKICTEQSNSPPPIRRQPTWSRSSL	S	0.997787	314	17.15936	17.1765	16.97477	17.16791	17.14897	17.01509	17.05155	17.06981	17.26105	0.017797448	-0.023921967	-0.007111231	-0.016810735
806	Q14669	TRIP12	thyroid hormone receptor interactor 12	9320	ENSG00000153827	VGGKRGRAQTAPTKTSPRNAKKHDELWHDGV	S	0.970531	1157	17.15877	17.26197	17.26334	17.29491	17.38431	17.35294	16.94798	16.87652	17.35139	0.814129729	0.16939799	-0.116027832	0.285425822
1276	H0YE29	ARHGAP1	Rho GTPase activating protein 1	392	ENSG00000175220	LKYDDFLKSTQKSPATAPKPMPPRPPLPNQQ	T	0.682733	180	17.15749	17.16673	16.58498	17.0998	17.39255	17.2139	16.5748	16.12743	16.92347	1.060968588	0.427832921	-0.265685399	0.693518321
471	M0QZD8	AC099489.1;LOC400499	vitellogenin	400499	ENSG00000188897	GILGQAGLRWVTSVRSAASLDGGIQVQKGRV	S	0.871373	843	17.1567	16.73965	15.38606	17.67783	17.45234	16.63112	18.29809	16.95356	16.82637	0.464136549	-0.931871414	-0.826292674	-0.10557874
670	P31947	SFN	stratifin	2810	ENSG00000175793	___________MERASLIQKAKLAEQAERYE	S	1	5	17.14868	17.06581	16.78584	17.27821	16.8681	17.56347	16.96578	16.75199	17.53016	0.165070379	-0.082536697	-0.236483892	0.153947194
716	P61006	RAB8A	RAB8A, member RAS oncogene family	4218	ENSG00000167461	KAKMDKKLEGNSPQGSNQGVKITPDQQKRSS	S	0.999989	185	17.14788	17.42538	17.05505	17.37484	17.36607	17.29742	16.99833	17.16143	16.88548	1.155260291	0.194358826	-0.136669795	0.33102862
795	Q13796	SHROOM2	shroom family member 2	357	ENSG00000146950	ESTPADLGPRAQSPGSPLHARGQDSWPVSSA	S	0.994347	1039	17.1432	16.68647	17.19681	17.01116	17.22308	17.1426	16.66996	16.79525	16.83603	0.921169316	0.241749446	-0.116784414	0.358533859
926	Q8IVM0	CCDC50	coiled-coil domain containing 50	152137	ENSG00000152492	QTRNWEKQSRHQDRLSPKSSQKAGLHCKEVV	S	1	276	17.11888	16.81828	16.30198	16.8007	16.8007	17.20619	16.31465	16.58426	16.71398	0.480326265	0.208751043	-0.189482371	0.398233414
627	P10909	CLU	clusterin	1191	ENSG00000120885	KQTCMKFYARVCRSGSGLVGRQLEEFLNQSS	S	0.951647	100	17.11716	16.93606	16.82389	17.04963	17.55293	16.9788	16.83245	16.87147	16.88214	0.709618343	0.097016652	-0.234750748	0.3317674
630	P11717	IGF2R	insulin like growth factor 2 receptor	3482	ENSG00000197081	RETVISKLTTCCRRSSNVSYKYSKVNKEEET	S	0.798877	2347	17.11705	17.55981	17.27485	17.20581	17.26353	17.28717	17.26142	17.26572	16.8734	0.293970915	0.183726629	0.065069199	0.11865743
248	E5RIA0	SORBS3	sorbin and SH3 domain containing 3	10174	ENSG00000120896	__________MADGGSPFLGRRDFVYPSSTR	S	1	6	17.11665	17.22346	16.82028	17.34447	17.15857	17.15758	17.14897	17.27303	17.20858	0.478871926	-0.156733831	-0.166746775	0.010012945
680	P42356	PI4KA	phosphatidylinositol 4-kinase alpha	5297	ENSG00000241973	TISKKSGMSKKTNRGSQLHKYYMKRRTLLLS	S	0.993518	231	17.11594	17.22412	16.71102	17.1429	16.93341	17.44403	16.75447	16.73925	16.65899	0.989009824	0.299452464	-0.156424205	0.455876668
696	P50443	SLC26A2	solute carrier family 26 member 2	1836	ENSG00000155850	____MSSESKEQHNVSPRDSAEGNDSYPSGI	S	1	12	17.11513	17.40466	17.52014	17.58937	17.33289	17.4906	17.54359	17.64808	17.07159	0.095365268	-0.074442546	-0.124312719	0.049870173
1220	Q13555	CAMK2G	calcium/calmodulin dependent protein kinase II gamma	818	ENSG00000148660	KGAFSVVRRCVKKTSTQEYAAKIINTKKLSA	T	0.983883	37	17.09476	17.1334	16.82252	17.13491	17.1447	17.08846	16.87099	16.90619	16.877	1.153088752	0.132170359	-0.105792363	0.237962723
526	O60343	TBC1D4	TBC1 domain family member 4	9882	ENSG00000136111	DSGFDEQQEFRSRCSSVTGVQRRVHEGSQKS	S	0.721658	318	17.09053	17.19893	16.98715	17.39615	17.42136	17.22062	16.87304	16.71867	17.23762	1.097605332	0.149093628	-0.253843307	0.402936935
1255	F5GYU8	TMC5	transmembrane channel like 5	79838	ENSG00000103534	GSSETPKMTRGVLSRTSSIQPSFRHRSDDPV	T	0.868204	268	17.08618	17.50013	16.81278	17.66837	17.6375	17.35535	17.43591	17.15036	17.3449	0.75391435	-0.177365621	-0.420714696	0.243349075
635	P13716	ALAD	aminolevulinate dehydratase	210	ENSG00000148218	KFASCFYGPFRDAAKSSPAFGDRRCYQLPPG	S	0.905769	214	17.08015	17.09785	17.14051	17.51283	17.27849	17.22449	17.49678	17.16693	17.21409	0.771259685	-0.186429342	-0.232433955	0.046004613
1348	P49006	MARCKSL1	MARCKS like 1	65108	ENSG00000175130	ACSDEGTAQEGKAAATPESQEPQAKGAEASA	T	0.997338	148	17.07932	17.32887	17.14848	17.42857	17.05558	17.16899	16.9704	16.9266	16.85026	1.168052372	0.269802094	-0.032158534	0.301960627
734	P80723	BASP1	brain abundant membrane attached signal protein 1	10409	ENSG00000176788	APASDSKPGSSEAAPSSKETPAATEAPSSTP	S	0.526524	182	17.07713	17.23482	16.94031	17.46619	17.35802	17.20333	17.30269	17.39632	17.22619	1.070052526	-0.2243131	-0.258424123	0.034111023
1395	Q2M3M2	SLC5A9	solute carrier family 5 member 9	200010	ENSG00000117834	RNCPLSELEKEAHESTPEISERPAGECPAGG	T	0.963311	583	17.06834	16.71492	16.91625	17.00701	16.72454	17.02432	16.7009	16.55621	16.79791	0.674327686	0.214827855	-0.018786112	0.233613968
135	P49815	TSC2	TSC complex subunit 2	7249	ENSG00000103197	FDDTPEKDSFRARSTSLNERPKSLRIARPPK	S	0.978181	939	17.06613	16.72267	16.83874	17.27376	17.02118	17.01792	16.61741	16.66052	16.86145	1.283403407	0.162718455	-0.228437424	0.391155879
1400	Q58FG1	HSP90AA4P	heat shock protein 90 alpha family class A member 4, pseudogene	3323	ENSG00000205100	KNQSLRTSAKSTYGWTANMERIMKAQALRDN	T	0.929285	295	17.06087	15.55198	14.67552	15.95333	16.14227	16.05091	17.66893	17.08773	15.91135	0.487568307	-1.126548131	-0.286050479	-0.840497653
297	E9PKE3	HSPA8	heat shock protein family A (Hsp70) member 8	3312	ENSG00000109971	NHFIAEFKRKHKKDISENKRAVRRLRTACER	S	1	235	17.06023	17.23135	16.89546	16.76643	16.80751	17.36042	16.96533	16.76811	16.90195	0.2060097	0.183883031	0.084229787	0.099653244
302	E9PP50	CFL1	cofilin 1	1072	ENSG00000172757	SDGVIKVFNDMKVRKSSTPEEVKKRKKAVLF	S	0.883668	23	17.05049	17.18863	17.1342	17.38482	17.38633	17.30305	16.84928	16.83504	17.40923	0.724565006	0.093256632	-0.233627319	0.326883952
892	Q6PKG0	LARP1	La ribonucleoprotein domain family member 1	23367	ENSG00000155506	VPRQHYQKETESAPGSPRAVTPVPTKTEEVS	S	0.999964	444	17.04196	17.33246	17.27849	17.45491	17.38751	17.40806	17.27203	17.25451	17.30661	1.036321463	-0.060084661	-0.199195226	0.139110565
311	F2Z3C5	PRKCZ	protein kinase C zeta	5590	ENSG00000067606	FQAKRFNRRAYCGQCSERIWGLARQGYRCIN	S	1	118	17.03885	17.27694	16.42305	17.04815	16.88965	17.53344	16.71545	16.61784	17.07755	0.310289551	0.109336217	-0.244127909	0.353464127
503	O15551	CLDN3	claudin 3	1365	ENSG00000165215	EKKYTATKVVYSAPRSTGPGASLGTGYDRKD	S	0.990589	203	17.03703	16.97489	16.70685	16.9187	16.80864	16.77108	16.31651	16.79981	16.56169	0.93818266	0.346916199	0.073449453	0.273466746
494	O14828	SCAMP3	secretory carrier membrane protein 3	10067	ENSG00000116521	LPPPSAPSLQPSRKLSPTEPKNYGSYSTQAS	S	0.87911	50	17.02313	17.58079	17.30108	17.3423	17.12303	16.92451	16.91532	16.77095	16.84549	1.066231751	0.457747142	0.171719233	0.286027908
513	REV__Q6ZUS5	CCP110				EQSRRSLRGSINRIVNESHLDKEHDAVEVAD	S	1	257	17.01727	17.76435	16.96511	17.57186	17.9117	17.49193	16.47932	16.94684	17.39138	0.803927886	0.309728622	-0.409585953	0.719314575
490	O14672	ADAM10	ADAM metallopeptidase domain 10	102	ENSG00000137845	PPQPIQQPQRQRPRESYQMGHMRR_______	S	1	740	17.00996	17.18892	17.47279	17.15234	17.20686	16.92845	17.26865	17.48054	16.96894	0.160094937	-0.015487671	0.128002803	-0.143490473
1535	P57739	CLDN2	claudin 2	9075	ENSG00000165376	FSCSSQRNRSNYYDAYQAQPLATRSSPRPGQ	Y	0.849163	198	16.99888	16.06006	17.29688	16.14725	16.3756	16.00749	17.77507	16.6187	16.46988	0.547192593	-0.169277191	0.608496348	-0.777773539
240	F8W6W8	RIMS2	regulating synaptic membrane exocytosis 2	9699	ENSG00000176406	EAQGPSSYAQRTTNHSPPTPRRSPLPIDRPD	S	0.986902	240	16.99877	16.61956	16.91415	17.01182	16.44758	16.86254	17.14091	16.3138	16.88465	0.019338215	0.064372381	0.070178986	-0.005806605
860	Q5T0Z8	C6orf132	chromosome 6 open reading frame 132	647024	ENSG00000188112	QEVSTPSQARGEGSPSEATRLPTQGARSSAA	S	0.816468	738	16.99877	17.53701	16.77494	17.2547	17.3599	17.7339	17.02237	17.25109	16.94341	0.564569084	0.031281789	-0.345928828	0.377210617
1339	R4GN18	CD46	CD46 molecule	4179	ENSG00000117335	ADGGAEYATYQTKSTTPAEQRG_________	T	0.989943	72	16.99015	17.50207	17.51137	16.96002	17.19152	17.15827	17.5533	17.5785	17.30875	0.804653705	-0.14565595	0.231254578	-0.376910528
614	P05060	CHGB	chromogranin B	1114	ENSG00000089199	PSLELDKMAHGYGEESEEERGLEPGKGRHHR	S	0.999978	405	16.98593	16.98549	17.10503	17.09589	17.66837	17.36205	16.90759	17.27075	17.20486	0.752608018	-0.102252324	-0.349955241	0.247702916
1498	A0A0C4DFY5	GPRC5C	G protein-coupled receptor class C group 5 member C	55890	ENSG00000170412	QVTKSSPEQSYQGDMYPTRGVGYETILKEQK	Y	0.979219	362	16.97388	17.4338	17.3279	17.74544	17.52535	17.42489	17.13029	17.62728	17.41733	0.548375833	-0.146437963	-0.320033391	0.173595428
196	B7Z2Y2	COG2	component of oligomeric golgi complex 2	22796	ENSG00000135775	SVPLGQLREEVLSLRSSVSEGIRAVDERMSK	S	0.976461	45	16.96927	16.87111	16.60041	17.06624	17.00152	16.95413	17.09723	16.79221	16.96353	0.504101094	-0.137390137	-0.193696976	0.056306839
859	Q5T0Z8	C6orf132	chromosome 6 open reading frame 132	647024	ENSG00000188112	PEPDCPGELKATAPASPRLGQSQSQADERAG	S	1	373	16.96669	17.41568	16.46939	16.78953	16.90384	17.11136	16.56138	16.72667	16.43052	0.53034991	0.377728144	0.015673319	0.362054825
31	A0A088AWN2	WDR20	WD repeat domain 20	91833	ENSG00000140153	SVTTPGNSVPPPLPRSNSLPHSAVSNAGSKS	S	0.591105	463	16.96623	17.29948	17.03209	17.11197	17.12535	17.00251	16.70104	16.89368	17.0491	0.661047563	0.217992783	0.019322713	0.198670069
910	Q7Z6Z7	HUWE1	HECT, UBA and WWE domain containing E3 ubiquitin protein ligase 1;H	10075	ENSG00000086758	CIRIALPAPRGSGTASDDEFENLRIKGPNAV	S	0.999291	1907	16.95968	17.08535	16.67066	16.91088	16.90736	16.74675	16.3881	16.43539	16.91064	0.673755866	0.327183406	0.050233841	0.276949565

796	Q13796	SHROOM2	shroom family member 2	357	ENSG00000146950	GQSTPRQADAQCREGSPGSQQHPPSQKAPNP	S	0.682518	974	16.95526	17.12747	16.9259	17.32175	17.0442	17.21219	17.05346	17.02734	17.02821	0.886195444	-0.03345871	-0.189837774	0.156379064
939	Q8IY63	AMOTL1	angiomotin like 1	154810	ENSG00000166025	IASTTAASSAHAKTGSKDSSTQTDKSAELFW	S	0.946241	822	16.95299	17.41922	17.18959	17.86707	17.37975	17.53328	17.25774	17.42308	17.57223	0.82348403	-0.230414073	-0.406101227	0.175687154
23	C9JZJ7	MID1	midline 1	4281	ENSG00000101871	NIIDRFQKASVSGPNSPSETRRERAFDANTM	S	0.997992	96	16.94501	17.10993	17.23482	16.9886	17.17407	17.13571	17.11736	17.1994	17.31681	0.356105365	-0.114605586	-0.002871195	-0.11173439
1330	P10909	CLU	clusterin	1191	ENSG00000120885	QHPPTEFIREGDDDRTVCREIRHNSTGCLRM	T	1	250	16.94421	16.54839	16.86084	17.1437	17.52428	17.15482	17.18756	17.09723	16.77211	1.086695071	-0.234488169	-0.489788055	0.255299886
1525	K7EMR2	SLC44A2	solute carrier family 44 member 2	57153	ENSG00000129353	________MGDERPHYYGKHGTPQKYDPTFK	Y	0.942768	8	16.94054	17.47912	17.46643	17.67204	17.64336	17.35793	17.54502	18.27812	17.36564	0.438745874	-0.434233348	-0.262415568	-0.17181778
1453	Q9BV36	MLPH	melanophilin	79083	ENSG00000115648	DDDSFDRKSVYRGSLTQRNPNARKGMASHTF	T	0.999691	548	16.93526	16.93445	17.19873	17.22015	16.36104	16.81728	16.41673	15.95706	17.33848	0.257026728	0.452058474	0.22332255	0.228735924
784	Q12929	EPS8	epidermal growth factor receptor pathway substrate 8	2059	ENSG00000151491	QNSSSSDSGGSIVRDSQRHKQLPVDRRKSQM	S	0.998918	672	16.93341	17.04868	16.81253	17.0898	16.86701	17.29095	17.14549	16.90419	17.00635	0.274341514	-0.087135951	-0.151049296	0.063913345
317	F5GYU8	TMC5	transmembrane channel like 5	79838	ENSG00000103534	PDYSEFQSHPYHRASSRQPDYPGSQRNPDFA	S	0.843845	123	16.91275	17.15758	16.58571	16.99347	17.09486	17.01236	16.89238	16.89202	16.85099	0.309871098	0.006884257	-0.148217519	0.155101776
613	P05060	CHGB	chromogranin B	1114	ENSG00000089199	QAPEDLEWERYRGRGSEEYRAPRPQSEESWD	S	0.999286	367	16.90771	15.64872	16.41429	16.61094	18.0215	16.38512	16.64442	17.02356	16.0535	0.291044476	-0.250251452	-0.682277997	0.432026545
615	P05060	CHGB	chromogranin B	1114	ENSG00000089199	KEELVARSETHAAGHSQEKTHSREKSSQESG	S	0.999215	225	16.90642	16.17802	15.74128	16.17849	17.98593	16.36116	16.7125	17.00843	16.17954	0.196589432	-0.358247439	-0.566617012	0.208369573
354	Q15366	PCBP2	poly(rC) binding protein 2	5094	ENSG00000197111	IIGRQGAKINEIRQMSGAQIKIANPVEGSTD	S	1	287	16.90395	16.94707	16.85209	17.05473	16.7055	16.8031	16.49589	16.46004	16.7432	1.286825176	0.334659576	0.046593984	0.288065592
904	Q7Z6J0	SH3RF1	SH3 domain containing ring finger 1	57630	ENSG00000154447	SKKEKKGLLKLLSGASTKRKPRVSPPASPTL	S	0.978803	727	16.89936	17.20438	16.61669	16.86508	16.98371	16.90042	16.55537	16.89758	16.60397	0.448654161	0.221167247	-0.009592056	0.230759303
420	H7BYP9	MCF2L	MCF.2 cell line derived transforming sequence like	23263	ENSG00000126217	ALAKSPCPSPGIRRGSENSSSEGGALRRGPY	S	0.99994	220	16.89416	16.93549	16.75981	16.88703	16.82972	16.9063	16.63722	16.8906	16.56686	0.727423727	0.164927165	-0.011201223	0.176128387
1081	Q9H4A6	GOLPH3	golgi phosphoprotein 3	64083	ENSG00000113384	_______MTSLTQRSSGLVQRRTEASRNAAD	S	0.70733	9	16.87328	16.91508	16.99601	17.07368	16.87424	17.68695	16.71585	16.76176	17.06339	0.530184921	0.081120809	-0.283500036	0.364620845
1351	P50150	GNG4	G protein subunit gamma 4	2786	ENSG00000168243	______MKEGMSNNSTTSISQARKAVEQLKM	T	0.840894	10	16.87099	16.78481	16.24234	17.08119	16.7272	16.98671	16.35973	16.42942	16.69398	0.78653434	0.138336182	-0.298988342	0.437324524
70	A0A0C4DFY5	GPRC5C	G protein-coupled receptor class C group 5 member C	55890	ENSG00000170412	DIILPRATANSQVMGSANSTLRAEDMYSAQS	S	0.839856	448	16.85842	17.15135	16.90806	17.45683	17.16133	17.16516	17.25663	17.11695	17.10349	0.942305993	-0.186414083	-0.288497289	0.102083206
894	Q6W2J9	BCOR	BCL6 corepressor	54880	ENSG00000183337	EVPEDPLLKAKRRRVSKDDWPEREMTNSSSN	S	1	1166	16.85599	16.98982	16.72694	17.04473	17.21987	17.03853	16.42256	16.75251	16.8652	1.273870309	0.177489599	-0.243465424	0.420955022
1140	Q9UBY5	LPAR3	lysophosphatidic acid receptor 3	23566	ENSG00000171517	YVKRKTNVLSPHTSGSISRRRTPMKLMKTVM	S	0.918255	227	16.85404	17.04942	16.7204	17.10758	16.88405	16.83455	16.53036	16.75591	16.66066	0.998439468	0.225645701	-0.06743749	0.293083191
264	E7ESD9	RAD50	RAD50 double strand break repair protein	10111	ENSG00000113522	LQFRDVNGELIAVQRSMVCTQKSKKTEFKTL	S	0.5	99	16.84561	16.29022	16.32011	16.78902	17.07932	16.47708	16.42185	16.49718	16.41287	0.55828248	0.041347504	-0.296492894	0.337840398
1248	E7ESD9	RAD50	RAD50 double strand break repair protein	10111	ENSG00000113522	DVNGELIAVQRSMVCTQKSKKTEFKTLEGVI	T	0.5	103	16.84561	16.29022	16.32011	16.78902	17.07932	16.47708	16.42185	16.49718	16.41287	0.55828248	0.041347504	-0.296492894	0.337840398
621	P07900	HSP90AA1	heat shock protein 90 alpha family class A member 1	3320	ENSG00000080824	YPITLFVEKERDKEVSDDEAEEKEDKEEEKE	S	1	231	16.83738	17.09332	16.92856	17.0868	17.01672	16.7761	16.69724	16.74832	17.00174	0.325140331	0.137320201	-0.006786346	0.144106547
52	Q5TBM3	HSPH1	heat shock protein family H (Hsp110) member 1	10808	ENSG00000120694	NTCEPVVTQPKPKIESPKLERTPNGPNIDKK	S	1	73	16.83096	16.81303	16.78059	16.88536	16.6647	16.8856	16.42874	16.10556	16.85014	0.749337376	0.346716563	-0.003692627	0.35040919
1050	Q9C0C2	TNKS1BP1	tankyrase 1 binding protein 1	85456	ENSG00000149115	ETTQARTEAQDLCRASPEPPGPESSSRWLDD	S	0.999973	672	16.8296	17.00185	16.73991	17.34074	16.8291	16.76513	16.94592	16.66108	16.75695	0.239098389	0.069135666	-0.12120374	0.190339406
646	P17812	CTPS1	CTP synthase 1	1503	ENSG00000171793	SVGRLSHYLQKGCRLSPRDTYSDRSGSSSPD	S	1	331	16.8291	16.70225	16.91858	16.75081	16.65102	16.95311	16.9281	16.71102	17.1948	0.267099176	-0.127993266	0.031665802	-0.159659068
19	A0A087WZ40	MAP7	microtubule associated protein 7	9053	ENSG00000135525	RENVLFLTSGTRRAVSPSNPKARQPARSRLW	S	0.996703	345	16.82712	16.70428	17.14589	16.87328	16.72	16.78328	17.04356	17.21675	16.88322	0.596627114	-0.155409495	0.100245794	-0.255655289
222	C9JUG3	SSH3	slingshot protein phosphatase 3	54961	ENSG00000172830	TPRFRKVVRQASVHDSGEEGEA_________	S	0.99997	388	16.82327	16.5294	16.84916	16.93606	16.7563	16.90654	16.62899	16.82252	16.49883	0.553264177	0.083829244	-0.132356008	0.216185252
1363	P80723	BASP1	brain abundant membrane attached signal protein 1	10409	ENSG00000176788	EKDKKAEGAATEEEGTPKESEPQAAAEPAEA	T	0.999987	36	16.8129	16.9677	16.77017	17.12727	16.51759	16.60541	16.37111	16.5019	16.66996	0.635755549	0.335933685	0.100168864	0.235764821
120	H0YI78	KIF21A	kinesin family member 21A	55605	ENSG00000139116	ISRQSSLSEKKIPEPSPVTRRKAYEKAEKSK	S	0.796383	280	16.80032	16.82078	16.58571	17.07148	16.69289	16.54398	16.28077	16.153	16.95776	0.344743447	0.271759033	-0.033849716	0.305608749
805	Q14515	SPARCL1	SPARC like 1	8404	ENSG00000152583	TEPGEHQEAKKAENSSNEEETSSEGNMRVHA	S	0.872479	289	16.79208	16.41287	16.62742	16.5032	17.25608	16.6454	16.42405	16.31891	16.56547	0.505409363	0.174641927	-0.190774282	0.365416209
1274	H0YBE8	WWC1	WW and C2 domain containing 1	23286	ENSG00000113645	MARKRLEKDLQAARDTQSKALTERLKLNSKR	T	0.999546	164	16.78366	16.76474	16.62956	17.38423	16.7889	16.50442	16.68401	17.32676	17.25866	0.34039348	-0.363822937	-0.166526159	-0.197296778
218	Q9H2U9	ADAM7	ADAM metallopeptidase domain 7	8756	ENSG00000069206	KPASKDSRGIADPNQSAK_____________	S	1	752	16.76707	15.81057	16.68017	16.38605	15.70631	16.41886	17.41072	16.64386	15.6324	0.118692954	-0.143053055	0.248866717	-0.391919772
666	P29966	MARCKS	myristoylated alanine rich protein kinase C substrate	4082	ENSG00000277443	ASSTSSPKAEDGATPSPSNETPKKKKKRFSF	S	0.962017	145	16.76344	16.92926	16.3094	17.02778	16.62085	16.79716	16.60528	16.37434	16.89995	0.168672751	0.040847143	-0.147891363	0.188738505
862	Q5T0Z8	C6orf132	chromosome 6 open reading frame 132	647024	ENSG00000188112	CFGPQPGGPEMRRVNSAGRAPPGGLHAPRLS	S	1	1106	16.75955	16.55531	15.93857	16.05437	15.88877	16.76176	15.88701	16.07602	15.90299	0.436759424	0.462469737	0.182843208	0.279626528
749	Q02383	SEMG2	semenogelin 2	6407	ENSG00000124157	HGEKSVQKDVSKGSISIQTEEKIHGKSQNQV	S	0.962368	315	16.75525	16.21968	16.13799	16.9333	16.80196	16.32481	16.79004	17.11766	16.31035	0.355298563	-0.36837705	-0.315716426	-0.052660624
1437	R4GNF9	TSTD1	thiosulfate sulfurtransferase like domain containing 1	100131187	ENSG00000215845	KRGLQATQLARSLGYTGARNYAGAYREWLEK	T	0.999989	45	16.75434	16.80259	16.70171	16.77841	16.58295	16.96815	16.28831	16.46936	16.73991	0.748827563	0.253688812	-0.023622513	0.277311325
964	Q8NHG8	ZNRF2	zinc and ring finger 2	223082	ENSG00000180233	SPEDGGGGRDRPVGGSPGGPRLVIGSLPAHL	S	1	135	16.75356	16.90994	16.91275	17.09558	16.93203	16.97096	16.5586	16.60222	16.91882	1.085179351	0.165536245	-0.140775681	0.306311925
174	B1AKL4	EIF4ENIF1	eukaryotic translation initiation factor 4E nuclear import factor 1	56478	ENSG00000184708	HMHSQLEHRPSQRSSSPVGLAKWFGSDVLQQ	S	0.9905	927	16.75199	16.46229	16.9938	16.67535	16.73912	16.81753	16.74531	16.51038	17.0457	0.006877784	-0.031098684	-0.007973353	-0.023125331
1009	Q96KP4	CNDP2	carnosine dipeptidase 2	55748	ENSG00000133313	GSEIPLPPILLGRLGSDPQKKTVCIYGHLDV	S	1	87	16.74491	16.43793	16.14579	16.49755	16.4533	16.62228	15.8472	16.39138	16.38102	0.457611132	0.236344655	-0.081500371	0.317845027
363	H0Y5T1	CLASP1	cytoplasmic linker associated protein 1	23332	ENSG00000074054	GKKECDIVSRDGGAASPATEGRGGSEVEGGR	S	0.638146	960	16.74386	16.62214	16.76202	16.52966	16.69683	16.47013	16.53057	16.34878	16.64456	0.744674866	0.201367696	0.143796921	0.057570775
309	F2Z2K0	NSFL1C	NSFL1 cofactor	55968	ENSG00000088833	EDEEEEEGQRFYAGGSERSGQQIVGPPRKKS	S	0.999998	99	16.73727	17.1206	16.83393	16.9668	17.02204	16.82848	16.55378	16.82575	16.97017	0.244973703	0.11403211	-0.041840871	0.155872981
318	F5GYU8	TMC5	transmembrane channel like 5	79838	ENSG00000103534	ANLNHPGSRKNLEHTSFRINPYADSLGKPDY	S	0.970462	192	16.73278	17.34421	16.75669	16.96736	17.12918	17.3243	16.72973	16.87616	17.24971	0.19902486	-0.007305781	-0.195725123	0.188419342
1483	Q9UPX8	SHANK2	SH3 and multiple ankyrin repeat domains 2	22941	ENSG00000162105	CVEEVQCKPRDSQAETRADRSKKLFRHYTVG	T	0.999535	594	16.72933	17.21086	16.69112	16.79373	16.85939	16.98125	16.79182	17.02821	16.58493	0.051839717	0.075447083	-0.001022339	0.076469421
1509	E9PP50	CFL1	cofilin 1	1072	ENSG00000172757	FLWAPELAPLKSKMIYASSKDAIKKKFQGIK;IFWAF	Y	0.999925	117	16.7292	16.7059	16.97544	16.98882	16.97735	16.67314	16.00035	16.42292	16.96138	0.545279849	0.34196345	-0.076254527	0.418217977
496	O14896	IRF6	interferon regulatory factor 6	3664	ENSG00000117595	YEMFSGDFTRSFDSGSVRLQISTPDIKDNIV	S	0.998816	323	16.7288	16.8516	16.32745	16.86266	16.77674	16.93353	16.6197	16.54346	16.73503	0.571305945	0.003220876	-0.221694311	0.224915187
101	A0A0J9YWK3	COBL	cordon-bleu WH2 repeat protein	23242	ENSG00000106078	VNKRSNSKGCLTTPNSPSMHSRSLTLGPSLS	S	0.931229	284	16.7264	17.63517	16.96229	17.13039	17.18699	17.23201	17.19864	16.84904	17.48377	0.020763892	-0.069196065	-0.075175603	0.005979538
502	Q5TD90	KPNA5	karyopherin subunit alpha 5	3841	ENSG00000196911	_____________MASPGKDNYRMKSYKNKA	S	0.999997	3	16.72107	16.92207	16.59815	16.9266	16.86423	16.98782	16.56994	16.49607	16.83071	0.94874035	0.11485672	-0.179117203	0.293973923
458	J3QR09	RPL19	ribosomal protein L19	6143	ENSG00000108298	EERLQAKKEEIIKTLSKEEETKK________	S	0.970199	186	16.71706	17.81691	16.92555	16.54688	16.94524	16.21877	17.26508	16.9145	17.30572	0.662889885	-0.008595149	0.582878113	-0.591473262
1008	Q96KP1	EXOC2	exocyst complex component 2	55770	ENSG00000112685	VLGHLSQTASLKRGSSFQSGRDDTWRYKTPH	S	0.825746	432	16.71331	16.68127	16.27421	16.63069	16.86206	16.86979	16.27503	16.45505	16.70131	0.635332078	0.079130809	-0.231250127	0.310380936
1502	B7Z613	GPM6B	glycoprotein M6B	2824	ENSG00000046653	SSNWAYLKDASKMQAYQDIKAKEEQELQDIQ	Y	1	276	16.70765	16.51455	16.58198	16.55424	17.12636	16.52541	16.70036	17.14818	16.47272	0.125661446	-0.172360102	-0.133946737	-0.038413366
339	F5H4R1	MAP3K4	mitogen-activated protein kinase kinase kinase 4	4216	ENSG00000085511	ASIAAELQFRSLSRHSSPTEERDEPAYPRGD	S	0.515251	1251	16.70698	16.68934	17.00668	16.81616	17.24961	17.0897	16.73026	17.05494	16.877	0.491491828	-0.086398443	-0.250822703	0.16442426
884	Q6IWH7	ANO7	anoctamin 7	50636	ENSG00000146205	TVLIDVSPPEAEKRGSYGSTAHASEPGGQQA	S	0.996979	81	16.69792	16.89416	17.01998	17.21371	17.13059	16.70104	16.66094	16.90419	17.22788	0.107834355	-0.060317993	-0.144428253	0.08411026
717	P61278	SST	somatostatin	6750	ENSG00000157005	SQAAEQDEMRLELQRSANSNPAMAPRERKAG	S	0.999641	89	16.69602	16.88417	16.8204	17.41204	16.8012	16.63934	17.30893	16.93641	16.37152	0.054607999	-0.072087606	-0.150664647	0.078577042
607	P04279	SEMG1	semenogelin 1	6406	ENSG00000124233	NVVEVREEHSSKVQTSLCPAHQDKLQHGSKD	S	0.903799	237	16.69384	16.16013	16.82997	16.74031	17.44403	16.50869	17.01105	17.13791	16.67783	0.35230093	-0.380951564	-0.336363475	-0.044588089
1052	Q9C0C2	TNKS1BP1	tankyrase 1 binding protein 1	85456	ENSG00000149115	LSPSALKAKLRPRNRSAEEGELAESKSSQKE	S	1	1666	16.69003	16.80247	16.59091	16.85014	16.77069	16.70239	16.97343	16.75356	16.9237	0.837676849	-0.189095179	-0.079938889	-0.109156291
547	O75864	PPP1R37	protein phosphatase 1 regulatory subunit 37	284352	ENSG00000104866	PGSPSTPTEQRISVSSPGRGHKVFVVTRVES	S	0.879168	88	16.68934	16.60332	16.41364	16.78775	16.92172	16.58871	16.57694	16.20713	16.30196	1.163768658	0.206759771	-0.197292964	0.404052734
169	M0R088	SRRM1	serine and arginine repetitive matrix 1	10250	ENSG00000133226	PSPPPRRRSPSPRRYSPPIQRRYSPSPPPKR	S	0.99992	570	16.68319	16.35626	16.06183	16.7512	16.36963	16.54302	16.21673	16.51022	16.50728	0.213878062	-0.04431661	-0.187522252	0.143205643
1170	Q9Y3R5	DOP1B	DOP1 leucine zipper like protein B	9980	ENSG00000142197	PKTQRTSIHCLKQENSADDLHRWFNRKKTSF	S	1	1021	16.68291	16.39145	16.63736	16.87099	17.13971	16.68072	16.73013	17.0351	16.89344	0.916831457	-0.315649668	-0.326566696	0.010917028
636	P13716	ALAD	aminolevulinate dehydratase	210	ENSG00000148218	FASCFYGPFRDAAKSSPAFGDRRCYQLPPGA	S	0.5	215	16.67783	16.64864	16.69778	17.07608	16.79182	16.79867	17.04996	16.71773	16.76409	0.648529135	-0.169176102	-0.214110692	0.044934591
9	A0A087WVU6	HECTD1	HECT domain E3 ubiquitin protein ligase 1	25831	ENSG00000092148	SAGSTGRIPGLRRLDSSGERSHRQLIDCIRS	S	0.988494	178	16.66775	16.94306	16.68195	16.91986	16.8881	16.57965	17.09466	16.7988	16.99711	0.456152073	-0.199268977	-0.031618754	-0.167650223
1346	P40818	USP8	ubiquitin specific peptidase 8	9101	ENSG00000138592	SDAKKSVEDRGKRCPTPEIQKKSTGDVPHTS	T	1	500	16.66775	16.31138	16.42541	16.46753	16.29013	16.47534	16.33778	16.50224	16.45282	0.062751855	0.037233353	0.057182948	-0.019949595
1519	H0YDD9	ANO1	anoctamin 1	55107	ENSG00000131620	IVYEILKRTTCTKAKYSMGQGEGRKKDSALL	Y	0.924399	107	16.64653	17.15956	17.34568	17.16074	17.06592	16.76474	17.51384	18.14021	16.94832	0.524741606	-0.483533859	0.053455353	-0.536989212
271	E7EQR4	EZR	ezrin	7430	ENSG00000092820	FGDYNKEVHKSGYLSSERLIPQRVMDQHKLT	S	0.996044	149	16.64047	16.75369	16.14183	16.80738	16.47581	16.71451	16.23403	16.18148	16.37572	0.801511236	0.248250961	-0.15390714	0.402158101
776	Q09666	AHNAK	AHNAK nucleoprotein	79026	ENSG00000124942	GHHTVGLKLHRKGDRSPEPGQTWTREVFSSC	S	0.999961	93	16.62756	16.59655	16.21339	16.85927	16.53934	16.86448	16.67369	16.49997	16.59797	0.614009402	-0.111375173	-0.275194168	0.163818995
1410	Q68DQ2	CRYBG3	crystallin beta-gamma domain containing 3	131544	ENSG00000080200	HENRLPTYFRGYESPTLSKDYEGYPAPAMPD	T	0.499955	1936	16.62656	16.97029	16.94123	16.91193	16.7563	16.47967	16.3784	16.06485	16.81691	0.636491486	0.4259758	0.130061467	0.295914332
878	Q68DQ2	CRYBG3	crystallin beta-gamma domain containing 3	131544	ENSG00000080200	IAHENRLPTYFRGYESPTLSKDYEGYPAPAM	S	0.499955	1934	16.62656	16.97029	16.94123	16.91193	16.7563	16.47967	16.3784	16.06485	16.81691	0.636491486	0.4259758	0.130061467	0.295914332
1530	R4GN18	CD46	CD46 molecule	4179	ENSG00000117335	LQRRKKKGKADGGAEYATYQTKSTTPAEQRG	Y	0.991227	63	16.62371	16.58364	16.5706	16.84671	16.90595	16.40163	16.93905	16.97589	16.62614	0.463174417	-0.254377365	-0.125448863	-0.128928502
1381	Q13443	ADAM9	ADAM metallopeptidase domain 9	8754	ENSG00000168615	PSRQPGSVPRHVSPVTPPREVPIYANRFAVP	T	0.590284	761	16.62257	16.68811	16.53617	16.82376	16.8964	16.70212	16.93503	16.70887	16.36857	0.344170577	-0.055205663	-0.191811879	0.136606216
1294	J3QRU1	YES1	YES proto-oncogene 1, Src family tyrosine kinase	7525	ENSG00000176105	IADFGLARLIEDNEYTARQGAKFPIKWTAPE	T	0.999996	432	16.62199	16.71867	16.82972	16.92636	16.96477	16.56321	16.65843	17.19287	16.65899	0.089891051	-0.113304774	-0.094654083	-0.018650691
296	E9PK71	PKP3	plakophilin 3	11187	ENSG00000184363	PIAKPAYSPASWSSRSAVDLSCSRRLSSAHN	S	0.996241	65	16.6131	16.58715	16.55703	16.62028	17.06297	16.84132	16.54134	16.71679	16.59193	0.908962924	-0.030929565	-0.255764643	0.224835078
1493	C9JFU4	TMC4	transmembrane channel like 4	147798	ENSG00000167608	RRAHRQRNASRDQVVYGSGTKTDRWARLLRR	Y	0.903499	19	16.6118	16.99203	16.65899	16.95594	17.20514	16.59006	16.74754	16.92393	16.98125	0.176216686	-0.129965464	-0.162774404	0.03280894
981	Q8WV41	SNX33	sorting nexin 33	257364	ENSG00000173548	QHMAFRPKPPLERQDSLASAKRGSVVGRNLN	S	0.997129	169	16.61022	17.10544	16.24101	16.68784	16.74623	16.66858	16.23773	16.59057	16.52077	0.277074035	0.202531179	-0.048658371	0.25118955
994	Q96C19	EFHD2	EF-hand domain family member D2	79180	ENSG00000142634	LLRRADLNQGIGEPQSPSRRVFNPYTEFKEF	S	0.953279	74	16.6089	16.91649	16.83738	16.88632	16.92219	16.80209	16.56105	16.74478	16.72414	0.704578807	0.11093394	-0.082607269	0.193541209
1297	K7EQ87	EVPL	envoplakin	2125	ENSG00000167880	PAALEGYRCYRSASPTVPRSLR_________	T	0.599358	106	16.60247	16.25818	16.64681	16.61813	16.78187	16.74977	16.65648	16.62013	16.6075	0.652332311	-0.125553767	-0.214102427	0.08854866
1546	Q92567	FAM168A	family with sequence similarity 168 member A	23201	ENSG00000054965	GTFHLPVDTGTENRTYQASSAAFRYTAGTPY	Y	0.625205	85	16.60078	17.08067	16.11057	16.86701	16.79753	16.68058	16.52451	16.10546	16.51607	0.436656696	0.215326945	-0.184369405	0.39969635
923	Q86X29	LSR	lipolysis stimulated lipoprotein receptor	51599	ENSG00000105699	GSYVPLLRDTDSSVASVRSGYRIQASQQDDS	S	1	366	16.59881	16.587	16.59782	16.64962	17.01291	16.82302	16.42174	16.77726	16.68031	0.708598539	-0.031888962	-0.233973185	0.202084223
313	F5GY11	STARD10	StAR related lipid transfer domain containing 10	10809	ENSG00000214530	AESREERMGGAGGEGSDDDTSLT________	S	0.996416	238	16.59265	16.90454	16.60383	16.86641	16.81703	16.60846	16.42266	16.40606	16.57338	1.056162577	0.23297437	-0.063627879	0.296602249
960	Q8NFH8	REPS2	RALBP1 associated Eps domain containing 2	9185	ENSG00000169891	PTPPPRPQKTHSRASSLDLNKVFQPSVPATK	S	0.995993	353	16.58874	16.66206	15.84967	16.45016	16.11969	16.45493	16.2227	15.97817	16.5446	0.046665478	0.118332545	0.025230408	0.093102137
1071	Q9H0B6	KLC2	kinesin light chain 2	64837	ENSG00000174996	QPGGTGLSDSRTLSSSSMDLSRRSSLVG___	S	0.740611	610	16.58549	16.75251	16.68181	16.96229	16.53092	16.15598	15.96916	15.63685	16.58939	0.742498029	0.60813872	0.123540878	0.484597842
949	Q8N4C8	MINK1	misshapen like kinase 1	50488	ENSG00000141503	RVPLKPYAAPVPRSQSLQDQPTRNLAAFPAS	S	0.815259	601	16.57977	16.54897	16.50034	16.47012	16.58379	16.61655	16.04316	16.54582	16.46918	0.513217416	0.190309525	-0.013788859	0.204098384
124	A0A286YEX4	NFIC	nuclear factor I C	4782	ENSG00000141905	YLAYFVRERDAEQSGSPRTGMGSDQEDSKPI	S	0.96803	216	16.57516	16.66872	16.50438	16.75551	16.36895	16.31526	16.65074	16.89024	16.49972	0.333326645	-0.097482045	0.102846146	-0.200328191
90	A0A0G2JIW1	HSPA1B	heat shock protein family A (Hsp70) member 1B	3304	ENSG00000232804	GPGGFGAQGPKGGSGSGPTIEEVD_______	S	0.834668	634	16.55927	16.19478	16.13168	16.2988	16.30983	16.36391	16.15512	15.5254	16.01863	0.920769079	0.395525614	-0.028938293	0.424463908
1367	Q02383	SEMG2	semenogelin 2	6407	ENSG00000124157	HMIVIHHKGGQAHHGTQNPSQDQGNSPSGKG	T	0.676541	139	16.55667	15.71596	16.18907	16.62614	16.40534	16.26119	16.3026	16.14806	16.02618	0.36837313	-0.005045255	-0.276988665	0.27194341
1541	Q13443	ADAM9	ADAM metallopeptidase domain 9	8754	ENSG00000168615	DQLWRSYFRKKRSQTYESDGKNQANPSRQPG	Y	0.672994	736	16.55376	17.04676	16.99932	17.06213	17.45459	16.77995	16.99092	17.33612	17.16693	0.377939291	-0.298042933	-0.232274373	-0.06576856
903	Q7L8J4	SH3BP5L	SH3 binding domain protein 5 like	80851	ENSG00000175137	PDTDTLSLLSLRTVASDLQKCDSVEHLRGLS	S	0.984048	311	16.5498	16.63013	16.17728	16.97489	16.67452	16.56095	16.44472	16.37946	16.67659	0.563607832	-0.047856649	-0.284382502	0.236525853
341	F5H6E2	MYO1C	myosin IC	4641	ENSG00000197879	LVGKINRSLASKDVESPSWRSTTVLGLLDIY	S	0.998588	384	16.54836	16.76668	16.04659	16.45797	16.37201	16.6971	16.32887	16.33235	16.35922	0.164828464	0.113730748	-0.055154165	0.168884913
1425	Q8IUC4	RHPN2	rhophilin Rho GTPase binding protein 2	85415	ENSG00000131941	VSLLDSTSSMHNKSATYSVGMQKTYSMICLA	T	0.67067	606	16.54638	16.67604	16.40221	16.6781	16.51705	16.57949	16.22451	16.3088	16.64822	0.455946697	0.147702535	-0.050004959	0.197707494

1122	Q9P206	KIAA1522	KIAA1522	57648	ENSG00000162522	PHLEELHTQAQEGLRSLQHQEKQKLNKGGWD	S	1	83	16.53958	16.97824	16.48833	16.56308	16.90853	17.0596	16.54217	16.62799	16.69248	0.330557653	0.047836939	-0.175018946	0.222855886
244	D6REA1	SIL1	SIL1 nucleotide exchange factor	64374	ENSG00000120725	LKSALAKFKEGAEMESSKEDKARQAEVKRLF	S	0.5	153	16.53845	15.18259	14.78729	16.43843	17.06918	16.05935	16.23799	16.4937	15.98533	0.699871678	-0.736232758	-1.019543966	0.283311208
165	Q5TIG5	AFDN	afadin, adherens junction formation factor	4301	ENSG00000130396	TDSNHSSIAIQRVTRSQEELREDKAYQLERH	S	1	1275	16.53729	16.64723	16.01537	16.79348	16.7628	16.61913	15.97663	16.11169	16.32436	1.291649255	0.262408574	-0.325167974	0.587576548
1271	H7C3X8	SIK3	SIK family kinase 3	23387	ENSG00000160584	PSPEALVRYLSMRRHTVGVADPRTEVMEDLQ	T	1	372	16.53261	16.35616	16.62927	16.69112	16.72787	16.70158	16.42192	16.08311	16.54861	1.045785174	0.154803594	-0.200839361	0.355642955
463	K7EMQ6	NARS	asparaginyl-tRNA synthetase	4677	ENSG00000134440	EQMKSESREKKEAEDSLRREKNLEEAKKITI	S	1	20	16.52749	16.61468	16.3098	16.788	16.31529	16.88166	16.39255	16.17687	16.41785	0.627172954	0.154897054	-0.177663167	0.332560221
617	P06727	APOA4	apolipoprotein A4	337	ENSG00000110244	QMKKNAEELKARISASAEELRQRLAPLAEDV	S	0.999667	259	16.51818	16.47701	16.18208	16.17426	16.66039	15.80947	16.72214	16.63368	16.09024	0.200996129	-0.089593252	0.17772007	-0.267313321
1326	P04279	SEMG1	semenogelin 1	6406	ENSG00000124233	SQTEEKAQGKSQKQITIPSQEQEHSQKANKI	T	0.75165	331	16.51495	15.99252	16.21381	16.73066	16.96263	16.16591	16.63098	16.74018	16.25206	0.436229647	-0.300646146	-0.379306475	0.078660329
1126	Q9P270	SLAIN2	SLAIN motif family member 2	57606	ENSG00000109171	LGPGSPVRAGASIPSSGAASPRGFPLGLSAK	S	0.631729	59	16.51425	16.47611	16.57075	16.68551	16.34599	16.23085	16.25961	16.04042	16.62842	0.267593639	0.210886002	0.099584579	0.111301422
1014	Q96S53	TESK2	testis associated actin remodelling kinase 2	10420	ENSG00000070759	KSPCPRRTIWLSRSQSDIFSRKPPRTVSVLD	S	0.740713	340	16.50693	16.63651	16.51559	16.64104	16.79551	16.65088	16.64428	16.53937	17.0024	0.405497311	-0.175672531	-0.142798106	-0.032874425
1540	Q13443	ADAM9	ADAM metallopeptidase domain 9	8754	ENSG00000168615	PRHVSPVTPPREVPIYANRFAVPTYAAKQPQ	Y	1	769	16.50092	16.96545	16.45841	16.782	17.12666	16.48684	16.564	16.79981	16.66345	0.129368444	-0.034162521	-0.156908671	0.12274615
581	O96013	PAK4	p21 (RAC1) activated kinase 4	10298	ENSG00000130669	CTPAAPAVPGPPGPRSPQREPQRVSHEQFRA	S	1	291	16.49832	16.73859	16.43486	16.72601	16.78021	16.56873	16.15865	16.51004	16.64329	0.513929671	0.119929632	-0.134389877	0.254319509
786	Q13131	PRKAA1	protein kinase AMP-activated catalytic subunit alpha 1	5562	ENSG00000132356	EAKSGTATPQRSGSVSNYRSCQRSDSDAEAQ	S	0.820359	498	16.49568	16.55076	16.33598	16.80876	16.28148	16.45097	16.55879	16.33612	16.64526	0.032454791	-0.052583694	-0.052927017	0.000343323
714	P60981	DSTN	destrin, actin depolymerizing factor	11034	ENSG00000125868	DEVCRIFYDMKVRKCSTPEEIKKRKKAVIFC	S	0.9819	24	16.4949	16.48737	16.74136	17.03252	16.7563	16.65004	16.54756	16.29415	16.644	0.82712452	0.079308192	-0.238407771	0.317715963
1409	Q5XXA6	ANO1	anoctamin 1	55107	ENSG00000131620	PSGNRTLVRRVQHSDTPSGARSVKQDHPLPG	T	0.977263	88	16.49243	17.33333	17.88566	17.00865	17.64118	17.14081	17.77179	18.47401	17.05091	0.279900022	-0.528429667	-0.026404063	-0.502025604
1016	Q96TA1	NIBAN2;FAM129B	niban apoptosis regulator 2;family with sequence similarity 129 member	64855	ENSG00000136830	QRVLERVLKKYDYDSSSVRKRFFREALLQIS	S	0.800205	472	16.49154	16.59687	16.70442	16.78366	16.53415	16.66331	16.31755	16.10226	16.61382	0.838344609	0.253065109	-0.062763214	0.315828323
665	Q5T181	SHC1	SHC adaptor protein 1	6464	ENSG00000160691	___________MNKLSGGGGRRTRVEGGQLG	S	1	5	16.47967	16.75616	16.48693	16.47913	16.61497	16.67162	16.39894	16.55171	16.35886	0.502262252	0.137750626	-0.014321009	0.152071635
451	J3KRZ2	DYNC1LI2	dynein cytoplasmic 1 light intermediate chain 2	1783	ENSG00000135720	_________XTPTRASESPARGPSGSPRTQG	S	0.589135	7	16.47722	16.05511	16.79829	16.59344	16.45839	16.59164	15.8546	16.02842	16.2993	0.910239677	0.382768631	-0.1042792	0.487047831
792	Q13557	CAMK2D	calcium/calmodulin dependent protein kinase II delta	817	ENSG00000145349	KSLLKKPDGVKESTESSNTTIEDEDVKARKQ	S	0.740463	333	16.47546	16.34748	16.01245	16.82625	16.40065	15.93664	16.27992	16.54303	16.04346	0.0441241	-0.010336558	-0.10937945	0.099042892
94	A0A0G2JMZ6	KIF5C	kinesin family member 5C	3800	ENSG00000168280	TAVHAIRGGGGSSSNSTHYQK__________	S	0.863903	855	16.4728	16.44411	16.38082	16.60131	16.4467	16.22251	15.94011	16.06479	16.31056	1.145682728	0.327424367	0.009070714	0.318353653
235	D6RCR4	DBN1	drebrin 1	1627	ENSG00000113758	GAIGQRLSNGLARLSSPVLHRLRLREDENA_	S	0.995511	79	16.46751	16.58344	16.19125	16.53088	16.61094	16.67162	16.47264	16.2342	16.48636	0.615135124	0.016330083	-0.190414429	0.206744512
1066	Q9H0B6	KLC2	kinesin light chain 2	64837	ENSG00000174996	RRSGSFGKLRDALRRSSEMLVKKLQGGTPQE	S	0.5	556	16.46735	16.44181	16.18406	16.51496	16.24167	15.79017	15.97388	15.77874	16.50953	0.234368368	0.277023633	0.18214035	0.094883283
479	O00204	SULT2B1	sulfotransferase family 2B member 1	6820	ENSG00000088002	PNTSLEREPRPNSSPSPSPGQASETPHPRPS	S	0.706587	335	16.45807	16.27747	16.36905	16.2237	16.45377	16.2243	16.26739	16.51565	16.25949	0.096213499	0.020688375	0.067607244	-0.046918869
815	Q15056	EIF4H	eukaryotic translation initiation factor 4H	7458	ENSG00000106682	__MADFDTYDDRAYSSFGGGRGSRGSAGGHG	S	0.994197	14	16.45631	16.60305	16.38259	16.33419	16.28955	16.57158	16.30458	16.26854	16.4622	0.344691834	0.135540009	0.082209905	0.053330104
1344	P35240	NF2	neurofibromin 2	4771	ENSG00000186575	SDRGGSSKHNTIKKLTLQSAKSRVAFFEEL_	T	0.999789	581	16.45422	16.73542	16.42818	16.61956	16.53322	16.33994	16.2941	16.55823	16.72547	0.018180296	0.013341904	0.041700999	-0.028359095
1256	Q92934	BAD	BCL2 associated agonist of cell death	572	ENSG00000002330	SFKKGLPRPKSAGTATQMRQSSSWTRVFQSW	T	0.534691	139	16.44868	16.77828	15.96215	16.4099	16.33349	16.3904	16.33067	16.233	16.42861	0.02466851	0.065611521	0.018439611	0.047171911
1478	Q9UHR4	BAIAP2L1	BAR/IMD domain containing adaptor protein 2 like 1;BAI1 associated prc	55971	ENSG00000006453	MIEEIKTPASTPVSGTPQASPMIERSNVVRK	T	0.993936	257	16.44278	16.46173	16.16176	16.36292	16.22594	16.50758	15.98489	16.14105	16.45996	0.297367201	0.160128593	-0.01005427	0.170182864
804	Q14515	SPARCL1	SPARC like 1	8404	ENSG00000152583	NASNVNKHIQETEWQSQEGKTGLEAISNHKE	S	0.998109	170	16.44196	16.40311	16.21832	16.47706	16.85111	16.21215	16.53754	16.55133	16.34249	0.192199074	-0.122658412	-0.158981959	0.036323547
1416	Q7L7X3	TAOK1	TAO kinase 1	57551	ENSG00000160551	LKESKELQIKKQFQDTCKIQTRQYKALRNHL	T	1	581	16.44121	16.73714	15.9365	16.15078	16.29124	16.60435	15.88369	16.0705	15.71253	0.82975914	0.482711156	0.022826831	0.459884326
1104	Q9NSK0	KLC4	kinesin light chain 4	89953	ENSG00000137171	TKVAELLGESDGRRTSQEGPGDSVKFEGGED	S	0.793532	442	16.43822	16.80788	16.44537	16.64766	16.57786	16.22223	16.22955	15.77605	16.23276	1.037671742	0.484372775	0.081244787	0.403127988
32	A0A0A0MT38	DOCK9	dedicator of cytokinesis 9	23348	ENSG00000088387	RSYVKYAYKAEPYVASEYKTVHEELTKSMTT	S	0.999969	927	16.435	16.55217	16.06283	16.81878	16.60089	16.50502	16.44061	16.15658	16.10132	0.947505932	0.117166519	-0.291561762	0.408728282
711	P57739	CLDN2	claudin 2	9075	ENSG00000165376	SNYYDAYQAQPLATRSSPRPGQPPKVKSEFN	S	0.913341	207	16.43324	16.9917	17.29697	17.15086	17.06234	16.91882	17.42743	17.86563	16.80158	0.378117876	-0.457574209	-0.136701584	-0.320872625
787	Q13144	EIF2B5	eukaryotic translation initiation factor 2B subunit epsilon	8893	ENSG00000145191	SEQSMDSEEPDSRGGSPQMDDIKVFQNEVLG	S	1	544	16.43047	16.81403	16.64864	16.75134	16.66025	16.55352	16.17168	16.24485	16.71424	0.557251336	0.254121145	-0.023988724	0.278109868
1479	Q9UHR4	BAIAP2L1	BAR/IMD domain containing adaptor protein 2 like 1;BAI1 associated prc	55971	ENSG00000006453	ELEKKIELDVKYMNATLKRYQTEHKNKLESL	T	0.999982	118	16.4249	16.75251	16.36429	16.41257	16.35802	16.77056	16.30624	16.54128	16.39771	0.112712073	0.098825455	0.000186284	0.09863917
1389	Q15678	PTPN14	protein tyrosine phosphatase non-receptor type 14;protein tyrosine phos	5784	ENSG00000152104	CTEQSNSPPPIRRQPTWSRSSLPRQQPYILP	T	0.999927	323	16.42182	16.78788	16.62884	16.88047	16.74517	16.48333	16.42915	16.9741	16.8483	0.117182665	-0.137671153	-0.090144475	-0.047526677
1013	Q96RF0	SNX18	sorting nexin 18	112574	ENSG00000178996	ARALYDFRSENPGEISLREHEVLSLCSEQDI	S	1	20	16.418	17.05875	16.56837	16.827	16.50103	16.70455	16.69275	16.8119	16.30219	0.036584883	0.079427083	0.004183451	0.075243632
675	P40189	IL6ST	interleukin 6 signal transducer	3572	ENSG00000134352	LPRQQYFKQNCSQHESSPDISHFERSKQVSS	S	0.571985	763	16.41561	15.62417	14.93668	15.30292	16.64371	16.08294	16.62185	17.50549	16.01147	0.572812866	-1.054114342	-0.351039251	-0.703075091
1021	Q9BST9	RTKN	rhotekin	6242	ENSG00000114993	ALTGGPKRLATKLSSSLGRSSGRRVRASLDS	S	0.665289	170	16.40344	16.27745	16.09922	16.1115	16.35525	16.58054	16.06842	16.19233	16.25457	0.311318586	0.088265737	-0.089056015	0.177321752
26	C9IYE1	CADPS2	calcium dependent secretion activator 2	93664	ENSG00000081803	DAPGRAGGGGAARSVSPSPSVLSEGRDEPQR	S	0.982139	58	16.39909	16.89119	16.64456	17.26013	17.11908	16.67466	16.03155	16.01496	17.00229	0.699584402	0.295349121	-0.373010635	0.668359756
459	J3QQN1	PSMB10	proteasome subunit beta 10	5699	ENSG00000205220	CVITKTGAKLLRTLSSPTEPVKRGCPSSLPG	S	0.830431	110	16.3963	16.38983	16.27168	16.79411	16.56938	16.56993	17.17485	16.59019	16.59009	0.951619536	-0.432437261	-0.291867574	-0.140569687
827	Q16825	AL162171.1;PTPN21	protein tyrosine phosphatase non-receptor type 21;protein tyrosine phos	11099	ENSG00000070778	HGLEGLRLKERTLSASAAEVAPRAVSVGSQP	S	0.984933	660	16.39134	16.4921	15.95778	16.94306	16.67494	16.43726	16.31685	17.00405	16.70388	0.617779651	-0.39451313	-0.404678027	0.010164897
83	A0A0C4DGH6	UHRF1BP1L	UHRF1 binding protein 1 like	23074	ENSG00000111647	KISEDESSGLVYKSGSGEIGSETSDKKDSFY	S	0.79785	639	16.38254	16.7345	16.13216	16.50848	16.70685	16.73331	16.28016	16.30547	16.6683	0.391966332	-0.001578649	-0.23314476	0.231566111
1218	F8VWV9	TUBA1B	tubulin alpha 1b	10376	ENSG00000123416	TGKEDAANNYARGHYTIGKEIID________	T	0.9734	74	16.37533	16.42017	16.40043	16.61425	16.18923	16.40931	16.32253	16.1004	16.30533	0.450937528	0.155892054	-0.005617778	0.161509832
1163	Q9UQN3	CHMP2B	charged multivesicular body protein 2B	25978	ENSG00000083937	QKTRTFAVSSKVTSMSTQTKVMNSQMKMAGA	S	0.532307	45	16.37278	16.49846	16.21568	16.38519	16.32177	16.44582	16.08632	16.03108	16.48614	0.391293473	0.16112709	-0.021952947	0.183080037
799	Q14166	TTLL12	tubulin tyrosine ligase like 12	23170	ENSG00000100304	THVTKSLHSIIRHRESTPKVVSKYIESPVLF	S	0.990955	444	16.35213	16.09148	16.21472	16.23156	16.262	16.58559	15.99086	15.84242	16.35602	0.551428556	0.156339963	-0.140277227	0.29661719
1306	O14494	PLPP1	phospholipid phosphatase 1	8611	ENSG00000067113	FKERKEEDSHTTLHETPTTGNHYPSNHQP__	T	0.921251	271	16.35113	16.2738	16.06352	16.56005	16.37601	16.46993	16.2125	16.3547	16.31425	1.072083445	-0.064330419	-0.239178975	0.174848557
272	E7ERA6	RNF223	ring finger protein 223	401934	ENSG00000237330	GQQVWHTAVPPPRRSSSIASMPRSPSSAGSP	S	0.954845	19	16.34724	16.52358	16.18714	16.88274	16.59636	16.78749	16.24767	16.68729	16.72494	0.912470048	-0.20064799	-0.402880351	0.202232361
690	P49207	RPL34	ribosomal protein L34	6164	ENSG00000109475	____MVQRLTYRRRLSYNTASNKTRLSRTPG	S	0.982873	12	16.34101	16.31472	16.08697	15.85854	16.51625	16.16657	16.38998	16.11415	16.17989	0.030970727	0.019560496	0.067111969	-0.047551473
713	P60981	DSTN	destrin, actin depolymerizing factor	11034	ENSG00000125868	_____________MASGVQVADEVCRIFYDM	S	1	3	16.33919	16.38156	15.96952	16.47983	16.29649	16.23672	16.35277	16.29115	16.36975	0.202080277	-0.107800484	-0.107589404	-0.00021108
932	Q8IXS8	FAM126B	family with sequence similarity 126 member B	285172	ENSG00000155744	TPTVPRISRTAITTASIRRHRWRREGAEGVN	S	0.975707	321	16.32936	16.59389	15.96761	16.65046	16.35504	16.57089	16.20057	16.18633	16.32994	0.559033414	0.058006287	-0.228514353	0.28652064
1227	A4UGR9	XIRP2	xin actin binding repeat containing 2	129446	ENSG00000163092	SSGQQNPKPYMRKFKTPLMIAEEKYRQQKEE	T	1	2320	16.32606	17.64273	16.07496	16.94843	17.2082	16.78072	16.0455	16.27365	16.44106	0.530197151	0.42784373	-0.297868093	0.725711823
524	O60269	GPRIN2	G protein regulated inducer of neurite outgrowth 2	9721	ENSG00000204175	CRLRAPSAAAMQRSHSDLVRSTQMRGHSGAR	S	0.999423	122	16.31964	16.51604	16.70334	16.73079	16.6541	16.71813	16.36234	16.37028	16.56732	0.960593458	0.079692841	-0.188004176	0.267697016
1521	J3QKM5	WIPF2	WAS/WASL interacting protein family member 2	147179	ENSG00000171475	RSAPILEKPKGSSGGYGSGGAALQPKGGLFQ	Y	0.949464	74	16.31618	16.63069	16.13097	16.74004	16.32365	16.51799	16.19791	16.23399	16.48638	0.344769893	0.053186417	-0.167949041	0.221135457
127	Q13555	CAMK2G	calcium/calmodulin dependent protein kinase II gamma	818	ENSG00000148660	ITADQALKHPWVCQRSTVASMMHRQETVECL;ITA	S	0.782754	276	16.30942	15.99709	15.59132	16.20482	15.99099	16.1507	15.91006	15.60418	16.04925	0.306738181	0.111448606	-0.149558703	0.261007309
766	Q05655	PRKCD	protein kinase C delta	5580	ENSG00000163932	YSNFDQEFLNEKARLSYSDKNLIDSMDQSAF	S	0.997685	645	16.3085	16.75382	16.35789	16.9131	16.64808	16.19633	16.34619	16.09655	16.70144	0.137152159	0.092010498	-0.112433116	0.204443614
1171	Q9Y3R5	DOP1B	DOP1 leucine zipper like protein B	9980	ENSG00000142197	DEITMKSEFPLLRQHSVSSIRQLMPFFMTLN	S	0.976698	2236	16.30754	16.33823	16.35827	16.94043	16.40921	16.76396	16.30611	16.28964	16.57205	1.032114717	-0.054586411	-0.369852066	0.315265656
816	Q15477	SKIV2L	Ski2 like RNA helicase	6499	ENSG00000204351	AVGQPGGPRGDTVSASPCSAPLARASSLEDL	S	0.966505	245	16.30546	16.21176	16.01232	16.51388	16.50967	16.18662	16.2799	16.52499	16.13601	0.444456565	-0.137121836	-0.226877848	0.089756012
10	F8W7S5	RRBP1	ribosome binding protein 1	6238	ENSG00000125844	KKLQEQLEKAEDGSSSKEGTSV_________	S	0.715742	745	16.3003	15.74861	15.50317	16.68058	17.22581	16.5032	16.84561	17.34412	15.93979	0.912408681	-0.859145482	-0.952502886	0.093357404
835	Q4KMP7	TBC1D10B	TBC1 domain family member 10B	26000	ENSG00000169221	PLVAPPRRHGAPAAPSPPPRGSRAGPVVVVA	S	0.995421	22	16.29115	15.80695	16.12557	16.07813	16.04874	16.16691	16.10198	15.98711	16.10644	0.016032966	0.009385109	-0.023367246	0.032752355
977	Q8TED4	SLC37A2	solute carrier family 37 member 2	219855	ENSG00000134955	EILAWKVSLSRGSGSSMVLTHQ_________	S	0.880372	499	16.28122	16.59908	16.49534	16.89782	16.78915	16.51467	16.60522	16.59773	16.17104	0.610665191	0.000552495	-0.275330861	0.275883357
819	Q15678	PTPN14	protein tyrosine phosphatase non-receptor type 14;protein tyrosine phos	5784	ENSG00000152104	RQMKRGILHTDSQSQSLRNLNIINTHAYNQP	S	0.76769	465	16.27416	16.4565	16.57413	16.96263	16.48712	16.74609	16.3764	16.56925	16.82972	0.532191792	-0.156857808	-0.297014236	0.140156428
1362	P80723	BASP1	brain abundant membrane attached signal protein 1	10409	ENSG00000176788	DEKAKEKDKKAEGAATEEEGTPKESEPQAAA	T	0.999934	31	16.27248	16.64231	16.40977	16.68414	16.29085	16.08891	15.95085	16.0588	16.36441	0.498294477	0.316835086	0.086885452	0.229949633
212	C9J6P4	ZC3HAV1	zinc finger CCCH-type containing, antiviral 1	56829	ENSG00000105939	FLASASASAERSCTPSPDQISHRASLEDAPV	S	0.999138	275	16.2669	16.53488	16.21407	16.46456	16.32903	16.06512	15.87606	16.08831	16.33027	0.43034767	0.24040254	0.05238088	0.18802166
1025	Q9BUF7	CRB3	crumbs cell polarity complex component 3;crumbs 3, cell polarity comple	92359	ENSG00000130545	KLREKRQTEGTYRPSSEEQVGARVPPTPNLK	S	0.816522	97	16.26024	16.40856	15.96052	16.38038	16.07046	16.42009	16.00957	16.37818	16.15326	0.098928382	0.029442787	-0.080533028	0.109975815
1135	Q9P2Q2	FRMD4A	FERM domain containing 4A	55691	ENSG00000151474	VRSPHYVHSTRSVDISPTRLHSLALHFRHRS	S	0.996636	694	16.25803	16.44548	16.02349	16.44624	16.09589	16.26158	15.77409	16.075	16.2629	0.392745651	0.205005646	-0.025568644	0.23057429
863	Q5T0Z8	C6orf132	chromosome 6 open reading frame 132	647024	ENSG00000188112	QRAQKGRSVGAALGRSSLPGSLRDHSHQAEA	S	0.837516	862	16.25553	16.83923	15.55938	16.1048	16.55571	16.48238	15.5931	16.02081	15.4603	0.693103947	0.526643117	-0.162921588	0.689564705
1401	Q5JZ02	TP53RK	TP53 regulating kinase	112858	ENSG00000172315	GYRHPALEARLGRRRTVQEARALLRCRRAGK	T	1	81	16.25541	16.96387	15.82123	16.70374	16.96104	16.64583	16.1982	16.32666	16.12475	0.673666381	0.130301793	-0.423367182	0.553668976
801	Q14195	DPYSL3	dihydropyrimidinase like 3	1809	ENSG00000113657	TTTPKGGTPAGSARGSPTRPNPPVRNLHQSG	S	0.769519	636	16.25468	16.27394	15.8982	15.96747	16.08229	16.15666	15.99786	15.61882	16.03379	0.528243089	0.258784294	0.073465029	0.185319265
216	C9JI98	TMEM238	transmembrane protein 238	388564	ENSG00000233493	RWSAPAAAGQRPAPGSRRARRAARAPPPPAA	S	0.999985	137	16.25364	16.54876	16.10922	16.76293	16.41971	16.96104	16.01175	16.47905	16.6197	0.64758858	-0.066294352	-0.410685857	0.344391505
1454	Q9BW04	C1orf116;SARG	chromosome 1 open reading frame 116	79098	ENSG00000182795	ALPITQPTPRGGPEETITQQGRTPRTVTESS	T	0.995246	100	16.23371	16.55043	15.79756	16.31565	16.2589	16.67301	15.62039	16.22427	16.15883	0.462556291	0.192739487	-0.221948624	0.41468811
1505	D6R9B4	CD164	CD164 molecule	8763	ENSG00000135535	VIFFLYKFCKSKERNYHTL____________	Y	0.997073	160	16.22927	16.06205	16.28881	16.53644	15.86419	16.24254	16.52077	16.05378	16.22846	0.030943831	-0.074295044	-0.021014849	-0.053280195
1457	Q9BZH6	WDR11	WD repeat domain 11	55717	ENSG00000120008	SSPHSSPAHNKLATATGAKKALNKVKILITQ	T	0.790656	219	16.22733	16.41212	15.87051	16.45329	16.17707	16.39712	16.13471	16.08514	16.20614	0.391818178	0.027993838	-0.172504743	0.200498581
1432	Q8N2M8	CLASRP	CLK4 associating serine/arginine rich protein	11129	ENSG00000104859	LAEKKASIGYTYEDSTVAKVEKAAEKPEEEE	T	0.771173	171	16.22491	15.99914	15.9424	15.91308	16.49528	16.33544	15.89945	16.10826	15.89888	0.511213919	0.08661747	-0.192449888	0.279067357
395	H0YE29	ARHGAP1	Rho GTPase activating protein 1	392	ENSG00000175220	RQVLKYDDFLKSTQKSPATAPKPMPPRPPLP	S	0.806673	177	16.22272	16.08385	15.64194	16.22786	16.37557	16.08217	15.6494	15.72563	16.0563	0.78470295	0.172394117	-0.245695114	0.418089231
1375	Q12929	EPS8	epidermal growth factor receptor pathway substrate 8	2059	ENSG00000151491	PPESGLGRADPPYTHTIQKQRMEYGPRPADT	T	0.984763	605	16.2112	16.33577	15.99703	16.277	16.07548	16.37312	16.1146	16.17684	15.72672	0.440304608	0.17528216	-0.060530663	0.235812823
1456	Q9BW04	C1orf116;SARG	chromosome 1 open reading frame 116	79098	ENSG00000182795	SSYLSTEKDASPKTSTSLGKGSFLDKISPSV	T	0.995854	507	16.21006	16.46094	15.69732	16.09505	16.1186	16.69152	16.16048	16.22857	16.24178	0.112042132	-0.087505976	-0.178949992	0.091444016
1370	Q02952	AKAP12	A-kinase anchoring protein 12	9590	ENSG00000131016	VHMMAAAVADGTRAATIIEERSPSWISASVT	T	1	799	16.20749	15.86957	16.07721	16.00328	16.12147	16.18876	15.67361	15.85289	16.29426	0.182848011	0.11116759	-0.053080241	0.164247831
769	Q07866	KLC1	kinesin light chain 1	3831	ENSG00000126214	VEWNGDGTGSLKRSGSFSKLRASIRRSSEKL	S	0.990091	561	16.20671	16.42019	16.65564	16.69262	16.47374	16.49171	16.12793	16.06476	16.79981	0.194024671	0.09667778	-0.125174205	0.221851985
61	E9PMS6	LMO7	LIM domain 7	4008	ENSG00000136153	TTGVATTQSPTPRSHSPSASQSGSQLRNRSV	S	0.9793	1185	16.20562	15.81833	16.40468	16.6569	16.20985	16.50097	16.34126	16.0328	16.18623	0.549725742	-0.043885549	-0.313029607	0.269144058
81	A0A0C4DGE6	ARHGAP27	Rho GTPase activating protein 27	201176	ENSG00000159314	ELPPEESESSRVDFGSSERLGSWQEKEEDAR	S	0.961651	264	16.20503	16.6541	16.18094	16.29266	16.50933	16.11382	16.44283	16.55983	15.90188	0.010480377	0.045178731	0.041419983	0.003758748
1324	P04279	SEMG1	semenogelin 1	6406	ENSG00000124233	GNSPSGKGISSQYSNTEERLWVHGLSKEQTS	T	0.999999	162	16.20428	15.62879	15.55345	16.37524	16.57622	16.04597	17.08046	16.18714	16.58671	1.092334238	-0.82259655	-0.536970456	-0.285626094
370	F8WBF9	NDRG3	NDRG family member 3	57446	ENSG00000101079	IPSASMTRLARSRTHSTSSSLGSGESPFSRS	S	0.661021	236	16.20147	16.41915	16.05871	16.39373	16.24809	16.29862	16.44668	15.97799	16.18394	0.134540474	0.023572286	-0.087034225	0.110606511
168	Q5TIG5	AFDN	afadin, adherens junction formation factor	4301	ENSG00000130396	RADHRSSPNVANQPPSPGGKSAYASGTTAKI	S	0.999999	1182	16.19672	16.26488	16.05972	16.27939	15.94711	16.20614	15.6438	15.96052	16.05649	0.800240245	0.286837896	0.029561679	0.257276217
601	P04279	SEMG1	semenogelin 1	6406	ENSG00000124233	NALHKTTKSQRHLGGSQQLLHNKQEGRDHDK	S	1	103	16.19164	15.28063	15.51465	15.68467	16.7672	15.81701	15.6652	15.93542	15.96732	0.268530588	-0.193672498	-0.427321116	0.233648618
180	H3BV44	MYO9A	myosin IXA	4649	ENSG00000066933	RFSSVDEQAKLHKTMSQGEITKLAVRQKASD	S	0.665231	583	16.18793	16.56806	16.4135	16.81728	16.36907	16.15153	15.97583	16.37921	16.17338	0.357705941	0.213689486	-0.056131999	0.269821485
793	Q13576	IQGAP2	IQ motif containing GTPase activating protein 2	10788	ENSG00000145703	MPHEELPSLQRPRYGSIVDDERLSAEEMDER	S	0.999972	16	16.18793	16.23353	15.67138	16.33851	16.14043	16.06558	16.15288	15.94041	16.2117	0.151819022	-0.070717176	-0.150564194	0.079847018
1034	Q9BV36	MLPH	melanophilin	79083	ENSG00000115648	AHVMASHHSKRRGRASSESQGLGAGVRTEAD	S	0.917423	336	16.18325	16.22034	15.74439	16.12606	15.66609	15.77004	15.26913	15.87922	16.19378	0.199611167	0.268616676	0.195262591	0.073354085

1202	A0A088AWN2	WDR20	WD repeat domain 20	91833	ENSG00000140153	DRANSTQSRLSKRNSTDSRPVSVTYRFGSVG	T	0.545166	389	16.18185	16.31262	16.43567	16.94855	16.4242	16.58043	16.57174	16.53254	16.31031	0.769330034	-0.161483129	-0.341014862	0.179531733
171	B0QY95	MIEF1	mitochondrial elongation factor 1	54471	ENSG00000100335	LAVKRMYDRAISAPTSPTRLSHSGKRSWEEP	S	0.696352	59	16.17563	16.37067	15.91495	16.39143	16.16965	16.07778	16.22568	15.97073	16.35454	0.029090666	-0.02990373	-0.059204102	0.029300372
225	C9JZY6	UBE2H	ubiquitin conjugating enzyme E2 H	7328	ENSG00000186591	___________MSSPSPGKRRMDTDVVKLIE	S	0.668795	5	16.17141	16.56648	16.52471	16.5977	16.10292	16.69629	16.12283	16.06863	16.19913	0.654392586	0.290670395	-0.044768651	0.335439046
584	P01833	PIGR	polymeric immunoglobulin receptor	5284	ENSG00000162896	VGVARARHRKNVDRVSIRSYRTDISMSDFEN	S	1	673	16.16595	16.1287	16.04674	16.22266	16.05042	15.877	18.69347	16.27358	17.0024	0.890201898	-1.209351858	0.063768069	-1.273119926
42	B6VEX4	ABI1	abl interactor 1	10006	ENSG00000136754	PTVPNDYMTSPARLGSQHSPGRTASLNQRPR	S	0.72265	222	16.16304	15.94576	15.94679	16.20843	15.88955	16.18478	15.92875	15.85238	16.20205	0.124780186	0.024138133	-0.075719833	0.099857966
423	H7C155	RAF1	Raf-1 proto-oncogene, serine/threonine kinase	5894	ENSG00000132155	LQHSLPKINRSASEPSLHRAAHTEDINACTL	S	0.611756	503	16.15581	16.34067	16.01848	16.26351	16.2278	16.51593	16.02434	16.02306	16.25728	0.63512823	0.070092519	-0.164095561	0.23418808
664	Q5T181	SHC1	SHC adaptor protein 1	6464	ENSG00000160691	VEGGQLGGEEWTRHGSFVNKPTRGWLHPNDK	S	0.999999	29	16.15506	16.96871	16.22611	16.4949	16.02763	16.08688	15.81092	15.83664	16.02206	0.795115981	0.560083071	0.246822357	0.313260714
136	A0A2R8Y6L5	DYRK1A	dual specificity tyrosine phosphorylation regulated kinase 1A	1859	ENSG00000157540	SSGGSSGTSNSGRARSDPTHQHRHSGGHFTA	S	0.988331	529	16.15464	15.94247	16.10969	16.36241	16.40348	16.18642	16.38776	15.97571	15.95335	0.636930588	-0.036678632	-0.248504003	0.211825371
366	H0Y5T1	CLASP1	cytoplasmic linker associated protein 1	23332	ENSG00000074054	SSSQESLNRPLSAKRSPTGSTTSRASTVSTK	S	0.984111	353	16.15185	16.40762	16.07748	16.16914	15.91076	16.33551	15.96918	15.59336	16.26477	0.350537731	0.269877116	0.073845228	0.196031888
1305	O00161	SNAP23	synaptosome associated protein 23	8773	ENSG00000092531	DAQNPQIKRITDKADTNRDRIDIANARAKKL	T	0.999997	193	16.14356	16.42123	15.69351	15.70258	15.73859	16.67632	15.41634	16.32666	15.76114	0.10310201	0.251390775	0.04693826	0.204452515
1452	Q9BSW2	CRACR2A	calcium release activated channel regulator 2A	84766	ENSG00000130038	VQGQIPEAPPLKLTPTSPRGQPVGKEALCKE	T	0.937142	520	16.14135	15.87584	16.08032	16.35399	16.09102	15.7601	15.6608	15.89673	16.33687	0.04705287	0.067700386	-0.035866102	0.103566488
60	C9JUH3	CCM2	CCM2 scaffold protein	83605	ENSG00000136280	LETIGVKDGRGIITDSFGRHRRALSTTSSST	S	0.999981	287	16.13991	16.32501	15.72651	15.66169	15.4649	16.64414	15.36615	15.79223	15.81215	0.270154029	0.406964938	0.14023304	0.266731898
1101	Q9NS68	TNFRSF19	TNF receptor superfamily member 19	55504	ENSG00000127863	LHEYAHRACCQCRRDSVQTCGPVRLLPSMCC	S	0.999998	109	16.13859	15.98655	15.61327	16.49603	16.40439	16.20373	16.23205	16.20264	16.16755	1.26928808	-0.287941615	-0.455244064	0.16730245
1386	Q14247	CTTN	cortactin	2017	ENSG00000085733	EARRKLEEQARAKTQTPPVSPAPQPTEERLP	T	0.967274	364	16.13595	16.386	16.07305	16.57154	16.55625	16.23904	16.39307	16.22936	16.03607	0.650115819	-0.021166484	-0.257277807	0.236111323
1178	Q9Y3R5	DOP1B	DOP1 leucine zipper like protein B	9980	ENSG00000142197	MDPEEQELLNDYRYRSYSSVIEKALRNFESS	S	0.998862	16	16.13505	15.99223	15.54795	16.34833	15.64659	16.1618	15.34482	15.31518	15.85898	0.72128264	0.385416667	-0.16049703	0.545913696
838	Q53EL6	PDCD4	programmed cell death 4	27250	ENSG00000150593	QLRDLCPSRGRKRFVSEGDGGRLKPESY___	S	1	457	16.13419	16.32311	16.11634	16.06059	16.39518	16.44493	16.1795	15.90893	16.29255	0.280799009	0.064216614	-0.109024048	0.173240662
1388	Q15056	EIF4H	eukaryotic translation initiation factor 4H	7458	ENSG00000106682	DFLGGRGGSRPGDRRTGPPMGSRFRDGPPLR	T	0.999524	156	16.13286	16.33539	16.18437	16.35733	15.94501	16.25207	15.69169	16.08389	16.18462	0.414805196	0.230805715	0.032737732	0.198067983
316	F5GYU8	TMC5	transmembrane channel like 5	79838	ENSG00000103534	EPDYSEFQSHPYHRASSRQPDYPGSQRNPDF	S	0.956791	122	16.12942	16.25316	15.99021	15.98054	16.1083	16.26461	15.95363	16.50061	15.95685	0.002731104	-0.012762705	0.006450971	-0.019213676
474	M0R0Y8	CALR3	calreticulin 3	125972	ENSG00000269058	SIEYDWNLTSLKKETSPAE____________	S	0.5	139	16.12795	16.0989	16.0015	17.07462	16.36064	16.64273	17.37551	17.05155	16.22637	0.969147123	-0.808363597	-0.616549174	-0.191814423
1304	M0R0Y8	CALR3	calreticulin 3	125972	ENSG00000269058	GSIEYDWNLTSLKKETSPAE___________	T	0.5	138	16.12795	16.0989	16.0015	17.07462	16.36064	16.64273	17.37551	17.05155	16.22637	0.969147123	-0.808363597	-0.616549174	-0.191814423
523	O43896	KIF1C	kinesin family member 1C	10749	ENSG00000129250	RRPPSPRRSHHPRRNSLDGGGRSRGAGSAQP	S	1	1033	16.12761	15.78956	15.74504	16.06678	16.15256	15.99689	15.88018	15.89874	16.17206	0.37577832	-0.096255302	-0.184673945	0.088418643
484	O00418	EEF2K	eukaryotic elongation factor 2 kinase	29904;101930	ENSG00000103319	KRGELDDPEPREHGHSYSNRKYESDEDSLGS	S	0.976386	462	16.12626	15.59392	16.0936	15.84578	15.73664	15.67361	15.78614	15.61459	15.82548	0.375847197	0.195854187	0.185914358	0.00993983
1030	Q9BV36	MLPH	melanophilin	79083	ENSG00000115648	SVPEARDSPQSLTDESCSEKAAPHKAEGLEE	S	0.993322	231	16.11937	15.69144	15.78374	16.00933	15.54092	16.06817	15.76539	15.72225	15.71663	0.163373296	0.13009421	-0.00795269	0.1380469
970	Q8TE68	EPS8L1	EPS8 like 1	54869	ENSG00000131037	PYNILTPYPGPRLHHSQSPARSLNSTPPPPP	S	0.577834	543	16.11065	16.11018	15.83222	16.12076	15.92439	16.21794	15.98181	16.02451	15.79398	0.324889009	0.084246635	-0.070016543	0.154263179
213	C9JDY7	LAT2	linker for activation of T cells family member 2	7462	ENSG00000086730	SEKIYQQRSLREDQQSFTGSRTY________	S	0.975188	51	16.10969	16.06689	15.90365	16.49726	15.94018	15.98795	16.1046	16.16975	15.74131	0.125148097	0.021522204	-0.115054766	0.13657697
770	Q08462	ADCY2	adenylate cyclase 2	108	ENSG00000078295	DLKTYFVNTEMSRSLSQSNVAS_________	S	0.779222	905	16.10799	16.37133	16.28544	16.37434	16.55025	16.26812	15.96837	16.24087	16.35158	0.483105993	0.067982992	-0.142649968	0.21063296
1531	R4GN18	CD46	CD46 molecule	4179	ENSG00000117335	RKKKGKADGGAEYATYQTKSTTPAEQRG___	Y	0.99901	66	16.10286	16.19281	15.67259	16.46802	16.29643	16.21676	16.34192	16.43114	16.06783	0.747203474	-0.290876071	-0.337651253	0.046775182
1365	P84095	RHOG	ras homolog family member G	391	ENSG00000177105	ILLVGTKKDLRAQPDTLRRLKEQGQAPITPQ	T	1	125	16.10185	16.1063	16.11476	16.07828	15.98908	16.05462	15.59747	15.98067	16.10681	0.518580963	0.212651889	0.06697464	0.145677249
1223	A0A2R8Y549	PATJ	PATJ crumbs cell polarity complex component	10207	ENSG00000132849	GLVQLEIGRLRAGSWTSARTTSQNSQGSQQS	T	0.906274	1620	16.10042	16.15339	16.09261	16.02566	16.00858	16.42134	15.47136	15.75063	16.00961	1.023682791	0.371609052	-0.03638649	0.407995542
466	K7ELD9	SYNGR2	synaptogyrin 2	9144	ENSG00000108639	_____________MESGAYGAAKAGGSFDLR	S	0.99381	3	16.09789	16.24206	15.8774	16.41005	16.07554	16.15302	15.90183	15.90431	16.02187	0.760657037	0.129780769	-0.140420596	0.270201365
637	P13807	GYS1	glycogen synthase 1	2997	ENSG00000104812	PRPASVPPSPSLSRHSSPHQSEDEEDPRNGP	S	0.533065	588	16.09245	16.47363	15.92054	16.72081	16.4264	16.19025	16.09239	16.17991	16.26646	0.50965901	-0.017379443	-0.283612251	0.266232808
1335	P13639	EEF2	eukaryotic translation elongation factor 2	1938	ENSG00000167658	KAGIIASARAGETRFTDTRKDEQERCITIKS	T	0.998482	57	16.09156	16.15725	16.02877	16.77828	16.19533	15.92634	16.55075	16.68154	16.25639	0.534232325	-0.403701782	-0.207461039	-0.196240743
937	Q8IY63	AMOTL1	angiomotin like 1	154810	ENSG00000166025	VPSIAAATGTHSRQTSLTSSQLAEEKKEEKT	S	0.693715	759	16.08931	15.9978	15.91242	16.40023	16.34707	15.91259	15.68532	16.00103	16.05643	0.632204693	0.085580508	-0.220118841	0.305699348
1288	I3L4C2	BAIAP2	BAI1 associated protein 2;BAR/IMD domain containing adaptor protein 2	10458	ENSG00000175866	SPPQSQSKLSDSYSNTLPVRKSVTPKNSYAT	T	0.998242	373	16.08723	15.99296	15.51283	15.67951	15.29537	16.19904	15.46471	15.93643	14.99903	0.274616025	0.397620201	0.139702479	0.257917722
549	O76054	SEC14L2	SEC14 like lipid binding 2	23541	ENSG00000100003	PNPDDYFLLRWLRARSFDLQKSEAMLRKLGR	S	1	46	16.07736	16.57046	16.03772	16.43248	16.14858	16.12777	15.79543	15.68836	16.41442	0.303369928	0.262442589	-0.007762273	0.270204862
988	X6RAB3	USP6NL	USP6 N-terminal like	9712	ENSG00000148429	GVHHLSNGQRSVGRPSPLASGRRESGAPHRR	S	0.997999	419	16.07646	16.18204	15.13691	15.88981	15.82642	15.97808	15.92388	15.7499	15.92611	0.028890669	-0.06815815	-0.099635124	0.031476974
538	O75113	N4BP1;AC026470.1	NEDD4 binding protein 1	9683	ENSG00000102921	EEALSNERICQKRRFSDSEERHTKKQFSLEN	S	0.995371	288	16.07387	15.96287	16.12695	16.03015	16.0455	15.73923	16.1159	16.17861	16.32504	0.981164772	-0.151952108	0.11626943	-0.268221537
197	B7Z613	GPM6B	glycoprotein M6B	2824	ENSG00000046653	QDIKAKEEQELQDIQSRSKEQLNSYT_____	S	1	292	16.0639	15.85788	16.45211	15.92741	16.24592	15.88981	15.74199	16.47518	16.02995	0.039296384	0.04225572	0.103583972	-0.061328252
108	A0A1B0GU52	IL10RB	interleukin 10 receptor subunit beta	3588	ENSG00000243646	WCVYKKTKYAFSPRNSLPQHLKEE_______	S	1	36	16.06108	16.27051	15.80647	16.23803	15.81478	15.9265	15.90471	16.08526	15.79617	0.104940812	0.117308935	0.052914619	0.064394315
1182	Q9Y570	PPME1	protein phosphatase methylesterase 1	51400	ENSG00000214517	RVSMVGQVKQCEGITSPEGSKSIVEGIIEEE	S	0.956776	56	16.06105	15.94948	15.36458	16.29164	15.66289	15.87875	16.074	15.90023	15.7771	0.094584484	-0.125403086	-0.152718862	0.027315776
1438	Q8TB03	CXorf38	chromosome X open reading frame 38	159013	ENSG00000185753	LCLHQKLDSQEPGRQTPDRKA__________	T	0.999509	195	16.05985	15.97985	15.67298	16.26538	16.26129	15.98275	15.52102	15.83022	15.82972	1.190477462	0.177239736	-0.265578588	0.442818324
706	P52735	VAV2	vav guanine nucleotide exchange factor 2	7410	ENSG00000160293	PYKSRERSASRASSRSPVFTPRVIGTAVARY	S	0.94211	771	16.05945	15.96111	16.13248	16.2412	15.44628	16.08479	16.36871	15.96061	15.73355	0.058315166	0.030056318	0.126924833	-0.096868515
938	Q8IY63	AMOTL1	angiomotin like 1	154810	ENSG00000166025	PARSVPSIAAATGTHSRQTSLTSSQLAEEKK	S	0.978941	755	16.05691	15.78364	15.64276	16.59682	15.73994	15.75874	16.22161	15.8924	15.62617	0.105684859	-0.085621198	-0.204063098	0.1184419
712	P57739	CLDN2	claudin 2	9075	ENSG00000165376	NYYDAYQAQPLATRSSPRPGQPPKVKSEFNS	S	0.815024	208	16.05046	16.88346	17.22938	16.81841	16.92254	16.64766	17.23837	17.6918	16.31875	0.153359572	-0.361872355	-0.075099945	-0.28677241
1325	P04279	SEMG1	semenogelin 1	6406	ENSG00000124233	STQDELLVYNKNQHQTKNLNQDQQHGRKANK	T	1	270	16.04859	15.88648	16.74373	16.44481	17.06519	15.66156	16.65382	17.00744	16.46058	0.269938988	-0.481015523	-0.164246559	-0.316768964
631	P12036	NEFH	neurofilament heavy	4744	ENSG00000100285	LEALKSTKDSLERQRSELEDRHQADIASYQE	S	1	347	16.0468	16.43381	15.70377	16.21274	16.24965	16.38105	16.40503	16.41673	16.05408	0.317827136	-0.230481784	-0.219686826	-0.010794957
219	C9JKZ3	TMPRSS2	transmembrane serine protease 2	7113	ENSG00000184012	VLTQASNPVVCTQPKSPSGTVCTSKTKKALC	S	0.605245	71	16.04448	15.89699	16.62671	16.41178	14.7073	15.60026	16.6197	15.98315	16.1991	0.477361536	-0.077922821	0.616279602	-0.694202423
886	Q6P1L5	FAM117B	family with sequence similarity 117 member B	150864	ENSG00000138439	PAGGGGGGGPRTASRSTSPTRGGGNAAARTS	S	0.548541	91	16.03547	15.72593	16.0804	15.90903	15.69016	16.14655	15.83538	15.8964	16.11762	0.01295131	-0.002539635	0.032018026	-0.03455766
1124	Q9P206	KIAA1522	KIAA1522	57648	ENSG00000162522	GGSTAEASDTLSIRSSGQLSGRSVSLRKLKR	S	0.785567	433	16.01826	15.48787	15.86481	16.49182	15.98155	16.09902	15.74759	16.17055	15.83723	0.645814843	-0.128141403	-0.400478045	0.272336642
536	O60825	PFKFB2	6-phosphofructo-2-kinase/fructose-2,6-biphosphatase 2	5208	ENSG00000123836	NNFPKNQTPVRMRRNSFTPLSSSNTIRRPRN	S	0.999707	466	16.01378	15.8982	15.45031	16.13745	16.16754	15.79294	15.90703	16.03185	15.97307	0.399804768	-0.183221181	-0.245213509	0.061992327
1508	D6RBN9	RELL1	RELT like 1	768211	ENSG00000181826	______MNPGACWYIYILQKGLTQQGGEAVG	Y	0.5	10	16.01289	15.75817	15.81716	15.75898	16.34398	16.03123	16.29221	16.10754	15.77492	0.245836903	-0.195485751	-0.181991259	-0.013494492
1507	D6RBN9	RELL1	RELT like 1	768211	ENSG00000181826	________MNPGACWYIYILQKGLTQQGGEA	Y	0.5	8	16.01289	15.75817	15.81716	15.75898	16.34398	16.03123	16.29221	16.10754	15.77492	0.245836903	-0.195485751	-0.181991259	-0.013494492
567	O95685	PPP1R3D	protein phosphatase 1 regulatory subunit 3D	5509	ENSG00000132825	GCDPRLRPIILRRARSLPSSPERRQKAAGAP	S	0.993631	74	16.01057	16.15371	16.20778	16.21494	16.24358	16.04048	16.2371	16.48174	16.26024	0.794000903	-0.202339172	-0.042317073	-0.1600221
243	Q9UPQ0	LIMCH1	LIM and calponin homology domains 1	22998	ENSG00000064042	PKTLPLDKSINHQIESPSERRKKSPREHFQA	S	0.825221	1357	16.00874	15.75149	15.74384	16.0724	15.99616	16.07307	15.72542	15.86111	16.30925	0.333784875	-0.130571047	-0.212526957	0.08195591
290	Q7KZI7	MARK2	microtubule affinity regulating kinase 2	2011	ENSG00000072518	STNSVLSTSTNRSRNSPLLERASLGQASIQN	S	0.930215	486	16.00854	16.23695	16.01822	16.35263	16.45797	16.33015	16.02321	16.34089	16.51584	0.771357556	-0.205411911	-0.29234759	0.086935679
907	Q7Z6Z7	HUWE1	HECT, UBA and WWE domain containing E3 ubiquitin protein ligase 1;H	10075	ENSG00000086758	AMSLGQDIPMDQRAESPEEVACRKEEEERKA	S	1	1395	16.00477	15.68568	16.09879	15.94455	16.212	15.39292	15.92722	16.03019	16.42064	0.247934102	-0.196269989	0.07992204	-0.276192029
947	Q8N4C7	STX19	syntaxin 19	415117	ENSG00000178750	SLVASMRRFSLLKRESTITKEIKIQAEYINR	S	0.978585	92	15.98686	15.90247	16.14663	15.9407	15.41366	16.11272	15.48341	15.98388	15.32524	0.515222506	0.41447862	0.189624151	0.224854469
1004	Q96JZ2	HSH2D	hematopoietic SH2 domain containing	84941	ENSG00000196684	HLATVNLSSLLDVRRSTVISGPGTGKGSQDH	S	0.980324	182	15.9868	15.97918	15.78691	16.42039	16.41391	15.91188	15.76591	16.16563	16.03761	0.653648003	-0.072089195	-0.331097921	0.259008725
96	D6RJF3	PANK4	pantothenate kinase 4	55229	ENSG00000157881	LAYYSTVQHKVAKVRSFDHSGKVSLHCGHPG	S	0.999903	110	15.97605	16.14474	15.96876	16.27938	16.54379	16.40762	16.71277	15.94615	16.44891	0.714455658	-0.339427948	-0.380411466	0.040983518
157	C9JXU1	SHROOM1	shroom family member 1	134549	ENSG00000164403	LRPTVPARPPATHPRSASLSHPGGEGEPARS	S	0.510831	186	15.96835	16.49596	15.87652	16.13439	15.92156	16.0169	16.01378	15.98997	16.20732	0.055595826	0.043253899	0.08932972	-0.046075821
521	O43581	SYT7	synaptotagmin 7	9066	ENSG00000011347	EDALRSGGAAPSEPGSGGKAGRGRWRTVQSH	S	0.987086	122	15.96418	16.461	16.01931	16.76461	16.53452	15.89584	15.84877	16.21227	16.03622	0.380044312	0.115745544	-0.250157356	0.365902901
814	Q15056	EIF4H	eukaryotic translation initiation factor 4H	7458	ENSG00000106682	___MADFDTYDDRAYSSFGGGRGSRGSAGGH	S	0.949584	13	15.95431	16.23021	16.07454	16.18125	16.14689	16.06243	15.87503	15.75478	16.19048	0.442429693	0.146254222	-0.043835322	0.190089544
284	E7EX40	RAB11FIP1	RAB11 family interacting protein 1	80223	ENSG00000156675	PEAEPESKSEPRPPISSPRAPQTRAVKPRLH	S	0.770131	380	15.94681	15.83155	16.05388	16.74255	16.3786	16.57576	16.8764	16.12598	15.69841	0.709160217	-0.289509773	-0.621551196	0.332041423
1000	Q96CF2	CHMP4C	charged multivesicular body protein 4C	92421	ENSG00000164695	RKPGMSSTARRSRAASSQRAEEEDDDIKQLA	S	0.5	214	15.9462	15.92435	15.28901	16.00683	15.94887	15.85033	15.5411	16.07636	15.51008	0.23516695	0.010673205	-0.215491613	0.226164818
1001	Q96CF2	CHMP4C	charged multivesicular body protein 4C	92421	ENSG00000164695	KPGMSSTARRSRAASSQRAEEEDDDIKQLAA	S	0.5	215	15.9462	15.92435	15.28901	16.00683	15.94887	15.85033	15.5411	16.07636	15.51008	0.23516695	0.010673205	-0.215491613	0.226164818
914	Q86SU0	ILDR1	immunoglobulin like domain containing receptor 1	286676	ENSG00000145103	KPPSYRSLDITPGKNSRKKGSVERRSEKDSS	S	0.999896	434	15.94061	16.32925	16.06643	16.34735	15.92706	16.01443	15.52839	15.85028	15.76155	1.094416664	0.398687681	0.015817006	0.382870674
1378	Q13131	PRKAA1	protein kinase AMP-activated catalytic subunit alpha 1	5562	ENSG00000132356	RSIDDEITEAKSGTATPQRSGSVSNYRSCQR	T	0.992186	490	15.93221	15.87891	15.86868	15.97723	15.85138	16.33081	15.61019	15.48315	15.88517	0.977721753	0.233763695	-0.159874916	0.393638611
332	Q9Y485	DMXL1	Dmx like 1	1657	ENSG00000172869	NSSSGLHPPKKTLTRSMTSLAQKICGKKTAF	S	0.984371	1285	15.93212	16.13282	15.891	16.22811	15.97699	16.25612	16.13348	15.84215	16.53506	0.231708793	-0.184919039	-0.168422699	-0.01649634
1176	Q9Y3R5	DOP1B	DOP1 leucine zipper like protein B	9980	ENSG00000142197	SPFKTKSSESPSSSPSSPARKNGGEWDVEKV	S	0.959852	720	15.92866	16.01129	15.75303	16.14983	15.77697	15.95197	15.23855	15.53089	16.05848	0.498592081	0.288352966	-0.06192716	0.350280126
522	O43896	KIF1C	kinesin family member 1C	10749	ENSG00000129250	RYPPYTTPPRMRRQRSAPDLKESGAAV____	S	1	1092	15.91712	15.59849	15.59502	15.59179	15.91705	15.63135	15.46936	15.70052	16.09795	0.018048022	-0.052399635	-0.009850184	-0.042549451
312	F6UYG0	WNK1	WNK lysine deficient protein kinase 1	65125	ENSG00000060237	QPSLVGSKEEPPPARSGSGGGSAKEPQEERS	S	0.509758	183	15.91684	16.28989	16.23004	16.15638	16.10558	16.20503	15.81863	15.93682	16.25361	0.274562158	0.142568906	-0.010074615	0.152643522
1342	P22732	SLC2A5	solute carrier family 2 member 5	6518	ENSG00000142583	EVYPEKEELKELPPVTSEQ____________	T	0.91778	498	15.91413	15.98313	15.63552	16.10478	16.01579	16.13675	15.65153	16.0095	16.05793	0.564237717	-0.062060356	-0.241514206	0.17945385
626	P10909	CLU	clusterin	1191	ENSG00000120885	CLKQTCMKFYARVCRSGSGLVGRQLEEFLNQ	S	0.996718	98	15.90607	15.47801	15.59386	15.93648	16.67934	15.95842	15.69979	15.67082	15.73339	0.996137094	-0.042019844	-0.532099406	0.490079562
921	S4R3V8	LSR	lipolysis stimulated lipoprotein receptor	51599	ENSG00000105699	GSYVPLLRDTDSSVASEVRSGYRIQASQQDD	S	0.999992	337	15.9016	15.84784	15.53488	15.53725	16.02178	15.71604	15.45741	15.91403	15.63883	0.067367195	0.091346423	0.003081957	0.088264465
1377	Q12929	EPS8	epidermal growth factor receptor pathway substrate 8	2059	ENSG00000151491	KNKKGKRKGPGEGVLTLRAKPPPPDEFLDCF	T	1	317	15.9004	16.17407	15.63623	15.79261	15.74969	16.00026	15.57388	15.98797	15.95599	0.03293532	0.06428051	0.056042989	0.008237521
63	H7BYZ6	ASB3	ankyrin repeat and SOCS box containing 3	100302652;51	ENSG00000115239	EGNVKVLRKLLKKGRSVDVADNRGWMPIHEA	S	1	28	15.89981	16.00959	15.8219	16.29117	15.9377	15.88977	15.92539	15.7019	15.92437	0.420227112	0.05987517	-0.129113197	0.188988368
527	O60343	TBC1D4	TBC1 domain family member 4	9882	ENSG00000136111	VHEGSQKSQPRRRHASAPSHVQPSDSEKNRT	S	0.9525	341	15.89254	15.30656	15.45227	15.93429	15.782	15.7103	15.7003	15.72089	15.55821	0.477555994	-0.109341304	-0.258402824	0.149061521
30	C9J784	MDFIC	MyoD family inhibitor domain containing	29969	ENSG00000135272	IHHGAKHGSADNRKLSAPVSQKMHRKIQSSL	S	0.997574	69	15.88677	15.98618	15.79114	16.05178	15.77756	16.10607	16.31178	16.11468	15.40697	0.030209541	-0.056449254	-0.090445201	0.033995946
67	A0A0C4DFY5	GPRC5C	G protein-coupled receptor class C group 5 member C	55890	ENSG00000170412	SANSTLRAEDMYSAQSHQAATPPKDGKNSQV	S	0.861855	463	15.87669	16.0531	15.70139	16.10985	15.7204	15.91118	15.89907	15.62502	15.72633	0.292047334	0.12691625	-0.036755562	0.163671811
533	O60784	TOM1	target of myb1 membrane trafficking protein	10043	ENSG00000100284	AGMNLGSSSVRAGLQSLEASGRLEDEFDMFA	S	0.996133	310	15.8564	15.9511	15.86128	16.03261	16.07911	15.90428	15.67986	15.66725	16.21845	0.203061906	0.034406026	-0.115737597	0.150143623
1145	Q9UHR4	BAIAP2L1	BAR/IMD domain containing adaptor protein 2 like 1;BAI1 associated prc	55971	ENSG00000006453	IKTPASTPVSGTPQASPMIERSNVVRKDYDT	S	0.98336	261	15.85326	15.69664	15.64338	15.39814	15.97428	15.95944	15.83022	15.70633	15.62202	0.027628656	0.011568069	-0.046193123	0.057761192
348	F5H875	CHMP1A	charged multivesicular body protein 1A	5119	ENSG00000131165	SAVGESSVRSQEDQLSRRLAALRN_______	S	0.999999	124	15.85172	16.22861	15.29211	15.83459	15.63911	16.20684	15.67629	15.62268	15.83684	0.098215121	0.078878403	-0.102702777	0.181581179
1252	E9PCN4	EVC	EvC ciliary complex subunit 1	2121	ENSG00000072840	NLLKNLESNAQTPSETGSPSRRRKREVQMSK	T	0.507719	79	15.84799	15.29523	15.00048	16.2018	17.19633	15.78762	15.75919	15.60342	15.93	0.947109082	-0.382968267	-1.014017423	0.631049156
400	H0YJH0	SAV1	salvador family WW domain containing protein 1	60485	ENSG00000151748	RRESNRLSAPSYLARSLADVPREYGSSQSFV	S	1	34	15.844	16.24771	15.8265	16.44676	16.28964	16.03387	15.75567	16.34129	16.26318	0.342505535	-0.147313754	-0.284025192	0.136711438
1031	Q9BV36	MLPH	melanophilin	79083	ENSG00000115648	PEARDSPQSLTDESCSEKAAPHKAEGLEEAD	S	0.969229	233	15.83455	16.31861	15.96903	16.14211	15.87195	16.11417	15.11586	15.57669	15.92701	0.907948436	0.500874519	-0.002015114	0.502889633
190	C9JRC4	PPP1R7	protein phosphatase 1 regulatory subunit 7	5510	ENSG00000115685	____MAAERGAGQQQSQEMMEVDRRVESEES	S	1	12	15.83427	16.22323	15.54031	15.61488	15.31487	15.42699	15.53181	15.42098	15.69221	0.825833348	0.317605972	0.413692474	-0.096086502

874	Q9H4G4	GLIPR2	GLI pathogenesis related 2	152007	ENSG00000122694	KNLNREAQQYSEALASTRILKHSPESSRGQC	S	0.735196	48	15.83163	15.91861	15.44113	15.85822	15.293	15.79596	15.32175	15.70714	15.68332	0.114992974	0.159715017	0.081390699	0.078324318
105	A0A0U1RQQ1	CARMIL1	capping protein regulator and myosin 1 linker 1	55604	ENSG00000079691	VLKKVPSDKERDGQSSPQPSPRTFSQEACPT	S	0.9829	915	15.83138	15.68519	15.75431	15.77193	15.73039	15.81598	15.20262	15.3479	15.7446	1.08210661	0.325254758	-0.015806834	0.341061592
198	D6RE77	RAPGEF6	Rap guanine nucleotide exchange factor 6	51735	ENSG00000158987	MLDVQGGAHKKRARRSSLLNAKKLYEDAQMA	S	0.5	816	15.8296	15.58804	15.77512	16.10609	15.70236	15.65807	15.50618	15.42659	15.7211	0.586593398	0.17962869	-0.09125487	0.27088356
199	D6RE77	RAPGEF6	Rap guanine nucleotide exchange factor 6	51735	ENSG00000158987	LDVQGGAHKKRARRSSLLNAKKLYEDAQMAR	S	0.5	817	15.8296	15.58804	15.77512	16.10609	15.70236	15.65807	15.50618	15.42659	15.7211	0.586593398	0.17962869	-0.09125487	0.27088356
1539	Q12929	EPS8	epidermal growth factor receptor pathway substrate 8	2059	ENSG00000151491	QTDREHGSKTSAKALYEQRKNYARDSVSSVS	Y	1	45	15.82314	15.95946	15.85153	15.98386	15.68184	16.00062	16.03856	16.07427	15.47649	0.00432992	0.014938672	-0.010730108	0.02566878
998	Q96C24	SYTL4	synaptotagmin like 4	94121	ENSG00000102362	KLDHCLPLHGKISAESPTGLPSHKGELVVSL	S	0.758886	488	15.81586	15.62076	15.48803	16.01064	15.66395	15.72598	15.50541	15.44288	15.53497	0.968742613	0.147130966	-0.158637365	0.305768331
1475	Q9UBY5	LPAR3	lysophosphatidic acid receptor 3	23566	ENSG00000171517	FSQENPERRPSRIPSTVLSRSDTGSQYIEDS	T	0.788917	326	15.81583	15.27267	16.63453	16.07909	14.14975	15.06141	16.01831	15.50047	15.42564	0.382967707	0.259536107	0.810926119	-0.551390012
452	J3KRZ2	DYNC1LI2	dynein cytoplasmic 1 light intermediate chain 2	1783	ENSG00000135720	TPTRASESPARGPSGSPRTQGRGGPASVPSS	S	0.987267	17	15.81393	15.35052	15.62788	15.60658	15.20858	15.43817	15.32646	14.9443	15.23504	0.92724567	0.428841909	0.179667473	0.249174436
1249	E7EQB2	LTF	lactotransferrin	4057	ENSG00000012223	ADKGQFPNLCRLCAGTGENKCAFSSQEPYFS	T	1	182	15.81381	15.17816	15.97495	15.60273	15.6862	15.76648	15.98824	15.68489	15.47047	0.013386943	-0.058892568	-0.029495557	-0.029397011
103	A0A0U1RQF0	FASN	fatty acid synthase	2194	ENSG00000169710	TAHAFVSTLTRGDLSSIRWVCSSLRHAQPTC	S	0.987505	1541	15.80511	15.94068	15.56686	15.92247	15.62995	16.00838	15.51132	15.63078	15.93014	0.197223546	0.080138206	-0.082718849	0.162857056
1380	Q13404	UBE2V1;TMEM189-UBE2	ubiquitin conjugating enzyme E2 V1;TMEM189-UBE2V1 readthrough	7335;387522	ENSG00000244687	____________MAATTGSGVKVPRNFRLLE	T	0.953833	4	15.79875	16.01926	15.32618	15.54885	15.5205	16.11199	15.24989	15.40098	15.6515	0.338289638	0.280609131	-0.012380918	0.292990049
345	J3QS47	CYB561	cytochrome b561	1534	ENSG00000008283	LSMDFKTLTEGDSPGSQ______________	S	0.99999	221	15.79594	15.7657	15.95851	16.37188	15.78992	16.19441	15.90961	16.03293	16.03634	0.576760723	-0.152912776	-0.278686523	0.125773748
85	A0A0C4DGY6	DOCK7	dedicator of cytokinesis 7	85440	ENSG00000116641	DGNSYQDDQDDLKRRSMSIDDTPRGSWACSI	S	0.971017	70	15.79022	15.6874	15.22193	15.77618	15.62978	15.75676	15.28587	15.61999	15.45276	0.467072221	0.113644282	-0.154390971	0.268035253
200	C9K058	PPIL3	peptidylprolyl isomerase like 3	53938	ENSG00000240344	MVQTGDPTGTGRGGNSIWGKKFEDEYSEYLK	S	1	65	15.789	15.61477	15.67904	15.89114	15.48529	15.85655	15.32745	15.1909	16.07291	0.161935067	0.163849195	-0.050059636	0.213908831
1229	A6NMY6	ANXA2P2	annexin A2 pseudogene 2	304	ENSG00000231991	_____________MSTVHEILCKLSLEGDHS	T	0.5	3	15.78486	15.88369	15.39767	16.04685	15.83649	16.0898	15.97697	15.94265	15.91289	0.856696361	-0.255425135	-0.302303314	0.046878179
156	A6NMY6	ANXA2P2	annexin A2 pseudogene 2	304	ENSG00000231991	______________MSTVHEILCKLSLEGDH	S	0.5	2	15.78486	15.88369	15.39767	16.04685	15.83649	16.0898	15.97697	15.94265	15.91289	0.856696361	-0.255425135	-0.302303314	0.046878179
396	H0YER8	ASAP3	ArfGAP with SH3 domain, ankyrin repeat and PH domain 3	55616	ENSG00000088280	TLQLESREEHLSRKNSGCGYSIHQHQGNKQF	S	0.832236	91	15.78077	15.28598	15.7401	16.14637	15.85587	15.84206	15.97444	15.88403	15.77304	0.814943555	-0.274884224	-0.345814705	0.070930481
498	O15037	KHNYN	KH and NYN domain containing	23351	ENSG00000100441	GGGFCVHREPPGAHGSCHRAAQSRGASLLQR	S	1	331	15.77677	15.44666	15.18747	15.42797	15.87133	15.75768	15.4587	15.1191	15.43329	0.55181618	0.133268992	-0.215357463	0.348626455
481	O00308	WWP2	WW domain containing E3 ubiquitin protein ligase 2	11060	ENSG00000198373	HSGASARTTPATGEQSPGARSRHRQPVKNSG	S	0.999999	211	15.76658	15.94022	15.31044	15.68803	15.66952	15.99475	15.10587	15.31511	15.53704	0.870783298	0.353073438	-0.111688932	0.46476237
655	P21399	ACO1	aconitase 1	48	ENSG00000122729	VIDHSIQVDFNRRADSLQKNQDLEFERNRER	S	1	138	15.76554	15.49851	15.24956	15.67546	15.58895	15.85336	15.59744	15.1827	16.11425	0.127494424	-0.126929283	-0.201386452	0.074457169
133	A0A2R8Y549	PATJ	PATJ crumbs cell polarity complex component	10207	ENSG00000132849	PVITGLQNLVGTKRVSDPSQKNSGTDMEPRT	S	0.880042	1661	15.76446	15.85484	15.52632	16.02767	15.70041	16.103	15.49838	15.39814	15.78435	0.884951258	0.154912949	-0.228488286	0.383401235
1106	Q9NV70	EXOC1	exocyst complex component 1	55763	ENSG00000090989	FGLHGSSGKLTGSTSSLNKLSVQSSGNRRSQ	S	0.990353	458	15.75968	16.37436	15.80735	16.15583	16.33141	16.30684	15.61965	15.67943	15.94759	1.073589922	0.231577555	-0.284227689	0.515805244
1072	Q9H0B6	KLC2	kinesin light chain 2	64837	ENSG00000174996	GLSDSRTLSSSSMDLSRRSSLVG________	S	0.986051	615	15.75835	15.63464	15.53473	15.70704	15.86665	15.76839	15.98101	15.55827	15.84804	0.366882127	-0.153198242	-0.138118426	-0.015079816
877	Q68DQ2	CRYBG3	crystallin beta-gamma domain containing 3	131544	ENSG00000080200	QGMENMSEVKEKPCVSPTVGEKNLLVDPNSM	S	0.661669	1280	15.75833	15.78887	15.95635	16.01498	15.24885	15.77396	15.80766	15.44666	15.90351	0.109242828	0.115242004	0.155251503	-0.040009499
320	F5GYU8	TMC5	transmembrane channel like 5	79838	ENSG00000103534	YPGSQRNPDFAGSSSSGNYAGSRTHPDHFGS	S	0.749791	143	15.7576	15.69998	15.68803	16.14069	15.74683	15.40394	16.34596	16.12789	16.09822	1.096253494	-0.475486755	-0.048617999	-0.426868757
720	P62070	RRAS2	RAS related 2	22800	ENSG00000133818	LVRVIRKFQEQECPPSPEPTRKEKDKKGCHC	S	0.951527	109	15.75332	15.97639	15.48208	16.12477	15.84853	15.87368	15.67085	15.71896	15.92546	0.416069253	-0.034494082	-0.21172905	0.177234968
450	M0R3H9	BABAM1	BRISC and BRCA1 A complex member 1	29086	ENSG00000105393	EEEEEHSAEPRPRTRSNPEGAEDRAVGAQAS	S	0.994603	29	15.75005	16.08715	15.8739	16.01637	15.60105	15.90435	15.92671	15.65696	15.7728	0.13853356	0.118210793	0.063111623	0.055099169
389	H0YB27	PIK3R1	phosphoinositide-3-kinase regulatory subunit 1	5295	ENSG00000145675	EKKGLECSTLYRTQSSSNLAELRQLLDCDTP	S	0.601248	57	15.74935	15.6049	15.19533	15.75209	15.65684	15.50028	15.58376	14.65631	15.72898	0.206227769	0.193508466	-0.119879723	0.313388189
1311	O43581	SYT7	synaptotagmin 7	9066	ENSG00000011347	APVPGQTPHDESDRRTEPRSSVSDLVNSLTS	T	0.499951	173	15.74817	15.50993	15.40557	15.47491	15.74323	15.83398	15.31511	15.53357	15.16722	0.847543945	0.215920448	-0.129481951	0.3454024
517	O43581	SYT7	synaptotagmin 7	9066	ENSG00000011347	AVNTAPVPGQTPHDESDRRTEPRSSVSDLVN	S	0.499951	169	15.74817	15.50993	15.40557	15.47491	15.74323	15.83398	15.31511	15.53357	15.16722	0.847543945	0.215920448	-0.129481951	0.3454024
1160	Q9UQN3	CHMP2B	charged multivesicular body protein 2B	25978	ENSG00000083937	IEISGKMAKAPSAARSLPSASTSKATISDEE	S	0.998064	146	15.73577	15.75275	15.07803	15.40321	15.67827	15.73846	15.34783	15.27307	15.32045	0.415647929	0.208398501	-0.084467888	0.292866389
1105	Q9NV70	EXOC1	exocyst complex component 1	55763	ENSG00000090989	SKKFGLHGSSGKLTGSTSSLNKLSVQSSGNR	S	0.990101	455	15.7276	15.82692	15.29254	15.72118	15.63943	15.8066	15.51228	15.52286	15.54191	0.372155705	0.090003649	-0.106714884	0.196718534
170	M0R088	SRRM1	serine and arginine repetitive matrix 1	10250	ENSG00000133226	HRPSPPATPPPKTRHSPTPQQSNRTRKSRVS	S	0.935032	375	15.72558	15.12989	15.04895	15.44844	15.15644	14.23175	15.79873	14.87867	15.08957	0.17714914	0.045815786	0.355932554	-0.310116768
1115	Q9P1Y5	CAMSAP3	calmodulin regulated spectrin associated protein family member 3	57662	ENSG00000076826	WVIPGPTTGPKAASPSPARRVPATRRSPGPG	S	0.947566	758	15.71727	15.98235	15.94382	16.33584	15.84926	15.97934	16.01478	15.62134	16.02074	0.25438548	-0.00447305	-0.173665365	0.169192314
687	P46937	YAP1	Yes associated protein 1	10413	ENSG00000137693	SFFKPPEPKSHSRQASTDAGTAGALTPQHVR	S	0.743563	109	15.71604	15.64436	15.47991	15.84235	16.1329	15.82938	16.06097	15.70962	15.48724	0.62051779	-0.139174779	-0.321438789	0.18226401
747	Q02383	SEMG2	semenogelin 2	6407	ENSG00000124157	MGQKGGSKGQLPSGSSQFPHGQKGQHYFGQK	S	0.863433	37	15.70739	15.03802	15.76671	15.17192	15.39034	15.25588	16.14601	15.79586	15.76332	1.12675168	-0.397691409	0.231327375	-0.629018784
59	A0A0A0MT60	FKBP15	FKBP prolyl isomerase 15	23307	ENSG00000119321	SEEEEEKAEERPRRPSQEQSASASSGQPQAP	S	0.999995	981	15.7019	15.97347	16.20665	16.27232	15.67082	15.85903	16.11054	15.85837	16.18526	0.0764631	-0.090716362	0.026620229	-0.117336591
1407	Q5T0Z8	C6orf132	chromosome 6 open reading frame 132	647024	ENSG00000188112	______MKKKQTVQGTFSKLFGKKHTTTPST	T	0.999173	10	15.70182	15.69471	15.73357	15.63535	16.02115	15.89117	16.05352	15.6581	15.50056	0.165926478	-0.02736028	-0.139189084	0.111828804
1327	P04279	SEMG1	semenogelin 1	6406	ENSG00000124233	NGVQKDVSQRSIYSQTEKLVAGKSQIQAPNP	T	0.9916	378	15.69952	15.10169	15.42042	15.37518	15.40893	14.96781	15.72896	15.88741	15.20656	0.388308728	-0.20043246	0.15657107	-0.35700353
1337	P14625	HSP90B1	heat shock protein 90 beta family member 1	7184	ENSG00000166598	MDVGTDEEEETAKESTAEKDEL_________	T	0.837788	797	15.69697	15.36198	14.88374	15.50103	16.42136	15.50739	15.87779	15.78533	15.97015	0.630714379	-0.563525836	-0.495697657	-0.067828178
1078	Q9H2G2	SLK	STE20 like kinase	9748	ENSG00000065613	ACILESVSEKTERSNSEDKLNSKILNEKPTT	S	0.978936	372	15.694	15.52906	15.8476	15.80582	15.58352	16.07289	15.51847	15.29132	15.77844	0.476114758	0.160814603	-0.130521456	0.29133606
609	P04792	HSPB1	heat shock protein family B (small) member 1	3315	ENSG00000106211	AVAAPAYSRALSRQLSSGVSEIRHTADRWRV	S	0.964675	82	15.69052	15.29692	15.21421	15.87056	15.60851	15.44126	15.5545	15.68757	15.29688	0.327036885	-0.112435977	-0.23956426	0.127128283
1455	Q9BW04	C1orf116;SARG	chromosome 1 open reading frame 116	79098	ENSG00000182795	PLSSGEDPNSRLAPLTTPKPRKLPPNIVLKS	T	0.982146	294	15.69024	15.93475	15.26239	15.65433	15.74099	15.99212	15.12868	15.48828	15.44812	0.766848882	0.274102529	-0.166686058	0.440788587
294	R4GNF5	TCP11L1	t-complex 11 like 1	55346	ENSG00000176148	IKSDSSSPQRVQRPHSSPPRFVTVEELLETA	S	0.767452	55	15.68871	15.97446	15.38212	15.80665	15.65458	16.10673	15.6753	15.37874	15.87644	0.225802466	0.038271904	-0.174221357	0.212493261
755	Q02383	SEMG2	semenogelin 2	6407	ENSG00000124157	QDQEHGHKENKMSYQSSSTEERRLNYGGKST	S	0.825076	470	15.68019	15.05621	14.54466	15.36273	16.49475	15.31865	16.08516	15.78128	15.20262	0.428949572	-0.59599781	-0.631685257	0.035687447
1093	Q9NQT8	KIF13B	kinesin family member 13B	23303	ENSG00000197892	AVKEQLTGKGKLSRRSISSPNVNRLSGSRQD	S	0.994664	1379	15.67802	15.41782	15.54738	15.75718	15.11647	15.59065	14.72185	15.37052	15.47751	0.414051122	0.357783318	0.059642156	0.298141162
775	Q09666	AHNAK	AHNAK nucleoprotein	79026	ENSG00000124942	GKGKKSKFKMPKIHMSGPKIKAKKQGFDLNV	S	1	5031	15.67783	15.7489	15.51884	15.95429	15.70498	15.81486	15.5181	15.66675	15.51493	1.146057878	0.081929207	-0.176187197	0.258116404
851	Q5R3F8	ELFN2	extracellular leucine rich repeat and fibronectin type III domain containing	114794	ENSG00000166897	PPYKESSHHPLQRQLSADAAVTRKTCSVSSS	S	0.999861	619	15.67226	15.50879	15.30567	16.04919	15.55516	15.70155	14.96542	15.43609	15.52827	0.797224952	0.185642242	-0.273061117	0.458703359
693	P50151	GNG10	G protein subunit gamma 10	2790	ENSG00000242616	______________MSSGASASALQRLVEQL	S	0.5	2	15.67162	15.96181	15.70455	16.35551	16.3422	15.90011	16.38443	16.03913	16.06895	1.063564465	-0.384840965	-0.419942538	0.035101573
694	P50151	GNG10	G protein subunit gamma 10	2790	ENSG00000242616	_____________MSSGASASALQRLVEQLK	S	0.5	3	15.67162	15.96181	15.70455	16.35551	16.3422	15.90011	16.38443	16.03913	16.06895	1.063564465	-0.384840965	-0.419942538	0.035101573
1090	Q9HCS5	EPB41L4A	erythrocyte membrane protein band 4.1 like 4A	64097	ENSG00000129595	RTKSPKFPYTRRRNPSCGSDNDSVQPVRRRK	S	0.993032	445	15.67135	15.40298	15.48126	15.72425	15.54909	15.75008	15.48646	15.79444	15.87594	0.487661081	-0.200416883	-0.155942917	-0.044473966
990	Q96AQ6	PBXIP1	PBX homeobox interacting protein 1	57326	ENSG00000163346	QGPKEPPRKSGSFHSSGEKQKQPRWREGTKE	S	0.832078	551	15.66927	15.60467	15.25886	15.93827	16.2556	15.71048	16.19397	15.60374	15.9752	0.834599524	-0.413369179	-0.457184156	0.043814977
1474	Q9UBI6	GNG12	G protein subunit gamma 12	55970	ENSG00000172380	________MSSKTASTNNIAQARRTVQQLRL	T	0.953008	8	15.66872	15.14788	15.0574	15.3711	15.15086	15.43019	15.07652	15.01459	14.68612	0.783168644	0.365590413	-0.026048342	0.391638756
1266	F8W6G1	NRBP1	nuclear receptor binding protein 1	29959	ENSG00000115216	QQEEVTSPVVPPSVKTPTPEPAEVETRKVVL	T	0.554349	439	15.66359	15.71754	15.83913	16.37368	15.91837	15.67016	15.85628	15.67027	15.97022	0.337837164	-0.092169444	-0.24731795	0.155148506
695	P50151	GNG10	G protein subunit gamma 10	2790	ENSG00000242616	__________MSSGASASALQRLVEQLKLEA	S	0.898458	6	15.64563	15.53613	15.99696	15.99287	16.23437	15.65958	15.97925	16.00913	15.67187	0.288130777	-0.160511017	-0.236035029	0.075524012
1088	Q9HCC9	ZFYVE28	zinc finger FYVE-type containing 28	57732	ENSG00000159733	PGGEASPGRPRLRSGSDEEERVFFMDDVEGT	S	0.688226	280	15.64495	15.31469	15.1566	15.3138	15.67527	15.33102	15.60406	15.20492	14.89888	0.172603582	0.136126518	-0.06795152	0.204078039
152	H3BU16	JPT2	Jupiter microtubule associated homolog 2	90861	ENSG00000206053	AARSIPAGAEPGEKGSARKAGPAKEQEPMPT	S	0.999995	128	15.64436	15.42331	15.44116	15.84245	15.71612	15.6918	15.65947	15.47367	15.836	0.807105323	-0.153434753	-0.247177124	0.093742371
1503	C9J1X3	TNK2	tyrosine kinase non receptor 2	10188	ENSG00000061938	GDFGLMRALPQNDDHYVMQEHRKVPFAWCAP	Y	1	316	15.64104	15.55105	15.4795	16.33002	15.87167	15.57999	16.04072	15.86832	15.46384	0.477784148	-0.23376592	-0.370034536	0.136268616
643	P16152	CBR1	carbonyl reductase 1	873	ENSG00000159228	RVVNVSSIMSVRALKSCSPELQQKFRSETIT	S	0.5	149	15.63569	16.29951	16.06601	16.14661	16.17463	15.86004	15.79373	15.66372	15.88786	0.450096883	0.218629837	-0.060025851	0.278655688
777	Q09MP3	RAD51AP2	RAD51 associated protein 2	729475	ENSG00000214842	EDYHCAKVINAYEEQSKLLVREILGSQTALI	S	0.986485	459	15.63436	15.08804	15.63589	15.9008	15.89685	15.53524	15.33933	15.20277	15.41953	0.944763188	0.132218043	-0.324868202	0.457086245
128	A0A2R8Y2X6	BICDL2	BICD family like cargo adaptor 2	146439	ENSG00000162069	THEELLLLRRERREHSLELERARSEAGEALS	S	1	285	15.63305	15.41821	15.02505	15.9467	15.66206	15.62671	15.56775	15.47927	15.35139	0.813276288	-0.107363701	-0.386381467	0.279017766
651	P18669	PGAM1	phosphoglycerate mutase 1	5223	ENSG00000171314	QIKEGKRVLIAAHGNSLRGIVKHLEGLSEEA	S	1	189	15.63288	16.1543	15.79551	16.13653	15.96439	15.97715	15.80673	16.09789	16.02546	0.246782288	-0.115794818	-0.165122986	0.049328168
1022	Q9BSW2	CRACR2A	calcium release activated channel regulator 2A	84766	ENSG00000130038	PDGRVVSRPQRLGQGSGQGPKGSGACLHPLD	S	1	19	15.63044	15.49214	15.65866	15.96443	15.14007	15.51117	15.2906	14.81102	15.93429	0.127636032	0.248440742	0.055187861	0.193252881
506	O43182	ARHGAP6	Rho GTPase activating protein 6	395	ENSG00000047648	GQAAEREQQVTQKKLSSANSLPAGEQDSPRL	S	0.957033	724	15.62836	16.39783	15.50128	16.18998	15.81348	16.35106	15.63458	15.88565	15.84505	0.326366376	0.054059982	-0.275686264	0.329746246
8	E9PJZ9	RELA	RELA proto-oncogene, NF-kB subunit	5970	ENSG00000173039	__MRFRYKCEGRSAGSIPGERSTDTTKTHPT	S	0.998407	14	15.62742	14.71204	15.39003	15.89245	14.81623	15.54735	15.34759	14.74141	15.10644	0.183229525	0.178018888	-0.175510089	0.353528976
1430	Q8IY63	AMOTL1	angiomotin like 1	154810	ENSG00000166025	EADMTKWEQKYLEESTIRHFAMNAAATAAAE	T	0.988152	646	15.61859	15.74712	15.34228	16.32494	16.00187	15.85207	15.53792	16.11073	15.69076	0.8763841	-0.210472425	-0.490294456	0.279822032
107	K7ES72	CAPS	calcyphosine	828	ENSG00000105519	ATMEKLRAQCLSRGASGIQGLARFFRQLDRD	S	1	21	15.61767	15.41419	15.18604	15.47031	15.3809	15.73719	15.3387	14.86327	15.41907	0.496404672	0.198951721	-0.123503049	0.32245477
394	H0YDD9	ANO1	anoctamin 1	55107	ENSG00000131620	VYEILKRTTCTKAKYSMGQGEGRKKDSALLS	S	0.999979	108	15.61615	16.36169	16.68072	16.00852	16.46909	16.02883	16.34497	17.42169	16.3289	0.379277327	-0.479003588	0.050705274	-0.529708862
274	E7ES96	PSEN1	presenilin 1	5663	ENSG00000080815	EEWEAQRDSHLGPHRSTPESRAAVQELSSSI	S	0.606154	261	15.61016	15.77123	16.03497	15.79228	16.34225	16.03538	16.18288	16.00114	15.63637	0.277859162	-0.134676615	-0.251182556	0.116505941
1047	Q9BZQ8	NIBAN1;FAM129A	niban apoptosis regulator 1;family with sequence similarity 129 member	116496	ENSG00000135842	CAYIRGKTEAAIKNFSPYYSRQYSVAFCNHV	S	0.994653	29	15.60626	15.6305	15.45712	15.70652	15.46745	15.74846	15.63092	15.42836	15.48284	0.327261674	0.050586383	-0.076181412	0.126767794
322	F5GYU8	TMC5	transmembrane channel like 5	79838	ENSG00000103534	RPDYPGSLAEPNYPRSLSNPDYSGTRSNAYS	S	0.996955	77	15.59289	15.92114	15.01498	15.43375	15.58414	15.57994	15.47804	15.40421	15.67052	0.002238938	-0.007917722	-0.022935549	0.015017827
1156	Q9UPY5	SLC7A11	solute carrier family 7 member 11	23657	ENSG00000151012	LFIIWDKKPRWFRIMSEKITRTLQIILEVVP	S	1	481	15.59196	15.57899	15.83138	15.88486	16.01317	15.46084	15.62676	16.15677	15.68143	0.128597373	-0.15421168	-0.11884594	-0.03536574
647	P18206	VCL	vinculin	7414	ENSG00000035403	AWASKDTEAMKRALASIDSKLNQAKGWLRDP	S	0.968036	272	15.59009	15.51948	15.14318	15.51665	15.60087	15.20277	15.40411	15.04683	15.41742	0.165845067	0.128132184	-0.022514343	0.150646528
1075	Q9H0M0	WWP1	WW domain containing E3 ubiquitin protein ligase 1	11059	ENSG00000123124	ECIPSTSAELESEARSILEPDTSNSRSSSAF	S	0.999342	91	15.58305	16.12004	15.18546	15.50361	15.54318	15.97026	14.70536	15.5336	15.48633	0.376817029	0.387749036	-0.042836825	0.430585861
585	P03928	MT-ATP8;ATP8	mitochondrially encoded ATP synthase membrane subunit 8;ATP syntha	4509	ENSG00000228253	NKPWEPKWTKICSLHSLPPQS__________	S	0.846707	63	15.57637	15.52836	15.74257	15.70461	15.87594	15.76785	15.90186	15.94471	15.46477	0.328328796	-0.154678663	-0.167031288	0.012352626
138	A0A2R8YD75	SNX27	sorting nexin family member 27	81609	ENSG00000143376	__________IVKSESGYGFNVRGQEPREC_	S	0.998613	6	15.57513	15.55684	15.2027	15.53351	15.75228	15.8881	15.87992	15.77672	15.52726	0.71180982	-0.283079465	-0.279743512	-0.003335953
285	E7EX40	RAB11FIP1	RAB11 family interacting protein 1	80223	ENSG00000156675	ESSSASDVMSHKRTASTDLKQLNQVNFTLPK	S	0.975715	132	15.57027	15.63376	14.86298	15.08053	15.86341	15.5695	15.1727	15.12695	14.67513	0.531042111	0.364076932	-0.148810069	0.512887001
295	E9PJP2	SOGA3	SOGA family member 3	387104	ENSG00000214338	DFNGSANPLMREQSESLSELRQHLQLVEDET	S	0.596903	641	15.56591	16.73331	15.22573	16.49837	16.15571	15.9359	16.49204	16.23557	15.62457	0.142076287	-0.275741895	-0.355009397	0.079267502
1464	Q9H2G2	SLK	STE20 like kinase	9748	ENSG00000065613	VFFKMTGESECLNPSTQSRISKFYPIPSLHS	T	0.740918	1186	15.56036	15.87426	14.99828	15.83385	15.70663	15.64473	15.25956	15.55183	15.41445	0.413201524	0.069020907	-0.250769615	0.319790522
1226	A0AV02	SLC12A8	solute carrier family 12 member 8	84561	ENSG00000221955	RKLESSQPRQGEGNRTPESQKRKSKKATKQT	T	0.999168	79	15.55657	16.72294	16.77546	16.68373	16.71652	16.482	16.54123	16.60638	16.89273	0.212896388	-0.328460058	-0.275767008	-0.052693049
1180	Q9Y4H2	IRS2	insulin receptor substrate 2	8660	ENSG00000185950	QPIAAPPKPEAARVASPTSGVKRLSLMEQVS	S	0.660757	1100	15.55549	15.82287	15.72792	16.07976	15.8867	15.7609	15.71304	15.95613	16.03036	0.589391291	-0.197750409	-0.207025528	0.009275119
1461	Q9H0B6	KLC2	kinesin light chain 2	64837	ENSG00000174996	GSWYKACKVDSPTVNTTLRSLGALYRRQGKL	T	0.974331	450	15.55537	15.87347	15.63733	16.08354	15.7052	15.40335	15.02639	14.90745	15.65606	0.813189385	0.492086093	-0.041972796	0.534058889
794	Q13796	SHROOM2	shroom family member 2	357	ENSG00000146950	YSEESTPADLGPRAQSPGSPLHARGQDSWPV	S	0.635217	1036	15.55438	15.79175	15.63257	15.81163	15.92012	16.10771	15.50022	15.76818	15.80741	0.990850995	-0.032370885	-0.286918958	0.254548073
1234	B0QZ77	MMS19	MMS19 homolog, cytosolic iron-sulfur assembly component	64210	ENSG00000155229	SLIGKKEGRRKKLPCTETERGSKAKRGNSAC	T	0.986581	106	15.553	15.47056	15.33403	15.43967	15.79637	15.54361	15.70949	15.78642	15.72526	1.089462486	-0.287859281	-0.14068381	-0.147175471
210	C9J4C4	FLCN	folliculin	201163	ENSG00000154803	_______MNSRMRAHSPAEGASVESSSPGPK	S	0.999986	9	15.5404	15.275	14.76973	15.06638	15.23287	15.50162	15.08377	15.22912	14.99528	0.117505454	0.092320442	-0.071914037	0.164234479
112	A0A1B0GTW1	TJP2	tight junction protein 2	9414	ENSG00000119139	DYDYHSSSEKLKERPSSREDTPSRLSRMGAT	S	0.499998	499	15.54004	15.69968	15.60281	15.82757	15.80768	15.3278	15.44278	15.65874	15.60287	0.071409509	0.046045939	-0.040170987	0.086216927

113	A0A1B0GTW1	TJP2	tight junction protein 2	9414	ENSG00000119139	YDYHSSSEKLKERPSSREDTPSRLSRMGATP	S	0.499998	500	15.54004	15.69968	15.60281	15.82757	15.80768	15.3278	15.44278	15.65874	15.60287	0.071409509	0.046045939	-0.040170987	0.086216927
1179	Q9Y4H2	IRS2	insulin receptor substrate 2	8660	ENSG00000185950	TFSGSAGRLCPSCPASSPKVAYHPYPEDYGD	S	0.593338	619	15.52851	16.04529	15.81698	16.13137	16.12283	15.90539	16.2791	15.65357	16.07427	0.340042716	-0.205384572	-0.256266912	0.050882339
1481	Q9ULR3	PPM1H	protein phosphatase, Mg2+/Mn2+ dependent 1H	57460	ENSG00000111110	AASLRGGVGAPGSPSTPPTRFFTEKKIPHEC	T	0.900621	224	15.52289	15.74581	15.32966	15.59292	15.86436	15.59027	15.20987	15.20262	15.67074	0.574331819	0.171705882	-0.149731318	0.3214372
1095	Q9NQT8	KIF13B	kinesin family member 13B	23303	ENSG00000197892	EQLTGKGKLSRRSISSPNVNRLSGSRQDLIP	S	0.837625	1382	15.52056	15.15897	15.13711	15.30453	14.92597	15.46094	14.94403	14.50891	15.19353	0.586403754	0.390057882	0.04173247	0.348325411
925	Q8IV36	HID1	HID1 domain containing	283987	ENSG00000167861	LQRRRRTPEPLSRTGSQEGTSMEGSRPAAPA	S	0.980641	365	15.51755	15.49611	15.23855	16.11557	15.3166	14.90666	15.50621	15.17372	15.65517	0.002248197	-0.027630806	-0.028873761	0.001242956
1010	Q96PM5	RCHY1	ring finger and CHY zinc finger domain containing 1	25898	ENSG00000163743	ICESYNTAQAGGRRISLDQQ___________	S	1	208	15.5173	15.96396	15.68278	15.79396	15.83289	15.74065	15.81391	15.55564	15.95199	0.051781013	-0.052500407	-0.067821821	0.015321414
768	Q07866	KLC1	kinesin light chain 1	3831	ENSG00000126214	QERNNCLADSRALSASHTDLAH__________	S	0.727527	631	15.51428	15.80418	15.87649	15.73156	16.0289	15.52589	14.94036	15.21424	15.90193	0.486008293	0.379475276	-0.030462583	0.409937859
145	A0A2R8YFI4	PANK2	pantothenate kinase 2	80025	ENSG00000125779	AVGASAEGTRRDRLGSYSGPTSVSRQRVESL	S	0.572298	66	15.51305	15.97403	15.24284	15.54388	15.68669	15.23993	15.39844	15.58939	15.82426	0.057642479	-0.027390798	0.086472511	-0.113863309
393	H0YBT1	BAG4	BCL2 associated athanogene 4	9530	ENSG00000156735	SYSTEVPSTYRSSGNSPTPVSRWIYPQQDCQ	S	0.989027	26	15.50482	15.88867	15.4731	15.83353	15.48935	15.68321	15.54442	15.61459	15.61884	0.065915694	0.029578209	-0.04649957	0.076077779
1468	Q9HCM3	KIAA1549	KIAA1549	57670	ENSG00000122778	KAIREELLRLSQKQSTVQNFHS__________	T	0.777275	712	15.49925	14.66617	13.5173	15.76459	15.11415	14.69158	14.66461	14.27722	14.04832	0.466821491	0.230859121	-0.629197439	0.860056559
957	Q8ND76	CCNY	cyclin Y	219771	ENSG00000108100	CEDKYKDLRRSARKRSASADNLTLPRWSPAI	S	0.589104	270	15.49813	15.22532	15.28117	15.22634	15.38458	15.02877	15.05371	15.24176	14.99126	0.626573554	0.239297867	0.121644974	0.117652893
372	F8WD55	TRPM8	transient receptor potential cation channel subfamily M member 8	79054	ENSG00000144481	NRRNDTLDSTRTLYSSASRSTDLSYSESDLV	S	0.906256	27	15.49751	16.2988	15.35284	16.1023	15.9996	15.80818	15.85185	15.9848	15.95386	0.231714817	-0.213785807	-0.253650347	0.03986454
467	K7EM11	GIPC1	GIPC PDZ domain containing family member 1	10755	ENSG00000123159	GWDMISQRSAGGRPGSGPQLGTGRGTLRLRS	S	0.999992	27	15.49495	15.39961	15.13787	15.21769	15.07284	15.50006	14.93138	14.93129	15.21565	0.734318866	0.318036715	0.080610593	0.237426122
961	Q8NFW9	MYRIP	myosin VIIA and Rab interacting protein	25924	ENSG00000170011	VNFNPQLASRETSDSSEPEEAPHTTDRRARR	S	0.882858	511	15.48618	15.62901	15.73828	15.96177	15.65489	15.59193	15.2243	15.1238	15.95292	0.309650991	0.184147835	-0.118372599	0.302520434
242	Q9UPQ0	LIMCH1	LIM and calponin homology domains 1	22998	ENSG00000064042	KLPDVKKDDMSARRTSHGEPKSAVPFNQYLP	S	0.837701	97	15.48463	15.27168	15.2183	15.06184	15.17008	15.68286	15.0521	14.97204	15.38337	0.218244251	0.189036369	0.019946734	0.169089635
123	A0A1W2PR06	SLC7A2	solute carrier family 7 member 2	6542	ENSG00000003989	YDQPKCSPEKDGLGSSPRVTSKSESQVTMLQ	S	0.902195	480	15.48186	16.01234	15.72089	15.9786	15.65167	15.38788	15.40621	15.2313	15.68874	0.384788877	0.296281497	0.065646172	0.230635325
122	A0A1W2PR06	SLC7A2	solute carrier family 7 member 2	6542	ENSG00000003989	SYDQPKCSPEKDGLGSSPRVTSKSESQVTML	S	0.499944	479	15.48186	16.01234	15.72089	15.9786	15.65167	15.38788	15.40621	15.2313	15.68874	0.384788877	0.296281497	0.065646172	0.230635325
889	Q6P597	KLC3	kinesin light chain 3	147700	ENSG00000104892	LGQLRQYDPPAESQQSESPPRRDSLASLFPS	S	0.627481	165	15.47573	16.18762	16.3015	16.1218	16.26849	16.21434	15.93293	15.60038	16.36559	0.174693412	0.021986008	-0.213256518	0.235242526
1144	Q9UHR4	BAIAP2L1	BAR/IMD domain containing adaptor protein 2 like 1;BAI1 associated prc	55971	ENSG00000006453	MKKQKVKTIFPHTAGSNKTLLSFAQGDVITL	S	0.983057	354	15.47056	15.96585	15.46869	15.90077	15.45166	15.45603	15.2547	15.46732	15.18216	0.608677585	0.333642324	0.032211939	0.301430384
760	Q02383	SEMG2	semenogelin 2	6407	ENSG00000124157	WSGQNAKGKSGQSADSKQDLLSHEQKGRYKQ	S	0.71838	533	15.46897	14.26715	13.82853	14.93378	14.87776	14.70989	15.34145	15.48038	14.65581	0.369589747	-0.637660027	-0.318925858	-0.318734169
421	H7BYT1	CSNK1D	casein kinase 1 delta	1453	ENSG00000141551	RHSRNPATRGLPSTASGRLRGTQEVAPPTPL	S	0.900587	331	15.46471	15.76622	15.5258	15.84115	15.36619	15.54243	15.48385	15.23852	15.44602	0.417948637	0.196112633	0.00232029	0.193792343
692	P50150	GNG4	G protein subunit gamma 4	2786	ENSG00000168243	____MKEGMSNNSTTSISQARKAVEQLKMEA	S	0.601028	12	15.46429	14.77921	14.22852	15.27358	14.84412	15.49567	14.31847	14.73127	14.69496	0.564612679	0.242440224	-0.380448659	0.622888883
1472	Q9UBF8	PI4KB	phosphatidylinositol 4-kinase beta	5298	ENSG00000143393	SDATASISLSSNLKRTASNPKVENEDEPVRL	T	0.852268	292	15.45988	15.77682	15.14458	15.46199	15.50084	15.69398	14.91429	14.76937	15.56051	0.643257077	0.379033724	-0.091844559	0.470878283
185	F8WCJ4	ACSS2	acyl-CoA synthetase short chain family member 2	55902	ENSG00000131069	____MGLPEERVRSGSGSRGQEEAGAGGRAR	S	0.632388	12	15.45959	15.31483	15.4443	15.69933	15.52099	15.68573	15.58575	15.17454	15.50274	0.749202799	-0.014767647	-0.229107857	0.21434021
1017	Q96TA1	NIBAN2;FAM129B	niban apoptosis regulator 2;family with sequence similarity 129 member	64855	ENSG00000136830	YLELIGNSLPGTTAKSGSAPILKCPTQFPLI	S	0.99307	133	15.45895	15.47839	15.42315	15.64256	15.43104	15.59085	15.03403	15.6508	15.48404	0.217242167	0.06387647	-0.101319631	0.165196101
1169	Q9Y3R5	DOP1B	DOP1 leucine zipper like protein B	9980	ENSG00000142197	DGTQSLAANDSSRKNSWEPKPITVPQFKQML	S	1	681	15.43866	16.28334	15.63402	16.63354	15.95642	15.74368	15.6086	15.31232	16.01155	0.363642399	0.141182899	-0.32587115	0.467054049
16	Q9P2T1	GMPR2	guanosine monophosphate reductase 2	51292	ENSG00000100938	KDVLLRPKRSTLKSRSEVDLTRSFSFRNSKQ	S	0.883704	46	15.4295	15.16589	15.47934	16.0031	15.536	15.77836	15.82053	15.09544	15.0756	0.690375614	0.027720451	-0.414244334	0.441964785
802	Q14247	CTTN	cortactin	2017	ENSG00000085733	SAVGHEYQSKLSKHCSQVDSVRGFGGKFGVQ	S	0.999925	113	15.42515	15.30032	15.75282	15.96267	15.80529	15.73296	15.87908	15.2197	15.6529	0.543077645	-0.091129303	-0.340877215	0.249747912
22	B7Z2K3	FAM13C	family with sequence similarity 13 member C	220965	ENSG00000148541	SQSECQVRAGTPAHESPQNNAFKCQETVRLQ	S	0.96892	131	15.42394	15.84882	15.74497	16.00775	16.03858	15.85545	16.04386	16.0109	15.90421	1.167634886	-0.313749949	-0.294685682	-0.019064267
1092	Q9HCS5	EPB41L4A	erythrocyte membrane protein band 4.1 like 4A	64097	ENSG00000129595	ADPNNRRSRHRSRSRSPDIQAKEELWKHIQK	S	0.5	543	15.42101	15.44404	15.13855	16.08574	15.37599	15.3682	15.20804	15.11382	15.21659	0.696022296	0.155048688	-0.27544117	0.430489858
1091	Q9HCS5	EPB41L4A	erythrocyte membrane protein band 4.1 like 4A	64097	ENSG00000129595	NQADPNNRRSRHRSRSRSPDIQAKEELWKHI	S	0.5	541	15.42101	15.44404	15.13855	16.08574	15.37599	15.3682	15.20804	15.11382	15.21659	0.696022296	0.155048688	-0.27544117	0.430489858
558	O94919	ENDOD1	endonuclease domain containing 1	23052	ENSG00000149218	QIQDEERMVQSQKSSSPLSSTRSKRSTLLPP	S	0.898842	303	15.41531	15.37796	15.30603	15.47573	15.68009	15.50283	15.36765	15.4196	15.12291	1.054522596	0.063044866	-0.186451594	0.24949646
881	Q6GQQ9	OTUD7B	OTU deubiquitinase 7B	56957	ENSG00000264522	SPTASAGDEPRSTPESGDSDKESVGSSSTSN	S	0.762183	464	15.40275	15.64639	15.16777	15.59292	15.27525	15.69439	15.10681	14.95005	15.32654	0.815212342	0.277836164	-0.115218163	0.393054326
1309	O15498	YKT6	YKT6 v-SNARE homolog	10652	ENSG00000106636	GEKLDDLVSKSEVLGTQSKAFYKTARKQNSC	T	0.99539	179	15.39687	15.40028	15.10489	15.72744	14.99978	15.45761	14.88613	15.28544	15.35889	0.189907513	0.123860359	-0.094263395	0.218123754
250	E5RJY1	NDRG1	N-myc downstream regulated 1	10397	ENSG00000104419	LDGTRSRSHTSEGTRSRSHTSEGTRSRSHTS	S	0.810836	61	15.39111	15.28044	15.44695	15.63126	14.83294	15.74804	15.30983	15.08551	15.82003	0.003310736	-0.032289187	-0.031243324	-0.001045863
40	A0A0A0MRB5	MADD	MAP kinase activating death domain	8567	ENSG00000110514	NRAQKLLRPNSLRLASDSDAESDSRASSPNS	S	0.994356	818	15.39057	15.22155	15.32955	15.58382	15.26052	15.26129	14.93935	15.08912	15.57943	0.16967689	0.111256599	-0.054649353	0.165905952
1372	Q07866	KLC1	kinesin light chain 1	3831	ENSG00000126214	RNNCLADSRALSASHTDLAH___________	T	0.605042	633	15.38097	15.75929	15.67005	15.66927	15.63509	15.52086	15.39681	15.09461	15.68552	0.385807497	0.211123466	-0.004975319	0.216098785
1398	Q5T4P9	PLEKHO1	pleckstrin homology domain containing O1	51177	ENSG00000023902	SLSRPWEKTDKGATYTPQAPKKLTPTEKGRC	T	0.904511	71	15.37731	15.22042	14.93397	15.22185	14.63651	15.59269	14.82526	15.0756	15.16589	0.082673846	0.154981931	0.0268809	0.128101031
1301	Q8TE68	EPS8L1	EPS8 like 1	54869	ENSG00000131037	ATQEELQRDRSPAAETPPLQRRPSVRAVIST	T	1	187	15.36878	15.61068	15.28887	15.16302	15.17325	15.39429	14.9595	15.31441	15.46356	0.324431674	0.176952362	0.179253578	-0.002301216
610	P04792	HSPB1	heat shock protein family B (small) member 1	3315	ENSG00000106211	VAAPAYSRALSRQLSSGVSEIRHTADRWRVS	S	0.506344	83	15.356	14.76176	15.40517	15.81406	15.35494	15.61755	15.43248	15.08891	15.45359	0.622217464	-0.150681178	-0.421203613	0.270522436
361	H0Y7R3	WNK2	WNK lysine deficient protein kinase 2	65268	ENSG00000165238	VKKATAFLQRPSRAGSLGPETPSRVGMKVPT	S	0.993945	1456	15.35428	14.80469	14.86805	15.21599	14.93263	15.17621	15.24822	14.89055	15.32706	0.123635257	-0.146271706	-0.099269867	-0.047001839
1260	F5H442	TSG101	tumor susceptibility 101	7251	ENSG00000074319	VTTVGPSRDGTISEDTIRASLISAVSDKLRW	T	0.823994	225	15.34218	15.55567	15.13783	15.19768	15.40929	15.56617	15.33863	15.32917	15.11439	0.170229219	0.08449618	-0.045820872	0.130317052
1532	P16104	H2AFX;H2AX	H2A histone family member X;H2A.X variant histone	3014	ENSG00000188486	KAPSGGKKATQASQEY_______________	Y	0.8768	143	15.3363	14.80549	15.14314	15.1333	15.3327	14.47858	17.54751	14.93461	16.26708	0.726548051	-1.154756546	0.113449732	-1.268206278
1134	Q9P2Q2	FRMD4A	FERM domain containing 4A	55691	ENSG00000151474	TRLHSLALHFRHRSSSLESQGKLLGSENDTG	S	0.865129	711	15.3306	15.79365	15.12662	15.7761	15.62665	15.464	15.34869	15.14055	15.47795	0.451614711	0.094562531	-0.205290794	0.299853325
1221	Q13555	CAMK2G	calcium/calmodulin dependent protein kinase II gamma	818	ENSG00000148660	VCQRSTVASMMHRQETVECLRKFNARRKLKG	T	1	287	15.31157	16.08585	15.29985	16.5522	16.05676	15.98112	15.98264	16.36649	16.38399	1.043625515	-0.678617477	-0.630940119	-0.047677358
114	A0A1B0GTW1	TJP2	tight junction protein 2	9414	ENSG00000119139	FGPSRDRDRDRSRGRSIDQDYERAYHRAYDP	S	0.999883	303	15.31139	14.95465	15.29724	15.1021	15.36786	15.55947	15.2139	15.07397	15.02015	0.466630866	0.085083961	-0.155379931	0.240463893
1444	Q96A22	C11orf52	chromosome 11 open reading frame 52	91894	ENSG00000149300	QLQQNLPKGHETTGHTYERVLQQQGSQERSP	T	0.996158	52	15.29487	14.63827	14.94951	15.09811	14.91942	14.93069	14.43886	15.00562	14.77262	0.315531984	0.221846898	-0.021860123	0.243707021
1506	D6RA31	SNCA	synuclein alpha	6622	ENSG00000145335	QGVAEAAGKTKEGVLYVGSKTKEGVVHGVAT	Y	0.999931	39	15.29322	14.90788	15.0086	15.21174	14.96276	14.22844	14.55471	14.83225	15.18832	0.181656687	0.21147569	0.268919945	-0.057444255
181	F5H672	ISCU	iron-sulfur cluster assembly enzyme	23479	ENSG00000136003	SALLLRSPRLPARELSAPARLYHKKVSQICE	S	1	29	15.29056	15.68907	15.31561	15.26583	15.37663	14.9993	14.4241	14.76186	15.21822	1.055728931	0.630350749	0.217824618	0.412526131
719	Q5T6W2	HNRNPK	heterogeneous nuclear ribonucleoprotein K	3190	ENSG00000165119	GGRPMPPSRRDYDDMSPRRGPPPPPPGRGGR	S	1	260	15.28602	15.55752	14.50296	15.25303	15.3899	15.06773	15.18321	14.82655	15.13374	0.095155227	0.0676651	-0.121388753	0.189053853
362	H0Y7R3	WNK2	WNK lysine deficient protein kinase 2	65268	ENSG00000165238	RIWPALQPKEQQDVGSPDKARGPPVPLQVQV	S	1	164	15.28598	15.13787	14.67204	15.70539	15.7364	15.2889	15.52742	15.46579	15.43264	1.196309096	-0.443318685	-0.544934273	0.101615588
1512	F5GYU8	TMC5	transmembrane channel like 5	79838	ENSG00000103534	GPLNNPDYPGTRSNPYSVASRTRPDYPGSLA	Y	0.643935	55	15.27979	15.43362	15.0756	15.42505	15.55396	15.75627	15.0323	15.0736	15.49404	0.892299475	0.063022931	-0.315422058	0.37844499
376	G3V2B0	MPP5	membrane palmitoylated protein 5	64398	ENSG00000072415	DCPGDLGTRMMPIRRSAQLERIRQQQEDMRR	S	1	53	15.27554	15.41154	15.13619	15.43319	15.44976	15.64566	14.82804	15.72393	15.12912	0.326919094	0.04739666	-0.235112508	0.282509168
1383	Q13557	CAMK2D	calcium/calmodulin dependent protein kinase II delta	817	ENSG00000145349	KKPDGVKESTESSNTTIEDEDVKARKQEIIK	T	0.638204	337	15.27514	15.2991	14.98984	15.15335	15.45024	15.3214	15.29699	15.11553	14.95147	0.371624084	0.06669871	-0.120305697	0.187004407
281	E7EX29	YWHAZ	tyrosine 3-monooxygenase/tryptophan 5-monooxygenase activation prote	7534	ENSG00000164924	KNVVGARRSSWRVVSSIEQKTEGAEKKQQMA	S	0.954894	64	15.26386	15.71634	15.18274	15.41002	15.4076	15.63072	15.156	15.06036	15.21006	0.825264185	0.245504061	-0.095135371	0.340639432
556	O94875	SORBS2	sorbin and SH3 domain containing 2	8470	ENSG00000154556	VQSSPNLLAAGRDSQSPDSAWRSYNDGNQET	S	0.669665	40	15.2625	15.36098	14.81378	15.56894	15.13314	15.58014	14.82382	15.29921	15.46659	0.299840316	-0.050781886	-0.281651815	0.230869929
1127	Q9P270	SLAIN2	SLAIN motif family member 2	57606	ENSG00000109171	SPVRAGASIPSSGAASPRGFPLGLSAKSGGG	S	0.998964	63	15.25978	15.35235	15.15153	15.69441	14.88598	14.88836	15.11886	14.69256	15.22291	0.178112368	0.243112246	0.098302841	0.144809405
667	P29966	MARCKS	myristoylated alanine rich protein kinase C substrate	4082	ENSG00000277443	STSSPKAEDGATPSPSNETPKKKKKRFSFKK	S	0.700944	147	15.25772	15.3435	15.2858	15.72758	15.37048	15.0824	15.40238	15.37382	15.5404	0.171336874	-0.143192927	-0.097813288	-0.045379639
1270	G5E9G7	NRXN2	neurexin 2	9379	ENSG00000110076	ERPPALLGSQGLRGATADPLCAPARNPCANG	T	1	201	15.25669	14.28113	14.31026	15.69664	15.73166	14.78591	15.56698	15.20881	14.55513	0.575094773	-0.494277	-0.788713137	0.294436137
262	E7EMZ9	TACC2	transforming acidic coiled-coil containing protein 2	10579	ENSG00000138162	_MGNENSTSDNQRTLSAQTPRSAQPPGNSQN	S	0.976479	15	15.25433	15.2527	14.95588	15.54478	15.13571	15.14776	14.77237	14.72611	15.26605	0.580984268	0.232791901	-0.121782303	0.354574203
1520	I3L4C2	BAIAP2	BAI1 associated protein 2;BAR/IMD domain containing adaptor protein 2	10458	ENSG00000175866	SNTLPVRKSVTPKNSYATTENKTLPRSSSMA	Y	0.999517	386	15.25414	15.69789	15.81718	15.42895	15.41125	15.63501	15.94821	16.20747	15.14645	0.17418051	-0.177637418	0.098003705	-0.275641123
18	E5RHB5	PLEKHA2	pleckstrin homology domain containing A2	59339	ENSG00000169499	EGSEPGSHTILRRSQSYIPTSGCRASTGPPL	S	0.94545	184	15.25108	15.46729	15.31366	15.5329	15.66509	15.38677	16.02226	15.24922	15.44521	0.267160112	-0.228222211	-0.184245427	-0.043976784
449	J3KPH8	HDAC7	histone deacetylase 7	51564	ENSG00000061273	LPSDPPEHFPLRKTVSEPNLKLRYKPKKSLE	S	0.974733	211	15.24808	15.66422	15.64864	15.25915	15.68283	15.68135	15.00031	15.34027	15.24522	0.745945014	0.325048447	-0.020797729	0.345846176
300	E9PMV1	PLEC	plectin	5339	ENSG00000178209	FRAKIERARSDEGQLSPATRGAYRDCLGRLD	S	0.999887	627	15.24046	15.62171	15.28041	15.61079	15.80029	15.5153	15.73116	15.52592	15.57755	0.767052535	-0.23068428	-0.261271477	0.030587196
1236	B1APY4	ROR2	receptor tyrosine kinase like orphan receptor 2	4920	ENSG00000169071	FLVCMCRNKQKASASTPQRRQLMASPSQDME	T	0.852086	298	15.23025	14.78515	15.07682	15.58443	14.9614	15.11906	15.36977	15.04003	15.26825	0.239798041	-0.195281029	-0.190898259	-0.004382769
1526	P04279	SEMG1	semenogelin 1	6406	ENSG00000124233	LNQDQQHGRKANKISYQSSSTEERRLHYGEN	Y	0.86607	288	15.22686	14.96551	15.45882	15.28334	15.54897	15.20243	15.97354	15.57675	15.55929	1.074636893	-0.48612531	-0.127847354	-0.358277957
1536	Q02383	SEMG2	semenogelin 2	6407	ENSG00000124157	HSQDQEHGHKENKISYQSSSTEERHLNCGEK	Y	0.86607	348	15.22686	14.96551	15.45882	15.28334	15.54897	15.20243	15.97354	15.57675	15.55929	1.074636893	-0.48612531	-0.127847354	-0.358277957
1524	K7ELD9	SYNGR2	synaptogyrin 2	9144	ENSG00000108639	__________MESGAYGAAKAGGSFDLRRFL	Y	0.989436	6	15.22464	15.14059	15.27979	15.41769	15.25628	14.67309	15.28475	15.08759	15.08323	0.056322324	0.063151677	0.099322001	-0.036170324
417	I3L3F2	PHKB	phosphorylase kinase regulatory subunit beta	5257	ENSG00000102893	PDAVVSPSSAFLRSGSVYEPLKSINLPRPDN	S	0.972479	20	15.22279	15.73748	14.56707	15.55719	14.99974	14.90139	15.79383	15.31036	15.71481	0.405397131	-0.430553754	0.023009618	-0.453563372
1020	Q9BS40	LXN	latexin	56925	ENSG00000079257	GTPHRVFEVQKVKQASMEDIPGRGHKYHLKF	S	1	41	15.215	15.54152	15.29002	15.61342	15.61342	15.68628	15.06567	15.44624	15.50885	0.88418213	0.008594195	-0.288857142	0.297451337
1296	K7ERC3	ADGRL1	adhesion G protein-coupled receptor L1	22859	ENSG00000072071	LKTSAMRSNTRYYTGTQSRIRRMWNDTVRKQ	T	0.993914	18	15.21018	15.67882	15.55357	15.78284	15.68085	14.97127	16.10341	16.0992	15.13487	0.191874956	-0.298305194	0.002531052	-0.300836245
173	B1AHL2	FBLN1	fibulin 1	2192	ENSG00000077942	AVWKVGRASQAEGTASARAQRRGMVQEQCCH	S	0.956496	93	15.20698	16.06371	16.41449	15.73079	16.014	16.26997	16.20218	15.04717	16.16785	0.04297572	0.089326223	-0.109859149	0.199185371
275	E7EX73	EIF4G1	eukaryotic translation initiation factor 4 gamma 1	1981	ENSG00000114867	SVTAFFKWLREAEEESDHN____________	S	1	1433	15.20469	15.16275	15.05901	14.9643	15.13735	15.18301	14.84422	14.64024	15.25816	0.408011243	0.22793897	0.047259013	0.180679957
1200	E5RFQ9	STK3	serine/threonine kinase 3	6788	ENSG00000104375	LDSHTMVKTSVESVGTMRA____________	T	0.999741	225	15.18936	15.0911	14.71703	15.52604	15.24745	15.38893	14.81638	15.27383	15.30192	0.729786313	-0.131546974	-0.388304075	0.2567571
788	Q13200	PSMD2	proteasome 26S subunit, non-ATPase 2	5708	ENSG00000175166	IYKTHLENNRFGGSGSQVDSARMNLASSFVN	S	0.62433	204	15.17039	15.77288	14.82555	14.96045	14.60218	15.04819	14.93779	14.34402	14.66461	0.735066261	0.607470512	0.386003494	0.221467018
1103	Q9NSK0	KLC4	kinesin light chain 4	89953	ENSG00000137171	QPSAAPLQVSRGLSASTMDLSSSS_______	S	0.916922	534	15.16667	15.53327	15.43195	15.50956	15.12549	15.25366	15.18681	14.96082	15.24474	0.519844502	0.246511141	0.081060092	0.16545105
121	A0A1W2PNX9	KCNMA1	potassium calcium-activated channel subfamily M alpha 1	3778	ENSG00000156113	KCGCKRLEDEQPSTLSPKKKQRNGGMRNSPN	S	0.898717	104	15.1629	15.90539	16.04433	15.66345	15.86307	15.67576	16.24865	16.02687	16.39092	0.86132513	-0.517940839	-0.029888153	-0.488052686
475	M0R1L7	CHMP2A	charged multivesicular body protein 2A	27243	ENSG00000130724	IQAVSLKIQTLKSNNSMAQAMKGVTKAMGTM	S	0.997505	98	15.16145	14.99086	15.0814	15.4409	15.18045	15.21322	14.74015	15.02098	15.39861	0.375777967	0.024654706	-0.200289726	0.224944433
155	A6NKB8	RNPEP	arginyl aminopeptidase	6051	ENSG00000176393	_________MASGEHSPGSGAARRPLHSAQA	S	0.999727	7	15.15434	15.66069	15.31416	15.60166	15.4072	15.31511	15.54828	15.61422	15.20438	0.049957168	-0.079228719	-0.064925512	-0.014303207
673	P35579	MYH9	myosin heavy chain 9	4627	ENSG00000100345	FVVPRRMARKGAGDGSDEEVDGKADGAEAKP	S	1	1365	15.14314	15.57725	15.2547	15.8115	15.52405	15.64231	15.4135	15.16746	15.56923	0.800283875	-0.058364868	-0.334255219	0.27589035
1499	A0A0J9YYI4	SERINC5	serine incorporator 5	256987	ENSG00000164300	LTSTTRSSSDALQGRYAAPELEIARCCFCFS	Y	1	343	15.13751	15.58344	15.93457	15.46652	15.54711	15.36229	15.74769	16.50996	15.15979	0.187322796	-0.253978411	0.093199412	-0.347177823
246	E5RGY9	SNAP91	synaptosome associated protein 91	9892	ENSG00000065609	NTLEGKKPGNKSGAPSPLSKSSPATTVTSPN	S	0.867692	298	15.13643	14.7402	14.24874	15.23017	15.01559	14.98072	14.30649	14.67414	14.62571	0.830453583	0.173013687	-0.367034594	0.540048281
733	P80723	BASP1	brain abundant membrane attached signal protein 1	10409	ENSG00000176788	APSSKETPAATEAPSSTPKAQGPAASAEEPK	S	0.759218	195	15.13322	15.42075	15.32436	15.53603	15.33619	14.90905	14.98642	15.04401	15.27234	0.254251716	0.191853523	0.03235054	0.159502983
1029	Q9BV36	MLPH	melanophilin	79083	ENSG00000115648	QGHSLHLSSVPEARDSPQSLTDESCSEKAAP	S	0.586184	223	15.13182	15.2106	14.9527	15.17407	15.02976	14.61597	14.60322	14.8538	14.94668	0.52723393	0.29713726	0.158434868	0.138702393
489	O14545	TRAFD1	TRAF-type zinc finger domain containing 1	10906	ENSG00000135148	VTEGIPRLDSQPQETSPELPRRRVRHQGDLS	S	0.818318	415	15.1317	14.89501	14.52472	15.23751	14.80685	15.22178	14.76741	14.38242	14.47034	1.009889445	0.310419083	-0.238236109	0.548655192
895	Q6ZTQ3	RASSF6	Ras association domain family member 6	166824	ENSG00000169435	HAKDEPDSPVLYRTMSEAALVRKRMKPLMMD	S	0.991842	155	15.12566	14.97804	15.17804	15.50081	15.5201	15.14183	15.32073	14.99841	15.55465	0.529307448	-0.197351774	-0.293665886	0.096314112

507	O43182	ARHGAP6	Rho GTPase activating protein 6		395	ENSG00000047648	VPGQFMLVGHLSSSKSRESSPGPRLGKDLSE	S	0.612832	511	15.12178	15.08788	14.74609	15.14545	14.88665	15.62242	14.62474	15.08514	15.02202	0.357156893	0.074616114	-0.232926369	0.307542483
1522	J3QRU1	YES1	YES proto-oncogene 1, Src family tyrosine kinase		7525	ENSG00000176105	RGIFLVRESETTKGAYSLSIRDWDEIRGDNV	Y	0.689607	199	15.11325	15.15014	15.21504	15.90623	15.59006	15.0435	15.31784	15.31844	15.6193	0.4844897	-0.259046237	-0.353784243	0.094738007
1376	Q12929	EPS8	epidermal growth factor receptor pathway substrate 8		2059	ENSG00000151491	SIPPPPRAPAPAPPGTVTQVDVRSRVAAWSA	T	0.961023	223	15.10099	15.23777	14.81943	15.41835	15.60583	15.27692	15.87246	15.60652	15.17376	0.924219386	-0.498181025	-0.380968412	-0.117212613
75	H7C3Q7	ABI2	abl interactor 2		10152	ENSG00000138443	DYVPSPTRNMAPSQQSPVRTASVNQRNRTYS	S	0.999829	93	15.09791	15.77854	15.18534	15.61494	15.55495	15.5051	15.32931	15.2048	15.29624	0.475097069	0.077149073	-0.204399427	0.2815485
995	Q96C24	SYTL4	synaptotagmin like 4		94121	ENSG00000102362	EAKNLTAAKAGGTSDSFVKGYLLPMRNKASK	S	0.918729	549	15.09433	15.13378	15.28951	15.24589	14.96817	15.01877	15.03222	15.06015	14.83076	0.621678226	0.198165258	0.094933192	0.103232066
1166	Q9Y3R5	DOP1B	DOP1 leucine zipper like protein B		9980	ENSG00000142197	KSEEPAGKRDRDGTQSLAANDSSRKNSWEPK	S	0.996521	670	15.08986	15.62579	15.68055	15.55354	15.4263	15.18832	15.26165	15.28323	15.48693	0.090913353	0.121464729	0.076018333	0.045446396
287	E9PAM4	AL355315.1	novel protein	NA		ENSG00000249967	PPVIVETARSHQRSSSESYTQSFQSRKPFFS	S	0.947885	432	15.08502	15.1104	14.68486	14.95215	14.91153	14.82272	14.83871	14.83763	14.91294	0.142680052	0.097000122	0.064624786	0.032375336
841	Q5JSH3	WDR44	WD repeat domain 44		54521	ENSG00000131725	TFKETENTAYKVGNESPVQELKQDVSKKIIE	S	1	50	15.08477	14.88531	14.82312	14.95037	15.10783	15.33221	14.71081	14.23878	15.07033	0.680547654	0.257761637	-0.199071566	0.456833204
966	Q8TC07	TBC1D15	TBC1 domain family member 15		64786	ENSG00000121749	SNALPTLSASGARNDSPTQIPVSSDVCRLTP	S	0.800787	658	15.08069	15.44999	15.19595	15.17948	14.62342	15.06179	15.81117	15.31313	15.35328	0.964124656	-0.25032107	0.287309329	-0.537630399
1265	F8VWT9	HECTD4	HECT domain E3 ubiquitin protein ligase 4		283450	ENSG00000173064	SPGDVAGIGWERTEGTPPPPGQPAKGRVYFT	T	0.849028	2368	15.08015	15.08982	15.088	15.17796	15.23021	14.94777	15.2991	15.004	15.42764	0.33921685	-0.157588641	-0.032656988	-0.124931653
700	P51159	RAB27A	RAB27A, member RAS oncogene family		5873	ENSG00000069974	TVGIDFREKRVVYRASGPDGATGRGQRIHLQ	S	0.999913	56	15.07565	14.90633	15.25978	15.37419	14.98922	15.02029	15.15616	15.02133	15.11146	0.027481677	-0.015732447	-0.047313054	0.031580607
622	P08575	PTPRC	protein tyrosine phosphatase, receptor type C;protein tyrosine phosphata		5788	ENSG00000081237	QHIGNQEENKSKNRNSNVIPYDYNRVPLKHE	S	0.999737	814	15.07556	15.48114	15.44262	15.31995	15.53904	15.33123	16.5756	15.35552	16.09426	0.864549544	-0.675354958	-0.063634872	-0.611720085
21	H0YKB1	TJP1	tight junction protein 1		7082	ENSG00000277401	DHSGRSHDRPPRRSRSRSPDQRSEPSDHSRH	S	0.699812	313	15.07548	15.12888	15.40723	15.04593	14.8257	15.25919	15.00799	14.93976	15.24633	0.256326669	0.139169375	0.160256068	-0.021086693
962	Q8NFW9	MYRIP	myosin VIIA and Rab interacting protein		25924	ENSG00000170011	PQSCSTKVADEGTSASPGGYRAPAALWRSQS	S	0.987462	280	15.07259	14.63979	15.31306	15.91716	15.24908	15.13571	14.97715	14.84931	15.28746	0.519776476	-0.029489517	-0.425501188	0.39601167
1413	Q7L1Q6	BZW1	basic leucine zipper and W2 domains 1		9689	ENSG00000082153	EAGLKELSEYVRNQQTIGARKELQKELQEQM	T	1	250	15.06777	15.14836	14.85107	15.39356	14.92119	14.86877	14.62799	14.34679	15.26858	0.306317983	0.274611473	-0.038776716	0.313388189
1045	Q9BYG4	PARD6G	par-6 family cell polarity regulator gamma		84552	ENSG00000178184	IQKREEAERGSLGAGSLCRRRRALGALRDEG	S	0.999986	110	15.06474	15.6027	15.18352	15.49876	15.39232	15.64532	14.96235	15.26569	14.83408	1.05657174	0.262948036	-0.228477478	0.491425514
12	A0A087WW74	SASH1	SAM and SH3 domain containing 1		23328	ENSG00000111961	SCDPPGVTGLNKNRRSLPVSICRSCETLEGP	S	0.999983	223	15.06006	15.86935	15.84073	15.78882	15.35587	15.43414	14.79847	15.14283	15.40919	0.573359295	0.4732186	0.063772519	0.409446081
1462	Q9H246	C1orf21	chromosome 1 open reading frame 21		81563	ENSG00000116667	KIEKGRDYCSEEEDIT_______________	T	0.986686	121	15.05765	15.3832	14.60629	15.21576	15.20434	14.46875	14.52381	15.07079	14.81743	0.110821987	0.211702983	0.052762667	0.158940315
887	X6RKU3	FBXO42	F-box protein 42		54455	ENSG00000037637	GYDLKIGLSLAPRRGSLPDQKDLRLGSIDLN	S	1	206	15.05723	15.69602	15.14175	15.62251	15.44511	15.33309	15.0593	15.17243	15.40594	0.332227246	0.085775057	-0.168570836	0.254345894
118	A0A1B0GTW1	TJP2	tight junction protein 2		9414	ENSG00000119139	GRSFRSGYSERSRLNSHGGRSRSWEDSPERG	S	0.998486	222	15.05689	14.89888	15.00856	15.0289	14.79396	15.08788	14.94297	15.25133	14.91153	0.065208194	-0.04716746	0.017867088	-0.065034548
1155	Q9UPX8	SHANK2	SH3 and multiple ankyrin repeat domains 2		22941	ENSG00000162105	QQMPSKPEGAAKTIGSYVPGPRSRSPSLNRL	S	0.875738	466	15.05125	15.8919	14.76586	15.0308	15.39614	15.61249	14.5235	15.29677	15.05308	0.243679334	0.278552691	-0.110139211	0.388691902
27	C9IYE1	CADPS2	calcium dependent secretion activator 2		93664	ENSG00000081803	PGRAGGGGAARSVSPSPSVLSEGRDEPQRQL	S	0.597498	60	15.05002	15.5651	15.04844	15.52736	15.67077	15.43107	15.02626	14.79847	15.31618	0.975331821	0.17421786	-0.321876844	0.496094704
1232	Q5TIG5	AFDN	afadin, adherens junction formation factor		4301	ENSG00000130396	MHTDSNHSSIAIQRVTRSQEELREDKAYQLE	T	0.920538	1273	15.0484	15.14267	15.12948	15.14386	14.92967	15.39017	15.15379	15.21947	14.85341	0.064359206	0.031294823	-0.04771773	0.079012553
1181	Q9Y4P8	WIPI2	WD repeat domain, phosphoinositide interacting 2		26100	ENSG00000157954	QTYGAAAGKGTYVPSSPTRLAYTDDLGAVGG	S	0.830557	395	15.04204	15.25352	15.07761	14.97136	14.96036	15.24867	14.84882	14.88355	14.78095	1.248305463	0.286616007	0.064256668	0.222359339
1277	H0YFE9	C2CD5	C2 calcium dependent domain containing 5		9847	ENSG00000111731	AGKEGGPFKALLRQQTQSALEQRMNQKLEMH	T	0.961467	142	15.04106	15.20098	14.91373	15.37293	15.66719	15.23545	15.53187	15.32952	15.00004	0.733287566	-0.235220909	-0.373271624	0.138050715
638	P13807	GYS1	glycogen synthase 1		2997	ENSG00000104812	VPPSPSLSRHSSPHQSEDEEDPRNGPLEEDG	S	0.983422	593	15.03896	14.82307	14.69577	15.17469	15.20835	15.26048	14.89093	15.20839	14.95183	1.260936888	-0.164448102	-0.361905416	0.197457314
606	P04279	SEMG1	semenogelin 1		6406	ENSG00000124233	HTYHVDINDHDWTRKSQQYDLNALHKATKSK;YT	S	0.999587	82	15.0323	14.15948	14.37402	14.62096	15.17079	14.61649	14.38229	14.86767	14.64954	0.196986773	-0.111228943	-0.280813217	0.169584274
1147	Q9UKE5	TNIK	TRAF2 and NCK interacting kinase		23043	ENSG00000154310	EEDIPPKVPQRTTSISPALARKNSPGNGSAL	S	0.984408	596	15.02816	15.7876	15.41887	15.45337	15.59278	15.48523	15.57282	15.94805	15.25185	0.111507369	-0.179361025	-0.098913829	-0.080447197
912	Q86SU0	ILDR1	immunoglobulin like domain containing receptor 1		286676	ENSG00000145103	RGPKSWALERRELDPSWSGRHRSSRLNGSPI	S	0.998588	309	15.0257	14.95124	15.05558	15.11162	15.13979	15.39302	14.21576	15.00282	15.02911	0.675451384	0.261610031	-0.203972499	0.46558253
161	H7BZ52	ZSWIM8	zinc finger SWIM-type containing 8		23053	ENSG00000214655	CQNWRGWRKQSAGPNSPTGGGGGGGSGGTR	S	0.908827	53	15.02375	14.47984	14.34006	15.00852	14.74405	14.41858	14.52858	14.51767	14.95092	0.041159027	-0.051170031	-0.109161059	0.057991028
986	X6RAB3	USP6NL	USP6 N-terminal like		9712	ENSG00000148429	SGAPHRRHEHSPHPQSRTGTPERAQPPRRKS	S	1	443	15.009	15.02955	14.47409	15.40255	14.81613	15.2263	14.92486	14.75076	15.05206	0.413684655	-0.071682294	-0.310783068	0.239100774
323	F5GYU8	TMC5	transmembrane channel like 5		79838	ENSG00000103534	DYPGSLAEPNYPRSLSNPDYSGTRSNAYSAA	S	0.995997	79	15.00519	14.63753	14.70784	14.78023	15.12517	15.13487	14.99572	15.29354	15.09197	0.836991787	-0.343559265	-0.229903539	-0.113655726
175	B1ALM5	TMEM9	transmembrane protein 9		252839	ENSG00000116857	YTEQLHNEEENEDARSMAAAAASLGGPRANT	S	0.931766	140	15.00492	15.23392	15.18994	14.75332	14.92402	14.8261	15.17707	15.51871	15.01759	1.205190533	-0.094864209	0.308445295	-0.403309504
1262	H0Y3C5	HCK	HCK proto-oncogene, Src family tyrosine kinase		3055	ENSG00000101336	IADFGLARVIEDNEYTAREGAKFPIKWTAPE	T	1	411	15.00444	15.74341	15.98366	15.85462	15.89186	15.48073	15.39322	16.4917	15.77977	0.143810986	-0.311058044	-0.165230115	-0.145827929
686	P46059	SLC15A1	solute carrier family 15 member 1		6564	ENSG00000088386	NRLEKSNPYFMSGANSQKQM___________	S	0.997923	704	15.00268	15.58816	15.25477	14.86733	14.9191	14.97073	14.72222	15.11963	14.93498	0.907302766	0.356260618	0.3628184	-0.006557782
959	Q8NE71	ABCF1	ATP binding cassette subfamily F member 1		23	ENSG00000204574	PKPAKPEKNRINKAVSEEQQPALKGKKGKEE	S	1	166	14.99396	15.02393	15.44401	15.22219	15.30264	15.19299	15.24195	15.15042	15.26488	0.106197471	-0.065118472	-0.085307121	0.020188649
633	P12036	NEFH	neurofilament heavy		4744	ENSG00000100285	HSWTRTSVSSVSASPSRFRGAGAASSTDSLD	S	0.616994	63	14.99024	15.11695	15.05057	15.04183	15.2696	15.14446	14.14195	14.6451	14.98566	1.120754137	0.461684545	-0.099376361	0.561060905
356	F8W8V9	KIF1A	kinesin family member 1A		547	ENSG00000130294	KLPASRSIRNLFGSGSLRASESNRVTGVYEL	S	0.931173	1421	14.98602	15.24611	14.71677	15.56772	15.18735	15.03982	14.72334	15.33476	15.16812	0.294829873	-0.092443784	-0.281997999	0.189554214
641	P15924	DSP	desmoplakin		1832	ENSG00000096696	PHTGLLLLSVQKRSMSFQGIRQPVTVTELVD	S	0.981461	1610	14.98375	15.11272	14.65676	14.9788	14.8262	14.75029	14.74746	14.67419	15.18755	0.030809794	0.048012733	0.065982183	-0.017969449
1184	Q9Y5K6	CD2AP	CD2 associated protein		23607	ENSG00000198087	PEKPLILQSLGPKTQSVEITKTDTEGKIKAK	S	0.980216	256	14.98054	15.39212	15.20903	15.20713	15.3319	15.14203	14.71548	15.23358	15.16628	0.271616206	0.155449867	-0.033119837	0.188569705
387	H0Y873	AGAP3	ArfGAP with GTPase domain, ankyrin repeat and PH domain 3		116988	ENSG00000133612	PAEVLSSSPKLDPPPSPHSNRKKHRRKKSTG	S	0.897301	67	14.97938	14.76674	14.65536	15.01989	14.79918	14.89197	14.42436	14.50034	14.95292	0.51843992	0.174616496	-0.103189468	0.277805964
1118	Q9P206	KIAA1522	KIAA1522		57648	ENSG00000162522	GTPTLPPKGLAGPPASPGKAQPPKPERVTSL	S	1	556	14.97732	15.42072	14.5329	15.23833	15.40115	15.02228	14.96244	14.80766	15.13499	0.276702776	0.008618673	-0.243603706	0.252222379
1550	Q9H1C7	CYSTM1	cysteine rich transmembrane module containing 1		84418	ENSG00000120306	YGWQGGPQEPPKTTVYVVEDQRRDELGPSTC	Y	0.999994	64	14.97311	15.61445	15.31628	15.60869	15.56917	15.59724	15.68817	15.69851	15.69392	1.02819908	-0.392252922	-0.290417353	-0.101835569
965	Q8TAB5	C1orf216	chromosome 1 open reading frame 216		127703	ENSG00000142686	NGYASSSLSIDSRSSSPEPACGTPRGPGPPD	S	0.809211	126	14.95837	15.32062	15.47336	15.71741	15.28912	14.86196	14.36701	14.55968	15.23429	0.659665519	0.530459086	-0.038711548	0.569170634
800	Q14195	DPYSL3	dihydropyrimidinase like 3		1809	ENSG00000113657	RRGQGREESREPAPASPAPAGVEIRSATGKE	S	0.999915	102	14.9557	14.35066	14.4901	15.0507	15.01332	14.58649	14.81278	14.73042	14.45648	0.370543281	-0.067740758	-0.284684181	0.216943423
1391	Q16825	AL162171.1;PTPN21	protein tyrosine phosphatase non-receptor type 21;protein tyrosine phos		11099	ENSG00000070778	MPLPFGLKLKRTRRYTVSSKSCLVARIQLLN	T	0.958494	16	14.95424	15.1267	14.75682	15.63311	15.43746	15.14506	15.45137	15.24789	15.72393	1.260989613	-0.528475761	-0.459287643	-0.069188118
984	Q92625	ANKS1A	ankyrin repeat and sterile alpha motif domain containing 1A		23294	ENSG00000064999	TCSPTEDATMGSRSESLSNCSIGKKRLEKSP	S	0.882714	647	14.9405	14.90928	15.1773	15.34048	15.10345	15.42124	14.52326	14.21743	15.20121	0.919559088	0.361731847	-0.279362361	0.641094208
1257	Q9Y485	DMXL1	Dmx like 1		1657	ENSG00000172869	SSGLHPPKKTLTRSMTSLAQKICGKKTAFDP	T	0.964375	1287	14.9354	15.25724	14.8933	15.27812	15.25739	14.88812	15.63572	15.10161	15.48991	0.690297356	-0.380432765	-0.112565994	-0.26786677
431	I3L1D5	ALOX15B	arachidonate 15-lipoxygenase type B		247	ENSG00000179593	HFTEEAPRRSIATFQSRLAQISRGIQERNQG	S	0.964375	630	14.92963	15.65919	15.36509	15.68552	14.91714	15.36584	15.40414	15.25842	15.94201	0.137501963	-0.216888428	-0.004863421	-0.212025007
976	Q8TED4	SLC37A2	solute carrier family 37 member 2		219855	ENSG00000134955	KEILAWKVSLSRGSGSSMVLTHQ________	S	0.8498	498	14.91639	15.47959	15.34942	15.75898	15.59278	15.27223	15.48076	15.59243	15.42134	0.512223864	-0.249708811	-0.292860985	0.043152173
64	A0A0A6YYH1	ARPIN-AP3S2;C15orf38-	ARPIN-AP3S2 readthrough;C15orf38-AP3S2 readthrough		100526783	ENSG00000250021	______________MSRIYHDGALRNKAVQS	S	0.987228	2	14.91036	15.39818	15.02137	15.10714	15.12791	15.23818	14.92245	15.09618	15.1872	0.085103456	0.041361491	-0.047774315	0.089135806
1338	R4GN18	CD46	CD46 molecule		4179	ENSG00000117335	RRKKKGKADGGAEYATYQTKSTTPAEQRG__	T	0.901301	65	14.90487	15.57752	15.0518	15.38472	15.39594	15.53013	15.21818	15.4847	15.45924	0.409770882	-0.209310214	-0.258864403	0.049554189
1303	K7EP32	UBXN6	UBX domain protein 6		80700	ENSG00000167671	LEQKQSRAWGPTSQDTIRNQVRKELQAEATV	T	0.999898	51	14.88841	15.57403	14.69485	15.17415	15.22351	15.48969	14.40195	15.32365	14.95737	0.30948741	0.158105214	-0.24335289	0.401458104
980	Q8WUM4	PDCD6IP	programmed cell death 6 interacting protein		10015	ENSG00000170248	FTWKDAFDKGSLFGGSVKLALASLGYEKSCV	S	0.999987	108	14.8768	15.12348	14.44637	14.93733	14.88331	14.95579	14.85268	15.19833	14.42862	0.052489821	-0.010991732	-0.109923999	0.098932266
336	F5H442	TSG101	tumor susceptibility 101		7251	ENSG00000074319	__________MAVSESQLKKMVSKYKYRDLT	S	0.977608	6	14.87012	15.56528	14.10009	14.75316	14.88536	15.23811	14.2048	15.11354	15.03553	0.035351773	0.060541153	-0.113713582	0.174254735
1273	H0Y704	ZNF185	zinc finger protein 185 with LIM domain		7739	ENSG00000147394	QGDPAVPAQQPADPSTPERQSSPSGSEQLVR	T	0.765341	206	14.86988	14.47965	14.15252	15.22784	14.93217	14.9684	15.01555	14.64824	13.89539	0.604362652	-0.019039154	-0.542116165	0.523077011
1006	Q96JZ2	HSH2D	hematopoietic SH2 domain containing		84941	ENSG00000196684	PKDRKVPTRKAERSVSCIEVTPGDRSWHQMV	S	0.843577	241	14.86201	15.49047	14.01898	14.98067	14.79472	15.15331	14.95229	14.93875	15.02842	0.074134838	-0.182665507	-0.1857481	0.003082593
282	E7EX40	RAB11FIP1	RAB11 family interacting protein 1		80223	ENSG00000156675	VYLEQPEAKGEIKDSSPSSSPSPKGFRKKHL	S	0.891329	191	14.86147	14.94805	14.27845	15.01502	15.41128	15.46135	14.82918	14.93475	14.91942	1.15789406	-0.198459625	-0.599894206	0.40143458
1069	Q9H0B6	KLC2	kinesin light chain 2		64837	ENSG00000174996	PTQPGGTGLSDSRTLSSSSMDLSRRSSLVG_	S	0.975154	608	14.85511	14.79624	14.50767	15.03325	14.78581	14.21417	13.4298	13.80896	14.6381	0.853405546	0.760721842	0.041932106	0.718789736
383	H0Y5N2	SMPD2	sphingomyelin phosphodiesterase 2		6610	ENSG00000135587	RIDYVLYKAVSGFYISCKSFETTTGFDPHRG	S	0.892015	148	14.85438	15.51222	15.29484	15.3349	15.38627	14.86254	15.47558	15.6685	15.06015	0.159597952	-0.180928866	0.025908152	-0.206837018
791	Q13443	ADAM9	ADAM metallopeptidase domain 9		8754	ENSG00000168615	ESDGKNQANPSRQPGSVPRHVSPVTPPREVP	S	0.999164	752	14.85428	15.23085	15.05918	15.30457	15.17754	15.47659	14.87589	15.09564	14.86443	1.190917073	0.102780342	-0.271461169	0.374241511
989	X6RAB3	USP6NL	USP6 N-terminal like		9712	ENSG00000148429	KFTFKVQPPSHARYPSQLDGEARGLAHPPSY	S	0.989998	640	14.84848	14.82307	14.76415	15.14923	14.04106	15.11256	15.1766	15.00325	14.64515	0.066114942	-0.129772186	0.044279416	-0.174051603
447	J3KPH8	HDAC7	histone deacetylase 7		51564	ENSG00000061273	PLAQGGHRPLSRAQSSPAAPASLSAPEPASQ	S	0.951212	543	14.84618	14.55878	14.67535	14.73328	15.08518	14.87537	14.73983	14.70908	14.30457	0.678200145	0.108941396	-0.204507192	0.313448588
1374	Q12929	EPS8	epidermal growth factor receptor pathway substrate 8		2059	ENSG00000151491	VRPPESGLGRADPPYTHTIQKQRMEYGPRPA	T	0.585421	603	14.83763	15.51585	14.83989	14.89354	14.93194	15.32471	14.80952	15.73894	14.92477	0.028326352	-0.093281746	0.014396667	-0.107678413
1183	Q9Y5K6	CD2AP	CD2 associated protein		23607	ENSG00000198087	DDSLPIKRERHGNVASLVQRISTYGLPAGGI	S	1	80	14.82665	14.37511	14.63175	15.22215	14.82282	14.88803	14.55441	14.65161	15.01428	0.664249679	-0.128934224	-0.366500854	0.23756663
913	Q86SU0	ILDR1	immunoglobulin like domain containing receptor 1		286676	ENSG00000145103	SHSPHWPEEKPPSYRSLDITPGKNSRKKGSV	S	0.994136	425	14.81438	15.74617	15.07823	15.56864	15.14414	14.99126	14.73513	15.03286	15.10255	0.237459526	0.256081263	-0.021748225	0.277829488
1485	Q9Y2Q0	ATP8A1	ATPase phospholipid transporting 8A1		10396	ENSG00000124406	SRAEGYEKTDDVSEKTSLADQEEVRTIFINQ	T	0.5	28	14.79436	15.02297	14.18797	15.16408	15.10954	14.95169	14.64566	14.42986	15.0083	0.568999834	-0.026169777	-0.406670252	0.380500476
885	Q6IWH7	ANO7	anoctamin 7		50636	ENSG00000146205	IDVSPPEAEKRGSYGSTAHASEPGGQQAAAC	S	0.526309	84	14.79391	14.76134	14.3592	15.37887	14.71215	15.03845	14.81042	14.38761	14.65262	0.768363539	0.021265348	-0.405005773	0.426271121
559	O95049	TJP3	tight junction protein 3		27134	ENSG00000105289	SPRLRRESSVDSRTISEPDEQRSELPRESSY	S	0.805497	310	14.78989	15.24433	14.79487	15.27124	15.40624	15.39393	15.05668	15.25026	15.3082	1.165561398	-0.262016296	-0.414106687	0.152090391
342	F5H6E2	MYO1C	myosin IC		4641	ENSG00000197879	GKINRSLASKDVESPSWRSTTVLGLLDIYGF	S	0.926305	386	14.78336	15.22208	14.43567	14.87229	14.60733	15.50084	14.77314	14.859	14.70028	0.133458707	0.036229134	-0.179785728	0.216014862
593	P04279	SEMG1	semenogelin 1		6406	ENSG00000124233	GENGVQKDVSQSSIYSQTEEKAQGKSQKQIT	S	0.673272	316	14.77674	14.47294	13.65296	14.66439	14.93549	14.14776	14.39198	14.32657	14.25422	0.17729436	-0.023375511	-0.28166612	0.258290609
1107	Q9NVD7	PARVA	parvin alpha		55742	ENSG00000197702	________MATSPQKSPSVPKSPTPKSPPSR	S	0.944942	8	14.77324	15.09964	14.6615	15.10959	15.17496	15.10693	14.56891	14.82476	15.07004	0.74079738	0.023558299	-0.285701434	0.309259733
237	F8W6W8	RIMS2	regulating synaptic membrane exocytosis 2		9699	ENSG00000176406	KMVAIVGLSRKSRSASQLSQTEAGGKKLRST	S	0.612167	1113	14.77061	14.83333	14.41587	14.8961	15.11187	14.96326	14.36325	14.30335	14.80816	1.127692383	0.181687991	-0.317133904	0.498821894
1026	Q9BV36	MLPH	melanophilin		79083	ENSG00000115648	SDEESIRAHVMASHHSKRRGRASSESQGLGA;SDI	S	0.607831	329	14.76999	14.68256	14.63345	15.29336	14.43039	14.77957	14.41343	14.36489	14.72339	0.40557195	0.194762548	-0.139106115	0.333868663
153	A6NIT2	PHKA1	phosphorylase kinase regulatory subunit alpha 1		5255	ENSG00000067177	QVPSVRVEIHLPRDQSGEVDFKALVLQLKET	S	1	699	14.76829	15.06848	14.88393	14.82878	14.51742	14.91821	14.52961	14.74284	14.93816	0.280625841	0.170033137	0.152096113	0.017937024
501	O15127	SCAMP2	secretory carrier membrane protein 2		10066	ENSG00000140497	SQGIFSSRTFHRAASSAAQGAFQGN______	S	0.870917	320	14.75796	14.57949	14.08157	14.80115	14.22543	14.60947	14.12064	14.75812	14.61085	0.016515377	-0.023530324	-0.072343508	0.048813184
688	P48307	TFPI2	tissue factor pathway inhibitor 2		7980	ENSG00000105825	KIPSFCYSPKDEGLCSANVTRYYFNPRYRTC	S	0.999188	157	14.75353	14.9438	14.45024	15.3471	15.22536	14.50971	15.08962	15.63911	15.25669	0.775591652	-0.612615267	-0.31153361	-0.301081657
1049	Q9BZQ8	NIBAN1;FAM129A	niban apoptosis regulator 1;family with sequence similarity 129 member		116496	ENSG00000135842	EAAIKNFSPYYSRQYSVAFCNHVRTEVEQQR	S	0.99658	37	14.75118	14.05299	14.5136	15.20949	14.95056	14.79538	14.91639	14.95119	14.3893	0.792388478	-0.313035647	-0.54588445	0.232848803
464	K7EJC2	GMIP	GEM interacting protein		51291	ENSG00000089639	DVDSVGGGSESRSLDSPTSSPGAGTRQLVKA	S	0.642468	181	14.74956	14.69109	14.46167	14.62987	14.7483	14.80992	14.66395	14.5907	14.63776	0.327360192	0.003302574	-0.09525617	0.098558744
74	A0A0C4DFY5	GPRC5C	G protein-coupled receptor class C group 5 member C		55890	ENSG00000170412	VIPEVSQVTKSSPEQSYQGDMYPTRGVGYET	S	0.999962	356	14.74693	15.18662	15.01489	15.5397	14.81658	15.31359	14.38755	15.0493	14.16341	0.852638301	0.449395816	-0.240478834	0.689874649
1446	Q96B36	AKT1S1	AKT1 substrate 1		84335	ENSG00000204673	EDTQVFGDLPRPRLNTSDFQKLKRKY_____	T	0.969798	116	14.74405	15.14494	15.35146	15.23028	15.2243	15.47075	14.99872	14.5977	14.84926	1.009380672	0.264918009	-0.22830073	0.49321874
1488	Q9Y570	PPME1	protein phosphatase methylesterase 1		51400	ENSG00000214517	ARVSMVGQVKQCEGITSPEGSKSIVEGIIEE	T	0.608867	55	14.7431	14.66617	14.49985	14.96045	14.18758	14.53236	14.83999	14.40261	14.35941	0.046020937	0.102367719	0.076239268	0.026128451
1051	Q9C0C2	TNKS1BP1	tankyrase 1 binding protein 1		85456	ENSG00000149115	HLFQDSTEPRASRVPSSDEEVVEEPQSRRTR	S	0.769177	1620	14.74263	14.44379	14.65139	14.6483	14.87296	14.65615	14.68245	14.30699	14.27692	0.75015883	0.190480232	-0.113203367	0.303683599
1056	Q9C0H2	TTYH3	tweety family member 3		80727	ENSG00000136295	SMRAKYLATSQPRPDSSGSH___________	S	0.721272	348	14.74068	14.45353	14.30385	15.27219	14.74662	14.48438	15.27405	15.32175	14.74599	0.842743468	-0.614578565	-0.335045815	-0.27953275
258	E9PGG1	DYNC1I2	dynein cytoplasmic 1 intermediate chain 2		1781	ENSG00000077380	MSPSSKSVSTPSEAGSQDSGDGAVGSRTLHW	S	0.983042	95	14.71011	14.44579	14.73941	14.79994	14.29275	14.61241	14.69479	15.04764	14.29326	0.050033475	-0.046791395	0.06340313	-0.110194524
1015	Q96S99	PLEKHF1	pleckstrin homology and FYVE domain containing 1		79156	ENSG00000166289	AAQQRQEEAEEQGAGSPGQPAHLARPICGAS	S	1	227	14.71	15.25636	14.68491	15.0824	15.18859	15.04648	15.10619	15.17559	14.65905	0.23482701	-0.09652106	-0.222068151	0.125547091

20	F5GZI3	PARD3	par-3 family cell polarity regulator	56288	ENSG00000148498	HRPRPRIIRGRGCNESFRAAIDKSYDKPAVD	S	1	637	14.70757	15.07129	14.35838	14.35535	14.2799	14.51459	14.55537	14.24563	14.82962	0.398995364	0.168875694	0.329134305	-0.160258611
409	H3BUW6	ZFYVE19	zinc finger FYVE-type containing 19	84936	ENSG00000166140	ARLAALKDERQGSIPSTQEMEARLAALQGRV	S	0.522884	41	14.69659	14.92542	14.63844	14.56254	15.1745	14.8734	14.63385	15.04068	14.57501	0.097415945	0.003636678	-0.116664886	0.120301565
642	P16104	H2AFX;H2AX	H2A histone family member X;H2A.X variant histone	3014	ENSG00000188486	VGPKAPSGGKKATQASQEY____________	S	0.97698	140	14.69604	14.74299	14.95751	14.29088	14.11334	14.15782	16.55246	14.49116	16.67893	1.185747791	-1.108668963	0.611502647	-1.720171611
528	O60343	TBC1D4	TBC1 domain family member 4	9882	ENSG00000136111	SRGANRMRGRLGSVDSFERSNSLASEKDYSP	S	0.685836	591	14.69174	15.40674	14.83812	15.42633	14.87695	15.10456	14.98744	14.9557	15.11793	0.109719184	-0.041488012	-0.157078743	0.115590731
1397	Q53EL6	PDCD4	programmed cell death 4	27250	ENSG00000150593	SDSGSDALRSGLTVPTSPKGRLLDRRSRSGK	T	0.968165	93	14.68929	14.06911	14.48111	14.72601	14.83694	15.01193	14.02998	14.24889	14.5789	1.128181012	0.127245267	-0.445120494	0.572365761
221	C9JP16	CRTAP	cartilage associated protein	10491	ENSG00000170275	KRCKQGLPAFRQSQPSREVLADFQRREPYKF	S	0.962096	135	14.68573	14.46359	13.60026	14.7345	14.88651	14.47713	14.95633	14.44178	13.67794	0.248433283	-0.108821233	-0.449515343	0.34069411
413	H3BTK3	CARHSP1	calcium regulated heat stable protein 1	23589	ENSG00000153048	NVVPSPLPTRRTRTFSATVRASQGPVYKGVC	S	0.923984	52	14.67027	14.40368	14.90961	15.09861	15.01162	14.98384	13.75854	14.5695	14.85219	0.758226915	0.267778397	-0.370169957	0.637948354
873	Q9H4G4	GLIPR2	GLI pathogenesis related 2	152007	ENSG00000122694	PLKLCKNLNREAQQYSEALASTRILKHSPES	S	0.972371	43	14.64875	13.42443	13.48872	14.74241	13.42154	14.12832	13.79614	13.43788	13.84941	0.157443495	0.159490267	-0.243457158	0.402947426
1471	Q9P206	KIAA1522	KIAA1522	57648	ENSG00000162522	QEKQKLNKGGWDHGDTQSIQSSRTGPDEDNI	T	0.596925	102	14.64571	15.12989	14.76062	15.3805	15.39583	14.73814	14.73249	14.94928	14.78545	0.550134155	0.022999763	-0.326085726	0.34908549
1	H0Y710	BAG6	BCL2 associated athanogene 6	7917	ENSG00000096155	QGAERASPEPQRENASPAPGTTAEEAMSRGP	S	0.997107	116	14.6429	15.36601	14.70617	15.94976	15.45632	15.26235	15.19264	15.44963	14.73143	0.75621576	-0.219537735	-0.651116371	0.431578636
735	Q00532	CDKL1	cyclin dependent kinase like 1	8814	ENSG00000100490	___________MMELSTDTGWNQKSNFPPCD	S	0.859717	5	14.64239	15.07523	15.03458	15.38808	15.29297	14.35721	13.82177	14.40607	14.8473	0.596000477	0.559018453	-0.095352809	0.654371262
576	O95835	LATS1	large tumor suppressor kinase 1	9113	ENSG00000131023	HEIPTWQPNIPVRSNSFNNPLGNRASHSANS	S	0.997301	464	14.6407	14.74084	13.90905	14.7203	14.19729	14.58942	14.66128	14.34117	14.25022	0.024776574	0.012636185	-0.072142601	0.084778786
650	P18669	PGAM1	phosphoglycerate mutase 1	5223	ENSG00000171314	__MAAYKLVLIRHGESAWNLENRFSGWYDAD	S	1	14	14.62422	15.06785	14.41118	15.08589	15.07807	14.80307	14.88508	14.56582	14.66484	0.524455368	-0.004156431	-0.287922541	0.283766111
497	O14920	IKBKB	inhibitor of nuclear factor kappa B kinase subunit beta	3551	ENSG00000104365	LLKIACSKVRGPVSGSPDSMNASRLSQPGQL	S	0.763155	613	14.6154	14.66295	14.32249	15.21303	14.55045	15.07322	15.32938	14.68634	14.90006	0.682601869	-0.438313166	-0.411952655	-0.026360512
708	P54107	CRISP1	cysteine rich secretory protein 1	167	ENSG00000124812	RRVVPPASNMLKMSWSEEAAQNARIFSKYCD	S	0.996806	68	14.61039	13.02348	13.48457	13.72366	13.50047	13.91233	15.62085	14.37789	14.50177	0.950095887	-1.127357801	-0.006005605	-1.121352196
193	C9JHX5	STEAP2	STEAP2 metalloreductase	261729	ENSG00000157214	_____________MESISMMGSPKSLSETFL	S	0.98465	3	14.60964	14.73556	14.66005	14.81293	15.03733	15.32355	14.38613	15.10987	15.38542	0.407265036	-0.292060534	-0.389520645	0.097460111
1074	Q9H0K1	SIK2	salt inducible kinase 2	23235	ENSG00000170145	AIYFLLVERLKSHRSSFPVEQRLDGRQRRPS	S	0.847438	343	14.58496	14.81478	14.60432	14.95796	13.56796	14.51619	14.38485	13.99991	14.84598	0.151963803	0.257774671	0.32065169	-0.062877019
1132	Q9P270	SLAIN2	SLAIN motif family member 2	57606	ENSG00000109171	SRSGAVQGAGSLGPGSPVRAGASIPSSGAAS	S	0.992019	48	14.56825	14.5945	14.19998	14.53667	14.21379	14.54569	14.32643	14.34041	14.21591	0.293941652	0.159994443	0.022193909	0.137800535
1293	J3QRU1	YES1	YES proto-oncogene 1, Src family tyrosine kinase	7525	ENSG00000176105	LDNGGYYITTRAQFDTLQKLVKHYTEHADGL	T	1	237	14.56796	14.62131	13.95383	14.13939	14.44611	14.14919	13.96993	13.97996	14.18867	0.496886342	0.334847768	0.136133194	0.198714574
279	E7EX17	EIF4B	eukaryotic translation initiation factor 4B	1975	ENSG00000063046	WRSEETQERERSRTGSESSQTGTSTTSSRSK	S	0.961487	422	14.56534	14.84701	14.58291	14.8005	14.29835	14.79421	14.11569	14.25481	14.78703	0.340040912	0.279239655	0.0340662	0.245173454
1136	Q9UBF8	PI4KB	phosphatidylinositol 4-kinase beta	5298	ENSG00000143393	SVPARIPENRIRSTRSVENLPECGITHEQRA	S	1	413	14.54321	14.76477	14.27124	14.71714	14.42771	14.6853	14.92806	14.49079	14.92708	0.381143092	-0.255567551	-0.083639463	-0.171928088
188	C9JEN3	TMBIM1	transmembrane BAX inhibitor motif containing 1	64114	ENSG00000135926	GPGHGYDGEERAVSDSFGPGEWDDRKVRHTF	S	0.919762	83	14.52778	14.79817	14.12896	14.42633	14.8804	14.54303	14.25525	14.25636	13.93027	0.808981032	0.337676684	-0.131615957	0.469292641
865	Q5TH69	ARFGEF3	ARFGEF family member 3	57221	ENSG00000112379	ELLRQDKRPRSGSTGSSLSVSVRDAEAQIQA	S	0.546631	2100	14.52381	14.51015	14.88574	14.63038	14.3005	14.82967	14.04993	14.09466	14.529	0.780625228	0.415368398	0.053049405	0.362318993
109	A0A1B0GTL5	RAB11FIP5	RAB11 family interacting protein 5	26056	ENSG00000135631	DSAQSPKLFTHKRTYSDEANQMRVAPPRALL	S	0.98724	307	14.51958	14.75744	13.65015	14.27874	14.47687	14.65139	13.82267	14.20656	14.48991	0.165305298	0.136011442	-0.159942945	0.295954386
1210	A0A0C4DFY5	GPRC5C	G protein-coupled receptor class C group 5 member C	55890	ENSG00000170412	SYQGDMYPTRGVGYETILKEQKGQSMFVENK	T	0.999409	371	14.51354	14.50879	14.98308	14.97665	14.94663	14.91322	13.57566	14.47826	14.33553	1.275304712	0.538652738	-0.277030945	0.815683683
257	E9PGG1	DYNC1I2	dynein cytoplasmic 1 intermediate chain 2	1781	ENSG00000077380	VPPPMSPSSKSVSTPSEAGSQDSGDGAVGSR	S	0.769885	91	14.50897	14.1989	14.38734	14.71553	14.61638	14.27467	14.16797	15.5023	14.36461	0.151847661	-0.313220978	-0.170453072	-0.142767906
824	Q16348	SLC15A2	solute carrier family 15 member 2	6565	ENSG00000163406	_______MNPFQKNESKETLFSPVSIEEVPP	S	1	9	14.50594	14.81808	14.36919	14.99329	14.65782	15.05884	14.56278	14.76886	14.14856	0.709154021	0.071005185	-0.338914235	0.409919421
682	P42702	LIFR	LIF receptor alpha;LIF receptor subunit alpha	3977	ENSG00000113594	DIPNPENCKALQFQKSVCEGSSALKTLEMNP	S	0.999984	887	14.50296	15.09424	14.529	14.86723	14.86626	14.83017	14.00571	14.90181	14.71124	0.261807402	0.169147809	-0.1458203	0.314968109
656	P21730	C5AR1	complement C5a receptor 1	728	ENSG00000197405	RKSLPSLLRNVLTEESVVRESKSFTRSTVDT	S	0.998006	327	14.5009	14.43639	14.84426	14.90124	14.49073	14.00466	17.00405	14.66378	16.00147	0.993529138	-1.295915286	0.128308296	-1.424223582
1328	P04732	MT1E	metallothionein 1E	4493	ENSG00000169715	__MDPNCSCATGGSCTCAGSCKCKECKCTSC	T	0.835575	14	14.49848	15.13799	14.64397	15.01127	14.4296	14.34956	14.07431	14.54436	15.14478	0.067521723	0.172329585	0.163334846	0.008994738
909	Q7Z6Z7	HUWE1	HECT, UBA and WWE domain containing E3 ubiquitin protein ligase 1;H	10075	ENSG00000086758	ELEDLIDELLERDGGSGNSTIIVSRSGEDES	S	0.989625	2362	14.49748	15.0671	14.79101	14.37585	13.82058	14.32207	14.41217	14.04874	14.54043	1.0622332	0.451413155	0.612361908	-0.160948753
550	O76054	SEC14L2	SEC14 like lipid binding 2	23541	ENSG00000100003	RKYYVRDQVKQQYEHSVQISRGSSHQVEYEI	S	0.997133	198	14.47744	14.52564	14.12501	15.20606	14.64183	14.35769	15.05443	14.779	14.81613	0.762245242	-0.507158279	-0.359160423	-0.147997856
220	C9JKZ3	TMPRSS2	transmembrane serine protease 2	7113	ENSG00000184012	PSPVPQYAPRVLTQASNPVVCTQPKSPSGTV	S	0.993033	61	14.47618	14.51792	14.39801	14.39547	14.66284	14.93337	14.47649	14.08032	14.41713	0.704152714	0.139387449	-0.19985644	0.339243889
398	H0YFE9	C2CD5	C2 calcium dependent domain containing 5	9847	ENSG00000111731	SSGPSTPLKNQTYSFSPSKSYSRQSSSSDTD	S	0.855448	98	14.46282	14.65447	14.31295	14.92648	14.43189	14.48822	14.45063	14.59735	14.45488	0.182593069	-0.024206161	-0.138780912	0.11457475
360	F8W912	CCDC125	coiled-coil domain containing 125	202243	ENSG00000183323	QVSRISNYRRQSSTDSNSELSNEELRQCLNE	S	0.955992	103	14.46206	14.21113	14.41118	14.6605	13.87987	13.89699	14.37999	13.82078	13.35728	0.446511275	0.508773804	0.215666771	0.293107033
1079	Q9H2G2	SLK	STE20 like kinase	9748	ENSG00000065613	EEITESSSTEEMEVRSVVADTDQKALGSEVQ	S	1	655	14.45892	14.87656	14.45802	14.60334	14.78642	15.00075	14.32073	14.53089	14.64808	0.569270801	0.097935041	-0.199000359	0.296935399
866	Q5VSL9	STRIP1	striatin interacting protein 1	85369	ENSG00000143093	LPEDSIKVIRNMRAASPPASASDLIEQQQKR	S	0.996867	71	14.45674	14.85599	14.50804	14.43515	14.3025	14.06483	14.11105	14.3728	14.06449	1.126403341	0.42414252	0.339432398	0.084710121
335	Q9Y485	DMXL1	Dmx like 1	1657	ENSG00000172869	LKVWYNVENWRTAVTSPDGSSEKQSQGEIDF	S	0.982725	208	14.45295	15.3394	14.48809	15.24284	15.13053	15.07744	15.39607	15.2939	15.349	0.916503277	-0.586179733	-0.390124321	-0.196055412
736	Q01970	PLCB3	phospholipase C beta 3	5331	ENSG00000149782	VKNKKRHRPSAGGPDSAGRKRPLEQSNSALS	S	0.999055	413	14.43404	14.5446	14.20892	14.64723	14.39077	15.00742	14.01297	14.45095	13.88226	0.9818983	0.280458768	-0.285954793	0.566413562
1333	P11229	CHRM1	cholinergic receptor muscarinic 1	1128	ENSG00000168539	SSSERSQPGAEGSPETPPGRCCRCCRAPRLL	T	0.976272	254	14.42751	13.84931	14.40288	14.49517	14.09086	14.49742	14.6883	13.7789	14.2304	0.059933477	-0.005967776	-0.134581566	0.12861379
1358	P60880	SNAP25	synaptosome associated protein 25	6616	ENSG00000132639	EREQMAISGGFIRRVTNDARENEMDENLEQV	T	1	138	14.42055	13.09302	14.19606	14.45635	14.89378	14.60473	14.87743	14.12566	15.07443	0.718503126	-0.789293289	-0.74840641	-0.040886879
428	H7C4B6	BACH1	BTB domain and CNC homolog 1	571	ENSG00000156273	_____XPWLGIRISESPEPGQRTFTTLSSVN	S	0.995743	11	14.41568	14.35073	14.89945	15.17574	14.66584	14.65788	14.52466	14.27634	14.67043	0.49019795	0.064805984	-0.277865728	0.342671712
974	Q8TEA8	DTD1	D-aminoacyl-tRNA deacylase 1;D-tyrosyl-tRNA deacylase 1	92675	ENSG00000125821	SKERNTPRKEDRSASSGAEGDVSSEREP___	S	0.89338	197	14.40833	14.34963	14.69843	14.48129	13.71167	14.5629	13.72035	14.21834	14.21728	0.454861059	0.433475176	0.2335097	0.199965477
78	A0A0C4DG33	PEX1	peroxisomal biogenesis factor 1	5189	ENSG00000127980	GKDQLFSQPPVLRTASQEGCQELTQEQRDQL	S	0.834096	1124	14.40468	15.09898	14.19653	15.11862	14.7791	15.14808	15.01149	15.10685	15.02033	0.769948045	-0.479495684	-0.448540052	-0.030955633
184	H0Y5J4	DBNL	drebrin like	28988	ENSG00000136279	IFKQKERAMSTTSISSPQPGKLRSPFLQKQL	S	0.866585	204	14.40401	14.50637	14.55489	14.60716	14.47927	14.72989	14.14569	14.6434	14.75848	0.109849524	-0.027437846	-0.117016157	0.089578311
397	H0YFA4	CRIP2	cysteine rich protein 2	1397	ENSG00000182809	ERKASGPPKGPSRASSVTTFTGEPNTCPRCS	S	0.712089	99	14.39393	15.06781	14.54671	14.65877	14.76134	15.07682	14.31543	14.50414	14.82675	0.294859741	0.120712598	-0.162824949	0.283537547
1073	Q9H0B6	KLC2	kinesin light chain 2	64837	ENSG00000174996	PYGEYGSWYKACKVDSPTVNTTLRSLGALYR	S	0.98334	445	14.39285	13.99506	14.11504	14.28605	13.8608	13.85759	13.639	13.96262	14.55878	0.085381474	0.114184062	0.166172345	-0.051988284
215	C9JI98	TMEM238	transmembrane protein 238	388564	ENSG00000233493	PSALARLARKLSRRWSAPAAAGQRPAPGSRR	S	1	124	14.38188	14.60322	14.02193	13.97154	14.22769	13.86109	13.84872	14.35748	13.36482	0.513399455	0.478671392	0.315571785	0.163099607
748	Q02383	SEMG2	semenogelin 2	6407	ENSG00000124157	LHHGEKSVQKDVSKGSISIQTEEKIHGKSQN	S	0.798772	313	14.37463	14.41237	14.89107	15.33532	14.78249	14.31423	14.15054	15.19963	14.32804	0.119151506	-4.76837E-05	-0.251320203	0.251272519
954	Q8N9U0	TC2N	tandem C2 domains, nuclear	123036	ENSG00000165929	PGSPGLSKSMFDLTNSSQRFIQRHDSLSSVP	S	0.844399	181	14.37082	13.89169	14.00974	14.39279	13.59607	14.20228	13.77839	13.65642	14.51095	0.027654488	0.108831406	0.027038574	0.081792831
291	Q5JWQ2	NRP1	neuropilin 1	8829	ENSG00000099250	GVKLKKDKLNTQSTYSEA_____________	S	0.972758	103	14.37062	14.21917	15.11411	15.24607	14.96484	15.01742	15.06588	15.81173	15.07347	0.908041026	-0.749059995	-0.508143425	-0.24091657
416	H3BTD6	EDC3	enhancer of mRNA decapping 3	80153	ENSG00000179151	NIPKRTDVKSQDVAVSPQQQQCSKSYVDRHM	S	0.984837	18	14.3681	14.55842	14.44333	14.78678	14.72563	14.76503	14.9376	14.59339	13.90351	0.339161137	-0.021550179	-0.302529971	0.280979792
864	Q5T0Z8	C6orf132	chromosome 6 open reading frame 132	647024	ENSG00000188112	PRSFKVPPPTPVRTSSIPVQEAQEAPRKEEG	S	0.987585	314	14.3614	15.03905	14.06516	14.29613	14.93691	14.23631	13.92007	14.45411	14.12533	0.259365562	0.322031339	-0.001248995	0.323280334
553	O94819	KBTBD11	kelch repeat and BTB domain containing 11	9920	ENSG00000273645	ASPEERACPEEPAAPSPEPRVWLEDPASPEE	S	1	107	14.35728	14.43892	14.25879	14.98589	14.23347	14.35735	14.32994	14.61258	14.54333	0.164350872	-0.143621445	-0.173905373	0.030283928
950	Q8N5J2	MINDY1	MINDY lysine 48 deubiquitinase 1	55793	ENSG00000143409	VETIRACSMPQELPQSPRTRQPEPDFYCVKW	S	0.999997	103	14.34339	14.92741	14.44631	14.75353	15.28692	14.65743	14.65128	15.0152	15.009	0.446570699	-0.319453557	-0.326923052	0.007469495
840	Q53TN4	CYBRD1	cytochrome b reductase 1	79901	ENSG00000071967	AARKRNLALDEAGQRSTM_____________	S	0.530075	226	14.32573	14.67777	14.46142	14.78775	15.06794	14.59333	14.51958	13.98744	14.09737	1.292504932	0.286842028	-0.328034401	0.614876429
676	P40189	IL6ST	interleukin 6 signal transducer	3572	ENSG00000134352	SSPDISHFERSKQVSSVNEEDFVRLKQQISD	S	0.852888	778	14.31868	15.09857	14.12283	14.23422	15.08779	15.01035	14.56653	15.04776	14.21143	0.101050539	-0.095215797	-0.264095942	0.168880145
1219	A0A286YFL2	PHGDH	phosphoglycerate dehydrogenase	26227	ENSG00000092621	VINAAEKLQVVGRAGTGVDNVDLEAATRKGI	T	1	78	14.30884	14.96118	14.3903	14.78315	14.0182	14.63934	13.89548	14.53667	14.33183	0.218298195	0.298780123	0.073209127	0.225570997
1086	Q9HAS3	SLC28A3	solute carrier family 28 member 3	64078	ENSG00000197506	TNTKQDEEQVTVEQDSPRNREHMEDDDEEMQ	S	0.990145	63	14.30128	14.21181	14.15664	14.81198	14.46939	13.97199	14.54043	14.5243	14.2735	0.252871008	-0.222833951	-0.194541931	-0.02829202
150	A2RRE0	WDR7	WD repeat domain 7	23335	ENSG00000091157	RPPRPSTPDLSKARGSPPTSSNIVQGQIKQV	S	0.737413	260	14.29742	14.46557	14.30392	14.6665	14.30092	13.55866	12.75154	13.48872	14.21348	0.718414992	0.871053696	0.180274328	0.690779368
1429	Q8IY63	AMOTL1	angiomotin like 1	154810	ENSG00000166025	LRPARSVPSIAAATGTHSRQTSLTSSQLAEE	T	0.564496	753	14.2849	14.56707	13.76394	15.02726	14.3528	14.20297	14.44249	14.54702	14.64397	0.338953198	-0.339190801	-0.32237498	-0.016815821
1319	O95835	LATS1	large tumor suppressor kinase 1	9113	ENSG00000131023	SYENVDSGDKEKKQITTSPITVRKNKKDEER	T	0.580989	611	14.28446	14.71306	14.67772	14.8009	14.34367	14.46237	13.79705	14.18611	14.28944	1.001074323	0.467545509	0.022767067	0.444778442
104	A0A0U1RQF0	FASN	fatty acid synthase	2194	ENSG00000169710	LKPNTSVQFLRLGMLSPEGTCKAFDTAGNGY	S	0.946811	207	14.28164	15.29641	14.74704	15.15221	14.78305	14.7006	14.44689	14.82858	14.46576	0.226520953	0.194621086	-0.103588104	0.29820919
430	I3L0N3	NSF	N-ethylmaleimide sensitive factor, vesicle fusing ATPase	4905	ENSG00000073969	KVVNGPEILNKYVGESEANIRKLFADAEEEQ	S	0.999995	293	14.28099	15.01659	14.10263	13.54352	13.43202	14.09704	13.81027	13.88312	13.9184	1.117201262	0.596140862	0.775876681	-0.179735819
721	P62820	RAB1A	RAB1A, member RAS oncogene family	5861	ENSG00000138069	QIWDTAGQERFRTITSSYYRGAHGIIVVYDV	S	0.811738	78	14.27219	14.29103	14.38963	14.66356	14.00194	15.00048	14.14736	14.21439	14.57708	0.218620333	0.004672368	-0.237709363	0.242381732
385	H0Y704	ZNF185	zinc finger protein 185 with LIM domain	7739	ENSG00000147394	PAQQPADPSTPERQSSPSGSEQLVRRESCGS	S	0.964593	212	14.26993	14.92898	14.79391	14.24436	14.66523	14.73121	13.92277	14.44158	14.02825	0.826387306	0.533404668	0.117339134	0.416065534
1440	Q8TDW5	SYTL5	synaptotagmin like 5	94122	ENSG00000147041	STGSSDLNDQEPGPRTPKSSRSNGVTPGTQS	T	0.985633	247	14.26701	14.40826	14.87748	15.12957	14.65581	14.92731	14.28865	14.54858	14.78566	0.644251434	-0.023380915	-0.386648496	0.363267581
427	H7C284	MFSD6	major facilitator superfamily domain containing 6	54842	ENSG00000151690	SEIQPLQGTNENRENSPAGRAQPVPCETHSD	S	0.926246	33	14.26312	14.3568	14.6082	14.05223	14.28005	14.47744	14.14784	14.05943	14.4669	0.265308007	0.184647242	0.139466921	0.045180321
53	X6RE05	UBR4	ubiquitin protein ligase E3 component n-recognin 4	23352	ENSG00000127481	RVLRPRNKRRHVTLPSSPRSNTPMGDKDDDD	S	0.617843	361	14.25864	14.27612	14.35473	14.56415	14.60252	14.52845	14.4166	13.78095	14.36256	0.82701195	0.109795252	-0.268544515	0.378339767
1428	Q8IY63	AMOTL1	angiomotin like 1	154810	ENSG00000166025	GHGYYMAGGTSQKSRTEGRPTVNRANSGQAH	T	0.847764	180	14.2534	14.15434	14.83269	15.27485	14.61321	14.51724	14.88011	14.89785	14.81568	0.599839833	-0.451066017	-0.388287544	-0.062778473
307	E9PR85	PELI3	pellino E3 ubiquitin protein ligase family member 3	246330	ENSG00000174516	_____MVLEGNPEVGSPRTSDLQHRGNKGSC	S	1	11	14.24956	15.05257	14.77782	15.02899	14.49754	14.54671	14.84201	14.69191	14.34312	0.018002324	0.067638715	0.00223573	0.065402985
472	M0QZR4	ARHGEF1	Rho guanine nucleotide exchange factor 1	9138	ENSG00000076928	GPPRDGDGVPGGGPLSPARTQEIQENLLSLE	S	1	919	14.24934	14.00273	13.74368	14.56326	14.18921	13.79513	14.92773	14.66111	14.0228	0.550476581	-0.5386343	-0.183950106	-0.354684194
898	Q76N89	HECW1	HECT, C2 and WW domain containing E3 ubiquitin protein ligase 1	23072	ENSG00000002746	NHLTHRQHLQRLRSYSAGEASEVSRNRGASL	S	0.994618	1041	14.23175	14.36571	13.96434	15.01846	14.05604	14.7066	14.54225	14.10812	13.99072	0.459085036	-0.026430766	-0.40643851	0.380007744
823	Q16204	CCDC6	coiled-coil domain containing 6	8030	ENSG00000108091	RILQEKLDQPVSAPPSPRDISMEIDSPENMM	S	0.999999	244	14.2231	14.54243	14.73392	14.9792	13.90294	14.59374	14.74662	14.52037	14.55944	0.043315889	-0.108996709	0.007851601	-0.11684831
929	Q8IWU2	LMTK2	lemur tyrosine kinase 2	22853	ENSG00000164715	STDARTHSLDNRSQDSPGESEETLRLTESDS	S	0.900926	886	14.21735	14.37014	13.93839	14.47852	14.36817	14.19152	13.82028	13.93507	14.27714	0.669996639	0.164461772	-0.17077446	0.335236231
1151	Q9UPU5	USP24	ubiquitin specific peptidase 24	23358	ENSG00000162402	SRPFRNVSRQTSRQMSLCGTPEKSSYRQLSV	S	0.994517	1285	14.21082	14.59374	14.17703	15.09424	15.04597	14.43977	14.45366	14.98966	14.95442	0.85194749	-0.472048442	-0.532795588	0.060747147
1310	O43182	ARHGAP6	Rho GTPase activating protein 6	395	ENSG00000047648	ATEGRAHPAVSRACSTPHVQVAGKAERPTAR	T	0.548849	618	14.20166	14.58848	14.41343	14.68404	14.69191	14.56695	14.55746	14.1782	14.95742	0.283102487	-0.163168907	-0.246438344	0.083269437
461	J3QRY4	PSMD11	proteasome 26S subunit, non-ATPase 11	5717	ENSG00000108671	__MAAAAVVEFQRAQSLLSTDREASIDILHS	S	0.65102	14	14.1848	13.57211	13.08788	14.70941	14.05206	14.09383	14.57495	14.09984	14.63612	0.964007082	-0.82204024	-0.670170466	-0.151869774
1150	Q9UNW1	MINPP1	multiple inositol-polyphosphate phosphatase 1	9562	ENSG00000107789	QSCQTSEECELARANSTSDEL__________	S	0.886386	281	14.18364	14.85501	14.15434	15.32874	14.77329	14.82486	15.01785	15.18309	14.82426	1.055831717	-0.610737801	-0.577967962	-0.032769839
1463	Q9H2G2	SLK	STE20 like kinase	9748	ENSG00000065613	FLSKTKDSGSISLQETRRQKKTLKKTRKFIV	T	0.998821	785	14.15869	13.54352	14.27641	14.33922	13.94663	14.68513	14.09019	13.96921	13.87757	0.416952184	0.013881683	-0.330789884	0.344671567
570	O95716	RAB3D	RAB3D, member RAS oncogene family	9545	ENSG00000105514	_____________MASAGDTQAGPRDAADQN	S	0.999969	3	14.15498	13.64914	14.24198	13.62628	14.0921	14.28179	14.22889	14.54581	14.18294	0.420368299	-0.303845723	0.015314102	-0.319159826
591	P04279	SEMG1	semenogelin 1	6406	ENSG00000124233	RLHYGENGVQKDVSQSSIYSQTEEKAQGKSQ	S	0.825893	312	14.14187	13.89794	13.21155	13.88817	14.56582	13.3681	14.2128	13.75311	13.50755	0.048887007	-0.074032466	-0.190241496	0.11620903
942	Q8IZ41	RASEF	RAS and EF-hand domain containing	158158	ENSG00000165105	LAREVKKRTDKDDSRSITNLTGTNSKKSPQM	S	0.943151	719	14.13162	14.74426	13.59922	14.3581	13.78167	14.15529	13.39674	13.71918	13.88446	0.48758052	0.491572062	0.060008685	0.431563377
715	P61006	RAB8A	RAB8A, member RAS oncogene family	4218	ENSG00000167461	ARDIKAKMDKKLEGNSPQGSNQGVKITPDQQ	S	0.999832	181	14.13041	15.07108	13.91858	14.11138	14.16671	14.01777	14.18611	13.72888	14.10722	0.287016213	0.365956624	0.274739901	0.091216723
834	Q3KQU3	MAP7D1	MAP7 domain containing 1	55700	ENSG00000116871	RTHPSAAVPVCPRSASASPLTPCSVTRSVHR	S	0.941935	329	14.1073	14.67959	14.25518	14.33071	14.48407	14.86302	14.33309	13.99639	14.64515	0.223130922	0.022480965	-0.211911201	0.234392166
401	H0YM86	DCAF11	DDB1 and CUL4 associated factor 11	80344	ENSG00000100897	PRMLHQRERGLCHRGSFSLGEQSRVISHFLP	S	0.999135	121	14.09713	14.03531	13.98059	14.44061	13.91989	14.1642	14.18681	14.22882	14.11309	0.295460159	-0.13856411	-0.137223244	-0.001340866

11	F8W7S5	RRBP1	ribosome binding protein 1	6238	ENSG00000125844	QEQQQQMAELHSKLQSSEAEVRSKCEELSGL	S	0.96188	265	14.07623	13.92741	13.88931	14.24867	14.63759	13.97011	15.06659	14.38499	14.21515	0.771742174	-0.591260592	-0.321136793	-0.2701238
663	P27987	ITPKB	inositol-trisphosphate 3-kinase B	3707	ENSG00000143772	RLGRARSPSPCPFRSSSQPPGRVLVQGARSE	S	0.534972	183	14.06878	14.36673	14.20105	14.4283	13.72686	13.81017	13.00016	13.26152	13.8877	1.17458811	0.829061508	0.2237463	0.605315208
299	E9PPF2	TYK2	tyrosine kinase 2	7297	ENSG00000105397	ILTVAQRSQAPDGMQSLRLRKFPIEQQDGAF	S	1	499	14.06819	14.60194	14.54007	14.40601	13.49897	15.14867	13.71639	14.0317	13.73513	0.420098774	0.575659434	0.052182833	0.523476601
1345	X6RJP6	TAGLN2	transgelin 2	8407	ENSG00000158710	NQLQEGKNVIGLQMGTNRGASQA________	T	1	180	14.06743	13.99736	13.63186	13.98317	14.22091	14.49947	14.04003	13.82943	13.65184	0.80294263	0.058451335	-0.335634232	0.394085566
1394	Q2M2I8	AAK1	AP2 associated kinase 1	22848	ENSG00000115977	EPAIQAPVRQQPKVQTTPPPAVQGQKVGSLT	T	0.596191	605	14.05782	14.25842	14.25842	13.97244	14.11171	14.05477	13.49286	14.4931	13.41455	0.350872114	0.391382535	0.145246188	0.246136347
774	Q09666	AHNAK	AHNAK nucleoprotein	79026	ENSG00000124942	KFNFSKPKGKGGVTGSPEASISGSKGDLKSS	S	0.973562	5731	14.05333	14.13675	14.05757	13.47725	14.30471	14.13899	14.03832	13.54858	13.65855	0.376487067	0.334068616	0.108900388	0.225168228
1164	Q9Y281	CFL2	cofilin 2	1073	ENSG00000165410	_____________MASGVTVNDEVIKVFNDM	S	0.909668	3	14.0292	14.06348	13.76446	14.45057	13.40514	13.92954	13.66011	14.02877	13.36987	0.192199356	0.266129812	0.023963928	0.242165883
158	C9JXU1	SHROOM1	shroom family member 1	134549	ENSG00000164403	VPARPPATHPRSASLSHPGGEGEPARSRAPA	S	0.860423	190	14.02393	14.84696	14.90063	15.15616	14.9796	15.00891	14.97773	14.64796	15.27947	0.538747951	-0.377879779	-0.457719485	0.079839706
505	O15552	FFAR2	free fatty acid receptor 2	2867	ENSG00000126262	RAFGRGLQVLRNQGSSLLGRRGKDTAEGTNE	S	0.5	297	13.98308	14.11699	13.81388	14.322	14.00088	14.34755	14.55465	14.51274	14.20488	1.241863814	-0.452769279	-0.252157847	-0.200611432
504	O15552	FFAR2	free fatty acid receptor 2	2867	ENSG00000126262	RRAFGRGLQVLRNQGSSLLGRRGKDTAEGTN	S	0.5	296	13.98308	14.11699	13.81388	14.322	14.00088	14.34755	14.55465	14.51274	14.20488	1.241863814	-0.452769279	-0.252157847	-0.200611432
278	E7EX17	EIF4B	eukaryotic translation initiation factor 4B	1975	ENSG00000063046	SNPPARSQSSDTEQQSPTSGGGKVAPAQPSE	S	0.956606	509	13.96624	14.08854	14.23122	13.71864	14.0759	13.93884	13.94608	13.43111	13.57459	1.051344793	0.444737752	0.184204737	0.260533015
797	Q13796	SHROOM2	shroom family member 2	357	ENSG00000146950	SEPEKMNEVGLTRGYSPHQHPRTSEDTVGTF	S	0.979246	779	13.96344	14.20465	13.54025	14.27954	14.28793	14.29462	13.85097	14.65827	14.20762	0.506793701	-0.336174965	-0.384584109	0.048409144
978	Q8WUD1	RAB2B	RAB2B, member RAS oncogene family	84932	ENSG00000129472	DVHNEANGIKIGPQQSISTSVGPSASQRNSR	S	0.932062	127	13.95556	13.47307	13.8451	14.23272	12.96002	13.01789	14.25967	13.2162	12.56622	0.140006652	0.410545985	0.35436662	0.056179365
1098	Q9NR34	MAN1C1	mannosidase alpha class 1C member 1	57134	ENSG00000117643	RSRLRHPVLGTRADESQEPQSQVRAQREKIK	S	0.937486	164	13.94178	14.71306	13.30563	14.57607	14.8011	14.86549	14.77144	14.77741	14.48003	0.880153964	-0.689466794	-0.7607282	0.071261406
691	P50150	GNG4	G protein subunit gamma 4	2786	ENSG00000168243	_______MKEGMSNNSTTSISQARKAVEQLK	S	0.885118	9	13.91373	14.37985	13.88531	14.48759	13.98735	14.41818	14.45969	14.31437	13.57436	0.151679367	-0.056506157	-0.238075574	0.181569417
684	P45974	USP5	ubiquitin specific peptidase 5	8078	ENSG00000111667	AEAAMDISEGRSAADSISESVPVGPKVRDGP	S	0.693415	760	13.89983	14.63294	13.42035	14.68343	14.36844	14.57252	14.66233	14.34624	14.45295	0.665842138	-0.502799034	-0.557090759	0.054291725
915	Q86V87	FAM160B2	family with sequence similarity 160 member B2	64760	ENSG00000158863	IAFVKFPPHDPRQNVSPAPEGQV________	S	1	736	13.88674	13.20927	13.41164	14.46614	13.97343	13.36878	13.98522	13.28076	13.86118	0.290680951	-0.206500371	-0.433563232	0.227062861
1470	Q9NSK0	KLC4	kinesin light chain 4	89953	ENSG00000137171	PSAAPLQVSRGLSASTMDLSSSS________	T	0.935422	535	13.86728	13.6808	13.51508	13.84569	13.42758	13.90124	13.79269	13.43957	13.9719	0.013923363	-0.046999931	-0.037114143	-0.009885788
491	O14745	SLC9A3R1	SLC9A3 regulator 1	9368	ENSG00000109062	______________MSADAAAGAPLPRLCCL	S	1	2	13.85895	14.01985	13.46404	14.30706	13.79482	13.11104	13.39071	14.25695	13.63334	0.002855653	0.020617167	0.043306986	-0.022689819
930	Q8IWY9	CDAN1	codanin 1	146059	ENSG00000140326	CPTPELGSPLPSRTGSLTDEPADPARVSSRQ	S	0.834409	284	13.85555	14.32453	13.07994	14.2431	14.46875	13.91924	14.27976	13.99806	13.42836	0.28778699	-0.148719788	-0.457022985	0.308303197
1224	A0A2R8YDC1	ABCC4	ATP binding cassette subfamily C member 4	10257	ENSG00000125257	LKKDNEESEQPPVPGTPTLRNRTFSE_____	T	0.879767	659	13.84323	14.84921	13.48029	14.81603	14.47149	14.3526	13.83131	14.14458	14.34907	0.386219963	-0.050743421	-0.489128113	0.438384692
952	Q8N9U0	TC2N	tandem C2 domains, nuclear	123036	ENSG00000165929	PSSSSSRKNSQGSNRSLDTITLSGDERDFGR	S	0.910413	211	13.81107	13.45173	13.34471	14.2762	13.55519	13.23751	12.4896	13.42548	13.9771	0.158073278	0.238444964	-0.153794289	0.392239253
462	K7EMU2	PRKAR1A	protein kinase cAMP-dependent type I regulatory subunit alpha	5573	ENSG00000108946	LQKAGTRTDSREDEISPPPPNPVVKGRRRRG	S	0.99673	83	13.7788	14.05918	14.10738	14.06255	13.96578	13.82217	13.42849	12.88461	13.82108	1.082968228	0.603725433	0.031614621	0.572110812
1209	A0A0C4DFY5	GPRC5C	G protein-coupled receptor class C group 5 member C	55890	ENSG00000170412	PRATANSQVMGSANSTLRAEDMYSAQSHQAA	T	0.546984	452	13.77035	14.89349	14.04388	14.31472	13.85973	14.34603	14.48696	14.53867	14.53077	0.283508573	-0.282893499	0.062414487	-0.345307986
1100	Q9NRM7	LATS2	large tumor suppressor kinase 2	26524	ENSG00000150457	AKGDKGGKDKKQIQTSPVPVRKNSRDEEKRE	S	0.964524	576	13.75999	14.03918	13.59828	14.43957	14.07213	13.42351	13.81688	13.83979	13.8896	0.10193334	-0.049607913	-0.179253578	0.129645665
537	O75083	WDR1	WD repeat domain 1	9948	ENSG00000071127	NNPSKPLHVIKGHSKSIQCLTVHKNGGKSYI	S	0.999755	322	13.7528	13.98371	13.70552	13.91989	14.19406	13.65475	13.42482	13.3143	13.7687	0.866789436	0.311402003	-0.108889898	0.420291901
438	I3L456	RABEP1	rabaptin, RAB GTPase binding effector protein 1	9135	ENSG00000029725	KSDNDMFKDGLRRAQSTDSLGTSGSLQSKAL	S	0.734665	94	13.73851	14.34658	13.56177	14.16828	13.24441	14.17344	13.62822	13.72473	13.87527	0.046344534	0.139550209	0.020243963	0.119306246
1041	Q9BW04	C1orf116;SARG	chromosome 1 open reading frame 116	79098	ENSG00000182795	LPRNIHIARSQNFRKSTTQASSHNPGEPGRL	S	0.577197	149	13.73015	13.27719	11.93965	13.41561	12.8575	13.76984	12.9969	13.40048	12.0431	0.174123721	0.168839455	-0.365317345	0.534156799
876	Q5XXA6	ANO1	anoctamin 1	55107	ENSG00000131620	PCNHHNTKACPDSLGSPAPSHAYHGGVL___	S	0.985836	974	13.72824	13.7121	14.05723	14.66622	14.30549	13.81017	13.12093	14.93493	14.31196	0.163371805	-0.290088018	-0.428106308	0.13801829
280	E7EX29	YWHAZ	tyrosine 3-monooxygenase/tryptophan 5-monooxygenase activation prote	7534	ENSG00000164924	AGMDVELTVEERNLLSVAYKNVIGARRASWR;TEL	S	0.99999	45	13.72153	13.60478	13.96127	13.88388	13.90398	14.27205	13.44333	14.11935	13.74546	0.371829011	-0.006854375	-0.257442156	0.250587781
948	Q8N4C8	MINK1	misshapen like kinase 1	50488	ENSG00000141503	ERLQRQLQQEHAYLKSLQQQQQQQLQKQQQ	S	1	478	13.6937	13.33651	14.56027	13.41032	13.9438	13.35494	13.04467	13.69534	13.45507	0.31361351	0.465136528	0.293810209	0.171326319
62	E9PMS6	LMO7	LIM domain 7	4008	ENSG00000136153	GVATTQSPTPRSHSPSASQSGSQLRNRSVSG	S	0.816403	1187	13.67673	14.43632	13.79106	13.76746	13.87958	14.07757	14.00615	13.72526	13.85856	0.049883175	0.104712168	0.059833527	0.044878642
256	E7EMC7	SQSTM1	sequestosome 1	8878	ENSG00000161011	GGKRSRLTPVSPESSSTEEKSSSQPSSCCSD	S	0.514304	277	13.67021	14.05291	13.84431	13.83427	13.84137	13.90463	13.87306	13.77787	14.13611	0.087788999	-0.073202133	-0.00428009	-0.068922043
1212	A0A0G2JIW1	HSPA1B	heat shock protein family A (Hsp70) member 1B	3304	ENSG00000232804	GFGAQGPKGGSGSGPTIEEVD__________	T	0.993431	637	13.66955	13.61506	13.93378	13.38033	13.21034	14.07915	13.79695	13.79928	13.50829	0.12870132	0.037954013	0.182857831	-0.144903819
17	A0A087WY82	F11R	F11 receptor	50848	ENSG00000158769	TKKGTSSKKVIYSQPSARSEGEFKQTSSFLV	S	0.999824	264	13.65721	13.81127	13.28245	13.18519	13.7363	13.02041	13.62114	14.08456	13.1193	0.214236355	-0.024686813	0.269681295	-0.294368108
1199	Q9P2T1	GMPR2	guanosine monophosphate reductase 2	51292	ENSG00000100938	GADIIKVGIGPGSVCTTRKKTGVGYPQLSAV	T	0.971867	205	13.65116	13.57388	13.47611	13.42062	13.46135	13.51853	13.95256	13.10317	13.57329	0.054802797	0.024045626	0.10022068	-0.076175054
1053	Q9C0H2	TTYH3	tweety family member 3	80727	ENSG00000136295	FQNPRCENTPLIGRESPPPSYTSSMRAKYLA	S	0.872048	325	13.64476	13.89888	13.27748	14.16577	14.25185	13.80786	14.37048	13.78238	14.18635	0.889064344	-0.506031672	-0.468120893	-0.037910779
1228	H3BU16	JPT2	Jupiter microtubule associated homolog 2	90861	ENSG00000206053	PGGKTSDIFGSPVTATSRLAHPNKPKDHVFL	T	0.700825	86	13.63424	13.37978	12.95108	13.27002	13.47586	14.05994	12.70109	13.22358	13.3443	0.518356845	0.232049306	-0.280233383	0.512282689
65	A0A0B4J1Z0	COBLL1	cordon-bleu WH2 repeat protein like 1	22837	ENSG00000082438	FSFFQRSKKKRDQTASAPATPLVNKHRPTFT	S	0.821684	269	13.63265	14.31649	14.21234	13.11996	13.33762	13.77982	14.18789	13.92898	13.89747	1.137471356	0.049050331	0.641361872	-0.592311541
233	D6R8Z7	TSC22D3	TSC22 domain family member 3	1831	ENSG00000157514	SGLQRGSSGENNNPGSPTVSNFRQLQEKLVF	S	0.76872	42	13.62617	13.74126	13.32671	13.97289	13.53138	13.70542	13.40048	13.69218	13.71843	0.229560813	-0.038983981	-0.171851794	0.132867813
1005	Q96JZ2	HSH2D	hematopoietic SH2 domain containing	84941	ENSG00000196684	QAPKDRKVPTRKAERSVSCIEVTPGDRSWHQ	S	0.997671	239	13.62045	13.12341	12.61946	13.89737	13.24279	14.0402	13.01228	13.56224	13.25639	0.600110784	-0.155860265	-0.605680466	0.449820201
478	O00161	SNAP23	synaptosome associated protein 23	8773	ENSG00000092531	SGKAYKTTWGDGGENSPCNVVSKQPGPVTNG	S	0.993834	110	13.60698	13.48822	13.3253	13.58988	13.29806	13.15747	12.84404	13.55375	13.39634	0.192892528	0.208793004	0.125027974	0.08376503
778	Q12923	PTPN13	protein tyrosine phosphatase non-receptor type 13;protein tyrosine phos	5783	ENSG00000163629	DIERASFRSLNLQAESVRGFNMGRAISTGSL	S	0.999995	897	13.58296	14.56903	13.94736	14.44184	13.6591	14.07732	13.74924	13.77479	13.77859	0.231477037	0.26557827	-0.026303291	0.291881561
54	X6RE05	UBR4	ubiquitin protein ligase E3 component n-recognin 4	23352	ENSG00000127481	VLRPRNKRRHVTLPSSPRSNTPMGDKDDDDD	S	0.539533	362	13.58191	14.50953	13.44489	14.70309	14.08788	13.70887	14.03015	13.5394	14.0162	0.179261986	-0.016476949	-0.321173032	0.304696083
668	P29966	MARCKS	myristoylated alanine rich protein kinase C substrate	4082	ENSG00000277443	EKGEPAAAAAPEAGASPVEKEAPAEGEAAEP	S	1	101	13.58167	13.54702	12.98893	13.26913	13.19262	13.41917	13.50146	12.75603	13.14569	0.202202277	0.238142649	0.078902245	0.159240405
1431	Q8IYJ3	SYTL1	synaptotagmin like 1	84958	ENSG00000142765	KTAVKKRNLNPVFNETLRYSVPQAELQGRVL	T	1	333	13.54194	13.75405	14.06937	14.1899	14.00044	13.95037	13.32221	12.84778	14.18952	0.514296979	0.335284551	-0.258451462	0.593736013
1450	Q96KN1	LRATD2;FAM84B	LRAT domain containing 2;family with sequence similarity 84 member B	157638	ENSG00000168672	HPAEPEEGDSNVARTTPPPGRPPAPSSEEED	T	0.519928	289	13.53976	14.53217	13.75154	13.91177	13.17393	13.62365	13.63436	13.85526	13.45507	0.295409694	0.292931875	0.371378581	-0.078446706
761	Q02790	FKBP4	FK506 binding protein 4;FKBP prolyl isomerase 4	2288	ENSG00000004478	TDTEMKEEQKSNTAGSQSQVETEA_______	S	0.969398	451	13.53515	14.23586	13.9532	13.77674	13.96894	13.6364	13.4921	13.77077	13.41402	0.534796134	0.349106471	0.114046097	0.235060374
195	G3V1D0	EML3	EMAP like 3	256364	ENSG00000149499	RPLQPPQRADTPRRNSSSSSSPSERPRQKLS	S	0.665943	128	13.52748	13.85077	13.5326	13.72611	12.95743	13.83891	13.80655	13.68278	14.32587	0.424452502	-0.301447868	0.12946701	-0.430914879
154	A6NKB8	RNPEP	arginyl aminopeptidase	6051	ENSG00000176393	_____________MASGEHSPGSGAARRPLH	S	0.909574	3	13.48306	13.14208	12.96353	13.30791	12.82197	13.12425	13.19854	13.44048	13.33302	0.339337196	-0.127789497	0.111509641	-0.239299138
1404	Q5R3F8	ELFN2	extracellular leucine rich repeat and fibronectin type III domain containin	114794	ENSG00000166897	GEKLPTAKGLEAGLDTPKVATKGNYIEVRTG	T	1	493	13.4818	13.89879	13.23485	13.33957	14.08348	13.61264	13.11961	13.08731	13.29045	0.759865759	0.372688611	-0.140084267	0.512772878
144	C9J591	ARHGEF5	Rho guanine nucleotide exchange factor 5	7984	ENSG00000050327	SQQRLESLSETPGPSSPRQPRKALVSSESYL	S	0.499925	136	13.47002	14.22212	13.73978	14.11301	13.46582	13.91849	13.75666	14.22536	13.43528	0.001906615	0.004873594	-0.021800677	0.026674271
143	C9J591	ARHGEF5	Rho guanine nucleotide exchange factor 5	7984	ENSG00000050327	QSQQRLESLSETPGPSSPRQPRKALVSSESY	S	0.499925	135	13.47002	14.22212	13.73978	14.11301	13.46582	13.91849	13.75666	14.22536	13.43528	0.001906615	0.004873594	-0.021800677	0.026674271
552	O94804	STK10	serine/threonine kinase 10	6793	ENSG00000072786	QVAQEKQVAEQGGDLSPAANRSQKASQSRPN	S	1	438	13.46799	12.58939	13.20985	13.52209	13.79228	13.3319	13.17479	13.50159	12.96672	0.539643656	-0.125288328	-0.459679604	0.334391276
435	O14641	DVL2	dishevelled segment polarity protein 2	1856	ENSG00000004975	ESVVSLRRERPRRRDSSEHGAGGHRTGGPSR	S	0.5	169	13.43957	13.94535	13.85428	13.80977	13.17891	14.16011	13.38613	13.79665	13.25939	0.192469297	0.265679359	0.030139605	0.235539754
436	O14641	DVL2	dishevelled segment polarity protein 2	1856	ENSG00000004975	SVVSLRRERPRRRDSSEHGAGGHRTGGPSRL	S	0.5	170	13.43957	13.94535	13.85428	13.80977	13.17891	14.16011	13.38613	13.79665	13.25939	0.192469297	0.265679359	0.030139605	0.235539754
583	P01303	NPY	neuropeptide Y	4852	ENSG00000122585	RSSPETLISDLLMRESTENVPRTRLEDPAMW	S	0.946794	82	13.42035	14.18139	14.38667	16.31791	14.26905	14.10697	14.37958	14.57979	14.17532	0.379352763	-0.382091204	-0.901840528	0.519749324
967	Q8TCT7	SPPL2B	signal peptide peptidase like 2B	56928	ENSG00000005206	PGSPAESEGRDQAQPSPVTQPGASA______	S	0.978431	583	13.39727	12.77246	13.35755	13.43567	13.5243	13.40714	12.34829	13.12051	12.1351	1.291062751	0.641128222	-0.279939334	0.921067556
922	S4R3V8	LSR	lipolysis stimulated lipoprotein receptor	51599	ENSG00000105699	DSSVASVRSGYRIQASQQDDSMRVLYYMEKE;SS	S	0.997829	376	13.37082	13.26774	12.67814	13.78698	13.96335	13.37694	13.58508	13.78984	13.94727	1.219130715	-0.668497403	-0.603524208	-0.064973195
1011	Q96QR8	PURB	purine rich element binding protein B	5814	ENSG00000146676	__________MADGDSGSERGGGGGPCGFQP	S	0.499996	6	13.32601	13.62525	13.29016	13.54774	14.27787	13.32559	13.33804	14.26913	13.31458	0.158068962	-0.226775805	-0.303256671	0.076480865
1012	Q96QR8	PURB	purine rich element binding protein B	5814	ENSG00000146676	________MADGDSGSERGGGGGPCGFQPAS	S	0.499996	8	13.32601	13.62525	13.29016	13.54774	14.27787	13.32559	13.33804	14.26913	13.31458	0.158068962	-0.226775805	-0.303256671	0.076480865
1043	Q9BXB4	OSBPL11	oxysterol binding protein like 11	114885	ENSG00000144909	PPLKSRSFSLASSSNSPISQRRPSQNAISFF	S	0.663488	181	13.32094	13.43424	12.3025	13.12986	13.10903	11.96221	13.05024	13.1493	12.30962	0.075935954	0.18283844	0.285524368	-0.102685928
476	O00161	SNAP23	synaptosome associated protein 23	8773	ENSG00000092531	___________MDNLSSEEIQQRAHQITDES	S	0.875072	5	13.30035	13.69534	13.75008	14.16365	14.16318	14.07498	14.16781	13.42035	13.63764	0.996587948	-0.160013517	-0.552012126	0.391998609
477	O00161	SNAP23	synaptosome associated protein 23	8773	ENSG00000092531	__________MDNLSSEEIQQRAHQITDESL	S	0.5	6	13.30035	13.69534	13.75008	14.16365	14.16318	14.07498	14.16781	13.42035	13.63764	0.996587948	-0.160013517	-0.552012126	0.391998609
1359	P62633	CNBP	CCHC-type zinc finger nucleic acid binding protein	7555	ENSG00000169714	NCYRCGESGHLARECTIEATA__________	T	0.994299	155	13.26731	13.85389	13.23056	13.88655	13.51225	13.49123	13.35824	13.98175	13.87872	0.264638612	-0.288979212	-0.179419835	-0.109559377
267	E7EWE1	UBA5	ubiquitin like modifier activating enzyme 5	79876	ENSG00000081307	SGDGGGGRVRIEKMSSEVVDSNPYSRLMALK	S	0.499918	45	13.26155	14.11399	14.11805	14.02167	14.34589	13.45185	15.02864	14.32172	14.45854	0.828873243	-0.77176857	-0.108611107	-0.663157463
266	E7EWE1	UBA5	ubiquitin like modifier activating enzyme 5	79876	ENSG00000081307	RSGDGGGGRVRIEKMSSEVVDSNPYSRLMAL	S	0.499918	44	13.26155	14.11399	14.11805	14.02167	14.34589	13.45185	15.02864	14.32172	14.45854	0.828873243	-0.77176857	-0.108611107	-0.663157463
1399	Q53TN4	CYBRD1	cytochrome b reductase 1	79901	ENSG00000071967	ARKRNLALDEAGQRSTM______________	T	0.5	227	13.25927	14.05672	13.56736	14.08837	14.34638	13.58238	13.88798	13.0028	13.04234	0.648285299	0.316744804	-0.37792333	0.694668134
1318	O95716	RAB3D	RAB3D, member RAS oncogene family	9545	ENSG00000105514	_________MASAGDTQAGPRDAADQNFDYM	T	0.823201	7	13.2338	12.39312	13.50296	13.17394	12.72709	13.79482	13.40288	13.33874	12.81736	0.051157993	-0.143033663	-0.188659032	0.045625369
68	A0A0C4DFY5	GPRC5C	G protein-coupled receptor class C group 5 member C	55890	ENSG00000170412	KEQKGQSMFVENKAFSMDEPVAAKRPVSPYS	S	1	389	13.22885	13.73005	13.4264	13.71446	13.19496	13.41416	12.79492	14.01777	13.10266	0.051765877	0.156645139	0.020571709	0.13607343
1527	P04279	SEMG1	semenogelin 1	6406	ENSG00000124233	KQQRETKNSHQNKGHYQNVVEVREEHSSKVQ	Y	1	220	13.2148	14.13531	14.2393	14.57637	14.3172	14.27627	14.24733	14.19029	13.5216	0.48322483	-0.123270035	-0.526808103	0.403538068
1261	J3QS47	CYB561	cytochrome b561	1534	ENSG00000008283	SQAEEQALSMDFKTLTEGDSPGSQ_______	T	0.866189	214	13.2086	13.31416	12.98306	13.37762	13.42312	13.76062	13.53867	14.26048	13.66622	1.168671869	-0.653184573	-0.351841927	-0.301342646
544	O75592	MYCBP2	MYC binding protein 2;MYC binding protein 2, E3 ubiquitin protein ligase	23077	ENSG00000005810	AYDGEVLLRSIVKQVSTENDSTLVHRFPLLV	S	0.877354	1624	13.17592	13.01392	13.64937	14.18131	13.33915	14.21826	13.91036	13.58531	13.65855	0.779330479	-0.43833828	-0.633171717	0.194833438
48	G3V578	NDRG2	NDRG family member 2	57447	ENSG00000165795	MASSCMTRLSRSRTASLTSAASVDGNRSRSR	S	0.727016	245	13.16828	13.35879	13.79076	13.16571	14.34402	13.75947	13.11441	13.36044	14.624	0.097426574	-0.260338465	-0.317124049	0.056785583
807	Q14678	KANK1	KN motif and ankyrin repeat domains 1	23189	ENSG00000107104	AASQINVCGVRKRSYSAGNASQLEQLSRARR	S	0.830677	167	13.14641	13.57766	13.1211	13.53418	13.96515	14.02722	13.49773	13.20709	13.62993	1.112831316	-0.163190842	-0.560459455	0.397268613
33	J3QQX0	GPS1	G protein pathway suppressor 1	2873	ENSG00000169727	SARVDSHSKSPPREGSQGELTPANSQSRMST	S	0.998719	62	13.14601	13.10963	13.27826	13.57045	13.33357	11.57861	12.74872	12.99826	13.44979	0.091640491	0.11237971	0.350424767	-0.238045057
146	F6SJA5	ARHGEF7	Rho guanine nucleotide exchange factor 7	8874	ENSG00000102606	LFPNVLLMLSASPRMSGFIYQGKLPTTGMTI	S	0.992216	34	13.07357	13.6562	11.98196	13.45288	13.97835	13.09602	12.70263		12.46987	0.472067481	0.31766208	-0.605175972	0.922838052
634	P12277	CKB	creatine kinase B	1152	ENSG00000166165	__________MPFSNSHNALKLRFPAEDEFP	S	0.572351	6	13.07078	13.62217	13.53041	13.33483	13.47053	13.62217	13.45571	13.03604	13.76942	0.020889554	-0.012608528	-0.068060875	0.055452347
1158	Q9UQB8	BAIAP2	BAR/IMD domain containing adaptor protein 2;BAI1 associated protein 2	10458	ENSG00000175866	SQGLDDYGARSMSSGSGTLVSTV________	S	0.953951	514	13.06861	12.89802	12.97327	13.30521	14.06853	12.97289	13.30592	13.85623	13.30107	0.582824028	-0.507774989	-0.468909899	-0.038865089
1044	Q9BXR0	QTRT1	queuine tRNA-ribosyltransferase catalytic subunit 1	81890	ENSG00000213339	_______MAGAATQASLESAPRIMRLVAECS	S	0.999578	9	13.00512	12.72707	12.77563	13.35397	12.51392	13.12131	12.47657	13.1306	13.33637	0.072430141	-0.145240784	-0.160462697	0.015221914
514	O43520	ATP8B1	ATPase phospholipid transporting 8B1	5205	ENSG00000081923	SAYAFSHQRGYADLISSGRSIRKKRSPLDAI	S	0.499998	1222	13.00475	13.62537	12.39799	13.47028	13.68398	13.59211	13.35204	13.40913	13.23972	0.633071323	-0.324259122	-0.572752635	0.248493512
1332	P11137	MAP2	microtubule associated protein 2	4133	ENSG00000078018	REHAKARVDHGAEIITQSPGRSSVASPRRLS	T	0.537499	424	12.99172	13.54122	13.19909	13.56974	13.22772	12.57762	13.3184	12.59565	12.71802	0.25736232	0.366649628	0.118982315	0.247667313
1102	Q9NSK0	KLC4	kinesin light chain 4	89953	ENSG00000137171	GTEPRPSSSNMKRAASLNYLNQPSAAPLQVS	S	0.999788	513	12.98861	12.7608		12.88969	13.01009	11.83218		12.29198		0.536179596	0.582732677	0.2973876	0.285345078
1283	H3BTQ8	SPINT1	serine peptidase inhibitor, Kunitz type 1	6692	ENSG00000166145	NYLTREVYRSYRQLRTQGFGGSGIPKAWAGI	T	0.994941	154	12.96985	13.71457	13.33064	13.7601	13.3559	13.47206	13.25923	13.18374	13.61551	0.175567115	-0.01447614	-0.190998713	0.176522573
194	C9JHX5	STEAP2	STEAP2 metalloreductase	261729	ENSG00000157214	___________MESISMMGSPKSLSETFLPN	S	0.679505	5	12.94217	13.16828	13.39607	13.56784	12.21778	12.85235	11.93542	13.78515	13.52748	0.055931493	0.086156845	0.289516131	-0.203359286
189	C9JEN3	TMBIM1	transmembrane BAX inhibitor motif containing 1	64114	ENSG00000135926	______________MSNPSAPPPYEDRNPLY	S	0.968659	2	12.90411	12.56655	12.06878	13.81648	12.44755	13.27783	13.09492	12.56274	12.66133	0.496382138	-0.259852091	-0.667472521	0.40762043

943	Q8IZ41	RASEF	RAS and EF-hand domain containing	158158	ENSG00000165105	NSYSKFNRSLHINNISPGNTISRSSPKFIGH	S	0.954809	377	12.89455	13.16341	11.6632	13.38289	12.62507	12.45589	12.81186	11.35017	12.97441	0.109430998	0.194910685	-0.247562726	0.442473412
455	J3KRZ2	DYNC1LI2	dynein cytoplasmic 1 light intermediate chain 2	1783	ENSG00000135720	SFFNSLLSKKTGSPGSPGAGGVQSTAKKSES	S	0.703288	72	12.80846	13.45082	13.28197	13.24216	11.67371	13.49098	13.38734	13.01486	12.74995	0.116503411	0.129701614	0.378138224	-0.24843661
1206	A0A0B4J1Z0	COBLL1	cordon-bleu WH2 repeat protein like 1	22837	ENSG00000082438	QRSKKKRDQTASAPATPLVNKHRPTFTRSNT	T	0.997002	273	12.79999	13.9702	13.50544	13.42062	13.45276	13.85009	13.13558	13.03978	13.29433	0.361394278	0.268644969	-0.149279277	0.417924245
419	H3BSP9	MT2A	metallothionein 2A	4502	ENSG00000125148	PNCSCAAGDSCTCAGSCKCKECKCTSCKKSK	S	0.966926	18	12.6922	13.09446	13.40488	13.96678	13.27218	12.93221	13.15632	13.24771	13.39487	0.230974744	-0.202452342	-0.326544762	0.12409242
355	F8W7D1	CPAMD8	C3 and PZP like alpha-2-macroglobulin domain containing 8	27151	ENSG00000160111	PVNEKLEAYILDPRGSRMIEWRHLKPFCCGI	S	1	173	12.66709	13.2645	12.1809	13.02372	13.84725	13.24687	12.88585	12.1474	13.13316	0.603209736	-0.017969767	-0.668450673	0.650480906
899	Q7L1Q6	BZW1	basic leucine zipper and W2 domains 1	9689	ENSG00000082153	QMKKFVEWLKNAEEESESEAEEGD_______	S	0.982415	345	12.38985	12.3396	11.17673	11.34513	12.85602	12.39425		11.99149		1.104994363	-0.022759755	-0.2297376	0.206977844
439	I3L4C2	BAIAP2	BAI1 associated protein 2;BAR/IMD domain containing adaptor protein 2	10458	ENSG00000175866	SLSPPQSQSKLSDSYSNTLPVRKSVTPKNSY	S	0.562697	371	12.31195	13.04567	12.38179	13.12803	12.59949	13.42535	13.09727	13.26284	12.87665	0.63390775	-0.499120712	-0.471153895	-0.027966817
512	O43246	SLC7A4	solute carrier family 7 member 4	6545	ENSG00000099960	LRFQKSSPPSSPGPASPGPLTKQQSSFSDHL	S	0.682838	427	12.30754	12.82408	13.54122	14.30763	13.38343	12.93413	12.29039	13.59933	13.27497	0.297697897	-0.163951874	-0.650783857	0.486831983
1504	C9J1X3	TNK2	tyrosine kinase non receptor 2	10188	ENSG00000061938	GKTMPTTQSFASDPKYATPQVIQAPGPRAGP	Y	0.909737	859	12.28257	12.03765	12.70438	12.47294	13.42901	11.26796	12.88476		12.39909	0.325629552	-0.300394217	-0.048439662	-0.251954556
234	E5RGH4	HNRNPH1	heterogeneous nuclear ribonucleoprotein H1	3187	ENSG00000169045	NNVEMDWVLKHTGPNSPDTANDGFVRLRGLP	S	0.990332	27	12.19177	13.26281	13.31345	13.65452	13.48872	13.30862	13.13043	13.07456	13.52332	0.514590515	-0.320089658	-0.561277707	0.241188049
1131	Q9P270	SLAIN2	SLAIN motif family member 2	57606	ENSG00000109171	NEQLRSRSGAVQGAGSLGPGSPVRAGASIPS	S	0.999696	43	12.03641	12.39038	12.41798	13.47877	12.54569	12.80858	12.85183	13.18481	12.91696	1.203322732	-0.702939987	-0.662752787	-0.0401872
882	Q6IQ22	RAB12	RAB12, member RAS oncogene family	201475	ENSG00000206418	ALQRRAGGGGGLGAGSPALSGGQGRRRKQPP	S	0.999631	21	11.99788	13.06579	13.47472	13.29921	13.55039	13.40288	12.92424	13.23226	13.74819	0.395885014	-0.45543162	-0.571360588	0.115928968
1215	A0A0U1RQF0	FASN	fatty acid synthase	2194	ENSG00000169710	ELSSKADEASELACPTPKEDGLAQQQTQLNL	T	0.999973	2202	11.88108	13.11594	13.23866	13.24377	13.43006	12.86651	12.90666	12.92323	12.94218	0.260878962	-0.178803762	-0.434887568	0.256083806
1187	Q9Y5S2	CDC42BPB	CDC42 binding protein kinase beta	9578	ENSG00000198752	KHSTPSNSSNPSGPPSPNSPHRSQLPLEGLE	S	0.696917	1690	11.79377	14.01306	13.25099	13.33497	13.36263	13.37205	13.46467	13.47662	13.73407	0.206660707	-0.539182345	-0.337276141	-0.201906204
530	O60343	TBC1D4	TBC1 domain family member 4	9882	ENSG00000136111	QCSDGEGRKRTSSTCSNESLSVGGTSVTPRR	S	0.796832	691	11.73534	12.02635				12.32244	12.52853	11.73034	13.16238	0.559144721	-0.592900594	-0.441587448	-0.151313146
2	A0A087WT53	MAGI1	membrane associated guanylate kinase, WW and PDZ domain containi	9223	ENSG00000151276	NTLERREKHEKRRDVSPERRRERSPTRRRDG	S	1	1043										0	#DIV/0!	#DIV/0!	#DIV/0!
1494	A0A087WU84	OPRK1	opioid receptor kappa 1	4986	ENSG00000082556	RQSTSRVRNTVQDPAYLRDIDGMNKPV____	Y	0.99987	355										0	#DIV/0!	#DIV/0!	#DIV/0!
6	A0A087WUX9	AR	androgen receptor	367	ENSG00000169083	ETSPRQQQQQQGEDGSPQAHRRGPTGYLVLD	S	1	96		11.35926	12.11794	12.08567		13.20324	11.54081	11.53654	11.49265	0.145809659	0.215262254	-0.905860901	1.121123155
51	A0A0A0MSJ8	PPP6R2	protein phosphatase 6 regulatory subunit 2	9701	ENSG00000100239	DFQPFCCSESGPRCSSPVDTECSHAEGSRSQ	S	0.486277	374										0	#DIV/0!	#DIV/0!	#DIV/0!
50	A0A0A0MSJ8	PPP6R2	protein phosphatase 6 regulatory subunit 2	9701	ENSG00000100239	TDFQPFCCSESGPRCSSPVDTECSHAEGSRS	S	0.486277	373										0	#DIV/0!	#DIV/0!	#DIV/0!
56	Q4FZB7	KMT5B	lysine methyltransferase 5B	51111	ENSG00000110066	FATDSGFEILPCNRYSSEQNGAKIVATKEWK	S	0.991974	204										0	#DIV/0!	#DIV/0!	#DIV/0!
1495	Q4FZB7	KMT5B	lysine methyltransferase 5B	51111	ENSG00000110066	MFATDSGFEILPCNRYSSEQNGAKIVATKEW	Y	0.99061	203										0	#DIV/0!	#DIV/0!	#DIV/0!
57	A0A0A0MT60	FKBP15	FKBP prolyl isomerase 15	23307	ENSG00000119321	VRRVKFARDSGSDGHSVSSRDSAAPSPIPGA	S	0.932397	326										0	#DIV/0!	#DIV/0!	#DIV/0!
41	A0A0A0MTJ6	CTNNA2	catenin alpha 2	1496	ENSG00000066032	DSDFEQEDYDVRSRTSVQTEDDQLIAGQSAR	S	0.337714	270										0	#DIV/0!	#DIV/0!	#DIV/0!
1204	A0A0A0MTJ6	CTNNA2	catenin alpha 2	1496	ENSG00000066032	FEQEDYDVRSRTSVQTEDDQLIAGQSARAIM	T	0.944279	273										0	#DIV/0!	#DIV/0!	#DIV/0!
66	A0A0C4DFY5	GPRC5C	G protein-coupled receptor class C group 5 member C	55890	ENSG00000170412	VMGSANSTLRAEDMYSAQSHQAATPPKDGKN	S	0.478526	460										0	#DIV/0!	#DIV/0!	#DIV/0!
77	A0A0C4DG27	HLCS	holocarboxylase synthetase	3141	ENSG00000159267	DDPKALGEEPKQRRGSASGSEPAGDSDRG__	S	0.999924	79										0	#DIV/0!	#DIV/0!	#DIV/0!
80	A0A0C4DGE6	ARHGAP27	Rho GTPase activating protein 27	201176	ENSG00000159314	SIHKSSQDGDTPAQASPPEEKVPAELDEVGS	S	0.994777	98										0	#DIV/0!	#DIV/0!	#DIV/0!
84	A0A0C4DGU2	MMACHC	metabolism of cobalamin associated C	25974	ENSG00000132763	PGNPSRARSWLSPRVSPPASPGP________	S	0.839833	218										0	#DIV/0!	#DIV/0!	#DIV/0!
1213	A0A0G2JM79	NOSTRIN	nitric oxide synthase trafficking	115677	ENSG00000275326	VSKASSGGQSNPGSSTPAPGAAQLSSRLCKA	T	0.451049	316										0	#DIV/0!	#DIV/0!	#DIV/0!
92	A0A0G2JM79	NOSTRIN	nitric oxide synthase trafficking	115677	ENSG00000275326	IVSKASSGGQSNPGSSTPAPGAAQLSSRLCK	S	0.451049	315										0	#DIV/0!	#DIV/0!	#DIV/0!
100	A0A0J9YW81	KCNN2	potassium calcium-activated channel subfamily N member 2	3781	ENSG00000080709	RRRSSSTAPPTSSESS______________	S	0.497725	582										0	#DIV/0!	#DIV/0!	#DIV/0!
99	A0A0J9YW81	KCNN2	potassium calcium-activated channel subfamily N member 2	3781	ENSG00000080709	RRRRSSSTAPPTSSESS_____________	S	0.497725	581										0	#DIV/0!	#DIV/0!	#DIV/0!
98	A0A0J9YW81	KCNN2	potassium calcium-activated channel subfamily N member 2	3781	ENSG00000080709	SSRRRRSSSTAPPTSSESS___________	S	0.387762	579										0	#DIV/0!	#DIV/0!	#DIV/0!
25	A0A0U1RQT1	ACAP2	ArfGAP with coiled-coil, ankyrin repeat and PH domains 2	23527	ENSG00000114331	YREKGDESEKLDKKSSPSTGSLDSGNESKEK	S	0.499644	386										0	#DIV/0!	#DIV/0!	#DIV/0!
24	A0A0U1RQT1	ACAP2	ArfGAP with coiled-coil, ankyrin repeat and PH domains 2	23527	ENSG00000114331	AYREKGDESEKLDKKSSPSTGSLDSGNESKE	S	0.499644	385										0	#DIV/0!	#DIV/0!	#DIV/0!
106	A0A0U1RR07	SYTL2	synaptotagmin like 2	54843	ENSG00000137501	LKARGAPRGILKRNSSSSSTDSETLRYNHNF	S	0.953567	279										0	#DIV/0!	#DIV/0!	#DIV/0!
110	A0A1B0GTW1	TJP2	tight junction protein 2	9414	ENSG00000119139	RKPSPEPRAQMRRAASSDQLRDNSPPPAFKP	S	0.810354	1037										0	#DIV/0!	#DIV/0!	#DIV/0!
111	A0A1B0GTW1	TJP2	tight junction protein 2	9414	ENSG00000119139	RGRPHERARSRERDLSRDRSRGRSLERGLDQ	S	1	251										0	#DIV/0!	#DIV/0!	#DIV/0!
116	A0A1B0GTW1	TJP2	tight junction protein 2	9414	ENSG00000119139	SRGPRSRSREHPHSRSPSPEPRGRPGPIGVL	S	0.99842	353										0	#DIV/0!	#DIV/0!	#DIV/0!
117	A0A1B0GTW1	TJP2	tight junction protein 2	9414	ENSG00000119139	GPRSRSREHPHSRSPSPEPRGRPGPIGVLLM	S	0.999865	355										0	#DIV/0!	#DIV/0!	#DIV/0!
125	A0A286YFF8	MON2	MON2 homolog, regulator of endosome-to-Golgi trafficking	23041	ENSG00000061987	__________MSGTSSPEAVKKLLENMQSDL	S	0.486837	6										0	#DIV/0!	#DIV/0!	#DIV/0!
15	A0A2R8Y695	IQSEC1	IQ motif and Sec7 domain 1	9922	ENSG00000144711	AFRQYQMNKNFERLRSSMSENRMSRRIVLSN	S	0.492715	163										0	#DIV/0!	#DIV/0!	#DIV/0!
142	A0A2R8YDT1	GLUL	glutamate-ammonia ligase	2752	ENSG00000135821	PACSDTPRAGRCLSPSPSPSPRKLRCLDQLE	S	0.489999	84										0	#DIV/0!	#DIV/0!	#DIV/0!
141	A0A2R8YDT1	GLUL	glutamate-ammonia ligase	2752	ENSG00000135821	HQPACSDTPRAGRCLSPSPSPSPRKLRCLDQ	S	0.489999	82										0	#DIV/0!	#DIV/0!	#DIV/0!
1205	A2SY06	MARK3	microtubule affinity regulating kinase 3	4140	ENSG00000075413	TPDRIRFPRGTASRSTFHGQPRERRTATYNG	T	0.499027	151										0	#DIV/0!	#DIV/0!	#DIV/0!
55	A2SY06	MARK3	microtubule affinity regulating kinase 3	4140	ENSG00000075413	ATPDRIRFPRGTASRSTFHGQPRERRTATYN	S	0.499027	150										0	#DIV/0!	#DIV/0!	#DIV/0!
129	A6NNI4	CD9	CD9 molecule	928	ENSG00000010278	DICPKKDVLETFTVKSCPDAIKEVFDNKFHI	S	1	111										0	#DIV/0!	#DIV/0!	#DIV/0!
1233	B0QY71	MAFF	MAF bZIP transcription factor F	23764	ENSG00000185022	RELNRHLRGLSAEEVTRLKQRRRTLKNRGYA	T	0.997506	21										0	#DIV/0!	#DIV/0!	#DIV/0!
1240	B5MC48	CLHC1	clathrin heavy chain linker domain containing 1	130162	ENSG00000162994	RSDKEFLESVQRYIITETERLGCSEEGPA__	T	0.997886	33										0	#DIV/0!	#DIV/0!	#DIV/0!
1501	B5MC48	CLHC1	clathrin heavy chain linker domain containing 1	130162	ENSG00000162994	IICRSDKEFLESVQRYIITETERLGCSEEGP	Y	0.998355	30										0	#DIV/0!	#DIV/0!	#DIV/0!
202	B8ZZI9	AFTPH	aftiphilin	54812	ENSG00000119844	QTSDNLSEECQLARKSSGTGTEPVAKLKNGQ	S	0.471178	172										0	#DIV/0!	#DIV/0!	#DIV/0!
203	B8ZZI9	AFTPH	aftiphilin	54812	ENSG00000119844	TSDNLSEECQLARKSSGTGTEPVAKLKNGQE	S	0.471178	173										0	#DIV/0!	#DIV/0!	#DIV/0!
205	B8ZZT4	VAMP8	vesicle associated membrane protein 8	8673	ENSG00000118640	___________MEEASEGGGNDRVRNLQSEV	S	1	5		11.41785		11.62013	11.72988		12.5555	12.08345	11.36976	0.583363073	-0.585049947	-0.257149696	-0.327900251
207	B9DI82	PLCH2	phospholipase C eta 2	9651	ENSG00000149527	ESKIRDCEDPNNFSVSTLSPSGKLGRKSKAE	S	0.419432	401										0	#DIV/0!	#DIV/0!	#DIV/0!
1241	B9DI82	PLCH2	phospholipase C eta 2	9651	ENSG00000149527	SKIRDCEDPNNFSVSTLSPSGKLGRKSKAEE	T	0.419432	402										0	#DIV/0!	#DIV/0!	#DIV/0!
28	C9IYE1	CADPS2	calcium dependent secretion activator 2	93664	ENSG00000081803	RAGGGGAARSVSPSPSVLSEGRDEPQRQLDD	S	0.412766	62										0	#DIV/0!	#DIV/0!	#DIV/0!
164	C9IZZ8	ITGB1BP1	integrin subunit beta 1 binding protein 1	9270	ENSG00000119185	VDSSLGGLSRSSTVASLDTDSTKSSGQSNNN	S	0.939543	41										0	#DIV/0!	#DIV/0!	#DIV/0!
162	C9IZZ8	ITGB1BP1	integrin subunit beta 1 binding protein 1	9270	ENSG00000119185	TKSKSVDSSLGGLSRSSTVASLDTDSTKSSG	S	0.327913	36										0	#DIV/0!	#DIV/0!	#DIV/0!
163	C9IZZ8	ITGB1BP1	integrin subunit beta 1 binding protein 1	9270	ENSG00000119185	KSKSVDSSLGGLSRSSTVASLDTDSTKSSGQ	S	0.327913	37										0	#DIV/0!	#DIV/0!	#DIV/0!
1230	C9IZZ8	ITGB1BP1	integrin subunit beta 1 binding protein 1	9270	ENSG00000119185	SKSVDSSLGGLSRSSTVASLDTDSTKSSGQS	T	0.754689	38										0	#DIV/0!	#DIV/0!	#DIV/0!
209	C9J235	RUFY4	RUN and FYVE domain containing 4	285180	ENSG00000188282	LEKKGESSRKHRYPQSMWEPEGKELQLDQEE	S	0.999204	284										0	#DIV/0!	#DIV/0!	#DIV/0!
211	C9J6P4	ZC3HAV1	zinc finger CCCH-type containing, antiviral 1	56829	ENSG00000105939	HLCKLNLLGRCNYSQSERNLCKYSHEVLSEE	S	0.992147	101										0	#DIV/0!	#DIV/0!	#DIV/0!
1242	C9JKZ3	TMPRSS2	transmembrane serine protease 2	7113	ENSG00000184012	QYYPSPVPQYAPRVLTQASNPVVCTQPKSPS	T	0.993053	58		12.61717	11.52052	12.27132	12.38769	13.37082	12.78179	12.29864		0.234851161	-0.471367836	-0.607767105	0.136399269
76	C9JXI9	KRIT1	KRIT1 ankyrin repeat containing	889	ENSG00000001631	IEDAYVAVIRPKNTASLNSREYRAKSYE___	S	0.852603	22										0	#DIV/0!	#DIV/0!	#DIV/0!
232	D6R8Z7	TSC22D3	TSC22 domain family member 3	1831	ENSG00000157514	CCLDLAHRSGLQRGSSGENNNPGSPTVSNFR	S	0.497807	34										0	#DIV/0!	#DIV/0!	#DIV/0!
231	D6R8Z7	TSC22D3	TSC22 domain family member 3	1831	ENSG00000157514	NCCLDLAHRSGLQRGSSGENNNPGSPTVSNF	S	0.497807	33										0	#DIV/0!	#DIV/0!	#DIV/0!
1244	D6RCK3	MOB1B	MOB kinase activator 1B	92597	ENSG00000173542	PEGSHQYELLKHAEATLGSGNLRMAVMLPEG	T	0.793607	40										0	#DIV/0!	#DIV/0!	#DIV/0!
251	E5RJY1	NDRG1	N-myc downstream regulated 1	10397	ENSG00000104419	GMGYMPSASMTRLMRSRTASGSSVTSLDGTR	S	0.1999	35										0	#DIV/0!	#DIV/0!	#DIV/0!
1247	E5RJY1	NDRG1	N-myc downstream regulated 1	10397	ENSG00000104419	RSRTASGSSVTSLDGTRSRSHTSEGTRSRSH	T	0.714384	49										0	#DIV/0!	#DIV/0!	#DIV/0!
47	E7EMV7	TNIP1	TNFAIP3 interacting protein 1	10318	ENSG00000145901	KQQVKIFEEDFQRERSDRERMNEEKEELKKQ	S	1	425										0	#DIV/0!	#DIV/0!	#DIV/0!
261	E7EMZ9	TACC2	transforming acidic coiled-coil containing protein 2	10579	ENSG00000138162	SLLVLQGDSGKRSSDSEEAFETPESTTPVKA;VGC	S	0.444141	1904										0	#DIV/0!	#DIV/0!	#DIV/0!
269	E7EQB2	LTF	lactotransferrin	4057	ENSG00000012223	_____MGLCLAGRRRSVQWCAVSQPEATKCF	S	0.999791	11										0	#DIV/0!	#DIV/0!	#DIV/0!
276	E7EX17	EIF4B	eukaryotic translation initiation factor 4B	1975	ENSG00000063046	MPAPPPKENAWVKRSSNPPARSQSSDTEQQS	S	0.754031	494										0	#DIV/0!	#DIV/0!	#DIV/0!
292	E9PGB3	TACC2	transforming acidic coiled-coil containing protein 2	10579	ENSG00000138162	SQESPVKSSPVRMSESPTPCS__________	S	0.839512	664										0	#DIV/0!	#DIV/0!	#DIV/0!
310	F2Z305	DCAF6	DDB1 and CUL4 associated factor 6	55827	ENSG00000143164	LFDPKDDTARELKTPSAEERREELRQPPVKR	S	0.994256	294										0	#DIV/0!	#DIV/0!	#DIV/0!
34	F2Z357	RAP1GAP	RAP1 GTPase activating protein	5909	ENSG00000076864	GHVSQEPKSENSSTQSSPEMPTTKNRAETAA	S	0.467523	541										0	#DIV/0!	#DIV/0!	#DIV/0!
304	F5GXG5	EXPH5	exophilin 5	23086	ENSG00000110723	VEDAMGNYMLNKFSPSSPESANECSKVLSDS	S	0.43934	885										0	#DIV/0!	#DIV/0!	#DIV/0!
305	F5GXG5	EXPH5	exophilin 5	23086	ENSG00000110723	EDAMGNYMLNKFSPSSPESANECSKVLSDSA	S	0.43934	886										0	#DIV/0!	#DIV/0!	#DIV/0!
319	F5GYU8	TMC5	transmembrane channel like 5	79838	ENSG00000103534	QPDYPGSQRNPDFAGSSSSGNYAGSRTHPDH	S	0.778725	140										0	#DIV/0!	#DIV/0!	#DIV/0!
1254	F5GYU8	TMC5	transmembrane channel like 5	79838	ENSG00000103534	EPNYPRSLSNPDYSGTRSNAYSAASRTSPDH	T	0.49664	86										0	#DIV/0!	#DIV/0!	#DIV/0!
326	F5GYU8	TMC5	transmembrane channel like 5	79838	ENSG00000103534	SSETPKMTRGVLSRTSSIQPSFRHRSDDPVG	S	0.494779	269										0	#DIV/0!	#DIV/0!	#DIV/0!
324	F5GYU8	TMC5	transmembrane channel like 5	79838	ENSG00000103534	LAEPNYPRSLSNPDYSGTRSNAYSAASRTSP	S	0.49664	84										0	#DIV/0!	#DIV/0!	#DIV/0!
328	F5GZK9	DCP1B	decapping mRNA 1B	196513	ENSG00000151065	QGVVRSLSYEEPRRHSPPIEKQLCPAIQKLM	S	1	157										0	#DIV/0!	#DIV/0!	#DIV/0!
329	F5GZR9	SLC3A2	solute carrier family 3 member 2	6520	ENSG00000168003	______________MSQDTEVDMKEAAAAAK	S	0.9868	2										0	#DIV/0!	#DIV/0!	#DIV/0!
337	F5H442	TSG101	tumor susceptibility 101	7251	ENSG00000074319	QPPVTTVGPSRDGTISEDTIRASLISAVSDK	S	0.87135	222										0	#DIV/0!	#DIV/0!	#DIV/0!

347	F5H858	PHACTR2	phosphatase and actin regulator 2	9749	ENSG00000112419	TGSKASASPSTSSTSSRPKASKETVSSKAGT	S	0.427183	120	0	#DIV/0!	#DIV/0!	#DIV/0!
351	F8VWT9	HECTD4	HECT domain E3 ubiquitin protein ligase 4	283450	ENSG00000173064	LLLHVTIAAQSGLTRSISGTPAETPACKSAS	S	0.496136	1361	0	#DIV/0!	#DIV/0!	#DIV/0!
238	F8W6W8	RIMS2	regulating synaptic membrane exocytosis 2	9699	ENSG00000176406	LSRSHPRTGSVQTSPSSTPVAGRRGRQLPQL	S	0.41481	902	0	#DIV/0!	#DIV/0!	#DIV/0!
239	F8W6W8	RIMS2	regulating synaptic membrane exocytosis 2	9699	ENSG00000176406	SRSHPRTGSVQTSPSSTPVAGRRGRQLPQLP	S	0.41481	903	0	#DIV/0!	#DIV/0!	#DIV/0!
1267	F8W912	CCDC125	coiled-coil domain containing 125	202243	ENSG00000183323	FPQVSRISNYRRQSSTDSNSELSNEELRQCL	T	0.310396	101	0	#DIV/0!	#DIV/0!	#DIV/0!
358	F8W912	CCDC125	coiled-coil domain containing 125	202243	ENSG00000183323	DTFPQVSRISNYRRQSSTDSNSELSNEELRQ	S	0.489659	99	0	#DIV/0!	#DIV/0!	#DIV/0!
359	F8W912	CCDC125	coiled-coil domain containing 125	202243	ENSG00000183323	TFPQVSRISNYRRQSSTDSNSELSNEELRQC	S	0.489659	100	0	#DIV/0!	#DIV/0!	#DIV/0!
367	F8WAN1	SPECC1L-ADORA2A	SPECC1L-ADORA2A readthrough (NMD candidate)	101730217	ENSG00000258555	AALRQGMGLSRRSSTSSEPTPTVKTLIKSFD	S	0.222494	834	0	#DIV/0!	#DIV/0!	#DIV/0!
1268	F8WAN1	SPECC1L-ADORA2A	SPECC1L-ADORA2A readthrough (NMD candidate)	101730217	ENSG00000258555	LAALRQGMGLSRRSSTSSEPTPTVKTLIKSF	T	0.222494	833	0	#DIV/0!	#DIV/0!	#DIV/0!
368	F8WAN1	SPECC1L-ADORA2A	SPECC1L-ADORA2A readthrough (NMD candidate)	101730217	ENSG00000258555	ALRQGMGLSRRSSTSSEPTPTVKTLIKSFDS	S	0.222494	835	0	#DIV/0!	#DIV/0!	#DIV/0!
1514	F8WD55	TRPM8	transient receptor potential cation channel subfamily M member 8	79054	ENSG00000144481	MRNRRNDTLDSTRTLYSSASRSTDLSYSESD	Y	0.971573	25	0	#DIV/0!	#DIV/0!	#DIV/0!
374	G3V1D1	FTH1	ferritin heavy chain 1	2495	ENSG00000167996	SGLAEYLFDKHTLGDSDNES___________	S	0.987148	109	0	#DIV/0!	#DIV/0!	#DIV/0!
378	G5E9C7	MAP2K2	mitogen-activated protein kinase kinase 2	5605	ENSG00000126934	GQLIDSMANSFVGTRSYMAPERLQGTHYSVQ	S	0.996305	135	0	#DIV/0!	#DIV/0!	#DIV/0!
379	H0Y332	STXBP5	syntaxin binding protein 5	134957	ENSG00000164506	IELYGSNDPYRREPRSPRKSRQPSGAGLCDI	S	1	26	0	#DIV/0!	#DIV/0!	#DIV/0!
382	H0Y572	DOCK9	dedicator of cytokinesis 9	23348	ENSG00000088387	ARNQEGLGPIVHDRKSQTLPVSRNRTGMMHA	S	0.932576	42	0	#DIV/0!	#DIV/0!	#DIV/0!
183	H0Y5J4	DBNL	drebrin like	28988	ENSG00000136279	EIFKQKERAMSTTSISSPQPGKLRSPFLQKQ	S	0.480062	203	0	#DIV/0!	#DIV/0!	#DIV/0!
1238	H0Y5J4	DBNL	drebrin like	28988	ENSG00000136279	HPREIFKQKERAMSTTSISSPQPGKLRSPFL	T	0.449558	200	0	#DIV/0!	#DIV/0!	#DIV/0!
1239	H0Y5J4	DBNL	drebrin like	28988	ENSG00000136279	PQPGKLRSPFLQKQLTQPETHFGREPAAAIS	T	0.99986	220	0	#DIV/0!	#DIV/0!	#DIV/0!
1515	H0Y5N2	SMPD2	sphingomyelin phosphodiesterase 2	6610	ENSG00000135587	GVRIDYVLYKAVSGFYISCKSFETTTGFDPH	Y	0.952354	146	0	#DIV/0!	#DIV/0!	#DIV/0!
364	H0Y5T1	CLASP1	cytoplasmic linker associated protein 1	23332	ENSG00000074054	GSYASLGRIRTRRQSSGSATNVASTPDNRGR	S	0.319647	428	0	#DIV/0!	#DIV/0!	#DIV/0!
365	H0Y5T1	CLASP1	cytoplasmic linker associated protein 1	23332	ENSG00000074054	YASLGRIRTRRQSSGSATNVASTPDNRGRSR	S	0.319647	430	0	#DIV/0!	#DIV/0!	#DIV/0!
1516	H0Y767	SBF2	SET binding factor 2	81846	ENSG00000133812	ETTFYNAVQEQVRSLYLSAKEDNHAPHLKQK	Y	0.994416	292	0	#DIV/0!	#DIV/0!	#DIV/0!
386	H0Y767	SBF2	SET binding factor 2	81846	ENSG00000133812	FWETTFYNAVQEQVRSLYLSAKEDNHAPHLK	S	0.989871	290	0	#DIV/0!	#DIV/0!	#DIV/0!
1517	H0Y8H0	DDX60L	DExD/H-box 60 like	91351	ENSG00000181381	DELLHWHAQRLLSDDYDRIKCHVDEQSRDPH	Y	1	225	0	#DIV/0!	#DIV/0!	#DIV/0!
388	H0Y8H0	DDX60L	DExD/H-box 60 like	91351	ENSG00000181381	KTSDELLHWHAQRLLSDDYDRIKCHVDEQSR	S	1	222	0	#DIV/0!	#DIV/0!	#DIV/0!
1211	H0YA91	CAST	calpastatin	831	ENSG00000153113	EKEESTEVLKAQSAGTVRSAAPPQEKKRKVE	T	0.999378	284	0	#DIV/0!	#DIV/0!	#DIV/0!
392	H0YBE8	WWC1	WW and C2 domain containing 1	23286	ENSG00000113645	ALRSLSGTPKSMTSLSPRSSLSSPSPPCSPL	S	0.855306	312	0	#DIV/0!	#DIV/0!	#DIV/0!
391	H0YBE8	WWC1	WW and C2 domain containing 1	23286	ENSG00000113645	QSQYVCRLNRSDSDSSTLSKKPPFVRNSLER	S	0.444988	727	0	#DIV/0!	#DIV/0!	#DIV/0!
390	H0YBE8	WWC1	WW and C2 domain containing 1	23286	ENSG00000113645	PQSQYVCRLNRSDSDSSTLSKKPPFVRNSLE	S	0.444988	726	0	#DIV/0!	#DIV/0!	#DIV/0!
1275	H0YE29	ARHGAP1	Rho GTPase activating protein 1	392	ENSG00000175220	AHALTTEGIFRRSANTQVVREVQQKYNMGLP	T	0.995223	241	0	#DIV/0!	#DIV/0!	#DIV/0!
1278	H0YLS6	PSMA4	proteasome subunit alpha 4	5685	ENSG00000041357	______MSRRYDSRTTIFSPEGRLYQVEYAM	T	0.80756	10	0	#DIV/0!	#DIV/0!	#DIV/0!
1279	H0YNB1	HERC1	HECT and RLD domain containing E3 ubiquitin protein ligase family mer	8925	ENSG00000103657	SASEGGGLMTRSESLTAESRLVHTSPNYRLI	T	0.895665	477	0	#DIV/0!	#DIV/0!	#DIV/0!
407	H3BMF5	SLC9A3R2	SLC9A3 regulator 2	9351	ENSG00000065054	PWEPKPDWAHTGSHSSEAGKKDVSGPLRELR	S	0.25	124	0	#DIV/0!	#DIV/0!	#DIV/0!
406	H3BMF5	SLC9A3R2	SLC9A3 regulator 2	9351	ENSG00000065054	DPWEPKPDWAHTGSHSSEAGKKDVSGPLREL	S	0.25	123	0	#DIV/0!	#DIV/0!	#DIV/0!
405	H3BMF5	SLC9A3R2	SLC9A3 regulator 2	9351	ENSG00000065054	AHDPWEPKPDWAHTGSHSSEAGKKDVSGPLR	S	0.25	121	0	#DIV/0!	#DIV/0!	#DIV/0!
1280	H3BMF5	SLC9A3R2	SLC9A3 regulator 2	9351	ENSG00000065054	PPAHDPWEPKPDWAHTGSHSSEAGKKDVSGP	T	0.25	119	0	#DIV/0!	#DIV/0!	#DIV/0!
137	H3BS66	SMIM1	small integral membrane protein 1 (Vel blood group)	388588	ENSG00000235169	RDGVSLGAVSSTEEASRCRR___________	S	0.456203	33	0	#DIV/0!	#DIV/0!	#DIV/0!
411	H3BTK3	CARHSP1	calcium regulated heat stable protein 1	23589	ENSG00000153048	PPTHQASVGLLDTPRSRERSPSPLRGNVVPS	S	0.993691	26	0	#DIV/0!	#DIV/0!	#DIV/0!
410	H3BTK3	CARHSP1	calcium regulated heat stable protein 1	23589	ENSG00000153048	LPTRRTRTFSATVRASQGPVYKGVCKCFC__	S	0.999998	58	0	#DIV/0!	#DIV/0!	#DIV/0!
412	H3BTK3	CARHSP1	calcium regulated heat stable protein 1	23589	ENSG00000153048	SVGLLDTPRSRERSPSPLRGNVVPSPLPTRR	S	0.753152	32	0	#DIV/0!	#DIV/0!	#DIV/0!
414	H3BUH7	ALDOA	aldolase, fructose-bisphosphate A	226	ENSG00000149925	HRIVAPGKGILAADESTGSIAKRLQSIGTEN	S	0.458657	36	0	#DIV/0!	#DIV/0!	#DIV/0!
1282	H3BUH7	ALDOA	aldolase, fructose-bisphosphate A	226	ENSG00000149925	RIVAPGKGILAADESTGSIAKRLQSIGTENT	T	0.458657	37	0	#DIV/0!	#DIV/0!	#DIV/0!
1285	H7C1F9	RALGAPA2	Ral GTPase activating protein catalytic alpha subunit 2	57186	ENSG00000188559	RSHPDVTEPMRFRSATTSGAPGVEKARNIVR	T	0.725318	532	0	#DIV/0!	#DIV/0!	#DIV/0!
426	H7C1W4	AC022384.1	novel protein	NA	ENSG00000272410	EGWSQNSRSFRFSRSSEEREVKEKRTFQEEM	S	1	397	0	#DIV/0!	#DIV/0!	#DIV/0!
424	H7C1W4	AC022384.1	novel protein	NA	ENSG00000272410	ASEEGWSQNSRSFRFSRSSEEREVKEKRTFQ	S	1	394	0	#DIV/0!	#DIV/0!	#DIV/0!
425	H7C1W4	AC022384.1	novel protein	NA	ENSG00000272410	EEGWSQNSRSFRFSRSSEEREVKEKRTFQEE	S	1	396	0	#DIV/0!	#DIV/0!	#DIV/0!
432	I3L202	ZDHHC1	zinc finger DHHC-type containing 1	29800	ENSG00000159714	AESVDEIPVAQTRLGSAALAAPRGRGRQPTL	S	1	307	0	#DIV/0!	#DIV/0!	#DIV/0!
1287	I3L4Z8	PCTP	phosphatidylcholine transfer protein	58488	ENSG00000141179	LDMEGRKIHVILARSTSMPQLGERSGVIRVK	T	0.364229	21	0	#DIV/0!	#DIV/0!	#DIV/0!
433	I3L4Z8	PCTP	phosphatidylcholine transfer protein	58488	ENSG00000141179	DLDMEGRKIHVILARSTSMPQLGERSGVIRV	S	0.364229	20	0	#DIV/0!	#DIV/0!	#DIV/0!
448	J3KPH8	HDAC7	histone deacetylase 7	51564	ENSG00000061273	KKSLERRKNPLLRKESAPPSLRRRPAETLGD	S	0.971662	237	0	#DIV/0!	#DIV/0!	#DIV/0!
1291	J3KRL0	MYO1D	myosin ID	4642	ENSG00000176658	IVTRINDIIEVKNYDTTIHGKNTVIGVLDIY	T	0.871506	370	0	#DIV/0!	#DIV/0!	#DIV/0!
454	J3KRZ2	DYNC1LI2	dynein cytoplasmic 1 light intermediate chain 2	1783	ENSG00000135720	PRTQGRGGPASVPSSSPGTSVKKPDPNIKNN	S	0.44434	33	0	#DIV/0!	#DIV/0!	#DIV/0!
453	J3KRZ2	DYNC1LI2	dynein cytoplasmic 1 light intermediate chain 2	1783	ENSG00000135720	SPRTQGRGGPASVPSSSPGTSVKKPDPNIKN	S	0.44434	32	0	#DIV/0!	#DIV/0!	#DIV/0!
456	J3KS54	FLII	FLII actin remodeling protein	2314	ENSG00000177731	KDPMARKMRLRRRKDSAQDDQAKQVLKGMSD	S	1	435	0	#DIV/0!	#DIV/0!	#DIV/0!
457	J3KSJ8	PPP1R1B	protein phosphatase 1 regulatory inhibitor subunit 1B	84152	ENSG00000131771	EHSSPEEEASPHQRASGEGHHLKSKRPNPCA	S	1	55	0	#DIV/0!	#DIV/0!	#DIV/0!
460	J3QQQ0	LIPG	lipase G, endothelial type	9388	ENSG00000101670	YLKTRAGMPFRGNLQSLECP___________	S	1	386	0	#DIV/0!	#DIV/0!	#DIV/0!
1214	K7EKH8	ERC1	ELKS/RAB6-interacting/CAST family member 1	23085	ENSG00000082805	RSPRLPRSPRLGHRRTNSTGGSSGSSVGGGS	T	0.269615	35	0	#DIV/0!	#DIV/0!	#DIV/0!
468	K7EMD6	SGTA	small glutamine rich tetratricopeptide repeat containing alpha	6449	ENSG00000104969	FEAAATGKEMPQDLRSPARTPPSEEDSAEAE	S	0.879964	77	0	#DIV/0!	#DIV/0!	#DIV/0!
1307	O14545	TRAFD1	TRAF-type zinc finger domain containing 1	10906	ENSG00000135148	HVTEGIPRLDSQPQETSPELPRRRVRHQGDL	T	0.499885	414	0	#DIV/0!	#DIV/0!	#DIV/0!
437	O14641	DVL2	dishevelled segment polarity protein 2	1856	ENSG00000004975	RTGRPEERAPESKSGSGSESEPSSRGGSLRR	S	0.939022	618	0	#DIV/0!	#DIV/0!	#DIV/0!
492	O14745	SLC9A3R1	SLC9A3 regulator 1	9368	ENSG00000109062	LAEAALESPRPALVRSASSDTSEELNSQDSP	S	0.414211	288	0	#DIV/0!	#DIV/0!	#DIV/0!
493	O14745	SLC9A3R1	SLC9A3 regulator 1	9368	ENSG00000109062	EAALESPRPALVRSASSDTSEELNSQDSPPK	S	0.414211	290	0	#DIV/0!	#DIV/0!	#DIV/0!
495	O14863	SLC30A4	solute carrier family 30 member 4	7782	ENSG00000104154	YRQEVDRTCANCQSSSP______________	S	0.91611	428	0	#DIV/0!	#DIV/0!	#DIV/0!
499	O15037	KHNYN	KH and NYN domain containing	23351	ENSG00000100441	PGEEAWEREVALRPQSVGGGARESAPLKGKA	S	1	291	0	#DIV/0!	#DIV/0!	#DIV/0!
510	O43246	SLC7A4	solute carrier family 7 member 4	6545	ENSG00000099960	ATSIIVLRFQKSSPPSSPGPASPGPLTKQQS	S	0.480636	421	0	#DIV/0!	#DIV/0!	#DIV/0!
511	O43246	SLC7A4	solute carrier family 7 member 4	6545	ENSG00000099960	TSIIVLRFQKSSPPSSPGPASPGPLTKQQSS	S	0.480636	422	0	#DIV/0!	#DIV/0!	#DIV/0!
529	O60343	TBC1D4	TBC1 domain family member 4	9882	ENSG00000136111	DGRAQGVRSPLLRQSSSEQCSDGEGRKRTSS	S	0.823787	673	0	#DIV/0!	#DIV/0!	#DIV/0!
525	O60343	TBC1D4	TBC1 domain family member 4	9882	ENSG00000136111	KRKLNLQDGRAQGVRSPLLRQSSSEQCSDGE	S	1	666	0	#DIV/0!	#DIV/0!	#DIV/0!
1313	O60675	MAFK	MAF bZIP transcription factor K	7975	ENSG00000198517	ARTVARGPVAPSKVATTSVITIVKSTELSST	T	0.5	133	0	#DIV/0!	#DIV/0!	#DIV/0!
532	O60675	MAFK	MAF bZIP transcription factor K	7975	ENSG00000198517	TVARGPVAPSKVATTSVITIVKSTELSSTSV	S	0.5	135	0	#DIV/0!	#DIV/0!	#DIV/0!
1315	O60675	MAFK	MAF bZIP transcription factor K	7975	ENSG00000198517	RGPVAPSKVATTSVITIVKSTELSSTSVPFS	T	0.5	138	0	#DIV/0!	#DIV/0!	#DIV/0!
1314	O60675	MAFK	MAF bZIP transcription factor K	7975	ENSG00000198517	RTVARGPVAPSKVATTSVITIVKSTELSSTS	T	0.5	134	0	#DIV/0!	#DIV/0!	#DIV/0!
539	O75351	VPS4B	vacuolar protein sorting 4 homolog B	9525	ENSG00000119541	_______________MSSTSPNLQKAIDLASK	S	0.31345	2	0	#DIV/0!	#DIV/0!	#DIV/0!
541	O75351	VPS4B	vacuolar protein sorting 4 homolog B	9525	ENSG00000119541	____________MSSTSPNLQKAIDLASKAAQ	S	0.25	5	0	#DIV/0!	#DIV/0!	#DIV/0!
540	O75351	VPS4B	vacuolar protein sorting 4 homolog B	9525	ENSG00000119541	______________MSSTSPNLQKAIDLASKA	S	0.31345	3	0	#DIV/0!	#DIV/0!	#DIV/0!
1316	O75351	VPS4B	vacuolar protein sorting 4 homolog B	9525	ENSG00000119541	_____________MSSTSPNLQKAIDLASKAA	T	0.31345	4	0	#DIV/0!	#DIV/0!	#DIV/0!
543	O75494	SRSF10	serine and arginine rich splicing factor 10	10772	ENSG00000188529	QSRSQSRSRSKSRSRSWTSPKSSGH______	S	0.525546	252	0	#DIV/0!	#DIV/0!	#DIV/0!
542	O75494	SRSF10	serine and arginine rich splicing factor 10	10772	ENSG00000188529	KSQSRSQSRSRSKSRSRSWTSPKSSGH____	S	0.896844	250	0	#DIV/0!	#DIV/0!	#DIV/0!
545	O75607	NPM3	nucleophosmin/nucleoplasmin 3	10360	ENSG00000107833	LDDFQLQPPVTFRLKSGSGPVRITGRHQIVT	S	0.911181	126	0	#DIV/0!	#DIV/0!	#DIV/0!
546	O75607	NPM3	nucleophosmin/nucleoplasmin 3	10360	ENSG00000107833	DFQLQPPVTFRLKSGSGPVRITGRHQIVTMS	S	0.909929	128	0	#DIV/0!	#DIV/0!	#DIV/0!
1317	O75874	IDH1	isocitrate dehydrogenase (NADP(+)) 1, cytosolic;isocitrate dehydrogenas	3417	ENSG00000138413	VCPDGKTVEAEAAHGTVTRHYRMYQKGQETS	T	0.847453	311	0	#DIV/0!	#DIV/0!	#DIV/0!
551	O76083	PDE9A	phosphodiesterase 9A	5152	ENSG00000160191	KRIDDAMKELQKKTDSLTSGATEKSRERSRD	S	0.852866	352	0	#DIV/0!	#DIV/0!	#DIV/0!
554	O94819	KBTBD11	kelch repeat and BTB domain containing 11	9920	ENSG00000273645	HLLAAALGPAGERAGSRPQSPSGDADARGDA	S	0.999049	310	0	#DIV/0!	#DIV/0!	#DIV/0!
557	O94875	SORBS2	sorbin and SH3 domain containing 2	8470	ENSG00000154556	SSTTLTKSFTSSSPSSPSRAKGGDDSKICPS	S	0.80701	402	0	#DIV/0!	#DIV/0!	#DIV/0!
565	O95218	ZRANB2	zinc finger RANBP2-type containing 2	9406	ENSG00000132485	GSSSPRKRSYSSSSSSPERNRKRSRSRSSSS	S	0.166659	280	0	#DIV/0!	#DIV/0!	#DIV/0!
560	O95218	ZRANB2	zinc finger RANBP2-type containing 2	9406	ENSG00000132485	SRSHRGSSSPRKRSYSSSSSSPERNRKRSRS	S	0.166659	275	0	#DIV/0!	#DIV/0!	#DIV/0!

564	O95218	ZRANB2	zinc finger RANBP2-type containing 2	9406	ENSG00000132485	RGSSSPRKRSYSSSSSSPERNRKRSRSRSSS	S	0.166659	279									0	#DIV/0!	#DIV/0!	#DIV/0!
563	O95218	ZRANB2	zinc finger RANBP2-type containing 2	9406	ENSG00000132485	HRGSSSPRKRSYSSSSSSPERNRKRSRSRSS	S	0.166659	278									0	#DIV/0!	#DIV/0!	#DIV/0!
562	O95218	ZRANB2	zinc finger RANBP2-type containing 2	9406	ENSG00000132485	SHRGSSSPRKRSYSSSSSSPERNRKRSRSRS	S	0.166659	277									0	#DIV/0!	#DIV/0!	#DIV/0!
561	O95218	ZRANB2	zinc finger RANBP2-type containing 2	9406	ENSG00000132485	RSHRGSSSPRKRSYSSSSSSPERNRKRSRSR	S	0.166659	276									0	#DIV/0!	#DIV/0!	#DIV/0!
568	O95685	PPP1R3D	protein phosphatase 1 regulatory subunit 3D	5509	ENSG00000132825	PRLRPIILRRARSLPSSPERRQKAAGAPGAA	S	0.5972	77									0	#DIV/0!	#DIV/0!	#DIV/0!
569	O95685	PPP1R3D	protein phosphatase 1 regulatory subunit 3D	5509	ENSG00000132825	RLRPIILRRARSLPSSPERRQKAAGAPGAAC	S	0.64358	78									0	#DIV/0!	#DIV/0!	#DIV/0!
574	O95817	BAG3	BCL2 associated athanogene 3	9531	ENSG00000151929	PPPSPGPSAVPSSPKSVATEERAAPSTAPAE	S	0.942291	389									0	#DIV/0!	#DIV/0!	#DIV/0!
573	O95817	BAG3	BCL2 associated athanogene 3	9531	ENSG00000151929	SPFRSSVQGASSREGSPARSSTPLHSPSPIR	S	0.999736	279									0	#DIV/0!	#DIV/0!	#DIV/0!
572	O95817	BAG3	BCL2 associated athanogene 3	9531	ENSG00000151929	LRAASPFRSSVQGASSREGSPARSSTPLHSP	S	0.781377	275									0	#DIV/0!	#DIV/0!	#DIV/0!
1320	O95835	LATS1	large tumor suppressor kinase 1	9113	ENSG00000131023	YENVDSGDKEKKQITTSPITVRKNKKDEERR	T	0.463315	612									0	#DIV/0!	#DIV/0!	#DIV/0!
575	O95835	LATS1	large tumor suppressor kinase 1	9113	ENSG00000131023	ENVDSGDKEKKQITTSPITVRKNKKDEERRE	S	0.463315	613									0	#DIV/0!	#DIV/0!	#DIV/0!
580	O96013	PAK4	p21 (RAC1) activated kinase 4	10298	ENSG00000130669	LLLDEFENMSVTRSNSLRRDSPPPPARARQE	S	0.867431	99									0	#DIV/0!	#DIV/0!	#DIV/0!
579	O96013	PAK4	p21 (RAC1) activated kinase 4	10298	ENSG00000130669	LTLLLDEFENMSVTRSNSLRRDSPPPPARAR	S	0.78657	97									0	#DIV/0!	#DIV/0!	#DIV/0!
1529	P04279	SEMG1	semenogelin 1	6406	ENSG00000124233	YGENGVQKDVSQRSIYSQTEKLVAGKSQIQA	Y	0.88416	375									0	#DIV/0!	#DIV/0!	#DIV/0!
588	P04279	SEMG1	semenogelin 1	6406	ENSG00000124233	QDQQHGRKANKISYQSSSTEERRLHYGENGV	S	0.433506	290									0	#DIV/0!	#DIV/0!	#DIV/0!
616	P05060	CHGB	chromogranin B	1114	ENSG00000089199	RSETHAAGHSQEKTHSREKSSQESGEETGSQ	S	0.998041	231									0	#DIV/0!	#DIV/0!	#DIV/0!
1329	P05060	CHGB	chromogranin B	1114	ENSG00000089199	QASAIKKEELVARSETHAAGHSQEKTHSREK	T	0.898699	219									0	#DIV/0!	#DIV/0!	#DIV/0!
1331	P10909	CLU	clusterin	1191	ENSG00000120885	DQCDKCREILSVDCSTNNPSQAKLRRELDES	T	0.494382	282									0	#DIV/0!	#DIV/0!	#DIV/0!
625	P10909	CLU	clusterin	1191	ENSG00000120885	KDQCDKCREILSVDCSTNNPSQAKLRRELDE	S	0.494382	281									0	#DIV/0!	#DIV/0!	#DIV/0!
632	P12036	NEFH	neurofilament heavy	4744	ENSG00000100285	GSSSGFHSWTRTSVSSVSASPSRFRGAGAAS	S	0.875405	57									0	#DIV/0!	#DIV/0!	#DIV/0!
1334	P13473	LAMP2	lysosomal associated membrane protein 2	3920	ENSG00000005893	IAYVIGRRKSYAGYQTL______________	T	0.998793	409									0	#DIV/0!	#DIV/0!	#DIV/0!
640	P14621	ACYP2	acylphosphatase 2	98	ENSG00000170634	______________MSTAQSLKSVDYEVFGR	S	0.499835	2									0	#DIV/0!	#DIV/0!	#DIV/0!
1336	P14621	ACYP2	acylphosphatase 2	98	ENSG00000170634	_____________MSTAQSLKSVDYEVFGRV	T	0.499835	3									0	#DIV/0!	#DIV/0!	#DIV/0!
652	P19086	GNAZ	G protein subunit alpha z	2781	ENSG00000128266	AARRSRRIDRHLRSESQRQRREIKLLLLGTS	S	0.980631	27									0	#DIV/0!	#DIV/0!	#DIV/0!
654	P20366	TAC1	tachykinin precursor 1	6863	ENSG00000006128	PQQFFGLMGKRDADSSIEKQVALLKALYGHG	S	0.951046	76									0	#DIV/0!	#DIV/0!	#DIV/0!
657	P22732	SLC2A5	solute carrier family 2 member 5	6518	ENSG00000142583	VYPEKEELKELPPVTSEQ_____________	S	0.5	499									0	#DIV/0!	#DIV/0!	#DIV/0!
669	P31323	PRKAR2B	protein kinase cAMP-dependent type II regulatory subunit beta	5577	ENSG00000005249	YFGELALVTNKPRAASAHAIGTVKCLAMDVQ	S	0.999947	364									0	#DIV/0!	#DIV/0!	#DIV/0!
671	P31949	S100A11	S100 calcium binding protein A11	6282	ENSG00000163191	GLAMACHDSFLKAVPSQKRT___________	S	0.989385	101									0	#DIV/0!	#DIV/0!	#DIV/0!
681	P42685	FRK	fyn related Src family tyrosine kinase	2444	ENSG00000111816	EADKSTVIENPGALCSPQSQRHGHYFVALFD	S	0.928868	37	12.24935	11.39677		12.92998	12.34632	12.7217	12.16022		0.002068256	-0.617899895	-0.81509161	0.197191715
685	P45974	USP5	ubiquitin specific peptidase 5	8078	ENSG00000111667	AAMDISEGRSAADSISESVPVGPKVRDGPGK	S	0.789153	762									0	#DIV/0!	#DIV/0!	#DIV/0!
1349	P49006	MARCKSL1	MARCKS like 1	65108	ENSG00000175130	PSQGAEAKGEVPPKETPKKKKKFSFKKPFKL	T	1	85									0	#DIV/0!	#DIV/0!	#DIV/0!
689	P49006	MARCKSL1	MARCKS like 1	65108	ENSG00000175130	ANGQENGHVKSNGDLSPKGEGESPPVNGTDE	S	0.999984	41									0	#DIV/0!	#DIV/0!	#DIV/0!
1350	P49207	RPL34	ribosomal protein L34	6164	ENSG00000109475	_MVQRLTYRRRLSYNTASNKTRLSRTPGNRI	T	0.468342	15									0	#DIV/0!	#DIV/0!	#DIV/0!
134	P49815	TSC2	TSC complex subunit 2	7249	ENSG00000103197	GEDSRGQPEGPLPSSSPRSPSGLRPRGYTIS	S	0.621929	1449									0	#DIV/0!	#DIV/0!	#DIV/0!
699	P50443	SLC26A2	solute carrier family 26 member 2	1836	ENSG00000155850	_____________MSSESKEQHNVSPRDSAE	S	0.499885	3									0	#DIV/0!	#DIV/0!	#DIV/0!
698	P50443	SLC26A2	solute carrier family 26 member 2	1836	ENSG00000155850	______________MSSESKEQHNVSPRDSA	S	0.499885	2									0	#DIV/0!	#DIV/0!	#DIV/0!
1352	P50443	SLC26A2	solute carrier family 26 member 2	1836	ENSG00000155850	SYPSGIHLELQRESSTDFKQFETNDQCRPYH	T	0.741499	37									0	#DIV/0!	#DIV/0!	#DIV/0!
1353	P51148	RAB5C	RAB5C, member RAS oncogene family	5878	ENSG00000108774	MAIAKKLPKNEPQNATGAPGRNRGVDLQENN	T	1	191									0	#DIV/0!	#DIV/0!	#DIV/0!
701	P51159	RAB27A	RAB27A, member RAS oncogene family	5873	ENSG00000069974	KSWIPEGVVRSNGHASTDQLSEEKEKGACGC	S	0.751964	198									0	#DIV/0!	#DIV/0!	#DIV/0!
709	P55072	VCP	valosin containing protein	7415	ENSG00000165280	RDHFEEAMRFARRSVSDNDIRKYEMFAQTLQ	S	0.743787	748									0	#DIV/0!	#DIV/0!	#DIV/0!
1534	P57103	SLC8A3	solute carrier family 8 member A3	6547	ENSG00000100678	VADKRLLFYKYMHKKYRTDKHRGIIIETEGD	Y	0.916721	265									0	#DIV/0!	#DIV/0!	#DIV/0!
1355	P58546	MTPN	myotrophin	136319	ENSG00000105887	DEVKDYVAKGEDVNRTLEGGRKPLHYAADCG	T	1	31									0	#DIV/0!	#DIV/0!	#DIV/0!
1356	P60709	ACTB	actin beta	60	ENSG00000075624	DYLMKILTERGYSFTTTAEREIVRDIKEKLC	T	0.472998	202									0	#DIV/0!	#DIV/0!	#DIV/0!
1357	P60709	ACTB	actin beta	60	ENSG00000075624	YLMKILTERGYSFTTTAEREIVRDIKEKLCY	T	0.472998	203									0	#DIV/0!	#DIV/0!	#DIV/0!
722	P62857	RPS28	ribosomal protein S28	6234	ENSG00000233927	PIKLARVTKVLGRTGSQGQCTQVRVEFMDDT	S	0.679471	23									0	#DIV/0!	#DIV/0!	#DIV/0!
1361	P63092	GNAS	GNAS complex locus	2778	ENSG00000087460	TRAKYFIRDEFLRISTASGDGRHYCYPHFTC	T	0.493127	335									0	#DIV/0!	#DIV/0!	#DIV/0!
724	P63092	GNAS	GNAS complex locus	2778	ENSG00000087460	VTRAKYFIRDEFLRISTASGDGRHYCYPHFT	S	0.493127	334									0	#DIV/0!	#DIV/0!	#DIV/0!
726	P63218	GNG5	G protein subunit gamma 5	2787	ENSG00000174021	______________MSGSSSVAAMKKVVQQL	S	0.49824	2									0	#DIV/0!	#DIV/0!	#DIV/0!
728	P63218	GNG5	G protein subunit gamma 5	2787	ENSG00000174021	___________MSGSSSVAAMKKVVQQLRLE	S	0.45544	5									0	#DIV/0!	#DIV/0!	#DIV/0!
727	P63218	GNG5	G protein subunit gamma 5	2787	ENSG00000174021	____________MSGSSSVAAMKKVVQQLRL	S	0.49824	4									0	#DIV/0!	#DIV/0!	#DIV/0!
1364	P80723	BASP1	brain abundant membrane attached signal protein 1	10409	ENSG00000176788	PSSKETPAATEAPSSTPKAQGPAASAEEPKP	T	0.436236	196									0	#DIV/0!	#DIV/0!	#DIV/0!
752	Q02383	SEMG2	semenogelin 2	6407	ENSG00000124157	QAQEYGHKENKISYQSSSTEERRLNSGEKDV	S	0.433506	410									0	#DIV/0!	#DIV/0!	#DIV/0!
751	Q02383	SEMG2	semenogelin 2	6407	ENSG00000124157	DQEHGRKAHKISYPSSRTEERQLHHGEKSVQ	S	0.484112	291									0	#DIV/0!	#DIV/0!	#DIV/0!
756	Q02383	SEMG2	semenogelin 2	6407	ENSG00000124157	DQEHGHKENKMSYQSSSTEERRLNYGGKSTQ	S	0.346682	471									0	#DIV/0!	#DIV/0!	#DIV/0!
746	Q02383	SEMG2	semenogelin 2	6407	ENSG00000124157	VMGQKGGSKGQLPSGSSQFPHGQKGQHYFGQ	S	0.333333	36									0	#DIV/0!	#DIV/0!	#DIV/0!
1369	Q02383	SEMG2	semenogelin 2	6407	ENSG00000124157	EYGHKENKISYQSSSTEERRLNSGEKDVQKG	T	0.413118	413									0	#DIV/0!	#DIV/0!	#DIV/0!
745	Q02383	SEMG2	semenogelin 2	6407	ENSG00000124157	AAVMGQKGGSKGQLPSGSSQFPHGQKGQHYF	S	0.380638	34									0	#DIV/0!	#DIV/0!	#DIV/0!
764	Q02952	AKAP12	A-kinase anchoring protein 12	9590	ENSG00000131016	EGEEKQEKEPSKSAESPTSPVTSETGSTFKK	S	0.411882	178									0	#DIV/0!	#DIV/0!	#DIV/0!
1371	Q02952	AKAP12	A-kinase anchoring protein 12	9590	ENSG00000131016	EEKQEKEPSKSAESPTSPVTSETGSTFKKFF	T	0.411882	180									0	#DIV/0!	#DIV/0!	#DIV/0!
1538	Q05655	PRKCD	protein kinase C delta	5580	ENSG00000163932	KTGVAGEDMQDNSGTYGKIWEGSSKCNINNF	Y	0.966644	334									0	#DIV/0!	#DIV/0!	#DIV/0!
1373	Q08495	DMTN	dematin actin binding protein	2039	ENSG00000158856	VELPRSRERSLSPKSTSPPPSPEVWADSRSP	T	0.328046	66									0	#DIV/0!	#DIV/0!	#DIV/0!
772	Q08495	DMTN	dematin actin binding protein	2039	ENSG00000158856	HVELPRSRERSLSPKSTSPPPSPEVWADSRS	S	0.328046	65									0	#DIV/0!	#DIV/0!	#DIV/0!
773	Q08495	DMTN	dematin actin binding protein	2039	ENSG00000158856	ELPRSRERSLSPKSTSPPPSPEVWADSRSPG	S	0.328046	67									0	#DIV/0!	#DIV/0!	#DIV/0!
779	Q12929	EPS8	epidermal growth factor receptor pathway substrate 8	2059	ENSG00000151491	PVPVSKVPANITRQNSSSSDSGGSIVRDSQR	S	0.484662	659									0	#DIV/0!	#DIV/0!	#DIV/0!
781	Q12929	EPS8	epidermal growth factor receptor pathway substrate 8	2059	ENSG00000151491	PVSKVPANITRQNSSSSDSGGSIVRDSQRHK	S	0.324372	661									0	#DIV/0!	#DIV/0!	#DIV/0!
780	Q12929	EPS8	epidermal growth factor receptor pathway substrate 8	2059	ENSG00000151491	VPVSKVPANITRQNSSSSDSGGSIVRDSQRH	S	0.324372	660									0	#DIV/0!	#DIV/0!	#DIV/0!
789	Q13237	PRKG2	protein kinase cGMP-dependent 2	5593	ENSG00000138669	QASPDKVPLEVHRKTSGLVSLHSRRGAKAGV	S	0.496732	110									0	#DIV/0!	#DIV/0!	#DIV/0!
1379	Q13237	PRKG2	protein kinase cGMP-dependent 2	5593	ENSG00000138669	LQASPDKVPLEVHRKTSGLVSLHSRRGAKAG	T	0.496732	109									0	#DIV/0!	#DIV/0!	#DIV/0!
1382	Q13443	ADAM9	ADAM metallopeptidase domain 9	8754	ENSG00000168615	RDQLWRSYFRKKRSQTYESDGKNQANPSRQP	T	0.437374	735									0	#DIV/0!	#DIV/0!	#DIV/0!
1384	Q13557	CAMK2D	calcium/calmodulin dependent protein kinase II delta	817	ENSG00000145349	DGKWQNVHFHRSGSPTVPIN___________	T	0.49025	474									0	#DIV/0!	#DIV/0!	#DIV/0!
1385	Q14195	DPYSL3	dihydropyrimidinase like 3	1809	ENSG00000113657	TPKGGTPAGSARGSPTRPNPPVRNLHQSGFS	T	0.535517	638									0	#DIV/0!	#DIV/0!	#DIV/0!
803	Q14247	CTTN	cortactin	2017	ENSG00000085733	HEYQSKLSKHCSQVDSVRGFGGKFGVQMDRV	S	0.999315	117									0	#DIV/0!	#DIV/0!	#DIV/0!
812	Q15036	SNX17	sorting nexin 17	9784	ENSG00000115234	CWRVTSSVPLPSGSTSSPGRGRGEVRLELAF	S	0.199877	310									0	#DIV/0!	#DIV/0!	#DIV/0!
810	Q15036	SNX17	sorting nexin 17	9784	ENSG00000115234	TRMRCWRVTSSVPLPSGSTSSPGRGRGEVRL	S	0.247325	306									0	#DIV/0!	#DIV/0!	#DIV/0!
811	Q15036	SNX17	sorting nexin 17	9784	ENSG00000115234	MRCWRVTSSVPLPSGSTSSPGRGRGEVRLEL	S	0.199877	308									0	#DIV/0!	#DIV/0!	#DIV/0!
813	Q15036	SNX17	sorting nexin 17	9784	ENSG00000115234	WRVTSSVPLPSGSTSSPGRGRGEVRLELAFE	S	0.199877	311									0	#DIV/0!	#DIV/0!	#DIV/0!
1387	Q15036	SNX17	sorting nexin 17	9784	ENSG00000115234	RCWRVTSSVPLPSGSTSSPGRGRGEVRLELA	T	0.199877	309									0	#DIV/0!	#DIV/0!	#DIV/0!
1542	Q15054	POLD3	DNA polymerase delta 3, accessory subunit	10714	ENSG00000077514	SYTLGVHVNQAKQMLYDYVERKRKENSGAQL	Y	1	42									0	#DIV/0!	#DIV/0!	#DIV/0!
1543	Q15054	POLD3	DNA polymerase delta 3, accessory subunit	10714	ENSG00000077514	TLGVHVNQAKQMLYDYVERKRKENSGAQLHV	Y	1	44									0	#DIV/0!	#DIV/0!	#DIV/0!
353	Q15366	PCBP2	poly(rC) binding protein 2	5094	ENSG00000197111	LINVRLSSETGGMGSS_______________	S	0.5	335									0	#DIV/0!	#DIV/0!	#DIV/0!
352	Q15366	PCBP2	poly(rC) binding protein 2	5094	ENSG00000197111	YLINVRLSSETGGMGSS______________	S	0.5	334									0	#DIV/0!	#DIV/0!	#DIV/0!
818	Q15648	MED1	mediator complex subunit 1	5469	ENSG00000125686	ETVEDMVKKNLPPASSPGSKNPELGSG____	S	0.991221	545									0	#DIV/0!	#DIV/0!	#DIV/0!
817	Q15648	MED1	mediator complex subunit 1	5469	ENSG00000125686	AETVEDMVKKNLPPASSPGSKNPELGSG___	S	0.991655	544									0	#DIV/0!	#DIV/0!	#DIV/0!
822	Q15836	VAMP3	vesicle associated membrane protein 3	9341	ENSG00000049245	______________MSTGPTAATGSNRRLQQ	S	0.499391	2									0	#DIV/0!	#DIV/0!	#DIV/0!
1390	Q15836	VAMP3	vesicle associated membrane protein 3	9341	ENSG00000049245	_____________MSTGPTAATGSNRRLQQT	T	0.499391	3									0	#DIV/0!	#DIV/0!	#DIV/0!
826	Q16825	AL162171.1;PTPN21	protein tyrosine phosphatase non-receptor type 21;protein tyrosine phos	11099	ENSG00000070778	LSHGLEGLRLKERTLSASAAEVAPRAVSVGS	S	0.439937	658									0	#DIV/0!	#DIV/0!	#DIV/0!

1392	Q16825	AL162171.1;PTPN21	protein tyrosine phosphatase non-receptor type 21;protein tyrosine phosp	11099	ENSG00000070778	AGLSHGLEGLRLKERTLSASAAEVAPRAVSV	T	0.439937	656									0	#DIV/0!	#DIV/0!	#DIV/0!
1393	Q2M2I8	AAK1	AP2 associated kinase 1	22848	ENSG00000115977	TTPPPAVQGQKVGSLTPPSSPKTQRAGHRRI	T	0.968457	620									0	#DIV/0!	#DIV/0!	#DIV/0!
829	Q2M2I8	AAK1	AP2 associated kinase 1	22848	ENSG00000115977	PAVQGQKVGSLTPPSSPKTQRAGHRRILSDV	S	0.782398	624									0	#DIV/0!	#DIV/0!	#DIV/0!
1396	Q32MZ4	LRRFIP1	LRR binding FLII interacting protein 1	9208	ENSG00000124831	KEEDSERYSRRSRRNTSASDEDERMSVGSRG	T	0.333333	65									0	#DIV/0!	#DIV/0!	#DIV/0!
830	Q32MZ4	LRRFIP1	LRR binding FLII interacting protein 1	9208	ENSG00000124831	EGVAKDNAKIDGATQSSPAEPKSEDADRCTL	S	0.49721	689									0	#DIV/0!	#DIV/0!	#DIV/0!
833	Q32MZ4	LRRFIP1	LRR binding FLII interacting protein 1	9208	ENSG00000124831	DSERYSRRSRRNTSASDEDERMSVGSRGSLR	S	0.333333	68									0	#DIV/0!	#DIV/0!	#DIV/0!
832	Q32MZ4	LRRFIP1	LRR binding FLII interacting protein 1	9208	ENSG00000124831	EEDSERYSRRSRRNTSASDEDERMSVGSRGS	S	0.333333	66									0	#DIV/0!	#DIV/0!	#DIV/0!
831	Q32MZ4	LRRFIP1	LRR binding FLII interacting protein 1	9208	ENSG00000124831	GVAKDNAKIDGATQSSPAEPKSEDADRCTLP	S	0.49721	690									0	#DIV/0!	#DIV/0!	#DIV/0!
839	Q53HC5	KLHL26	kelch like family member 26	55295	ENSG00000167487	LCGMVYATGGRNRAGSLASVERYCPRRNEWG	S	0.500392	426									0	#DIV/0!	#DIV/0!	#DIV/0!
87	Q5JRC6	PHF6	PHD finger protein 6	84295	ENSG00000156531	_____________MSSSVEQKKGPTRQRKCG	S	0.666667	3									0	#DIV/0!	#DIV/0!	#DIV/0!
86	Q5JRC6	PHF6	PHD finger protein 6	84295	ENSG00000156531	______________MSSSVEQKKGPTRQRKC	S	0.666667	2									0	#DIV/0!	#DIV/0!	#DIV/0!
88	Q5JRC6	PHF6	PHD finger protein 6	84295	ENSG00000156531	____________MSSSVEQKKGPTRQRKCGF	S	0.666667	4									0	#DIV/0!	#DIV/0!	#DIV/0!
842	Q5JSZ5	PRRC2B	proline rich coiled-coil 2B	84726	ENSG00000130723	VPSGGPVPSPQTYRPSSASPSGKPSGSAVNM	S	0.905129	257									0	#DIV/0!	#DIV/0!	#DIV/0!
843	Q5JSZ5	PRRC2B	proline rich coiled-coil 2B	84726	ENSG00000130723	PSGGPVPSPQTYRPSSASPSGKPSGSAVNMG	S	0.905129	258									0	#DIV/0!	#DIV/0!	#DIV/0!
845	Q5JTZ5	C9orf152	chromosome 9 open reading frame 152	401546	ENSG00000188959	ESMVNAVWINKERRSSLSLEEADSEVEGRLE	S	0.33333	88									0	#DIV/0!	#DIV/0!	#DIV/0!
846	Q5JTZ5	C9orf152	chromosome 9 open reading frame 152	401546	ENSG00000188959	MVNAVWINKERRSSLSLEEADSEVEGRLEEA	S	0.33333	90									0	#DIV/0!	#DIV/0!	#DIV/0!
844	Q5JTZ5	C9orf152	chromosome 9 open reading frame 152	401546	ENSG00000188959	AESMVNAVWINKERRSSLSLEEADSEVEGRL	S	0.33333	87									0	#DIV/0!	#DIV/0!	#DIV/0!
265	Q5JXL7	SEPT7;SEPTIN7	septin 7	989	ENSG00000122545	KANWEAQQRILEQQNSSRTLEKNKKKGKIF_	S	0.970759	74									0	#DIV/0!	#DIV/0!	#DIV/0!
848	Q5M775	SPECC1	sperm antigen with calponin homology and coiled-coil domains 1	92521	ENSG00000128487	VDAAGRWPGVCVSRTSPTPPESATTVKSLIK	S	0.494948	729									0	#DIV/0!	#DIV/0!	#DIV/0!
1402	Q5M775	SPECC1	sperm antigen with calponin homology and coiled-coil domains 1	92521	ENSG00000128487	EVDAAGRWPGVCVSRTSPTPPESATTVKSLI	T	0.494948	728									0	#DIV/0!	#DIV/0!	#DIV/0!
849	Q5R3F8	ELFN2	extracellular leucine rich repeat and fibronectin type III domain containin	114794	ENSG00000166897	QRVSFLKPLTRSKRDSTYSQLSPRHYYSGYS	S	0.49071	741									0	#DIV/0!	#DIV/0!	#DIV/0!
1403	Q5R3F8	ELFN2	extracellular leucine rich repeat and fibronectin type III domain containin	114794	ENSG00000166897	RVSFLKPLTRSKRDSTYSQLSPRHYYSGYSS	T	0.49071	742									0	#DIV/0!	#DIV/0!	#DIV/0!
854	Q5SQI0	ATAT1	alpha tubulin acetyltransferase 1	79969	ENSG00000137343	PHPTARLLLAADPGGSPAQRRRTR_______	S	1	292	12.83639	13.31132	12.05596	11.59446	12.42511	11.86801		12.3807	0.155231993	0.949495316	1.048675378	-0.099180063
855	Q5T0B9	ZNF362	zinc finger protein 362	149076	ENSG00000160094	______________MSRSSPSGKGHSRMAEP	S	0.486942	2									0	#DIV/0!	#DIV/0!	#DIV/0!
856	Q5T0B9	ZNF362	zinc finger protein 362	149076	ENSG00000160094	____________MSRSSPSGKGHSRMAEPRF	S	0.486942	4									0	#DIV/0!	#DIV/0!	#DIV/0!
858	Q5T0Z8	C6orf132	chromosome 6 open reading frame 132	647024	ENSG00000188112	LNGRQAEATRASPPRSPAEPKGSALGPNPEP	S	1	279									0	#DIV/0!	#DIV/0!	#DIV/0!
1405	Q5T0Z8	C6orf132	chromosome 6 open reading frame 132	647024	ENSG00000188112	STPSQARGEGSPSEATRLPTQGARSSAAFPP	T	0.374751	741									0	#DIV/0!	#DIV/0!	#DIV/0!
1231	Q5TIG5	AFDN	afadin, adherens junction formation factor	4301	ENSG00000130396	MQEFRSSDGRPDSGGTLRIYADSLKPNIPYK	T	0.739539	249									0	#DIV/0!	#DIV/0!	#DIV/0!
45	Q5VW31	NFIB	nuclear factor I B	4781	ENSG00000147862	___MNSGVNLQRSLSSPPSSKRPKTISIDEN	S	0.883718	13									0	#DIV/0!	#DIV/0!	#DIV/0!
1408	Q5VWT5	FYB2	FYN binding protein 2	199920	ENSG00000187889	TPEEPRKKLETKGAQTLPSQKHVVAPKILHN	T	0.758319	193									0	#DIV/0!	#DIV/0!	#DIV/0!
879	Q68DQ2	CRYBG3	crystallin beta-gamma domain containing 3	131544	ENSG00000080200	SDGSDTTEQESTNLPSPNKSIRHEHLQLPES	S	0.710814	457									0	#DIV/0!	#DIV/0!	#DIV/0!
880	Q6AWC2	WWC2	WW and C2 domain containing 2	80014	ENSG00000151718	SSLGSLASSRGSLNTSSRGSLNSLSSTELYY	S	0.705571	358									0	#DIV/0!	#DIV/0!	#DIV/0!
890	Q6P597	KLC3	kinesin light chain 3	147700	ENSG00000104892	FPSWHLDKAPRTLSASTQDLSPH________	S	0.475231	497									0	#DIV/0!	#DIV/0!	#DIV/0!
1411	Q6P597	KLC3	kinesin light chain 3	147700	ENSG00000104892	PSWHLDKAPRTLSASTQDLSPH_________	T	0.475231	498									0	#DIV/0!	#DIV/0!	#DIV/0!
893	Q6PKG0	LARP1	La ribonucleoprotein domain family member 1	23367	ENSG00000155506	RVDPPMGEEGNHKRHSVVAGGGGGEGRKRCP	S	1	963									0	#DIV/0!	#DIV/0!	#DIV/0!
288	Q7KZI7	MARK2	microtubule affinity regulating kinase 2	2011	ENSG00000072518	ASAAVSAARPRQHQKSMSASVHPNKASGLPP	S	0.450606	533									0	#DIV/0!	#DIV/0!	#DIV/0!
289	Q7KZI7	MARK2	microtubule affinity regulating kinase 2	2011	ENSG00000072518	AAVSAARPRQHQKSMSASVHPNKASGLPPTE	S	0.450606	535									0	#DIV/0!	#DIV/0!	#DIV/0!
1415	Q7L576	CYFIP1	cytoplasmic FMR1 interacting protein 1	23191	ENSG00000273749	ITILDKYLKSGDGEGTPVEHVRCFQPPIHQS	T	0.996695	428									0	#DIV/0!	#DIV/0!	#DIV/0!
902	Q7L7X3	TAOK1	TAO kinase 1	57551	ENSG00000160551	SMGVRNSPQALRRTASGGRTEQGMSRSTSVT	S	0.49344	826									0	#DIV/0!	#DIV/0!	#DIV/0!
905	Q7Z6J0	SH3RF1	SH3 domain containing ring finger 1	57630	ENSG00000154447	GGGSGTNCTNALRSQSSTVANCSSKDLQSSQ	S	0.488928	101									0	#DIV/0!	#DIV/0!	#DIV/0!
906	Q7Z6J0	SH3RF1	SH3 domain containing ring finger 1	57630	ENSG00000154447	KDLQSSQGGQQPRVQSWSPPVRGIPQLPCAK	S	0.981212	125									0	#DIV/0!	#DIV/0!	#DIV/0!
1417	Q7Z6Z7	HUWE1	HECT, UBA and WWE domain containing E3 ubiquitin protein ligase 1;H	10075	ENSG00000086758	TSAAGSSEQPRAGSSTPGDAPPAVAEVQGRS	T	0.450661	2889									0	#DIV/0!	#DIV/0!	#DIV/0!
908	Q7Z6Z7	HUWE1	HECT, UBA and WWE domain containing E3 ubiquitin protein ligase 1;H	10075	ENSG00000086758	DTSAAGSSEQPRAGSSTPGDAPPAVAEVQGR	S	0.450661	2888									0	#DIV/0!	#DIV/0!	#DIV/0!
1418	Q86WC4	OSTM1	osteoclastogenesis associated transmembrane protein 1	28962	ENSG00000081087	QKKRKLILPKRLKSSTSFANIQENSN_____	T	0.447965	324									0	#DIV/0!	#DIV/0!	#DIV/0!
916	Q86WC4	OSTM1	osteoclastogenesis associated transmembrane protein 1	28962	ENSG00000081087	KKRKLILPKRLKSSTSFANIQENSN______	S	0.447965	325									0	#DIV/0!	#DIV/0!	#DIV/0!
1422	Q86WZ0	HEATR4	HEAT repeat containing 4	399671	ENSG00000187105	DLHDDVRIKAITTCATAALERPRIATSQRDS	T	0.333333	504									0	#DIV/0!	#DIV/0!	#DIV/0!
1421	Q86WZ0	HEATR4	HEAT repeat containing 4	399671	ENSG00000187105	SLGDLHDDVRIKAITTCATAALERPRIATSQ	T	0.333333	501									0	#DIV/0!	#DIV/0!	#DIV/0!
1420	Q86WZ0	HEATR4	HEAT repeat containing 4	399671	ENSG00000187105	QSLGDLHDDVRIKAITTCATAALERPRIATS	T	0.333333	500									0	#DIV/0!	#DIV/0!	#DIV/0!
928	Q8IWE4	DCUN1D3	defective in cullin neddylation 1 domain containing 3	123879	ENSG00000188215	___MGQCVTKCKNPSSTLGSKNGDREPSNKS	S	0.500001	13									0	#DIV/0!	#DIV/0!	#DIV/0!
1426	Q8IWE4	DCUN1D3	defective in cullin neddylation 1 domain containing 3	123879	ENSG00000188215	__MGQCVTKCKNPSSTLGSKNGDREPSNKSH	T	0.857937	14									0	#DIV/0!	#DIV/0!	#DIV/0!
927	Q8IWE4	DCUN1D3	defective in cullin neddylation 1 domain containing 3	123879	ENSG00000188215	____MGQCVTKCKNPSSTLGSKNGDREPSNK	S	0.500001	12									0	#DIV/0!	#DIV/0!	#DIV/0!
1427	Q8IWY9	CDAN1	codanin 1	146059	ENSG00000140326	PTCPTPELGSPLPSRTGSLTDEPADPARVSS	T	0.473106	282									0	#DIV/0!	#DIV/0!	#DIV/0!
1545	Q8IY63	AMOTL1	angiomotin like 1	154810	ENSG00000166025	KLHKFEKELQRISEAYESLVKSTTKRESLDK	Y	0.999861	433									0	#DIV/0!	#DIV/0!	#DIV/0!
936	Q8IY63	AMOTL1	angiomotin like 1	154810	ENSG00000166025	HKFEKELQRISEAYESLVKSTTKRESLDKAM	S	0.999921	435									0	#DIV/0!	#DIV/0!	#DIV/0!
935	Q8IY63	AMOTL1	angiomotin like 1	154810	ENSG00000166025	AATAAAERDTTIINHSRNGSYGESSLEAHIW	S	0.999997	670									0	#DIV/0!	#DIV/0!	#DIV/0!
941	Q8IZ41	RASEF	RAS and EF-hand domain containing	158158	ENSG00000165105	KLHDSNDGLRSALENSYSKFNRSLHINNISP	S	0.533897	363									0	#DIV/0!	#DIV/0!	#DIV/0!
1433	Q8N4P6	LRRC71	leucine rich repeat containing 71	149499	ENSG00000160838	KSQMVGISNSALVDKTDKTQTMKTPKGLGKK	T	0.999782	147									0	#DIV/0!	#DIV/0!	#DIV/0!
1434	Q8N4P6	LRRC71	leucine rich repeat containing 71	149499	ENSG00000160838	GISNSALVDKTDKTQTMKTPKGLGKKKEKSW	T	0.685754	152									0	#DIV/0!	#DIV/0!	#DIV/0!
1435	Q8N9U0	TC2N	tandem C2 domains, nuclear	123036	ENSG00000165929	SSSRKNSQGSNRSLDTITLSGDERDFGRLNV	T	0.877696	214									0	#DIV/0!	#DIV/0!	#DIV/0!
963	Q8NHG7	SVIP	small VCP interacting protein	258010	ENSG00000198168	RRQKEAASRGILDVQSVQEKRKKKEKIEKQI	S	1	46									0	#DIV/0!	#DIV/0!	#DIV/0!
1439	Q8TD16	BICD2	BICD cargo adaptor 2	23299	ENSG00000185963	REGQGGAGRTSPGGRTSPEARGRRSPILLPK	T	0.499609	573									0	#DIV/0!	#DIV/0!	#DIV/0!
968	Q8TD16	BICD2	BICD cargo adaptor 2	23299	ENSG00000185963	EGQGGAGRTSPGGRTSPEARGRRSPILLPKG	S	0.499609	574									0	#DIV/0!	#DIV/0!	#DIV/0!
972	Q8TE68	EPS8L1	EPS8 like 1	54869	ENSG00000131037	______________MSTATGPEAAPKPSAKS	S	0.457873	2									0	#DIV/0!	#DIV/0!	#DIV/0!
1442	Q8TE68	EPS8L1	EPS8 like 1	54869	ENSG00000131037	_____________MSTATGPEAAPKPSAKSI	T	0.457873	3									0	#DIV/0!	#DIV/0!	#DIV/0!
973	Q8TEA8	DTD1	D-aminoacyl-tRNA deacylase 1;D-tyrosyl-tRNA deacylase 1	92675	ENSG00000125821	SSKERNTPRKEDRSASSGAEGDVSSEREP__	S	0.350534	196									0	#DIV/0!	#DIV/0!	#DIV/0!
1445	Q96A65	EXOC4	exocyst complex component 4	60412	ENSG00000131558	ELEYIHALTLLHRSQTGVGELTTQNTRLQRL	T	0.733767	937									0	#DIV/0!	#DIV/0!	#DIV/0!
991	Q96BT7	ALKBH8	alkB homolog 8, tRNA methyltransferase	91801	ENSG00000137760	SDVGDLTLSKRGLRTSFTFRKVRQTPCNCSY	S	0.333333	330									0	#DIV/0!	#DIV/0!	#DIV/0!
1447	Q96BT7	ALKBH8	alkB homolog 8, tRNA methyltransferase	91801	ENSG00000137760	TSDVGDLTLSKRGLRTSFTFRKVRQTPCNCS	T	0.333333	329									0	#DIV/0!	#DIV/0!	#DIV/0!
1448	Q96BT7	ALKBH8	alkB homolog 8, tRNA methyltransferase	91801	ENSG00000137760	VGDLTLSKRGLRTSFTFRKVRQTPCNCSYPL	T	0.333333	332									0	#DIV/0!	#DIV/0!	#DIV/0!
992	Q96BY7	ATG2B	autophagy related 2B	55102	ENSG00000066739	SGVSLFWDEFSASAKSSPVCSTAPVETEPKL	S	0.489144	239									0	#DIV/0!	#DIV/0!	#DIV/0!
993	Q96BY7	ATG2B	autophagy related 2B	55102	ENSG00000066739	GVSLFWDEFSASAKSSPVCSTAPVETEPKLS	S	0.489144	240									0	#DIV/0!	#DIV/0!	#DIV/0!
1002	Q96I34	PPP1R16A	protein phosphatase 1 regulatory subunit 16A	84988	ENSG00000160972	DNPEVVRPHNGRVGGSPVRHLYSKRLDRSVS	S	1	418									0	#DIV/0!	#DIV/0!	#DIV/0!
1449	Q96JZ2	HSH2D	hematopoietic SH2 domain containing	84941	ENSG00000196684	LATVNLSSLLDVRRSTVISGPGTGKGSQDHS	T	0.499295	183									0	#DIV/0!	#DIV/0!	#DIV/0!
1451	Q96NL3	ZNF599	zinc finger protein 599	148103	ENSG00000153896	HGQELWTVKRGLSQSTCAGEKAKPKITEPTA	T	0.956328	78									0	#DIV/0!	#DIV/0!	#DIV/0!
1018	Q96TA1	NIBAN2;FAM129B	niban apoptosis regulator 2;family with sequence similarity 129 member	64855	ENSG00000136830	ELIGNSLPGTTAKSGSAPILKCPTQFPLILW	S	0.998218	135									0	#DIV/0!	#DIV/0!	#DIV/0!
1019	Q99959	PKP2	plakophilin 2	5318	ENSG00000057294	ATYEGRWGRGTAQYSSQKSVEERSLRHPLRR	S	0.9418	132									0	#DIV/0!	#DIV/0!	#DIV/0!
1028	Q9BV36	MLPH	melanophilin	79083	ENSG00000115648	HVMASHHSKRRGRASSESQIFELNKHISAVE	S	0.482491	337									0	#DIV/0!	#DIV/0!	#DIV/0!
1027	Q9BV36	MLPH	melanophilin	79083	ENSG00000115648	AHVMASHHSKRRGRASSESQIFELNKHISAV	S	0.482491	336									0	#DIV/0!	#DIV/0!	#DIV/0!
1042	Q9BW04	C1orf116;SARG	chromosome 1 open reading frame 116	79098	ENSG00000182795	SYLSTEKDASPKTSTSLGKGSFLDKISPSVL	S	0.497921	508									0	#DIV/0!	#DIV/0!	#DIV/0!
1037	Q9BW04	C1orf116;SARG	chromosome 1 open reading frame 116	79098	ENSG00000182795	PAGLPQNARAEDAPLSSGEDPNSRLAPLTTP	S	0.493234	281									0	#DIV/0!	#DIV/0!	#DIV/0!
1038	Q9BW04	C1orf116;SARG	chromosome 1 open reading frame 116	79098	ENSG00000182795	AGLPQNARAEDAPLSSGEDPNSRLAPLTTPK	S	0.493234	282									0	#DIV/0!	#DIV/0!	#DIV/0!
1048	Q9BZQ8	NIBAN1;FAM129A	niban apoptosis regulator 1;family with sequence similarity 129 member	116496	ENSG00000135842	EKVKLRVLKQYDYDSSTIRKKIFQEALVQIT	S	0.464924	489									0	#DIV/0!	#DIV/0!	#DIV/0!
1459	Q9BZQ8	NIBAN1;FAM129A	niban apoptosis regulator 1;family with sequence similarity 129 member	116496	ENSG00000135842	KVKLRVLKQYDYDSSTIRKKIFQEALVQITL	T	0.464924	490									0	#DIV/0!	#DIV/0!	#DIV/0!
1458	Q9BZQ8	NIBAN1;FAM129A	niban apoptosis regulator 1;family with sequence similarity 129 member	116496	ENSG00000135842	ALKEECRALTKGLEGTIRSDMDQIVNSKNYL	T	1	308									0	#DIV/0!	#DIV/0!	#DIV/0!
1055	Q9C0H2	TTYH3	tweety family member 3	80727	ENSG00000136295	TPLIGRESPPPSYTSSMRAKYLATSQPRPDS	S	0.33053	333									0	#DIV/0!	#DIV/0!	#DIV/0!
1549	Q9C0H2	TTYH3	tweety family member 3	80727	ENSG00000136295	RESPPPSYTSSMRAKYLATSQPRPDSSGSH_	Y	0.994402	338									0	#DIV/0!	#DIV/0!	#DIV/0!

1460	Q9C0H2	TTYH3	tweety family member 3	80727	ENSG00000136295	ENTPLIGRESPPPSYTSSMRAKYLATSQPRP	T	0.33053	331									0	#DIV/0!	#DIV/0!	#DIV/0!
1054	Q9C0H2	TTYH3	tweety family member 3	80727	ENSG00000136295	NTPLIGRESPPPSYTSSMRAKYLATSQPRPD	S	0.33053	332									0	#DIV/0!	#DIV/0!	#DIV/0!
1059	Q9C0H5	ARHGAP39	Rho GTPase activating protein 39	80728	ENSG00000147799	TLKQNTESPRASAESSPGRGSSVSREGSTSS	S	0.499021	123									0	#DIV/0!	#DIV/0!	#DIV/0!
1058	Q9C0H5	ARHGAP39	Rho GTPase activating protein 39	80728	ENSG00000147799	QTLKQNTESPRASAESSPGRGSSVSREGSTS	S	0.499021	122									0	#DIV/0!	#DIV/0!	#DIV/0!
1061	Q9GZN6	SLC6A16	solute carrier family 6 member 16	28968	ENSG00000063127	MVHLCMKPITYMSWDSSTSKEVLRPYPPWAL	S	0.306182	645									0	#DIV/0!	#DIV/0!	#DIV/0!
1060	Q9GZN6	SLC6A16	solute carrier family 6 member 16	28968	ENSG00000063127	VTMMVHLCMKPITYMSWDSSTSKEVLRPYPP	S	0.902333	642									0	#DIV/0!	#DIV/0!	#DIV/0!
1062	Q9GZR1	SENP6	SUMO specific peptidase 6	26054	ENSG00000112701	STHHTDGLSKIRLNYSDESPEAGKMLEDELV	S	0.53533	909									0	#DIV/0!	#DIV/0!	#DIV/0!
1063	Q9H0B6	KLC2	kinesin light chain 2	64837	ENSG00000174996	GGTPQEPPNPRMKRASSLNFLNKSVEEPTQP	S	0.5	581									0	#DIV/0!	#DIV/0!	#DIV/0!
1070	Q9H0B6	KLC2	kinesin light chain 2	64837	ENSG00000174996	TQPGGTGLSDSRTLSSSSMDLSRRSSLVG__	S	0.385772	609									0	#DIV/0!	#DIV/0!	#DIV/0!
1064	Q9H0B6	KLC2	kinesin light chain 2	64837	ENSG00000174996	GTPQEPPNPRMKRASSLNFLNKSVEEPTQPG	S	0.5	582									0	#DIV/0!	#DIV/0!	#DIV/0!
1076	Q9H1K6	TLNRD1	talin rod domain containing 1	59274	ENSG00000140406	_____________MASGSAGKPTGEAASPAP	S	0.364276	3									0	#DIV/0!	#DIV/0!	#DIV/0!
1466	Q9H2P9	DPH5	diphthamide biosynthesis 5	51611	ENSG00000117543	GLARVGADDQKIAAGTLRQMCTVDLGEPLHS	T	1	185									0	#DIV/0!	#DIV/0!	#DIV/0!
1082	Q9H4E7	DEF6	DEF6 guanine nucleotide exchange factor;DEF6, guanine nucleotide exc	50619	ENSG00000023892	LTRWGSQGNRTPSPNSNEQQKSLNGGDEAPA	S	0.46313	600									0	#DIV/0!	#DIV/0!	#DIV/0!
871	Q9H4G4	GLIPR2	GLI pathogenesis related 2	152007	ENSG00000122694	NTKKMGVGKASASDGSSFVVARYFPAGNVVN	S	0.499658	126									0	#DIV/0!	#DIV/0!	#DIV/0!
872	Q9H4G4	GLIPR2	GLI pathogenesis related 2	152007	ENSG00000122694	TKKMGVGKASASDGSSFVVARYFPAGNVVNE	S	0.499658	127									0	#DIV/0!	#DIV/0!	#DIV/0!
875	Q9H4G4	GLIPR2	GLI pathogenesis related 2	152007	ENSG00000122694	QQYSEALASTRILKHSPESSRGQCGENLAWA	S	0.410799	55									0	#DIV/0!	#DIV/0!	#DIV/0!
1087	Q9HBV2	SPACA1	sperm acrosome associated 1	81833	ENSG00000118434	IINWAAVKAFWGAKASTPEVQSEQSSVRYKD	S	0.537194	256									0	#DIV/0!	#DIV/0!	#DIV/0!
1096	Q9NQU5	BUB1B-PAK6;PAK6	BUB1B-PAK6 readthrough;p21 (RAC1) activated kinase 6	106821730;56	ENSG00000137843	KSLRTAPATGQLPGRSSPAGSPRTWHAQIST	S	0.494038	346									0	#DIV/0!	#DIV/0!	#DIV/0!
1097	Q9NQU5	BUB1B-PAK6;PAK6	BUB1B-PAK6 readthrough;p21 (RAC1) activated kinase 6	106821730;56	ENSG00000137843	SLRTAPATGQLPGRSSPAGSPRTWHAQISTS	S	0.494038	347									0	#DIV/0!	#DIV/0!	#DIV/0!
1469	Q9NR45	NANS	N-acetylneuraminate synthase	54187	ENSG00000095380	ELEFKFNRKALERPYTSKHSWGKTYGEHKRH	T	0.88909	72									0	#DIV/0!	#DIV/0!	#DIV/0!
1109	Q9NWM3	CUEDC1	CUE domain containing 1	404093	ENSG00000180891	_______MTSLFRRSSSGSGGGGTAGARGGG	S	0.329371	9									0	#DIV/0!	#DIV/0!	#DIV/0!
1110	Q9NWM3	CUEDC1	CUE domain containing 1	404093	ENSG00000180891	______MTSLFRRSSSGSGGGGTAGARGGGG	S	0.329371	10									0	#DIV/0!	#DIV/0!	#DIV/0!
1108	Q9NWM3	CUEDC1	CUE domain containing 1	404093	ENSG00000180891	________MTSLFRRSSSGSGGGGTAGARGG	S	0.329371	8									0	#DIV/0!	#DIV/0!	#DIV/0!
1123	Q9P206	KIAA1522	KIAA1522	57648	ENSG00000162522	SGGSTAEASDTLSIRSSGQLSGRSVSLRKLK	S	0.961405	432									0	#DIV/0!	#DIV/0!	#DIV/0!
1119	Q9P206	KIAA1522	KIAA1522	57648	ENSG00000162522	SNSVVPPPQGGSGRGSPSGGSTAEASDTLSI	S	0.431151	415									0	#DIV/0!	#DIV/0!	#DIV/0!
1129	Q9P270	SLAIN2	SLAIN motif family member 2	57606	ENSG00000109171	AVNRFSPSPRNSPRPSPKQSPRNSPRSRSPA	S	1	353									0	#DIV/0!	#DIV/0!	#DIV/0!
1125	Q9P270	SLAIN2	SLAIN motif family member 2	57606	ENSG00000109171	SLGPGSPVRAGASIPSSGAASPRGFPLGLSA	S	0.333247	58									0	#DIV/0!	#DIV/0!	#DIV/0!
1128	Q9P270	SLAIN2	SLAIN motif family member 2	57606	ENSG00000109171	AVYPAVNRFSPSPRNSPRPSPKQSPRNSPRS	S	1	349									0	#DIV/0!	#DIV/0!	#DIV/0!
1139	Q9UBI6	GNG12	G protein subunit gamma 12	55970	ENSG00000172380	_________MSSKTASTNNIAQARRTVQQLR	S	0.797881	7									0	#DIV/0!	#DIV/0!	#DIV/0!
1137	Q9UBI6	GNG12	G protein subunit gamma 12	55970	ENSG00000172380	______________MSSKTASTNNIAQARRT	S	0.330688	2									0	#DIV/0!	#DIV/0!	#DIV/0!
1473	Q9UBI6	GNG12	G protein subunit gamma 12	55970	ENSG00000172380	___________MSSKTASTNNIAQARRTVQQ	T	0.330688	5									0	#DIV/0!	#DIV/0!	#DIV/0!
1138	Q9UBI6	GNG12	G protein subunit gamma 12	55970	ENSG00000172380	_____________MSSKTASTNNIAQARRTV	S	0.330688	3									0	#DIV/0!	#DIV/0!	#DIV/0!
1141	Q9UBY5	LPAR3	lysophosphatidic acid receptor 3	23566	ENSG00000171517	KRKTNVLSPHTSGSISRRRTPMKLMKTVMTV	S	0.451949	229									0	#DIV/0!	#DIV/0!	#DIV/0!
1476	Q9UBY5	LPAR3	lysophosphatidic acid receptor 3	23566	ENSG00000171517	IYVYVKRKTNVLSPHTSGSISRRRTPMKLMK	T	0.360249	224									0	#DIV/0!	#DIV/0!	#DIV/0!
1142	Q9UDT6	CLIP2	CAP-Gly domain containing linker protein 2	7461	ENSG00000106665	LRESVLNSSVKTGNESGSNLSDSGSVKRGEK	S	0.331187	202									0	#DIV/0!	#DIV/0!	#DIV/0!
1477	Q9UDT6	CLIP2	CAP-Gly domain containing linker protein 2	7461	ENSG00000106665	RVIPLRESVLNSSVKTGNESGSNLSDSGSVK	T	0.331187	198									0	#DIV/0!	#DIV/0!	#DIV/0!
1143	Q9UDT6	CLIP2	CAP-Gly domain containing linker protein 2	7461	ENSG00000106665	ESVLNSSVKTGNESGSNLSDSGSVKRGEKDL	S	0.331187	204									0	#DIV/0!	#DIV/0!	#DIV/0!
1146	Q9UK41	VPS28	VPS28 subunit of ESCRT-I;VPS28, ESCRT-I subunit	51160	ENSG00000160948	KTMQALEKAYIKDCVSPSEYTAACSRLLVQY	S	0.987668	62									0	#DIV/0!	#DIV/0!	#DIV/0!
1148	Q9ULR3	PPM1H	protein phosphatase, Mg2+/Mn2+ dependent 1H	57460	ENSG00000111110	RAASLRGGVGAPGSPSTPPTRFFTEKKIPHE	S	0.405185	223									0	#DIV/0!	#DIV/0!	#DIV/0!
1149	Q9ULS6	KCNS2	potassium voltage-gated channel modifier subfamily S member 2	3788	ENSG00000156486	GEIRINVGGFKRRLRSHTLLRFPETRLGRLL	S	1	32									0	#DIV/0!	#DIV/0!	#DIV/0!
1482	Q9ULS6	KCNS2	potassium voltage-gated channel modifier subfamily S member 2	3788	ENSG00000156486	IRINVGGFKRRLRSHTLLRFPETRLGRLLLC	T	1	34									0	#DIV/0!	#DIV/0!	#DIV/0!
241	Q9UPQ0	LIMCH1	LIM and calponin homology domains 1	22998	ENSG00000064042	PSPDVVLRGSSDGRGSDSESDLPHRKLPDVK	S	0.945858	72									0	#DIV/0!	#DIV/0!	#DIV/0!
1157	Q9UQ03	CORO2B	coronin 2B	10391	ENSG00000103647	YLAGKGDGNIRYYEISTEKPYLSYLMEFRSP	S	0.710362	296									0	#DIV/0!	#DIV/0!	#DIV/0!
1553	Q9UQ03	CORO2B	coronin 2B	10391	ENSG00000103647	HMLYLAGKGDGNIRYYEISTEKPYLSYLMEF	Y	0.632738	293									0	#DIV/0!	#DIV/0!	#DIV/0!
1552	Q9UQ03	CORO2B	coronin 2B	10391	ENSG00000103647	THMLYLAGKGDGNIRYYEISTEKPYLSYLME	Y	0.632738	292									0	#DIV/0!	#DIV/0!	#DIV/0!
1484	Q9UQB8	BAIAP2	BAR/IMD domain containing adaptor protein 2;BAI1 associated protein 2	10458	ENSG00000175866	GLDDYGARSMSSGSGTLVSTV__________	T	0.54475	516	12.95545	12.39882	12.00222	11.45666	13.34221	12.47142	13.25995	12.62427	0.348682862	-0.108076572	0.410105864	-0.518182437
1162	Q9UQN3	CHMP2B	charged multivesicular body protein 2B	25978	ENSG00000083937	KMAKAPSAARSLPSASTSKATISDEEIERQL	S	0.577106	151									0	#DIV/0!	#DIV/0!	#DIV/0!
1168	Q9Y3R5	DOP1B	DOP1 leucine zipper like protein B	9980	ENSG00000142197	KRDRDGTQSLAANDSSRKNSWEPKPITVPQF	S	0.497526	677									0	#DIV/0!	#DIV/0!	#DIV/0!
1174	Q9Y3R5	DOP1B	DOP1 leucine zipper like protein B	9980	ENSG00000142197	ARGSPFKTKSSESPSSSPSSPARKNGGEWDV	S	0.483077	717									0	#DIV/0!	#DIV/0!	#DIV/0!
1167	Q9Y3R5	DOP1B	DOP1 leucine zipper like protein B	9980	ENSG00000142197	GKRDRDGTQSLAANDSSRKNSWEPKPITVPQ	S	0.497526	676									0	#DIV/0!	#DIV/0!	#DIV/0!
1175	Q9Y3R5	DOP1B	DOP1 leucine zipper like protein B	9980	ENSG00000142197	RGSPFKTKSSESPSSSPSSPARKNGGEWDVE	S	0.889039	718									0	#DIV/0!	#DIV/0!	#DIV/0!
1173	Q9Y3R5	DOP1B	DOP1 leucine zipper like protein B	9980	ENSG00000142197	TARGSPFKTKSSESPSSSPSSPARKNGGEWD	S	0.369491	716									0	#DIV/0!	#DIV/0!	#DIV/0!
1222	Q9Y3X0	CCDC9	coiled-coil domain containing 9	26093	ENSG00000105321	TKEGAASPAPETPQPTSPETSPKETPMQPPE	T	0.327102	385									0	#DIV/0!	#DIV/0!	#DIV/0!
130	Q9Y3X0	CCDC9	coiled-coil domain containing 9	26093	ENSG00000105321	KEGAASPAPETPQPTSPETSPKETPMQPPEI	S	0.327102	386									0	#DIV/0!	#DIV/0!	#DIV/0!
333	Q9Y485	DMXL1	Dmx like 1	1657	ENSG00000172869	LAIQQGKQKPSGLTRSTSMLISSGHNKSSNS	S	0.353168	572									0	#DIV/0!	#DIV/0!	#DIV/0!
334	Q9Y485	DMXL1	Dmx like 1	1657	ENSG00000172869	IQQGKQKPSGLTRSTSMLISSGHNKSSNSLK	S	0.332621	574									0	#DIV/0!	#DIV/0!	#DIV/0!
1258	Q9Y485	DMXL1	Dmx like 1	1657	ENSG00000172869	AIQQGKQKPSGLTRSTSMLISSGHNKSSNSL	T	0.353168	573									0	#DIV/0!	#DIV/0!	#DIV/0!
1487	Q9Y4H2	IRS2	insulin receptor substrate 2	8660	ENSG00000185950	AQDLDRGLRKRTYSLTTPARQRPVPQPSSAS	T	0.6839	579									0	#DIV/0!	#DIV/0!	#DIV/0!
1490	Q9Y5K6	CD2AP	CD2 associated protein	23607	ENSG00000198087	FGDIFKEGSVKLRTRTSSSETEEKKPEKPLI	T	0.315856	231									0	#DIV/0!	#DIV/0!	#DIV/0!
1186	Q9Y5K6	CD2AP	CD2 associated protein	23607	ENSG00000198087	DIFKEGSVKLRTRTSSSETEEKKPEKPLILQ	S	0.315856	233									0	#DIV/0!	#DIV/0!	#DIV/0!
1185	Q9Y5K6	CD2AP	CD2 associated protein	23607	ENSG00000198087	GDIFKEGSVKLRTRTSSSETEEKKPEKPLIL	S	0.315856	232									0	#DIV/0!	#DIV/0!	#DIV/0!
1554	Q9Y5K6	CD2AP	CD2 associated protein	23607	ENSG00000198087	PPTKASNLLRSSGTVYPKRPEKPVPPPPPIA	Y	0.995576	409									0	#DIV/0!	#DIV/0!	#DIV/0!
825	REV__Q16512-3	PKN1				LRIEELRHHFRVEHAVAEGAKNVLRLLSAAK	S	0.558965	228									0	#DIV/0!	#DIV/0!	#DIV/0!
920	S4R3V8	LSR	lipolysis stimulated lipoprotein receptor	51599	ENSG00000105699	GGQGSYVPLLRDTDSSVASEVRSGYRIQASQ	S	0.330936	334									0	#DIV/0!	#DIV/0!	#DIV/0!
919	S4R3V8	LSR	lipolysis stimulated lipoprotein receptor	51599	ENSG00000105699	AGGQGSYVPLLRDTDSSVASEVRSGYRIQAS	S	0.330936	333									0	#DIV/0!	#DIV/0!	#DIV/0!
730	U3KQD7	MT1X	metallothionein 1X	4501	ENSG00000187193	__________MDPNCSCSPVGSCACAGSCKC	S	0.491256	6									0	#DIV/0!	#DIV/0!	#DIV/0!
731	U3KQD7	MT1X	metallothionein 1X	4501	ENSG00000187193	________MDPNCSCSPVGSCACAGSCKCKE	S	0.491256	8									0	#DIV/0!	#DIV/0!	#DIV/0!
619	V9GYZ6	EPRS	glutamyl-prolyl-tRNA synthetase	2058	ENSG00000136628	KTGKEYIPGQPPLSQSSDSSPTRNSEPAGLE	S	0.205794	889									0	#DIV/0!	#DIV/0!	#DIV/0!
620	V9GYZ6	EPRS	glutamyl-prolyl-tRNA synthetase	2058	ENSG00000136628	TGKEYIPGQPPLSQSSDSSPTRNSEPAGLET	S	0.205794	890									0	#DIV/0!	#DIV/0!	#DIV/0!
618	V9GYZ6	EPRS	glutamyl-prolyl-tRNA synthetase	2058	ENSG00000136628	KEKTGKEYIPGQPPLSQSSDSSPTRNSEPAG	S	0.205794	887									0	#DIV/0!	#DIV/0!	#DIV/0!
867	X6R5W5	DENND4C	DENN domain containing 4C	55667	ENSG00000137145	EIESYMNLKSPLGSKSSSMELHREENRESGM	S	0.333333	37									0	#DIV/0!	#DIV/0!	#DIV/0!
869	X6R5W5	DENND4C	DENN domain containing 4C	55667	ENSG00000137145	ESYMNLKSPLGSKSSSMELHREENRESGMTT	S	0.333333	39									0	#DIV/0!	#DIV/0!	#DIV/0!
868	X6R5W5	DENND4C	DENN domain containing 4C	55667	ENSG00000137145	IESYMNLKSPLGSKSSSMELHREENRESGMT	S	0.333333	38									0	#DIV/0!	#DIV/0!	#DIV/0!
985	X6RAB3	USP6NL	USP6 N-terminal like	9712	ENSG00000148429	SGRRESGAPHRRHEHSPHPQSRTGTPERAQP	S	1	438									0	#DIV/0!	#DIV/0!	#DIV/0!
1419	X6RHS9	PROSER2	proline and serine rich 2	254427	ENSG00000148426	PVTHRKSDASDMNSDTSPSCRLRAFSRGGSL	T	0.493292	17									0	#DIV/0!	#DIV/0!	#DIV/0!
917	X6RHS9	PROSER2	proline and serine rich 2	254427	ENSG00000148426	VTHRKSDASDMNSDTSPSCRLRAFSRGGSLE	S	0.493292	18									0	#DIV/0!	#DIV/0!	#DIV/0!
674	X6RJP6	TAGLN2	transgelin 2	8407	ENSG00000158710	PNWFPKKSKENPRNFSDNQLQEGKNVIGLQM	S	1	163									0	#DIV/0!	#DIV/0!	#DIV/0!
