# Supplementary material for: Quantitative proteomic biomarkers from extracellular vesicles of human seminal plasma in the differential diagnosis of azoospermia
Source: Clin Transl Med. 2021 May 28;11(5):e423. doi: 10.1002/ctm2.423 (PMC8161617; doi:10.1002/ctm2.423)
Supplement: Supplementary file 6 — Supporting Information [file CTM2-11-e423-s006.pdf]

Supplementary Table 4A. Differential proteins of spEV from NS, NOA and OA patients.

Gene name	Significant pairs	Tissue distribution in male reproductive system according to Human Protein Atlas	Cluster in heatmap
IGKV3-20	OA > NS; OA > NOA	Not expressed	C1
IGHV1-18	OA > NS; OA > NOA	Not expressed	C1
IGHV3-30-5	OA > NOA; OA > NS	Not expressed	C1
ELSPBP1	NS > OA; NOA > OA	Epididymis*	C2
SLC5A12	NS > OA; NOA > OA	Epididymis	C2
CAMP	NS > OA; NOA > OA	Epididymis	C2
DEFB129	NS > OA; NOA > OA	Epididymis*	C2
BSPH1	NS > OA; NOA > OA	Epididymis*	C2
EDIL3	NS > OA; NOA > OA	Epididymis; Seminal vesicle; Testis; Prostate	C2
SPINT3	NS > OA; NOA > OA; NS > NOA	Epididymis	C2
CES5A	NS > OA; NOA > OA	Epididymis; Ductus deferens; Testis; Seminal vesicle; Prostate	C2
DEFB126	NS > OA; NS > NOA	Epididymis; Ductus deferens; Testis; Seminal vesicle; Prostate	C2
MFAP4	NS > OA; NOA > OA	Prostate; Epididymis; Testis; Ductus deferens; Seminal vesicle	C2
BSG	NS > OA; NOA > OA	Epididymis; Testis; Prostate	C2
PATE3	NS > OA; NOA > OA; NS > NOA	Epididymis; Ductus deferens; Testis; Seminal vesicle; Prostate	C2
MYO3B	NS > OA	Epididymis; Prostate	C2
SPAG11B	NS > OA; NS > NOA	Epididymis*	C3
ECM1	NS > OA; NS > NOA	Epididymis; Seminal vesicle	C3
RNASE13	NS > OA; NS > NOA	Epididymis; Ductus deferens; Testis; Seminal vesicle; Prostate	C3
MUC15	NS > OA; NS > NOA	Epididymis; Seminal vesicle	C3
PTGDS	NS > OA; NS > NOA	Epididymis; Testis; Seminal vesicle	C3
WFDC8	NS > OA; NS > NOA	Epididymis*	C3
DEFB118	NS > OA; NS > NOA	Epididymis*	C3
DEFB121	NS > OA; NS > NOA	Epididymis; Testis	C3
ACRBP	NS > OA; NS > NOA	Testis*-SPG; SPC; RS; ES; LC	C3
PGK2	NS > OA; NS > NOA	Testis	C3
ATP2B4	NS > OA; NS > NOA	Testis; Epididymis; Seminal vesicle; Prostate	C3
TEX101	NS > OA; NS > NOA	Testis*-PAC; RS; ES	C3
DEFB106A	NS > OA; NS > NOA	Epididymis*	C3
WFDC11	NS > OA; NS > NOA	Epididymis; Testis	C3
CCT6B	NS > OA; NS > NOA	Testis; Epididymis; Ductus deferens; Seminal vesicle; Prostate	C3
CRISP2	NS > OA; NS > NOA	Testis*-SPG; SPC; RS; ES	C3
ROPN1B	NS > OA; NS > NOA	Testis; Epididymis;	C3
HK1	NS > OA; NS > NOA	Seminal vesicle; Testis; Epididymis; ; Prostate	C3
AQP5	NS > OA; NS > NOA	Testis	C3
ATP1B3	NS > OA; NS > NOA	Testis; Prostate; Epididymis; Seminal vesicle;	C3
CYB5R2	NS > OA; NS > NOA	Testis	C3
AKAP4	NS > OA; NS > NOA	Testis*-ES	C3
PKM	NS > OA; NS > NOA	Testis; Epididymis; Seminal vesicle; Prostate	C3
HSPA1L	NS > OA; NS > NOA	Testis*-ES; RS; PAC	C3
HSPA2	NS > OA; NS > NOA	Testis	C3
SLC2A14	NS > OA; NS > NOA	Testis	C3
GLIPR1L1	NS > OA; NS > NOA	Testis*-ES; RS; PAC	C3
PSMA8	NS > OA; NS > NOA	Testis	C3
LELP1	NS > OA; NS > NOA	Testis*-ES	C3
LDHC	NS > OA; NS > NOA	Testis*-ES; RS; SPC	C3
HIST1H2BA	NS > OA; NS > NOA	Testis*-SPG; SPC; RS	C3
ACR	NS > OA; NS > NOA	Testis*-ES; RS; PAC	C3
SPACA4	NS > OA; NS > NOA	Testis*-ES; RS	C3
PGAM2	NS > OA; NS > NOA	Testis	C3

Note: * represents tissue specific proteins

SC-Sertoli cell; LC-Leydig cell; SPG-spermatogonia; SPC-spermatocytes; RS-round spermatids; ES-elongated spermatids; PAC-pachytene spermatocyte

Supplementary Table 4B. Differential phosphoproteins of spEV from NS, NOA and OA patients.

Gene name	Significant pairs	Tissue distribution in male reproductive system according to Human Protein Atlas
SEMG1	NOA > OA; OA > NS	Seminal vesicle*
EEF1D	NS > OA; NS > NOA	Testis; Epididymis; Seminal vesicle; Prostate
PRM2	NS > OA; NS > NOA	Testis*-ES
CCT6B	NS > OA; NS > NOA	Epididymis; Ductus deferens; Testis; Seminal vesicle; Prostate
LRRC37B	NS > OA; NS > NOA	Testis; Epididymis; Seminal vesicle
CANX	NS > OA; NS > NOA	Testis; Epididymis; Seminal vesicle; Prostate
SPACA1	NS > OA; NS > NOA	Testis*-ES; RS
P3R3URF;LOC11(	NS > OA; NS > NOA	Testis; Epididymis
ATP8A1	NOA > OA	Testis; Epididymis; Seminal vesicle; Prostate
SLCO6A1	NS > OA; NS > NOA	Testis*-RS; ES; SC; LC
PAK4	NS > OA; NS > NOA	Testis; Epididymis; Seminal vesicle; Prostate
TMC5	OA > NS; NOA > NS	Testis; Epididymis; Seminal vesicle; Prostate
PALM3	NS > OA; NS > NOA; NOA > OA	Testis; Epididymis; Seminal vesicle
SERPINC1	OA > NS	Not expressed
ARHGEF37	NOA > OA; NOA > NS	Testis; Epididymis; Seminal vesicle; Prostate
ATP1A4	NS > OA; NS > NOA	Epididymis; Ductus deferens; Testis; Seminal vesicle; Prostate
LTF	OA > NS	Epididymis; Prostate
SPAG11A	NS > OA; NOA > OA	Epididymis*
SGK3	NS > OA; NOA > OA	Testis; Epididymis; Seminal vesicle; Prostate
H1FNT	NS > OA	Testis; Epididymis; Prostate
FNTA	NOA > NS; OA > NS	Testis; Epididymis; Seminal vesicle; Prostate
DUOX1	NOA > OA	Testis; Epididymis;
DNAJB2	NOA > OA	Testis; Epididymis; Seminal vesicle; Prostate
ATP9A	NS > OA; NOA > OA	Testis; Epididymis; Seminal vesicle; Prostate
GPR64	NS > OA	Epididymis*
PCTP	NOA > OA	Testis; Epididymis; Seminal vesicle; Prostate
TLN2	NOA > OA	Testis; Seminal vesicle
MVP	OA > NS	Testis; Epididymis; Seminal vesicle
KLC3	NS > OA; NOA > OA	Testis; Epididymis
KIAA1522	NS > OA; NOA > OA	Testis; Epididymis; Seminal vesicle; Prostate
KCNQ1	OA > NOA	Testis; Epididymis; Seminal vesicle
PHYHD1	NOA > OA	Epididymis; Ductus deferens; Testis; Seminal vesicle; Prostate

Note: * represents tissue specific proteins

SC-Sertoli cell; LC-Leydig cell; RS-round spermatids; ES-elongated spermatids
