# Supplementary material for: Quantitative proteomic biomarkers from extracellular vesicles of human seminal plasma in the differential diagnosis of azoospermia
Source: Clin Transl Med. 2021 May 28;11(5):e423. doi: 10.1002/ctm2.423 (PMC8161617; doi:10.1002/ctm2.423)
Supplement: Supplementary file 7 — Supporting Information [file CTM2-11-e423-s002.pdf]

Supplementary Table 5A. Enriched gene ontology terms in differential proteins of spEV in cluster 2 from NS, NOA and OA patients.

GO ID	Description	Gene Ratio	BgRatio	GeneRatio/ BgRatio	MaxFC (amongCluster)	p-value	FDR-*q*	geneID	Count	Gene Sets	Cluster in heatmap
GO:0007338	single fertilization	3/12	144/18493	32.1	1	1E-04	0.00831	BSPH1/DEFB126/ELSPBP1	3	BP	C2
GO:0048240	sperm capacitation	2/12	29/18493	106	1	0.0002	0.00831	BSPH1/ELSPBP1	2	BP	C2
GO:0009566	fertilization	3/12	177/18493	26.1	1	0.0002	0.00831	BSPH1/DEFB126/ELSPBP1	3	BP	C2
GO:0061844	antimicrobial humoral immune response mediated by antimicrobial peptide	2/12	71/18493	43.4	1	0.0009	0.0279	CAMP/DEFB126	2	BP	C2
GO:0042742	defense response to bacterium	3/12	319/18493	14.5	1	0.001	0.0279	CAMP/DEFB126/DEFB129	3	BP	C2
GO:0050829	defense response to Gram-negative bacterium	2/12	87/18493	35.4	1	0.0014	0.0327	CAMP/DEFB126	2	BP	C2

GO ID	Description	Gene Ratio	BgRatio	GeneRatio/ BgRatio	MaxFC (amongCluster)	p-value	FDR-*q*	geneID	Count	Gene Sets	Cluster in heatmap
GO:0008028	monocarboxylic acid transmembrane transporter activity	2/10	53/17632	66.5	1	0.0004	0.0185	BSG/SLC5A12	2	MF	C2
GO:0005201	extracellular matrix structural constituent	2/10	155/17632	22.8	1	0.0033	0.0266	EDIL3/MFAP4	2	MF	C2
GO:0005342	organic acid transmembrane transporter activity	2/10	156/17632	22.6	1	0.0033	0.0266	BSG/SLC5A12	2	MF	C2
GO:0046943	carboxylic acid transmembrane transporter activity	2/10	156/17632	22.6	1	0.0033	0.0266	BSG/SLC5A12	2	MF	C2
GO:0015129	lactate transmembrane transporter activity	1/10	6/17632	294	1	0.0034	0.0266	SLC5A12	1	MF	C2
GO:0008201	heparin binding	2/10	163/17632	21.6	1	0.0036	0.0266	BSPH1/ELSPBP1	2	MF	C2
GO:0004771	sterol esterase activity	1/10	7/17632	252	1	0.004	0.0266	CES5A	1	MF	C2
GO:0008514	organic anion transmembrane transporter activity	2/10	209/17632	16.9	1	0.0059	0.0347	BSG/SLC5A12	2	MF	C2
GO:0005539	glycosaminoglycan binding	2/10	222/17632	15.9	1	0.0066	0.0347	BSPH1/ELSPBP1	2	MF	C2
GO:1901681	sulfur compound binding	2/10	242/17632	14.6	1	0.0079	0.0369	BSPH1/ELSPBP1	2	MF	C2
GO:0005537	mannose binding	1/10	22/17632	80.1	1	0.0124	0.0478	BSG	1	MF	C2
GO:0050431	transforming growth factor beta binding	1/10	22/17632	80.1	1	0.0124	0.0478	SPINT3	1	MF	C2
GO:0004806	triglyceride lipase activity	1/10	24/17632	73.5	1	0.0135	0.0478	CES5A	1	MF	C2
GO:0008509	anion transmembrane transporter activity	2/10	330/17632	10.7	1	0.0142	0.0478	BSG/SLC5A12	2	MF	C2

Supplementary Table 5B. Enriched gene ontology terms in differential proteins of spEV in cluster 3 from NS, NOA and OA patients.

GO ID	Description	Gene Ratio	BgRatio	GeneRatio/ BgRatio	MaxFC (amongCluster)	p-value	FDR-*q*	geneID	Count	Gene Sets	Cluster in heatmap
GO:0030317	flagellated sperm motility	6/32	89/18493	39	1	9E-09	2.8E-06	AKAP4/ATP2B4/LDHC/PGK2/I	6	BP	C3
GO:0097722	sperm motility	6/32	89/18493	39	1	9E-09	2.8E-06	AKAP4/ATP2B4/LDHC/PGK2/I	6	BP	C3
GO:0007338	single fertilization	6/32	144/18493	24.1	1	2E-07	3.4E-05	ACR/AKAP4/GLIPR1L1/HSPA	6	BP	C3
GO:0009566	fertilization	6/32	177/18493	19.6	1	5E-07	8.6E-05	ACR/AKAP4/GLIPR1L1/HSPA	6	BP	C3
GO:0006090	pyruvate metabolic process	5/32	113/18493	25.6	1	1E-06	0.00017	HK1/LDHC/PGAM2/PGK2/PKM	5	BP	C3
GO:0006754	ATP biosynthetic process	5/32	116/18493	24.9	1	2E-06	0.00017	HK1/LDHC/PGAM2/PGK2/PKM	5	BP	C3
GO:0009206	purine ribonucleoside triphosphate biosynthetic process	5/32	127/18493	22.8	1	2E-06	0.00021	HK1/LDHC/PGAM2/PGK2/PKM	5	BP	C3
GO:0009145	purine nucleoside triphosphate biosynthetic process	5/32	128/18493	22.6	1	3E-06	0.00021	HK1/LDHC/PGAM2/PGK2/PKM	5	BP	C3
GO:0009201	ribonucleoside triphosphate biosynthetic process	5/32	133/18493	21.7	1	3E-06	0.00022	HK1/LDHC/PGAM2/PGK2/PKM	5	BP	C3
GO:0009127	purine nucleoside monophosphate biosynthetic process	5/32	141/18493	20.5	1	4E-06	0.00025	HK1/LDHC/PGAM2/PGK2/PKM	5	BP	C3
GO:0009168	purine ribonucleoside monophosphate biosynthetic process	5/32	141/18493	20.5	1	4E-06	0.00025	HK1/LDHC/PGAM2/PGK2/PKM	5	BP	C3
GO:0009142	nucleoside triphosphate biosynthetic process	5/32	145/18493	19.9	1	5E-06	0.00026	HK1/LDHC/PGAM2/PGK2/PKM	5	BP	C3
GO:0046394	carboxylic acid biosynthetic process	7/32	412/18493	9.82	1	5E-06	0.00026	ATP2B4/HK1/LDHC/PGAM2/P	7	BP	C3
GO:0016053	organic acid biosynthetic process	7/32	413/18493	9.8	1	5E-06	0.00026	ATP2B4/HK1/LDHC/PGAM2/P	7	BP	C3
GO:0009156	ribonucleoside monophosphate biosynthetic process	5/32	155/18493	18.6	1	7E-06	0.00029	HK1/LDHC/PGAM2/PGK2/PKM	5	BP	C3
GO:0006096	glycolytic process	4/32	74/18493	31.2	1	8E-06	0.0003	HK1/PGAM2/PGK2/PKM	4	BP	C3
GO:0006757	ATP generation from ADP	4/32	75/18493	30.8	1	8E-06	0.0003	HK1/PGAM2/PGK2/PKM	4	BP	C3
GO:0009124	nucleoside monophosphate biosynthetic process	5/32	167/18493	17.3	1	9E-06	0.0003	HK1/LDHC/PGAM2/PGK2/PKM	5	BP	C3
GO:0042866	pyruvate biosynthetic process	4/32	78/18493	29.6	1	1E-05	0.0003	HK1/PGAM2/PGK2/PKM	4	BP	C3
GO:0072330	monocarboxylic acid biosynthetic process	6/32	293/18493	11.8	1	1E-05	0.0003	HK1/LDHC/PGAM2/PGK2/PKM	6	BP	C3
GO:0006735	NADH regeneration	3/32	25/18493	69.3	1	1E-05	0.0003	HK1/PGAM2/PKM	3	BP	C3
GO:0061621	canonical glycolysis	3/32	25/18493	69.3	1	1E-05	0.0003	HK1/PGAM2/PKM	3	BP	C3
GO:0061718	glucose catabolic process to pyruvate	3/32	25/18493	69.3	1	1E-05	0.0003	HK1/PGAM2/PKM	3	BP	C3
GO:0061615	glycolytic process through fructose-6-phosphate	3/32	26/18493	66.7	1	1E-05	0.00031	HK1/PGAM2/PKM	3	BP	C3
GO:0061620	glycolytic process through glucose-6-phosphate	3/32	26/18493	66.7	1	1E-05	0.00031	HK1/PGAM2/PKM	3	BP	C3
GO:0046031	ADP metabolic process	4/32	84/18493	27.5	1	1E-05	0.00033	HK1/PGAM2/PGK2/PKM	4	BP	C3
GO:0006165	nucleoside diphosphate phosphorylation	4/32	92/18493	25.1	1	2E-05	0.00045	HK1/PGAM2/PGK2/PKM	4	BP	C3
GO:0046939	nucleotide phosphorylation	4/32	94/18493	24.6	1	2E-05	0.00047	HK1/PGAM2/PGK2/PKM	4	BP	C3
GO:0009135	purine nucleoside diphosphate metabolic process	4/32	96/18493	24.1	1	2E-05	0.00048	HK1/PGAM2/PGK2/PKM	4	BP	C3
GO:0009179	purine ribonucleoside diphosphate metabolic process	4/32	96/18493	24.1	1	2E-05	0.00048	HK1/PGAM2/PGK2/PKM	4	BP	C3
GO:0006007	glucose catabolic process	3/32	32/18493	54.2	1	2E-05	0.00048	HK1/PGAM2/PKM	3	BP	C3
GO:0009185	ribonucleoside diphosphate metabolic process	4/32	98/18493	23.6	1	2E-05	0.00049	HK1/PGAM2/PGK2/PKM	4	BP	C3
GO:0019359	nicotinamide nucleotide biosynthetic process	4/32	106/18493	21.8	1	3E-05	0.00063	HK1/PGAM2/PGK2/PKM	4	BP	C3
GO:0019363	pyridine nucleotide biosynthetic process	4/32	106/18493	21.8	1	3E-05	0.00063	HK1/PGAM2/PGK2/PKM	4	BP	C3
GO:0072525	pyridine-containing compound biosynthetic process	4/32	109/18493	21.2	1	4E-05	0.00068	HK1/PGAM2/PGK2/PKM	4	BP	C3
GO:0006734	NADH metabolic process	3/32	38/18493	45.6	1	4E-05	0.00068	HK1/PGAM2/PKM	3	BP	C3
GO:0007339	binding of sperm to zona pellucida	3/32	38/18493	45.6	1	4E-05	0.00068	ACR/HSPA1L/TEX101	3	BP	C3
GO:0009132	nucleoside diphosphate metabolic process	4/32	116/18493	19.9	1	5E-05	0.0008	HK1/PGAM2/PGK2/PKM	4	BP	C3
GO:0009152	purine ribonucleotide biosynthetic process	5/32	239/18493	12.1	1	5E-05	0.00088	HK1/LDHC/PGAM2/PGK2/PKM	5	BP	C3
GO:0046034	ATP metabolic process	5/32	250/18493	11.6	1	6E-05	0.00106	HK1/LDHC/PGAM2/PGK2/PKM	5	BP	C3
GO:0009260	ribonucleotide biosynthetic process	5/32	253/18493	11.4	1	7E-05	0.0011	HK1/LDHC/PGAM2/PGK2/PKM	5	BP	C3
GO:0006164	purine nucleotide biosynthetic process	5/32	258/18493	11.2	1	8E-05	0.00117	HK1/LDHC/PGAM2/PGK2/PKM	5	BP	C3
GO:0046390	ribose phosphate biosynthetic process	5/32	259/18493	11.2	1	8E-05	0.00117	HK1/LDHC/PGAM2/PGK2/PKM	5	BP	C3
GO:0072522	purine-containing compound biosynthetic process	5/32	271/18493	10.7	1	9E-05	0.0014	HK1/LDHC/PGAM2/PGK2/PKM	5	BP	C3
GO:0009166	nucleotide catabolic process	4/32	140/18493	16.5	1	1E-04	0.0014	HK1/PGAM2/PGK2/PKM	4	BP	C3
GO:0009435	NAD biosynthetic process	3/32	53/18493	32.7	1	0.0001	0.00142	HK1/PGAM2/PKM	3	BP	C3
GO:0035036	sperm-egg recognition	3/32	53/18493	32.7	1	0.0001	0.00142	ACR/HSPA1L/TEX101	3	BP	C3
GO:0019362	pyridine nucleotide metabolic process	4/32	145/18493	15.9	1	0.0001	0.00142	HK1/PGAM2/PGK2/PKM	4	BP	C3
GO:0046496	nicotinamide nucleotide metabolic process	4/32	145/18493	15.9	1	0.0001	0.00142	HK1/PGAM2/PGK2/PKM	4	BP	C3
GO:0019320	hexose catabolic process	3/32	54/18493	32.1	1	0.0001	0.00142	HK1/PGAM2/PKM	3	BP	C3
GO:0009205	purine ribonucleoside triphosphate metabolic process	5/32	280/18493	10.3	1	0.0001	0.00142	HK1/LDHC/PGAM2/PGK2/PKM	5	BP	C3
GO:1901292	nucleoside phosphate catabolic process	4/32	146/18493	15.8	1	0.0001	0.00143	HK1/PGAM2/PGK2/PKM	4	BP	C3
GO:0009167	purine ribonucleoside monophosphate metabolic process	5/32	286/18493	10.1	1	0.0001	0.00144	HK1/LDHC/PGAM2/PGK2/PKM	5	BP	C3
GO:0009199	ribonucleoside triphosphate metabolic process	5/32	286/18493	10.1	1	0.0001	0.00144	HK1/LDHC/PGAM2/PGK2/PKM	5	BP	C3
GO:0009126	purine nucleoside monophosphate metabolic process	5/32	287/18493	10.1	1	0.0001	0.00144	HK1/LDHC/PGAM2/PGK2/PKM	5	BP	C3
GO:0009144	purine nucleoside triphosphate metabolic process	5/32	287/18493	10.1	1	0.0001	0.00144	HK1/LDHC/PGAM2/PGK2/PKM	5	BP	C3
GO:0072524	pyridine-containing compound metabolic process	4/32	150/18493	15.4	1	0.0001	0.00144	HK1/PGAM2/PGK2/PKM	4	BP	C3
GO:0016052	carbohydrate catabolic process	4/32	152/18493	15.2	1	0.0001	0.00149	HK1/PGAM2/PGK2/PKM	4	BP	C3
GO:0009161	ribonucleoside monophosphate metabolic process	5/32	300/18493	9.63	1	0.0002	0.0017	HK1/LDHC/PGAM2/PGK2/PKM	5	BP	C3
GO:0006733	oxidoreduction coenzyme metabolic process	4/32	162/18493	14.3	1	0.0002	0.00183	HK1/PGAM2/PGK2/PKM	4	BP	C3
GO:0009141	nucleoside triphosphate metabolic process	5/32	307/18493	9.41	1	0.0002	0.00183	HK1/LDHC/PGAM2/PGK2/PKM	5	BP	C3
GO:0046365	monosaccharide catabolic process	3/32	64/18493	27.1	1	0.0002	0.00193	HK1/PGAM2/PKM	3	BP	C3
GO:0009123	nucleoside monophosphate metabolic process	5/32	322/18493	8.97	1	0.0002	0.00221	HK1/LDHC/PGAM2/PGK2/PKM	5	BP	C3
GO:0019674	NAD metabolic process	3/32	71/18493	24.4	1	0.0002	0.00255	HK1/PGAM2/PKM	3	BP	C3
GO:0009988	cell-cell recognition	3/32	72/18493	24.1	1	0.0003	0.00262	ACR/HSPA1L/TEX101	3	BP	C3
GO:0034404	nucleobase-containing small molecule biosynthetic process	4/32	184/18493	12.6	1	0.0003	0.00273	HK1/PGAM2/PGK2/PKM	4	BP	C3
GO:0009165	nucleotide biosynthetic process	5/32	344/18493	8.4	1	0.0003	0.00283	HK1/LDHC/PGAM2/PGK2/PKM	5	BP	C3
GO:1901293	nucleoside phosphate biosynthetic process	5/32	347/18493	8.33	1	0.0003	0.0029	HK1/LDHC/PGAM2/PGK2/PKM	5	BP	C3
GO:0046434	organophosphate catabolic process	4/32	203/18493	11.4	1	0.0004	0.00379	HK1/PGAM2/PGK2/PKM	4	BP	C3
GO:0006006	glucose metabolic process	4/32	204/18493	11.3	1	0.0004	0.00381	HK1/PGAM2/PGK2/PKM	4	BP	C3
GO:0009108	coenzyme biosynthetic process	4/32	217/18493	10.7	1	0.0005	0.00474	HK1/PGAM2/PGK2/PKM	4	BP	C3
GO:0019318	hexose metabolic process	4/32	244/18493	9.47	1	0.0008	0.00726	HK1/PGAM2/PGK2/PKM	4	BP	C3

GO:0042026	protein refolding	2/32	26/18493	44.5	1	0.0009	0.00827	HSPA1L/HSPA2	2	BP	C3
GO:0051188	cofactor biosynthetic process	4/32	279/18493	8.29	1	0.0013	0.0115	HK1/PGAM2/PGK2/PKM	4	BP	C3
GO:0051085	chaperone cofactor-dependent protein refolding	2/32	31/18493	37.3	1	0.0013	0.0115	HSPA1L/HSPA2	2	BP	C3
GO:0009150	purine ribonucleotide metabolic process	5/32	488/18493	5.92	1	0.0014	0.0121	HK1/LDHC/PGAM2/PGK2/PKM	5	BP	C3
GO:0005996	monosaccharide metabolic process	4/32	287/18493	8.05	1	0.0015	0.0124	HK1/PGAM2/PGK2/PKM	4	BP	C3
GO:0007340	acrosome reaction	2/32	33/18493	35	1	0.0015	0.0125	ACR/ROPN1B	2	BP	C3
GO:0051084	'de novo' posttranslational protein folding	2/32	36/18493	32.1	1	0.0018	0.0147	HSPA1L/HSPA2	2	BP	C3
GO:0007286	spermatid development	3/32	146/18493	11.9	1	0.002	0.0166	HIST1H2BA/HSPA2/ROPN1B	3	BP	C3
GO:0042742	defense response to bacterium	4/32	319/18493	7.25	1	0.0021	0.0173	DEFB106A/DEFB118/DEFB12	4	BP	C3
GO:0006458	'de novo' protein folding	2/32	40/18493	28.9	1	0.0022	0.0174	HSPA1L/HSPA2	2	BP	C3
GO:0048515	spermatid differentiation	3/32	152/18493	11.4	1	0.0023	0.0179	HIST1H2BA/HSPA2/ROPN1B	3	BP	C3
GO:0006732	coenzyme metabolic process	4/32	351/18493	6.59	1	0.003	0.0236	HK1/PGAM2/PGK2/PKM	4	BP	C3
GO:0032781	positive regulation of ATPase activity	2/32	55/18493	21	1	0.0041	0.0314	ATP1B3/HSPA2	2	BP	C3
GO:0061077	chaperone-mediated protein folding	2/32	60/18493	19.3	1	0.0048	0.0368	HSPA1L/HSPA2	2	BP	C3
GO:1902305	regulation of sodium ion transmembrane transport	2/32	62/18493	18.6	1	0.0051	0.0378	ATP1B3/ATP2B4	2	BP	C3
GO:0000821	regulation of arginine metabolic process	1/32	3/18493	193	1	0.0052	0.0378	ATP2B4	1	BP	C3
GO:0010751	negative regulation of nitric oxide mediated signal transduction	1/32	3/18493	193	1	0.0052	0.0378	ATP2B4	1	BP	C3
GO:0045763	negative regulation of cellular amino acid metabolic process	1/32	3/18493	193	1	0.0052	0.0378	ATP2B4	1	BP	C3
GO:0008037	cell recognition	3/32	213/18493	8.14	1	0.0058	0.0422	ACR/HSPA1L/TEX101	3	BP	C3
GO:0006457	protein folding	3/32	218/18493	7.95	1	0.0062	0.0441	CCT6B/HSPA1L/HSPA2	3	BP	C3
GO:0044282	small molecule catabolic process	4/32	431/18493	5.36	1	0.0062	0.0441	ATP2B4/HK1/PGAM2/PKM	4	BP	C3
GO:0007525	somatic muscle development	1/32	4/18493	144	1	0.0069	0.0458	PGK2	1	BP	C3
GO:0015670	carbon dioxide transport	1/32	4/18493	144	1	0.0069	0.0458	AQP5	1	BP	C3
GO:0140199	negative regulation of adenylate cyclase-activating adrenergic receptor signalir	1/32	4/18493	144	1	0.0069	0.0458	ATP2B4	1	BP	C3
GO:1901896	positive regulation of calcium-transporting ATPase activity	1/32	4/18493	144	1	0.0069	0.0458	HSPA2	1	BP	C3
GO:1903276	regulation of sodium ion export across plasma membrane	1/32	4/18493	144	1	0.0069	0.0458	ATP1B3	1	BP	C3
GO:1903278	positive regulation of sodium ion export across plasma membrane	1/32	4/18493	144	1	0.0069	0.0458	ATP1B3	1	BP	C3

GO ID	Description	Gene Ratio	BgRatio	GeneRatio/ BgRatio	MaxFC (amongCluster)	p-value	FDR-*q*	geneID	Count	Gene Sets	Cluster in heatmap
GO:0097223	sperm part	8/34	188/19659	24.6	1	9E-10	9.7E-08	ACR/ACRBP/AKAP4/ATP2B4/	8	CC	C3
GO:0001669	acrosomal vesicle	5/34	106/19659	27.3	1	1E-06	5.6E-05	ACR/ACRBP/GLIPR1L1/SPAC	5	CC	C3
GO:0031514	motile cilium	5/34	177/19659	16.3	1	1E-05	0.00046	AKAP4/ATP2B4/LDHC/PGK2/I	5	CC	C3
GO:0035686	sperm fibrous sheath	2/34	6/19659	193	1	4E-05	0.00119	AKAP4/PGK2	2	CC	C3
GO:0045121	membrane raft	5/34	304/19659	9.51	1	0.0002	0.00307	ATP1B3/ATP2B4/GLIPR1L1/H	5	CC	C3
GO:0098857	membrane microdomain	5/34	305/19659	9.48	1	0.0002	0.00307	ATP1B3/ATP2B4/GLIPR1L1/H	5	CC	C3
GO:0098589	membrane region	5/34	316/19659	9.15	1	0.0002	0.0031	ATP1B3/ATP2B4/GLIPR1L1/H	5	CC	C3
GO:0036126	sperm flagellum	3/34	90/19659	19.3	1	0.0005	0.00689	AKAP4/ATP2B4/PGK2	3	CC	C3
GO:0097729	9+2 motile cilium	3/34	95/19659	18.3	1	0.0006	0.00717	AKAP4/ATP2B4/PGK2	3	CC	C3
GO:0097228	sperm principal piece	2/34	22/19659	52.6	1	0.0007	0.00722	AKAP4/ATP2B4	2	CC	C3
GO:0031225	anchored component of membrane	3/34	169/19659	10.3	1	0.0031	0.0307	GLIPR1L1/SPACA4/TEX101	3	CC	C3

GO ID	Description	Gene Ratio	BgRatio	GeneRatio/ BgRatio	MaxFC (amongCluster)	p-value	FDR-*q*	geneID	Count	Gene Sets	Cluster in heatmap
GO:0044183	protein binding involved in protein folding	2/27	23/17632	56.8	1	0.0006	0.0378	HSPA1L/HSPA2	2	MF	C3
GO:0051787	misfolded protein binding	2/27	23/17632	56.8	1	0.0006	0.0378	HSPA1L/HSPA2	2	MF	C3
GO:0051082	unfolded protein binding	3/27	127/17632	15.4	1	0.0009	0.0391	CCT6B/HSPA1L/HSPA2	3	MF	C3
GO:0015662	ATPase activity, coupled to transmembrane movement of ions, phosphorylative	2/27	33/17632	39.6	1	0.0012	0.0391	ATP1B3/ATP2B4	2	MF	C3
GO:0043531	ADP binding	2/27	39/17632	33.5	1	0.0016	0.0436	PGK2/PKM	2	MF	C3
GO:0042623	ATPase activity, coupled	4/27	357/17632	7.32	1	0.002	0.0451	ATP1B3/ATP2B4/HSPA1L/HS	4	MF	C3
GO:0016887	ATPase activity	4/27	445/17632	5.87	1	0.0044	0.0468	ATP1B3/ATP2B4/HSPA1L/HS	4	MF	C3
GO:0019829	cation-transporting ATPase activity	2/27	66/17632	19.8	1	0.0046	0.0468	ATP1B3/ATP2B4	2	MF	C3
GO:0004040	amidase activity	1/27	3/17632	218	1	0.0046	0.0468	ACR	1	MF	C3
GO:0016774	phosphotransferase activity, carboxyl group as acceptor	1/27	3/17632	218	1	0.0046	0.0468	PGK2	1	MF	C3
GO:0022853	active ion transmembrane transporter activity	2/27	67/17632	19.5	1	0.0047	0.0468	ATP1B3/ATP2B4	2	MF	C3
GO:0042625	ATPase coupled ion transmembrane transporter activity	2/27	67/17632	19.5	1	0.0047	0.0468	ATP1B3/ATP2B4	2	MF	C3
GO:0048029	monosaccharide binding	2/27	74/17632	17.6	1	0.0057	0.0468	ACR/HK1	2	MF	C3
GO:0004082	bisphosphoglycerate mutase activity	1/27	4/17632	163	1	0.0061	0.0468	PGAM2	1	MF	C3
GO:0004619	phosphoglycerate mutase activity	1/27	4/17632	163	1	0.0061	0.0468	PGAM2	1	MF	C3
GO:0004340	glucokinase activity	1/27	5/17632	131	1	0.0076	0.0468	HK1	1	MF	C3
GO:0004396	hexokinase activity	1/27	5/17632	131	1	0.0076	0.0468	HK1	1	MF	C3
GO:0004459	L-lactate dehydrogenase activity	1/27	5/17632	131	1	0.0076	0.0468	LDHC	1	MF	C3
GO:0005134	interleukin-2 receptor binding	1/27	5/17632	131	1	0.0076	0.0468	ECM1	1	MF	C3
GO:0008865	fructokinase activity	1/27	5/17632	131	1	0.0076	0.0468	HK1	1	MF	C3
GO:0019158	mannokinase activity	1/27	5/17632	131	1	0.0076	0.0468	HK1	1	MF	C3
GO:0042806	fucose binding	1/27	5/17632	131	1	0.0076	0.0468	ACR	1	MF	C3
