# Supplementary material for: Quantitative proteomic biomarkers from extracellular vesicles of human seminal plasma in the differential diagnosis of azoospermia
Source: Clin Transl Med. 2021 May 28;11(5):e423. doi: 10.1002/ctm2.423 (PMC8161617; doi:10.1002/ctm2.423)
Supplement: Supplementary file 8 — Supporting Information [file CTM2-11-e423-s003.pdf]

## Supplementary Table 6. Enriched gene ontology terms in differential phosphoproteins of spEV from NS, NOA and OA patients.

| GO ID | Description | Gene Ratio | BgRatio | GeneRatio/ BgRatio | MaxFC (among Cluster) | p-value | FDR-*q* | geneID | Count | GeneSets |
|---|---|---|---|---|---|---|---|---|---|---|
| GO:0007286 | spermatid development | 4/30 | 146/18493 | 16.9 | 1 | 8.71E-05 | 0.03 | SPACA1/PRM2/SEMG1/H1FNT | 4 | BP |
| GO:0048515 | spermatid differentiation | 4/30 | 152/18493 | 16.2 | 1 | 0.000102 | 0.03 | SPACA1/PRM2/SEMG1/H1FNT | 4 | BP |
| GO:0015914 | phospholipid transport | 3/30 | 74/18493 | 25 | 1 | 0.000231 | 0.0454 | ATP9A/ATP8A1/PCTP | 3 | BP |

| GO ID | Description | Gene Ratio | BgRatio | GeneRatio/ BgRatio | MaxFC (among Cluster) | p-value | FDR-*q* | geneID | Count | GeneSets |
|---|---|---|---|---|---|---|---|---|---|---|
| GO:0005548 | phospholipid transporter activity | 3/28 | 65/17632 | 29.1 | 1 | 0.000147 | 0.0223 | ATP9A/ATP8A1/PCTP | 3 | MF |
| GO:0004012 | phospholipid-translocating ATPase activity | 2/28 | 23/17632 | 54.8 | 1 | 0.000603 | 0.0445 | ATP9A/ATP8A1 | 2 | MF |
| GO:0051082 | unfolded protein binding | 3/28 | 127/17632 | 14.9 | 1 | 0.00105 | 0.0445 | CCT6B/DNAJB2/CANX | 3 | MF |
| GO:0043492 | ATPase activity, coupled to movement of substances | 3/28 | 132/17632 | 14.3 | 1 | 0.00117 | 0.0445 | ATP9A/ATP8A1/ATP1A4 | 3 | MF |
| GO:0005319 | lipid transporter activity | 3/28 | 146/17632 | 12.9 | 1 | 0.00157 | 0.0476 | ATP9A/ATP8A1/PCTP | 3 | MF |
