# Supplementary material for: Quantitative proteomic biomarkers from extracellular vesicles of human seminal plasma in the differential diagnosis of azoospermia
Source: Clin Transl Med. 2021 May 28;11(5):e423. doi: 10.1002/ctm2.423 (PMC8161617; doi:10.1002/ctm2.423)
Supplement: Supplementary file 9 — Supporting Information [file CTM2-11-e423-s009.pdf]

Supplementary Table 7A. Isotope-labeled heavy synthetic peptide sequences used for relative targeted quantification by PRM.

Gene Name	Peptide Sequence	Precursor ion (m/z)	Purity Grade
ACR	GSQS**C**WVAGWGYIEE**K**($^{13}C_6$,$^{15}N_2$)	932.9243++	crude
LDHC	SAETLWNIQ**K**($^{13}C_6$,$^{15}N_2$)	599.3213++	crude
CES5A	VVDGAFFPNEPLDLLSQ**K**($^{13}C_6$,$^{15}N_2$)	666.3538+++	crude
DNM1L	LHDAIVEVVT**C**LL**R**($^{13}C_6$,$^{15}N_4$)	549.9757+++	crude
HSPA1L	ARFEEL**C**ADLF**R**($^{13}C_6$,$^{15}N_4$)	768.8787++	crude
SPAG11A (pS44)	LFF**C**HS(p)GE**K**($^{13}C_6$,$^{15}N_2$)	606.7535++	crude
EDIL3	DFGHVQFVGSY**K**($^{13}C_6$,$^{15}N_2$)	696.3453++	crude
BSPH1	TYEGYW**K**($^{13}C_6$,$^{15}N_2$)	477.7259++	crude
ACRBP	YMEEEILGFG**K**($^{13}C_6$,$^{15}N_2$)	662.3227++	crude
SLC5A12	TWPLPLSTDQ**C**I**K**($^{13}C_6$,$^{15}N_2$)	783.9074++	crude
HIST1H2BA	ESYSIYIY**K**($^{13}C_6$,$^{15}N_2$)	587.2995++	crude
SPACA1 (pT257)	AST(p)PEVQSEQSSV**R**($^{13}C_6$,$^{15}N_4$)	797.8528++	crude
TEX101	AGTETAILAT**K**($^{13}C_6$,$^{15}N_2$)	542.3104++	crude
AKAP4	DQSKTEGSV**C**LF**K**($^{13}C_6$,$^{15}N_2$)	753.8710++	crude
GLIPR1L1	FEHND**C**LD**K**($^{13}C_6$,$^{15}N_2$)	593.2578++	crude
DEFB129	**C**LMGLG**R**($^{13}C_6$,$^{15}N_4$)	408.7083++	crude
CRISP2	AVSPPASNML**K**($^{13}C_6$,$^{15}N_2$)	561.8069++	crude
LELP1	**C**QPS**C**L**K**($^{13}C_6$,$^{15}N_2$)	450.7115++	crude
CAMP	AIDGINQ**R**($^{13}C_6$,$^{15}N_4$)	448.7448++	crude
ELSPBP1	F**C**ETNEYGGNSL**R**($^{13}C_6$,$^{15}N_4$)	778.8373++	crude
BSG	SESVPPVTDWAWY**K**($^{13}C_6$,$^{15}N_2$)	836.9084++	crude
SPACA4	ATS**C**GLEEPVSY**R**($^{13}C_6$,$^{15}N_4$)	739.8446++	crude
TENM2	NLLSIDYD**R**($^{13}C_6$,$^{15}N_4$)	559.7894++	crude

Supplementary Table 7B. Isotope-labeled heavy synthetic peptide sequences used for absolute targeted quantification by PRM.

Gene Name	Peptide Sequence	Precursor ion (m/z)	Purity Grade
SLC5A12	TWPLPLSTDQ**C**I**K**($^{13}C_6$,$^{15}N_2$)	783.9074++	pure
SLC5A12	EFLVGG**R** ($^{13}C_6$,$^{15}N_4$)	394.2205++	pure
HIST1H2BA	ESYSIYIY**K**($^{13}C_6$,$^{15}N_2$)	587.2995++	pure
HIST1H2BA	AMSIMNSFVTDIFE**R**($^{13}C_6$,$^{15}N_4$)	885.92512++	pure
